# Design of a multi-epitope vaccine against six Nocardia species based on reverse vaccinology combined with immunoinformatics

**DOI:** 10.3389/fimmu.2023.1100188

**Published:** 2023-02-02

**Authors:** Fei Zhu, Caixia Tan, Chunhui Li, Shiyang Ma, Haicheng Wen, Hang Yang, Mingjun Rao, Peipei Zhang, Wenzhong Peng, Yanhui Cui, Jie Chen, Pinhua Pan

**Affiliations:** ^1^ Department of Respiratory Medicine, National Key Clinical Specialty, Branch of National Clinical Research Center for Respiratory Disease, Xiangya Hospital, Central South University, Changsha, Hunan, China; ^2^ Center of Respiratory Medicine, Xiangya Hospital, Central South University, Changsha, Hunan, China; ^3^ Clinical Research Center for Respiratory Diseases in Hunan Province, Changsha, Hunan, China; ^4^ Hunan Engineering Research Center for Intelligent Diagnosis and Treatment of Respiratory Disease, Changsha, Hunan, China; ^5^ National Clinical Research Center for Geriatric Disorders, Xiangya Hospital, Changsha, Hunan, China; ^6^ Department of Infection Control Center of Xiangya Hospital, Central South University, Changsha, Hunan, China

**Keywords:** *Nocardia*, immunoinformatics, reverse vaccinology (RV), multi-epitope vaccine, molecular docking, molecular dynamics (MD) simulation

## Abstract

**Background:**

*Nocardia* genus, a complex group of species classified to be aerobic actinomycete, can lead to severe concurrent infection as well as disseminated infection, typically in immunocompromised patients. With the expansion of the susceptible population, the incidence of Nocardia has been gradually growing, accompanied by increased resistance of the pathogen to existing therapeutics. However, there is no effective vaccine against this pathogen yet. In this study, a multi-epitope vaccine was designed against the Nocardia infection using reverse vaccinology combined with immunoinformatics approaches.

**Methods:**

First, the proteomes of 6 Nocardia subspecies Nocardia subspecies (Nocardia farcinica, Nocardia cyriacigeorgica, Nocardia abscessus, Nocardia otitidiscaviarum, Nocardia brasiliensis and Nocardia nova) were download NCBI (National Center for Biotechnology Information) database on May 1st, 2022 for the target proteins selection. The essential, virulent-associated or resistant-associated, surface-exposed, antigenic, non-toxic, and non-homologous with the human proteome proteins were selected for epitope identification. The shortlisted T-cell and B-cell epitopes were fused with appropriate adjuvants and linkers to construct vaccines. The physicochemical properties of the designed vaccine were predicted using multiple online servers. The Molecular docking and molecular dynamics (MD) simulation were performed to understand the binding pattern and binding stability between the vaccine candidate and Toll-like receptors (TLRs). The immunogenicity of the designed vaccines was evaluated via immune simulation.

**Results:**

3 proteins that are essential, virulent-associated or resistant-associated, surface-exposed, antigenic, non-toxic, and non-homologous with the human proteome were selected from 218 complete proteome sequences of the 6 Nocardia subspecies epitope identification. After screening, only 4 cytotoxic T lymphocyte (CTL) epitopes, 6 helper T lymphocyte (HTL) epitopes, and 8 B cell epitopes that were antigenic, non-allergenic, and non-toxic were included in the final vaccine construct. The results of molecular docking and MD simulation showed that the vaccine candidate has a strong affinity for TLR2 and TLR4 of the host and the vaccine-TLR complexes were dynamically stable in the natural environment. The results of the immune simulation indicated that the designed vaccine had the potential to induce strong protective immune responses in the host. The codon optimization and cloned analysis showed that the vaccine was available for mass production.

**Conclusion:**

The designed vaccine has the potential to stimulate long-lasting immunity in the host, but further studies are required to validate its safety and efficacy.

## 1 Introduction

The genus *Nocardia*, a group of obligate aerobic gram-positive actinomycetes, is a common etiological agent of nocardiosis that widely exists in the soil, decaying plants, and other organic organisms (1). In 1888, Edmond Nocard isolated an acid-resistant filamentous pathogen from the diseased cattle with symptoms of primary suppurative pulmonary infection and granulomatous abscess (2). In 1889, Trevisan dubbed this strain “*Nocardia farcinica*” and introduced the *Nocardia* genus in detail (2). To date, 120 strains have been identified, of which 54 can cause nocardiosis, and based on the widely accepted Brown-Elliott classification, the *Nocardia* genus has been divided into 9 groups (3, 4). When immunity is significantly compromised, *Nocardia* can invade the body *via* trauma and the respiratory tract, leading to suppurative skin infection and lung infection, or even spreading to organs such as brain and kidney through hematogenous dissemination to result in a systemic infection (5). Notably, the case fatality rate of patients with disseminated infection is 16.2%-38.2% and may be over 50% if the central nervous system (CNS) is affected (6, 7). In recent years, with the increase in the usage of immunosuppressive drugs, organ transplantation, and the incidence of chronic lung diseases, the incidence of *Nocardia* infection has shown an upward trend.

*Nocardia* has been reported to be capable of surviving as a facultative intracellular parasite within macrophages and evading damage by host neutrophils and monocytes (8, 9). Some *Nocardia* infections are asymptomatic, combined with a long incubation period, making them difficult to identify in clinical specimens (10, 11). Moreover, the co-occurrence of bacterial and fungal infections will further complicate the diagnosis (12). In addition to immune escape and diagnostic difficulty, another issue worth noting is the heterogeneity in the genome size (ranging from 6 to 10 million base pairs (bps) among distinct *Nocardia* species (13, 14). *Nocardia* varies from different species in antimicrobial susceptibility patterns and virulence. Currently, clinically relevant *Nocardia* species have been categorized into 13 antimicrobial susceptibility patterns (15). Nevertheless, the identification of *Nocardia* species necessitates cutting-edge equipment and extensive professional expertise, which is generally difficult to achieve in clinical practice (16, 17). At present, antibiotic therapy is the mainstay of treatment for *Nocardia*, but its duration of treatment is very lengthy. It is generally recommended that patients with normal immune function should receive 6–12 months of antimicrobial therapy, but those with weakened immune systems or CNS dissemination should be treated for at least 12 months (18). Notably, the development of resistance in *Nocardia* to preferred antibiotics, such as trimethoprim-sulfamethoxazole (TMP–SMX), amikacin, and third-generation cephalosporins, is becoming highly common (19, 20). Thus, it is important to develop effective prevention strategies as quickly as possible.

Vaccination is one of the most cost-effective ways to protect people and communities against infectious diseases. However, no vaccine study on human nocardiosis has been reported yet. Traditional vaccine development is a costly and time-consuming procedure, and is fraught with uncertainty regarding the vaccine’s specificity, allergenicity, and toxicity, especially for pathogens with complex species and strains (21). Owing to developments in structural biology, genomics, proteomics, and computational science, the field of vaccines has undergone revolutionary changes during the past decades. Reverse vaccinology (RV), as a new technique, is able to locate immunogenic antigens from the pathogen’s entire genome sequence (22). It has many advantages over traditional vaccination methods (23, 24). First, convenience – the whole process starts with the analysis of genome sequence, with no need to cultivate microorganisms. Second, safety – it can avoid the spread of pathogenic microorganisms. Third, comprehensiveness – all protein antigens expressed by the pathogens in different periods and environments can be analyzed and used as candidate antigens, even when little is known about the pathogenic mechanism and immune response of the microorganisms. Moreover, RV can be combined with other methods such as immunoinformatics or various biophysical analyses to construct a novel multi-epitope vaccine. It is worth mentioning that the vaccines developed using this approach against several additional infections, including *Brucella melitensis* and *Acinetobacter baumannii*, had been tested in animal experiments, and encouraging experimental results were obtained (25, 26).

In this study, we developed a multi-epitope vaccine against *Nocardia* infection by combining RV with immunoinformatics techniques. All the proteomes of 6 *Nocardia* subspecies (i.e., *Nocardia farcinica*, *Nocardia cyriacigeorgica*, *Nocardia abscessus*, *Nocardia otitidiscaviarum*, *Nocardia brasiliensis* and *Nocardia nova*) that have a much wider range of popularity were employed to identify the ideal T-cell (CTL and HTL) and B-cell epitopes to be used in the final vaccine construct. The binding mode with immune receptors, the immune effect, the population coverage, and the potential for mass production of the designed vaccine were then assessed using immunoinformatics methods. This study not only gives theoretical reference and data for future research on the *Nocardia* vaccine but also offers new ideas for vaccine development against other similar pathogens.

## 2 Methodology

### 2.1 Target proteins screening

#### 2.1.1 Proteome retrieval and core proteins screening

A total of 218 complete proteome sequences of the 6 *Nocardia* subspecies (i.e., *Nocardia farcinica*, *Nocardia cyriacigeorgica*, *Nocardia abscessus*, *Nocardia otitidiscaviarum*, *Nocardia brasiliensis*, and *Nocardia nova*) were obtained from the NCBI (National Center for Biotechnology Information) database on May 1st, 2022. The Bacterial Pan Genome Analysis (BPGA) tool v1.3, which utilizes USEARCH to cluster proteins with a sequence identity threshold of 0.5, was employed to identify core proteins (27, 28).

#### 2.1.2 Screening of essential proteins, virulence proteins and resistance-related proteins

To identify the essential proteins, all selected proteins were submitted to the BLAST v2.12.0+ software to perform sequence alignment between selected proteins and proteins in the Database of Essential Genes 10 (DEG 10) based on the filtering criteria of E-value<10-4, bit score>100 and sequence identity>30% (29). The DEG 10 (http://origin.tubic.org/deg/public/index.php) is a database containing frequently updated essential proteins of prokaryotes (30). Moreover, the virulence proteins and resistance-related proteins were identified *via* BLASTp from the Virulence Factor database (VFDB) (http://www.mgc.ac.cn/VFs/) and The Comprehensive Antibiotic Resistance Database (CARD) (https://card.mcmaster.ca/), respectively, with the filtering criteria set to E-value<10-4, bit score>100 and sequence identity >30% in both cases (31, 32). All of the above sequence data was downloaded from DEG, VFDB and CARD on May 1st, 2022.

#### 2.1.3 Screening of human non-homologous proteins

To avoid inducing autoimmune reactions, we checked the homology between the chosen proteins and the human proteome. First, we obtained the Homo species reference proteome (taxid: 9606) from the NCBI database and then built a local database using the local BLAST v2.12.0+ software based on the obtained data. Then, sequence alignment between selected essential proteins and the established local database was performed using BLASTp. Following the exclusion criteria of E-value<10-4 and bit score>100, the essential proteins with homology to the human proteome were excluded.

#### 2.1.4 Analysis of antigenicity and subcellular localization

VaxiJen2.0 (http://www.ddg-pharmfac.net/vaxijen/VaxiJen/VaxiJen.html) was utilized to assess the antigenicity of the chosen proteins based on the physicochemical properties of protein amino acids (33). To ensure that the final epitopes included in the vaccine construct have better binding ability to lymphocyte receptors or antibodies, we increased the screening threshold for antigenicity to 0.5. Moreover, to validate the results, ANTIGENpro (http://scratch.proteomics.ics.uci.edu/) was also used to predict the antigenicity of chosen proteins (34). The membrane and secretory proteins are easily accessible to lymphocyte receptors and antibodies, indicating that they may serve the ideal vaccine targets. Hence, two web servers, PSORTb v3.0.2 (https://www.psort.org/psortb/) and Cell-PLoc 2.0 (http://www.csbio.sjtu.edu.cn/bioinf/Cell-PLoc-2/) were employed to predict the subcellular localization of the selected proteins. PSORTb v3.0.2, a bacterial localization prediction program, can be used to predict the cytoplasmic membrane, outer membrane, periplasm, extracellular and other subcellular localizations (35), and Cell-PLoc 2.0 can also be used to predict subcellular localizations of proteins in many organisms (36).

#### 2.1.5 Prediction of transmembrane helix, peptide signal and physicochemical properties

The DeepTMHMM server (https://dtu.biolib.com/DeepTMHMM) was employed to predict the transmembrane helix, and the results suggested that its performance was superior to that of previous versions (37). Only the selected proteins with transmembrane helix ≤1 would proceed to the next screening step. The SignalIP 6.0 server (https://services.healthtech.dtu.dk/service.php?SignalP-6.0), which can predict 5 types of signal peptides and their cleavage sites in bacterial and archaeal proteins, was used to predict the signal sequence of selected proteins, and only the proteins without signal sequence were considered for vaccine construction (38). Physicochemical properties such as molecular weight and instability index were calculated *via* the ProtParam server (https://web.expasy.org/protparam/) (39). To facilitate protein purification during vaccine development, only proteins with appropriate molecular weight (<110KDa) and ideal stability (instability index<40) were selected for epitope identification. Further, the toxicity and allergenicity of proteins were predicted by ToxinPred2 and AllerTOP v.2.0 servers, respectively (40, 41). Finally, the models of selected proteins were predicted *via* Robetta server (42), the quality of which was validated through PROCHECK (https://saves.mbi.ucla.edu/) (43), ERRAT (https://saves.mbi.ucla.edu/) (44) and the ProSA-web (https://prosa.services.came.sbg.ac.at/prosa.php) (45) servers.

### 2.2 Epitopes screening

#### 2.2.1 Prediction of cytotoxic T lymphocyte epitopes

The CTL epitopes were restrictive in binding to MHC class I molecules. To design vaccines with a wide population coverage, we selected human leukocyte antigen (HLA) class I alleles with a high frequency of expression in this study. Each HLA-I allele covered more than 5% of the global population, which was calculated by the Population Coverage tool in the Immune Epitope Database (IEDB) (http://tools.iedb.org/mhci/). There were high frequency HLA-I alleles (HLA-A*01:01, HLA-A*02:01, HLA-A*03:01, HLA-A*11:01, HLA-A*23:01, HLA-A*24:02, HLA-A*26:01, HLA-A*31:01, HLA-A*68:01, HLA-B*07:02, HLA-B*08:01, HLA-B*18:01, HLA-B*35:01, HLA-B*40:01, HLA-B*44:02, HLA-B*44:03, HLA-B*51:01, HLA-C*01:02, HLA-C*02:02, HLA-C*03:03, HLA-C*04:01, HLA-C*03:04, HLA-C*05:01, HLA-C*06:02, HLA-C*07:01, HLA-*C07:02). The NetCTLpan1.1 server (https://services.healthtech.dtu.dk/service.php?NetCTLpan-1.1) was utilized to predict CTL epitopes by combining MHC-binding peptide prediction, proteasome cleavage site prediction and TAP transport efficiency prediction (46). In this server, the threshold for epitope identification was set to 1%, which indicated that CTL epitopes had good binding to HLA-I alleles.

#### 2.2.2 Prediction of helper T lymphocytes epitopes

NetMHCIIpan 4.0 (https://services.healthtech.dtu.dk/service.php?NetMHCIIpan-4.0), which predicts peptide binding to any major histocompatibility complex (MHC) II molecules of the known sequence using Artificial Neural Networks (ANNs), was employed to predict HTL epitopes, with the 14 most prevalent HLA II alleles (HLA-DRB1*01:01, HLA-DRB1*03:01, HLA-DRB1*04:01, HLA-DRB1*04:04, HLA-DRB1*04:05, HLA-DRB1*07:01, HLA-DRB1*08:02, HLA-DRB1*09:01, HLA-DRB1*11:01, HLA-DRB1*13:02, HLA-DRB1*15:01, HLA-DRB3*01:01, HLA-DRB4*01:01, HLA-DRB5*01:01) in human selected as the binding targets (47). In this study, the epitopes with a percentile rank<1%, which are considered stronger binders, were chosen for further analysis.

#### 2.2.3 Prediction of B-cell epitopes

B-cell epitopes are essential for eliciting humoral immune responses and neutralizing antibody responses. In this study, we utilized three servers to identify B-cell epitopes, and only overlapping epitopes were chosen for further analysis. The first server is ABCpred (https://webs.iiitd.edu.in/raghava/abcpred/), which uses the artificial neural network (ANN) to predict epitopes with an accuracy of 65.3% (48, 49). The second server is Bepipred2.0 (http://tools.iedb.org/bcell/), which employs a random forest method to predict B-cell epitopes from antigen sequences (50). The third server is ElliPro (http://tools.iedb.org/ellipro/), which was used to predict potential B-cell epitopes based on the 3D structure of proteins (51).

#### 2.2.4 Antigenicity, toxicity, allergenicity, and surface accessibility of selected epitopes

The antigenicity of selected epitopes was predicted using the VaxiJen 2.0 server, and the epitopes with antigenicity>0.5 were further submitted to the ToxinPred2 (https://webs.iiitd.edu.in/raghava/toxinpred2/algo.html) and AllerTOP v.2.0 (https://www.ddg-pharmfac.net/AllerTOP/) servers to check for their toxicity and allergenicity, respectively. The screening threshold of the ToxinPred2 server was set to 0.6, and its performance appeared to be superior to that of other servers. AllerTOP v.2.0 predicts the antigenicity of epitopes using the k closest neighbors (kNN) algorithm with an accuracy of 88.7% (41). The accessibility of epitopes on protein surfaces was observed by the surface mode of the PyMOL v2.4 software (52). Based on the above prediction results, all epitopes located in signal peptides, transmembrane helices, and intramembrane regions were excluded.

#### 2.2.5 Immunogenicity analysis of CTL epitopes and validation of the binding of HLA class I alleles

Immunogenicity is the capacity of an agent, such as an epitope or antigen, to stimulate an immune response within the body. In this study, the Class I Immunogenicity tool (http://tools.iedb.org/immunogenicity/) in the IEDB was employed to evaluate the immunogenicity of CTL (the higher the immunogenicity score, the greater the probability of triggering a host immune response) (53). TepiTool (http://tools.iedb.org/tepitool/) from IEDB was applied to validate HLA class I alleles that bind to CTL epitopes using a consensus algorithm that integrates four methods (54, 55), and it is generally believed that epitopes with IC50 ≤ 500 nm have moderate binding to HLA class I molecules.

#### 2.2.6 Prediction of cytokine induction of HTL epitopes

IFN- γ is a key player in cellular immunity, with the capacity to improve antigen processing and presentation, stimulate T-cell differentiation, increase leukocyte transport, and enhance antibacterial activity (56). IL-2 and IL-4 are also essential players in driving T-cell and B-cell proliferation and differentiation (57, 58). Hence, in this study, we evaluated the capacities of all selected HTL-epitopes to induce IFN-γ, IL-2, and IL-4 secretion by using IFNepitope (https://webs.iiitd.edu.in/raghava/ifnepitope/help.php), IL2Pred (https://webs.iiitd.edu.in/raghava/il2pred/), and IL4Pred (https://webs.iiitd.edu.in/raghava/il4pred/) servers, respectively (59–61). Epitopes that can induce the production of IFN-γ and at least one of IL-2 and IL-4 were finally selected.

### 2.3 Molecular docking between T-cell epitopes and MHC molecules

The MHC molecules as essential molecules in the human body are involved in the presentation of antigen molecules, the interaction between immune cells, and the differentiation of T cells. It is therefore necessary to investigate the interactive models between T-cell epitopes and MHC molecules. First, the selected T-cell epitopes were submitted to the PEP-FOLD 3 server (https://bioserv.rpbs.univ-paris-diderot.fr/services/PEP-FOLD3/) for generating peptide 3D structures (62). The 3D structures of MHC class I molecules (HLA-A*01:01, HLA-B*58:01, HLA-B*15:02, HLA-B*35:01) and MHC class II molecules (HLA-DRB1*01:01) were directly obtained from the RCSB PDB website (https://www.rcsb.org/) (63), while the structures (HLA-DRB1*03:01, HLA-DRB1*04:05, HLA-DRB1*07:01, HLA-DRB4*01:01) not available on the website were modeled through the SWISS-MODEL server (https://swissmodel.expasy.org/) (64). The quality of the model was validated using PROCHECK, ERRAT, and the ProSA-web servers. Then, the Swiss-PdbViewer software was used for the energy minimization of receptors and ligands (65), and Autodock Vina in the PyRx v0.8 was employed to perform molecular docking between T-cell epitopes and MHC molecules (66). The docking results were subsequently analyzed with LigPlot+ v.2.2 (67).

### 2.4 Vaccine construction

Compared to conventional vaccines, multi-epitope peptide vaccines are weakly immunogenic. Human beta-defensins (HBDs) are small molecular cationic antimicrobial peptides produced by epithelial cells that exhibit antibacterial and immunomodulatory activity, which is considered to be part of the innate and adaptive immune response (68). Hence, to enhance the immunogenicity of the vaccine candidate, β-defensin 3 was added to the vaccine’s N-terminus as an adjuvant. Further, for avoiding the formation of newly connected epitopes and ensuring the independent immunogenicity of each epitope, appropriate connectors were added between the epitopes. Then, the EAAAK linker was utilized to connect adjuvants with the vaccine (69), and the CTL, HTL, and B cell epitopes were linked by AAY, GPGPG, and KK linkers, respectively. The HEYGAEALERAG linkers, acting as cleavage sites for the proteasomal and lysosomal systems, were chosen as spacer linkers to connect CTL, HTL, and B cell epitopes (70). The PADRE sequence, which can facilitate antibody responses, was linked to the C-terminal of the vaccine using the EAAAK linker (71).

### 3.5 Features of vaccine construct

The VaxiJen2.0 server was used to predict the antigenicity of the vaccine candidate, which was then confirmed by the ANTIGENpro server. The allergenicity and toxicity were checked by AllerTOP v.2.0 and the ToxinPred2 server, respectively. Thereafter, the ProtParam tool was employed to check the physiochemical properties of the designed vaccine, including molecule weight, instability index, the number of amino acids, theoretical isoelectric point (PI), aliphatic index, and GRAVY and half-life. The potential for soluble expression of the vaccine candidate in *E. coli* was evaluated by the SOLpro server (http://scratch.proteomics.ics.uci.edu/) with an accuracy of about 74% (72). Also, the DeepTMHMM and SignalP6.0 servers were employed to predict possible transmembrane helices and signal peptides in the vaccine, respectively.

### 2.6 Secondary and 3D structure predictions, 3D structure refinement and validation

The secondary structure of the vaccine was predicted by the PSIPRED 4.0 server (http://bioinf.cs.ucl.ac.uk/psipred/) with an accuracy of 84.2% (73). The putative 3D structure of the vaccine was generated using the Robetta server (http://www.robetta.org/), which executes comparative modeling if a suitable template is in the database and executes *ab initio* structure prediction otherwise. Then, the primary structures were submitted to GalaxyRefine for refinement (74). The GalaxyRefine server (https://galaxy.seoklab.org/cgi-bin/submit.cgi?type=REFINE) can improve the structure quality by rebuilding side chains and relaxing the overall structure, and its main parameter MolProbity was used to evaluate the quality of refined models (the lower the MolProbity score, the better the quality of the model). Furthermore, the quality of refined models was validated by the PROCHECK -generated Ramachandran plot, the ERRAT score, and the Z score generated ProSA-web.

### 2.7 Disulfide engineering of vaccine

To assist protein folding and improve the stability of the protein structure, the Disulfide by Design (DbD) v2.13 server (http://cptweb.cpt.wayne.edu/DbD2/) was employed to conduct disulfide engineering of the designed vaccine, which could check residue pairs with the ability to form disulfide bonds if the amino acid mutated to cysteine (75). The potential residue pairs were then selected based on the criteria of chi3 value -87° or +97° ± 30 and energy value< 2.2 kcal/mol. Subsequently, the PROCHECK, ERRAT, and ProSA-web servers were used to assess the quality of the model with selected disulfide bonds.

### 2.8 Vaccine-Toll-like receptors docking

The molecular docking of the designed vaccine with the receptor can help clear the binding mode between two molecules. Toll-like receptor 4 (TLR4), a type I transmembrane protein expressed in a variety of tissues including monocytes and macrophages, plays a key role in activating innate immunity by directly recognizing pathogen-related molecular patterns (PAMP), such as lipopolysaccharide (LPS), lipoteichoic acid (LTA), double-stranded RNA (dsRNA), etc (76). TLR2, another member of the TLR family, can recognize various microbial components from gram-positive bacteria such as lipopeptide, peptidoglycan, and fatty acids, and regulate the phenotype and function of dendritic (DC) cells, T cells, natural killer (NK) cells, macrophages and other immune cells (76, 77). Hence, the molecular docking between the multi-epitope vaccine and TLR2 (PDB ID:2Z7X) or TLR4 (PDB ID:4G8A) was realized by the ClusPro 2.0 server (https://cluspro.org/help.php), which ranks the cluster of docking complexes according to their central and lowest energy scores (78). Then, the best docking model was submitted to the HADDOCK 2.4 server (https://wenmr.science.uu.nl/haddock2.4/) for refinement, with the parameters set to default (79). Besides, the PDBsum server (http://www.ebi.ac.uk/thornton-srv/databases/cgi-bin/pdbsum/GetPage.pl?pdbcode=index.html) was used to analyze the interacting residues between the vaccine and TLRs (80).

### 2.9 Molecular dynamics simulation and analysis

#### 2.9.1 MD simulation

The MD simulation was run using the Gromacs v2022.1 software to understand the dynamics and stability of vaccine–TLR docking complexes (81), and all MD simulations and corresponding analyses were run in triplicate for ensuring the accuracy of the results. During the initial phase, the AMBER14SB_PARMBSC1 force field was added to generate the coordinate file and topology of the vaccine-receptor complex (82). The complex was then solvated by adding transferable intermolecular potential 3P (TIP3P) water molecules, followed by the addition of counter ions (Na+ and Cl-) to neutralize the charge of the simulated system. Subsequently, energy minimization was performed until reaching the desired threshold (the maximum force ≤1000.0 kJ/mol/nm). To achieve a stable temperature and pressure environment, NVT and NPT simulations were run for 200ps and 1ns, respectively, and the system’s temperature and pressure were equilibrated to 310K and 1atm, respectively. The modified Berendsen thermostat was employed to regulate the temperature of the system (83), while the Parinello-Rahman barostat was used to equilibrate the pressure (84). Subsequently, the MD simulation was run for 100 ns with an isothermal and isobaric system. At last, the LINCS algorithm was used to constrain the hydrogen bonds (85), and the Particle-Mesh Ewald summation scheme was executed to calculate long-range electrostatic interactions (86). Finally, the VMD v1.93 software was used to generate the movie of the MD simulations (87).

#### 2.9.2 MM-PBSA calculation

To calculate the binding free energy estimates between the vaccine and TLRs, the last 10 ns of the trajectory was extracted and further submitted to the gmx_MMPBSA v1.56 tool (88). Besides, the energy contribution of all residues within 4 Å of the receptor and ligand was also analyzed using the gmx_MMPBSA v1.56 tool. In this study, the MM-PBSA method was adopted to predict the binding free energy of the complex, whose mathematical explanation is:


$$\begin{array}{c} \Delta G_{bind,solv}=\Delta G_{bind,vaccum}+\Delta G_{solv,complex}-(\Delta G_{solv,ligand}\\ +\Delta G_{solv,receptor}) \end{array}$$

#### 2.9.3 Principal component analysis and dynamical cross-correlation analysis

The principal components (PCs) are eigenvectors that determine the direction of motion, and the extent of residual motion during the MD is defined by the corresponding eigenvalues. In this study, PCs were mainly projected to two dimensions, the principal component 1 (PC1) and the principal component 2 (PC2). Then, the DCC analysis was performed to understand the correlation between the motions of the residues of the vaccine chain and the motions of the residues of the TLRs chain (89). The last 50 ns of the trajectory was extracted and used for the analyses of PCA and DCC, which were operated using the Bio3D package in R version 4.21 (90).

### 2.10 Immune simulation

The immune simulation of the designed vaccine was performed using the C-ImmSim server (https://kraken.iac.rm.cnr.it/C-IMMSIM/index.php?page=0), which can simulate the immune response generated by a mammalian thymus (T cells), bone marrow (lymph and bone marrow cells), and a lymphoid organ (91). The simulation results could help us estimate the immunogenicity of the vaccine and the immune response it might induce in the human body. According to previous studies, the administration scheme adopted in this study was 1000 vaccine units at a time, once every four weeks, for a total of three injections. The total number of steps was set to 1050, with 1, 84, and 168 time steps for the first to the third injection, respectively. The host MHC molecule combinations were HLA-A*01:01, HLA-B*07:02, and HLA-DRB1*01:01, and other parameters were kept unchanged.

### 2.11 Population coverage

The proportion of the population that will respond to the vaccine (population coverage) is another important indicator to consider in the vaccine design process. Distinct epitopes bind to different HLA alleles, and the distribution frequency of distinct alleles varies across different regions and ethnicities. Based on the distribution frequency of different HLA alleles and the HLA alleles bound to the vaccine, the global population coverage of the vaccine was calculated using the Population Coverage tool (http://tools.iedb.org/population/) in IEDB (92).

### 2.12 Codon optimization and cloning

To improve the efficiency of vaccine expression in the selected expression host, the vaccine constructs were submitted to the online tool VectorBuilder (https://www.vectorbuilder.cn/tool/codon-optimization.html) for codon optimization, and *E. coli* (Strain K12) was selected as the expression host in this study. The codon adaptation index (CAI) and percentage GC content, generated by the VectorBuilder server, were utilized to evaluate protein expression levels. The values of CAI vary from 0 to 1, and a high CAI value indicates a high-level expression of genes. The recommended range of GC content varies from 30% to 70%. Then, the Xhoi and BamHI restriction endonuclease sites were attached to the vaccine’s C terminal and N terminal, respectively. At last, the final sequence of the vaccine was inserted into the pET-28a (+) vector using the GenSmart tool (https://www.genscript.com/gensmart-design/).

### 2.13 Prediction of the vaccine mRNA secondary structure

The Transcription and Translation Tool (http://biomodel.uah.es/en/lab/cybertory/analysis/trans.htm) was applied to obtain the mRNA sequence of the vaccine. Two online servers, Mfold v2.3 (http://www.unafold.org/mfold/applications/rna-folding-form-v2.php) (93) and RNAfold (http://rna.tbi.univie.ac.at/cgi-bin/RNAWebSuite/RNAfold.cgi) (94) were used to predict the secondary structure of the vaccine mRNA. The main output result was the minimum free energy (ΔG Kcal/mol), and the lower the minimum free energy, the more stable the folding structure of mRNA.

## 3 Results

### 3.1 Pan-proteomics analysis of primary data

**Figure 1:**
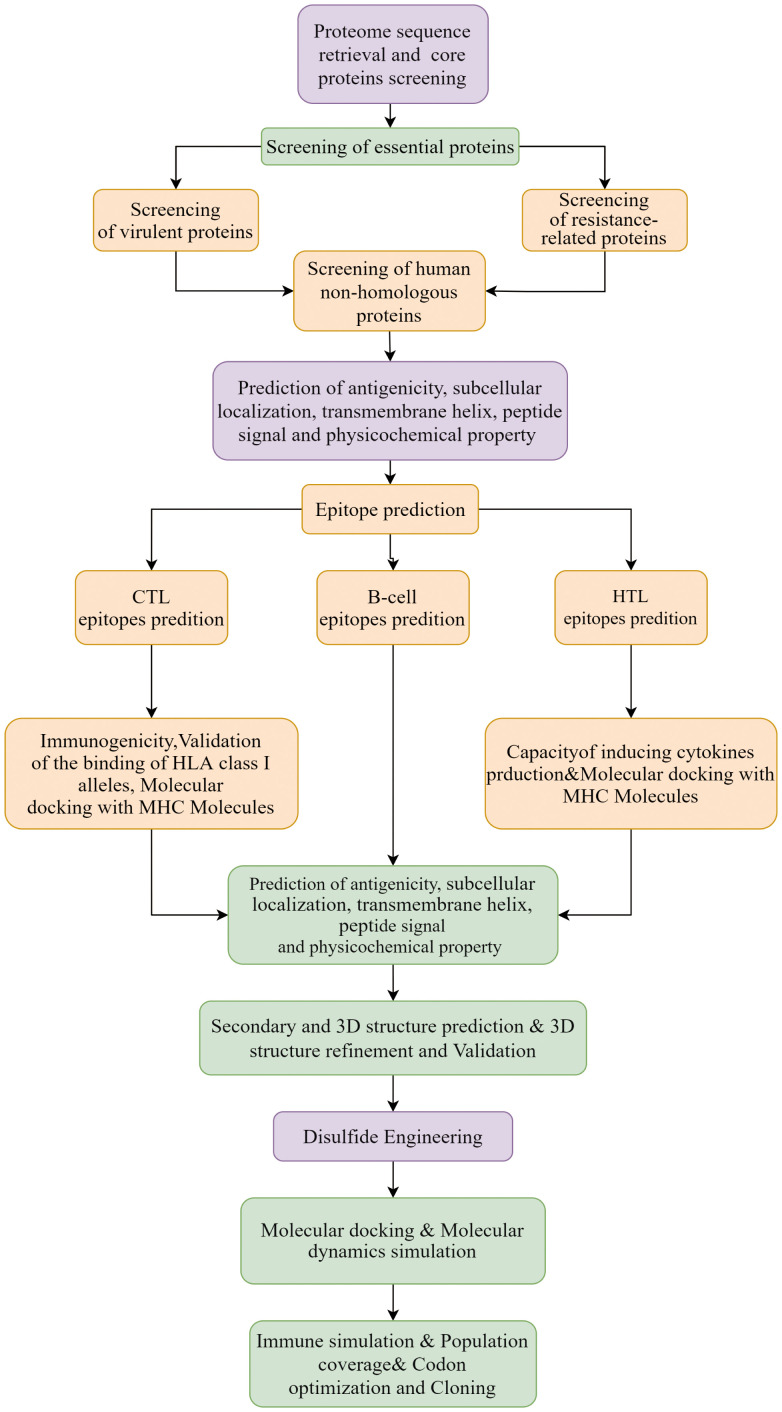
Workflow designed for 6 common *Nocardia* subspecies vaccines.

A total of 218 proteomes available for the 6 representative *Nocardia* subspecies were obtained from the NCBI database. The detailed information was shown in **Supplementary Data Sheet 1**. The results of pangenome analysis predicted by the BPGA tool revealed that 1,336 core proteins were found in the 218 proteomes (**Supplementary Data Sheet 2**). The flow chart for the design of a multi-epitope vaccine against 6 *Nocardia* subspecies was presented in **Figure 1**.

### 3.2 Selection of essential, virulence, resistance, and non-homologous proteins

The results of BLASTp indicated that there were 711 essential proteins out of the 1,336 core proteins (**Supplementary Data Sheet 3**), and there were 100 virulent proteins (**Supplementary Data Sheet 4**), 63 resistant proteins (**Supplementary Data Sheet 5**), and 28 proteins presenting with virulent and resistant functions among the 711 essential proteins (**Supplementary Data Sheet 6**). Only 76 non-homologous proteins remained after removing proteins with homology to the human proteome (**Supplementary Data Sheet 7**), of which 3 ideal proteins were identified after analyses of antigenicity, subcellular localization, transmembrane domains, signal sequence, allergenicity, toxicity, and physicochemical properties. From **Table 1**, it could be seen that all these 3 proteins were antigenic, non-allergic, non-toxic, non-homologous with human proteins, stable and of a suitable molecular weight. Also, all the 3 proteins were essential for bacteria, and two of them were associated with antibiotic resistance, and one with bacterial virulence. After sequence alignment with the NCBI database using BLASTp, it was further found that one of the 3 proteins was a transglycosylase domain-containing protein, one was a penicillin-binding protein, and the other was type VII secretion-associated serine protease myosin-like protein. The structures of the target proteins were shown in **Supplementary Data Sheet 8**; **Figures S1**–**S3**.

**Table 1:** The target protein from 6 common *Nocardia* species.

Protein	Protein name	Antigenicity		Localization		No of Residues	Mol.Wt.kDa	Instablity Index	Stablity	transmembrane helix	Signal	Allergic	Toxicity
		Vaxijen	ANTIGENpro	PSORTb	Cell-Ploc								
CORE_REP|Org125_Gene1111	transglycosylase domain-containing protein	0.7858	0.963922	Unknown	Cell membrane, Extracell	973	100.1	39.62	stable	1	0	–	–
CORE_REP|Org97_Gene925	penicillin-binding protein	0.52	0.943565	CytoplasmicMembrane	Cell membrane	831	87.01	38.03	stable	1	0	–	–
CORE_REP|Org5_Gene928	type VII secretion-associated serine protease mycosin	0.6548	0.640472	Extracellular	Extracell	503	50.57	35.71	stable	1	1	–	–

### 3.3 Epitopes screening

**Figure 2:**
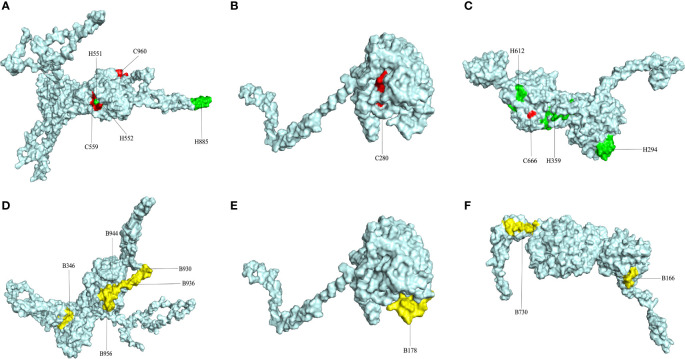
The plot of surface positions of all final selected epitopes. **(A)** The Cytotoxicity T Lymphocytes (CTL) and Helper T lymphocyte (HTL) epitopes of CORE_REP|Org125_Gene1111. **(B)** The CTL and HTL epitopes of CORE_REP|Org5_Gene928. **(C)** The CTL and HTL epitopes of CORE_REP|Org97_Gene925; **(D)** The B-cell epitopes of CORE_REP|Org125_Gene1111. **(E)** The B-cell epitopes of CORE_REP|Org5_Gene928. **(F)** The B-cell epitopes of CORE_REP|Org97_Gene925. Each CTL epitope is shown using a combination of C and its starting position. Each HTL epitope is shown using a combination of H and its starting position. Each B-cell epitope is shown using a combination of B and its starting position.

After the prediction of antigenicity, immunogenicity, toxicity, allergenicity, and the ability to induce the generation of INF-γ, IL-4 and IL-2. Then, we identified 4 ideal CTL epitopes from 356 epitopes (**Table 2**), 6 desirable HTL epitopes from 305 epitopes (**Table 3**), and 8 appropriate B cell epitopes from 61 epitopes for designing a multi-epitope vaccine (**Table 4**). All selected epitopes were located on the surface of the target protein and visualized by PyMOL v2.4 (**Figure 2**).

**Table 2:** The selected CTL epitopes for the vaccine construct.

Protein	Start	Peptide	Antigenicity	immunogenicity	Allergic	Toxicity	Tepitool		
							MHC I alllele	IC50(nM)	Rank%
Gene1111	959	STDGTGDGY	2.8514	0.14952	–	–	HLA-A*01:01	10.79	0.02
							HLA-A*30:02	482.39	0.69
	558	KSGAVRAYY	1.2303	0.17015	–	–	HLA-A*30:02	8.84	0.01
							HLA-B*58:01	65.32	0.25
							HLA-A*30:01	90.7	0.43
							HLA-C*16:01	98.36	0.3
							HLA-B*15:25	163.17	0.85
							HLA-B*57:01	181.13	0.27
							HLA-A*29:02	288	0.44
							HLA-B*15:01	306.85	0.84
							HLA-A*11:01	411.25	1.3
							HLA-C*03:02	489.86	0.91
Gene928	279	ISPAWFSPY	1.2337	0.21557	–	–	HLA-C*03:02	16.69	0.07
							HLA-B*15:25	23.66	0.14
							HLA-C*16:01	27.04	0.09
							HLA-C*12:03	44.51	0.1
							HLA-A*30:02	50.05	0.07
							HLA-C*14:02	58.29	0.22
							HLA-A*29:02	60.57	0.15
							HLA-B*15:01	62.86	0.23
							HLA-C*12:02	109.67	0.09
							HLA-B*15:02	189.88	0.22
							HLA-C*02:02	294.87	0.12
							HLA-C*02:09	294.87	0.12
Gene925	665	SMAGAAYIY	0.5453	0.19637	–	–	HLA-A*29:02	5.26	0.02
							HLA-B*15:25	7.5	0.03
							HLA-A*30:02	11.77	0.02
							HLA-B*15:02	16.58	0.02
							HLA-B*15:01	19.31	0.06
							HLA-B*35:01	72.23	0.13
							HLA-C*03:02	97.12	0.3
							HLA-C*14:02	210.44	0.53
							HLA-C*16:01	272.86	0.63
							HLA-A*11:01	340.63	1.1
							HLA-A*32:01	352.38	0.29
							HLA-A*03:01	436.2	0.86

**Table 3:** The screened HTL epitopes for the vaccine construct.

Protein	Start	MHCII alleles	Peptide	Rank_EL%	IC50(nM)	Antigenicity	Allergic	Toxicity	IFN-γ	IL-4	IL-2
Gene1111	885	DRB1_0101	KPSQVEILPGITIPV	0.35	16.11	0.7823	–	–	+	+	–
	551	DRB1_0301	TAVVSVDPKSGAVRA	0.02	65.27	1.2461	–	–	+	+	–
	552	DRB1_0301	AVVSVDPKSGAVRAY	0.1	71.21	1.1765	–	–	+	+	–
Gene925	294	DRB1_0405	DTLIQNIPSRADEFR	0.33	150.99	0.7800	–	–	+	+	–
	612	DRB1_0701	EPGLANTLANALSQD	0.12	58.97	0.5813	–	–	+	–	+
	359	DRB4_0101	GYLIRTTLDPAVQNS	0.28	33.33	0.5034	–	–	+	+	+

**Table 4:** The selected B cell epitopes for the vaccine construct.

Protein	Start	B cell epitope	Antigenicity	Allergic	Toxicity
Gene1111	956	GVGNSTDGTGDGYTNS	2.4246	–	–
	930	PADGSSPSTSNSGDTS	1.7579	–	–
	346	GSTITQQYVKNAMVGN	0.6965	–	–
	936	PSTSNSGDTSGNSRSQ	2.2641	–	–
	944	TSGNSRSQRPGAGVGN	2.1359	–	–
Gene928	178	LQYEAKNHRDDDTGKVAAGGYGD	1.3327	–	–
Gene925	731	RGSNNAQIPDVNGMSE	1.1028	–	–
	166	SGEVQQG	2.1954	–	–

### 3.4 Molecular docking between T cell epitopes and MHC molecules

**Figure 3:**
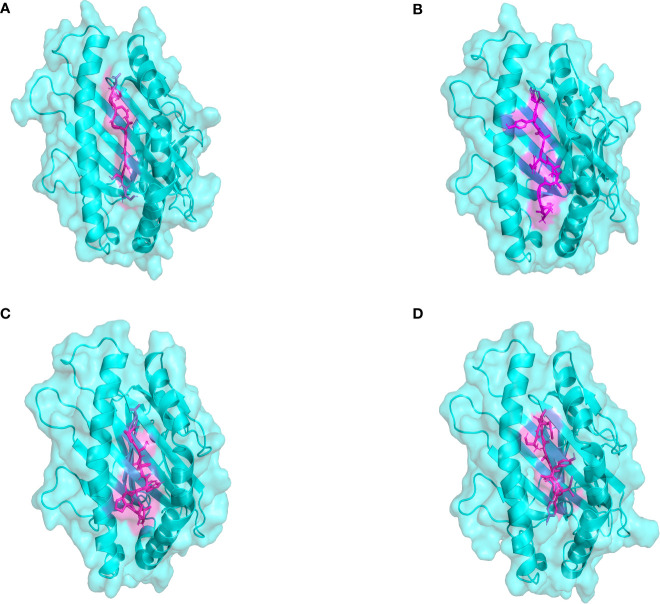
The plot of the binding patterns of CTL epitopes with MHC class I molecules. The MHC I allele molecules were presented in blue and the CTL epitope peptides were presented as magenta. **(A)** The STDGTGDGY epitope bind with HLA-A0101 **(B)** The KSGAVRAYY epitope bind with HLA-B5801 **(C)** The ISPAWFSPY epitope bind with HLA-B1502 **(D)** The AETASPERI epitope bind with HLA-B4402.

The docking results of Autodock Vina revealed that the binding affinity of CTL epitopes to MHC I alleles varied between -9.1 and -8.4 kcal/mol (**Figure 3**), whereas the binding affinity of HTL epitopes to MHC II alleles varied between -6.6 and -4.4 kcal/mol. According to relevant research, the binding energy is often used to determine the likelihood that a ligand will bind to a protein target, with a larger negative binding affinity value indicating a stronger interaction between the receptor and ligand. From the above, it was suggested that T cell epitopes had a good affinity for MHC allele molecules in this study. Furthermore, the predicted results of Ligplot+ v2.2 also indicated that there were strong interactions between T-cell epitopes and MHC allele molecules, as detailed in **Supplemental File 8 Tables S1, S2**. The quality verification results of MHC molecules modeled by the SWISS-MODEL server are shown in **Supplementary Data Sheet 8**; **Table S3**.

### 3.5 Vaccine construct and features

**Figure 4:**
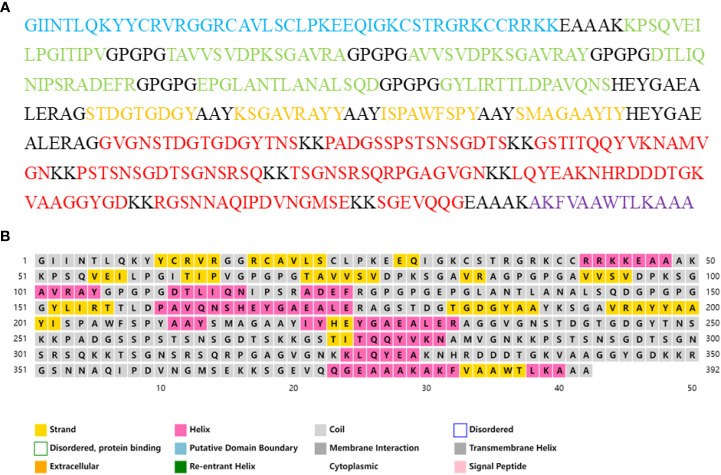
**(A)** The amino acid sequences of the vaccine construct. The adjuvant is colored blue, the HTL epitopes are colored green, and the CTL epitopes are colored yellow, The B-cell epitopes and the Pan HLA DR-binding epitope (PADRE) are colored red, all linkers are colored black. **(B)** The secondary structure of the vaccine construct.

The final vaccine sequence is shown in **Figure 4A**. According to the projected results of VaxiJen2.0 and ANTIGENpro, the antigenicity scores of the vaccine were 1.1966 and 0.9672, respectively. The results from the ToxinPred2 and AllerTOP v.2.0 servers revealed that the vaccine was non-toxic and non-allergic. Analysis of physicochemical characteristics revealed that the vaccine construct was composed of 392 amino acids, with a molecular weight of 40.30 kDa, and its theoretical pI was 9.47. The instability index of the vaccine was 26.07, indicating a stable nature. The aliphatic index of the vaccine was 55.64, suggesting that the vaccine was thermostable. The GRAVY value of the vaccine was -0.677, which demonstrated a hydrophilic nature. The developed vaccine had an estimated half-life of 30 hours in mammalian reticulocytes, over 20 hours in yeasts, and over 10 hours in *E. coli*. According to the prediction results of the SOLpro server, the probability of the vaccine being soluble when overexpressed in *E. coli* was 0.784691. Neither signal peptide nor transmembrane helix was detected in the vaccine construct, so there was no obvious difficulty in expressing the vaccine *in vivo* (**Supplementary Data Sheet 8**; **Figures S4**, **S5**).

### 3.6 Secondary and tertiary structure predictions, tertiary structure refinement and validatio

**Figure 5:**
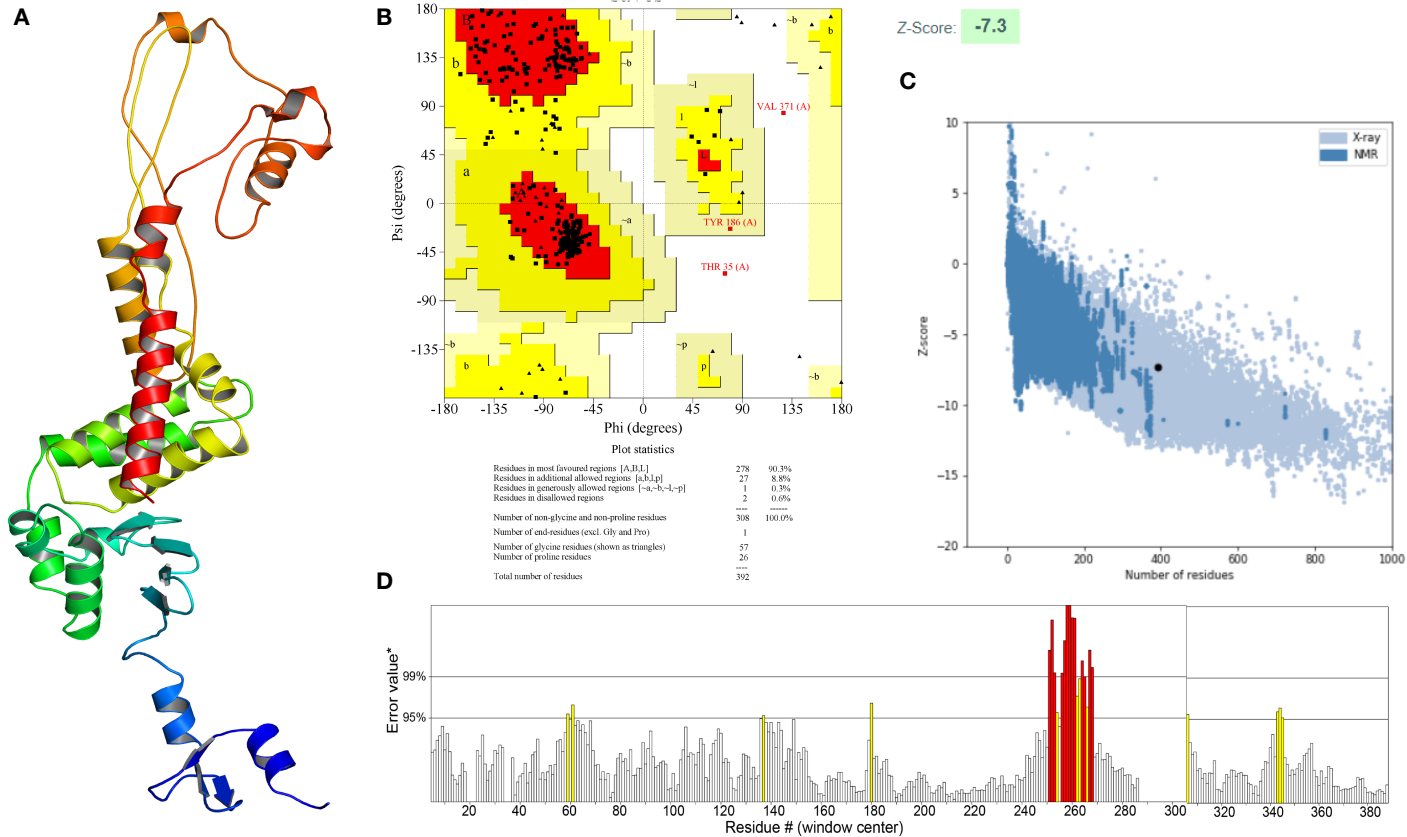
**(A)** Three-dimensional (3D) structure of the vaccine. **(B)** The Ramachandran plot of the refined 3D model generated by the PROCHECK server, the red-colored regions are the most favored regions, the dark yellow and light yellow regions are the additional allowed and generously allowed regions, the white regions are the disallowed regions. **(C)** The Z-score plot of the refined 3D model generated by the ProSA-web server. **(D)** The ERRAT score of the refined 3D model generated by the ERRAT server.

The secondary structure of the vaccine, as analyzed by the PSIPRED 4.0 server, was composed of 65.05% coil, 19.39% α-helix and 15.56% β-strand (**Figure 4B**). The Robetta server was employed to generate the primary 3D structure of the vaccine, which was further refined by GalaxyRefine(**Figure 5A**). The best refined vaccine model, with a MolProbity of 1.897, was validated by the PROCHECK, ERRAT, and ProSA-web servers. The Ramachandran plot generated by the PROCHECK server revealed that the vaccine had 90.3% residues in the most favored regions, 8.8% residues in additional allowed regions, and only 0.3% residues in generously allowed regions (**Figure 5B**). The ERRAT score of the vaccine was 93.017(**Figure 5D**), and the Z-score generated by the ProSA-web server was -7.3(**Figure 5C**). The PROCHECK server was employed to examine the stereochemistry of protein structures in detail. Over 90% of residues in the most favored regions suggested a good quality of the vaccine model. The ERRAT server was then utilized to distinguish correct and incorrect regions in the protein structure based on characteristic atomic interactions, and a high ERRAT score indicates a good quality of the structure. The Z score generated by the ProSA-web server was used to indicate the overall model quality, and the lower the Z score, the better the model quality. All of the above analysis results suggested that the developed vaccine had a reliable and stable 3D structure.

### 3.7 Disulfide engineering of vaccine

The inter- and intra-chain disulfide bonds were evaluated by the DbD2 server, which reported 52 pairs of amino acid residues with the ability to form disulfide bonds. Given that 90% of native disulfide bonds have an energy value< 2.2 kcal/mol, 2.2 kcal/mol was set as the threshold for screening disulfide bonds in this study. According to the energy value and chi3 value, only 3 residue pairs (i.e., LEU6-TYR9, GLU28-ARG43, and GLY234-ALA376) were chosen and they were not falling into any of the epitope regions, which could minimize the effect of disulfide engineering on vaccine immunogenicity (**Supplementary Data Sheet 8**; **Figure S6A**). Analyses of the Ramachandran plot, ERRAT score, and Z-score were shown in **Supplementary Data Sheet 8** (**Figure S6B–S6D**), which indicated that the model with disulfide was of good quality.

### 3.8 The analysis of molecular docking of vaccine-Toll like receptors

**Figure 6:**
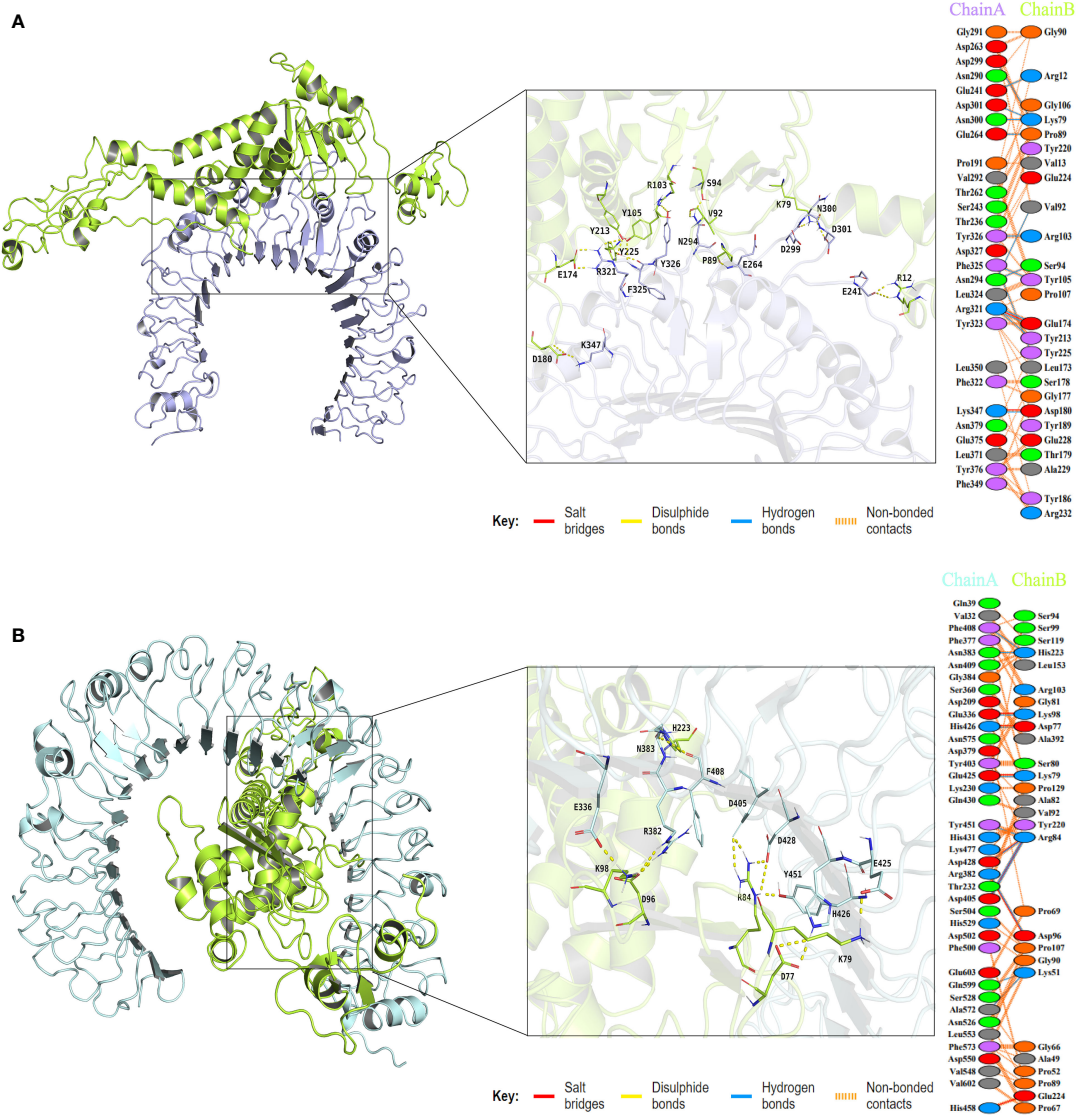
The plot of visualized analysis and the interacting amino acid residues of the docking complex. The TLR2 receptor is colored lightblue, the TLR4 receptor is colored palecyan, and the vaccine is colored limon. **(A)** The visualization of the vaccine and TLR2 complex and Interacting residues. The chain A refers to TLR2 receptor and the chain B refers to vaccine. **(B)** The visualization of the vaccine and TLR4 complex and Interacting residues. The chain A refers to TLR4 receptor and the chain B refers to vaccine.

To assess the binding affinity between the vaccine construct and TLRs (TLR2 and TLR4), molecular docking was performed using the Cluspro2.0 server. The server generated a total of 30 candidate models of the vaccine-TLR2 complex with different binding energies, from which the top-ranked model with the lowest binding energy score of -837.3 was selected for further refinement. Also, a total of 30 model complexes of vaccine-TLR4 were generated, and the top-ranked model with the lowest binding energy score of -835.1 was chosen. The HADDOCK 2.4 server was then used to refine the vaccine-receptor complex, and the HADDOCK scores of the vaccine-TLR2 complex and vaccine-TLR4 complex were -194.9 ± 1.0 and -243.4 ± 1.7, respectively. The low HADDOCK scores revealed the good binding between the vaccine and TLRs. The analysis results of the PDBsum server revealed that there were 14 hydrogen bonds and 2 salt bridges formed between the vaccine and TLR2, and 12 hydrogen bonds and 7 salt bridges formed between the vaccine and TLR4 (**Supplementary Data Sheet 8**; **Table S4**). For the vaccine-TLR2 complex, the amino acid residues of the vaccine involved in salt bridge formation were GLU174 and ASP180, and the residues involved in hydrogen bonds formation were ARG12, PRO89, VAL92, SER94, LYS79, TYR213, GLU174, TYR105, TYR225, ARG103, and ASP180 (**Figure 6A**). For the vaccine-TLR4 complex, the amino acid residues of the vaccine involved in salt bridge formation were LYS98, ASP96, ARG84, LYS79, ASP77, and GLU224, and the residues involved in hydrogen bonds formation were LYS98, ASP96, HIS223, ARG84, LYS79, ASP77, and LYS51 (**Figure 6B**). The above results suggested that there were strong interactions between the designed vaccine and TLRs (TLR2 and TLR4).

### 3.9 MD simulation

**Figure 7:**
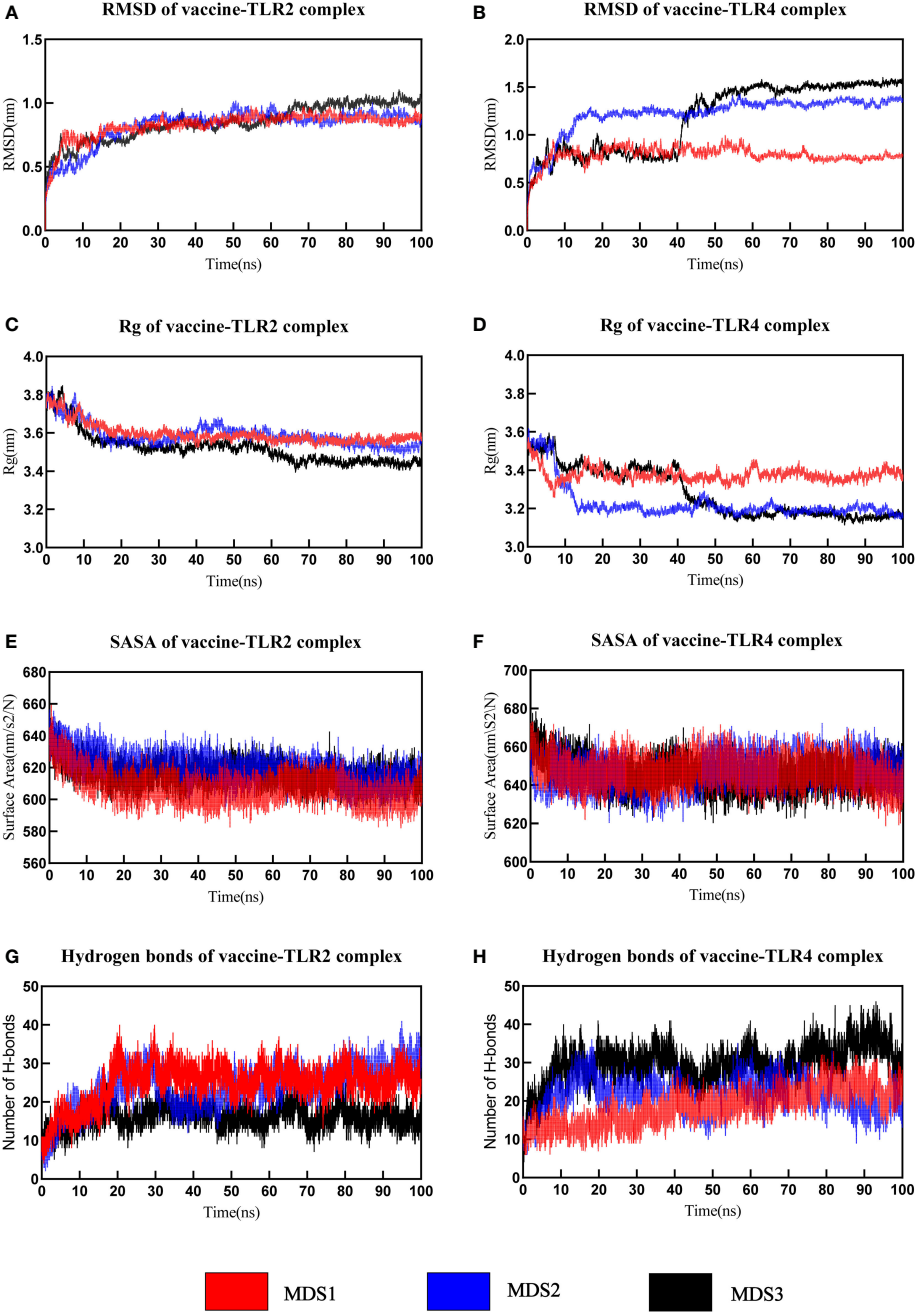
The analysis of molecular dynamics simulation of vaccine-TLRs. **(A, B)** RMSD (root mean square deviation) plots **(C, D)** Rg (Radius of gyration) **(E, F)** SASA(solvent accessible surface area) **(G, H)** Hydrogen bonds.

MD simulation was performed to understand the stability of vaccine-receptor complexes at the atomic level using Gromacs v2022.1. A primary analysis of trajectories was performed, including the calculation of the Backbone root-mean-square deviation (RMSD), the radius of gyration (Rg), root mean square fluctuation (RMSF), total solvent accessible surface area (SASA), and the number of hydrogen bonds. RMSD reflects the conformational fluctuations of vaccine-receptor complexes based on the initial structure. For the vaccine-TLR2 complex, the RMSD of the first and third simulations reached relative stability around 10 ns, but the RMSD of the second simulation reached stability around 15 ns (**Figure 7A**). Overall, the RMSD curves for the three simulations have similar trends. The mean RMSD values of the three simulations were 0.826 ± 0.101nm, 0.821 ± 0.139nm, and 0.850 ± 0.140nm, respectively. For the vaccine-TLR4 complex, the mean RMSD values of the three simulations were 0.791 ± 0.085 nm,1.223 ± 0.190 nm, and 1.182 ± 0.356 nm, respectively. In the first and second simulations, the RMSD reached equilibrium around 10ns and 20ns, respectively. In the third simulation, RMSD reached the first equilibrium around 10ns, fluctuated again at 40 ns, and reached the second equilibrium around 45 ns (**Figure 7B**). Rg characterizes the compactness of a protein structure. The Rg value of the vaccine-TLR2 complex decreased at first and then remained at a constant level at about 15ns in all three simulations (**Figure 7C**). For the vaccine-TLR4 complex, the Rg values of the second and third simulations were lower than those of the first simulation, indicating a more compact structure of the complex during the second and third simulations (**Figure 7D**). The analysis of Rg also showed that the vaccine-TLR4 complex underwent two conformational changes before reaching equilibrium in the third simulation (**Figure 7D**). Overall, the results of Rg indicated that the structures of both the vaccine-TLR2 complex and vaccine-TLR4 complex were compact and stable over time during the three MD simulations. The RMSF value reflects the flexibility of a single amino acid of a protein throughout the MD simulation. RMSF analysis revealed that the residues 380-392 of the vaccine chain and the residues 27-100 of TLR2 in the vaccine-TLR2 complex were highly flexible in all three simulations (**Supplementary Data Sheet 8**; **Figure S7A and S7C**). For the vaccine-TLR4 complex, it was found that the residues 250-360 of the vaccine chain were highly flexible in the first simulation, while the RMSF of the vaccine had lower values in the other two simulations. Residues 250-360 did not interact with the receptor and the vaccine structure is not compact enough resulting in high flexibility of the residues during the first simulation (**Supplementary Data Sheet 8; Figure S7B**). Meanwhile, residues 27-300 of the TLR4 had higher flexibility in the third simulation and the RMSF of TLR4 was relatively lower than that of the vaccine in the other two simulations (**Supplementary Data Sheet 8; Figure S7D**). The analysis of the total solvent accessible surface area showed the change in the complex surface area with respect to time. The curve trend of the SASA plot was opposite to that of RMSD indicating that the better interaction between receptor and ligand led to a lower surface area of the protein complexes (**Figures 7E, F**). The results of the analysis of SASA once again revealed the stable binding between the vaccine and TLRs (TLR2 and TLR4). The vaccine-TLR2 complex presented about 10 hydrogen bonds at the beginning of the simulation. After 20 seconds, the number of hydrogen bonds formed in the vaccine-TLR2 complex increased and then maintained relative stability in all three simulations (**Figure 7G**). During the three simulations, the number of hydrogen bonds of the vaccine-TLR4 complex increased from 10 at the beginning to about 25 hydrogen bonds at the end of the simulation (**Figure 7H**). The formation of hydrogen bonds suggested that the vaccine had a good affinity for TLRs. Finally, the movies of the MD simulations generated by VMD v1.93 were shown in the **Supplementary Data Sheet 9-14**.

### 3.10 MM-PBSA calculation

The tracks of 100ps extracted from each interval during the final 10ns simulation period were processed with the calculation of MM-PBSA and several binding interaction energies, including van der Waals, electrostatic, polar solubility, solvent accessible surface area (SASA), and binding energy (ΔG_binding_) of vaccine-receptor complexes. The binding energy of the vaccine-TLR2 complex was -161.07 kJ/mol, -208.24 kJ/mol, and -148.16 kJ/mol, respectively. For the vaccine-TLR4 complex, the binding energy were -203.16 kJ/mol, -176.99 kJ/mol, and -240.85 kJ/mol in three simulations, respectively. The detailed energy analysis presented in **Table 5** revealed that the vaccine had a good affinity for both TLR2 and TLR4 molecules. For the vaccine-TLR2 complex, residues (LYS79, ALA92, and ARG232) in the vaccine chain contribute significantly to vaccine binding to the TLR2 receptor, particularly residues (ARG232) (**Supplementary Data Sheet 8; Figure S8A, S8C and S8E**). For the vaccine-TLR4 complex, MM/PBSA decomposition analysis showed that residues (PRO69, LYS77, ARG84, PRO89, SER99, TYR105 and LYS389) in the vaccine chain contributed to the binding of the vaccine to the TLR4, with a greater contribution from the residue (ARG84) (**Supplementary Data Sheet 8; Figures S8B, S8D, S8F**). However, the residue (GLU224) negatively affected the binding of the vaccine to TLR2 and TLR4 receptors. Overall, the results of all MM-PBSA calculations indicated that the vaccine can stably bind to the TLR2 and TLR4.

**Table 5:** The results of binding free energy, estimated by MMPBSA for the vaccine-TLRs complex.

Complex	Molecular dynamics simulation	van der Waal energy (kJ/mol)	Electrostatic energy (kJ/mol)	Polar solvation energy (kJ/mol)	Solvent accessible surface area energy (kJ/mol)	Binding energy (ΔGbinding) (kJ/mol)
vaccine-TLR2	MDS1	-271.97 (11.34)	-1871.02 (98.55)	2013.21 (92.74)	-31.29 (1.04)	-161.07 (15.29)
	MDS2	-215.32 (9.69)	-1634.28 (71.58)	1665.53 (65.57)	-24.17 (0.73)	-208.24 (15.68)
	MDS3	-216.43 (10.92)	-1775.62 (72.26)	1868.89 (70.43)	-24.99 (0.96)	-148.16 (12.45)
vaccine-TLR4	MDS1	-244.11 (11.39)	-3138.56 (77.46)	3206.89 (76.91)	-27.38 (0.90)	-203.16 (14.52)
	MDS2	-210.10 (11.81)	-3400.95 (93.34)	3460.52 (93.45)	-26.45 (1.54)	-176.99 (14.30)
	MDS3	-257.86 (12.71)	-3850.23 (182.20)	3899.90 (168.15)	-32.65 (0.94)	-240.85 (22.88)

### 3.11 PCA and DCC analysis

**Figure 8:**
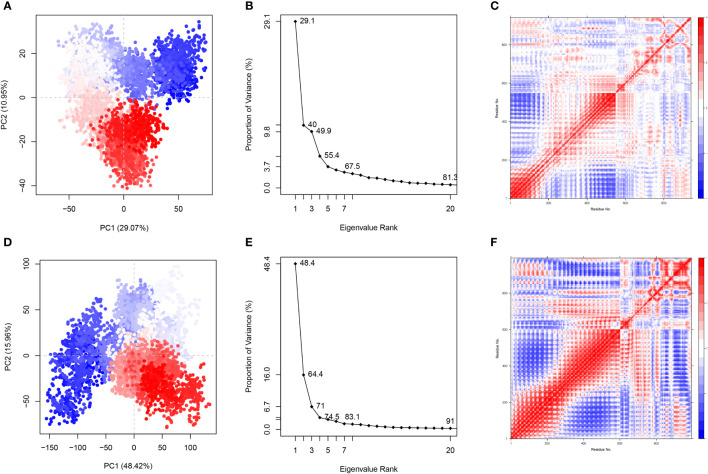
Principal component analysis(PCA) and dynamical cross-correlation (DCC) analysis for first MD simulation. **(A)** The plot of PC1 and PC2 about vaccine-TLR2 complex **(B)** The plot of PCA analysis about vaccine-TLR2 complex **(C)** The DCCM plot of vaccine-TLR2 complex **(D)** The plot of PC1 and PC2 about vaccine-TLR4 complex **(E)** The plot of PCA analysis about vaccine-TLR4 complex **(F)** The DCCM plot of vaccine-TLR4 complex.

To study and evaluate the association between different protein conformations, PCA was conducted for the trajectories of all C atoms in 50-100ns in the simulated system. PCA translates atomic coordinates into a limited number of PCs (commonly 2 or 3 PCs) that signify the directions in which the structure set has the greatest collective variability. The PCA scatter plots revealed that the color of instantaneous conformations changed from blue to red over time. In the direction of PC1, the successive color shifts (from blue to white to red) indicated that the conformation in both systems gradually evolved to another state during the simulation. PC1 and PC2 characterized 40.02%, 45.89%, and 49.24% of the system’s motility with respect to the vaccine-TLR2 complex (**Figure 8A**; **Supplementary Data Sheet 8; Figure S9A, S9C**) and 64.38%, 50.86% and 47.68% of the system’s motility with respect to the vaccine-TLR4 complex (**Figure 8D**; **Supplementary Data Sheet 8; Figures S9E, S9G**) in three MD simulations, respectively. Over 80% of the motion of both systems in 20 dimensions could be explained (**Figure 8B, E**; **Supplementary Data Sheet 8; Figures S9B, S9D, S9F, S9H**).

The dynamical cross-correlation matrix (DCCM) was utilized to display the information about interchain and intrachain residue-residue contacts and motions. **Figures 8C**, **G** and **Supplementary Data Sheet 8**; **Figure S10** depict the DCCM matrices of the vaccine-TLR2 and vaccine-TLR4 complexes. The color gradients from blue to white to red in the figures correspond to the correlation coefficients of -1, 0 and 1, respectively, and a deeper shade of the same color represents a stronger correlation. For example, the correlation coefficient of light blue is -0.5 and that of light red is 0.5. DCC analysis of the vaccine-TLR2 complex in three MD simulations showed that the residues surrounding 1 to 51 (550 to 600 in a plot) and 151 to 231 (700 to 780 in the plot) from the vaccine were positively correlated with the residues surrounding 200 to 548 (200 to 548 in a plot) and 300 to 400 (300 to 400 in the plot) from the TLR2 chain, respectively. (**Figure 8C**; **Supplementary Data Sheet 8 and Figure S10A and S10B**). For the vaccine-TLR4 complex, the residues surrounding 9 to 79 (610 to 680 in the plot) and 99 to 199 (700 to 800 in the plot) were positively correlated with the residues surrounding 500 to 601(500 to 601 in the plot) and 200 to 400 (200 to 400 in the plot) from the TLR4 chain, respectively (**Figure 8F**; **Supplementary Data Sheet 8 and Figure S10C and S10D**).

### 3.12 Immune simulation and population coverage

**Figure 9:**
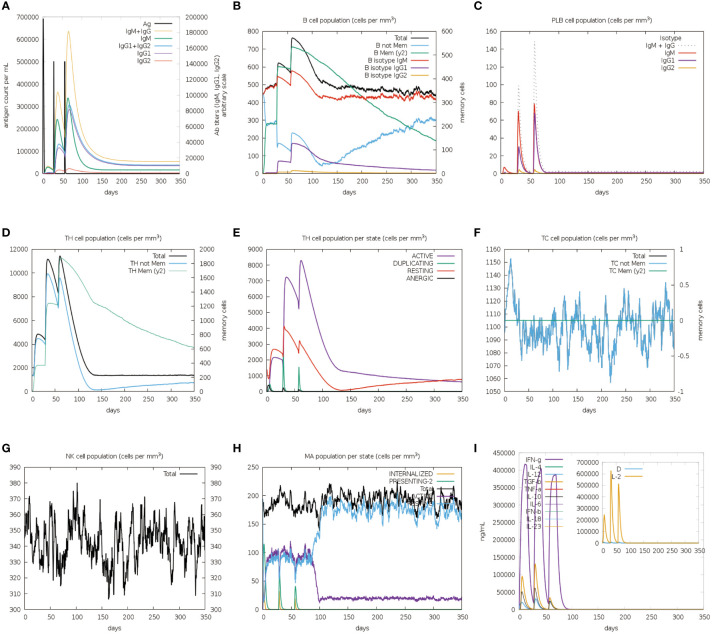
*In silico* immune simulation spectrum. **(A)** Changes in antibody titers after three vaccine injections; **(B)** Changes in B cell population after three vaccine injections; **(C)** Changes in plasma B-cell population after three vaccine injections; **(D)** Changes in helper T cell population after three vaccine injections; **(E)** Changes in helper T cell population per state after three vaccine injections; **(F)** Changes in cytotoxic T cell population per state after three vaccine injections; **(G)** Changes in natural killer (NK) cell population after three vaccine injections; **(H)** Changes in macrophages (MA) population per state after three vaccine injections **(I)**.

The immune stimulation of the vaccine was carried out using the C-ImmSim web server, which predicts whether the vaccine can induce significant adaptive immunity in the human body. Following the first injection, lower lgM antibody titers, a B-cell isotype lgM population, and a slight B-cell isotype lgG population were detected. After the second and third injections, higher levels of IgM antibody with IgG1, IgG1+IgG2 and lgG+lgM as well as a substantial number of memory B cells were found (**Figures 9A, B**). In addition to the B-cell, the plasma B cells, the total, memory and active TH-cell (helper T cell) populations also increased during the secondary and tertiary immune responses (**Figures 9B-E**). Besides, TC cells (cytotoxic T lymphocytes) were observed during the immune simulation, with the maximum TC cell count exceeding 1,150 cells per mm^3^ (**Figure 9F**). Further, the NK cells and macrophages were activated after exposure to the vaccine (**Figures 9G, H**). **Figure 9I** showed that the vaccine was able to activate a wide range of cytokines, including IFN-γ, IL-2, and IL-4. In summary, the designed vaccine was capable of inducing the generation of a large number of immune cells and a variety of cytokines, indicating that it can successfully induce adaptive immunity and memory immunity in the host body.

Population coverage analysis suggested that 89% of the world’s population might respond to the designed vaccine. More specifically, its population coverage in Europe, the United States, and China reached 93.47%, 88.91%, and 86.63% (**Supplementary Data Sheet 8; Table S5**), respectively. The results of immunization simulation and population coverage demonstrated that the developed vaccine was highly immunogenic and able to protect the vast majority of the global population.

### 3.13 Codon optimization and cloning

**Figure 10:**
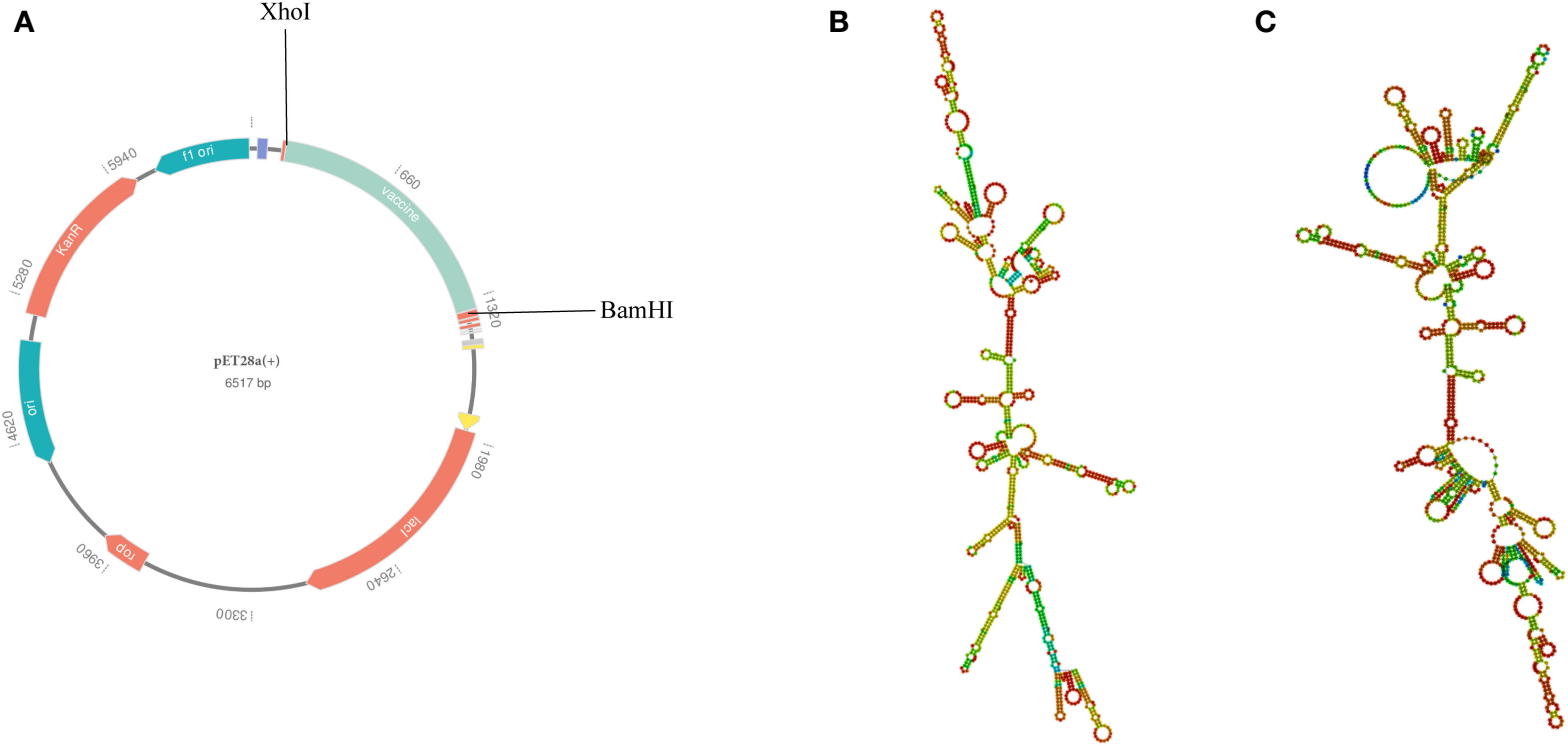
**(A)** In silico cloning of the multi-epitope vaccine into the pET28a (+) expression vector; the light green part presents the vaccine’s codon. **(B)** The best secondary structure of the vaccine mRNA. **(C)** The centroid secondary structure of the vaccine mRNA.

To ensure high-level expression and easy production of the vaccine, codon optimization was performed *via* the VectorBuilder server. The cDNA length of the optimized vaccine was 1,176 bps and a stop codon was added to the Terminal of cDNA (**Supplementary File 8**). Both the CAI value of the vaccine (i.e., 0.89) and the GC content (i.e., 59.46%) were desirable, indicating a high gene expression potential and excellent expression ability in *E. coli* (K12 strain). Then, the GenSmart tool was used to clone the cDNA sequence of the vaccine into the pET28a(+) vector (**Figure 10A**).

### 3.14 Secondary structure of vaccine mRNA

According to the RNAfold server, the best secondary structure of the vaccine mRNA had a minimum free energy of -474.30 kcal/mol (**Figure 10B**), whereas the centroid secondary structure had a minimum free energy of -407.80 kcal/mol (**Figure 10C**). The minimum free energy of the optimal secondary structure of the vaccine mRNA was determined to be -413.80 kcal/mol by the mFold v2.3 server (**Supplementary Data Sheet 8; Figure S11**). Because mRNA is more stable when its minimal free energy is lower, the vaccine was predicted to be stable after expression *in vivo*.

## 4 Discussion

So far, *Nocardia* is still a significant threat to aquaculture animals and immunocompromised people, such as the elderly, transplant recipients, HIV-infected individuals, malignant tumor patients, and patients treated with glucocorticoids (4, 95–97). Moreover, *Nocardia* infections have also been found in people with normal immune function (98, 99). Due to the elevated incidence and fatality rate of nocardiosis, and increased *Nocardia* resistance to existing antibiotics, the need of developing a vaccine to mitigate *Nocardia* infections has been underscored.

Because there is a large number of *Nocardia* species and many of them are pathogenic to humans, our goal was to develop a broad-spectrum multi-epitope vaccine that can protect against the majority of *Nocardia*, but it is a huge challenge for traditional vaccinology. Compared with traditional techniques, RV is capable of performing batch analysis of thousands of biological parameters within a shorter time while ensuring more reliable results, thereby opening up a novel door to the research and development of vaccines (23, 24). To enable the designed vaccine to take effect on most pathogenic *Nocardia* strains, 218 complete proteome sequences of 6 common *Nocardia* species were downloaded from NCBI, and the proteins containing 50% similar sequences from all strains were included for further analysis. Then, less significant proteins, such as the human proteome, essential genes, virulent factor proteins, antibiotic-resistant proteins, and human interacting proteins, were subtracted from several other databases. Besides recognizing the virulent and antibiotic-resistant factors, the identification of potential vaccine candidates must also focus on checking their antigenicity, allergenicity, subcellular localization, and other physicochemical properties, such as the aliphatic index, GRAVY, and instability index. Finally, 3 outer membrane proteins, including one virulence factor protein and two antibiotic resistance proteins, were selected for the identification of epitopes.

Epitope selection is a critical step in the development of multi-epitope vaccines. In this study, the antigenicity, toxicity, and allergenicity of all predicted epitopes were evaluated, and both the T-cell (CTL and HTL) and B-cell epitopes were included in the multi-epitope construction, which can avoid unsatisfactory immune effects due to insufficient immune response that is induced by T-cell epitopes or B-cell epitopes alone while ensuring safety. It is well known that CTL cells can only recognize the antigens presented by MHC class I molecules, while HTL can only recognize the antigens presented by MHC class II molecules (100). Therefore, the capacity of the epitope to bind to HLA alleles is a crucial predictor of whether it can effectively induce an immune response or not. It is worth mentioning that we performed molecular docking between all ultimately selected T-cell epitopes and HLA alleles, and the docking results suggested that the affinity between them was strong, indicating that the epitopes could be effectively presented *via* MHC molecules. IL-2 is indispensable for the proliferation as well as differentiation of naive T cells into effector T cells (58). Both IL-4 and INF-γ are important activators of T helper cells, and IFN-γ also participates in the activation of macrophages (56, 57). The potential of HTL epitopes to induce the IL-2, IL-4, and INF-γ generation was examined in this study. With all that mentioned, the eventually-selected epitope has the potential to construct an effective multi-epitope vaccine that is characterized by few side effects.

Adjuvants can not only improve the immunogenicity of multi-epitope vaccines but also help induce a more durable immune response (101). In this study, the β-defensin, which can attenuate toxicity and enhance systemic antibody responses when administered together with antigens, was incorporated into the vaccine construct as an adjuvant (102). It is well known that TLRs can recognize different PAMPs and play an indispensable role in the innate and adaptive immune response (77). The binding affinity with TLRs is an important reference factor in evaluating the immune effect of a multi-epitope vaccine candidate. From the docking results, we observed stable interactions and high affinity between the multi-epitope vaccine and TLRs (TLR4 and TLR2), indicating that the vaccine candidate can effectively trigger the body’s immune response. Furthermore, the docked complexes were molecularly simulated to assess the stability and agility of the vaccine-TLR complexes in a simulated biological environment. In the three MD simulations of the vaccine-TLR2 complex, the RMSDs all remained stable in the last 80% of the simulation period, showing that the structure of the vaccine-TLR2 complex was relatively stable. For the trend of RMSD of vaccine-TLR4 complex, although there were differences in the three simulations, RMSDs all reached equilibrium at 50ns in the three simulations. The Rg values of two vaccine-TLR complex systems also tended to stabilize after 50 ns, which further confirmed the stability of the complex. In all MD simulations, there were quite negative correlations between RMSD and SASA, which verifies the good binding between vaccine and receptor (TLR2 and TLR4) on the other hand. In all MD simulations, more hydrogen bonds were formed in both the vaccine-TLR2 and vaccine-TLR4 complexes, demonstrating high affinity of the vaccine chain to the TLR2 and TL42 chains.

The MM-PBSA analysis indicated that very little energy was required to keep the two complexes stable. The DCC analysis revealed the existence of positively correlated contacts between vaccine-TLR2 amino acid residues and vaccine-TLR4 amino acid residues, indicating that the associations between the vaccine and TLRs (TLR2 and TLR4) were good. According to the above analysis, the designed vaccine is able to firmly bind to TLRs and successfully transport into the body in the natural environment, thereby inducing effective immunity. In the process of immune simulation, we observed the proliferation of TC, TH and B cells, and the production of lgA, lgG, INF- γ, IL-2 and other cytokines with the injection of the vaccine. In addition, memory B cells were also observed following repeated exposures to the vaccine, which suggested that the vaccine was capable of inducing not only cellular and humoral immunity but also long-term immunity against *Nocardia*. Finally, to evaluate the feasibility of mass production, codon optimization and cloning were implemented for the vaccine construct, and the values of CAI and GC contents suggested that the designed vaccine could be well expressed in the E. coil K12 strain. Besides, the lower minimum free energy scores of the secondary structure of vaccine mRNA suggested that the vaccine was expected to behave stable in the body.

## 5 Conclusion

Nocardiosis poses a major public health threat due to the expansion of susceptible populations, underdiagnosis and lack of adequate awareness. In this study, we designed an antigenic, non-allergic and non-toxic multi-epitope vaccine against *Nocardia* using RV and immunoinformatics techniques. The results of molecular docking and MD simulation show that the designed multi-epitope vaccine has a strong affinity for different innate immune receptors of the host, and that the vaccine-receptor complex is dynamically stable in the natural environment. In addition, the designed vaccine has the potential to induce humoral immunity and immunity. Finally, the codon optimization and cloned analysis show that the vaccine is feasible for mass production. To sum up, the designed vaccine may have a good protective effect on *Nocardia*, but the proposed candidate vaccine needs to be tested both *in vivo* and *in vitro* to determine its efficacy and safety.

## Data availability statement

The datasets presented in this study can be found in online repositories. The names of the repository/repositories and accession number(s) can be found in the article/**Supplementary Material**.

## Author contributions

Conceptualization: FZ. Methodology: FZ. Validation: SM and HY. Formal analysis: PZ, WP, MR and YC. Writing—original draft preparation: FZ and CT. Writing—review and editing: JC and CL. Supervision, PP and JC. Project administration, PP and JC. Funding acquisition, PP. All authors contributed to the article and approved the submitted version.

## References

[1] Brown-Elliott BA Brown JM Conville PS Wallace RJ Jr. Clinical and laboratory features of the nocardia spp. based on current molecular taxonomy. Clin Microbiol Rev (2006) 19(2):259–82. doi: 10.1128/cmr.19.2.259-282.2006 PMC1471991 16614249

[2] Conville PS Brown-Elliott BA Smith T Zelazny AM. The complexities of nocardia taxonomy and identification. J Clin Microbiol (2018) 56(1):20171226. doi: 10.1128/jcm.01419-17 PMC5744224 29118169

[3] Hamdi AM Fida M Deml SM Abu Saleh OM Wengenack NL. Retrospective analysis of antimicrobial susceptibility profiles of nocardia species from a tertiary hospital and reference laboratory, 2011 to 2017. Antimicrob Agents Chemother (2020) 64(3):20200221. doi: 10.1128/aac.01868-19 PMC7038297 31818815

[4] Wang H Zhu Y Cui Q Wu W Li G Chen D. Epidemiology and antimicrobial resistance profiles of the nocardia species in China, 2009 to 2021. Microbiol Spectr (2022) 10(2):e0156021. doi: 10.1128/spectrum.01560-21 35234511 PMC9045199

[5] Yu S Wang J Fang Q Zhang J Yan F. Specific clinical manifestations of nocardia: A case report and literature review. Exp Ther Med (2016) 12(4):2021–6. doi: 10.3892/etm.2016.3571 PMC5038476 27698688

[6] Baldawa S Nayak N Kukreja S D'Souza D Diyora B Sharma A. Cerebral nocardiosis. Asian J Neurosurg (2014) 9(4):245. doi: 10.4103/1793-5482.146661 25685238 PMC4323985

[7] Takamatsu A Yaguchi T Tagashira Y Watanabe A Honda H. Nocardiosis in Japan: A multicentric retrospective cohort study. Antimicrob Agents Chemother (2022) 66(2):e0189021. doi: 10.1128/aac.01890-21 34902263 PMC8942482

[8] Davis-Scibienski C Beaman BL. Interaction of nocardia asteroides with rabbit alveolar macrophages: Association of virulence, viability, ultrastructural damage, and phagosome-lysosome fusion. Infect Immun (1980) 28(2):610–9. doi: 10.1128/iai.28.2.610-619.1980 PMC550977 6995313

[9] Beaman BL Beaman L. Nocardia species: Host-parasite relationships. Clin Microbiol Rev (1994) 7(2):213–64. doi: 10.1128/cmr.7.2.213 PMC358319 8055469

[10] Poonyagariyagorn HK Gershman A Avery R Minai O Blazey H Asamoto K. Challenges in the diagnosis and management of nocardia infections in lung transplant recipients. Transpl Infect Dis (2008) 10(6):403–8. doi: 10.1111/j.1399-3062.2008.00338.x 18823356

[11] Lebeaux D Freund R van Delden C Guillot H Marbus SD Matignon M. Outcome and treatment of nocardiosis after solid organ transplantation: New insights from a European study. Clin Infect Dis (2017) 64(10):1396–405. doi: 10.1093/cid/cix124 PMC10941331 28329348

[12] Baio PV Ramos JN dos Santos LS Soriano MF Ladeira EM Souza MC. Molecular identification of nocardia isolates from clinical samples and an overview of human nocardiosis in Brazil. PloS Negl Trop Dis (2013) 7(12):e2573. doi: 10.1371/journal.pntd.0002573 24340116 PMC3854972

[13] Yasuike M Nishiki I Iwasaki Y Nakamura Y Fujiwara A Shimahara Y. Analysis of the complete genome sequence of nocardia seriolae Utf1, the causative agent of fish nocardiosis: The first reference genome sequence of the fish pathogenic nocardia species. PloS One (2017) 12(3):e0173198. doi: 10.1371/journal.pone.0173198 28257489 PMC5336288

[14] Mehta H Weng J Prater A Elworth RAL Han X Shamoo Y. Pathogenic nocardia cyriacigeorgica and nocardia Nova evolve to resist trimethoprim-sulfamethoxazole by both expected and unexpected pathways. Antimicrob Agents Chemother (2018) 62(7):e00364-18. doi: 10.1128/aac.00364-18 29686152 PMC6021631

[15] Zhao P Zhang X Du P Li G Li L Li Z. Susceptibility profiles of nocardia spp. to antimicrobial and antituberculotic agents detected by a microplate alamar blue assay. Sci Rep (2017) 7:43660. doi: 10.1038/srep43660 28252662 PMC5333629

[16] Muñoz J Mirelis B Aragón LM Gutiérrez N Sánchez F Español M. Clinical and microbiological features of nocardiosis 1997-2003. J Med Microbiol (2007) 56(Pt 4):545–50. doi: 10.1099/jmm.0.46774-0 17374898

[17] Wang HL Seo YH LaSala PR Tarrand JJ Han XY. Nocardiosis in 132 patients with cancer: Microbiological and clinical analyses. Am J Clin Pathol (2014) 142(4):513–23. doi: 10.1309/ajcpw84aftuwmhyu 25239419

[18] Wilson JW. Nocardiosis: Updates and clinical overview. Mayo Clin Proc (2012) 87(4):403–7. doi: 10.1016/j.mayocp.2011.11.016 PMC3498414 22469352

[19] Uhde KB Pathak S McCullum I Jr. Jannat-Khah DP Shadomy SV Dykewicz CA. Antimicrobial-resistant nocardia isolates, united states, 1995-2004. Clin Infect Dis (2010) 51(12):1445–8. doi: 10.1086/657399 21058914

[20] Tremblay J Thibert L Alarie I Valiquette L Pépin J. Nocardiosis in Quebec, Canada, 1988-2008. Clin Microbiol Infect (2011) 17(5):690–6. doi: 10.1111/j.1469-0691.2010.03306.x 20636427

[21] Nabel GJ. Hiv vaccine strategies. Vaccine (2002) 20(15):1945–7. doi: 10.1016/s0264-410x(02)00074-9 11983251

[22] Moxon R Reche PA Rappuoli R. Editorial: Reverse vaccinology. Front Immunol (2019) 10:2776. doi: 10.3389/fimmu.2019.02776 31849959 PMC6901788

[23] Monterrubio-López GP González YMJA Ribas-Aparicio RM. Identification of novel potential vaccine candidates against tuberculosis based on reverse vaccinology. BioMed Res Int (2015) 2015:483150. doi: 10.1155/2015/483150 25961021 PMC4413515

[24] Naz A Obaid A Shahid F Dar HA Naz K Ullah N. Chapter 16 - reverse vaccinology and drug target identification through pan-genomics. In: Barh D Soares S Tiwari S Azevedo V, editors. Pan-genomics: Applications, challenges, and future prospects, (Elsevier Science and Technology: Academic Press) (2020). p. 317–33. doi: 10.1016/C2018-0-00570-8

[25] Li M Zhu Y Niu C Xie X Haimiti G Guo W. Design of a multi-epitope vaccine candidate against brucella melitensis. Sci Rep (2022) 12(1):10146. doi: 10.1038/s41598-022-14427-z 35710873 PMC9202987

[26] Chiang MH Sung WC Lien SP Chen YZ Lo AF Huang JH. Identification of novel vaccine candidates against acinetobacter baumannii using reverse vaccinology. Hum Vaccin Immunother (2015) 11(4):1065–73. doi: 10.1080/21645515.2015.1010910 PMC4514290 25751377

[27] Chaudhari NM Gupta VK Dutta C. Bpga- an ultra-fast pan-genome analysis pipeline. Sci Rep (2016) 6:24373. doi: 10.1038/srep24373 27071527 PMC4829868

[28] Edgar RC. Search and clustering orders of magnitude faster than blast. Bioinformatics (2010) 26(19):2460–1. doi: 10.1093/bioinformatics/btq461 20709691

[29] Camacho C Coulouris G Avagyan V Ma N Papadopoulos J Bealer K. Blast+: Architecture and applications. BMC Bioinf (2009) 10:421. doi: 10.1186/1471-2105-10-421 PMC2803857 20003500

[30] Zhang R Ou HY Zhang CT. Deg: A database of essential genes. Nucleic Acids Res (2004) 32(Database issue):D271–2. doi: 10.1093/nar/gkh024 PMC308758 14681410

[31] Chen L Yang J Yu J Yao Z Sun L Shen Y. Vfdb: A reference database for bacterial virulence factors. Nucleic Acids Res (2005) 33(Database issue):D325–8. doi: 10.1093/nar/gki008 PMC539962 15608208

[32] Alcock BP Raphenya AR Lau TTY Tsang KK Bouchard M Edalatmand A. Card 2020: Antibiotic resistome surveillance with the comprehensive antibiotic resistance database. Nucleic Acids Res (2020) 48(D1):D517–d25. doi: 10.1093/nar/gkz935 PMC7145624 31665441

[33] Doytchinova IA Flower DR. Vaxijen: A server for prediction of protective antigens, tumour antigens and subunit vaccines. BMC Bioinf (2007) 8:4. doi: 10.1186/1471-2105-8-4 PMC1780059 17207271

[34] Magnan CN Zeller M Kayala MA Vigil A Randall A Felgner PL. High-throughput prediction of protein antigenicity using protein microarray data. Bioinformatics (2010) 26(23):2936–43. doi: 10.1093/bioinformatics/btq551 PMC2982151 20934990

[35] Yu NY Wagner JR Laird MR Melli G Rey S Lo R. Psortb 3.0: Improved protein subcellular localization prediction with refined localization subcategories and predictive capabilities for all prokaryotes. Bioinformatics (2010) 26(13):1608–15. doi: 10.1093/bioinformatics/btq249 PMC2887053 20472543

[36] Chou KC Shen HB. Cell-ploc: A package of web servers for predicting subcellular localization of proteins in various organisms. Nat Protoc (2008) 3(2):153–62. doi: 10.1038/nprot.2007.494 18274516

[37] Hallgren J Tsirigos KD Pedersen MD Almagro Armenteros JJ Marcatili P Nielsen H. Deeptmhmm predicts alpha and beta transmembrane proteins using deep neural networks. bioRxiv (2022). doi: 10.1101/2022.04.08.487609

[38] Teufel F Almagro Armenteros JJ Johansen AR Gíslason MH Pihl SI Tsirigos KD. Signalp 6.0 predicts all five types of signal peptides using protein language models. Nat Biotechnol (2022) 40(7):1023–5. doi: 10.1038/s41587-021-01156-3 PMC9287161 34980915

[39] Wilkins MR Gasteiger E Bairoch A Sanchez JC Williams KL Appel RD. Protein identification and analysis tools in the expasy server. Methods Mol Biol (1999) 112:531–52. doi: 10.1385/1-59259-584-7:531 10027275

[40] Sharma N Naorem LD Jain S Raghava GPS. Toxinpred2: An improved method for predicting toxicity of proteins. Brief Bioinform (2022) 23(5):bbac174. doi: 10.1093/bib/bbac174 35595541

[41] Dimitrov I Bangov I Flower DR Doytchinova I. Allertop V.2–a server for in silico prediction of allergens. J Mol Model (2014) 20(6):2278. doi: 10.1007/s00894-014-2278-5 24878803

[42] Kim DE Chivian D Baker D. Protein structure prediction and analysis using the robetta server. Nucleic Acids Res (2004) 32(Web Server issue):W526–31. doi: 10.1093/nar/gkh468 PMC441606 15215442

[43] Morris AL MacArthur MW Hutchinson EG Thornton JM. Stereochemical quality of protein structure coordinates. Proteins (1992) 12(4):345–64. doi: 10.1002/prot.340120407 1579569

[44] Colovos C Yeates TO. Verification of protein structures: Patterns of nonbonded atomic interactions. Protein Sci (1993) 2(9):1511–9. doi: 10.1002/pro.5560020916 PMC2142462 8401235

[45] Wiederstein M Sippl MJ. Prosa-web: Interactive web service for the recognition of errors in three-dimensional structures of proteins. Nucleic Acids Res (2007) 35(Web Server issue):W407–10. doi: 10.1093/nar/gkm290 PMC1933241 17517781

[46] Stranzl T Larsen MV Lundegaard C Nielsen M. Netctlpan: Pan-specific mhc class I pathway epitope predictions. Immunogenetics (2010) 62(6):357–68. doi: 10.1007/s00251-010-0441-4 PMC2875469 20379710

[47] Reynisson B Barra C Kaabinejadian S Hildebrand WH Peters B Nielsen M. Improved prediction of mhc ii antigen presentation through integration and motif deconvolution of mass spectrometry mhc eluted ligand data. J Proteome Res (2020) 19(6):2304–15. doi: 10.1021/acs.jproteome.9b00874 32308001

[48] Saha S Raghava GP. Prediction methods for b-cell epitopes. Methods Mol Biol (2007) 409:387–94. doi: 10.1007/978-1-60327-118-9_29 18450017

[49] Saha S Raghava GP. Prediction of continuous b-cell epitopes in an antigen using recurrent neural network. Proteins (2006) 65(1):40–8. doi: 10.1002/prot.21078 16894596

[50] Jespersen MC Peters B Nielsen M Marcatili P. Bepipred-2.0: Improving sequence-based b-cell epitope prediction using conformational epitopes. Nucleic Acids Res (2017) 45(W1):W24–w9. doi: 10.1093/nar/gkx346 PMC5570230 28472356

[51] Ponomarenko J Bui HH Li W Fusseder N Bourne PE Sette A. Ellipro: A new structure-based tool for the prediction of antibody epitopes. BMC Bioinf (2008) 9:514. doi: 10.1186/1471-2105-9-514 PMC2607291 19055730

[52] Delano WL. The pymol molecular graphics system. Proteins (2002) 30:442–54.

[53] Calis JJ Maybeno M Greenbaum JA Weiskopf D De Silva AD Sette A. Properties of mhc class I presented peptides that enhance immunogenicity. PloS Comput Biol (2013) 9(10):e1003266. doi: 10.1371/journal.pcbi.1003266 24204222 PMC3808449

[54] Paul S Sidney J Sette A Peters B. Tepitool: A pipeline for computational prediction of T cell epitope candidates. Curr Protoc Immunol (2016) 114:18.9.1–.9.24. doi: 10.1002/cpim.12 PMC4981331 27479659

[55] Moutaftsi M Peters B Pasquetto V Tscharke DC Sidney J Bui HH. A consensus epitope prediction approach identifies the breadth of murine T(Cd8+)-cell responses to vaccinia virus. Nat Biotechnol (2006) 24(7):817–9. doi: 10.1038/nbt1215 16767078

[56] Kak G Raza M Tiwari BK. Interferon-gamma (Ifn-Γ): Exploring its implications in infectious diseases. Biomol Concepts (2018) 9(1):64–79. doi: 10.1515/bmc-2018-0007 29856726

[57] Dinarello CA. Historical insights into cytokines. Eur J Immunol (2007) 37 Suppl 1(Suppl 1):S34–45. doi: 10.1002/eji.200737772 PMC3140102 17972343

[58] Malek TR. The main function of il-2 is to promote the development of T regulatory cells. J Leukoc Biol (2003) 74(6):961–5. doi: 10.1189/jlb.0603272 12960253

[59] Dhanda SK Vir P Raghava GP. Designing of interferon-gamma inducing mhc class-ii binders. Biol Direct (2013) 8:30. doi: 10.1186/1745-6150-8-30 24304645 PMC4235049

[60] Dhanda SK Gupta S Vir P Raghava GP. Prediction of Il4 inducing peptides. Clin Dev Immunol (2013) 2013:263952. doi: 10.1155/2013/263952 24489573 PMC3893860

[61] Lathwal A Kumar R kaur D Raghava GPS. In silico model for predicting il-2 inducing peptides in human. bioRxiv (2021). doi: 10.1101/2021.06.20.449146 PMC12267595 40670434

[62] Lamiable A Thévenet P Rey J Vavrusa M Derreumaux P Tufféry P. Pep-Fold3: Faster de Novo structure prediction for linear peptides in solution and in complex. Nucleic acids research (2016) 44(W1):W449–W454. doi: 10.1093/nar/gkw329 PMC4987898 27131374

[63] Sussman JL Lin D Jiang J Manning NO Prilusky J Ritter O. Protein Data Bank (PDB): database of three-dimensional structural information of biological macromolecules[J]. Acta Crystallographica Section D: Biological Crystallography (1998) 54(6):1078–84. doi: 10.1107/s0907444998009378 10089483

[64] Waterhouse A Bertoni M Bienert S Studer G Tauriello G Gumienny R. Swiss-Model: Homology modelling of protein structures and complexes. Nucleic Acids Res (2018) 46(W1):W296–303. doi: 10.1093/nar/gky427 PMC6030848 29788355

[65] Guex N Peitsch Mc Fau - Schwede T Schwede T. Automated comparative protein structure modeling with Swiss-model and Swiss-pdbviewer: A historical perspective. Electrophoresis (2009) 30(S1):S162–73. doi: 10.1002/elps.200900140 19517507

[66] Trott O Olson AJ. Autodock vina: Improving the speed and accuracy of docking with a new scoring function, efficient optimization, and multithreading. Journal of computational chemistry (2010) 31(2):455–61. doi: 10.1002/jcc.21334 PMC3041641 19499576

[67] Laskowski RA Swindells MB. Ligplot+: Multiple ligand-protein interaction diagrams for drug discovery. J Chem Inf Model (2011) 51(10):2778–86. doi: 10.1021/ci200227u 21919503

[68] Dhople V Krukemeyer A Ramamoorthy A. The human beta-Defensin-3, an antibacterial peptide with multiple biological functions. Biochimica et Biophysica Acta (2006) 1758(9):1499–512. doi: 10.1016/j.bbamem.2006.07.007 16978580

[69] Chen X Zaro Jl W-C Shen WC. Fusion protein linkers: Property, design and functionality. Adv Drug Deliv Rev (2013) 65(10):1357–69. doi: 10.1016/j.addr.2012.09.039 PMC3726540 23026637

[70] Athanasiou E Agallou M Tastsoglou S Kammona O Hatzigeorgiou A Kiparissides C. A Poly(Lactic-Co-Glycolic) acid nanovaccine based on chimeric peptides from different leishmania infantum proteins induces dendritic cells maturation and promotes peptide-specific ifnγ-producing Cd8(+) T cells essential for the protection against experimental visceral leishmaniasis. Front Immunol (2017) 8:684. doi: 10.3389/fimmu.2017.00684 28659922 PMC5468442

[71] Alexander J del Guercio M-F Maewal A Qiao L Fikes J Chesnut RW. Linear padre T helper epitope and carbohydrate b cell epitope conjugates induce specific high titer igg antibody responses. J Immunol (2000) 164(3):1625. doi: 10.4049/jimmunol.164.3.1625 10640784

[72] Magnan CN Randall A Baldi P. Solpro: Accurate sequence-based prediction of protein solubility. Bioinformatics (2009) 25(17):2200–7. doi: 10.1093/bioinformatics/btp386 19549632

[73] Buchan DWA Jones DT. The psipred protein analysis workbench: 20 years on. Nucleic Acids Res (2019) 47(W1):W402–w7. doi: 10.1093/nar/gkz297 PMC6602445 31251384

[74] Heo L Park H Seok C. Galaxyrefine: Protein structure refinement driven by side-chain repacking. Nucleic Acids Res (2013) 41(Web Server issue):W384–8. doi: 10.1093/nar/gkt458 PMC3692086 23737448

[75] Craig DB Dombkowski AA. Disulfide by design 2.0: A web-based tool for disulfide engineering in proteins. BMC Bioinf (2013) 14:346. doi: 10.1186/1471-2105-14-346 PMC3898251 24289175

[76] Takeda K Akira S. Toll-like receptors. Curr Protoc Immunol (2015) 109:14.2.1–.2.0. doi: 10.1002/0471142735.im1412s109 25845562

[77] Vijay K. Toll-like receptors in immunity and inflammatory diseases: Past, present, and future. Int Immunopharmacol (2018) 59:391–412. doi: 10.1016/j.intimp.2018.03.002 PMC7106078 29730580

[78] Kozakov D Hall DR Xia B Porter KA Padhorny D Yueh C. The cluspro web server for protein-protein docking. Nat Protoc (2017) 12(2):255–78. doi: 10.1038/nprot.2016.169 PMC5540229 28079879

[79] van Zundert GCP Rodrigues J Trellet M Schmitz C Kastritis PL Karaca E. The Haddock2.2 web server: User-friendly integrative modeling of biomolecular complexes. J Mol Biol (2016) 428(4):720–5. doi: 10.1016/j.jmb.2015.09.014 26410586

[80] Laskowski RA Jabłońska J Pravda L Vařeková RS Thornton JM. Pdbsum: Structural summaries of pdb entries. Protein Sci (2018) 27(1):129–34. doi: 10.1002/pro.3289 PMC5734310 28875543

[81] Kutzner C Páll S Fechner M Esztermann A de Groot BL Grubmüller H. Best bang for your buck: Gpu nodes for gromacs biomolecular simulations. J Comput Chem (2015) 36(26):1990–2008. doi: 10.1002/jcc.24030 PMC5042102 26238484

[82] Maier JA Martinez C Kasavajhala K Wickstrom L Hauser KE Simmerling C. Ff14sb: Improving the accuracy of protein side chain and backbone parameters from Ff99sb. J Chem Theory Comput (2015) 11(8):3696–713. doi: 10.1021/acs.jctc.5b00255 PMC4821407 26574453

[83] Berendsen HJC Postma JPM WFv G DiNola A Haak JR. Molecular dynamics with coupling to an external bath. J Chem Phys (1984) 81(8):3684–90. doi: 10.1063/1.448118

[84] Parrinello M Rahman A. Strain fluctuations and elastic constants. J Chem Phys (1982) 76:2662–6. doi: 10.1063/1.443248

[85] Hess B Bekker H Berendsen HJC Fraaije JGEM. Lincs: A linear constraint solver for molecular simulations. J Comput Chem (1997) 18(12):1463–72. doi: 10.1002/(SICI)1096-987X(199709)18:12<1463::AID-JCC4>3.0.CO;2-H

[86] Darden T York D Pedersen L. Particle mesh ewald: An N·Log(N) method for ewald sums in Large systems. J Chem Phys (1993) 98(12):10089–92. doi: 10.1063/1.464397

[87] Humphrey W Dalke A Schulten K. Vmd: Visual molecular dynamics. J Mol Graph (1996) 14(1):33–8. doi: 10.1016/0263-7855(96)00018-5 8744570

[88] Valdés-Tresanco MS Valdés-Tresanco ME Valiente PA-O Moreno EA-O. Gmx_MMPBSA: A New Tool to Perform End-State Free Energy Calculations with GROMACS[J]. Journal of Chemical Theory and Computation (2021) 17(10):6281–6291. doi: 10.1021/acs.jctc.1c00645 34586825

[89] Arnold GE Ornstein RL. Molecular dynamics study of time-correlated protein domain motions and molecular flexibility: Cytochrome P450bm-3. Biophysical Journal (1997) 73(3):1147-59. doi: 10.1016/S0006-3495(97)78147-5 9284282 PMC1181014

[90] Grant BJ Rodrigues Ap ElSawy KM McCammon JA Caves LSD. Bio3d: An r package for the comparative analysis of protein structures. Bioinformatics (2006) 22(21):2695–96. doi: 10.1093/bioinformatics/btl461 16940322

[91] Rapin N Lund O Bernaschi M Castiglione F Castiglione F. Computational immunology meets bioinformatics: The use of prediction tools for molecular binding in the simulation of the immune system. PloS one (2010) 5(4):e9862. doi: 10.1371/journal.pone.0009862 20419125 PMC2855701

[92] Bui HH Sidney J Dinh K Southwood S Newman MJ Sette A. Predicting population coverage of T-cell epitope-based diagnostics and vaccines. BMC bioinformatics (2006) 7(1):1–5. doi: 10.1186/1471-2105-7-153 PMC1513259 16545123

[93] Zuker M. Mfold web server for nucleic acid folding and hybridization prediction. Nucleic acids research (2003) 31(13):3406–15. doi: 10.1093/nar/gkg595 PMC169194 12824337

[94] Gruber AR Lorenz R Bernhart SH Neuböck R Hofacker IL. The Vienna rna websuite. Nucleic Acids Res (2008) 36(Web Server issue):W70–4. doi: 10.1093/nar/gkn188 PMC2447809 18424795

[95] Maekawa S Yoshida T Wang PC Chen SC. Current knowledge of nocardiosis in teleost fish. J Fish Dis (2018) 41(3):413–9. doi: 10.1111/jfd.12782 29341219

[96] Valdezate S Garrido N Carrasco G Medina-Pascual MJ Villalón P Navarro AM. Epidemiology and susceptibility to antimicrobial agents of the main nocardia species in Spain. J Antimicrob Chemother (2017) 72(3):754–61. doi: 10.1093/jac/dkw489 27999029

[97] Steinbrink J Leavens J Kauffman CA Miceli MH. Manifestations and outcomes of nocardia infections: Comparison of immunocompromised and nonimmunocompromised adult patients. Med (Baltimore) (2018) 97(40):e12436. doi: 10.1097/md.0000000000012436 PMC6200467 30290600

[98] Wintheiser GA Venable ER Temesgen Z. Disseminated nocardia in an immunocompetent host. Mayo Clin Proc (2021) 96(4):847–8. doi: 10.1016/j.mayocp.2020.11.019 33814090

[99] Li J Cao J Wu Y Wan N Pan L Chen Y. A case of an immunocompetent young man obtaining community-acquired disseminated nocardia brasiliensis. Heart Lung (2014) 43(2):164–7. doi: 10.1016/j.hrtlng.2013.12.002 24594251

[100] Miller JF Vadas MA. Antigen activation of T lymphocytes: Influence of major histocompatibility complex. Cold Spring Harb Symp Quant Biol (1977) 41 Pt 2:579–88. doi: 10.1101/sqb.1977.041.01.067 302179

[101] Kwissa M Kasturi SP Pulendran B. The science of adjuvants. Expert Rev Vaccines (2007) 6(5):673–84. doi: 10.1586/14760584.6.5.673 17931149

[102] Dhople V Krukemeyer A Ramamoorthy A. The human beta-Defensin-3, an antibacterial peptide with multiple biological functions. Biochim Biophys Acta (2006) 1758(9):1499–512. doi: 10.1016/j.bbamem.2006.07.007 16978580

