# Supplementary material for: Design of a multi-epitope vaccine against six Nocardia species based on reverse vaccinology combined with immunoinformatics
Source: Front Immunol. 2023 Feb 2;14:1100188. doi: 10.3389/fimmu.2023.1100188 (PMC9952739; doi:10.3389/fimmu.2023.1100188)
Supplement: Supplementary file 1 [file DataSheet_1.docx]

Supplementary Material

# Supplementary Data

## The DNA sequence of the designed vaccine:

GGTATCATTAACACCTGCCAGAAGTGCTATTGTCGCGTGCGTGGCGGCCGCTGCGCCGTGCTGAGCTGCCTGCCGAAAGAATGTCAGATCGGCAAATGTAGCACCCGCGGCCGTAAATGTTGTCGTTGCAAAAAAGAAGCCGCAGCGAAAAAACCGAGCCAGGTGGAAATCCTGCCGGGCATTACCATTCCGGTTGGCCCGGGTCCGGGTACCGCAGTGGTTAGCGTGGACCCGAAAAGCGGTGCAGTGCGCGCCGGTCCGGGTCCGGGCGCAGTGGTGAGCGTCGATCCGAAAAGCGGCGCCGTGCGTGCGTATGGCCCGGGCCCCGGCGATACCCTGATTCAGAACATTCCGAGTCGTGCGGATGAATTCCGTGGCCCGGGCCCGGGCGAACCGGGTCTGGCCAATACCCTGGCGAACGCGCTGAGCCAGGATGGCCCGGGTCCGGGCGGTTATCTGATTCGCACCACCTTAGACCCGGCCGTGCAGAACTCACACGAATACGGCGCGGAAGCACTTGAACGCGCTGGCAGCACCGATGGCACCGGCGATGGCTATGCGGCGTACAAAAGCGGCGCCGTTCGTGCCTATTATGCGGCGTACATTAGTCCGGCGTGGTTTAGCCCGTATGCCGCCTATAGCATGGCGGGCGCGGCCTATATTTATCATGAATACGGCGCGGAAGCACTGGAACGTGCGTGCGGTGTGGGCAATAGCACTGATGGCACCGGCGATGGCTATACCAATAGCAAAAAACCGGCGGATGGCAGCTCCCCGAGTACCAGCAACAGCGGCGATACCAGCAAAAAAGGCAGCACCATTACGCAGCAGTACGTGAAAAATGCAATGGTGGGCAACAAAAAACCGTCAACCAGCAATAGCGGTGATACCTCAGGCAATAGCCGCAGCCAAAAAAAAACCAGCGGCAACTCACGTTCTCAGCGTCCGGGCGCCGGAGTGGGCAACAAAAAACTGCAGTACGAAGCTAAAAACCACCGTGACGATGACACCGGCAAAGTGGCGGCGGGCGGCTACGGCGATAAAAAACGCGGCAGCAATAACGCCCAGATTCCGGATGTGAACGGCATGAGCGAAAAAAAAAGCGGCGAAGTTCAGCAGGGCGAATGCGCCGCGAAAGCCAAATTTGTTGCGGCGTGGACCCTGAAAGCCGCAGCGTAA

## The RNA sequence of the designed vaccine

GGUAUCAUUAACACCUGCCAGAAGUGCUAUUGUCGCGUGCGUGGCGGCCGCUGCGCCGUGCUGAGCUGCCUGCCGAAAGAAUGUCAGAUCGGCAAAUGUAGCACCCGCGGCCGUAAAUGUUGUCGUUGCAAAAAAGAAGCCGCAGCGAAAAAACCGAGCCAGGUGGAAAUCCUGCCGGGCAUUACCAUUCCGGUUGGCCCGGGUCCGGGUACCGCAGUGGUUAGCGUGGACCCGAAAAGCGGUGCAGUGCGCGCCGGUCCGGGUCCGGGCGCAGUGGUGAGCGUCGAUCCGAAAAGCGGCGCCGUGCGUGCGUAUGGCCCGGGCCCCGGCGAUACCCUGAUUCAGAACAUUCCGAGUCGUGCGGAUGAAUUCCGUGGCCCGGGCCCGGGCGAACCGGGUCUGGCCAAUACCCUGGCGAACGCGCUGAGCCAGGAUGGCCCGGGUCCGGGCGGUUAUCUGAUUCGCACCACCUUAGACCCGGCCGUGCAGAACUCACACGAAUACGGCGCGGAAGCACUUGAACGCGCUGGCAGCACCGAUGGCACCGGCGAUGGCUAUGCGGCGUACAAAAGCGGCGCCGUUCGUGCCUAUUAUGCGGCGUACAUUAGUCCGGCGUGGUUUAGCCCGUAUGCCGCCUAUAGCAUGGCGGGCGCGGCCUAUAUUUAUCAUGAAUACGGCGCGGAAGCACUGGAACGUGCGUGCGGUGUGGGCAAUAGCACUGAUGGCACCGGCGAUGGCUAUACCAAUAGCAAAAAACCGGCGGAUGGCAGCUCCCCGAGUACCAGCAACAGCGGCGAUACCAGCAAAAAAGGCAGCACCAUUACGCAGCAGUACGUGAAAAAUGCAAUGGUGGGCAACAAAAAACCGUCAACCAGCAAUAGCGGUGAUACCUCAGGCAAUAGCCGCAGCCAAAAAAAAACCAGCGGCAACUCACGUUCUCAGCGUCCGGGCGCCGGAGUGGGCAACAAAAAACUGCAGUACGAAGCUAAAAACCACCGUGACGAUGACACCGGCAAAGUGGCGGCGGGCGGCUACGGCGAUAAAAAACGCGGCAGCAAUAACGCCCAGAUUCCGGAUGUGAACGGCAUGAGCGAAAAAAAAAGCGGCGAAGUUCAGCAGGGCGAAUGCGCCGCGAAAGCCAAAUUUGUUGCGGCGUGGACCCUGAAAGCCGCAGCGUAA

# Supplementary Figures and Tables

## Supplementary Figures

### Supplementary Figure 1


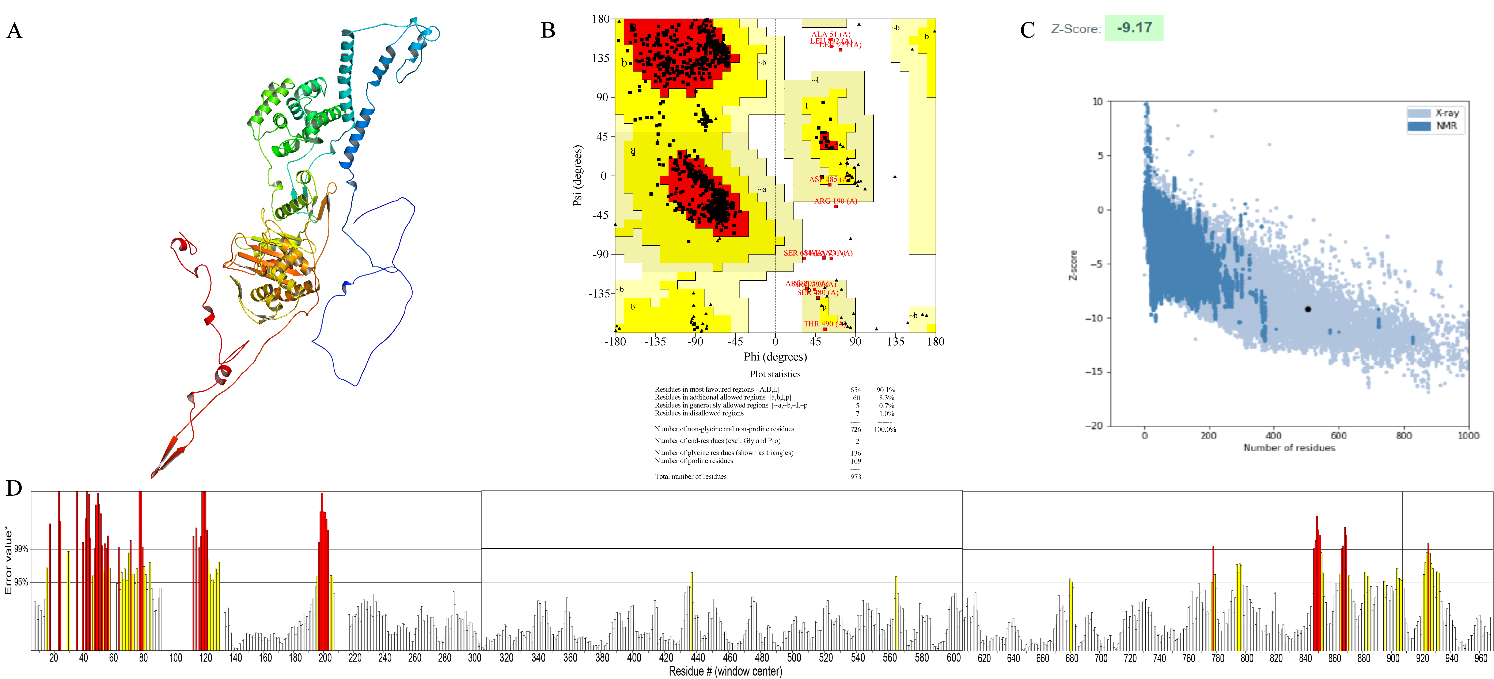


**Supplementary Figure 1.** (A) Three-dimensional (3D) structure of the target protein CORE_REP|Org125_Gene1111. (B) The Ramachandran plot of the refined 3D model generated by the PROCHECK server, the red-colored regions are the most favored regions, the dark yellow and light yellow regions are the additional allowed and generously allowed regions, the white regions are the disallowed regions. (C) The Z-score plot of the refined 3D model generated by the ProSA-web server. (D) The ERRAT score of the refined 3D model generated by the ERRAT server.

### Supplementary Figure 2


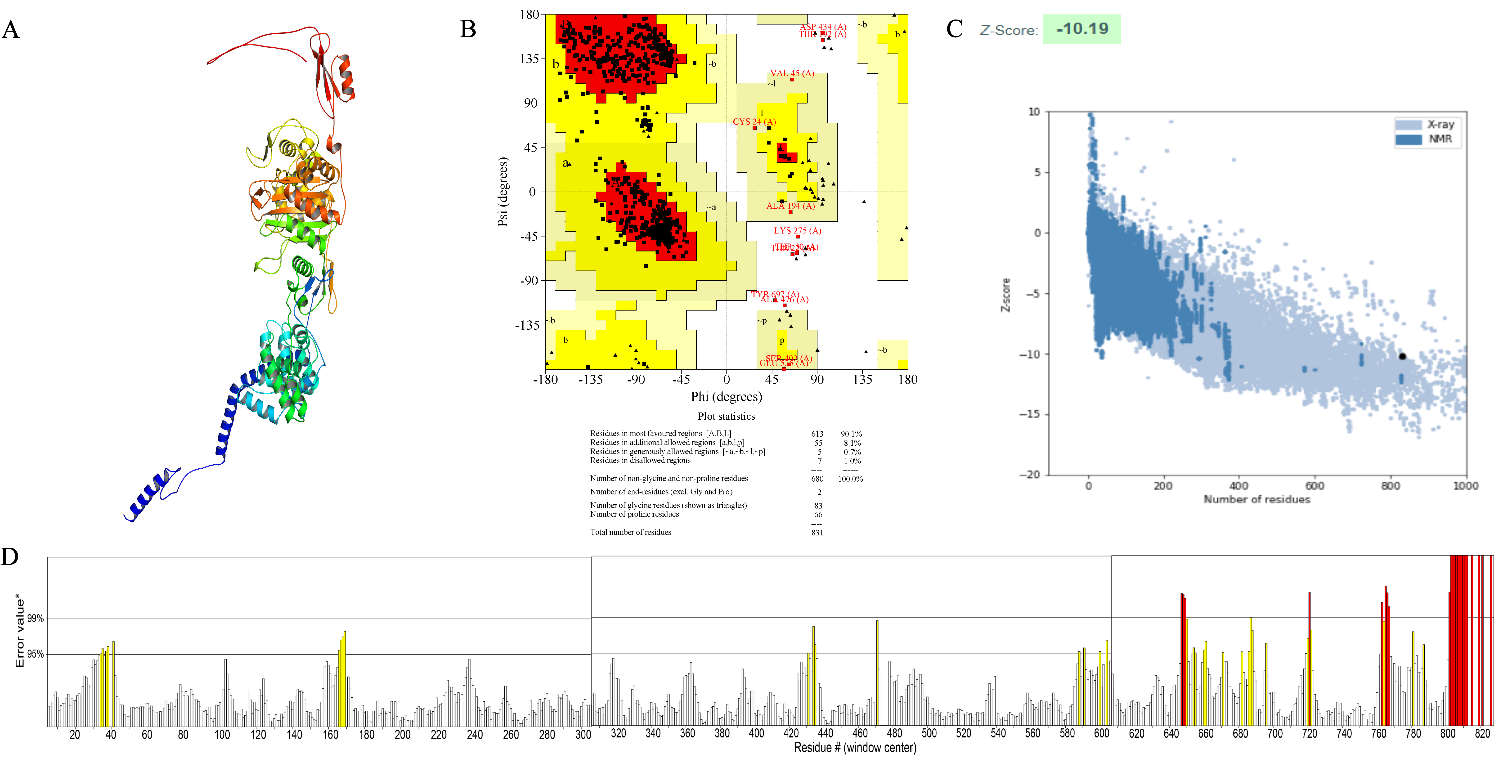


**Supplementary Figure 2.** (A) Three-dimensional (3D) structure of the target protein CORE_REP|Org97_Gene925. (B) The Ramachandran plot of the refined 3D model generated by the PROCHECK server, the red-colored regions are the most favored regions, the dark yellow and light yellow regions are the additional allowed and generously allowed regions, the white regions are the disallowed regions. (C) The Z-score plot of the refined 3D model generated by the ProSA-web server. (D) The ERRAT score of the refined 3D model generated by the ERRAT server.

### Supplementary Figure 3


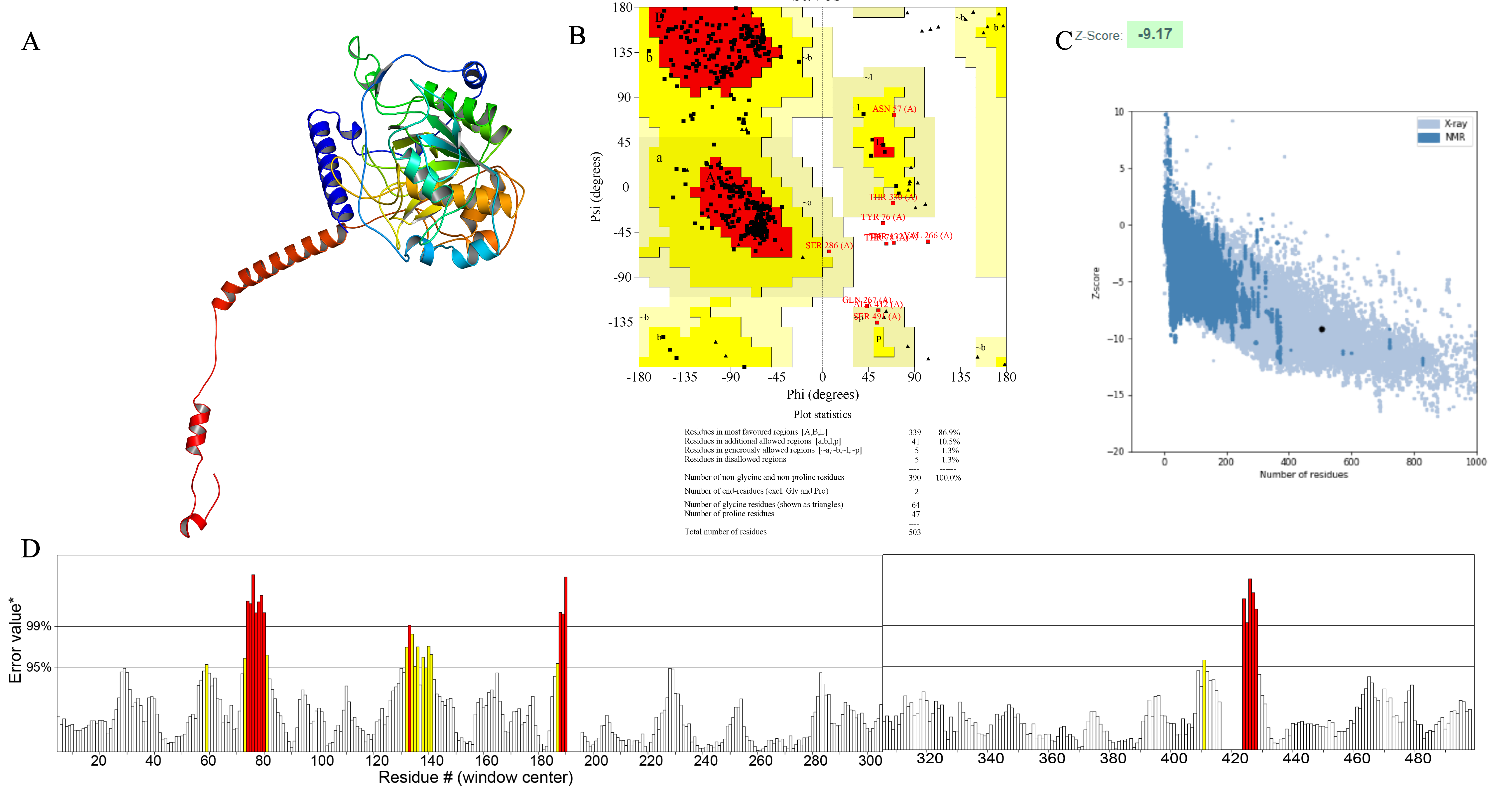


**Supplementary Figure 3.** (A) Three-dimensional (3D) structure of the target protein CORE_REP|Org5_Gene928. (B) The Ramachandran plot of the refined 3D model generated by the PROCHECK server, the red-colored regions are the most favored regions, the dark yellow and light yellow regions are the additional allowed and generously allowed regions, the white regions are the disallowed regions. (C) The Z-score plot of the refined 3D model generated by the ProSA-web server. (D) The ERRAT score of the refined 3D model generated by the ERRAT server.

### Supplementary Figure 4


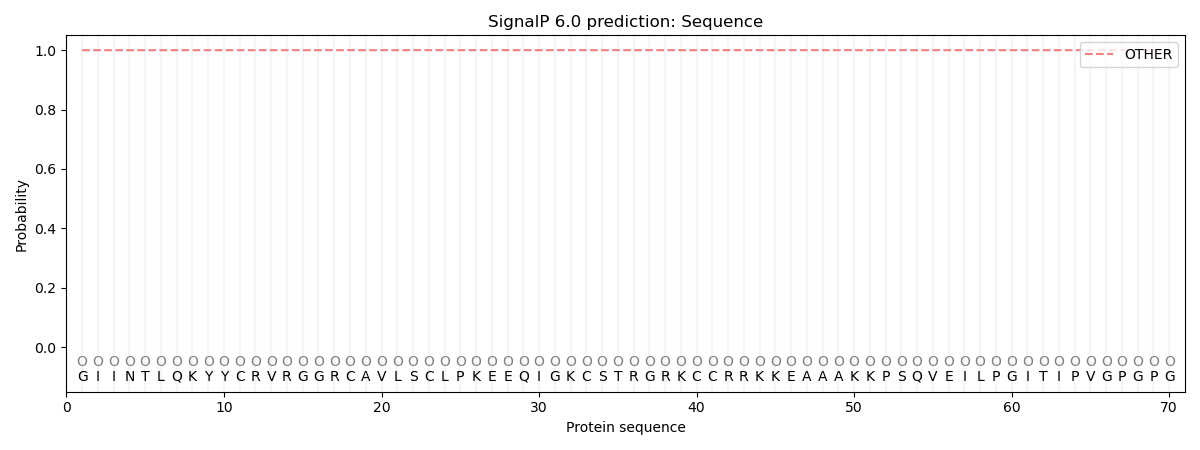


**Supplementary Figure 4**. The plot about the signal peptide prediction of the vaccine

### Supplementary Figure 5


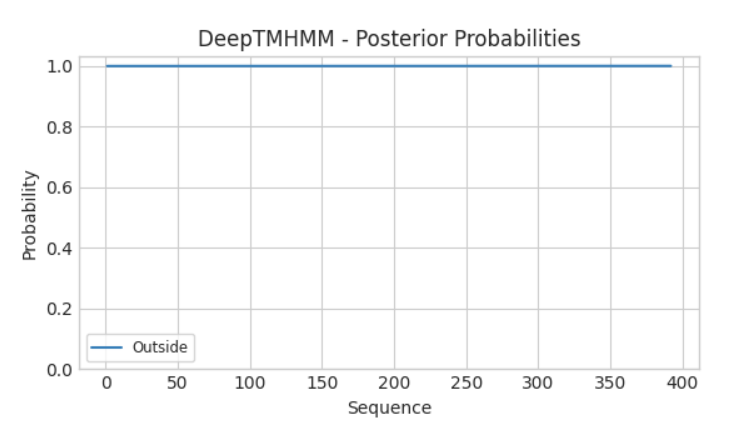


**Supplementary Figure 5**. The plot about the transmembrane helix of the vaccine

### Supplementary Figure 6


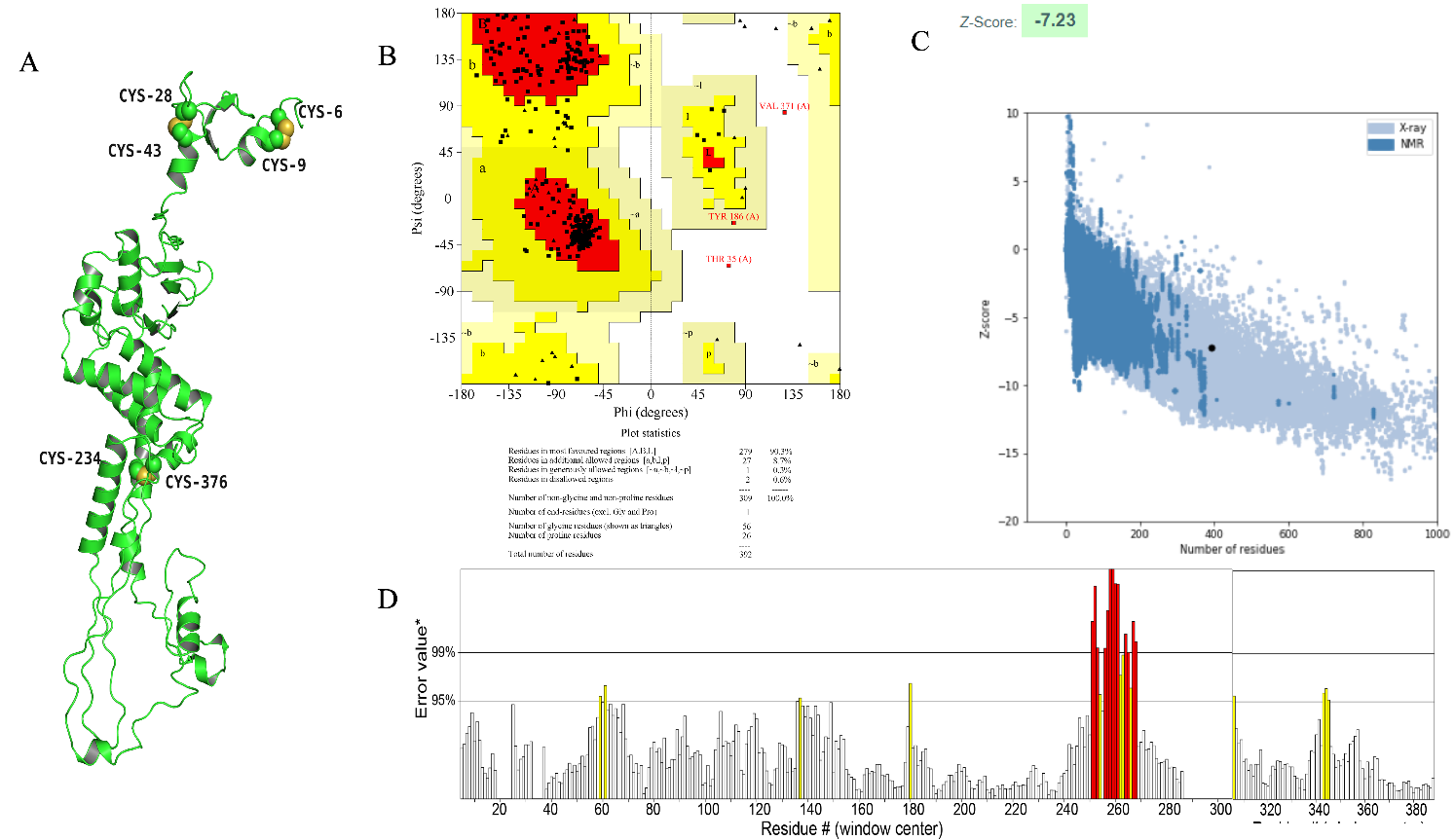


**Supplementary Figure 6**. (A) Three-dimensional (3D) structure of the vaccine after disulfide engineering, the yellow spheres represent disulfide bonds. (B) The Ramachandran plot of the refined 3D model generated by the PROCHECK server, the red-colored regions are the most favored regions, the dark yellow and light yellow regions are the additional allowed and generously allowed regions, the white regions are the disallowed regions. (C) The Z-score plot of the refined 3D model generated by the ProSA-web server. (D) The ERRAT score of the refined 3D model generated by the ERRAT server.

### Supplementary Figure 7


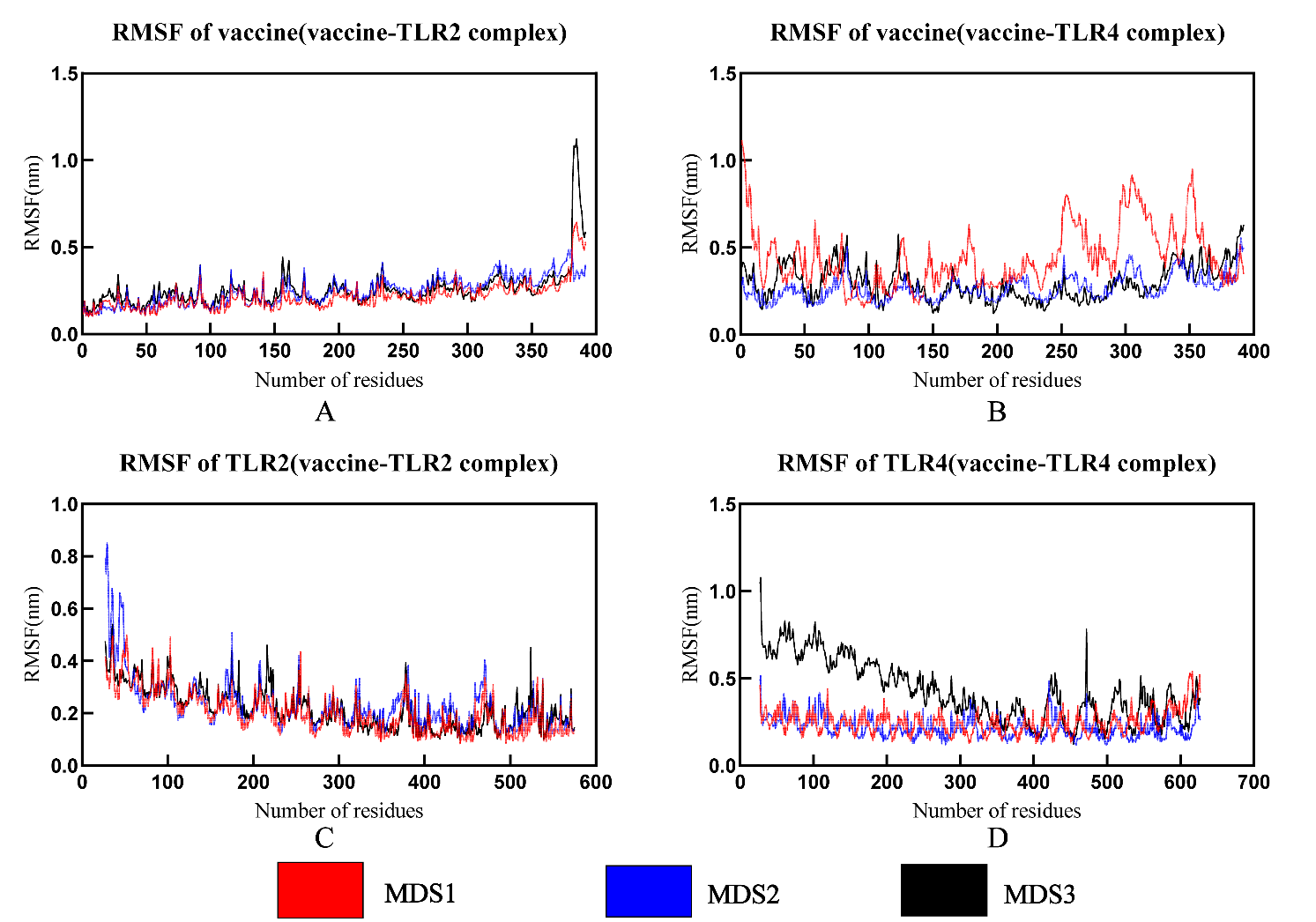


**Supplementary Figure 7**. The RMSF plot of the vaccine-TLRs. (A, C) The RMSF of vaccine-TLR2 complex. (B, D) The RMSF of vaccine-TLR4 complex

### Supplementary Figure 8


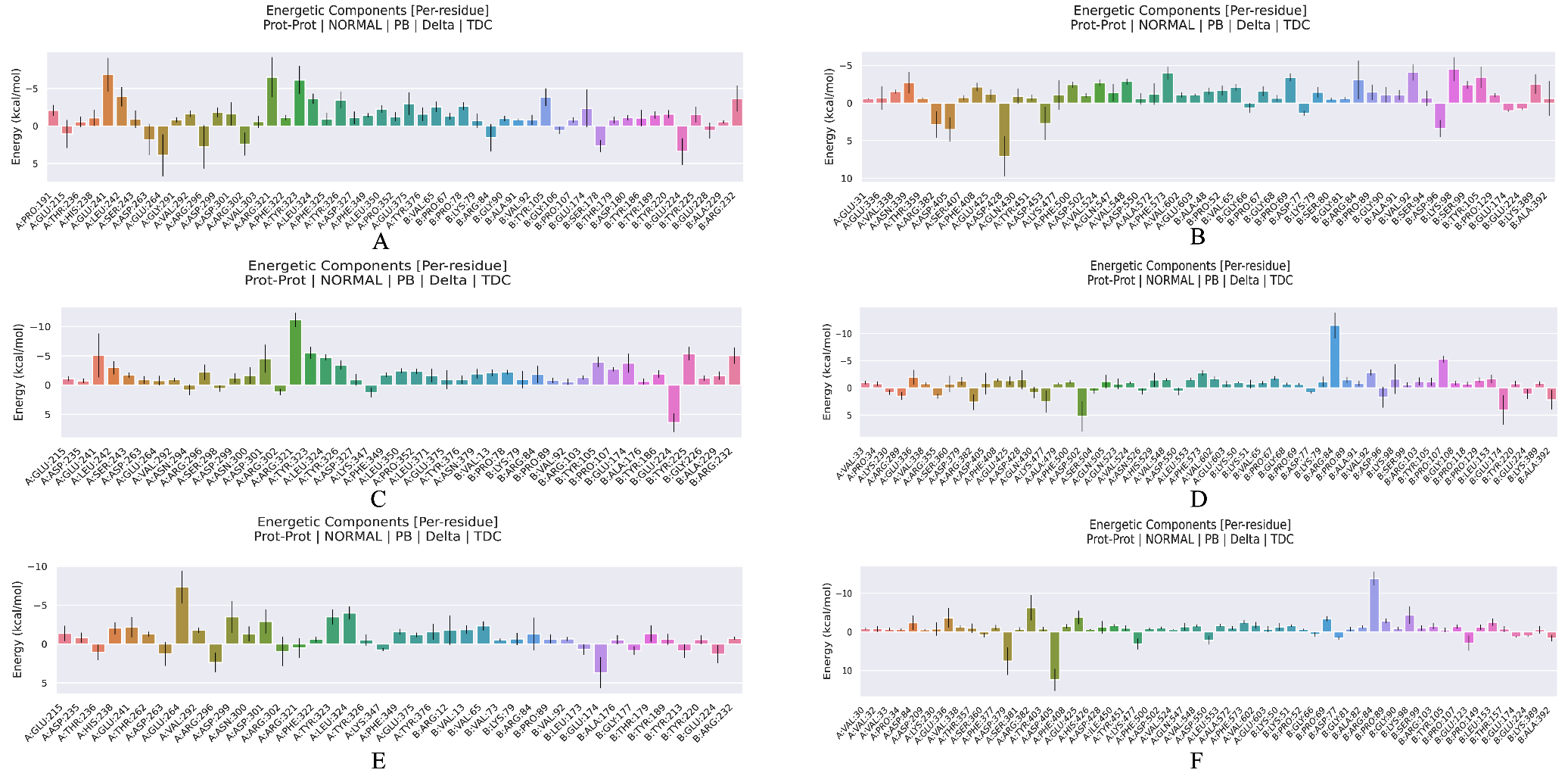


**Supplementary Figure 8**. The MM-PBSA decomposition analysis of the vaccine-TLRs. (A, C, E) The MM-PBSA decomposition analysis of the vaccine-TLR2 complex for the first, second and third molecular dynamics simulation. (B,D,F) The MM-PBSA decomposition analysis of the vaccine-TLR4 complex for the first, second and third molecular dynamics simulation

### Supplementary Figure 9


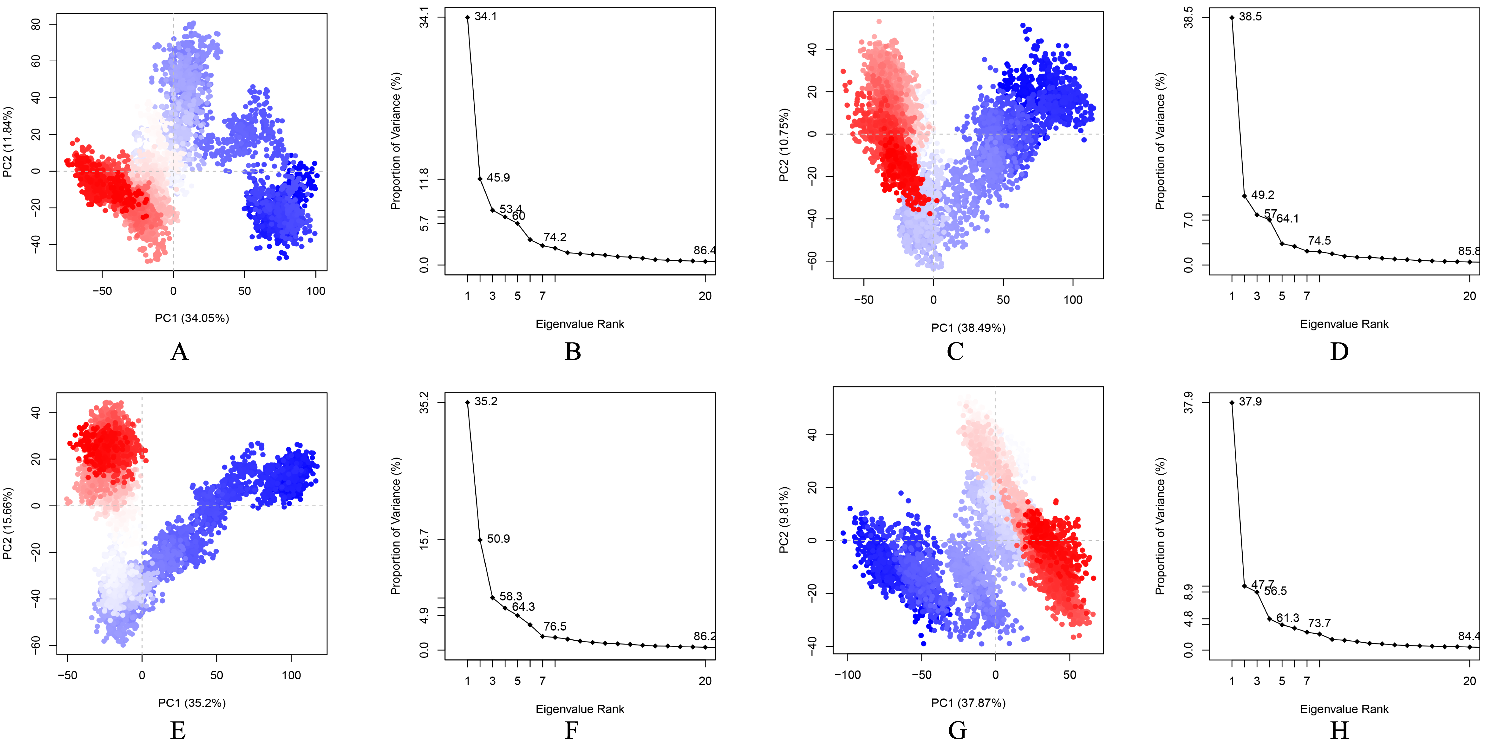


**Supplementary Figure 9**. The PCA analysis of the vaccine-TLRs for second and third molecular dynamics simulations. (A, B) The PCA plot of the vaccine-TLR2 complex for second MD simulation. (C, D) The PCA plot of the vaccine-TLR2 complex for third MD simulation. (E, F) The PCA plot of the vaccine-TLR4 complex for second MD simulation. (G, H) The PCA plot of the vaccine-TLR4 complex for third MD simulation.

### Supplementary Figure 10


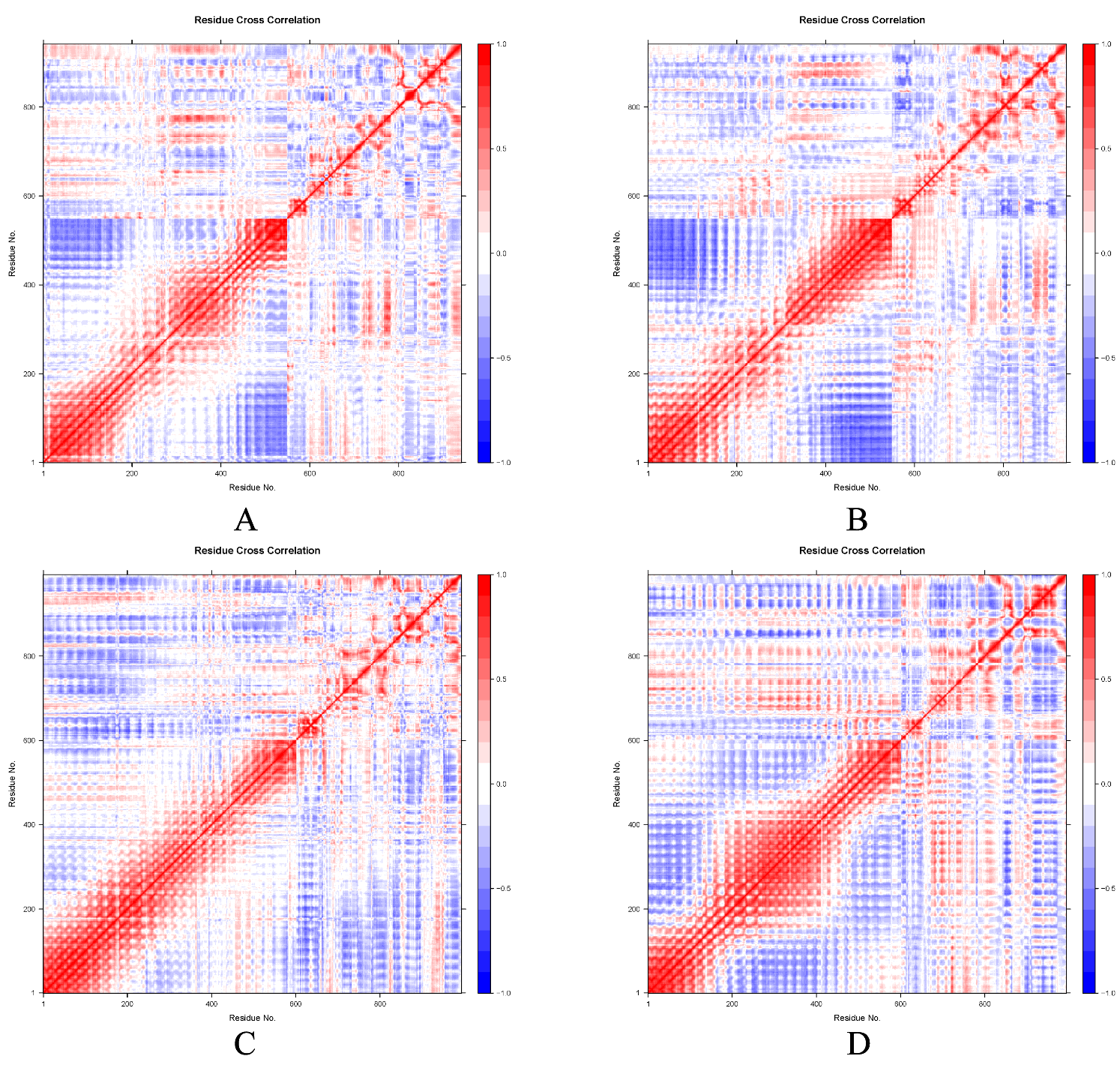


**Supplementary Figure 10.** The DCC analysis of the vaccine-TLRs for second and third MD simulations. (A)The DCCM plot of vaccine-TLR2 complex for second MD simulation. (B)The DCCM plot of vaccine-TLR2 complex for third MD simulation. (C)The DCCM plot of the vaccine-TLR4 complex for second MD simulation. (D)The DCCM plot of the vaccine-TLR4 complex for third MD simulation.

### Supplementary Figure 11


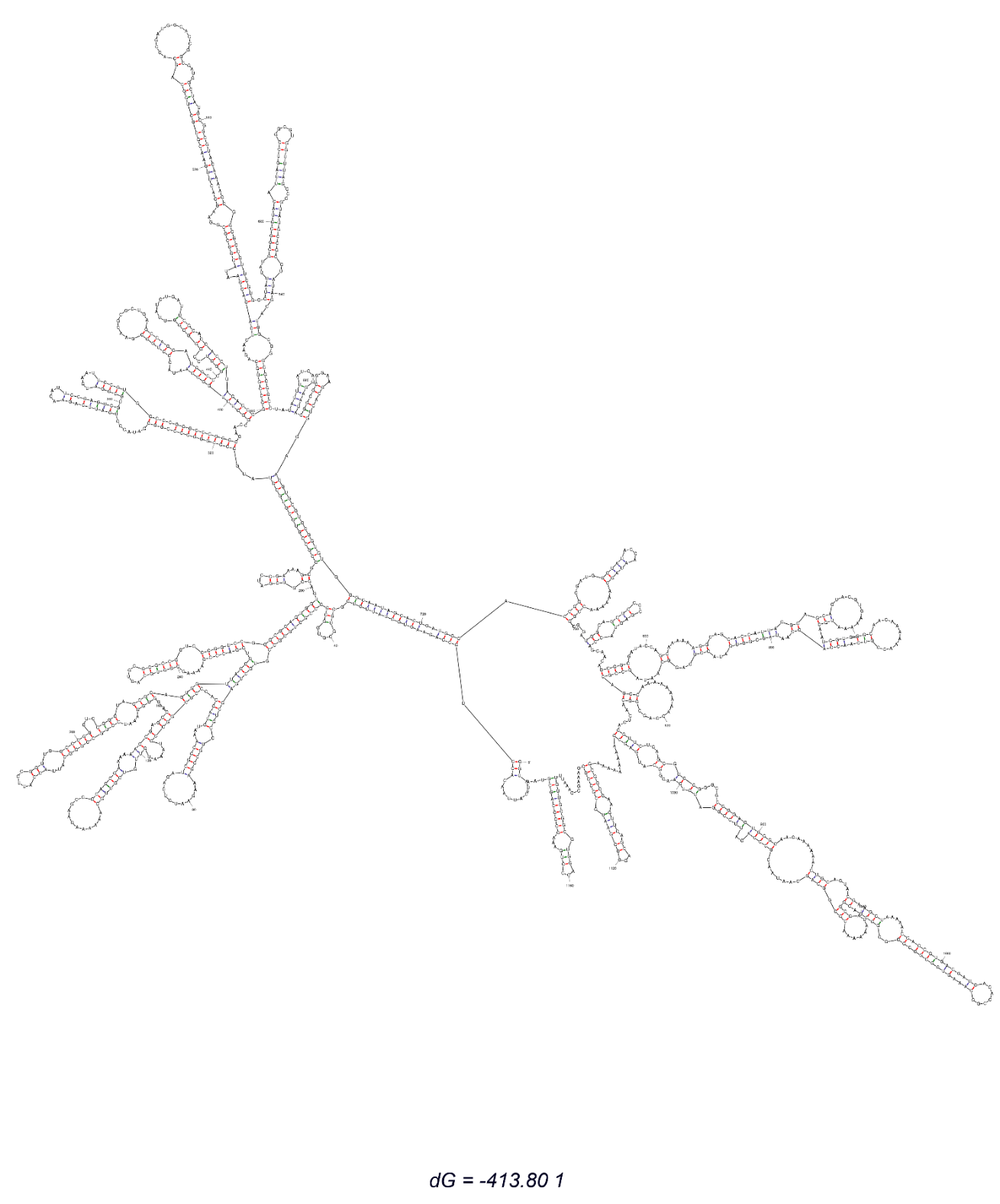


**Supplementary Figure 11**. The plot of secondary structure of the vaccine’s mRNA generated by mFold v2.3

## Supplementary Tabels

## 2.1.1 Supplementary Table 1

**Table S1**. Docking results of CTL epitopes with MHC I alleles

|  | **Peptide** | **MHC** | **PDB_ID** | **Chain** | **Binding affinity** | **Hydrogen_bond** | **Salt bridges** |
| --- | --- | --- | --- | --- | --- | --- | --- |
| CTL epitopes | STDGTGDGY | HLA-A*01:01 | 4nqx | A | -9.1 | Ser1-Tyr7,Ser1-Tyr171,Thr2-Asn66,Thr2-Arg163,Asp3-Tyr99,Gly8-Trp147,Tyr9-Tyr84,Tyr9-Asp116 | Asp3-Arg163 |
|  | KSGAVRAYY | HLA-B*58:01 | 5v5l | A | -8.5 | Lys1-Tyr7,Lys1-Glu63,Lys1-Tyr171,Val5-Arg97,Arg6-Tyr9,Arg6-Asn66,Tyr8-Asn77,Tyr9-Trp147 | NA |
|  | ISPAWFSPY | HLA-B*15:02 | 6vb2 | A | -9.8 | Ser7-Glu152,Ser7-Trp147,Pro8-Trp147,Tyr9-Ser77,Tyr9-Asn80,Tyr9-Lys146,Tyr9-Tyr84,Tyr9-Ser116 | NA |
|  | SMAGAAYIY | HLA-B*35:01 | 7m8u | A | -8.4 | Met2-Lys146,Ala5-Arg97,Ala6-Tyr9.Ala6-Asn70,Tyr9-Ala150 | NA |

## 2.1.2 Supplementary Table 2

**Table S2**. Docking results of HTL epitopes with MHC II alleles

|  | **Peptide** | **MHC** | **PDB ID** | **Chain** | **Binding affinity** | **Hydrogen_bond** | **Salt bridges** |
| --- | --- | --- | --- | --- | --- | --- | --- |
| HTL epitopes | KPSQVEILPGITIPV | HLA-DRB1*01:01 | 5v4n | B | -6.6 | Lys1-Tyr309,Pro2-Tyr309,Gln4-Gln313,Val5-Arg320,Ile13-Thr326,Pro14-Thr326 | NA |
|  | TAVVSVDPKSGAVRA | HLA-DRB1*03:01 | NA | B | -4.9 | Thr1-Glu38,Ser5-Asp86,Val6-Trp90,Ser10-Ser40.Ala12-Glu38,Arg14-Glu38 | NA |
|  | AVVSVDPKSGAVRAY | HLA-DRB1*03:01 | NA | B | -5.6 | Ala1-Tyr39,Ser4-Phe36,Lys8-Glu38 | Arg13-Asp57 |
|  | DTLIQNIPSRADEFR | HLA-DRB1*04:05 | NA | B | -6.4 | Thr2-Cys44,Leu3-His42,Gln5-Val40,Arg10-Leu82,Arg10-Ser86,Ala11-Glu38,Asp12-Glu38,Glu13-Phe36,Phe14-Arg35,Arg15-Pro34 | Asp1-Lys41 |
|  | EPGLANTLANALSQD | HLA-DRB1*07:01 | NA | B | -4.4 | Glu1-Phe36,Gln14-Glu57 | NA |
|  | GYLIRTTLDPAVQNS | HLA-DRB4*01:01 | NA | B | -5.2 | Tyr2-Asn62,Arg5-Arg35,Thr6-Tyr61,Gln13-Glu38,Ser15-Arg100 | NA |

## 2.1.3 Supplementary Table 3

**Table S3**.The quality validation of the MHC II allele molecules

| MHC II molecules | Template | Seq Identity | Ramachandran plot | | | | ERRAT score | Z score |  |
| --- | --- | --- | --- | --- | --- | --- | --- | --- | --- |
|  |  |  | Residues in most favoured regions | Residues in additional allowed regions | Residues in generously allowed regions | Residues in disallowed regions |  |  |  |
|  |  |  |  |  |  |  |  |  |  |
| HLA-DRB1*03:01 | 3pdo | 89.90% | 90.40% | 9.00% | 0.00% | 0.60% | 97.207 | -5.52 |  |
| HLA-DRB1*04:05 | 3o6f | 93.17% | 87.10% | 10.70% | 1.10% | 1.10% | 88.601 | -5.21 |  |
| HLA-DRB4*01:01 | 3pdo | 86.87% | 92.30% | 7.10% | 0.00% | 0.60% | 94.253 | -5.15 |  |
| HLA-DRB1*07:01 | 3pdo | 89.90% | 89.70% | 9.70% | 0.00% | 0.60% | 95.349 | -5.51 |  |

## 2.1.4 Supplementary Table 4

**Table S4.** The docking results and analysis of the vaccine-TLRs complex

|  | Vaccine-TLR2 complex | Vaccine-TLR4 complex |
| --- | --- | --- |
|  | Cluspro 2.0 server | |
| Center weighted Score | -733.3 | -743.4 |
| Lowest Energy weighted Score | -837.3 | -835.1 |
|  | HADDOCK 2.4 server | |
| HADDOCK score | -194.9 +/- 1.0 | -243.4 +/- 1.7 |
| RMSD from the overall lowest-energy structure | 0.6 +/- 0.4 | 0.6 +/- 0.3 |
| Van der Waals energy | -103.9 +/- 3.6 | -105.8 +/- 4.0 |
| Electrostatic energy | -336.1 +/- 19.7 | -528.6 +/- 21.1 |
| Desolvation energy | -23.8 +/- 1.4 | -31.9 +/- 1.4 |
| Restraints violation energy | 0.0 +/- 0.0 | 0.0 +/- 0.0 |
| Buried Surface Area | 3058.0 +/- 95.7 | 3533.9 +/- 47.8 |
|  | PDBsum server | |
| Number of Hydrogen bond | 12 | 12 |
| Number of Salt bridge | 2 | 7 |

## 2.1.5 Supplementary Table 5

**Table S5.** The population coverage of vaccines in different regions

| Area | Population coverage |
| --- | --- |
| World | 89.31% |
| Europe | 93.47% |
| North Africa | 90.21% |
| Central Africa | 80.50% |
| South Africa | 85.92% |
| West Africa | 88.79% |
| East Africa | 81.38% |
| South Asia | 90.87% |
| North America | 88.49% |
| China | 86.63% |
| United States | 88.91% |
| Germany | 93.43% |
| Italy | 97.61% |
| Spain | 83.13% |
| Russia | 81.09% |
| India | 88.60% |
| Japan | 80.39% |
