# Supplementary material for: Design of a multi-epitope vaccine against six Nocardia species based on reverse vaccinology combined with immunoinformatics
Source: Front Immunol. 2023 Feb 2;14:1100188. doi: 10.3389/fimmu.2023.1100188 (PMC9952739; doi:10.3389/fimmu.2023.1100188)
Supplement: Supplementary file 9 [file Table_2.docx]

>CORE_REP|Org119_Gene7073#

MSSLATDSVDQCSSGESSAQPFVLSPAQTALWYAQRIRPDVPLTIAQYVEIHGDLDVGRLLYAIERFGAESEVGKLRLAEIDGIPHQIVDPARRPGWARVDLRGERDPHAAALRWMHEYTGSPIDLERDPLTANVVLRTGDSDYIWYSRAHHIVIDGYGAMNALTRTAEIYTALENRTEPVVSRAAPLAEIYADEVRYRETSRFRADRDYWLEQLAGAGEPMSLGGSTVTAATQDAGRRIAAGVLDDRAQAAMDAAVTTFGTANSALFVAALGAYVRSVTGNPDVVLSLPVSARTTVSLRRSAGVVSNVVPIRLRFGAETTLAEVVKATELQITGALRHQRYRHDDIRRDCGYSRDARGFFGPMVNIMLFHDELTFGSLVGSLNVLATGPVEDLSVNLYNGVGGRIHVDFEANPRLYGEAEVSVHHDRFLDFLTRFLGAAPDTHAETLTAITAAEHERVLHEWNATEAPRQPGTLAELFAERAAACPDAIALESGDDTADPSPVHPVTTLTYRELDERANRLARLLIERGAGPETVVGLCLRRSIDLVVGMYAIVKTGAAYLPLDPEHPADRLDQIVRQASPVCVLTAARDELAMPESAAALAIDTVELSGYRGAPITDAERTAALRADHLAYVIFTSGSTGKPKGVGVSHAAIVNRLRWMQHEYSLDRTDVVLQKTPATFDVSVWEFFWPLQIGARLVVAAHDGHRDPAYLARLIAEKGITTAHFVPSMLSVFVTDTDVRGCTALRQVFCSGEALPAATVRDFHAALPRPALHNLYGPTEAAVDVTYWPCPADPATVPIGSPVWNTQTYVLDSRLRPVPPGVVGELYLAGVQLARGYLGQPRLTADRFVANPFGAGVRMYRTGDLARWQLGTDRPGVLEYMGRSDFQVKIRGLRIELGEIEAALLDDARVARAVCVAHPGRNGDELVAYVVATPAAGRLDTTALLTELRRTLPAYMVPSALLELDELPLSANGKIDRKALPAPVGVRATGRSTAEPRTEVERVLARVFAEMLGTEVGVEDSFFDLGGNSLVAARAVARINAALGTGLTIRDLFEASTIAALTQRFATHPADVSSPKLVAAQRPERIPLSLAQQRLWILNRFAEHAAAYNMPLAVRIEGALDVEALRAGLVDVIERHESLRTTFPESAEGAVQLVHPAAEIPLTLDPIDAAGADVAELATEFAGYGFDLRSQAPIRVALYRTGPDQWVFLVVLHHICGDGWSIAPLARDLMTAVAARGAGAAPQWAPLPVQYADFALWQRELLGNESDPASALSGQLTHWRSALAGLPDQLDLPLDRPRPLRRSTTGGRVDFTISPEIRRAASELAAARGVSMFMVLHAALATLLSRLCASTDIAIGTPIAGRSDPALDELVGMFVNTLVLRTEIDPAAGFDRMLDVVRETDLNAFANADVPFERLVEVVNPERSAARHPLFQVMLSYDRDPDLRIELPGVRAEVLPIVSDIAKFDLQLVVHDDVTDGPLTAEFGYATDIFDRATVESFARRFVAVLNAVVAAPSMPIGDLSILDRREIANLVPIAGAPAEPFTTLARLLTDTAERVPDAVAVRYLGVDTTYRELDESSNRLARVLIEHGAGPEVVVAIALPRGLDAITAVWAVAKTGAAYVPIDPSYPGERIAHMIGDSGAILGLTDAACLAAMPEWPAPRGKHRKNYVDWLVLGSAELAAEAVHCSTAPITDADRHHSLCTVHPAYLIYTSGSTGKPKAVVVTHAGLASLANEQTHLFGVTDSARTLHFSSPSFDASVLELLLGFAAGATIVVAPAGMYGGAELATLLRTERVTHAFVTPAALATVPTDGLDELEAVIVGGEACSEELVETWSAEHRMHNMYGPSEATVAATATGPMVPGRPVPLGQPIRGMRLFVLDGRLHPVPPGTPGELYLSGPGLARGYHGRYGLTAQRFLANPHGRRGERMYRTGDLVVVETGGQVRFLGRADDQIKIRGFRIELREIDHVLRAHPGVNFALTVVHTDEHGQPRLASYVTVDHPVAAADLTETARQRLPGYMVPASVTVLAELPVTPAGKLDRKALPEPVFATGGSSRAPATELESRVAGVFGEILGRPVTGAEDSFFDVGGNSLLATRLAAALHAEFGVDLPVRVIFEAPTVAGVAERLTEAPRTQRLALAVQTTRPGRIPLSLPQQRLWFLNRYSPESSAYNIAFVIRIAGDLDVAALRAALTDLVERHEVLRTVFPEDSAGAQQVVLPTARALPAIEAIDTDEAGATAALGALAHRGFDLIRDTPLRMTLLRTGSERYLLGIVVHHIAADGWSLGPLTRDLAAAYVARHGGAAPAWTPLPVQYADFGLWQRACLGDEGEPGSLAAEQLAYWRSALADLPAELPLPYDRPRPAEPTQYAGAVPFTVPDPVQRALAELAKEQGVSMFMVLRSALAVLLRSVTGGRDIVIGTPVAGRTDTKLDELVGMFVNTLVLRSDVDPDRPFAGLLRADRDTELAAMAHADIPFERVVEELASGTTRGRHPLFQVALTVQDGPVPTLELPGLELRAEELDIALAKFDLELRVAHIGCDAGPGEPGRAFEFVYAAELFDEATIHTLADRFLRVLAAVTADPRVLVRDIDTRTERERRLLAPATGGPTTPQCTLAAYFTATAHMHPHRTAVRSGATTLTYAELDKRSNRLARALLARDIGIGDRVALGLTRSVESVLTVLAVVKTGAAFVPVDPNYPADRVRHMLADAGCWVGVTVGAHAERLRTAAADGPATDWLLLDDPAVRAELETYDDALVDDLDRMCTIEAADLAYLIYTSGSTGKPKGVAVTHAGLSNFADELRDRMRVDRESRTLHFASPSFDAAVLDLLLAVGSGAAMVLCPPDVYGGDELAALLERERITHTFMTPAALATIDHERWPLPHLRALMVGGEACAPDLVARWAPGRTMLNGYGPTETTIVATIATLTAEQPVTIGTLVRGARALVLDERLRPVPAGVPGDLYLGGHGVARGYFDRFGLTALRFVADPFGPAGARLYRTGDVVRWNDAGELCYLGRSDHQVKVRGFRIELGEITAALGEHPAVRFAHTEVRQIAGADRIVAFVQPADEHTGVDVEAVRDRLGAQLPAHMVPASITVLERIPLTPVGKLDSAALPEPQLAVAAATREPSTPSERLVARVMGELVGVDAVRADDSFFDIGGNSLLATQLVARLAAASNTRLEVRTVFAAPRVAELAAHLDSGPAGARSRPALVRQARPDRIPLSAAQRRLWFLNRFNGIGEAAADGADLSAGAYNVPVVLRMNGKLNVDALVVALHAVQDRHETLRTVFPEVGGEPTQRVLDLVTAAITLFVATVRPDEVDDAVRRFAAPGFDLAGVVPMRAALISVSPDGDRGVRNPAEVSDEHVLVLVVHHIAMDGQSLAPLALDVATAYRAACADRSPEWDELAVQYVDYTLWQQDTLGTEDDPDSVIRRQLDYWRHQLDGVPELLTLPADRRRPPVPSYRGGLVECEIDAFTHRDLHRVATSNNVSMFMVLHAALAVLLHRMSATDDITVGTPIAGRGHPALDRLIGMFVNTLVLRTRIDPDARFTDLLHTVRDVDLDAFAHADLPFERLVEVLNPARSQAHHPMFQVMLSVQNHPVGGLELPGLRIEAADVDTGIAKFDLQFTLTEAQTPERDPAGITLSVNYASDLFDEQTALRLGHRLARLLAAVAANPTTAVGDLELLDPAEWSGLAPVRGAEPDRPVTFPEVFAAAAAVDRAAIALRADGTQISYDALDRWTNRLARVLMRRGVGPETLVALGIPRSVESVATVLAVAKAGAAFVPVDPNYPAPRIAHMLSDSGAALGITLSAHRDELPGDVEWIVLDDPIFRGLVLDSPDGPIAAAERTAPLRIDNPAYVIYTSGSTGTPKGVVVTHGGLSNFAAETAQRFDVRPGCRVLHFATPSFDAAMLDLLLALGGAATLVITPPGVVGGEDLARVFIDEAITHAFITTSALGTVDPTGVTALRHVLVGGEALPPDLVTRWAPNRNLYNVYGPTETTIVTVISQPMTPGGPITIGGPIRGVSATILDGRLHPAPVGVTGELHLAGSALARGYLNRPGLTAQKFVANPFGKPGERMYRTGDLVRWWTGQGSPAAGRDHGGSREIEYVGRTDHQVKIRGFRIELGEIDAALAKHGGVEFATTIGHRTPAGSTALVSYVKARNGIGLTAAELTEHVAGLVPNYMVPQSIMLLDRVPLSPVGKLDRKALPEPVFSAADGYRAPATPTEVALCAAFAAVLGVETVGADDGFFELGGNSLLATKVVAQVRANGLDLPVQAMFGEATPAAIAARLDGSGAGIVAALGPVLPIRPNGKAAPLFCVHPAIGLAWCYSGLLAHLAPDRPVYGLQAPHVAGEDGFASIAEAAQQYVAHIKSIQPTGPYHLLGWSLGGLIAHEVAVQLQEAGDEVALLSMMDSYRLSDAWLEHAIPSVAEIIEEFGSDQLDAPLDPAMNLRDAAELLRARPGPFAALTVEHLERLYAGYTNGTLLAHGFRPRVFDGDLLFFTAAADEINRADPERTAAAWQPFVTGAIRDHELPCRHSAMTAPESLAAIGQVLRGALDGAAVLLPAGAQPAKNGVRRTKSGARKEKQR

>CORE_REP|Org15_Gene5536#

MADNEGTQTTETTGDANAEVEQQTTATEQSVTEPGGTAKSGGTDMSVAELREWLQRWVADATGQPVEQITVDRPMEEFGLASRDAIALGGDIEELTGVLLNPTIVYQHPTIAALAERVINGEPEAPEEAADDAFYTAGYQPGAAHDIAIVGLSTRLPGAGDTPESTWDFLINRGDGIRELPEGRWSEFLADPDIAAAVENGNTLGGYLDQDAIKGFDAEFFAMSPVEVERVDPQQRLMMELTWEALEHARIPANTLKGESVGVFIGTSTNDFQLVASLGLGKSDPDAPASADAYALTGGSTAIIANRVSYFYDFRGPSVAVDTACSSTLVAVHDAVRALRNGDADVALAGGVNMLLAPAITLGFDSIGAVAKDGHIKAFSSDADGMVRSEGAGMVVLKRLADAERDGDRILAVVKGTAVNSDGRSNGLPAPNPEAQVDVLRRAYRDAGIAPSTVDYIEAHGTGTPIGDPIEADALGRVVGRGREDDKPVLLGSAKTNFGHLESGAGAAALAKVILALQHNVIPPNIGYAGPSPFIPFDQAHLKVVDEPTEFPRYSGTATIGVSGFGFGGTNAHVVIQEYVPAASVESKEEAAQIASAETIEAELDNEATDVLAGAEAILEGSDPLAEPEPVAEVAEWTQERTEPLPVILPVSAYLPSRRRRAASDLADWLESEAGQAAPLEDVARSLAKRNHGRSRGVVLAKTHEEAVAGLRAIAAGKPGPGVFTADSPAAQGSMWVLAGFGSHHRKMGKQLYLENSIFARTVDEIDELVVDEAGYSVKEMILDDAQDWDVGTSQVGVFAIQLGLAALLRAHGAEPAGVVGHSQGEAAGAYISGGLPLEDAVRVICARSRLMGEGEQMITDDQVRNMALVEYSAEDIEKVLPEYPDLEVAVYAAPTNTVIGGPPDQVHAIVARAEAEGKFARVLQTRGAGHTSQMDPLLGELAAELAGIEPTKVTTDLYSTVHKATVYKAGSDPVHDVDYWVTNMRGSVYFTNAIRRAVDAGITTYLELAPNSVALMQVMGTTFAAGVHNAALIPTLKRKEDEAAGVISALAQLYVQGHPVDLVSLLPAGDYADVPRTAFLRKEYWPKVSIATGSGSGRAPGAHVALPDGRHAWEVAASAVTDLAGLVNAAAAQVLSEVALGATIAHSPLPASGTLTTTLTPHPGGASVQVHVREDNVFRLLFDAVVTASAPSTNGTSAPAVQPAPAPAETDSGTADLVVAESFGERWDPNGTQTVEERLATIAAESMGYAVEDLPMEIPLMELGLDSLMAMRIKNRVEYEFDIPSLQVSAVRDASLNEVGKVLRYAIEHRDEVAAMAEKQATEGGSLTVDDNFVAAARAAMEAGEDPAAAVTQQVEAAEPKPVESATPAGSADAVAEAADVDQKASATAGSASQAAEPKVEAADAKDGAADAKTGSGKGTAPAQAAAVFGGGQVAGAKEPEADVPPRDAAERLTFAAWAMVTGKSAGGIFNTLPILDEDTADKLAARLSDRVGSTIDVDDVLDCETIEQLSDIVRRHQDSATEVEGFIRPLRPRPEGSTAIPVFVFHPSGGNTLVYEPLLKRLPEGTPMYGFERIEGSIPERAREYAAEIRKIFPSGPYALYGWSLGAVFALQVAQIMRAEGDDVRLVGLIDLALPVEDEDPSPEGRRARIERFQAFAQKTYGIEGQLDDEMLQELADASDEEQLEIIMGLLKFADVKIPGGVMEHQRTSWLDSRDLQKAQPSHYEGDVTLYLADRYHDGMIELEPRFAERKPNGGWDDYIPNLEVIHIPGDHLQIIDEPRVAQIGADLTRKLAAISTEADDAPGKGEQ

>CORE_REP|Org214_Gene5614#

MTVSSELSSAAWAAGLPQALRGELATLEEAYFRHVDAGDVDCAITGITQIFRRHMELAMHRPAGRALVRVYHPDDSSGLGAAVQVVTDDMSLLVESVTASLSRLGVSVSEVIHPIFEVERDADGRLEQAAPHEVDGNGTAGLRESWMHLQLHPATSRAQLSRIESGLPNVIADVRQVIGDTEAIKDVQSKLAEDLELAAKSGKAPFSDTDLIDTANLLRWLASGNFTVLGYARYRLSSDQEQQSSNALPGTCLGVLRPDVGTDFQVPINALDRPLLILTQGLVPATVHRSVYPYFIGVADFDESGTIVGEHLFIGVFTVTAVHENVLDIPVIERRVRSVIEESGFDLDSFSGQAMLEVIQSFPRTELFSSDDDTMRKTAVAVLNIGLRRQVRLFLRADGYGRFVACMVYLPRDRYTTRVRLEMQEILVRELGGVDIDYSARVAESDLASVYFTVRMPAAEHGAPRYAAAPAADTSEANRLRIQGLLAEASRTWEDHLNDEVSTSTMLDPAVVQRYAVAFPDAYKEDFEADRALADIVRLEHLRDGGIDQYLYRNAGSDPGSWRFSLYVGGAGISLSQVLPVLQSLGVEVVDERPYQLELEPQPGGDPSSGGERWIYDFGLLARPELLRSALDRDLDAELLESSKRAAVLEAEVRGLRERFTEAFEAAWYGRAEADGLNELVLRARLPWRAVSILRAYAKYLQQAGFPYSQANISRVLLTYPDVARLFVDLFGARFDPDTVSAEHAAELETQVRGRIDEVVSLDADRILRAILNLIRATLRTNYYVTDAEGMPRDYLSMKVEPREISELPKPRPQFEIFVYSPRVEGVHLRFGPVARGGLRWSDRLEDFRTEVLGLVKAQAVKNAVIVPVGAKGGFVVKQPPAATGDPVADRQALGAEGVACYRTFISGLLDVTDNVDRATGKVLPPARVVRRDGDDTYLVVAADKGTATFSDIANDVAKRYGFWLGDAFASGGSAGYDHKAMGITAKGAWESVKRHFLEMDIDTQTEDFTVVGIGDMSGDVFGNGMLLSEHIRLLAAFDHRHIFLDPNPDTAASYAERQRMFQLPRSSWADYDAKLISAGGGVWDRTVKSVPISPQARKALGLGDDVESLSPPELVRAILLAPAQLLWNGGIGTYIKSSTESNADVGDKSNDPVRVNGNQLRVKVIGEGGNLGATALGRIEFCRNGGKMNTDALDNSAGVDCSDHEVNIKVLLDGVVSSGELAEPDRNPLLASMTDEVAQIVLEDNVAQNFLMGISRTDAPQMLNVHMRLIDDLEERRGLDRELEALPSEAQMQRMLEEGVGLTSPELANLMAHVKLSLKADLLQTDLPDSAYFTTRLPDYFPTPLRDRFGAAIKKHRLRREILTTMIVNEMVDYGGITYAHRLSEETGATATDSVRAFAAATEIFGLPEMWARIRAADATTSVRDLLELETKRTLDRASRWFLSNRPQPIAVGAEINRYCRDVQELAPKVPGWLRGHHVSTLTDQSAELIARGAPTDLATEVFGLLNLFPLLDIVDIADITDRDGDEVGALYYALNEHLKIDWLLQAVSHLERGDRWHSLARLALRDDMYASLRSLTLDVLSAGDPEESADEKIAYWESKNQSRLGRARAALSELFESGTHDLATLSVAARQVRSMVSGVGAQSEVAAR

>CORE_REP|Org108_Gene3171#

MARQARAEITRDSVLAGAADVFLRLGYANASLSEIIAQSNVTKGALYFHFGSKEELARAVVDQGNERLVSSCQGFFDPRVPALEACIGITYVVADLSMNDPMVGAMLKLTHQIGDYRGAAGDNIAKSWGDTYRLLAERAIAQGDLMPDLDPETIGLLLHGVTTGVHIVAVGTEAIDQMATRMERAWYFLLPAIVPPGEALLLPRVRRPSPAPLRAVILRTGAQPGGPQGGREHAATEERQNGRAGRFARSLQELSMSAPRPPLAGLAAAAGADIALRTVSDLVGTSQVELVAPAAARAFVAGTVATSKPVVVVTATGREADDLTVELTEILGEAVAQFPSWETLPHERLSPGADTVGRRLAVLRRLAHPEDPVFPEPLRVVVTTVRSLMQPMASGLGDIEPIVLRVGTETDFDELLTRLVEFAYTRVDMVGKRGEFAVRGGILDVFPPTADHPVRVEMWGDEVSELRPFSVADQRSLPELSIDVVVAPPCRELLLTEAVRDRAAEVAAANSADAALVEMLEKLAQGIPVDGMEALLPVLRPGALSLLTEELPEGTHLLLCDPEKIRTRAADLMRTGAEFLEASWTAASFGGDAPLGAHGLDLAASAYRSLPEIHDSATEHDLPWWTLSPLTSGDSSEVVLPVQAAPAARGSDELVATIFASLRAHVTTGGRAVVVVAGHGTAQRVLERLADADVPAAALDPGAEPETGVVGVLCGSLHDGVVFEDAGLVVVAESDLTGNRVTAPTEGKRLPAKRRNQVDPLALNAGDMVVHDQHGIGRFVEMIERTVGGARREYLVIEYAPGKRGQPGDRLFVPMESLDQLSRYVGGEMPSLSKLGGSDWANTKRKARKAVREIAGELVQLYAARQAAPGHAFGPDTPWQQEMEDAFAFTETVDQMTAITDVKADMEKPVPMDRVVCGDVGYGKTEIAVRAAFKAVQDGKQVVVLVPTTLLAQQHLQTFTERVAGFPVTVKGLSRFTDPAESREVLEGMASGEVDIVVGTHRLLQTGVRWKDLGLVIVDEEQRFGVEHKEHIKALRTHVDVLTMSATPIPRTLEMSLAGIREMSTILTPPEERHPVLTYVGAYSDKQVTAAIRRELLRDGQVFYVHNRVSSIDKAAKRIRDLVPEARVVVAHGQMNEDTLESTVQGFWQREFDVLVCTTIIETGLDISNANTLIVERADTLGLSQLHQLRGRVGRSRERGYAYFLYPPEKPLTETAYDRLATIAQNSDLGAGMAVAMKDLEIRGAGNVLGAEQSGHVAGVGFDLYVRLVGEAVEAYRAAADGKPITTEETKEVRIDLPVDAHIPPDYITSDRLRLEAYRKLAAAHDDSTLAAVVEELVDRYGPLPVEVGRLVSVAKLRLLAREYGVTEIAVTGTTVKISPLNLPDSKQLRLKRIYPSATYKAASGVVGVPLPRVQDSVGADRLRDVPLLQYLADLLLALDGKAQGAVDLTVATEVSVAR

>CORE_REP|Org42_Gene3301#

MGGAVSGGGVKVGEEGRAAARGELRAAGGVGVVGGERGSDDDGAVQARLDGRLVPAAVGCWVVTVLVLGAGWRVGVMVAVGAAVAAIGLWVGLMWAVAHRRERWRAVAVVALGAVLLGAGFAVAAAWREHRVQTHPLRAVQAGMSVRVVVTPVDDPKPVRGASFGGERTWLVRASLREYQHDSTVVRGGGAVVILATGSAWAKLPPGQPVEFRARPSPPRLRDLTVVTLRALGDPSLAGPLPWWQRLAGSVRADLVAASAAALPAGAAGSLPALVVGDTSALSDEVRRDFETAGLQHLTVVSGANFTILLTVVLFLTRVLTLGPRTTVCVAAGALVMFVVIARPDPSVLRAAAMGGVTLLALLTGRRRQALPALCAAVIGLLAVWPELAMSAGFALSVLATGALILLAPSWSDWLRAKGMWRLPAEILAVSAAAFVVTTPIVVALTGKVSLVAVVANVLVAPVIAPITVIGAAGAVLATAWMPLAELALRCAAPPMWWLLAVAEYCAAVPGATVTVPAGSTGGLIACAVVAAGIWLLRSAIVRRLLAAVLISAVAVLIPVRLWFPGWPPDGWVLAACDVGQGDGLALSAGPGSAVVVDVGPDPRTIRTCLNRLGITRIPLLVLTHPHADHIAGLDGALDGRDIGAVAVGPGELPGYSTDSAAVRQEPGSPLSPQPAPPTHCGSPAGLPERSGTAAYSPPPPSNTADWPPAGLPGVGMRPALGAEPGSQPTTATSASGADRHPPVVDRTDTGPAELAKTVHRVGIPVVELTAGCRLTIGDLTLDVLAPQAPRSRRTAALDLDTANDRSIVLAAHTAAGRILLTGDIEAATQRSLLASGAPIRADILKVPHHGSRTTTTEFLRAVHPRLALISAGATNTFGHPHPAILADLEALGTTIARTDRDGIITVRPNGPALEVRTTGPASAPRSRRPPRRRLPARRERDARPAPGAHQGGRAPPASASVCQPGGAALSPADRPQREVVLDRRRRSCRGSRGRALPTARHRSGFHLVDRHQPSRPGAPIASGGVRAQPGRPAPPIPEPPNAHQSLSDHDRRIGGVSERPAAVHLVLGDEELLIERAIASVTAQVRAGAPDPDGVPVDRLRAGEASTAELAELLSPSLFAEDRVIILESAAEAGKDAVAVITEAAADPPDGVVLMVVHSGGGRAKALAPALHKAGAVVHNCAKLSKASERAEFVRAEFRAAGARVSGEVVQAVIEAVGSELRELAAAASQLAADTGGKIDVAAVRRYYSGKAEVTGFDVAELAVTGDRPGAMEALRWANDRGVPHVLLADALADSVHTIAKVGSAGRGDPFKLAGPLGMPPWKVKKAQAQSRGWTPATIGSALQVVATLNADVKGGAADSAFALEHALMQILDLHGR

>CORE_REP|Org107_Gene6262#

MATEGFVRRPRIAPPRAPGGEVALTPPPEVTRALPAPLMMKLMPVVMVVAVIGMIAMMAMMGRNLLANPLSMMFPMMMLMSMVGMMAGFRGGTGKRAVELNEERKDYFRYLDQVRKDVRRTGNKQLETLVWSHPEPADLPSLIGTRRMWERRPNDPDFGHVRVGMGSHRLATKLARPETGPLEDLEPVSTVALRRFVRTHSVVHGLPTAVSLRAFPAINISGSPEDSRMLVRSMLMELVTFHGPDHLAVAIVCADPDGAWGWAKWLPHLQHPTQRDGMGSARMMYTSLGELETALAAELMERGRFMRNPQPTQGRLHLVVIIDDGYVNGNERLISESGLDSVTVLDLTAPEGGLAARRGLQLIASDGDVSARSAAGVEKFATADMVSPAEAEAFSRTLSRYRLATAAQIVSLGEGSTADPGLMALLKIPDAAQIDPARVWRPRTARERLRVPIGITPDGTPVEIDIKESAENGMGPHGLCIGATGSGKSEFLRTLVLSLVTTHSPDALNLVLVDFKGGATFLGLDSLPHVAAVITNLEEELSLVDRMKDALAGEMNRRQELLRSAGNYANVTDYEKARAAGVPLDPLPALFVVVDEFSELLSQKPDFAELFVMIGRLGRSLHVHLLLASQRLEENKLRGLESHLSYRIGLRTFSANESRAVLGITDAYHLPSVPGAGYLKSDASDPLRFNASYVSGPYVAPQGTVTGEDGTPVGGQRLALFTAAPVEMPAPPEEEEASPLDLPPSPTNPMLELPPPPSALGLPGAPGSDEGIPDSLLDVVVKRLTGHGRPAHEVWLPPLDESPTVDMLLPDPDWRSPVNRHGQLWMPIGVIDKPYEQRRDVLTISLAGAQGNVAVVGGPQSGKSTTLRAIIMAAAATHTPQHVQFYCLDFGGGSMAGLVGLPHVGSVAGRLDSDRVRRTIAELTSLMRQREERFAELGIESMAEFRRRKFAAAAHVPEGAASSGNPLADDRFGDVFLVIDGWAVIREEFDVLESQINAIAAQGLSYGIHVIIGASRWAEIRPVVKDQIGTRLELRLGDPTDSEMGRRTAFQVPVGRPGRGLTPEQLHMLIALPRLDSDSDPSTLADGVSRARQELAELHAGRHAPEVRMLPMQFSRDELLATTRAQGIELSPTKVVVGLGESELQPLVLDFQTEPHFMAFADVESGKTTLLRNIVMGVVENSDPEQAKIIMIDYRRTMLGVVEGEHLAGYSTSSQTCGPMIQEVAEFLSKRIPGSDITPQQLRDRSWWEGPEIYIVVDDYDMVATGGINPFAPLIEYMPQARDIGMHFVVTRRMGGVSRALYDPIIGGLKNMSVDTLIMSGSRDEGKIIGEIRPSKLPPGRGTLASRSKGQEMVQIAYLPPV

>CORE_REP|Org24_Gene2063#

MSAEPCRARHPKEGRTVILLLSTSDTDLLSARASGAEYRLANPARLLPEDLPALLAGADLVIVRILGGVRAWEEGLETLRASGVPLVALGGEIAPDAELMEQSTVPGGVAADAHNYLAAGGPLNLRQLHNFLSDTVLLTGHGFEPPVEMPRWGELERTARPVAADAPTVAVVYYRAQQLAGNTAYVDALCTAIEAAGARPLPLYCASLRAAEPELLARLREADALVVTVLAAGGTKPATASAGGEDEAWDVGALADLDVPILQGLCLTSSRAQWEDNDDGLSPLDVATQVAVPEFDGRIITVPFSFKEFDADGLSAYVPDPERAARVAGIAVRYARLRHIPNADKRVVLMLSAYPTKHARIGNAVGLDTPASAIRLLTEMRAAGYDLGAPGEIPGLEQGDGDALIHALIAAGGQDPDWLTAEQLEGNPIRIGADTYTAWFDTLPDDLRENVIEAWGPPPGELYVDRSADPKGEIVIAALRFGNVVLIVQPPRGFGENPVAIYHDPDLPPSHHYLAAYRWLAAPEGFAADAMVHLGKHGNLEWLPGKTLGMSASCGTDAALGDLPLIYPFLVNDPGEGTQAKRRAHATLVDHLIPPMARAETYGDISRLEQLLDEHANISALDPAKLPAIRQQIWTLMRAAKMDHDLGLEERPDEDVFDDMLLHVDGWLCEIKDVQIRDGLHVLGQAPAGETEVDLVLAMLRARQLWGGEVSVPGLREALGLSESGDESRNRVDSFEARARALVAALQAADWSVDAIDAIVDAQITAGGTGDNAGATPALADTAASGDAAAPGAALAGDTEKRGDSVAAEAGRSAAGGTTVVPGNAVAPGGAAASAGSTAPAVGPDAVRAVLRFAATEVVPRLRQTEVEIQRVLHALAGGFIPAGPSGSPLRGLINVLPTGRNFYSVDPKAVPSRLAWETGQAMADSLLERYLADHGEYPRSVGLSVWGTSAMRTAGDDIAEVLALLGVRPVWDEASRRVTTLEPIALDELGRPRIDVTVRISGFFRDAFPHVLALLDDAVRLVAGLDEPAESNYVRAHAQSDLAAHGDERRATTRIFGSKPGTYGAGLLQLIDSKSWRTDDDLAQVYTTWGGYAYGRDLDGAPAADDMRSAYRRIAVAAKNTDTREHDIADSDDYFQYHGGMVAAVRALTGKNPEAYIGDSTRPDSVRTRTLSEETTRVFRARVVNPRWLDAMRRHGYKGAFEMAATVDYLFGYDATTNVVADWMYEKLADSYVFDEVNRKFMEQSNPWALHGIAERLLEAAERNLWERPEDETLARLKQIYLETEGELE

>CORE_REP|Org134_Gene4052#

MDPRARSARAGHEAGSAVSDTASWRPELASDSVEPAADAEEAGTEPAQPGTDSAPTGGPAVDAAPGPGTSTGPDASAGSDTTTGPGAAARPATSGDGAAPSADHPEELDTQENNPIAIEDELEEMGLEGAREIGHGGFGVVYRCVQRALDRVVAVKVLSSDIDAESRERFLREEHAMGRLSGHPNIVDILQVDVTATGRPFIVMPYATRGSLEVVVRDNGPLGWSDTLRAGVKLAGAIESAHRAGILHRDVKPANILLSSYGEPQLTDFGIARVPGGFRTSSSMITGSPAFTAPEVLKGDEPTVRSDVYGLGATLFALLTGHAAFERQAGEKVVAQFLRITTQPVPDLREQDIPADVAAAIEQAMAQNPRDRPASAYEFGEMLRAIQRTHGQMADEMALLDTEDEAEAAAAPSGNRTGPAVTARRSWPLNLSPPTPRPGYDPAPTTTFPPTAATKFRPPTPAREPVQRTRLLDILRTGGRRRLALIHAPAGFGKSTLAAQWRGELTADGVAVAWIGIDSDDDNEIWFLAHLIEAIRRVRPDIGTGLDQVLEEQPADAVAYAITTLIDDVHAGGATVVVVVDDWHRITDPGTRRVMDSLLDNGCHHLRFVVTSRDQSGLPISRMRVRDELVGIGSAELRLTREETRQILVDRNRFTLDDAQIDELHRATDGWPAAVQLISLALRGNPDPDPLIQHLAEGGHGVREYLAENVIDALEPRMVDFLTAISIAEKVSGSLAAALSDDPEAEHLLEQAEQRELFVRRVEYDPEWFRVQPLFAEHLRARLERTDPARVKVLHRKAARWYAEHQLLRKSVDHAVSATDLKMALDLLESGGMDLIDGSRLATLLGTVSKLPVQQVASRSKLLMAVARANVNLQQSGAARSALGRLSSVLSRGSSGDADVVRQRCQAAVLAAADQVARDHTEGVMDQIGDCLDHPDELPAWTVSTAANLTSFVRLCEFDFDGARSIQDWAAEYHERSKDPLGSVFGLCSRGAVAFEQLDIATAARCFQQAWDTARARSGQRSHAVRVAAALLGELHYRRGELDAADRLLDESHELVARVGPIDFLISTFVIGARVKAVRGDMATAASRLAEGQRIAVEQDLPRLAAQVRAERVRLGPAAETSGPQTATERWNGNVIRDTGSTGHRLTGTAALTAEAEEIAAIRELLADGGIDDQDRAVRRARALYGRTHELHRPRAQLDTSLLLAECLAAAGWVGEAAAQLVPAVTTCAELDWTRPLLDAGPGVVAILRVLRNDLPSELPIRFVDELLA

>CORE_REP|Org45_Gene3532#

MHIHRAERADTLVDALAQLLARPLDDPFAAEVVAVPAKGIERWVIQRLATVLGSATGGDGIAANIEFPDPASLVGAVLAEATGLTPDTDPWAPERLVWTLLPVLDAVLAEPWCGVLSRHLGRGDAAGHKVGRRYATAARIADLFDGYGTQRPGMIAAWADGLDTDGTGRELPEDLRWQPRLWRTLREAVGVPSPAERLSAACAHLRAEPGVVSLPRRISLFGVTRLTTDQLEVLSALSAGREVHLWLVHPSPTLWTELASNGADAQADSADRSGSNAAGDTSASVRAVSDRERVDRADGSSPSSEMPDSLVRVRHPLLAALGRDVRELQQRLHAYDHTDTYHPPAAISPGTTASPGRAERSGARTVEAASDTPELGRPVDSAAGHVIEPTADCAEGGATEFSDVAGSDRGDRAVAAGFVAAATPAVGTGESATGRRSVERGAVAGASSVRGVASGRTLLSALQAAIREDRWPVAVEPELAGDGTVGVHACHGPARQVEVLRDCLLHIFAADHTLQPRDVVVMCPDVESYAPLVRAAFGQWSLDTAEAGHPGHALRVRLADRAQRAVNPLLGVIGTLLELADGRVTVTEVLDLAAAEPVRLRCGFDDDDLERLREWAAETGARWGIGQRQRQAFGLGDFAQNTLNAAVDRILLGVAAGESGADWLDLALPLDDVDSGDIDLAGRFAEFVDRLAVCLRDLRGPTLAEPAGTARPAGEWAAVLGRALDLLTDVTRAQAWTGVQARRELAAALEHAGDVPLRLPDIAALLATRLAGRASRANFRTGELTVCTMVPMRSVPHRVVVLLGLDDEVFPRAGGIDGDDVLARHRCPGDRDPRSEDRQLLLDAIMAAGERLVLLHTGSDPVTGAHRPPAIPLAEVLDTVRAHVGRAAMARIVRRHPLQPFDAANFRADDPFSFDPVALAGAVAARRPQRSRPVFLAAPLPSAAPGDVELTDLVAFAEHPVRAFLWQRLGIRVPEEEEEIDDRLPIALDGLTKWSMGERMLAARLNGVEADMLRAAEWRRGTLPPARMGAAVLDVIEGTVDQLVRVARPMHEIPGRTIDVAVDLGDGRRLTGTVADVHDDALLRATYSRLAPKHRLAAWVRLLALAASGSHQSWRALTLGRGQFRNPVWQSTLTAPDAATARAILRQLVHLRDAGLTEPLPIAPSATAVYADRRCKGASVDDATLSAEQDFDSAYGERTDRYLRQVWGPALRFPDLTRAPAEHGGDEPTRFGELARMLWNPLLANENQGRP

>CORE_REP|Org176_Gene3205#

MSTASFVHLHNHTEFSMLDGMAKIGPLFAEAERLGMPAVGMTDHGNMFGAAEFFTHASKTGITPIIGIEAYVAPESRLSTRRVFWGEPGQRADDVSGSGAYLHMTMFARDATGLRNLFELSSLASIEGQLGKWPRMDAELIAAHADGIIATTGCPSGEVQTRLRLGQFDDAYEAAGRWREIFGPDNFFLELMDHGLSIERRVREDLLTIGDKTGIPPLATNDCHYVLPGQAAAHEAMLCVQTGKTLSDPTRFRFGGNGYHLKSAAEMRALWDDEVPGACDATLAIAERIEPYDEIWRFRDRMPVFPVPDGHTEDSWLRHEVDAGLRRRFPGTVPDTYRDRAAYELDIIRDKGFPAYFLVVGDLVAHARAVGIRVGPGRGSAAGSLVAYALAITNIDPIEHGLLFERFLNPERPSAPDIDIDFDDRRRGEMIRYATDRWGADRVAQVITFGTIKTKAAIKDAARVNYGQAGFAVADRITKALPPAIAAKDISVAGIMDPAHERYAEAAEVRALIESDPDIRRIYDTAAGLEGMVRGAGVHACAVIMSSEPLIDVVPLWRRPQDGALITGWDYPSCEAIGLLKMDFLGLRTLTVIGDCLDNIRANRGLDIDLDTLTHTDPNTYAMLARGDNLGVFQMDSTGMRELLLRMAPTEFNDLVASNALYRPGPMGVGAHWAYADRKNGREAITPIHPELDEPLRDILGETYALIVYQEQIMQIAQHVAGYSLGQADLLRRAMGKKKPEVLAMEFENFRNGMRGNGYSDAAVTALWEAVLPFAGYAYNKSHAAGYSLIMYWTAYLKANHPAEFMAALLTSIGDDKDKSALYLADCRKRGIRVRPPDVNSSAATFTSVGTDIRFGLGAIRNVGGPVVEAITRARDTGGPFTTFSDYLDRVDATACSKKVTESLIKAGAFDSLGHRRKGLHRIHDDAIEAALGVKKAAARGQFDLFAADDAEDETASVFHIAVPDEEWEKPQLLAFEREMLGLYVSGHPLDDVAGALAAAVDAPIATVLAGSVPHGRTVALGGMIAQVERRITKKGDPWAVVRLEDLDASTEILFFPAAYARAATTLIEDAVVLVTARVDIRDGRRSLIADALTVPELARAGGTPLTLSLRTDTCTRDNVRALREILQRHPGNTEVQILYHARTGPARYGLDPALRVHLSSGLLGDLKALLGPAAVAAGSTVTAVNGAYGPGTSSVRGGRITSTSAIGPA

>CORE_REP|Org80_Gene4825#

MGWSNGPPSWSELERVLSGRPGRHDPDSMYPGDGGDSPAWSRKRGEYLADGDDVRPLGPVVPYAELHAHSAYSFLDGASHPEELVEEAARLGLEAIALTDHNGFYGAVRFFEAAREWDIATVYGAELTLAATESRSPVDPPPHPSRPARAGLRSLPVDAESASAEPSPRDTAAPRPHSGAPDSAARTGEPDPPGVHLLVLARGQEGYRRLSREIAAAHMAAGEKGILRYDLDTLTAAAGGHWQILTGCRKGALRQALEQDLRAGSEPVRAEAALRDLVERFGAERVSVELTHHGLPADDERNAHLIALADRLGLPVVATTGAHFASPAQRHRAMALAAIRSRSSLDEMAGWLAPTGGAHLRSGAEMARLFADCPQAVANAVALARECAFDLKLIAPELPPFPVPEGHDENSWLRELTLQGAAERYGAPQENPAAYRQIEHELAVITGMKFPGYFLVVHDIVTFCKNNGILCQGRGSAANSAVCYAIGITNVDPVRNNLLFERFLSPARDGPPDIDIDIESDRREEAIQHVYHAYGREYAAQVANVITYRGKSAVRDAARALGFSTGQQDAWSKQVSRWTGVGAETGTDIPERVLELAADIEGLPRHLGIHSGGMVICDRPIADVCPVEWARMPGRSVLQWDKDDCAAAGLVKFDLLGLGMLSALHYMIDLVREHEGVTVELHKLDLAETAVYEMLSKADSIGVFQVESRAQMATLPRLKPREFYDLVVEVALIRPGPIQGGSVHPYIRRRNGQEDWDFDHPSLQKTLARTYGVPLFQEQLMQIAVDVAGFTAAEADQLRRAMGSKRSPERMERLKARLYQGMRDLHGITGEVADRIYEKLYAFANFGFPESHSQSFAALVFYSAWFKLHHPAAFCAGLLRAQPMGFYSPQSLVADARRHGVVVHGPDINASRAEPTLEQRGTQVRLGLAAVRYIGAELAEKIVAAREESGPYTSLLDLTGRVELTVRQAESLATAGALDSVTTVASTRNGSPSGAAIPVRAATNRTPPASIPARSEPPPGGAAPGASSVRRTALWAAGAAAAERSDRLPGLVAAAEAPALPGMSALELAAADVWATGISPGSYPTEFLRAELDAMGVIPADRLLSVPDGSRVLVGGAVTHRQRPATAAGVTFLNLEDETGMVNIVCSVGLWTRYRRLAQTARALIIRGRIQNAEGAVSVYAETLRQLDLRMAGKSRDFR

>CORE_REP|Org31_Gene4357#

MFTKVLVANRGEIAIRAFRAAYELGVGTVAVFPYEDRNSVHRLKAAESYQIGEQGHPVRAYLSIDAIIDAAKSAGADAVYPGYGFLSENPDLAAACAREGITFIGPSAEVLELAGNKARAIAAAKAAGLPVLRSSVPTADVDELLAAAQELEYPIFVKAVAGGGGRGMRRVAEPAQLRESIEAASREAESAFGDPTVFLEQAVVNPRHIEVQILADQHGNVMHLFERDCSVQRRHQKVIELAPAPNLDLALRDRICADAVAFAKQIGYSCAGTVEFLLDERGNHVFIEMNPRIQVEHTVTEEITDVDLVQSQLRIAAGETLEQLGLSQDKITIRGAALQCRITTEDPANGFRPDTGRITAYRTPGGAGIRLDGGANLGAEIGAYFDSMLVKLTCRGRDFPAAVARAGRALAEFRIRGVTTNIPFLQAVLDDPDFKTGRVTTSFIDERPQLLTLRGSADRGTKILNYLADITVNKPHGERPTTVYPHDKLPPIDLTVPPPDGSRQRLLRLGPEGFARDLRAQKAVGVTDTTFRDAHQSLLATRVRTNGLLGVAGHVARLTPELLSIEAWGGATYDVALRFLYEDPWERLALLREAVPNICLQMLLRGRNTVGYTPYPEQVTRAFVSEATATGIDIFRIFDALNNVDQMRPAIDAVRETGTAIAEVAMSYTGDLSNPDETLYTLDYYLKLAEQIVDAGAHVLAIKDMAGLLRAPAAATLVKALRSNFDLPVHVHTHDTPGGQLATYLAAWQAGADAVDGASAAMAGTTSQPALSAIVAAAAHSEHDTGLNLQNVCDLEPYWEALRKVYAPFESGLPAPTGRVYTHEIPGGQLSNLRQQAIALGLGDRFEEVEAKYAAADRLLGRLVKVTPSSKVVGDLALALVGTGVDIEDFAADPGRFDIPDSVIGFLRGELGTPAGGWPEPFRSRALAGRGPAKPETPLTPADEAGLAGSSEQRRTTLNRLLFPGPTSEFLAHREKYGDTMGLSANQFFYGLRHGEEHRVQLEKGVTLLIGLEAIAEPDERGMRTVMCILNGQLRPVAVRDRSIASDVPAAEKADKGNAGHIAAPFAGVVTLAVSEGDAVAAGDTIGTIEAMKMEAAITAPRAGTVGRVAIGQVQQVEGGDLLIELTMGESGAGDQLRSVRNDQSE

>CORE_REP|Org63_Gene886#

MRGSQIALRTVPDAATAVLTKPPAAPVVTRNDFRTARLIALVAGLLGALFALATPFLPVTQTTAVLNWPQGGTLGNVQAPLMSQVPIDLKATIPCETIAQLPERGGMLLATAPPQGDRAALEAMFVRVSETSVDVVDRNAVVVSADRSRMGECAALSISSDSERTYAVFTGLTKQVERPVEGGAPGATELATVPVEGQLGGDLRPQVVGVFSDLKGAAPAGLAFDMTVDTRFSSSPTAIKLVAMIAAVLCTLIALAALARLDGSDGRGHRRFLPANWLKPTWADGAVAGTLLLWHFAGANTSDDGYILSMVRVAPHAGYMANYFRWYGVPEAPFGWYYYVIQVFSEISTASPWVRLPALACAILCWLVISREVVPRLGRGVRTSKVALWTGGLVFLAFWLPFDNGLRSEPIVALGALLTWVSIERAIATGRLLPAAVAILVAAFTLAAAPTGLMCVAALLAGIRPLVRIVVRKHRQFAALGAGRWGSTLPLLAPIAAAGVLVLTVVYSDQTFAGIQEANRVRQVTGPNLAWYEDYLRYYYLFVETVDGSLSRRFAFLVMLLCLFTTMLVLLRRRQVPGIASGPTWRLMGVVFGTIFFMMFNPTKWTHHFGAYAGIAGSLAAVTAVAVSASALRARKNRAIFLAGLLFVLAVAFSGINGYWYVSSFGVPWFDKRISLQGYQSNTVMLMLFGLALALVGWYALREDYTKPQPSAKTARGRRIRRFAAIPLTVVAALMVALEVLSLVKGAVSQYPAYSLARSNIDALGGSTCGLANDVLVEPDPNGGRLEPIIDPARPLTDPLAGVDSVGFDPNGVPNDLSADSVEVKPGTGNTSTQSVGAAFAEGQSAGTGGGQGALGVNGSTVALPFGLDPASTPILGSYQNGMQQPANVTSSWYQLPARSADKPLVVISAAGRILSFDDTGAMKYGQSLTVDYGKHLPDGTVQKLGTYLPRDIGPFPSWRNLRVPLDEIAPDADAVRIVANDPILIGDQWLAFTPPRMPKLQTLNSLLGSQQPILLDWAVGLQFPCQRPFDHENGVAEVPGYRILPDRPLAISSTNTWQAEEFGGPLGFAQMLAKSTTVPTYLKHDWARDWGSLERYDQYDRNAVPAKLDTGTTTRSGLWSPGNLRVF

>CORE_REP|Org113_Gene3340#

MPRRNDLQHILVIGSGPIVIGQACEFDYSGTQACRVLRSEGLRVSLVNSNPATIMTDPEFADSTYVEPITWEFVEKVIVAEKAKGTPVDALLATLGGQTALNTAVALHENGILEKYDVELIGADFEAIQRGEDRQKFKDIVAKVGGESARSKVCYTMDEVRETVAELGFPVVVRPSFTMGGLGSGMAYNDDDLDRIAGGGLAASPTANVLIEESILGWKEYELELMRDGRDNVVIVCSIENVDPMGVHTGDSVTVAPAMTLTDREYQKMRDLGIAILREVGVDTGGCNIQFAVDPRDGRLIVIEMNPRVSRSSALASKATGFPIAKIAAKLAIGYTLDEIVNDITKETPACFEPTLDYVVVKAPRFAFEKFPGADPTLTTTMKSVGEAMSLGRNFSEALGKVLRSLETKAAGFWTQPDGRWTDVAEVLADLRVPIEGRLYQVERALRLGASVEEVAEASGIDPWFVAEIAGLVELRGEIAQAPVLDEPLLRFAKHNGLSDRQIAALRPELAGEDGVRELRHRLGIRPVYKTVDTCAAEFEAKTPYHYSTYELDPAAESEVAPQPDREKVLILGSGPNRIGQGIEFDYSCVHAAQTLSEAGYETVMVNCNPETVSTDYDTADRLYFEPLTFEDVLEVYHSESESGRVAGVIVQLGGQTPLGLAQRLTDAGVPVVGTSAAAIDLAEDRGEFGQVLVAAGLPAPKYGTATTFAQAKEIAARIGYPVLVRPSYVLGGRGMEIVYDESSLEGYISRATELSPEHPVLVDRFLEDAIEIDVDALCDGEEVYLGGVMEHIEEAGIHSGDSACALPPITLGRSDIESVRRSTAALAQGIGVKGLLNVQYALKDDVLYVLEANPRASRTVPFVSKATGVQLAKAAARVMLGTSIAQLRKEGILPAEGDGGHAPMDAPVAVKEAVLQFHRFRRPDGTGVDSLLSPEMKSTGEVMGIDTDFGTAFAKSQSAAYGSLPTEGTVFVSIANRDKRAMVFPVKRLHDLGFRILATEGTAEMLRRNGIPCERVRKHSDPEFPAGSAGAADEAPVPSVVDQIKDGEIDIVFNTPYGNSGPRVDGYEIRTAAVGANIPCITTVQGAAAAVQGIEATIHGGIGVRSLQELHAVLRGHEER

>CORE_REP|Org23_Gene2846#

MRGAEGGEVLDYHALNAMLNLYGPNGEIQFDKDREAAHQYFLQHVNQNTVFFHNLDEKLDYLIDENYYEREVLDQYSREFIKSLFQQAYAKKFRFPTFLGAFKYYTSYTLKTFDGKRYLERFEDRVCMVALTLAAGDEQLARELVEEIIDGRFQPATPTFLNSGKKQRGEPVSCFPAGTPVDTIDGPRAIETLRPGEKVLSHDGSYRAVEALIENPNDQALVEISHFGHKEPIRCTPEHPILVWTTREVDTLIDGDGADPFNGFVWLAARDVQPTDFIVTTAPLGERDRRVFDLMEYTGEGVYEEVDGQIRKVNTDAKHRNKQRHNQRFVPVNRFVEESYELGLILGWYVAEGHVSKRSTDVSVPNGVHFTLGAHEVEYHVELGMAFKQVFGVDLSLHTNHSDQSTRMVCNSKIVASLLLSLAGTGYNAKRLSADVLTADEDFQRGLLAGLFRGDGCSTSGGMMLDLVNPELIDQVQLILRRLGIMSVVRSYINQAGNVTGQVFVPGLPGNNEDFIFDIGKNLHSYVGRKGVKRTTYQVVHGRHVYGVRSIGRTTETPEKVYNLHVEGTHTYTIRGTVVHNCFLLRIEDNMESIGRSINSALQLSKRGGGVALLLSNIREHGAPIKKIENQSSGVIPIMKLLEDSFSYANQLGARQGAGAVYLHAHHPDIYRFLDTKRENADEKIRIKTLSLGVVIPDITFELAKKNEDMYLFSPYDVERIYGKPFADIDVTEKYYEMVDDKRIRKSKIKAREFFQTIAELQFESGYPYIMFEDTVNRANPIAGKITHSNLCSEILQVSTPSEFNDDLSYSKVGKDISCNLGSLNIAKTMDSPDFAKTIETAIRALTAVSDQTHIYSVPSIEQGNNQSHAIGLGQMNLHGFLARERIHYGSEEGIDFTNIYFYTVVYHALRASNRIAIERGSYFGGFPESKYASGEYFDKYTDQVWEPKTDRVRQLFDEAGVHIPTQDDWRELKASVMEHGIYNQNLQAVPPTGSISYINHSTSSIHPVASKIEIRKEGKIGRVYYPAPYMTNDNLEYYEDAYEIGYEKIIDTYAAATQHVDQGLSLTLFFKDTATTRDLNRAQIYAWRKGIKTLYYIRLRQMALEGTEVEGCVSCML

>CORE_REP|Org101_Gene4427#

MQAGREGAVRALISAQPGYLEESPFVSVYLYKWGKFAFRRKWIVLPLWLVLLGALGAGSHLLSKSMSDEFSMPALPSERATEILDKQFPGMSAQFGIDAVSGTYVLAAPAGTKLTDKDNSAAIDALITDLRALVVGDDRHRLVAEKAGAALQNPVAATQAMGCLTTADPAACAGAPLNVLSKDAPATVAVLTVPFDIASSMDITDEQRQLAYTVADPARARGLVVELGGSIAREQEQPSGQAELIGMGVALVVMVIAFGAIMAAFVPIVTAIVGLGAAMLVISLSTAVVEVPSFTTFLASMIGIALSIDYALFIVSRYKHELRVAANPEEAAGISVGTAGSAVVFAGLTVIVALGALSIVGVNFLTFMGLGGAVAAFFAVLTAITLMPALLGAFGRFLFKPKLPLVARHDPEDDTSVTNGMRVGRLIGKRPWLALIVAVAALAALAMPALQLQLGLPGEDSLPTESTARQAYDIRTNGFGEGSNGVLTVAADLEQVPEGERKAAVTALRDRLAEFPEMDYVTTPQFSANGLGAMLSGVPKSGPNDQDTKDLVRDARAAEGELSERYGIRYGITGTTAIYADMDHVLLGKIVPYLAIVAGAAFVLLILVFRSILVPLTAALGFLLSMAATFGATVLIFQEGTFGLIADPQPIVSFLPIMLIGLVFGLAMDYQVFLVTRMREEFVHGKSPRDAMIAGYHHGARVVTSAAIIMISVFGSFLLETDVTAKSMGFALAAGVAIDAFVVRMVLVPALLAIMGKASWWMPKWLDRILPDIDVEGAKLRALPRKRSGAAELEPVGAEPAPALVAVNTVAVQQLPDVESGQFVASSGRTIFGRVCRDDGYPVPDAALTLIDQRGQQVSRASADGDGNYAIEPPAPGGYVLIVSANRHQPAAVNVTVAEGGAHHVDVTLQGSGELSGVVRTAAREPVAQATITVTDLRGEVVGVAVSAADGGYACKGVLAGTYTLVAVAARMRPTATTLTVPESGRLRFDVELAPMAMLWGTVRANGRAVYDARITVLDWSGTIVGTAHTDEDGRYAVADLPEGDYTVEARGYPLVTGQVTITGSQVDHDVRLGFDIDEHAEMS

>CORE_REP|Org19_Gene3335#

MADETSTRNAYPRVDFTAADAGSRQGASVSFPELERRVLDYWAADDTFRASIDNRAEGCDEFVFYDGPPFANGLPHYGHLLTGYVKDLVPRFQTMRGKRVERRFGWDTHGLPAEIEAEKQLGITDKSQIDAMGLAEFNAACKSSVLRYTNEWRDYVTRQARWVDFDNDYKTLDLDFMESVMWAFKSLYDKGLVYQGFRVLPYSWYEQTPLSNQEARLDDAYRMRQDPAVTVDMLLEVAADHPLHELDGANALIWTTTPWTLPSNLAIAVHPDITYAHVRGKDGKRYVLATERVSHYARELGTADDGLEVLSEHSGAALAGLRYRPPFDFFLGHPHAHRVLNADYVTTDSGTGVVHLAPAFGEEDMDVASANGIEVVQPLDAGGKFTSMVPPYEGLMVFDANPVIIKDLKAAGKLLRHETIEHSYPHSWRSGQPLIYMAVPSWFVAVTKFRDRMVELNQQITWVPEHIRDGQFGKWLENARDWNISRNRYWGAPIPVWVSDDPAYPRVDVYGSLDELERDFGVRPADLHRPGIDELVRPNPDDPTGKSMMRRTPEVLDCWFESGSMPYAQVHYPFENKEWFDGGAVTSDTAGTSVARAHSPGDFIVEYNGQTRGWFYNLHVLSTALFDRPAFKSVVAHGIVLGDDGLKMSKSKGNYPDVNEVFDRDGSDAMRWFLMASPVLRGGNLIVTERGIREGVSHALRPLWNAWTFLQLYASKPGVWRTDSAHVLDRYILAKLAAARDVMTEALEVYDIATACDELRSFADALTNWYVRRSRSRFWEEDRDAIDTLHTVLEVTCRLAAPLLPLITEVIWRGLTGERSVHLTDWPGETELPRDPELVSAMDEVRSVCSTVLSLRKAQNLRVRLPLSEVTIAAPDAERLRPFADLVADEVNVKKVDLTTDVAAHGRFELVVNARAAGPRLGKDVQHVIKAVKSGQWREDDGVVKALVPGHDDGIALLPEEYTQRLVAAEPESTAALPGNAGLVVLNSEVTEELEAEGWARDLIRDLQETRKSLGLDVSDRITVVLEVPQERAEWARTHRDLIAGEILATALDFGAAGAGAADVVGGVRAAIAKA

>CORE_REP|Org53_Gene3008#

MVRPPSARSAVPGVGRLGLLDPTAAASLRELGWDNVESIPVLWALSRAPDADLALNTLMRLREALGSDWQRLDSAIRTDTSLRGRLFALLGSSTALGDHLVAEPAAWEVLRRGDLPDRDELLADLLAAVQATPEAGPHAGPMLFRAGIAGPEAVALLRCRYRDQLMLLAALDLAATVENEPVLPYRVVGRHLTDLADAALTAALAVAVARVCKDQPCPVRLAVIAMGKCGARELNYVSDVDVVFVAEPADATATRLAAEMMSVGSQAFFEVDAALRPEGKQGALVRTLDSHLTYYKRWARTWEFQALLKNRPMTGDLELGREYRDAVMPMVWTASERPDFVPEVQGMRRRVEDLVPAELRERELKLGRGSLRDVEFAVQLLQLVHGRVDENLHVASTVDALSALAAGGYVGRDDAANLTASYEFLRLLEHRLQLQRLKRTHTLPADDDEEGMRWLARAAHIRPDGRQDAMGVLRSEIRRNAVRVRRLHAKLFYRPLLEAVVRMDPDALRLSPDAAVRQLAALGYAAPENAFGHLKALTGGVSRKGRIQALLLPTLLEWLGETPNPDAGLLAYRRVSEALDEQTWFLRELRDEGAVAQRLMIVLGSSEFLPDLLINAPETIRMFADGPHGPLLLGPQPEEVARGILTAAARYDDPNRAVAAARSLRRHELARVASADLLGMLEVPQVCRALSSVWVAVLDAALAAVIRAGEAESGEPAPAAFAVIGMGRLGGMELGYGSDADVLFVCEPRPGVDETKAVKWANTVAERVQRLLGAPSTDPPLHVDAGLRPEGRSGALVRTLSAYQAYYGQWAQSWEVQALLRAHQVAGDQELGVRFLHAVDKVRYPAGGVSEDAVREIRRIKARVDSERLPRGADPATHTKLGRGGLADIEWTVQLLQLRHAHEVESLHNTATLETLAAIEKAELLAAEDVALLRDSWLLATKARNALVLVRGKPSDQLPGPGRLLSAVATVAGWPNNDGGSEFLDHYLRITRRARAVVERVFGS

>CORE_REP|Org144_Gene5995#

MKFSAKDLVGSVQRLMATAQNGLEVIRFGGLTHDVESSPFEVVERRRMYRLRHYFPDDTTPDRPVVLLVPPLMVNADIWDVNAEDGAVGILHRGGIDCWVVDFGSPAKEEGGWERDLADHVLAVSSAIDAVTEATGSSVHLMGYSQGGMFAYQVAAYRYGKGVQSIVTFGSPVDIVAGMPFGLPYGMVSDVADFLADHVVTRLPITDSMVRIGFQMLDPMKTAKARIDFLRQLHDREALLPKERQRRFLNSDGWVGYAGPAAADLLKQFVAHNRLMLGGFVIRDHPVSLAELKCPILAFVGEVDDIGQPGAVRGIVRAAPNAEVYEATLVAGHFGLVAGSTATNHTWPLVRQWVDWLERDTLLPPEIHPMVDQVETNRPRSAATRVVHTAASLAEAGAGVGKALEGIANNTVRGSVELAGEAARALPRLTRLGMIQPHTRISLGRLIAEQGRRAPLKDLFLFDDRVHTNAAVNVRIDNVVRGLISVGIRPAMRVGVIMETRPSALATVAALSRLGAVAVLLAPGSELVRALELTGVDTVVADPENLRHAAETGARVLVLGGGDARQLDLPANGRVIDLEQIDPAQVKLPGWYRPDPGLARELAFVLVTGTGDRLETKYITNHRWALSAFGTATTADLNRRDTVYCLAPLHHSSGLLVSLGGAIAGGSRIALARSLDPARFAEEVHRYGVTVVTYTWTMMRDILDAEVFPTGPYSPTPSASPTANPQWHPIRLFIGSGMPAGLWRRTAEQFEPARVVEFYASIEGDVVLANVKGAKRGCKGRPVPGTARVELVAYDPVTEEIQTDEAGYARRCADNEVGLLIGKATEGVDISAGGLRGVFAPGDSWMPTENLFRRDADGDYWLIDRKDTVIHTRRGPVFGQPIVDVLNDITAVDMEVAYGLAVGDHCIAVAAVCVRKGLRLEPKDVTEALRALDPDQRPDVVYVVDEIARSASYRPSTRAVQAAGRPEPGPDTWWYNRASDAYEILTEQDAAAVLGDR

>CORE_REP|Org163_Gene5077#

MSTPVRGADLTDLIHSAAPAKSAGTNPAPAPAASRDPNGSHPPQPGGRVRVIREGVASYLPDIDPEETSEWLESFDEMLDREGPGRARYLMLRLLERAGERRVAIPSLTSTDYVNTIPTENEPWFPGDEEVERRFRAWIRWNAAIMVHRAQRPGIGVGGHISTYASSAALYEVGFNHFFRGKDHSGGGDSIFIQGHASPGIYARAFLEGRLSSDQLDGFRQEYSHGGPGHGLPSYPHPRLLNNFWEFPTVSMGLGPMNAIYQARFNHYLHDRGIKDTSDQHVWAFLGDGEMDEPESRGLAHVAAMEGLDNLTFVVNCNLQRLDGPVRGNGKIIQELESFFRGAGWNVIKVIWGREWDALLGADRDGALVNLMNSTPDGDYQTYKANDGAYVRDHFFGRDPRTKALVQDLSDQEIWNLKRGGHDYRKVYAAYAAAMAHKGQPTVILAKTIKGYTLGKHFEGRNATHQMKKLTLQDLKDFRDLQRIPISDAELEKDPYLPPYYHPGMEAREVQYMLDRRKALGGFLPERRAASKPLKLPGDEAYRSVRKGSGKQNVATTMALVRLMKELLRDKEIGKRIVPIIPDEARTFGMDSWFPSLKIYNRNGQLYTSVDAELMLAYKESAVGQILHEGINEAGSTASFTAAGTSYATHGEPMIPLYIFYSMFGFQRTGDGLWAAADQLARGFVLGATAGRTTLTGEGLQHNDGHSLLLASTNPAVVTYDPAFAFEIAHIVRDGLRRMYGGGTPPQGAAPLPGTHPHGSAGEFGGEDVFYYITLYNEPYPQPAEPEGLDVAGLLKGIYLYKRGGEGAVRAQILVSGVTVPDGLRAQALLAQEWGVQADVWSVTSWGELRKEALDKEIAALRNPGADPGVPYVTEALSRADGPYVAATDWMRAVPDQVRKWVPGDFTTLGTDGFGFSDTRPAARRVFNVDAQSIVVAALAGLGRTGGIDPAKAVEAAAKYRIDDVDAAPKPAASAEEELA

>CORE_REP|Org125_Gene1111#

MEIQAVTSPYDDGPNGGRPPRGPQSGPGGQPPRPAGGNPPGARPLPPRRQAPPPGPGGPRGGQPGGPPNPAGGPPRRPGPPPGGDRTPPMRGPAGGPPRTGGQPTVRGGQPNPAGGPPRRSANPAPRPGAGATQKIAKPGEQKPQATQKIAAGTLGEAMAQRGPRSTAANRSAPGGAGTRSGSGPGTGGRRAVAGGTPPSGPPPRKGNGGGDGPGGSAGKGPKTKKKSPWRIVRRVIYVLVALAIVVPSAVFLIAYTTVSIPQPGDLKTPQVATILASDGTTQISKIVPPEGNRTDVTIDQIPPHVRNAVIAAEDRDFYSNPGFSISGFARAARDNLMGKDTAGGGSTITQQYVKNAMVGNQHSLSRKMRELVISAKMARQWSKDDILTAYLNTIPFGRGTFGIDAAAKAYFGKSVEQLTVEEGAMLAATINQPYGLDPENNPKGAEQRWNYVLDGMVKAGSVPAAERAKMVYPKVLPSSANNDDSESKTAGPNGLIKRQVLSELSEAGISDTQLNTEGLQITTTIDQKAQQAAIDSVHKNMQGERDEVRTAVVSVDPKSGAVRAYYGGDNATGWDFANAGLQSGSTFKVFGLAENLELGKPLSTMYDSSDLTVNGIKITNAEGETCGTCTIAEALKRSLNTSFYRMELDMPDGPAKIAAMAHRMGIPDTIPGVGQTLTEPDGSGPNNGIILGQYQVRPLDMASAYATIAASGVYHKPHFVQKVVTADGQVLLDRGQVAGEQRISAAVADNLASAMQPIAASSRNHGLAGGRPSGSKTGTTQLGDTGQNKDAWMIGFTPSLSTAVWVGTADGVALKTPGGSIMYGSGLPSDIWKDTMDGALEGTPKENFPKPAAIGGQAGVPSYSAPYTAPTTTQQEYQPPVVVKPSQVEILPGITIPVPGIQPNPRSQPQQNQPQSQDTGPLPGQPVAPADGSSPSTSNSGDTSGNSRSQRPGAGVGNSTDGTGDGYTNSHR

>CORE_REP|Org30_Gene1262#

MTEPRIESEQDATRPLREDIRFLGGVLGDTIRDHEGPEVFDLIERVRIEAFRVRREEVGRSAVADMLDAVDIAVALPLIRAFSYFVLLANLAEDLQRDRRRAAHEAAGEPPQDSSLAATYRKLDAAALPGAEVADLLTDALVSPVITAHPTETRRRTVFDVQTRITELMRRRQHYPDRERAALELEIRRQVLTLWRTALIRLARLRIQDEIAVGLRYYELTLFDVIPAINAEVRAALRSRWPDADLLPRPMLRPGSWIGGDRDGNPFVTAEVVRTAAGQAAGVAFGRYLRELVELEKTLSQSGRLVQVSDAVAELATAGYADPATHADEPYRRALHRVRDRLISTAERALGTDVGSTGLLGLGLGPASGVGLWVGSVTRAAAVGPAHGGGPVADAAEIGGQKTSVSAAPAYPGPQSLLDDLDAIDASLRASGDGLLADDRLAALRHAVETFGFHLQGLDMRQNSEVHEQVVAELLAWAGVHPDYASLPEHERVRILSAELSTRRPLLGPHARLSELATKELGIIRAAAEVVATFGEPAIPNYIISMCTSVSDMLEAALLLKEGGLLDPGASDSPPRCAAGIVPLFETIEDLGAGAATLSAALEVPVYRELVAAKGMRQEVMLGYSDSNKDGGYLAANWALYRAELDLVEVARKTGIRLRLFHGRGGTVGRGGGRSYDAILAQPAGAVHGSLRLTEQGEVIAAKYAESGSAHRNLESLIAGTLESTLLDVEGLGDDAEPSYQLMDDLAARARAAYTRLVHDTPGFVEYFRESTPVAEVGDLNIGSRPASRKPTNSVADLRAIPWVMAWSQARVMLPGWYGTGSALEEWIDGDPQRLATLSGLYRRWPFFRTVLSNLAQVMAKSDLEIAARYAELVEDTALREQIFGMIGEEHARTIRMHAAITGNDQLLSDNPSLAESIHNRFPYLEPLNQMQVQLLRRLRGGDDSELVKRGILLTMNGLATALRNSG

>CORE_REP|Org31_Gene6076#

MVTEDRMPAALAGRLAGEVDTSERRRAEYSSDASNYRVPPRAVIFPRTDEDVRTTLEFARAEGLPVTARGGGTSVAGNAIGAGLVLDFSRHMRQIIELDPQARTARVQPGVVLAQLQSAAKRYGLRFGPDPSTQNRCTLGGMIGNNACGPRAVAWGRTSDTVRELRILDGTGTERRLANDLTVLPGLPEFTTANLAVLRTDLGRFDRQASGYGLEHLLPERGSSIAKAFVGSEGTCGLLLDATVELVRLPRASVLTVLGYPDIATAADDVAAVMAGKPTAVEGIDARLVDAVRAHRGTVPELPRGGGWLFVETTGDTEDEAMAAADQLRRGVGALDARIVTDPVAAAALWQIRADGAGLAGRTPDGRPAWPGWEDAAVPPERLGAYLREFAELTAAHGVEGLLYGHIGDGCIHVRLDLPIAEAPQRFRRFLFDAAELVVRHGGSLSGEHGDGRARSELLALMYSRPVLDVFAGYKALFDPDDVLNPGVLVAPRPLDADLRVAGLAPLPSAGGFAFPHDDGDLSTAVHRCVGVGKCRADTRASGGFMCPSYLATGDEKDSTRGRARVLQEVARGALAWSSDAVAESLDLCLSCKACASDCPAGVDVATYKSEALHRRYRRRPRPIDHYSLGWLPRWLRLATMVPRAANALAEIGVLRRIGLRAAGIDPRRPVPRLAARSFRRIWRDHGGEPPALDPAHLDQAHLDTAHLDTAHHDQGNAPAARPEVMLWVDTFTDAFDPEIAMAAARLVQSLGYRVRIPARRVCCGLTWISTGQLDGARARLRATLNALDEHVRGGGVVVGLEPSCTAVLRSDLPELLPDDPRAAPTARAVRTLAEFLAEQPRWRPPRRPDLSVVVQPHCHHHAVLGFEADRQLLAAMGITVTEITGCCGLAGNFGMRRGHYDISVAVAENGLLPALRSADAETILLADGFSCRTQAGQLAGRPARHLAQFLVRE

>CORE_REP|Org134_Gene4320#

MDPSTAWSIRPTWEYVVSRTFADRHIGPDRAELDRMLPVVGVGSLDDLATAAIPAGILDDSVLAALPAAVSEHEALAELAALAHSNTVATSMIGLGYYDTLTPPVLVRNLLENPAWYTAYTPYQPEISQGRLEALLNFQTMVSDLTGMEVANASMLDEATAAAEAMTLLRRANRSASARLLIDTDLFPQTRTILYTRAEPLGIEIVEADLSGGQVPDGEFFGVLAQVPGASGRIVDVAPIVEAAHERGALVAVGADLLALTLITPPGELGADACFGTTQRFGVPMGFGGPHAGYLSVHAKHARQLPGRLVGVSVDADGAPAYRLALQTREQHIRREKATSNICTAQVLLAIVAAMYASYHGADGLRAIARRVHGHATAIAAGLDGAVVHERFFDTVLAHVPGGAEAVVGKAKSRGINLRLVDADHVGIACDEATTDAHVAAVLESFGTALPSEKRDAAPVSIENRTSEFLTHPAFTRYRTETAMLRYLRRLSDKDIALDRSMIPLGSCTMKLNSTAEMEAITWPGFARVHPYAPVEDAPGLLRLIADLEGWLSSITGYDSVSLQPNAGSQGEYAGLLAIRRYHLDRGDTHRDTCLIPSSAHGTNAASAAMAGLRVEVVACRDNGDVDLDDLRAKIADHADRLACIMITYPSTHGVYEHEVAELCALVHDAGGQVYIDGANLNALVGLARPGRFGGDVSHLNLHKTFCIPHGGGGPGVGPVAVRSHLEQYLPGDPLESGSHAVSAAKYGSASILPITWAYIRMMGADGLRRATLSAIASANYIARRLDEHFPVLYTGENGMVAHECILDVREITKQTGVTVDDVAKRLADYGFHAPTMSFPVAGTLMVEPTESEDLAELDDFIEAMIAIRREIDQVGAGVWPVTDNPLRGAPHTAASLVGEWDHPYSREIAVYPRGLDHSRAKVWPPVRRIDGAFGDRNLVCSCPPLDAYTD

>CORE_REP|Org6_Gene3522#

MTRTIEGEQRAGLDRRTLLARSAVGTVALATAGTVGTVGQGVAAAPALAAPDSGYRVGLGISDITGPAAECGMMGYSQFGQDTAGIHLRPRARAFVFEAGAARVVFAVAENGMIVQSVHRGVLLELARRFGDRYTEQNVLLTSTHSHATCGGASHDYAYNLSVLGFQQQVYDAEVQGIVEAIVAAHEDLSPATLALGRAELHDASVNRSRVAFERNPAEDRAHFPGAIDPAVTALSIRKGGREVGAITWFATHNTSMTNQNRLISSDNKGYAAFAHEHIEHGVRYLDGAPEFIAAFAQTNAGDMSPNLNLRPGSGPTDDEFANTRIIGERQHAASKAALAQAAPVRGPVQSLLCYIDLADIAVDGRFTPDGQPRRTAPAAAGVSLIAGSVEDGPGLPGAPIPEGVRNPLIDALGDPRRPAPSWLADAQAPKVIAAPLGLLPPVPWVPNVVPIQLVRIGELYLAAAGGEFTIVAGLRVRRAVAAALGVDLEQVLLQGYANAYHQYVTTPEEYDAQQYEGGSTLFGRYTLPAYQQEFTRLATALATDGTVARGPAPRDVSHLQPNLVPAPGPDTALPDLTFGDVLVQPAPGYARASRWWPSSSRPTRNTTRAATAPSSRCSAGRRTAGCAWPTRGSGRSASTGARPARPPRSPGSPGTFPPTPHRADTGSCTSPVGSPPTAAGIRSPASPTNSTSRERSEPGLGALVPAPGGAALNQEDHQADQADDQAHDAHRASGLPPRAADPDHDQRDQPQQAADGEHGPRVGQPRRDLRHVRGHHPSVAGQIVRSDAGGSRIVWRVTTPSDVRALAGELSLAVVRLTRHLRGRRTDSPVSLTQLSALATLAREGAMTPGALAAKERVQPPSMTRVIASLSDLKLVERKPHPTDGRQIIVSLSESGRALIADEASAREAWMTEQLSGLTGEQLDVLEQAVAIMKQIVAESE

>CORE_REP|Org140_Gene5012#

MCRSSAGTAPAGPPKPVMPGRTEPNAPSIPPCWPPAVRASARSASTRRTPVTEIDTPDTPAVPPPPPQHWFADVLASDGGVVRLRPITPDDAERLQQFHAALSDRTRYLRYFGPYPRISPKDLYRSTHVDHHNRVGLVAELGESIIAVGRYELLDRTGPRAAEVAFVVADGHQGRGLGSILLEHLAGAAAENEIETFVAEVLAENTVMVTVFREAGYQVERSRDGSVLHLEFAIDPTEALLSVRDARERASEARSVGNLLTPRSIAVIGATPSAGRVGGAVLTNLLSGAFQGPVFPVNPARRSVRGVHAYATVREIPDEVDLAVVAVPAESIGSVLDDCMAKGVKGLVVLTAGFGETGEAGLEAERALVAAARGHGMRVVGPSALGIANTDPAIALNATLAPVLPGRGRIGFFCQSGPLGAAILGEAAARNLGLSTFVSAGNRADVSGNDLLQYWDTDPDTDVVLLYLESFGNPRKFSRIARRVARTKPIVAVSSGRLAARAQPAGDMDRSIVRDLFAQAGIVQVDSISELFDCAALLGYQPLPEGSRLAVIGNSAALGWLAVDAARGEGLVAGEPVDLGPQATPGEYFDAVLAQLRSADVDSVIVVFAPPVPLPTGEFAAAIRSASEAVPEAGKPILTTFIAEQGIPNLLAVRGAGASAVRGSIPSYPDPERAARALARVRRYAEWRDRPQSAVVRPDGIDSVRATELVAGWMAESGGRRLTDLETVALLECYGMSVVEFREVRDADAAVAAAEELGYPVAAKATSDAWRRRPDLSGVRLDLWRPEAVRQAYTDLVALSGDPALHIQRMATKGVGCVLRVQDDPSFGSVIEFGLSGLIIEMLGDRAYRALPLSPDEAAALIDAPRAAPLLSGTPASPRVDKAALVELAQRISALFDDLPEMRELYFDPVLASPTSAEILYARARIGPQPSRFDTGPRRLG

>CORE_REP|Org37_Gene6665#

MASTTRPDGDPETRARRQSVLLRQDELLELVPRQRPTQQRSRQVFDAVLRAFRELLVEVGFDSSTCEEAAVRAEVPISTLYQFFDNKYVIVCELNRQDLIAATQELADFPGEVPSMDWLRHMNKWIDHLGELWMSDPSRREVWLAMQSTPDTRATGALHQRELAEALQQMLRPLMPRTPRARRTVVAQVLVHVVRSMLSFSVQEGHTRSQAVAELKRLMVAYLLLAEKESRTRPRGETRPGSDNSDKPLFENGVDAVSGEDCDGGPRPRTAHLANIREQNLGHSINVSGGFFQGKEQILLPRRRPAQARSKRKFDALLHASRELLVEIGFESFTCEEAAVRAEVPISTLYQFFDNKYVIVCELNRQDLIAATQELADFPGEVPSMDWLRHMNKWIDHLGELWMSDPSRREVWLAMQSTPDTRATGALHQRELAEALQQWVRPLMPLTPRAHRAMMSQVFVHILYAMLSFSVQEGHTHSQAVAELKRLMVASLLLAEQESRSRPGDGEPSPLNPAVDARRDQYLAAIAQGRGELREAEHLYTRALMVFEELGDRPAIAHSCLALAAIAHSRGDDREAMELYTRGLVIQEELGDRAAVAATYRELAVLAHERGDSREAEDRYIHARVVFEELGDRAGMAGTYHQLGVLAQDRGDCLEAEQQYLRALAIENELGDRAAVAATYRELAVLAHERGDSREAEDRYMHARVVFEELGDRAGMAGTYHQLGVLAQDRGDYEGAEHLYIQALALVEELGDLAGIARGNGQLGIVAQLRGEMDRAEHQYTRALSIKEELGDRAGMASSYHQLGILAHLRGDYSSAESWYTRALSIVEEFGDRSGVARSCGQLAVLAHDRGDYSKAEGLYARALGIFEAQGDRAEMARSYRNLAILAQDQGNDSKAKHLMTRALAVAREFDEPHTSREDSRSTMKYRQESEREGGVGR

>CORE_REP|Org100_Gene3344#

MRSLREPTRRAKYVAARPLTNRDLAQVLRVLDTDPVASCMVVARLQEFGLDARGAQGELWTREGPAESLCFSGANLVPLRGDHEALRAFADRAARWPRMCSSLVGRQELALPLWEMLSERWGPPRELRGTQPLLALAHPALYDPDAEVRRARPDELDRYLEAAIAMFIEEVGVDPRAGDGGRAYRRRIQSLIESGRAWARFEDGRVVYKAEIGSLSRRTGQIQGSGCIRTGAGAGSARRARRRSRTRWSPPAARPASTSTISTPSPAAPTAGSGSGRWRPSPPSCSTERAPRGARFGAPQEFAVRWPVPSAAMSTRILIPVVVAMLVAAAVVGCSSGPQGPVPVAESFLSAFAARELDAAAELTSQPEKASAALASAWEKLQAEELTARSGAARVTGDTATVDYSYEWRLPKNRVWSYTGQLQMGRSEGRWTVRWTSSNIHPRLGDTQTLALRTTPAPRARVNEQSGSDVLVPGEVTRVAFSPADAPDPAKVAGALSAALLRFDKKLTPEAILRAARAADGPYTVALLSEVEYNEVGALLIGLPGVRLNQEWDMVSTERGFAPDLLTQIRKTVIAEVDGKAGWSVVTQNANGVDTDVLTEVPAQPAPSFALSIDRYVQNAAQRAVDVPKEQTMMVVIRPSTGAILAVAQNEAADRDGPMATIGQYPPGSIFKTVTAAAAMHEGLATPDTIVPCPSQIVIGERTIPNYNMFSVGSVPMATAYERSCNTSFAKLASELPADALHVTAAGLGVGPDYTVVGLPTTSGSVPPAEDMVQRSEDGIGQGKVVVSPFGMALMAATVANGSAPVPWLIGGRETIVDGERPAIDPAVIEGLRLMMRKVITGGTATRIVDQGEVYGKTGEAEVEGGSHSWFVGYRGDIAFATLLVKGGSSDNAVAVTRDMFAALPPEY

>CORE_REP|Org31_Gene3647#

MTHHRSHTGELAKFSTELKFSLDPFQRTACAALEAGHGVLVCAPTGAGKTVVGEFAVHLALAAGGKCFYTTPIKALSNQKFAELTERYGRDTTGGGKSMVGLLTGDQSINPDAPVVVMTTEVLRNMLYASSDALRGLSYVVMDEVHYLADRFRGAVWEEVILHLPPDVRLVSLSATVSNAEEFGAWMETVRGDTAVVVDETRPVPLWQHVMVGRRMFDLFDTDSTDQKVLVDEDLVRFIKQRELADRANSWGGPRNGRGGPRRDFRPLPRPEVLAKLDEEGLLPAITFIFSRAGCDGALAQCLRSRLDLGRDEDRAQVDAIIDKHTGELPKADLEVLGYWEWRESLHRGLAAHHAGMLPAFRHTVEELFVNGLVRAVFATETLALGINMPARTVVLERLVKFNGESHAELTPGEYTQLTGRAGRRGIDVEGHAVVLWQPEVDTSAVAGLASTRTYPLRSSFRPGYNMSINLIDRMGAAESRALLERSFAQFQADRSVVGLVRGIERNESALRKLRDQLGGAEGGFLEYISLRERIKQRERQLEQQGRSDRRGAAVDALVALRRGDVVAIPSGRRAGLAVILEPDATPGDPRPLVLTADKWAGRISVADFPVPAEPLGHMRLPRRVDHRTAQARRDLASALRSTGISAPGRPRRGKRSAAADDRELATLRRSLRSHPAHTRPDREQMARVGERYNRLLRETETMRQKVAATTNSLARTFDRIVGLLQERGFVHDSEVTADGRRLARIYSESDLLVAECLRQGLWRGLGPAELAGVVSILVFESRQDGGYLGASGPTAPIRRAVGETIRVWTELRSDEARHKLPPTREPDLGFVTGVYKWARGDGLAESLLASGDQAAPLSAGDFVRWCRQVIDLLDQIHGNADDPEVAATAAKAVRAIRRGVVAVDAA

>CORE_REP|Org134_Gene3863#

MDSTLAPETHGGHRPEPEPRSGNSPQAQHAPQAQESPQAPDTAAGGRAAAQGGLRAKQQRLTAEQQQRRTEAGHRRAEAEQRKSEETERLIDGLNPQQRAAVVHTGAPLLIVAGAGSGKTAVLTRRIAYLLAARGATPGQILAITFTNKAAAEMRERVIGLVGPRANNMWVSTFHSSCVRILRMQSALLPGLNSNFSIYDADDSRRLLTMISRDQEIDTKKYSARLLATAISNLKNELISPEQATADAESDDAELPGLVARVYTEYQRRLRAANALDFDDLIGETVALLQSHPQVAEYYRRRFRHVLVDEYQDTNHAQYILVRELVGHHASRPAADTAGADDAAAGAAAAAGSDDDWAEPDEHRVPPSELCVVGDADQSIYAFRGATIRNIEEFERDFPDAETILLEQNYRSTQHILSAANAVIARNEGRREKRLWTDSGEGDLITGYVADNEHDEASFVAREIDRLVDAGEATYGDVAVFYRTNNNSRALEEIFIRMGLPYKVVGGVRFYERKEVRDVVAYLRVLENPDDAVSLRRILNTPRRGIGDRAEACVAVHAEQRDIGFAAALRDAADGNVALLNTRAQRAISGFLELLEEIRAAGARPDADFPDVGNVVEAVLDRTGYRAELEASDDPQDGARLDNLNELVSVAREFSSEANNNAEAARAEGMLPEAADGEPEPGSLAAFLERVSLVADTDQIPDEGAGVVTMMTLHTAKGLEFPVVFVTGWEDGQFPHMRALGDPAELAEERRLAYVGITRARRRLYLSRAVVRSGWGQPVSNPESRFLKEIPGHLIDWKRLEPKSSGGSRTGRRRGDEDFERDWTEGWSEPRPGVRERRPAPRAGGVKRNNVDLVLAVGDRVSDDKYGLGRVVAADGVGPLATVTIDFGTAGKIRLIPQFSRTLVKL

>CORE_REP|Org163_Gene5199#

MKFACAAPVGWVFVQTHEIRRRFLDHFLRAGHTEVPSASLILADPNLLFVNAGMVQFKPYFLGQEEPPYPRATSVQKCVRTGDIEEVGVTTRHNTFFQMAGNFSFGDYFKEGAITLAWELISKPQDEGGYGFDPERIWVTVYQDDPETAEIWKRVAGMPEERIQFRDGKDNYWDMGVPGPGGPCSEIYYDRGPEHGRDGGPVADEDRYLEIWNLVFMQDVRGELSPKLGHPPVGSLPKKNIDTGMGVERIALLLQGVDNVYETDLLRPIIDKAEELTGRSYGFQHEDDVRFRVIADHARTAAMLIADGVNPGNDGRGYVLRRLLRRIVRSARLLGAEKPVMGEFMKIVSDLMAPSYPELATDFRRIETVAVGEETAFLKTLNTGSTLFDNTAAAVKAEGGSTIAGSDAFTLHDTYGFPIDLTLEMAAEAGLSVDEEGFRSLMAEQRKRAKEDAQARKHAHADLTIYKELVDRGATEFTGFDELTSEATVLALIADGVRVPTATVGQDVEVILDRSPLYAESGGQIADRGSITASSGLKLRVNDVQKIAKKLWVHKTTVEHGQVTEGDIVLAQADPAWRRGATQGHSGTHMVHAALRRVLGPNAVQAGSLNKPGYLRFDFNWQGQLSEQQKADIEAVSNDAVGADFPVNTFVTDLPKAKQMGALALFGENYGNEVRVVEIGGPFSMELCGGTHVQHSSQIGPITVLGESSVGSGVRRVEAFVGLDSYKYLAKERALLAGVASALKVPSEEVPGRVEQLVERLKVAEKELERTKMAAVLSSAGKFVEEAERIGRLLLVAVAAPEGVPAGDLRTLATDIRGRFGSEPAVVVLLGNADGKVPFVVAVNKPAQEFGVKAGDLVGSFGPSIAGRGGGKPEMAQGAGSDPSGIPAGLAAVRARVAELAG

>CORE_REP|Org44_Gene5774#

MDKTDPLTQKSRQALHDAQTKAVRFGHTEVDGEHLLLALLDDPDGLVPRLLAQAQADPDTLRTALETELGRRPKVSGPGAAPGQIFLTQRLVRLLDTAEREAKRLKDEYVSVEHLVIALIEEGTTTAAGRLLHEHGLTRDRFLQALTAIRGNQRVTSAMPEVAYEALDKYGRDLVADAAAGKLDPVIGRDAEIRRVVQILSRKTKNNPVLIGDPGVGKTAIVEGLAQRIHRGDVPEGLRDKTVFALDMGSLVAGAKYRGEFEERLKAVLNEVKAAEGRILLFVDELHTVVGAGAAEGAMDAGNMLKPMLARGELHMIGATTVDEYRKHIEKDAALERRFQPVLVDEPDEADAISILRGLRERLEIFHGVKIQDSALVAAVTLSHRYISDRFLPDKAIDLVDEACAMLRTEIDSMPAELDELTRRVMRLEIEEAALAKETDPASQSRLTELRKELADLRAEADAMRAQWEAERAALRKVQSLRQEIDQVRHDAELAERDYDLNRAAELRHGRLPELERRLDAEEQQLTAKQGRQRLLREVVTADEIAAIVSRWTGIPVSRLQEGERDKLLRLDEILHQRVVGQDEAVQLVADAIIRARSGIKDPRRPIGSFVFLGPTGVGKTELAKTLAAALFDTADNMVRLDMSEYQERHTVSRLVGAPPGYVGYEEGGQLTEAVRRKPYSVVLFDEIEKAHTDVFNTLLQVLDDGRLTDAQGRTVDFRNTVIIMTSNIGSEYLLEGATAGGEIKPEARERVMAALRGHFRPEFLNRIDDIVLFKPLTEAEIERIVELMTDELRGRLAERRMTLHLSDPARHFIAQQGFDPVYGARPLRRFIAREVETRIGRALLGGDVHDGATIHIGLSDGGLTVSFDNPNTGPSQDPADRVAAGTGS

>CORE_REP|Org114_Gene3624#

MIEDVTDLPNPSLPSVPQGPPAAGPGGAAGSSPVAPSASAMRRALRRARDGATLNLDEAVVLLHARDADLDDLCATAARVRDAGLRDSGYAGDVLPITYSRKVFIPLTRLCRDKCHYCTFVTVPGKLRAAGHGMYLEPDEVLDIARRGAELGCKEALFTLGDRPEERWPEARQWLDERGYDSTLDYVRAMSIRVLEETGLLPHLNPGVMSWAELSRLKPVAPSMGMMLETTSTRLFTEKGQAHYGSPDKDPAVRLRALTDAGRLSVPFTTGILVGIGENLTERAESILAIRKAHKAFGHVQEVIVQNFLAKSDTAMRDTPDADLQEFRATIAVTRILLGPKMRVQAPPNLVSLDECRALLAAGVDDWGGVSPLTPDHVNPERPWPNLEVLAQVTADAGYVLTERVTAHPKYVLAGHPWIDPRVSAHVAALADPATGLARPDTKPTGLPWQEPDHDWESVGRIDLNTAIDSEGRNTESRSDSALSDDGLGAFGDWETIREQVHELAAAAPERFDADVMAALRAAERDPAGLTDDQYLALATADGAALEAVTAFADQLRRDTVGDDVTYIVNRNINFTNICYTGCRFCAFAQRKGDADAFTLSSEEVADRAWEAWVEGATEICMQGGIDPELPVTGYADLVRAIKNRVPEMHVHAFSPMEVVNGASRGGQSIHDWLSALKEAGLDTIPGTAAEILDDEVRWILTKGKLPTSAWIEVITTAHRLGIRSSSTMMYGHVDNPKHWVGHLRVLRGIQDETGGFTEFVLLPFVHQSAPLYLAGASRPGPTVRDNRAAHALARIMLHGRIANIQTSWVKLGTTGTQLMLNGGANDLGGTLMEETISRMAGSEHGSAKTVAELTEIATGIGRPARQRTTTYGTPPRRSPVSLPVS

>CORE_REP|Org3_Gene2247#

MLLRGFTSVRDMGGPIFPLKAAIDAGKATGPRVWPSGAMISQTAGHGDFRTPEEKSRRFTGKQSRAEEIGATFIVDGRDEVLTASRENLRMGASQLKLTAGGGTSSAYDPIDVTQFTLDELRAAVDAASDWNTYVAVHAYTPKAVRRSVEAGVLCIEHGQLLDEPTVDLLAERGVLLSGQYLQPSNDSMPAERRAKREGIVAGNAIVWPMAKNAGVKLAWGTDFLFEPAMNAEQNRLPAPAERVVQPSGTAPPRHHRQRRTTGTERAPQPLPGNARRGRTQRAGRPDPRRRRPVGRHQPHRRPGQELPRDHERRRDPQEHLARPPPRSPGRLARRQPEAHTPTGTRFGIHGAVVGGDDVVDDGQAESGSGDGLGASSSLGLCMGRCPEGTRSGSRAEKGAKPRQRSFLARRHSRSGALPVQCPVKPRSRRIYDVRMLIHDTHGSSTSPPEPADVDRAGGRRISPYVVWGGIAAAVLVLVAGQLIAVHINALGPLTSLLRDYAGTPKSATTPWAGFLLALVGVTTRVRVSALAAAVAIDLVFVAVRTLDERPFTVGNGPTIVLTALAVIAAVRWSGVRRRTALRTIALGALLILATKVGEIWLDITAWVCPQVLDPYVEVADRALGNPSWLVGRALELAGPIPTGVVRWVYFELPVAAIVVAVWQLRGVTTGVWPQHHLVRTFLAIGLIGPIFYVIFPVVGPVLAYGPQGHGMEIADVWPNIIPALPISVESMPFDGITPRNCMPSLHTAWALALFIHSRRGPTWLRWGGTFWLVCTLTATLGLGAHYGLDLVAGAAFCLTIESVLRDPGRGWDRARIRVVAMGIATFAAVLLCVRYLAMPMATHPVPFGIAILGMLTALCVVFYRTWFAPQPPVVVAREAELEHR

>CORE_REP|Org118_Gene5430#

MNTTVEPTQRKTVVVVGHGMVGHRFVEALRSRDEAGRWQIVVLSEESQAAYDRVGLSSYVGAWEKSALALPGNEYAGDALVDLRLGVRADEIDRAARKVTTSSGDVIGYDALVLATGSYAFVPPVPGHDRPECFVYRTLEDLDGIRAAAQNAGPGAVGVVVGGGLLGLEAANALRLMGMTPHVVEFAPRLMPVQVDEGGGAILEKLVTDLGLHVHTGVGTSAIEPAEDGAGLRVSLSDESVIDASLVVFSAGVRPRDQIARDAGLEIGPRGGALTDLGMLTSDPNIYAVGEVAAVEGTCYGLVAPGYTTAEIVADRLLGGAGEFPGADLSTKLKLLGVDVASFGDAHATTEGALSVVLHDAAKGTYAKLVISDDAKTLLGGILVGDASQYAALRPLVGSELPAEPAALISPAGAELGADALPDEAQICSCNNVSKGAIVGAIHEGACDIAGVKSCTSAGTSCGGCVPMIKKLLEQSGVEMSKALCEHFTQSRSELFQIVQVTGIRTFSELIAKHGTGIGCDICKPTVASILASTSSDHILDGEQSALQDTNDHFLANLQKNGTYSVVPRMPGGEVTPEQLIEIGQIAKDFGLYVKVTGGQRIDLFGARVEQLPQIWQRLVDKGMESGHAYGKSLRTVKSCVGSTWCRYGQQDSVGMAVLLEKRYRGLRSPHKLKLAVSGCARECAEARGKDVGVIATENGWNLYVGGNGGLTPKHAVLLAGDLDDETLIRYIDRYLMFYIRTADRLQRTAPWQESLEGGIEHLKQVVCEDSLGIAAELEESMARHVAGYKDEWAAVLEDPAKLSRFVTFVNAPEEADPTIAFDESGERKTPVLLGLPDVPGGGSLRNHGGHRAGRDGHGMPELPAMPAATATPGK

>CORE_REP|Org198_Gene7432#

MRFGRVRGVKALRRFTVRAHLPERLAALGELATNLRWSWHPPTQDLFAELDPQRWLEMGHDPVRMLGEVPAARVDELAADPDYVRRVDAAAADLRDYLAAPSWFERRAGEEGVRGIAYFSMEFGVTEVLPNYSGGLGILAGDHLKAASDLGLPLIGVGLLYRSGYFRQTLSADGWQTEHYPDLDPQGLPLRLLTSEQSESETAPVLIHVAMPDQRVLRARVWIAQVGRVPLLLLDSDIAENDPELRAVTDRLYGGDQEHRIRQEILAGIGGVRAVRAYTAANGLPDPDVFHMNEGHAGFLGVERIREFVAAGKDYDTALAAVRAGTVFTTHTPVPAGIDRFPMPMVRRYFGGAHGESESAMLPGLSVDRIVALGREADPSVFNMAHMGLRLAQRANGVSKLHGEVSRAMFAGLWPGFDAAEVPIGSVTNGVHAPTWAAREWFDKAREHIGAELVEEARGWERLRDVDLGELWSTRNALRAILVAEVRRRVRASWLDRGAAEAELGWVDSVFDPDVLTVGFARRVPTYKRLTLMLRDPQRLRAQLLDPQRPMQLVVAGKSHPADDGGKALIQQVVRFADDPAVRHRIVFLPDYDMSMARYLYWGCDVWLNNPLRPLEACGTSGMKSALNGGLNLSIRDGWWDEMYDGENGWAIPTADGVSDEHRRDDLEAAALYDLFERTVAPRFYDRDAAGMPVRWVEMVRHTLQTLGPKVLASRMVRDYAVEYYAPAANAYQQATADDFAVARTIADYRRRVEAAWPSVKVIQVDSAGLPDTPIIGARLSLTARIDLGGLAVDDVVVQAMLGRVSPSDDLSDVVTIPMTHQGSDSGVAHFVVDTPVPLSGAVGYTVRVLPHNELLAGDAELGLVAAPNA

>CORE_REP|Org138_Gene4495#

MIDHDSRIPTATTAPRSVVALVDSTTQVERELVGSWLAEGGINQEFGTEAPVTQIDLDPTAIATRLVDRHDDPLVVPVRVLWLPPERDGVRRTTFTDLITLSNPRKPNRLMQRRLIGKAPDRHLVLTGQPARLSELRANNPGAAGAAEAFARAIVRAGIVALERAERAVIGDRYKVPRLVAEEILDSPEFLRRLDDIAAHTGTSPREMHRRAEKALRELVAAQSRLVSDLFTQAMRPVHASTWKVDDDPVGFERLRSLNRRYPLVFLPSHRSYVDAFVLGDVLARNDFPPNHVIGGANLGFWPMGPIARRTGTVFIRRSFGDDEVYKAVVEEYFAYLLAKRFNLEWYFEGGRTRTGKLRPPRYGLLNYLAAALRSGRVDDVMLVPVSITYERLNEIGAIADEQTGGKKQPEGLAWLARYVRNQQHSAGRVYVRFGEPLSARERLTAHGDPLVEPADPGPAVSNPRAAASNTTSIPSPQAADTAPTTSNPAAAKADPEPETSNSTPATSNPEAVSNPTPAISASAAGGNGAVPESESVVEEQERRAVQRLAFDVAVGINAVTPITVNALTTLVLLGVHERALTRDELRAAIAPVLGYIEYRDLPRGELDTLRDDHGLAVVLEQLAIAKVVTVYRGGLEPVYSIGAGAHLEAAFYRNSAVHWFVNRAILELAVLTAVEAPEGDQLRVGWEAAYRLRDLLKFEFFFPERAEFTSELTAEMLHVDPQWHRRTAAGTVGTEILAQLAGSGFMMAHRVLRSFFDAQLVVAERLAAHDPATAVDRKAIIDECLNVGRQMLLQQRLQSPESVSSELFSSALKLADNHGLLTPDPADPAELAARRTRFAGELRAIGGRITRAATLDPSNRLETL

>CORE_REP|Org12_Gene1466#

MSAPNLTRDQAIARAATISVENYRVELDLTGQPTGPAATAGETFFSRSTVTFTATPGARTFIDIVAAGVRSAVLNGTELDVSGYDESQGLTLTDLAENNVLVVEADCVYSHTGEGLHRFVDPTDDKVYLYSQFETADAKRMFACFDQPDLKATYDMTVTAPADWEVISNGAATATRTVGEVVEHTFATTPRMSTYLVALIAGPYAKWIDVYTDDRSTIPLGVYCRASLAEFMDAERLFTETKQGFAFYHRNFGVPYVFGKYDQLFVPEFNAGAMENAGAVTFLEDYVFRSKVTRASYERRAETVLHEMAHMWFGDLVTMKWWDDLWLNESFATFASVLCQAEATEYTGAWTTFANVEKSWAYRQDQLPSTHPIAADIPDLAAVEVNFDGITYAKGASVLKQLVAYVGLEPFLAGLRDYFAAHAFGNATFDDLVGALEKSSGRDLSGWGAQWLKTTGLNTLRPDFEVDADGKFTRFAVVQGGAEPGAGEYRVHRLAIGVYDDRDGKLVRTKRVELDLDAAERTEVAELVGVERGQLVLVNDDDLTYCSLRLDPQSLDTVVARIADIAESLPRTLCWSAAWEMTRQAEMRARDFVALVQRGIGAETEIGVVQRLLMQAQTALASYADPEWADQTGWPAFADRLLELARAAEPGSDHQLAFVNALTAARLSPWHSEVLGTLLLDSDPAAVELPGLAVDTDLRWRIVQALAAAGQLDAESIDTPVIDAELERDPTAAGRRQAAAAATARPQAEVKQKAWRTVMDDDSVPNITARAIVGGFAPIGQGELLAPYVQRYFDEIASVWERRSSEVAQTVVVGLYPHWAISEEAVAVADKFLADEHPPALRRLVIEGKAGIERSLRARAADIA

>CORE_REP|Org144_Gene202#

MTDTTLPPFGGSGGDRIDPVDIQQEMQNSYIDYAMSVIVGRALPEVRDGLKPVHRRVLYAMYDNGYRPDRGYVKSARPVAETMGNYHPHGDASIYDTLVRMAQPWSLRYPLVDGQGNFGSRGNDGAAAMRYTECRLTPLAMEMLREIDHETVDFIPNYDGRSQEPTVLPSRVPALLMNGSNGIAVGMATNIPPHNLTELAEAIYWALDNHDADEEATLAACMERVKGPDFPTHGLIVGSQGIHDAYTTGRGSIRMRGVVEIEEDNKGRTTLVITELPYQVNTDNFINSIAEQVRDGKIAGISDIHDESSDRAGMRIVVTVKRDAVAKVVLNNLYKHTQLQTSFGANMLSIVDGVPRTLRLDQMIRLYVKHQLDVIVRRTKYLLRKAEERAHILRGLVKALDALDEVIALIRRSANTDTARTGLMQLLDIDEIQATAILDMQLRRLSALERQKIIDELAKIELEIADLKDILAKEERQRAIVRDELAEIVEKYGDDRRTRIIAADGDVADEDLIAREDVVVTITETGYAKRTKTDLYRSQKRGGKGVQGAGLKQDDLVKHFFISSTHDWLLFFTNKGRVYRAKAYELPEANRTARGQHVANLLAFQPDEKIAQIIQIKNYEVAPYLVLATKNGLVKKSKLSDFDSNRSGGIVAVNLRDEDELVGAVLCSADDDLLLVSALGQSIRFSATDEALRPMGRATSGVQGMRFNASDELLSLNVVRPDTYLLVATAGGYAKRTAIEEYTPQGRGGKGVLTVQYDPKRGTLVGALIVEDDDELYAITSGGGVIRTVAKQVRKAGRQTKGVRLMNLGEGDTLLAIARNADEPDPDLLAGDTSDTGSSE

>CORE_REP|Org158_Gene3705#

MTQHLEQANAGQSNNDASATPPTPNSMPQRQGDPTSQRQSDPTSQRQGDPAAQRQGDTTAAQRPGDTTAQRPSLPVAQRQGGAPAAVPTSASRRVRARLARRMTGQRGIAAVKPVLEPLATVHRELYPKANLQLLQRAFDVADEKHAHQFRKSGDPYITHPLAVANILAELGMDTTTLVAALLHDTVEDTGYSLDELTNEFGQEVAHLVDGVTKLDKVNLGAAAEAETIRKMIIAMARDPRVLVIKVADRLHNMRTMRFLPPEKQAKKARETLEVIAPLAHRLGMATVKWELEDLAFAILHPKKYDEIVRLVADRAPSRDTYLAKVRAEIVNTLAASRINAIVEGRPKHYWSIYQKMIVKGKDFDDIHDLVGIRILCDEVRDCYAAVGVVHSLWQPMAGRFKDYIAQPRYGVYQSLHTTVVGPDGKPLEVQIRTQDMHRTAEFGIAAHWRYKETKGKHSNDSTEVDDMAWMRQLLDWQREAADPAEFLESLRFDLKSPEIFVFTPKGDVITLPQKSTPVDFAYAVHTEVGHRCIGARVNGRLVALERQLENGEVVEIFTSKAQNAGPSRDWQNFVVSPRAKAKIRQWFAKERREEALEAGKEAISKEVRRSGLPLQRLMSADAMSALAHELHYPDISALYAAVGESQVSAHHVVQRLMAQLGGVGDVENELAERSTPSTVPARQRGTGDAGVEIPGASGTVAKLAKCCTPVPGDEIMGFVTRGGAVSVHRTDCTNADSLRSEPERIIEVKWAPSPSSVFLVAIQIEALDRTRLLSDVTKVLADEKVNILSASVMTSGDRVAISKFTFEMGDPKHLGHLLNVVRNVEGVYDVYRVTSAA

>CORE_REP|Org102_Gene5670#

MNAANRPLRVGIVGAGPAGIYAADALMKSDAPAGFDGVSIDLYERMPAPFGLIRYGVAPDHPRIKGIITALHKVLDKPQVRLLGNIDYGVDITLDDLRGFYDAVIFSTGANADRALDIPGIDLDGSYGAADFVSWYDGHPDVPRTWPLDAQKVAVLGVGNVALDVARVLAKTGDELLPTEIPPNVYEGLKANQALEVHVFGRRGPAQAKFTPLELRELDHSPTIEVIVDPSDIDYDEGSEAARRHSKQVDMICNTLEQWAIRDVGDRPHKLFLHFFESPAEILGSGGKVVGLRTERTQLDGTGNCKGTGEYKDWDIQAVYRAVGYLSQNIPALPFDEQAGTVPNEAGRVLVDEGADGAARYLPQTYVTGWIKRGPVGLIGHTKGDANETIACLLDDVKDFTPAANPDPEAVTAFLEDKGIPFTTWAGWYRLDAHERALGEPEGRERVKVVEREDMLRASGVAVAPAVETAAILGALAGVHDPEINRPITELDMVAGVDIAPGNRVTVRVLLTVAGCPMRARLTRDIEAAVLSVPGTASVTVDFGVMTDTQRATLRQRLRGGDTPVIPFAQPGNRTRVYALASGKGGVGKSSVTVNLATVLARRGLRVGILDADIHGHSIPSMMGSTATPTQVDRMIMPPTAHGVRLISIAMFVSDNEPVVWRGPMLHRVLNQFLADVYWSDLDVLLIDLPPGTGDIAISLAQLLPTAEMIVVTTPQHTAARIAERAGAVATQTGQRVAGVIENMSWYEGPDGTRHLLFGSGGAEDVSARLTDILGADCPVLARIPLDPDVCAAGDEGIPMVLTHPESAAAQAISVLANRLDARRTRLAGQRLAVAPV

>CORE_REP|Org136_Gene4446#

MAQPGSHGDPTAEETSADNVGGGGGRSTPPSPYRLAEAPGPLDTDELAAIDAWWRAANYLSVGQIYLMANPLLREPLRPEHIKPRLLGHFGTVPGLNLVWVHANRAILARDLDAVFVAGPGHGGPGPNACAWLEGTYSELYSHVPRDIDGMRALFAQFSFPGGVPSHCAPETPGSFHEGGELGYSLLHAFGAALDHPNLTVFCVVGDGEAETGPLAGSWHADKFLNPARDGAVLPILALNEYKIANPTILARIPESELISLMRGYGYEPLVVSGRDPARVHQAMAEAMDTALERIAQIQRAARGDGDGSRPHWPMIVLRTPKGWTCPPLVDGEPVEGTFRAHQVPLPAARTDDDHRAVLEQWLRSYRPAELFDESGAPVSELMDLVPAGDRRMSANPVSNGGAQVRDLRLPDWRDFGVEVPAPGATHHEATRVLGGWLREVTARNPDNFLTFAPDELASNRLQDILEVTGRNWQAEIDEFDVGLDRQGRVIEVLSEHMCQGLLEGYLLTGRHGVFTCYEAFVHIVDAMFNQHAKWLDASAAVPWRRPIPSLNYLLSSHVWRQDHNGFTHQDPGFLDVVLNKKPEIVRVYLPPDANTLLSTFDHCLRSRHYVNVVVAGKQPQPDWLSVEAAALHCARGIGIWEWAGNNDELGTAPDVVLACAGDVPTLETLAAAAILREHLPQLRVRVINVVDLMRLLPGTDHPHGLPDSEFDTLFTHDRPVIFAFHGYPWLIHRLTYRRTNHSGLHVRGYKERGTTTTPFDMVMLNDMDRYHLVTDVIDRVAGLREKAGGLRQQMQDARLRARTWTREHGEDIPEVANWRWPAEIIARRNETSYIR

>CORE_REP|Org124_Gene1722#

MTAAQNLPVLFLTDPIVLPGMVVPIELDESAQAAIDAARAAKTDQVLVAPRLDEGYAAYGVVATIEQVGRLRGGAPAAVLKAERRAKIGHGVTGPGAALWVEAEPVEDVPADGRTKELAAEYKKLVVSVLQRREAWQVIDAVNQLSDPSAIADTAGYATYLTSEQKRELLETPEPAKRLATLIEWTKAHIAETEVSEKISEEVREGMEKSQREFLLRQQLNAIRKELGEDEPDGADDYRTRVEQADLPDSVREAALREVGRLERASDQSPESGWIRTWLDTVLELPWTVKTTDSTDVSAARAVLDADHHGLDEVKDRMVEYLAVRARRAARGLEVVGGRGSGAVMVLVGPPGVGKTSLGESVARALGRKFVRVALGGVRDEAEIRGHRRTYVGALPGRIVRAMKEAGSMNPVVLLDEIDKVGSDFRGDPAAALLEVLDPAQNHTFRDHYLDLDLDLSDVLFIATANVMETIPGPLLDRMELITVDGYTEDDKVAIARDFLVPRQLERNALTAEEVTVTEAALREIAADYTREAGVRQMERLIAKVLRKAATKLSEGGVADDDTVISLGLGYDPELGYDDPVHAGSDVASETAVGGADLPAGESRSTAAVSGESLTIDVGDLKDYLGRPRFTPDSVERTAVPGVATGLAVTGLGGDVLYIETNAVDGERSLTLTGQLGDVMKESAQIALTYVRSHLEEIGIEPSVLDRNIHVHFPAGAVPKDGPSAGVTMVTALVSLALGRQVRSDVGMTGEVTLNGRVLPIGGVKQKLLAAQRAGLKTVFIPARNEPDLDDVPAEVLAALDVRPVADVADILAYAIEPVEEPALDGRPLAATA

>CORE_REP|Org215_Gene4817#

MGGVVVGFERARNEQVAGGVVANGASDLVRARDQLLGEAGRNAGGGARTPRLDSEALRQALVDLFELWLTTKGSEVGITPDSGLAVVAVGGLGRREMLPYSDLDLVLLYDDVDPQRVAEVADQLWYPLWDAHIKLDHSVRTVPQALRVAADDLTAALGMLEARHIVGDQALSDLLVNGVRRDWRSGIRSRFDGLLEQAETRWRRSGEIAHRAEPDLKNGRGGLRDIQLLDALAIAQLTDAMPGLGPDVPGGGLELAHRRLLDVRTELHRVAGRGRDQLRAQDADEIGAALRIGDRFDLARTLSDAARTVSYSVDVGLRTAANALPRRGLARLRRMPVRRPLDEGVVEHAGEVVLARDARPQRDPGLILRVAAAAAQTGLPMSATTLNRLSEDAPELREPWPKEALNDLLVLLGSGRNAVDAIEALDRTGLWGRLFPEWGVVRDLPPRDAIHTWTVDRHLVEVVVRASALSTRVSRPDLLLLGALLHDLGKGRPEDHSVVGAELATQIGRRLGLWPSDVRTLSAIVRHHLLLPETATRRDLDDPNTVLTVVKALDGDAQLLELLHALAEADALATGPGVWSDWKASLIAELVRRCRLSMAGEELPEPDPIPAELVAKAEAGGVHVDLRAGDGRYTHVVTVVAPDTPGLLSDAAGVLAVHSLRVLSASLGGAGESAIDTFVVAPRFGDPPDPGLLRQELIRARNGDLDLAGVLARKEREAGGVQRSPYAQAEPRIIWSETADPGRVLLELRAEDRIGLLSRLAGALARVGANVLWAKAVTLGAAVVDVFCLDLGPGDTPQRRAEIAAALLAVVPPQQPKKSPESDTEMGSGRVF

>CORE_REP|Org49_Gene3403#

MIRAGWDRLRLFNIGELLAHRGRTLMSMIVMGVSAGLLVSVFSISGSITGSVDRLARSLSGDAALEVSGITDAGFEQSLLQEVRGVAGVGTAVPMLRAPIGADADRALLIGADASAAALGSDLDGPLREQAAKLPTVRNGVLVGTGMGYAEGESFDIGGTTVTVAGVLDDPAARKLNDGHLVITLLPVAQQITDRAGRLDSIQIVAAADTDVAQLRSALTDVVDGRAVVADPGMRAAQAGGAVQVVTLATLASSAAALIVSSFLIYNAMSMAVAQRRPMLSLLRAIGGRRAPMVRDLVAESALLGLIGGLVGAAVGVFMGRIAIHRLPAAMLNSVEARTEFILPGYAVPVAVAACVVASVAAAALAARQVYKVAPIEALAPVGTSSADTTNPILRWGAVALGTALVVGAIVIANADLGRISMAAVSMAIAGAVMFCFAATGPIVRGTAAVARLFGAPGALGATTVERAPRRVWATAMTVMIGVTAVVAIGNATRNMADSASESFAGLGTTDVFISPTAMEQFPTGPILPADLKEKIAAIPGVAGAGSAQMAFATLGGGRVMLQAFEEGQGRQAATAALSADTLRRMAAGEGIVVSRDVARTLAVQRGSTLELPTPSGIHRVEVLEVVPYFSAIAGVVILDLDILRQWYQRPGETIIGVDFLPDADPDQVFAAIRAAVPPEIHVDTGPEAVAAISASVSQGTSLSTSILWIVVAVSTIALLNTLMLSVLERRRELGVLRAMGTSRRFLLRTVLTEAAGIGVVGAAIGLAVGAAVHYLATITMSSSLSLDVEYQPSPLLLVYAVVALLLALLGSIPPAVRAARLPIVEAIAAD

>CORE_REP|Org97_Gene925#

MGRAQDRARIFWYARPCGSLATSCHGAPPTAHRACDGGPIRLGRVPISQTLARLAGACVLAAVLVAGLLFPLAGGFGYMSNRAADAVDNVSAELVAGTAPAVSTMVDATGAPIAWLYEQRRFEVPSDKIANDMKLAIVSIEDKRFAEHGGVDWQGTLRAFLTNTSSGEVQQGASTIDQQYVKNFQLLVVAKTDAERRAAIETTPARKLREIRMALTLEKELTKDEILTRYLNLVPFGNGSYGIQDAAQTYFGVDAKDLKVAQAAMLAGMVQSSSKLNPYTNPKGVLERRNTVLDTLIQNIPSRADEFRAAKEQPLGVLPEPKGLPRGCIAAGDRGYFCDYALQYLANAGISKDQMDKGGYLIRTTLDPAVQNSVKAAVTANTDPNLENIAEVTSIIAPGQDSHHILAMTSSRTYGLDQGAHQTVQPQPYSMVGDGAGSIFKIFTTAAAMEKGLGTSAQLDVPSFFAAKGMGNGGAAGCPPATYCVKNAGNYRSPMSVTEALAQSPNTAFVKLIQDVGVTPTVDMAVRLGMRSYAEAGTSGHGNQSLADMIKQQNLGSFTLGPVAINPLELSNVAATLASGGKWCPPSPIAEVIDRDGKQVPLTQQACEQVVEPGLANTLANALSQDAVGGTAAGSARAVGWNAPVSAKTGTTETHRSSAFLGFTNSMAGAAYIYGDSPTPGEICSFPLRTCGDGNLYGGNEPARSWFGGIKPVLDKFPPPALPPLDDKYVRGSNNAQIPDVNGMSESEARSVLIGAGFQVSTVTTPGSAAKGTVTATTPNGSAIPGSVITVLVSDGTQREIPKPGPPPAPPVLPGLPQIPRLPPIPIPIPR

>CORE_REP|Org23_Gene4451#

MSAATADPDTADPDTGEPRTRVFGIRHHGPGSARSLRLALDHYRPDTILIEGPADADPLTAFVHDPGLVPPVAMLAYVPDAPTRAAFWPFAQFSPEWQALRYATEHHVPVRFCDLPATTVLALDAEPGDSIDPLAELASAAGYDDAERWWDAIVESTTDIDTFDALTEAMGALRDSIAHGAQPPPTDSRLSPGPAGPAPGARSATNSRSAAADQDPATDENEDPADCDEERGNTEFRRGDGNAGVRGSEPGATSPAMPAAGIDAHTLRREAYMRQTIRKALKDGAQRVAIVCGAWHAPALVEPLGPAAPDARLLKGLPKAKARLTWVPWTHSRLSAASGYGAGVTSPGWYHHLFTETEQPIARWLTKVAGALRRHDLPVSSAHIIEAVRLSETLAALRDRPLAGLSEVTEATRAVLCDGDESMLRLVGAELVVGEAIGAVPATTPTVPLDADLRARARTLRLKQQPESTVMELDLRKDRDRLKSRLLHQLRILEVEWGTPTDSEVRATGTFRETWTLRWEPEFAVAVIEASRWGTTVASAAETKLLDTAGRDDQTVGDITDALGLALVADLGCAVGELIGRLESVAALDHDVTHLLAALPGLVRTLRYGDVRGTDTAALTHVADGMLIRICAGLPGAVTGLDTDAALEMRALIDAGHTAIHIRDDTEAREAWLAALHRIAERDDVHGAIVGRTVRLLCDADRIDQAESARRLAAALSIGSSAAAKAEWIDGFLGGRGLLLVHDRELLRLVDEWLRTLDDDQFVQTLPLLRRTFGAFESGERRAIGAAVRDGVSATAGAANVRQLDLERGVRAMRVVAEILGART

>CORE_REP|Org109_Gene6036#

MAGGASRTHTFHTSRACCGVRLVNPADSALRAQPRTGLSYGTSLLNRVLVGGPDGDPRLTHVVELPARAADRSAWPAWASPEVIAAIRDTGIDAPWRHQVDTADSAFHGRNVVVSTGTASGKSLGYQLPVLTALQADPKATALYLSPTKALGADQLRTVGALTHEGPLRDVHPATYDGDTPAEIRQWVRANARWVFTNPDMLHLGILRSHQRWARVLRKLRYVVIDECHAYRGVFGSHVALVLRRLRRIAARYGADPVFVLCSATSAEPAAAASRLIGAPCVAVTRDGSPQGPRTVALWEPPLLTAMTGENGAPVRRAATAEAARIMADLVGEGARTLTFVRSRRAAELTAMEAKRLLAEVDPDLAARVAPYRGGYLAEDRRALEAALSDGSLLGAATTNALELGVDIAGLDAVVISGFPGTVASFWQQAGRAGRRTQGSLVLLVARDDPLDTYLVHHPEALLDKPVEATITDPRNPYVLGPQLLCAALELPLTDAEVDDFGAREVLGDLSAQGLIRRRGAEDRARWYVTAETQPHDAVDVRGGIGAPVAIVDGETGRLLGTADAGRAQATLHQGAVHLHQGETYVVDELDPADGVAFVHAAEPGWTTSARQVTSIAVDAVTEYRAHGHVTAGLAQVRVTSQVIGYLRTLVTGEVLDLVELDLPPQTLPTRAVMYTVTPELLALAGIAPQDVPGALHAAEHAAIGLLPLVATCDRWDIGGVSTAEHPDTGLPTVFVYDGQAGGAGFAERGFAQLRQWLSATRLAIESCGCAAGCPSCVQSPKCGNGNHPLDKVAAARLLTAVLAELSADEDATLDS

>CORE_REP|Org101_Gene3463#

MTPPPMTPSDATETHVTDPLSLRRTQARPTTIRSTYRLQLRPDALTFADARAIAEYLQQLGISHLYLSPILTATKGSTHGYDVTDPTTVSAALGGPAGLKALSDEVRSRGMGLLVDLVPNHVGVADPRQNPWWWDVLRNGRESPFAHYFDIDWSAGNGAGGRLALPVLQSENDPAALTVDRSGAEPMLALHDLRFPIAPGTDGDNALRIHDKQHYRLVSWKAGVCTYRRYFTVGGLAAIRQEDPEVFEITHRELAAWCAHDIIDGVRIDHPDGLADPGAYLIRLRQIIGPNRLLLVEKVLSNREPLDATLPIDGTTGYDALADVGGVLIDPEGEPALTELSCRFAGHGSDRAWISETEHRIKRAVAETVLTADVRRLVAAIKRDARAESVDTMALTNATIEVLAFMPVYRTDYAPLAGMTAAVITEVERRNSELTNPLAVLTAALAMGGEAVTRFHQVGGAILAKAVEDTMFYQAARLVSLQEMGGNPARFGRSLIEFHLANIERAQRWPATMTTLSTHDTKRGEDVRARIGVLSQAAKDWARSVTAWQETAPGPDGATTLFLLQNMFGVWPPDGRPAAAIPGFRERLHQFAEKAIREAGTKTSWEEPDAAFEDEVHTWVDTVIDGPVGTELGDLVHRLAPHAWSDALAQKLLQLCGPGIPDLYQGCELWEDSLVDPDNRRPVDFALRAGMLQSLTGTPELDVTGAVKMWIVAYALWLRRERPDCFVGGTYAPLFGTGGEAQRLIAYTRGRPAEAPEIIVAATRHSVGLAETGWGDTALELPEGTWIDRLTGHTFQGRIRIERLFARLPVALLVR

>CORE_REP|Org106_Gene483#

MASRRAFDGGAGRPPVEVELSNLDKVLYPATGTTKGEVIAYYTAIAEAMLPHIAGRPVTRKRWPNGVEAPSFFEKNLAEHAPPWLERRVLEHSDRRVAYPLIDSEAGLAWIGQQASLEVHVPQWRFDGAAMGPATRIVFDLDPGPGVGLPECAEVALAVRDMIEDIGMHAFPVTSGSKGIHLYVPLDRVLSPGGASTVAKQVATNLEKLRPDLVTATMAKAVRRGKVFLDWSQNNPAKTTIAPYSLRGRAEPNAAAPRTWAEIEDAATLRHLRFDEVLARWRDDGDLLADLDPPLSRRGADALPGGADALAKYRSMRDPARTPEPVPAEPPSRGADNRFVVQEHHARRLHWDVRLERDGVLASWAVPKGPPTTPKQNRLAVHTEDHPLEYLHFHGVIPKGEYGAGEMTIWDTGTYETEKWRDDEVIVRFHGSKLTGRYALIQTNGNQWLMHLMREQPADAETPVGAETPVGAEDSGGPAVDAEIRDETAGDADAAGGRRSRTTAGAEVGRRGATPVPRGLSPMLAVSGEVGPLDGEQWCFETKWDGFRLIAEIDAGAVTLRSRAGNVVTDRYPRIAAVLAEELAGHRAVLDGEAVVFDEHNVAQVALLQADPARAEFVAFDVLYLDGTSLLRKRFADRRRVLEALGAAAPSLRVPPALDGPGAEALRYSEEHGLEGVIAKRWDSVYLPGKRGHSWVKQRNWRIQPVVVGGYRRSGARDFKSLLVGIPHDGELIYVGRVGTGFGEQDMTALARRLRGLERKTSPFANALTAEERKEAVWVRPTITGRVRFMNWTDTGRLWHPAWLGEDD

>CORE_REP|Org150_Gene1805#

MNRKTVFRNLAIVAGILLVIYLVSYFSNDTRGWKNVDTSVALTQLADKQNVKQVQIDDKEQQLRITLKQGNDATGGQNQIMAKYPGGSEVSAQILRDVQQSGAPFNTAVKQDSWFTQVLLFVLPMVILLGLFVFVMARMQGGGRGGMMGFGKSKAKQLSKDMPKTTFADVAGADEAVEELYEIKDFLQNPARYQALGAKIPKGVLLYGPPGTGKTLLARAVAGEAGVPFFTISGSDFVEMFVGVGASRVRDLFDQAKQNSPCIIFVDEIDAVGRQRGAGLGGGHDEREQTLNQLLVEMDGFGDRTGVIIIAATNRPDILDPALLRPGRFDRQIPVGNPDLAGRRAILRVHSQGKPIAPDADLDGLAKRTVGMSGADLANVINEAALLTAREHGNVITGPALEESVDRVIGGPRRKSRIISEHEKKITAYHEGGHTLAAWAMPDIEPVYKVTILARGRTGGHAMTVPEDDKGLMTRSEMIARLVMAMGGRAAEELVFHEPTTGASSDIDQATKIARAMVTEYGMSARLGAVRYGQEQGDPFLGRTMGTGSDYSHEVAREIDEEVRNLIEAAHTEAWAILNEYRDELDALAIALLERETLHRKDLEQVLATVDKRPRITAFNDFGERVPSDRPPVKTPRELAAERGESWPEEAETRKPNQQPAPQPVPANGYPQEGHRNPAPAPTGYSYPAPQPGNYPRPAGGPGYPRQSTHGSRPDYGAPAGWSAPGWPPQEDPSAGGSPQQPEYGGAPQGGGSQGYGEPQRYGEPQRYGGGHRRPEWPGDQDGSTQNGTQDHGDWDGPNSHR

>CORE_REP|Org121_Gene5397#

MSSATRANSKQAARHAADSHDAIRVVGARVNNLKDVSIELPKRRLTVFTGVSGSGKSSLVFSTIAAESQRLINETYSSFVQGFMPTLARPEVDVLDGLTTVITVDQQRMGSDPRSTVGTATDANAMLRILFSRLGKPHIGSPQAYSFNVASISGAGAVNIERAGRTIRERRSFSITGGMCPRCEGRGSVSDIDLTQLYDDSKSLAEGAFTIPGWKSDSFWTVRVYAESGFVDPNKPIRKYTKRELNDFLYKEPVKVKVDGVNLTYEGLIPKIQKSFLSKDKESMQPHIRAFVERAVTFTTCPDCAGTRLSEEARSSKIKRKNIADLCAMEIRDLAEWVRALKEPSVQPLLDSLVATLDSFVEIGLGYLSLERPSGTLSGGEAQRVKMIRHLGSALTDVTYVFDEPTIGLHPHDIQRMNDLLRQLRDKGNTVLVVEHKPETIAIADHVVDLGPGAGSGGGTICYEGSVEGLRASGTITGRHFDDRAALKESVRKPTGALEIRNATRHNLRGVDVDIPLGVLCVVTGVAGSGKSSLVHGSIPASEGVVAVDQTPIRGSRRSNPATYTGLLEPIRKAFAKANDVKPALFSANSEGACPNCNGAGVIYTDLGMMAGTASTCDVCEGKRFDASVLDYHLGGRDISEVLAMSVREAEEFFGSGEARISAAHAILARLVDVGLGYLTIGQPLTTLSGGERQRLKLATHMADKGGVYVLDEPTTGLHLADVENLLNLLDRLVESGKSVIVIEHHQAVMAHADWIIDLGPGAGHDGGKIVFEGTPAELVAARSTLTGEHLADYVGA

>CORE_REP|Org141_Gene5255#

MVSGSLLLCGIDLTVLHVAVPSVSRDLRPSAAQLLWIVDVYSLALAAMLVTCGTLGDRVGRRRMVLSGFLTFGLASAACALSTSTAQLIAARAALGVGAAMIMASTVAIIRVVFTDGRERAFAIGVWTSAHSVGATIGPLVGGLVAERWGWNAVFLVNIPVIIVILAVGARVIPESKNPAPRRWDLASVALSIAGLASVVYALKQAGEHAGVSTAILVTALSGAALLYAFVHRQRRLAEPLLDLSLFADRRFATAAVCVIGCFGSYVALLFFLTQWLQQVGGYSPLHAGLALMPLAAANAVGAVTAPRTASRWGNRGALTAALLLFALAYAVIAAVGDTAHYGTILPALLAAGYGAGIVMTLGADAIMSAAQPERSGEAAAIQETSFELGAGLGVAVLGTVMTVVYRTGMPHVPGLGPDERVIVGESFTAAQDLTAHLPSATADAVLDAARQSYDHGFTTIAVIATVTLVITAAMAAVLLRCKQNEPRNYRFQGGQASVPDTTHPPVTVLGLGAMGQAFVATLLKGGRTVTIWNRTPGKDAELVTAGARTTAAVDEAVTASPVIIAVLLDHRSVHSTLDPIADQLAGRQLINVTSTTAEESRELAFWAAGHGIEYLDGGIMAAPSMIGQPGASILYSGSRAVFDDHRGTLDLLASAEYFGTDAGMASMLDFSLLSAMYGMYGGFFNGVAMTRSVGVSAEAYAERAAAWVKAMTDYLPMLGKLIDARDYENGVQDIAFHKAAVDAIVRATRDAGAAPDFLAPLQHLIDRQIAEGNSALAFEHTVEEIV

>CORE_REP|Org45_Gene1385#

MATIEYLRTDPALPPVGIVDRSPLTPAKKGIFLAIAVLGAIAWAIVAFMRGENVNAVWIVVAAVSTYILAYQFYARLIQWKITKPRDDVATPAEAMENGKDFMPMDRRVLFGHHFAAIAGAGPLVGPVLAAQMGYLPGTIWIIVGVVLAGAVQDYLVLWASSKRRGRSLGQMARDELGPVGGVAAIVGVLVIMMILLAVLGIVVVKALAATENPVTGALEGGSPWGVFSIAMTIPIALFMGIYLRFVRPGKVGEVSVIGFTLLMLAIVSGNWVAESGWGRDWFTLSAATIGWLLIFYGFFASVLPVWLLLAPRDYLSTFMKIGTIFLLAAGILITMPVLKAPAVSEFASNSDGPAFAGSLFPFLFITIACGALSGFHALVSSGTTPKLLEKQSQARMIGYGGMLMESFVAVMAIVTASIIDQHLYFAMNASGALTGNTPETAAAYVNSLGLQGDPITAAQLTQAAEDIGEKKIVSLTGGAPTLAVGMSEVLHQFLGGAGWKAFWYHFAIMFEALFILTTIDAGTRVARFMVSDSLGNLGGPFTRFKDASWRPGAWLCSAIVVAAWGSVLLMGVTDPLGGIYTLFPLFGIANQLLAAIALTVVTVIVVKKGLLKWAWIPALPLAWDLIVTMTASWQKIFSADPKIGYWKSHDNAKIKLDAYEAARDSGQLPAGVADAAALEKQISDLEKIVRNTFIQGTLSIIFAVLVLIVAVVGVLVCVRAIRRGGSETTESPEEPSKIFGPSSFLATKAEKEVQQEWDELIASGQIRAPGARSGVHAH

>CORE_REP|Org163_Gene810#

MHRTGPSRDIDESELSVTPPAKQAAGVTAVGVALKRSVEEMGVIRTARTLARVNQVHGFDCPGCAWPEPTGHRRPAEFCENGAKAVAEEATLRTVTPEFFAAHSIAELSEKSGYWLGQQGRLTHPMVLRPGDTHYSPIAWDEAHRLIADTLRGLASPDEAVFYTSGRTSNETAFLYQLLVRSYGTNNLPDCSNMCHESSGAALSGSIGIGKGSVSIDDFAKADLIVVAGQNPGTNHPRMLSALAAAKAKGARIIAVNPLPETGLLAFRDPQTVKGVTTGVPLADDFLQIRLGGDMALFQALGRLLLEAEDRAPGTVVDRAFVDAHCAGYAEYEKHVRAVDLATVLEATGLSTAELEHTAEVFARSRNIILCWAMGLTQQADAVATIEEATNLLLLRGMIGKPGAGVCPVRGHSNVQGDRTMGIWEKMPAAFLDALDREFGITSPREHGFDTVAAIRAMRDGRAKVFFGMGGNFVSATPDTAVTEAALRGCALTVQVSTKLNRSHVVHGRTALILPTLGRTDLDLAPDGTKNQVSVEDSMSMVHLSTGRLKPVSDHLRSEVAIVCELARELFGAEHAVPWARFARDYDTIRDAIARVVPGCTGYNRKVRQRNGFQLPHPPRDAREFRTATGKANFAVNELTWLPVPDGKLILQTLRSHDQYNTTIYGLDDRYRGIHNGRKVVLVHPDDITALGFTDGDLVDVVSEWTDGTERRVEGFRLVPYPTPRGNAAAYYPETNPLVPLDHVAKRSNTPVSKAVTIRFEPSTGGKPVPAHADRDPA

>CORE_REP|Org5_Gene2538#

MSTTALAEPAFEGRPAAPEPEPSAAGHPRRVVFRWGTVTVVGVIVGYVAVLLANARHFYTDDTESQYTGLWLMMGRHLRDGHLPLMAPEQWMTGNYTMEEAGLFNPPQLLIDLIAPSIDNLALLATVVKLVFAIIAALGVYRICLVYGSRASWAAVAGIAFPLSGWFLFFDEASWVTSLTGIAWMLHAWASAVRYARGPAGDRSRYGAGAFGCGPIPLFVFLYLAISVEYVFPAVEAVLMLAAVAVGELVVLRAWRPVVRLAVVAGAAGLAGLMTYLPAMLSATVSWRGNSQINNDRFLTVPWSESLNASLPSAMPAFTSWWGYVQPLPVTYIAWFLIPALAFVDWDRARRAWRELISVGLFSIMFLMWAAGPGTVGPLRWPARVLPMVAVGLLILVCVLLGRFGTVRDVRARVIAAAVLVGLLFVRTFSADPHDVGWHLLSVLVVGAVGALAVWLGVRRGTAAAALVAIVAMFPIAYWQVSSVQPTPMGWNLPVHRAEAEAAFPKFDGVTIQLGDRALIQPGERTLEGAYGSLVFGNYAKDVRRDYVNGYTPNGHFWFGDMLCMRWDSSVCPDAFRRLFTPEPSTGRPPVDLMKLDRVVLQRALFPDAGNQPPPPGWKWADYPGHERYIRVLERENGPVSTSGGRISDAQGVQAVSLSESDRSSRARVSSPDGGRVVFARLNWPGYHATLDGRELSIAQVSKSFVAVDIPAGTANATLDVTWEPPGWRIGAAGAVGGLLVLILMQVLYLRTRRRDTASAVPEPEAAEPVS

>CORE_REP|Org87_Gene3703#

MTQTSERKSSAVEVEPGVFESVALIDNGSYGTRTVRFETGRLARQAAGSVVAYLDDETMLLSATTAGKTPKDQFDFFPLTVDVEERMYAAGRIPGSFFRREGRPSTDAILTCRLIDRPLRPSFVDGLRNEVQVVVTVLSLDPKDLYDVVAINAASASTQIAGLPFSGPVGGVRVALIPDQGANSAGGGQWVAFPTVEQLEGAVFDMVVAGRVVESGDVAIMMVEAEATEKVIELVEGGAQAPTEAVVAEGLEAAKPFIARLCRAQQDLAELAAKPTEEFPLFPPYGPDVYEAVEGAAEAELGEALSIAGKQEREEKIDEIKLAVLDRLAEQFAGREKELGAAFRSVTKKLVRQRILTDGFRIDGRGLADIRALSAEVAVVPRAHGSALFERGETQILGVTTLDMVKMAQQVDSLGPETSKRYMHHYNFPPFSTGETGRVGSPKRREIGHGALAERALIPVLPSQEDFPYAIRQVSEALGSNGSTSMGSVCASTLSLLNAGVPLKAPVAGIAMGLVSDTVTNDKGEQEVRYVALTDILGAEDAFGDMDFKVAGTREFVTALQLDTKLDGIPSQVLAGALSQAHDARTTILDVMAEAIATPDEMSPYAPRVTAIKIPVDKIGEVIGPKGKVINQITEDTGANISIEDDGTVFVGATDGPSAQAAIDAINAIANPQLPKVGERFLGTVVKTTAFGAFVSLLPGRDGLVHISKLGNGKRVAKVEDVVNVGDKLRVEIADIDNRGKISLVPVDENADEPAADAVDAGTE

>CORE_REP|Org125_Gene2067#

MATLSDRLDHVLGVKAAEPLADAFDMHIVEDLLRHYPLRYATQGQPLTEEAPEEGAHITVVGRVRKTELRPMRQRRGKLLKVELDTGSAKPVEITFFNGDKVSYLVKQGVRAMMSGTVHWWRPDRWNLSHPSYLILPETAESVDSLTSVRGGGALRGLAESAKGAGGVDISFFEREYIPVYPATAKVQSWDILACVRQVLDQLDPIDDPLPADLREDHELLPVSDALRLIHLPEHKSDIDQARQRLRFDEALALQLVLAQRRHDAAGRTARPCPPRSDGIAAEFEQRLPFELTAGQNKVIAEISGDLSRPHPMHRLLQGEVGSGKTIVALHAMLQVVDAGLQCALLAPTEVLAAQHYRSLRSMLGDLGAAGELGAADRATKVVLLTGSMSASAKKAALLDVVTGTAGIVIGTHALIQDAVEFFDLGMVIVDEQHRFGVEQRDALRAKAKDGITPHLLVMTATPIPRTIAMTTLGDLETSTLTELPRGRSPITTRVVPARMKPAWVERAWERIREEVAAGRQAYVVCSRIGDEEDDGAPKKGAKSKGKSRKQADEGTGEAPATHAAIDVFDTLRTGPLADLRLGLLHGRLPTDDKDRVMRSFNDGDIDVLVCTTVVEVGVDVPNATVMVIVDADRFGVSQLHQLRGRVGRGKHPGLCLLITETSPMGTAMARLEAVAGTLDGFELSVLDLRQRREGDVLGSAQSGTARSLKLLSLLDDLDVITTAQVLAREVVDADPGLTDHPGLANMMHAAVDSERLEYLAKS

>CORE_REP|Org113_Gene4327#

MERVTTTELRESAEGLLRELAGPEARLREDQWTAIEALVVHRRRALVVQRTGWGKSAVYFIAARLLRTQGRGPTVIVSPLLALMRNQVASARRAGVVAETINSGNVTDWDEIHARVASGEVDVLLVSPERLNNPDFRDSVLPKLAADAGLVVVDEAHCISDWGHDFRPDYRRIRTLIADLGEDIPVLATTATANDRVVTDVATQIGTDTLVLRGTLDRESLYLSVVRIPDAVQRTTWLSRQLAELPGSGIIYTLTVAAAHDLADVLTDHGYRVAAYTGRTDPGERETLEQALLDNEVKALVATSALGMGFDKPDLGFVVHIGAPSSPIAYYQQVGRAGRGLGTAAAATPTDTRGTAPSTNAHTMPDTGQPGTPADTQGTAPDTNVRAVPDAAPPGASVDPHADTSGTNVRAEVILLPGPEDRQIWNYFASVAFPREPVVRSVLAALDYERPLSTVALEPLVELSRSRLEMVLKVLDVDGAVHRVRGGWLATGQEWAYDTERYERLDRARAAEQQAMLDYQSTSECRMNFLRHQLDDPGLAADAPGCGRCDNCTGHRFDVEVGADEVAAIRARLDRPGIDLAPRKQWPTGLSKLGIPLSGKISDGPETGRVLGRLSDLGWGQRLRTLLDAPDGPAPDHIVDACIAVLREWDWTQRPGAIMALQSATHPKLAADLSARLAQIGRLTDLGVLHTRPDRPPVSAANSAHRVAALFDSWEPPDLTGLDTPVFLVDTHTDTGWTLTLAARTLRLAGAPAVLPLALATPT

>CORE_REP|Org98_Gene6070#

MSTDTDATPISDIPPGSAWPERLSSISRHDLPASIVVFLVALPLSLGIAIASDAPIAAGLIAAAVGGIVVGFLGGSPMQVSGPAAGLTVVVAEVIHQFGWQTTCFITAAAGLLQIVFGVSRIARAALAIAPVVVHAMLAGIGVTIALQQVHVLLGGASRSSAFENITELPGQLLTPHGDDFVIGLIVIGIIVAWRRVPDKVRLIPGPLVAVLVGTVLSLVLPGNPDRIKLDSSLFDAIGLPALPSGNWGGVVPAVLTIALIASVESLLSAVAVDKLHTGGRTNFDRELLAQGAANMTSGMLGGLPVTGVIVRSSTNVAAGARTRASTILHGVWILVFSIALVGVVQQVPKSALAGLLIVVGVQLVKLAHIQLAHRTGDLAVYAVTMVSVVFLNLLEGVLLGLAMAFAMLLWRVVKVSVRATPVAGTDRWIVRVDGTCTFLALPKLTKELATVPAGTDVLVELTVDFLDHAGYEAIHDWARQHESTGGNVEFVEIGTARMEHAMTRPPRRGRARGILDEVLGPWRERNGDAVAAGVAAYHRSHAHVMRPHLDQLRDRQDPHSLFLTCADSRIVPNVITNSGPGDLFTVRNVGNLHPADGSDASVEAALSFAVDNLKVHNVVVCGHSSCGAMKTLLAGTSAGPGLDTWLAHARPSLDAYRAGHPVRAVAAAAGYDETAQLSMVNVAVQLEILQRHPVIRQAAAARGLTVSGLFFDISTACVLEVTTDSISEIGRGPLKGAIDADRGKSGSTGIRPTRSPAQSRTA

>CORE_REP|Org96_Gene538#

MTLSHIEEPGDVAHLVGNFAEVPLTEHDPEPAAVDAAQVEEFVRTAAAANNYTPEQLTWSTPEGIDVPPVFTKADRDAVAAAGYPLDSVPGVAPFVRGPYPTMYVNQPWTIRQYAGFSTAADSNAFYRRNLQAGQKGLSVAFDLATHRGYDSDHPRVQGDVGMAGVAIDSILDMRQLFDHIPLDQVSVSMTMNGAVLPILALYVVAAEEQGVKPEQLAGTIQNDILKEFMVRNTYIYPPKPSMRIISDIFAYTSAKMPKFNSISISGYHIQEAGATADLELAYTLADGVEYIRAGIDAGMEVDKFAPRLSFFWAIGMNFFMEVAKLRAGRLLWSELVSKFEPKNSKSLSLRTHSQTSGWSLTAQDVYNNVARTCIEAMAATQGHTQSLHTNALDEALALPTDFSARIARNTQLLIQQESNTTRPIDPWGGSYYVEWLTHQLAERARAHIAEIEAHGGMAQAISEGIPKLRIEEAAARTQARIDTGQQPVIGVNKYQVEEDQAVEVLKVENSRVRAEQIEKLRRLRAERDQDAVDRALAELTRAAAATQGGMENNLLALAIDAARAKATVGEISDALEKVYGRHQAEIRTLSGVYRDEAGKVTNITIAGKLVEEFAEAEGRRPRILVAKMGQDGHDRGQKVIATAFADLGFDVDVGPLFQTPEEVAQQAADNDVHIVGVSSLAAGHLTLVPALRQALADVGRPDIMVVVGGVIPPGDFDELYQAGAAAIFPPGTVIADAAIDLLRKLAGELGHELEGSAAE

>CORE_REP|Org77_Gene3469#

MTIATTDEHKAVQESMSGWAAAVRPIATMRDDTTGFWRMYWGQLTDLGIFRVAVDEQAGGAGGSITDLAVLVEQAAHDLVGGPVLTTALAGVVTGGRLDEQQPCGVALDTVVESASSLSAVDRPRQGDGGELVLNGVWETVLGAAPGAAVLLPVRIADGSRWCLIPAEAPGVTIEPLPALDPSTPLARVRCAEVQVPAADVFAPDFAVEDLIVALTTAELAGVAGWCLETAVEYAKVREQFGKPIGSFQAVKHICAWMLCRTELIRSVAADAAAAADDALANPAGSELPIAAAIAAAISLDAAVDTAKDCIQVLGGIGFTWEHDAHFYLRRATALRQLLGGGGYWRARVTELTRAGARRTTGADRILADVATETASSGEGVGAAVGVAAAVGNAAAVSLELGAAATGVAAEVAAIAALPVDQQRKAMVEAGLVMPHWPEPYGRAADPMTGLLISEELARAGLETPDLAIGGWAVPTLLQHGTPEQMERFVWPTLHGDVVWCQLFSEPGAGSDLAALRTTATKVDGGWMLRGQKVWTSLADTANWGICLARTDPAAPKHKGISYFLVDMRSAGIEIRPLVQITGEAKFSEVFLDDVFVPDECVVGALGNGWKISRATLSAERIAMGGKGIGQVLEELVGKLPATGPGTELINDRLGRFVADATAGLLLEQRGAALILAGADAGPQSSVRKLVGVRHRQEVAEFAVEVAGPAGALDNEATKEFLLTRCLSIAGGTEQILLTLAGERILGLPRDGS

>CORE_REP|Org45_Gene2052#

MSTVEGRTRGALLARQAGGALGAAVLAAVVAAAVAGVGLVAFASVEWPAFNSSNVTRALTTVGQVVAVAMLVAAIWLVRARKWPWVAKVLSWGGISAFVTVTLGMPLGATKLYLFGLSVDQEFRTQYLTRLTDSAALRDMNYVDLPPFYPAGWFWAGGRFANLFGLDGWEAFKPWAIIALAVAAALALVLWSELIRADWAIAVAAATTAVTVAYAAPEAYSAVLVVLLPPVLVLAWGALHRPAEYGGAEYQTDAARATGSKAADSGAVSTDAAGAKVAGAKAVGSGAGSSETAGTSATGSGAASPGVASTSTASTEAASTSTASTGATASGMVGAVPTAGGWGAVLGTGLFLGLAATFYTLYFAAAVFAVCLMAVAAAGIALWQRHFALRVHRRTKREVPGVWRLLWPILLRLIAIGVVAGLLALVVWLPFLLRVLDEGFPSSGTAFHYLPEGGARLPLPMFEFSLLGGLCLIGVIWLVLRVGSSRRAQALAVGVVAVYLWCLLSMLVTAAGTTLLSFRLEPVLMVLLAAAGAFGFVEGARAIYQVLNEPERFRAVVAAVAVLGAIGFGQQIPEILAPEITTAYTDTDGDGVRADKRPPSAVSYYDEIDAALREQIGRPRDETVVLTADLSFLSIYPYFGFQALTSHYANPLADFPARADEIKRWSTLETPEELLDALSTAPWRAPDAFLFRQSGDNYTLRLAEDVYPNQPNVKRYQVTFPATLFDDPRFTVTDIGPFTLVVVEH

>CORE_REP|Org97_Gene3126#

MIVSVSETETVPQQPRLPAAPPAATPPPTTAAAAQLPADRYLNRELSWLDFNARVLALAEDPSEPLLERAKFLAIFSSNLDEFYMVRVAGLKRRAEAGLSVRSADGLSPTEQLTLIAERTQELAGRHARVFLDQVRPALADEGIAIIGWADLDDDERRRLSGYFLDQVFPVLTPLAVDPAHPFPYISGLSLNLAVTVKDSETGGEHFARVKVPDNVDRFVRVRRTLNETPGARRDSDITAVPRLAAFLPMEDLIAAHLDQLFPGMEVVEHHSFRITRNADFEVDEDRDEDLLQALERELARRRFGSPVRLEVSDDMTEHMLELLLRELDVDPGDVIQVPGLLDLSCLWQVYGVDRPLLKDAPYVPATPPAFGERETPRNVFAALREGDVLVHHPYDSFSTSVQRFIEQAAADPQVLAIKQTLYRTSGDSPIVNALIDAAEAGKQVVALVEIKARFDEQANIKWARALEQAGVHVVYGLIGLKTHCKTCLVVRREGATIRRYCHIGTGNYNPKTARLYEDVGLLTAAPEIGADLTDLFNSLTGYSRKANYRNLLVAPSSVRSGIVERIRRETELAAQGVPARIRLKANAIVDEQIIDALYRASQAGVPVQIVVRGICGLRPGVPGMSDNIEVRSILGRFLEHSRILHFQAQDEYWIGSADMMHRNLDRRVEVMAQVKDPKLRERLAVVFDSALDPATRCWVLQPDGSWAAQPSPDADSRGVQVRDHQEFLMRLRRPDQQ

>CORE_REP|Org46_Gene6654#

MASSCSPPNKPLPGDRAGHHGVAVTGRTTRVAPQAATARAASRSSGASTSERQETVAYRADLDGLRGVAIGLVVIFHVWFGRVSGGVDVFLVLSGFFFTGLLLRRADSTGSPGVGTTLRRTVRRLLPAMVVVLAAVVVASVIVRPYTQWWELSAQTLSSLLYVQNWRLALTWSDYLAADPSVSPLQHLWSMSVQGQFYLAALATVAVAAWTTRRSMRSAALRPVLAVTVGVLGVVSFWYAWRGGQTQQGWNYYDSIARCWELLAGALLAAIAPLLSPPRMARAGLAALGLFGVVGCGWLILDGANRFPGPAALLPVAAAAGVIVSGNNLPLDQRPWPNRILATPTARWLGDIAYPLYLWHWPILIFYLTERGQPHAGVAGGIVIVTLSIVLAWVTHRWVEEPLRLRSRPRAEAAGAEGTTISRRVAGVAVVALGAVVIAAAGGWLTVMARINPPHAVGALDPRLYPGAEALASGAAVPQAPMRPTVFEAPGELPPPTVDGCIADWDTREVITCTYGVPDAERTLAVVGSSHAEHWLPALQVLAGEYSFRIQVYLKMGCPLTLAEDAMYKGEPIPDCRDWSREVIDRLGADRPDWVFTTGTRPREDIGDETPPEYLDVWSALSERGLNVIAIRDTPWLRREKVRYMAIDCLAKGGDRIGCGMRRQDALDEVNPALEPASRYPNVFPVDLSDAVCEPTVCAVIEGNVLIYHDEHHFTVSYSRSLADALGRRLQPLLGWW

>CORE_REP|Org7_Gene5304#

MALDSGTGTISDLGGVAAPIAKTVPTERAHHGDVFVDEYEWLRDKENPEVISYLEAENAYTEAQTAHLAGLRDSIFDEIKSRTQETDLSVPTRMGDYWYYSRSFEGKQYGVHCRCPIAADAEGIDAWTPPQLEAGTEVPGEQILLDSNVLAEGHDFFALGAYSISHDGNLLAYSVDTNGDERYVLRFKDLRTGDLLPDEVAETAPGATWSLDGTHVFYQTVDESWRPDTVWRHRLGTAPDADVKVFHEPDERYWVSVVSTRSEKFLMIWVGSKITTEGWVLESDNPEGEFRVILPRREGVEYSAEHAVVGGEDRFLILHNDVVDGVKAENFVLADAPVADPSNLTLLIGHRDDVRLEDVDAFADHLVLSYRREALTRVTVWPLTESGYGERKELDFDLELFSVGAGANPEWAQPTLRIGLSSFITPVQVFDYVPATGDLLLRKEQPVLGGYDANDYEQHRDWAVAEDGTRIPISLVWKKGGLAHSRLASAPGVPLDAPKPLLLYGYGSYEASMDPSFSVSRLSLLDRGMVFAVAHVRGGGEMGRLWYENGKTLTKKNTFTDFVSCARHLIDTGVTAADRLIADGGSAGGLLMGAVANLAPELFTGILANVPFVDPLTSILDPSLPLTVIEWDEWGNPLADKDVYDYMKSYAPYENIEAKDYPAILAITSINDTRVLYVEPAKWVAKLRATKTGDAQLLLKTEMSAGHGGVSGRYEKWKEVAFEYAWVLDRVGLAGA

>CORE_REP|Org103_Gene2295#

MTTSAIHHTRDSGGIVTLTIDDPNQRVNTMNSLFVESLAAELDAIENDADVTGVILTSAKKTFFAGGDLNDLRAARRDRIDEFAAFVQRNSVLLRRLEKLSVPVVAAINGSALGGGLELALAAHHRIVVDAPGVTLGLPEVTLGLLPGAGGVVRTVRLLGVQAALRDVLLSGKKHPVAGALELGLVDATVATIEELIPAATAWIREHAGARQPWDTEGFRIPGGAPGERGAPLHATLPALAATLRAQTKGAPAPAQANILAAAVEGAQVDVDNALAIEARYFLDLAIGQIAKNMIQANFFDMQVVNGPRGRDTAREPWLPRKAIVLGAGMMGAGIAYQCAVSGIDVVLKDVTPEAAERGKGYSLRVLDKRVRAGQISAATRDEVLARITPTADVAAAAGADLVIEAVFEDPALKADVLREIEPLLAPDALIGSNTSTLPITGLAENVSAPDRFIGLHFFSPVDRMPLLEVIKGGRTSSETVSRALDLARTIGKTPIVVNDSRGFFTSRVIGTFVNEALAMLGEGVPAPVIEQATTQAGYPAPALQLADELNLELLRRVRDASRVAAEAAGGSWDPHPAEAVLDRMLGEFGRAGRLAGSGFYEYEDGARTRLWPGLRAAFGSPRADLPFTDLKERMLFVEAIESVKCLDEGVLESVPDANIGSLLGIGYPGWTGGVLQYIDGYPGGVAGFVRRAEELAAAYGARFAPPSSLVAVARDGGTLADAHRERQPALS

>CORE_REP|Org101_Gene3640#

MAFATEIPTEGYEDRAAGATPLAHSEHRPVGEIERTEGQFQVVSDHQPAGDQPAAIAELERRITAGERDVVLLGATGTGKSATTAWLIERLQRPTLVMAPNKTLAAQLANELREMLPNNAVEYFVSYYDYYQPEAYIAQTDTYIEKDSSINDDVERLRHSATSSLLSRRDVVVVASVSCIYGLGTPQSYLDRSIQLEVGTEVDRDALLRLLVDVQYTRNDMAFTRGSFRVRGDTVEIIPSYEELAVRIEFFGDEIEALYYLHPLTGDVVRQVEMLRIFPATHYVAGPERMERAVRDIEAELEERLAELERQGKLLEAQRLRMRTQYDLEMIRQVGFCSGIENYSRHIDGRPAGSAPATLLDYFPDDFLLVIDESHVTVPQIGGMYEGDMSRKRNLVEFGFRLPSAVDNRPLTWEEFADRIGQAVYLSATPGPYELGQVGGEVVEQVIRPTGLVDPQVVVKPTKGQIDDLVHEIRVRTERDERVLVTTLTKKMAEDLTDYLLGLGVRVRYLHSEIDTLRRVELLRQLRLGEYDVLVGINLLREGLDLPEVSLVAILDADKEGFLRSSTSLIQTIGRAARNVSGEVHMYADKITDSMQHAIEETDRRRAKQVAYNTEMGIDPKPLRKKIADILDQVYKEADETEVEVGGSGRNASRGRRAQGEPGRAVSAGVYEGRDIKSMPRAELADLVKELTAQMMNAARELQFELAGRLRDEIADLKKELRGMDAAGLS

>CORE_REP|Org81_Gene2367#

MTERIQVGGLQVAKVLHDFVENEALPGTGVDSAAFWSGAEAVINDLAPRNRALLTERDDIQAKIDEWHRANPGTGYDKAAYKQFLTEIGYLRPEPADFQIGTENVDAEIATTAGPQLVVPVSNARFAINAANARWGSLYDALYGTDAISEENGAEKGTGYNKVRGDKVIEWARNFLDDAVPLITGSHVGSTKYSIEDGELVVGLEDGTDIGLADPSALVGYLGDPANPTSVLLKHHGLHIEIQIDPSSPIGSTDTAGVKDVVLESAVTTIMDFEDSVAAVDAEDKVLCYHNWLGLMKGDLAEEVSKGGKTFTRTMNPDRVYTALDGSQLVLHGRSLLFVRNVGHLMTSDAILDADGNEVPEGIMDGLLTVLIAKHALNGDTKLKNTRTGSIYIVKPKMHGPDEVAFTNELFDRIEQVVGLPANTLKVGIMDEERRTTVNLKACIHAAKDRVVFINTGFLDRTGDEIHTSMEAGPMVRKAEMKGQQWILSYEDFNVDTGIATGLPGKAQIGKGMWAMPDLMADMLVQKVGHPKAGANTAWVPSPTAATLHATHYHLVDVVKRQAEIAKGGARASVDQILEIPLAADTNWSAEERQQELDNNSQGILGYVVRWIDQGVGCSKVPDIKDVGLMEDRATLRISSQLVANWLRHGVVSEDEVIASLERMAPVVDRQNAGDPSYRPMAPDFASSIAFQAAKELVLEGTKQPNGYTEPILHRRRREAKEYNAKFGA

>CORE_REP|Org79_Gene341#

MAQEVLKDLNKVRNIGIMAHIDAGKTTTTERILFYTGINRKVGETHDGGATTDWMEQEKERGITITSAAVTCFWNNNQINIIDTPGHVDFTVEVERSLRVLDGAVAVFDGKEGVEPQSEQVWRQAAKYDVPRICFVNKMDKLGADFYYTVGTIVDRLGAKPLVMQLPIGAEDDFDGVVDLIDMKALLWPGKVETGTPPQIQEIPEDLKEKAEEYREKLLETVAESDEELMEKYFGGEELTKEEIQAAIRKLTIASEVYPVFCGTAYRNKGIEPILDAVVSYLPSPIDIGEVHGTSVDGEEDLTRKPSVEEPFSALAFKIAVHPFFGKLTYVRVYSGQAIPGEQMLNSTKSKKERVGKLFQMHANKENPVEHADAGNIYAFIGLKETTTGDTLCNPDHPIILESMDFPDPVIQVAIEPKTKADQEKLGTAIQKLAEEDPTFTVQLDEETGQTVIGGMGELHLDVLVDRMKREFKVEANIGSPQVAYRETIRKKVESLDYTHKKQTGGSGQFAKVIVTIEPYSPDPEELEEGESASYKFENAVTGGRVPKEYIPSVDAGIQDAMQYGFLAGFPLVNIKATLEDGAYHDVDSSEMAFKLAGSQVLKEAVAKAKPVLLEPVMAVEVVTPEEYMGTVNGDISSRRGQVFAMEDRSGAKVVKAKVPLSEMFGYIGDLRSSTAGRANFTMVFDSYAEVPQSVAQEIIDERNGNK

>CORE_REP|Org105_Gene3911#

MLAVTRSDCHRFTKLCDMADLAPSTVYIASPEGDTGKSTVALGVLQMLCATTARVGVFRPITRSTDEPDYILELLLEHSTADIEYAQAIGVTYEQVHADPDAAISEIVMRFHEVAKVCDAVVVVGSDYTDVASPSELRYNARIAVNLGAPVLLVVRGSERSPDEVKQLAELCSSELSAEHAQLVAIIANRCAPDQLDQVCAALSGFAVPSWTLPEVPLLIAPTMAELCAAIDGEMYSGDPELLHREAMKIMVGGMTAEHILERLEDGEVVIAPGDRSDVLLSVVNAHEAEGFPSLSGIIMNGGLLPHPAIARLMTGLKPRLPILTTSLGTYDTAGAAHRTRGRMSADNPRKVDTALALMEQHVDAGEFLRRLEVPRSTVVTPQMFEYQLIERARADRKRIVLPEGDDDRILRAAGRVLQRKIADLIILGDENAIRARAAELGVDIADAEVLDPRTSEHLEDFAREYTELRKHKGMTLERARETVTDISYFGTMMVHKGIADGMVSGAAHTTAHTIRPSFEIIKTVPGVSTVSSVFLMCLADRVLAYGDCAVVPDPSSEQLADIAISSAATAERFGIDPRVAMLSYSTGESGSGADVDKVRVATKLVRERAPQLLVEGPIQYDAAIEPTVADAKLPDSEVAGRATVFIFPDLNTGNNTYKAVQRSAGAIAIGPVLQGLRKPVNDLSRGALVADIVNTVAITAIQAQGE

>CORE_REP|Org163_Gene4874#

MRSATVQRDQARTRNAKESAAVTTSAPISPVALVRVPAGTTAGAAVREAGLPTKGPETVVVVRVDGELKDLSWTPDTDVDVEPVAANTDDGRNVIRHSAAHVLAQAVQQEFPGAKLGIGPYIKDGFYYDFRVERPFTPEDLAKLESRMKKIVKGAQRFSRRVVEVEDARVELAGEPFKLELISDKSGIDDPEVMEVGGKELTIYDNLDPRTGEKIWGDLCRGPHIPTTKFIPAFKLTRSSAAYWRGDQSREDLQRVYGTAWESQEALDEHLHLLAEAERRDHRKLGLELDLFSFPDELGSGLPVFHPKGGIIRKELEEYSRRRHVAAGYEFVNTPHITKGHLFEVSGHLDWYRDGMFPAMHLDAEFNEDGTVRKPGQDYYVKPMNCPMHNLIFRARGRSYRELPLRLFEFGSVYRYEKSGVVHGLTRVRGMTQDDAHIYCTKEQMHSELTDTLRFVLDLLKDYGLDDFYLELSTKDPKKFVGSEEIWEEATETLSKVASASGLELVPDPGGAAFYGPKISVQAKDALGRTWQMSTIQLDFNLPERFDLEYTASDGTKQRPVMIHRALFGSIERFFGVLTEHYAGAFPAWLSPVQVVGIPVAEAFAPHLDRVIERLQDEGVRAQVDRSDDRMQKKIFNNTAQKVPFMLLAGERDVNANAVSFRFRDGTQVNGVPVDDAVATIVAWLANRENASPTADGFEIRSSKGGA

>CORE_REP|Org14_Gene504#

MPELPPVPTLLATAVEALGGTARTGQQTMSAAVAHSIDTKEHLAVQAGTGTGKSLAYLVPSLRHAVQSGRTVVVSTATIALQRQLVDRDLPRLAQALEKPLGRRARFAILKGRNNYLCLHKINSAIPDEPPETELFDAFAISRLGREVQRLNEWASDTETGDRDELVPGVSDRAWRQVSVSARECLGKSRCPFGTDCFAERARAESGQADVVVTNHALLAIDAISGIQVLPEHDVVVIDEAHELVDRVTGVATAELSTATITAAAKRCTKLVDEGDLDRLEGAAEAWHELLEDLPAGRWDELPAGGDQVLALLRDAAWTVRTALAPPGSGAPQGDPEAAAARNMAVAAVEEVHDTAVRALTAFEEPDPAARRDVLWLAVDEVRGVARRTLHMAPLSVGGLLRSRLFGTATVILTSATLQVGGSFDGLAVTWGLPPQTPDRAGTDVPGADRADKGIRRIDPAMANGAEAPADTSAMRWNSLDVGSPFDHAKSGILYVAKHLPAPGRDGLAPSYLDEIERLIEAAGGRTLGLFSSMRAAKAASEILRERLDTPILCQGEDATGTLVRRFADDPATSLFGTLSLWQGVDVPGPSLSLVILDRIPFPRPDDPLLVARQQAVQARGGNGFLTIAANHAALLLAQGTGRLLRSVHDRGVVAILDSRLVTARYGGYLRASLPPYWETSDPEVVTKALRRLTATAADLTAAAEK

>CORE_REP|Org101_Gene6880#

MRELVTDVIDVGADFPVARKALWDLFLEPQTYPRLFAGIGACELVEESPDSRIVRVRIGTAESGIRTHQLRLTVRRWYESFELQCPGTGSFVSVRLRGDEERTKIVVTVFAPGRLHPGIAEGSNAAVMSWVNAGLRRAVDVIRGARTSTVVNAENSPLRRQVSVAKQMVTVGVGRTSSLATGVKQARSLAKWGFNLAGGYATAAAYAPDRIAIIDDSGTRTFAEMHTRTSALAGALAALDLGFGDTIGLLARNHAGMVECMVAAGKLGVDVALLNVGLSGRQIEDIVQRHRLAALFVDGDLEQLVHYLHADLPRFNTDGRPPVPGRATLDDLIAEGERPFRLPNRAGRLIVLTSGTSGTPKGARRPEPKGFGTIAALLSRIPLPMAEPMLIPAPLFHTWGLAGLQISTPLRATVVLPERFDAEDCLRLVAEHRVASMIVVPTMVHRILDLPAAVRDRYDTASLRAVVSCGAPLAGATVLQFMDVYGDILYNVYGSTEVSWATIATPDDLRTAPTTAGRPPLGTRIAVLGPDQRPVPLGVTGHIFVGNHMLFDGYVNSAPPTEADGMLDTGDLGYLDVAGRLFIAGRDDEMIISGGENVFPRPVEEALAHLPQVSEVAVVGVPDQEFGQRLAAFVVKREGAGLDSDMIRSYIRHRLSRFSVPRDVTFLPTLPRGETGKIIKHLLTGGPPAEGSAQSPLGGPHLVT

>CORE_REP|Org63_Gene3321#

MTDTDLLATPGVESNPTGDRESESGQISKMIENTDVARSGLTGMLLPQLRALAGELGIRGTSGMRKGDLIAAIKENQAAGKSAKVEKPAKSEAKSAATAKADAPAKNAAPAESAPAKAEQATLDVTPAASAAPTEAPAPAKTAPADSAPAAESASSKATEATETTEESGRESGQRGRGRQRRGRDQARSASAETAPAETRADEPKQDSEQGGERRRERGQGERQSERGQGERGERGQGERGERGQGERGERGERGQGERGERGERGQGERGQSQNGSAGGRGGDDEEGGRGRRGRRFRERRRGRDRDGGGGEARELEIREDDVLQPVAGILDVLDNYAFVRTSGYLAGPNDVYVSMNLVRKNGLRRGDAITGAVRAPRDGEQANQRQKFDPLVRLDTVNGGDVEAAKRRPEFGKLTPLYPNQRLRLETQPNKLTTRVIDLIMPIGKGQRALIVSPPKAGKTTILQDIANAIATNNPEVYLMVVLVDERPEEVTDMQRSVRGEVISSTFDRPPSDHTSVAELAIERAKRLVEMGRDVVVLLDSITRLGRAYNNSSPASGRILSGGVDSTALYPPKRFLGAARNIENGGSLTIIATAMVETGSTGDTVIFEEFKGTGNAELKLDRKIAERRVFPAVDVNPSGTRKDELLLSPDEAAVLHKLRRVLSGLDSHQAIDLLIDRLKKSKNNLEFLMQVSKTAPGALDE

>CORE_REP|Org51_Gene2871#

MWSGARKSCRPCGRPDPSDRRENRPVSSYPHLFEPLDLGFTTLRNRVVMGSMHTGLEDRAWDIDKLAAYFAERARGGVGLIITGGYAPNRTGWLLPFGAKLTTTTEAYRHRTVTRAVHAHGAKIALQILHAGRYSYLPGSVSASSIKAPINPFRPRKLSARGIEQTIRDYVRCAELARLAGYDGCEIMGGEGYFLNQFLAPRTNKRTDEWGGSAANRRRLPLEIVRRIRAAVGPEFILIFRLSMAELVEGGQTFAEIRELARELERAGATIINTDIGWHEARVPTIVTSVPRAAFVEFTAKIAREVSIPVCASNRINMPEVAEEILTRGDAQLISLARPLLADPDWVAKAAGGREDEINTCIACNQACLDHAFQRKTVSCLLNPRAGHETDLVLAPTRRTKHIAVVGAGPAGLAAAVNLAERGHRVDLFEAEDRIGGQFDIARRIPGKEEFEETLRYFDRMIAKTGVRLHLNTRATAEDLLAARYDEVVLATGVRPRVPDIPGIDHPMVLTYAELVREAKPVGRRVAVIGAGGIGFDVGEFLTVDGHPTLKLDEWKQEWGVDADDERAPGQLRAPRPAPAAREVVLLQRKDSPFGRSLGKTTGWVHRAALRAKGVEQVGGVNYERIDDEGLHISFGERRARPRVIRVDNVVVCAGQESVRELAEPLRAAGVRVHLIGGAELAAELDAKRAIDQGTRLAARL

>CORE_REP|Org9_Gene5213#

MTTPRRPRRTASRPAGAPDPAEQRRAAEAAAERAESARPAEPAAEPAAEPAADRPGGSAGEPTAPAGTDQPTAPAKAERPARGDRGDARTQRQDKPARGDSGEGRSAGGRRSGRGGRQGRGRAEQLPAQPPVVAAQDRLGAPPKLPKNGLRVFALGGIGEIGRNMTVFEYGGKLLIVDCGVLFPEDQQPGVDLILPDFRPIEDRMDDIVAIVLTHGHEDHIGAVPFLLRNRSDIPVLGAKFTLALVAAKCREHRLHPKLIEVTEGETTSHGPFECEYFAVNHSIPDALAVAIRTPAGVALHTGDIKLDQLPLDGRLTDLAGFSRLGDEGVDLFLVDSTNAEVPGFVTPEREIGGVLDTVIGKARGRVIVASFASHVHRIQQVVDVAQKYGRRVCFVGRSMVRNMQIAQDLGYLTVPDGVVVDLDVAATLPGDRLVLISTGSQGEPLSALSRMARGDHRQINIRPDDLVVLASSLIPGNENSVFAVVNGLARLGASVITQQNAKVHVSGHASAGELLYLYNAVRPTNAMPVHGEWRHLRANAALAVATGVPEERVVLAEDGVVVDLVDGIASIVGRVPVGHVYVDGLSVGDVGESTLSDRLVLGEGGFISITVAIDETTGKAVSAPELSGRGFSDDPTALAEAAELVEAELLRLAGEGITDTHRIAQGVRRVVGRWVADTYRRRPMIVPTVIGV

>CORE_REP|Org101_Gene6585#

MRSGRQVAQRFVSVARVVTAVAVLVVGGLFAVDSLSGAAAAEPSSDSEGQRSERWTALHDGPQPYADVHIDWDVPIRMSDGVVLKANVYRPMDAAGQIEQRPLPTIVNLTPYSKLASNLVDSALAIPNLQPMAVDLIRRMDLSGTPVSGFEDLLHALDGGTVHTVLGIDRDLIRSGYTQVIADVRGTGFSQGMWDTLGAREQLDTREVIEWAAAQPWSTGKIGMNGGSYAGINQLRAAENAPAALKAIFPVTPGSDLMRDVVAPGGGIGTTFMPLWLSNVNQMKVLPDVRSMLDGTFDWQWFHDRMADPSTNYDLLVQALVTPSLDAVPPALAELLDENSELRQGILGHPERITVPTFVYSGWHDIFANSATKLYNAIPLAPSRKQLIVGDTYHANPGSGTGFPGAPPRLPVLQRVWFDKWLKDIDHGIDDFGPVTVWEQGGGWITLGEFPQTGVTHRRVYLTAAPSGTATSVYDGSLSTARPEETETLTVAPGLSTVCSRDAAQGSAGLTAALDMCAKDSRIAEQNALTFTGAPVAEPTVISGPINVHLNTVHDATDGYWSVTVNDVAPEGTSTVLSTGQLTASMRAVDEAKSARSANGDFTAPYNPITLDRLLPVVPGEPTALDIAVIPVQAVLQPGHRLRVDVFAGNSPKALAFRPLLNNTELKPQSVLLDPAAPSFVNLPTSRPLD

>CORE_REP|Org141_Gene5357#

MDESAGARPAPRRPRGSCARRFRAGVGCDHRRRYHLGVTEHVEQLEFQAETHQLLELMIHSVYSNKDTFLRELISNASDALDKLRLESYKDKDLHVDTSDLHIELEVDTDGRVLTVRDNGIGMSRAEVVDLIGTLAKSGTAQLRKQLSEAKSEAAAEELIGQFGIGFYSTFMVADKVTLTTRRAGETEATRWVAEAGSSTYSIETVEDAPQGTAVTLQLKAADEDDHLFDYTQEWKLREIVKKYSDFIAWPIRMQVERTVTEGEGEDAQEQTVVEEQTLNSMKALWTRPKSEVSDEEYHEFYKHVSHAWDEPLEIIPLKAEGTFEYQALLFIPSQAPFDLFTREHKRGVQLYVKRVFIMDNCEELMPEYLRFVKGVVDAQDLSLNVSREILQQDRQIQMIRKRLVKKVLSTVKDVQGAEDQDNYQTFWREFGRVLKEGLLSDFDNRDTILAVSSFASTASESDLATLAQYVERMKDGQGSIYYMTGESRQQVESSPHLEAFRAKGLEVLILTDPVDEMWVGSVPEFDGKPFVSIAKGEVDLETEEEKKESEQLREQQDKDYAELLGWLGKTLADSVKEVRLTNRLTTSPACLVGDVFDFTPMLERMYRASGQALPETKRILELNPTHPLVTGLREAYDTRKQDADEGKVPELGETAELLYGTAVLAEGGELKDPAKFAHILTDRLTRTL

>CORE_REP|Org102_Gene3397#

MASYDEPEPDSNLRAGYGDLPDPPGDESNGGPAVPPRDGLGDPPDVPDDYLPSSSGPSHRSIFPPIDDYAFLSDCETNCLIARNGSVEWLCLPRPDSPSVFGAMLDRSAGHFRIGPYGRNVPAARRYLPGGLIVETTWQTETGWLIVRDALVLGPWHNTTQRSRTHRRTPMDWDAEHMLLRTVKCVNGVVELEMSCEPAFEYHGAPARWEYTGEVYEQATARGGDDCPALTLTTDLRLGLEGREARARTRMQEGDQVFVALSWSELPPPRTFADAAEKMWQTTECWRQWITLGRFPDHPWRGYLQRSALTLKGLTYAPTGALLAAATTSLPETPGGERNWDYRYTWVRDSTFALWGLYTLGLDREADDFFAFLHDVCRDDNGDPVALQVLYGIGGEREIREYELPNLSGYDGARPVRIGNAAYQQDQHDIWGTLLDSVYLHVKSRQQVPETLWPMLERQVQAAIENWRKPDRGIWEVRGEPQHFTSSKVMCWVALDRGAKLAELHGEYDYAKKWRDIADEIHADVLEHGVDSRGVFTQTYGNDALDASLLLVVLNRFLPPDDHRVRATVLAIADELTENGLVLRYRTETTDDGLSGAEGSFTICSFWLVSALVEIGELQRARHLCERLLGYASPLKLYAEEIDTRTGRHLGNFPQAFTHLALINAVMHVIRAEESHGAGQFNPAHPGR

>CORE_REP|Org110_Gene6706#

MSSALDPSASPDLESNRPAATYAVVIALSAAIAAVVAALVVGLSAAQALSLLGIPDPGALTTYGLPAVRALADLSAALTVGSLLFAAFLVPPQASGLLDVGGYRAVRRASNFALLWACCAALLIPLTVSDTTGQPVRDTLDPVGLWRAIDQIELAGAWRTTVLFALIVAVGARLALRWGWTPVLFGAAIATMMPLALTGHSSSGGAHDVATNSLILHLVSAAVWVGGLFALLAHARRGGAHTDLAARRFSLTATFAFATIGVSGVINSWVRVPWDELFTSTYGRLVLAKAAALVLLGLFGYAQRRAALPALAADPKDRGALIRFAGVEVLVFAATMGLAVGLGRTPPPPPTSIPTPAEVELGYNLAGPPTVARMLFDWRFDLIFGTLAIILAIGYLLGVRRLRARGDAWPIGRTIAWLSGCVVLLLATSSGVGRYAPAMFSVHMGAHMALSMLAPILFALGGVVTLALRALPPAGRGGAPGPREWILAAVHNPVSRFLTHPIVASVIFVGGFYALYLGGIFDTFADSHGAHLLMNLHFLLSGYLFYWVVIGIDPKPRQVEPLTKLAMVFGSLPFHAFFGIALMSMTTVLGGWFYRGLGLGWNGDLLGDQRTGGSLAWASGEVPLVVVMLALLIQWSRSDKRLAQRTDRAADRDHDADLAAHNAMFAELAKRDRGPQKSAGDPAQP

>CORE_REP|Org158_Gene1247#

MIGQILEGRYRIDAPIARGGMSMVFRGEDTRLDRPVAIKVMDPKFAADPQFLTRFELEARAVAKLKHPALVAVYDQGVDGDHPFLIMELVEGGTLRELLRERGPMPPHAVRAVIEPVMQAIGVAHSSGLVHRDIKPENVLISDSGEVKIADFGLVRAVAAANITSASVILGTAAYLSPEQVTSGHADARSDVYAAGVLIFEMLTGRTPFTGDNSLSIALQRVENDVPSPSHHISGVPPEFDELVAHATAREPAHRFADGNEMAAEIRRIAQVLQLPAYRVPAPQESAEHLSARYRVGPTPAPAAPAESRSRPAPVGAADMTTRLPAEPPTTRVPQAAAPPAHQHTRVMTAARELPPDYAQSAAHAPPPTLPPHGDQPPRNGYLADRGRSRRTAVLWLGAVVVLALLLGIGGWWLGVGRYEAVPAIAGMDRERAVATLQAAGFDTEVRDKASDTIPMGNVVGTDPSAGTKVVKGSTVAVLISSGKPKVPDIRPGQDVQSVKQAIRDAGLTPVDAGEVSSTAAEGTVAKVDPDPGTILPMGADVKVYTSKGSAPVELPNVRGKTEEEARAALDAVGIEVTGTRVEFDSKVKAGEVAGTDPAAGTTIDSSQGVVLLISNAVEVPGLLGSSVGDARAKLEALGLGVSVRQLAPSDSSIVISQSSVPGAKVEPGSTITLVALP

>CORE_REP|Org109_Gene6329#

MSPPLPWAPSPCILRSSARRSATAYCGRGGYAEYLPRFDTVRRATSSTGGALAVSSAPITARSRRFPRRRQESATGLDRTVEFPSVKVGAGKRAAWRTLLPAVAVLTLISSCAANPPPPIESTDSPKTTPVKPAETTVVVALDTLGTAFNPHLRSDQSPATSAIASLVLPSPFRPVLDPARPGATAWVPDSSLLISAEVTAQEPFTITYKLRNEASWSDGAPIAAEDFRYLWQEMISEPGVVDPAGYRLISDVNSSAGGKTVTVVMSQPYPGWHELFSDLLPSHLLKDAPGGFARGMNGQVNGVRVSGGPFGIRSADPGRDEMLLERNDRFWGTPAMPGQILLRRGGTTAQLAGSLRTGDVQMALVHGGVATQAQLGAIPSVRTAIMPQSRVLQLVLNGRKGELSDPRVRSGVLALLDPALLATVGAQTGNWVEPARAQVLAPSDPGYAPTAPPRPSAEEAFALLAAAGYGRAPEPPPATSPTSPAPQPRTVGKDGKPLVVRIGAVDRDATALAVANTAADQLRSAGIDATVRSVAADELYGKELIEGTVDAIVGWEVAGSDPATVLASRYGCPPPALPGATGPAQAIAEAAQRAPSNLAGVCDPALQPAIDEALRGGDVARVLAEAEPKLWAMATVLPIVQDNAVAASGPRVDGASLSGAIQVGVFGDASMWRRIP

>CORE_REP|Org24_Gene1345#

MCGLLGYLTVDTSGAPEGTTAEAIAAQLHEALVCQRHRGPDERGTWHDEHMVFGFNRLSIIDIEHSHQPLRWGPPENRQRYAMTFNGEIYNYLELREQLTAEHGAEFGADPMFATEGDTETIAAAFHYWGPEAAARLRGMFAFAIWDTETRKLFIARDPFGIKPLFLATGPGGTAFSSEKKSLLDLLPQLGLSDALDPRALEHYTVLQYVPEPETLHRDVRRLESGCYAWVEPGQAPKITRYFDPRFRVVPFAKPGEVTAQPPTTRPRPAAQRPNTAEYRYREIAEALEDSVAKHMRADVTVGAFLSGGIDSTAIAALAIRHNPNLLTFTSAFEREGYSEADVAAETAAAIGAKHYIRTVSPEEFAASIPEIVWYLDEPVADPALVPLYFVAKEARKHVKVVLSGEGSDELFGGYTIYREPLSLKPFEYLPKPLRRLAGRLSERIPDGTRGKSLLHRGSLTLEDRYYGNARSFNDAQLRSVLRDFRPEWTHRDVTDPIWAMQGRDWDPVARMQHLDLFTWLRGDILVKADKMTMANSLELRVPFLDPEVFAVAEKIPVDQKITKDTTKYALRRALEDIVPPHVLHRAKLGFPVPLRHWLRGPELYDWARQQIIDSATDHLLDKTAVLGMLDAHRAGTSDHSRRLWTLLVFMIWHGIFVEQRIKPEIQEPTYPVSL

>CORE_REP|Org144_Gene6421#

MVSHQNDVGTGHGAATGSAQSDTGAAKLEKVVIRFAGDSGDGMQLTGDRFTHEAAAFGNDLATQPNFPAEIRAPQGTLPGVSSFQIQIADYDILTAGDQPDVLVAMNPAALKANLADLPRGATLILNTDEFTKRTLAKVGYRADPLDDDTLSDFVVHRVPMTSLTMGATESTGVGKKDGQRAKNMFALGLLSWMYGRPIGGTEQFMREKFAARPEIAEANVLAFRAGWNYGETTESFATTYEIAPAKLPPGTYRQITGNTALAYGLVAAGQLAGLPVFLGTYPITPASDILHELSKHKNFGVTTFQAEDEIAGIGAALGASLGGSLGVTSTSGPGLALKSETIGLAVMTELPLLIIDVQRGGPSTGLPTKTEQADLLQALYGRNGESPVAVLAPRSPADCFATAVEAARIALTYRTPVLLLSDGSIANGSEPWSIPNVTELAPIDPAFEPAGAETDPFLPYARDPETLARPLAVPGTKGRAHRIGGLEKADGSGNISYDPANHELMVRLRQAKIDGIGVPDLEVDDPDGRAELLLIGWGSSYGPIGEACRRARRRGVPVAQAHLRHLNPLPANLGAVLRRYRTVVAPEMNGGQLALLLRGKYLVDVRPWTKVAGTAFSAQELVGVIDAALDGSLEEMEHDKAFAARARATYTTQPDSSANRAQPGDVRPTGGNE

>CORE_REP|Org113_Gene5495#

MPEVERHKYDVVVIGAGGAGLRAVIEAREHGLSVAVVCKSLFGKAHTVMAEGGCAASMGNANEKDNWQVHFRDTMRGGKFLNNWRMAELHAQEAPDRVWELETYGALFDRTPDGRISQRNFGGHTYPRLAHVGDRTGLELIRTMQQKIVSLQQEDYAESGDYEARIKVFAECTITDLLKDTSGIGQGPEEAAGVTTEGPRSRSNDTGAISGAFGYWRESGRFVLFESPAVVLATGGVGKSYKTTSNSWEYTGDGHALALRAGASLINMEFLQFHPTGMVWPPSVKGILVTEGVRGDGGVLKNTEGKRFMFDYIPAVFKGQYAETEEEADQWLRDNDSARRTPDLLPRDEVARAINEEVKAGRGTEHGGVYLDIASRLPREEILKRLPSMHHQFKELADVDITSEPMEVGPTCHYVMGGIEVDPDTGAATVPGLFAAGECSGGMHGSNRLGGNSLSDLLVFGRRAGLGAATYVEQLEKRPAISNSDIDAAAKLALSPFDPPASGTGENPYTLHTDLQQTMNDLVGIIRKEHELEQANGHLQELRERYGNVTVEGHRQFNPGWHLAIDLRNMLLVSECVAQAALLRTESRGGHTRDDHPQMDANWRNRLLVCRVDPADADRTVPSVVVTSEDQKPMRSDLLALFELSELEKYYTPAEVAAHPAAAESSAKGDE

>CORE_REP|Org5_Gene139#

MSLSISPDEMLVAGDEHEEGAAAQSPNRARDRDAARRFSLLSRSVFVGGVIVTLLLFGIGAWQRRWIADDGLIVLRTVRNLMAGNGPVFNAGERVETNTSAAWTYVIWFFGWISDARLEYVSLVVALTLSLLAIVFAMVGSARLWRPVTGAAPTLLLPAGALVYIAVPPARDYATSGLENCLVIFWLGVLWWLLLRWSQDERPRLLNLLLAGFWAGLCWVIRPEMTVIGGLALVVLFFSRMPRTRLRPLFTRALLVLVGGLVPVGYQIWRMGYYGLPYPNTAVAKEAGGAKWQQGLKYLWDLVGPYYLWIPLLVLIVVAVALLVRARRGRAVADAGKAPATGRLTRLQRWLRSPAAVVTVLVGGGLLLVIFNIRVGGDFMHGRMLLPQLFCLMLPVSVLPVRLPVASAGSDRPGWLRWSFALPLIAWAGTVGWALFAANTTANTAGGQISASGIVDERIYYVLNSGHDHPVLAEDYLDYPRMRAMVQDIAANPNGGLLINSPSYMMWYVAPPPLPIPPAGYGHTVYFLNLGMTSMNVPLSVRVIDQEGLAYPLAAHTDRLVDGRIGHDKNLYPDWVVVDTGMVDQHPWMPWFLDEKWVIQARTALTCPATQDLLASYRAPLTLDRFKHNLIQSLHFAKYRIDRVPKYEIQRCHLVDPTTPPPVPN

>CORE_REP|Org24_Gene4315#

MRGGPNGRPVRTDPVVPQRLPQRRNVLSLGSSRRQRGANRQRAVATSRRREPQGRTASLRGRRGGVRGLGFGWVSCSASGLWNRLIPARECPRRTVGSRPGPLFRSRGGCGPSLGNGPESAGGPRNARVVAVSERARSRVLAVWCPDWPAVAAAAVAGVPVTRPVGVFSANRVVACTAVARAEGVRRGTRRREAQARCPELLVVQDDPDRDARAFEPVVAAVDATVPGVEVLRPGLLVLAARGAARYFGSEEAAAERLVDAVAAVGVECQIGIADELSTAVFAARRAILVPPGGGAAFLAPLPIGELAVEPALSAPERADLVDLLHRLGLRRIGDFAALTPAEVSSRFGVDAIAAHRCALARPERRPGTRPPAADLTVEYRCDPPIDRVDAAAFAGRMLAAQLHELLAAAAVACTRLSIQAETAAGEHLSRTWRCAEPLTPDSTADRIRWQLDGWLTRRTRGGGRADPPAPTAPITLLRLEPVEVVSAGALQLGLWGGVGEEEARARRALARVQGLLGGEAVRVGVLSGGRGPEERITMVTLGEELVPAADPAQPWPGRLPEPAPAVILAHRPRVRLEAADGTPVWVTDRGLFTTDPTHLHWGSRSWRLLGWAGPWPLDDRWWTHRSPQGYVARAQVLLDGDTRALLLLAYDHAWHVEGLYE

>CORE_REP|Org112_Gene6280#

MSRGRDSGRDRAENGRGRGRFGGGRGNSGGGRASSGKRAASARSAAGPARARKPSRPRPAPGLDASTRFRFGVGRIVMLVALLVAALQLLWIQSVSAPRLSAEAASQRTVHQIDAATRGPILDRNGKSLAFTVNAKALTFQPVRVRKDLQEAHDENSAKPEPDQRMQAIAKYIHDKLGTAAPEQDLLKKLRSDEPFVYLVRNVDPRVAADISLKFPEVGTERQDLREYPGGSLAANVIGATGWDGHGQIGLESALDAILAGTDGSHTYDRGSDGAVIPGSWRDRQPAVNGYGVELTLDSDLQYYVQQQTQQAKELSGAQAASAVVLDARTGQVLAMANDSTFNPALGPQHWSSSSLGNPSVQEVYEPGSVNKIVTAAAAIEYGLTTPDEVLQVPGNIFMGGVTVNDAWQHGVMPFTTTGIFGKSSNVGTLMLAQRIGEDRYYDMLQKFGLGQRTGVGLPGESAGVVPSREQWSGSTFANLPIGQGLSMTTLQMTAMYQAIANDGVRVPPRIVKSKIDPDGNRTEEEPPEGVRVVSPETAATLREMFQAVVQRDPMGVQMGTGVPAAVEGYQVAGKTGTAQQIDPGCRCYSTSSYWITFAGMAPADNPRYVIGLMLDAPVRSSDGSGGQSAAPLFHAIASWALQRDRVPPSPPAKPLILQAS

>CORE_REP|Org101_Gene1761#

MIDILTPAANGIQGRVCPVRHDNDFEDFMEQSATQAVTETKDRATAVDAPASLRSDHRAPDRLVLQRGIFTGPSAKVSDELYAVVKGRAHRERQALRLEKGAAAHTNTYFGRFAASYWQRWTTVTEVRVTMVLDVVKKAKLRLVASDIAGHRRIIDTAQVTASGPVTLSATLDQYVDGGAIWLEFDAVGGDLGITEVSWTSAAPDHIRPVAIAICTFNRAEDCAHTVAALASDAVVLGAIDAVYVVDQGTDLVQNRPLYQEVAPTFGDKLRYIRQPNLGGAGGFTRGLYEVSAANEHADVILMDDDILCEPETVLRLNAFANMTVEPTLVGAQMLFLLNPDYLNVGAEEVHLQDLRHGQKVPKALRNTSMLKRNQERRVDAGYNAWWTCLIPAEVVAEIGLPVPIFFQWDDVEYGIRARESGFVTVTLPNAAVWHADFYWKDYDDWARYFSTRNSLIVGALHTDLDGKAITRKLFRELSEQLVAMQYGLVHTTLQGIEDFLQGPKVLQDGGIAALAAARTSRADYAETKKHPASTPPVRSGDIQLRRATGEPSRPLLVLIKRAINQWFGRTQHGVIGVTREDAYWWHVSLFDHVVVTDASQSGVRVRQRDKARARQLLRRTFHVLRRLRRELPTLQQQYRAAVPDLTSRANWERLYGITPE

>CORE_REP|Org8_Gene457#

MSRTRGTWTSVVAAILLVAGMATACSSDDTDEAADVCATTPNGTLVAASPTGPTGSKDISTNPELSTGYRSGMVAARTKTFAVATANTLASKAACEVLRDGGTAADALITAQTMLGLVEPQSSGIGGGAFLMYYDAASKSVEAYDGREVAPAAATENYLRWVSDTDRTEPKPNTRASGRSIGVPGVLRMLEMVHREHGKTGWRELFDPAIGLADRGFSISPRLAAQVAEQAKNLALDEAAKAYFLNPDGTPKPADTLLTNPAMAKTLGAIASEGAQAFYTGAIAQDIVAAATSTSGGRTPSLITTADLAGYQAKKRTALCTDYRNHQICGMPNPSSGGSTVAATLGILENFDLAALPPDNLGAGSDTARNGGKPKAEAVHLIAEAERLAYADRNKYVADTDFVPLPGNSLQTLLNKDYLKQRSALIDRNRSMGTAQPGDFGPVPLGVGPQPPEHGTSHISVVDQYGNAAAMTTTVESEFGSFHLVDGFVLNNQLTDFSADPLGTDGAPVANRLQPNKRPRSSMSPTLVFDKAPDGARGNLTHVAGSPGGSVIIQFVVKTLVGMLDWGLDPQQAVSALSFGAGNSPATGVGGEHPSINTADNGDHDALVLRLRELGHQVSVAPQSSGLSALTRDGTAWVGGADPRREGAVLGDNR

>CORE_REP|Org15_Gene5535#

MSPCCVCLGGEGMTDETFDDYLDETGNIAIPEGRTLVDYVEKHTRNDANDLAYRYIDYSRERDGEYQDLTWKEFGVRLRAVAARLQQVTKPGDRVAILAPQGLDYVISFFAAIYAGTIAVPLFDPDEPGHTDRLHAVLGDCTPSAILTASSSAAGVRQFFRPLPAAQRPRIIAVDAVPDTLGESWVRPDLAVDDIAYLQYTSGSTRTPAGVEITHRAVGTNLLQMVHAINLDWNSRGVTWLPLYHDMGLLCVILPAIGGKYITIMSPSAFVRRPGRWISELAAVSDGAGTFAAAPNFAFEHAAARGLPKNGETLDLSNVIGLINGSEPVTTSSMKKFNEAFAPYGLPKTAIKPCYGMAEATLFVSATRAEDEAKVIYVDRNELNAGRVVKVDHSAPNAIAQVSCGYVALSQWAAIVDSESIDSPEGAQELPEGRVGEIWLHGNNIGIGYWGREEETRQTFKNLLTNRQAEGSHAAGAPDDAIWLRTGDYGVYVDGELYITGRVKDLVIVDGRNHYPQDLEFSAQEASKMLRPGFIAAFSVPANQLPAEVFAADSHAGLKYDADDASEQLVIVAERGPGAHKADSQPIADAVRGALSQRHGVTVRDVLLVPAGSIPRTSSGKLARRACRAAYLEGTLRGGYQQQAFPDAPDEE

>CORE_REP|Org140_Gene4746#

MCCPNRTCRRADAGGAAVTSIQIAQRGTGLLRAFNEAGVLSAADVHVAVRLGRLGREESEAALFAAALAVRAVRSGSVCLELARMREIGIDADETWDTTVDPASLPWPEFDDVLAALRVSPLVIGGAAGPLRPLRLVEDRRAGGPLLYLDRYYQQEQTIRRVLTERSDRHPVVVPAVVRRELDRLFATPATEAGSTAPDRQRLAAALAATHWTTVVAGGPGTGKTHTIARIIALLDAHQRANPKAPALRVALAAPTGKAAARLQEAVRDQAADLGLPELSASTLHRLLGWQRGRGTRFRYHEFNRLPYDVIVVDETSMVSLTMMSRLMAALRPDTRLVLVGDPDQLASVDAGAVLADLVAGPVVGAPNPVLDQIIGRAAEPSADPEALTELEQTRLRGGIVRLTRGRRFGGRIADLAVAVRAGDSETALGLLREGGDALSLCEPEDVAAVRADVITAARRVTEAALAGDAAAALTALESHRLLCAHRQGPFGVERWDRMAGEWAAAAGAGPESGQNTWYPGQPLLVTANDHEARIYNGDTGVVIRQPDGSLRVALQRGSEPYLVHPTQFPSVVTVFAMTIHRSQGSQYDAVTIVLPEPESTLLTRELLYTAITRARGHVRIIGTDAAIRAATARRVLRASGLSHRVE

>CORE_REP|Org163_Gene4912#

MIWIRCAIRRELVQVGVLSRVDTPDDLRRLTVPQVRELAEEIREFLVRKVAATGGHLGPNLGVVELTIALHRIFDSPADPLIFDTGHQAYVHKILTGRKEQFDSLRKQGGLSGYPSRAESAHDWVESSHASAALSYADGLAKAFALSGQDRHVVAVVGDGALTGGMCWEALNNIAAAPDRPVVVVVNDNGRSYAPTIGGLAERLTALRTQPAYEHALDAGKRILKSIPRVGESAYSMVHAVKAGIKDAVSPQELFSDLGLKYVGPVDGHDVVALEAALRRAKDFGGPVVVHAVTQKGRGYAPAENHVADQMHACDPIDPLTGVPVGGPKARGWTSVFSEELIAQAERRADIVAITAAMPGPTGLAAFGERFPDRMFDVGIAEQHAMASAAGLALGGMHPVVAIYSTFLNRAFDQLLMDVALLKQPVTVVLDRAGVTGSDGASHNGMWDLSVLGIIPGIRVAAPRDAATLREELAEALAVNDGPTALRFPKGSVAEDISAVERLDGIDVLRTAEPEGGSVQAVHGDVLLVAVGSFAAAALEAANLLDSEGISVTVIDPRWVLPVSDTLLKLAENYRLVVTLEDGGLHGGIGSTVSARLRNSGLDVPTRDLGVPQQFLDHASRGELHTELGLTGPDIARRISGWLAAR

>CORE_REP|Org105_Gene4310#

MTTPKNLSSRYELGEIIGFGGMSEVHKARDLRLSRDVAIKVLRADLARDPTFYLRFKREAQNAAALNHPAIVAVYDTGEAEVDGGPLPYIVMEYVDGETLRDIVRGKGPLPPRRAMEIIADVCAALDFSHKAGIVHRDMKPANIMINRSGAVKVMDFGIARAIADAANPMTQTAAVIGTAQYLSPEQARGESVDARSDVYSVGCVLFEILTGEPPFTGDSPVAVAYQHVREDPRLPSLVHEGVPRELDSVVLKAMSKNPANRYQTAAEMRADLIRVLGGQKPSAPMVMTDEDRTTILGSEEPAPRSYHTVDNHDRSAYRDNDDTGEPEPVDPPSQRRTAYLTLGAVAAVIVAIALFWVLIGPGSKPDQVAVPDLSNSSVQQAEQKLEDLGFHVAIQEKPDARVAPGNVIATQPLGGSRVDEGSTITLQVSTGPAQVQVPRLTGLTRQEAEQKLNAIGLRLDPQVDKEASSTAELDKVIGQNPAEGASVEVDRAVKVTIGSGPEQVRVPNVVGQDIEVAEPNLVEGAQFKVVVQEVASSRPKGEVIATSPAGGSTAEKGSTVTVQVSLGAEFTMPSLVGLNASHAVDRLRQAGWAGSTTQIVQNTQVTLDSANVGKVLNQQPAAGSSVGRNSTIVIYTGVLPLGPP

>CORE_REP|Org136_Gene4665#

MTSPQEKAAAARKAAEEAARIAAEAAAAAEAAEAEAATAESGGAPAASGATAAESGAGSSGSGGAAAGSGGATDAVGGAAAGSGGAAESARGTTASSDVAASAGGAAGASGGPVGPATESGGADSGSDDDAPEGGGSSAAQEIAAGYAVEGAALELGTVVVDGTVDRTARVRIPLRTMNRHGLVAGATGTGKTKTLQGIAEQLSRAGVPVVLADVKGDLSGLSRPGEQNEKLAQRAVETAATDWAPTGFPTEFVSLGTGGLGVPIRATITSFGPILLSKVLGLNETQESTLGLIFHWADKQGLGLLDLKDLRAVIQHLTSPEGKADLQGIGGVSASTAGVILRSLVNLEADGGDTFFGEPELDPADLLRTEGGQGVITLFELGAQAARPAMFSTFLMWVLADLFQTLPEVGDVDKPELVFIFDEAHLLFADASKAFLDQVEQTVKLIRSKGVGVFFCTQLPTDIPNAVLSQLGARIQHALRAFTPDDQKALSKTVRTYPKTDTYDLEQALTSLGIGEAIVTVLSERGAPTPVAWTRIQPPRSLMDTIGADAIKSRALASALHGKYGQTVDRESAYEMLAANVAAAEPEAEPQPVPGRSPSAEEDSAAERIMKNPAVKSFLRSAATVAGREITRTLFGTRKRR

>CORE_REP|Org105_Gene5106#

MYRTGHADAIYVAAGPNSSVSAAVMKISGFHAMTGNRQAQRAFDAGILSLGLSIDGQESTRDLEYAKLAFQRATEWDPTMCDAWLGRAAAGEVTDEVIRNLHRTSTSTLYREQRRLGLAPRALAGRFVSGLYIDYPLASYTEIWLAYAANLIGSKQYDEAERVLDELAEYRAGMLSDPDREIDDRISAYIRGVLHFNTQRWPDVMSVLAGSAEWEDPYLATGAHVMVGSACAQLGLFGEAIRRMEQAENGPIPAARTTAMFCRGLCLRETGSEDEAQALFEQVYSQAPDFTANTEAMRDKSYRITITTKESIDARTDRWDPASAPSVEQLQTADAEDRAKKILTEARAELDRQIGLTAVKTQVAKLQATAQLAKIRAEKGMASVPRGNHLAFTGPPGTGKTTIARVVAKIYCGVGLLKTDKVVEAKRMDFVGQHLGSTAIKTDKLIDTAMDGVLFIDEAYTLIQTGLSGGDAFGREAVDTLLARMENDRDRLVVIIAGYDGEIDRLLAANDGLASRFAKRLQFPSYTPPELGQIGKLIASSRDSELSEDAVRLLEQACERLYNSERTDQSGQPRRGIDLAGNGRFVRNVIEAAEEEREFRLANDESLDLTAVDESVLMRIEAPDMEAALAGVLSSLGVS

>CORE_REP|Org12_Gene4561#

MSPAVLVSAPADLSAFGHDPWWLVVVKSVGIFIFLLLIPLLAVVIERKVVAWMQMRVGPNRVGPRGSLQSIADGVKMLLKEDIVPAMVDKPIYILAPIVALIPAVMAFAVIPLGPEVSIFGTRTPLQLTDMPVGVLYILAMTSIGVYGIVLAGWSSGSTYPLLGGLRSTAQVISYEIAMAACFGAVFLLAGTMSTSGIVERQWGTWNVWLLLPSFVIYAVAMVGETNRAPFDLPEAEGELVGGFHTEYSSLKFAMFMMAEYINMGTVSALATTLFFGGWHAPFPLNLWDGANSGWWPLLWFTLKLWTFLFVFIWLRGTLPRLRYDQFMNLGWKLLIPVSLLWVMIVATLKVVQDNGHDVQTTGLVTAGVIISVGLLAMMLRAGRAGDNPTPETAGPQQFSDFPVPPMPETAPTATKAGLLDPIGGFWVTFVTMFKKKNTEFYPEEKVPTAPRYHGRHQLNRHPDGLEKCIGCELCAWACPADAIYVEGADNTDSERYSPGERYGRVYQINYLRCIGCGLCIEACPTRALTMTNEYELADDNRADLIYEKDRLLAPLGDGMIPPPHAAYPGATEEDYYLGAVPAAPGTDRDTGLGSTGNAVARPSGSRGSDLVGPDPADPAVDAAGRQPAATGAQGGAQ

>CORE_REP|Org15_Gene5181#

MGTGNLLWLLPALPAAGALILLLAGHLSDRWGHWLGCATAVASFGVAVWAFAEMLGRAGADRAVSHNFFSWIPVAGLQAEFSLQLDQLTMCFVLLITGVGSLIHIYSVGYMSHDPARRRFFAYLNLFLAAMLILVMADNYLVLYLGWEGVGLASYLLIGFWHEKPSAAAAAKKAFVVNRVGDMGLAIALFLMFATFGSVDFGHVFAGVPQASDGTLTALGLLLLLGACGKSAQVPLQSWLGDAMEGPTPVSALIHAATMVTAGVYLIARSNAIFDAAPAARAGVLVVGAVTLLFGAVIGCAKDDIKKALAASTMSQIGYMVLAAGLGPAGYAVAIMHLLTHGFFKAGLFLGAGSVMHAMNDETDMRRYGGLRRYLPITFVTFGLGYLAIIGVPPFAGFFSKDRIIEAAFGYGGANGITLGAAALLGAGITAFYMTRVMLLTFFGEKRWTASKSGMEPHPHEAPAVMTGPMIVLAIGSVFSGGVFVFGSSLQNWLAPVVGTEHAESAVPAWAVTVAALVVVAIGVAVAYRQYAWRPVPLTAPQDVTPLTAAARRDLYGDAFNEAALMRPGTHLTRSLVFLDNRGIDGIVNTTAAVIGGLSARIRRVQTGFVRSYALSMFTGAALVVAALLAVRLL

>CORE_REP|Org216_Gene3071#

MPELRSRTVTHGRNMAGARALMRASGVPAADIGAKPVVAVANSFTEFVPGHTHLQPVGRIVGDAIRRAGGIPREFNTIAVDDGIAMGHQGMLYSLPSRDLIADSIEYMVQAHCADALVCISNCDKITPGMLLAAMRLDIPTVFVSGGPMEGGRATLADGTVRRLDLITAMSEAVNDDTSDADLATIEENACPTCGSCAGMFTANSMNCLVEALGLALPGNGTTLATHTARRDLYEAAGETIMAITRRYYDRDDASVLPRAIASRAAFDNAMALDLAMGGSTNTVLHLLAAAHEAGLDYTLADIEKRSRAVPCLCKVAPNGSHLMEDVHRAGGIPAILGELRRGGHLHTTVRAVHSESLDGWLAEWDVRGPNPAQAAVDLFHAAPGGVRSATAFSQSARWASLDLDAESGCIRDVAHAYSEDGGLAVLRGNLAVDGAVVKSAGVPADLHVFTGEAVVAESQEEAVTAVLSGRVRPGTVLVIRYEGPRGGPGMQEMLYPTAYLKGRGLAGSVAVVTDGRFSGGSSGLSIGHVVSPEAAAGGTIAAVADGDRITIDIPSRTLRLEVDDAEIARRLAHRRRTGYRPRSRHRPLSTALRAYALLAQSADKGGVRRLPPDELGGPEAAFDTQTRAG

>CORE_REP|Org207_Gene4008#

MAKAVGIDLGTTNSVIATVEGGQPTVIPNSEGSRTTPSVVAFTDQGERLVGQLARRQAILNPKGTVASAKRFIGRRFEEVATERDTVSYEVVSGSNGAARFDVRGKQVAPEEISAAVLRKLVDDASKYLGEKVTEAVITVPAYFNDAQRQATKDAGKIAGLNVLRIINEPTAAALAYGLDKKKNETVLVFDLGGGTFDVSLLDVGDGVVEVRATAGDTHLGGDDFDRRVVDWLAEEFRKDYGIDLRTDPQALQRLYEAAEKAKVELSSVSQTTINLPFITADAGGPKHLNTTLMRSKFDQLTGDLVERCMGPVQQAMADAKVTANDIDEVILVGGSTRIPAVQQLVRRLTGGKDPNMTVNPDEVVALGAALQSAVIKGEMSDVLLLDVTPLSLGVETLGGVMTKVIERNTTIPARRSEVFSTAEDNQNAVDVVVLQGERERAADNRVLGRFRLENIRPAPRGVPQVEVTFDIDANGILNVSAKDKDTGAEQTITISESSNLDQSEVERMVADAERHRGEDAKIRERVDARNTLDTIAYQVEKRLSELGEAAPAHDKARAEMLIGDARQAIKDDTVGIERLRELTSELQQLFYGLDTAAGGTAGNQAGGGARRDGGGGDDVIDAEFTSE

>CORE_REP|Org65_Gene1884#

MCGIVGYVGYRDALGVVVDALRRMEYRGYDSAGVAILDGAGAIAVERKAGRLANLEAELGEAGAGAFAGSTGMGHTRWATHGAPTDRNAHPHRDEAGAVAVVHNGIIENFAPLRRELEDAGVELRSDTDTEVAVHLVSRAYAEGPTAGDFEASALAVLRRLEGAFTLVFTHADHPDKIIAARRSTPLVVGVGKGEMFIASDVTAFIEHTREAVELGQDQAVVITADSYRVTDFAGNDAGSRTRPFTIDWDLAAAEKGGHDYFMLKEIEEQPAAVAETLMGHFDTGQGGSGRIVLDEQRLADQELRDVDKVFVVACGSAYHSGLLAKYAIEHWTRLPVEVELASEFRYRDPVLDRSTLVVAISQSGETADTLEAVRHAKEQKARVLAICNTNGAQIPRESDAVLYTRAGPEIGVASTKAFLAQVTANYLVGLALAQARGTKYPDEVAREFAELEAMPKLVARVLETAPQVRAIARELAKVPTVLFLGRHVGYPVALEGALKLKELAYMHAEGFAAGELKHGPIALIEDGLPVIVVMPSPKGRAVLHSKLLSNIREIQARGARTIVIAEEGDDTVRPFADDLIEIPSAPTLFQPLLSTVPLQIFAAEVAQARGYDVDKPRNLAKSVTVE

>CORE_REP|Org9_Gene416#

MVDTDHMPAPAMAPAAAPTGHARVLAWVAEVADLTAPEDVVWCDGSRQEWDRLTARLVDKGTFVALSGKPNSFWCVSDPEDVARVEDRTFICSRDKRDAGPTNNWVDPVDMRTVMTEHYRGAMAGRTMYVIAFCMGPLDAEDPKYGVQITDSEYVAVSMQIMTRSGAPVWNQLGQDAEFVQCLHSVGAPLSPGQADVAWPCDHTKYIAHFPEDRTIWSYGSGYGGNALLGKKCFALRIASVLARDEGWLAEHMLILKLTSPQGRTHYVAAAFPSSCGKTNLAMLEPALEGWKAETVGDDIAWLRLGPDGRLYAVNPEAGFFGVAPGTGAKTNPNAIATIEQGNSIFTNTALTDDGDVWWEGLTDTPPQHLTDWRGNDWTPESATGPAAHPNSRYCTPIEQCPSVAPEWDDPAGVPLSAIFFGGRRATTIPLIAESFDWTHGVFTASVLSSETTAAAAGQVGVVRRDPMAMLPFLGYHVGDYFAHWLRLGEAADPGKLPKIFQVNWFRRDADGRFLWPGFGDNVRVLKWALERIEGTAAADATAIGYVPVPSSLDLSGFSEAGKRSARAALEVHHAEWADEVASIEDWYASIGADTLPGPLGDQLAALKNRLAHTPARPTGTTAGAS

>CORE_REP|Org5_Gene6313#

MNNSSVDSVPSGGDDRPEGADERNHEGPADAATGYRDGSAAPAASESGAAEVSEPTFADLGIDDRLLAAIADVGYESPSPIQAATIPPLLSGADVVGLAQTGTGKTAAFAIPILMGLDKRPKPPQALVLAPTRELAIQVAEAFGRYSAHLPGIHVLPIYGGQNYAVQLQGLRRGAQVVVGTPGRVIDHLERGTLDLTQLRYLVLDEADEMLKMGFQEDVERILRDTPAEKQVALFSATMPSVIRKISKQYLKDPVEITVKSKTSTNTNITQRWVHVSYQRKLDALTRILEVEPFEAMIIFVRTKQATEELAEKLRARGYSAAAINGDIAQNQRERTIGQLKSGTLDILVATDVAARGLDVDRISHVVNYDIPHDTESYVHRIGRTGRAGRTGEALLFVAPRERRLLDAIERATRQPLTEMQLPSVDDVNAQRVVKFHDAITENLASPNLALFRKLIEDYEAEHNIPLADIAAALAIGGHDGENFFMEPEAEPIRPPRRERAPREERERRSEGPQRHRATGADMATYRIAVGKRHRVVPGAIVGAIANEGGLRRSDFGHISIRPDHSLVELPADLPSETLDALRRTRISGVLIQLQLDQGPPSHRPIGRGPRREGGRKHDRRKPRS

>CORE_REP|Org136_Gene2886#

MTTTDIGYAHPSGTTVYTVPQAFQQTLTLRPDQIALRTVGGTQEITWREYGERVRALAAGLAGLGVGHGDTVGIMLTNRPEFNLIDTAALHLGATPFSIYNTSSAEQITHLFTNAANKVVVTEQKFLDVIKASGVPVEHLIVVDGPVTGALTFDDVEAAAAGDFDFDAAWQAVQPDDLATLIYTSGTTGPSKGVEITHRNVLAQVLALVSGPLSVGIDDRIVSYLPAAHVADRISAHAMNLLTGIQLTTVPDPREVAAALPDARPTVFFGVPRVWQKIKAGIEAKLATETGVKKSLAEWAIATGIAAARADLAGTGRPLTLRIQHPIADALVLSKLRAALGLDQLKVASSGAAPIPAETLEYFLGLGFTVSEVWGMSETTGVGTYTELDKPRPGTVGRPVDGLELRLDADGEVLVRGPIVTRGYRNMPDKTAEAFDDDGWLRTGDVGTLDADGYLRIVDRKKELIINEAGKNIAPSNIENSVKAASSLVGQVVAIGDAKPYIAALIVLDPDIAAVRAKELDATDTDIAGLATRREILDEVLAAVQAGNKKLSRVEQIKRFTVLGTVWEPGGDELTPKMSLKRQPIAAKYAEQIASLYADPAPEGVIRGRRSIGRISGGYGVAAW

>CORE_REP|Org13_Gene1651#

MHRQVRSSDGRGTHHHARRGCRPRGDSPLSAAPVEIQPVPQAERPFPARMGPKGAAMWKIVTTTDPKLLGVMYIFTAISFFLIGGLMALLMRAELARPGLQFLSNEQYNQLFTMHGTLMLLFYATPIVFGFANCVLPLQIGAPDVAFPRLNALSYWLYLFGATVATAGFITPGGAADFGWTAYVPLSLAVHSPGVGADLWVMGVAVSGVGTILGAVNMITTVVCLRAPGMTLFRMPIFTWNILVTSILVLEAFPILTAALMGLEVDRHLGGHIYDPATGGPILYQHLFWFFGHPEVYIVAIPFFGIITEILPVFSRKPVFGYTALVYATIAIAALSSAVWAHHMFATGAVLLPFFSLMSFFIAVPTGVKFFNWIGTMWKGHLTFETPMLWSLGFVTTFLFGGLSGVLLASPPLDFHITDTYFIVAHFHYVLFGTIVFATFGGIYFWFPKFTGRFLDERLGRLHFWTTFLGFHTTFLVQHWLGAEGMPRRYADYLPADGFTTLNTISTIGSFILGFSMITFVWNVFKSYRYGQVVTADDPWGYGNSLEWATSSPPPRHNFYELPRIRSERPAFELHYPHMIERMRAESHTGWGSAGRSHAAALTEAPAAGPQESNRADESDAE

>CORE_REP|Org210_Gene6389#

MPAPAAGRSRGDGPVGHYKSNVRDLEFNLFEVYGLETVLDSGAFAELDGETARTMLAEAARLAEGPVAESYAETDRHPPIFDSDSHSVRIPEPFKRSVRAWQDAQWWRVAKSEAIGGVPAPSMLGWAINELVLGAQPAAYMYLSGPMMADVLAGIGTEQQRRWAAQAVERNWGATMVLTEPDAGSDVGAGRTTATEQQDGSWHLEGVKRFITSADSDDLFDNIMHLVLARPAGAGPGTKGLSLFIVPKFHFDHDTCEPGERNGVFVTNVEHKMGLKASATCELTFGGHGIPAVGYLVGGVHNGIAQMFEVIEEARMMVGTKAIATLSTGYLNALDYAKTRVQGSDPARAADKAAPKVTIIHHPDVRRSLMMQKAYAEGLRSIYLYTAGHQDPVVARHISGADDDLAARVNDLLLPIVKGVGSERAYQYLTESLQTFGGSGYLQDYPIEQYIRDAKIDSVYEGTTAIQAQDFFFRKIARDNGIALAHVLGQVRATAESDADRGRLKNEKVLLSHALEDVQEIIGRLTTHLLEAAVEPREVYKIGLNAVRLLLAVGDLLVAWRLIVGAETALTAIEAGQNTAFYAGKVAVASFFANSALPHLTAELAVVSATDATVMDLEEAGF

>CORE_REP|Org4_Gene3693#

MRTRSVPWLRAVSVPPPRSPEGSMSNLINLEQVSKSFGITPLLDNVSLGVHAGERIGVVGLNGGGKTTLLEVLTGLEPPDSGRVSRVGGLRLAVVTQRGVLPAGATVGSVVLAGLADDLNGVAGGPDEVAEHEWAANPRIRSVLEGIGIAGLGLQTSIDNLSGGERRRVALAAALVRDLDLLVLDEPTNHLDVEGVQWLAAHLLERRSALVVVTHDRWFLDTVATDTWEVVGGKVESYEGGYGDWIFARAERARQADASEARRSNLARKELAWLRRGAKARTSKPRYRVEAAEALIADVPPPRDSVSLAAFARKRLGRVVIELEDTTLTTPDGRELVRDLTWRLAPGERVGLVGVNGSGKTTLLRTLAGDTEPAAGKRIQGQTVQIGWLRQELDDLPTDMRVLEAVQQVAQRIMLGDKEISAGQLAERLGFSPARQRTPVGDLSGGERRRLQLTRILMAEPNVLLLDEPTNDLDIDTLQQLEDLLDNWAGTLVVISHDRYLIERICDTTWALFGDGKLTNLPGGIDEYLKKRAAQGQSATRAADKPTGPVTDAAAQRAARKELSRLERAIEKFDEREQRLHTALADAAIDPDKLVTLNAELKQVVADKEAAEERWMELAEDV

>CORE_REP|Org157_Gene2630#

MPIASDPSPGAETVPQYAAWRKGVAGVLAKARKVDVADLPDEPEQLLTQTTYDGLAVAPLYTRRDERPEPPLPGVFPFVRGRDATRDVHRGWDVCADITETDAAAANREILAGLENGLSAVRVGVGEHGVPVAELPTALRGLLFELAPLSLAAGAALPEAAAQLYAVLDDYRVDDRAAIRIGLGAAPLTSRFAGAADVDSDRAVELAKQAVARTETVRAITVDGTVFHNAGASDAQELGAVVAAGLEYLRALTGAGVDIADALGQLEFRLAATDDQFATIAKFRAARTVWARVAHVCGAPDFGGAPQHAVTSAAMMSKRDPWVNMLRTTLAAFGAGVGGADSLTVLPFDCALPPGELGVSKSFSDRMARNTQLLLLEESHLGHVQDPGAGSWYIEDYTAKLAAKAWEFMQELEKAGGYRAALDAGLLGQRIAETKATRDADVAHRRTAVTGVNEFPNLAEKPLSEQARTPGEIARYGAAFEALRDRSDAYLEANGARPKALLVPLGTVAEHNVRVTFIANLLASGGIASVNPGPLEVSAVEAAAKEAGAPIAVLCGSDARYGAEAGAAVAALRAAGVETVLLAGAEKAVADLDAAQRPDGFLTAKIDAVAQLSGLLEKVGA

>CORE_REP|Org39_Gene3915#

MAKKKVQDPAVPGPDATLYLIYAGFALFGTLWVALQLGNQLSTPPQDLPINPIALGFGLGRGELTWPTASTVIVVLVVLAAVTYLVVKRKMKKRKSVGRLAVDDKADHMGSGSAIGPLTEAGVRDKAKQLGMRLGRDDTPGVPIGVAVADGQMVYGSYEDLHVDIWGPRQGKTTSRVIPAILSAIGPVLTTSNKRDVVDATRDVREGKGSRTFVFDPQGVADEEPTWYWDPLAWVDAHSPGCEVRAVRLAGHFADGDDSSGPQATTDSYFDQEAEDLLAGLFLAAAVDNRPIIQVWDWVNNWRDTEPVELLRRAGQHFMASGLAQQFNVDDRTRSGIFGSAKKMVRCLKLSNVHPWITPGDTRTRDGRTVNRIQFDELEFIEGNGTLYSLSLEGRGSAAPLVSALTEAVIDVATHKASRSPGGRLPIPMLAVLDEAANVVRWKDLPKQYSHFGSRGIVVMTVLQSWAQGARCWGVDGMEALWAAANIKVLGSGVDDNKFLQERSEQIGEYETISASVSESKGGKSYSRSLSSSKTFNVHALATLPRGRAIVFSSGTPPVLVRTVPWWEGEYAAEVKKSIAHHDPSPHKKTEIADLIGAPSLIKAPPGQDAGQIEEVKPL

>CORE_REP|Org102_Gene5286#

MRRNLLRLATAGSVDDGKSTLIGRLLYDSKALFSDQLSAIEKFSTARGDQAPDLSLVTDGLRAEREQGITIDVAYRYFATPRRKFVIADTPGHVQYTRNMVTGASTADLALILVDARKGVSEQTRRHAFLSSLLGVGHLVLCVNKMDLVDFSRHRFDEIREEFADFATKLEVRDLSFLPLSALHGDNVVEPSPHTPWFPGPPLLRHLEDVHIASDRNLIDARLPVQYVIRPSGETRRSYAGTIAGGVFKPGDEITVLPSGRATRVGQIWGPGGSKVEEACAGMAVSLTLDDELDIGRGDMLARPGNQPHQDRELDAMVCWFSDDTALRPGDRYAIRTAAQTAQVRVRALDYRLDVNTLHRVEDAPRLALNDIGRVTLHSTQPILFDPYRANRATGSFILIDERTDQTVAAGMITGRTPVQPQARGPVTWHRSAVERTQRLSQGGTLWLTGLSGSGKSTIAVELERQLIAAGRPAYLLDGDNLRHGINGDLGFGDDERRENIRRVAEIAALFADSGTIAIVSLISPFAAERENARKIHADKGLEFHEIFVDTPLATCEDRDPKGLYAKARAGEITRFTGIDSPYERPEHADLVVTPADGTPTAIAELIRRELGIAEHR

>CORE_REP|Org113_Gene5471#

MALREFISRHRIVSAAAAPAALGVAAIVAASWGLMATPEHRDTRLTSSVTECHDMVTISVAGRNDSPNPNSTAMLVDANGNALPAALSGDYHSTWVDPVVNAPADKVDPGSYAAVYIAYPANMNSYEDAVTTGVSNTQQVMHDIAQACPDTKFSIVGYSEGADVARRVAMGIGHDEPGTDGKYDVVDPNHVLGVVILADAGRTAGDGPFPGAQDPFGHPDGFDQNYQNGNTPISGQGAMPDNAGDFGSLNGKIASFCSEGDLTCAAPQNISLLQLAANVGRQLNVDALQNEGLTPATGQDVAVVLGQIAMNAFSYIATQPNWMASDETFLQVLLKVSDPEYKPGQNPPDKAVAQSISTNEMSPLAYLPKKVFNEIVGLIVTNQNTIPVIMSDPYQLTLGPNGTGHHFDYWRDSDSANGKPMTSAEYAAAWLTHLAQQAQAGEPVDTKAQPTEADVATAYKTVTDTQAEKKKNADEAAAAAKEKKTDKTTTTKDPKATKDPKPSKDAASTKETTTPSKDATPSKDATPSKDTAPTEDPKATEDSKPTPTNPGTPEQAPATTTPATTTPAKPQPRTEVAAPVQTTEPKKPEPTKEQPGKDEPTKDQPTTAERTPAPAK

>CORE_REP|Org180_Gene5150#

MYQSPNGRPGPENPREDGPVRSDLISRRDLEFLLYEWLRVEELTGRARFADHSRETFDGFLDLCEDLATRYFAPHNKANDAQEPRFDGERVTIIPEVKDAVQAFGKANLVGAAMDAEVGGMQLPSVVEAAGFAWFQAANVGTAGYVFLTIGNANLLAAHGSPEQVDRFVKPMVEGRFTGTMCLSEPQAGSSLADIVTRAEPQEDGTYRLFGSKMWISGGDHELTENIVHLVLAKIPGGPAGTKGISLFVVPKYLVDADGSLGARNDVTLAGLNHKMGYRGTVNTVLNFGEGRHTPGGAAGAVGYLVGEPHRGLSYMFHMMNEARLGVGLGATALGYTGYLKSLRYARERQQGRAVTAKDPATPQIPIIEHADVKRMLLAQKSYVEGALALLLYCNRLVDLARTDPASAREHTLLLEILTGVAKSWPSQWCLEANNLAIQVHGGYGYTREYDVEQHYRDNRLNPIHEGTHGIQGMDLLGRKVIQHEGASLRLLAQRIAASVAAARALGGEPAELAEALDASWQRLVTVTTAMLSAGDPAATMANSTIYLEAFGHIVLAWIWLEQAVAAAGHDGDFYAGKRAAARYFFRYELPRTGPQLDLLAALDRTTLEMRDAWF

>CORE_REP|Org10_Gene3860#

MTESDRTESTAPGCDPAELRTLFLFEQLDDEQLAWLCADGRIELIEPGPVYRQGDPATCFYVLIEGELRLTKLAGGMEIELNRTDHRGVYAGAWTAYLGEQAEPTYNSSLYVTRPSRFFVLDAEIFARMMHAWFPMAVHLLEGAFFGNRNAHQRVAERERLMALGSLSAGLTHELNNPAAAAVRATSGLRERVAGMRHKLGMLAEGRFAPEVLVTLVRLQEEAAEQVAKAPALTPLEAADREDALGDWLDEHGIADGWELAPNFVQAGFDVDWLERVHGTLEGCSETVFEGAIRWLNYTIETELLMNEIADSTARISTLVGAAKQYSQMDRAPFQVVDIHELLDSTLVMLNRKLGDGVRVVKDYDRTLPALPCFAAELNQVWTNLIDNAVYAMGGEGTLTLRTYRENDCAVVEVGDTGPGMPEEVRRRVFEPFFTTKPVGEGTGLGLDISFRIVVNKHDGDIRVESAPGDTRFVVRLPLHRDIPARNPAPTPRNRQTETMTAIEGIDPSVPPSGPGCVECEASGGWWVHLRRCAQCGHIGCCDTSPSQHATAHHRQTGHPFIQSYEPGEDWYWDFRTEEMFTEGPELAAPHSHPAAQGVPGPSGRVPADWREHIH

>CORE_REP|Org49_Gene6287#

MSRSRTLPPGHRVSDVACPVPTVVAGRVGSVRTRTRLFALFAVSTLTLTGCGLFDDDTTERMTSVATSFADALTNDNPSAAAAVTTDPTQAAATLGALYDGLGTDARFEVRGVDKDAGTFTLAATWKFGANEWSYTTTGKAADTGDGFKVTWDPAAVAPGLDAGPLSYSPVYPDPARVLDSTGGELMAQQVVTLVNLSPGVDTVAVANLLSPIAPSITATSLQSEIDAAQGNPVTAITLRESDLAPIRDALTALPNITLAPQTRLLTTDRDLASPTLSGLSELWQQQADAAAGWAVRASTPQGTERVAGRDPQPTTDIRTTLDLGLQRAAEAALTPITQPAAIVAMQPSTGNVVAVAQNSAADAEGPIALTGLYPPGSTFKTVTVSAALQAGSVTPDSIVACPGSANIEGRRIPNDNNFDLGEVPLHTAFARSCNTTMGRLAVDLPPDALTTAAAQLGLGIDYVTPGLTTVTGSVPTADTPALRVEEGIGQGKVTASPFGMALVAAALAHGSVPAPTLVAGQPGTPDRTPPVLPAGIADQVRTMMRETITDGTATQLRDIPGLRGKTGTAEFIDDTHAHGWFVGIDGDLAFAVFVYDANSSGPAVDAAGRMLRQR

>CORE_REP|Org49_Gene2014#

MRPSTLPPRTTGRRPPARNRPGRRALTGSRATPPGTARICPWRCSTARRIRLPIVAVSESARRRIIEWGRRALGLDVPPSDHTAAALHSVETDVPGLDKRETLQLARIRLLGATGAVIMAISALGVGAQPVRQNPTSGLRIIGFFARAHTSTLAMCMIGTVLVVMAWLLLGRFAIGGWGGNPRHRLSRSQMDRTLLLWIIPLSVAPPMFSNDVYSYLAQSEIAVRGLDPYEVGPADGLGLNNVLTNNVPNIWRETPAPYGPLFLWMGKGIAVVTGDNIIAGVWLHRLLVLGALALIVWALPRLARRCGVAAVSALWLGAANPLVLLHLVGGVHNDALMLGLMLAGLEICLRAIEDAYPFDQRAWAILLGGAGLIALSSTIKIVSLLALGFVGMALARRLGGGFRMVVKVGLILGVVAGVTILFVTTASGLGFGWLYTLNTASAVRSYLSLPTAIGIATGFGGVLLGLGDHTTAVLSITRPIAATLAAVVIVRMLFATWTGRLHAVGALGVSLGALVLLFPVVQPWYLLWAIVPLAAWANRPAFRVPAIALSVVVSLLVMPRGADFYVFQIVQSAIATVIVGLAFIFLTRNALPWRNQPGVSAPSQEATAYGVRS

>CORE_REP|Org46_Gene2776#

MRTLYPAIEPYESGMLAVGDGQSVYWEVSGNPDGKPVVFLHGGPGGGTAPFHRRFFDPAAYRIVLFDQRGCGRSTPHLADGASLEHNTTGHLIADIEALREHLAVERWQVFGGSWGSTLALAYAQRHPERVTELVLRGIFLLRRKEIDWYYNGAAGYVYPDEWEKFLAPVPEDERGQDLVEVYHRLLHSPDEDLARAAAIAWSTWEGATSSLLPHPDRVAETAEPRFALAFARIENHYFRHGGFLDEGQLLRDIAAITHIPAVIVQGRHDIVCPAVSAWELHRAWPGSVLHIVDDAGHAANEPGITHHLVEATDRFSRVGVSTVTTAADALIGALRDDIDRLSAAEPEVRADAEDSVHQMRVATRRLRSVLRSYGTLLAKKPAAAMNAELKWLAGLLGEARDAEVRADRFAALLAEHGEQAQPADLDAVTARLVNAERDRYRAAHDEVLAALDGKRYRELHDELARWRTAPPLRHSRAEAPATDVFGEVLRRDLDRVASLVRAEPTVDPHERVELLHDIRKSAKRLRYSCEAAEHVIGDEAAERGRRAKKLQTVLGDHRDAVESHSAIVRRAAEAAAADEDAGLYDILAAAEDAAAGRELSRYPATAAALLG

>CORE_REP|Org97_Gene4674#

MLSGHRRRAKHDTRRYGVRHTSPPTIEAKLLTGSVYQVTLAAATVPSTRSGRVEVVDVGVGIGSILQGVQRKAVEVTEAVRALNVLRERGLIDPRNLGETVLSAKEGRRLGPQATMVRHAARMWPQRPALVDEHGSMTFRELDDNSTRLARGLQSLGVEPGTVIGILARDHRGLLLAMAAAGKAGARLALMNTGFAKPQFAQVCEREHVKVVLHDSEFVGLLDALPPELPRVITWVDDGAELPTGAITIEELIAAHPPTELPLPAKPGGFVILTSGTTGLPKGAPREAVSPIASAQITDRIPFPAQGTMLIVSPIFHSTGQATWLIATALGATTVMTRRFDAERTLALIAEHEVDLLVAVPTMLHRMVELDPAVRERYDLSSLRSIVLAGSALSPELTVRATEAFGPVLYNLYGSTECAVATVAQPADLALAPGTAGRAPVTCEVALFDENGKRVHGANVKGRIFIRSGAPFGGYTDGRHKQIIDGYMSSGDVGHFDEHGLLFVDGRDDDMIVSGGENVFPQEVENLLLERADIFDAAVVGVDDVEFGKRLRAFVVPEPGATLDPDEIKAYVKGTLARYKVPRDVVFLDDLPRNATGKLLRRALVEYQIT

>CORE_REP|Org103_Gene5940#

MYSWDSSNAVSPVSSDNADSATSAAPGPFASADAAAHVRRDARPLVGRSERARNAAVRPGFGLALVMDAVTVVPTPANEPVHSYAPGSPERERLLARLSEISAETLDVPLVVGGKHRPGIGERHDIRAPHRHDLVLGTYTDTTHSEAQAAIDAALAAAPDWRSLPFDDRAAVFLRAADLLAGPWRETLAAATMLGQSKSVAQAEIDAPCELVDFWRFNVAFAREILAKQPQSSPGVWNRMEYRPLEGFVYAITPFNFTAIAGNLPTAPALMGNTVVWKPSPTQTLSAYYTMRLLEAAGLPPGVINMVTGDGVQLSEIALADPRLAGIHFTGSTATFQYLWQEVGANIGRYHGYPRLVGETGGKDFVLAHPSADPAALSTALIRGAYEYQGQKCSAASRAYIARSVWREMGEQFLATVEELRYGDVADLSNFGGALIDRRAYDKNVAAIERARSAGVTVAVGGTYDDTDGWFVRPTVLVCDDPADESFRTEYFGPILSVHVYDDGEPGAYSAILAEVESAAPYALTGAVFAQDRKAIEQACTALRFAAGNFYINDKPTGAVVGQQPFGGARASGTDDKAGSPLNLLRWVAPRTVKETFAPPTDHRYPHMRT

>CORE_REP|Org150_Gene6526#

MHMDHMSATRPEAGAQEARRPVRIANCSGFYGDTLSAARDMVEGGPIDVLTGDYLAELTMFILYRARHKDPATGYAKTFLTQLEEVLGTCLDRGIRIVANAGGLAPGVLADEIGKLAARTGLAPKIAYVDGDDIVGRLGDMRSAGESFTHVDTGRPLADRTDAPVTANAYLGGWGITAALRAGADVVVTGRVTDAALVSGPAAWWHDWAPDDFDRIAGAIAAGHVIECGPQACGGNYPFFEEITDRRYPGFPLAEVAADGSSVITKHPETGGVVSTGTVIAQLLYEIGGPAYLGPDAVSHFDTVELTQQGPDRVSISGVRGSAPPDRLKVAMNFLGGYRNTMTFVITGLDIEAKARWARRQMFALIGGEDRFDAVDVELLRFDHIDADKNAEATAHLRVSVKSSDERVAGRAFSGVATAMAVGGYPGLHTTTPPTPASAFGVYWPTLVRRDLVEHRVTLPDGSRHVVPDPPLLASQCGTATFTDRPRQPVVSAGPTRRVPLGTVVGARSGDKGGNANVGLWTRDDDVYSWLRTWLDVGRFRELVPEAAGLEVRRYELANLRALNFVIVGLLGDGVAASTRPDPQAKGLGEYVRSRYIEIPESLLDGHRG

>CORE_REP|Org118_Gene1593#

MSPADAFVSGTRTITAPSKPAPADQPAWNKQKNSSMPTFRYRPFAEEVPGGSPAFDAAPIPFDRTWPDRVVDRAPGWCAVDLRDGNQALIDPMSPARKRRMFDLLVRMGYKEIEVGFPSASQTDFDFVREIIEDGAIPDDVSIQVLTQCRPELIERTFEACQGAANVIVHFYNSTSILQRRVVFRADRDAVKKIATDAAKLCLEIEQRYPDTNWRYEYSPESYTGTELEYAREVCDAVSEIIAPTPEKPLIINLPATVEMATPNVYADSIEWMSRNLARRDSIVLSLHPHNDRGTAVAAAELGYQAGADRIEGCLFGNGERTGNVCLVTLGMNLFSRGVDPQIDFSDIDEIRRTVEYCNQLPVHERHPYGGDLVYTAFSGSHQDAINKGLDAMKDTADASDSDVDDIVWEVPYLPIDPKDVGRTYEAVIRVNSQSGKGGVAYIMKTDHGLVLPRRLQIEFSQAIQKITDGEGGEVTPKEMWDVFAEEYLNPVLPLERIRQKMTAAETDSGTDTITAVVKVDGAEQEIVGSGNGPLASFVDALATIGYDVRVLDYSEHAMSSGDDAQAAAYVECAIGDKVTWGVGIATSITTASLRAVVSAVNRALRAR

>CORE_REP|Org4_Gene5021#

MSESENGSRPVALIDRDWEEQHMSPIENSDSAAWRELEAVRAEAAALRRQLADSPDRARELEARIDSLTIRNTKLMDTLKEARQQLVALREEVDRLGQPPSGYGILIGVYDDQTVDVFTSGRKMRLTCSPNIETSTLEYGQTVRLNEALTVVEAGVYDAVGEIGTLREILDDGRRALVVGHADEERVVWLAGPLAKVAEMDDLEDPDSPIRKLRPGDSLLVDTKAGFAFERIPKAEVEDLVLEEVPDVDYGDIGGLGRQIEQIRDAVELPFLHKDLFREYALRPPKGVLLYGPPGCGKTLIAKAVANSLAKKIAEARGEDAKEAKSFFLNIKGPELLNKFVGETERHIRIIFQRAREKASEGTPVIVFFDEMDSIFRTRGSGVSSDVETTVVPQLLSEIDGVEGLENVIVIGASNREDMIDPAILRPGRLDVKIKIERPDAESAQDIFSKYLVEDLPLHADDVAEFGGDKAMCIRAMIDRVVERMYAESEDNRFLEVTYANGDKEVLYFKDFNSGAMIQNIVDRSKKYAIKSVLDTGNPGLRIQHLYDSIVDEFSENEDLPNTTNPDDWARISGKKGERIVYIRTLVTGKNASASRAIDTESNTGQYL

>CORE_REP|Org5_Gene5463#

MTSTSRYGNALQASAQQRLVCAPVITRLSHLFLRTLRDDPADAEVPSHKLLVRAGYVRRIAPGVYSWLPLGLKVLRRIEDVVREEMNGIGGQEISLPALLPRDPYETTNRWTEYGDALFRLRDRKGADMLLGPTHEELFALTVKGEYNSYKDLPVTLYQIQTKYRDEERPRAGILRGREFIMKDSYSFDLDEDGLAASYAAHRGAYQRIFARLGVEYVIVAATSGAMGGSASEEFLATSPIGEDTYVTCLESGYAANVEAVVTPAPAEIPVEGRPEAVVHDTPGTPTIASLVEWANGAGIAEGYGRPVTAADTLKNVMVKLRHPDGKTEVVGIGVPGDREVDDKRLGASLEPAEVELLTDEDFTANPFLIKGYIGPKALLENGVRYLVDPRVVTGTSWITGADASGKHVVGLVAGRDFTPDGTIEAAEVREGDPSPDGRGVLHAARGIEIAHIFQLGYKYTDAFEVDVLGENGKPVRLVQGSYGVGISRMVAVVAEQMHDEKGLRWPSEIAPFDVHVVVANKDEAARAGAEQVVAGLDAQGLDILFDDRTASPGVKFKDAELLGMPWIVVIGRGWADGKVELRNRFTGEAEDIPADSAVESVLAKIRA

>CORE_REP|Org2_Gene853#

MPKRHGLDPARLRLPEDGDWATIRDHLVERLPRVDPARIDELLRDGGIVDLAGPIAPDAPYVPGGAVWFHRDLPAETDVPFEITIVHRDDTLLVVDKPHFLATIPRGQHILQTALVRLRRDLELPELIPAHRLDRVTAGLVLFIIDPARRGAYQTMFHKRTVRKEYEAIAPYDPALALPRVVRSRIVKEKHVLAAQEVAGEPNAETAIELLEHRDGLGRYRLRPLTGRTHQLRLHMNSLGIPILGDDFYPVLTDKPVDDFTRPLQLLAATLEFTDPISRAPRRFETTRTLQAWTDPARTSGAVARAAPGVAHSIETSSSQSSSGMATAHRDRWIGRIRVARPQMTDRSAPPATIRTLGGMLAPELADEAPPRHKLHAYLDVAVVVLALAGTNLIAHFTTAWASIVTVPVAAVVLLALMRRRGMHWHELGLSPRQWKRGTLYALGAVGVVLAAVAIGALLPVTRPFFLADRYATISGALIASMIVIPLQTVIPEELAFRGVLHGTLDRVYGARGVFAAGSLLFGLWHIASSLGLTSSNRGLAGFVGGGVAGQIAGILLAVLATAAAGVVFTWLRRRSGSLLAPIALHWSVNGAGALAAAIVWHTTLS

>CORE_REP|Org195_Gene4993#

MTSSTDAGPTDQPTPESQPGHRARPERPASERRVRPDRPRPEATSTGPRVRTGKPVRRKAEGQWALGYREPLNPNEQSKKDDNPLNVRARIENIYSKTGFERIDKGDLRGRFRWWGLYTQREQGYDGSWTGDENIDLLEAKYFMMRVRCDGGALNVAQLRTLGQISTEFARDTADLSDRENVQYHWIEVENVPEIWKRIEAVGLKTTEACGDCPRVVLGSPLAGESLNEIIDPTPAIDEIVRRYIGKKEYSNLPRKFKTAISGQQDVVHEINDVAFVGVVHPEHGPGLDLWVGGGLSTNPMLAKRVGVWVPLDEVPDVWEAVVSVFRDYGYRRLRTKARLKFLIKDWGIEKFRQVLEDEYLKRKLIDGPAPEQPTKPIDHVGVQRLRNGLNAVGFSPIAGRVSGTVLTEVAAAVERIGSDRIRFTPYQKLIVLDVPDDKVDALIDELEPLGLQARPSLWRRNLMACTGIEFCKLSFAETRKRSQALVPELEERLADLNAQLDVPITININGCPNSCARSQIADIGFKGQLVDDGDGNQVEGFQVHLGGSLGFDSAFGRKLRQHKVTTQELGDYVERVVRNFVKHRADGERFAQWAVRADEADLR

>CORE_REP|Org141_Gene4813#

MIVELAWTHVEPQRGSRPVTAADAIVLAGGRASRMGGVDKPAIVIGGRSMLNVALGAVSGCVQTVVVGPHRPELDPTVRQVREVPPGAGPVAAIGAGLRALGTEAAPRVVVLAADLPFLTEWAVADLLRRSDQSGADAVFAADESGRPQYLIGVWRRSALTARLTRLDSLVNQPMKALVPDDTVMVTLDGVTDCDTDEEVRAARAVIDGGHDPGHQRPPLLLNEARQTLRDNLTRLTPYHTDLFEVAGAALAAPIRAAGPLPRFDVSAMDGYAVAGDGPWRLRADIGFAGGQRPVGLLPGEAVRIATGAHVPDGTTGVLRDEFAGVTDDHQLRRLPDSPIRSDIRRRGEDLDRGDLIADEGAPVTAALISAAAAVEVPEAAVRGPVRARIIMTGDEIRASGPLHGGQTRDSIGPILPDLLARHGIRTVDRVHLRDTPHGFDEVLAGTDGFDLLVVVGATGSGAADQLRAALSRADAHVLVHRLRLRPGGSTVVAELSTGATVLGLPGNPFAAVAVLTALAGSLVEGRTGSPPAHPLLGPLHNASEIAGQVPRIVPAVADPRGGWRGEPAVRTAHLGGLLGRDGMVVVPADAHDGALVEFLPLPG

>CORE_REP|Org41_Gene2160#

MSESSERNSGAVAARPVQEHRYDVVIVGAGGAGMRAAIEAGPRVRTAVLTKLYPTRSHTGAAQGGMCAALANVEEDNWEWHTFDTVKGGDYLVDQDAAEIMAKEAIDAVLDLEKMGLPFNRTPEGKIDQRRFGGHTRDHGKAPVRRACYAADRTGHMILQTLYQNCVKHDVQFFNEFYVLDLVLTETDRGPVATGVVAYELATGDLHVFHAKSIVFATGGSGRMYKTTSNAHTLTGDGMAIVFRKGLPLEDMEFHQFHPTGLAGLGILISEAVRGEGGILRNADGERFMERYAPTIKDLAPRDIVARSMVLEVLEGRGAGPNKDYVYIDVTHLGEDVLEEKLPDITEFSRTYLGVDPVKELVPVFPTCHYVMGGIPTRIRGEVLRNNDDIVPGLYAAGECACVSVHGANRLGTNSLLDINVFGRRAGIAAAEYAERTDFVEMPENPAQMVQDWLALILSDHGNERVADIRTELQRSMDNNASVFRTEDTLKQALTDIHALKERYSRITVQDKGKRYNSDLLEAVELGFLLELAEVTVVGALNRKESRGGHAREDYPDRDDVNFMRHTMAYKEGTDLLSDIRLDFKPVVQTRYEPMERKY

>CORE_REP|Org105_Gene3157#

MMREFEAPASYTIPEDANNSDNVFRHAEQSPNAVLFQVPNGSGGLRDVTATEFAKTVTGVAKGIIASGIELGDRVAIMSATRYEWAVLDFAIWAAGACTVAIYDSSAAEQAKWILQDSATKLLVVENDKHRATIDEIESGSLPELKEISQIDKGAIDELISRGADLDDRLVHERRAQVGASSPATLIYTSGTTGRPKGVMLTHANLWAESKSDRIALGKFIVEGKKTLLFLPLAHVFARAVALAAFDAKVIVAHTSDWTTLVDQFASFKPHFILSVPRVFEKVFNSAKQKAHDGGKGKIFDLAAETAIAYSEGLDNGGPDLVTKVKHFVFDKLVYSKLRVALGGQCEAAVSGGGPLGARLGHFFRGVGVTIYEGYGLTETTAAITVNTPEHIRVGSVGRPIEGHAAKIAEDGELLLKGSVVFDGYWGNAEATEEAFEDGWFKTGDLGAIDADGFVTITGRKKEIIVTAGGKNVSPALLEDSLRAHPLISQVMVVGDGQPFVGALITLDPEALPGWKERNGVSADTPMEQLVQNPALVAEIDAAVAETNKKVSKAEQIKKTRILTVDWTQETGELTPKMSLKRAVVMKQYAAEVEKIYS

>CORE_REP|Org101_Gene1042#

MALALSTGECQVGTVRPGSIDTLGRPSRAPNLASVMGRSNLRSSCVSAIYMEDLNNAMAKTIAYDEEARRGLERGLNALADAVKVTLGPKGRNVVLEKKWGAPTITNDGVSIAKEIELEDPYEKIGAELVKEVAKKTDDVAGDGTTTATVLAQALVREGLRNVAAGANPLGLKRGIEKAVEAVTAKLLDTAKEIDTKEQIAATAGISAGDSSIGELIAEAMDKVGKEGVITVEESNTFGLQLELTEGMRFDKGYISGYFVTDPERQEAVLEDPYILLVGSKVSTVKDLLPLLEKVIQAGKPLLIIAEDVEGEALSTLVVNKIRGTFKSVAVKAPGFGDRRKAQLADIGILTGGEVITEEVGLSLETAGIELLGQARKVVITKDETTIVEGAGDAEAIKGRVAQIRAEIENSDSDYDREKLQERLAKLAGGVAVIKAGAATEVELKERKHRIEDAVRNAKAAVEEGIVAGGGVALLQSAPALDDLTLTGDEATGANIVRVALSAPLKQIAFNAGLEPGVVAEKVSNLPAGHGLNADSGAYEDLLAAGVADPVKVTRSALQNAASIAALFLTTEAVVADKPEKAAAPAGDPTGGMGGMDF

>CORE_REP|Org113_Gene3852#

MPSDNSDFTINRRGLLALGGMAAAATATALGAPRAWAAPSATADADVIVVGAGLAGLVATSELAAAGRRVLLLDQEPEQSFGGQAHWSLGGLFFIDSAEQRLAGIKDSFDLARGDWFRTAGWDRGPDDTLGEDYWGKRWAENYLQFAAGEKQAWLRGLGMNWVPVVGWAERGQADGGIGNSVPRFHITMGTGPGVVEPFEKLVRDAAGKNVTFAFRHQVDELVVTGGAISGVRGTVLEPSGAARGTPSSRIKVGEFEFRAAQVIVTSGGIGANHELVRRNWPARLGKAPARMITGVPAHVDGRMLAIGESAGARLVNRDRMWHYTEGLKNYAPIWPGHGIRVLGAPSSMWFDAEGRQLPAPGIPSVDTLGTLDLIMRTGYDYSWFVLNKKIISKEFTLSGSEQNPELTNKDLAAYLANRALTDTPAPVKAFMDKGQDFVVADTLTELVAGMNKLTGADLIRLDSLRDQIAYRDNELGNPNSTDPRIVAIRRSRDYIGDNLFRTTDPHPILAPDAGPLIAIRMNILTRKTLGGLQTDLSGRVLDARGEPIRGLYAAGEVAGFGGGGVHGYRSLEGTFLGGCLFSGRQTGRAAAKESA

>CORE_REP|Org138_Gene6165#

MTQTDLHVAPSTLPAFTPLTTREAVSPRCDTPLPRHETALSPHDPALLSDREGALSELGGALSDCASAVSDRSGVNPGRDGAPPERAALSAQGGDLSDREGAGSGQVRPRAESGGAPPEREGFARGREPVTEYVAAQEVPVQQSASTHDEDRAPYVPLRDIVDPHRVGPTLEWLDDHAPHVIADELARMDAVTAGMVFRLLDKDRALDVFEELEPVDQQQILSGLRDERFRELVEEMDPDDRARMLREAPAKVAKKVLAGLSPRERRMTAQLLGYPEGSVGYYMTPEVVALPRNLPVAQALQWVRTKGGNAETVYTLPVVDGGRRIIGVVELRDLVLSSPDAMIADLVAAEPVFVRATDSAEKAARLMQGANLINLPVVDSEDRLVGLLTIDDAIEVIEAADSEDVARQAGAAPWEGHYMAAGVFQLARYRAMWLTLLLVAATLTVSVTDLFEGTLEQAAHLALFIPLIIGAGGNAGAQAATSCVRAVAVGEVRGSDLFRVVWRECRVGLLLGTMLALIGVVIGGLFVGMEIAAVVGITLVLICAWAATIGGTMPLLAKKLRIDPAVISAPMVTTLVDATGLIIYFTTAKLVLGI

>CORE_REP|Org1_Gene577#

MRCHGGRAGIVTAHTVRVLEKADTLTRLRARGVVVVRRTEDVIDLNYVGLVVATVFFALSVTPSLVPRDWLFQGLISGINAALGYGLGCLLEWLFRLWVRPRLKVPPAPTWVRYAVKTAVLLTAALTAALMLVQSARWQREITALMGMEGTTTPAYLRTGLLSLAVGVLVVAGYRTVREIILFLARQLNRWVRVPRELAPAVGALVLVVAAVTIFNGVASRAFFAVANSAFSVRNDHTSPNAVQPQQPERSGSPESLAAWDTLGFEGRWFVSHGPTASRIAAVTGRPAREPIRAYVGLESAEDGEDQAELAVRELERTGAFDRQVIVVVTTTGTGWVNSLAAGAIEYMFGGDTAIVASQYSYLPSVLSFLADRGKAAAAGERLFDAVHEHWSQRPPDQRPKLFVYGESLGSQGSEAAFDGLADLRAKVDGALWVGPPNSNRLWEQFVARRDPGSPEVLPVYADGLVVRFAANPPDLAVPGPEWRSPRIAYLQHASDPIVWWSTDLIFSRPDWLSEPRGSDVSSQMRWAPFVTFWQVTADLTNAQGVADGHGHRYGSLVLDAWAAIAQPPGWTPELAEQVRFQLEAAEEFERVVK

>CORE_REP|Org98_Gene7027#

MLTEIRIDGLGVIATATAQFHAGLTCLTGETGAGKTMVVTSLHLLSGARADAGRVRLGAPRAVVEGRFTVDDVNDAARAEVAQVLEAAAAEPDDDGSVIAIRTVGSDGRSRAHLGGRGVPASVLADFTASLLTVHGQNDQLRLQRPDQQLSALDQFAGDAVGTALRKYQVLRRSWLDARTELLERTARSRELALEADRLKHSLNEIDAIAPEPGEDVRIVDEVRRLSDLDSLRDAAATAHGALAGPADTPEDGSGALEALGTARARIEAADDPALVALAPRLADAIAVVIDVTTELSGYLSDLPSDPGALDSLLTRQAELKTLTRKYAPDIDGVLAWAQEARTRLGSLDVSEEALAKLAAEVDTAADRVREAAKKLSGVRAKAAGKLAAAVSAELGGLAMGKARLEVEVRPLLAGAQDTAPLTVDGQELHAGHTGIDEAEFRLSAHSGAQSLPLSKSASGGELSRVMLALEVVLASSDHGATMVFDEVDAGVGGRAAVEIGRRLARLARTHQVIVVTHLPQVAAFADTHLVVDKSDDGKGAVNSGVRALTNDERVVELARMLAGLDDTETGRAHAEELLATARAEKAGAEAATR

>CORE_REP|Org105_Gene5302#

MNAHRVLRSPLGKIPIFRTDPMSTWGFLLTGSPSWVNRRYWHWLHSEPMTSVQQQPTPGPAGAPDIHTTAGKLADLRNRLEEAKHPMGEAAVDKVHAKGKMTARERILALLDEGSFVELDALARHRSVNFGLENNRPLGDGVVTGYGTIDGRDVCIFSQDVTVFGGSLGEVYGEKIVKVMDLALKTGRPLIGINEGAGARIQEGVVSLGLYGEIFHRNIQASGVIPQISLIMGPAAGGHVYSPALTDFVVMVDQTSQMFVTGPDVIKTVTGEEVTMEELGGANTHMTKSGVAHYVASGEQDALDYVKDLLSYLPSNNRAEAPRFPATDPIDGAIEDSLTEEDLELDTLIPDSPNQPYDMHEVIRRLLDDDEFLEVQAERAMNIIVGFGRVDGRSVGIVANQPTQFAGCLDIDASEKAARFVRTCDAFNIPIITLVDVPGFLPGTGQEYNGIIRRGAKLLYAYGEATVGKITIITRKAYGGAYDVMGSKHMGADVNLAWPTAQIAVMGASGAVGFVYRKQLAEAAKEGADVDALRLELQNEYEDTLVNPYVAAERGYVDAVIPPSHTRGQIVSALRLLERKMVTLPPKKHGNIPL

>CORE_REP|Org145_Gene4689#

MGGSCACPVLTLPVRNHCRSERCRFVRLPVRETGRAESAQHCRRTARRSQPVITATDLEVRAGVRTLLSAPGPALRVQAGDRIGLVGRNGAGKTTTLRILAGEGEPYAGKILRSTEIGYLPQDPREGDLDVLARDRVLSARGLDTLIRDMEKQQALMAEVADEAEREKAVRKYGRLEERFSALGGYVAESEAARICHSLGLPDRVLGQPLRTLSGGQRRRIELARILFSASDGSGGRSDRILLLDEPTNHLDADSITWLRGFLQNHDGGLIVISHDVELLEAVVNKVWFLDAVRGEVDVYNMGWKKYLDARATDEQRRRRERANAEKKASALKAQAAKLGAKATKAVAAQNMVKRAERLLDELDEVRVADKVARIKFPEPAPCGKTPLMAENLTKVYGSLEIFTGVDLAIDRGSRVVVLGLNGAGKTTLLRLLAGVEQPTAGQLVPGHGLKVGYFAQEHDTLDDQATVWENIRHAAPDAGEQDLRGLLGAFMFSGPQLDQPAGTLSGGEKTRLALAGLVSSAANVLLLDEPTNNLDPISREQVLDALRTYAGAVVLVTHDPGAAEALSPERVILLPDGTEDHWSAEYLELIQLA

>CORE_REP|Org119_Gene804#

MTKKPESQFLGPEQRRTAWERFGKDHFDVVVIGGGVVGAGIALDAATRGLQVALVEARDLASGTSSRSSKMFHGGLRYLEQLEFGLVREALRERELALSTLAPHLVKPLRFLYPLTHRAWERPYVAAGLVLYDTMGGAKSVPGQRHLSRMGALRLSPGLKRSALIGGVSYYDTVVDDARHTMTVARTAAHYGAVIRTSTQVVGFLREADRVVGVRVRDSEDGRTAEVRAHVVINATGVWTDEVQALAHQRGRFHVRASKGVHIVVPRDRIVSDAAIILRTPTSVLFIIPWGTHWIVGTTDTDWNLDLAHPAATKADIDYLLDRVNEVLVTPLTHDDIDGVYAGLRPLLAGESDETSKLSREHAVARVAPGLVGIAGGKYTTYRVMAYDAVDEAAQDIPARVSPSITEKVPLLGADGYFALVNQTVQLAEAYGVHPYRVKHLLDRYGSLIDEVMAMADGKPELLQPITDAPSYLQVEAVYAAAAEGALHLDDILARRTRISIEYSHRGADCAEEVAQLVAPVLGWDDAEIDREVTTYRARVEAEIRSQTQPDDASADALRIAAPEPRPEILEPVPADGSASKAVRQPNS

>CORE_REP|Org20_Gene4022#

MTFDYDVVVVGSGFGGSVTALRLTEKGYRVGVLEAGRRFADEEFAETSWDARKYLWAPALGCFGIQRLTLLKDTFIMAGAGVGGGSLVYANTLYEPPDKFYRDRQWAHITDWKAELAPHYDQAKRMLGVTTNPATTPSDRVLAEVAEEMGVAESYRSTPVGVLFGGKGVRPGENLPDPFFGGVGPARATCTHCGECMTGCRHNAKNTLVKNYLYLAEQAGATVHPLTTVTDVRPRPGGGYTVSTVRTGRWVRKARRTFTAEQVVFAAAALGTQKLLHKLRDRGSLPDISPRLGELSRTNSEELLSVRSRRKDSDFTKGVAITSSIHPDDDTHIEPVRYGKGSNAIGLIGTAMIDPDGRTGKVRLWARTMRRLGLRDALHLQNPRGWSEQMIGLLVMQSVDNSITTYTKRGLFGRTMTTRQGAGEPNPTWIPAGHEVAHRVADKIDGIAGAGWSALFDIPMTGHFIGGCVIGDSPDTGVVDPYHRMYGYRGLHVIDGSTISANLGVNPSLTITAQAERAVALWPNKGEADPRPEPGQPYRRIAPVPPRNPVVPASAPAALRLPIVEITGPATESEPAAGSPA

>CORE_REP|Org216_Gene3082#

MLIRLLRTYLSPYRAQLAGVVALQLVSVIAMLYLPSLNADLIDNGVTKGDIDYIWHTGLWMLAVTAVQIVASASSVFLGAQAAMSAGRDLRAALVHRVGTFSAREVGLFGAPSLITRNTNDVQQVQLLVVMSVTVLVMAPIMCVGGIIMALREDLKLSWLLLIAVPALALAMGLVVARLVPGFREMQARIDVVNRVLREQITGIRVVRAFVRERQETWRFGLANTDLTEASLRVGRLMALMFPVVMLISNVTTVAVIWFGGHLIDDGELQIGSLTAMLSYIMQILMAVMMASFLAMMAPRAAVSADRIGAVLTTESSVVPPEFPKPFAGDPGRVEFAAAEFAFPGAEKPVLRGIRFTVEPGTTTAIVGSTGAGKTTLLNLIPRLIDVTAGAVYVGGTDVRELDMELLREQIGLVPQKAYLFSGTVASNLRYGRPEATDEELWRALEIAQAADFVRDMPQGLETPVAQGGTTVSGGQRQRLAIARALVRRPRVYLFDDSFSALDVATDARLREALRPETRDASVIIVAQRVSTIRDADQIIVLEDGEMAGIGTHEQLLRDCAEYQEIVASQLSAQEEVR

>CORE_REP|Org128_Gene3333#

MSATPFRGWWRANCDLNPISVSASRGRVRPVSYTHGVWDAPLLGETIGANLDRTVAIHGDRDALVDRVTGVRWSYREFAAEVDAVALGLLEAGIGKGDRVGIWSPNRAEWTLVQFATAKIGAILVNINPAYRSEEVRYVITQAGIRMLISAREHKSSNYAEIIGRVRPECPDLEQVVLFDSAAWEALVAAGRAADPSRLAEAGTRLTADDPINIQYTSGTTGFPKGATLSHHNILNNGYFVGELCGYTEADRICIPVPFYHCFGMVMGNLAATSHGAAMVIPAASFEPRATLAAVAEERCTSLYGVPTMFIAELAHPNFESFDLSSLRTGIMAGSPCPVEVMKQVIERMGMAEVSICYGMTETSPVSTQTRRDDTITQRTATVGRVGPHLEIKIVDPDTGSTVPRGEPGELCTRGYSVMLGYWNDPDKTGEAIDAARWMHTGDLATMDDDGYVAITGRIKDMVIRGGENIYPREIEEFLYTHPDILDAQVVGIPDPKYGEELVAWIRVREGAATVDAPTLAQFCDGRLAHYKIPRYVHVVDEFPMTVTGKVRKVDIRATSVRLFGVPELDQQQTGEQ

>CORE_REP|Org163_Gene4686#

MRVVGGVGENGGESIGGGTVNDGPLIVQSDKTLLLEVDHASADAARQAIAPFAELERAPEHVHTYRVTPLALWNARAAGHDAEQVVDALVSFSRYAVPQPLLVDVVDTMARYGRLQLVKHPAHGLTLVSLDRAVLEEVLRHKKIAPMLGARIDDDTVVVHPSERGRIKQMLLKIGWPADDLAGYVDGEAHSIELDYATDGWHLRDYQQMAADSFWAGGSGVVVLPCGAGKTMVGAAAMAKAKATTLILVTNTVAGRQWRRELLARTSLTEEEIGEYSGERKEIRPVTIATYQVITRRTKGEYKHLELFDSRDWGLVIYDEVHLLPAPVFRMTADLQSRRRLGLTATLVREDGREGDVFSLIGPKRYDAPWKDIEAQGWIAPAECIEVRVTLTDAERMTYATAEPEERYKLCSTAHTKIAVVESILAQHRDAPTLVIGAYLDQLDELGAALDAPVIQGSTKTKDREELFDAFRRGEIPVLVVSKVANFSIDLPEASVAVQVSGTFGSRQEEAQRLGRLLRPKQDGGQAHFYSVVARDTLDAEYAAHRQRFLAEQGYAYRITDADDLLGPAIG

>CORE_REP|Org24_Gene2076#

MREISPEWNCGAGHPGGTVLGTAGGWSGVRVGRAADWRLVSTQLLVGGRIYSSSNPDATAMAVSDGIVVWVGQDKPGRALHPDAEVVDLDGAFVAPAFVDPHVHITALGLQLTGLDLSRAASLAECLELVREFARTRPQGAILGDGWEETRWPERRAPTSAEIDAAAPGRLVYLTRVDAHSAVVSSALLDAVPGVTEAAGFTRGEPLRENAHHRVRAAVLDGLDRAQRDRARRAALDHAAAHGIVAVHECAGPEISGHADVAELLEFSHGVEVRVYWGEAVRTADEARTLVKELGVHALGGDLFVDGSLGSHTARLRTPYADRDTRGTAFLDADAVAAHLRACTEAGIQAGFHVIGDAAMDIVVDGFTRAAAELGGPAVATRGHRVEHAEMVDAEQIAQLAAWGVIASVQPGFDAAWGGVDGMYAARLGADRAATLNPFAAMAAAGISLAIGSDAPVTPLHPWAAVRAAAHHRTPGHGISPRAAFTAATRGAWRAGGVRDGVSGTLVPGAPASYAVWAADDLVVAASADSVQRWSTDPRSRVPGLPPLDPTAPLPRCLRTVHRGVTIHGD

>CORE_REP|Org50_Gene288#

MRIRSTRGCTRRRSARWAVPVVALGLVVAGCGANDGESATGLSDALGTTSDINPKNRDEVREGGNLRLAVTSFPANWNTLSTDGNDGEIGDIERPLMPRAFDVDAAGNLTVDKDFFTDVALTGTNPQQVTYTINPQAVWSDGSPITWEDIAAQAHALSGRDKRFLIAITNGFEFVDKVERGVDDRQAVLTFNHPFGEWRGQFAGDTALFPKSVTADPESFNKGLVDRLGPSAGPFVVQSTDRSQGRIVLGRNPKWWGETPKLDTITYSVLDHAAWVGALQNNELDLIRLASIDEVKTVRNTDGLVIRRAPGNRWRHITFNGAPGSILADPRLRVAIAKAIDRQGIATATQNGLVENPKPLNNHIFLQGQDGYQDNSAPVAYDPDQAARELDALGWKLNGDVREKDGRKLEIRDVMYNDPLWIQIAQIIQQNLARIGVKLTIDTKPGAGYFTDVIIPGDFDAAQFIFSGDAFAMSDIRQIYYYDPNDLQGNYGRIGSPELNALIERTLTELDPKKAIELANEVDRKVFEEGHSLPLTQSDGSYGARADLANIGSPGLASYDYTKIGFVK

>CORE_REP|Org5_Gene6751#

MSSSSLHVRVGVCNLGLVSSDITATAAWRKLHDHHSAIADRHLREFFADDPDRGRELIVEAGELRVDYSKHRITRETLDLLLDLAATAGVARRRDAMFAGEHINTSEDRAVGHVALRLPAGASMMIDGADAGVAVHEVLRRMGDFTDGVRSGQWRGATGERITTVVNIGIGGSDLGPAMLFQALRHYADAGISARFVSNIDPADLTAKLDGLDPARTLFVVASKTFSTLETLTNATAARRWLVAALGEDAVAEHFVAVSTHAQRVADFGIDTANMFEFWDWVGGRYSVDSAIGLSIMVVIGRERFAEFLAGMHSIDEHFVSAPPERNAPILLGLLGVWYSNFFGAQSRAVLPYSNDLARFPAYLQQLTMESNGKSVRLDGSPVTTSTGEIFWGEPGTNGQHAFYQLLHQGTRLVPADFIGFARPTDDLATRDGSGSMHDILMSNLFAQTKVLAFGRTAAEIEAEDGDAPGFDPALVPHRVMPGNRPSTTILAPQLTPSVVGQLIALYEHQVFVEGTIWGIDSFDQWGVELGKQQALALEPLLTAAEDPAPQSDSSTDALIRWYRGNR

>CORE_REP|Org105_Gene607#

MAGAARAPATLVPNARAVSYAICHISRRFGVGAIDTGQLIAEHYRLVERIGSGGTGVVWRAIDERLQRSVAVKQIHIKPSLPEAERDVLRQRAIREARNAARFQHPNAIVVFDITEHNGDPCLVMEYLKSRSLAQVLSAQGAVPLNQVARIGEQVASALIAAHQAGIVHRDVKPGNVLLDDHGTVKITDFGISRAAGDVTLTETGLICGTAAYLAPEVARGADPTPAADVFALGATLFHALEGEPPYGASSNPLAVLYAAANGQVSEPRNAGPATDFLLQLLSPQPEDRPTMRMARDQLAAFADAGADAVPAGFVPASEAYGRRNGDGATEVVATRALRSRAAAAGPATERQPAPRRSSVAVDTAAHRPVEPARDHPDTAAHPRTTTQPRPASPAAGKRRAVLIGALVGAVVAVSALLVSAFNSADDDSSPQASASSVATAPSGDASTSAAVPLLGQTPSVGGKVTDIGAAGQLVERFYSDPESSWSLLTPAAQKVYTDQQGFRQYWSADGRTIQSFGRIYAPNGVNADGSVDMRVTGLTYGGQSKNPDLRIIDAGGGRLLIDSDTR

>CORE_REP|Org210_Gene6047#

MCKSSLVRRKNTFYFGPVSYNIADLVEHTIDLVPDRVALADDVRSVTYAELEDRANRLAHYLQEQGVQPGDKVGIYSRNTIEAVEAMVAIFKARAVMINVNFRYVENELQYIFDNSDMVALIHERRYSDKVSAVRPNTPNLKTVIAVNDGTEAEVSLPADSVEYEAALAASHSERDFGDRSNDDIFMIYTGGTTGMPKGVMWRHEDWWRVLGGGINFITGEAIEDEWQQAKAGAAGGQMVRYPIPPMIHGGSQSATFHGLFDGGKTIMLPEFGAHTVWQAIDRHGVNLIFITGDAMARPMLDALKEGHPETGEPYQHANLWAMASSAALFSPALKDEFIELLPNTVITDSIGSSETGFGGLSVAAKGATHTGGPRVKIDASTAVIDEEGNPVAPGSGQVGVLARTGNIPLGYYNDPVKTAATFKEFNGVRYSIPGDFARVEEDGTVTMLGRGSVSINSGGEKIYPEEVEGALKCHPEIFDALVIGVPDERWGQRVAAVVQCRGADRPTLEELRPVLTREISSYKLPRSLWFVDEIKRSPAGKPDYRWANEHAGSRPADEESQAASK

>CORE_REP|Org129_Gene4814#

MSFVVTQPCCNDAACVQVCPVNCIRPTPDDPDFRSTEMLYIDPRTCIDCGACMEACPVDAIHPEDELPDDQLRYREINADYFRRNPLDAGTPPAPQPPVKVPKPDSPLRVAVVGSGPAGVYAAAELMARMPSGGIDIEMFDRLPTPWGLVRAGVAPDHLGTKAITEVFRRIAAKPGFRFHLNVEIGRDLTHDELLAHHHAVIYAVGALEDRKLDIPGADLPGSVAATEFVAWYNGHPDYANRAFDLTGERAVIVGNGNVALDVARLLVSDPDALVRSDMAEHALEALRESNIREVVVLGRRGVAQAAYTTPELLALGRIPGVDVVVDPRELDLGGVAGEDPSFSTDLKTRVAVEYSDSTTTPGNKRIVLRYLASPVRLLGADHVEGVEIVRNELVRTERGTLEARPTGTTEILDAGLVLRSIGYRGTAVPGVPFDADAGRIPNIDGRVLDPDTGAALAGVYTTGWVKRGPSGVIGTNKLCAQNTVAALIDDLLSDRLPQPKPGRDRDALSALVVERRPEVVDGKGWLAIDAAERKGGRDRGRPRVKITDTAEMVEIAGAAMHPVG

>CORE_REP|Org113_Gene5713#

MRPEVVVVRWIFMLDRARVTKGDKVILDDVSLTVLPGAKIGVVGPNGAGKSTVLRVMAGLELTAGGEAVLAPGITVGILAQEPELDETATVRGNVEAAVAGTQALLARYTEIAERLADDADEELLAELGALQEQLDRRGAWDLDSRLDQAMDALRCPPPDAGVTTLSGGERRRVALCRLLLQRPDLLLLDEPTNHLDAESVQWLEQHLSGYPGTVVAVTHDRYFLDNLAEWILELDRGHAHPYRGNYGIYLDTKATRLRVEGRKDAERLRRLRRELEWIRSGPAARQAKGAARLRRYEEMAAAADGARVRTFDEIRIPPGPRLGGLVVEADHVDKSFGDHTVIRDLSFSLPRNGIVGVLGPNGAGKTTLFRLLIGELTPDAGKIRIGDSVEISYVDQNRVRIDPGRTAWDVVSGGHAVIGVGTMEVPSRAYLAAFGFRGVDQQKPSRLFSGGERNRLNLALTLKQGGNVLLLDEPANDLDTETLDSLENAIDEFAGCVIVTAHDRWFLDRLATHILAWEGTAADPGRWFWFEGNFAAYEQNKLARLGPDAARPHRLTHRRLTRD

>CORE_REP|Org5_Gene1415#

MVAPSSSRKPQKVNDMTAAADPQAFAARITADLTGPGGPFEMGVEEVLGAPIPLMRNRRRSMADLFHAAAAWGDHDYLVTADRRLTFAEHGAAATALARGLAERYGVGKADRIGILAANTPEWVMTFWAAQLLGAIPVGYNAWWAPREIAYGLEHTQPTVVVADAKRAALLTDTGIPVLTMESDLPALVADHPGPAPEAAIDEDDPAVILYTSGTSGRPKGVVHSHRNLLAVCDYHRFTDAMMVAFRGQQLGSGPSPRRFLLTSPLFHIASLHNLIVPRLATGATVVMHTGSFDPDRVLALMERQRVTNWGAVPTMVSRMLDCDLSRYDLSSLVAFSLNAAPSSPAFHQRLRSELPMAEVALTTSYGLTESGTAATVATPPVLAAFPDTVGMPIIGVSVEIRDPDNKPLPDGEEGEICVRSPYVMLGYWNDPAATAQAIDAERWLHTGDFGILEQGRLRLSGRRSDLILRGGENVYPIEIENALDEHPEVLESAVLGVPHDDLGQEVAAVVVVTDPGAVTEEQLRAFTAERLAYFKVPARWVITAQPLPRNATGKVVRREIEI

>CORE_REP|Org52_Gene3143#

MSPALAAGLQIASVVAVLALVYVPLGDYMARVYTSSSDLRAESWLYRLARVDPRAEQTWYGYAGSVLGFSLAGVLVLYVLQRIQGVLPLSHGLAGVSPAVAFNTAVSFVTNTNWQSYVPETTMSPLTQSAGLAVQNFVSAAVGMAVAVALIRGLVRVGRGGEVGNFWVDLTRGTLRILLPLAFVIALILLSQGVIQSYRSGFTGVGLDGRPVTTALAPVASQEAIKELGTNGGGVLAANSAHPFENPTPLSNVVQILAILLIPVALTRTFGTMIGNRRQGLTVLAVMAGIYAVILGVTTAAESGARGAAATAAGAMLEGKEVRFGIPGSVLFAVSTTGTSTGAVNSAHDSMSPLGGGAVLVNMLLGEIAPGGVGSGLYGILVLAVIAVFVGGLLVGRTPEFLGKKLRRREITLAALAVLVMPALVLIGTAITVILPDTAAALGNSGDPGTPGAVHGFSEVLYAYASASNNNGSAFGGLTVTSDWFQSSLGLCMLFGRFLPILFVLALAGSLAAQPRTPATAGTLPTAGAGFAGLLTGTVVLVAALTFFPVLALGPIAEALQ

>CORE_REP|Org202_Gene4973#

MRVRRPGRLDPVSTTLHARGLSAGHGERTLFDDLDLTIAPGDVIGLVGVNGAGKSTLLRMLAARETPTGTITLSPPDATVGYLAQEPERVPGETVLDFLGRRTGVTAAQRAMDAAAERLAEGGTDEYSPALERWLALGGADLEARAQEVAADLGLAESLADGLGTPMTALSGGQAARAGLASVLLSRYDILLLDEPTNDLDLDGLARLEDFVRGVRVPLVVISHDREFLARTVNRIVELDLAQQQVGLYDGGYEAYLAEREIARRHAREAFEEYADTRAALETRAQMQRNWLEHGVRNARRKARDPRKLDSDKAGRKMRAEATEKQAAKARQTQRRIERLEVVEEPRKEWELRMTIAAAPRSGAVVATATDAVVTRGDFRLGPVTTQIDWADRIVLTGANGAGKSTLLGLLLGRIAPDSGSAALGSGVEIGEVDQARSLFRGTTPLAERFGREMPDWPDAEIRTLLAKFGLRGPHVLRACDTLSPGERTRAALALLQARGVNLLVLDEPTNHLDLPAIEQLEQAVDSFTGTLLLVTHDRRMLDSVRATRRWHLRDGLLHED

>CORE_REP|Org129_Gene4552#

MCAHERSLVPVQPSPQVLRPAVALSTPVQAVAELTGARLNSGDPAGIEITGIEQRSNAVQPGDLFAGLAGAKAHGARFAADAVERGAVAVFTDAAGAELIGELDVPVLVHDDPRAVLGELSAAVYGHPSRRLQVIGITGTSGKTTTSYLVEAGLTAAGLSTALIGTIETRIARRVDPNPEETAAGASAAVEYRRVPSALTTPEAPQLHAMFALMVEQGVRAVVMEVSSHALALGRVDGVHFSVGAFTNLSQDHLDFHADFEDYFAAKRRLFVPDPGAPQRQVAADTCVICVDDAWGRRLAREAGGRARVVTVATADCPTGETEPEWTVTGAAALADGGQQFTAIGPAGEISARLRLPGRYNIANGLLAIAVCAAAGVDAAVAAAALGEVDVPGRMQRVNAGQDFLALVDYAHKPAAVESVIATLRRHLRDSGGRLAVVVGAGGDRDAGKRPLMGATAARGADLLVITDDNPRTEDPAEIRAAIRAGALGIAEAERGEVREIGDRAAAIAAAVDWARRGDVVLVAGKGHETGQEIAGVKYPFDDREVLGQALERKTKDLTVS

>CORE_REP|Org5_Gene948#

MAESSGTDSRPLFGRISLPNLLAAQVLGLIVGLIVLIAGVPGWYALGAAIVAGLLILIPIGKRTIASWIATLWRYFTRQDYDLGDTVDFRGPDGRSLGLYWEGSRVVAVVEVLPPRGGLTRIDRNTVHASHLLPLPELAQCLSQHDILLSGIDIISHGHRSRSGTPAGPIYESLLGPLPATAHRTVWLAISFDALACPEATSRRGGGTEGAGRAVTIATQRIVRALEDADCSSRVLTAPEIRQAVWQISAGVDPRELTQRWRYAELGNSVNIGAAVDPKQLGSDLLAQLWVAPSRGTTVTVRLRPGTSAETVNIGAAWRLTARELPEKTNTKGMVSLSGRHRQSLLAHLPIAIPGMDDTVPMSQYPIDVVGVLHLPSSGCGQLIGSDAEGNGVAVRLVGQGIAAVYVAGELYLAQQLVFRALAVGERILIRTDRPQAWEQLVSTIGNPERLTIAMETHQSDAGFTAAVVDGVLAPAPHAGVTTIYLTGDPMGWPATKPDLSIHQPGAMGNHVVMRTGTAQVDLTLVSIPRETTYIGDPRGRRPAPQQQQQPRRRPAPARR

>CORE_REP|Org106_Gene4102#

MGVPRKLETSAFFGVLVAGLVGIPAPTAAAAPHPWMPAPVNTCGETGFDPLARLPMPGQPAPPPVQIPSEIEIPVPIPELTLVPVPDPPPDYTRVAADALPKDPCGDPCPDLREPVEPETAPPAEDDTKAEEGTEETPEEEEELSVDVPGSADTGSGSSAGSGSSAGSGSSAGSGDSGSSFAGVQVPRISIRPEIEPIPIPVPGGPQQDPQPAPPEVVHPVEPGPLAPAPAAPVVDTAELVEQVTGHGSENRTDVRWAINGTDLGIMWETKPGEVAMVFGDTFGEGWEYGGAGGSDWRSNALAYSTDTDLSDGVRIDRMVQDSRCHAAELLSSRKIKNWETTVIPTSGFALGNRQYMSYMSVNRWSRIPGMWWTNYGGIAYSDDNGSTWVKDQHAKWDNLFGLGRFQVAAMVPQGDYVYMFGTPNGRVGVVGLARVPKEHVLNKSAYQYWVNGDWAPAAENQATPLFLGIASELSVRYDAGTERWQMVYLDSARGAIVLREATTPQGAWTDGVPLVSTSDYPKAYGGFIHPWSTGDELYFTMSAWDSYNVYLMRSPVRSR

>CORE_REP|Org4_Gene1527#

MGGVSDMATQLGNKVSAQADSADERYRAAAFLKRSINKVFPTHWSFLLGEIALYSFIILLLSGVYLTLYFDPSMTEVVYDGSYQPLRGVTMSRAYETALNISFEVRGGLFVRQVHHWAALLFAASIIVHLFRVFFTGAFRKPREANWVIGSLLLILAMFEGYFGYSLPDDLLSGTGLRAAFSSITLGMPIIGTWLHWLMFGGDYPGTIIIPRLFIAHVLLFPGIMLALIAAHIALVWYQKHTQYPGPGRTEKNVIGARIVPVFSLDQGAFFAFTLGIVAIMSGVFQINAIWTMGPYNPAQISAGSQPDFYMMWTDGMMRLIPPWELYLGRYTVPAPVWGALLMGVVFTVLITYPWIEKRLTRDTAAHHNLLQRPRDVPVRTAIGAMAIAFYVVLTLSCVNDIVAYKFDISLNATTWTGRIGLLLLPPLAYFVAYRVCLGLQRSDRAVLEHGVETGVIKRLPHGEYIEIHQPLGPVDEHGHPIPLAYQGAPVPKKMSKLGLAGKPGTGSFLRADPWQESERNHETDHAEEHKQLAVLRDYQERDQRNGNGSHGNGSSDS

>CORE_REP|Org18_Gene2143#

MLSHPERLPLLTASTDGAVSSGPRGLPAEVSRRRTFAVISHPDAGKSTLTEALALHAKMISEAGAIHGKAGRKSTVSDWMEMEKARGISVSSTALQFNYRAAGSDIDNVINLVDTPGHSDFSEDTYRVLTAVDAAVMLIDAAKGLEPQTLKLFQVCRHRGIPVITVINKWDRPGRAPLELLDEIDERIGLTPTPLFLPVGIAGDFRGLLRRGPDGEAVEYIHFTRTAGGATIAPEESLTPEQAQAREGEAWETAAEESELLSATGQDHDQELFLAGQTSPVIYASAMLNFGVRQLLETLVALAPAPAGRRDVDGGMRETSDPFSAVVFKVQAGMDTAHRDRLAFMRIVSGEFERGMVVTHAQTGRPFATKYALTVFGRERATVDTAYPGDVVGLVNATALAPGHTLFVDKKVEFPPIPSFAPEHFAVLRAQSAGKYKQFRKAIDQLDSEGVVQVLRNDARGDASPVLAAVGPMQFEVVTARMQAEYNVETQMDHLPYTLARRTDAASAEELGRQRGVEVFTRSDGVLLALFSDKWRLQYIEKEHPGLTLEPLVATAD

>CORE_REP|Org215_Gene1101#

MTPPPQTTPLALHDAARAYGDAPAVVDGAVRLSWAELLDSVRETARALLARGIGSGDRIGIWAPNTHHWVTAVLATHYVGAVIVPLNTRYVAEEAADVLARVDAKALFIAGPFLGRDRLAELRAAAPDLKIGTTIVIPGDTGPGGTDDAGTAVTDDAAAIGTGGVGVSAAGDAAARTGDDTLTWRNLAALAEQVSAADAIARAESVSPDDLSDILFTSGTTGRSKGTLIAHRQALAGARAWSECATLNSTDRYLVVPPFFHNFGYKAGILACLVTGATIVPQATFDVPETMRLVQDHRITVLTGPPTIYQTILEHPARRDADLSSLRVAVTGAATVPVVLIERMRTELEFDVVLTAYGLSESGGFGTMCRPEDDAETIANTCGRAIGDFEVALADNGEVLIRGSQVMLGYLDDPVATADTIDCDGWLHTGDVGTLDGRGYLKITDRLKDMYICGGFNVYPAEVEQALARLDGVAETAVIGVPDERMGEVGKAFVVRKAGSGLTADDVVAHAKTLLANFKVPRYVEFRDQLPYSAAGKVLKRQLRDDTGRAENEERA

>CORE_REP|Org1_Gene1663#

MDFLLHPCASAVGDGHVGVGTGFAVRGTRGQRLIAGPAGGNWQDRGMGLRRGCGVLVAALAVVAAGCTIERGEQAGPMPAAPAGLERFYEQAVPWGPCAGFTDDQVRLPPNAQCARIEVPVDYADPAGPTAQIALSRIPASGAKIGSLLLNPGGPGVSGLDTVAIANQTPLSERFDRVGFDPRGVGASTPAITCLTPPEADAERAERPEDNTPAGIAAAEADNRDYAAKCVQRSGAELLEHVGTREVVQDMDVIRAVLGDPKLTYLGYSYGTKLGSLYAEKFPDRVRALVLDGAVDSSQDPVQESLRQAAGFQRAFDAYAADCARTPDCPLGTDPAQAVARFRELVDPLWERPAATTDPRGLSYNDAITGVTQTLYTDDLWQVLTLGLQELRDGRGDTLLQLADLYDGRRDDGTYRNTQDAFNAIRCVDDPRVTDPAVAARQDTEYRKAAPFLDDGRGTGAAPLELCAAWPVPNSGEPHSISVQGLPTTVVVSTTEDPATPYQAGVDLAAQLGAALVTFRGNRHTAALVAGNECLDSAVIAYLVDLTIPPAGLTC

>CORE_REP|Org3_Gene3222#

MGVVGASTASRFDTTVQARGIRRRLSVAPATVCGVSIPTSGESIGSGRAPHRVDAEFTSLPLVALADAALSAATAAGASHADLRVHRLVTQSIRLRDGRVEAVTDAAELGLAVRVIVDGTWGFASHAALTPESAAEVARRAVTVATTLRALNRERVELADEPRYDGVEWVSAYDLDPFTVPTTDKVALLQEYSQRLSAADGVDHVTASVLQVKEQTFYADTAGSSITQQRVRLHPQLEAITVDSAAGVFETMRTLAAPAGRGWEYVTGADGIWDWAGELARMPEWLAEKVKAPSVVAGPTDLVIDPTNLWLTIHESIGHATEYDRAIGYESAYAGTSFATPDLLGMLRYGSPIMHVTGDRTEPHGLATVGYDDEGVAGQRWDLVRDGLLVGYQLDRVFAPRLGLDRSNGCSYADSPHHVPIQRMANVSLQPDPHRDTSTEELIARVEDGIYIVGDKSWSIDMQRYNFQFTGQRFFRIRDGKLDGQLRDVAYQATTTDFWGAMEAVGGPSTWQLGGAFNCGKAQPGQVAAVSHGCPSILVRGINVLNTRTEAGQ

>CORE_REP|Org24_Gene6553#

MVPSRRRSPARCSTPDWRGAERYERARWTGAGMLTNGALIADRYRLHRLIATGGMGQVWEALDTRLDRRVAVKVLKAEFSADPTFRHRFRTEAKTTAQLNHPGIAGIYDYGETMDPAGGETAYLVMELVSGEPLNAVLNRLGRLSVAQGLDMLEQTGRALQVAHAAGVVHRDVKPGNILVTPTGQVKITDFGIAKAVDASPVTKTGMVMGTAQYIAPEQATGEDATAASDVYSLGVVGYEALAGQRPFTGDGALTVAMKHVRETPPPLPPDLPPNVRELIEITMAKEPGQRYTSGGEFADAVAAVRAGRRPPPPSGLAGPMTSGATRVLPPGPTVILPTAARGDAATVRYPTPPHAQRAQQPPVATAMMQGPNTPPPGTPPIGGRTAEQGGGRFTNSQKALAGLGVGALVVGAAAAFVLLSGDPPDSTPPTKTSAVVVPPPVPTTTTTTEPPTTTRYVPPPPTVPPTTEEPLPTTTEPPPTTTTQPPTTTPQQPSTTKPAPTTTVKPSKTTIEPPFEIPSWPPTVPGGAGGAFGTTRPAHSSTPAPAAQQGLP

>CORE_REP|Org175_Gene4706#

MTDQPSNDNDSSDYALPDPSGPAPAPNGSVPNGSVANGAGPIDTEVAEPEPIQAQPIGAGGGNSGFVVVANRLPVDLEKLPDGSTRWKRSPGGLVTALEPVLRSNKGAWVGWAGVPDVDVDPIIEDGLELHPVPLTAQEVEDYYEGFSNGTLWPLYHDVIVRPVYDRKWWAAYVQVNRRFAEATAKVAAEGATVWVQDYQLQLVPKMLRMLRPDLTIGFFLHIPFPPVELFMQMPWRTEIIEGLLGADLIGFHLPGGAQNFLYLARRLAGQPTSRGNVGVRSKLGVVQVGFRNVRVGAFPISIASAELDEHSRRRSVRERAAKIRAELGNPKNILLGVDRLDYTKGIDIRLNALEELLMEGRVDPSDTVMVQLATPSRERVQSYIKMRGDIERQVGRINGEFARVGYPVVHYLHRPIPREELIAFFVAADAMLVTPLRDGMNLVAKEYVACHSGLNGALVLSEFTGAAAELRQSYLCNPHDLDSVKDAIASALTDDRDTKRRRMRSLRRQVLTHDVDRWARAFLDALAHDQVAGSALLSDEEDMYSEPHR

>CORE_REP|Org169_Gene3867#

MLSTMQHEQLSLATLLRYSSTFLGDSTVSTWTGSGVRTMTYREMGDEAGRLANALRGLGIGLGDRVGTFMWNNNEHMVAYIAVPAMGAVLHALNIRLFPEQLTYVSNHAEDRVVIVDGSLVPVFAKVLPSLRTVRHVIVANGEADQLEPPAGVSVHSYIDLIEAQTSVYDFPVLDERSAAAMCYTSGTTGDPKGVVYSHRSNWLHAMQVCASNGLGLTGSDTILAIVPQFHANAWGLPYAALMSGASLLMPDQYLQPNPLLRMMADQRPTFAGAVPTIWGGVLAALATNPQDISYLRTVAVGGAAVPPSMMRIFDEQHGVGILHAWGMTETSPLGSVAHPPAGAEGEEQWAYRCTQGRFPASVQARLVNDDGEVVPNDGKSLGELEVRGPWITGSYYAPDGNLIDEEKFDEGWLRTGDVGRISANGYLTLVDRSKDVIKSGGEWISSVDLENAVMGHPSVAEAAVIGVPDEKWDERPLAVIVLRSDVASDAVETRARELREYLSTKFAKWQLPERWAFVSEIPKTSVGKFDKKKLRAQHAGSDLAVITLI

>CORE_REP|Org13_Gene6328#

MTEYPWLTTLWVLPLAGAVVVLAVPAGRRTVARVTGLVLSLATLAVAIVVAVRFDPGGPQYQLVESHRWIPAFGAGYTLGVDGIALVLLLLTAALVPLLILAGWKDDREAGGGRRVAHIYVALTLIVESMVLISFVSLDILLFYVFFEVMLIPMYFLIGGFGPRTSGGESAAAELALRQQRSRAAVKFLLYNLFGGLIMLAAVIGLYVLTARAHLGGAGGTFDFRAVTAAANSGQLGAGPAVLNALFLGFMFAFAVKAPLWPLHTWLPGAAVSATPASAVLMMAVVDKVGTFGMLRYCLLLFPAASTTYAPMISVLAVIGILYGALLAIGQTDVMRLIAYTSISHFGFIILGIFAMTNQGGSGATLYMVNHGISTAALFLIAGFLVSRRGTRVIAEFGGVQKVAPVLAGTFLIAGLATLSLPGLAPFVSEFLVLAGTFTRYPVAAVFASGALVLAALYVLWMYQRMMTGPVRKGNERLQDLLPRELLVVVPLLAALLVLGAYPKPVLDRINPAVAGTLTTIGKHDPAPTVAPDAATVPAATTPSGGNHR

>CORE_REP|Org33_Gene4362#

MHATPPAWQHGWCMRKSETFDITSTSGANDDADQAEFTSDIDLTDGDVTDENDDYAREGDVLAERAARMKRNGWTAEPTAGELQLEERSSLRRVAGLSTELTDITEVEYRQLRLERVVLVGVWTSGTAAQAEASMAELAALAETAGSQVLEALIQRRDKPDPATYIGSGKADELRAVVLETGADTVICDGELTPAQLTALEKVVKVKVIDRTALILDIFAQHATSSEGKAQVSLAQMEYMLPRLRGWGESMSRQAGGRAGSNGGVGLRGPGETKIETDRRRIRERMAKLRREIREMKTARETKRARRASSGIPQVAIVGYTNAGKSSLMNALTGSGVLVQDALFATLDPTTRRAELDDGREVVFTDTVGFVRHLPTQLVEAFRSTLEEVTGADLLLHVVDGSDPDPAGQIKAVREVIADVIKESGAAAPPELLVVNKLDAISPMRRTELRGLLPDAEFVSAHTGAGVDGLRARLNEVLGGLDVEVGVLLPYTRGDLLARVHADGRILESAHEEGGTRLRARVPHALAAALSEYAHAGAAGEPVGAERT

>CORE_REP|Org118_Gene6962#

MQVAAHSAAGRAPGSATDPQSPRTYEVRTYGCQMNVHDSERLSGLLEDAGYTKATGGQTADLVVFNTCAVRENADNKLYGTLGHLAPIKAERPGMQIAVGGCLAQKDRDTVVRKAPWVDVVFGTHNIGSLPVLLERARHNEQAQVEILESLEAFPSTLPARRESAYAGWVSISVGCNNTCTFCIVPSLRGKEVDRRPGDVLAEVQALVDQGVLEVTLLGQNVNSYGVNFAEPALPQGHPADFAPEHRDRGAFAKLLRACGSIDGLERVRFTSPHPAEFTDDVIEAMAQTPNVCPQLHMPLQSGSDRVLKAMRRSYRKDRYLGIIEKVRAAMPHAAITTDIIVGFPGETEEDFQETLDVVRQARFTSAFTFQYSIRPGTPAATMADQVPKAVVQERYDRLIELQEQISLEANRALIGTEVELLVAEGAGKKNAATARMSGRARDGRLVHFRPGDAAIRPGDIVTVDITEAAPHHLIADGPVHTHRRTRAGDAHERGVLPKTAPIGVGLGLPRIGAPETPPAAAGCETLPAAAGCETPPAAAGCDTGCGA

>CORE_REP|Org113_Gene4117#

MTAQAWILIAAIVAVLLVAFVAGFVLYKRRRVSIAPAAEQDKELTDRSGGYTASGGFSFSQGGAGSGTLTPPRPEPVPIERTDDEGQPHIGDDAAVPRDSARRTITDVRLPEPETLTDQQSGGATATAPVAEPETDGAAAPAEPATEIETPVDSAPADTTPTETALTETAPAETAPTEAAPTDTAPAETALPDTAPAETTPPKAAPDDTIAPAPQQPVTTDGGPATAPADAAPVAEPAIAEIEPTAGRLTKLRGRLSRSQNAVGKSLLGLLGGGDLDEDSWEEIEDTLVMADLGTSVTTTVVERLREELAARSVRTSEQARQVLRDVLVEALRPELDRSIRALPHADHPSILLVVGVNGTGKTTTTGKLARVLVADGRRVLLGAADTFRAAAADQLQTWGERVGADTVRGKEGADPASVAFDAVTAGISEGVDAVLVDTAGRLHTKTGLMDELGKVKRVVEKKAEVDEVLLVLDATVGQNGLTQARVFAEVVDITGVVLTKLDGTAKGGIVFQVQHELGVPVKLVGLGEGADDLAPFEPGAFVDALLG

>CORE_REP|Org138_Gene2201#

MIEMTLREIADVVGGTLHDCPDPEVTVTGAVEFDSRRIGSGDLFLALPGARVDGHDYARQAVAAGAVAVLAARPVGVPAIVVTPPSAAPAARDGDTGADGDAAPEDRRTSRALALAADTDGSGAAVLAALAKLARTSVDRLVAAGGLTVVGVTGSSGKTSTKDLLAAVLSPLGPVVAPPGSFNNELGHPWTALRADADTRFLVLELSARGRGHIRALTEVAPPGIGVVLNVGTAHLGEFGSREAIAETKGELVEALPATGLAVLNADDPLVSAMAARTAARVVQVGQAAGADLRATDVTLDEQARARFTLRRGDESVDVTLAVHGEHQVGNALAAAAVALECGADLATAAAALGAAQAVSERRMDVRTRADGVTVINDSYNANPDSVRAALKALVTMAKSGDTARRSWAVIGEMGELGEESVLEHDRIGRLAVRLDVDRFIVVGAGRPVRALFQGAVQEGSWGEEAVHVPDIAAAVELLDSELAPGDVVLVKASKSVGLWAVAEHLTAAAGPDARPAAGSDSRPAADSKSRPAADSIARPAAAEEAAR

>CORE_REP|Org162_Gene5667#

MGRENADPIPPGVPPVSQAGRPVVLIADKLAQSTVDALGDGVEVRWVDGPNRAELLAAVPEADALLVRSATTVDAEVLEAGKKLQIVARAGVGLDNVDVPAATERGVMVVNAPTSNIHTAAEHAVTLLLAAARQIPAADATLREHTWQRSKFNGVEILGKTVGVIGLGRIGQLFAQRLAAFETKIIAYDPYTSPARAAQLGIELVSLDEVLERADFISIHLPKTPETKGMLNAETIAKTKKGVIIVNAARGGLIDEQALADAITSGHVRAAGIDVFETEPCTDSPLFDLPQVVVTPHLGASTAEAQDRAGTDVAKSVQLALAGEFVPGAVNVTGGSVTDNVAPWLEIVRKQGALLGALADELPVSLEVQVRGELAADDVAVLELSALRGVFSALIEDAVTFVNAPSLAKDRGLEAAVTTHTESPTHRSLVDLRAVFGDGRTLNVAGTLTEPQQVQKIVNINGRNYDMRAEGLNLAVLNYEDRPGQLGRLAGKLGEAGIDILAAQLTQDLDKEGATVVLRVNQEVPAEVQASIAEAVGAAKVAQVDLS

>CORE_REP|Org102_Gene2110#

MPGERSGGEKNDFRRTIGWCSVSTPNPSSSGTADSAASGQAKAAAARAAVEHDVPEQMRIRQEKRERLLAEGREAYPVVVERTHALAEIRTAYPDLAPDTQTGLMVGIVGRVIFMRNTGKLCFATLQEGDGTKLQAMISLNGVGAESLAAWKADVDLGDFVSVHGEVIASRSGELSVMADSWAMAAKALRPLPVAHKEMNEESRVRQRYVDLIVRPEAREMARTRVAAVRALRNALERRGFLEVETPMLQTLHGGAAARPFVTHSNALDLDLYLRIAPELFLKRCVVGGLEKVFEINRNFRNEGADSTHSPEFAMLETYEAYGTYDDSATMMRELIQEVAQEVYGTQVVTLADGTEYDLSGEWTTVEMYPSLSESIGVEVTPETTVEELLALADRVGLEIPEGKGYGHGKLVEELWEHVYGDKLYAPTFVRDFPVETSPLTRQHRSKHGVTEKWDLYVRGFELATGYSELVDPVIQRERFVDQARLAAAGDDEAMRLDEDFLAAMEHGMPPTTGTGMGIDRLLMALTGLGIRETILFPIVRPSAR

>CORE_REP|Org63_Gene992#

MLTFRGAALGALLLAVVGLSATAPVAQAQPAPDGVTITADLKLNREGVLEVVEQVSVPPEGSFRMSLPLRLKVSDDAERIFRVTDVDTEGAGTATVANDQFTIEAEPGESTFRYSVQNTVSGAPGTQVFHWLGVLNTDIASISASLISPSFEMGIVDCKLGPPGNTRPCADVKIEADGVLFLEQTDLHKGDAIDLTLQLPPGTVPNNADVRDSGDAGPFTVTTPVLVAFGVLLVALAGLVAFVLRARRQDAAMDGGSETIDPLLRDGDRVQFTSPDGALPGEAGLLLDGYVDPVDIAATVVDLAVRRYIRITPLSDSDWRITRVNTPDDQLRDYEKAVYHALLPDGSDAVTLTDLRKPGRVDSGPVRTALVADAVARGNFLDRRRPGFAVWLGGALVVAGVAATVALALTSGHALVGVAIALGGVATLLAPKYLPARTARGLELARQIRALQRGLETTRREQIPPIDQETVFSRALPFMVLGARADNWIRAFRDLDPSADAQPGLYWFGGFERDRNLQRFAGHFPFFITALEGLFATAGDPHR

>CORE_REP|Org50_Gene1273#

MPVSEPPESPTAAAPAGADPVSAAGPAMSDPAVGGVSDRPALATAVAASPELAAVVERWNPQGMALLRAVHGSGAAGSVTVIGPADVNTTLLRTELARFEPRVTLSEPVADPNAAETASPAGAAPPAVALILLDAGTTLGADLLGVIHRLRADGTHLLLAMNGIHAYRDWRAVRTRDLELLAEQGAADLDIVPVSARLAAAARTAGDAGLLDRSGLGALHARLTAAAAAGGDRPGAVTTRVLADTRQRVVEQVAALRSGAEPARLRERRAVLLAGRDGGRATAMSTLRGQLHLARVDLMTDIGARVRALHATARAELDRLRTAEIAAYPARLQRAVTELTGAFDHIIDQRLAELSARITGADRDTTPRRRDPAPRVGPDPEPRHRGVEDHLMIALGASAGVGLGRLLVAPFSLVPALDVATVPVTLLLGGGAAAWVVRARGQLAERAHIRQWVADALVNVKAQLEQRVATALVEAETVLADHVVQASTARMVETDRRVAQLEAELRRIAAAQPGQLAACERDIRVLDRWLPPVETNDQLGTQ

>CORE_REP|Org42_Gene4328#

MSGSESAGSRVGHATVTPPTDCVVVIGAGLAGLAAALYLRGSGRSVTVLERADHVGGRVGRYRFDDYEIDSGATVLTLPELIDDALAAVGHDRESVGTPLRIHQLAPSYHARFADGSDIRVFADPDQMAAEVARTCGPDEAGRYRRLRQWLAQIYRAEFGEFMDTNFDSPLDMVRLPRKRAALLELVRLGGFGRLGPRVRHFLRDPRLTRLFTFQALYAGMPPNQALAVYGAIPHMDTSLGVYFPEGGMRAVAETMAEALVAAGGRLELGAEVTGIDYAGRRARRVRLADGTARDCDAVVVTADLGAIERFGLKRRRGLRASPSAVVAHGTIPAGIAERWPIQAHHTIDFGAAWEHTFAEIAAPRGGRLMSDPSLLLTRPALSDPGLYIDRADGRYEPFSLLAPCPNLDSAPLDWAELGPAYLRELLGVLEQRGYHGLATHFRLDHLDTPQTWLDQGMLSGTPFSAAHLFRQTGPFRPRNLPRGSDNVVIAGCGTTPGVGVPTALLSGKLAANRLIGPENPRRGETPRTIGTAPREQAVN

>CORE_REP|Org150_Gene4572#

MSTTTRSETSPIRSRLDTTSQTFGTNREAQLRNLAELDDQLDLARAGGGERYIRRHHERGRLLARERIELLLDRDAHFLELSSLAAWGTEFTTGASVVTGIGVVAGVEVAIIAHDPTVRSGAMNPWSLKKTLRALEIARTNRLPVINLVESGGADLPNQANLFVQAGQIFHDLSDLSARGIPTIALVFGNSTAGGAYVPGMCDHAVLVDQQAKVFLGGPPLVKMATGEDADDEDLGGAAMHSRVSGLADHFAVDEHDAIRIGRRIVSELNWRKQGPGPTLPADPPLYDPEELLGIAPADFRVPFDPREVIARVVDGSRFGEYKPEYGTSLVTGWASIHGFPVGILANANGVLFSEEAEKATEFILLANQTDTPLVFLQNTTGYMVGTTYEQRGIIKDGAKMINAVTNSKVPHFTINMAASFGAGNYGMSGRAYSPRFMFAWVGAKLAVMGAAQLAGVLSIVGKAAAANSGREFDEQADTRRRQEIEAQIAAESHSFFISGKVYDDGVLDPRDTRTVLGIALSAAHSGPVEGRRGYGVFRM

>CORE_REP|Org119_Gene7095#

MLVTYGSPVLVQRLVARPGGDGVEPSWRGRTQSIRRPPAPEPHHLTDARVRLPYSGSDAGGGVPESTRVHPKPGCCADGGSTPFSLVRAETTTGVQQVRSGGILASSRWRPSRVRLAASLAAISALALTGCGTDVDDITVGPGKGWPAAFHDGRNAGTSPVTGAKKIALSWSRPIGGPIAEPVTIGPDGQFFLTTLSRDCMLFSGQMATGRKRFCSKLGPSAISAPSVVDGATNVYVGDDDAVNSYNYLGQPRWRTPIGGTPVSTQFTGDGRLLVVTQSGQVDVLSRQTGERTVPTTQLLGEPDFLEYPNLTRPAAGQGLDDCRTGGPQCPVANISAVDAASGRFFVTVWKPGHPAAALVALRYADNKIQQEWSAELLSGGSGTSPVLSSDGKTLYVGDNSKRLIAVDTADGRTKWVHQLEWAPQGGFSVSDAGLIIPAGDDGYLLALRDTGDAAETVWERKDLALRGTPVQTAGGTGYTTAAIGDGLNLITFDTKTGATIDSDVLPGAQGSTTGTSIGPKGEVLVATRIGELFTFEPER

>CORE_REP|Org210_Gene4354#

MSRRVTDNGRSDALEGGSLRDHGERREGRDGRSRSAAGARHVHTLIVGSGFSGLGLAIRLSRQGRDDYLVLERGNDVGGTWRDNTYPGAACDVPSQLYSYSFALNPNWSRSFSKQPEIQSYIQGVADRHGVRDKHIFDCEMTGARWNEEQARWEVQTSKGAFTADILVSAVGALCEPNLPDIKGINDFRGRIFHSARWDHDADLTGERVAVIGTGASAIQIVPSIAPKVAHLDVYQRTAPWLLPRIDRPYTLPERLAFKYVPGVQKLSRAAIYAARETQVVGLAKFPPAMLALEGLAWLKLRLEVPDAQLREKVTPNFRIGCKRMLISNEYYPALGRDNVDVVTDGIREIRANSIVTADGTEREIDALIVATGFHVTDSPVYETISGRDGRTLTELFDEIGQQGYKGAAIHNFPNMFFLLGPNVGLGHTSMVYMIESQINYIADAIATFDQRGLRTVEVRKDAQDSYNRDLQDRMSNSVWLNGGCASWYLDKHGNNTTLWPDFTFRFRKLTEKFDVAAYDTTRSTDGAAGPDLKVVAAQ

>CORE_REP|Org24_Gene2768#

MERTAHGDNRTAHGGRGQLTVTGAPTTVTTGNPLRDERDSRVPRIAGPCSMVIFGVTGDLSRRKLLPAIYDLANRGLLPPGFALVGFARRDMSDDEFADLVHESIKSSARTTFREEVWQQLREGLRFVQGTFEDDGAFHRLATTLKDLDRDRGTGGNHAFYLAIPPTEFPVVLDQLSKNGLAQPAPGAGDPAPWRRVVIEKPFGHDLDSAQELNALVNRVFPEQTVFRIDHYLGKETVQNILALRFANQLFDPIWNANYVDHVQITMAEDIGLGGRAGYYDGIGAARDVIQNHLLQLLALTAMEEPVSFQPKQLQIEKIKVLSATKLVEPLDETTARGQYTAGWQGSEPVAGLLQEEGFDPDSTTETYAAITLAVETRRWAGVPFYLRTGKRLGRRVTEIAVVFKRAPHLPFDQTMTEELGQNALVIRVQPDEGITMRFGSKVPGSSMEVRDVNMDFSYGEAFTEDSPEAYERLILDVLLGVPSLFPVNEEVELSWRILDPVLERWAADGRPEPYEAGTWGPESADEMLARSGREWRRP

>CORE_REP|Org218_Gene4924#

MTITQDPRASAAVSDNYAAHQALVGELRERLAATALGGPEKARQRHIARGKLLPRQRVDQLLDPGSPFLELSPLAANGMYDDECPGAGVITGIGRVSGRECVIVANDATVKGGTYYPLSVKKHLRAQEVALQNHLPCVYLVDSGGAYLPHQDEVFPDREHFGRIFYNQANMSAKGIAQIAAVMGSCTAGGAYVPAMSDEAVIVRDQGTIFLGGPPLVKAATGEVVSAEELGGGALHSRTSGVTDHLADDDQDALRIVRRIVSTLGPRPESPWEVRTPIEPAAPVEELYEVVPVDLRTPYDVREVITRIVDGDPDGGSGFHEFKAEYGKTLVTGFAHIHGHPVGIVANNGVLFSESAMKGAHFIELCDKRKIPLLFLQNITGFMVGRDYEAGGIAKHGAKMVTAVACARVPKLTVVIGGSYGAGNYSMCGRAYSPRFLWMWPNARISVMGGEQAASVLSTVRGDQLDSSGKPWTEEDQEAFKAPIRDQYERQGNPYYSTARIWDDGVIDPADTRTVLGLALSVCAQAPLEPVSYGVFRM

>CORE_REP|Org152_Gene4600#

MDPAARKPIKRALVSVYDKTGLIELASGLHAAGVELVSTGSTAGKIADAGIPVTKVEDLTGFPETLDGRVKTLHPRVHAGILADTRREEHVDQLVELGVEAFQLVVVNLYPFTQTVASGATVDECVEQIDIGGPSMVRAAAKNHPSVAVVVDTRDYDDVLVSVRDGGFTLARRTELAAKAFQHTATYDVAVASWMTSVAVPAAAPATASEGAASEGAESEGAQRFPEWIGATWTRQSVLRYGENPHQAAALYTDAAGPAGLAQAQQLHGKEMSYNNYTDADAAWRAAYDFEAPAVAIIKHANPCGIAVGADIAEAHRKAHACDPVSAYGGVIAANREVSVEMAEQVAEIFTEVIVAPGYADGAVDVLRRKKNVRILIAEAPRRKGAELRPVSGGVLLQDRDILDAAGDAPANWQLVAGDAASAETLADLEFAWRACRAVKSNAILLAHDGASVGVGMGQVNRVDAVQLAVQRAGDRAKGSVAASDAFFPFSDGPQQLVAAGITAIVQPGGSIRDKDTIDLCREAGVTLYFTGARHFAH

>CORE_REP|Org15_Gene6278#

MSDPRASGVRGRESADSTEQLDTGDRVHVTKSTGARVETAVPSNGNESSPDTYWRRAGRFRHRISRRLSAVPLRVTLALALVSLTGLGLLISGVAVTSAMRNVLMDNVDRQLFGAAHDWAGPDAPPPQRLPGPVGRERPPGLFYVRIEDPSGKVRSLFPTGPSVPDFPADLGKHPRTIGSVGNPDEHWRAERVTTPGGSSWVAIRLSETENIIDRLIGLQVAVGLMVLAVLAIVAQFVIRRSLRPLGEVEKTAAAIASGDLHRRVPVQGTNTEVDRLSQSLNGMLSQIQSAFAATEASEESARRSEARMRRFVADASHELRTPLTTIKGFAELYRQGALADPDMFMDRIERESKRMSLLVEDLLMLARLDAQRPVERRPVDLLALASDAVHNARAVDAAQRPEEPRRPIDLEIRPGTGTLEVRGDEARLRQVLGNLVNNALLHTPPEAAVTVALTPAPDEVVIEVADTGPGLPTEDAERIFERFYRTDTSRSRDSGGTGLGLSIVQALVAAHGGTVSVRSAVGQGTTFAVRLPRSQE

>CORE_REP|Org142_Gene323#

MSAAGRSTNRYVLAIDQGTTSSRAIVYDAAGQLVSVAQREHRQLYPAAGHVEHDAAEVRRNVEGLIGRVITAAGIQARQVVGLGIANQRETTVLWDRHTGRPVRNAIVWQDTRTEDLVARLAQRPDADEVQVRCGLPVLNYFAAPRIRWLLDSDERLRARAERGEVLFGTMDSWLIWNLTGGANGGVHVTDVTNAGRTLLMNLHTLDWDERLLEFFEIPRAMLPEIRSNAEIYGYTAIEPAGIPIAAALGDQQAALFGQTCFAAGEAKCTYGTGSFLLSNTGTAPVRSGHGLLTTVAFRIGDEPAHYALEGSIASTGSLVQWLRDRLGLIESAPEIETLAATVTDNGGCYVVPAFSGLFAPRWHAEARGVIAGLTSYITRGHLARAVLEATAWQTREVVEAMNADTGQSLRELRVDGGMTSNNLLMQQISDALGVPVERPLFAETVSLGAAYAAGLAVGLWPDMEGLRRNRHTAARWLPALTAADRDREYRHWSRAAALSYHWTTETGSAPGDERLRPPYTVRTTASSDAPASDRP

>CORE_REP|Org2_Gene7009#

MLQTGAKVFWWIGPIIVLIVIPVLDWVVGEDGTNPRDEDYELLSNDRYYRWCTYLFLPVQLIGLLIACAMWAGHELSVVDKLGLAATLGFVSGIGINAAHELGHRVENAERWLAKVALAQSFYGHFFVEHNRGHHVRVATPEDPASGRLGETLWEFLPRSVFGSFRSAIELERVRLARKNRRWFSVHNHILQAWSMSVVLFGTLMALFGIGILPWLILQAVIGASLLETVNYIEHYGLLRGKRPNGNYARCSPRDSWNSDRLVTNIFLFHLQRHSDHHANPGRRYQTLRSSMQAPQLPAGYATMILFAVVPPLWRAVMDHRVLEHYEGDVTRANIHPRKRRQLLERYRGWATRTPENAAAQTNAPAQSDAPAQANGAEQTSGTARANGAAQADGADGAARAASVPSANDGAQGAAQSNGAVRGGDGAVPSNFAAHTNGAAQASGAARSNGAGAAQANRGAQADGVVQGSDVRPGGSGAAVDGVASGSGVTSETGVPSGNSGSPCNGAVQRNTVVQRNGSAQRREATVAESGAI

>CORE_REP|Org2_Gene1014#

MTPAPLAVDVQEGSIRMSNPVSGERTAVRPSTGGDDPHKIAMLGLTFDDVLLLPAASDLIPSSVETSSQLTREIRLRTPLVSSAMDTVTEARMAISMARAGGMGVLHRNLAAADQAAQVETVKRSEAGMVTDPVTCRPSDTLAEVDAMCARFRISGLPVVDETGSLVGIITNRDMRFEVDQNRRVEEVMTKAPLITAQEGVTAEAALGLLRRHKIEKLPIVDGNGRLRGLITVKDFVKTDQYPNATKDRDGRLLVGAAVGVGEDAWSRAMTLADAGVDVLIVDTAHGHQAQVLQMVTKVKAEVGDRIQVVGGNVATRAGAAALVEAGADAVKVGVGPGSICTTRVVAGVGAPQITAILEAVAVCKPAGVPVIADGGIQFSGDIAKAIAAGASTVMLGSLLAGTAESPGELILVGGKQFKSYRGMGSLGAMQGRGQAKSFSKDRYFQDDVLAEDKLVPEGIEGRVPFRGPVNQVIHQLVGGLRAAMGYTGSQSIAHLQDAQFVQITAAGLKESHPHDITMTVEAPNYTGRG

>CORE_REP|Org45_Gene1567#

MSASDRTATPGASAPAFYITTAIAYPNGAPHIGHAYEYISSDALARFKRLDGFDVFFMTGTDEHGQKVQQAAKAAGVPEREYAAGNSDVFERMDKALDVSFDRFIRTTDEDHHAASIAIWERMAANGDIYLDTYSGWYSVRDEAFYTEEEITVLDDGTRVSTETRTPVEWTEESNYFFRLSSYQDRLLELYETRPDFIAPATRRNEIVSYVKAGLKDLSISRTTFDWGVPVPGDPEHVMYVWVDALTNYLTGAGFPNTDSAAFQKFWPADLHIIGKDITRFHCVYWPAFLMSAGIELPKRVFVHGFLYNKGEKMSKSVGNVVDPMELVDTYGLDAVRFFLLREISYGQDGSYSHEAIVGRINTDLANEYGNLAQRCLKMVARDFGPVAPTPGEFTEDDRALLDRANGLLEKVRAEFDQQQIHLGLEQLWLMLGETNRYFSAQAPWTLAKAGTPEGTAREGTILYVTMEVLRIVSILVQPVIPGSANKILDLLGQTGRTFADIATPIQPGIALPAPEVVFPKFVEPKA

>CORE_REP|Org16_Gene1684#

MRVAETQRPVLVVDFGAQYAQLIARRVRESSVYSEVVPHTATVEEIADRQPLAVILSGGPSSVYAEGAPQLDPRLFDLDIPVFGICYGFQAMAQALGGTVAHTGTREYGRTELNIDGGVLHGGLPTVQPVWMSHGDAVTDAPAGFEVTGTTAGAPVAAFENRARRLAGVQYHPEVLHSPHGQQVLSRFLHELAGIPASWTPANIADALVEQVREQIGDGHAICGLSGGVDSAVAAALVQRAIGDRLTCVFVDHGLLRAGEREQVQRDFVAATGAKLVTVDAVEKFLGELKGVTDPEEKRKIIGREFIRSFEDAVAEVVKSTGTEDGDGGPAVEYLVQGTLYPDVVESGGGSGTANIKSHHNVGGLPEDLEFELVEPLRLLFKDEVRAVGREVGLPEEIVARQPFPGPGLAIRIIGEVTPDRLETLRQADAIAREELTAAGLDAQIWQCPVVLLADVRSVGVQGDGRTYGHPIVLRPVSSEDAMTADWTRLPYEVLERISTRITNEVAEVNRVVLDVTSKPPGTIEWE

>CORE_REP|Org214_Gene7760#

MGCRTRPVQCDTVSRGRRGHRRRGAHPALAAADHATGRRDPGDPGADGRRGTHLRRRAHRARPGSAGRHRHRPARRRRAHAHRRRAGRPGRLPLRAARHRAPARPRNRPPRRAVHRDQPARRQGHRDRGRTRDRARHTARRTLALLRRSPDGNRRSHSRPARARHRDRGHRHHREDAAEFRRAVAADARLHVGRSGGGALRKREYDESDVRVRPGKSSRPRTKTRPQHNDAEPAMVVSVDRGRWGCVLGGDPGKQIVAMRARELGRTPIVVGDQVDVVGDLSGKPDTLARIVRVTDRRTVLRRTADDTDPFERIVVGNAEQLFIVVALADPPPRTGFVERCMVAAFAGGLRPVLCLTKHDLDAASEFAAAFEDLDLTIVYGGIEDPLEPVLELLHDRLTAFIGHSGVGKSTLVNRLVPDAYRAVGAVSGVGKGKHTSTQSVALPLPAGGWVIDTPGVRSFGLAHITPDDVVAAFSDLAAAIEDCPRGCTHLGPPADPECALDQLPGKERRVAAIRVLLNALNSNENY

>CORE_REP|Org85_Gene6549#

MTQHRDSDSTRGGGRATGGPVYARPGAPDALMSYQSRYDNWIGGQWVAPVKGQYFENPTPVTGENFCEVARSTAEDIELALDAAHAAAPAWGKTSAAERAAILNKIADRIEANLDAIALAEAWDNGKPIRETLAADIPLAVDHFRYFAGAIRAQEGSLSEIDAETVAYHFHEPLGVVGQIIPWNFPILMATWKLAPALAAGNAVVLKPAEQTPASIMFLWSIIGDLLPPGVVNIVNGFGVEAGKPLASSNRIAKIAFTGETTTGRLIMQYASQNLIPVTLELGGKSPNIFFSDVMSADDDFLDKALEGFTMFALNQGEVCTCPSRSLIQADIFDRFLELAALRTKAVRQGDPLDTETMIGAQASNDQLEKVLSYIEIGKGEGAQLVTGGERALLGGDLNGGYYVQPTMFTGRNAMRIFQEEIFGPVVSVTSFTDYDDAISIANDTLYGLGAGVWSRDGGTAYRAGRDIQAGRVWTNTYHQYPAHAAFGGYKQSGVGRENHKMMLDHYQQTKNLLVSYAPKAMGFF

>CORE_REP|Org151_Gene7020#

MVRKVAASVVDTQAKLDELVKILAIAAEPAGEAGIAKREKKSIPSVRQRVHMLLDPGTFIETSALARQPDQKDALYGDGLVTGRGLIGGRPVVVIAHDQTVYGGSVGITSARKFMRALQFAFDNACPVVTINDSGGARIQDAVGSIASFGDISRVLEKLSGYVPQVSIILGKCAAGSVYGPINTDVLIGTRDSYMFVTGPEVIKAVNGEDITAEALGGAKVQAERGTLHHVAETEEQAYEWARQYLSYMPTSCLEQPLIVNPGLEPEITATDRELDTIIPDSDRTGYDMHEILLRIFDDGEFHEIRAAFAPNLITGFARVDGVPVGVIANQPLVLGGSIDAACSDKSTYFIRLCDAFNIPLVFVVDTPGVLPGLEQEANGVIIRGGRVPRAIIEATVPIINLVVRKSYGGAYGMMAARQVGADISFAWPTARIAVIGAESAVDLIGKRQLAAVPEEQRAAAREFMVNHYNETIATPWIAAERGYIDAVIEPSRTRLEIRHALRLLREKPTVKPEFNPRKHAVYPM

>CORE_REP|Org5_Gene6966#

MSERSRATKKHPGRILFVGSGPGDPALLTVRAREVLRRAELAFTDPDVDKGVLAMIGTAVEPGPEGESTVDVRPALGEPAEVAKTLVHEARAGHDVVRLVSGDPMTTDAVIAEVNAVTRSHMAFEVLPGLPSATTVPAYAGIALGSSHTEVDVRGEVDWASVAAAPGPLVLHATSGHLAETASALVENGLAPQTPVAVTVRGTTRQQRTIEATLATLNSAASELVGPLVVTVGKEVEKRTKMSWWESRALYGWTVLVPRTKEQAGEMSEKLVMHGAIPMEVPTIAVEPPRSPAQMERAVKGLVDGRYQWVVFTSTNAVRAVWEKFGEFGLDARAFSGVKIACVGEATADKVRSFGINPELVPSGEQSSEGLLADFPPYDDVFDPVNRVLLPRADIATETLAEGLRDRGWEIDDVTAYRTVRASPPPAETREMIKTGGFDAVLFTSSSTVRNLVGIAGKPHARTIVACIGPKTAETAIEFGLRVDVQPETAQVGPLVDALAEHAAHLRAEGLLPPPRKKSRRSR

>CORE_REP|Org45_Gene2547#

MSARRIPGGAAPTRSRATWKTDPARTGGPAYLRRRRPGAGITEQSARAITLDEAVAAIRSGPQGPEIAAVFDFGGTVVHGFDPPSLTRRLLRRDRDPVAAGLLGSIRGARSEGEYERFLQHTMHAWAGLPEHRLEELGATLFQGTVYGHLYPEAWRLIREHETAGHTLVLVSALTRFQVRPAADELGIPTVLCTAMAAQDGVLTGHVEGKPLWRNGKADAVRRFALAEGIDLTRSWVYADSAADLPLLGVAGRPVAVNPDPRTVLEATEKEWPILHFRPRTTPRPTDYARTVAGFAALLGGALFGVAAKAHTKQRREMADSLLNHAAESTLRGTGVRVRVTGREYARAPRPAVFIFNHQSQFDMVVVAEVLGGGITGIAKKEITRNPIFGPLMRFVEVTFIDRADTAAAKAALAPVVQTLRGGLSIVIAPEGTRSRTPRVGAFKKGAFHIAIQAGVPIIPVVIRNAGEIAWRDSAVVRKGVVDVAVLPPIDVSGWDPAAMDADIERVRQLFVDTLLEWPTGD

>CORE_REP|Org80_Gene4558#

MWWTCSSDICGASSKWKGHRGCCTPFAVSVSCCGRRNERAVVSAADTPGSSDAAARPVGAGPPDRRAAAAGSRRRRSYSLRTRVAGAAAAGAILIITILSVITLQAIERVNVEQTDQQLTLASRLVLIDPVIAVGLVNLIGPNENLALTVRDDGELTATTAIELPDLPTGSHTVTVDGASYRVLTTTENQQAGRTVSLGIPNADAARATAQQQRWVLAGGLVAIAAAAGLGWLFGGRAVRPIVDLTRQVGARSGYRDPEHPPQPVDGSGVLEAEQLADAVNTMLSRVDQAQGETAAALETARDFAAVSAHELRTPLTAMRTDLEVLRTLDLDETQRAEILDDLHRSQGRVEATLSALERLASGDLTHERDHVDTDVGDLCDQAAHDAMRHFPGLTVRIDTDAELVTRGLPAGLRLAVDNALANSVKHGGATEALVSAHRAPDGHIIVSIDDNGRGIPVQERQAVFDRFYRGTQATKGGSGLGLALVAQQAQLHGGQAYFDDGTLGGVRLVLDLPARPARTG

>CORE_REP|Org103_Gene853#

MRQKGDVPDDARPGTLLRTRGGGLLSAAAEQRKEGGAEVRDRDVLDRVPTGLFIGGGWREADDGARFLVEDPATGARLADVADGGPAEMAAALDAAAEAQRSWAATPRRQRSDLLRAAYEEVLRRLDEFALLITLEMGKPLAESRGEVRYGAEFLRWFAEEAVRVGGRWGRSPEGATRMLTMKEPVGPTLMITPWNFPLAMATRKIAPAVAAGCTMVLKPAEQTPLTALAFAELLRTAGLPDGVLNVVTTTRAPETIAPLLRDQRLRKLTFTGSTAVGKRLLAQAADQVLRVSMELGGNAPFLVFEDADLDRAIEGAMVAKMRNVGQACTAANRLLVHEAVAEKFAAGFAAAMAAQKIGRGTEPGVQVGPLIDARARDKVARLVADAAGDGARVLTGGSAVDGPGYFYAPTVLTDIGDHPVAREELFGPVAPIATFGSVEEAVAMANATEYGLASYVFSENLSRVLEVVENLQFGMVGVNQGVVSNAAAPFGGIKHSGFGREGGIEGIEEYLATKYVGIAS

>CORE_REP|Org5_Gene7475#

MRDSYDVVVVGGGHNGLVAAAYLARGGLSVLVLERLDHTGGAAVSERIFPGVDARLSRYSYLVSLLPDRIVRDLDLRFHTLPRSISSFTPVGDTGLLVDTADPQRTRASFRRVTGSDADHVAWQQFYDLTTRAARRLFPTLLGPLPTRTELRRTLGDAEAWEVLFERPLGETIEARFGDDTVRGVVLTDALIGTFTHAHDPSLLQNRCFLYHVIGGGSGEWNVPVGGMGALTDALADAARRTGAHLRTSCVVTGIDTDGRTARVRFETAGRIESVAARHVLVNAAPATLARLLGEPGEPAPEGSQLKINMVLTRLPELRAGIDPAEAFAGTFHIAEGYDQLERAYREAASGRLPTTPPAEIYCHTLTDPSILGPEADGRGLHTLTLFGLHTPASLFDSEPELVRSELIDSTLRQLDSVLTEPIRDCLALDADGNPCIEAKTPPELEAELGLPGGHIFHRDLSFPFRTDDADTVAARWGVHTGHDNIFRCGAGTVRGGGVSGIGGHNAAMAVLETLRLAAPI

>CORE_REP|Org139_Gene2204#

MAIGAPSGGDIGESRVEDFAARGPRRRRILRLPVVTQAFGRLVGDRQATADTIVAAPTPLQPIDLTDDAKVAEVLDLAVRMGEVVLASGTSVNDTTTTVRFIAATYGLARCDVDVTFDAIRIWADRGRSLPPASSMRIVHYRGMDFTRLAAVDRLTRRIRTQVVDPEDARAALDAITSAPHPYPRWMATFGWSLLAASIAVLLGAGALVAVVSFAATAAIDRVNRVLNRYGLPFFFQHMVGGAIAATPAIVLATLAEPLGIDVDPTLIIAAGITVLLSGLQLVGSVQDAITGSPITAAARMLEVVMMTGGIVAGIALALRVGQILGATSPPLDLTPARVFTDLPVKLLAGAMAALAYALACYAERRALVAAALSGAVGTVVFLLVQHAGFGPVVSSGAAATIIGLAGGLMARRALTPPLIVAVAGITPLLPGLSVYRGLYGTLNDELLIGANQLLAALGVGCALAAGVTLGEWFDRTVVRPPVLRRFGNLRRPIVRRRRRPAGLPPYLRKEIPPYLRKQK

>CORE_REP|Org175_Gene5205#

MNPPSDPAAGGGGSPSARPATASHQQDRAQPPAAADPAARTRAHSDADAPPLSQPGAGPEQPDPAIASGAADAVPPSSDPADAGPRRILALALPTLGVLVAEPLYLLFDLAVVGRLGALALAGLAVGGLILAQVSSQLTFLAYGTTARAARRHGAGDERGAVAEGVQASWLAAGIGLVIVAVVQLFAVPIVGAISGGGDIADEALDWVRIALFGVPLILLSMAGNGWMRGVQQTRRPLTYVVAGLALSAVLCPVLVHGLVGAPRLELPGSAVANVAGQAVTAGLFVWALVRERVELRPHPSVMRAQLVLGRDLIARSLAFQACFVSAAAVAARFGAASVAAHQLVLQLWNFLALTLDSLAIAAQTLVGAALGAGNASGARSLARRISGWSEIFALALAAIFAAGAAVIPPLFTDDPAVLDRTGVVWWFFVALIPVAGIVFALDGVLLGAGDAAYLRTTTLGAALLGFLPAIWLSLVFDWGIAGIWSGLVAFMVLRLVAVVWRALSGRWATVGAEVPRVAR

>CORE_REP|Org29_Gene4463#

MARTTSKRQAKSGANETVAPLGSSRRGADEPAPMRPPTPLTRTVSLRWRVTLLAASVVAIAVAVTSIAAYAMVARALYGDVDAQLRARAATMINGDIDSMAFQSLGVATLFSNNIGVGLIYPFSVSSPPSTPEGERTLDSLPVYIPPQPTKPPIGTEEIAVAKGEHTSSLRTYNNQRVLARRMDSGVTLVISQRLEPTREVLDRLAWLLFVVGGCGVLLAAAAGTAVGRTGLRPIARLTAATERVARTDDLTPIPVTGDDELARLTESFNTMLRALAESRDRQRRLVADAGHELRTPLTSLRTNMELLIAAGRPGAPRIPDEDMAELRMDVVAQIEELSTLVGDLVDLAREDAPETVYERVDLGEVAERALERARRRRGSIEFVAALRPWFVYGHEAGLERAILNVLDNAAKWSPAGAQVRVSMAEVGRGLLELSVDDAGPGIPPAERELVFERFYRTTASRSMPGSGLGLAIVKQVVTKHGGTITIDTSERGGALIRIVLPGEAGAPVATAEDEPDP

>CORE_REP|Org19_Gene2296#

MGTETVENRRHRVVVIGSGFGGLFACKHLEHDNVDVVLISKTSTHLFQPLLYQVATGILSTGEIAPATRIVLRKHHNTQVILGEVHDIDLVNKTVTSKLLNQDTVTSFDSLIVATGAQQSYFGNDRFATYAPGMKTIDDALELRARILGSFEEAELAKTQEERDRFLTFVVVGAGPTGVELAGQIAELADRTLVGTFRNIDPRDARVLLVEGAGAVLAPMGPKLGGKAQRRLEKMGVEIQLNAMVTDVDARGVTVKDKDGTERRIESACKVWSAGVQASELGKMLAERSKGTETDRAGRVVVEPDLTIKGYPNVFVVGDLMAVPGVPGQAQGAIQGATYAAKQIKAEVAGKQTPDQRKPFKYFNKGSMATVSRFNAVCQIGKLEFSGFLAWLIWLVLHLYYLIGYRSRTVTVFQWFVAFLGRNRGQMAATEQWVFARLALEAMNGNETDARDVQAEVGNTTPPAAPGEPSAKSAAATPDGEKASGTSESAGSGESTATSKSGASSESTTSGSSQPKAG

>CORE_REP|Org5_Gene6285#

MPQNRSASSPRPRGAGAPLAGGRVPRARGVRPVAGPPVNRPGRILAVAAAAVVFIVTGFGWHSVDSLVSGIERIGNLGLGGGHDGAVDILMVGIDSRTDAHGNPLSDQERAMLHAGDEVGTNTDTIVLIRVPNDGSSATAISVPRDSYVDIPGLGKGKINSAYGATKEAARQKLADQGLSDSQIEEKSTQAGRQALIKSVANLTGITVDHYAEVGLLGFVLLTDAVGGVQVCLNNPVDEPLSGADFPAGEQRLSGPQALSFVRQRHDLPRGDIDRIVRQQVFMASLVNQSLNAKILANPGKLRELSDAVGRTIVLDKGWDVVSFMHQLQDLSGGKVNFETIPVQNLDATTSDGESVVKVDPKAVKSFVAAAVGGKSDEHRDSDAVAPDTVTTDVYNSGSTSGLATQVAQALTGKGFHTGSVANWTGEPVRSSRVLAASTSDAKAKAVAEALGGLTVIADPELSQGAIRVVLADDYSGPGSDAGSLFDLSGTSQTSGAPTPVPPAPPIDAGQNGPKCVN

>CORE_REP|Org190_Gene4366#

MTCRVVLLRHVVDRQEVSVSALDVSRWQFGITTVYHFLFVPLTIGLAPLIAGMQTAWVITGKEHWYRLTKFFGKLFLINFALGVATGIVQEFQFGMNWSEYSRFVGDVFGAPLALEGLVAFFMESTFIGLWIFGWSRLPKLVHLATIWMVAIGVNASAYFIIAANSFMQHPVGARYNPETGRAELTSIVELLTNNTALAAFPHVVAGSFLTAATFVAGIAGWWMVRNARSGDEQKLTEARTMWRPAARASLVVIALSGVALIYTGDVQGKLMFEQQPMKMASAESLCHTATNPDFSVLTVGTHNNCDSVTHVIEVPYVLPWLAEGKFTGVTLDGVVDLQQAYNEKYGVGDYRPNLFVTYWSFRAMIGLAGGSALLAIAGLWVMRRGRVPDQRWFSWLSLLAIPTPFLANSAGWVFTEMGRQPWVVVPNPTGDPNLRLLVQDGVSNHSATTVWVSLITFTIVYGLLAVVWFYLMRRYVIEGPDKAAAPAPAKGPDDTGAPGAKPRTEEPAVEQLSFAY

>CORE_REP|Org4_Gene3225#

MTAYRTRGVMVGYQDRTGMVRELTHFIGGQHVAGTSGRFGDVYDPNLGQVQARVPLASKDEVAAVVANAEAAQRVWAAFNPQKRARVLMKFLTLVQDDMDNLAALLSAEHGKTIADAKGDIQRGLEVIEFATGIPHLLKGEYTESAGTGIDVYSMRQPLGVVAGITPFNFPAMIPLWKAGPALATGNAFVLKPSERDPSVPLRLAELFLEAGLPAGVFNVVNGDKEAVDALLHDPRIKAVGFVGSTPIAQYIYETATANGKRAQCFGGAKNHAIVMPDADLDDVADQLIGAGYGSAGERCMAISVAVPVGQETADRLLAKLTERVHKLNIGRSDDPGADYGPLVGKDGVDRVHNYVQIGIDEGAELVVDGRGVTVPGAEDGYFVGATLFDNVTPEMRIYKEEIFGPVLSVVRAKDYEEGLRLANEHEYGNGVAIFTRDGDTARDFAARVQVGMVGINVPIPVPIAYYTFGGWKRSGFGDLNQHGPDSIRFYTKTKTVTQRWPSGLKESNAFVIPTMD

>CORE_REP|Org12_Gene746#

MTGGNETAEVGTAADPVNHTGQAPAELPELLRRVHMVGIGGAGMSGIARILLARGGEVSGSDAKESRGVLALRARGAQVRIGHDASALDLLEGGPSAVVTTYAAIPKTNPELVEANRRGVPVLMRPTVLAELMRGHHTLLVSGTHGKTSTTSMLVVALQHCGFDPSFAVGGELNEAGTNAHHGTGGYFVAEADESDGSLLQYDPDVAVVTNIESDHLDFFGSDEAYVQVFDDFVARLVPGGLLVVCLDDPGSLALARRVADRVAAGELDIRVAGYGSAEAVGVPVPMQARLVAWEPRDVGGVATVQLGDEPAPRTLRLSVPGRHMALNALAALLAARDAGADVDEILQGLEGFGGVHRRFQFVGRENGVRVFDDYAHHPTEVRAVLGAAAELVRQEAADGARSRQGRVIVVFQPHLYSRTATFAEEFGAALSLADEVVVLDVYGAREKPLPGVNGALVAQAVTKPVHYQPDMSRVGRQAASLARAGDVVITMGAGDVTMLGGQILDGLRVRPSTGR

>CORE_REP|Org42_Gene4664#

MIIDPSGRGPTMRQLLIAGVCGLVVFAVILGFLMARYQGYFVPKVNVTANLTTTGDGLPQDADVKFRGVLVGAVDSVSVAAKGELQQVNIELKPEYVDGIPDNVTARVVPSNLFAVTSVELVYNGQSDGHLEEGSVIEEDTSKGTVALQDTLTTVQNILDQIDPMQFGRVLGTLSYALDGSGRVPGSTIERLDRWLTTVDESIPDLGVLLGDFSASAHALNESAPELMDVLASSVTTARTIADRRSELAALISGASVTADKINHLFAMNPNSGKEVTAGLNATIGALASDPSAITNAIANLNSSTRKLNTVFTWGPQRQMVWNAGLTFTPWQPNTVEDCPRYGDLAGPSCATAPAVADPGVLPEAMRPGRLKSAEGLPILPPLPGMPAIPGVTTQVTAPTAPAPTAAVPNPFAGTPLEGLFPNLVPGAPAPAAPQPPAPAPAAQPGADQPEQGETAPAASTERPGVKPAARPIAYTDDQALTALLGRRASTLEYMMLSTALEGGTVQVTGEEAGR

>CORE_REP|Org10_Gene3434#

MAIARAGLPRLMAMSAPTVELMDYADVVARYEPVLGMEVHVELSTATKMFCGCPTDFGAEPNTQVCPVCLGLPGSLPVVNEKAVESAIRIGLALNCSITPWGRFARKNYFYPDQPKNYQISQYDEPIATDGHLDVVLDDGSVFRVDIERAHMEEDTGKSVHVGGATGRIHGASHSLLDYNRAGVPLIEIVTKPITGAGERAPEVARAYVTALRDLLKSLGVSDVKMEQGSLRCDANVSLMPVGASEFGTRTETKNVNSLKSVEVAVRYEMRRQAAVLAAGGAIVQETRHFHESDGTTSPGRRKETAEDYRYFPEPDLEPIAPSPEWIEELRATIPEYPWLRRARIQQEWGVSDEVMRDVVNAGALDLIIATVEAGAPANEARSWWVAYLSEKAKERGVALDELPITPAQVAEVVKLVESKTVNSKVAKQVVDIVLAGEGEPAAVVEAKGLGMVSDDSALQAEVEKALAANPDIADKIRAGKVQAAGKIVGDVMKATRGQADAARVRELVLAACS

>CORE_REP|Org102_Gene2469#

MVAENTDSMSRDVFATGHPALYRRCMSREPSILIIGAGFAGLGMALELRRHGIGNFTLLEKAAELGGVWRENTYPNAACDVPSPLYSWSFEPKSDWPRRFSHQRDIHEYMRAVADKYRIPEHIEFGVEVTDAEFDERAGVWRVTTADGATRTADILIPAVGQLSRPAMPNLPGIESFTGAAFHSAQWDHSVDLTGKRVACIGTGASAIQYIPAIQPNVEHLTLFQRSAAWILPKFDTEYSALHHALFKYVPPVRLAERFAIWSFFEVLALALTDIPAIKSPVIAIADRHREKQVPDPELRAKLTPDYAAGCKRGLFSNEYFPALAQPNVTVETTAIEAVTPTGIRTADGVEHAVDVIVYGTGFKGTEFLAPMNIYGLGGRKLSDEWAAEGARAYLGMSVPHFPNMFMMYGPNTNVGSGSIIYMLEAQARYIRQAIGYLARRPGSFVSARPNVEQSWDDWLQHRLKDTPWNFCSSWYRNASGRITNNWPGATVLFRWKTRTFEPGDYEVAARA

>CORE_REP|Org5_Gene968#

MAATSVGGECASSGLGGAEEESDLRPGEVGDMAKRPVTAVLAALVLMVGVLTGCTVTTGGHAVSIYDDPFQVAGLPTTSGPSGPRPGVPDAALTAAGGDKGAVDTLALNAIDDIQSYWRGEYHNEFDGDFAPVTKFYSWSAKAPRSQETQFCKDTTYHLVNAAYCRLDNSVGWDRAVLLPMMQDSFGKMAVVMVLAHEYGHAIQTMSHIVGAKDPVIVKEQQADCFAGAFMRHVAEDKAPHFTINTSDGLNNVLAATVAIRDADPEDPESVHGSAFERVTAVQIGFTDGPKGCKAIDMKDIQRRRKNLPQSFGDDANRGELAITKDSLKELSKAMAAVMPIPAEPTYDYHGALMNCSNGADTVPVTYCPATNTIGTDVPALAQRGKANADEQDGFPTRVGGDYNAYVVFVSRYALAVQRNARQQLTGAKTGLRAACLSGVITAKLADPHRGPGQGDIALSPGDLDKAVSGLLSDGLAASDVEGKTVPSGFSRVDAFRAGVLGTQEICEARYT

>CORE_REP|Org184_Gene4905#

MSATAPAWVRFTFVVSTGPDPAQLPAQSDPAQLAPDERRTRLLAAAAVTLGSLGDVLTPLGELFAARGFQLYLVGGSVRDAILGRLGTDLDFTTDARPEQVQQMMRGWADHLWDTGGLAFGTVSAAKDDQQLEITTFRSDSYDRVSRNPEVTFGDTLEGDLVRRDFTVNAMAVKIGADGALEFVDPLGGMDALLAGVLDTPSAPQESFGDDPLRMLRAARFVSQLGFTLHPRVQTAITEMAGEIERITAERVRTELDKLIAGAHPIDGINIMCETGLAQIVLPEVPAMKLEIDEHHQHKDVYWHSLTVLEQAIDQEEGDPDLVLRWAALLHDIGKPDTKRNEPGGGVSFHHHEAVGAKMVRKRMRALKYPKQFTEDVARLVFLHLRFHGYGKGQWTDSAVRRYVTDAGDLLPRLHKLVRADCTTRNKRRAAALRATYDDLEHRIARLQEQEDLDRVRPDLDGNAIMELLDLKPGPDVGKAWKYLKELRLDRGPLTRDEAEAALLEWWKTQQ

>CORE_REP|Org142_Gene5069#

MLDEGRTQFYGGHVPAPLTTRQQVNGYRFLLRRLDHALVRRDVRMLHDPMRSQLRSLLVGAVLGLLVVAGAAILAFIRPQGAIGDAKIVMGKDSGALYVVVADNDGGNTLHPVLNLASARLISGSSESPASVKDDKLADMPRGPLLGIPGAPSALPGSAQGTSSEWSLCDTVELSITGSAASASGVDTAVLAARPDLSERIRRADPDEAVLVRRSDRTYLIYEGKRAQVDPENSAIARALSLSGERPRPAGAGLLGAATPVPPIAVPEIPNAGKPGPGALSDIPVGGVISVAATGRGERAELYVVLADGVQHISDFTADVIRTANSQGMSQIETVPPDALTGIAVLSQLPVDHFPAAAPTILSAEDAPVTCVSWSKTEQSDADAVDGPTDRASAALLVGARLPLPEGAQPVSLATADGSGDRVDQAYLRPSSGEFVHVTGMEPGSPRRGSLFYIADNGIRYGVPDIDTAMVLGLGDAPALAPWAIVGQLVPGPTLASTDALTRHDVLPQSN

>CORE_REP|Org12_Gene2002#

MSDVPFGFSNRDDDPDRDKRDDQAGSGANNPFAFGMGSSGAAGFDPAAFGQMLTSLGQMFSTMSQPGSEASPVNYDVAKRLARQQLGSTVAPITDGAQRAVSDAAHLAELWLDAATTLPAGATKAVAWTPNDWIEQTLPTWKRLCDPVAEQVSGMWSSTLPEEAKQFAGPMLGMIGQMGGLAFGSQLGQALGQLAQEVLTSTDIGLPLGPAGTAALLPSAISEFSKGLERPESEILVYLAAREAAHQRLFAHVPWLRQQVLGAVEDYARGIRMDFSAIEEAAQNIDPMSLADPSKLEELLSQGTFEPQTTPEQKAALERLETLLALIEGWVQVVVAEAVGDRLPGAGALAETLRRRRATGGPAEQTFATLVGLELRPRKLREAATLWQRLTTDAGMSARDAVWAHPDLLPSSEDLDKPSGFIDSVIGAGANVFDDPLAQLAETEARERREGAAAQQERPNKDAEGDTNTAGETNTAGDKKAEGTAAQDDSGSGANPSDAGDERGENEDR

>CORE_REP|Org154_Gene2389#

MAESAQPRLAAHPGGTGRTSVLTSYDPRTGEAVGEYPVQRTAEVARAVRAARAAEKWWGGLGFGGRKRWLLDWKRAIVRRSGELVELICTETGKPEADAAIEVVLAIENLDWAARNAARALGRRSLGRNWLTRNHKATVGYLPLGVVGVLGPWNNPVYTPMGSIAYAMAAGNAVVFKPHELTTGVGVWLAESWRALAPDQPVLQAVTGDDATGLALCRAEVDKVAYAGTEAGAREVIAGCAETMTPVVVERDDKGAMIVHVDAKLDDAAEAAVYGAMANAGQNPSGVQCAYVADSVYDSFLHLVIAQARRLRPGADRRASYGPMIMEAQADVVRRQVRDALARGGRAVVGGLESIREPYIEPIVLAEVPEESLAVTGEAIGPVLVVNRVASMEEAAERVNATGNAVAVSVFTRDVHSIEAFAERLRVGVVTINSATAYTGIPALPYGGVGEYGHGHSHGDEGLREFSRTLSIARKRYRGTVNLTTFDRHPRHLRAATAIFQLRHARRP

>CORE_REP|Org39_Gene5783#

MIDKRQQQRDDPGKTIPAVTRVLIIGAGLSGIGTAIRLRCAGIEDIVVLERATGPGGAWRDNVYPGAHCDIPSVLGSFSLARNPRGSHEYSSGADILAYIHDVIARHGLERRIWYGRTVIGLDFDEAAGTWRVRTSTSGGEEVITARSVVMAVGPLSNTSRPDIVGIDGYRGHKVYSARWDPSLDVTGLTVAVVGTGATAVQVIPELVNRARHVTVFQHTPRWVLPHPQYRVPAWNRSLFEKLPLTKDLTRTAYFWAHEAMRSGVVWPTGLTTALEQVAKLQLRRQIKDKWTRRQLTPNYRANCQQLLVSNAYLPALDRDHCKLLTFPIVRLTERGILTVDGVERCFDTIVFATGFDVPCKIGTPFPIRGRDAHLLREEWAEGACAYKSVHVSGYPNLHFTFGPNSCSGRNSALFFLEAQIDYIVESVRMLERWGLRYLDARKSAQDRFNAGIRQRFSGTTRNSRCASWHPTEDGFNPTIFPGSARQFRAQMDEFTLSDYHAVSLSE

>CORE_REP|Org144_Gene6750#

MSERRERDAALEHLDVVIVGAGLSGIGAAYRLQTECPGKTYAVLEARAALGGTWDLFRYPGIRSDSDMFTLGYPFKPWRDAKSIADGPSILHYINETAAENGIDRHIRYGTKVIAADWSSETTRWTLTLEQRDGAGAVARRELTCGFLYSCAGYYNYDQGYTPEFPGRSSFSGQVVHPQFWPEELDYSGKRVVVIGSGATAVTLVPSMADAAERVTMLQRSPTWISAVPRRDKRADKIRELLPPQLAHRVIRTKNILFSIGFYQYCRRRPESARNLLTRLNLRILGDKQMVAEHFTPSYNPWDQRLCAAPSADFFRAIKKGKAEVVTDHIDAFVPEGIRLKSGRVLPADIVVTATGLQLQAFGGIEPRVDGETVALAEQFVWQGTMVSGVPNFAVCLGYTNASWTLRADLSSRLVCKIINHMDRRDRAAVVPKPDGALVEQPLLELTSGYIQRAIGTFPRQGDRRPWKVRQNYLLDSVFTLHTDLDKTLAATPRSAVRSSDLLATH

>CORE_REP|Org104_Gene5315#

MTPITVSRRTTADGNGRVVLPIVTIGRVQFLPGHQPPYDLTYDDLFLVPNRTDVASRFDVDLSSVDGSGTTIPIVVANMTAVAGRRMAETVARRGGIVVLPQDLPLDAAADTIGYVKSRSLTADTPVSMEPEHSVAEALALMHKRAHGAVVVVEDGKPVGVVTEASCTDVDRFARLREVARTDFVSAPASTSPRALFDLLEAEHAQLAVLTTEDGALAGVMTRTGAVRAGIYQPNVDAEGKLRIAAAVGINGDVAAKAKSLVDSGADLLVIDTAHGHQEKMLEALRAVAGLGLGVPLAAGNVVSAQGTRDLAEAGADIVKVGVGPGAMCTTRMMTGVGRPQFSAVAECAAAAKEVGVHVWADGGVRHPRDVALALAAGASNVMIGSWFAGTYESPGDLRVDRDGNAYKESFGMASKRAVAARTATDSGFDRARKALFEEGISSSRMRLDPERPGVEDLIDHICSGVRSACTYAGARTLPEFHHRAVLGVQSAAGFAEGRPLPSGW

>CORE_REP|Org5_Gene2466#

MSTTDHNPSGATQHMPTTVTSPQVAVNDIGSAEDFLAAIDKTIKYFNDGDIVEGTIVKVDRDEVLLDIGYKTEGVIPSRELSIKHDVDPNEVVSVGDEVEALVLTKEDKEGRLILSKKRAQYERAWGTIEELKEKDEAVKGTVIEVVKGGLILDIGLRGFLPASLVEMRRVRDLQPYVGKEIEAKIIELDKNRNNVVLSRRAWLEQTQSEVRSEFLHQLQKGQVRKGVVSSIVNFGAFVDLGGVDGLVHVSELSWKHIDHPSEVVEVGMEVTVEVLDVDLDRERVSLSLKATQEDPWRQFARTHAIGQIVPGKVTKLVPFGAFVRVEEGIEGLVHISELAERHVEVPDQVVAVGDDAMVKVIDIDLERRRISLSLKQANEDYHAEFDPSKYGMADSYDEQGNYIFPEGFDPETNEWLEGFDKQREEWEGRYAEAERRHKMHTAQMEKMAADAAAEAANGGGAGNYSSESGAQASSSSSSSSESAGGSLASDAQLAALREKLSGNA

>CORE_REP|Org49_Gene3138#

MSTLSELLAEHTDLPGAAVDHLQRVVGDWQLLADLSFADLTLWVGAGPVSDGADVVCVAQCRPTTAPTVHPEDLVGSLAAHDDHHQVFDALISGDIVRVDVDSEATGVYHPHPVHAVREAIPVRVGDDVIAVLGRDTDVQRRKMRSNLEVAYMSCADDLCQMVNDGTFPTLEDRTGSHSSPRAGDGFIRLDTEGTVVYASPNALSAYHRMGLQNDLVGQDLALTTRSLITDPFDAQEVVGDIQAALAGKAGRRMEVEARGATVLLRTLVLRPHGELAGAAVLVRDVTEVKRRDRALLSKDATIREIHHRVKNNLQTVAALLRLQARRTENEEARLALTESVRRVTSIASVHEMLSMSVDEEVDLDEVVDRLLPIMADVATVHTARIKVRRAGSLGVFSAERATPLVMVLTELVQNAIEHAFDAGEDGVVTIRSERSARWLDVIISDDGRGLPDGFSLEGSDRLGLQIVRTLVTAELGGSIGLHPGKDVGTDAVLRVPLGRRSAR

>CORE_REP|Org7_Gene2823#

MTAVADRSLGPELARTQTISTDTDVLVLGLTSSENGPAIVPEDLFGDVLTAEVRAELLDQLGAVGAKGKTEELTRVPAPAGLDGVTSVLAVGLGAAEKIDAEQIRRSAGVAARALSGTELVVTTLSGLDIGAAAEGFYLGAYTFTPFKSDKSAPKPDERPVARVELLVPEPEFGEQELFRAQLIAEAVATARDFVNTPPSHLYPAEFASRAQELAEAAGLQVEVLDEKALEAGGYGGVLGVGKGSSRPPRLVRITYAGGPKKVALVGKGITFDTGGISIKPAQNMDNMTSDMAGAAAVIATTLLAARLSLPVTVTATVPMAENMPSATAQRPGDVLTQYGGITVEVLNTDAEGRLILADAIVRASEDDPDYLIDVATLTGAQMVALGTRTPGVMGTDEFRDRVAAVSRAVGENGWAMPLPAELRADINSKIADLANVAPHRWGGMLSAGLFLKEFVPEGVQWAHLDVAGPAYNTGGPFGYIGKGGTGVPVRTLITVLEEIGAE

>CORE_REP|Org5_Gene928#

MVRNGRAAGWLRVAVAGAVLGAASGAGPAVMVGAGPASAVAPPAIDDGALGQAQAVNAKNGPPDETEKRAICAEPYLTGAVPRDPPLPQRILDLDRAWKFSRGAGQKVAVIDTGVNRHPRLPDLQPGGDFVTAGDGTEDCDGHGTLVAGLIAARPSPEDAFSGVAPEAQILAIRQLSLQYEAKNHRDDDTGKVAAGGYGDVLTMAAAVVRAVDMGATVINISEVSCSPAGSGTADGPLGAAVKYAADRNVVVVAAAGNLDQSACSVQNQTSGWNGVSTVISPAWFSPYVLSVASTDPDGATSPFSIHGPWVGVAAPGRTIISLDSKPGGTGLVDTEHGDEGPLTIDGTSFSAAFVSGLAALVRSRFPDLSAAQVIDRIERTAHNPGAGRDDRVGFGLIDPLAALTAQLPPPADRTGALPRAIAPPAPDPGPDPVPRRVAVIGSIALLALLVIGWAAALPYRRGRPGRGTGDPADGFVGTAETASPERISASSGPAGTDSPGGE

>CORE_REP|Org5_Gene949#

MPSKPTTRWQVSGYRFLVRRMEHALVRRDVRMLHDPMRSQSRAYAVGLVLGIVALAGCGVLALLKPQGSIGDNKILLGKDSGAVYAVIDGVVHPALNLSSARLAVGEPAKAVSIKESELAKKPRGALIGIPGAPSSLNFDGSGKGRAWSICDGLKNDGSQDLSTTVIAGDPSLGSKASRLGEGAALLVQGRDAAYLVYDNQRARVDMNDPKVTEALGIRGKTPRPISPGLLNAIPEVLPIEPPKIVDPGGMPTYSLNNHRIGDVVHVATKDQYYVVLRTGLQSISPLTADIIRNSNTAVSTDPEIDQSQAVQQNVSNELPVQKYPVKAPTIVEAKDQPVACMSWKPVAGASDKTDGSKRATLAVITGYSLPIPDNAQTTPLAQADGSGQNVDAFYSTPGSGFFVQTTGIETDSQRRDSMFFIADTGVRYGIKDANAQKALGMDAEKAKPELAPDQIVGLLAAGPTLGRQEAMVAHDGVAPDPAPAKQLVQSKQDQQAQQQSPN

>CORE_REP|Org5_Gene3985#

MTFELLRAAGVRAPVRAAVREMRNLPRSCRYGGGVHFTNGGSVIEQYPLSGWSRTAPTVARVLRSRDLDVVARAVREAGPRGVIARGLGRSYGDPAQNGGGLVVDMTVFDRVHTIDPDSGVVDVDAGVSLDALMRAALPHGLWVPVLPGTRQVTVGGAIASDIHGKNHHSQGSFGNHVLSMDLLAADGTVRTIGPDGADADLFWATVGGMGLTGIVVRARIRMKHTETAYFIVDCDRTTDLDETMRLLTDGSDEGYEYSVAVPDTISTGAKLGRAGFSRGNLATVDQLPPRLRRDPLHFAAPQLLTVPDIFPSGMVNNLTTRIAGELTYRVFGKQGRGMIQNITQFLHPLDVLGEWNRAYGRRGFLQYQFSMPYGAEDQLADAVRTIARSGHRSFLNVFKRMGPSSRAPLSWPHPGYMLSLDFTLAPGVNEFCADLDRRVLAAGGRLYFAKESRTTPEMIRAMYPRLEEWRTIRDAVDPERTFVSDMARRLRLVDDEPVLAR

>CORE_REP|Org162_Gene2884#

MPADISAPPSRGPAPTGGKTPTVIRLLVLATFVVILNETIMINAIPRLMHDLDVTERAAQWVSTAFMLTMAAVIPVTGWFLQRVSTRQAYAIAMGVFLAGTALSAVAPTFAVLLVGRIIQAGGTAVMMPLLMTTLMTVVPEQDRGRVMGNVTLAISVAPAMGPVISGLVLQAGSWRWLFVLVLPIAGTVTWLGLRRLDNIGEPQTGDIDWLSVAFAAFGFGGLVYGLSKFETDHVAVPALLVAAGLALIAVFAFRQLRLQRSGVPLLDLRILLSGTYTKALVLMSVAFLAMLGSMILLPLYLQNLRHLSPLETGLLVMPGGLAMGLLGPTVGRLFDRFGGRPLVIPGAVGVTVALAGFTQISMSMPYWQLLALHILLMISLAGLFTPVFTLGLGALPPHLYSHGSSMLGTLQQVAAAFGTALVVTVMSARMTQLMETGTEPVTAQLDGMRLAFAVSAALSVLVIVTAILLPSRAPAPEETGEDDASEAETAESAAPLLVKD

>CORE_REP|Org5_Gene2339#

MVEHTPRALRSPGPMLEFLRGRDVLVAGWGVSGRSLIEPLRDIGARPVVTDAGEKAMAEAAELGLDTATGAELLEPDALNRFALVITSPGWRPDSPVLVSAVTEGIPVWGDVEFAWWVDQARLYGPVRKWLVITGTNGKTTTTQMTHAILRAAGLASVACGNIGLPILDALRRTPGPQILAVELSSFQLHWAPSVRPEAGVVLNVAEDHLDWHGGLDAYAAAKARALTGRVGVVGLDDAVAAALARKSKARRTVGFRVGVPADGELGVVDGKLLDRAFTKAAILAEVGDISPPGPAGVADALAAAALTRAIDVAPQFVKEGLAEHKVGPHRAAFVRELSGVGFVDDSKATNPHAARSSILAHPQVIWIAGGLLKGAHIDDLVEEVADRLVAAVVFGKDAAVIAAAMARHAPDVPVVELGSGDDDRMSGELSTASLVAEIDGADAVMARAVRIAAGYAGRGDTVLLAPAAASLDMFADYTHRGRSFVAAVQALDERDIGSQQ

>CORE_REP|Org43_Gene4984#

MADVAVVGSGPNGLAAAVVLASAGLSVEVFEAAATAGGGCSTAELTLPGFHHDVCAGAHPMASASPFFRAFDLAAHGVELLAPPASYAHPLDGGRAGVAWRDLDRTVADLGPDGPAWRSFFEPLVRDWPGVVGVAMSDLRHLPPDLPTAVRFGLRLLEQGSPLWNLRFRGDVAPALLTGVATHAITSPRALPAVGAGVLLGTLAHAAGWVIPRGGSQAIADALIAELERLGGSVHTGHRVDSLDEFGGARAIVLDTSPAELLRLAQDRLPAGYARRLRRFRYGGAACKVDFALSGPVPWAAPDCAQAGTLHLVGSRAEAMAAEGAVASGRHAERPYVLAIQPGVVDDSRAPAGKYTFYTYAHVPHGSDLDVTDAVIAQVERFAPGFRDLILAHNTRTAAELPTHNANYVGGDISAGAMILPQVLFRPAPRWNPYATPLPGVYLCSSATPPGPGVHGMNGLNAARHVLRREFDITTDPLELLGTAVRTGARRLRPLTRSGGV

>CORE_REP|Org36_Gene4556#

MQRIIGIEVEYGISTPTEPTANPILTSTQAVLAYAAAEGVPRAKRTRWDYEVESPLRDARGFDLSRMNGPAPVIDADEVGAANMILTNGARLYVDHAHPEYSAPEVTDPLDAVIWDKAGERVMEAAARHASSVPGAPRLQLYKNNVDGKGASYGTHENYLMNRDTPFNSIIVGLTPFFVSRQVICGSGRVGIGQSGDHAGFQLSQRSDYIEVEVGLETTLKRGIINTRDEPHADADKYRRLHVIIGDANLAEMSTYLKVGTTALVLDLIESGEDLSDLQLARPVTAVHTISHDPTLRATVALADGRELTGLALQRLYHERVAKFVHREGNDDPRVADILDNWAMVLDLLERDPMECANLLDWPAKLRLLEGMRSREGLNWGAPKLHLMDLQYSDVRLDKGLYNRLVARGSMKRLVSEQQVLDAMTNPPTDTRAYFRGECLRRFGADIAAASWDSVIFDLGGDSLVRIPTLEPRRGTKAHVGKLLDGVDTAAELVEQLTT

>CORE_REP|Org127_Gene6933#

MMAPALRGYFTVDEVRAAEAELFERVAAGVPMRRAAYGLANVVAAELRERTGGVAGRSVTLLVGSGDNGGDALWAGAALRRRGAAVTAVLLSPERAHAEGLAALRRAGGRIREAGAGSAAELLGTPDLVVDGIIGISGRGALRPRAAELVAAIEAPIVAVDLPSGVDPNTGAVDGPAVRAAVTVTFGSYKPVHALAAPWCGRIQLVPIGLRLPEPQLAALEPVSIGRDWPVPGPRDDKYTQGVTGIHAGSDTYPGAAVLCTGAAVAATSGMVRYAGTAAAQVLAQFPEVIAAESISGTGRVQSWVFGPGAGTDDAARERLSEVLATDLPVVVDADGLTLLAATPELVTGRGAPTVLTPHAGEFARLTGRELGPDRVAAVRELAEKWQVTVLLKGRSTLVATPGEPTLVNEAGGSWAATAGAGDVLSGVIGALLAAGRVPGWAAAAAARVHALAANLAAHADSDAAAPISSTPLLHHLRAAVRTLRSLADAADGLPADSF

>CORE_REP|Org3_Gene1380#

MTGPDETDGPDFAREAGNAEPEPQHGTGAPLGSGPSPVDLAEMALVEAELDRRWPETKIEPSLTRIATLMDLLGSPQQSYPAIHIAGTNGKTSVTRMIDALLTALHRRTGRITSPHLQLATERISIDNAPITPARYVEVYRELAPYIEMIDQQSAAAGGPAMSKFEVLTGMAYAAFAEAPVDVAVVETGMGGTWDATNVIDGQVAVITPIGLDHTEYLGPDLTAIAREKAGIIKRAPESLIPRDNVAVIAEQDPEAMDVLLRRAVEVDAAVAREGAEFRVLARKIAVGGQQLELQGLGGVYDEIFLPLHGEHQARNAVLALAAVEAFFGAGAQRQLDVDAVRAGFASVTSPGRLERMRSAPTIFIDAAHNPAGAKALAATLTSEFDFRKLVGVVAVLGDKDAAGILEALEPVFDEIVVTTNGSPRALDVDSLTDLAVQRFGDERVVPAYTLPDALETAIAIAEDVADTGEMVSGAGVIVTGSVVTAGAARALFGKEPA

>CORE_REP|Org10_Gene811#

MTVTEHRSPELASRQPADPSALLSPRRAKIVCTLGPAVATDDKVRALVETGMDIARLNFSHGDHPDHQANYDRVRSAAAATGRSVGILADLQGPKIRLGRFADGATVWATGDTVRITVEDCPGDHDRVSTTYIHLADDARPGDRLLVDDGKLALTVTAVDGPDVVCTVVEGGPVSNNKGLSLPGMDISVPALSDKDIDDLEFALRLGADLVALSFVRSAADIDRVHEVMDRVGRRVPVIAKIEKPEAVDNLEEIVLAFDALMVARGDLGVEVPLEQVPLVQKRAVQLARENARPVIVATQMLDSMIDNSRPTRAEASDVANAVLDGADAVMLSGETSVGKYPLETVATMARILAAVEQNSTAVPALTHVPRTKGGVLSFAARDIGERLNAKALVAFTQSGDTVRRLARLHTPLPLLAFTPVPEVRHQLALTWGTEAFLVDRVHTTDDMIRQVDTALLHLDRYQHGDLVVIVAGSPPNTVGSTNLIHVHRIGTDDYTSY

>CORE_REP|Org5_Gene7432#

MTLGHERNRLASEAIRVATAEQLSGPERIPPADVPRATAAQRAYLKVTSQNRGMNGPGFTPTQLAARAAYLLRGNDLGTMTSAAPKLYPHMWSWDAAFVAVGLAPLSVERAVVELDTLLSAQWRNGMIPHIVFANGVDGYFPGPARWECRRLAANAPAGPDTSGITQPPVHAIAVQRILDHSRRHGRTTRAVAEEFLDRRWVDLMRWHRWLAHGRDPKQHGRITLYHGWESGMDNSPRWDRAYANVVVGDLPPYRRADLDVVDPAQRPSNREYDRYLWLVEQMRRCGYDDFQLASAMSFAVEDVFVTAIFALACDVLAGIGEDYRMPNADVRELYEWADYFRAGVIATTDDRTGAARDFDLRADQWIGTETLAMFAPLLCGGLPRTTERSLLRLFEGPRFCGHPDLRYALPPSTSPVSKDFRAREYWRGPVWPVMSWLFSWVFARRGWAERSFMLRAEGLRQAGDGSFAEYYDPFTGDPLGSMQQSWTAAAVLDWLG

>CORE_REP|Org17_Gene3747#

MANQELFRKSTCADAARSSRSGIRAGGSIVEDLPLSIDRDAPRPLSVQVADELRAAATAGRLRGGERLPSSRALAERLGVSRTVVTAAYDQLHAEGWISGRHGSGTYLTAAPASVPEPVAASALSDAVGELLDLAPGAPCVAAIDRAAWRRAWRAASDRLPSARKDRGGEPDYLAMITEHLLRHRGLAPGTDSVVLATSGTSSAVGELAHAVLRRGDTVAVEDPGYLRAVGAFRAAGIEVLPIPLDDKGIRVDRIPADVRAVYCTPAHQFPLGARMPAARRVELIDFARRTGALIIEDDYDGELRYDSAPLPLLASLDPSRVIHLGTTSKILSTTLGVGWLVASAAVTDAVTAHRESTGTCPAPAGQLVFAELARHGDLARHLRRLRREMPPRRALVVAELRARGLDVVGDHAGSHVLVPLESAAVEEAAIAHARDRGVRLDGLARHHADRPHTFGAPIGYAALPSAELRAAIIIAAECLAAASYAGPMRPPFHGWQ

>CORE_REP|Org119_Gene5784#

MSAPAPPSAGAFPSPPGGFAPAPPPSTRRNVELLLLAGAAVITTAALFLVEASQEQSLTWDIAKYGAAYLGLFGVAHLAVRRFAPFADPLLLPIVALLNGLGLVLIHRLDLADQQTAVYNSWSMPSPDANKQILWTGLGMVVFVVLLIALRDYRTLARYSYTLGLVGLVALAMPALLPSRFSEINGSKNWIKVPGFNIQPAEFAKILLIIFFASVLVAKRDLFTAAGRHLLGMEFPRGRDLGPIVVVWIVCVGVLVFEKDLGTSLLIFGTVLVMLYIATERVGWLIIGGALLGLGFVFAYQTFGHVQVRTQTWLHPFDDYNNTGYQISQSLFGLATGGLAGTGLGSGRPNQVPFAKTDFIITTIGEELGLIGLTAVLVLFLVFIVRGLRTALAVRDSFGKLLAAGLAFTIAIQLFVVVGGVTKLIPLTGLTTPFMSYGGSSLLANYALLALLIKVSDAARAPAPARKSVPAAPIADATTELLRKPEGGRPEAGPAT

>CORE_REP|Org210_Gene726#

MPQQTAVVVLAAGAGTRMRSKTPKVLHSLAGRSMLEHALHAANEIDPTALITVIGHDREQVGAAVNSVAAELGREITSAVQEQQLGTGHAVQCALTALPADFAGDLLVTSADVPLLDGHTLSALLDEHRSYQPRSAVTVLTFVPEDPNGYGRIVRDADGGVLEIVEHADATPEQAAINEVNSGVYVFDVAVLRTMISRLTTANAQHELYLTDVLKLAREAGNPVHGARLVDAAKVTGVNDRVQMAQAARTLNRYILERHMRAGVTVIDPATTWVDASVRIGRDAVLRPGVQLLGNTVIGEDAEVGPDSTLTDVLVGEGAKVVRTHGEGATIAAAATIGPFAYLRPGTIVGESGKIGAFVETKNASIGAHSKVPHLTYVGDATIGEYSNIGASSVFVNYDGVKKHHTVVGSHVRTGSDTMFVAPVTVGDGAYSAAGTVLRRNVPPGALAVSGGAQKNIEGWVQRYRPGTAAAQAAAEAIAADDRASQATEQKDGNTE

>CORE_REP|Org118_Gene6959#

MGRTRAGNEPAPTRARPWSRSARGAASAPPASAAPSADAARTPLTTSPPVTDAAAWQSIAESIERHRVSGAATPSPAFEENAVNGVDDPSPPGVTTSAAYAEPEGAGAHRRSSEAVDDTGADRNSRAEGRDVNTRRRAAREILRGRRVAPSGRRSRRRGKAPDIESDGGRTIVDPASDTDNPSAHGVDHEIREPDSGPAWSPTGSRGAGGNARERRRSPGRGAFRDPIAHTVAAEEPLARAGRVPGTLRDAGEFALHAVRKWADPRERELRRRRRVRRRSLRWSAASGVTALGTAGLVAISAPVWAVVVVGGGAAALVTGAAVSTRRYLELRRNPLPPAAFVPRRLPAAQSAARAPIARLVRAERAFHALGRQIEASGRIPADDFADTVATAASGAAALHALAGDIAAMEKALAVVATSHAGAGLRQQSGAMVGRLESGVTEYEQLVAAAGQILAAGASEAPAPADEFGWTMFTLREAADRLDGWAQALTDLADRH

>CORE_REP|Org52_Gene4515#

MTATAAATGRQPGTPVFTHHTRGRWLEHWEPDNPEFWEAGGKRTARKNLAFSVFAENLGFSVWVIWGTVVTSMGAAGFPFLAGLGQGNPVAVSNALLLTSTPTLVGAALRIPYTFAIPRFGGRAFTAFSAAMLLVPTLGLAWFVNQPGTPMWVFMVLAALAGFGGGNFSSSMANISFFFPEGKKGAALGINAAGGNLGVAQTQLVLPLLITLGTHLTAKDPAGYRFGITLSVLVWVPFILIATVGALRYMDSIATAKSDGKSYKLALTNRHTWVMSFLYIGTFGSFIGFSFAFPTLIKANFPSLAGIGWITTLGNLAFLGALVGSFSRPFGGWISDKVGGARITVFVFGGMAVAVAAIMAALELKSFPLYLIAFLVLFVLTGIGNGSTYRMIPSIFSAESKKYAAEHDIDPADAAASAKRQAGAAIGVIGAIGASGGYLLQQALRLSNINFGSMAPAFWAYAAAFLVMAGVTWFYYLRSSFAIGRFTSLAYANV

>CORE_REP|Org106_Gene2979#

MLENPAATTQFPVTQRAFGLAILVLSGLQLMVVLDGTVVIFALPRLQDQMGLSSAGSAWIVTAYGLTFAGLMLLGGRLGDAFGRKRMLIAGVGLFTVASLLCGLAHWQAMLIAARALQGAGAAIAAPVAFALVATTFAPGKARNQAIAIVGSMVGIGSVGGLVVGGALTQLSWRWIFLINVPIGALIILGAIYCLADTGHHRVALDARGAVLGTLACAAIVFGATEGPELGWSHPAVIGALIGGAILLVVFVIAERNVDDPLLPWSLFDSRDRVTTFVLILLAGGVLGAMTYFVAQFLQNVLGYGPLQAGVASIPFTVGIGIGGALASKLAMTVAPRWLLFGAALVLAVGLLFGSTLDGEVSYLPTLLPLLIVIGFGVGVAMVVTPLCVLVGVPPSDIGPLSAVGQMFMNLGTPMAIGILTPVAVSRTLSLGGTTGKVSAMTDAQIVALGEGYTLVLAVCAGVAAVIGLIALTLRFTPEQIARAQHAQEEAQRS

>CORE_REP|Org113_Gene4603#

MTTSELSASPTLTPDVRNGIDYKVADLSLAEFGRKEIRLAEHEMPGLMALRREYAEVQPLKGARISGSLHMTVQTAVLIETLVELGAQVRWASCNIFSTQDHAAAAVVVGPHGTVDEPKGTPVFAWKGESLEEYWWAAEQMLTWDGEPANMILDDGGDATMLVLRGAQFEKAGVVPPEDETHSTEYKVFLNLLRASLEANPGKWTAIAESVKGVTEETTTGVLRLYQFAAAGELAFPAINVNDSVTKSKFDNKYGTRHSLIDGINRGTDVLIGGKKVLICGYGDVGKGCAESLAGQGARVQVTEIDPINALQALMDGFDVVTVDQAIGNADIVITSTGNKDIIGLDDMKAMKDQAILGNIGHFDNEIDMAALESSGATRLNIKPQVDLWTFGESGKSIIVLSEGRLLNLGNATGHPSFVMSNSFSNQVIAQIELWTKPEEYDNEVYRLPKALDEKVARIHVEALGGTLTKLTKDQAEYIGVDVEGPYKPEHYRY

>CORE_REP|Org127_Gene5066#

MSDTLGVRPQPAADITDWDYEADVVIAGYGIAGVAAAIEAARAGADVLVLERTGGWGGAASLSGGFIYLGGGTPLQKALGFEDTPENMEKFMLAALGPGVDEAKIHDYCQGSVEHFNWLVAQGVPFKEEFWGEPGWEPPHDEGLMYSGGENAAPFKDIATPAPRGHLPQMQNKRIGEQGGGYMLMKPLTDAAESLGVRAEYDIRIQRLVVAADNRVVGVVAKRYGKEITVRARRGVVLATGSFAYEQRMIEGYAPRLIGRPAAAIEEHDGIGIRVAQALGAELAHMDATEVAFFGDPQMMVRGILVNGRGQRYIAEDTYPGRIGQATLIQQDNQAYLIIDEAALEEALKTETSSPFFRQPPTWAAETVEELESDMGLPAGSLQATVEVYNRHAESGSDPLLGKKPEWVKPIGTPVAGFDMRNFTAGFTLGGLRTDLDSRVIHVTGEPIPGLYAAGRCTSGVCAGGYASGTSLGDGSFYGRRAGRAAANDGPASS

>CORE_REP|Org138_Gene6221#

MTAKVGDSVNARMDQLDDARGTVRAPDLARVTILAKHTQVDTAIPLDVPVALVIPSVVDMVAQHSRANDFDHDDDEDVEPHEWVLARIGEPPLANSLSLTEQGVRDGELLMLESAEHIAPTPLFDDIMYNVAIADADHYRGWTPGTARLTGSVLAVAATLVGCLGLLAAPDAVPGWVSGAVALFVTVLLVVASTVLARLYEDGGTALVLGGCALPLAATAGMLFVPDHYGWANLLLGAALLGAVAILAWRGAGVSPGLFLGVATVTVYAVPAALVGLLTDLPVRSIGAGAAGLGLAGLALAPRLSMLLAGLPLPPVPAPGTPIDPTEDDPDDHRALPTLEALRAAAEAARRFLTGLVAATTLVTATGALAAADPAGTDVYRPGLALALVCAAVLMFRSRTYAGAAQAVPLVAGGAAIVLLILAGAALRMQQPLLVFGAAMVVLGATLVLGMILPNQQATPPMRRAVELLEYAFVAAVIPLVFWVTQLFALVRGL

>CORE_REP|Org113_Gene2983#

MAEHTAPTAPTTPSSGATIDPGDLAACLRVLDQAATLPDGHADSVAVQRAVAHMFKRFKKRRRTASREEVAAADRAVVAATATGSPNRIDDETAGIPLSANTVGDTAGTLRRARPCYICKQRYTRVDHFYHQLCPDCAARGHAKRNARTDLTGRRALLTGGRAKIGMYIALRLLRDGAHTTITTRFPNDAIRRFTAEPDSAQWLHRLRIVGIDLRDPAQVVALADDVAAQGPLDIIINNAAQTVRRSAGAYAALVDAESAPLPAGELPDTITFGTTTQAHPNALTASLDATSLPATLSAADVTDLALVAGSASLERISQGIAIDAGGLVPDLAHTNSWVQTVAEVDATELLEVQLCNSTAPFILISRLRPALAASPARRKYVVNVSAMEGVFARGYKGPGHPHTNMAKAALNMLTRTSAREMFETDGILMTAVDTGWITDERPHYTKIRLAEEGFHAPLDLVDGAARVYDPIVRGEAGTDLFGCFLKDYEPSNW

>CORE_REP|Org105_Gene2089#

MRPLLSNAVREQTARPRTSRGLCAQPAGVTALADRFSRGQGIRAQRRARARPYGDDPTVARHRPAAFAEVRPGVRIVTMHQLADTGRSCPVARAMPMDTDGPRISLYAAEFAADPHRAYREMRERYGALVPVELAPGVPATLVIGYRTAVRILNDPDHFPADPRVWQKDIPSDCPVLPLLEWRPQALRSTGAEHARYRQAYTEAINGVDLHTLHSTVEKIAIPLINTFCESGSAELISQYAFPLTFAVLNEILGFPAELGQRVATGMQAIFDGIDAERGNAMITEAVMELVADKRVNPGDDIATRLLNHPAALTEEEMIHQLVTLYGAGIEPQQNLIVNTVLLMLTDDRFAGSVLGGSLSTRDALDEVLFTDPPMANFCVSYPRQPILIDGVWLPAHQPVVISMTGCNNDPEVSAGRYLDNRSHLSWSAGPHACPAKSVAYLVVQDAIDQLLDVLPEMRLAVPADQLSWRPGPFHRALAAMLVVFPPSPPLSVR

>CORE_REP|Org5_Gene5433#

MTKKQSSARATNSASSARASGSKARTARSTRATTPASAPSADAAPLLSTEQGGSSLVLAADGSPSDSGVRRTVLPGGLRVVTEHVPGVRSASIGVWVGVGSRDEGPSVAGAAHFLEHLLFKATPTRSALQIAEAVDAVGGELNAFTAKEQTCYYAHVLDDDLPLAVDVVSDVVLNGLCRSVDVDVERQVVLEEISMRDDDPEDLVGDSFLTALFGDHPIGRPVIGTVDSIEQMTAAQLRGFHLRRYRPDRMVVAVAGNIEHEHTVELVYRAFGDRLDPLCAPAPRREGRFRTRSQPRLYRSYRDSEQAHLVFGVRAFGRHEGQKRWPLSILNTVLGGGLSSRLFQRIREERGLAYSVYSSVDTFADTGAFSVYLGCQPENLGEVASLARGVLEEVAADGISDAECARAKGSLRGGLVLGLEDSGSRMNRIGRSELSYGNHRSVSETLARIDAVTTEEVSAVAASLLARPYAAAIAGPYRRGRELPGAVRRLVD

>CORE_REP|Org5_Gene4509#

MPNCAPRSTSRPRGSCVPEPRRWTSALSEVVGTVEGVSEGLFEAPEVESPGEMPGGSSALPPVGRMSPLAVRMRPANLDEVVGQQHLLGPGSPLRRLIDGSGAASVLLYGPPGTGKTTLAALISTATGRRFEALSALSAGVKEVRAVIDLARRRLLAGEQTVLFIDEVHRFSKTQQDALLAAVENRVVLLVGATTENPSFSVVSPLLSRSLVLQLKSLSDNDIREVLRRAIVDERGLGGQYTVSEAALDHIVRIAGGDARRSLTALEASAESSLDGTVDVDLVEASVDRAAVRYDRAGDQHYDVISAFIKSLRGSDVDAALHYLARMISAGEDPRFIARRLMIQASEDVGMADPTALQTAVAAAQVVQLVGMPEAQLALAHATIHIATAPKSGAVAAAVGAALSDISAGKAGAVPPHLRDGHYAGAAALGNAQGYRYPHDHPDGVLAQQYPPDELIGADYYAPTDHGYEREVGPRVTKLRRIVRGRAGAPDR

>CORE_REP|Org114_Gene3042#

MNTPLRRVAMAVMIMVVALLANATYVQVIKADSLRSDPRNVRVLMDEYSRQRGQISAQGTVLASSVATDDRYKYLRTYPTDPAAYAPVTGFYSMQYGSTGLEHAEDSVLNGSDNQLFGRHLVDLVSGRDPRGGNVVTTIDPVMQKVAYEQLTSKGYTGSVVAIEPSTGRILTMVSTPSYDPNQLSSHDGAAVTQTWNDLQQDPRSPMLNRAVSQTYPPGSTFKVVVTAAALSAGVAQPDDQFTAASRITLPDTATTLENYNGNHCGPGDSQTASLTTAFKLSCNTAFVELGIKVGSAKLKDEAAAFGIGQHEGIPIPVADSTVGTIPDGAALGQSSIGQRDVALTPLDNAVIAATIANGGVRMRPYLVDQLQGPDLSVLSTTKPMSVGQAVNAQVASQLTTLMMESEKNTQGGGRSPYTIASKTGTAEHGSDPRNTPPHAWYIAFAPAQNPKIAIAVIVENGGDRALAATGGSVAAPVARAVLDAGLAGG

>CORE_REP|Org103_Gene1246#

MFVGKVLAMEKMYAGGRLRALREQRKLSQSGLAKMLGLSVSYVNQLENDQRPLTVPVLMRLTTTFDLEVNFFAPETDARLLADLQGVFAENPEAGPLTSGELDDLLTRAPAAARLLIHQHRRLRAADDQLDQLTAGIDKPASAPRAAMPYEDVRDYFYDRRNHIPALDLAAEELFDRNGFTLGGLDLQLARLLRDQHDVAVRIRSEDPAKPGPKRIYDPATRTLTLARRLTAGQRAFQLATQLAFITQTEVIEELLAETAALPAESRRLLRIGLASYFAGALILPYGRFLDAAEKLRYDIDLLSAQFEVGFETVCHRLSTLQRRGRRGVPFFFVRTDRAGNISKRQSATAFHFSRVGGSCPLWVVHDAFATPGRIRTQIAQMPDGRTYLWLARTTGERSPGYRAPSRDFAVGLGCDLTFADKLVYSQGLPIHDPSEAVPIGAGCRVCERGDCAQRAFPQIGRPLAADENRPATTPYAPASPAVGDRADQW

>CORE_REP|Org12_Gene4594#

MRCPRSASKTTATIGIVDVVASVRAEGSVTLRSPTPRPARAHPASAPAAAHTPARTTARIPSPPACEPSRAAGAATGPHAVRDLFPALADDGPVYLDSAATTQKPLPVIEAIEGYHRHHTANSGRGTYPWATTLTRAIEGVRADTARFLHADPDEVVFTAGATAGLNAIALAWGLTTLADGDEILYSPRDHASNVYPWLQLRATLAHFGRRLRLVPYRTTALGEADIDDIAAKLGPRTRLLTLSHLHHVYGARNTLEELRDRIDARVAVCFDCSQSAGHIPIDVRELGADFAVLSAHKMFAAPGTGVLFCHRRVHDQLSPFLPGGNSGVSVRDSALLPARMPHRLEGGTHNIPGILALGAALRVLDSIGIDTIERHNRMLTRRLVDGMRALPGLRLLPGPGHAPCDTGYGIVSFTLDGITATDLGFVLAELGFLVRTGAHCVPADTAGENAEVVAAEADSVRVSTHVYTTAEEIDRFLGCLTTIATEVR

>CORE_REP|Org13_Gene4665#

MRSLRSTRSELPESRGWTGTPPRARRHRDLIVAVATLLAVALVAACSSSDKVTATPRPAPVVGDWNGVLPLPNQQLPIGVTFTEDGGSVTVPAQGIFDLPLEQVDTNPDAIAFTIPGLPGDPHFTGRYDRHADTVTGTFTQSGHDLALTMHRGKVPAPLRPQEPHPPWPYLSEDVTYRSDGITIAGTLTRPRGPGPFPAVVLVTGSGPQDRNEEIAGHKPFLLLADTLTRAGYAVLRTDDRGVGGTGGQLDTCSYTDLTDDIMAGLGYLRSRPDIDGNHIGLLGHSEGGYLAPLAATRPDSRLAFVIMMAGPAVSGSDVLLAQNDLLLRSEHADADAIRKQVGYITTLTTLIRTGDDEQIRRFATEHNDSLPPEQRQPQSAIDQLTTPYFQALVDYDPAPALQALRIPVLAFYGTKDMQVPAAQSAPAARGDLAGNPNADVHVFDGLNHLMQPADTGSPKEYPTIETTIAPEVLSYITTWLGKYVTPVP

>CORE_REP|Org128_Gene2505#

MTSTTAASRESDAPQNQALTQEETIASLGTYGYGWADSDIAGASAKRGLSEDVVRDISAKKNEPEWMLDIRLKALRIFDRKPMPNWGSNLEGIDFDNIKYFVRSTEKQAESWEDLPEDIKNTYDKLGIPEAEKQRLVAGVAAQYESEVVYHQIREDLESQGVIFLDTDTGLREHPEIFQQYFGSVIPAGDNKFSALNTAVWSGGSFIYVPPGVHVDIPLQAYFRINTENMGQFERTLIIVDEGAYVHYVEGCTAPIYKSDSLHSAVVEIIVKEGGRCRYTTIQNWSNNVYNLVTKRAKAGAGATMEWIDGNIGSKVTMKYPAVWMTGEHAKGEVLSVAFAGEGQHQDTGAKMLHLAPHTSSTIVSKSVARGGGRASYRGLVQVNKGAHGSKSTVKCDALLVDTVSRSDTYPYVDIREDDVTMGHEATVSKVSEDQLFYLMSRGMTEDEAMAMVVRGFVEPIAKELPMEYALELNRLIELQMEGAVG

>CORE_REP|Org153_Gene2292#

MSKFIDRVVLHVRAGKGGHGCASVHREKFKPLGGPDGGNGGNGGDVILEVDPNVHTLLDFHFHPHAKASNGKPGEGGNRDGKQGTDLLLKVPDGTVVLGADGEVLMDLVGAGNRFIAARGGRGGLGNAALASKARKAPGFALLGEEGEERDIVLELKSVADVGLVGFPSAGKSSLVSVLSAAKPKIADYPFTTLVPNLGVVASGDTTFTVADVPGLIPGASEGRGLGLDFLRHLERCAVLAHVVDCATLEPGRDPVSDADALEAELAAYKPALSADADLGDLADRPRVVILNKTDVPDAAELAEMVTDEFTARGWPVFQISAVSRAGLRPLTFALADMVRKYREEHPKAAPKRPVIRPIAVDETGFSVIADPEEPGGFIVRGTRPERWVRQTQFDNDEAVGYLADRLARLGVEDELVRLGAEPGAPVTIGDVTFEWEPQISAGVDMVPTGRGTDIRLEQTDRVSAAERKHASRVRRGLVRDDEDEA

>CORE_REP|Org75_Gene2079#

MAVSLGKAGHTPAARESGYAAHRAGVDRLLASYRAIPPEATVRLAKKTSNLFRARAANPAPGLDVSGLTRVIAVDPEARTADVAGMTTYEDLVATTLPYGLAPLVVPQLKTITLGGAVTGLGIESTSFRNGLPHESVLEMDVLTGAGEILTVTPDGEHADLFRGFPNSYGTLGYTVRLKIELEPVPPYVALRHVRFHDLRELEAAIAAVVEDRSYDGERVDYLDGVVFTATESYLTLGRQTDEPGPVSDYTGMDIYYRSIQHDSPHPKRDRLTVHDYLWRWDTDWFWCSRAFGTQNPKIRRFWPKRYRRSSFYWKLVALDHKYHIGDKIEARKGNPPRERVVQDIEVPVERTADFVSWFLREIPIEPIWLCPLRLRAEGPAVSGAGAAGTRAWPLYPLERDRTYVNVGFWSAVPTVPGQPEGAANRAIERTVTEFDGHKSLYSDSYYDKDEFAALYGGDSYTELKKRYDPDQRLLDLYSKAVQRK

>CORE_REP|Org198_Gene7470#

MRGHNGTERQRGGIVSAHPAGPRHAEIPHAPSLPERPREPQQMIDLPANVWPRNASRDSDGVVRLAGVPVHELAAEFGTPLFVVDEDDFRSRCRDMVRAFGPNARVHYASKAFLCGEIARWIRDEGLSLDVCSGGELAIALHAGFPAERIALHGNNKSATELEAAVTAGVGHVVVDSLIEIERLEAIAGRAGVVQDVLVRVTVGVEAHTHEYISTAHEDQKFGFSIAGGDAMEALARVFEADNLRLVGLHSHIGSQIFEIDGFEIAARRMLRLLHDAIEKFGVERTAQISTLDLGGGLGISYLPNDDPPPLDDFAAKVRDLVAAEAASIGLPEPKIAVEPGRAIAGPGTVTLYEVGTTKDVSLDGGLRRRYISVDGGMSDNIRPALYQADYDCRLVSRTSEAAAVVARVVGKHCESGDIVIRDTWMPEDVGPGDLVAVAATGAYCYSMSSRYNQLTRPAVVAVRDGQPRLILRRETVADLLSLEV

>CORE_REP|Org81_Gene2965#

MKVTCSSPTTAIVSGPPTGLPRRVLSCAGGHRGEPCVRSRGEPCVRSLGGLCFGSGGESGFAASRESGVAAGDVSGATVSRGSGGAVRREFDAAGDRAAVERSAESVGVDRRGGPVGVRPADRHTADVPAADPARAATEPSGSVDHQRLRHHGDVDARPGMVDFAVNVQGTAPPEWLRQRLAGRLEELGRYPDSGEESATRAAVAARHGRRAEEVLLLAGAAEGFAMLPRLGARRAAVIHPSFTEPELALREAGVPVTRVVLEPPYTLDAGLVPEQADLVVVGNPTNPTSVLHPAEALLALRRPGRIVVVDEAFADAVPGEPETLSGIDLPDVLVLRSLTKTWALAGLRCGYALGAPQVLARLNQGRPHWPLGSLQLEAIAATSAPAAVVETRRKAEVIAADRAAMIPRLRTLGIDVHEPAAGPFLLIRVPDAELLRKRLADKGIAVRRGDTFPGLAAGFLRVAVRPPAEVDRLVAAIEDVGL

>CORE_REP|Org31_Gene5564#

MAKQVPTSRLARGTKLGAVAASSVIRTQRARLSMRGRSEAVRAKMAEESMIRTTEQVVMVLGTMKGVAMKLGQMMSVLDLDLVPDAHRERFQKRLAVLRNAAPSVSFESMRQVIEDDFGQPLDAVFAEFEAEPVAAASIGQVYRARLRDGRQVAVKVQYPGIDAAVRADLKNLAMFRRVLQSAMPWVTPAVLDELRLNMESELDYQAEANTQLQIAELYAGHPFIVVPRSLPELSTTRVLVTEYVAGKGFEEIRQLPDAERDRIGEIIYRFYVGSLFTFNEFCGDPHPGNVLLAEDGRVGFLDFGLFNRMDPGHVQFELTCLRAAAEDRAEDLRELMIERGVIDSPEEIGAEECLEYVLAASEWCLIDEELTITPELASGAFLLAVDPRASEFAGMKQQNLPPEHLFSRRADFLTFGMLGQLGCTANWHRISREWLYNEPPVTELGRAHHVWLAEHPPVAPKKSRAKSSKAGKSTKAARPQA

>CORE_REP|Org152_Gene795#

MSVLLVGISHRSAPVAVLEKVAITEDDRPKLTDKMLASSHVSEAMIVSTCNRVEIYAVVDAFHGGLGEISDMLTRHSGLPLPELTKHAYVRYAEAAAEHLFAVASGLDSMVVGEQQVLSQIRAAYAASDAQQAAGRTLHELAQHALRVGKRVHSETGIDRAGASVVSVALDRARAVLGAAELSREAAASVSENAVAPYDSALIGRTALVLGAGAMGGLAVAQLARAGVGRIIVVNRTLERAQRLARTATDMHGVTADAMEMSRMVEAMAVADVVVTCTGAVGAVVTLADVHRALAGRPHQHLVICDLGLPRDVEHAVAGLPGVTVIDMETLQRDPSAGAAADDTVAARGIVADELAKYLAGQRMAEVTPTVAALRQRAAEVVEAELLRLDSRLPSLAADDREEVARTVRRVVDKLLHAPTVRVKQLASTPGGDSYAEALRELFELKPGAAQAVAAPMEIAALGELADDFTAAHLGDEQGPNS

>CORE_REP|Org5_Gene6276#

MTVARSAESVHAVVKAYDVRGVVGEQIDAAFVRDVGAAFARLMRDSATRIAIGHDMRESSPELAAAFADGVLDQGLDVVHIGLASTDQLYFASGHLQCPGAMFTASHNPARYNGIKLCKANALPVGQETGLATIADELIEGVPAGPGPRGTATEQNLLEAYAEFLRGLVDLSGIRPLKVAVDAGNGMGGYTVPAVLGAVSQLTIEPLYFELDGSFPNHEANPLDPKNLVDLQKFVRETGADIGLAFDGDADRCFVVDERGEPVSPSAVTALVAERELAKEPGATIIHNLITSQSVPELVTELGGTPVRTRVGHSFIKQQMASTGAIFGGEHSAHYYFRDFWGADSGMLAALHVLAALGGSDGPEGGSLRNHEGPRGRRIEHSEKDDRTMSELSSSYSTYAASGEINSTVADAKDRTLAVVTAFEGRARSVDRLDGVTVRLPGNAWFNLRASNTEPLLRLNVEARSQEEVDALVTEILSIVRG

>CORE_REP|Org63_Gene113#

MDSVSQRLDLRPNRIAVLSVHTSPLAQPGTGDAGGMNVYVLQTAVELARRGTEVEIFTRATASNLPPVQEAAPGVLVRNVVAGPFEGLDKHDLPTQLCPFTAEVLRQEARHLPGYYDLVHSHYWLSGQVGWLARDRWRVPLVHTAHTLAAVKNAALAEGDCPEPATREIGEKQVIAESDRLVANTAEEARQLVELYGADPERIDVVPPGADLTLYRPGDKAAARAALGLSADEQIVAFVGRIQPLKAPDVLVRAAAEVLRADPERPLRVLIVGGPSGSGLDRPDALIELAAELGIAARVSFLPPQPPQRLVLVYRAADLVAVPSYNESFGLVAIEAQASGTPVLAADVGGLGTAVRHDVSGLLVPGHRTSDWANALRHLLDDPGRLHRMGERAVAHAANFSWAHTADGLLASYAAALAGFRDERSALGGRGLAHSLVRDDAYDRAAADRTNLAGERTAALLAESSQARSRALWRRRMGVRR

>CORE_REP|Org13_Gene5367#

MTIMAPAQADATTDPRDPLGRLQRFFDPGTVLPLHPRDKSGVLAAIGEVDGVRTVAYCSDATVMGGAMGVDGCKHIVDAIDTAIDSRIPVVGIWHSGGARLAEGVEALHAVGTVFEAMVRASGLVPQISVVVGFAAGGAAYGPALTDVVIMAPEGRVFVTGPDVVKSVTGENVDMATLGGPETHGKKSGVCHIVADDENDAMHRGRRLVSMFAEQGEFDLSAAAHGDVDLKAMLPESAKRAYDVKPVVRELLDKIPSPDGNDESSFEEMQGGYARSIVTGLGRLGGRTVGVLANNPLRMGGCLTSESAEKAARFVRLCNSFGIPLVVVTDVPGYLPGVGMEWEGVVRRGAKLLHAFAEARVPRVTLVTRKIYGGAYIAMNARSLGATAVYAWPESEVAVMGAKAAVGILHKKAIAKAPEEEREALIERLTAEHETIAGGVGRALSLGVVDEVIDPAKTRSTIAAALASAPAQPSHNKNIPL

>CORE_REP|Org39_Gene2740#

MKLTRFVKIQLVIFSVLTVIGITVMSGTYVQLPAMFGIGRYDVTVRLAATGGLYENANVTYRGTTVGKVTAVRLTPEGVDAELSIDSDYKIPSDVDAWVRSVSAVGEQYVDLLPVDNGTGGNLSDGSVIPVERTKLPQDVGTLLEQTDRLLNSVADTKLRQVIDDAFLAFDGAGPDLQRFLDSASLLVQEARNNSEATKALIDQIGPLLDTQIESDAAIRSWTADLATLTDQLREHDPALRNVLSKTPGAAGSATRLFQDLNPTLPLLARNLASVGQVSYVYDPGIEQLLVVFPPLVGALLTIALNGPLEYGAMVDFMVSVNDPQGCTTGFLPADQRRSAALRDVPDTPGGLYCKVSQDAPFAVRGIRNTPCMEVPGKRAPTPELCRDPQGFVPLGDNPPFGPPNPVAPSGDTGGEQPQGGAPGAPAGATQPASVETRSYDPRTGTVLGPDGRMYRQANIGPDGSGVIPTSLTAMVQEQMR

>CORE_REP|Org127_Gene3048#

MSPSVTPSSNSDTLAGDGIWTDETDWEIADFEGDADAHEHVPMPTVAVVGRPNVGKSTLVNRILGRREAVVEDIPGVTRDRVSYEASWAGRRFLVQDTGGWEPDAKGLQQAVARQAELAMQTADAILLVVDATVGATATDEAAVKALRRSKTPVILVANKVDGEKAEADAAVLWSLGLGEPRMVSAAHGRGTGDLLDDVLAVLPETPREGSGGGGPRRVALVGKPNVGKSSLLNKLSGDERSVVHDVAGTTVDPVDSLVELGGKTWRFVDTAGLRRKVGTADGTEFYASLRTKAAIEAAEVAIMLIDASEPITEQDLRVIGMVADAGRALVLAFNKWDLVDEDRRYQLEREVERELVRVPWAQRVNISAHTGRAVQKLVPAMETALESWDQRIPTGRLNTWLKEVIAATPPPMRGGRLPRVLFATQATTRPPTFVLFTTAFLEAGYRRFLERRLREEFGFDGSPVRISVRVREKRDRSKK

>CORE_REP|Org19_Gene6498#

MGRPEEAAECYRGGRPIGLQTTLPNGWQTQADQAAPVHRYAEPVNDRSRVADPDELPATTSSARSTGGLTTRQRRLLLIAIALFVVSAIVSWAAHIWTGYIDLQVYRNGARTWLDGGDLYGPMPKVYGIGLPFTYPPLAALFFAPLALMPLAVAQWLVLLTSMASLAVTLWLVLVRIRPEMDRSTRVILLIGALAVLGLSEPVRQTYNFGQINLILMAAVALDALVRKPFWPRGMLIGITVAVKLIPAGYLLYFLLRRDWRACLTLIGSAIGAIALAYLLFPHDSTEYWFHTLIDTGRIGPPQYAGNQSLKGFAFRLGVSDGAATAIWIGLSLIAIGLAALWMKRLLDAGHQVSALLVNSAAVLLVSPVSWSHHWVWVAPALLVAGDLIARMPAADAPDTPGRAARRRRMWIAITAVITVLFMVGPQWVLPHNADRELRWAWWQQIIGSSYVLVTFAALVIAAIAYRPSGVRNAASAAE

>CORE_REP|Org169_Gene5623#

MFVSLVPNVLATRYASPQLVQLWSPENKIVLERRLWLEVLRAQTELGAAGTEAVTPEVLEDYERVLGEVDLASIAERERITRHDVKARIEEFNALAGHEQIHKGMTSRDLTENVEQLQIRLSLEHVYEHGVAVAARLAERAAEYQSLVMAGRSHNVAAQATTLGKRFAGAADEVLIALHRVRELIDRYPLRGIKGPMGTAQDMLDLFDGDAAKLAQLEQKVAGHLGFATVLTSVGQVYPRSLDHDVISALVQLGAGPSSFAHTVRLMAGHELVTEGFQPGQVGSSAMPHKMNTRSCERVNGLQVVLRGYGSMAAELAGAQWNEGDVFCSVVRRVALPDAFFAIDGMMETFLTVLGEFGAYPAVIERELNRYLPFLATTRILMAAVRAGVGRESAHEVIKEHAVAVALAMREQGREPDLLDRLAADDRMPLDRAGLEAALADRTAFIGAAEAQVGDVVAQVQKLIDANPEAARYTPSPIL

>CORE_REP|Org102_Gene1165#

MSSKPTSDDAAERDTGAPHASDPDATLVGGTGNEVSHEPGTALPDPTAEHRPEAPGGDHATEILGTGETAHNARPAHAPDAPGADDTTRAFGDRESVSDSAPTEVLGANPTAGNNDTTTAFPGRVSVSDSAPTEVLGNSPDAAYPQHAEMRGAQENPADHRTEVLGPTGGPNPSGHQDTAALGAAGDPYPSDRQDTTALGPYGTETFDAAALAAGAAPGGEQQPPTTPPPPVGPNAAGSGGGSSWMRGRTLLVVALVVSLLAIAALAGGEAYARRTVENCITSQFEQEMGSRIDVSFGAKPMLITMFDGKVSSVTVNSDDTKFGPAVGMVVHAKFNDIEVVDNGRGGGTIGSSSADVTWSNDGIAKTLGGLVSGVQSNPTKDTLTFAVLGGLAGLEVKPQVVGGKVEVTTEAASLLGFGLPTDLVEGIVDLMAESLQSYPMGLRPTEVQVTDSGLRVLLAGGKTELPAAQGDSSDFRC

>CORE_REP|Org110_Gene7274#

MHTFDNVTYLVNGLYIIAFGMFIYGLMGLTGPKTAVRGNLIAAVGMFIAVVATLISVRHTSNWILIVAGLVVGIALGVPPAQFTKMTAMPQLVAAFNGVGGGTVALIAWSEFINSQGFSHFDEEPTVHIVIGSLFAAIIGSVSFWGSLIAFGKLQEILPGRPIGLGKLQQPLNLLLLLGAIAAAVVIGVGATNDGVSQLWMIAVLVLAGVLGLAVVLPIGGADMPVVISLLNALTGLSAAAAGLALNNTAMIVAGMIVGASGTILTNLMAKAMNRSIPAIVAGGFGGGGTAPGAGGDGENKQAKATSAADAAIQMAYANQVIVVPGYGMAVAQAQHAVKEMAALLEAKGVEVKYAIHPVAGRMPGHMNVLLAEAEVSYDALKEMDDINGEFGRTDVALVIGANDVTNPAAREDASSPIYGMPVLNVDQAKSVIVLKRSMNSGFAGIDNPLFYADHTSMLFGDAKKSVGAVTEELKAL

>CORE_REP|Org85_Gene7073#

MTLRLFDTDTRTTREFAPLVPGRASVYLCGATVQGEPHIGHVRSGVAFDVLRRWLLAHDYDVWFIRNVTDIEDKILHKAAEAGRPWWEWAATYERAFDNAYETLGVLPPSIEPRATGHITQMVDLMQRLIERGHAYASAGNVYFDVRSYPEYGSLSGHRLDDVHQGESAGEGKRDPRDFTLWKAAKPGEPTWPSPWGPGRPGWHLECSAMAEFYLGPEFDIHCGGMDLVFPHHENEIAQSKAAGDGFANYWLHNGWVTLGGEKMSKSLGNVLSVPNVLKQVRAVELRFYLGSAHYRSMLEYSDKALHDAAQTYQRIEAFVHRTADRAGDIPVGKWTDAFAAAIDDDLAVPKALAEIHRVVHEGNKALESGAVDSARDLAGQLRAMLGILGVDPLDPHWFTPSDSSAAIGALDVLVRAELDRRQQARAAKDWASADAARDRLQAAGIEVTDTPNGPEWALAAAQHLPPDAQQPGKAD

>CORE_REP|Org56_Gene2351#

MAERPRTLAEKVWDQHVVVRGAGEGAQREPDLIYIDLHLVHEVTSPQAFDGLRAAGRPVRRPDLTIATEDHNVPTVDIDKPIADPISRTQVETLRRNCEEFGVRLYPMGDIEQGIVHVVGPQLGLTQPGMTVVCGDSHTSTHGAFGALAMGIGTSEVEHVMATQTLSLRPFKTMAINIDGELPPGVTSKDVILAVIAKIGTGGGQGYVLEYRGEAVRAMSMEARMTMCNMSIEAGARAGMVAPDEVTYEFLKGREHAPTGADWDAAVAAWEALKTDPDAGFDAEVHLDASTLTPFVTWGTNPGQGAPLGDVVPNPEDFADENERAAAEKALTYMDLEPGTPLREVPVDTVFVGSCTNGRIEDLRAVADVLKGRKVADSVRMLIVPGSMRVRAQAESEGLGEIFTAAGAEWRQPGCSMCLGMNPDQLSPGQRCASTSNRNFEGRQGKGGRTHLVSPQVAAATAVRGRLSAPADLN

>CORE_REP|Org5_Gene7017#

MAEPIFQTRMGIMEQSGGRTPVVADAAANLESGPEVGNPTRPPARDATASSFAELMDESGKRRDLRKMKIVATGFLLAATVVYLICAWLGSRGLGGGWVGYVRAASEAGMVGALADWFAVTALFRHPLGLPIPHTAIIRKKKDQLGTSLGDFVRTNFLSPDTVVAKVESAQISLRLGTWMADPGHAARVSEETSTILRAVIGALRDADVEQVIDQTIVKRIAEPQWGPPIGRVLTELLAENRQQPLVDLLAERAHQWALGSQETLDRIVMRDSPSWAPKFANILLSEKIYRELVEFTWKIRSQPDHEVRLAANRFLEDFARDLQYDEAMIAKAERVKAEIMGREEITGMAHATWRAAKRMILESADDPGSTLRRKITENVQQLGQRLVDEPELRKQVDAWVERGVRYLVANYGSEIATLISDTVARWDADEASRKIELQAGRDLQFIRINGTVVGSLAGLAIYAVSHLLFPGF

>CORE_REP|Org154_Gene4464#

MRCCCPHRPAGARHGCNVEDVQRRIMGIETEFGVTCTFHGHRRLSPDEVARYLFRRVVSWGRSSNVFLRNGARLYLDVGSHPEYATAECDSLHQLVTHDRAGERVLEELLIDAEQRLAEEGIGGDIYLFKNNTDSAGNSYGCHENFLVVRAGEFSRISDVLLPFLVTRQLICGAGKVLQTPKAATFCLSQRAEHIWEGVSSATTRSRPIINTRDEPHADAEKYRRLHVIVGDSNMSETTTMLKVGTAALVLEMIEAGVAFRDFALDNPIRAIREVSHDLTGRRPVRLAGGRQASALDIQREYYARAVEHLRNRDRDPQIDQVVDLWGRALDAVEAQDFAKVDTEIDWVIKRKLFQRYQDRYDMELSDPKIAQLDLAYHDIKRGRGVFDLLQRKGLAKRITEDEAVDAAVDTPPQTTRAKLRGDFITAAQEAGRDFTVDWVHLKLNDQAQRTVLCKDPFRSVDERVDRLIASM

>CORE_REP|Org112_Gene2784#

MVATNTRETAESADAADSADTTAARPVKKAAAKKAPAKKAAAKKTAAKKTAAKKTAKATKATKAAKKAAPKKAGEGADGAETENLDDESLEIDDLGDLEVDEEDLGDEELEVEDDEAEDEAEEAEAETEEEADEPTAKDKASGDFVWDEEESEALRQARKDAELTASADSVRAYLKQIGKVALLNAEEEVELAKRIEAGLYATEKIREYADKGEKLNVQLRRDLNWIMRDGNRAKNHLLEANLRLVVSLAKRYTGRGMAFLDLIQEGNLGLIRAVEKFDYTKGYKFSTYATWWIRQAITRAMADQARTIRIPVHMVEVINKLGRIQRELLQDLGREPTPEELAKEMDITPEKVLEIQQYAREPISLDQTIGDEGDSQLGDFIEDSEAVVAVDAVSFTLLQDQLQSVLETLSEREAGVVRLRFGLTDGQPRTLDEIGQVYGVTRERIRQIESKTMSKLRHPSRSQVLRDYLD

>CORE_REP|Org101_Gene365#

MVGNMADETQYRIEHDTMGEVRVPVDALWRAQTQRAVENFPISGRGLERAQIRALGLLKGACAKVNKDLGLLDPAKADAIIAAANEIAAGAHDDQFPIDVFQTGSGTSSNMNANEVIASIAKANGVTVHPNDDVNMSQSSNDTFPTAVHLAATEAVITDLVPALEHLRLALLDKSTEWRTVVKSGRTHLMDAVPVTLGQEFGGYTRQIAASIDRVMATLPRLGELPIGGTAVGSGLNAPDGFGGKVVAELVRATGIDALREARDHFEAQAARDGLVEASGAVRTVAVSLTKVANDIRWMGSGPLTGLGELQLPDLQPGSSIMPGKVNPVLPEAVTQVAAQVIGNDAAVAFGGANGAFELNVYIPVMARNLLESIRLLANVSRLFADKCVHGLVANVEHLRTLAESSPSIVTPLNSAIGYEEAAAVAKEALKNKKTIRQTVIDRGLLDEKLTEAELDRRLDVLSMAKVKDGK

>CORE_REP|Org6_Gene4755#

MPGMRHDRLPDGFGVRIDPRVRAYSGNRILIGGTPARVLRLAPEAAEMIGDGYLEVTGPKSAVVARRLLDSGVANPRPRLLPSTDDVTVVVPLHNNPEGLARMLAVLRGHHVIVVDDGSDQPVRIPETRGTRCRVTVLRHDTAHGPAAARNAGLRAATTEFVAFLDSDVVPRSGWLEVMLGHFSDPEVALVAPRIVALDAESNALARYEHTRSSLDLGRREAAVHSRGPVSYVPSAAMLVRRQALLAVGGFDESMRVAEDVDLCWRLERAGRRLRYEPAAHVAHDHRVAFRAWFGRKMFYGTGAAPLARRHGPVAVSPLSLPYWTALAAVLFATLTRWGLLGGLVALATALVRLRRVFAGLDNPTRIAALYLARGFFAGLWRIASAMCRHYWPITLLAVLVSRRVRRIAVTMAVADGLADWFTHRDAGGLDPVRYLVYKRLDDLAYGTGLWVGAARARSLDALRPAFSRR

>CORE_REP|Org56_Gene2410#

MRRNKVAYPLLGVPDTGDTAWMLASSALVLLMTPGLAFFYGGMVRSKNVLNMIMMSISAMGIVGVLWSLYGFSEAFGDNKLGLIGNPAQFFGLKGLIGTNAVKASPADPSTGASAVDQVNIPLAGTIPMTVFVAFQLMFAIITVALISGAVADRMKFRAWVVFAIAWSTIVYFPVAHWVFDFDVKDAAGNIVHHGGWIANKLQAIDFAGGTAVHINAGAAGLALCLVLGKRKGWPKTPMRPHNLPFVMLGAGLLWFGWFGFNAGSSVSSNGLAGSTFLTTTFATCAAMLGWLVVEKFRDGKPTSLGAASGIVAGLVAITPSCSSVNVLGALVIGAVAGVLCALAVGLKFKLGFDDSLDVVGVHLVGGVVGTLLIGLFLAPESGAASGGAKGLFYGGGFDQLGKQAVGAFTVLAFSFVVSLILGLIIKYTIGIRASEEDEFKGMDESEHAETSYDFAAVGGTARTAVKEA

>CORE_REP|Org26_Gene4804#

MMMSTRDSATKRRPDSGRSQDSAVGLNQPKRDWMGAAMRVMTTLTGSELAEKYNLRKPIERVTYEGTKTGFRTLGAATRAFNKVAGGGQPKRLATNEAKNKDYFDLTPTDEQQMIVETVREFAAEILRPAAHDADEAAAAPKDLLGRAAELGITLINVPEELEGAASERGAVTNSMVAEALAHGDMGLALPILAPSGVAVALSQWGTDAQQQTYLPAFTGENVPQASVVISEPRALFDPFALQTKAVRSPSGYRLSGVKSLVPAAADAELFIVGAELDGRPALFIVESDAQGLVVEADPSMGLRAAGLGRLILDNVAVGSDALLGDGDGKQHAEDYADAVRLARLGWASLAVGTGQAVLDYVIPYVNEREAFGEPISHRQAVAFMVANIAIELDGLRLVTLRGASRAEQGLSFAREAALAKKLATDKGMQFGLDGVQLLGGHGFTKEHPVERWYRDLRGIGVAEGVVLV

>CORE_REP|Org53_Gene4027#

MTAARSVSASSATSHSTGTGKTAAVSSDSKGARSSHSQASAKTFVIDTSVLLSDPWAFTRFGEHHVVLPLVVISELEGKRHHHELGWFAREALRNLDDLRLLHGRLDQQVPIGTEGGTLQVELNHTDPSVLPVGFRTETNDSRILACALNLAAEGRRVVMVSKDIPLRVKAGAVGLHADGYHAQDVVTSGWSGMVELDVASSQIDQLYAESVIDLDAARELPCHTGIRLLGGSSSALGRVTPDKRVQLVREREAFGLHGRSAEQRIALDLLLDESVGIVSLGGKAGTGKSALALTAGLEAVLERRTQRKVVVFRPLYAVGGQELGYLPGSESEKMGPWAQAVFDTLDGLASPEVMEEVLSRDMLEVLPLTHIRGRSLHDSFVIVDEAQSLERNVLLTVLSRLGSGSRVVLTHDVAQRDNLRVGRHDGVAAVIEKLKGHPLFAHITLTRSERSPIAALVTEMLEEYGPNA

>CORE_REP|Org67_Gene2964#

MTRIAIIGGGPAGYEAALVAAQHGAQVTLIDRDGIGGACVLWDCVPSKTFIASTGMRTDLRRARDLGITLDPSQAAVQLPEVNARVKALALAQSSDIRSKLLSAGVTLISGTASFTDPAPGRAPHRITVRPTGERAGERVIDAEVVLIATGASPRVLPGAEPDGERILTWRQLYDLRELPETLVVVGSGVTGAEFVSAYTELGVQVKLVSSRDRVLPGEDADAALVLEEALAERGVELVKHARADAVERTADGVVVKLSDGRTVAGTHALMTVGSTPNTGDLALDKVGIELDRGGYLRVDRVSRTAVSGIYAAGDCTGLLPLASVAAMQGRIAMYHALGEGVSPIRLKTVASAVFTRPEIATVGVSQTAIDNGEVPARTVMLPLNTNPRAKMSGLRRGFVKIFCRPATGVVIGGVVVAPIASELILPIALAVQNNLTVNDLAQTFSVYPSLTGSVTEAGRLLMRHDDLD

>CORE_REP|Org16_Gene180#

MAKTYVGARLRQLRTERGLSQISLAKKLEISASYLNQIEHDVRPLTVPVLLRISEVFGVDTSFFASQDDTRLIAELQEVVMDTELGIEADAQEIADMVSAHPSLARALVNMHRRYRNTTAQLAAATEDRFSDGSGSGAISRPHEEVRDFFYQRQNYIHELDTAAEELATRMRLHGGDLRRELTRRLTTGYGVQIVERIDLGEGVLHRYDPEARKLEIAPHLSGGQRVFKLATELAYLECGDLIDKLVEEGNFASEDTRTLAKLGLANYFAAATVLPYSHFHEVAEDFRYDIERLSAFFAQSYETICHRLSTLQRPKLRGVPFSFVRVDRAGNMSKRQSATGFHFSSAGGTCPLWNVYETFAYPGRIMTQIAQMPDGRKYLWIARTVERRATRYGQPSKTFAIGLGCELRHAGRVVYADGLDLTDPQATPIGAGCRVCERANCPQRAFPPLGKSLDISEHRSSISPYVLR

>CORE_REP|Org102_Gene1432#

MTTTLHPSSEPPRPVGEGTESAEVAGAIESNSAPAGAGADISAPPRDDSTEATTASDTGSMAATSSAFAMAPRHGDAAASGIERDAVERAANVASEPGAASTEFPRRPAGFLPALEGMRGMAALGVVVTHVAFQTGATSLPMVGRVLERFDMAVAVFFALSGFLLWRPHAAAARGLGTAPTAGRYLLHRAARILPAYWAVVCAVLILLPTAASTAGFRVWLANLGLVQVFVPLTLTDGLTQMWSLSVEMAFYLLLPLLAVAVAWLRGDRARWRVPVLLAFGTLCLTWNLIPVPTPDAINSDNWLPGYLPWFAAGMLLAELSDLAVPRLRRLAGNPWILWTIALVALLLSATDLGGLPGLTRGAPWQYVLKMAFGAIIGFTLLAPLVLRPDIRHRWLESRTAAMLGRWSYGVFLWHLAVLSIVFPVFAIVPFSGDFPQVLALTIALTLPLAAASYALIEEPVRRWARRFG

>CORE_REP|Org151_Gene6489#

MSVKSTVEQLSPTRVRINVEVPFEELKPDFDRAYKALAKQVKIPGFRPGKAPAKLLEARLGRGAVLEQVVNDVLPGRYSEAVTAGQVKVIGQPEIEITKIEDGEELAFTAEVDVRPEITLPAYDGIEVTVDAFTIGDEDIEEQLLSLRQRFGTLTGVERAVQDGDFVSIDLSATVDGEEVPEAATTGLSHEVGSGQLIEGLDEALIGLNSGESKEFTSTLVAGEHAGKEAVITVTVQSVKERELPAADDDFAQLASEFDTLDELKEDLRTRVERSKKVQQAGEIRDKVLETLLEQVEVPLPEAVVKAEIDAVTHDAVHGFDHDEAKLAEALEAQGSSREEFDKDAKESAEKSVKTQLLLDAIAEADNTQVGQEELTERILFQSQRYGLAPEQFIQQVQQAGQLGAIFADVRRGKALAGVVGKVKVTDSAGNAVDTAEMFGAPEDSAPEIEADGVVEVESDETAGAKAE

>CORE_REP|Org215_Gene5410#

MVTCRDSIVIGSCISHIGRLDEVHEVRRHRPVRRRRHRNRRRDRIRCTGGSAHRTAAGLRAGARAGSGCRLHHRAGRGGRRHRHQRHRRSVQRRRRRAGREPDQRIRCGGGAGAADRKSRWGGRADRRGRRRQRSGTVADPRGRAARARAGHLRATVVLRRIAARRARRGRGRGDRRRHRPARAGRLRDSRRHHRRAHRPAGRRWSAADRLGLRLLQRAALRALILGGTREARVLAETASGERGFEIVSSLAGRVRDPLLPVGQVRIGGFGGVDGLRTWLSDNRIEAVVDATHPFAAGITDHAAAAAASLGLPILHVRRPGWTQRPGDSWIRVPDLSAAATAVAGLGDRIFLTIGRQGVSAFAALRGHWFLIRAIDPPEGALPPRHELLLARGPFTVADETALLTAHRITALVTKDSGGAQTEAKLDAARARGLPVVVVDRPPLPTGARSVDSVAGAWDWLRAAQRLS

>CORE_REP|Org4_Gene3246#

MNAVSQGDSVSGGGRSAVGPNSAQMVWGRDQPIGVAVLGMGNVGTEVVRILREHAEDLRSRVGAPVVLRGVAVRDLATDRGIPTALLTTDADALVARDDVDLVVEVIGGIDPPRRLILAALNAGKSVVTANKALLADYTGELAAAAERNRADLYFEAAVAGAIPVVRPLIQSLAGDRVNRVVGIVNGTTNFILSAMDETGADYADTLAEATRLGYAEADPTADVEGFDAAAKAAILASLAFHTRVTAADVYREGISKISSEDLETASALNCTVKLLAICERVAAGPGEPSPEEGGKERVSVRVYPALVPRKHPLAAVSGAFNAVVVEAENAGRLMFYGQGAGGAPTASAVLGDLVMAARNKFYGGRAPGESVYAELPIAPIGDTPTRYHVNLQVEDRPGVLAAVAGEFAKHGVSISTVRQEGHGTGARLVVVTHHALESALADTVAALAEMESVTSITSVLRLEGTEE

>CORE_REP|Org102_Gene5974#

MNSMIAAPEVVETALRHSRADEAVVIVTDAHDASLRWAGNSMTTNGSSISRDWAVVSIFRDGPRVARVGSVGSTSVDPAEIESVVRASEAAARDAEPARDAMPLLTPETVGPAGPDGELPWNGSPAGTDITVFEGLARDLAAGFDGADRLYGFAHHQMHSSWLGTSTGIRRRWVQPTGSVEINGKRGVGAELASAWVGAGTLDFTDVDTPALLGELTRRLDWSARRVELPAGRYETLLPPSAVADLMIYMAWSMEGRGAQEGHTAFSRPGGTRIGERLTEIPLTLYSDPSAAGLEYRPFVATPSSSESLSVFDNGLTARRVDWLRDGAIANLVYPRATAAEFDAAVTPPGENLLMTGGTDASLAEMVARTERGLLLTCLWYIREVDPATLLLTGLTRDGVYLVEDGAVTAAVNNFRFNESPLDLLRRVGEAGRTEITLPREWKDWFTRTAMPALRIPDFHMSSVSQAT

>CORE_REP|Org142_Gene2971#

MGNIAAENTDLAIELTAAAEAELPGFAAPIFRPADEGFDAEVAGFQTAYQHRPAVVVAARHAEDVRAAVEFAARHRLPVAVQATGHGLSVAASGGVLISTRRMTGIEIDADAATARVGAGVQAGALIDAAAQHGLAPLNGSSPSVGVVGYVLGGGLGLLGRTFGYAADHVRAIELVTADGRMRTLRPGDELFGAVLGSGGNFGVVTALELGLVPVTTVFGGQLMFDTALVPRALEVWREWTATVPDTVTSTVAMLAFPDIPQVPQPLRGRFVASIRVAIDGSAEEGERLVAPLRAIGTPMKDDLRTMPYIESHTIHSDPADPHAYAATNALLDDLTPEAVEALLAVAGPDSGLGAVIDIRHLGGALRAPGHDDLALDYREAGYVVRAITLPEPGAEAVEPAAAVRAALAPWTVGHSLNFLYGAPGMAGVAQTRAGYRADTYARLAALKSKYDPHNMFRFNRNIRPER

>CORE_REP|Org102_Gene679#

MEGMRIADHVVDLIGNTPLVRLNSVVGPNSGLVAAKVEYLNPGGSSKDRIAVKMIDAAEQAGLLRPGGTIVEPTSGNTGVGLALVAQQRGYKCVFVCPDKVSEDKRNVLRAYGAEVVVCPTAVAPEDPQSYYNVSDRLVREIPGAWKPDQYSNPGGPDSHYETTGPEIWRDTEGKVTHFVAGVGTGGTITGTGRYLKEVSGGKVQIIGADPEGSVYSGGTGRPYLVEGVGEDFWPSAYDPAVPDEIIAVSDADSFDMTRRLAREEGLLVGGSCGMAVVAALRVAERDPDAVVVVLLPDGGRGYLSKIFNDDWMSSYGFLRSRLDGSAATEPLVGDVLRGKSGALPDLVHTHPQETLRDAIEILREYGVSQMPVVGAEPPVMAGEVAGSVSERDLLSAVFEGRAHLTDSVKQHMSPAFPLIGSGEPVSAATKALEETDALMVVEDGKPVGVITRHDLLGFLSTGALGH

>CORE_REP|Org102_Gene1398#

MDIVVRHWSGKEARALRDAKRMSIREFAAHLGVHERLVSKWEAGGIRVHPRPINQAALDTSLARSDDVVRARFAALIDQPLIDRPGPGFSARQSSSAQAIYSSDAELLSLVDTGAMRGDALAAISERDLIMAAAHEASEHAGRAESTNVGSTTLEQLDADVTRIANDYVHVPPVPMMVEMLRVRRRVYRLLEGHQRPADTSHLYLLAGTLSGLLANASTDLGYYDAAGEQARAAWAYAELCGHNGLRAWTRGMQALIEYWSERPRRAVLLAQNGQEYAESTTAQVRLHNIEARIWSRLGSSADTENCIRAAEAARGSSGANDSLHDEVGGVFGFNEPKSQYYAGATYIHLGQAEPALQATRRAIELYANGPTEQRSYGAEALARVDSAAAHLINGSLDGATEALQPVLQLDEDKRIAQLEERLTGLRQRLAGPTFREAIEARRLDERIEEFCGTTAAKGIPPGDQPR

>CORE_REP|Org13_Gene5085#

MEDHVTTPESVRLTSAVDPATAVSRLACSGHFSDYIVYERPGRWVFAAAPLGRVELDTDELRVSTSAGSARERWTGRPVDALERALDSLRVTCGPGASGTAYGWIAFEFCADALGAQRHLTERADLAHVIIPRIEVTVTESGVGVDGATAAEVEVIEQLLLSPAEPLPTPHPVDVRADTCGYRARVAAAVAEIAAGDYQKVILSRRVELPFRVDLPASYRLGRAHNTPARSFLLRLGGLAAAGFSPELVVSVDDEGVVTTEPLAGTRALGQGVAADLAARNDLESDPKEIVEHAVSVKTSFAEIASIAEPGTTTVADFMAVRERGSVQHLASTVRGRLARHRTRWDALDALFPAVTASGIPKRAAVDAVFRHDAARGLYSGAVVTLSESGSLEAALVLRAVYQDADAAWVRAGAGIVAQSRPDREFEETCEKLGSVAPYLVPAISHGPGSTPRSPMVTGRRSHRS

>CORE_REP|Org141_Gene3803#

MGRLFGTDGVRGLANESLTPELALRVSGAAAQILSRGKKRALAVVGRDPRASGEMLEAAVTAGLTAAGVDVLSVGVLPTPAVAYLTGLYDACLGVMISASHNPMPDNGIKIFAAGGHKLDDAIEDRIEAVMAEAPLRPTGAGIGRVLGASGARDHGLAIPDQYSVAGTHERYVEHLVEATGHELNGLTVVVDCAHGAASEVGPAAYREAGATVIAISADPDGLNINDGCGSTHLDQVRRAVREHGADLGLAHDGDADRCLAVDADGNVVDGDAILAILALAMRDAGELAENTLVATVMSNLGLHIAMREAGITMRTTAVGDRYVLEELRRGRFTLGGEQSGHVVFPAHGTTGDGILTGLKLMGRMASTGRTLADLASVVQTVPQILVNIPVADKAAVMAAADVLDAVADAERELGETGRVLLRPSGTEQLVRVMVEATDLAQAQRLADDLAERVAAVQRISAPSH

>CORE_REP|Org141_Gene4341#

MATPAVDGRKGYVSATGGVPGSQQRSRAVSEVVELVSTLIRFDTSNTGELATTKGEQACAEWVAEQLREVGYTTEYVESGAPGRGNVFARLKGADSSRGALLMHGHLDVVPAQAEDWSVHPFSGAVRDGYVWGRGAVDMKDMVGMMLAVARQFKIEGTVPPRDLVFAFLADEENGGKWGSHWLVDNRPDLFDGITEAVGEVGGFSLTVPRPDGGERRLYLVETAEKGLGWMRLRAKARAGHGSFLHEDNAVTILAGAVARLGTHTFPLVVSDSVAEFLAAVSQESGLEFDPSGPDIEGTLAKLGSISRIIGATLRDTANPTMLNAGYKANVIPQTAEAVVDCRVVPGRQAEFEREVDELIGPDVEREWITKLDSYETTFDGHLVDAMNAAILAHDPQGRTVPYMLSGGTDAKAFARLGIRCFGFAPLQLPPELDFAALFHGVDERVPVDALEFGTRVLEHFLLHS

>CORE_REP|Org112_Gene6545#

MRTVPTATAPRPPRTPRRNPRPAPHRTRPTARTPPPWEPPAPAVVAAPTRIPTPTTAVVSRWRRSWPTCAPRATANRTHGTSGAGRRPRVPRGRRPAVSSGTSHSSWPPRIGTDILVGVTSGTRVARDWNGTFERTRVTSDKAVCGTDPAPARLTVGIVSAGRVGSALGAALERAGHVVFGVSGISDASVYRARTRLPDSEILPAEEVARRAELLLLAVPDSELAGLVSGLATADAVRPGTIVAHTSGANGIGVLAPLTARGALPLAIHPAMTFTGHDEDVSRLGNACFGITAADDIGYAIAQSLVIEMGGEPVRVAEEHRTLYHAALAHGSNHLVTLILDAVEALRAALAGPGLLGQQLVDDQPGGLAERVLAPLASAALDNALRRGPSALTGPVARGDVDAVAAHLNALESTDAELAAGYRALSLRTAQRARTNPALLELLASPSDRRDAAEGSDQAKEGN

>CORE_REP|Org162_Gene1548#

MIPCSTDVGRRRSGGTDVEVGNDRANDPTDAEQLAQRYRSLVEHSPDGVVVHERGILVYANPAIVRLLGADSTEDLVGQPVTRFVDPKSVPGMLARIGRLTEAGAASEPAEMTLVRTDGSLLDVETVSVLTAWHNRLAYQVVIHDLSAQRAAEAAQRRAEQHFTTVVSQLEEGVVVIDRQGRIESINPAALRIFGHEGEDLVGTPIYALPLTLLDANAMTLPPTRHPVARTLATGETVVGYVFGVDRPDGQRRWLSGSSRLLNPGDPQSSAVSSFNDITEFRASRRQLEYQATHDPLTGLANRALVLSRLAAALGTTEDLPVSTVLFIDLDGFKSINDTLGHAIGDTVLQIVAQRLQRGLRTDDIVGRIGGDEFLVLLSGRTLGEDLEALVARLRQTMAEPIIARGHRIQVDASIGITPLHPGDSRTPEAVLHDADVAMYRAKPPGHRDSSPVTRRPNNTHAS

>CORE_REP|Org150_Gene1525#

MRKLAARRATVGRASVARASVKTAAVALASTLLLGPWIATSPALADPAPAQSAADTLPPELVQAIGRDLKMSPAEYLDRAARAQQLRDYAREFRSSHPQAFAGAWLGADGKPVMAVTSLDAARIANSAGYQTRLAPISADNLESSLDQLVKWIGGLPRELSAGINSVAIDFLNSQLVLSVANTPAGHLLNLPTLIANVKVVLSPDGGGPVEHRPMAGDTYISAPTSLDDSALKSVDVCSFGFNSIDAAGNALNISAGHCDPNVGKGNSEAGVYLPNVRNLKASPQFGTFVKAQLGGSTGLDYSVIKLNERAITAGMDQPSVRGGNGTTLTLTGTAEPITGAPICKSGQSSTFTCGFVVADRVETALFTAEGESKTVRGFASSACTLGGDSGGAIVSGTLALGITSGSNAADAPDCRAANMDLAQYGGTATLGIPIRQILSDIDANSGGGLGAGITVRTRPNAG

>CORE_REP|Org102_Gene2285#

MTDTAGAEDDPRIAALRAAVRKHMPQAKADLAELVSFKSVADPRQFPAEECRKAAQWVADAFVAEGLTDAGLHTTPDGTDTVVARRAAPAGKPTVMLYSHYDVQPPLDDAAWHTPVWQLTERDGRWYGRGAADCKGNIVMHLTALRALREVLGDKGFPVGLTLVSEGSEEQGTGGLERFVPEHADLLRSDTILMCDTGNFAVGVPTFTETLRGNVNVVVTVETLASPMHSGMFGGAAPDALAALIHLLATLRDEHGNTTVDGLANDQVWDGVQYPPEQFRADATVLPGVDLVGSGTPADMLWARPALTVLGIDAPKVVGSSAAIQSTAAARLNLRIPPGTDPGQALRLLAAHLERHTPWHARVTVETEGVGAPFRSAAGGSARTAMAAALAAAYGRPATTEGQGGAIPLCNVFADTYPDAEIMLLGVEEPKCLIHAPNESVDPTEIEHMALAEALFLASYAD

>CORE_REP|Org119_Gene7103#

MSHPAAEVACPTAALARCETLVGVRFRADRLGGPDSGPPAVSGGGRDARRRAGPGAPGSVVSEPVARSGRPARAGASGAVPPLDAEDRLDVGTPPRRRPGSARRSGVEPVVDPLAPQSDSPSPRRDHNGVEIYDPLGLYDRPDRSHQPLRARWDPTAPDEGRTRERPERTAKKQSALGRFVSTYGWRAYALPVLFAVTVLVIVDAVRGGDPIVGTGNTPGLGQLSPHTRNAGIIGLPSGDGHFPADLPTGALPQGGSFAETGAGTWHMVPGSTGQIGTGTDSVFRYTVEIEDGVDTSGFGGDESVAKLIESTLANPKSWTHDPKFAFRRVDQGDAEFRISLTSRESTRKACGFEIPIDSSCYNADLGRVVLSEVRWVRGAIAFEGDIGSYRQYQINHEVGHAIGYHQHQPCETDGGLAPVMMQQTFGTKNNDIAVLEPGGVVPMDGKHCRFNPWPYPRG

>CORE_REP|Org215_Gene1707#

MNSLFLLALAIVLVPLGGIFAALDSSLNTISAARVDDMVRAERPGAARLAHIITDRPRYVNLMVLLRVLCEITATVLLAAVLLDWMDQLWALVVTAAVMVLVDYLVIGVGPRTLGRQHAYSLALAASLPLQAIGTLLGPVSRLLILIGNAITPGKGFRNGPFASEIELREVVDLAGERGVVADDERRMIQSVFELGDTAARAVMVPRTEMVWIESEKTVAQAMSLAVRSGHSRIPVIGENVDDIVGVVYLKDMVPYADRSRKVRVHEVMRAAVFVPDSKPLDDLLDEMQRRRNHMAVLVDEYGGIAGLVTIEDVLEEIVGEIVDEYDQNEVPDVEDLGNGKYRVSARLSVEDLGELFGMAIEEEDVDTVGGLLAHELGRVPLPGSKAVAHGLVLKGEGGSDARGRVRVHTVVVKRAAEKTGAEKSDAGRSTSERVDGESAGVNGVGGSGANEDGEADD

>CORE_REP|Org39_Gene6539#

MGGLHPFDGSRTPGGCQFDSACVSGPRRRRCRPAGGRGVRAVVGGGRATVGSRETRRRESRVTVTEFRARTAPSRWPRAGPSEVDLVRTLAVVGGGVIGLSVAWRAAESGWRVTLYDPAVGSGASWVAGGMLAPLSEGWPGEDAALEFGAASLTRWPDFAARLKSVTGAEVFTAAETLTVALDAADAADLRTIADWVNAKLAESGAEADPAVGSALRLLDRAGVRSVEPGLDRRVRAGLLSPAEPAVDNRSLVTALREACVAVGVEVRAEEVAALRELPHDRVVLATGASARLWPDLPVRPVKGEILRLRRRLSAPPPPNRVVRARVHGRPIYLVPRPDGLVLGATQYEAGFDTVVTVGGVRDLITDAEAIFPGVGEYEFAEATAGSRPGTPDNLPLIGYLDERVIAALGHGRNGILGVPVTADAVLALLADTELPAARAASPTRFRVPEPHFAGGKQ

>CORE_REP|Org96_Gene5513#

MEQDTGQAVARRGLRARLRAQAATMSGTARFDLATVFVVLVLYTVAWPTLHLTHVVAPGAQPFVAALAAFPLLLVRINPALGWAVSAGSALVIALAIPHQPANEMPFQVVHVLSLIVLLFAVGLRAPMQLVLLAWASTSLLFGTTMPGEGEAFANAAWGWPIALTVVVLFAMLIRWLVLSRRQLVRQEEENELERARRAILEEKARIARDLHDVVAHHMSLVVVQAQTAPYRVAGVNEAARAEFESISTTAREALNEIRGMLGVLRSDGVAPEHSPQPKAGEVLALFEGARRAGVDIDWTVDGDLSRVADTTGLALYRIVQESLSNASRHAPGAAVRVTLRCADTLDLLVVNGPAAAPAGSAGNGGHGIAGMRARALAAGGELVAESTADGGFEVRARMPLTPDPVVTVAAPAVPAASVAPAASVAPAASVAPAASVAPDSAVVAQPAPVDASGRGA

>CORE_REP|Org101_Gene5725#

MTDQKPESFPLRRSVAASAMGNATEWFDYGVYAATATYLTDAFFPGELGTLGTMLGFAVSFVLRPLGGMVWGPLGDRIGRKAVLATTILLMAAATGAIGILPTHSSVGVFAPILLIGLRVVQGFSTGGEYGGAATYLAECASDKRRGFLGSFLEFGTLAGFVGGSATVLACQLAIGSDAMHDWGWRIPFLLAVPLGLVGWYLRSRLDESPVFTEVAEVAEQTDQEHRPGGLHGLRELVTTYRRELLTLGGLVVALNVVNYTLLTYQPTYLQKTIGISESGTTAMMLIGQTVMMVTLPFFGRLSDRVGRRPMWLFSLVGLAVLALPMYWLMGQGTAWAITGFIVLGLLYVPQLSTISSTFPAIFPTQVRYAGFALAYNVSTAAFGGTAPLVNEAAIESTGWSLFPAAYMIGASLIGLVAWCFLRETAGTSLRGTEVPDAGEDAPAIIPAGPAGAALAP

>CORE_REP|Org101_Gene854#

MAHEHRHYAVYQTLWHTIFGAPRAPAPPGTRASIEVMGSPEVTPASGDALPAQAEVLVVGAGPAGSASAAWAARAGREVVLLDSAVFPRDKTCGDGLTPRATAELEHLGLGEWVRAHTVNHGLRMTGFGREALLSWPAGAFPTYGSAVPRTELDDKLRETAMKSGARMIDGTKVIDVTRDGDRVTGVTVRTATGTHTIGCTLLIVADGVRSPVGRLLGRTWHRQYAYGTAARAYIKSGRSDDQWITSHLELRDAAGALVPGYGWVFPLGNGEVNIGVGSLATEQRPSHIALKPLLEHYTKQRFEEWQFEGSLRAVASALLPMGGAVSHVAGRNWVLVGDAAGCVNPLNGEGIDYGLEGGHMLAGLLDEPDLTTIWPELLRARYGRTFSVARRIAGLATHPKMVPIGGPPVMRSKYLQRTAVRVMGNLVTDEDVDLTARAWRAAGRMSMRVDDLPPFA

>CORE_REP|Org126_Gene7010#

MPRSGDERSWSRTGRSAHRAAAPGCARADDAGADDARADDARADDAGADDARADDFDAQVAPADPALLAAADAAVTAAESDLIALSHSIHAEPELAFAEHRSVAKTLVPLRERGFGIETPVADLDTAFVATYGSGDLVVGICAEYDALPEIGHACGHNIIAAAAVGAGLALAEVADRCGLTVKVFGTPAEESGGGKVLMLERGVFDGVAMALMVHPGPLDIVGARSLALADLSVSFHGREAHASAAPEYGRNAGDAATVAQVALGLLRQHLRPGQQLHGIVESGGVAPNIVPGHAELLYYLRADDSASLDDLLQRASACFEAGALATGCTHEIRALAPTYTELTPDSALLCAYREQIIGMGRMPLAPDLEAARPLGSTDMGNVTNVIPGIHPVIGIEANGAVTHQREFAAACVTPSADLAVADGARALARTAIQVAGDQLHRDRLLERSIQRQEEIR

>CORE_REP|Org117_Gene5341#

MNRRVRDSGRDDGYGRRTALAREWESGVETLLVVGAGPKALAVAAKSHVLRQLGLSAPRVIAVEAHAVGGNWLASGGWTDGRHRLGTSPEKDIGFPYHSTWARGHNREINEAMMAFSWTSFLVEHGTYAEWIDRGRPSPQHHVWAKYLQWVARKIDLELVLGKVRTIRQRPTDGGAGWSVEVAGADGATTELEADGLMITGPGQSTKALAKHPRVLSIAEFWDLAGKRKLPISSRAAVIGGGETAGSALDELVRHEMLTISVISPMATIYTRGESYFENSLFSDPTKWNALSIQERRDVIRRTDRGVFSVRVQESLLGDNRVHHLQGRVTRIVGQGDGVAVTLRNEMRADQVHNFDLVVDATGGQPLWFLDLFDSESADLLELAVGGPLTQQRIESSIGYDLAVTGLGAKLYLPNMAALAQGPGFPNLSCLGELSDRVLRAEPARVRAGARQLAAQ

>CORE_REP|Org39_Gene2821#

MMLRVSVIGTGYLGATHAAGMAELGFDVLGVDNNAAKAAALAAGRVPFHEPGLPELLSKHVDQGCLSFGTSLAEAASFADVHFLCVGTPESPTGAADLTQLYTAIEGLVPHLTRNCLIVGKSTVPVGTAEALAARVDELAPRGIAVELAWNPEFLREGHAVYDTLHPNRLVFGVSTPDAEWALRQVYAAAIAEGAPVVVTNLPTAELVKVSANAFLATKISFINAMAELCEITGADVNLLADALGHDDRIGRKFLGAGLGYGGGCLPKDVRALIARAGELGVPESVRFLDSVDAINLRRRERVVRETLEILGDERPTGRVAVLGAAFKPLSDDVRDSPALDVAVRLHHAGIEVTVYDPEANRSASRIAPQLRYAPNATAAVLGADVVLHLTEWREFRELDPALLSSVARRRVLIDARNTLDPEPWLSAGWDFRALGRLVARRADAGRVDPLLESA

>CORE_REP|Org30_Gene3982#

MAVAIALFTRDLRVRDNPALTAAARSAEVLPVFVVDETICASDYLSPNKATFLAATLADLDDQLRRLGGCLLLRGGDTVREVCQLVRQYSVDEVHVAADVSGYSRRREDRLRARLTQLGCRLRVHDSGTTVAAPGHLLPSGGTDHFAVFTPYFRRWSSMGMRAPLPAPRRIHLPDGVGTDRVPTAAELRVGEVSPELAPGGESAGRRAARNWFRDGIAAYSDLHDDLAADATSRLSPYLHFGCLSAVELVHRSDSSSPGGAAFVRQLAWRDFHHQMLAARPSAAHSDYRSRNDRWSDDDALLAAWRAGRTGYPIVDAGMRQLAAQGWMHNRARLITASFLTKTLYVDWRAGAKHFMGLLADGDVANNQLNWQWMAGTGADTRPNRILNPIRQADRYDPEGDYVRRWVPELARLRGSDIHQPWRLTQDATTGYPARIVDHEHAAAEFRTLRQRDSH

>CORE_REP|Org4_Gene7917#

MPLQQFESFIRSAVGHIDLNEEVESEMSNAGTPKTAAEIQQDWDTNPRWKGVTRNYTAEQVSKLQGTVVEEATLARRGSEILWDLVNNEDYINSLGALTGNQAVQQVRAGLKAIYLSGWQVAGDANLSGHTYPDQSLYPANSVPSVVRRINNALLRADEIAKVEGDDSVKNWLAPIVADAEAGFGGALNAYELQKAMIAAGAAGVHWEDQLASEKKCGHLGGKVLIPTQQHIRTLTSARLAADVADVPSVIIARTDAEAATLITSDVDERDREFLDGTRTAEGFFGVKNGIEPCIARAKAYAPYADLIWMETGVPDLEVARKFAEAVRGEFPDQLLAYNCSPSFNWKAHLDDATIAKFQRELGAMGFKFQFITLAGFHSLNYGMFDLAYGYAREGMTAFVDLQEREFKAASERGFTAIKHQREVGAGYFDTIATTVDPNTSTAALKGSTEEGQFH

>CORE_REP|Org56_Gene4749#

MSSVDVLSRSKGRLDSYFGIARLGSTMKRELMAGTVTFLAMSYVLAVNPAVLGDHGQLGSRGIPTQAVFTATAVAAVVGTLVMGVWARYPIALAPGMGLNAFFAYSVVLGMGIDWQVALSGTLLSGIIFFVLAVTKIREKIIDAIPLQLKLAVGAGIGMFVAFLGFKNAGIVVSDPATFVHLGDFTKGTTLLALFGLLVTVVFLVLGWHGAVLYGIVCTTVVGIVSGLVHLPHQVVALPHGLDQTFGQAIVNLPHAFTGQMAIVVLTMLFVDFFDASGTLIGIANQAGLLGPDGKLPRAAQALAADSIGTAAGAIIGTSTTTAYVESTAGVSAGGRTGLTAVSTAGWFLAAMFFFPIFAVVADVPAVTAPALIVVGVLMSRALGDIDWSKLEFAIPAFITVIMMPLTYSIANGIAMGLTFYPVVMVARRRGREVHPVMWVLMAVFLAYFFFLAE

>CORE_REP|Org5_Gene7373#

MRPASPRDSSAVARASVTGARETPAAGISEVEPPAHSSQPLRERTFSAPDSSELANLSAPGDPVREVLLLCWRDTGHPQGGGSERYLERVGAQLAARGVKVTLRTARYRGAARRERIDGIEISRAGGRFSVYPRALAAIAAGRMGFGPLRGLRPDAVIDTQNGIPFFARVVSGAPSVVLVHHGHREQWPVAGRLVGRIGWWIESWLSPRVHRNDQYLTVSLPSAEELASLGVDAARIAVVRNGAEPVPGQSPTGAEPIRTPHPSIVVLSRLVPHKQIEDALEVVAGLRGRLPGLELDVIGDGWWADNLKTRARELGIADAVNFHGFVDEPRKHELLSRAWVQVLPSRKEGWGLAVIEAAQHGVPTIGYRSSRGLTDSIVDGVTGVLVDDVFQLTETTGELLADPEMRVVMGEKARTRAREFSWEQTGFGVGSVVAAAARGEFVSGLVAGRSAE

>CORE_REP|Org212_Gene3834#

MVSAPGDHAGVSLEGLRILPVTGLPEFRPGDDVAERIAAAAPWLADGDILVVTSKIIAKAEGRVVPAPVDPEERDAVRRALVEQEAVRVLARKGRTLITENKLGIVQAASGVDGSNVEKDELVLLPADPDASAAALRAGLAERLGVRVAVVVTDTMGRAWRNGQTDAAIGAAGLRVLHDYAGAVDGQGNELHVTQVAVADELAAAADLVKGKLRGVPVAVVRGLPTQEDGSTAADLVRAGEEDLFWLGTAEAVERGRREAVLLRRSVRTFADTPVEPDTIRAAVSVALTAPAPHHTRPVRFVWVRDAQRRQRLLAAMADKWRADLRADGLDPERIERRVGRGRILFDAPEVLIPCCVPDGAHSYPDERRQAAETTMFTVAVGAAVQGLLVALATEGVGSCWIGSTIFAPEVTRAELDLPADWNPLGAIAVGYPTEELAPRPPRDPGDGLVER

>CORE_REP|Org5_Gene1113#

MSTIGRSPAGREDDAAEGDSAGTGTPISASRQTTTGLSGRTSGSAAGPEAGTCRHTGLATGRQTEVAADQAAVESWLREHGDDLIGWRRHIHANPELSRAEHATTEFVESWLVKADLEPRILPTGNGLICDIGPSGPRLALRADMDALPLQEYTGRPFASTVPGVSHACGHDAHTAILLGTALALAELDELPVGVRLVFQHAEEVMPGGAIDMVAAGAMDDVSRVFALHCDPRLEVGRIGVRVGAITSAADTVELVLDSPGGHTSRPHLTSDLVYAIGTVITGLPGLLSRRIDPRTSTVMVWGAVSAGKAPNAIPQTGMLTGTVRTGDHATWSLLEPMVREIVDGLLAPTGVRYQLNYKRGVPPVVNDEFCTRMFEDAILGLGPDALSDTPQSGGGEDFSWYLEEVPGAMARLGVWSGEGPQLDIHQPTFDIDERALAAGVRVLTNLVLQAR

>CORE_REP|Org101_Gene2174#

MVGAWCWTASPKLSAVRVLVIGSGAREHALVLALRRDPAVTGIVAAPGNAGIAQHAQTRPVDPCSAEAVVALATDVAAELVVIGPEVPLVLGVADAVRAAGIACFGPSAAAARIEGSKAFAKDVMAAAGVRTAHSEIVDNPADLDAALDRFGPTWVVKDDGLAAGKGVVVTADRSAARDHGAELLEQGHPVLLESFLDGPEVSLFCLVDGETVVPLLPAQDHKRVGDGDTGPNTGGMGAYTPLPWLSPDAVTTIIEDVVKPVAAELVRRGSGFSGLLYAGLAMGVAGPAVVEFNCRFGDPETQAVLALLESPLGELLAATANGTLAEVEPPRWRDGSAITVVVAAENYPGRPRIGDVISGAGDGAIDDTAAVLHAGTALREDGALISAGGRVLNVVGVGADLAEARTNAYARITAIKLPGSHYRTDIGLAAVEDRIAVPDRASASSGQTRES

>CORE_REP|Org101_Gene3287#

MLTLADMDRQKEFVLRTLEERDIRFVRLWFTDVLGYLKSVAIAPAELEGAFEEGIGFDGSAVEGFARVSEADMVARPDPSTFQVLPWSTSKGHQHSARMFCDITMPDGSPSWADPRHVLRRQLNKAGDVGFSCYVHPEIEFFLLENGPQDGSQPIPADSGGFFDQAVHDSAPNFRRHAIDALESMGISVEFSHHEGAPGQQEIDLRYADALSMADNVMTFRYLIKEVAIDEGVRATFMPKPFAQYPGSAMHTHMSLFEGEANAFHDPDDPINLSVTARAFIAGILEHAPEISAISNQWVNSYKRLIHGGEAPTAASWGRSNRSALVRVPMYTPNKSSSRRIEIRSPDSACNPYLTFAVLLAAGLRGIEKGYTLPPEAEDDVWSLTAAERRAMGFRELPGTLDEALQAMERSELVAETLGEHVFDFFLRNKRREWADYRSQVTPYELKEYLGL

>CORE_REP|Org113_Gene5852#

MGTSGSDGGTATKTTVLITVTGPDKPGVTSVLLAALSRHGVSLLDVEQVVIRGRLTLGVLVTSPGDPEELQDQLEEAMATVGMEVEVEIGANSVSGAPLSTHAVVVLGSPVTARAFSTIARTLAAQGANIDSIRGIADYPVTGLELMVTAPTTVPDDAATLTTPVAAGPDPSTASSGGSRGRAAASGPDPSIAETRLRTALAEVAAKENVDVAVERAGLARRAKRLIVFDVDSTLIQGEVIEMLAAHAGVEDEVRKVTEAAMRGEIDFAESLRQRVATLTGLDETVIDRVAERIELTAGARTTIRTLRRLGFRCGVVSGGFRQVIEPLAHELELDFVHANTLEVVDGKLTGKVIGEIVDRPGKAVALRRFAAEAGVPMEQTVAVGDGANDIDMLNAAGLGIAFQAKPALREVADTALSHPFLDAVLFILGVTRDEVEAADARDGLLRRVPLS

>CORE_REP|Org116_Gene5304#

MCRASLLVQPAVLELIVERVSVCRRRHLVVETRSARPNLPDGFDVTDPDIYAERVPVEEFAELRRTAPIWWNPQPPEVGGFHDDGFWVVSKHADIKEVSRRSDVFSNFENTAIPRFNDDISREQIELQRIVLLNMDAPEHTKLRKIISRGFTPRAINGLRAELSAKAEQIVKAAAAAGSGDFVTQVACELPLQAIAELIGIPQEDRMKVFNWSNQMTGYDDPDNDADPVTASAEVLGYAYQMAAARKACPADDIVTTLIEADVDGDKLTEEEFGFFVIMLAVAGNETTRNAISHGMIAFLENPDQWELYKKERPATAADEIIRWATPVTSFQRTALVDTELGGVQIKKGQRLVLLYRSANFDEDVFENPYKFDIMRADNPHLSFGGTGAHFCIGANLARLEIDLIFNAIADHLPDITRLGDPKRLRSGWLNGIKEFPVDYKTAARCPVSH

>CORE_REP|Org24_Gene3427#

MSGPLGHAVGLNLVQNTSAASHDRAGGDPNFAPRAGGDEPYLVGLDLNGRRVVVVGGGTVAQRRLGLLIASGADVHVITRATTPAVEGMATSGQLTLTLRDYVDGDLDGAWYALACTDEPDTNAAVVAEAERRRVFCVRADAARFGTAVTPATARYDGLTLGVLAGGEHRRSAAVRTALLEALQSGVVTDESEPTAPGVALIGGGPGDPDLITVRGRRLLARADLVVADRLAPPELLAELGPHVEVVDAAKIPYGRAMAQEAINDALIDGAKAGKFVVRLKGGDPYVFGRGFEELEACAAAGVPVTVVPGVTSAISVPALAGIPVTHRGVTHEFVVVSGHVAPDHPDSLVDWPALARLRGTIVLLMAVERIEQFAAALLAGGRAADTPVTVVQEGSLRTQRTLRAQLSTVAERVRAEGIRPPAIIVIGPTAGFTAGTPDAEAVSIDQGAS

>CORE_REP|Org57_Gene3374#

MRLVRRTGCTETGYRSSHRLAPVIRSSYDFSVTATLPETTASPAGTTARRRRALVEGRLLVLAAIIMSALVLRVAVTAFSPLAEEIGHEIGYGTAVVGVFGMIPTLMFSLSGLLTPLMVRRLGLERTALAAMLMAGLGMLIRVLVSGTTELFVFSALALGGMGIGNVVIPPLVKRYFPDRLAIVSALYITMVQIGTVLPALVAVPVAEAHGWRISLGMWALLGFAAAVPWFGVLRDRRGRDTADTTALPADGHTTGKAWRSPVAWGMAGMFGMTSLTTYSMFTWLPTIFADAGASAAFGGTMVALFAVVGLIAALTAPTVAARMTNPFPVVIGCAVCFFVAFTALLIAPMSAPILWVIVLGLGPSTFPMALTLINLRTRTPAGSASLSGFTQGVGYAVACAGPVLFGMLHTATGGWAAPFAFLGVAVLVLLAGAWQACKPRMLEDTWSAR

>CORE_REP|Org126_Gene2423#

MLLPRIRLSSAAGRAPRTRLRSAAVAASAAVLVLGPLAATSSADPAPAPAPNLPADLVAAVQRDLKISPEEYLHRADVAQHVAAFTATAQRQFPQAFAGAWLDDAGKAVVALAPGQGLDEAKKAAQDAGFGVKDVAKSQTVLRSEKNAFQQWLSGQPEAVVQAVRGVVIDTVNNSIAVRVDKAGVPMPGFVDPAHVIVMAAPPVGPPESDAQVKPVAGEQPNAPRAGGDAYASVAGKMQLVCSTGFNGEDRNGNPVNITAGHCDPNIPAAGTANAPGMFELLPGNHIGAQLGSFQKSILGNQDYSIVSIDGGSRDRFANNLVRVPGAAPIAITGVADPVVGAPVCKSGSRTGFSCGVVNAVDQTVQVGDRDLTQAFSANICALPGDSGGPIVTGTMALGISSASSVADYPICEIPNLIGALTGDAPQLFAQPVNQVLSDNPGLRVRTN

>CORE_REP|Org190_Gene4684#

MPEADGAGRDVDSGETDSTTTQVSADAETETAVPLLVPADGVPPVLSTPAEIAEAATRIAAGTGPLAVDAERASGFRYSARAYLIQLRRAGAGSFLIDPIPVADALAPLAEAINDLEWVLHSADQDLPGLAELGLRPAALFDTELGGRLAGFDRVGLAAMVERLLGRALRKGHGAADWSTRPLPEAWLNYAALDVELLLELREAVAIALHQQGKTEWAAQEFEHVRLTEPPAPKADRWRRTSGIHTLRRARQLATVRELWTTRDELARHRDIAPSRILPDSAIIAAANAEPRTIAQLRELPVFGGPRQRRYSREWLSAVERARTLPDTELPPLSQPYDGPPPVNRWERRDPVAAARLTRARAAMGELSTEVLVPVENLLTPDVVRRLCWDGLPSYDFGPDSATELGKQIDEFLRSAGARPWQRELAVPRLTVALIVAEPPHEPADGD

>CORE_REP|Org181_Gene5426#

MPSAPTTSPPGSGECQTGRVTALDAVSLITTKRDGGQLSDEQIDWVIDAFTRGAVADEQMAALAMAILLRGMTRRETARWTAAMIASGQRMDFTDLPRPTVDKHSTGGVGDKITLPLAPLVAACGAAVPQLSGRGLGHTGGTLDKLESIPGWRADVPVARMRDILADPAIGAVICAAGADLAPADKRLYALRDVTGTVESVPLIASSIMSKKIAEGTAALVLDVKVGSGAFMKDRATATELATTMVELGADAGVRTVALLTAMDSPLGRTAGNALEVAEAVAVLAGGGPADVVELTLALAREMVALAGLDTDPADVLASGRAMDHWRAMVRAQGGDPDAPLPRATHTEILRADRDGVLTRLDAMGVGVAAWRLGAGRARQGDPVQHGAGVALHAGVGDRVTAGQALCTLHTDTPEAFDSAAAALREGIEIGDAAAGTGPLVLDRIG

>CORE_REP|Org16_Gene5537#

MKVAQTPSDANGAGDAKPVLSYPGGEYAMTVAQAVEGNDGIDLGKLLASTGYVTYDPGFTNTAPTKSAITYIDGEAGILRYRGYPIEQLAASSNFIEVSYLLIYGELPTQAQLEDFTDRIRRHTLLHEDLKRFFDGFPRNAHPMPVLSSAVNALSAYYQDSLDPRDPEQVELSTIRLLAKLPTIAAYSYKKSVGQPFLYPDNSLSLVENFLRMTFGFPAEPYEVDPEVAAALDMLLILHADHEQNCSTSTVRLVGSSDANLFTSVSGGINALWGPLHGGANQAVLEMLDDIKANINGGTVDAAVKDFIRKVKNKEDGVKLMGFGHRVYRNYDPRATIVKKTADQILGKLGVQDPLLDIAKALEEAALTDSYFVDRRLYPNVDFYTGVIYRAMGFPTRMFTVLFAMGRLPGWIAHWREMHSEPLKIGRPRQIYTGYGARDYGDIAGR

>CORE_REP|Org163_Gene2588#

MTTRIELARVDLRGRTPSVAELRAALPRGGVDVDSVLHQVRPVVEAIRDQGVSAALEFSERFDGVIPPTVRVPAAELEGALERLDPAVRAALEESIARARKVHADQRRTDKTTEVVPGGTVTERWVPVERVGLYVPGGNAVYPSSVVMNVVPAQTAGVGSLVVASPPQAQFGGLPHPTILAAAQLLGVDEVWAVGGAQGVALLSYGGVDTDGAQLEPVDLITGPGNIYVTAAKRLCRGLVGIDAEAGPTEIAILADATADPVHVAADLISQAEHDVLAASVLVTDSAQLADAVDAALTAQLTVVKHAHRVGEALRGKQSGTVLVDDIEQGLRVVNAYAAEHLEIQTTDAPAVAARVRSAGAVFVGAYAPVSLGDYCAGSNHVLPTAGCARHSSGLSVQTFLRGIHVVEYTEAALKDVAGHVVALANAEDLPAHGQAVQARFEALS

>CORE_REP|Org19_Gene3907#

MALNKASSNKARKVRARAASRRSSLVSRAALATASVALLTAGLTSACSSGDGSNPTGQNLGGRGPITYVEGKDTTETGAVKQLIDRWNAAHPNEQVTFKEQSNDASQQYDDLAQHMRAKQSDYDVMALDVPWTAEFAAKGWIQPLKDSFALDTSTLLSPTVASATYQGTLYAAPRNTNGGLLYYRKDLVPNPPKTWPELLADCNIARDHGIGCYAGQFAPYEGLTVNAAEVINAYGGSFVGPDGKTATVNSPQSRAGLQVLTDAYKNGDIPKEAISFKEPESQNAFASGKLLFLRTWPNFYGVAQADSSAVKGNFGVSPLPGKDGIGASTLGGYNAAISAYSKHKATALDFLRFLISEDAQRIVAEGAFPSVRSSMYDDPALIAKFPYLPALKDSIASAVPRPVTPFYPAVSKAIQDNAYAALTGKKSVDDAIIGMQKGIEAAGS

>CORE_REP|Org2_Gene44#

MVRARPADSVGRAQPVVTGRLAALAGVLALLVTFVVPSWIGVVVATAVLAAAVLFDLGTVGRAGDLGLSRPPLTTVRLGRATEVALVAVNNGAKPLRGTVWDDWPDSARAENRTHRLDLAPATLVRWHTTLTPAQRGDRVAGAVTVRLIGPLGLAGTQTRRRVPARVRALPQFRSERLLRSKVKRLQHLEGRNVANLRGQGTEFDSFREYVAGDDVRAIDWRATARATDVLVRTWRPERHRHMLMLLDTGRISAGRVGDGTRLDAAVEAALLLGGLAAAAGDSVDLLAFDRAPRAEVRGISGKGLQLKLMHAMAGVTPELVDTDSAGLVRAAIQRTRRRSLIVWFTSLDGAAVEENLLPVLPTLAQRHRVLIVSVTDPDIAAAAARRTDRADLYPAAAAETILAERALVQESLRRTGIAVVAASPDRLPEALADEYLELKQSGTM

>CORE_REP|Org92_Gene1865#

MVAHETALALIQLGAVFFGLGLLGRVAARIGMSPIPLYLLGGLAFGTGGLVELHQVDEFIHLASEIGVVLLLLLLGLEYSASELVTGLRKSWAAGVLDFALNATPGVLVAFALGWGFTGAIAMAGVTYISSSGIVAKVLNDLGRLGNRETPVILSILVFEDLVMAGYLPVLTAVLAGVGFVAGLQTLGIALAAVTVVLVVALRYGRFVSLIVDSKDREIFLLKLLGSALLVAGIASAVQVSAAVGAFLLGIAISGSTAHNATKLLEPLRDLFAALFFVLFGLSTDPATIPPVLGWALLLAVVTTATKVATGWWAAQRAGASRLGRARAGTALVAHGEFSIVIAGLAVTAGAVPAEFAALATTYVLLMAVAGPVAARVVEPVMGLLTARQRAARAAASPAAETVLEPGGPVAPRDRIAGAGLPEDGRRVPADRLDPDDESVGDPA

>CORE_REP|Org105_Gene421#

MNRQDRPRTRARAVVSLRAMSTPHTVAAEEHLPRDTADVVRTVRDSRQRAEPLTVCGGELADDGVSVPDQRAVVSMRRMNSVLDINLGRGTVRVQAGARLSEIDRRLGAHGLGLPIVGDHRDITAGGFASVGGVSTASHKYGMFIDQIVDLEYVDPDGRIGTCGRDHHTERFHRILGAGGRAGIITALTLDTVEVDKDHSWLSTNAHRFLDFDSFVEYAHGQVARPGEAVLQVGRWVDTAPLKVSRPVGTGHVQLGTVRFGQWSSLYPTAPTRSLRARREVGTRARKSLGVIASAAGGRAGMPVRNAAAGAVMLTPKVLTLRDAEYLADTVISSSERGPAYRVGVFAPLSSYTSVFYRLHDLFAGHRERTGCFTVISAMTYGVRSKFLRAESDTRGLPSEDHGLITFTCRLRPASLPSEQLRDIVTTIDEICRSENALRYESGE

>CORE_REP|Org162_Gene4624#

MPCDLPSKQPKECRFVAIIEQVGAREILDSRGNPTVEVEIALDDGTLTRAAVPSGASTGEHEAVELRDGGDRYQGKGVQKAVEGVLDEIAPAVIGLDAVEQRTVDQTLLDLDGTPDKSRLGANALLGVSLAVARAAAESSGLELFRYVGGPNAHVLPVPMMNIVNGGAHADTGVDVQEFMIAPIGAPTFKESLRWGAEVYHSLKSVLKSQGLSTGLGDEGGFAPDVAGTRAALDLIASAIEKAGYKLGTDVALALDVAATEFYTAGEGYKFEGSVRSAAQMNEFYSELLSAYPIVSIEDPLSEDDWDGWVALTDAIGDKVQLVGDDLFVTNPERLEEGIAKGAANALLVKVNQIGTLTETLDAVDLAHRNGYKTMMSHRSGETEDTTIADLAVAVGSGQIKTGAPARSERVAKYNQLLRIEDALGDSARYAGDVAFPRFAFEG

>CORE_REP|Org151_Gene2264#

MTDPTVSAVDPSQWSFETKQVHAGQAPDATTGARALPIYQTTSYAFRDTDHAAALFGLAEPGNIYTRIMNPTQDVVEQRVAALEGGVAALLLASGQAAETYAILNLAAAGDHIVSSPHLYGGTYNLFHYTLPKLGIEVSFVDDPDDLEQWRAAIRPNTKAFYGETIANPSSAIFDIPGIAAVAHAAGLPLLVDNTVATPYLIQPLAHGADIVVHSATKYLGGHGSAIAGVIVDGGTFDWTVTDAQGQSRYPGFTTPDPSYHGAVFADLGAPAFALKARVQLLRDLGAAVSPFNAFLISQGLETLSLRVERHVANATAVAEFLRTHPDVISVSYAGLPTSPWYERAKQLAPKGAGAIVAFELRGGVDAGKKFVDGLVLHSHVANIGDVRSLVIHPASTTHSQLTPDEQLRAGVTAGLVRLAVGIEGIDDILADLRAGFTAAAT

>CORE_REP|Org5_Gene7339#

MDFAARADDVPDPAWSFAAVGQRRGVADGRQGGGKRPRVGSGGANDYGRNMRNVAGIVLAVAAITLTGCSGGNSSNPATTSGAAPGTPSRTGATAATAPTTTAGEQNCAAGYLAQFSTRQKLAQLLTVGVKNAADAEQTVRDEQVGGIFVGSWTDQSMLADQQIEQVKAAAHTPLMVTIDEEGGRVSRLKNQLGPAPSAREAAQTMSADEYYQQSLARGQEMKKLGITVNFAPDVDVSDEPDDEVIGDRSYSEDPQVVTQFADAYIRAMHDAGLGAVMKHFPGHGHGSGDSHTGAVRTPPLDEMQQVDLVPFRNLIDSGAAVMVGHLDVPGLTDPNVPASISPQAMALLRQGTGYGAAPYNGPIFTDDLSGMAAITARMGIEEAVATALEAGADDALWISTDAVSSVLDRLEQEVRDGKLTMRQIDDSVLRVAKFKGIQLPC

>CORE_REP|Org105_Gene2713#

MINRGVVRMRNTKAVQAAEPEFVEVVIVGSGFGGLAAAKQLAKSGVPYVLISSTPEHLFQPLLYQVATGVLAADEIAPPIASILRRHEKADVRLGKVTAIDPDAAELVYETADGPRRIRYGSLIAATGANQSYFGRDDFAEKTFALKTIDDAKRLRAQIDHVFTQAKHADKETRERLLSFVVVGAGATGVEVAGQLAELAKRYYHQDVSVTLVEGAGEVLPPFGGGLSEYAKQSLTKGGVEVLLGTFVTDIEPGKVTVKDKQGVEHRIAAETVVWSAGVQASGFTKILAEATGAETDRAGRLLINPDLTVGGYADIYAIGDMTSLKGYPGQSPVAMQEGRHAADIIRRKKLPGTEFEYWDKGSMAVIRRRSAIAKVSDKIKFKGLIAWYMWLAVHLFYLVGFRNRFMAVMGWLVAFTGNGRPGFAEIDKDRPAVGHKPPIAA

>CORE_REP|Org1_Gene899#

MRDEPRSRPVSRSSRRCASDEGASIGDSTARHQGAANPPRRRSGKSNTERTTHVRDMVEIGMGRTARRTYELDDVDIVPSRRTRSSKQVSLAWQLDAYRFEIPLVAHPTDALVSPRSAVELGRLGGLGVINGEGLWARHADVETKIEQLTELAAQGRFDRAVALLQQLHAAPMQPDLLAAAVAEVRAAGVTVAVRVSPQNARTLTPALLQAGIDLLVVHGTIISAEHVGDGEPLNLKTFIAELDVPVVAGGVSDHRTALHLMRTGAAGVIVGYGSYPGATTTGEVLGIGVPMATAIADAAAARRDYLDETGGRYVHVIADGDIATSGQLAKAIACGADAAMLGVPLAVAAEAPGRGWYWPSAAAHPSVPRGSLLQVGDGWDLGAEDEADEAARPPLERVLFGPSDDPFGSLNLVGGLRRSMAKAGYSDLKEFQKVGLSVRA

>CORE_REP|Org5_Gene5326#

MSVATPGSPATPASAAAPARAGLTMAGQPLSSPLKDVWSLSRHMVGHFVENVAPCGTLPGDAIHGDITTITRTCLELAISLLDGRDIPEKTERLKDAAAAWAREGVPIDTIHHAIHEGFKIGFDLVVTNATTRNTTHGNSNGSATSDEPTLTLTHTDYTNLLTGAKLLVEILDTMTTAVSHAYVRELRAVVSEHHNAVHTLTSALLGGHPTSTMARECGIEIADNYHILALHIPPHPDQHNPHLDPTIIARRKLRRIQAALATNTHTTTLSLLSTDGGTLLIPTTHTTPTDLDPLITHLSHAARVPITATTTTTTTTDIPTATDQTHQLLDMVTRLESIPGLYRFDDLALEYQLTRPGPGRDHLGTLLNPLDHHPELLTTLQTHIANNLNRQRTARLLHVHTNTVDYRLKRIAQLTGFDPTQASGLWYLRSALVARTYATT

>CORE_REP|Org2_Gene7412#

MAAANYGLSMGADAVPARHGEGSDADGLDPDGPGSRLTVTSEVGTLRTVLLHRPGDELRRLTPRNNDQLLFDGIPWVERAQQEHDAFTGVLRERGVEVLLLADLLAETLAVSGAARIQGISAAVDARRLGHSLADQLAAFLRGVRARDLANILMAGMTFDELPFGPDATSLVRRMHHGADFVIDPLPNLLFTRDSSFWVGPRVAITSLALPARIRETSLTDLIYAFHPRFLGVRRAYESHTAPIEGGDVLLLGPGVVAVGVGERTTPAGAEALARSLFDDDLAHTVLVVPIAQNRATMHLDTVCTMVDQDALVMYPAVRDSLCAFTIERDEDYSARNGDGRVSMSGPDPFLVAAAKAMGIDKLRVIDTGLDGVTAEREQWDDGNNTLALAPGVVAAYERNEMTNARLEDAGIEVLRIPGSELGSGRGGPRCLSCPLSRDDL

>CORE_REP|Org101_Gene6166#

MATGARAELAHMTVSSMLDTSTNSRSVSLGRSGPSRWRRGIVAASKQSAYEGASDRILRRFGLAIGIAGVIVAVVELPEIAGQSRFLDAGWTIVTMVLAFGLFPVLAVVSISLSRQLIQLVAGAAAVSFLAALVVIPLSYPHPAPDVSSVWLYRVLALGVLAAVLAWRPLPAVAYLVVGSAVASLSNGLVVPHTTPLVWAGDFVRAAGLCALFLWCAIYAKAAADRVDRESEIESRRAAAVAGAAARDRERARFAALIHDAVLSTLLDASRAGTDSPVLRRQAEQTLEQLDECRVGEVEPNRLDAQSATGFLRSAVHEVNAGIRFTARRWSGFDDLRLPVDAAGTIAAALTEAVRNSLRHATVPGREVQRTVTVTISAGGIRVVFRDDGAGFDMSQVPADRLGISVSILGRMRQLAGGAGFVESQPGEGTTVTLVWGSDG

>CORE_REP|Org101_Gene6844#

MADPALRTDLDVPLHEFDPLVAELVGRELGRQQHGLEMIASENYAPLAVMQAQGTVLTNKYAEGYPGRRYYGGCEHVDELESLALTRLRALFGAEYANVQPHSGAQANAAVMHALLRPGDRILGLALDHGGHLTHGMKINFSGRLYDVAAYHVRAEDQLIDMAEVARLAREHRPKLIVAGWSAYPRHLDFAEFRRIADEVGAYLMVDMAHFAGLVAAGLHPSPVPHAHVVTSTTHKTLGGPRGGFILATAELGKKLDSAVFPGQQGGPLEHVIAAKAVAFKMAAEPAFRDRQERTLTGARLLADRLLAEDCRAAGIGLVSGGTDVHLVLVDLRAAELDGKQAEDLLHSVGITVNRNAVPFDPRPPIVSSGLRIGTPALAARGFDRAAFVEVADIIATALRVGRPWREFSVRVEVLTQKFPLYAGMRQHLPSATDAARELA

>CORE_REP|Org49_Gene64#

MRCVPRGAEERTSERPSVRRYRSAEAPRNPHLSRHRRRSGRGSELLCGSTCSSSILFGGTDTFGDRPPPRPHGGPSSPVSPITPAGDRCGCRRCLAQIQYTANRCADTWLRSAPAFAIVLTVDALASLLEGPRARGAFVMRSSFDPPWSLRIQDEAPLTVVAVVRGGGWIVPDARDGKPVAPCRLRTGDVAVFRGPDHYTVADDPGTAPRVIIHPGQITTTPDGEVLCETLSLGVRSWGTAADAETVLVTGTYEQESATGRRLLRALPPIVVLGSGEFDSRVLDLLVDEAAKDLPAQGAMLDRLLDLLTIAALRAWFARSDAPAWYHAYADPLVGKALRLMQHNPAHSWTVAALAAEVGVSRAALARRFTELVGEPPMAFLTEWRLALAADLLHESDATLEAIARRVGYGSAFALSTAFKRHFGVSPRDHRVAAARERTA

>CORE_REP|Org152_Gene4802#

MPVSVTAATAAGLCEFIDASPSPFHVCRTVAAELDDHGFTRLSESAPWPSSSAGRYYVVRGGSLVAWADGGPGAPSGFRGGQAAPFRVIGAHTDSPNLRVKQHPDLASAGWQLVGLEPYGGAWLNSWLDRDLGISGRLSVRDGNVVRERLIRIDEPILRVPQLAIHLSEDRGGVKLDPQRHVNAVWGVGVEPRSFLAFVAERSGIDPEAVLGWELMTHELVPAKLIGRDLDLVSAPRLDNQGTCYAGLRAFLAAIAEPGAAVPVLAMFDHEEVGSQSDRGAQSSLLPTVLERIVLSRGGGRAEYLAALAGSVCASGDMAHATHPNYPDRHEPMHRIEVNGGPVLKVNQNLRYATDATGAGAFALACSQADVPLQRYVHRADLPCGSTIGPFTAARTGMPTVDVGAAQLAMHSAREMMGAADVPAYAAALAAFLTPEIGR

>CORE_REP|Org26_Gene2801#

MRDGTTRLIGPGVNERAESPAGLLVEYPAGERAGDPLARGADGVLRYGNLTPALTELLDLQVHAFATREAVVEVGGPRLTYRDLWHSASRIAGGLQEHGIGYGDRVAVHLPVGARWVQAFLGALLSGAVPVLVHDGLPAAVAERVIADSGADFVLGGGARGCGSTRTGSVADTELPDGAAFIDDGAALDDLALLCYTSGTAPGPALPKGVELTNENLLSAIRSVVGALDLPTDGLRNLVLLPLAHASGCVDQLLPTFAVGGTVVLAPDTGRLAETLAAERIDMIAATPRILGALLPELAAERADGLRTEGVARISTAGHRAERAASGVDATELRAIFPDARQWAVWGATETSGIGLAVDDSLADPGGTVLGLPFGGTELALWGPRAGDGHGELLCRGPNVTRRYWNDPKATADRFTGSWFHTGDQVTIGADGLVRRSA

>CORE_REP|Org103_Gene5459#

MACDLRGTSRTRRWSRGRRPYDSGVSVPQAVLLAVLAAVVGLAVGGLLIPYVNARQAARRQADSGLTMSQVLDLIVLASESGIAVVDQYRDVVLVNPRAEELGLVRNRLLDERAWAAVEKVLATGESAEFDLTAKNPLPGRSRIAVRGVARPLSQEETGFTVLFADDDSEQARMEATRRDFVANVSHELKTPVGAMSLLAEALLESADDPEAVRHFGQRVLGESRRLGKMVTELIALSRLQGAEKLPELEVVDVDTVVMQAVDRSRTAAEAAGITVSTDRPSGLEVLGDETLLVTALSNLVENAIAYSPPGSHVSVSRSLRGKYVAMAVTDRGIGIAKEDQERVFERFFRSDKARSRATGGTGLGLAIVKHVAANHNGEITLWSKLGTGSTFTLRIPAHLEADSGDDDVDADGAAVSTKENGSRPSGPGRPNGVEARR

>CORE_REP|Org161_Gene3242#

MFVGKGGVGKTTLACASALAYARAGQDVLLASLDQAHSVGDAFGFRFPHDPGAVAGIVRVAPGLDVIELDSLALLEDRYREVVRMLSAGGTHTHDLGLDPGALEPAELTGLPGVQELLALTELAAFADEDDWDVLVVDCPPSADLLRIVSAPQTLLDYLDRLWPPHARAMSAAGPDPRRAILAATVARIVAAVTRVRDLLADHDRTGLRLVTVAERVAVAETRRVRSAAALLGLRLDAVVVNKMLPATPAPAGPLEAAHPAVHWYRNRRAEQQAVVDRLRAELDDVPVLLAQHSGPEPVGAEALSLLSYEPLDAPPPEHTARENARKRGIPDGEPDTRAESTRVGARSREPVVRWESGTGVDAVYALRMRLPVVDAATLRLGRVEDDLIVGADGVRRRVRLAPVLRRCTVEGAELDAGHLTVRFRPDPALWPAEGPVR

>CORE_REP|Org209_Gene2601#

MITAETNVFESLESNVRGYCRNWPTVFTTAKGAWLQDEDGKDYLDFFAGAGALNYGHNNPVLKQPLIDYIASDGITHGLDMSTAAKRKLLETLRDTVFAPRGLDYKVQFPGPTGANAVEAALKLARKVTGRETVLSFTNAFHGMTLGALSVTGNAAKRAGAGVPLVHAAHMPYDGYFDNTTADFQWMERVLDDTSSGFDRPAAVIVETVQGEGGINVARVEWLQHLAQLCAEREILLIVDDVQMGCGRTGPFFSFEVAGITPDIVTLSKSIGGYGLPLALVLFKPELDQWAPGEHNGTFRGNNPAFVTAQVALETFWSDGALEAATKAKGEKVATELATVAGHFPGLSTRGRGLVHGIAFEDPSQAGKVCQVAFERGLLVETSGSSDEVVKLLPPLTITDDELDQGLQILTGAIDTVCTGWGRLHHRAPAEGGDRR

>CORE_REP|Org80_Gene5458#

MRRRILRSMLTVLMLTTVALGVPLTYTAWLWVEDITRNDLQNRLERIAVEVIAQEREDGRVHDGLDLRTVRPLVPDDGKLTIIYPAPHDNASQVDIGPERVQDPLLESLSMGASGSLRLEVPSAPMHARQRQAVAVVGLAVLASLSAAVAVAVVTARRVADPLRDVAARAARLAMGDFRPDPRRHGIAELDRVSDVLDSATVEIAGRLQREHALVADVSHQLRSRLTAVRLRLDELSAHRDPDVVHEAEEAMAQVDRLTEAIDDLVRASRDEDATDRDPVPVMDELRGIVTEWTHPFTEAGRTLQLIGDDQLRAPITGSRLREAVAVLIDNALMHGGGTCTVSVRTVRPGGDREPLVCVEIADEGDGVSDELAPHIFDRGFSGAGSTGVGLALARALIEADGGRLELQRRRPALFAVFLGAPTATRQANGVVGEPR

>CORE_REP|Org94_Gene5589#

MRLRYWFRWLSAQGAPRLVLRTQARRGDPFARLVGGREGIEDPYPLIEQLRGDGGPVRTPLSWAAFDHELCRAILRDNRFGVRSPQSFTAFEPLKRLAARSPLPPNPVEPPSMLVIDPPEHTAMRKPVAAAFTPRAIGRLRDRVASVTTELLDALPSHGSVDLVAAYASQVPIAIISEMLGFPDADRQMFLGWGDRMTPLLDIGIPWRAHKRALLAMEVMNDYLDRHIARLRREPGDDILSALVTAGDLDDHELKASASLLMGAGFETTVNLIGNGVVQLLAHPDQLARLREEPDLWPNAVEEILRIDSPVQSTARTALTDVELDGALLRRGHTVVLSLAGANRDPKVFADPERFDVARPNAKDHLSFSSGIHVCLGASLARMEGVYALRALFERFPDLALAEPPHRRALFTLHGYERMPVHLGKRAAAREMSPLS

>CORE_REP|Org15_Gene2657#

MPAIVLIGAQWGDEGKGKATDLLGGRVQWVVRYQGGNNAGHTVVLPNGDNFALHLIPSGILTPGVTNVIGNGVVIDPGVLLDELAGLEQRSVDTSRLLLSADAHLIMPYHVAIDKVTERFLGNKKIGTTGRGIGPCYQDKVARVGVRVADVLDEKILTQKVEAALEFKNQVLVKIYNRRALDPQQVVDEVLNQAEGFKHRISDTRLLLNQALENGETVLLEGSQGTLLDVDHGTYPYVTSSNPTSGGAAVGAGVGPNKITTVLGILKAYTTRVGSGPFPTELFDQSGEYLAKTGGEVGVTTGRARRTGWFDAVIARYATRVNGITDYFLTKLDVLSSLDRVPICVAYEIDGERVEQMPTTQTEFHHAKPIYEEMPGWWEDISGARSFDDLPANARAYVERLEELSGARVSCIGVGPGRDETIVRHDILNEQLSSR

>CORE_REP|Org129_Gene4082#

MWDFRTEPDFQAKLDWMDTFVREECEPLDLLFPHIGQPYNTENAAARAILKPLQDRVREQGLWACHLGPDLGGQGYGQVKLALMNEILGRSMWAPTVFGTAAPDTGNAEILAMFGTAEQKSRYLQPLLDGDIVSCFSMTEPQAGADPKEFVCAARRDGDEWVISGEKWFSSNARYAAFFIVMAVTDQDASPYRRMSMFVVPAETPGIEIIRNVVVMPDREELDEGTHGYIRYNDVRVPADAILGGAGQGFEVAQARLGGGRVHHAMRTVGKCRRAFDMMAERVLSRHTQGELLADKQMVQQFIADSWIELAQFRLLVLQTAWIIDNEPHGTARTDIAMCKVAMAKIFADIISRAVQIHGSLGVTAELPLYEWWTSVPSLALADGPTEVHKATVAKQVLKGYRPAAGLFPSEHIPTRREAARARYADILKEHGLA

>CORE_REP|Org24_Gene2681#

MTATGHDSRLAGPHDSPLAEVAALDVARIRADFPILSRTVRDGKPLVYLDSGATAQRPTAVLDAERDFLVQRNAAVHRGAHQLAEEATDAYEGARADIARFVGVDADEIVFTKNATESLNLVTYSFADNRFPYRVGPGDEIVITELEHHANLVPWQELARRTGATLKWYGVTDDGRIDLDSLELSPATKVVAFTHQSNVTGAVAPVEELVRRAKAVGALVVLDACQSVPHMPVNFRELGIDYAAFSGHKMLGPSGVGVLYGRREILADTPPFITGGSMIETVFMEESTYAPPPQRFEAGVPMTSQVVGLGAAVRYLDAVGMEAVAAHEHALTEAALLGLGKLDGVRIIGPTENVNRGGAVAFVVDGVHAHDVGQILDDEGVAIRVGHHCAWPLHRRFGVAATARASFAVYNTLDEVDTLVAAVRKAQTFFGVA

>CORE_REP|Org100_Gene1857#

MDKLPGVSERFLVTGGNRLVGEVAVGGAKNSVLKLMAAALLAEGTTTITNCPDILDVPLMAEVLRGLGCEVTITDDAPGDRSVVTITTPAEPKYHADFPAVTQFRASVCVLGPLMARCKRAVVALPGGDAIGSRPLDMHQAGLRLLGATSEIEHGCVVARAEELRGARIRLDFPSVGATENILMAAVLAEGETVIDNAAREPDIVDLCNMLVQMGARISGAGTSVLTIQGVERLHPTEHRVIGDRIVAATWGIAAAMTMGDVRVTGVNPKHLALVLDKLRSAGARISFDVDGFRVVQPDRPRAVNFSTLPFPGFPTDLQPMAIGLAAIADGTSMITENIFEARFRFVEEMIRLGADARTDGHHAVVRGIPRLSSAPVWSSDIRAGAGLVLAGLVADGTTEVHDVFHIDRGYPNFVEQLQSLGGLVERVGGAE

>CORE_REP|Org185_Gene4863#

MLARIRHAADVAANTQVECVGPDRHGHTGAVSSPASTASHTDASVPVLRDFGGGPFGIYVHVPFCATRCGYCDFNTYTAGELGSSSSPQSWMTALRGELATAARQFAALPSATPEVATIFVGGGTPSLLGGDGLAEVLDAVRAEFTLAADAEITTESNPESTSPAFFERIRSAGYTRVSLGMQSAAQHVLAVLDRTHTPGRAVAAAKEARAAGFEHVNLDLIYGTPGERDSDLDASIDAVLEAGVDHVSAYSLIVEDGTALSRRVRRGELPAPDDDVLAARYERLDARLSAAGLTWYEVSNWAASDAARCRHNLGYWDGGDWLGAGPGAHSHLGGVRWWNVKHPARYADCVAEGGLPAAGWESLTDDERYLERIMLTVRLRTGLPMSDLHPGGKAKAMQIIADGRAALRDDHLVLTEQGRLLADGVVRDLVS

>CORE_REP|Org16_Gene2197#

MTGEKHTLATGDTQNAPENDRLIAVVGIGADGWPGLSPRVRDEIAAAQVLFGSRRQLDLIPADASIAQRRAWPTPLLPALPELLAAHRGSRICVLASGDPMFYGIGVTLANLLGPQAIRVYPQPSSATLACARLGWASAHTPVVSIVGRPLETVLPALADGRRLLVLSADEHSPAQVAELLRCNGFGESRLTVLEQLGGPAERVVAATAAQWSRPPGDPLNIVAVEAVRDPAAPRLTRLPGLPDASYGGDGQLTKAEVRTLSIAALAPAPGELLWDVGGGSGTIAIEWCRTHPDCRAITFERSAARRDQIAANAAALGVPAIVVRGEAPADLPAAGDPAPDAIFLGGGLTQDGLFATCWDRLRPGGRLVANAVTAESEALLLRWAATHGGELRKFQIYRGEPLGGFTAWRPHLPVAQWIAVKPADRSVSPE

>CORE_REP|Org49_Gene2684#

MPRQTQIGLMSHAELVSEHETQDANYAKLKTEKLTLDLTRGKPAPEQLDLSADLLSLPGADDYRDASGTDVRNYGGLHGLPELRAIFGELLNIPVENLLAGNNASLEIMHDMVVFAMLHGTADSERPWVQEPVRKFLCPAPGYDRHFAITQSLGFEMIPIPMRHDGPDVHAIAELVAADPTVKGLWAVPNYSNPTGVTFSEEVVRELVSMPTAAADFRLFWDNAYAVHPLTDTADPVLDVLGMAAAAGNPNRPFVFASTSKITFAGAGVSFVGASTANLGWYLKHAAKQSIGPDKVNQLRHLRFFKDAEGVRTHMQKHRAILEPKFALVLRILEDRLGASKVASWTEPKGGYFISLDVLEGTASRVVALAKDAGIALTPAGASFPYGRDPDDKNIRIAPSFPKESELEKAMDGLATCVLLAATEKLLADGK

>CORE_REP|Org123_Gene6248#

MAQDDRFTRAFDTAGSDFDRLGVHLWNPIGSATVAATAPRPGERVLDACCGTGASALPAARAVGAGGHVDAVDLSAALIGELARHAAALPQVRTHVADATTWPHDGYDVVQAVLGVFFFPDMAAGTERLISRARPGGRVGCTIWRRGSMVLAGKHLGNAIAAVTGTPVRERPEHPVDRIDNAEAFGSWLTERGLSDVTVVEHPLRLALTPELAWLIVTGSGFVGALADLDPDQVAAVHRRYLDSLACRRRHRHRRDHADRCRYSRCGKLPAYQGRYSGLMTVVKIVGDCAASAESAFRYVNDYRNLPRFLHGIQSFTPVGSRTEGVGAVFDGTMKLGPATLHSRVEVVRWEEGAAIGIKSIKGFDLESTFLFHPRGEDRSTVDAIVDYRVPGGLAGKALGRTIEPFVKIAVKHTNDNLLRQIAEFHARGAA

>CORE_REP|Org176_Gene7174#

MPEARAAGPVTGVAALPRASDGVAQRSGHEPDAGGHFGVYGGRHVPEALMAVIEEVTAEYEKSRLDDSFLNELDRLQRDYTGRPSPVFECTRLAEHAGGARILLKREDLNHTGSHKINNVLGQALLAKRMGKARVIAETGAGQHGVATATACALLGLECVVYMGAVDTARQALNVARMRLLGAEVVSVTSGSQTLKDAINEALRDWVTNAEDTYYCFGTAAGPHPFPMLVRDFQRIVGMEARAQVQASTGRLPDAVVACVGGGSNAIGIFHAFLDDADVRLIGYEAAGDGVDTGRHAATFTGGTPGAFQGAYSYLLQDEDGQTIESHSISAGLDYPGVGPEHAYLKDVGRAEYRPITDTEAMDALLLLSRSEGIIPAIESAHAVAGALQLGKELGPDAIILVNLSGRGDKDMDTAARWFGLFDTEPQEADQ

>CORE_REP|Org10_Gene4638#

MSASVKPRLAGRQPSSCGGVLRVMTGLPKVGGKRARRRFSRHDRVVRVTVSTDQSPCPSATGAELLPPPDGTLAIVPVGDIRLESGAVIPDVHLGVQRWGELSPGLDNVVLVEHALTGDSHVVGPADDVHQLPGWWNGMVGPGAPMDTDEWCVIATNVLGGCKGSTGPGSTAPDGKPWGSRFPAISIRDQVTAEAALFDRIGIHRLAAVVGGSMGGMRVLEWMVGAPERVAAALVLAVGARATADQIGTQTTQIAAITADPDWQGGDYHDTGRAPTTGMGIARRIAHLTYRTEDELDHRFANHAQDGEDPFDGGRWAVQSYLEHQAEKLCRRFDPATYVLLTEAMNRHDVGRGRGGVAAALAATPVPCVVGGVDSDRLYPLHTQQELADLLPGCARLEVVHSRDGHDGFLTETAAIGKLLVETMRLARAHR

>CORE_REP|Org126_Gene3874#

MRSGHDSALLRRRAALERQWARWVPSPRDLPASPAVCPLESGPAASDAPDADAPDAEAAQPRADVAQSWVRSRAFVDPATDCAPAVAGDVASKWADSPLRGPIAELSDQLHSITHDAGFVAAVTDEAGTILWSDGGQVMRRRAERVGFTPGGRWDENHMGTNALSLALHTGRPSSVFSAEHLVEALHGWVCYCAPIRAADGRQLGVLDLSTTWDRSHPLAMSTVLSLTAAVEAKLQGGVANTAPGVRLECLGGAALTRGGRPLRLRRRLLEILTLLALEPDGYTPERLQLAVYGDRPTGSSTLKADVSHLRRATGGDISNRVYRLTTPVSCDATDMLAALAAGDTATAVRLYRGPLLPGSQTPGVVEWREYLEVGIRTAVLSAGEAEYALDYGAKAPGDIEIHEHALRLLPARDARRAVAVARLHSALRS

>CORE_REP|Org5_Gene4512#

MRVMSIVSGSWPHRPDTPGDARKVAEPLAESDLERVTWLAMTALLAERAAEVVSAAQQLLTKRMGAAVKLSDPIELSGSGRTTVLRVRVAENSFSLPRTLIVKQVRGAAQERRIGGLAPGVASIDSAFLREAVSYQFTTALSSEHRPGAYLLAHSLPDRLLILSDLGENMSLTSVLQSGAEPATRNAQMAFAQALGRMHAATVGREADFVALLRRADVGRRVDGIAQQAEASVGEVPGMLAREVGIEVPGEIAERIVRGNRLFAGGRFRAFSPSDLCPDNVILNDEGARFLDYEWGGFRDATLDIAYALVSFPGCLCDFELSRERAQQMVEAWRSEVVGVWPALADDDLLAERILEARLIWVWLTTYWFLPADHSRIAAAREHGLSVPRSAALINRWAALAEDARCTGDDTLGDFAEHVSATLEELWEG

>CORE_REP|Org4_Gene6788#

MIGSNDPAPPATTHTPGHGVVHGDDHPSGDLLERAAARADPPDRAADEVTSTPALHTSDHSGVSHDVQMPEEPDATNPEPPSESTAAEQTAQSRPQDVSDTEAAGVSSAARVPGDAGASVDAAVPVDAQASGGAAGPGVGVGAGDSVAGGAQETEQKSGFHLRLSNFQGPFDLLLTLISSRKLDVTEVALHQVTDEFIAYTKALTAALSDDATTTLRADKILDQTTEFLVVAATLLDLKAARLLPSGEMTDAEDLELLEARDLLFARLLQYRAFKQVAELLGELEAVALRRYPRAVGLEERFADLLPEVTLGVDAHEFAAIAAAAFRPRPVPKVGLDHLHNHAVSIAEQAALVLERLKLAGKGGWTTFTELVADCTVPVEIVARFLALLELYRGKTIEFDQPDPLGPLSISWIGDDVQTSTVTIEEDYG

>CORE_REP|Org132_Gene5347#

MQAALGKGSRRDPLTAIRESGRGTKIALALGTVAVVVLAGVLWWVFSSYNTTRITAYFDKSIGIYEGSEVRILGVPVGKVDSVTPQGDQVKVTMHVDRKYDIPADAKAAQITPSVVSDRYIQLTPVYKGGPKMPRNATIPRDRTATPVEVDRLYKSIQELSDALGPNGANKDGAVNELVRTGAANLTGNGDALANSLTQLSHAARYLSDARGDIFDTIKNLQVFVHTLAVNDQQVREFNTQLADLAGFLSGERENLGQALNLLSIALGDVARFIDNNRDLVADNADALTKLTQTLADQRQDVANALPVLPLALSNLINIHNGESGTLDMRANFTDLQNPFGAVCKMLDLGQLRPGDPKFDAISRQMRPILDQCKVITDQIKDGVQTPSLVLPFGILSGENIQKTPAPGSVPGTPSDRQPPSQQEGGQR

>CORE_REP|Org12_Gene1520#

MFEWSETDEMIRAAVRAFIDKEIRPNLDALDSGAMPPYPILRKLFGEFGIDVMGAEAIEKLLAKQRAAEAAPDAAQRDKKLRSGGDPFGEQQSLMAVLISELSGVSMGLVAAMGVSIGLGAATIMSRGTLAQKERWLADIVTLKKIAAWAITEPDSGSDAFGGMKTSVKRDGEDYILNGQKTFITNGPYADVVVVYAKLDEGDGGAGGRSAGSAPIDKRDRKVLTFVLDKGMEGFTQGKPFKKMGLHSSPTGELFFDNVRVGKDRLLGETEEHKGGDGRESARTSFVAERVGVGFMALGIINECHRLCVEYAKTRVLWGQEIGRFQLVQLKLAKMEIARINVQNMVFNTLERGRAGKPPTLAEASAIKLYCSETATEVAMEAVQLFGGNGYMQEYRVEQLARDAKSLMIYAGSNEIQVTHIAKGLLGR

>CORE_REP|Org49_Gene558#

MPRPHGDGRGNAPPPVAQRRRNDHGRPGRRACGRDHSPTDATIDLVTTSLETAASSLRIGPYPVDPPVVLAPMAGITNVAFRTLCREFGSATSIYVCEMITARAVVERNEKTLHMMSFGPDESPRSMQLYGVDPKTLGEAVRIVVGEGWADHIDLNLGCPVPKVTRLGGGAALPYKRELFRAIVREMVAAAAPAGVPITVKFRIGIDDDHITYLDTGRIAEAEGAAAVALHARTAAQRYSGEADWTAIARLKEAVTTIPVLGNGDIFSADDAVTMMAKTGCDGVVVGRGCLGRPWLFAELEAALRGEPVPAPPNLGKVGEILYRHASLLADHDGEDKGMRDLRKHMAWYLMGFPVGSELRRRFATVGSLAELSDLIGQLDPTAPFPKDAEGPRGRQGSPGKVALPHGWLDDPDDCAVPTAADVMHSGG

>CORE_REP|Org78_Gene5482#

MALTPDQITAIDAAHVWHPYGGFPATTEPLVVASASGVRLTLADGRELVDGMSSWWAAVHGYRHPVLDAALVAQSQRMSHVMFGGLTHEPAARLTELLVQLTPEGLDKVFLCDSGSVSVEVAVKMCLQYWRSLGKPGKRRLLTWRGGYHGDTFTPMSVCDPEGGMHALWTDALAEQVFVGMPPAEYRPGYVAELEAALAAHADELAAVVVEPVVQGAGGMRFHDPRYLADLRRLCDAHDVLLVFDEIATGFGRTGELFAAEHAGVRPDVMCVGKALTGGYLTLAAALCTTRIAETISAAHGGLMHGPTFMGNPLACAVAVASVELLLARDWRGEVRGIEDGLRAGLAPVRDLPGVVDVRVLGAIGVVELDRPVDMRAATAAAVAAGVWLRPFRNLVYTMPPFISTAADVAAITRGIAAAVAAGDVPS

>CORE_REP|Org39_Gene1705#

MARIGDGGDLLKCSFCGKSQKQVKKLIAGPGVYICDECIDLCNEIIEEELAESSEVKLDELPKPTEIRDFLEQYVIGQDAAKRNLAVAVYNHYKRIQAGDKGRDPRGEPVELAKSNILMLGPTGCGKTYLAQTLAKMLNVPFAIADATALTEAGYVGEDVENILLKLIQAADYDVKRAETGIIYIDEVDKIARKSENPSITRDVSGEGVQQALLKILEGTQASVPPQGGRKHPHQEFIQIDTTNVLFIVAGAFAGLEKIVQDRIGKRGIGFGAEVRSKAEVDTTDHFAEVMPEDLIKFGLIPEFIGRLPVVASVTNLDKESLVRILAEPKNALVKQYVRLFEMDGVDLEFTQDALEAVADQAILRGTGARGLRAIMEEVLLPTMYDIPSRDDVAKVVVTADTVNDNVLPTIVPRKRQQGPERREKSA

>CORE_REP|Org118_Gene827#

MQLRGGGTIEQVSPTSQSPHQPPRILEQSLKLQNVLYEIRGPVHAHAARLEAEGHRILKLNIGNPAPFGFDAPDVIMRDMIAALPYAQGYSESKGILPARRAIVTRYELVPGFPEFDVDDVYLGNGVSELITMTMQALLDSGDEVLIPAPDYPLWTAMTSLAGGTAVHYLCDESNGWQPDVADIESKITDKTKALLVINPNNPTGAVYSSEVLQQLVDLARKHQLLLLADEIYDKILYDDTKHISLASLAPDLLCLTFNGLSKAYRVAGYRSGWLVITGPKEHAAGFLEGIDLLASTRLCPNVPAQHAIQVALGGYQSIEDLILPGGRLLEQRDVAWEKLNMIPGVSCVKPKGALYAFPRLDPEVHDIHDDGKLVLDLLLQEKILMVQGTGFNWPQHDHLRIVTLPWARDLAVAIERFGNFLSSYRQ

>CORE_REP|Org5_Gene1930#

MSEAKNTGPRRDTETLSHLDDEGRARMVDVSAKAKTARTAVAAGVLRTTPEVVALVRADDMPKADVLATARIAGIAGAKKTSELIPLCHQLALSSVHVRFDFTDDAITIEARAKTKGPTGVEMEALTAVAIAGLTLHDMVKAVDPAAVLDGVRLLTKDGGKHGHWERLGEPVDHATSGRSSHAHDHTRPGQAQTAAPSPGASADTPAGHGPSGRPHDSAHGAMEPTPAPLATAAHSGRPSEPVDDAAAHSGHAHGRGPGTHSGVARAGEGSRSAVVVVASTGAAAGTRVDTTGPVLMDWLAGLGFSVRGPLVYADAEIAAGLRDALEGAPGLVITTGGTGAAPSDATPEATLAVLDRELPGVAESIRQRGTAAFPLAALSRGVAGLSGATVIVNLPGSPGGVRDGIAVLEPLLDHLLAQVAGGGRHE

>CORE_REP|Org217_Gene187#

MVAEHGEVDLAAARPDSGPTAAAAPAGNGSGATANRSGGEAGVRRRPKDRKAQIVRAAARAFSERGYYPVGVDEIAAEVGISGPALYRHFANKYALLVAAAEEGARHLLQVAQAADDPALDPEPRLDAVIKAISEHTIDIRREAGLYRWERRYLEREDRLRIRRIYDELNDTIAAPIARLRPGADPADLRMLSAAVMSAVASIAAHRTALSGARLLPLLRDMCWAILRTELPPAPVDTEDEPAPRGLPVTSKREQLLTEAIRIFGRQGYHEASIEEIGAAVGINASSVYRYFSSKADLLAAAFHRTGDRVSVAITEALAEATSRPDAVRRIAARQAKLTFAMPEIMPVYYAEFSNLPQAEQHKLRAIQRQNVLEWANLLDGDPIEARFRVHAAIGQVIDVGRLIRFDSRPAQLARVTALMEAVLLG

>CORE_REP|Org162_Gene3635#

MNSPRSRVLASGIRRRGSAFGRRVAAVATALGAVTLLAGCGDGAYSIPLPGGPSVGDDPLHLDIRFADVLDLVPQSTVKVDGVAVGRVDAIELAPDGWTASVKAVVRNTVDLPANARAEVRQTNLLGEKFIELSVPTHDPAPARLADNAVIPVANTRTATEVEQVLGALSLLLNGGGVAQLQPIVVELNKALEGRESTVRDLLEQANTLIDGLNRQVDDITRALDGLDTLSSRLSGQTTQISQVLDELPTGIRILEEQRPQLIQLLGQLDRVGQAGFDVLDNSKDDLIRDLNSLRPTLQALGSGADDLVTAFPLIPTYPFPDEAIKSAFGGQVNTWLSVDLQIGTLLSNLGVGKQDPVYIPPNGRPVPVDGSNPYYNGNGPRPGWPTVSLLPLPPTVVRPQLPAGLPIPTDPLGSLLDQLGVGGPR

>CORE_REP|Org105_Gene2202#

MPLITMDRFPGRERLHGKMDGVLRWITAGESHGPALVAILDGMVAGVEVTSDEISAQLARRRLGYGRGARMKFEADKVTIVGGVRHGRTMGGPVAIEVANSEWPKWTTVMSADPVDPAELADLARNAPLTRPRPGHADYSGMLKYGFDDARPVLERASARETAARVAAGTVARAFLRQAFGVEVVSHVISIGTAANTTGHVPTAADLDAIDASPVRAFDAEAEAAMIAEIEAAKKDGDTLGGVVEVVVEGLPVGLGSFTSGENRLDSRLAAALMGIQAIKGVEVGDGFETARRRGSQAHDEMKPGPDGVLRSTNRAGGLEGGMTNGEALRVRAAMKPISTVPRALSTVDMSSGEEAVAIHQRSDVCAVPAAGVVAESMVALVLAQAALEKFGGDSLTETCDNITSYVKRISSRPHVAPTDADSRAR

>CORE_REP|Org7_Gene3766#

MRERGGVVQNYSEPDYERLVVERPKTAGLGAPGSEFEVGHRTEFARDRARVLHSAALRRLADKTQVMGPRDGDTPRTRLTHSLEVAQIGRSIGEGLGCDPDLVDLAGLAHDIGHPPYGHNGEKALDHFADAHGGFEGNAQNLRILTRLEPKVLDPAGVSAGLNLTRASLDAALKYPWGRTGPGTKFGAYDIDAERLAWIRKGAPERRRSLECQIMDWADDVAYSVHDVEDGVIAGRIDLRALADPWEQEALASLGRHKHYSLSAEELVAAAQRLSELPVVAAVPAYDGTLASSVALKRLTSELVGRFATGAITATRETAGTGPLSRYGADLEVPLIAAAEVAVLKTVALHYVMSDRDHKLRQAGQRDQIQAVATRLLATAPNGLDPLLLPWWHAAADDTARVRVIVDQIASYTESRLERVAALLGV

>CORE_REP|Org66_Gene1451#

MLVTSTDSVSGANLAKDKPEPASGTLERDVQTLEKAIYEVKRVIVGQDRLVERLLVGVLARGHVLLEGVPGIAKTLAVETFARVVGGSFSRVQFTPDLVPTDLIGTRIYRQGREEFDTELGPVVANFVLADEINRAPAKVQSALLEVMAERHVSIGGKTYPMPDPFLVMATQNPIESEGVYPLPEAQRDRFLFKVVVDYPSVEEEREIIYRMGVTPPEAKQILGPEDLIRLQKVAANTFVHHALVDYVVRVIAATRKPLDYGMADVANWIAYGASPRASLGIIAAARAVALIRGRDYVVPQDVVEVIPDVLRHRLVLSYDALADEVSPEDVIRRVLQTVGLPQVAPQAVPAGAQAAPAGPPQQHQQQIPQPPAQPGQGQQGQGQPGQGQQGQGQPGQGQPGQQGQGAPNQGAQAPMAPAGTNQPK

>CORE_REP|Org158_Gene2874#

MRHRDTSTATEAELRWRPAPLVYMLAVASAPALVLAIVLGKWQLVVFAAPMLGVLATAPLQQSRTRIQVDGAGILRCFETEEVTLAVATFVESGHALLRLHPEPIPGMEMRVEEAVDSGTAPAGLRVALSSPRWGRFPVPIRVSALSPAGLAVASVRLPAGEVFVYPIADPQRMRLPRTELPERIGTHLTRRHGPGVEFADVRAYAPGDQLRTVNWPVSARRGRLFVTERFTNRAADVVVLVDTSLQAPGPASDSLELSVRGAAQVAQSALQAGDRTAVVCLGKSPRWLRPDIGRRQFYRIVDAVLDVGEEHIPTSGTLAPHTAVPIGAIVVAFSTLLDTQFALALIDLRKRGHVVVVVDVLRGAPFADGLDHTLARMWQLERASMYRDMGTVGVDIVAWPEGTRLDQVMRLIPEHRRTVRVRR

>CORE_REP|Org102_Gene2841#

MPAIPGRQPAGTGPRGTMTILSNVSARSFDSSAMPTVTMIGGGQLARMTHQAAVALGQRLRVLAERPDDPAAQVTPEVVLGTHTDLAALRKAAVGSHAVTFDHEHVPTEHLEALIAEGVNVQPPPGALVYAQDKLAMRRKLAELGVPVPVFTAVASAADAVAFGDEHGWPVVLKAVRGGYDGRGVWMPADAAEATRIADDQLAHGVALLAEAKVDLKRELSAMVARSPFGQAAVWPVVETVQRNGQCAVVIAPAPELPEQRATEASALALRLASELGTTGAMAVELFETHAGELLVNELAMRPHNSGHWSMDGAVTGQFEQHLRAVLDYPLGDTAPLAPVTVMANILGAPEAPAMSMDERLHHLFARMPDARVHLYGKGERPDRKIGHINILGDDVAATREQAERAAHWMSHAVWTDGWDPHHE

>CORE_REP|Org4_Gene4198#

MLLSDRDIRAEIAAGRLGVEPLLETLIQPSSIDVRLDGMFRVFNNTRYTHIDPAQQQDELTSLVEPAEGEPFVLHPGEFVLGSTLEVCTLPDDLAGRLEGKSSLGRLGLLTHSTAGFIDPGFSGHITLELSNVANLPITLWPGMKIGQLCLLRLTSPAEHPYGSATAGSKYQGQRGPTPPAPTSTSPSPPARSPTATPSAYCHSAGQRGKTVRDRRSPLVGHRTLGVVVGAVCALGVSTACSDTDNAAGLTTSKSATSAVAAPGAVRVAVPPAADGVVPRVVAFDPCFRVDDSRIAEAGFDPASRERNATEVTAMSSLTKIGCSFRGLSSGAQVADFLSITTSTETLPEVTGSARNEVVETTSIGQRPTTIYRSMPAACDAAVESPDGALQISLIVPPGAAEAPKACDRIRDVASKIAVALDVG

>CORE_REP|Org142_Gene3706#

MASGDIVPIELGLTDGDLVTLWAPRWRDGDDEWEAFLGHEDALYGFESVAELAAFIRTDSDNDLVDHPAWKVVAGLSAVELEPEENFTFDLIGVPELVASDPDAETVAELEDTLAMVRNIGEVCELEVVTKFFGSHPVLGALPGGVNAFIGRDGEELWDQIGAAVAKDWDAVVDAIDSVVQTPDVDAEAVSVAEAELLAAEENVIDADDAADTDSDSDYEPVDLTDDSNEDEEDEEDDVEDDSLWHEVGIDPIKIVTGEGTYFTLRCYLDDEPIFLGTKGAIAVFGTERALARYLADDHEHDLARVSTFSEVQTAAVDGSLEVEVTDENVYVLPGLADDLGDGPESVDIEQLDLAVELFTDAADYADDDAVEQGLAQSTPLGWYVSYLLNPDPSRMAPNPPFTAEAQAWRELERNFEARLNQK

>CORE_REP|Org144_Gene454#

MADISVRGRIALRAAAAASWASQKAGRGKGSMIGGLIALQIDKTIMDQLGRGKRTVLITGTNGKSTTTRMTTAALGTLGAVATQADGANMDAGIVAALSVHRGAPLAAIEVDELHLPHVTDSLNPAAVVLLNLSRDQLDRVGEINMIERKLRAGLARHPATVVIANCDDVLVTSIAYDHPNVVWVAAGSGWSMDATSCPRSGEPIVWEDAPAGARGGEAGKHWRSTGADFARPEPDWWLEGNDLVGPDGVRLPLELALPGRANRGNAAQAVAAAVALGAGAADAVTATGTVREIAGRYRTVQVGDHAARLLLAKNPAGWQEALSMIEPAAAGLVIAVNGQVPDGEDLSWLWDVRFEHFEGVQVVASGERATDLAVRLTYAGVEHTTVSNPVRAIASCPAGHVEVLANYTAFRDLNRDLDGRTA

>CORE_REP|Org158_Gene6393#

MRWLWTVLRQGVIVDLDWSPADLAFRDEVRSFLDEKLTPDLRRAGQLATSVYPDHEASMRWQHILHERGWAAPAWPVRHGGCDWSLTQHYIFSRECTLAGAPNLSPMGIRMVAHAIMAFGTEEQQNYFLPGILTGEVFFCQGYSEPESGSDLASLSMAAVDDGDDFIVTGSKIWTTHATEANWIFALVRTSKQAKKQQGITFLLIDMRTPGIEIRPLVMTSGEQVQNQVFFDQVRVPKKNVLGQIDDGWTVAKYLLIFERGGGAAAPALQVMAEELAQAAATQPGPDGTPLIDDPGFARRLADARIRADVLEVLEYRTLSAISRGKDPGSAASTIKILGTELSQVLTELALEAAGPRGRAYQPHATMPGGPVVDFTAPADGFVSGEEWQAVAPLRYFNDRAGSIYAGSNEIQRNIIAKATLGL

>CORE_REP|Org14_Gene597#

MTAGTVGEVDTVREAVHEAARRARVASRTLAQLTTAQKDAALHAAADALLAAKDAVLAANAEDIAIAEAGGTAASLLDRLRLTEPRIDGIASGLRQVAGLPDPVGEVLRGSTLANGLEIRQVRVPLGVVGMVYEARPNVTVDAFGLALKSGNAALLRGSSSAARSNAALVEVMREALVAQGLAADAVQLLPSEDRSSVTHLIQARGLVDVVIPRGGAGLINAVVRDARVPTIETGTGNCHVYVHAAADLEMAESILLNSKTRRPSVCNTAETVLIDRAIAETAVPRLIDALERAQVTIHGDLPGLVPATEEDWADEYLSLDIALKVVDGLDAAVDHINEWGTGHTEAIVTADLKAAREFTARVDAAAVMVNASTAFTDGEQFGFGAEIGISTQKLHARGPMALPELTSTKWIVWGDGQIRPS

>CORE_REP|Org119_Gene4741#

MHELVGALRSYAWGSRTALAQLCGRPVPSAHPEAELWFGAHPADPAHVRIADHTTSLLDFVAADPIRELGPAAAEFGGKLPFLLKILAAEEPLSLQAHPSAAQARAGFERENRTNVALDSPMRNYRDDNHKPELVVALDRFEALAGFRNPRRTVELLRALQVPGLESYANLLAAQPDSDGLRTLFTTWITLPQPVLATLLPAVLDGCVRYLSGKGKREFTAEARTALELAEAYPGDAGVLAALLLNRLTLEPGQGLFLAAGNLHAYLRGLGVEIMANSDNVLRGGLTPKHVDVPELLRVLDFEPIDLPVVLPEPAGDGSVRYATPAPEFALRRFDLVAGSGQVPLTAAGPGIVLCTAGSVRLLQGTTELALQRGAAAWISATDTDIRAQAPDGDAQLFCACVGGVGAAGLPATLTGTDQHRG

>CORE_REP|Org109_Gene6011#

MFVRTLSVRDFRSWEYAELELSPGRTVFLGSNGNGKTNLLEAIGYLATLGSHRVATEAPLIRTGTERARIGATVVNAGRELRIDVELNQGSPNRAQINRSPVRRTREILGILQTVLFAPEDLALVRGDPGERRRFMDELCTTRLPRLAAVRGDYDRVLRQRSALLKTAGRQARSKADLSTLDVWDGHLAEHAAVLLAQRLRLVHDLAPYLARSYASIAPESRPASIVYRSAALPPEFLDPARPPRPEDTGELEAIVLRELAAARPKELERGVCLVGPHRDELDLMLGDSPAKGFASHGESWSFALALRLGAFELLRATGAEPVLLLDDVFAELDRRRRAALAAVAADAEQVLITAAVPEDVPAELAAVPLRVETAGGPDSRVSRIVAPGWSESGEPGNDDAEYPPLGREGDPLTGAPESRTP

>CORE_REP|Org132_Gene1794#

MPVENVAVAGEARIPAVNKGELFASFDVAAFEVPSAHDEAWRFTPLRRLRGLHDGTAVRDGRAGIEVAVSDGDARGASAVTQAAASGPDASTAAVDGSVIDGVTVETVGRDDARLGEGGVPTDRVAAQAYSGFEQATVVSVGAETEVDRPVVVRVTGPGADKTAFGHLQIRLGNFAAATVVIDQRGSGTYAENVEFVLGDSAKLTVVAVQDWDDDAVHVTAHHAKLGRDAVLRHTDVTLGGDLVRLTATVRYDGPGGDAELLGLYFADDGQHFEQRLLVDHAQPHCKSNVLYKGALQGDPSSAKPDAHTVWVGDVLIRAEAEGTDTYEANRNLVLTDGARADSVPNLEIETGEILGAGHASATGRFDDEQLFYLRARGIPEEAARRLVVRGFFHEIIQKIAVVEIRERLESAIETELAAIGA

>CORE_REP|Org25_Gene3927#

MKILMVSWEYPPVVVGGLGRHVHHLAIELAAAGHEVVVLARRPTGTDPSTHPTHSYIADGVLVVAVAEDPPFFDFGEDMLAWTLAMGHAMVRAGVALGKPGIGDGWTPDVVHAHDWLVAHPAIALAEYYDVPLVSTIHATEAGRHSGWVSGRVNRQVHSVEWWLANESDALITCSASMQDEVERLYGPDRIPLTVIRNGIDVGAWTFRPRPPRSGPPRLLYVGRLEYEKGVQDAIAALPRIRRAHPGTTLTIAGVGTQFEWLRERARGHRVARAVTFAGQLDHTELLGWLHGADAIVLPSRYEPFGIVALEAAAAGTPLVTSTAGGLGEAVIDGVTGASFEPADVDGLVQTVRAVLDDPAAAQERAYAARERLTADFAWDVVAAETAQVYTAAKRRVRSPLGRPVITMRPLPERDPGQPV

>CORE_REP|Org216_Gene4894#

MIDLRLLREDPNAVRASQRARGEDPALVDALLEADAARRAAVATADNLRAEQKAMSKQIGKAPKEERSALLARAQELSVKVKEAEAAQHAADADLDAAHRALSNVVLPAVPAGGEDDYVVLETVGTPPEFDFEPKDHLELGEALGLMDMERGAKVSGSRFYFLTGHGALLQLGLLQLAAQKAVANGFTMMIPPVLVRPEVMAGTGFLGRHAAEVYHLADDDMYLVGTSEVPLAGYHADEILDLSAGPKRYAGWSSCFRREAGSYGKDTRGIIRVHQFDKVEMFVYTTPDQAEAEHERLLAWEREMLAAIEVPYRIIDVAAGDLGSSAARKFDCEAWVPSQQTYRELTSTSNCTTFQARRLSVRYRDENGKPQIAATLNGTLATTRWIVAILENHQRADGSVRVPAALVPFVGTDVLRPPA

>CORE_REP|Org215_Gene3561#

MSKLVRYQLIAFGLIAVLGVVFVGAKYVRLDNMLGFGQYRVQVRIVDTDDKRATTGNLSPGAEVTYRGVPVGRVGKQEIIPDGVLITLELDSGAPKVPQSAKAVVANRSAIGEQYVDLVPSSAGAPYLRDGSVIDGARTPIPVEDLLASVNHFASTTDLVALSTTITELGKAFDGKGDELQVLVDSLARFTETGVDALPQTLQLIRDAQTVLTTQAEQSPAIRQFSDGLDRLSAQLRSSDPDVRRLIGTGTDAGSQISQLLRESGDALTRDLANLRTLLLTISPKFYALGPVLQMLPLLSIGASATAPGDGTTHFGLVLETNNPPACTVGYEGTQRILDEMRAQNPDFDDSRDDFPFNTEAKCLVPQGNPTAVRGGERAEFADPSVPQPWDDNPKVDPEKLNLNPVATQLATLLGVTPKR

>CORE_REP|Org12_Gene4586#

MRTLDTSFSTRNGGFREVVVTAVEITTSIGADTESTWQALLSGASGIKVLTDEDITRHDLPNAIGGKLIHDPTADLDRVRKRRMCYVQQMSYAMGQRLWETAGAPEVDKDRLGVCIGTGLGGADVIVEANDTMREHGYRKVSPFAVPMSMPNGVSGVVGLDIGARASLVTPVSACASGNEALVHAWRSIVLGDADMVVAGGVEGYINPMAIAGFTMARALSSRVDEPERASRPFDRDRDGFVFGEAAALLLVESEEHARARGATPLARLLGAGLTADGYHMVAPDPEGLGCARAMRRAIETAGVSAADVDHVNAHATGTSIGDLAEAKGIAAAIGTHPAVYAPKSALGHSVGAVGALEAAISVLTLRDQVIPPTLNLDNQDPEIDLDIVHDKPRHTDVEFAMNNSFGFGGHNAAVLFGRY

>CORE_REP|Org19_Gene113#

MNTPSFVIVGASLAGAKAARALRDNGFDGSLTLIGDELWYPYERPPLSKDYLQGKVDRDTVFVHPPRWYTTHEVDLRLDTTATAIDRGNHLLTLGNGEQLPYDKLLLTTGASPRRLPLPGADAHGVYYLRSLDDSNRLRELLHTASRIALVGGGWIGLEVAAAARAAGVEVTVVERSPLPLQAVLGPDIAAVFADLHRDHGANLHLAATLAEITTCDGAATGLRLADGSRIDADAVIVGIGATPNTGLAGDSGLDVDNGIVVDPALRSSDPDIFAAGDVANAYHPFYSRHIRVEHWANALHQPDTAAATMLGRDASYQRLPYFYTDQYELGMEYTGYCEPDQHYQVVVRGDLQERRFIAFWLRDDRVAAGMNVNIWDVTEPIQTLIRTRTVVDPARLADPDVPLTDLTTPASSRTERDMN

>CORE_REP|Org23_Gene581#

MREHMADSREFDLILFGATGFVGKLTAQYLLGAAPADAKIALAGRSVDKLTRVRDELGPAAAAWELVVADAADQPALDALAARTTVVVTTVGPYLRYGLPLVAACAKAGTHYADLTGEPLFIREAIDRYHEEAVLTGAKIVNSCGYDSVPSDLSVYQLYRRTVEDNTGELTDTTLIASLKGGVSGGTIDSGRAMMEAVAADPSKQSVLSHPYALSPDKSMDPDVGRQSDQALQRASSIDPSLDGWVGTFVMAMHNTKIVRRSNGLLGWVYGKNFRYREVMSAGKSPVAPLVAAGMSGGIVATMAAGALLSRVSFGRKLLDRVLPKPGTGPSEQARTSGWFTMKTYAHTTSGAKYVATFAGQGDPGYQATAVLLGESGLCLAFDGAKLPELAGVLTPAAAMGDALTERLRAAGMTIEVERG

>CORE_REP|Org181_Gene5629#

MKGTVVTTAQVPDVLAPADLGPVRLRNRIIKSATFEHMAPGALVSDQLVEFHREVAAGGVGMTTVAYCAVAPEGRTEADQIWMREAAVPGLRRLTDAVHAEGAAASAQIGHAGPVANAKSNGLPALAASRRISPMGPQLTHAATMADIARIRAAHGEATRLAIESGFDAVEIHFGHNYFASSFLSPVLNKRTDAYGGPLRNRARLIREVAAEVREASGGRIAVLAKLNMDDGVPGGFWLDEAAQVAQWLEADGSLDAIELTIGSSLLNPMYLFKGEAPLREFAEVMSPVMKVGVKVAGKLFLHTYPYRDLFMLEAARQIRAAVSMPLVLLGGITDKAAMDTAMAEGFEFVAMARALLREPDLINKIQADHEAKSLCIHCNKCMPTIFYDTRCVLRTRPTPVPLADPVRAGTPVAGNGVSR

>CORE_REP|Org15_Gene4950#

MSTEQLQQRWSSALMNNYGTPKVALVRGSGAVVYDAEGKRYVDFLGGIAVNSLGHAHPAILEAVAQQLATLGHVSNLYVSEPVLELAERLLAHFGDGTGPIGQGSEEAAGVTTEGPRSGRIGTARAFFCNSGTEANEAAFKIARLTGRHTIVACEEAFHGRTMGALALTGQPSKRAPFEPMPPGVVHVPYGDAAALAAAVDSDTAAVFLEPIMGESGVIVPPPDYLAEARRITSERGALLILDEVQTGICRTGPFFAHQAAGIVPDVMTLAKGLGGGLPIGAVLAQGPAAELLTPGLHGTTFGGNPVSAAAALAVLRTIDEQGLAAHVESVGKTLIDGIEELGHPLIDHVRGAGLLIGIQLTQDVSAKVEEAARAAGYLINPPKPNVIRLAPPLILTEAQAQGFLVDLPGILDAAFQESE

>CORE_REP|Org116_Gene2646#

MANRVFVVGVGMTKFEKPGRRKNADGSDWDYPDMARESGTAALADAGIAYTDVQQAYVGYVYGESTSGQRAVYELGLTGIPIVNVNNNCSTGSTALYLAAQAIRGGLADCTLALGFEKMQPGSLGATFTDREQPMMRHMDLLAQISEVKFPPAPWMFGAAGREHMQKYGTTAEQSARIGHKNHAHSVNNPYAQFQDAYTLDEILAAPMIYDPLTKLQCSPTSDGSGAAVLASEAFVARHDLAGQAVEIVGQAMTTDVESSFAAGTAAALIGYDMNVAAAQQVYEQSGLGPRDFQVIELHDCFSANELLLYEALGLCGAGEAGALVDAGDTTYGGRWVVNPSGGLISKGHPLGATGLAQCAELSKGHPLGATGLAQCAELTWQLRGTADKRQVDGVTAALQHNIGLGGAAVVTAYQRAER

>CORE_REP|Org105_Gene3182#

MRVARARPSVYVESVSFAHAAAMTGPADRLRVAMLTREYPPEVYGGAGVHVTELVSELRALAEVTVHCMGAPRDDAVVHQPDTHLYAANPAIQMMSAQLRMADATGEVDVVHSHTWYTGLAGHLSATLYGIPHVLTAHSLEPRRPWKAEQLGGGYRLSSWSERNAVEHADAIIAVSAGMRRDVLDAYPAVDPARVHVVHNGIDASVWHPGPPEAGGEPFLWQLGVRTDRPIAAFVGRITRQKGVAHLLAAARDFDSEIQVVLCAGAADTPELAAEVASAVEELSRRRGNVFWVQDMLPTEQIRQVLAAATVFVCPSVYEPLGIVNLEAMACGTAVVASDVGGIPEVVADRNTGRLVHYDPAAPSEYERGLAEAVNELAADQVLASEYGAAGRARAVAEFDWSRIAAQTLEVYDRVRKP

>CORE_REP|Org118_Gene6684#

MARVLRSFQVTNHRSLAEPQELRLVSSRAGALPAPVTAVHGAAASGKSGLVDALAHMRDAVLHSVTGWDPYAGPVRTPHLGFADRPSEFVATFVAEGTPYTYGFRLDNADVTAEWLHTHPHNRKRVIFEREGEQIRIGPMFEPVRFGITALVPLVRPNALLLSLTGQMYAEALVPAYRWFSAQLDVQRGPADPHSIAERLGGHISRSADNAARLLMLLRSAELGIEDLLLAENDPMYADYLRDIDGEIAGMAKELDACAASPAHALRVRETLGVEPVALERELSNLRAARDTLYARMVARRGVGLGVVHEGIDAAFDIADESTATLALLRLLPTVLDALDTGRVLAVDDVDTHLTGDTADRLIQLFQNPETNSRGAQLIFTTRNRTLIDRGNGRSQSRRTRVWQVRRTAAGTSELSAG

>CORE_REP|Org5_Gene4499#

MDNRNGCEHPTSNTHRQAPDRARDGDGVTAPQYDSCSVTSMSSDDGARRPDHAARRAGLRDLLVENGVDALLVTDLVNIRYLTGFTGSNAALLVHSWDGSQVEDEGPRTVICTDGRYITQVGEQVPDVRAEIARASARRLVELAGEWQIGRVGFESHVVTVDQHRAFVEQGSGLQFVPAPGLVEQLRTVKDAYEVDRLRTACAAADAALAALLERGGIRPGRSEREVARELEWLMFEHGADGIAFETIVAAGANSAVPHHRPTSAVLAAGDFVKLDFGAVVGGYHSDMTRTLVLGEPSDWQREIYQLVYEAQRAGREALAPGVRCADVDAASRAVIEAAGYGDLFVHGLGHGVGLQIHEAPPVAKTATGTLLDGVAVTVEPGVYFPGRGGVRIEDTLVVRAGEPELLTHTGKDLTVVD

>CORE_REP|Org2_Gene6466#

MNPRPASISARPVDEYRDSIEQLLRPLAARAVEDVAVPHALGRQLADDVRAPVDLPVFRNSAMDGYAVRAASVAVAPVTLPLAGVVAAGNAGQTPLPPGAAMKVMTGAPIPPGADCVVPVEDARADEGTVTVERGRSAGEFVREPGTDVHAGDLLARAGTTLAPRHIAALAAVGLPAVAVVRPVLAAIITTGDELVPAGTELRPGQIYNSNGIALAAALTANGVTVVSVEHSTDDPAQFRKLLAAATGSADVVFTSGGVSKGDFEVVKDVLEPLGGQFGPVAVQPGGPQGRTVVDGVPVLSFPGNPVSTMVSFEVFARPILRRLAGLAPVPSYDLPLRNAVARSPQGKRQFLRGKLIHPESGDLPASQQLPEAVEVVSGPGSHLIASMAWADVLIDVPAAATTLPAGTVVRVWTL

>CORE_REP|Org37_Gene1103#

MSAQHVRRADLCVVGLGPTGRAVAHRAMRAGLDVVAVDPRPERLWPPTFSCWVDELPAWLPHSAIAQRIEAPTVWTQTPHRIERPYCVLSKPGLRDALPLDDATVLAGRATSVDAHRVDLQDGSTITAATVVDTRGLASPGARRTASAHGIFVDAERAAPMVGDGEGLLLDWRDENGAGPDEPPSFLYAVPLGDGTVIFEETSLGLRGGMPQHELRRRTLHRLAAHGIRLRGDEPSEAAHYPLDQPPPPRRGSVRGSDPVPFGSRGGMMHPCTGYSVADSFALVDTLVDAVRTGSDPVAALWPWQARLVYWMRMRGLYGLGRLTTAQSISMFEAFFTASPRQQRALLSAHDDYAALGAVLFMTVSRTWPFRWRYDLVGWANRNRWVGYDYPAATPDQTLDAALDAALPEDGSAGG

>CORE_REP|Org198_Gene6103#

MSDPSRSEIVSPVTDAPNATTAPRADDAASLSAITHDEIFAGHLGGKLSVELSSPLETQRDLSIAYTPGVAQVSRAIAQDEELSKRYTWTERLVVVVSDGTAVLGLGDIGPRASLPVMEGKAALFKKFAGLNSIPIVVDTKDVDEIVDLLVKLRPSFGAVNLEDISAPRCFEIEKRVIEALDCPVMHDDQHGTAIVVLAALNGAAKVQGRGIEGLKVVVSGAGAAGVACTNILLAAGVRDVTVLDSKGIVSRERTDLNEVKAELATRTNPRGLNGGAAEALNGADVFLGLSAGLIAEELIASMAPESIVFAMSNPDPEIHPEVARKYAAIVATGRSDFPNQINNVLAFPGVFKGALDAGARRITEGMKIAAADAILSVVADELGPDKIVPSPLDPRVAPAVAEAVAAAARAEGVA

>CORE_REP|Org102_Gene1805#

MLGSTRLPRALPIRWAPVTVASPFDLIVVGSGFFGLTIAERTANLLGKRVLVVERRYHLGGNAYSEADPETGIEIHKYGAHLFHTSNKRVWDYVNQFTEFTGYQHRVFAMHKGQAYQFPMGLGLLSQFFGRYFTPDEARKLIAEQSAEIDTKDAANLEEKAISLIGRPLYEAFIRDYTAKQWQTDPKELPPGNITRLPVRYTFDNRYFNDTYEGLPKHGYTAWLAKMAESDLIEVRLDTDWFEVRDEIRAQNPDAPVVYTGPLDRYFDYAEGELGWRTIDFETEHLETGDFQGTSVMNYNDADVPYTRIIEPRHFHPERDYPTDKTVIMREYSRFAQTGDEPYYPINTPDDRAKLLAYRERAKTETAAAKVLFGGRLGTYQYLDMHMAIGSALNMFDNVLRPHLESGAPLVDTAE

>CORE_REP|Org186_Gene601#

MTNGNSTPSLRKDGGRDEDRAQSAVDDFAYGSGAPRHGFFNRLYTGTGAIDVIGKRRMWYAITALIVLISLASMLVRGFNFGIDFEGGSRIQFPAGDATTSEVETVYHNTLGTDPVSVQTVGSGSTATMLIRSEALSTEQADQLASALFTEFQPLGNDGQPSRAAISTSDVSETWGDQITRKALIALLVFLVIVSVYIAVRFERDMAIAAMAALVFDVGVTAGIYSLVGLEVTPATVIGILTILGFSLYDSVVVFDKVEENTRGILHLNRRTYAEQANLAVNQTLMRSINTALIGILPIVGLIVIAVWMLGVGTLKDLALVQLVGLLVGTYSSIFFATPLLVSLKERWGPVAAHTRKVLAKRSNVASARAAAEADRRVSVATGRPAAPGGPARPRPNAAPRPGARPSGKRHRRN

>CORE_REP|Org141_Gene4249#

MTSDDAHTPPPVPEPSRDGDPSRPAAGRPGRGSAGRRSAGAAFGRSKPRSGRRSTSAAVSAEGAGNSTSSGSKLRLSAPKLRWGLLAVAVGAVSAAMVTLFVTGFENDSGLEAHNPAVAVPAGAEKTFGTATRGDCLGWTAPDRSDLVEVDCADKHMFEVTADIDLSRYPGKEFGPGSRFPDSLRFTELKEEHCVTAAQQYLGGKFDPRGRFVVGLMYPSPEGWAKTGDRKLRCGLQAAGLTGAASAPLPTTGSVLDNDQSKVFAPGMCLGINQNLPTDPVDCTQPHAVEIVATVDLGARFQGPPPPKEEQDKFVEGECARLSTEYLGGPDVLRNKTLTLFFDYIDARSWLAGSRKLDCMVGKGADQEGFAPITGSAKGEIQINGQAPVPPPSDGRYTPPPLPGAAPLPAQPR

>CORE_REP|Org101_Gene3371#

MTCGSPDGSAAERRVFSQRRGCERAPGNPTLEATLSAGTGTQTPHRTSAAELIAPIPGTPAFVAAVEGALTRFFADRRGTVEELGPIFVEAADALELFVLRGGKRTRPAFAWTGWLGAGGDAAGPDAAAVLTACAALELVQACALIHDDIIDSSRTRRRFPTVHVDFETRHRDRGWAGDSAHFGASVAILVGDLALAWADDMVHASGLEPTAIARFAPVWAGMRTEVLGGQLLDINGEAGGDESVEAALRINRYKTAAYTVERPLHLGAAIADADADLVAAYRTFGTDIGIAFQLRDDLLGVFGDPAITGKPSGDDLREGKHTVLLAEALRRADESDPAAAKLLRTSVGTDLDADEVAHLRTVLTDLGAADEVERRIAELTDAGLAAIATSTATPAAKEQLRAMALAATKRTA

>CORE_REP|Org105_Gene1973#

MTGAPGRPACAPSPAGAAATHTLNGMRRFSLWLRGKPIVADSMLAGVLLVLDVLGVSTAHNKVAFVALSLVLPLPMILRRVYPRLMAAAMLGLSITTTAVSYLTEEESEHVALLGLGIMLYTLVAYVGRREGLLFLAGLVLDTGLSMLATGFPAGTDLAFVIVLYALCWTLAEFIGARHAYDTEVAARLAVADYDRERRAHDAVSAERTRIARELHDVVAHAVSVIIVQADGAKYALRHDPDAAEQAVTTIAATGREALRELRRTVALLRTEHAPDQLPQHGTAGLAKVVQMMRGTGLAVELEMTGELDDIAPEISLGVHRIVQESLTNTLRHAGAHPKAWVRVQRRDDDVLIEVTDSGGVPVEEPENRITGSGLGLAGMRERVAVLGGSMEAGRAPDGSWRVRATIPLVQPD

>CORE_REP|Org150_Gene6053#

MSTSTGSSPGTDTAAGNGPPGGGSLTEPFTAAIAEAEKLIAAADFINDEKDLAEGYDYLAGSIAACLQLSGTHGTSHPYFVSSTGPHAKMGLDNPDTLYYHANVEPGAEYLLTGVRGSTVDLSFQVLRGDYTATDVPNGDDAFDDRRLQIAADGSYQLRFGPPKADAGPNYFVLGEGASMLAVREVYGDWNAETKGSIRIERLDTVGTAAPALGVDQLRKRYRAAARALVQRIHTWFNFPKWFYLDLPVNTLTEPRLTPGGLSTQYSSVGHFDLDDDQALVITVPKSDAPYQGFQLGSRWYISLDYVNHQTSLNSAQAQVDPDGMIRMVVSARNPGITNWIETTGRRRGILQFRWQRVAREMTPADGPQFTLVPFDEVAAQLPHYELNRIDEQGWRARIADRQRGFADRMLG

>CORE_REP|Org88_Gene1874#

MQSWSDTPIPTVPGAGPPLRLYDTADRQVRPVTAGATASMYVCGITPYDATHLGHAATYLTFDLINRIWRDGGHEVHYVQNVTDVDDPLFERAARDGVDWRELGTSEIELYREDMAALRIVPPRDYIGAIESVDEVVEFVGKLVASGAAYTVDDAEFPDIYFRADATEQFGYESGYDRATMERLFAERGGDPDRPGKRDTIDALLWRAARPGEPSWPSPFGPGRPGWHIECAAIAVNRLGTEFDIQGGGSDLIYPHHEYSAAHAEALVAGRRFARHYVHAGLIGLDGEKMSKSKGNLVLVSTLRRAGVDPAAIRLGLLAGHYRQDRMWTDAVLDEAGARLDRWRCATALPAAGAATDTIARLRQHLADDLDTPKALAAVDNWADEALTYGGTDTDAPAAIATAVDALLGVRL

>CORE_REP|Org150_Gene4138#

MINLELPKKLRASANQAHQVAAEIFRPISRKYDLAEHEYPVELDTMAAMVEGLADSGTQDISGATGGRKAKSGEQAADSHSTELLGNSNGGNMSALLNALETCWGDVGLMLSIPYQGLGNAAIAAVATDEQLERFGKVWAAMAITEPSFGSDSAAVSTTAVLDGDEWVLNGTKIFVTAGSRATHIVVWASVDKSKGRAAIKSFVVPRDAKGLTVARLEHKLGIKASDTAELRLEDCRIPADNILGSPEVNVEKGFAGVMQTFDNTRPLVAAMAIGVGRAALEELRTILEESGVEISYDIPANNQSAAAAEFLRLESDWEAAYLLALRAAWMADNKKPNSLEASMSKAKAGRMGTDVTLKAVELAGAVGYSQRTLLEKWSRDSKILDIFEGTQQIQQLIVARRVLELSSAQLK

>CORE_REP|Org176_Gene2589#

MFIDLTTEQRRLRDELRAYFADLVTPEEEAEMAVNRHGDAYRAVVRRMGRDGWLGVGWPKEYGGQGFGPVEQQIFFNEAVRADVPLPLVTLLTVGPTLQQFGTAEQKQRFLPGILAGDIHFAIGYSEPEAGTDLAALRTSAVPDESGDWIVNGQKIFTTGAHEADYIWLACRTGSTESRHRGITILIVDTADPGYSWTPIITCDGAHHTNATYFDNVRVPANMLVGEENRGWKLITTQLNHERVSLGPSGKIEQLYDRVRDWAQPRGVLAELDVRRALGRIHAMVRLNELLNWQVAAASDGRRSQGPAGSRSATASGPGTPAAGVDGDQSRVIADASATKVYSTESLQEAGRLAEEIVGRYGDPADPATGELLTWLDRRTKQNLVVTFGGGVNEVMRELVASAGLRLPRVPR

>CORE_REP|Org49_Gene3373#

MKSAFPGPVGAQTVYLDHAATTPMFPVAVEAMTAVLGTAGNASSLHGSGRAARRLLEEARESIAANLGARPSEVIFTSGGTESDNLAVKGIYRARRDAEPRRRRILVSAVEHHAVLDAVEWLEQHEGADVTWLEVDSEGVVSPRTLRAALESYADEVALVSVMWANNEVGTVQPIVELSSVAQEFDVPMHSDAVQAAAQLPVDFAASGLSAASFAGHKVGGPHGIGVLLLGRTVPCVPIVHGGGHERDLRSGTSDVPAAVGLAAALRETVRGMASRTVELTRLSDRLIAGVRELVPDAVLNGATGERRLPGNVHFTFPGCEGDSLLMLLDAAGIECSTGSACNAGVAGPSHVLLAMGVEPALARGSLRFSLGHDSTDTDVDAVLAALPQVVERAKAAGLASVGSAPHAKGGY

>CORE_REP|Org140_Gene4764#

MVRGKGTSVPEQAFIYEAVRTPRGKQRGGALHSMKPVDLVSGLIDEVLARHGGLDPADVDDVVLGVVTPIGEQGSVIARTAALNSGLQETVPGTQINRFCASGLEAVNLASAKVASGFDDLVLAGGVESMSRVPMGTDGGALFADPATAFDHHIVPQGVSADLIATIEGFSREDVDAYAAESQNRAEKAWASGYFAKSIVPVKDINGVTILDHDEHRRPGSTVESLGKLKPAFAALADMAGFDDVALQKYPSVEKINHVHTGGNSSGIVDGSSLVLIGSEQAGERNGLTPRARVVTFAQIGSEPTIMLTGPTPATELALKKAGLSVDDIDVFELNEAFASVVLKWMKDLKIPHEKVNVNGGAIAMGHPLGATGAMIYGTCLDELERTGGRYGLITLCVGGGMGIATIIERL

>CORE_REP|Org50_Gene671#

MSGGQGRQKSARRWSMRTIAGPVIVVAVAALALTGCSASDATEAAQAAIDRNPITELIKPKLMSPVKDGEVGVSPGVPMTFKVEDGKFTNVSLVSPQGKTVNGKLAADGRSWETTEVLGYGKTYQLKADAIGLGGANSATLSFTTSSPGNQTKPYLIPGEGEVVGIGQPVAVQFDENIPDRKAAQDAIKITTEPPVEGAFYWVNNREVRWRPEHFWAPGTKVTIDVNVYGRDLGNGLYGQDNIHSFFTIGDAVIFTADDDTKQVTVEQNGQVIRTMPTSMGKDSTPTDNGIYIVADRHEKIIMDSSTYGVAVNSPDGYKTPVDFATRLSYSGIFFHSAPWSVGAQGYSNTSHGCLNLSPANAQWVFQNAKRGDITIVKNTVGGTLSGVDGLGDWNIPWPVWKAGNADDNR

>CORE_REP|Org144_Gene5981#

MSEKEPNDMGRPDEGHDGGKPSTEQVTGAGAHVAKTDGHALTEPTEAELDAMSRDELVKLGTERDGVDVAYRRERFPVPGTRAEKRAERAVAFWFAVSGIAAAALVGVFLFWPWEFKANKEDGHAAYSLFTPLVGITFGVSVLVIGIAVVLIRKLFIPAELSIQDRHDGPSPEVERRTLVAELSDALDSSTLARRKLITRTAGAGVGVLGIGALLVFVGGMVKNPWAKGDKSPLWVSGWTPDYEGETIYIRRDTGRPEDVVLVRPEDLDAGAMETVFPWKEKWRGDEHATLQSLRGIRNAVMLIRLRTEDAQKAIKRKGQESFNYGDYFAYSKICTHLGCPTSLFEQQTNKILCPCHQSQFLATEWGKPVFGPAARALPQLPITVNSEGFLVANGDFIEPLGPAFWERRS

>CORE_REP|Org163_Gene5468#

MLTGHFFGAVAGSMIFGGVLGDNGVGERFGRGGRAMDEERQQWTGVRRRPRRGRAFILVVTFVLATAVFLGYRGDLPAPNRPYPAEPVASVAFTPPAPLDPGLVTAAVRPALVNINASAERSGPGAAGSGIVLTADGEVLTSHHVVKGADTVIVTDVGNGKVYNAVVLGYDSEADIALLDLPAAAELPIATIGTSTGLRLREEVLAIGNAGGTGGTPTAVHGPLTDLDSAIVAVNAADLSRKALSGMLEVAAAVTPGQSGGALVDRNASVVGVIAAASGDGARTADQPANGYAVPIDAAMRVVQQIRSGTPTDTVHVGPTATLGVLISNALPVGTGARVDVALHGMPAYAAGLAAGDVITSLDDHVVTSAQSLRAALNTRKPNDTVRLGVTGAAGERTVRVVLVAGPPN

>CORE_REP|Org129_Gene4770#

MAETAQRSGKVREIDVGSTPTRYARGWHCLGLAETFRDGKPHAVQAFGTKLVVWADSNDELKVLDAYCRHLGGDLSMGEIKGDSIACPFHDWRWGGNGRCTSIPYARRVPPLARTRAWTTLERNGQLYVWHDHEGNPPPDDVTIPYIEGPYTDADGNPVEERNSDWTPFTWNTMLIEGANCREIVDNVVDMAHFFYIHFAFPTYFKNVFEGHVATQFLETKGRPDIGMAAKYGGETLLKSEASYFGPSYMINPLTNIYGGYQIKVQLINCHYPVTQDSFVLQYGLSVEKPKGIDDETAEKLAKSMTDFFGDGFLQDVEIWKHKSKIDNPLLCEEDGPVYQLRRWYDQFYVDVADIDPKMVQRFEFEVDTTKANEHWEAEVAENLRRKKEEEEEEAAGKTDPAVKQEAGA

>CORE_REP|Org120_Gene4992#

MTTFAAASNANGRLVRNQGVTGPLGFRAAGIAAGIKASGKPDLALVFNEGPEYAAAGVFTSNKIKAAPVLWSQQVLTGKRLRAVILNSGGANACTGPGGFQDTHQTAEELAAALSNWGTETGAGEIAVCSTGLIGDRLPMDKVIPAITEIVHEMGGGLSGGLDAAHAIMTTDTVPKEAAFHHRDKWNVGGMAKGAGMLAPSLATMLVVLTTDAAVSADQLDQALRNATARTFDRLDVDGSCSTNDTVLLLANGASEVTPSQADLDAAVLAVCDDLAAQLMADAEGVTKRVLVTVAGAVNEDEAVAAARTVARDSLVKTALFGSDPNWGRVLAAVGMAPVTLDPNRISVSFNGNPVCIDGAGAPGARDVDLSGMDIEVRIELNVGDAQATIRTTDLSHGYVEENSAYSS

>CORE_REP|Org134_Gene3544#

MELKVQVYRHASSIPGGCPPPTTPVVAGSGIVNAQVETVVDLDAIAHNVRILREHAGDAAVMTVVKADGYNHGAVEVGRAALAAGAAELGVTTISEAVHLREAGITAPILCWLNNSGADYGAGIAADIEIGISSMSQLRAVEAAARRLGRTATLTLKVDTGLNRNGVSVTEYRDVLTALRPLVDEQVLRFRAIFSHLAHADQPHHPTIDVQRDRFVDAIATAKEYGLVPEVTHLANSAAALTRPDLAFDMVRPGIAMYGLSPVPELGDFGLRPAMTFQAEISLIKHVAAGEGVSYGHEWIAPHDTTVALIPAGYADGVSRRLGGRCEVWVRGARRPSIGRVCMDQMVIDLGDNLDGVAEGDTAILFGTGESGEPHAQDWADLLDTIHYEVVCSPRGRVVRRFRGGQQ

>CORE_REP|Org104_Gene2232#

MSPREGTTAMSAPIVIVGAGLAGLRTAEELRRAGYEGDLVLLGDEARLPYDRPPLSKQFVRGETDDTTLRPAEFFTDKRIELRLGTTATGVDTATRRVLLADGSALAYDHLVIATGLRPRTLPGLPTPAGVHVLRDHADATALRDESASATAALVIGAGFIGCEVAASFRARGLDVVLVEPQPTPLASVLGEQVGGLVARMHRAEGVDLRCGTGVRTLLSDDRGRVRGALLSDGAEVRADLVVLGVGSRPAVEWLADSGIALAEQAAGGGVLADEVGRTSVERVWAVGDVAAWRHETGAQQRVEHWTNAGEQAKLVACALLGAEPPTAARVPYFWSDQYDVKIQALGTPSADDDVSVAADDGRKFLAYYSRAGALTAVVGAGMTAQVMKARAKVAAGAPVADLLATT

>CORE_REP|Org25_Gene399#

MTEAVIVATARSPIGRAGKGSLTGMRPDDLAAQTIKAALDQIPECDAHAIEDLYLGAWEHTGEQSENIARRVAVQLGLDGVPGATVNRACASSVQTTRMAANAIKAGDGEIFVSAGVECVSRYPQHNGVGAGDERFHNPTFDRARARTAQFAETGAPWTDPRAESLLPDVYITMGQTAENVASYRGVTRAEQDEFAVRSQQLAEKAIADGFFAREITPVTLPDGTVVRVDDGPRAGTTVEKLAGLSPVFRDSGTVTAGNACPLNDGAAALVLMSDRKAAELGLTPLARVVATAASGLSPEIMGLGPVEASQRALAKAGLSIGDIDLVEINEAFAAQVIPSYRELGIELERLNVNGGGIALGHPFGATGARITTTLLHTLRERDKQFGLETMCVGGGQGMAIIFERLS

>CORE_REP|Org5_Gene7379#

MLAGKMYGPCRQSAGVLTEFPPKGVSPVPRATAPSVTPGFVNDHGGSTALELDQLVATVRVGADPRIADQLSAAVACYRRPLRIQVAGRAGAGRTTLLRALALMSAEETAPVDEPGAPDPVLDADLVIYALAGSLQPADRRILESLRAESTLVVLNKADAVGSRWGDAVNAAEQATHGLGVPVAPVVAELAVRTRSGTPAEEDLRTLRRHAAGSGTALTLSPELFVDESAGLDVAERRAVLERWGLYGVSCALVALRHEPDLGPQQLLQVLHAASGIDPVHVGLHDRYEQIGALRGSELLDELARIAARAVPRDDGGRARDLIEDYLVGEEALWIGVRAGLAVPEVRHLAAGYPAAMPRDADDALLRAQRWRAVAASDMPAVARRAAIRLHNGYMRMWERMSSAGL

>CORE_REP|Org56_Gene5939#

MDLVGPRAEFGAGVNATVRLEVSDDAPFGPDNLPYGVFAPPGGDFRVGARIGSDVVDLSVLLDDSTFARPDLNAFLAQGPRRWHTVRERLREAVRGPLPAAVIHPLASVRSKLPIAIGDYVDFYASIDHATRLGRFLRPGGEPLLPNWRHLPVGYHGRAGSVVVSGTPVIRPCGQRRTSSGATEFAPSARLDIEAELGFVVGAGSELGTSIAVDAFPDHVFGVALVNDWSARDIQAWEGQPLGPFLGKSFATSLAAWITPLAALESARIALPEQTPEPLPYLRGQQDWGFDIDVRVHWNGVPVSAPPYRAMYWSGAQMLAHLTANGAGTRPGDLFASGTISGPRRDQSGSFIELSGNGAEPVDVGGSERTFLADGDTVLLTATAPGPGGSPMALGEVSGRILPSRR

>CORE_REP|Org1_Gene3792#

MSLPMTAEFTGWHVLAEFGGVDAALCDDLERLESALRESLIAAGVTICDVVHKKFEPQGVTVLALLSESHASIHTYPESGDIFVDVFTCGSIGAGATKAVELLRDALAPANVRMQVIQRGHGAQRIEEPVGAGLTRIWDLQDVIVDTHTPFQHMVIARTEQGISLFSDDDRQSTEFSQLTYHEAMMVPAFVLAEKLDTVLIIGSGEGVASQMSVAAGATHVDHVDIDQLEVELCAQHLPYGYTSEELAAAVRGEGPITVHYADGWDFLEQAQQAGTRYDVIVIDLPDERVEDAQHNRLYEAEFLSRCRALLAPGGVLSAQAGCATMWRNETLKRSWQRFHEQFGTVVHYGSDEHEWSFLFGLVDEIADPVPGMVDRLATLPYRPETIDGRALVRGAIEPHALRASR

>CORE_REP|Org101_Gene7494#

MTTVPTHPVGSASVRPRSVIVSGARTPVGRLLGGLKDFSGSDLGGFAIKAALEKGGVAPEQVDYVIMGQVLTAGAGQIPARQAAVAAGIPMDVPALTLNKVCLSGINAIALADQLIRAGEYEIVVAGGQESMSQAPHLLEKSREGFKYGDVTLRDHMAYDGLYDIFTDQPMGALTEQRNDTEPVSREEQDAFAAASHQRAAEAWKNGLFDDEVVPVAVPQRKGDPVLVAADEGIRADTTAESLAKLRPAFRKDGTVTAGSASQISDGAAAVVVMSKAKAEELGLSWLAEIGAAGVVAGPDSTLQDQPANAIAKACAREGISPADLDLVEINEAFAAVGVASTRKLGIDPAKVNVNGGAIAIGHPLGMSGARILLHLVLELKRRGGGVGAAGLCGGGGQGDALIVRV

>CORE_REP|Org170_Gene222#

MSKIKVEGTVVELDGDEMTRIIWQFIKDKLIHPYLDVNLEYYDLGIEYRDKTDDQVTVDAANAIKKHGVGVKCATITPDEARVEEFGLKKMWRSPNGTIRNILGGTIFRAPIIISNVPRLVPGWTKPIIIGRHAFGDQYRATDFKVFQGGTVTLTFTPDDGSEPIVHEVVKMPEDGGVVMGMYNFKKSIEDFARASFNYGLQQNYPVYMSTKNTILKAYDGMFKDTFQEIFDAEFKSQFDAAGLTYEHRLIDDMVASSMKWEGGYVWACKNYDGDVQSDTVAQGFGSLGLMTSVLLTPDGQTCEAEAAHGTVTRHYRQHQQGKPTSTNPIASIFAWTRGLEHRGKLDNTPEVIGFAQTLEDVVIKTVEGGQMTKDLALLVGGDQGYLTTEEFLAALDANLARALR

>CORE_REP|Org101_Gene1143#

MSTPADDLVLVINSGSSSIKYQLLDPESSAVTASGMVERIGEENGGIEHHADGASTEHRGPIADHTAGLRLVFEMFADTGHDLAAAGVRAVGHRVVHGGEVFYRPTLIDDKVVAAISKLSSLAPLHNPANVAGIESARTLLPGVPQVAVFDTAFFHGLPDAAKTYAIDAKVAAAHGIRKYGFHGTSHEYVSGQVAELLGRDPAELNQIVFHLGNGASASAIRGGRPVDTTMGLTPLEGLVMGTRSGDLDPGIVAHLVRSADMDIDQIDTLLNRDSGIKGLSGVNDFRELQRLIDGGDSAARLAYDVYIHRLRRYLGAYLVDLGGVDAITFTAGVGENSPQVRADALAGLSRFGIEVDAAANTAKDRTARRISPPDAEVAVLVVPTNEELAIARAAHDVAEAEAPR

>CORE_REP|Org19_Gene688#

MLSELKIPRPRPWGRVNGYARDVSERPSGDGENASRAARPGDARDRTGGSGSRRNGTAGRRGKRGTGSGADNRRNGEADRRNAQADRRNAEAEAGDTTRADTVLPVDRPESGVDPGLDPDVEIVIPLTSADEAESRLRSFGRRRTDQLSSLMAAANQRADVIGVIRKARESLPGDPAFGDPLSLSGPGGARAVARAADKIVGDNPSAAKELGLGALQVWQAMLERVGRGKGNAEITVVFTDLVAFSRWSLSAGDEATLELLRRVARAIEPPIVDRGGQVVKRMGDGVMAVFASPDSAVRAVLTAKKNLDRVEVAGYRPRMRAGLHTGTPREIGGDWLGVDVTIAARVMEAGGNGNTMISETTLEALEPSTLEELEVAAKPYRRSMFAAPLNGVPEGMRIFRLAGD

>CORE_REP|Org5_Gene5772#

MSGFEQPFDQALRAGERPLCDLTRGKLRAQDLNWCRINQVSAWSMLPECGRADPMASEEVGPMSSTERRRLEVLRAIVADYIANKEPIGSKTLVDKHNLGVSSATVRNDMAVLEAEGYITQPHTSSGRIPTDKGYRQFVDNIAEVKPLSSAEKRAIMGFLESGVDLDDVLRRGVRLLAQLTRQVAMVQYPTVSASTVRHLEVVALNPARLLLVVITDTGRVDQRLVELGAVIDDEDLAALRGMLGKAMDGKRLSAASSAVAELPEQAPGRLRDVLIRVSTVLVETLVEHPEERLVLGGTANLTRNAGDFGFPGSLRAVLEALEEQVVVLKLLAASQEPGTVTVQIGEETMVEQMRGTAVVSTGYGMPGTVLGGMGVVGPTRMDYPGTIASVAAVARYIGEVLAER

>CORE_REP|Org64_Gene5402#

MKLPALLSKNTETLPGRTGIARVDRNTRRLLRRVGPGDIAVVDEMDLDRITADRLVEAGVVAVVNTSPSISGRYPNLGPEVLVANDIMLVDTVSSDAFTKIKDGSKIRIHDGVVYADKLTKKEPEALVEGIELTEAAIAERMIEARNGLADHLEAFAGNTIEFVRTESALLIDGIGVPELELDMKQRHVVVVADGPDHAEDLKRLKPFIKEYAPIMVGVGRGADTLRKQGYRPDLIVGDPEEITSATMKCGAEVILPADTDGHAKGLERIQDLGIGATTFPSSGAPADLALLLAEHHGAALIVTVGAAASLDDFFDRGRRDSNPATFLTRLKVGTKLMDAKAVATLYRNRMSGVALAMVVLAALIAVIVVLLASNTGTEVLDWAVDTWNRFARWCQDLVGAGQR

>CORE_REP|Org45_Gene5718#

MRRSQQGGGDAAGGSAGFDSGDNERVSDVVKVLLLGSTGSIGTQALEVIAANPDRFEVVGLAARGGNTELLASQIAATGTGNVAVADPAAAAKLGVPLAGPHAAAQLVRDTDADVVLNALVGSLGLEPTLATLESGRRLALANKESLVAGGSLVTRAAAPGQIVPVDSEHSALAQCLRGGRAEEVERLVLTASGGPFRGWTTEMLESVDPAAAKAHPTWSMGLMNTLNSASLVNKGLELIETHLLFGIDYDRIDVTVHPQSIVHSMVTFTDGSTLAQASPPDMRLPIALALGWPDRVPGAATACDFSQAATWTFEPVDNTVFPAVELARRAGKAGGCVTAVYNAANEIAVQAFLDGVIRFPDIVRTVARVVESADRWSAEPSTLDEVLAADTWARDCARAFVRN

>CORE_REP|Org105_Gene3394#

MGKRSADRADIVVGMLNEQRIREDTPGVGHGLVFLDSAGSSLPPRVVTETVIAHLRREAAVGGYRAANERLGDLAAVKESIAALINASPAGIALSDSATRSWADFFYSVPLGPGDRILISGSDYASNAIAALQRARASGATVEHIPSDPTGQLDLDAFAGLVDERVKLVSLLHAPTNGGLVNPAAEATRIAHEAGALVLLDACQSAGQIPLDVAELGVDALSATGRKWLRGPRGTGFLYVRPELAARMEPARLDLHSAEWTAPDDYRLAPDAGRFEFWEHDVAARLGLGAAVDYLLELGPDEVYAAIAARAEYLRKGLAEITGVTVRDLGIRHSGIVSFTVDSVAPVQVRDRLAAEDITVTVSHRSSTLLDMAGRALDAVVRASPHCFVDFAELDRFLTAVAEL

>CORE_REP|Org87_Gene3819#

MDAHPLVTEYLRLGLAFDRLEEGFVDAYTGDPALRREVANAPAPQPRELARRAAELRAALPDSGLPAERVEFLDAHLRALECSGRKFAGDDIGFVDEVRAYFDVEIAPGNVEDYLDAHRRMDEVLAGDGMLAERIAAHRRADEIPPERLSACVEAFSSALREKVRERYPLPDHEHVTYEVVGDKPWSGFNYYLGNFHSRVAINSDLKQHMANLPHLIAHESYPGHHTEHCRKEAGLVAAGQAEQTLFLVNTPQCLMAEGLADLALRSIVGPGWGRWAQEIYADLGLRFDGERAERLAQASAQLLSVRQDAALLLHDRRRDEREVAEFLQRWSLVTPDRARQQLRFLSSPLWRAYISTYVEGYRLLGDWLDRAGDPAERGERFRRLLDEPLTPAAVRRELSASV

>CORE_REP|Org138_Gene5469#

MLATTGARLRDAVPLAELTTLRVGGPAPVADCAGTEALVATVRALDAADIPVLLVAGGSNLLVADEGFPGVVVRIANAGVRILTGADDAVAAVAGNDARAAATRTDAETGAVVATPAAGGIGVTDMPGGGDVVRVIAEAGANWDAVVAETVVAGYGGLECLSGIPGSAGATPVQNVGAYGVEVASLLTRVQLLDRASGEITWVQPSELGFGYRTSVLKHSDHAVVLAVEFALRADGSSAPLRYRELAAALGAEEGESRPAAEVRAAVLRLRAGKGMVLDPADHDTWSAGSFFTNPVVPAARVDEVRAAIAARVGPDVAVPTYPAPDGVKFSAGWLIERAGFAKGFPDESAPARLSTKHTLALTNRGAAKASDVVALARTVRDGVAERFGIRLEPEPVTVGLTL

>CORE_REP|Org45_Gene3101#

MVSSSDLLSQGVFSTAAVVPLRELLLVLLVATVVTYLSTGGVRVGAIAFGAVAVPRERDVHVKPIPRMGGVGIYLGVLAAVLFAHQLPALRRGFDYPADIPAVVVAGTLIVLVGIIDDRWGLDWLTKLVGQVTAAGVMAVMGLSWVAIYNPFTNTTVVLDQLQGGLVTVGITVTMINAMNFVDGLDGLAAGLGLIAAAAVFVFTVGLLYEQGGSTDTYPPALLAAALAGGCLGFLAHNFQPARIFMGDSGSMLIGLMLAAVSTGASGRIPLQGYGTRDIVGLLSPLLLVGAVMFIPVLDLVLAIVRRVRAGVSFSTPDKMHLHHRLLQIGHSHRRVVLLIYLWVSVLAFGAVGSSLMDRRLVVLLFAGGLVFALVITAVPSMGELTLKAGRKPPRGPDAARG

>CORE_REP|Org33_Gene789#

MTAASSEPVIFDPYDYAFHEDPYPVYARLRAEAPLYHNPELDFWALSRHADVTAAFRDATRLSSANGVSLDPAAWGPHAHRTMSFLAMDDPRHMRMRKLVYKGFTPRRVAEMETRIREITLSYLEPALERGRLDWIDEFAGKLPMDVISELMGVPEPDRAEIRRLADLVVHREDGVLDVPDAAIDASLRLVGYYADMVKDRRADPTDDLTSALLDAEIDGDSLSDDEIIGFMFLMVVAGNETTTKLLGNAVYWAARNPAEYAKVAADPDRVPDWVEETLRYDTSSQMVARSATTDIDYHGGTIPAGAKVLLLIGSANRDSAAFDDADSYRIDRHDTSALASFGAGVHFCLGAHLARLEANVALREFATRVTEYTVVTEGIERVHSTNVRGFAHLPITVEVH

>CORE_REP|Org14_Gene2625#

MTYDVARVRGLIPSLGDGWIHLDPQAGMLVPDSVSRAVSTGFRTSAFSHTNRHAAARRSGAILDAAREAVADLVGGDPAGVVLGPDRAVLLAWLAESLSSRLGLGTGIVLSRLDDEANVAPWLRIANRYGAHVRWAEVEIETCEMPAWQFEELIGPTARLVAVTAASPIVGSAPAVRVAADRVHEVGGLLVADCFGAAPYALIDIDELNADVVALSAPAWGGPQIGALVFRDPAFLDRIPSMSLNPYAKGAERLEVGGHQYALLAGLTTSIDYLAGLDEQATGSRRERLEISITSLQDYHDQLFEHLMEVLDAVPDLTVIGRASTRIPTVSFTIAGMQAEKISAELADHRIGTVSGAHGGSRLLDALGVNDEGGAVTLGLAPYTTKFEIEQLGRALNSLEK

>CORE_REP|Org113_Gene287#

MSRPSPLVPPATPLLDIEAIIADRTARVVVCCGSGGVGKTTTAAAIALRAAEQGRKVVVLTIDPARRLAQSLGVSDLGNAPQRVQLPETAAGELYAMMLNMRRTFDEMVLEHTTPEKAEQIFANPFYQTVASSFGGTQEYMAMEKLGQLVAKRDWDLVVVDTPPSRNALDFLDAPKRLGNFLNGRMIRVIMAPGRGVGRFVTGAMSLAVRGVSTIVGGQMLKDASLFLQSLESMFGGFQDRAERTYAMLSRPGTHFVVVAAAEPDALREASFFVDRLSTDSMPLAGLVLNRTHPVLCTLPAAQAITGADRLSPGAGSAADGTPAEAEAADATTDPATLTADILRIHAHRAVTAKRERHLLHRFTGAHPRVPIVTVTALPFEVSDLEALRAVGDQLAGNPTA

>CORE_REP|Org9_Gene877#

MPLLPVISATRRRIFRRNVANRAREVHDGGMRIGVPGEVKAQEFRVALTPAGAAELVRHGHEVLVERGAGAGSGFGDEAYRAAGARVVAEADEVWGEAELVLKVKEPIEPEYHRMRREQVLFAYLHLAASKECTEAILRSGITAIAYETVRAADGSLPLLAPMSEVAGKLAAQVGAYHLLAPLGGAGMLLGGVPGVRPAEVVVLGGGVAGGNAAAVAAGMGARVSVLDTDLVRLRAPDARFQGRVSTIASNAAEVERAVLRADLVVGSVLVPGARAPKLVTQAMVAAMRPGSVLVDIAIDQGGCFEGSRPTTHANPTFYVANSLYYCVANMPGAVPHTATVALTNATLPYVLAIADLGWREACSARPDLAAGVTAEDGRLLSPQVAAAHGLGEARPMGLAG

>CORE_REP|Org5_Gene7177#

MRSPRGRRTRSGLNLTGSERFTTAVAYDAVRDTGKQRMRDGWTRLRRSALPIVQCAVGAALSWFIAHRVVGHPQPFFAPMAAVISIGVSFGARLRRSVELVCGVAVGIGIGDFFIGRVGTGPWQIALVVAVAMGTAVFLDKGAVIPMQAASSAVLVATLMPPHTGGGLNRMVDALVGGLVGIVVVAVIPLHPVRRARQQAADILAVVGAALSGCADGLLEQDAEKVRAALQSVRGTQPQIDGLRNAVEGGREVSRISPLYWNSRPRLEVLRDAIEPLDNAVRNTRVLLRRALTLVRDDEILDPRMVAEVEELGQAVDVVRRYVLAEPGREPDAADATRVLRRVAKGATKDLVEGAGLSSHVVFAQLRSIVVDLMQVCGVKRLSAIALLPPTVRNPYVAPRD

>CORE_REP|Org194_Gene5107#

MQCSGTRRQRWLVSNSQRHRFLASGLTLALGVLVGGGYLLFDVMRVRPPGSTYTVTIQLDRSGGLQAGNNVTWRGYRIGEITAVELTDAGAAVAVRAEVDNRYRIPKDTAVQVHALSAAGEQYIDFVPNTDRGPYLDDGAVVPFDAARTSTPVPLSEVLIDTNELIAQIDPDKFAVILDELDIALSGGPDQFRAFVNGISLAVAGLDNLLPQTTNLITSLRTIAATTSQAQPDLGTLTRNSRILIDQVTAADAEIRALLDDTPGMIEVAARTLDRNADPITSLATNLSAVVRAAQLRIPALRALFPSLLIGTSAMGVPAHDGEFYTIVDIWPRPFCQYPTKQTPQYVVQDGTFRRWNYCVNPPAEQQIRGSGNAPRPNVPDNGANQPTGVDPNERTLPPVR

>CORE_REP|Org102_Gene2411#

MNAERNTGTTDIEVVRDRLTGAARTVVGAVAGAVGIGWLVRAAWGLPSAMGAGISAIQPTATASVRYRNRQFHNTEPSSQIASGSGFSLLNSVLTKRNLGRAPGPIPLVTPELPEQAADLAVTWYGHATALVEIDGYRVLTDPVWSERVSPSPLIGPARQHPVPQRLDELPPLDAVIISHDHYDHLDRDTVQGLVASQSAVFIVPLGIGAHLRHWQVPDERIVEMDWGTSVSLSTLQRAHEGTDLTITCTEARHFSGRGLVRNTTLWASWSLAGPRHRVYFGGDTGYTKAFSEAGAKLGPFDLTLLPIGAYDAAWPDVHMNPEEAVRAHADLNLGNAAHGLLVPIHWATFNLAFHGWSEPVQRMVAAADAAGTAVAVPKPGQRIDRNGVPPGQTWWKDVSR

>CORE_REP|Org2_Gene8017#

MTTRKAEDPQHATARSLGERSRANRDAVATAAVWSERADMAESAIISRHLRALWALPGTQLGVVGWPATKRERAFGSWHYWWQAHLIDCAVDAANRTPTPVRRKRIAAIARSHRIRNLTGWTNRYYDDMAWLAIALERAERIAGVTEARSGLLALEKPLYEGWNPEVGGGLPWRIGSDYYNAPANGPAAIALLRLGRQLRAQEMADWLDETLRDPESGLILDGIHLPAGEIERPVFTYCQGVVLGVETELAVHTGEARHIERVHRLLGAVEEHMTTRGVINGGGGGDGGLFNGILARYLALVALMLPGEDEARAADRRAAAAIVRASATAAWANRLQVEGEPLFGHDWSRPATLPGGTAGAGHFTPGGSVTASRVPERDLSVQLSGWMLMEAAYQVSAAGL

>CORE_REP|Org120_Gene4212#

MRGNTDSWRYRGDEVIGRDDVGTIDDLHASATKVVGLDDFGADDYRAGLEVLLASYAKDAELTPFGNKVNRAFLRGALIARLLSESAWQRFPEHAEVAIERPVFVTGLPRSGTTAVHRLLNADPAHQGLEMWLTEMPQPRPPRDTWASNPVYQRIEAAFEKHHVEHPEFMGVHHISAEQVEECWQLLRQSAMSVSYECLGYLPTYSEWLREQDWTPAYRRHRRNLQLIGLPDAGRRWVLKNPSHLFALDAIFEVYPDALIIQMHRDPRTIIASVCSLNEQASAGWSDKFRGPVVGAAQLDLWARGADRFLADRQRHNAAQFCDVYYDDFVADPIGTVAGIYRHFDLPLTAQATAAMTALHAESTSGAARPVHRYTLEDFGLTAEQVDARFADYRAEHFPKA

>CORE_REP|Org94_Gene5557#

MSERVVLAYSGGLDTSVAISWIGKETGAEVVAVAIDLGQGGEDMNVVRQRALDCGAVESIVIDARDEFAEQYCLPTIQANALYMGQYPLVSAISRPLIVKHLVEAAKFHGADTVAHGCTGKGNDQVRFEVGIGALAPDLNVIAPVRDYAWTREKAIAFAEENKLPINVTKKSPFSIDQNVWGRAVETGFLEDLWNAPTKDVYDYTADPTVNFEAPDELIITFDKGVPVAIDGRPVSVLEAIVELNHRAGRQGVGRLDMVEDRLVGIKSREIYEAPGAITLITAHQALEHVTIERELGRYKRQVEQRWGELAYDGLWFSPLKRALDAFVQDTQQHVSGDIRMVLHGGSAVVNGRRSEQSLYDFNLATYDEGDTFDQSLAKGFVQIHGLSSKVAARRDLNQK

>CORE_REP|Org114_Gene7015#

MTTASGGRPTGGLSGRAAIVGIGATDFSKDSGRSELRLAAEAVTAALADAGLTPADVDGLTTFTMDTNTQAAVARATGIPSLKFFSNIPFGGGAAAATVQQAAMAVATGVADVVVAYRAFNERSGNRFGQFATHLATGNPSSSGVDNAFSYTHGLGTPAAQVAMVARRYMHVYGATSADFGRVAVADRKHAAVNPAAHFYGKPITLEDHQSSRWIAEPLHLLDCCQETDGGVALVITSAERARDLPNKPAVILGAAQGSGADQYVMTSYYRDALTGLPEMGLVGDQLWSQSGLTPADMQAAILYDHFTPFVLMQLEELGFCPRGEAKDFIADGAIELGGRLPLNTHGGQLGEAYIHGMNGIAEGVRQIRGTSVNQVDGLENIIVTAGTGVPTSGLILSTN

>CORE_REP|Org39_Gene4732#

MTRPPYGQGPISPGGRPVPPPPGRRPGAPGPRPEPGYPQRPGPGYPQRQGFPPPPGYQPHPGPGGPQASPYGIPLSPPLPPVGYTPHQRPPTQPPAPRGPTPYGPTAHPTPPPRRRRWGTVLLIVIVVVTGVLLRTALSAGVDKLGAGDSVDTGYGPDIGALGVAETAGNPLLTDTDATLVPAECDYAPWSTQVEQARAFFDSAATCLESGWRAVFAEVGLSFEAPKVTVTASTTGITTLCTGNSSNFAAFYCSADKTIYMPISQLQTDVFKNNWVVYLSVFAHEYGHHVQAQSGILGKVHAQRREAGVRSDTGLELSRRTELQANCFDGMYLSSSRDGGALTSAQIGVARNDAYHRGDAPGDMRDHGTTQNGGAWFELGLDENRTFACNTFTAPASAVN

>CORE_REP|Org19_Gene2810#

MSSPQSLPPLVEPAAELTRDEVARYSRHLIIPDVGMDGQKRLKNAKVLVIGAGGLGSPALLYLAAAGVGTLGIVEFDEVDASNLQRQIIHGESDIGRSKADSARDSILEINSGVEVVLHKIRLEPENAVDLFAQYDLIVDGTDNFATRYLVNDAAVLAGKPYVWGSIYRFEGQVSVFWEDAPDGPNGEKRGINYRDLYPEAPPPGMVPSCAEGGVLGVLCASIGSIMVTEAIKLITGIGETLLGRLMVYDALDMNYRTIKLRRDPERQPITELIDYEAFCGVVSEEGQAAAVGSTVTARELKDMLDAGKDVAIIDVREPVEWDIVRIDGATLIPKDRILSGEALAELPQNTPIVLHCKTGIRSAEALAALKRAGFSDATHLQGGIVAWANQVDPSLPVY

>CORE_REP|Org214_Gene3195#

MSSKNGGFSVRGVREWLEAPAASVAERGGKLNVLRGAVARVTTPLLPDDYLHLANPLWSARELRGRIVDVRKETADSATLVIKPGWGFDFKYEPGQYIGIGVLVDGRWHWRSYSLTCPPNWSDPGVGGKRVISIAVKAMPEGFLSSHLVSGVPVGTVVRLAAPQGGFVLPYPPPERVLFLTAGSGITPVMAMLRAMDRRDLVTDVVHLHSARTAQDVMFGAELRDLHDRYATTHPADSVGDRPPASFTSHLHLTGEQGKFALADLDTKFPDWRERQTWACGPAAMLDEIEHHWREAGLADQLHVERFEIERSAVGEGGTVSFGKTGRTVEVDGATSLLEAGESAGVQMPFGCRMGICQTCVVTLSSGYVRDLRNGDEHREGDKVQTCISAAAGDCTLDV

>CORE_REP|Org121_Gene3999#

MNSVSRSDSVGGGGRATGGRWNTPERRELRATVRSFAEREILPYMDEWERDGEIPRELHKKAGALGLLGIQFPESAGGSGGDGIDAMIVCEELHQAGASGGLFASLFTCGIAVPHMIAAGNPEQIERWVRPTLAGEKIGSLAITEPGGGSDVGHLTTTARRDGDHYIVNGAKTYITSACRADYVVTAVRTGGPGSQGISLLVVEKGTPGFTVSRKLDKMGWRASDTAELSYVDVRVPAANLVGAENSGFFQIAGAFVSERVGLAVQAYSSAQRCLDLTLDWVRSRETFGRPLISRQAVQNTVTEMARRIDVARVYTRDVAQRSANGETDLIAEVCFAKNTAVEAGEWVANQAVQLFGGLGYMRESEIERQYRDMRILGIGGGTTEILTGLAAKRLGYQS

>CORE_REP|Org14_Gene5086#

MHRLRHGDTGPAVAEVRSNLASLGFHPHPHGTDRSGGQGEYWKDSDAVFDRELDSAVRAFQQQRGLLVDGVVGPATYRALKEASYRLGARTLIYQLSAPLYGDDVATLQRRLQDLGFYVHRIDGYFGPHTHDALTAFQREIGLSADGICGPDTLRSLELLGARVTGGNPHRIAEEEVVHRAGPQLTGKRIVIDPGMGGEDKGLAVPTEFGDVYESEILWDLASRLEGRMAATGMETFLSRPWGANPTDAERAETSNTFDADLMISLRCAANLSSSAGGVASFHFGNSHGSVSMIGQVLAGFIQREIVARTSLQDCRTHARTWDLLRLTKMPTVQVDIGYLTNEYDASVLTNPRMRDVIAEAILISVKRLYLLGQDDQPTGTYTFAELLAEELAAADRM

>CORE_REP|Org39_Gene327#

MRILHTSDWHIGRTFHGVDLLADQACSLEAMAELVAAEGVEVVVVPGDVYDRSIPSADAIAVCNRGFEAMRAAGATIVATSGNHDSPTRLGALGSFAAAGGLHLRTTIAEVARPVVLADARGDVAFYGIPYLEPDITRAELGVPQARSHAEILDAAMRRIRDDLAGRAGTRSVVLAHAFVVGGEATGSERSIAVGGVETVPLRAFDGIDYVALGHLHSPQTLSDSVRYSGSPLPYSFGERSHRKAVWIVDLDADGLGEVRRVDLPVVRGLSQLAGTLDELLTEPEFAVAENDYVAATLTDHARPVDALRKLRGRFPHAVHVEWVRPEGNPELRYRERVQGRRDIEIAHSFLSDVRGEPSGSELQWVERALAAAVREPERESTAAEGLFAEPGTTELTA

>CORE_REP|Org10_Gene4669#

MAAGRQVRDRAATGVWTAPVSPAESAGRGYRVDMTGPTPLTLASIRRAPKALLHDHLDGGLRPATVLELAAECGYDELPADTEAELAAWFREAADSGSLERYLETFAHTVAVMQTSEGLRRVARECVLDLAADGVVYAEVRYAPEQHLERGLSLDEVVEHTLAGFREGERAAAAAGQPIVVTCLLTAMRHAARSREIAELAVRWRDRGVGGFDIAGAEAGYPPTRHLDAFEYMRANSAHFTIHAGEAFGLPSIHEALAFCGCDRLGHGVRITDDIRVGDSVADAELGLVARYVRDKRIPLELCPSSNVQTGAVPSLDKHPFDLLARLRFRVTVNTDNRLMSDTSMSQEMHKLVQTFGYGWSDLERFTINAMKSAFIPFPERLRIIDDIIKPGYAVLIG

>CORE_REP|Org12_Gene3677#

MSQKRTGCTEGAAETLVADRSAHFRLVRKCAVAYETVGRSSGRCFVTGYRLAIVGVPTAVPVSQEPGLKLGWPDRADKARLHYVSGKGGTGKSTVAGALALALAAGGRRVLLVEVEGRQSIAQLFDLPPLPPTETKIATADGGGEVMALTLDIEHAFLEYLDMFYNLGFAGRAMRRMGAIEFVTTIAPGLRDVILTGKIKECAVRTDKSGRRVYDEVVVDAPPTGRIAGFLDVTKAMAEVAKGGPIAGQAEGVAALLHSEQTIVHLVTLLEALPVQETSDAIAELTESDFRIGTVIVNRATEGFLPAPVRARVATGDVDLDAVRAGLAEAGITVDDNDFQGLIREAVEHSATLQAQDDSSAELAKVDISRLYLPALPDGMDLGGLYELAEHLSAQGVR

>CORE_REP|Org132_Gene576#

MRIAYTEQQEQLRAELREYFAKLITPERREALSSQTGEYGHGNVYREVVQQMGHDGWLTLGWPKEYGGQDRSTMDQLIFTDEAAVAGAPVPFLTINSVAPTIMHYGTEEQKKFFLPKISAGELHFSIGYSEPGAGTDLASLRTTAVRDGDDWVINGQKMWTSLIQYADYIWLAARTDPTAKKHKGISMFIVPTSAEGFSWTPVHTMAGPDTSATYYQDVRVPHSAMVGPENGGWALVTNQLNHERVALTSAAPLALAVAQTTEWARDTKDSTGARVIDNEWVRLNLARVHAKVEFLKLLNWEIASGADAGGDAAPRPWDASTCKVLGTELATEAYRLLMEILGPQAYLRQDSPGAQLRGRLERMHRACLILTFGGGTNEVQRDIIAMTALKQPAAAR

>CORE_REP|Org69_Gene1199#

MNDTPDRTAAEAVDGEFSARRWRLVLGAAAEPELGGLGSADDAAMDGALSTLYDSDPPAGAGPRSAGLQGSAPRVARWLGDIRTYFPSSVVEVMQRDAIERLDLTQLLLEPELLEAVEPDVHLVGTLLGLNRVMPETTRSTARMVVERVVRDIERRMAARTVAAVSGALDRAARVSRPRPRDIDWDRTIRKNLAHYLPEQRTVVPERLVGYGRRAKAVRRDVVLAIDQSGSMASSVVYASVFGAVLASMRSLRTSLVVFDTEVVDLTEHLSDPVDVLFGTQLGGGTDINRALAYCQTLMTRPEETLFVLISDLYEGGIRDDMLRRVNALKESGVQVLVLLALSDDGAPAYDHDNAAALDALGIPAFACTPDRFPDLLALALNRGDIRTWANTTTGRS

>CORE_REP|Org4_Gene2732#

MSAPSTPRRPYTPLRDAYVIDAVRTPVGKRGGALAAVHPADLGAAALRGLLDRNSIDPGNVDDVIVGCVDNVGPQAGNVGRTAWLAAGYPEEVPGVTVDRQCGSSQQAINFGAQAIMSGTAEVIVAGGLQNMSAIPISAAMYAGKEYGFDSPFVGAAGWDHRYGTAEVTQFRAAQLIAEKWGISREDMERWALRSHERARDAIKNGRFDREIVPVGDFWIDQGPRETTLEKMASLPPLAEGSPLTAAVASQISDGASATLLASEWAVEAYGLTPRARIHHVSARGADPIFMLTAPIPATKWALEKTGLTIEDIDVIEINEAFAPVVLAWLKETGADPEKVNVNGGAIALGHPLGATGAKLFATLLNELERRNGRYGLLTICEGGGTANVTIIERLPN

>CORE_REP|Org168_Gene5410#

MSGGVSETTPIPGLHAATADLDPPLAALDLPTLRANAADLERRAHGTPIRVAAKSVRCRAVLETVLGAGLTRSGGFAGIMSYSLREALWLAGLGAGDVLLGYPSVDRAALAALAADESALRAITLMVDDVAQLELARAAMGTDRVRPRVCLDVDASLRLGPLHLGVRRSPVRTPEQAADLARAARAANFDVVGVMTYEAQIAGLPDRNPAVRLVKKASAAEIGRRRAAVLDAVRSVVGPLEIVNSGGTGSIEVSVGDTQVTEVTAGSGLYVPTLFDHYRSFTPRPALFFATSVLRRPAPSIATVFAGGYIASGPAGASRVPKPVWPKGLRLIGTEGAGEVQTPLSGASELRIGDRVWFRHAKAGELCERFDDLHLVEADGTRTTVPTYRGEGKNFG

>CORE_REP|Org142_Gene4996#

MRRDSVRRPGERRGSRPVSEDRVIFSRPYRAAAEVENLRAVLDSDHSHGDGRFTKTATAKIKAITNSPHALLTTSCTHALELGALLLELGQDDEVIVPSFAFTSAATAVALRGATCVFVDIDPATGNIDPMSVADAVTDRTKAVLVMHYGGVAADMAPLLEIAGEHGLALIEDNAHGLGGTWRGRALGTIGTIGTQSFHDTKNVHCGEGGALLLSDEILMGRAEIIREKGTDRARFLRGQVDKYSWQDIGSSYLPSELNAAVLDAQLAEFDRIQTGRHRVWDAYASALPEWARRNDVRLMQVPGDREHTAHLFYLRLPSEDIRDTMIRHLADRGIVAPFHYVPLDSSPAGLKYGRTPVPCTHSAEFSATIVRLPLWPMLGDDQIQRVVDAVTAFAV

>CORE_REP|Org142_Gene4386#

MWDAIQYCLEYRPEEIPVAVDRLLPTDEAKDLIQLTRDVADKVLAPIVDEHERSETYPEGVFATLGEAGLLTLPYPEEWGGGGQPYEVYLQVLEEIAARWTAVAVAVSVHSLSIHPLMAFGTEEQKQRWLPEMLGGTTIGAYSLSEPQAGSDAAALACRATGVDGGYRITGSKAWITHGGIADFYNLFARTGEGSKGISCFLVDKDTEGLSFGKPEQKMGLHAVPTTSARYDDAFVPSERRIGNEGQGLQIAFSALDSGRLGIAAVAVGLAQAALDEAVAYAQERVTFGRKIIDHQGLGFLLADMAAAVDSARATYLDAARRRDAGLPYSRNAAVAKLVATDAAMKVTTDAVQVLGGYGYTRDFRLERYMREAKITQIFEGTNQIQRLVISRHLAG

>CORE_REP|Org51_Gene384#

MALSAGTRRTVACLLVGLGALLIVMALLIPTYTVDKLAKTPLDLEITTIANSQQGQDSLVLDSKSLTAPEGSAKVDSNVPLISQRFLTVEEPSNATEMTVQAGQTLRRTDRQGDTGLLTASIDRVTIDRKTGMPVDTEPNGSIAVTTNAAGESIADPVQHTGLQYRFPIGTEKKSYPYFDLNARATFDANFIEETEINNLKVYHFQQTVPVTSMWDVVQAPTNRLTLPAAKWGLEGGDTPVTMTRYYTNVRDLWIEPQTGTVVKGSEQLHLFYGRSPQQEDVTALKSTLVFDENTIESQIAIAKDNIDTLSLFGRVVPIILGIVGVIALIAGALLGIRGAKNPAPAGGGFGPRGGAGPSRGPAPTGGAAAAPGSGAVRRGEDDAPTEQINIKKNL

>CORE_REP|Org174_Gene5298#

MSRVERKLLVVDARSAALVRANLREVIDSPHGPERLISAFYGHLFAENPGLRDLFPPAMDVQPKRLTTAIQFVLDHLEDWDRAQNFLEQLARDHRKYGVEAAHYDLAGRALLDAFRTYNGPAWTRELEEGWRDVGILISASMAIGANSDTSQPYWEATVVGHRRVLDDLAIVRLQSDTPVPYQAGQYVPVAVPQRPKMWRYFSPAIPSNPYGEIEFHVRKVRGGWVSPAIVNETQVGDRWLIGGPLGGLHVDRNSGKDVLMIGAGTGIAPLRAQLIEMSQRGVNPRVHFFIGGRYPCDLYDVENMWQLSQSNPWLTIVPVCEQKTNPWWYPHPATDAPYGMHRTLIGNLGAVVASFGAWEDRQIQIAGSAPMIADTRRALIAVGTPEHIITSDPI

>CORE_REP|Org27_Gene1522#

MNPAFVIDAVRSPMGRARANGALADVHPVELLAQVVSALIARTRVDPGEVEDLLVGCVTQSAEQSGNIGRMAWLAAGLPEHVPAVTIERKCGSGQQALQFAAQGVMAGSYDIVIAAGVESMSRVPIGSNRQGADIYGPSVTERYAPGLVSQGVAAELVAQRWGIGRARLDEFAARSHELAHAADAAGAFRREIVPVTVPGTGAVVDRDETIRPGTTADKISGLAPAFRTDELAERFPELDWRVTAASSSQLTDGASAVLIASERAAGRMGWQPRARFHGFHACGDDPLLMLTAPIAATRTLLARTGLGLDDIDHVEVNEAFASVPLAWADELHADLDRLNPRGGAIALGHPLGATGCRLLTTMLHAMEDNGGRYGLQTVCEAGGMANALVLRRE

>CORE_REP|Org109_Gene492#

MTTTAGGFPAAVLGTGQTHHVTKRTDVSMAGMCREAIDRALADAGLTIADIDAVVVGKAPDLFEGVMMPELFMADALGATGKPLLRVHTAGSVGGSTGIVATNLVQAGVHKRVLAVAWEKQSESNAMWALSIPVPFTMPVGAGAGGYFAPHVRSYIRRSNAPGHIGAMVAAKDRRNGAKNPLAHLKQPDITLESVLASQMLWDPIRFDETCPSSDGACAIVLGDADAAAEVEATGRKVAWVHATAMRTEPTTYAGRDQVNPQAGRDAAAALWEAAGITNPLEEIDVAEIYVPFSWFEPMWLENLGFTPQGDGWKLTDKGETEIGGTLPVNPSGGVLSSNPIGASGLIRFAEAAKQVMQRAGDYQVEGARKALGHAYGGGSQYFSMWVVGSERR

>CORE_REP|Org192_Gene1814#

MAMQQEWADKDYYGDLGVSSSASAADIKKAYRKLARENHPDSNPGDKKAEEKFKRVAEAYDVVGDEQKRKEYDQLKSMISSGGFGRFGRGGGSGFPGGFRGTETEFDLSDIFGSAAGGQAGDSGLGDIFGGFFGGRGGAGRNARPSRGADVETEITLDFREAAKGTTIPVELTGDAPCTTCHGSGSKDGKTHTCQMCSGSGYIRENSGAFGMARPCTNCGGTGEIIEDPCDTCGGTGTVRRTRSITVRIPAGVIDGQKVRLAGQGEAGPNGTPAGDLFVTVHVRNDEVFTRSGDDLEVTVPVAFSEVALGATITVPTLDNPVKVKVPAGTPNGRTLRVKGRGIPKRSGAGDLMVTVEVKVPKDLDPSATSALRAYAQAEKDSGFDPRAGWAGL

>CORE_REP|Org102_Gene3646#

MGGVISAARPGTRATALGWDTGEVMTAPLPLVFDAPRRGMPPRHLADLDAEERRAVMADLGLPKFRADQIARQYYGRLQADPEQMTDLPADMRAKVGEALFPPLLTPVRHIACDDGSTRKTLWKAGDGTLLESVLMRYPDRATLCISSQAGCGMACPFCATGQGGLNRNLSTAEIVDQVRAAAAALRDGEVAGGPGRLSNIVFMGMGEPLANYKRVVNAVRRITSPAPDGLGISQRNVVVSTVGLAPAIRKLADEDLSVTLAVSLHTPDDELRDTLVPVNNRWPVAEVLDAARYYADKSGRRVSIEYALIRDINDQPWRADMLGKKLHKALGSRVHVNVIPLNPTPGSKWDASPKPVEREFVRRVEAQGVPCTVRDTRGQEIAAACGQLAAEG

>CORE_REP|Org150_Gene5131#

MPSLDNAGSHTPREDSGATDAAASQAGTDAAAAHADTGAAASRAGTDPAASRVGTDAAAPRAGSDAAGGADTAVRVSGADPAVVVDDVRKSFGEVQALQGISFTAARASVLGILGPNGAGKTTTVKILSTLLRPDSGSASVAGHDVVADAAGVRRSIMMTGQYAALDENLSGRENLELFGRLMGLPKKDARRRADTLLEEFDLVGAGKRAVRHYSGGMRRRVDIACGLVVRPEVVFLDEPTTGLDPRSRQGVWDLVNALKEQGITVLLTTQYLEEADVLSDNIIVIDKGTVIAEGTADELKEKTGGSYCEVVPLDPTQLRKAVTALGELVPEALRHEFAGDRISIPAPDGASTLAEAVRRLDAAGLELADIALRRPSLDDVFLSITGHSGGHQ

>CORE_REP|Org113_Gene518#

MSAIPTVARLRPFGATIFAEMTELAVRHDAVNLGQGFPDTDGPAAMLEAARTAIADGVNQYPPGRGMPVLRRAIAADRRLRYGTDYDIDREVLVTVGATEALAAAVLGLVEPGAEVVLIEPYYDSYAAVVALAGATRRTAHLVPDGTGFALDLDSLRAAITPKTRMLLLNTPHNPTGAVFSRADLEAIADLAREHDLIVVSDEVYEHLVYDGNTHISIATLPGMYERTVVVSSAAKTFSVTGWKIGWACGPAPLIDGVIAAKQFLSFVGGGPFQPAVAYALEHEQAWVRDLRDSLSDKRIRLSEALGDAGFTVQRSDATYFVCADISPLTSADALTFCRELPERLGVAAVPLSVFADDRPSWDRLIRFAFCKKDETLDEAVRRLHAAHGAARS

>CORE_REP|Org155_Gene1979#

MELSSMKFRVAREDFAESVAWVARSLPSRPPVPVLGGVLLVADEDGLTVSGFDYEVSAQMRVAAEVAGPGQVLVSGRLLADITKALSNKPVDVSVDGTRVLISCGSAKFSLPTMPVEDYPQLPEVPQQSGELNAEVFAEAVAQVAVAAGRDDTLPMLTGIRVEIEGPQVVLAATDRFRLAVRHIEWQPARPDIETAVLIPARTLSESAKTLGATDAPVQLSLGTGAGADGLLGIVNAGRRTTTRLLDAEFPKFRQLLPKEHTSIATLQVATLTDAIKRVALVAERGAQVRLEFSGEGLLLSAGGDDAGRAEEWLEADFRGEPLTIAFNPGYLIDGLSALHSDRVTFGFTTPSRPAVLLPASEDEEPQPLDSGSFPALDSAYIYLLMPVRLPG

>CORE_REP|Org218_Gene1339#

MRSITAAQRRARLGVRHRLAHPQRSADVADIARSLVVLHATDPATVFLSVAARGAGITPADIEQALYEQRSLLRMLAMRRTMFVAPVELVPVLQAACADALADKQRRTYGKFLEQAGIGDGDVRSWWAEVEEQTHRALLARGAATGAQLSKDVPLLRTQVNTAPDKAYSRPTNITTWVLVTLGAEGRIVRGRPNGSWSSSQYTWSPIESWLPGGVPVIPADDARDELVRQWLHAFGPALVGDIKWWTGWTLGEVRTTLARLDITEVELDEGTGVILTDDLDPVPEPQPWAALLPALDPTPMGWQSRAWYLGDHAAALFDRNGNIGPTIWCDGRIVGGWAQRKDGEIALRLLEDVGSDAVAMIESEAERIGAWFGDVRPVPRFRTPLEKELTA

>CORE_REP|Org31_Gene5179#

MSERSERTEGTAGLGFSTRAVHAGFDPDPQTGAVNVPIYASSTFAQDGVGGLRGGFEYARTGNPTRSALEANLAALESGRYGRAFASGMAATDCAVRATLRPGDHIVIPDDAYGGTFRLIDKVFSQWGIEHSPAHVFDVDEMRAAIRPNTKLVWVETPTNPLLSIGDIPALADVAHAAGAKLVVDNTFATPYLQQPLLLGADIVTHSTTKYLGGHSDVVGGALITNDPELDAAFAFLQNGAGAVPGPFDAFLTMRGTKTLAVRMDRHCDNAETLVEFLAGHPAIAQVIYPGLPEHPGHQVAAKQMRRFGGMISVRLHGGADAAREFCSRTKVFTLAESLGGVESLIEHPAAMTHASTEGSALEVPADLVRLSVGIEDAADLLADVEQALAS

>CORE_REP|Org56_Gene2793#

MGRTLLITNDFPPRPGGIQSYVHSLALRMPPEDLVVYAPRWRGDSHLRFDAQQPFRVVRHPTTLMLPTPLVLRRAATLLRDERCDTVWFGAAAPLALMSPALRRFGADRILASTHGHEVGWSMLPGARQVLRVIGDHTDVVTYVSKYTRRRFAAAFGAQAALEYLPPGVDSTVFRPDPAARAELRARYGLGERPTVLCLSRLVPRKGQDMLILAMREIRRRIDGAVLVIAGGGPYEEKLRALVRALDLESDVVFTGRVPAAELAAHHTLADVFAMPSRTRGAGLDVEGLGIVYLEASATGVPVVAGNSGGAPETVLEGRTGTVVDGRDERAVAAAVVDILSDRDAAARMGAAGCEWVADQWRWDVLGGRLRGLLDPGAHSGVLGSDGSVR

>CORE_REP|Org194_Gene679#

MTQEVVERVEALLPTLRDRAQEAEDLRRLPEESVKDLQETGFFKLLQPRQWGGHAADPVVFYDTVRKIASACGSTGWVAGIIGVHNWHLALFDQRAQEEVWGEDTDVRISSSYAPMGAGLVTEDGSGYTVNGSWAWSSGSDHADWVVVGGPVIKNGKPVDFGSFLIPRTEYRIDDVWNVVGLRGTGSNTVVVENVFVPKHRFLSFRAMSDLKSPGLEQNTDPVYKMPWGTIHPTTISTPIVGMAYGALEAHVEHQGKRVRAAYAGENAKDDPFGKVRIAEAASDIDAAWRQLSGNVADEYAHLVAGREVPFDLRARARRDQVRATGRAISSIDKLFEASGATALANGTPLQRFWRDAHAGRVHAANDPERAYVMYGTHAFGLPIADTMV

>CORE_REP|Org37_Gene2611#

MRCLDVRSARIASAAPAAPTEPGTAATPALRFATTARNRSGSTEPGGRQRTARGGTRWLLAVLGVVLLAGVTTACGGSGVTGEQQGATTATLANPDPTPIGPEPVPALPVTVHSFDGAEVTVTSADRIVAVDRYGTLAQTVWALGLGSKLVGRSTSAAFPAVRDVPNVAGGNGSINIEAVAAQRPTVFLTDTTSATPTMRDQLRALGITVVYFDPQRTIDGVGPQIRSVADALGVHDQGVKLADRTHAEIAAATAAMPKRQPPLKIGFVYLRSTAITMLAGPGSGADSLVTALGGVDAGTAAGLTEPFTTITSEAMISAAPDVLLVMSDGLKSVGGVDGLEKVPGVAQTPAGRDHRVVDMSDAVLLSFGPETGHVLEALSKAVYGTQPA

>CORE_REP|Org150_Gene5117#

MDFTRDESQDAVAEVVVSLLEHDSARDLALWPALVDSGLLALPLPEQHGGDGMGLLEVSTLLTELATDAVAVPALSTLGFGVLPLVGHVGDDLAAKVFPAVAKGAIMTAALHEPGAPFVAEPQTKAVADGDSVRITGRKIAVPYAEQAQWILVPTDRGIAVVDGDANGITRTESPSSTGAPEFSVAFDDTVIPAAQLLDIPLEDLHRIALATLGAVADGLLKGVLALTSEHLRTRQQFGRPLAEFQAVAQEIADVYVVSRALQVAATSANWSLAQYVGRSPARAWTDGVGERSGGAKEGRTEPSGAATRDAGGGAKDTARVDDDLDVLAFCVASELPAAMQRCHHLHGGIGVDVTHPLHRYYSQAKDIARWLGGASFRLDRLGARCTSI

>CORE_REP|Org101_Gene7468#

MNTSRGGQPAGWVSTGGRQPAEPAGWVSTGVAPQAGVHSRWPGLIAAYRDRIAGARDWEPVTLLEGGTPLVPAPHLSELTGCEVYLKVEGLNPTGSFKDRGMTVAITDAKYQGQKAVLCASTGNTSASAAAYATRADMTCAVLIPQGKIAMGKLAQAVMLGAKIIQVDGNFDDCLELARKVTSDFPSIGLVNSVNPARIEGQKTASFEICDVLGRAPDVHALPVGNAGNITAYWRGYREYYADGVTTQLPRMLGVQAAGAAPLVHGAPVSDPETIATAIRIGAPASWNAAVAAKEESGGAFRAATDEEILAAYRLVAASEGVFVEPASAASVAGLLAARTEGWLDSGLTVVCTVTGNGLKDPDTALLGMPQVQAIPVDPVAVAAELELA

>CORE_REP|Org15_Gene4571#

MDLFEYQAKELFVKHGVPSSEGRVTDSAEDARAIATEIGKPVMIKSQVKVGGRGKAGGVKYAATPDDAFTHASNILGLDIKGHVTKKILVAEAKDIAEEYYISFLLDRANRTYLAMCSVEGGMEIEEVAATKPDRLAKVPVDAVKGVDLAFARSIAEQGHLPADVLDAAAVTIQKLWEVFVAEDATLVEVNPLVRTPENEILALDGKVTLDENADFRHPDHAEFADRDATDPLELKAKENDLNYVKLDGEVGIIGNGAGLVMSTLDVVAYAGEKHNGVKPANFLDIGGGASAEVMANGLDVILNDAQVKSVFVNVFGGITACDAVANGIVKALEMLGSEANKPLVVRLDGNKVEEGRKILVEANHPLVTLAQTMDEGADKAAELAAAK

>CORE_REP|Org72_Gene4483#

MRPVAVISDAIAADTVSVPPEAATVTLADLPLRESLRGHTPYGAPQYDIAVRLNTNESPHPPSTAMIDDLLASIRAVAAELHRYPDRDALALRADLAAYVTRRTGVEVSADNIWAANGSTEILHQLLLAFGGPGRSALGVTPSYAMYRIAAECLGTAWLSVGEPADSAPDIEQMVTAITEYQPDIVFVTTPHNPTGALLAPSDLERLLRIAPGLVIVDEAYGEFSAAPSAIGLIDEYPAKLVVTRSLSKALAFAGARVGYLVATPAVIEAMLLVRLPYHLSTLGQTAARVALRHADEALARAAEVVAERQRVSRSLHEMGFRVRDSEANFLLFGPFPDPPRAWQRYREHDVLIRDVGIPRTLRVTIGSPEENDRFLAVSAGLLTGESH

>CORE_REP|Org210_Gene809#

MGTPVIVEAARTPIGKRNGWLAGLHAAEVLGAAQRGVLERAQLDPALVEQVIGGCVMQVGEQGNNVTRTAWLHAGLPWQVGATTVDCQCGSAQQANHLIAGQIAQGAIDIGVACGVESMSHVPLGANVGENAGPRRPASWDIDMPNQFEAAERIAKRRGITRDDIDEFGVRSQRLAAQAWAEGRFDREVLTIAGAPQVDKEGTLTGETLDVNRDQGLRETTRESLAKLKPVLEGGIHTAGTSSQISDGAAAVLLMDEQAAARAGLKPRARIVTQCLVGAEPEFHLDGPVQATTRLLEKSGMSIADIDLFEINEAFASVPLSWASVHKPDMDRVNVNGGAIAIGHPVGSTGSRLITTALHELERSDKSIAMVLMCAGGALATGTIIERL

>CORE_REP|Org215_Gene2838#

MRVAVVAGPDPGHAFPAIALCLRFLEAGDEPVLFTGPRWFDAAKQVGIGVRRLKGLAPRAEDDDADAGQRIHERAAFISTEILPDMSAMLPDLVVSDVLTAGGGMAAERMKVPWVELSPHPLYLPSKGLPPIGSGLAPGEGLSGRARDAVLRGMTARAIRQGEEQRERARAGIGLPPEDPGPAARLVATLPALEVPRPDWPDNAHLVGPLLWEPTANVLDLPPGDDPLVMVAPSTAHTGVSGMVDTVLEALDGAGVRVAISMLDTPPAELPPWATAGLGRQDELLRHAAVVIGGGGHGLLAKSLLAGVPVVTVPGGGDQWELANRAARHGSSLLVRPLTAEAVRTAVRRILDEPTFAERARKASADASVVSDPVPLCHEVAAAARTHR

>CORE_REP|Org6_Gene5593#

MSTPVPTRERRARRCFRNVWGMRLVAVGRVAMCVALGSALLAGCARFDDSASSPFTPEPTFSPADPRPPDQPPSSTTRPSGPCIDPDPSVVATCLDTTGGLVGVGHGALVAERRTGRILEVVDPDTPPVEVATVSVDGSGDGGLLDIALSPTYGEDGLIYAYITTGSDNRVVRLAEGGPPKDILTGIPKGATGNRGALEWATPDRLMVLTGDAGNPGLANSPGSLAGKLLRLDSPAPGSAAPQIVAAGIGTPGDLCRDGSDNVWFTDRTAVEDRLQRMDPSGAVSVAWTWPDRPGVAGCAVAADGVAVALTYAKALAIAPTDPNTLAVTTAPTLMVQDRYGQLGGATIGPDGTVWVGTVNKAEGTPGPNDDRVVRVPPPSGGGGGGPD

>CORE_REP|Org101_Gene5781#

MRRAARGGGDLSPAGPVGACTLTRMPLSAFVRTRNRTATPGSGSARVAPRRLAAVVALAATVVAAGCSKTDDASTIVRTTTNIAGAGVVGLERDTTRACPLPSAPDAANGSTRTVTHAAGVSEVPADPKRIVVLTTSALDATCAVGLWERVVGAVTIDGPSPQPAYLGTGVLKIPGVGTAAQPNPALIAAQHPDLIIGDIPTATASFDALQAIAPTVLVGANNSWQAEFTALAAGLGRKAAADAALEDYRTAATDTGNVLSSGQTQASVLRFTGDTNQIQGSNSFAGQILADAGVQRPQAQRGTTFDVRPDEFAGKLEGDLIYVLLAGADGKKHGEQVMRSAAFKDLGASTDKRVFAVEDTVWHGNGLTAARALLTDLTGTLNGFVTD

>CORE_REP|Org1_Gene5478#

MPGPVVVSDTSPTVPASTGLWSGIMNGFLLARPDGVLRASGVRAAFDAVNDARAALRTGGAAIVVGALPFDPARPAALVAPAEQVHTAGPWRPAALPPLPRVQVVSEFPSAGEHLARVTKLVEQLDDPDTELRKVVAARSVLAEADGVLEPETVAAQLAARHPGASVFAVDLTAAGRTGATLIGASPELLVARRGRTVTLHPLAGTAPRRADPDADAAQAAELLDSAKNREEHSYVIEWIRDVLTPLCTELRIPEGPRLVETHDVWHLATPIVGTLREPAPTALDLAVLLHPTPAVCGTPTAAALETITRIEGDRGFYGGAVGWCDADGDGTWVVAIRCAELAADGRSLRAYAGGGIVAASQPQAELDETTAKLRTFLGGLDCAVPTH

>CORE_REP|Org132_Gene4629#

MSDFLSTGTLPEEYRELALTVRDFANQVVAPVAAKHDAAHTFPYEVVSGMADMGLFGLPFPEEYGGMGGDYFALCLALEELGKIDQSVAITLEAGVSLGAMPIYRFGNEAQKQEWLPQLTSGRALAGFGLTEPGAGSDAGGTRTTAVRDGDDWIINGSKQFITNSGTDITRLVTVTAVTGESEGKKEISTILVPTDTPGFVAEPAYNKVGWHASDTHPLSFTDVRVPQSNLLGELGRGYANFLRILDEGRIAIAALSVGAAQGCVDESVRYAGEREAFGRAIGRNQAVAFKIARMEARAHAARTAYYDAAALMLAGKPFKKQAAIAKLVASEAAMDNARDATQIFGGYGFMNEYAVARHYRDSKILEIGEGTTEVQLMLIGRELGL

>CORE_REP|Org80_Gene5463#

MAGNPDFDLFKLEDFHDELRAAIRGLAEKEIAPYAKDVDGNARFPEEALTALNAAGFNAVHVPEAYGGQGADSVATCIVIEEVARVCGSSSLIPAVNKLGTMGLILNGSEELKQKVLGDLVNGKMASYCLSEREAGSDAASMRTRAKQDGDDWVINGSKCWITNGGKSEWYTVMAVTDPDKGANGISAFMVHKDDEGFVVGPLEHKLGIKGSPTAELYFENCRVPGDRIIGEPGTGFKTALQTLDHTRPTIGAQAVGLAQGALDAAIAYTKDRKQFGKAIADFQNTQFMLADMAMKVEAARLMVYTSAARAERGEQNLGFISAAAKCFASDVAMEVTTNAVQLFGGAGYTTDFPVERMMRDAKITQIYEGTNQIQRLVMSRALLKG

>CORE_REP|Org85_Gene5120#

MSFVETEEQQALRAAVAALAAKYNYRDYVLPKARANEPLTELWDEAGKLGFLGVNLPEEYGGGGAGLYELALVMEELSAQGAGLLLMVVSPAICGTIITKYGTDEQKQTWLPKLGDGSAKMVFGITEPDAGSNSHQITTTARRDGEDWILNGRKIYISGVDQAEAVLIVSRTEDHKTGKLKPALFIVPTDAEGFHKTPQEMDIIEPDHQFTLFLDDVRLPANALVGKEDAALMQLFAGLNPERVMGAAMAIGLGRYAIDRAVQYAKERTVWKTPIGAHQGISHPLAQVKIELELAKLMMRKAATLYDLGDEMGAAEAANMAKYAAAEASIKALDQAIQTHGGAGLTKEYGLAAMLAAARIGRIAPVSREMVLNFVAQYSLGLPKSY

>CORE_REP|Org103_Gene5508#

MSNTLRTLPMPTGSGVGDVLPHLREAMEGNGPAWLPIPTTDRREARRLADALRPGDPIDDDVALVVTTSGTTGVPKGAMLSSSALRASGTATHDRLGGPGTWLLALPTHHIAGLQVLMRSILAGTEPTVLDVSGGFLPEALAGAISGMRGERRYTSLVPTQLIKAIEEPEATAALADLDAVLVGGAATPAPVYERARELGINVVRTYGMSETCGGCVYDGVPLAGTLVRIEDGRVVLGGPMIAKGYRGQPDHPAFAEPGWFRTEDAGTYDNGVLQVTGRLDEAITTGGLLVIPQVVEAVLVTHPAISECVVLGLPDERLGQRVAVAVVPAEGARPTLEELREHVVRELDAIAAPRELAILDELPLHGPGKPNRNKLRELLLTRSHT

>CORE_REP|Org17_Gene4449#

MSTAITLGMPAAPAAVLAPRRKTRQLMVGTVGVGSDHPISVQSMTTTKTHDVNATLQQIAELTASGCDIVRVACPRQEDADALPMIAKKSQIPVIADIHFQPRYIFAAIDAGCAAVRVNPGNIKEFDGRVKEVAKAAGAAGIPIRIGVNAGSLDKRMLEKYGKATPEALVESALWEASLFEEHGFGDIKISVKHNDPVVMVEAYRQLAAQCDYPLHLGVTEAGPAFQGTIKSAVAFGALLSEGIGDTIRVSLSAPPAEEVKVGGQILQSLNLRPRKLEIVSCPSCGRAQVDVYSLANAVTAGLEGLEVPLRVAVMGCVVNGPGEAREADLGVASGNGKGQIFVKGEVIKTVPEHQIVETLIEEAMRIADEMGTDAETGDPVVTVG

>CORE_REP|Org14_Gene2957#

MDLELDSAAAQFQLEVREFLRANVPAEPLPSMDTREGFEAHRAWEHTLADARLSVVSWQREYGGRDASLLEWVLFEEEYYAAGAPGRVSQNGIFLLAPTLFEHGTPEQLERILPRMARADDIWAQAWSEPEAGSDLAGIRSSAKRVEGGWVLNGQKTWSSRASFADWAFGLFRSETEEERRSKGLAASRHSGLTYVMFPLSADGVTVRPIPQLDGEPGFAEIFLDNVFVPDRDVIGEPGAGWRVAMSTSSNERGLSLRSPGRFNATAARLIELWRETADPADTAARNRVVDAWIGAEAYRLNTLGTVTRLSEGGKLGAESSINKVFWSELDIAMHETALDLLGASAEQSSAWTDGYLFSLAGPIYAGTNEIQRNIVAERLLGLPR

>CORE_REP|Org135_Gene889#

MFTLNDDERAIRETARDFADEFLAPHALEWDEHMHFPIEVLRKSGSVGLGGIYVAEDVGGSALRRLDAVRIFEELATGCPAVAAYISIHNMAAWMIDAYGDDGQRFRWLPGMTSMEILGSYALTEPGVGSDAAALTTKAVRDGDDYILNGAKQFISGAGANDVYVMMVRTGEEGPRGISALIVPADTPGLTVGPNEKKMGWKAQPTRQVILTDARVPVANRLGAEGDGFRIAMNGLNGGRLNIAACSIGGAQAALDKTVPYLAQRQAFGAPLLKNQALQFDLADMRTQLEAARTLLWRAADALDADADDKVELCAMAKRFATDAGFEVANKALQLHGGYGYLAEYGLEKIVRDLRVHQILEGTNEIMRVVVARSMTSAARGAGAA

>CORE_REP|Org210_Gene5906#

MRDRACTKRFPEEQVNFKRSGALLGVLAAAGTLTLTACGSDDNSAATGNTTKVDVACGGKKALKASGSSAQKNAMDRFIAAYEQNCDGAKLDYTSSGSGAGVNEFVGGQTDFGGSDSALDPKKDEPKKAADRCGAPAWNLPTVFGPIAITHNLDGVTNLTLDGPTAAKIFNGTITKWDDPAIKGLNQGVNLPSDEIHVIFRSDESGTTDNFQRYLDAASNGAWGKGAGKAFAGGVGEGAKGNEGTSAAIKSTKGSITYNEWSFARSQNLSTAQIITDAAVKPVALNTESAGKAIAAAKIVGQGNDLIIDTNSFYKPTDPGAYPIMLATYEIVCSKYADADTGKAVKAFLTSAITNGQNGLEDSGYVPIPDAFKTKLTTAINAIS

>CORE_REP|Org203_Gene3689#

MAPVESDDGGGAVTTLAELIEVLDAAYPPKLAESWDSVGVVCGDPAETVRRVVFAVDATADVVDEAIEWGAQALVVHHPLLLRGVDSVAADTPKGAVLHRLIRSGCALFSAHTNADSADPGVSDALAATLGLTVTGPLDPKPAAAVDKWVVQVPRTDTERVLAALFAAGAGASGKYRDAAWKVAGIGQFRPMEGADPAIGAIGEIAYVDEDRIELVAPPAARGAVLAALRSAHPYEEPAFHLTERAPLPGSLGIGRIGTLPEPETLRAFTARAAAALPKTTWGVRAAGDPERLIQTVAVCGGAGDSYLDTVTRRKVDAYLTSDLRHHPADEHLRRHGPALIDAAHWATEFPWCAQAESIVRAELPDLETRVSTLRTDPWTVSAS

>CORE_REP|Org144_Gene3837#

MLTHPVGSTPCYSPEPTSPFLVIYPERVRENYRALHAAMPAARIRFAVKASPVPELIQVLDEEGAEFDVASIGEIELCLELGVEPATLCYGNPIKKAADIARAYALGVRRYAFDTEDDLLRITEHAPGSQVECRFLASAPESRTPFGTKFGCAPAEALRLLVRARDLGLVVAGPYFHVGSQQLDPNAWRIGIEQAGRIVEALADKDIHVTSVNIGGGLPIAYADPAPALDEIATVVGTAAAEYLPATAALVVEPGRALVGSAGVIHAEVVGVRIAPDGRRWVYLDIGRYNGMAETENEYIAYRFVTDRDGDPVDEAVVAGPTCDGDDVLYQRTRVLLPTTLQAGDRVTILDTGAYTASYSSVSFNGFPPLTVHVSGAEPTRPAG

>CORE_REP|Org105_Gene5334#

MLCDTGHNPDSDVQEAVKEMLGKPSYPVQLIQPDGRRVLDREHAAVVADVGPDRLRDLYEDLVVTRRIDTEATALQRQGQLGLWAPLLGQEAAQVGSARALRPDDYVFCSYRESAVAYCRGVDPARLTRMWRGVAHSCWDPDAVNMTNPAIVVGAQGLHATGYAYAAHLEGADIATIAYFGDGATSQGDIAEALGFAASWSAPVVFFCQNNHWAISEPVRLQSATPIAQRALGYGIPSVQVDGNDVLAVLAVTRQALARAHAGGGPSFIEAITYRMGPHTTADDPTRYRSDAETEEWKRRDPIDRVHRLLDRENLLDEQFEQRVRDKADEIATVVRTATIDMPDPDPMELFDHVYSTEHPLIAEQRRAYAQHLAAHAPTEGVPS

>CORE_REP|Org144_Gene4802#

MTDIEEVLGRLRRYPDVEALNLYAVDAADRLILDVAADALTTEDSGKIAVIGDAYGALTIGAIAAHDLRAVRVHQDLLTGELALANNARALGFADRYTAHPLGAGLLDGVRVVLLRLPRALAGLSEVADAIARYADPEVVVFAGGRDKYLTKSMNEVLAQSFSEVRASRGRQKSRTLLVTGPKPVGSPPFPVRDRLDDLDIDVVAHGAAFSGARLDIGTRFLLQHLKWMKPDAREAIDLGCGTGILAVALAKARPAIKVVGTDQSAAAVASARATVVVNEVADRVSVVRDDAMSSAAANSADLVLCNPPFHVGAAVHTGSAIKMFAETGRVLRPGGELWTVFNSHLNYRGVIERMVGHTDVVGRNRKFTVTRSVRGLHDAQQR

>CORE_REP|Org9_Gene214#

MCRVPIRREPEGPLTLKKTASILLSSLAGATALLLTATTPAAANPNAINPIPVLNGTNGLPNLLGRTKAVFQVTGMASPNNTHAYNVLGTDLGIMWDNGHGEMLTAFGDTAGVGFPNLLAGSTWAWRSNILVRSHTKNPANGIYFDSVVRDVFGQARDLIPSPKIPFVEISRIPTAGISVNGVQYMSLMSVKSWDTVGQWTTNFSGLAASADNGETWADLGHTRRPNEGGNANFQMNAFLKSGGYIYEYGTRSGRNNAAFVARVREEHIENLGEYEYWDGNGWRKNDVNAAAPIMHGVGELSVMYNDYLGQYISLTTDPFNSVVMRRASSPEGPWSAPEVLIDTRELPTAYAPSIFPYQTGRDLYFLTTVHSQYNVVLMRTTL

>CORE_REP|Org112_Gene5694#

MSVVTRVTDLIGHTPLFELAATDTGTRLYLKLENLNPTGAAKIRMARAMVDDAEHRGLLSPGGHIIESTSGNTGLGLAVVAAERGYRFTAVVDHHACRDKLRAMKAMGAELVFVAEEGDDSLSTSAREELAEKMAREECEAALDAGTAPNAYFTEQHNNDANALGYYALADELLDELGRVDVLISAVGTGGSMFGTARRLRERGIEPLLYGVEPVGSIAFGGPAGPYWQSGTGTPEGADPGKIVAEDLGLLKEGVKVSDVEAFATARVLAAKLGLMIGGSAGGSVFAALRRLDDFPAGSTVVTIVCDGGEKYLDTVFDDEWMSDRDLLDAETERAVAALLDRLPSAARRDTASAADAARTPRATHTILLPADEREPVSGVAAK

>CORE_REP|Org102_Gene6006#

MRRGGAIPRGRAGAAQRRWARLAAARCRGRGHARDGTDRVVRHRLGAHTTLYRVAISKVERLMNLVIALLSTRQFLTAERIRESVAGYEESASDEAFSRMFERDKNELRDLGIPLEIGPVSRFSSVEGYRINRDAYELPDIDLTREEAAAVAVAVQLWESPELATAVEGALLKLRAAGVHVEPENGVASVPAVPARTRGAEPVLGRLLAAIDAGQAVRFPHRTSGDEYIERDVLPWGVVTQHGRWYLVGHDNARDDVRTFRISRIGERVTPYGPVNAVHKPENVDLREIVTRVTSQAPITGSATVWVAKGRGQEIRRLGATMEEREIGGRPGSVVEVPVRSRDWLARLLTGLGPDALVLAPEELRDTVIGRLRSVLEQTEVTA

>CORE_REP|Org105_Gene3199#

MAEVPGAERSLTTLSRVTLDLHADPIALTAALVDIPSVSRDELAVTDAVEAALRTQTTGFEIVRDGNVVLARTDRGLPTRVVLAGHLDTVPIADNVPSRMDTEGGEPVMYGCGTVDMKSGDAVFLHLAATIAEPAHDLTLIFYDCEEIAAEFNGLARIERERPEWLDGDLAILGEPSGGWVEAGCQGTLRARLTTAGTRAHSARAWLGDNAIHRLAPVLGRLSEYRAREVDIDGCVYREGLSAVRVAGGVAGNVVPDAAEVDVNFRFAPDRSVAQATDHVREVFAGLELDFQVTDAAPGALPGLTAPAAKDLITRVHAHGAAGVRAKYGWTDVSRFAARGVPAVNFGPGDPNLAHKRDERVPLAQITQVTAMLRSYLTGASS

>CORE_REP|Org6_Gene3417#

MTTSPAVPGGQATVPSDFVSGLEGVVAFTTDIAEPDKDGGALRYRGVDIEDLVGSRVTFGDVWALLVDGEFGHGLPPAEPFPLPVHTGDVRVDVQAGLAMLAPIWGYQPLLDIDDQTARENLARASVMALSYVAQSARGIYQPAVPQKKIDECNTVTERFMTRWKGDPDPRHIEAIDAYWVSAAEHGMNASTFTARVIASTGADVAASLSGAIGAMSGPLHGGAPARVLPMIEEVEKTGDARALVKGILDRKEKLMGFGHRVYRAEDPRARVLRATAQRLGAPRYEVAAALEQAALAELRERRPDRAIETNVEFWAAVILDFAEVPAHMMPAMFTCGRTAGWCAHILEQKQLGKLVRPAAIYTGPGPRKPAEVAGWSDISHL

>CORE_REP|Org56_Gene2495#

MSGESDTDPGQRRQLTTSARDLDDLAERLTRWLAGKLTGTTAPVVTGLSRPQAGGMSSSSVMFEASWERDGRTETGSYVARMPPEEGSFPVFETYDLDTQYAVMAGVGAHSDVPVPRLCWFEPDESVLGTPFFVMERINGRIPEDNPPYVFVGWVFDATPQQRMRITHATVDIIARVHAIADPAATFPALAGAGSSLRRHFDAQRHWYRWALADDGYRIPLLERGFDWLEQHWPADPGPDVLNWGDARPGNIIFDEFDPAAVLDWEMATLGPRELDVAWLIFIHRFFQDIATRFDQPGLPDYLRRDDVVARYEELTGHRLRDLEWYLVYAALRHGIVMARIKRRMIHFGEDTDTDDRDDYVMHRAALEALLDGTYQWDRGDR

>CORE_REP|Org105_Gene4411#

MLVRRGPRVSDEEAGVVAIRLLTGRSGERSSRMRSARRPGRTTRSGSWVKRAAIGVAAAMLVPMGVSIAGPAAPASAAFNPAGFDFWVDSGMGPIKSRIFRAKDGNTNRVVYALDGLRAPETLSGWEIDTNVAQLLTDWNINVVMPVGGMSSFYADWNAPSSFAGIPPGTGSSSGSGALNALAAGPGKSYRYQWETFLTQNLRWALRDRLGFNPNRNGVFGLSMGGSAALTLAAYHPDQFSFAGSYSGYLNISAPGMREAIRLAMLDAGGYNVDSMAPPWGPQWLRMDPFVFAPLLRDNNTRLWVSAGSGLPGPADGPTAGTVNGMALEALALANTRAFQLRMATLGANNVVYSFPNVGIHAWSYWAEEVARMTPDLSAHIG

>CORE_REP|Org105_Gene3985#

MDFSLTDEQQLLRDSVAGFLTARYELEKSRSAAKSVAGWQPEIWRGFAEELGILGATLAERVDGMGGGPTELMVIAEELGHALVVEPFVDTVVVGGGLLSRAGGEQADAVLRDIVAGSARIAFAALEPSAGESAHDISLTARRDGDEWVLDGSKIVVTSAPLATHLIISARTSGERRDRDGVSLFLTEFDTSAPSAGLEVHSYRTIDDRQAADLTFTGFRLPATALLGAEGEATANIEATLEEAIAAVAAESVGLMRKVVADTVEYSKQRQQFGQPIGQFQVLQHRMVDMYMELEQATAAAYLAAFALSASPSERARAISATKVTIARAARFIGQQSVQLHGAMGMTEELAIGHYFKRLTAIENEFGSSAYHLHRYARLTRP

>CORE_REP|Org43_Gene3725#

MAQNENIAALAAAGVSVWLDDLSRDRIRSGNLAELVRTRGVVGVTTNPTIFQGALSKGHAYDAQLKELAAQGADADADAAIRTITTDDVREACDVLAPLFEATGGLDGRVSIEVDPRFAFDADKTVAQAVDLWKTVDRPNLFIKIPATEEGLPAITAVIAEGISVNVTLIFSVQRYRAVMGAYLDGLRKARVAGHDLAKIHSVASFFVSRVDTEIDKRLAAIGTPEALELRGKAGIANARLAYAEYQDVFDGGAHTSTYQHLAAAGARRQRPLWASTGVKNPDYPDTMYVTELVAPNTVNTLPEKTLEAVADHGEIRGDTVSGTAAEAAEVFERLRAVGIDLDDVFAVLEREGVEKFEASWAELLSATAEELRAAASGSEGN

>CORE_REP|Org142_Gene4497#

MGLSDTMTDASGRVPGSSPAPLDEFPIHQTPLSLARVASSDRNFYDRSYFNAHDRDGGTLLISGFGVYPNLGVTDAYVALRDGDTVRTVRFSDALGDRSLDMRVGGYRIEVLEPLQRLRVVCEHEELSADLTWTGAFPTVQEQPHLILNGNRPIIEASRFAQVGSWSGTLQLDGREITVDPAVWTGTRDRSWGIRPVGETEPPGRAAAEPSGGFWWLYMPLRFDDFAIVVIVQEEPDGRRTLNDATRIWPDGRTEQLGWPRIAIDYRSGTRLPTAARIELTTPDGKPIEVEIRTVTDIPLHVGCGYGGDPDWQHGQWKGRDWTSSDRYDLTDPAVAGRIPYGVIDHVAYARCGEAEGWGLFEHASIGRHDPTGFADFLSVAP

>CORE_REP|Org91_Gene792#

MGGAGIERVVFHGSPRPTIGVEWEIALVDKVTRDLSNTAAAVFDSVGDLRAHDGTPQVTKELLRNTVELVTGVHNSVGEAMDDLSATMNTVRRAADPLGVDLFCAGTHPFAQWSAQQLTRSPHYDELIERTQWWGRQMLIWGVHVHVGVSHQDKVFPILNSLLLSYPHLLALSASSPMWSGSDTGYASNRALMFQQLPTAGLPFQFENWTQFEYFVHDQFKTGVFEQLGGMHWDIRPAPKWGTIEVRVCDGISTRAELAAMAAFIHCLIVDLDRRVDNGEQLPTLPPWHVQENKWRAARYGLDAIVIVDADSNERLVTDDLDELLNRLEPTAKLLGCADELASVAEIPVRGASYQRQRKVAAAAQGDLVAVVDALVKELDQ

>CORE_REP|Org168_Gene2393#

MARAAWSDDEVEAVRELARNFFEKEVVPHEEKFVEQGHPDRHLYHRAGELGLLCTAIPAEYGGGGGTFAHEAAIIEEQTLAGDGALGMPVHSSIIAPYLAEFGSEELKRRVLPKAASGEMVLSIGMTEPGTGSDLQNIKTRAVREGDEYVITGSKIFITNGWLCDGIIIAAKTDPTKGAAGVSLIFAEVGDDTPGFTRGRILSKIGGKGQDTAELFFDGLRVPASNLLGEAEGQGFYQMMQLLAQERLVTAVIAVPMMEKAVQLTVEYTKGREAFGKPLYAMQNTKFELAECATIARVARTFLDDAIVKHLRGELDIPTAAMTKYWITDQLGGVVDRCLQLFGGYGYMTEYPISQLYTGARVLRILAGSNEVMKDLIARSL

>CORE_REP|Org145_Gene4877#

MVVLRPSHRGVPVRISVPTTKIRAAAKAVVIGAAALTLGSCSLLPDSVSLQDKTRITADFENIAGIYEGNPVTVLGLEVGTVDKIVPKGTLVEVHMTINGDVKIPADAEAAIVSPSIVTNRHIELTPVYTQGEEMADGTHLPKARTRTPVELDTLIKTIDQFAAALKPQEGSEGLGPLSGRVLYPMLDGNGEKIRDTLNALSGALKVGVDNKDAISNIIVKLNELTTMLAENDQSVRDFSNRVTQMSGLLAEQAPGLQDTLDQLNAFLVNTSTTFAEHQDELQGTLTGLTNVTNQLRANAYGLTEVVDVAPLALQNIDRIVSREHGWVRLHALIGTALNGEIVSLFCERIQMRADGCRTGNMQDFGPDYGLTAALLGLTK

>CORE_REP|Org37_Gene3504#

MAWDRCGAGAAACAGCVRGAVLDIVSNGCSTGWVRWQNQNPAADDGALPGLERAGFVRSVQTPEFEGVTFHEVLCKSALNKVPQRSAVPFEWTINPMRGCSHACRYCFARPTHEYLDLDAGRDFDTQIVVKTNVAAVLRQELRRRSWRREPVALGTNTDPYQRAEGRYRLMPGIIAALTDAGTPFSILTKGTLLRRDMPLLVQSAQAVPVSLAVSIATVDEELHRAVEPGTPGPRARLELVRVLTEAGFDVNVMVAPVIPYLTDDVAHLDRLLGAIAASGGARATVLPMHLRGSTRGWFLQWLAEHHPALLRRYRQLYGRGAYVTPEYSAWLRQRVDPLLTRYRLDRHRERPARGRAAGLSEPGDDIQALRSDPQLALFG

>CORE_REP|Org63_Gene1320#

MTGDTGGSRAGTDAALVLEDGRVFRGQAYGAVGQTLGEAVFCTAMTGYQETLTDPSYHRQIVVAAAPQIGNTGWNDEDDESAKIWVAGYVVRDPARRASNWRATTTLPDELERQRIVGIAGIDTRALVRHLRTRGSMKAGIFSGDALAGPDELVARVNGQPSMLGADLAGEVSTDALYTIEPDGEHRCTVVAVDLGIKTNTPRMFAQRGMRVHVVSSSTPLEQILELKPDGVFLSNGPGDPATADAAVELTRGVLGKGLPLFGICFGNQILGRALGRDTYKMKFGHRGINIPVVEHETGRISITAQNHGFALEGERGERFDTPFGTAEVSHVCANDGTVEGVRLVDGRAFSVQYHPEAAAGPHDAAYLFDRFAGLMEGA

>CORE_REP|Org101_Gene2264#

MTRAADIDPRAHPSPGHHPSSNRCAMHADLDDQRTMSVSPLRSPAEVRRVHPITDELAGTVRKGRAATVDVLNGADDRLMVIVGPCSVHDPAAALDYARRLAAKAAELDDRLHVVMRVYFEKPRTTLGWKGLINDPHLDGSFDVNTGLGIGRKVLVDITALGLPVACEFLDPITPQYIADLVSYGAIGARTAASQVHRQLSSALSMPVGIKNGTDGDVQVAVDGVRAAAASHVFPGTDLDGRAALIRTTGNPDCHVILRGGSTGPNYDAASVAEACLRLEKAALPQRLVVDASHGNSNKDHNKQVDVVTDIAERLAAGEPGVVGVMLESFLVAGRQDLTLGKAADLTYGQSITDACLDWETTASQLDRLADAVAQRRNR

>CORE_REP|Org105_Gene3211#

MSTGLSSTGHAARVRVSSLLPDFPWDTIAGAKAKAAAHPGGIVDLSVGTPVDPVDPLIRAALNSVAEVPGYPTTHGTTALREAAVAALRRRYGITGIDQAAVLPVIGTKELIAGLPRLLGFGAGDLVVIPEVAYPTYEVGGLLAGTRIARADGLTQLGPESPALIYLNSPSNPTGKVLGVEHLRKVVAFARERGAIVVSDECYLGLSWEGRAVSVLDPEVCDGDHTGLLAVHSLSKTSNLASYRAGFVTGDAELIAELLEVRKHSGMMVPLPIQAAMTAALGDDAHENQQRERYRARRETLRTALLAAGFRIDHSEAGLYLWSSRDEPCRDTLDWLAERGILAAPGDFYGPAGAKHVRIALTATDERIAEAASRLGAG

>CORE_REP|Org105_Gene4449#

MPDFTESAFKATKATSIYFRYLRNSPMPAVPEARPRRRKVSRVRLLVTGGAGFIGANFVQQTVTERPEVTVTVLDALTYAGNRASLEPVADRIDFVHGDISDLDLVDELVSGVDAVVHFAAESHNDNSLTEPWPFVQTNIVGTYSLLQAVRRHDVRYHHVSTDEVYGDLDAADPAFTEQTAYNPSSPYSATKAASDLLVRAWTRSFGVRATLSNCSNNYGPYQHVEKFIPRQITNLIDGVRPRLYGAGHQIRDWIHVDDHNRAVWDVLERGRIGQTYLIGADGELDNKTVVRLILEAFGRDPDDFDHVTDRPGHDQRYAIDASLLRDELGWRPRYADFRAGLADTIAWYRANEDWWRPHKESTERAYAAAGEKTISPN

>CORE_REP|Org1_Gene658#

MRMTTAFPTIPDDLKPADGRFGCGPSKVRPEQLESLVRVGGSVFGTSHRQKPVKDVVARVRSGLRELFSLPDDYEVVLGNGGTTAFWDAAAFGLIRERSLHLTNGEFSSKFAAVAKGNPFIGDPIVVSAEPGSAPEPVADPAADLIGWAHNETSTGVAIPVQRPAGSEHALIAIDATSGAGGLPVTITDADVYYFAPQKCFAADGGLWVALMSPAALARVEEIKSSGRWTPEFLSLPVAIDNSTKEQTYNTPAIATLLLFADQIEWLNGNGGLDWAVKRTADSSSRLYQWAESSEYATPYVTDPAHRSQVVGTIDFADSVDAAQVAKILRANGIVDTEPYRKLGRNQLRIGMFPAIDPDDVSQLTRSIDWVVEKLS

>CORE_REP|Org161_Gene4102#

MEIVHPDVSADLAELDATLKTVESVLDIEELRRRIDELEHQAADPDLWNDQDHAQRVTSELSHAQGELRRVEDLRRRLEDLPVLYELAEGEEGEARTAALEEADAERAALHSDVEAMEVRTLLSGEYDKREALVNIRSGAGGVDAADWAEMLMRMYIRWADRHGYPVEVYDTSYAEEAGIKSATFAVKTPYAYGTLSVEMGTHRLVRISPFDNQGRRQTSFAEVEVLPVVETTDHIEVPETEIRVDVYRSSGPGGQSVNTTDSAVRITHIPTGIVVTCQNEKSQLQNKISAMRVLQAKLLERKRQEERAEMDALKTNEGASWGNQMRSYVLHPYQMVKDLRTNYEVNNPSAVLNGDIDGFIESGIRWRMRESQAS

>CORE_REP|Org30_Gene3953#

MSSHTDSRFAGDVYADRLERAVQLMRAAHLDALLITPGPDLRYLIGSAADSFERLTCLVIPADKSTPSVVIPKLELASLDGSAVSDLGLQVADWVDGIDPYQIVKSALHVGSRVAVTDSMPALHLLPLAESFSGLPVSATPVLRELRMIKDAAEIEALREAGAAIDRVHARMGEWLLPGRTEAEVAADIREAIVAEGHTEAEFVIVGSGPNGAIPHHMQSERRLQQGDVVVIDIGGPVPTGYNSDCTRTYVLGEPRSEVATRYAELEDAQAAAVAAVRPGVSAESVDAAARDPLKAAGLGAAFVHRTGHGIGLSVHEEPYIVEGNELELRPGMAFSVEPGIYFRGDWGARIEDIVVVTEDGCESMNQRPHGLTVL

>CORE_REP|Org1_Gene1712#

MCGDQEGVSVAHKASEEIGARPVRGRQGRILRRAGLAVSLGITAMLVSGCSIDNVWLRFGWPSGVTPQATRMRELWTWSIIAALAMGVLVWGLTFWTVVFHRKKKDSPEFPRQTGYNVPLELTYTAIPFVIIAVLFYFTVVVQNYVHEKVADPDVTVDVTAFQWNWKFGYREVDFKDGGYQFNGIDTAREEAAQAQLKEYEERVDTEHGHPQPGPVHGKPENDILSYLHYDTVETVGTSTEIPVLVLPTGKVIEFQLAAADVIHAFWVPEFLFKRDVMPNPKENHSDNVFQITEIEKEGAFVGRCAEMCGTYHSMMNFEVRAVSPEKFTRYLDERRAGKTNAEALAAIGESPVATSTRPFNTDRTVKSAAAPEAE

>CORE_REP|Org1_Gene3714#

MKIGMVCPYSFDVPGGVQAHVVELARVFLERGHKVSVLAPASEGTPLPDFVVSAGRAVAIPYNGSVARLSFGPMAYTRIRRWIDGNDFDVLHIHEPNAPSLSMLALKIAEGPIVATFHTSTTRSLVLSTFQGVLRPYHEKISGRIAVSELARRWQVEALGSDAVEIPNGVDVPAFARAPMLPGYPRPGGTVLFLGRYDEPRKGMQVLLAALPELVARHPDVEILVVGRGDEQRLRREAGRHARHLRFLGQVSDAEKASAMRSADVYVAPNLGGESFGIILIEAMAAGTAVVASELDAFRRVLRDGTAGMLVPVGDDVALAGALDTLLTDTERREALVRRANQVVGEYDWPVVAEQILRVYETVTVGDTRVRAAG

>CORE_REP|Org85_Gene6646#

MPIPSNQTPRSAVVPNGRSARSHGISVVVPIYRGEDTVGGLVAELHELTRPSTTAGGVTFQVDEIILVHDHGPDRSDVVLQELERAYPEVRTIWLSRNFGQDAATIAGMSAANGDWIVTMDEDGQHDPRFIGTFLDAALTERAELVYSKPSNTRPHGFLRNLTSRGAKLVLATLFAFPDSTRFESYRLIRGAIGRQLAEVASNGVYLDVALTWVVGNVAQVPVVLRAEGREESGYNYRRLFSLFWKMVLCSGTRGLRLVSMLGVTLALGGGLLAAFIVYEALTTDNWAPEGWASMIVVLLLVSGAILFSLGLIAEYLGVALHILVGKPLYLTVDSPTPRPQPLPSAVEDPAADATAELRTLTTTANRGNDRVDH

>CORE_REP|Org45_Gene5914#

MRVLAAMSGGVDSAVAAARAVDAGHEVVGVHLALSTAPGTLRTGSRGCCSKEDAGDARRAADVLGIPFYVWDFADRFKEDVIDDFVASYAAGETPNPCLRCNEKIKFSALADRAVALGFDAVVTGHYARLADGVLRRAVDADKDQSYVLAVLTAEQLARAMFPVGDTPKPLIREEAATRGLAVANKPDSHDICFIPSGDTRAFLGAKIGVRPGAVLDADGTKLADHEGVHGFTIGQRKGLGLPGPAADGKPRYVTDIDPETGTVRVGSAADLEVWTVLAERAVWTSGAVPDGPIECVAQVRAHGGTAPAVAEPADGGLVVRLRQPLTGVARGQAVVLYRPDAERGDQVLGSGTISGTERERHSYATELVSDATA

>CORE_REP|Org30_Gene3886#

MPRLAPVTTATRPSSSAGIPLTVSGHYRETETRSRVREVVVDVAHRFVTTKGVRLHVAEQGQGYPVVFCHGFPHTWYVWHRQMEAVAKAGFHALALDMRGYGRTDIPDDADAYTNEAVIGDLLALLDDIGAEQAVFVGLDFGAALLWELALRAPERVRGLVVLNNPFTPRSPRVPSSYWSKMAEKHFLHLEYFREPGIADAALAAHPREFLARVYYALSGDYHYLDTWQNPPGISYLEALPQAPALPWSWLSTDEFDTLAAEFERTGFTGGLSWYRAIDRNWELTADFAGATVTLPVYFVYGENDPDMEGFSGRDPLDTMRAYVPDLRSVEKVNGAGHLVQLERADAVNAFLLSSLADLGVAGALAENQVESA

>CORE_REP|Org46_Gene6162#

MVDLINLVQTLTSPHPLDRYLELVRPTLTVRDMRAEITHVRRSAPGSVTLTLRPPRQWKGHVAGQYVQIGVVIDGVRHVRCYSPVNPEGGRDRRIQLTVKAHPDGLVSQYLYRHAAAGMVVDLTPADGVFRLPEPRPERVLLISGGSGITPVLSMLRTLAAEDHPGEVVFLHYAKSPAVLPHRAELDAIARRHRNFRIELRYPHRIQDVAPRVDPDAPWVDLAPVKGGGYFDYDELERVAPWFAAAQTYVCGPQSLMDAVRTIYQAEQLEDRLHTEEFTIALAPVDAAEAHGTVNFSASGVSARNDGATLLEQAESAGLSPEYGCRMGICFSCTAVRRSGCTRNLRTGETDSDPDQPIQLCINAPVGDVEVDI

>CORE_REP|Org101_Gene5082#

MRGQRYRLAVAPGPLHRPPVYGVMLSAMRIGLSINYSGGFKEAAAEVVDLERAGLDIVFVPEAYSYDAVSALGYLAAKTSRLELASGILQIYTRTPSLTAMTAAGLDFVSDGRYILGLGASGPQVIEGFHGVPYDAPIGRTRELVEICRKVWRRERLEYHGKYYQIPLPEGQGTGLGKALKLINHPVRERIPVLLASLGPKNVELTAEIAEGWQPVFFLPEKAKDVWGDALAAGLAKRDPQLGDLQVYAGPALAIGDNVEPLLAFVKPYLALYIGGMGAKGKNFYHTLATKYGYGAEADRIQELYLAGKKEEAAKAVPDALARDVSLVGPAGYVKERIAAFAEAGATVLNVVPMAATPAERVKLIEQLRGLCD

>CORE_REP|Org102_Gene5145#

MADKLLLVTDEKLLQGPIHAVHVELGATFAPFGGWEMPVSYAGTVGEHTAVRTAVGLFDVSHLGKATVKGAGAAAFVNSALSNDLGRIRPGKAQYTLCCTDEGGVIDDLIAYYVSDDEIFLVPNAANTAAVVAELAKASPDGVTVTDEHREYAVFAVQGPKSVEVLTALGLPTEMEYMAYADAEWEGRPVRVCRTGYTGEHGYELLPRWADAEALFRALVAQVRAAGGQPAGLGARDTLRTEMGYPLHGHELSLEISPVQARAGWAVGWKKPEFWGKAALEQEKAAGPKRMLLGLKALDRGVLRQGQAVMRGDERVGETTSGTFSPTLKIGIALALLDTAAGLAEGDEVEVDVRGRRLRCEVVRPPFVQAKTA

>CORE_REP|Org5_Gene2511#

MIVHNHHMADTSSGPVLRVAVAGASGYAGGEVLRVLLGHPEYRSGRLEIGALTAGSNAGTTLGALQPHLLPLADRVLEETTAQVLAGHDIVFLGLPHGQSAAIAEQLPESTVIIDCGADFRLTDPEAWETYYQTPHAGSWPYGLPELPGARERLRGATRIAVPGCYPTVSSLALAPAVGAGIVEPEVTVVAVSGTSGAGRKLDVGLLGSEVMGSVRAYSIAGAHRHTPEIAQNLTAAAAATGADIGDVTVSFTPVLAPMPRGILATCTARLRPDAAGAVADPAAVRAIYDKAYGDEPFIHLLPEGVLPQTGSVVGSNAITLQVAVDTAARTLVVIGAVDNLTKGTAGAAVQSMNLALGFDEAAGLSTVGVAP

>CORE_REP|Org3_Gene1213#

MVSSVRVGGGRHLTSHDKYLILGDMSVIRSAGLRGFRATVAELGGDAEEFAIACGLPVAALDTDDMLVPDQAVSAVLELAAHRLDCPDLGLRMSARQDLAMLGPLALAIRSSPALADVLECSSRYLFVHARSLSLVLEPDPYGDRGVAALRYGVRAAAAIPIQGTDLGLAFVHRTIQRLIGDRYGLRSVELPYRPPAPLSVYEEFFGAPVRAGRRDALLRVPSSLAARQLSGGDENLHRLAMEFLAQQTAATGSSAVPTVRAAVKQLLGTTPPEIGVVAGLLTMHPRTLQRRLSAEGTTFAAVLDDVRRSETRRYLTTTDIAMSQIASLVGLTEQATLTRCCRRWWGHPPTAIRKDPALAREQTSVPATALT

>CORE_REP|Org102_Gene1037#

MNLNSAVTLNTEVVQDIEADMVDSGEAFDPAAHAAAMVGHHYRVADYYEVGREKVREYARAVQDYHPVHWDEDAAREYGYDGLVAPLTFISLVGILAQRKLFEQVVTGYDLSQIMQTDQILEFHRPIKAGDRLSCIVYLHSFRQAFGGDIIVTKNDVVAQNDELVLTTYTTLIGRSGGDIDPNLSDAVRNVLMHGIGPDERPDHQAHADAANQVHAAPVPVQQTQGDVPAKHAIRFDDVTVGQELPTRIVRLTRGDLVNYAGVSGDANPIHWSDDVCKLVGLENVVAHGMLTMGLGGGFVTSWLGNPGAVKEYNVRFTSPVYVPVDRAAEIEYTGKVKSMDPETRTAVVAIVAKSQGRKIFGRATATVQLA

>CORE_REP|Org198_Gene7039#

MSHDGAERQRGTVVRQILFAAAIALAVSILLTPLLIKMFAKQGFGQEIRVDGPASHQAKRGTPTMGGVAIIVGMWAGYLGSHLIGIGYNADGPSASGLLVLGLATALGGVGFVDDFIKIRKQRNLGLTAAGKYLGQLTSAVVFGVLALQFRGASGLTPASRHLSYVRDISTVTMGVVVFLVFVCLVVVAWSNAVNLTDGLDGLAAGSMSLVLGGYVVITFWQYYHACETKPETGCYNVRDPLDLALVCAAGAAACVGFLWWNAAPAKIFMGDTGSLALGGLLAGLSITTRTELLMIVIGALFVAETLSVVLQVAVYRTTRNRLFKMAPFHHHFELSKWAETTVIIRFWLLAAIASAVGLGLFYSEYLSAVG

>CORE_REP|Org103_Gene5490#

MSPDHDQPAERREPLEADNPAADTTADLTVALPAADATAAPTADSAVPVPSATEAEPGFDPDRPAPTVAEQAAPPDAPAAEAPAAEQAVAPDAPAAAQSAPTSAEETEAGFPTIDAPFSEAEARGSAAADADSPAVDRAARATVAHTETRAPQADSLDADDEVETIVHVLRHGEVHNPNGILYGRLPGFGLSVTGRAQAGAVARALADHDIALVIASPLQRAQETAEPIAAQHGLLVRTDENLIEAGNTFEGLRVSVGDGALRKPRHWWKLRDPFTPSWGEPYLQIAHRMLAAVNKARVEAAGHEAVLVSHQLPVWTLRRFLQGQRLWHDPRTRQCSLASLTSLVYRGDTLVDIVYSEPAGGSDPTVHGA

>CORE_REP|Org102_Gene2492#

MKRRCETLIRRSVRNGVAVGRRRGARAAALIAATAVLTGLTAGIGTQSAVADPIIDNKKLLADPVAPDGSRITKAEMKDERNIRLYVYSAAMDATYPVDVQRPADTSEPRPTLYLLNGAGGGQDDASWQKKTDIVNGFLGDKNVNVVQPIGGKWSYYTDWQQPDPNLGVNKWTTFFTEELPPLIDAALGTNGINAIAGISTSGTTVLQLPEKAPGLYKAAAAYSGCAQFADPVGREFMRLTVEVWGGGEMENMYGPADSPAWVENDPVVNADKLRGVELYISTGNGLPGQYDTLNGEYALPGAYGLANQLIIGGAIEAGTQYCTANLKNKLDSLGIPATYNFRNSGTHSWGYWNDEFKNSWPVLARGLGL

>CORE_REP|Org167_Gene3195#

MYALLLRVMFLVPPERIHHLAFAAMRVAARFAPIRALVRRLAVVDDPILHSTVFGVPFRAPLGLAAGFDKNAEGVDVWGPFGWGFAEIGTVTAQAQPGNPAPRLFRLPADRALINRMGFNNHGAARAAEQLRARTATVPIGANIGKTKVVEPAGAAADYATSAALLGPLADFVVVNVSSPNTPGLRDLQAVESLRPLLRTVLETVTDGGRSVPVLVKIAPDLSDEDIDAVADLAVELGLAGIVATNTTIRRDGLHTDPEDVAAMGAGGLSGAPVADRSLEVLRRLYRRVGDRLALISVGGIETPEQAYQRVLAGASLLQGYTGFIYGGPFWTRKIHRGLAELLRRDGYTSLAEAVGAEHRGPQPQADTA

>CORE_REP|Org159_Gene5055#

MFLVSLTLGIVGLPNVGKSTLFNALTKNDVLAANYPFATIEPNVGVVPLPDPRLNKLAEIFSSERIVPATVSFVDIAGIVKGASEGAGLGNKFLANIREADAICQVVRVFADDDVVHVDGRVDPSADIEVIETELILADLQTLEKAVVRLEKEAKVKKDRKPVADAAKAAQEILDSGTTLFAAADKVDTELLKELSLLTTKPFLYVFNADESVLTDEAKVAELKASVAPADSVFLDAKVEAELLELDEESAIELLESIGQTEPGLHALARAGFHTLGLQTYLTAGPKEARAWTIHQGDTAPKAAGVIHTDFERGFIKAEVVAYNDLLEAGSMAAAKAAGKVRMEGKDYVMADGDVVEFRSGVASPSKNK

>CORE_REP|Org22_Gene5576#

MDLQEFWFVLIGVLFTGYFVLEGFDFGVGMLMPVLGRGAVSDRGARGPSVVTQAAASGPDAHAADTRRRVVLNTIGPVWDGNEVWLITAGGAMFAAFPEWYASLFSGFYFPLLLLLVALILRICAIEYRGKIDDPVWRARCDLGIGIGSWVPALAWGWVFANIVRGVPLDADHQMTGSFLDLLSPYALLGALTTGLLFALHGAVFLSLKTGGEVREDAMRTGRLLLAPTALVVGGFGLWTQLAYGADWTWIPLGLAVLGLAVAAVAHFAERDGWAFTGTALVIVAASALLFGSLFPDVLPSTIDPAFSLNVDNASSTPYTLKVMSWAAVIVTPVVLLYQGWTYWVFRKRITVEQIPPGIGLSRQPVVEE

>CORE_REP|Org155_Gene3783#

MLIRRLARPLLASAFVVDGVDTLMHPEPRVKTASAVVQQGHEKLPSDVAQKLPSDPDLLVKATAVTQVAGGAMLALGKAPRLAALALAATVVPATLTQQDFWSESDPERRAAKRTAFLKDISLLGGLMIASADTEGKPSLGWRGRRAAKGAAAAVSAALPFGASAQEGSGEALRQQLQHAAERGREIAGVAASKGAALAETAQERGPVLAEAARHRGAELAEAAKHRGAALAETAQQRGPVLAEAAKQRGAALAGTAQQRGPVLAEAAKQRGAALAGTAQQHGPEWAEIAKSRGAEIADVAKHRGAEFADVAKHRGAEWADIARHRAAELAAAAREQGAQLADSGAQAAASARDQMEPPKRGFWRKSS

>CORE_REP|Org5_Gene6973#

MGFGVGSDMRDGHTAGRDDQPPKVVLVTGASRFFGGNIVTRLVADPAVERVIAVDARMPSRDLLRRMGRAEFVRADIRNPMIRKVIDGNQVDTVVHPAALSRPPASGGRPAMKDYNVFGAMQLTAVCQKAPSVRRVVVRSSSAVYGCGPKDPAKFTEEMSARTPPHGWFARDMIEVEGFARGLARRRPDIAVAILRFPPIVGPRLASRGLQYFRSPITPTIFGRDPRMQLLHEEDAIAALAHAARSAPGGTFNVAGDGALALSQAIRRAGRVELPVPMSVFQTVGRSLMGPVMREFTTEQIDYFHFGCGLDTTRMRTELGFAPRWTTVQAFDDFIGGAALRPVIDPRWIDAAETRLLGLVGAGTGAHR

>CORE_REP|Org162_Gene5526#

MNRHDGAPAPGGATPPAGAGQQPASSADHRNAAPHPGGTTPHRSSERQLADAAQLTGAATARRAVPLLRDVTLRDGLQLTGKVLPTEHKVEIVRRLLGLGVPELEIGSLARPDLVPPMANSLEVVAALSPEELRRCWLWVATPRHVEKAAAAGARNFQYCFSVSDAHNRANIGRATEDSVAAMPAAVELARAVGGRIQLCLATAFTCPFDGPVDPERVLAIAADPRTAGADDVVLADTLGQAHPGQVAALVAAVRARNPQRRIVFHGHDTWGLGVANSLAAAAAGADVVDGSLGGLGGCPFAPGASGNTSSEDLLFATRPDWFTPAVLGELVRMSEALLTELGEPNRSRTVEGARSKAQAFEWVIRG

>CORE_REP|Org189_Gene3692#

MTAAAQFTRIPHPAPLPEQRVQEILTAPGFGRYFTDHMVSIDYTEAEGWTNARVEPYGPLSMDPATMVFHYGQAIFEGLKAYRQPDGGVSCFRIDANAARFRRSARRMAMAELPDELFIESVRQLLEVDERWVPAAGGEESLYLRPFMFATESGLGVKPAAAYKYLLLGSPAGAYFPRGVKPVRVWLSTDYVRAAPGGTGEAKVAGNYAASLLAQAEATEKGCDQVVWLDACERRYVEEMGTNNLFFVFGSGSDARLVTPELSGSLLPGITRDSLLTLAADSGYQVEERKISVEEWRKGAESGEITEVFACGTAAVITPVGWVRSGDEEFAIGGGEPGEVTMALRETLTGIQRGTFADIHQWMRRL

>CORE_REP|Org126_Gene4339#

MRYGTARAAGLLIGFAADRIFGDPRRGHPVALFGTAAAAVESAGYRDARAAGVVHEIVLVGGVVALSAGAEIAARPSGITRGSGVVSAWRNRHGGVAGGRRGGCAGVRTAVVQGRPGRHAGLGTIVITAVGTWIALGGTTLARTGREMADRLEAGESTGAREVLPSLCGRDPEALDADGLARAAVESIAENTSDATVAPLVWGAVAGVPGLLGYRAINTLDAMVGYRNERYSNFGWAAARVDDLANLLPARVSGVLTVMLAPLVGGRPDDAWRAWRRDAGAHPSPNAGVAEATMAGALGVALGGRTEYRHGTEMRPVLGDGRTPRVPDLRRAVRLSNGVQLTAAVVAAVTAYALGRRRSARAELPE

>CORE_REP|Org6_Gene5329#

MRWDGHVSGAVPGRYADSETSRVGGVNRRILTLLAALVPVLVLGVLGTVFTVPFVALGPGPTFDTLGEFDGKQVVEVSGAELDPTTGHLNMTTVSVRDGLNLFEAFGFWASGRHGIVPRAEVYPPGVPREEIDRSNEQEFKDSEGAAEVAALHYLRLPTVVLVRQVGEESPAKDVLRPGDEFVSINGAPVTSPQDVVNAVSSQPPGAPLTVVFRRDNAEQTATVTLAARPDDASKGFLGVTPGEGARPPMDVTFNLADIGGPSAGLMFSLALIDKLSPGELGGGKFIAGTGTIEQDGKVGPIGGIQYKMMAAREAGAEAFLVPAANCNEARQRIPDGLRLVKVETLDGAVQSLAALGSGGETPTCG

>CORE_REP|Org163_Gene5601#

MDSYQGEGSTVTQPSAIDDILAEYAGLETQLADPSLHNDAGAARRVGKRFAELAPVMATYRKLETVRGDLSAAQELAADDAAFAAEIPDLERQVEELEQALADLLAPRDPHDGDDVVLEVKSGEGGEESALFASDLARMYVRYAERHGWKVEILDVALSDLGGYKEATLSIKSRDASRDGVWSRFKFEGGVHRVQRVPVTESQGRIHTSAAGVLIYPEPDEIEEVQIDESDLRIDVYRSSGKGGQGVNTTDSAVRITHLPSGIVVTCQNERSQLQNKARAMQVLAARLQALAEDQADQEAAAGRASQIRTVDRSERIRTYNFPENRITDHRIGFKAHNLDAVLDGDMDALLDALGKADREARMAAE

>CORE_REP|Org102_Gene3743#

MSEAVDADNSTATSADPSPVMNDSGVAPVVAGIGAEEPSSPVDLSAAVDVLDTARSVTVLCHVQPDADTIGSGLALAQVLHRRGVPVRVSFAEPAEVPVSMRSLPGIELLVPPDRVPAEVDVLVAVDCGSVGRLGTLRDRLAGARVSLVLDHHRSNTRFGTVNVIDESAESTAGLVVRVLDAWGESIDRPIAHCLFAGLVTDTGSFKWPRPGSHTLAERLLATGIDGAAITRTLMDTHPFAWLPMLSKVLGSARLEPAAAGGAGLVYAFVRRDDTAGVRSEEVESVVDIVRTTAEADIAAVFKESRTTPDLWTVSLRSHARPDGTPGVDVARIATTLGGGGHRYAAGYTTSGTPDQLVATLLAELG

>CORE_REP|Org145_Gene4685#

MNTVQVARPPRARGAVALRRSPPEGISAVTQAPVQTDILEIAREQVLERGEGLTQDQTLAVLRLGDDRLEELLGLAHEVRMKWCGPEVEVEGIISLKTGGCPEDCHFCSQSGLFQSPVRAAWLDIPSLVEAAKQTAKTGATEFCIVAAVRGPDARLMAQVAAGVEAIRNEVDIQVACSLGMLTQEQVDQLAAMGVHRYNHNLETAKSHFPNVVTTHTWEERWDTLRMVREAGMEVCCGGILGMGETLEQRAEFAAQLAELEPDEVPLNFLNPRPGTPFGDLEVLPAAEALKAVAAFRLALPRTILRFAGGREITLGDLGAKQGILGGINAVIVGNYLTTLGRPAESDLDLLGELKMPIKALNETL

>CORE_REP|Org144_Gene6103#

MAARIAQTSGAEHTAILGLGVYRPARVVTNDEVAGPINSSDEWIRTRSGIKTRRFASAVETVQSMSVAAARGALESAGVDADQVDCVIVATSTHLLLTPAAAPRIATELGMNGSAAFDVSAGCAGFCHALALASDLVRCGTAGHVLVIGVEKLTDTINPTDRSTAFLFADGAGAVVVGPSDVPGIGPTVWGSDGTQAHAIRQDKDWVEFFREIEEKGTDAVRPYLAMEGTAVFRWAAHSLEKVCRDAVDRAGLSTDDLNAMIPHQANGRIIEIMARVLELPENCALANDIEETGNTSAASIPLAMESLLRKGESQPGDTALLIAFGAGLSYAAQVVTLPRFAAPAAPITADTSDIESVDAEAATV

>CORE_REP|Org106_Gene362#

MNAPTPRLRTALTDLVGIEHPVVQTGMGWVAGPSLVSATANAGGLGILASATMTYEELEAAIAKTKAQTDRSFGVNIRADASDANERIDLLIRERVKVASFALAPKKDLIAKLKDAGVVVIPSIGAAKHAVKVASWGADAVIVQGGEGGGHTGPVATTLLLPSVLDAVDIPVVAAGGFFDGRGLAAALAYGAAGVAMGTRFLLTQDSSVPDAVKQEYLNRHLQDTVVSLKVDGMPHRVLNTELVQRLEHSGRWRGFAAAVSNAARFKSMTGMKWSTIVKDGLAMRKTKDLTWSQVIMAANTPMLLRAGLVEGNTQAGVLAAGQVTGIIDDLPTCKELIERIVTEAEERLDALASLRASAQPDASG

>CORE_REP|Org216_Gene3268#

MLRRSLLRGLLKCKCVRCRRRHSLVRHRAVRPGRCGGAAARRCGMVVLNPEGPSAQARLLTAACRGVVRPVLRAAPITRATIPVGALAIDGLARLRPHPRGIEREQVTMPGFAMEIIRPAGAARAMRHGALLYLHGGGFAVCGLETHRPVAASLARRTGLPVVNVAYRQLPVRSITESIDDCLAAYRWLLRHGAEPDRIVFAGDSAGGYLTFATALRALECGLPAPAGLVGLSPLLDLDYAAKRDYVNVARDPYIPLSALAAVVRLGAEREGRLDPLLSPVNGALAHLPPVLLVAAEDEVLRFDAELMAARLDAAGVPNSVELWRGQVHAFMSIAPGLPESRAALGRVARFVRGRLADSQRARTA

>CORE_REP|Org81_Gene5027#

MRGTNTAEPEVLIERRDGLGLITLNRPKAINALNHPMALAILDALREWAADDEVRTVVLTGAGERGLCAGGDIVAIHNDAKNAVAQADTAAADSPSGRFWRDEYMLNALIGRYPKPYVAVMDGIVMGGGVGLSGHASHRIVTERSKIGMPETGIGFIPDVGGTYLLSHAPGEIGTHVALTTARMSAGDAIAAGFADYFVPAEQLPALLEALRDNDADTAIAKFAQAAPESELMAQRDWIDACYSADSVEEIVARLRTHDAPEAAKAAADVLTKSPVALKVTLRSLRNARAATHLEEVLNEEYRVSVASLSTHDLVEGIRAQVVDKDRNPQWNPATLADVSVAEVDTYFAELGDKELGLTAPEGK

>CORE_REP|Org152_Gene6790#

MSGFPRERRKPRQDETGSGTAGATPRDEPSKACGECPGNQRRECDPQVTTIRELRSRIRAASSVRKITKAQELVAASRLTKARARVAAAEPYAREITRVLTELASASTLTHPLLTERPAPRRAAVLVITSDRGMCGSYNARVLERTEELLTTLRTAGKEPVLYVMGAKGLTYFGFRRRPVDGSWTGFSHSPTYADAADACRHLVDAFMAGADGDVSTPDGTGSMAGVDELHIVHTRFVSMLSQVPEVRRLAPIQVTFADESFEMGPDSFSDSPTAEVHAQYEFEPDADRLLSALLPKYINARIYASLLDAAASESAARRTAMKAASDKATNVVDSLTRSANSLRQAQITQEITEIVGGAEALA

>CORE_REP|Org210_Gene1197#

MNDADHSDDTSASGKSGDSEEPTLVALGGGHGLYATLTAARRLTERITAVVTVADDGGSSGRLRAELGMLPPGDLRMALAALAEDPDGVWARTAQHRFGGTGALAGHSVGNLVLAGLAEVLGDPVAALDEMAAILRCVGRVLPMSPTALTIEADVSGLEADPRVSRCIRGQVAVATTPGKVRRVRLIPSDPPASPEATSAIEHADVVVLGPGSWFTSVIPHMLVPDLREALMDTHAVKVLVLNLAAEPGETTGFSAERHLHVLSQHAPDFAVDHVLVDSGSVPEGREREHVARAAEQLRARVTFADVAEAGTDRHHPGKLAAALDQVIRQPRPELAGLRVEGRHPVQQVRSVLGGKERVSWR

>CORE_REP|Org31_Gene1130#

MTEPDVSAPLTHPGPGTATVRTRVHELTIVEVIRETPDTVSLVFEVPEPLVPRFRYRPGQFLTLRIPSERTAAVARCYSLSSSPHLDDDLIVTVKRTEGGYASNWLCDNAVPGMRMTTLPPSGVFTPKSLDADLLMIAAGSGITPIMSIIKSALLAGTGTTYLFYANRDACSVIFGAELTAMMTEYPDRLIVDHWLESGMGLPTAEALTERLWPYTGFDAFLCGPTAFMRTARAAMLAAGMTERHVHTEIYRSLTGDPFADIVVADDDAERPATATVELDGRRLELAWPRHTPLLDLLLAGGHDAPYSCREGACSACACTVRSGEVRMLRNDTLVDADLALGLTLACQSVPLTDHVDIAFDQ

>CORE_REP|Org169_Gene6025#

MHSSGRRRTGRWTGADPGRAARPKRSSYYTGPVSFDNVRGRSTPGRPSRTPSGTPSVVGQLGRRPDGTIPFSVEFNPPRDAAAEARLWRAAREFERMHPAFVSMTYGAGGSTRDRTARITGQLARETTLLTVAHLTAVGHSVAELRSIVGSYADAGIRNMLVLRGDPPGDPLGEWRKHPDGVEYAEELVRMVCELGDFHVGVASFPQGHYRSPDLEHDTRYLVSKLRAGAEYSITQMFFDVEHYLRLRDRVAAYDAEQGAKPIIPELMPITSLRTVQRAEELSGRPLPARVMQRLEQAAGNDPEANRNAVRAVGIEIATEIGQRLIDEGAPCLHFITLNFAKATTEVLTNLGYTVTPAAVSA

>CORE_REP|Org5_Gene5551#

MLRVAWVVVSRCRADCVPRSCLPSSVGASVRRCVAGPLAAARWLARGGYVGDGGGMEELPGDLRDLLDQYERHLRLGRNRSAHTVRAYLGDARALLNHLCDRSPDASVGEIDLPLLRSWLAELAAGGAARTTMARRASAARTFTAWLTHTGQLVADPGPRLGSARAHRVLPAVLGRDQADAAMTAAESGAAQQDPMALRDRLIVEMLYATGIRVSELCGLDIDDVDRARRLVRVLGKGNKERSVPFGGPADRSLEAWLNFGRPHLATAESGRALLLGRRGRRLDQRQARTVVHDVVSAVPGAPDLGPHGLRHTAATHLLEGGADLRVVQELLGHASMATTQLYTHVSIERLRHVHDQAHPRA

>CORE_REP|Org25_Gene2925#

MSARLASPDTTGRPAAWAGSAYRGQPTEYTGRFHRSAGTAPARLTLLVVAVLLLITGCGLEAGSAVPLRVGPGSIQPVPALKGVPITVGSKDFTEQNILGYLIEFAMVAAGADVRDLTNIQGSNSLRDAQLHGQIDIAYDYTGTGWMNYLGNETPVPGELAQFDAVRDADLADHDMAWAAMAPMNNTYALVTNAATATETGVRTLSDYARLVATAPGRAATCVGTEFNVRQDGFPGMARKYGIDNDAVPKRLVQDALVYTSVADGRQCSFGSVAATDGRIPSLGLILLDDDQHFFPTYNAALVMRRDFAEAHPEVITVMAPISVLLTNETITELNRQVDVDGREPSEVARGWLVAEGFVTEG

>CORE_REP|Org31_Gene5472#

MNAIKQQLRGLGGPLTKLVIFVIVTLFATTVLALSIANYSGGGTAFKARFTDVTSLNKGDEVRIAGVRVGKVTGVSIVDKRLAEVEFELTDRDWLPASTIATIRYRNLVGQRYIALEQGAGEQGRKLNKGGTIGLENTRPALNLTTLFNGFRPLFRTLTADDVNKLSFEIIQVFQGEQGTIHDLVTTTASLTNKIADKDAVIGELVRNLTAVLDTVNKRDDQFDQLIVNTEALVSGLAAERDTIGRSVTSLGNLASATGDLLVPVRPTLQGSIAGLSQLTGTLNERKDEVDEALTNLPIKMEKLGRVGSYGSWFQFYLCGIDIVVGPGAVDAPQLNLPAGLPTINQPLYTNAAPRCSGKAR

>CORE_REP|Org99_Gene2641#

MSQAKKGGLGRGLAALIPTGPDTIPNGLTTPTAPPGTAKPKGLVTPNGLGTAAANVIIGVDPAGAKPASPLRSDTQAEELTSPSGAVYREIPPDQIEPNPKQPRQVFEEDALAELVHSIREFGLMQPIVVRRLEPGVDKYQLVMGERRWRACQEAGLEAIPAIVRETADDALLRDALLENIHRVQLNPLEEAAAYQQLLEEFGVTHEELAARIGRSRPVVTNMIRLLKLPIPVQRRVAAGVLSAGHARALLGLEAGPDAQEALAARIVAEGMSVRATEEAVTLANREPDSAATPPAPKRKPIHMPGLQDVAEKLSNSFDTRVTVSLGKRKGKIVVEFGSVEDLERIVGLMQQQQLQMSSE

>CORE_REP|Org132_Gene2671#

MDAMVLVLGVSAGAGGARAMLTHSDQPHLPPIDRCVVARRAGAGVEEPVFQAIDAMRASAADRDEFVTGIAVTSRCALHAEAIRAAAGRSRLTIIDEPPAQLRYLRFSGQLPHEGAVLLYDLGSSGLTLTEADCRTQAILAGKRSTLLGGDGHDALLRWHLAHGGVEIDNATGREYKEQLSGTRVVTAGDPRSGMRIVVTRSDFHDLVVAGIHHSVSYVRQLIEETGVPPRAVALLGGCTRSPSIRESLEQLLDLPVIYDPEPDFVSARGAVLMATEQPSARRVRGIRFTPPQCGSAASTPRAAVSRRKVVAALAVTAALGATVTGLLVTGHDAARPAHDGTPARPVEVAGSSAEPPPGK

>CORE_REP|Org4_Gene703#

MGVDLPRVAEPGKIAAMRIGVLTGGGDCPGLNAVIRAVVRTANGRYGDAIVGFEDGWRGLLEDRKIQIHNDDRTDRLLAKGGTILGTARTNPDVLRAGLGRIKRTLDDNGIDALIPIGGEGTLTAASWLSDEGVPVVGVPKTIDNDIDCTDVTFGHDTALSIASEAIDRLHTTAESHQRVMLVEVMGRHAGWIAVNAGMAAGAHLTLVPEVPFDVDQVCTMIKRRFQRGDKHFICVVAEGSHPAEDSGFALRAGGIDEFGHERFTGVAQQLGAEIERRIGKEVRTTVLGHVQRGGSPTPYDRVLATRFGLHAAEAVHAGQFGQMVALHGSAIELVPLSEATKQLKRVPSERYQEAEAFFG

>CORE_REP|Org5_Gene6478#

MLWERRACVSGRGARGHVHARKLVHMSSAIPLNVGIARSADASGVGAGKRVLLAEPRGYCAGVDRAVETVERTLEKHGAPIYVRKEIVHNRHVVETLRDRGVVFVDETDEVPEGSVVVFSAHGVSPAVHESAAARNLHTIDATCPLVTKVHQEAKRFARDDFDILLIGHEGHEEVEGTAGEAPDNVQLVDGPDAVDGVHVRDEDKVIWLSQTTLSVDETMETVQRLRARFPKLQDPPSDDICYATQNRQVAVKAMAPECDLVIVVGSRNSSNSRRLVEVALNAGAAASYLVDFAREIDPSWFEGVRTVGVTSGASVPEILVRGVLDLLAEHGYGEVQPVTTANETLVFALPRELRTSARR

>CORE_REP|Org31_Gene4493#

MPGGHNGQVTLVEMGIPAVRSGRPSLDGRPDTPVLLDRFGRVARDLRVSITEKCSLRCTYCMPEEGLPAIPQDELLTVAEIVRLVRLAVRELGVQEVRFTGGEPLMRRDLEQIIAGCHEQVPHVPLAMTTNGVGLEHRARGLAAAGLHRVNVSLDTVDRAGFATLTRRDRLGSALAGIRAARDAGLAPVKINAVLMRETLSGAADLLQWCLDEQCELRFIEEMPLDADHEWARANMVTAAELLEVLGTRFALTAAGRADPSAPAETWLVNGGPATVGIIASVTRKFCDTCDRTRLTADGMLRSCLFSDQEYDLRRVLRSGADDHELATLWRGAMWNKWAGHGIDAEGFVPPERTMGAIGG

>CORE_REP|Org4_Gene2114#

MAAHRRQGTLRLDTKRLVGGALAAGVLATTTVYGAGPVGADPVALPATAADAVQRMVDLSRQSEQLNEQALNAQSDLDTKLGLQREADAKLAASTDQVNRARDEVRKYQPIIDRTAIAAYQGARTNRLFAVLVSDSPQQLLDQMSTLDVLAAQTSDQLALYKKATDAAEGAEADARRASDEARAAADKAETVRGELERKRSDLSGAIVQVVQAWTGLSTKDKSALAGPAFPPGFDRDTLLQGLVPGSGTSALAASLTRIGDPYVWGATGPHQFDCSGLVQWAFKQVGKDVPRTSSQQASYGTPVAQNDLQPGDVVFFYNDISHVGIYAGNGLMVHASTFGVPVAVAPISTTPYHSARRY

>CORE_REP|Org105_Gene2969#

MSALLDDLGDRARSGLGRLTDRPDFALVGVLRIPEAQTSPWISLTRRVLFAIGLLFTAALVVYVGRDGYRDASENPLSFLDALYYATVSLSTTGYGDISPVTPLARLVNIIVITPLRVLFLIVLVGTTLSVLTERSRQAFKIQRWRHSVRNHTVVIGYGTKGRTAIDAMLGDGASPSEIVVVDTDSVALEAAANAGLVTVHGSASQSDVLRLAGVQNAASIVVATNRDDTAVLVTLTARELTKSAKIVASIREAENTHLLRQSGADSVVVSSETAGRLLGIATTKPTVVEMIEDLLTPEQGFAVAEREVEPGEVGGSPRHLSDIVLGVVRAGELIRVGEPEVDALDHGDKLLYLRRAGR

>CORE_REP|Org134_Gene157#

MHDRGGTVGRTADALLLLSFGGPERPEDVMPFLENVTRGRGVPRERLAEVADHYLHFGGVSPINELNRQIIAAVEDELSAAGTDLPVYFGNRNWHPMVEDTLAQMTADGVRSALVFPTSAWGGYSGCLQYQEDIVRARGAVGDGAPELTKLRQYFDHPLFIESFADAIRAAVQQIPADRRDRIRLVFTAHSIPISADVSAGPPADGGRLYSRQVSDAARLCVAATGFTDYDLVWQSRSGPPQVPWLDPDIVDHLENLSGKGVDAVVVCPVGFVSDHLEVIWDLDNEAAEKAAELGMAFARAGTPGTDPRFAQLVVELIREQMDDAPARCLGSVPGYGGTVNGQACAVDCCKPPARPGR

>CORE_REP|Org12_Gene4210#

MRAKRGGAEPPTSATPARCPVWPTYSRWQAVSMRPKPGGAEPPPWASPALLPCGDKSRSDRQPLIRTVQSGAAAAWSTVDTGHSTRHPGGVLRSTRILPVLLVLVAAALLLPARASADAAIDDTTSHRIDELVALRAGATGASKGDLLDLLSRQFLGTPYAANTLIGSATQPEQFVADFRQVDCFTFLDYVEALSRTTDRNRFEANLIDTRYAAAQVDYTHRKHFFTDWAQVADVAATDITASLTPAAITVPKHLNAKGDGGVYLPGIPVVDRDITYIPSAAVDEDVTSGLRTGDYIGAYADQPGLDVTHVGILIKTPSGPVFRNASSLAANNRVVDTPLGEYLQTVPGIVVLRPQPA

>CORE_REP|Org79_Gene5061#

MRQKIIVDVDTGVDDSLALLYLLASPEAEILGIVSTAGNVGAAQVAVNNLAWLELCRAPEIEVALGSAEPLVIALRTTEDTHGPQGVGYAELPAPSRKVSDRNAPDLWVESAREHPGEIIGLCTGPLTNLALALRREPALPRLLRRLVVMGGAFNHPGNTTPTNEWNIHVDPEAAKEVFDAFSAAPPDRRPLICALDITETIEMRPEHLTRLAERAGSVPAERVSPSDPPGARSATSNPIIRHLTDAVRFYFDFHHSYDLGYLAHMHDPFAAAVALDPALARTRPATVDVELTGTLTRATTVADWAGMWGREPNADIVVGTDPELFFDRLISRVGDFAAAVYPARTGLIPAHAQEAP

>CORE_REP|Org138_Gene4545#

MNGQRSSVINVGIVGIVLAVTISLASLQFDRLPFLASGVQYTAHFGDAGGLVPGDQVQVAGVRSGRVEDVRLDGAKVLVRFSIDGGITLGDRTSAAIKTNTVLGRKSLEVTPAGDGRLRRDEPIPLERTTSPYSLNDALGDLATTVHGLDMEQVNTTLDALSATFADTPAPLRSALDGITALSRSINTRDQALTELLSKAQGVTQVLSDRAAKISALLVDGNQLLGELDARRTALGQMIQYVNGLAQQLSGFVNDNEAQLQPTLDRLNSVLGMLQRNEQNLSDALDALGPYAGALGEQVGGGPYFQAYISNATSKGLQPLVDALVWPEHLPEDLRSYLDNSPQQPPGLYPALTEPGK

>CORE_REP|Org210_Gene2958#

MLECSREQAEVLVPERSKDARTGFIAGRFGEFPARWGQGLSDQLTRIARSPFGPSEEEVRANLAGEASADAALALHDAELAESYGAEADSELPPHARPPRDLTAAAFFDVDNTMVQGASIVHFARGLAARKYFKTSDLVDMAWKQVKFRVTGKESQGDMASGKEKALSFIAGRSTAELAALGEEIYDEIIADKIWPGTRALAQMHLDAGQQVWLVTATPVELAQVIAKRLGLTGALGTVAESVDGVFTGRLVGDILHGLGKAHAVRTLAIREGLNLKRCTAYSDSHNDVPMLSLAGTAVAINPDSDLREVAKNRGWEIRDFRTGRKAAKIGVPTALALGAAGGAAAAVLTRRREQHG

>CORE_REP|Org38_Gene4207#

MVREQTLSLETDGTQQTATLAGQRFRVRDHYEVGREKIREFARAVQNHHGAHQQESDALRLGHENVIAPPTFASVIGSAGTRSLLESVLTEYDLSQILQTDQVFQAYRPIQAGDRLSSEILIESIRQFGDNDFVVVRSALLNQHGELALIGSTTIVARRGVDVDPSVADVVDNIMMHGQVVEFPSAGSEAADGSALIPLGTGTLPGPAPDRVPAPVHTLPDFDRLSVGDQLAPGIFRLTRGDLANYAGVSGDANPIHFSDHAAELVGLPTVVAHGMLTMGLASGYLTAWLGDPTAIEKFSVRFAGFVPVAPNAASTVEFTGRIKALDPRTRTATIVLGGTSEERKLFGRAIAEVRFS

>CORE_REP|Org81_Gene3324#

MAALGAMMPLTERITDGPSGFGGVEATPEWAREIKRLARERNATILAHNYQLPEIQDVADHVGDSLALSRIAAEAPEDTIVFCGVHFMAETAKILSPEKTVLIPDQRAGCSLADSITADELRAWKAEHPGALVVSYVNTTAAVKALTDICCTSSNAVDVVASIDADREVLFLPDQFLGAHVKRVTGRENMHIWMGECHVHAGINGDELNEQARTHPDAELFVHPECGCATSALYLAGAGEFPADRVHILSTGGMIDAAKAAAARSSRSGESGTVNQVLVATEVGMLHQLRKAAPGVDFQAVNDRASCKYMKMITPAALLRSLVENRDEVHVDPETAALARNSVQRMIEIGNPGGGE

>CORE_REP|Org15_Gene6315#

MGVSVQVPEWTTELAPATAARIVELLERAGAADGVAPISEQAVLSVTASGTVESATAPAGPAATDSGSAAPASPETDPAAADHDHGDVEPPTGRTRHLPVERDGELVAYANLVPAHGDHPAMAEAVVDPRARGRGIGATLVAAALQAGGPGARIWAHGNLAPARAVAGRLGLTIARELWQMRRPLTQAPDGDQATVELPELEVPADIVLRTYAGPADDAEILRVNNAAFDWHPEQGGWTEAEIAVRRAAPWFDPKGLFIAADPADPSHILGFHWTKVHEPDETSDAVGEVYVVAIDPAAQGRGLGRVLTLAGLHYLRDRGLGAVILYTEADNTAAVHTYTRLGFETAHIDAAYTAR

>CORE_REP|Org5_Gene6981#

MRVHHEQVDGITSHIEHTEPHMNTVPGRVPGPEDEPEPAPSGGAGTGTGAGKLLASGDVTAGASAHAPVSPNEPSGGLRPAAKLDPAPEVDLDFPREWIEFVDPANDEHLIAADLTWLLSSWTCVFGTPACQGIIEGRQDDGCCSHGAFLCDDDDRRKLQKAVKQLTPQDWQLMDEARDSDGKIRKKLYLEEDDLDDEPAIRTRRYEGACIFLNRPGFEGGIGCALHTMALRTGVEPLTVKPEVCWQLPIRRTQDWVDRPDGEEILRTTITEYDRRGWGPGGLDLNWYCSGSPDAHTGSRPVWQSYAPELIELIGQPAYDELARHCRRREGLGLIAVHPATTAAQAKAEAERSGNI

>CORE_REP|Org3_Gene2083#

MVWPAPDNEWVDQMTEPREIAERLLDAQVEYLLAEVSGERFTEVVARDVAAVLEVADTIVFRDVVSIDQAEQTVATVIDLIGGSPVIADMVGVFADAIYDNIAGSHDYTLGQVVEREPVEALLEKIFAMHQAQERILDRLTESPLVATVASKFVDKLINDFMQANRERAEKIPGVSSLMSLGQSAANRAKKVAADNTFIGDMAGKGALFALRRTNNAIREMLRDAPVHGAAMEFWDLHADEPVSGLRDYLTQKDLRELVLICYEIAVTTREKEYFGLLVDECVQVFFSKYGDYTLGAMLPELGLTAEDVSTEILRYGPAVIEAAKKNGVLAGLIRERLEPFYLSDQVLAILGGAAS

>CORE_REP|Org207_Gene1186#

MIVAQEQGNVGAAATGTPLRIGTRGSLLAMTQAGTVRDALIAAGRPAELVVVKTPGDMSSDPVQKIGVGVFTSALRDELAAGTIDLAVHSYKDLPTAPDPRFVIAAIPPREDPRDALVARDGLVLGELPAGAKVGTSAPRRVAQLRALGLGLDIVPLRGNLDSRLARVTDGELDAVVVARAGLSRIGRTAVITEALEPVQMLPAPAQGALAVECRSEDAALIEALAELDDAATRAAVVAERALLAELEAGCTAPVGALAEVVESLDDDGRIVEELSLRGCAAAVDGSEVLRASVVGDPERAAELGRALARELLELGARELLVEVAATEPGARSGTGAVRPPETDLSNPSPMENPQ

>CORE_REP|Org129_Gene2372#

MSTPTRRRLPDAPFLAAATGAGPGRRPVWFMRQAGRSLPEYRELRAGIGMLESCFDPELVCEITLQPIRRHGVDAAILFSDIVVPLKAAGIELDIVPGVGPVIANPVRSVDDVRALPRLRREEVGAITDGVRLLLDELGETPLIGFAGAPFTLASYLVEGGPSRNHERTKALMLGDPQTWHALLGVLTDITVEFLRAQIAAGVDAVQLFDSWAGALSLAQYREFVLPHSERVFAEIGEAGVPRIHFGVGTGELLGAMGEAGADVVGVDWRVPLTAAARRVGPGKALQGNLDPAVLFAGSSVVEREIRRIAHEADEAITLGATGHIFNLGHGVLPDTDPGAITAAVELIHSLPTPL

>CORE_REP|Org34_Gene1989#

MGGDLFGVALTIVLLAGNAFFVGAEFALISARRDRLEALAAQGKRNANTVIRAGENLSMMLAAAQLGITICSILLGRVGEPAVAHLLEGPFHLVGLPDQLLHPVAFTIALAIVVVLHILFGEMIPKNIALAGPERTALLLVPIHLLWLRLARPLIATYNLAANLSLRMLRIEPKDELDATVSSVELAEMIGESRSEGLLDEEEHRRLTQALGTSERVVGDVMVGLDTTRTVPLRGNGTTLGDIETAVAETGFSRYPVSADDGSLVGYLHVKDVLDLVADDTAGPSTPIPRTDIRPLPTVLAGTPLYEALARLRRTSSHLGRVVDNRGNTVGIVALEDLVEEFVGTVRDGTHRVIE

>CORE_REP|Org81_Gene1827#

MGTVQEPEIRDWDFPRGVASAALMVGYARERGVGAELMLRGTGLTAQTLADPDLQIDARTELAVVRNLVRELGDEPLLGVDVGRRYRITTFGIFGFACVSSPTLGEAISFALRYLELSFTFCIPVARWRPGEFVARVHDERVPADVRRFLVQRDVTAMHQMLCDLLGRKVALVRAEFDFPAPAGAELIAEIYGVTPAYEQSHNLFALDPVELEQPLPQANESTWAMCLAQCRDLVSRRRARTGIAAEVRERLVPRGGVDGFAAPPGIDSVAKDLNMSTRTLRRHLDAAGTSYRALLDEVRRALAEEMLTATPLSVSDVAIRLGYAEASTFIHAFKRWTGTTPSAYRRNRVTPLVR

>CORE_REP|Org105_Gene3522#

MNAAEPQLRTNNRNIVQDVEHENASRRTPLSAAIRATADAGPARTFDPTNRARDTRSLSVPAGNLNRVPELPEVEALAQFLREHAVGAVVGRVDVAALSAVKTFDPPVTALSGRDVTGAGRWGKFLGLECSGIWLITHLSRGGWLRWTDNPSATPPKPGKGPLALRVHFFTPEGATPAFDLTEAGTKKRLAVYIVEDPQQVPGISRLGPDALEVTEDEFAQILDASTQRIKTALVEQSLLAGIGNAYSDEILHTARISPFATAKSLSPEKVSQLYEAMRTVLLDAVQRSVGQDAARLKGEKRSGMRVHARTGLPCPVCGDTVREVSFAERSFQYCPTCQTGGKILADRRMSRLLK

>CORE_REP|Org5_Gene6774#

MRGLSVPTRTLLIMGEPQPILDPLSRAATFLVATIDEGGEQTVRDLLADLPGLRRSVGFRVPDAHLSCVTSIGSAAWDRLFAGPKPAELHEFPGYFGARHEAPATPGDLLFHLKSDMPDACFELAMVLSSRLKGAATIVDETVGFRYFEQRDLLGFVDGTENPEGADAVAAALIGDEDPDFAGGSYVVVQKYLHDLDAWNALSVEEQQLVIGRTKLEDLELSDADKPANSHVAVNTLIDPDGTERDILRANMPFGSVREGEFGTYYIAYAATPDVTERMLVRMFLGSDEAPYDRILDFSTAITGTLFFTPSAGFFDDLPDAPDQDRESTAATELPAAAPTGDGSLGIGTLKRIPL

>CORE_REP|Org125_Gene5591#

MTVHDEVTAAGQSDSTGPRSGNEVPPGDTSSMPSESSNKPAGRDTGGDSRRGETVFRSLATAAGATIVAAIALIALFLLIRAVPSVAANKANFFTSAEFNVTNADNMHFGIRDLFMVTVLSSLLALLIAVPLGVGIALFLTQYAPKVLSRPFAMLVDLLAAVPSIVFGLWGFLVLAEKLAPFEQFLNDKLGWFFLFKDGNVSISGGGTIFTAGVVLAVMILPIITSVSREVFHLTPRAHIEAAQALGATKWEVVRMTVLPYGRSGVIAGSMLGLGRALGETIAVLIVLRTAASPGHWSLFDGGYTFASKIASAASEFSQALPTGAYIAAGFVLFALTFVVNALARIAAGGKVNG

>CORE_REP|Org28_Gene1985#

MADDDSTATPDPATAPGPVRALREIGFWLERERAKTHRVQAYRRAADIVAGLSDEERAARRAANSWRELSGIGPKTAAVIEQAYSGVVPPYLVELRAAAESIGIEGRPLRELLRGDLHTHSNWSDGGSPIAEMMRIAAALGHEYCALTDHSPRLTVANGLSAERLRRQLDAVAELNTELAPFRILTGIEVDILDDGSLDQDPDLLDELDIVVASVHSRLRDDRETMTKRMVYAVANPLVDVLGHCTGRLVMGGRGTRPESEFDAELVFEACRRFGTAVEINSRPERQDPPSRLMRLAIEVGCEFAVDTDAHAPGQLDWQGYGCARAVANEVPPERVINTKPLDELLAWTRESGA

>CORE_REP|Org71_Gene3907#

MPCRADVTLDRPASSPRRQELQPPGTRAADQNGNAVSIDNRGERIDQAELARRFEEHRPYLRRLAYSTLGSITDAEDVVQEAWLRLQRQHESGDVAEIENLRAWLSTVTGRLALDYLGSARVRREQYVGEWLPEPTVVDWDDPADKISQDERVTMALLVVLESLSPAERTAFVLQDVFGMTGPEVAEVVGRTPAAVRQLASRARKRVEDGTPRFPASPDEHEKVVSAFALAWRSGDMSALLGVLDTGVTLTADGGGKVPAVRKPVTGAELIAKLLLGWYSSPSARSWGRMVLVNGRPGLVVFDGTHTGVFSFTVDAGRITAIDVIRNPDKLHDVPDSGEPWFLPASGNDLQATD

>CORE_REP|Org139_Gene5133#

MGYRCRFGTVVRIRSAGVDQRSVPEAAIIRQALDEIGRVIVGKQQAVQSIMAAVLAGGHVLIEDLPGLGKTTIARTFAAVLGLETTRVQFTPDLLPADLVGATVYNAAAGRFEYRPGPIVTNVLIADEINRTPPKTQSALLEAMAEGQVSTDGVTRPLPAPFLVIATQNPIEHEGTYPLPEAQLDRFAMCLGLGYSTAAQEKALLRQRMSAAGGVRPRQIADAATVDRLRAATSRVDVDDDILDYIVALVRATRTHVQVEVGASPRAELDLLQVARAHALLQGRDFVIPEDVKTVAPEVVSHRVSLRPEAWMRRIRGRTIVAEVLSRTPAPRLREAAASAAGALGLAGAVDSR

>CORE_REP|Org5_Gene5803#

MSRRSSSRFRFSARRGRIAVALTALAAVGLTAACSGGSSDTPGGATPGGGAGNLTLFAYSVVKPGYDKVIAEFNKTDPGKGAQIQQSYGASGDQSRKVKDGAQADVVSFSVEPDITRLVDAGIVDSNWNADANKGVPFGSVVVMAVRKGNPKGIHDWNDLLKPGVEVVTPNPFSSGSAKWNLLAPYAAESNGGQNPQAGLDYLGKLISKDHIKVQPKSGREATETFLQGTGDVLLSYENEAIFSERSGDPIEHVIPPTTFKIENPVAVTKNAKNPAAAVAFKDFLYSQAGQKAWAEAGFRPVDPQVAEQYAKDFPKPQKLWSIADLGGWKQVDKQLFTPDTGSVAVLYDKATK

>CORE_REP|Org80_Gene3445#

MTFRPRRSSPPVKGRHLDHGSGRSALVIIKEDSAVKTPREDQRSTERPAKPSGELASGHGTDPSTEAFIAHRNLLFTVAYEMLGSAADAEDVLQETWLRWIKVDPRQVRDERAYLVRITTRQALNRLRTMNRRKESYVGPWLPEPLLTTPDLTADVEFAESMSMAVMLVLETLSPTERAVFVLHEVFDIGYDDIAAAVEKSPAAVRQIAHRARRHVDARRPRSVVSVREARAALESFRRAVETGDPQVLLDVLAPEVVLVSDGGGIKQAALRPVSGADRVVRYILGGIGRTEATVTVEPTVLNAGPALVFRLDDEIDGVMAFRFEEGSITGLYFVRNPQKLTRIETETTLTVR

>CORE_REP|Org64_Gene326#

MLISQRPTLTEEVIAENRSKFTIEPLEPGFGYTLGNSLRRTLLSSIPGAAVTSIRIDGVLHEFTTVPGVKEDVTDIILNLKGLVVSSEEDEPVTMYVRKQGPGTVTAGDIVPPAGVVVHNPDMHIATLNDKGKLEIELVVERGRGYVPAVQNKASGAEIGRIPVDSIYSPVLKVTYKVEATRVEQRTDFDRLILDVETKNSISARDALASAGKTLVELFGLARELNVEAEGIEIGPSPAEADHIASFGLPIEDLDLTVRSYNCLKREGVHTVGELVARTESDLLDIRNFGQKSIDEVKVKLHALGLSLKDSPASFDPSSVVGYDASTGTWSDSGTFSDNDGGEQDYAETEQL

>CORE_REP|Org114_Gene7067#

MSGESGRLHDAKTREILLVAHPGRAELTETAHRVAKIFEQFGIGLRVLADEAYSTRFDTDEQGRPDGYPVRVMEHGPEAAIGCEMVLALGGDGTFLRAAELAREANVPVLGINLGRIGFLTEAEAENLDEALAQVVRRDYRIERRMTIDVTVRVDDTITERGWALNEASIENSARMGVLEVVLEVDGRPVSSFGCDGVLVATPTGSTAYAFSAGGPVVWPELEALLVIPSNAHALFARPLVTSPDSRIAVETVATGHDAIVFLDGRRTLALPRGGRVEAVRGTEPVLLVRLDSAPFADRMVRKFQLPVTGWRGRSSARMGAEEEARVPTQRPRAGDVEHRRTESTSADRDQD

>CORE_REP|Org117_Gene3998#

MSTAAAAYAVSAADGSFEKVTIARRELGPHDVLIDVKYAGICHSDIHTARDEWGGTRYPCVPGHEIAGLVAAVGSAVTKYRPGDRVGVGCMVDSCGQCPPCLADEEQYCLRGATMTYNTPVPEEVQPGGHTLGGYSTQVVVTENFVVRIPEGIGLDVAAPLLCAGVTLFSPLRHWQAGPGKRVAIVGMGGLGHIGVKLAAAMGAEVTVLSHSLSKQEDGKRFGAHHYYATGDKQTFRDLRNRFDLIINTVSADLPIDSYMRLLRLDGTLVILGLPENPLSVRPFTLANYRRSLAGSMIGGIAQTQEMLNFCAEHGIGAEIELISADEIDNAYDRVVASDVRYRFVIDAATM

>CORE_REP|Org144_Gene5066#

MSTSAVSEASERAIDAACDESTVVAGVDLGDTDLAATVRAGLEEVEKLLVAELSDGEEFLQEAALHLAKAGGKRFRPLFTLLTGQLGPRASDPALVTAGTVVELVHLATLYHDDVMDEATVRRGAPSVNSRWGNNIAILAGDYLFAHASRLTSTLGPDAVRIIAETFAELVTGQMRETMGARETQDPVEHYLRVVWEKTGSLIAAAGRFGGTFSGASLDHVERLARLGDAVGTAFQISDDIIDISSATEQSGKTPGTDLREGVHTLPVLYALRDEGADGDRLRKLLAQPLSTDAEVEEALELLGRSHGMVLAKEKLHGYADLAHAELSALPQGPANDALERLVRYTIERVG

>CORE_REP|Org13_Gene6761#

MRFGRSAAPVSHSALAPESAPRGRRGAQRGWRTRLLAAGAAMALPIAAGIMAPAAIAAPVHAPVHQTPAGGYDELMVPSSMGPIKVQVQWARNGGNAALLLLDGLRARDDRNAWSFETNAQQMFGNDNVTLVMPVGGQSSWYADWQGPSNTNGQKFTYKWETFLTKELPDFLSNYGVSRTNYAVAGLSMSGPAALRLAAFHRDQFKYAASFSGPLNWNAPGMREAIRVMMLDAGRFNVDSMAAPWSPQWLRSDPMVFAPQLRGLPMYISAASGLPGQYDHPNGLVGAFNTGNAMGIELISMVSTHSFKARLDSLGIPAAYDFPPTGTHAWLYWQDELAKARTGILAALNA

>CORE_REP|Org113_Gene376#

MNYVLQPFSQELDVRVTDTSLRDGSHHKRHQFTATEVRDIVAAVDAAGVPVIEVTHGDGLGGSSFNYGFSKTPEQELITIAAQTAKQAEIAVLMLPGVGVKEDIKISQDNGASIVRIATHCTEADVSIQHFGYARDLGLETVGFLMMSHSQPPEVLAKQARIMADAGCQCVYVVDSAGALVLDQVTDRIAAVVAELGDDAQVGFHGHENLDLAVANSIYAVKAGAKQIDGSARRFGAGAGNTPVEAFIGVADKIGIKTGIDFFAIADAAEDVVRPVMPQECLLDRQALMMGYAGVYSSFLKHAERQAERYGVSAAEMLVRAGKRKLVGGQEDQLIDIALELQREQAGASA

>CORE_REP|Org35_Gene172#

MTEHHEAPGGASKALRSSDGPEGGSSRNHEAANRTHKDIPQATVARLATYLRVLAMLADDGVLIVSSEELAVAAGVNSAKLRKDLSFLGPNGVRGVGYDVAKLRTRIEDVLGLSEGHRVVLVGAGNLGRALVGYGGFRRRGFTVVGLFDNDPALIGRTVAGLRVRDAAELDAVIATLEPTIAVIAVPDDAAQQVCDALVAAGLQSILSFAPCELVVPATVEVRRVDLAVEMQMLSFERVRNAESDRWPSDGHAGGSERNHGGPREATDSIQPAAVGERGDSIPVAHPLGSGRVARRAANTHPQPAHPGRAHPAAATQHPATPQHPATPQHPATSHSATEPTSKGSVVTP

>CORE_REP|Org4_Gene1586#

MSEPTGQDTPIETGAAAQVDSASATPGDPAAEPVVGGSDSSAVTPAGTGPDARIVAVVVTHKRRELLAESLKVIASQSRPVDHLIVIDNANEAEVAELVRDQPIESTYLGSAHNLGGAGGFALGMLHALSMGADWVWLADDDGRPDGAEVLATLLDCARRHGLVEVSPVVCDIDEPDRLAFPLRRGVVWRRLRSELGDEDFLPGIASLFNGALISAKAVDVIGVPDLRLFVRGDEVEVHRRLVRSGLPFGTCLQTAYLHPNGAAEFKPILGGRMHTQYPDDPVKRYFTYRNRGYLMSQPGMRKLLPQEWIRFSWFFLVTRRDPAGLREWFHLRSLGRHEQFGKPDPRG

>CORE_REP|Org136_Gene5349#

MITFSATTVLVLALIAAVVVAIGLWAYSTANRLDRLHVRSDQSWQALDGALARRAVVARAVAMAIAGPVSDPALAEQAKQLSTLADRAERAGRADRETVENQLSAALSAVDIGQLRPQLVAELADAEARVLIARRFHNDAVRDTLALRTRRPVRLLHLGGTAPLPTYFEITERATPAAATGLEVDTIRTSARTVLIDEQDRVLLMRGNDPKVPEVSFWFTVGGGVEPGESLRAAAVREIHEETGYTADPAALRGPIWRRVAVFPFNGELIRSEELFFALRVRQFDPRPANLTYVERRSITGHRWCTAADITTFDAAGETVYPYHLDELIAEAAAAADADTDPEVRSIR

>CORE_REP|Org37_Gene1714#

MTAPTPATSRRREAPDRNLALELVRVTEAGAMAAGRWVGRGDKEGGDGAAVDAMRQLVATVSMRGTVVIGEGEKDEAPMLYNGEAVGDGTGPEVDFAVDPIDGTTLMSKGSPGAIAVLAVAERGAMFDPSAVFYMEKIAVGPEAADVIDLSVPIAENLRRVAKAKNSLVSDLTVCVLDRPRHARHIQEVRDAGARIRLISDGDVAGAIACARPESGTDMLVGIGGTPEGIIAAAALRCMGGALQGKLAPTDDAEKQKALDAGHDLDRILTTEDLVSGENVFFSATGVTDGDLLRGVRYYSGGASTQSIVMRSKSGTVRIIDAYHRLTKLHEYASVDFVGDETAIPPLP

>CORE_REP|Org15_Gene485#

MTSYAPAEALAIEADELVKVFGEQRAVDGVSLAVPQGAVYGVLGPNGAGKTTTIRMLATLLRPDGGRARIFGHDVVAEPTAVRSLIGVTGQYASVDEKLSATENLIIFSRLLGLSRSEAKRRAAELLEEFGLTEAATKALENFSGGMRRRLDLAASLIATPPLLFLDEPTTGLDPRTRAQMWETIRRLVREGATVLLTTQYLDEADQLADRIAVIDHGRVIADGTSDELKGSVGQSALQITVADRDVIERARTLIGEFLSRADGKLVEASISPEAGRVTAPLSDPSVTADLLIRLRDNDIRVDEITVSKPSLDEVFFALTGHAAESDAAESDSAESDSAGSNSEGTAA

>CORE_REP|Org101_Gene2563#

MIIMGIESSCDETGVGIVRRHADGSCELLADEVASSVDQHARFGGVVPEIASRAHLEAIVPAMRRALAVAGIAKPDALAVTIGPGLAGALLVGVAAAKAYAAAWDVPFYALNHLGGHVAVDTLEHGPMPPCVALLVSGGHTHLLHVTDLAEPIVELGSTVDDAAGEAFDKVARLLGLGFPGGPALDAAAAQGDPGAIAFPRGMTGPRDARYDFSFSGLKTAVARYVEAAQRSGLTAADLPIPNIAASFQEAVADVLTMKAVRAAQDVGVDTLVLGGGATANSRIRSMAEERCAAAGLTLRVPKPRLCTDNGVMIAALGAHVIAGGAPPSALTVATDPGLPVSVSRVS

>CORE_REP|Org159_Gene5229#

MTEAAAPATTASRDADAALTALGTPLRPFRFAAAGEGNKQEGGARKFVQTAQQAEEYGFDTFVVPDHLGEQIGPIAALGALTQATEKIRLGTSVLANGFRHPVVLAKDLATIDVLSKGRLEVGLGAGWIKEEFENAGISYESPGVRLEKLDEALTILDVLLRGQECTFEGKHYQVRGVKGTPRPRQGPRPPICTGGGGPKMLRLAAKHADIVSVVPVTTKNGKGLLSGITIEKAVEKVNLIKEAAGDRFADIELNWAITAVVITDDREKTAEMALSALDRGLHPNLEVDVQLSVEDILNSPYVAIGTFEEIAEQIRRVRQLTSMSYVGVFPTQMDAFAPVIPLLRDE

>CORE_REP|Org19_Gene5036#

MSIRKQALGFGAFAIVSILVTVVIWNTLARTVNGPTDRYSAIFSDVLGLRPNDDVRIAGVRVGKVAEIGFDVNPQTNKHLAKVTFDVQRDQHLYTDTKALVRYQNLIGQRYLALAPGTAPDSRLLAAGGRIPLERTEPSFDISAFLNGFQPLFENLLPEQVNDLSTTLIQALQGDGVSLSTFITQAAALASDFERRDAILKDIITNLSGVMSGLARRSDELETLITQTRALISGLYDQGQSLLASTTRIATASESLVAMVQQVQPKLVPAQNSTRDALTLLIDNGAKLDQAAIDLPGILSDVGKFSGDGTYATAYLCSLDVSLYGILFPRGLFSQIGGHAQSAVCRP

>CORE_REP|Org153_Gene5410#

MDELMPEAGRGPQVSLVDVASYLPGEPVGTEYFTQFSRSDRMAKNVMFRSPKTRYHVGRDETAVDMVEQAVTPLVERHGADFLAGLDVLITHTQLPDNPVMGCGPEVARRLNARPGWVFDLHNGGCAAFVHMMALAQTILRTTDARTALIAATQNCAGPVFTQSEIRKLAQAPVPGDGCGVGLLVRDDSAPILDIECRTYPEFAGDMDFTTNGDRKYWEPGEGQGCVSFTESKITKVFARGNRLVPEVALAVCDRIGVKGRDIDTFVTNQPNRLFLRNWHDALELPAERHPDTFDSCGNLFAAAIPVTLDVENRAGRLRNGSVVLMSAFAHAGDFAAAAAVRWGAAR

>CORE_REP|Org75_Gene2308#

MVADRGTQYGVGAAPPPSGPCGAGDGWLRPPAAAARTLPACAVTSPRMCRDMSADRSGRTGKALVAAALAMLALTSCTIDTGDDAQAAEPPVATTTSTPADQQRIEITLPGTLAADFQQWKAGLRGRAGLAVMPVGGERMVTFGDWTSGPAWSTMKIPLVIAAERADAGASTYAMSAAVTASDNAAADTLWQGLGGGKQAAEAVEAVLREAGDSTTAVPPTRVRAEHSAFGQAEWSLAEQVRFASRLPCLSGADTVLNLMAQIVPSHRWGLGTVGGAEFKGGWGPDTSGDYLVRQFGIIDGPGGRIAVALAAQPESGAFSDGMTALNAMAALITDHLDELAGGSCPS

>CORE_REP|Org116_Gene5513#

MATAPGAVARSVTWVTTRSYAVRVSARRPALGRSHPFAVLVGPIRLAVIQRRRRDRSPVTGESDPSGTTTGRLVLAATPMGDIGDASQRLRDALTTADVVAAEDTRRTRALAKALGVEITGRVVSFYDHVENARIPALLDDIAAGRTVLLVTDAGMPSVSDPGYRMVAACVDRDLPVTCLPGPSAVTTALALSALPVERFCFDGFPPRKSGARRAWLRTLRTEPRACVFFEAPHRLADCLADAVEVLGPDRRAAVCRELTKTYEEVVRGTLADLATWAVDGARGEITVVLAGATPTATDPTTLVPEVEALVEDGLRLKDACAQVSSATGVSRRELYDAVLSARTEG

>CORE_REP|Org114_Gene2607#

MPIATPEIYAEMIARAKENSFAFPAINCTSSETVNAAIKGFADAGSDGIIQFSTGGAEFGSGLGVKDMVTGAVALAEFATVIAAKYDVTIALHTDHCPKDKLDTFVRPLLAISADRVKSGQNPLFQSHMWDGSAIPIDENLEIAKELLKQAHAANIILEVEIGVVGGEEDGVENAINDKLYTSPEDFEKTIDALGAGENGKYLLAATFGNVHGVYKPGNVKLKPEVLAEGQRVAAAKLGLGADAQPFDFVFHGGSGSLKSEIEDSLRYGVVKMNVDTDTQYAFTRPVAAHMFSNYDGVLKVDGEVGNKKVYDPRSYLKKAEANMAARVVEACNDLKSAGRSISAK

>CORE_REP|Org30_Gene3136#

MSSPASSPVAAGERKQRVLSGIQPTSSSFHLGNYLGALQYWVTMQDDYDALYFIPNMHAITVPQEPKELRLRTRRSVAQLLAIGIDPERSTLFVQSQVPEHAELTWVLSCLTGFGEASRMTQFKDKSVKQGAENATVGLFTYPVLMAADILLYRPHQVPVGEDQRQHLELTRNLAQRFNTRFKKTFVVPEPHIVKGTAKIYDLQDPTAKMSKSANTDAGLINLLDDPKVTAKKIRSAVTDTEREIRYDPDAKPGVSNLLVILGSLTDTPIVTLEKEYEGKGYGDLKSDVADALVEFVTPLQAKVEEYMADQGELDRILAAGAERAREIAGNTLAQVYDRVGFLTR

>CORE_REP|Org16_Gene3362#

MGVRVGVVGATGQVGAVMRKLLEERDFPADEVRFFASARSAGKKLPWRGGEIVVEDTETADPSGLDIALFSAGATMSRVQAPRFAAAGVTVIDNSSAWRKDPEVPLVVSEVNPEQTRNLVKGIIANPNCTTMAAMPVLKPLHDEAGLQRLIVSSYQAVSGSGLAGVEELATQARAVIGDAEKLTHDGSALQFPAPNKYVAPIAFNVLPLAGSLVDDGSGETDEDQKLRNESRKILGLPDLLVSGTCVRVPVFTGHSLSINAEFARPLSVERAKQLLADAAGVKLVDVPTPLEAAGKDESLVGRIRQDPGVPEGRGLALFVSGDNLRKGAALNTIQIAEVLLAQR

>CORE_REP|Org113_Gene7273#

MNILRRLTFLLRPSWAILAVVVVAFAYLCFTVLAPWQLGKNTSTSHRNDLIAASVKADPVPAAGLLESADGAAPAGTNPADTEWRRVIVTGSYVPGSTVVERLQHLDDQPAYGVLAAFRLDDGRIVLIDRGLVAAADGTRLPAIAEPPAGPQRLEGRVRRSEGTIPGKDPMVGDGLRQVYSVDTTQMSSVLGMRLTPFATGEQGGYLQLDSGQPGAFTPEPLPQLDAGPYLSYGLQWIAFGVMAPLGLGYFVYAEIRERRRDRAAASAEPTDPTTSAPVPSTAPDTGTAHTAATEPTAAAPTASATMTETPPAEMTNSPSATDETKKPHRTTADRLADRYGNRR

>CORE_REP|Org34_Gene2552#

MQLGMIGLGRMGANIVRRIVADGHTAVGYERHAPHIEELGAELGASFSGTTDLAEFVSRLETPRVVWVMIPAGATGAVIDQVAELLEPGDIIIDGGNIRYHEDIQRAERLAPKGIHYVDIGTSGGVFGRTRGFCLMIGGEAGPVRYLDPLLRSIAPGVDAAPRTPGRTGEPSPAEQGYLHCGPAGAGHFVKMVHNGIEYGAMAAYAEGLNILHKADYGAGYDSGAHSAEETPLEHPEYYRYDIDIPEVTEVWRRGSVVASWLLDLTAAALHADPNLDSFGGRVSDSGEGRWTIDAAIDIGVPVPVLSAALFQRFSSRGESHYADKMLSAMRKAFGGHNELPQG

>CORE_REP|Org7_Gene7659#

MNIEIEALRAIVADKGISIETVISAIESALLTAYRHTEGHQPNARIDINQKTGTVRVMARELDADGNVISEWDDTPEGFGRIAATTARQVVLQRLRDAENEKSFGEFSTHEGDIVGGVVQRDARANARGTIVVRIGSELHGAEGLIPPAEQVPGETYEHGDRIKCYVVGVSRGPRGPQITLSRTHPNLVRRLFALEVPEIADGSVEIVAVAREAGHRSKIAVRSTVSGVNAKGACIGPMGQRVRNVMSELAGEKIDIIDFAEDPATFVGNALSPSKVVSVTIVDPEARAARVVVPDFQLSLAIGKEGQNARLAARLTGWRIDIRSDAAPDMGGGTVRTEAHRS

>CORE_REP|Org101_Gene6126#

MPRSVISARPGVGGAEGVGVLKVGVVIPKLMAVSDIHVGHQGNRPVVEQIKADSPEDWLIVAGDVGEKTDDIRWALELLRGRFAKVIWVPGNHELWTTAKDPVQMTGAARYDYLVSICRDLDVLTPEDPFPAWEGAGAEQHGGSVTLAPMFVLYDYTWLPEGATTKAEGLAIARDRNVVATDEYLLSPDPYLTRDAWCNARVQVTKRKLDALPEGTPLVLINHFPLVRQPTDVLFYPEFALWCGTDQTADWHTRYNVVCSVYGHLHIPRTSHYDGVRFEEVSLGYPREWQRRGLPDRLLRQILPAPEYPPGTLNEWGGHFQVTPEMKAAAEEMRLKARRRRGL

>CORE_REP|Org5_Gene547#

MKLKMHSSTLKLGIFTLVMVIVLAFLVVVFSQMRFSRETGYHAVFTNSSGMLPGSKVRIAGVPVGSVKSVKVGKDHLAHVDFDVDTKYRLYVSTRATVRYENLVGDRYLELMEGPGTAQVLKKGGTIGTDKTKPALDLDMLLGGFKPLLRGLDPTQVNDLTGALLQVFQGQGGTLVSLLNSGGSFTKTLADRDALIGSVIDNLETVLATIDDHNKEFDTTLTELQRLISGLAADKDPIGAALPKLAGATGDLTGLLQEARPDLKETFGQLGQLSQNIDAHSSDIEWIFQQLPTTYRKLVRIGSYGSFLNMYVCGVTMLADGPDGKPMEVHLSGNQTTGRCEDK

>CORE_REP|Org50_Gene925#

MARKVPIMATERSELVQSVVESYLLDGARRYNRTEVAEQSGTTTALTTRLWMALGFPSSIGDEAVDYADADVAAVRNFEQLTVLSSADTRQQSATARTLGQGMARLAEWQVDLVLAEIEERIARADPGADPEETVRAATEGAIATLEQLNTYAWRRHLAAALSRSLDPGTSAGESVRELAVGFADMVGYTRLTRHLHPDELSMLLEAFESTTTAAITENGGWVIKNVGDEVMFATESATEAARIALAIQESTMMVGGTPDLRVAVAYGPVLQRFGDLYGSVVNIASRLTGVARPGTVLIDDHAAAALEGDPAFTIRHLRSVRVRGFNRLRPHLLRHNGKNGK

>CORE_REP|Org48_Gene2378#

MNDVAKVIHLHDVTAETRPRPAARRWPEPPPGAPAGRVTSLLERRAASEPPVPQSLGDLVRGKLGKQISATAEFARRRLAGDYRVDEFGFDADLLENVLLPALRPLAEHWFRVEVRGIENIPATGGALIVANHAGTVPVDGLMLQLAVHDRHVAHRALRLLAADLIFELPVLGVLARKAGHTLACREDAERLLRSGELTGVFPEGFKGVGKSYADRYKLQRFGRGGFVAAAVRTGVPIIPCSIVGSEEIYPKLADIKPLARLLGLPYFPVTPTFPHLGLLGAVPLPSKWYIEFGEPIPTTAYEPEAADDPMTMFEVTDQVRETIQQTLYKLLTKRRNPFTG

>CORE_REP|Org19_Gene37#

MAHPLWEQHCCLPILPSADVTELTRYPAGSYLSVNVGYSLHSIEDALAMIHLLRRKALADGRFRLVESVGDTEVASGPQGRIALAFDLEDSRPLDGDPDNIALFHSLGVRSLLPTYNHANAAGGGCLDPDDRGLTPYGRTIIRTLGEVGMFADGSHCSRRTGLDIADVAGGPMIYSHSNFAALWPHPRNIGDDQARACAATGGVIGINGVGIFLGRNRVEGRAARIEAMADHIAYGADLVGVEHIGIGSDFSFDGDQFNAEIAAAPENFSEDYTRWGPLQWVPPEDLLGLPPHPGTDVDNPHARPRVAGLDEVLARRGFIDSERAAIFHGNFARAAREVWR

>CORE_REP|Org127_Gene3382#

MSNSGAKKLTPEQLRKLYRPGELTVVHEPAGVSAVTSALRGVGRTVALVPTMGALHEGHLELVRRAKRTNQVVVVSIFVNPLQFGENEDFDKYPRTLDSDVALLREEGVALVFAPSVAQMYPDGPRTSVHPGPLGAELEGASRPTHFAGMLTVVAKLLQIVRPHEAFFGEKDYQQLTLIRQMVRDLNFDVDIVAVPTVRESDGLALSSRNRYLDEQQRELAITLSAALAAGRHAAGRGPDAVLAAARSVLDGATGVDVDYLELRGSDLGPIPSSGNARLLVAARIGATRLIDNVPVSVPPAVSDAASAGPSVVTQAAASGPVAPADGHNAVPDFQPAQADA

>CORE_REP|Org15_Gene4277#

MACRRAGRGGAWRSLARCRRAQPSRCDSGAAGSDRSPTPRPPLAAALGRRWHGRARCRECGRGLFGRQAVGTGCACVAGGIGANRRSVGEGGHRRRTGAHRRADRRGRTAGPACRCAAHRNRPRDRRIRRRHQAEIRDSFRRRTSRPHPAADRRRAAGPADRLPALRGRTGRNPVRIPRRPARLDQRLLRRSGGGGALVSTDQQALLDALNAEYTAVYAYGLIAAYASPERAKVVAEFTAAHRARRDATVDALKAAGASVPAPAAAYTPPFPVDDPIPAAKLAVTVETDTAVAWRAVVEHSTTPDRRHIGIEALTECATRLATWQSILGVNPATTPFPGQP

>CORE_REP|Org4_Gene4426#

MPEAMHSVRGETVRIGQQPGGNGAHGSAPTVLTERFTGPGLPSRVLRRVLAYIALTKPRVIELLLVATIPTMLLADRGTIDIRLILVTLFGGWMGAASANTLNCVADADIDKVMKRTAKRPLAREAVPTSHAFVFGVVLGLASFAWLWWQANLLSGALVVATILFYVFVYTLGLKRRTSQNVVWGGAAGCMPALVGWSAATGGIGWPAIALFGVIFFWTPPHTWALAMRYKEDYRAAGVPMLPVVATEQAVTKQIVIYTWLTVLTTLALVPATGVVYAAVALVAGAWFLLMAHQLYAGVRRGESVKPLRLFLQSNNYLAVVFCGLAVDSVLGWDTVGSFFG

>CORE_REP|Org195_Gene950#

MRSIWKGSIAFGLVNVPVKVYTATEDHDIRFHQVHAKDGGRIKYDRVCTVCGQSVQYTDIDKAYESPDGDKVVLNDEDFAKLPVAEKHEIPVLQFVPSDQIDPVLFEKSYYLEPDSSTPKAYVLLARTLEEIERTALVYFTLRQKTRLAALRVREGILVLQTLLWPDEVRSVEFESLDGVAEPRSQEIKMAETLVEAMSDDFDPDQFTDEYQIELKRLLDEAIASGTGKVPEQPEPVPSGMDAEVVDLVAALQRSLEASGRRTASGDGAAAEPKKTAKKAPAKSTASKSGAAKTTASKSGASKSASKSAAKSATSKSAAKSAAKKAPAKAAKKTAARKGA

>CORE_REP|Org78_Gene4613#

MTNSCFASITVQHERPASLTLRTGGDTRVSPNRKVEGAVSVRSVLGGSAVRAFAAVVGGALVLTGCTTNTEESGPAASKVQVDKVTEIADRLPDKIKQSGKLVVGVNVPYQPNEYRDADGKIVGFDVDLMDAVTAVLGIQAEYVESAFEKIIPAIQAGTYDVGMSSITDSKEREQQVDFTTYFNAGIQWAQQTGKPIDPANACGKRVAVQATTVEHTEEVPAKSAECVAQGKPPIDIKAFDEQSAATNALVQGQVDAMSADSPVTAYAIKQSGGKIEPAGPVFDSAPYGWAVQKGSPLAAVLQAAVNHLIRNGQYKQITENWGVQDGAITESVINGAVS

>CORE_REP|Org63_Gene4835#

MNANDPFARLPEAASFTVTSTTVADGGAWSPAQFSSGVPGGKDVSPQLSWSGAPEGTASYAVTVYDPDAPTGSGFWHWAVADIPATVTELPEGAGDDTGSGLPVGSFQLPNDARAARFLGAAPPPGHGPHRYFVVVHALDVESIGVPADSTPALLGFTMASHTLGRAVLTATAETPPAEGRPAHPERVEVSRLIPAPPDAVFAVLTDPKGHVDIDASGMLIDADGEPVQRPGDRFLVHMDREALGDVPLGRYEVEVVITELVPDEEIAWTVEAHGRRFGHIYGYRLAPSEGATLVTSYCDWSQIDEKWKRRLTFPVVPQSALKATLGILERTVRRRATL

>CORE_REP|Org46_Gene4582#

MSERSERTSDTGTRVRDLIIVGSGPAGYTAAVYAGRAELQPLQFEGTQFGGALMTTTEVENFPGFREGIMGPDLMEEMREQAKRFGAEIRTEDVDAIDLTGPVKKVVVGGETFEAYAVILAMGSAARYLNVPGEQRLLGRGVSACATCDGFFFKGQDIVVVGGGDSAMEEATFLTKFASSVTIVHRREEFRASRIMLERAKANEKIKFVLNAEVAEVHGDSSVTHLTLRDTRTGETSDLPATGLFVAIGHDPRSELVKGQVALDDEGYVLVQHPTTATDIPGVFAAGDLVDHTYRQAITAAGTGCRAAIDAERWLADQGDITSNTLDHAGESVAVPAN

>CORE_REP|Org119_Gene6972#

MTAQERALVVDHEGAQRANHDEAERERGAVMSEEPTGADLAEAVVKGPPKTLAGGLFKAIRPRQWVKNVLVLAAPLAAGTANEIDVLAHVGIAFVVFCMAASGIYLVNDALDVEADRAHPTKRFRPIAAGVVPVNLAYALSLVLLVGSIAGSFLASWHLAVVMAVYIGIQLAYCFGLKHQAVLDICIVSSGFLLRAVAGGAAANIDLSQWFLLVMAFGSLFMAAGKRYAELQIALATGAKIRRSLEYYTPTYLRFVWTLAATAVVVFYGLWAFQQDSLKDTNWYAISMIPFTIAILRYAVDVDGGQAGEPEEIALGDRVLQLLAIALIGAVGVAVYLT

>CORE_REP|Org19_Gene2534#

MAVEMFYDDDADLSIIQGRKVAVIGYGSQGHAHSLSLRDSGVEVRIGLKEGSKSRAKAEEAGLTVGTPAEVSEWADVIMLLAPDTAQAKIFTEDIEPNLKDGDALFFGHGLNIHFGLIKAPAGVTIGMVAPKGPGHLVRRQFVDGKGVPALIAIDQDPKGEGQALALSYAKGIGGTRAGVIKTTFKEETETDLFGEQAVLCGGTEELVKTGFEVMVEAGYAPEMAYFEVLHELKLIVDLMYEGGIARMNYSVSDTAEFGGYLSGPRVIDADTKERMKAILKDIQDGTFVKRLVANVEGGNKELESLRKQNAEHPIEVTGKKLRELMSWVDRPITETA

>CORE_REP|Org98_Gene6035#

MEYRTLHRGKRSPAALGRLGIALVLLATIGVFFLDRVPFLGGSTSYTAEFTEAAGLKTGNEVRIAGVKVGQVSDVRLDGDKVLVEFRTSGAWIGNETTASIQIKTVLGQKYLALDPKGSAPADPDSPIPLSRTLSPYDVVDAFTDAADNIDRIDTAQLATSMQVLSEAFATTPAEIRGSIDGMARLSATIATRDEQLRKMFDATGATTAVLAERNAEFERLLGSGGALLAELNIRQQAIHQLLTGAESLAVELSALVKDNKDQMGTALTNLRTAIGTLNDNQANIAKTLELAGPFYQLFSNVMGNGRWFEAVIVNLAPPALPEIPGIRPPIRTIGGN

>CORE_REP|Org201_Gene1319#

MGDAIVAEGLVKRYGQQVALDGLDLTVPEGTVTALLGPNGAGKTTTVRVLTTLLIPDGGRATVAGIDVLRDPRALRRRIGASGQYAAVDEYLTGFENLEMVGRLYHMGVQRSKERARELLDRFRLSDAADRPVKGYSGGMRRRLDLAGALVAAPPVLFLDEPTTGLDPRARLDLWDVIEELVAGGTTLLLTTQYMEEADRLADSIAVIDRGKVIAKGTADELKTMVGGDRIELTVDHVDNLAIAQQALAGLADGEIHLEPGLRRIIVPVSNGSQALVEAVGRLNDHSVKIHDVGLRRPSLDDVFLTLTGHEAEELINADDAADGLGALEATEGKTR

>CORE_REP|Org190_Gene3432#

MGATVVGSRSNVEVVCESVPVTGDQQAPVCVLGTGLIGGSLLRAAVGAGFRAWGYNRSAAGAEAARADGFDVTGDLPAVLRRAAETDALIVVAVPMPAVDQILSAVSTFAPDCAVTDVVSVKAPVAAAARRHGLGARFVGGHPMAGTSQSGWAATDPALFRGAAWAVGVDPGTHPQPWTRVVRLALACGSVVVPVVAEEHDRAVARISHLPHVLAEALAVAGAGGGELALGLAAGSFRDGTRVAATAPDLVRAICEPNAAALLEVLDETLTVLNAARDLLEEDGSLADLTEAGHDARQRYETTERWEITDIRPGDQDWLERLREAGQRGGVITRLD

>CORE_REP|Org43_Gene3438#

MTRAAVLGAGSWGTAFAKVLADAGTEVTIWARRPEIAEALATEHRNPAYLPDVQLPAVSATHDAAAALDGAQLVVLAVPSQSLRANLTGWRPALRAAIDEHDATLLSLAKGIETGTLLRMSQVIAEVTGAEERRIAVLSGPNLAREIAAGQPAATVIACSDAARAEAVQQASYTGYFRPYTNTDVIGCEIGGACKNVIALACGIAAGMGLGDNSIASLITRGLAEIMRLAVTLGAEPVTLAGLAGVGDLVATCTSPLSRNRSFGHVLGAGGSMEAAQQATHGQVAEGVKSCTSVRALAAAHEVEMPLTDAVHRVCHEGISVREAVGSLLGRRIKPE

>CORE_REP|Org210_Gene807#

MTGVIAKGSGPEAEPAPEVLSAELRMKFDYTRSVGPTIGRFLTGLREGRIVGVRGSDGRVIVPPAEFDPVTAERLTDFVDVSDVGTVQSWSWVADPLPGQPFDRPFAWALVKFDGADTAVLHALDVSSPDEVHSGLRVRVRWAAERTGSIHDIACVEPGETSSAPAGSTSSDADAAEPVTGIVTPIDLRYKHTASPTETRYLKALAEGRLIGGRADAESKVYFPPRGADPIDGRPTDEMVDLPDTGTITTFCIVNVPFMGQKIKPPYVTAYVLLDGADIPVLHRVLGCDPSEVRMGMRVKAVWKPREEWGYTLENVDHFEPNGEPDADYDSYRHHL

>CORE_REP|Org5_Gene2060#

MDFCGVRTKAVRKLHRRRHLLRALLRCFVRDVIALFAPGQGSQTPGMLAPWLDLPGARDRIELWSKAAGLDLLQLGTTATAEEITDTAVTQPLVVAAALLAFAEIPHGSVPADTIVAGHSVGEFAAAAVAGVISPDDAVKLAAIRGAEMAKACALVPTGMSAVLGGDEAAVLDRLAELDLTPANRNAAGQIVAAGRLDALAELAANPPEKARVRALPVAGAFHTSFMAPAQDAVAEAISQMVVPDEPIRTLLSNFDGKPVTSGKDAMEKLAAQVTRPVRWDLCTETVRVAGVSAVAELPPAGTLVGIAKRELKGTPNLALKTPENIPAFVELSATG

>CORE_REP|Org134_Gene5162#

MGRVRAIRLNGFGGPEVMEWAETPDPQAGPGEVLIDVAAAGVNRADVMQRKGHYPPPPGASEVPGLECSGVIAAVGDGVRGWSVGDRVCALLSGGGYAERAVAPAGQLLPIPDGLDLGAAAGLPEVAATVWSNLVMTAGLHAGQLVLIHGGGSGIGTHAIQVAKRLGARVAVTAGSAGKLERCRELGADILINYREEDFVAVIRAEQGSGGPGADIILDNMGAAYLARNVEALATYGQLVVIGLQGGVDAELNLAALLGKRAAVRATNLRGRPANGVGSKAEIIAEVREHVWPLVTEGAVVPVIHAELPINEVGDAHALLDSADTVGKVVLHIGDY

>CORE_REP|Org5_Gene7517#

MLGPMQFQAMVAHETEDGGVVLAREEVGEDFLGPGTVTIKVHYSSANFKDGLAITPRGGVVREYPIIPGIDLTGEVVTSEDPAFAPGDEVIAHGYEIGVSHNGGFAEYARVPAEWVVKLEGVSTREAAALGTAGFTAAMSVQALLDRGLTPVDGPVLVTGATGGVGSVAVDILSGLGFQVTASTGKTDAGELLSTLGAAATIGRLPEDPDAKPRPLAKAQWAAAVDSVGGKSLAHILSTIRYGGSVAVSGLTGGTALPTTVMPFILRGVSLLGIDSVNFPIEQRRALWTRLGKDLKPAHLSTLENIAPVTEAESVLRTIREGHHSGRTVLAVAGEF

>CORE_REP|Org101_Gene3105#

MGIRFCGRWNSPTSVLATASSSTWVDSRGDGPEAALSEVSIGTLAADSNSNGVSEGLLSELVEHLRQNRTVLREEWARRITDAQLLTAMTPDEMFSEATSVYDNYVAVLETGSVEALQDYARDLSERIIPRGVETDEVLGIVLLLRDVLARSLFEKYQTDFQLLNAVLDAYEPAANRIANTVGVSFVEERERIIRQQQEAIRELSTPVLQVREQLLILPIIGVLDSQRARQLTEQLLRAIRSNRAKVVVIDITGVPQIDSTVANHLVQTVDASGLMGANVIITGLSSEIALTLVTIGLDLSKMNAVGDLQGGIEEAERLLGYEVSRSGDTSRRPEA

>CORE_REP|Org148_Gene274#

MSSPESDRNRFVALPFTVVRKGYAQDEVRNYFDRFDAELRVTATDRDAAAAQARNLASQLEDARDEIDELRKEVDRLSVPPTTAEGMSDRISRMLRLASDEASEVRALAQAEAAEMVSIAEQQATEMRGKYESLLAETKEKREALEIEFEQTLANARTEAAKIIEAAQAEADRIGKEAEAKRKAAQQDFEVTMAERRTKLTRAMEELEATSRAEAAQRIKDATDEANRLITSATQTSERKIAHAKELAEEMRVLRGRVLAQLLGIRGQLDSVPAMLAAVNRESELLDGVPDQQRKSISGNNTKSVTGSRTKAIPEVTNEEDDVVDEERENAEISN

>CORE_REP|Org100_Gene3243#

MKLAVIPGDGIGPEVIAEALKVLDVVVPGVEKTEYDLGAKRYHATGEILPDSVLPELREHDAILLGAIGDPSVPSGVLERGLLLRTRFELDHHVNLRPSRLFTGVRSPLAGAPDIDFVVVREGTEGPYTGTGGAIRVRTPHEVATEVSTNTRFGIERVVRYAFAKAQARRKHLTLVHKTNVLTFAGSLWQRTVDEVGAEFPEVTVAYQHIDAATIHMVTDPGRFDVIVTDNLFGDIITDLAAAVSGGIGLAASGNIDASGTNPSMFEPVHGSAPDIAGQSKADPTAAILSVSLLLNHLGDTEAAARIDAAVAKDLAARSGTASTVEIGDRIAAAV

>CORE_REP|Org14_Gene5066#

MTTFGTRIRNRFQSNRFFWLGVIGAVLIVVLLVVSSSYKKLGVGTKDVQAEFVQTAGVQTGDKVNVAGVPVGTVAGAKLEGDHVLITLHVNNDVKLGPDTRASIKMATLLGARYVDLDPGNGKGLPGNRIHKSNTKVPYDLADVVQIGTPKFEALDTEKLAESLKVLGDQIDGSPQLTAQALDSVGALAKTINDRRDQVDGLLKDLDKVTKILGDNRNSLLLVITQGEAIAGRVMERQELLKQLLNNTATLTKQLQQIGAENNNQLGPTIEQLNTMAQGLQKNKDNLDKLLTIMPASLRQFNNVFGNGPYGEVGVPWLFPDNWLCFARVIEGCQG

>CORE_REP|Org113_Gene2521#

MSSRLSTRLSRLLNMVPYFLANPGISAAEAATELGVNTKQLMSDLNQLWMCGLPGYGPGDLIDLSFSEESIEVTFSAGIDRPLRLTSIEATALLVALRSIADLPGMVDPTAARSAIAKIESAISGETAARGESEVPVEAPAVTTVRSALADGRALRLVYYSASRDVVSERVVDPIRIVLVDNNSYLQAWCRQAEGVRLFRFDRIEEATELDEPADPPGHATDETAGLDLFSDDPAVALARLRIRGDYGWVLDQYPMHPVVVHADGDVEATMRFATLDWIARLLLGFGSGVTVLGPPELVNAVRDRSAAALAAYDDVGDLGDLGYDHSVIEEVGPA

>CORE_REP|Org162_Gene288#

MPIRLDARTATMIAATTREWVRTVYAGGNGTGAVPAMPPEELEAVFADVIDDLMELAASEPFPVLAARTIGRRLAAPPLESTRMLAPASRALLGLAPGESGSLLATRWMFVASQAIDSYAEELQQRALAQQERNLTEAVSVRVQEIAALQHRLRHEATHDALTDLANRSLLQERVRLMAADPARGVGLLLIDLDDFKQINDNYGHAVGDEVLVAIAERLRATCPPEAVICRYGGDEFVLAVPSRRNTLGELAMRVLAALCEPVASITGSVPVSASVGTAFCPPDRDCDFSDLLRSADRAMYSAKTAGKRQFAVSELDAEEPEPGGNRAGYAAVAG

>CORE_REP|Org140_Gene4760#

MKPRQGALVLSTAFTETFGIRHPIVQGGMQWVGRAELVAAVANAGALGMITALTQPTPEDLAKEIVRTRELTDQPFGVNLTILPAITPPPYDEYRQVIIDSGVKIVETAGSNPAPHLPDFHAAGIKVLHKCTSVRHAVKAQDAGVDAISIDGFECAGHPGEDDVPGLVLIAAAAEHLTIPMIASGGFADGRGLVAALALGADGINMGTRFMCTQEAPIHRAVKEAIVAGRETDTELIFRPLRNTARVARNAVSLEVVDILNKGGKFEDVRDLVAGTRGRKVLETGDLDGGIWTAGTVQGLIHDIPTVGELVDRIVADAETVITERLSGRLARVDV

>CORE_REP|Org144_Gene4159#

MTVRPDDPAPNPHATEAEVEAARKDTKLAQVLYHDWEAETYDDKWSISYDERCIEYARGRFDAAVGPAPLPYERALELGCGTGFFLLNLMQGGVAKTGSVTDLSPGMVKVALRNAQNLGLDVDGRVADAETIPYEDNTFDLVCGHAVLHHIPDVELALKECLRVLKPGGRFVFAGEPTTAGNFYARWLGRITWKATTTVTKLPQLAGWRRPQTELDESSRAAALEAVVDLHTFDPSDLEAMASSAGAVEVKASTEEFAAALWGWPVRTFEAAVPDEKLTMGYRMAMYKAWLRLSWLDENVMRRVVPRQFFYNAMITGVKPWGMEAGAAGANSSTE

>CORE_REP|Org5_Gene3382#

MASLSVRPGSVGAMPTPSASDRPLLLLDGASLWFRAFYAIPEKITAPDGRPVNALRGFTDMVAALITRHRPGRLVVCLDLDWRPDFRVALVPSYKAHRLDTAAGAAAGAEEVPDTLTPQVGMIADVLAAAGIATAGAAGLEADDVIGTLATRERDDEVVVVSGDRDLLQLVRDEAPLVRVFYVGRGLAKAELLGPAEVAAKYGVPQENAGPAYADMATLRGDSSDGLPGVAGIGDKSAATLISRFGSLEALVAAVDDPDSNLARGVRAKLVAAQEYLKAAAPVVRVVRDAEVELSGPDTLPTAPADPDRLDALATAYNAESPFKRLTAALAANV

>CORE_REP|Org49_Gene2429#

MNAGLGPAARYLLALCARGAHPRANRKTGEDTALRTTTYRRTGLLALAATAAAGLVLAAPAQAAPLWPGGPDIPGVPSAVMPQPEPGDTPNSPNAKAPVPPANFAAPNISPGDGEVVGVAQPIIINFKEPVTDHETAEKAIRITSTNKVSGHFYWFGDKQVRWRPESFWPAGSQITVEAGGTHVAYEIGDEFIATADDSTHEITVTRNGEVVRVMPTSMGKPGHETPNGTYITSERNRKMIMDSSTYGVPVTDPEGYKLEVEYAVRMSNSGIFVHSAPWSVAQQGVSNASHGCLNVSPADAQWFFENVKKGDPVVVVNTNGGTLNPGDGYGDWN

>CORE_REP|Org113_Gene7285#

MTVLPSHPCASASGSRSAGTPGRGPTVRAVRSGVLLMVLAVTLSALGVGCGDGTETTSDAEVEPSGFGNEVTMPIAAAVASLVTPGDDPRAPLRPEVPPGTSQQVTLHTEHHVEQQINDKDAGDFSPPAVTIPLTAHTDRGGVDLTLGAATSTDPALAQQLLSADGSHAAFQTSDDGTITWLRLTTTPTTPDGARAALERAFTQAVYHSLAFPAEPVGTGAVWTVHQQVSGDIQPSEVNQVTTARLVGRSGDVLTIALDITQTPKSPAWRLPNNAGSLDIVDYSVHGTGTITVDLGLPMPIAGALDIGGHLTYREPRSDVLLRQDIGTRLQWDS

>CORE_REP|Org1_Gene1864#

MRVPIPPSRRHGAPDLPPATEDNPAAAAQGTPIYSLSTSVRQRLRGFLGSKRRLPRKDSSQMTVLDSILDGVRADVAAREALLDFQSIKAAAAKAPAPLDARAALLEDGIGVIAEVKRASPSKGALADIPDPASLAKAYEDGGARVISVLTEGRRFGGSLDDLDAVRATVNIPILRKDFVVGPYQIHEARAHGADVILLIVAALEQDVLASLIDRTESLGMTALVEVHTEEEADRALEAGASVIGVNARNLKTLEVDRDVFARIAPGLPTEVIRVAESGIRGTADLLAYAGAGADAVLVGEGLVTSGDPRAAVSELVTAGTHPSCPKPARRGR

>CORE_REP|Org49_Gene4483#

MPIDETLAGYSNLAFKSAFVVYLLVLAMLIVQYASARKKLTAERELVTVGGSGSGSGSGSGDVLAANVPGKLAEKPAPTLAERFGNMAFAVLFVAIGLHLASIVLRGFAVHRFPLGNMYEFITMACAAAMVTGLVFMSDRRFRAMWVFLIVPVLILMYLAGNVLYAEAAPVVPALKSFWLPIHVTIVSIGSGIFLLSGVASLLFLFRMRQPDGQESDNLLGTLARRLPDARTLDRLAYKTTIVAFPLFGTGVILGAIWAEAAWGRFWGWDPKETVSFITWVVYAAYLHARATSGWRDTKAAWINIAGFTAMLFNLFIINIVVSGLHSYAGLN

>CORE_REP|Org170_Gene2897#

MSVSARRGSRSRRSRPVGCLVLLALAVLVVIVVVLAWYLLAGRLKEPEPGPKPPEERPTSQPASCPDVQMIAVPGTWESASNDDPYNPTANPASLMLNVTGPLREQFPAERVDIYTVPYVAQFSNPIAFPPDGQQSYNNSRSEGTRRMVDMLTDRHAECPLTTYVFAGFSQGAVIAGDIAAQVGAGNGPIPQDLLLGVTLIADGRRTGESGPPNAIPIGPVPPGVGAEVALAGLNVPGITMTGPRPGGFGAVADRTYTICAPTDLICDAPRDALRPTNIVGSLTTLIGAIGNPVHALYNGFVVDPNGTTATRWTANWASGLIEAAPRPPHS

>CORE_REP|Org203_Gene4892#

MEVPESGRGAGRGRVVRRGRVRENERVSTPNDLDHSPDLRVLPQVEWRERARAHRERIDELVGPYLRRRAAGAAHPVIDFLFTYYGHKPAQLRRWHPGFGVGLAEAAEYAGARGYHQLDMGSGMVATPDPAFLAKRRDTVEFVARLLRATAGRPAQLSCFGLHEWAMVYRSDELRHGAVPLRLGRAGTDAVVESMSLRCTHFDAYRFFTADAVGRNNQVLTRDDQPLREQPGCLHANMDLYKWGFKLVPLIDSDLLADCFELACAARELDMRASPYDLTEYGYEPVTIETAAGRSEYARAQSALAERAAPLRARLLRACERLLEAAAAPTH

>CORE_REP|Org2_Gene5546#

MTVLYHRDVLYRAFLRLVDKLPLPSLRVQRILAIAVILTQAGISVTGAVVRVTASGLGCPTWPQCFPGSFTPVGVSEVPVLHQAVEFGNRLLTFVVSLCAALIVLAVVRARRRRDVLVYAWLMPGGTLLQGIIGGITVRTGLLWWTVAIHLLASMLMVWLSVVLYAKICEPDDGIATVQAPAPLRWLTGLSAVAMSGVLIAGTLVTGAGPHAGDKSIERQVERLQVEIVTLVHLHSQLLVGYLALLIGLAFGLFAVGITPAVRKRLFVVLAIVCAQALIGVVQYFTDVPAVLVVFHVGGAAACVAATAALWAALHTREPVPAAVTVEQSV

>CORE_REP|Org130_Gene3075#

MNEKLSDILSKMLDLHTTADLLSYDFVQQAVLAAALLGLLAGVIGPLIVNRQMSFAVHGTSELSLTGAAAALLVGIGVGAGAIAGSVIAAVMFGLLGSKARERDSVIAVVMSFGLGLSVLFLWLGPSRAGSKFSLLTGQVVSVGGTGLTSLALCTVGVLAVLAFIYRPLLFASTDPEVAVARGVPVRALSVVFAVLLGVTAAFGVQIVGALLVLSLLITPAAAAAQLTASPLRATLLSVLFAEIAAVGGILLSLAPGVPVSTFITTISFVIYLACRFIGRPVVNARRRTALRTPAPAATAGPDPAPHIDASNDPDSRRNRAEEQLPVRG

>CORE_REP|Org148_Gene4438#

MSHDRSGSADSGDRPAGALPEGAGALPDEGGVSRETAVLSDGAPLSVSLSETDLRVIETLLAPLRAWTSPRFYGLENIPAEGPVLLVGNHNLLGGIDAPLLLPEVLRRRGRLIRGLAENVLIAVPGVRHLLHHYGSVRGTRQNCLALLERGEAVMVFPGGGREAVRRKNEKYHLKWEGRTGFARMAIEAGAPIVPIAMIGVDDAYDIVVDGDHPVLRPLRWVVEALGINRELTPPLVRGIGPTPLPRPERFYFAAGAPIDPAPWRDAPDLGAAAVELRAVVRKGLEEELRFLFAERDRDAGRTLAGRVRGDLSAFASGAIDRLRRFRLS

>CORE_REP|Org14_Gene5873#

MRIPFDPRRSRRRTNPDGTMSLVEHLQELRSRLLKSLLAVALTTILGFLWYSHSFLGIESLGDLLRGPYCSLPPEHRAQLTTDGTCRLLATAPFEQFMLRFKVAFTAGVVMACPIWLYQLWAFVTPGLYAKERKYAISFVASGVVLFVTGAVLAYWVVAHALSFLMGIGSNVQITALSGSQYFGFIIKLLIIFGVSFETPLLIIGLNMVGVLTYERLKKWRRGMIFGLFVFAAIVTPQDPFSMLALAAALTVLFEVAVQIARLNDRRRARRGDNWGALSDDEASPLAGPDDLDGVSPVEPARPVTATGPVSAEPAPKTPRPVSDYSDTL

>CORE_REP|Org1_Gene5346#

MLDSMIEVRGLTKHYGRTAAVEDLTFTVKPGQVTGFLGPNGAGKSTTMRMILGLDTPTAGTALIDGKPYHQLKQPLRTVGALLDAKWVHPNRSARAHLEWLAASNGIARSRVEEVLRLVGLSEVAGKNAGGYSLGMSQRLGLAGALLGDPKVLLFDEPVNGLDPEGILWIRRFMQRLASEGRTVLVSSHLLSEMAQTAEHLIVIGRGKLIADTPTKEFIERASEQTVRVRSPQLDQLRSLLTSNGMTVREDGTGAEGPALLVAGVTSDAVGKLAGANDITLFELSPQRASLEEAFMRMTGGAVQYHGEGAEAVGVPGPGGPYTAMGGAL

>CORE_REP|Org1_Gene3324#

MNHMSDPTTVKVAVIGSGNIGTDLMIKVIRHSRVLEMGAMVGIDPDSDGLARARRLGVPTTSDGVEGLLALPGFADIEVIFDATSAKAHAANAALLQPLGKRLIDLTPAALGPFVVPAVNVDEHRDAPNVNMVTCGGQATIPIVAAVSRVAPVAYAEIVASIASKSAGPGTRANIDEFTETTAHAVETVGGARRGKAIIILNPAEPPLIMRDTVLCLATAPDPATRSAIRESIEEMVARVAGYVPGYRLKQQIQITEIPPDQPVHTLAADGGPAPTHQVSVFLEVEGAAHYLPSYAGNLDIMTSAALRYAESIAATVTAAPADQGATR

>CORE_REP|Org4_Gene4494#

MTAAPEPPYRVATSPHAPPSFQAGELTDPRLTAALKTLELTVRRRLDGVLHGDHLGLIPGPGSEPGEARTYQPGDDVRQMDWSVTARTTHPHVRQMIADRELETWMVVDLSASLDFGTALCQKRDLAIAAAAAITHLTSGGGNRIGAVVATGERLVRVPARSGRVHAQSLLRSIATTPHARDGVRGDLRGGIESLRRPQRKRGLAVIISDFLGEIDWQRSLRAISARHDLLAVEIIDPRDLALPDIGDVVLHDPETGRTREFSVTPTLRADFAAAAQRHREQVEQALRSCGAPVLTLHTDRDWIADVVRFVSTRRHSLGAPSGRVPRQ

>CORE_REP|Org25_Gene4331#

MTLSDSSPANGSAPTAATVRHFLRDDDLTPSEQAEVLALAAELKGAPFARRPLEGPRGVGVIFEKNSTRTRFSFELGIAQLGGHAVVVDGRDTQLGREETLGDTGRVLSRYVDAIVWRTFEQTRLDEMAATATVPVVNALSDEFHPCQVLADLLTLTEQLGPLSGRKLAYFGDGANNMAHSLLLGGVTAGLHVTIAAPEGFAPLPWVVEAARARAAETGASVTLTDDPRIAAEGAHALVTDTWTSMGQENDGLDRVGPFRRFQINAGLLAKAQQDAVVLHCLPAHRGEEITDEVLDGPRSVVWDEAENRLHAQKALLVWLLDRQNGRR

>CORE_REP|Org176_Gene596#

MRSARGILVAVLVATALASGCTSDSSAPAPTKSPVYTDPPLPAKAVPVFTDSPVPLPPPGTPRCGDPTASLRPSGAGAAARGPTIDAIRARGRLLVGLDTGSNLFSYRDPVSGAIVGFDADIAREVARDLLGSPDLIEFRSLGSAEREAALQNRTVDLVAKTMTINCERREKVAFSTVYLHANQRVLAVKNSGIRSLADLAGRRVCIVSGTTSLEHIRRDQPAATILTVPSWADCLVVLQQRQVDAVSTDDAVLAGLAAQDPYTELVGGSISEEPYGIGIPKGNDDLVRFVNGTLERIRNDGTWVGLYQRYLPSLGPVPAPPAPTYQD

>CORE_REP|Org102_Gene1947#

MSERSDRPEDTTERPRDTADHAGPADTAGSSAAVAPSAGTGEFAAADGAEVASSSGVGEHVVAAASSQAADITAERIAAAARVEQAAAQGQRSSESTVRIGLVLPDVMGTYGDGGNAVVLRQRLRMRGYDAEIVEISLSEPVPDSLDIYTLGGAEDSAQRLATRHLQRYPGLQTAAGRGVPVLAICAAIQVLGHWYETSSGERVDGVGLIDVTTSPQAERAIGEVVTNPILAGLSQPLTGFENHRGGTKLGGAATGLARVTRGVGNGVGDGLEGVVQGSVIGTYMHGPALARNPELADYLLAKALGVDSLPPLDLPEVEQLRRERLRA

>CORE_REP|Org81_Gene4684#

MAMTAEVKDELSRLTVSQVSSRKAELSALLRFAGGLHIVGGRVIVEAEVDMGSIARRLRREIFELYGYGSDVHVLGAGGLRKTSRYVVRVSKEGEALARQTGLLDVRGRPVRGLPAQVVGGSISDAEAAWRGAFLAHGSLTEPGRSSALEVSCPGPEAALALVGAARRMGISAKAREVRGTDRVVVRDGEAIGALLTRMGAQDTRLTWEERRMRREVRATANRLANFDDANLRRSARAAVAAAARVERALEILGDDVPDHLAAAGKLRVLHRQASLEELGQLADPPMTKDAVAGRIRRLLSMADRRAKELGVPDTESAVTAELLEDA

>CORE_REP|Org8_Gene4316#

MAGNSQRRGAIRKGGTKKGAVVGSGGKRRRGLEGRGATPPAEARTKHPAAKRAAAAAKAAAAGRGGPRGGGSGRPAGRKNDDGPEMVLGRNPVVECLRAGVPAAALYVAVGTENDERLTESVKLAADAGISILEVPRTDLDRLSANGMHQGLALQVPPYRYSHPDDLLDQVRNSAEPALLVALDNISDPRNLGAVIRSVAAFGGQGVLIPQRRSASVTAVAWRTSAGAAARLPVARATNLTRTLKDWAAQGIQVVGLDAGGDTTLDDFDGREPTVVVVGSEGKGLSRLVRENCDAILGIPMAGPVESLNASVAAGVVLAEIARQRRL

>CORE_REP|Org215_Gene5280#

MDNSPGTPSRRAALGRRSATEESRHPLIGRSRGEAGEPRVGLRAVIREAVETLADAGVHSPHTDAELLAAHVLGVDRMRLMMVPLLTPEQLADFRALVARRAERVPLQHLTGTAAMGEIDLAVGPGVFIPRPETELLFAWALAQLEAVGHEHRPVVVDLCTGSGALALAIAHARPDAQVHAVELDPAALDWARRNALHRADQGDTPIDLHAGDVTAPDLLSHLNGTVDVVVANPPYIPESARLDPEVADHDPRRALFGGPDGLSVIRPMIGTIARLLRVDGVTAVEHDDTNGSDTAALFHSHGGFDAIVEHPDLAGKPRFVAARRTG

>CORE_REP|Org5_Gene4708#

MCVVSACSAASFPPDERLDTVAMDLDRMLQMIKDRQWALADIDWDAPGAELIEPDLHAKLKPFLSDLMWIENVGARGFAAMAKKAPTPTLKSIYEHFHAEEQKHANAELALMRRWGMLDGDEIPSPNTNVQLVINWLDRYSDGLSLSFLGTVIPMLEVALDGALIKFITDEVKDPVAQEVFNRINADESRHLAVDFEVMDMLGHADLRKRMIDFVGGWVNPSLLIGFLSYVPLLNKMRDNIVDMGVDEERLYGAMKRYRSAGDRSEYINRLPMFRVVAGHGARVMDRGNVSYHLFADTLVKATALIPFSLVHRTPTWSQELTYEPTA

>CORE_REP|Org121_Gene3069#

MTASWIDNQKNLMLFSGRAHPELAEQVAKELDVHVTPQTARDFANGEIFVRFEESVRGSDAFVLQSFPAPLNQWLMEQLIMIDALKRGSAKRITAVLPFYPYARQDKKHRGREPISARLVADLLKTAGADRIITVDLHTDQIQGFFDGPVDHMHAQLQLAEYVRTNYSLDNITVVSPDSGRVRVAEKWADSLGGSPLAFIHKTRDPLVPNQVKSNRVVGEVEGRTCILIDDMIDTGGTIAGAVKVLKDAGAGDVVIAATHGVLSNPAAERLAACGAKEVVVTNTLPITEEKKFPQLTVLSIAPLLARTIREVFENGSVTGLFNGNA

>CORE_REP|Org82_Gene6136#

MPNRYRPTSSDRYRTGGTGRHPGPCSTTEDSALSQHRVGPSSITVQSLLGEGGEAGRPKRHRAEPSATERVKAAATAAVAAGALIGAASQAAPALAYASPLLPGSHDSDEDEQAAPTVVKGSSILPVAEAKAAAEPVAEVAPEPAAVAGQIAAPVAAPFGIPNLPPEIAGPLAQAEEVLKGVQQQVAPAPQASAVRPVAGAVSSGFGSRWGAMHYGIDFADALGAPIHSVSNGTVIEAGPASGFGLWVRVLQDDGTTAVYGHVNEMFVHAGQRVNAGDVIATVGNRGQSTGPHLHLEIWDQAGTKIDPMPYLAAKGVPLGWGPSAH

>CORE_REP|Org174_Gene27#

MIGFLLRRAANYVVLLLLASFLTFAVAGLTFRPLDSLEQRNPRPPQAVIDAKAEQLHLDEPIPQRYLTWVSGAVRGDFGTTLAGQPVSEELGRRIGVSLRLLVIGSVLGTVLGVLIGAAGAIRQYRFSDYFTTIVSLVLLSTPIFLLATLLKYGALEINSLTGQRIFLYTGETSAHRIEGLWPQLLDRLQHLVLPTLALALGGMAGYSRYQRNAMLDVLQSDFIRTARAKGLTRGRALYKHGLRTALIPMATLFAYSLGGLITGATFTEKIFGWHGVGEWLVDAVNAQDIYVVVTVTVFTGLVVLVSGLLSDIVYAILDPRVRVG

>CORE_REP|Org138_Gene6180#

MTDRIVAPTGEDPARKVKKLYRSDVRRARRLLRGQFRVTPVFHTEVQGPHGPVPVTLKLEYLQHGGTFKVRGSLNALLGARAGADSVVLASGGNAGIAAALASAVRGLSCTVVVPESAPHTKVAAMWSHGAEVLWHGTTYAEAYRFATELAVERGALQLHAYDQPAIVAGAGVVGLEIEDQVRGRPPVLVAVGGGGLVSGIAVALGPRGRVIGVEPHGAPTLHAALAAGRPVEVEVSSVASDSLGASRIGAIAMEVAQRYGVESLLVSDDAIVMAREYLWREFRIVVEPAGATALAAIQSGVYVPKPYERPVIVLCGANTDLATL

>CORE_REP|Org108_Gene1873#

MEQRTVGRSGLRVSRIGLATHTWGTRTDADQAAVQLMAFVEAGGTLVDTSPVYAGGAAQRILADLLGDLVSRDDLVLSGCAGLHPRPVPPAADGTPPMPQVPGIGVDTSRRTLLRQLDRTLLELGTDHLDIWHIAAWDPRTPLEEVAATVELALRSGRVRYAGVRGFTAWQLASLAAMAPITVTQTPYSLLARTAEDDTVPAAAHHGVGMIATAPLAGGILTGKYRDGVPADSRGADEATAAEIRGRLDERATRVVDALVTAADGLATSPLAVALAWIRDRPGVASMFVGARDIGQLTGVLAAETLELPRAIAAALDDVSARTD

>CORE_REP|Org82_Gene6175#

MRAAQVSKLEGPEAVQIVDIPEPAAFPGGVVIDVHAAGVAFPDVLMTRGLYQMKPELPFVVGGEVAGIVREAPEDAHVRPGDRVVALTMLGNAMAETAVTPTQMVFRLPDNVSLEAGAGILFNDLTVHFCLRTRGRLAEGETVLVHGGAGGIGTSTLRMAAALGAGRVIAVVSTEAKAEVARANGATDVVLTDGWLAAVKELTGGRGVDIVLDPVGGDRFTDSIRSLASAGRLLVVGFTAGEIPTVKVNRLLLKNVEVTGAAWGEWVMTHPGYLQEQWAEVEPLLASGKIAPPEPVLYPLDKAAEAVASLDNRTATGKVVVTLR

>CORE_REP|Org18_Gene4392#

MSIAVSASIATDHLMRFPGRFADVLLADQLDHVSLSFLVDDLVIRRGGVGGNIAYAMGLLGRNPLLLGAVGADFSEYRQWLEAHGVDCSAVRISDSAHTARFVCTTDEDMAQIASFYPGAMSEARDISIAGLVEENRTLDLVLVGANDPEAMLRHTAECRELDIPFAADPSQQLARLDGDQTVQLIDGAAYLFTNEYEWGLLKQKSGLTEEEVASRVGIRVTTLGKNGVVVVDRDGSEVRVGVVPENAKVDPTGVGDAFRAGFLTGHTAGLSLERAAQLGSLVAVLVLETVGTQEWSLDPDDALKRLTQAYGPEAAAELEPLLR

>CORE_REP|Org112_Gene5835#

MTGRAGTVTHPTGTVASSIAVVTAASALTVTGAAAGRRAVGGAETPGDRGRPALIAVAHGSRDPRSAATMHAVVSDVAAARPDLDVRLAFLDLSTPSVEQVVDAVAADGHTHAVVVPLLLGKAFHARVDLPGLLAAAGARQRRLRLTQADVLGPDPRLIEALRDRVLESLTAGSSGEASHAARIDTRSLAVPRVDVDACAASPLTSGGHLGVAVAAVGSSSAAANARTAAVARQLAARTGWDTEICFATTEPTVTTALSRLRDRGAGQLLVAPWFLAPGLLTDRLANAASDIAHTAVIGAHPLLTQVVLDRFDTAAALPHALTA

>CORE_REP|Org158_Gene2483#

MTDRIAAVDCGTNSIRLLIADVAPAGEASATPHLTDVHREMRIVRLGQGVDATGSLHPEAIERTRAALHDYVDLMLDAGVSRVRMVATSATRDASNREDFFAMTREELGRVVPGAQAEVITGDEEARLSFAGAVGELSSADGPFVVVDLGGGSTEVVLGDSSGVQAAYSADIGCVRITERCLRGDPPTPEEVASGRFFASERLAQAFGVVPVERARTWVGVAGTMTTLAAVALDLPEYDSEKVHLTRLTLPQVRAVCDRLIGMTHDERAALGPMHPGRVDVIGGGAVITEVLADELARRAGIDALIVSEHDILDGIALSVAPRR

>CORE_REP|Org151_Gene5995#

MTDTVARYESLIATLGNTPLVGLRTLSPQWDGENHVRLWAKLEDRNPTGSIKDRPALRMIEQAERDGLLRPGCTILEPTSGNTGISLAMAAKLKGYRLVCVMPENTSVERRQLLTMFGAQIIDSPAAGGSNQAVARAKQLAAEHPDWVMLYQYGNPANALAHYETTGPEILADLPEITHFVAGLGTTGTLMGTGRFLREKVPSIEIVAAEPRYGELVYGLRNIDEGFIPELYDESVLTTRFSVGPFDAVKRTRELVSEEGIFAGISTGAILHAALGVARKAAKAGTRADIAFVVADGGWKYLSTGAYDGTLEEAEERLDGQLWA

>CORE_REP|Org60_Gene919#

MTAPTLEVHLTDDDLTAALRADARRGLTADPKWLPPKWFYDARGSELFEQITELPEYYPTRTERALLERVVGEIARAAQAQVLVELGAGSAAKTRLLLSALTAEGPLKTYVPQDVSATALRATAAEVAREFPGLAVHGVVSDFTDTLHNLPRGGRRMIAFLGGTIGNLVPAERAEFLTDVHDVLEPGEHLLLGAGLVIDPAILVPAYDDAAGVTAEFNRNVLHVLNARLAADFDPDDFRHVAVWDAENEWIEMRLEATADMRVDVADLGLVLDFARGEQLRTEISAKFRLEGLDTELSAAGFTLDKAWTDPDNRFTLVLATRG

>CORE_REP|Org170_Gene998#

MGTVLSVVPSPVTVRAPSKVNLHLGVGDLRPDGYHDLTTVFQALSLSDDLEIAPAASLTVRVTGEGAGEVPTDRTNLVWKAAVRLAHLAGRAPLVEISISKGIPVAGGMAGGSADAAATLVGLNELWDLGLSREELTAVAAELGSDVPFSLHGGTALGTGRGERLLPVLSRNTFHWVIALAKGGLSTPAVYHELDRLREIGDPPRLGAPQELMQALASGDPKQLAPLLGNDLQAAAVSLKPELRRTLRAGVSAGALAGLVSGSGPTCAFLCESEESAVQVAAELAGAGVSRSVRTATGPVPGARVVGGEGPHPQPWREGGNIG

>CORE_REP|Org114_Gene5945#

MARRARVDAELVRRGLARSREHAVELISAGRVLINGTVATKPATGVETATPLLVREEPDEVRWASRGAHKLLGALAAFEPQGVTVAGKRCLDAGASTGGFTDVLLSKGAAAVVAADVGYGQLVWRLRSDDRVEVHDRTNVRALTPELIGGTVELVVADLSFISLGLVLPALALCCAPGADLLPMVKPQFEVGKERVGSGGVVRDPALRAEAVRAVAAAAARLGLRTHGVVASPLPGPSGNVEYFLWLRKELSGADHSTGSITGAAADSSASAHPGVQSVPEDGAGTGLPAAPGAAAVGAAAYDAVEEERVAALIQRAVEEGPQ

>CORE_REP|Org105_Gene1446#

MRYAAPPLECAQRHARSACGDLGGEHVIDGGERVTGLKTGLEAVLARAHELPSPPARGAVTLRARGVSVDRRGGGAKARRVLAEVDFEVAAGEVVALVGPNGAGKSTLLAALAGELDPTEGSVELDGRPLTQWTPLDMARRRAVLPQSHTVGFPFSAGAVVAMGRAPWQRTALRERDQEIIAASMAATDVTHLAEQAFPTLSGGERARVALARVLAQDTATLLLDEPTAALDLGHQETVLRLADERAAAGAAVVIVLHDLGVAAAYADRVAVLDAGRIAADGPPRDVLTTELLTRVYQYPVEVLDHPVTGAQLVLPVRGGGGE

>CORE_REP|Org114_Gene686#

MRAIQVSEHGGPEVLRYTEVPDPVIGPKQLLVDTEAIGINFIDTYIRTGRYPQNVPYVPGAEGTGVVAAVGAEVTEFQAGDRVAWAAAPGSYAERVAVDEAVAIPVPEGIDVPVAASALLQGMTAHYLVESIYKPEPGEAVLVHAGAGGVGLIITQLLAKRGVRVITTVSSDEKEKLSREAGAAQVLRYGDELASRVRELTGGVGVAAVYDGVGASTFEASLASLRVRGMLALFGAASGPVPPFDLQRLNALGSLFVTRPTLAHYTRDRAELLWRARDVMNAIADGTLRIRVGATYPLAEAERAHRDLEGRKTTGSIVLLPR

>CORE_REP|Org113_Gene7331#

MNTRMSVYRSASNSSTHGSEEGVPGRPTGGLLLVHAHPDDESVTTGGTIAYYRRRGVPVTVVTCTLGEEGRVIGERYAQLVSSTADQLGGYRIAELTRALAALDAGEPWFLGGPGRWRDTGVVGTSTGPGLPDPLHSRAFASAGDEAVHELVRVLLAIRPQVVIGYDPHGGYGHPDHIRAHQITMAAVDATAGLGWATPKLYWAVTDATVLHRHLRSLSHRSAELPPGWRLPVTGELASVPSSSVTTTIDVSEVMPAKLAALRSHSTQISVSPTGREFALSNKIAQPILPEEHFILVRGRLDAGCPARHERDLLAGLSANTK

>CORE_REP|Org105_Gene2106#

MAGDLRVRRETGGEAVIADDPGRTATSEVREPLLDFGNEFRHTDVVKTVGLPNTDERAGHRAGTASSAAPATPGEGHTRAAIVQLLLEEGPITATAISNRLGLTPAGVRRHLDALIDSGQARASRSAPWQQKGRGRPAKQYQLTAAGRGRLGHAYDDLAGAAIRQLGEIGGEKAITEFARKRARTIVAGIDPVREHTQADTEAKAEEIAEAFTDAGFAATTRKVGTGVQICQHHCPVSHVAEEFPQLCQAELEAFRELLGTHVQRLATIANGDCACTTHVPLLVLTPGQSPHDPATEPNTASPSAVAQPATEATIDSGRSAE

>CORE_REP|Org128_Gene3250#

MTPSSVLNPTRTTGPGVAAPQYSLVVSSDLDHRRAAQRLRYRVFAGEPGFDIPFSAEGLDADRFDEHCDHLLVRDEASGEFVGCYRMLPPDKVRAAGGYYTATEFDLSAMDPEGRRIVEMGRACVVPDHRNGSVLTLMWAGILHYIQLTGYDWVMGCVSVPMRDTPADPPGANVAAVRDLLLSKHAGDPARRVRPYRPVVVDGRALDELAAPARPKLPPLLRGYLRLGAEICGEPAHDPDFGVADFVALLGLETINTRYLERLQSAAGAMDARASEGTDARASEGTDARASEGTDARASEGTDARASDADAARARDTGGGAR

>CORE_REP|Org113_Gene6136#

MTADAGSDATQAVPPATSCFRTAVVPAAGLGTRFLPATKTVPKELLPVVDTPGIELVAAEAAESGAQRLVIVTSPGKDGVVAHFVEDLVLESTLAERGKFHLLEKVRKAPGLLDVSSVVQEEPLGLGHAVSQAEQVLDDDEDAIAVLLPDDLVLPCGVLDVMTRVRRKRGGSVLCAIDVPKQEVSAYGVFDVVPVPDATNPDVLRVVGMVEKPKLADAPSTFAAAGRYLLDRAIFDALRRIEPGAGGELQLTDAISLLIAEGHPVHVVVHRGSRHDLGNPGGYLRAAVDFALERDEYGPALREWLQRRLAPDWNPQLTSPQ

>CORE_REP|Org113_Gene464#

MSVELVEVVRSGFRECVHRGSVAILDPDGDPAVALGEVHLPIFPRSTNKPMQAITLLRNGFEPVDEAELAISTASHFGESDHLALVRRLLDRFGFDESRLECPPDLPMGEAPRADALAGRAPADAARKIYMNCSGKHAAMLATCAINGWPVTGYTDPAHPLQQAVIATITDITGEPESDLGIDGCGLPIVPVSLINLARSYARLATADPGTPERRVADAIRHHPRVISGTDAPDLETMTATPGLVCKIGADGVHAGALPDGRAFAYKIDDGHDRARLPLTQAILQRMGVEWTEAHAELAGPPVLGGGVRVGIIRAIPGVV

>CORE_REP|Org112_Gene2009#

MSDKFEKPESAAATAAAAAAGTEPEPIKAIPLRRPGRWIAAAIILALLGLFLYGAATNPAYHWDTYANYLFDKRILEGALVTLELTVLAMVLGVVLGVVLAIMRLSPNPVLRSVSWVYLWIFRGTPVYVQLVFWGLFPGLYQTITIGVPFGPSFADFNVLDWRAPFLFAVIGLGLNEAAYMAEIVRAGVNSVGEGQREASVALGMSWSQTMRRTVLPQAMRVIIPPTGNELISMLKTTSLVTAIPLTTDLYGRARDIYGVNFQPVPLLLVAATWYLVVTSVLMVGQFYLERYYSRGSSRQLTGKQLRAMASQQHVVEEGK

>CORE_REP|Org101_Gene553#

MTNDTGGSNLWGGFVHLAISEYDVANDRTGQDIARAGSRAVERSSDVEQRQISNEARLIRGVFRAAGLAAGTVVRGGQWAVGTTYEVTKEITQAALDGESSADIAERTGNALRSIARSALGVTEGSVREIVSYVPTPNGPGNHSGAQQQALAVGSYLRSASTDELRRRGDALLARSADVYFTEDVHPAYDRILDELAPDEARILRFMAQNGPQPSVDVRTNRPLGIGSELVQGDLTSVPEQAGVRYPDRSRPYLINLNRLGLTLISEDPVVLSRYMVLEVQPVVEAALKKAGRAPKIVRKSLRLTEFGEDFCRTCFTIAN

>CORE_REP|Org102_Gene366#

MADIAVTVRLRAGSDRAVCPVWLRFSVPLRGYGTRVTMTAVEPPEHVRATFGLREVMPVPLGTWDGGWRCADVVLSPVTDHARAAWSAKVREGLRVDGLRLARPVRATDGRYVVSGWRADTFLDGVPEPRHDEVVSVSLRLHQATAQLERPRFLAQPPIAPWVDVDVFVAADRAAWEAVPLRSLKIGGMLPATSPDGERSLELIGQLATLRKPVTAAPQLVHGDLFGTVLFSGSFAPGLTDITPYWRPPSWAAAVIVVDALSWGGADDGLLERWSSLPEWPQMLVRAVMFRLAVHALHPRSTPEAFPGLNRTADMIRLLL

>CORE_REP|Org215_Gene3040#

MAEVLVLVEHAEGAPKKVTTELLTAARSLGTPAAVVVAAPGTADKLGDALAAAGAEKIYVAESDDAEGFLVTPKVDVLAGLSESASPAAILVAATAEGKEVSGRLAARIGSGLLVDVIAVNGDGSAVHSIFGGAFTVDAKATGDVPVISVRPGAIEAAPQNGAGEKVAVEVPAQEEGVVKVTAREPIVGGDRPELTEAAIVVSGGRGVGSADNFGKVVEPLADALGAAVGASRAAVDSGYYPGQFQVGQTGKTVSPQLYIALGISGAIQHRAGMQTSKTIVAVNKDEEAPIFEISDYGIVGDLFNVAPQLTEAVKAHKG

>CORE_REP|Org40_Gene2862#

MDIVELRTPTAIVRHGRGRLIVLRPGGDGDKVDGERVLDVPVTDLLKVRLNRRADDAVVIEIYLRYPTPVLTGVPADRTPLPVLIAEHDVPAAVEIVDRINDQILDFQRIDVAAPDLTPPAPEWNSGGPVRADVERAVRRMAPRKEAKSAINALHRLASADEYVLETALAVAHPPAGHGQGLLAATTQRLLFISIGDGERYAHELAVAAVMWARSTDGTASAPGEIDTLRTDTGIDVHDGNQTLHFTGLDRADTQRVTCAVNFAIRLESADGVAGPADPGTAQLYAEWELLVERHSLGMVDDTQFQRYGRGILRSLPGV

>CORE_REP|Org210_Gene5990#

MWPREPVGSAFVGKSKRNSPKPDSNRAQRLAERRAAQQQAASAVTRPFAGLAAECDLVALREFVPSATATLKLAAGVSAERPVTLATVLPGAVAALVRAGDEPTGFVGAQVQFQSENPAADLAAAILWTQAAEPGASLTAASEAAQDAVPPLTEVIDPKAPLDLTVHQNFQWWVPEGVTPDPQVAATIDQADQAIMPSDRLALGAESVGAAWWVDAGEKAHLRWVRPEDEDDLMLALARVHAAGGLHLGEGSRFAGSFRTHGLLVPVFDLDRERHPSEWLTPANEFGARLAEALASDAPMSSDERRSRDGLRSRQVTLR

>CORE_REP|Org5_Gene4083#

MACAGRDRPSIPGALPTSPPAPDRPDNRHRLPIPGTAARIRTTLVILEHMCSPSATLTVWAGSWLAGRSAPDDVLDALHAWSAHHSVAAGDPVTGGRTGLPWPTAEPLEAGSGIMMLLKVIREAMAAPGAQMRLVLPVPGDARGLPTGTEFATAAMDAEEGVIAGVPGGGGVALVPQWIGEDALQWTVFDAPVPVPVPDMALGEAEYAMREAVREAAEALMQLHTTAVGGDDDPRELIEDELADYSRHIYPDSAPLRARRILDSADHVAAILTVAQQTAPSSPSSASAVQAQETLLRPLWNAIRAARLAAVNAATGPR

>CORE_REP|Org4_Gene7796#

MTVLTNDALLAVADRAGVQTLPLALAVGPQQDSFEEWQRAQESAVATLIDDGLIDAHGEVDPELADVLFTLAHPEQELAARIYTGNGSRRVCVVRRAHMHAVAVRSGDDFDVRPVWTDGSAADLVRPVLAAMDACPPAEIPNFSVPAAELAERLDGAATSSELTDALYALGATDRDATTLGLTFGATHAYAEIVACAHEDGVTTRAPGAVAVYDTARGRIVAAPMVSPDQQVWSTVTPGTDHRVAQAVATLIEGLPGGGGCLSSSRGATSVVSYCNEIARMGQRSTKMTKGQRNVSHLSRWLGQGCWCRGRWRRQGRR

>CORE_REP|Org82_Gene3772#

MRIGGRRRTRPAPGRTPWPALLLAVPAGLLGVALAWALILPGPLPGEAVVRVAADGVGATALGLAALPRVSPRLRTPWRLLAVLAGVWAAAEFAVLVFEAAEVVGVPVTELGAAEFGTYLVDVSGGQVGIAILVGVATVACGSALAFRRPESASADLVLVFAAVALALRPITGHMSQQAFGSVLAAVHALAAGAWFGLLIALALVVRTRGEWAVVLPKYSAVALPLVAVVAVTGLLNGLIRVGGIAPFVSTGYGRILLAKTVVLLVLLALGWWWRRSWVPRAADHRMDAKDSLRRAIAEVVVMALAFGLAATLAVTA

>CORE_REP|Org24_Gene597#

MATARRGRRRSGRSHRVAARSARRARLTGVTLPSVTPVTGRLVVTGARGQLGRALLDLAPDARGYTHADLDITDLDAVRAALRCGDVVINCAAYTAVDRAETDIDAACAVNARGPMALAVACGEVGARLIHVSTDYVFPGTGSRPYETADPTGPTSVYGKSKLAGERAVADLLPETGHIVRTAWVYTGTGSDFVATMRRLERERETVDVVDDQIGSPTYAPDLAAALVELAEQPDAPRILHAANAGQASWFDLARAVFAGVGADPDRVRPCSTSAFPRPAPRPAYSVLSTASWTAAGLSPLRPWQDALNDALAAASD

>CORE_REP|Org172_Gene3028#

MVRRYTNALKTALLLGVLTALILTIGYALGGSTGLIVATVLSLVMNGAAYFYSDTIALHAMAARPVNEAQAPELHAMVRELATSAGQPMPRLYVSPIAQPNAFATGRSPRHAAVCVTDGILRLLTPRELRAVLGHELSHVYNRDILTSSVAAALAGILTSLANLALFLPIGSSTEDDDGPHPVAALLMLILAPVAAGLIQLAISRSREYQADVDGADLSGDPLALASALQKIDQWTRRLPLPADAPHAAYAHLMIAHPLSDDGVAALFSTHPPTAERIRRLRQLASQVAAGPSGPARPAPRSSWAGALAPTMRSLAR

>CORE_REP|Org158_Gene6059#

MASGTDGTLLCRYNRPRRDHLVPSNEQRRAAAKRKLERQLANRAARARKRKQLTIAASALGVVVAVAAGVGIYYLTRGDDDTATTATTSETPEASLASAPPSATPKPELVNCTYRDSGEAARPVDKPRADGIRTTGDDATLSVSMQTSQGPIGLTLNNAESPCTTNSFASLASQKYFDGTSCHRLSTSGLKILQCGDPTGTGMGGPGYAFDNEYPTDQYAPGDPTAQSVPVKYQRGVIAMANSGPSPDGTGTNGSQFFLVFGDSQLPPQYTIFGTIDETGLETLDKIAAAGDDGSMEPSPGGGKPNLQVTLESVQID

>CORE_REP|Org100_Gene4268#

MVAAETVRTVRSGADRQVGAQVADGGGIDHDALLDWYAAAARDLPWRRPGVTAWQILMSEIMLQQTPVARVQPIWLEWVRRWPVPSAMAASSQAEVLRAWGKLGYPRRALRLHECAGVLAAEHGDEVPADVDVLLGLPGIGAYTARAVACFAYGMRVPVVDTNVRRVVARAVHGRAEAGNPSARDLAETEALLPAQVEPAARFSAALMELGATVCTARNPDCGRCPLPHCAWVSAGRPASEVVRRTQKYEGTDRQARGRLLDVLRAASGPVERVRLDLAWTRDPGQRDRALDSLLVDGLIEQTADGLFALAGEGER

>CORE_REP|Org4_Gene1651#

MTEMIVNEASNVTQRALGISDFDTLAALESESIHVFREVAGEFERPVILFSGGKDSTVLLHLALKAFWPAPLPFALLHVDTGHNLPEVLEFRDRIVERYGLRLHVAKVEDYLADGRLTERPDGIRNPLQTVPLLDAISEHRFDAVFGGGRRDEERSRAKERIFSLRNAFGQWDPKRQRPELWNLYNGRHAPGEHVRVFPLSNWTELDIWRYIAREDIDLASIYYAHQRPVYQRDGMWMTPGVWGGPREGEALQTLSVRYRTVGDGSSTGAVLSDAADNEAILAEVAASRLTERGATRGDDRVSEAAMEDRKREGYF

>CORE_REP|Org113_Gene1830#

MELRQLRYFVTVVEEAGFTRAAQRLHLAQPGLSAQIRQLERELGQPLLDRSGRTVTLTAAGAAVLPHARAALAAAQQISHTADEFTGLLRGQVRIGLISGAATEEFDVATVLSAFHHDHPQIGISLTEDTTDRMLAAVARGALDIALVGLTGAPLDEGFGVDIVFETRLQAAVARENREFGDRIALADLRERSLICLPRGTGIRGVLEHACAAAGFEPRVDFEAAAPPLLIQLAAGGLGIAVVPALEPDQAAAAGVRMADITDPDLIGHLALVWRADRPLAPAAKVVLGQLRIALGRWKARHPISPGDGRGRNGSA

>CORE_REP|Org20_Gene4772#

MTGAQKRSGSDRDETIETTPPTGTASGASEPGQTPAEVDVERDEDVEAEQDAEVAAEAVAEAGSVTGYDDLPDLFDRLAAVEPGTAEHAALRDELISRCVPLADHIARKFSGRGEPFDDLTQVARVGLVHAVDRFDPARGSNFLSFAVPTIMGEVRRYFRDHTWAMRVPRRVKETHLRIGAAIDTLSQSLGRSPTAKEIAAELGVDPDEVTQAVIAGNAYQPSSIDAAALGRESDASLLDTLGEEEAQFERVEEYVAIRPLLAGLPERERRILTMRFFESMTQTQIAKQMGISQMHVSRILAKTLARLRELSARE

>CORE_REP|Org19_Gene6366#

MSALITPRDGRSCVVMGVVNVTSDSFSDGGRYLDPAVAVAHGVRLYEAGADIIDVGGESTRPGAVRIDPETEAQRVVPVIRGLVEAGVPTSVDTMRASVAAAAIDAGVSVVNDVSGGRADAEMVKVVAAAEIPWILMHWRANADHRHIGPADHYDDVVREVLAELSSQVDLAMAAGVHPSRLVLDPGLGFAKNAEHNWALLGALPELTAQGLPILVGASRKRFLGSLLGDESGPRPPDGREVATATISALAAQHGAWGVRVHDVRSSLDAIAVADAWRRAAESAERRAAESAERWAAESVERRAAEAGSHNQGSE

>CORE_REP|Org1_Gene3437#

MCFARRPRPVDPAVDPFTRGKVSTVVKISRQWKSLVAVGFAALTLSGLAGAPAQAAPETDALYNSAQRHFTDGNDGAGRADLRALLAIDPADAEALSLQAIWSHYAGDVPATADALARLQVVDPGLAGGTHHVLHAIGAAAGTLPNPLPALVGPQTGIVVLGYGLLPDGSLRPELVNRLTAAWIQAIASPFSPVVVTGGNPQNGITEAEAMRNWLVGRGLPAARIHVENRAGSTVQNALFSTRLLRDIGASSAVVVTSPNHIRRAVADFIVAGIPVVGAMTSLEQLVSQLPPPPRHAQRGIYLDATRTFQLATSR

>CORE_REP|Org17_Gene6792#

MPPRTNPPIIRSLPVTTPDTTSTTGTARVKRGMAEMLKGGVIMDVVTADQAKIAEDAGAVAVMALERVPADIRAQGGVARMSDPDLIDGIVNAVSIPVMAKARIGHFVEAQILQSLGVDYIDESEVLTPADYANHIDKWQFTVPFVCGATNLGEALRRITEGAAMIRSKGEAGTGDVSNATTHMRKIRAEIRHLQSLPEDELFVAAKELQAPYELVREIAETGKLPVVLFTAGGIATPADAAMMMQLGAEGVFVGSGIFKSGNPAERAAAIVKATTFYDDPDVLAKVSRGLGEAMVGINVEEIPEPHRLAERGW

>CORE_REP|Org151_Gene1532#

MDWVRPRPLGSAAVSDSDSASAGLAVVTVTYSPGEHLEHFITTLADATTEKPQVILADNGSTDGVPELVAEANSHVRLLRTGGNIGYGGAINRAVAEIDPAIEFIVIANPDIRWGTDAIDQLLAAAQRWPRAGAVGPLVLEPDGSVYPSARRVPGLLDGAGHAILGTVWKTNPWTRRYRQENEEISERAVGWLSGSCLLVRRAAFDSIDGFDSRYFMYMEDVDFGDRMGKAGWHNVFVPSAEVTHAKGHAAGRHPEKMLPAHHASAYRFQADRHPHWWQLPLRLALRAGLAVRSRIAVRSALRQQAREAGHPV

>CORE_REP|Org65_Gene817#

MNHLPQAPTANRIDSKDQHMSQQSSTASVSVIGLGPMGQAMVRAFLKAGTEVTVWNRSSAKVDAMVELGAKRANTVAEALDANEVTVISLTHYDAMYDVLGQATDHLAGKVIANLSSDSPEKARKGAEWVRSHGAEFISGGVMSAGDNIEHPASYIFYSGPREVFDTHAELLRPLSPPEYLGVDDGLAQVFYQALLIVFHPWMLAYDQALAVIARSGQDIDQFLPFAQRAAGAYPFFMEEYANQAKLGGWGDLSAFKMMDAGAQHIIDASEEVGVDATISHLAQGIWRKAIAATEEAGKPVSVFELFGGTKKS

>CORE_REP|Org9_Gene2292#

MHSAYARRMVDRVLVTLDGVVRDADEPLLFADDIGVLRGDGVFETVLVRDGDACAIEFHLGRLRRSAQALDLPEPELSRWREAVQTAAKEWGSEREGMMRLVLTRGRDTELGAPSSVTSGDLAAAVPVPTAYVLVVPVPERVAKARAEGVSVVTLARGISIDLAQAAPWQLLGAKTLSYATNMAALRFAHRMGADDVIFTSTENRVLEGPRSTVVIARDKELITPPAKNGVLPGVTQRALFTEAKKAGWECRYAPLFTADLLTCDSIWMLSSVTLAARVNSLDGLRMSAPDNAEEIIELVDRGVQRGGAIGDW

>CORE_REP|Org1_Gene4747#

MVMFSPPAAPLPTLCGKPVATDRALVMAIVNRTPDSFYDRGATFTDEAAMAAVDRAVAEGADLVDIGGVKAGPGSEVDAAVDIGGVKAGPGSEVDAAEETRRVVPFVAAIRAAYPDLLISVDTWRSEVARAAVAEGADLINDTWAGADPELVRVAAEHGAGIVCSHTGGAVPRTRPHRVRYADVVAEVTETVVAAAERAAAAGVRTDSILIDPTHDFGKNTYHGLELLRGLDVLVNSGWPVLMALSNKDFIGETLGVGLSERLEGTLAATAWSAAAGARVFRVHEVAHTRRVVDMIAAIQGIRPPARTLRGLV

>CORE_REP|Org84_Gene3006#

MTTETETTGVTDIGGTDIEFTKGHGTENDFVVLPDEDVRLDLTPARVAALCDRQRGLGADGVLRVARAGALLRAGVLDALPAGVSDTDWFMDYRNADGSIAEMCGNGVRVFAHYLAATGRAEGTEHVVGSRAGARPVTVHAAGPTHGEVTVAMGEVRALGASTATVAGWGYSGLGIDVGNPHLACVDPTLTAEALAKLDLTVSPGYDPDLFPHGVNVEILTPLDEQRAVDMRVYERGVGETRSCGTGTVAAAAAALSAEGFELATGSGAVTVRVPGGAVRVGLEAGSAWLRGPSVLVATGRLTADWWQTLG

>CORE_REP|Org46_Gene4697#

MTSEVLHRLSALDKAHVLADALPWLQKFRDKVVVVKYGGNAMVDEHLKQAFAADMAFLRTVGVHPVVVHGGGPQISAMLKKLGLQGEFRGGFRVTTPEVMDVVRMVLFGQVGRELVGLINSHGPFAVGISGEDAGLFTATRRTVEVDGEPTDIGLVGDVTEVNPDAVLDLIGAGRIPVVSTIAPDADGVVHNINADTAAAALAEGIGAEKLVVLTDVEGLYTNWPDRSSLTSRIDTAALAELLPRLDAGMVPKMEACLRAVSAGVPTAHVIDGRVPHAVLLELFTGEGIGTMVTPAPLIPSGSGAPDGTKQ

>CORE_REP|Org152_Gene1314#

MGEKRTPVVDALGGLLIVDKDGGWTSHDVVAKARRLLRTKKVGHAGTLDPMATGVLVLGVERATKMLGLLTLTTKAYTATIRLGQSTVTDDAEGEVTATTAAGHLTDAEIASGVAALTGDIQQVPATVSAIKVDGERAYARARAGEDVQLAARPVTVSRFDVLARREVDGADGQFVDLDVEVECSSGTYVRALARDLGARLGVGGHLTALRRTRVGPFTLEHARTLAELTAAAEAEEPLLSLDVDAAARTAFPVRAIDERQAEDLRNGRWLEPVGLSGVYAAIDPSGRAIALLQESGKRASSVMVVRPANL

>CORE_REP|Org7_Gene4566#

MGNEDQTPASESDGERDVSGLRLMAVHAHPDDESSKGAATTARYADEGHDVLIVTLTGGERGSILNPAMDTPGVLDRIDEIRREEMAAAARALGVRQQWLGFVDSGLPEGDPLPPLPEGSFALVPLEEATEALVRVVREFRPHVMTTYDELGGYPHPDHIRCHEVSMAAFEAAGDPERFPDAGAPWTPLKLYYDHGFTARRMEVFAEEYERMGEPFPLQEWLDRMRKYVPERGDVFSRVTTQIECAKYFPQRDDALRAHATQIDPNGAFFAIPLELQQRLWPTEEFELAKTRVRTALPETDLFAGIEDEQR

>CORE_REP|Org101_Gene1635#

MPRAGIGLDWSGMQVKKVVAALGASATVLSGLVFGSGSVSADPGCPSLYVVAIPGTWETGKDKKPQPGMLAGVTRNLPSSADVDYVTYAATAFPWEGEVYGNSKKEATDRARGLITDMAKSCGATKIALVGYSQGADAAGDLAAEIGTGLGPVPADRIAGVGLISDPRRSPTDVQVGAPAPGAGAGGPRVGGFGWLSDRTRTICALDDLYCATAPDDFVTRFAGFMAQASDMNPANMWRYQIEAGNIMTDLFAHGGVPALQAQLTESANEQRAKDLERFYKSQAHTLYGSYPVGGGQTATSWMHNWIAGMA

>CORE_REP|Org122_Gene986#

MARMSEPSPYVEFDRKQWRTLRKSTPLVLTEEELIGLRGLGEQIDLEEVAEVYLPLARLIHLQVAARQRLFAATATFLGEKHPDRQVPFVIGVAGSVAVGKSTTARVLQALLARWEHHPRVDLVTTDGFLYPTAELTRRGIMHRKGFPESYDRRKLLRFVTEVKSGAEEVCAPVYSHISYDIVPGKLHCVRQPDILIVEGLNVLQTGPRLMVSDLFDFSIYVDARIEDIEKWYVQRFLALRKTAFADPDAHFHHYASLTDEQATLAAQEIWNSTNRPNLVENILPTRPRATLVLRKDADHSINRLRLRKL

>CORE_REP|Org169_Gene5537#

MRESTRPRGHRGHAEQAAADSEGVTTMTSGTTQPATSLAPMTALHTGSGDPLLLLHGFLMSPHCWEDVASRLSATCEVFAPAFAGHWGGPDLSGWYIDVNLLADRVEEQLDELGWRTCHIAGNSLGGWVGFELARRGRARTLTAIAPAGGWHNPSLAQLRVGLEFLMLLPIVEIGKRLPAWIRFSAPVRRATAMLLSKNIAAAPRRGVEAAIMSATHCAAMLPLIVSGLRMAVLDDLSTVRTPVRLLMCEFDRIIPNRMYAKRFLRELPETADRILVHGVGHVPMLEAPDRIATLIAEHVYASRTRLRAV

>CORE_REP|Org150_Gene1780#

MSGSTNDSTSAAGFIGLGNMGAPMAERLLKRPGGLVVCDTRAEAVQPFVDGGAEPAKTAAEVAEHAGVISVVVLNDEQVRSVVTGPDGLLSTARPGTVIAVHSTISDRTAVELAGICAEHEVDFVDAPVSGGGAGAKKGSLAVMVGGSDAAFEKVREPFGYWAELVVHAGEVGAGTRMKLARNLLHFVSFTATAEAQRLAEAAGLNIVDLGKVVRHSDAITGGAGAIMLRDTTAPVAVDDFWHSIFTHTRDLGEKDLSLALALGERLGVELPFARMALSGLGDGLGVGPGDISRAARAATDTSEESNER

>CORE_REP|Org117_Gene770#

MTFNEGLQIDPDRASSGGGPGMGGKLALGGGAGGLILLVITLLLGGDPGSVLGQFTGAQDNGQVQPGTAGTPEHCRTGADANRYVDCRVVLTAQSLDAVWAGELPEQTGVRYSEPKLRLFSGAVATGCGNATSEVGPFYCPADQTAYFDVSFFQELVDRFGSSGGPLAQEYVVAHEVGHHIQNQLGDLGRAQQDPRGADSGAVRTELQADCYAGLWAHYADKTPAPGSSEPFLRPLTDTDIRDALSAASAVGDDRIQRAAQGRVNPEAWTHGSSDQRQKWFLTGYRTGRVDACDTYSASDLNNPPALR

>CORE_REP|Org204_Gene817#

MRETRTMPVPDGLDGMRVDAGLSRLLGLSRTAVAALAEEGSVQLDGVAAGKSDRLTAGAWLEVVFPEPKRELTIEAEPVEGMKILYADDDIVAVDKPVGVAAHTGVGWSGPTVVGGLAAAGYRISTSGAHERQGIVHRLDVGTSGVMVVAQSEHAYTVLKRAFKQRTVDKRYHALVQGHPDPSSGTIDAPIGRARGNDWKFAVTADGRPSVTHYDTVEAFQAASLLDIHLETGRTHQIRVHFSAIRHPCCGDLTYGADPRLAERLGLQRQWLHARSLGFQHPADGRYLEITSEYPADLTHALDVLRNA

>CORE_REP|Org1_Gene1776#

MTHKIGTDEGLSKTSTPGRHRGRRKVDQMSTLHKFKAYFGMVPLEDYEDDYVDDRAPRASERGGARGPRPYSERAGYGADRYGEDRYSADRFGPERFGAERFGPDRFGADRFDEDADYPEPAYKSYKSGYPVARRDDYPEDAYGEDRYEAPRRPTRIDAAPSSGRFRAGGGAPMLRGATRGALAVDPEAEERRLEERMRPEPVVARRPGIFEDGGPLSKITTLRPRDYSEARIIGERFREGNPVIMDLVELSNADAKRLVDFAAGLAFALRGSFDKVATKVFLLSPADVDVSAEERRRIAETGFYNQK

>CORE_REP|Org12_Gene3944#

MPGRSKPLSLPLPVARAVLGPMFRFAMHSRLPWEAQRYLLDAGSVLQTLPAGTRVHRMRLGGRPAERVTAGPADGPGAVLYLHGGGYTIGSPATHRSLAAHLSREIGCPVYVPDYRLAPEHPYPAALDDAEAAFSELVSTGLAPQQIAVAGDSAGGGLSLALALRLRDGHGMRPAALGLIAPWADPNELPARERDLVVNRAWSRLCAAAYLGDGDPLDPGYAPLLGSLDGLPATYVQVDEGELLHSQCVRLAAALRAAEVPVRFSVTRGLWHVSQLQASLVAPAALAARELAGFLGQSLQPAQTGSIG

>CORE_REP|Org12_Gene4391#

MSIFLNKDSKVIVQGITGGEGTKHTALMLKAGTQVVGGVNARKAGTTVSHTAKDGSAVELPVFGTVAEAIKETGADVSIAFVPPKFAKDAIIEAIDAEIPLLVVITEGIPVQDTAYAWAYNLEKGGAEGPKTRIIGPNCPGIITPGESLVGITPANITGKGPIGLVSKSGTLTYQMMYELRDFGFSTSIGIGGDPVIGTTHIDAIEAFEKDPETKLLVMIGEIGGDAEERAAAYIKENVTKPVVGYVAGFTAPEGKTMGHAGAIVSGSSGTAAAKKEALEAAGVKVGKTPSETAALAREILEKASITA

>CORE_REP|Org140_Gene5470#

MPSSKHCPGLRWNTAPDLGRQRCRWVPYGGRVRVSPSSAAGIGPSATAAAADPVQAADGAPKSKTRTRPAETRLGLVRRARRMNRKLALAFPDAHCELDFTTPLELAVATILSAQCTDVRVNLTTPALFAKYPDARAYAEANRAELEEYIRPTGFYRNKANALIGLGQALLENFDGELPHTMDELVKLPGIGRKTANVILGNAFGVPGITVDTHFGRLVRRWGWTAEEDPVKVEQAVGELIERKEWTLLSHRVIFHGRRVCHSRKPACGVCLLAKDCPSFGIGPTDPDAAAELVKGPEAEHLLELVGR

>CORE_REP|Org35_Gene2401#

MRNHPGPGSAGDSSRLRASGTVGVAMVTPFSAEGKLDVDAGVALAARLVDRGVDLLAISGTTGESPTTTESEKADLLRAVVDAVGSRATVIAGAGTYDTAHSVELARNAQRAGAHGLLVVTPYYSRPTQEGLIAHFTAVADATDLPVTLYDIPPRSIVPIASDTIRRLAEHPRIVAVKDAKGDLNAGAELIATTGLAFYSGDDTLNLPWLSIGATGFISVIGHLVPERLRELVDAYTAGDVVRAREINAGLVPLNAAMARLGGVAMSKAGLRLLGIDVGEPRLPQLMPGPDQLDLLSADLRAAGVLG

>CORE_REP|Org102_Gene6502#

MRAGHAVRTLAPGVIGSVGRGRHNQDMPDGPKPTSEHVGYSVRTVAERIGVPTATLRSWNRRYGIGPQQDRPGRHRLYTEADIAVLTRMVDLIRAGATPAGAAATARGPALSLGDKAALLTAAFALESRAVCALLDTHLRDYGVIDTWDRLCRPAFADIVARQLDGEGCVDVEHLLSWCIIATLHRAAPPPDTPPGPVVLACTSGETHSLPLEVLRAALAERGTGAHMLGPDVPTAALADTLARLPSPTTVLLWSQQESTALTSAIRVCADADATVYLGGPGWDTLILPESAIRLDSLSAAVDRLG

>CORE_REP|Org7_Gene176#

MSRSLGGTVVDASTAGRSDLDVLLGLLDLEQMDEDVFVGQHPEKVWSRTFGGQLVAQAIIAAGRTVGDRPVHAINAHFVRGGDTKKPIEYRVDRHRDGRAFANRTVTAYQDDQELFVMLAAFQDWNKGLEHGHPLPEVPDPETLPRVEESFQGLEDKLEMFIKAPHPIDMRYTNDPAWILKGTGERLNHNRVWMRTDGQLPDDPLLHVAALGYSSDTTVLDSIITTHGLSWGLDRIVAATVNHSIWFHRPFRFDEWALYATESPVASGSRGLATGRFFSRSGELLATTVQEGLIRHFPARSTTTAG

>CORE_REP|Org6_Gene5554#

MTLPYVKLLVGNLVYAVSRKPLRATPGRTPTSNVDGMSEAVIPAAVGDDEVMTADTGGEQTLPLTGERTVPGIAEENYWFRRHEVVYARLLSRCAGKTVLEAGSGEGYGADMIAGVAAAVVGLDYDASAAAHVRGRYPRVRMIRGNLAALPLPDAAVDVVVNFQVIEHLWDQSQFLRECLRVLRPGGELLISTPNRITFSPGRDTPLNPFHTRELNAAELDELLVEAGFRVESMTGVHHGATLRALDTKHGGSFIDAQIQRALAGQPWPAELTADVAAVTIDDFDLRADDIDASLDLVAVAVKPGS

>CORE_REP|Org102_Gene1292#

MITVFICSERVAPAIRAAAPRRVSGVELLPLTPNGKTPVRVLTIAGTDSGGGAGIQADSRTMALCGVHACVAVAAVTVQNTVGVSGFHEIPPQIVADQVRTVVTDIGIGAAKTGMLASTTIIEAVAGVCREVGIGGGGDIPLVVDPVAASMHGDPLLHAEALDAVRNTLFPLATVVTPNLDEVRLLTGVEVVDDRSARRAAEALHALGPRWAIVKGGHLRSSAYSTDLLFDGENCYELTAERIATGNDHGGGDTLAAALACALAHGYPVPDAFAFAKEWTRRCLEAAYDLGAGHGPVSPLWRLHEL

>CORE_REP|Org127_Gene4988#

MTDNSERICAGRTVIVTGAGRGIGRAHALAFAAAGANVVVNDLGAELDGAPSADSPAAQVVEEIVQAGGRAVVNGDDVADWAGAKRLIGQAVETFGGLDVVVNNAGIVRDRMLVNLAEDEWDAVIRVHLKGHFATMRHAIEYWRAESKAGRARDARIINTSSGAGLQGSVGQGNYAAAKAGIAALTITAAAEFGRYGVTVNAIAPSARTRMTETVFADMMARPDDGFDAMAPENVSPLVVWLGSPDSAGVTGRMFEVEGGKVALADGWRHGVAEDRGARWQPSELGPVVRELIAKATDPEPVYGA

>CORE_REP|Org113_Gene1455#

MTESDSDATVPGTRGNHVAGAGDPDAVSPAEVTAHPAVGISPASPSGEADEPRLDAQFRACHAELALGYGGGGDPDDPAQVQAMQMVLHIPKADPPLRSAVLEAAAASAVALCLDPRVGPGGEWESRYLAWKRSRIRKVARRARGAQWNAAGEVDGITIAADGAQARALVPGPVGAIDPRIRKLQIGGTDLEHDTPGAPDPELPVLWVNSALEMTVGKAAAQVGHASMLLAGALPVDQAFAWAQRGFRCAVRDADPAQWDQLCALVHGDAAPRTVAAVRDAGFTEVAPGSMTVIAVAPELQPLGL

>CORE_REP|Org30_Gene2108#

MRWSPFRPAEKGRATLAHNRIDHYPTRTPDPAPHVERHDATVWGRIEAPELAGFDTDGYTILDELISPDEVAYFAREIDRLAADPGLRGDERVIVEKSSNRVRSVFEVHRLSSAIAELAAEPRVAGLARQILGSEVYLHQSRVNYLPGFGGAGFYWHSDFETWHAEDGMPRPRAVSISIALTDNYPFNGSLMLMPGSHRTFVPCQGETPAENYRESLREQEIGVPAQSDIEVLAHKYGVAQFTGRAGSALLFDSNIMHGSANNITPFPRSNIFLVFNSVENTVEEPFAAPARRPTYIASREFTPV

>CORE_REP|Org86_Gene5258#

MHHRGQDVVQSPARRSHGQAGRGDLDGHPRGRVNRGDRGIHDVEPGGWGKVQGVSGAAGTPVADAVAARAAELSANLDALLARIEAACRASGRAPDSVRMLPVTKFFPARDVAILHDLGLREFGESREQEASAKVAELSGLDGIAWHMIGRLQRNKAKVVARWAHTVHSVDSERLATALDRGACAALAAGERAEPVRVLVQVSLDADPARGGVVPAELDALADRIAAAEGLQLAGLMAIPPLDAEPDSAFALLETLHTRILARHPGARELSAGMSGDLESAIAHGSTCVRVGTALMGARPITSG

>CORE_REP|Org81_Gene2585#

MNVGAPDRARWVGENPEYKETATVQRIGVIGGGTMGAGIAEVAARAGGSVLVLERDTEAADAAVARIEKSLGRAVKSGRLEQAAADQARARITLTTAIDDFADRELVIEAAPEIESLKTDFFTKLDGIVSPETILATNTSSIPVIRLANATANPGRVVGVHFFNPVPVLPLVEIVVTLKTDREVADRVTAYARDILGKRTIESKDQAGFIVNALLIPYLCSAIRMYETGFASAEDIDEGMVSGCAHPMGPLRLTDTVGLDVTLAVAESLYAEFGEPQYAPPVLLRRMVDAGYLGRKTGRGFYTY

>CORE_REP|Org47_Gene599#

MTDSSNSAGTEPAPGSGATAELSKPAAAGSRGPNWSWLRVTGSVGPLGIATAVLWLSIIVLLPLAALTVSAFDEGWAGFWDAVTSPVALASLRVTVFVSVIVALINVVMGTLIAWVLVRDDFPGKGIVNALIDLPFALPTIVASIVLLSLYGPESPIDIHLNATQPGLVVALAFVTLPFVVRSVQPVLIEVDKEVEQAALSLGADNWTTFRRIVLPTLTPAIISGGGLAFARAIGEYGSVVLIGGNIPRETQMASQYIQQQIEIDRPVAAAAVSVALLVIAFVSLLVLRLFAERSARKEQEAR

>CORE_REP|Org200_Gene1927#

MTASTAVETAPRPALPPRRPFQPGTRMWDETGLITFSLTAGSAFLLQTMEPTISAVVDEHSTFRTDPMGRAVRSLSSVMMWVYGGEEGVAEADRLRSMHASLNTTDASGFKHKALASFPWAWVLHTGTFAFTKNAKYFSRRPLTEAEKQEYYEESLQLMRNFSVAPKEIPANYAEFEKFFDDVVENHLQATGTARDYLRTIRSVAPPKQLPRFLWPLWKVLVDPIGRMQYFVTVGTTPEPARRKLGLTWTESDERKLRVLGWFIARLVPLLPERVRYFPIAYEARKLERDRARLRTVIQKRPI

>CORE_REP|Org66_Gene2436#

MAAAVSVHPRRHDLRRFERSAAQHHRRASARAPSGGSAVSGPLSVAPQPIPGHGLLTGRVAVITAAAGTGIGSATARRLLAEGADVVISDWHERRLGETEVELKGEFPERRVAAIACDVQSTTQVDELVRGAAAALGRIDIMVNNAGLGGETPVVDMTDEQWDRVLDITLNGTFRCTRAALNYFRAAGHGGVIVNNASVLGWRAQYGQAHYAAAKAGVMALTRCSAIEAAELGVRINAVAPSIARHAFLDKVSSSELLDRLSEREAFGRAAEPWEVAATIAMLASDYTTYLTGEVVSISSQRA

>CORE_REP|Org150_Gene5992#

MFPHRAPVPHSGTCCRSEPSARYGAIVSESVAETPAEPPAPKDASTVVLVRDGASGPEVFLQRRVKAMAFAAGMTVFPGGGVDRSDADAEIGWAGPDPTWWGRRFGVDRARAQALVCAAVRETFEECGVLLAGPTADTVVSDTVAYRSAREKLERRELSFGDFLANENLVLRADLLRPWDNWITPVVEPRRYDTYFFVAVLPEGQRADGATTEAHEVAWRTPAQALDRWRAGEDVLLPPTWTQLSSIAEFASTTEILAAERSISPIMPVFEPVDGQPMLQFPNNHRYFADMPDASRLQGSKRD

>CORE_REP|Org113_Gene136#

MRGIILAGGTGSRLHPITRGVSKQLVPVYDKPMVYYPLSTLMLAGVRDVLVITTPEDAESFRRLLGDGTQFGMSIDYVVQPEPDGLARAFVLGADHIGTDCAALVLGDNIFHGPGLGTRLRRFDGLDGGTVFAYRVSDPSAYGVIEFVGGKAVSIEEKPKLPRSSYAVPGLYFYDNDVVEIARGLRPSARGEYEITDINRTYLEQGRLRVETLARGTAWLDTGTFDSLLDAANYVRTIEERQGLKIGVPEEVAWRMGFIDDEQLSRLAEPLVRSGYGTYLMDLLTRGKNDGTTADEYRDEQDD

>CORE_REP|Org24_Gene1627#

MRLRDRFGALLLDLDGTLFRGHAPIPGASEALSSEGATQRLLYVTNNASRSGAGVARHLRELGFTATEDEVVTSAQAAAHLLAARLAPGSTVLVVGTDDLVAEVEDVGLQPVRRFNGIAPAAVVQGHSPHTAWADLAEAAYALRAGALWVAANTDATLPNERGLAPGNGSMVAALRTASDREPTVAGKPHAPLMEDALARAGTRSALVVGDRLDTDIDGALTAGLESLMVLTGVSTLADLNRRPDRLPTFVAESLDALNHPVAEHDPIAVDGGDIADRIAELLARHPGRAIPVATPPEPRTA

>CORE_REP|Org114_Gene6927#

MSSQDRMLTRIGGLLRQAESTENEHEAEAFMAAAQRLATRSSIDLAVARSHVASRERRPTPVQRVIPIGEPGKRGLRTYVQLFVAIAAANDVRCDVARTSTQVYAYGFDGDIDTCEALYASLLIQMVRASDQYIKSGAYKSGTVEKVVTEKRFGRTVRTRVRAPVAAVTARLNFQMAFAARIGRRLAEVKSEVETEAVRTDGAGDDAGNGGAAGGSAAGDGAGSAAAGTANGTALALRDKEVALTDFYRQTSEARGTWRGPEASAGYSSAARRAGDRAGRAARLGTAPELGAARGELPGGGS

>CORE_REP|Org4_Gene1605#

MLGYKGGTPMALPQGTTGSTMPRRQLGRHLRDLRNRARMTTRTAAQQLEWSEAKIWRIETGQTSLRSLDVEAMCKVYGAPSDLIGPLTALARETKARGWWTAYGDVIAEGFEVYIGLEEAATRLSTYENELVPGLLQTEDYTRALLTAARPDMPANELDRRVQLRMARQALVTRAHAPLHLDVVISESVLWRRIGGDAVTAAQLEHLRRMCDLPNVRIQVAPTDSGYHDGMDSGRFVMLEFPELRAGESPEPPVVYVESFTGPVYLDKENEIDRYRRALASIKSVAVDARDDIEQARSSRVF

>CORE_REP|Org191_Gene1599#

MTTLTKFDQPVKAPTFRDISTSRKVKNHIATAVVSICFAVALIPLGWVLWMVVSEGIGAVLSSTWWMNSQKGILPDQSGGGVYHAIYGTIIQSAVAAIIAVPLGIMAAVYLVEYGRGRLAKVTTFMVDILAGVPSIVAALFIFALWIATLGFPQSAFAVSLALVLLMLPVVVRSTEEMLKLVPDELREASYALGIPKWKTIVRIVVPTALPGMISGILLSLARVMGETAPVLVLVGYAKSINTNLFDGNMASLPLLIYQELANPEAAGRERVWGAALTLILLIALLYAAAAVVNKLLTRNR

>CORE_REP|Org203_Gene471#

MRASFLFGEVVEGLRRNVTMTIAMILTTAVSLTMLGGGLLAVRIADKTEQYFLDRLEVRLYLTEDVSATDPDCSLEPCSSLMADLKATEGVESVQFLNRDDAIREAKEKTFKDQPELAEYVADTPLPASLRVKMVDAQLYPTIYESFYDRPGVGMVRNDKDIVDRLVSLFDGLRNAAFGLAILQAVAALLLIANMVQIAAFTRRTEVGIMRLVGATRWYTQLPFLLEAVVAALAGSLLAVAGLFIARPLVVDRALGDLFASKVFPRITGDDIAMTALIIAPIGVAFAAVTAYATLRYYVRE

>CORE_REP|Org213_Gene3473#

MSSVQSTATVTTTPSEEASATSEQAGTEAGTTFHELPAVRGRSENSPLTWARHSVLQCKRLLIGWLRDPATTIQTLVYPAATLLMFKIVLGNSITTATGMPSVYGQVPMICLVAAMSGAVVSALGFKVEKTTGLLARFHTMPMNRAAGLTGRLLAEAVRVFITTLFVLAVGFALGFRFGQGPLAAIALIGIPVLFGVGFAVLVTALATLTEGVLLVSVIGIINTLLMFFNTGFVPVFAYPTWLQDVVANQPMSTAIDAMRGLSYGGPVAEPLLKTVAWTVGMIVVFAWPAVRGYRRAAETS

>CORE_REP|Org17_Gene4533#

MSANASVLYDVPGPKARRRHALYSVLVLAVLVVLGWLVWRAFDEKGQLTAEKWKPFVESEVWQTYILPGLRGTVVAAALAIVFAMVIGVVFGLLRLSDHRVVRWVAGVIVEVARAIPVLILMIFLYNWFAKDNLFASDQLALAAVVIALTVYNGSVIAEIVRSGIRSLPRGQTEAAQALGLRKGQMMRIILLPQAVTAMLPALISQMVVALKDTALGYQITYQEIVRQGQQLGAAEQNTVPALIVVAVIMIALNWALTVLATRVEQRLRSRRRGRTVLGVNSVLTDAAPGVDLSLSRTAAP

>CORE_REP|Org102_Gene972#

MRLAPTGKSADTRNATDAEIPGADTACMPPEHTRRLVHVPAWRDEGRIRWSYHPDMGELTAEAVRTALAELADPADAAHLRRFFKCGPGEYGEGDVFIGVRVPQTRKVAKQFGGLPLSEVDRLLDSEVHEHRLAALVILNARMATASRVRTGSAEEQCAIVELYLAAMRRGRINNWDLVDVSAEHILGPWLLEQPRDPLYELAASDSLWERRIALLTTFAFIKAGDASTTLALCERVLSDRRDLIQKAAGWMLREVGKRVDPTALIDFLEAHAADMGRTALSYATEHLTAEQRAHYRAQR

>CORE_REP|Org202_Gene3796#

MFTEAQLYSPVTRDRDGAVTVHLSDEHPGVRDPDYRARRNAIAALALGYTPGAALPRVDYTEEEQRVWRMVSTELARKHRTYASAEVLAAAERLALPTDHIPQLDEVSATLAPLSGFRYVPAAGLVPLREFFGSFAESVFHSTQYIRHHSAPLYTPEPDAIHEIIGHANQIAGPRFAAIYRTVGAAVARLRTEAALKFLADVFWFSMEFGVVRERGEIRCYGAGLLSSFGEIEEFRRARLRPLDVVAMGTEPYDITHYQPVLYCAESIGQIEDVIGGFFAEMDDETPLRARRVGSGSRG

>CORE_REP|Org58_Gene4507#

MTTDVSGDLDRSTATDAPFAAVCALTALPGPTPDAGRYRGVIDPVWTIGPKVHGGTMVAASAAAATAWLRADGSAPAGMAPIAASSDFLGAPEPGEVGYEVRTRKIGRQICLVDVTLTQNDTAKVRTAFTFGRLDDAEPRFAHRHDDMPVEPPAGAMEYEGSPLGKVVNVAKGADLALDREWARFLDGEQGVPRLRMWIRPFEGDQRDPDVAAYFAMMAADMSPPVPMNLGHFGWAPTVQMTTYLRRRPAPGWLRVVATTQEVGARMFDCDQLVLDSTGAVVAQSRQLALLPQPRDGRG

>CORE_REP|Org45_Gene3467#

MLVRVQNTAGTHAEKVLIDWLRTWKGRGDPHGVATVNCSLFHGDRLYPFDAVVWTPTSCVVIEAEALVERLEGELEVPLSGPWRGADKIINFEGGDRRTPLDKSRDHTFALQNWFAERGLGQRVVHGAVLVVPPPGSKVRVRQLWSDPSFEVLLGDDPARLREYFDSIASRGRPQWSANDVAWAFRGLGILPYLPAPQDLLNEGFLGPVDVTLWHGGPQQAQAEAYAEEQARLEREAERPHRRIMSVPAPWYSPWKLYPRRSGELDVGGSVMRVLLTIGMVVAAVWVIWFVLAALLTYG

>CORE_REP|Org120_Gene2356#

MIGPMKIRKAVIPAAGIGSRLLPLTKAIPKEMLPVGDKPVIEHTVRELVSSGITDITIVVSSGKSLIQDHFRPNPALVAQLRADGKTAYADAVEEVGELSRLGHITYLDQHGPYGNGTPVLNAARNLGDEPMLVLWPDDVFVADVPRAQQLINAYEQTGAPVLALMPMDPTESQRYGVPVVADDQGHGLLRITGLREKPKPEDAPSNYAAIGGYVVTPGVIEELRTQTRAWYEHRTGEVYLTDAINVHAADNPVYGQVIRGRWYDTGNPADYLVAQFASALANPQYGPLLRTLAEDTAS

>CORE_REP|Org4_Gene5905#

MTRPAARRTSPAVAAAISGAVDLSSLKQPPAGASGAAGGGDYAVTEANFETKVLRRSVQVPVVVALYSQRSPGSVELVRTLERLVGESGGAWDLATVEAESNMRIAQAFGVQGIPTVIAVAGGQPLADFQGAQPEAQVRQWLSAVVDAVAGKLPGGEAPEEAPEDPRFVAAETALEQGDMAGAEAAYEAIIAAEPGNEEAKGALRQLRFLARAQEIPETAIATADADPANVDAALDAADLELLSQLPEAAFERLIAVVKRTADDDRTKARTRLLELFELFDQAEPFVVAARRKLAAALY

>CORE_REP|Org1_Gene4754#

MRASKHIPLNDENGAVMTSPLRTPARHEPPAYDIRDHLDGSAAFFGAAANVIMQLSLPPVGYGVLESTVDSGKIMLHPIKRTRTTLTYLAVAMLGSDDERAAYRAAVDTAHRAVRSGPDSPVRYNAFDRELQLWVAACLYWGARDLYERMHGPMDPGTADGFYRAAERLGTTLQMHPAQWPPDRAAFDTYWREHLATTRIDPPVREYFWDLVNLKMFPRPVRLAVAPFHRWMVAGLLPERLREQMGMRWSPRDDRRLARLLTTVGAVEDRLPRPVKTFPVNAFLWDMRLRRRLGLPLV

>CORE_REP|Org3_Gene5341#

MMAATTMTKEKPAAPVTRNVPWTRRLGSARLYIGALIVLVWGLGPFYWMAVTAFRDPRYTFENTPWPTHVTLENFRDVFDTSRGNDFGQAMVNSVIIGSITTVIALLLGVLAAYALARLTFRGKYLVSGLILSASMFPVVVLVTPLFQLFTDLGWIGKYQAMIIPNISFVLPMTVYILAAFFAELPWELEEAARIDGATKMQAFRLVMLPLAAPAVFTTAILAFIAAVNEYLLARLLSSESTKPVTVAIAGFSGNNPLVQPYASIMAAGTLVTIPLVIMVLLFQRRIISGLTAGGVKS

>CORE_REP|Org109_Gene7387#

MRAAEEVVRPRPIGCVNVDTVSLSGKELAAAVNADTKARAAALTDRGTTPRLALIVANDDPASAWYVNSLRKAAERLGIACDTVDLGPEAGVAQIRAELTARGADAATDAIMLQTPLPAGVTLDDVSSAIVASKDVDGVSPLSLGLLAAGLDGFVPATSEAVVELLEHHEIPLAGRHVAVVGRSNVVGKPLAQLLLAKDATVTVCHSRTADLAAVTAAADVVVAAAGRIGLISGKHVREGAVVIDVGTNEAPDGKIVGDVDADSVRGKAAGLSPVPGGVGPVTTALLMRHVVIAAESR

>CORE_REP|Org105_Gene5273#

MQRIGTGGQILLTGSKRSGGFGHLAIRPERARADTVAVMLAEQAAAAIAERTGVPRHPVAVVLGSGWQDAAMEIGTPTASVSMSELPGIAVPTALGHVGMVHSIPVGETPVLVLMGRQHLYEGHAPDDVVRPVRAAIAAGAQTVLLTNAAGGIRPGLHVGEPVLISDHINLTGRTPLSGATFVNMVDAWDPRLRELARRVDPSLTEGVYAGLSGPQYETPAEIRMLGTMGADLVGMSTVLEAIACRALDARLLGISLVTNLAAGVTGEALSHAEVLAEGRAAAPRLGKLLRGVLEQL

>CORE_REP|Org49_Gene5817#

MSVGPRGHSDVPATQYEEESVKHIHAGKVRDLYEDGDELILVASDRVSVYDVVLPTPIPEKGALLTQLSNWWFRFFADVPNHLISTTDVPAEFAGRAVRAKKLSMVKVECIARGYLTGSGLAEYRRTGSVSGVALPPGLVEGDKLPEPIFTPTTKADEGHDEFITFDDVVNQEGREVAERLRDLTLDVYARGAEHAASRGVIIADTKLEWGWDGDVLTLGDEVLTSDSSRFWPADEYAPGRPQPSFDKQFVRDWSTSTGWNKEYPGPEIPADIVAATRAKYQQAYELITGETWTGVS

>CORE_REP|Org105_Gene1742#

MTGSGSLGDALRFGIVLFTSDRGITPAAAAKAAEATGFHSFYVPEHTHIPVKREAAHPQTGDESLPDDRYMRTLDPWVALATAAAVTERIELSTAVALPVEHDPITLAKTIASLDHLSGGRVVLGAGFGWNTDELTDHHVPPNKRRTVLREYLEAMRALWSQEEAEYHGDYVDFGASWAWPKPVRGSVPVLIGAAGTERTFGWIAQSADGWITTPGETDISDRLALLRKIWTDSGRPGHPRVVALDIKPDADRLAEWADCGVTDVLYGLPDKPEADVVAYLERLSDKLARIAGPAAG

>CORE_REP|Org101_Gene1038#

MSGSRRGSVPGMETVPIQMPDGSTVPVRLLPASGAHRHPVTPDAPRPVLVVVPGLGVPGEYYDGFALGLSRRGFDVAIGELRGNGASTPKPSAASTYGYHELVSVDFPAIFQVVRDRFPASTPYLLGHSMGGQLAVLYAARIRGRLGGLILIASGTPYHRGYRGLSGPGMLVGTAAVSLTANLAGFWPGDRISMGFGRQSKVLISDWARLARTGRFVPVGADIDYEERIARLKLPVLSITMTGDELTPPGSAEHLLAKLPKAEVTTWRAPEPLGHNGWIRDYTGTVDQIEKWLRDRQ

>CORE_REP|Org105_Gene5032#

MSGSGPEAVAERNHAGPPLTSDGQSVTAAATTEAGGAQLFAELPVAKLSSFAQWRALTGRIIWTMATKGELIVAIITPLVFTLGFYLPLRYVMKFQGIDYAQFVMPIIVLQTMAFTMMSNAQLSAFEALTGLSTRLQTMPIGTLVPLSARISAGVVRSVCSLTAALIYGHIIGFRFEAGWGQAILFCVFSLAISIVLSLGADALGSLTKSPESLSQALTLPTLIFGMLSCGFVPESGFPEWIRPFVRNQPISQLSFALRDMAADGVTWQVLWVPLVWLIGAAVIFAPLAVWASVRRS

>CORE_REP|Org196_Gene4574#

MTTGDTVTSTVELTYESTSRFAQVRPDLKLHYHEAGVGNGPTIVLLHGGGPGASSWSNFSRNIPVLAQNFHVIAVDQPGYGKSDKPTEHPQYFVHSASALKDLLDTLGITDRVHLLGNSLGGGTSVRFALDYPDRAGKLILMGPGGLSTNLFAPDPTEGVRLLSKFTYEPTRENIEAFIRIMVFDQSLVTDELIDERFAAAGTPESIAAMKAMGKSFASADFEKGMLWRDAYKLRQPVLLIWGREDRVNPLDGALVALKSIPRAQLHVFGGCGHWAQLEKFAEFNRLATDFLNGVK

>CORE_REP|Org25_Gene1095#

MNSAGAAVNSEDARLLVERVGPLATIQDLGRPGWFDSGVGVSGAADRGALRLANRLVGNPEGHAGIEVLLGGLTIRTRRHTTLSVTGAPAPARVDGRPVGHASVLELEPDQELSLGIAATGLRSYVGVRGGVDVTPVLGSRSRDTMSGIGPAPLRPGIELPIGPAPRSFPTVDLAPVPDLPAVLDVRAIPGPRDDWFTDADVLFAGRWTVSADVDRIGVRLRREAGPVLERRLGQELPTEGMALGAVQVPPSGQPVVFLADHPITGGYPVIAVLADADVDVMAQARPGQTLRFRRV

>CORE_REP|Org90_Gene3511#

MQKQIYTAPRRSPPNSEGRPVNNPLKGRTLLMSGGSRGIGLAIAIAAARQGANIALLAKTDVPHPKLPGTVHTAAAEIEEAGGQALAVVGDVRDEERVREAVDATVDRFGGIDIVVNNASAIDLSGSEQLSLKKFELMQQIQLRGTFLLTRTAVPHLRKSDNPHILSLSPPLNLSEKWLGEHPPYMLAKYGMTLLTLGFASEFRKEGIAANCLWPQTLIATAAVRNLLGGEEAVATARKPEIVADAAMLVLVGRAAATTGETFLDVDVLAENGITDLSEYGPSSDLAFDIFVDPAH

>CORE_REP|Org159_Gene5771#

MMTQAPAGREKTRVITLTEPPKNSRLFAKAALGAVPLLSARKPTLPDRAVRLDGLRVDPDHLAAYCRATGLRFGDALPLTYPFILTFPLAMQLVVARDFPFVAVGAVHAQNVIERTRDISVSEPLDIRTHIENLREHPKGLLVDAISDVKVGRELVWHQVTTFLHQQRTSLSGGPKQEPKPDEVPPPPLRTLRVDQKTITRYAAASGDHNPIHTSALGAKAFGFPRSIAHGMWSAATVLGAVEGRIPEQTTYSVKFGKPILLPSAVNLYADQVEGGWDLALRHPKKGYPHLTATLR

>CORE_REP|Org4_Gene6416#

MRETVCVYVHRLPPTTAGRKNAIDMAEFTTGEAVALELPIARIPTRAAAFLIDAVAQFALASVLFVLAVAVLLPNGADTAWLSVAVIVTLVTVLVGYPVACETLSRGRTLGKLLLGLRVVRADGGPIDFRHALTRGLAGAIVDFWMLGALGAVAVLTSLCSPNARRVGDVLAGTVVVHAQRALPVPLLAVPPPWLVGWTAQLELAGLPDDLALAVRQYLSRSKTLTPEVQHQLGSALVAAVCARLQVPAPSGYPPLQILGAIIAERQRRVLPPPLFPVFPRGPLGPPAVPGVAVR

>CORE_REP|Org125_Gene1023#

MTHRSFVNPHPPAPTCYRHPDRTTGLACTRCGRPACPQCLQPAAVGQHCAECVAQGRTDIRPVQPTVGGPFATARRTPYVTYALIAINVAVFAVTASQAHSVTDNHVSRLFYDWVLAPQWVAQGQWIRVLGSGFLHYGPLHLVVNMFALYILGRDTELVLGRSRFLAIYLVSLLGGSAAVMWLATDSATAGASGAIYGLFGATTVILLRLRQSPVQMLVLIAINLLISVSLPGISLWGHLGGLVAGTLAAAGILFLPEWLRARTRDSIRWIGWGAVAGLAVLALALIGAGAAVLA

>CORE_REP|Org151_Gene2073#

MRDKRMSLDEVVGELRSGMTIGIGGWGSRRKPMALVRAILRSDLTDLTVVSYGGPDLGLLCSAGKVRKAYYGFVSLDSPPFYDPWFAHARTSGALVAREMDEGMLKCGLEAAAARLPFLPIRAGLGSAVPDFWDGELRTVASPYPDADGRTETLIAMPALNLDAALVHLNLGDKHGNAAYTGVDPYFDDLYCLAAERRYVSVERIVDTDELVKTVPLQALLLNRMMVDGVVEAPGGAHFTLAGDSYGRDEKFQKHYVQSAKTPETWQQFVDKYLAVSEDEYQAAVREFAQEAQK

>CORE_REP|Org150_Gene6394#

MSTTSEYRVGDAMRQKRLGRKLFNAASGPVEGIAGLGDQLSFHLHGIAWIPRTLRRYRREMIRLVAEVSLGTGALAVIGGTIVIVGFLTAAAGYEVGQQGSNSLGRVGIEALSGFISAFFNTREAIPVVAGVALTATVGAGFTAQLGAMRVSEEIDALEVMSVPAVPYLVTTRILAGLIAIVPLYAIALFMGYASTQFVSIVLSGQSEGTYTHYFNVFLVPSDVIWSLVKVIAFALVVMSVHCYHGYHASGGPAGVGVAVGRAVRASLISIMIIDLIIGIAVYGGIHATVRVSG

>CORE_REP|Org12_Gene1519#

MATETAPRPVEFDDSGLNVWSDEERFEVTRERIAEYAAATNDPIPAHLSGDIASPVFAIVPVFEAMMMPVIDVVPMDIFGRVVHGEQDFHFHRPIRPGDRLVSRAKAVGYEGRENGTTITILIECRDSDGGLVNEQYLTAFFHNIDVGKRVGESAPAHKFDPELEAQPPLARVAAHVDDDQTYRYAPASGDPVPLHLDEQVAKDAGLPGIIAHGLCTMAMSSWAVLTEVAGSDVHRLKRFAVRFSTMVFPGDGLETRIWKVGSTGSDTTYAFRTARGTDLVLTDGLAVVADNS

>CORE_REP|Org138_Gene2183#

MSSSPPSAPDPSGPGIGSAQENQATKIRKRRRGKRRFAGGLVLLMGLVGAGFTASALTPDAQVATANEDQSALLREGKQIYDTSCVTCHGVNLQGVEDRGPSLIGVGEAAVYFQVSTGRMPAVRNEAQIMRKPPKFDARQTDALGAYIAANGGGPTVVRDADGEIAQESLIGGADLGRGGELFRMNCASCHNFTGKGGALSSGKFAPPLEPANEQQIYTAMLTGPQNMPKFSDRQLTPEEKRDIVAYVKDRTETQSEGGYGLGGFGPATEGLAAWIVGITLLVGSAMWIGSRS

>CORE_REP|Org128_Gene4969#

MRLPRSRVGGHPIHKVDAAREHATLPESSLPIGVSADYELPGAARSDVRTEVEVRPEAEVGPQARAVVANGADFDDTESVAGDAESVAGDAESVAGDAADDALSGTAAFDATGDRTMMPSWDELVREHADRVYRLAYRLTGDPQDAEDLTQETFIRVFRSLQNYQPGTFEGWLHRITTNLFLDMVRRRNRIRMEALPEDYDRVPSEGPGPEQVYHDARLDPDLQRALDALAPEFRAAVVLCDIEGLSYEEIGATLGVKLGTVRSRIHRGRQALREYLAHNGSQQRFAAEEKVG

>CORE_REP|Org127_Gene749#

MAVVTMKQLLDSGAHFGHQTRRWNPKMKRFIFTDRNGIYIIDLQQTLTYIDKAYEFVKETVAHGGTVLFVGTKKQAQESIAAEATRVGMPYVNQRWLGGMLTNFSTVHKRLQRLKELEAMEQTGGFEGRTKKEILMLTREKNKLERTLGGIRDMAKVPSAIWVVDTNKEHIAVGEARKLNIPVIAILDTNCDPDLVDYPIPGNDDAIRSAALLTKVVASAVAEGVQARASRASGDVKPEAGAGEPLAEWEQELLAQATPAAEGGEAAAEAPAETAAEAELKEEPATKTPADF

>CORE_REP|Org184_Gene952#

MFASGIRSSGIHALSVSSDVTPWLRSSGLEIVLLILGAVLFSRFATFVRDRVTSKIDAGFQSSDALVRTEAAKHRHALAQVVTWVVLTIVYVLVGMEVLQRLGFAVTGLVAPAAVLGAALGFGAQRIVQDILAGFFLITERQYGFGDVVRINVTGAADPAEGTVEDVTLRITTLRDADGQVIIVPNGQIVKVTNLSKDWARAAIDVPVSASADITRVNEILHKVGEEAYRDRRLEPLLLDEPTVMGVEDLTVDQMNIRMVARTLPGKQFEVGRELRVRVAAALRREGISETA

>CORE_REP|Org162_Gene2570#

MAESTPARTTAADASASARRRGSRGDSYEHLEPLFARLAAFESDDPRRERVRSEIVRLGLPLAEHIARRFANRGEPFDDLLQTARVGLVQAVDRFDHTRGAAFLSFAVPTIMGEVRRHFRDHTWSVRVSRHTKEIHGRIGSATEVLAHRLGRMPTARELAVELEVDVTEISRAMIAANCYTTDSLDMTVRDHDGDSSTPAVERLSTEEPCYRLLEDAMAVRPLIARLPQRERQILIWRYFGAMTQSQIADRLGISQMQVSRILSRTLTRLRDEALADPEPAEDDSRSALSVR

>CORE_REP|Org110_Gene4579#

MGEDWSRWLDEAPEAGESSGKPGRSKAPVVRLRRGKGGGSGADQPLQRPILVVGGCGGAGTTTTALGIAGELGMAGTPTVAVDATQAGSDLALRGADEHLHPISLQSWLYGRGDDEPAPLKECLSRATSGIGLLWRDSAPLRRRATYLTVARAVYDSGHTAVYDGGSPIAGRQLRPLLDDADVALVLAIPARADAANRLRVTLEWLDDQFGDSAEGQGGGIVGDTTIVISHQHPGTESRVAEHLREHLSGWVRDIREIPYDPHLARGELVRHASLAIETRRAYGRLLAGVAS

>CORE_REP|Org44_Gene2418#

MAWNPTQIPDQTGRTFVITGANGGLGAETTKALADKGATVVMACRNVAKAQQVADGIPGDVRVAELDLADLASVRAFAERAEEFDVLINNAGLMYIPFSRTADGFETQFGVNHLGHFALTGLLLDKIRDRVVTLASIAHRQTPKLWIDDLNYERRRYYRNLAYAQSKLANLMFARELQRRLAEAGSPKRSYAVHPGVSATELFARTETPLDRIAKPIIRLVGHPPAKAAHSTLFAATMPDADPGTYWGPNRLFQSQGPVEPSPSTRLSKNPELMRRLWAESERMTGVTYPV

>CORE_REP|Org47_Gene2557#

MATAAQWIEGARPRTLPNAIAPVIAGTGAAASIDGLVWWKAILALLVSLALIIGVNYANDYSDGIRGTDDERVGPLRLVGSGLASPAAVRTAAIVSLGVGAIFGLILVALTAWWLILIGAACLAGAWFYTGGSKPYGYRGFGEIAVFVFFGLIGVLGTQFVQAERVDWVGAVVAVAVGAFSSAVLVANNLRDIPTDTESGKVTLAVKLGDPRTRTLHLVLLAVPFIATLLLVARSPFALVGLLAIPLAVRANAPVRSGRGGLELIPALRDSGLALLAWSVLTAAALGLAAL

>CORE_REP|Org214_Gene5800#

MTRIAVIGGGRIGEALIAGLLESGRLAKDLVVVEPVTERAAQIAEQFSVRVTDSVADAAVGADLLVVAVKPADVDAVMTALGKAALSDNASVGNDRDQILVSLAAGVPTARLEAKLPAGFPVVRVMPNTPMLVGQGMSVIAPGRYARAQQLELVTDVLGAVGKVVTVAEAQMDAVTAVSGSGPAYFFLIVEAMVDAGVGLGLTREVATELVVQTMIGSAALLQESEQSAAELRAAVTSPAGTTAAAVRELERGGVRSAFLEALHAAKQRSAEQGGVSDGPVGVGTGVGAGA

>CORE_REP|Org5_Gene1239#

MTKVTGTAEPGPGIRHTVKVGSMYAASREASSRAREALSAALTGSEAVAATTGSELFAVVAVLDDQRSLRVALADKSVASSVRADLAERVFGGKISAATQAVLTTAVAQDWSRTRDLVDTLVLLGQEALLRAAADRGRIDAVEDELFRLGRTVEDNPDLEQALTDRGKPAQAKRDLLARLLTGKVEDVTMQLAEQAVGRAHGDVGVAFDQLSDLAASLRKQIVAHVRSATALTQQQRDQLAASLQRIYDKPVTIHVQVDPTLLAGVVVHIGDDVIDGSAIGRLQRLRQALA

>CORE_REP|Org5_Gene234#

MPDTLATPTVEDMAPTHSSSADDYSASGLRPDRFDRAGRDQLTDVNDLQAALPGIRDLRSWAHDALAVAPGESAVDIGSGTGSEVLVFADRVGPTGDAVGVEPDPNLLAAAERRAAEQNSTARFVTGDAYGLPFGAGTFDAVLCERVFQHLTAPNRAAGEIARVLKPGGRAVVVDSDWGTALVHPGDRGVVREVIETLISNTTNPFSGRRLPGLLTGAGLVVDDIGSHALVQDATIGAGALVARISAMAVARGSITEAQRHELLAELEAGARSGDIHLSVTMFAVLAHKPQ

>CORE_REP|Org176_Gene1443#

MAGKRTVLTVRLRRLAAMLHEMRENAQLSKEEVSAKTGINVTTLYRIETAQARPQRRTLMAMLDLYRIGEEQREDALELLSDALKPGMSRAYEGSVSEVYAAYINFESEALSARHYQTSIVPGLLQTYEYATAVIDTSMPKLEASVMESRAKARMDRAVNLTKEDPLELWVVMDEAAIRRTVGGPAVMRGQLDRLLQEIKRKNVILQILPFDAGAHPGMAGSFTLLDFPDPADPELVYVEGIAGDELIEGHTEIRRFGVIFDQLRAMALSPRDSAAMIVETARRMDGMDQ

>CORE_REP|Org105_Gene5129#

MSARIGHLPAPASGPGGTIVAMSSHARPAIAVIGGSGFYDFFDHEAVAVEVDTPYGAPSAPVAVGEVEGRPVAFLPRHGKRHEYSPHTLPYQANMWALRSLGVRRIFAPCAVGSLRADWVPGTVAVPDQLVDRTSGRPQTFFDAGGVHVSFADPYCDELRTAAIGAASDALPMRDSGTMVVVQGPRFSTRAESRWFAAQGWELVNMTGHPEAVLARELEMCYAAVALVTDLDAGLEEGDGVHATDVFAEFEKNITPFKALIRRAIGAVDGADTCARCRVHAGVSLPFDLP

>CORE_REP|Org113_Gene5858#

MSAAQDNSVSDTSAETTAYGAAPATSRRKTRAHHLQQWKAAGEKWSMLTAYDYSTAKLFEEAGIPVLLVGDSAANVVYGYDTTVPITVDELIPLVRGVVRGAPNALVVADLPFGSYEGSPEQALASATRFMKEGGAHAVKLEGGERVSEHIARLTASGIPVVAHIGFTPQSVNGLGGFRVQGRGDGAEQLVADAIAVQEAGAIAVVIEMVPAEIAGRLTHKLTIPVVGIGAGNDCDAQVLVWQDMAGYTSGKTAKFVKRFGRVGDELRSAAAAYAEEVARGTFPGPEHSF

>CORE_REP|Org184_Gene4302#

MRSIQTTGPVARRGGDVATIEEALEIERLERDIFRGASPKTQLQRTFGGQVAGQALVSAVRTVDPKFQVHSLHGYFLRPGNPDQPTVYLVERIRDGRSFCTRRVTGVQDGAAIFTMSASFHVGDDGPVHQDEMPVVQPPEELPDAKTTMSPERLWAMREWEHWDIRPVPQESVTQRPGVVSPQQVWFRYRHPLPDDPLFHVCTLAYMSDMTLLGSSKVNHADEPTQDASLDHAMWFLRPFRADDWLLYDQSSPSAGFGRALTGGKIFNRAGELVASVVQEGLIRTRRDK

>CORE_REP|Org155_Gene4641#

MGPGRRIAVRSTKEFAVPLDASGASLLAAVQASPRAVAAHDRQTWVGLFTADATVRDPVGARPHTGRAAIEKFFDTFIAPNTIEFEVAHDFTGPGIIVRDLHIRTTMSTGAQVLVPMHLRYDLTEIDGELRISHLAAHWELPAMVAQLLRTGTRGLGAGMQLGAALVRNQGLTGAAGMARGFTGVGRAGKRVAAELFDAAASGDTAHVRRLLGAGTVIECPAGTVVSADEFTERAEEGLRAGKVIAAGRSVTASVELDGAPAVIALEFDLDAPRIHHVVVFTELVPDQP

>CORE_REP|Org102_Gene5292#

MPDNPAPANLTPAEPTAADRAPADSAPLNLAAGAPAPGNPAQPVAFVTGAARGIGAAIAQRLAADGATVAVVDLDENSCAAAVDTIVAAGGKAIAVACDVTAEDQVDAAVDRVAAELGSLDILVNNAGVLRDNLLFKMSVAEWDTVMSVHLRGAFLCSRAAQRHMVAQRSGKIVNTSSVSALGNRGQANYSAAKMGIQGFTRTLAMELGPYGINVNAVAPGFIVTEMTAATAARLGVSSEELQAKTAEITPLRRVGQPADIADVVAFLASENAAFVTGQTIYVDGGRRL

>CORE_REP|Org117_Gene5952#

MPLLRVAVPNKGSLSESALTLLTEAGYRLPRVRNKELNCFDPENEIEFFFQRPRDIAVYVGAGTLDLGITGKDLLDDAAAPAESVLDLGFARSTFYFAARPDGPKSVADLAGRSVATSYPELVRKHLAQAGVSANVVVLQGAVENAVALGLADAIADVVETGTSLENAGLVTFGEPLMRSEAVLIRSTTAEWTDERAEAVTVMLDRLNGVLTARRYVMVDYDCPRAVLDAACALTPGIESPTVSPLADPDWVAVRSLVERKSINRTMDDLKKLGASAILATELAACRL

>CORE_REP|Org1_Gene3177#

MTAGDPMRLHPGHALSSFTEHLRALAPELLGPNRFAALDGATGSSGGTGAKDIAPHGTTIVAVSYRGGVLIAGDRRATQGNLLASRDMDKVYITDTFSAAGIAGTAGMAVELVRLFAVELEHYEKIEGVPLTFDGKANKLSKMVRDNLPAALQGLAVVPVLVGYDERAGDPDRAGRIVSYDVVGGRSEERFGYTAVGSGSMFAKTSLKKLYAKGIDQARALRIALESLYDASDDDTATGGPDLLRGIYPTAVVIDAEGALEVPESRLEEIARGIVADRTAAQEGSAGA

>CORE_REP|Org112_Gene446#

MSVRTRKPLVPGTQSPIREVPKSIERPEYVWKKTVNEGHEPWVQTPETIEKMRIASKIAAQALAEAGKAVAPGVTTDQLDAIAHEYLCDHGAYPSTLGYKGFPKSCCTSLNEVICHGIPDSTVIEDGDIVNIDVTAYIHGVHGDTNATFLAGDVDEEVRLLVERTEEATMRAIKAVRPGRALNVIGRVIESYANRFGYGVVRDFTGHGVGPTFHSGLVILHYDQPAVEAEIEPGMTFTIEPMINLGGIDYEIWDDGWTVVTKDRKWTAQFEHTLVVTDTGAEILTLP

>CORE_REP|Org144_Gene5001#

MGSRYTPPALRPFRLIGAAAQGPVRANQRAGHQAITFVAAIAAIPFALKHYRKEVLRLTADVGWGNGSLIVGGGTVGVVVILCGFGGITVGMESYTALNLLTMNPLTGAISGFATTREIGPILATLAFAIQAGCRFTAQLGAMRIAEEIDALESIAIRPLPYLVSTRMIAATLTIVPLYSVGLAVAYLMTKLSVLFLGGTSAGTYDHYFFQFLNGADVFFSVLKVMVFVLLSTFLQCYYGYVATGGPEGVGQAAGRAIKMVIVVMVFANLFLTLAIWGIDPGFRISG

>CORE_REP|Org139_Gene5071#

MGGDGLDEAGSERGAGVRPVLISADELRDALSDNRVRLLDVRWALGDPDGPQHYLDGHIPGAVFVDLETELAAPPSPARGRHPLPDIGQLEKCARSWGVCTGDTVVVYDATGGMAAARAWWLLRWAGVADVRILDGGLPAWTATGAELATGAEPDPADGDVELSPNHLPVIDADTAARWPGALLDARAGERFRGEQEPIDPRAGHIPGAISAPTAENLTADGHFRSPDELRKRFADCAGPVAVYCGSGVTAAHQIAALAVAGIDAALYPGSWSQWSNDPKRVVATGE

>CORE_REP|Org17_Gene5165#

MMALDPQLDPGELRTLIRTALDEDLRYGPDITSAATVPAEATVKAAMVSRQPGTVAGIDVGLLVLDEVIGAGNYEVTDRVADGTRVGPGDAVLTVVAPTRQLLTAERTMLNLVTHMSGIATATAAWVDAVEGTECRIRDSRKTLPGLRALQKYAVRVGGGVNHRMGLGDAALIKDNHVVAAGSVVAALRAVRELDPDIECEVEVDSLDQLDAVLAEDVELVLLDNFPLWATQAAVQRRNSRSPRTKLESSGGLSLESAADYARTGVDYLAVGALTHSVRVLDLGLDM

>CORE_REP|Org91_Gene1863#

MSADDVARVFAIEDKVERLKAATDGVAAAQQTINELTRIRRAVIRELHAEGWTFARIGAAAGLSRARIHQVSTQGPAPEGLFFGHGSLTVLTPRVRTGRLIGAPDPAAAPRLADLLRELGFAVTVEQFPPGHPLDLNRDGLIVLGGPELSPSLRQLIAADPRLRRSVARAGDGRRGIEDRAARRVYRPSGPDPHDIAYLARLPRPDGQGSILVIDGLHPPGSLGAVRLLATRLAGLHERAGTRRFSVLIAVRFDRATGEPLEAELLTPVYRHEPAELRPTAVRTRR

>CORE_REP|Org1_Gene1643#

MAKGAWSGFAGEAAACGVRSRLGCRGGRWPHPSIVCRSSPPLNVEPSIQAKLLQLAAVDAELTRIAHRRTVLPEQQEVARLEARRNEHKDAAVKVEIVLDDLDRDIKKLEGEIEAVRKREERDRGMLTSGSVGAKQLSEIQHELGSLERRRGVLEDELLEVMERREASASDHDHAGAQLTRTEQELADAQRQRDEALADLDVAQARCENDRGELVGLFPDELLAVYDRQRAQRGVGAALLQARRCGACRIELDRGEIARIAKTAADEVVRCPECGAILVRTKESGL

>CORE_REP|Org118_Gene1527#

MSGIYRDEAPLRDSGQVVDGAPGPAAGGRVPITVIEPEGYARGGIVVLHESRQFADVLLEMMKSLAGEGWIMVAPNLFHRFDRQKGHGDGAGTVNDRDLAAVGARVSDGPGAVATAVAGGAEVEQVFGADLFADFDACFDWLVGRGVYADTVGVLGFDNAGTAALLVATNRPIGAAVSVAAPGIMEPLTAEATALVDAAPQLQAPWLGLYGNDDPVNPADHVERLRDAAARAAVATLVVSYPGLHHRPDHPGFDMADFEQLPEDEKRLLIDAQTRIFDWFDSHLR

>CORE_REP|Org176_Gene4330#

MPLTPADVHNVAFSKPPIGKRGYNEDEVDAFLDLVEQELSRLIEENADLRQRVAELDAELADAKKNRGPGVVNAVKPPVPQAPPPQPEPIKPPVPAAPPAPMPAAPVAKDAPGADANLQAAKVLSLAQEMADRLTSDAKAEAESLLSNARANSERLVGDARTRSEAMIADARQKSDAMLSDAQTRSDSQLRQAKEKADALQADAERKHTEIMATITQQRSVLESRIEQLKTFEREYRVRLKSYLESQLEELENRGSAVPVDGGEAFADANTANNLAPASFAKGGK

>CORE_REP|Org162_Gene2632#

MLRAAQSGWRRPRFARRATLRRSLRLLGSFKFEQTDPAVFYGGVAADTADLVGDFFRDLTGRSLRGTVVLDVGGGPGYFADEFAKAGARYIPVEPDPSEMHAAGLSVPGAVRGSGMALPFRDDAVDICVSSNVAEHVPQPWVMADEMLRVTKPGGLMVLSYTVWLGPFGGHETGPWHYLGGEYAARRYRRKHGREPKNRFGRSLFAVRAADGLRWARSAPPDIEILAVFPRYHPRWAWWLVRIPGLRELLVSNLVVVAGKRNSTLEAAATPESAQSARAFGFAPR

>CORE_REP|Org138_Gene2622#

MPVSLHSTGKASFDHIYDRPDPREYYARMAELDYCIPELATPHFASLIADYRAATGIAAPTVLDLGCSYGVNAALLRLGISMAELTEHYRDADSDGDGDGDPAAALIARDRARLTTADRLPGVRFIGMDASRPALDYARAAGLLHDTVHADLEAADPTEAQRATLATADLVISTGCVGYITDRTLLRVARAHGGKLPWMAHFVLRMFSFDPIAARLAELGYRTERVPGSFRQRRFAAADERTQVLNTLADNGVDTEGHESDGWLYAQLYVSRPSPTLSFDHEDLS

>CORE_REP|Org3_Gene3438#

MTHDQESGAGSVQTFGDRLQHAMRQFGPLCVGIDPHPGLLDQWGLTDDVDGLEAFAEICVEAFDGCVALVKPQVAFFEVYGAGGIGVLERTIEVLRDSGTLVLADAKRGDIGSTMDAYARAWLGDGPLASDAVTVSPYLGFGSLDPALELAQANHRGVFVLAATSNPEGAELQRITAGDGRSIAQTIVDAAAARNTGDSFGSVGVVVGATLTEAPDLSALNGPILMPGVGAQGGGAESVRGLVPEHLLHGVVPNASREVLREGPSVPALRAKLAAMQEEFGFLQA

>CORE_REP|Org5_Gene2415#

MSAAADRAALLATASEVLDTATPRFVEGVGAPSAVQKGRGDFATALDLELERTLSQQLLERTGIPVHGEEFGGPELSSGTAWVLDPIDGTFNYSAGHPLSGMLLALVEDGQPVLGLTWVPLLEQRYAAAVGGPLLLNGKPLPPLESGRLADAMIGFGAFNIDAHGRIPGRFRFDLLGALSRLSSRMRMHGSTGIDLAYTASGILGGAVVFGHHPWDNAAGVALVRAAGGVVTDLRGEPWSITSRSVLAAAPGVHEELLEMIDSAVDRAAERDDTAGRDENSEGTQ

>CORE_REP|Org125_Gene2582#

MCGRYATTANPASLAVELDAVDETANGAPSDNAAAGKSAPGAGGASAGDKSGNGANYNVAPTNRILTVVRRHDHDHPDDDPALRIRRMRWGLIPVWTKAAEPGVPAKGKPLFNARADRAATAPSFRDSVKKRRCLVPMDGWYEWLTEPHPTGSGKAVKQPYYMSDAQGRRLYMAGLWSVWRDPAQRDMAPLLSCTILTTDAVGDLTRIHDRMPLMMPREHWDAWLDPDRPAPRELLEPPEPQVISSIVARPVSPLVNSVRNNGPQLLEPVAGAGGEQAGQTSLI

>CORE_REP|Org4_Gene3472#

MVRPSAGGRCLPAARAAVVGTASGPCNTVVDVIVALIDSGLGLLPTSAWLRKLRPDVDLLLQLDPDGAPWGPKPEQWTIDRVVRAAGMSIELGAEVIVIPCNTASVTALEHVRAEVGPDVPVIGTVPAIKPAAAVCRSVAVWATAATTASRYQADLIAKFGGNADVVGVACHGLADAIDRGDLVGARESIARAVAETPDDVEGVVLGCTHYPLVIDAIVAALPDGVRLFDSAQAVAAQTIRRMDALGRPTPGNGAVLVRNSGRPGELPASAAAFESGRILGAQG

>CORE_REP|Org1_Gene5227#

MWTMAQNWTIPATEALRADGVRIADLDTSATPGFDGDKTQGQDLLTERTAVLSDLQEKLYANGRSGDKRSVLLVLQGMDTAGKGGIVRHVIGSVDPQGVDHAAFGVPTPEEKRHHYLWRIRKALPRGGQLGVFDRSHYEDVLVVRVHDLVPRAEWEPRYDEINHFERELTDEGITLVKVAMFVSLDEQKRRLRERLERPDKYWKFNPADIDERAYWPAYQEAYQAMLDRTHTGHAPWYVIPADRKWYARLAVTELLIDALSTFQLDWPPAQFDVAEQLRRLDRA

>CORE_REP|Org1_Gene1097#

MHGAPARPGGPRGFSGDRFVSEISSWQHVGMRLTVLGCSGSVSGPDSPASGYLLTGPDMTPVVIDFGPGVLGALQRYADPGEVDIFLTHLHADHCLDLPGLLVWRRYHPTPPVGRAIVRGPSDSALRIGNASAEVGGECDDWSDVIDLRPWQEGETVEFGPGHTIAARRMFHPPESYGLRITTNAGRTFVYTGDTAMCDAVQELAQGADVLMAEASWTHDPANRPPGIHLSGTEAGRIAARAGVGELLLTHIPPWTSREDVIAEAKAEFTGPVHAVAPGEVFDL

>CORE_REP|Org102_Gene1101#

MNVTVLAGVGESFQQTASSGPLLLALGACVLAGLVSFASPCVVPLVPGYLSYLAGLVGAEAPPVSVESAKREAARGGSTALAEKTAKSRARLRVAGAAGLFVAGFTVVFVLATATVFGAIQVLNVNRELLQRVGGVVTIVMGLAFIGLIPALQKDTRMEPRRLSGIVGAPLLGAVFALGWTPCLGPTLSGVMAVSAGTDGTTAARGVALIVAYCLGLGLPFVILAFGSATALRGVGWLRRNSRTIQVIGGLLLVAVGIALVTGAWDQFVSWVRDAFVSNVTLPI

>CORE_REP|Org43_Gene3246#

MSRVSIDTHQAWVEFPIFDAKSRSLKKAFLGKAGGAIGRNQSDVVVVEALRDINLSLREGDRIGLVGHNGAGKSTLLRLLSGIYEPSRGSARIRGRVAPVFDLGVGMDPEISGYENIIIRGLFLGQTRKQMMSKIDEIADFTELGEYLHMPLRTYSTGMRVRLAMGVVTSIDPEILLLDEGIGAVDAEFMKKARLRLQELVARSGILVFASHSNEFLAQLCDSALWIDHGQIRLRGGIEEVVRAYEGPDAGNHVATVLREMAAERAGRAEGSADERELEQNAT

>CORE_REP|Org89_Gene2183#

MSDRTAGMTEPAARTRERTLSVTTLAAGEFHAPSLTDFFPPAVLFEGTPFELDRLMLVRLLMTAVLVAVMLLAFRSPRIIPRGLQNVAEIGLVFVKEQIAEEVLGKETGRKFFPLIATIFFTVLFLNFSGIVPLLNISSNARIGMPLVLAVVAYIAFNYVGIKKYGFFTYMRSSIVVPNVPPALHVLLIPIEFVSTFILRPFTLTVRLMANMLAGHIMLVLFFSATWYFLFDAAAWMKVFSPFSLLAGLGFTLFEMLVIFLQAYVFALLTAVYIGLAEHADSH

>CORE_REP|Org23_Gene5039#

MQRIHNAIQLTGLTDEELFQLYGYPSGLDRPWVRANFVSSIDGAVTSDGLSGGLGTDADRRVFLALRELADVVLVGAGTARAENYGPAKTDPSLRRRLHELGIGGHPDGAPPPIAVVTASAALDASGRLFPESPAAPAGTHSGAVPDSPGDPAAQTPGTQVSDQVAPLIITTAAAPAIRKRQLTDAGAEVIEAGDVAVSPQAVLDALAERGLTRVLCEGGPRLFGDLLAADCIDELCLTTAPVLIGGTAPRIALSREEFRVTMRPAHILLDEDATMLIRWVRT

>CORE_REP|Org5_Gene5557#

MRLRDNLFGVADESESVAVPGADDEPKDESASRRRRRGGKKRKQRPFWQELPILIVVAGVIAALVVNFIGRPYVIPSQSMEPTLHGCTGCVGDRIYVEKLSYDFGDPKPGDVVVFKGPSESWNKGYHSQRSSNVVKRGFQNFFSFFGLVPPDENDLVKRVIAVGGQTVQCCDAQGRVMVDGKPLDEPYANYKYPYQPGLPFATKVAGSLIVDPHGREFGPIKVPDGNLWMMGDNRNESLDSRGHVDDEYSGTVPIDDVRGRAVFKIWPPSRIGPVRSQNPQSN

>CORE_REP|Org128_Gene3051#

MRSGTTTVRAGRSSLRGVQRPPLDVDILRHAVAEQPDLSFFSRIDVVESTGSTNADLIGEAGDPSSDRRVLVAEYQDRGRGRHERSWVSPPRAQIAMSILVRLGGIEPAVLGWLPLLTGVAVVDAVRETTGLDANLKWPNDVLIGGRKVAGILAEVASGAGAPAVVVGVGLNVSLTEDELPVPHAVSLTLAGAENADRNELVLALLRAFARHFTEWRTENWNVAALADAYRARCATLGADVRAELPGGEVITGVATDIDAYGRLIIGDRSVSAGDVTHLRPA

>CORE_REP|Org5_Gene415#

MADATQSTTMAARDMPTLTTFPYPELDEREDDHWSGAQVSEDELRALSLGAFYSARWDAFHDALLLGPERDHPLGDRRELAIDTLTGAWGITDGTEAMASMEQLLEGMHGPLYALVHPLVTHALNSPERDRFGERADRHRAFLRQVGSFRGMDNPEALVRDYDIWTQAIKLDLTGHLVQPLPADIQAWDLARVVAVARMAFTAGYLEADAAWDYVMRALPPAQRRYRNWRQFGDAYLTGWTYWQACEDMAVLKSGGVDRRLELVRLWMRPTSPWRRIALQGE

>CORE_REP|Org4_Gene7954#

MGITVSLSRMALRDVVSADGTNIVYRVSGPAGARPLLLLHGWSANLLCWGRAANELATRYRVIAVDLRGHGYSDAPAAGYDDPKNWAADIAAVLAVERIDTGAILLGWSYGGIVLSDYLTAFGTGALAGVVYTGSMANLGRGVPGAAVGTAMQAAMPGVFEESAGRAVKAFGAFGNANTGPGADKGVDAQRLFGASLATVPAVRKALFYRTVDNTETLRALDIPVLVLHGTADPVVPIENGRYIAAAVPDARTSYWEGAQHGLFIEDRSRFVAEVSAFADGL

>CORE_REP|Org194_Gene3604#

MSNPGTGWEMAGPAWLFCPADRPERYAKAAAAADVVIIDLEDGVAEADKAAAREALIATPLDPDRTVVRVNAAGTVEHMLDLDAVARTGYRRLMLPKCESAEQITTLADYEVIALVESPLGALAVGRAVMARNAIGVMWGAEDLVAGLGGNSSRHADGSYRDVARHVRSQSLLAAKAYGKFALDSVYLDIPDLDGLAAEALDAVAVGFDAKVAIHPSQVPVIRRAYAPTGAEIDWARRLLAEVPNHRGVFTFEGRMVDAPVLRHAERIVRRAQGADSANVGS

>CORE_REP|Org102_Gene4695#

MPAVSERQRGRGGDAARNLAIPAGIGGYAAIILSDMAAGKGGKPSKEAKAAAKAARKQASKERRQQLWQAFQMQRKEDKLLLPLMIGALVGVTALFLIIGLIFDLQWFLLPIGVLLGALAAFIIFGRRVQKNVYAKAEGQAGAAAWVLDNLQGKWRVTPGVAATTQLDAVHRVIGLPGVILVAEGSPGRVKSLLAQEKKKVARLVGDTPIYDIVIGNDEGQVALKDLQRFLTKLPRNIDAKRMELIEGRLSALATRGGPALPKGPMPTGAKMKGMQRTIRRR

>CORE_REP|Org2_Gene75#

MNRSVPVGETFGQDNIRCRPEAYPDSGRQAGDPGAAHVMIATVLLSIGIVFLAELGDKSQLMALTFALRYRWWVVLGGIATASAAVHLLSVGVGYFLGSALPTRAIALVAALTFLAVGGWTLREHFGTADEDEPAPKSLRASTAPFFVVLSAFLLAELGDRTMFATAALATDYDWVGVWLGSTIGMVAADALAIAIGILVGKHLPERAIGIGSGLLFLYFGAMTLISTAAPDLGGLVVALLAATAPALGGAALLATRRRRRTTPAEPADQPAIPADPPTHR

>CORE_REP|Org113_Gene5243#

MRGDGDGFLVSPDGTRQWGRFGAAGLLLRAPRAGGGAVVLMQHRAPWSHQGGTWALPGGARDSHESSVHAAVREAQEEAGIAPDAVRVRGSRITSTAESGWTYTTVVADVPETLRTVANKESAELAWVPEEEVDDRPLHPGFAAAWPDLRASAARLDLGGIAEAEAIAALLPRTVDLAERGFLWLDAIVGAAVFTDLGGSADGAAGALSDAGERTARREPAASGPGDSGAPGSSVGVDGVARSARPRAAGSEVPTVRIIQDINRSAPSSEDLVLTLDQLLN

>CORE_REP|Org97_Gene4529#

MGNLREQIIAELGVAAEIEPKVEVRRRVEFLADYLSSTPATGFVLGISGGQDSSLTGKLCQLAVDELRARGQEATFVAVRLPYGAQADADDAQRALDFIGPDHVVEVNVKPGADAVAAATAEGVRELLGHETELRDFVRGNIKARERMIIQYAIAGQLNLVVVGTDHAAEAVTGFFTKHGDGGVDITPLTGLTKRQGAALLQELGAPPSLWEKVPTADLEDDRPALPDEEALGLKYAQIDDYLEGKDVAPEVAERVETIYCNTRHKRTVPVSPLDSWWKN

>CORE_REP|Org81_Gene3876#

MSVALLSSAENRTPMRGRDSHTKDARNTAAEVPVSMIERMTLILDAFDASTPTLTLLGLVERTGLPRSTVHRILDQMIKLRWLAHTSGGYRLGMRALELGGLTADHNEIRDAVSPLLHELSQRTGMVGHLAVLDGRDVVYLDKAGGRFAASLPTRLGGRMPAHATGLGKAMLACLEPNIAEAAVRARLPRLTPRTICDAEALSRELQQIRLRQGVAIDREEAVVGIACVAAPLRGRGTAPAAISLSGRADAMSFDRLARVVLEVAHEAGRTLFPRRAHWR

>CORE_REP|Org12_Gene1025#

MTTRAAAADEGAARDGLYSGELSIIGRVTTASNLTLVCEIDAPPVDGGSEPLRVVYKPVRGERPLWDFPDGTLAGREVASYLVSAALGWSVIPETILREGPLGPGMVQRWIETVDPGAAAPERLDLVDLVPAGAVPAGFREVLRAVDGTGARVSLVHADDPRLRRMAVLDVLLNNADRKGGHALEGVDGGVYGVDHGICLHSEPKLRTVLWGWAGEPVGEELLGDIAAFADRISGPIGSALAEHITDDEIEALEARTRELLDDPVLPQPVSSRPIPWPAF

>CORE_REP|Org160_Gene3260#

MRINRALRLGVGAIALALTAATTAGCGSGDDKTALDHAKEGKLTIGIKFDQPGLGQRNTDGTYSGFDVEVARFVAAKLGVQPDGITFKEAPSAQRETLIENGQVDFIVATYSITDQRKEKVDFAGPYYVAGQSLLVNADNTDITGPETIAGKTVCSVKGSTPAQNIEKNFPDTQLQTYDTYSLCLEGLNSGAVDAMTTDDIILAGYAAQTPGRYKLVGKPFTTENYGIGLKKGDQESRDKINDAIEAMITEGAWDKAFQDSVGRAANYPTPPAPQVDRY

>CORE_REP|Org149_Gene4169#

MEWVTRVQLPKPELVASDVDGTLIDPEERVTARTKAAVGAVVADGVPFVLATGRPPRWIGPVVDGLGFAPLCVCGNGAVIYDSAADRVLTSRTLDIETLGWIADLAEEVLPGCGLAAERVGASAHDAVTPQFVSSPEYEHAWLNPDDTAVARHEVIDAPAIKMLIRLPGARSGDMLAALAPVVGDRADITYSTDHGLIELSAPGVTKASGLVTVAERLGVDPAAAIAFGDMPNDIPMLTLAGRGVAMANAHPDALAAANEVTTTNAEDGVARVLERWWV

>CORE_REP|Org105_Gene3087#

MYLLDPPGRNTGEGVGGGRYGDCMRIGAHVRLDSDPIGWGEKLGADLIQLFVVDPQSWDKPQPHPRAEQILASPIDVVVHSSYQINVASLNNRLRMPSRNAVAQQAKAAADIGAFGLVVHGGHVRSDEEIEAGIVNWRKLFERQQDKGGFAVPILIENTAGGNHAMARHFDSIARLWDAVGEFGAGFCLDTCHAWAGGEDLVGVVERIRAITGRIDLVHLNSSRDEFNSGADRHANFADGTIDPQLLAEVCRTADAPVILETPAEGVADDMAYLREHVG

>CORE_REP|Org38_Gene1209#

MAIRKYKPTTPGRRGSSVSDFAEITRSTPEKSLIRPLHSKGGRNAHGRITTRHRGGGHKRAYRLIDFRRLDKDGIPAKVAHIEYDPNRTANIALLHYVDGEKRYIIAPKGVVQGTPIESGPTADIKPGNNLPLRNIPTGTTIHNVELRPGGGAKMARSAGSSIQLLGKEGTYATLRMPSGEIRRVDVRCRATVGEVGNAEQSNINWGKAGRMRWKGRRPTVRGVVMNPVDHPHGGGEGKTSGGRHPVSPWGQPEGRTRKPNRPSDKLIVRRRKSGKNKR

>CORE_REP|Org5_Gene5353#

MTDAADATPGAPGTGNTAEPDATTAAGASARTDAAPPMISMRNVDKHFGDLHVLRDVNLEVPRGQVVIVLGPSGSGKSTLCRTINRLEPIDSGTIAVDGVELPAEGRALAKLRADVGMVFQSFNLFAHKTILDNVLLGPVKVRRVDKKRARARAMELLERVGIADQADKYPAQLSGGQQQRVAIARALAMDPKVMLFDEPTSALDPEMVNEVLDVMVALAKEGMTMLVVTHEMGFARRAGDRVLFMADGRIVEDAPPETFFTAPASERARDFLGKILSH

>CORE_REP|Org19_Gene6391#

MTSRPRPCSTRFSPRPPDRIALTLNSRRVVALVPAAGRGVRLGESTPKAFVPVGGSPMLVHAVDGLITSGVVDRIVIMAPIEMIDAARELLAARAHAASSIPVDVVAGGVERTDSVRAGLAAAPEATHILVHDAARALTPPSLIARVVGALDAGHRAVVPGIPVADTIKAVDAAGDVTGTPDRSGLRAIQTPQGFDAALLREAYAVDLPATDDAGLVEAMGATVSVVPGDPLAFKITGPLDLRLANALVADDSAAAGGASGAASAETAITAATRAAVTG

>CORE_REP|Org1_Gene5539#

MSAEGSARRGVNTGQHSRAVLVTRASPWLSSSGMDRSSRRSTRALALSALTLGGLLAATAPAAAAPTGPDVSSWQHIDGRLIDWFAVKRAGHDFAMVKATEGLGYVNPYFVPDSLLMRAAGVARGTYHYARPELPPEPQAALYAATVLGQNGPLDLPPVLDLEHSGGLAPAALIDWTHRYLNTVRALTGRVPIIYTYPTFWRTAMADTDQFTGYPLWIADYRGNAQPEVPGGWPTWTFWQTTDSGSVPGIAGPTDLNVYSGAQGDFARYANMGGLFGSS

>CORE_REP|Org19_Gene2600#

MRNPLATPTGCGCPARHRRRREQHCAARSRRRPGAGVPQDTGGVVTSAERPPAATRVLVVDDEPQILRALRINLSVRGYEVITAATGAAALRAAAEKHPDVVVLDLGLPDIDGVEVLAGIRGWSSMPVIVLSARTDSSDKVQALDTGADDYVTKPFGMDELLARLRAAVRRSASTAEESAPIVETSSFTVDLAAKKVIRGGRDVHLTPTEWGVLEMLVRNQGKLVGRRELLREVWGPTYATETHYLRVYLAQLRRKLEDDPSQPKHLLTEAGMGYRFQA

>CORE_REP|Org31_Gene3788#

MGGLLEGKTILVTGIITDSSIAFHAAAVAQEQGAKVIITGIPERLRLIDRIAKRLPQEVPPAIPLDVTSEENLAELADKLRELAPEGIDGVLHSIAFAPRTLMGPEALPFLDGPGPDAAKAFEISAWSYASLARAVLPVMNERGSIVGMDFDPRTAMPFYNWMGVAKAALESVNRYVAREVGAAKKIRSNLIAAGPIKTLAAKAIAGTATDDAAKLNQLNEYWDGASPIGWDVDDPTVVAKSIVAMLSDWLPGTTASIIYVDGGASHNTWFPEDMSIN

>CORE_REP|Org127_Gene922#

MPGKRPAPEPPEPLSPLIDAHTHLDACGAEDAESVAAMVDRAAAVGVGRVVTIADDLDAARFAVDAAHWDPRVYAAVALHPTRANALDDAARAELEKLAADPRVVAVGETGLDYYWPGKLDGCADIEDQVEGFRWHIDLAKRLGKPLMIHNREADHDVLAVLLDEGAPDTVIFHCFSSDANMALACVAEGYLLSFSGTVSFRNAHELREAAKLVPDEQILVETDAPFLTPHPFRGAPNEPYCLPYTVRALAELREQDPAALAEITTANAERVYRLQRG

>CORE_REP|Org146_Gene108#

MARSDVLVSVDWAEENLNTPGVVFVEVDEDTSAYDNGHIEGAVRLDWKNDLQDPVRRDFVNQQQFSDLLSARGISNDDEVILYGGNNNWFAAYAYWYFKLYGHNNVKLLDGGRKKWELDGRPLSTEPVNRPATQYKASAPDLSIRAFRDEVIAAIGTKNLVDVRSPDEFSGKILAPAHLPQEQSQRPGHIPGAINVPWSKAANEDGTFKSDEELTEIYKEAGLDPEKETIAYCRIGERSSHTWFVLQELLGHQNVKNYDGSWTEYGSLVGAPIELGA

>CORE_REP|Org53_Gene1383#

MERVPQPDPDLLIDFTDVTIRRSGHTLVGPVTWQVELDERWVVLGPNGAGKTSLLRIAAAETHPTSGTANLLGETLGRVDVSELRPRIGLSSAALAGRVPRDEKVLDLVVSAGYAVLGRWRERYDDMDTDRAVDMLESLGAEHLSDRTYGTLSEGERKRVLIARALMTDPELLLLDEPAAGLDLGGREELVERLGDLAADPDAPAIVLVTHHVEEIPPGFTHALLLNEGEVVAQGLLDDVLTSENLSEAFRQAIALDRIEGRWFARRARRAGRHRSR

>CORE_REP|Org27_Gene1368#

MTTQTPDPIGVRGLPVVTGTTETLRLLVPHTLIQTQRLLLRWWRDPLTLMQSLLFPALLLVMLQTVLGRQISAFSGASALYGSVPMVALVGVMSGSLAGAITLGRERDAGLLARFWVLPVHRASGLAARIVAEGVRILACTVVLFAVGVVLGFRFEQGFAAAVALLGVPLLFGLAFATVVTTVAVFGARTAVVEAISLGSSMMMFFSTGFVPLAAYPGWARPIVEYQPMSHAINAMRGLSLGGPVREPLLATLAWSVGAILVFAIPAAIGYRRASRR

>CORE_REP|Org170_Gene778#

MANPFVKAWKYMMALFDSKIEEHADPKVQIQQAIEEAQRQHQALSQQAASVIGNQRQLEMKLNRQLDEVEKLNANARQAVTLADQATAAGDTEKAIQYTNAAEAFAAQLVTAEQSVEDLKVLHDQSLQAAAQAKKAVEQNAMLLQQKVAERTKLLSQLEQAKMQEQVSASLQQMDSTLSAPGSTPSLDAVREKIERRYATALGSAELAQNSVQGRMLEVQQASIQMAGHSKLEQIRASMRGDALPSGGATPAINPAQAQADPAQPQPQMNKGQTAQQ

>CORE_REP|Org120_Gene7219#

MGAVILRRDSSTATRPNPAGSVATSTVRPPSQHPSGARPPAIPAELVPNHVALVMDGNGRWAQERGLPRTAGHERGEAVLMDTVEGCIEMGVKWLSAYAFSTENWRRSPDEVRFLMGFNRDVIRRRRDEMNEMGVRVRWAGRRPRLWRSVINELEIAEEMTKHNTVMTLTMCVNYGGRAEIADAAREIARRVAAGEIDPEKVTEATVARFLDEPDMPDVDLFLRPSGEFRSSNFLIWQSAYAEFVYQDTLFPDFDRRNLWAACLEYASRDRRFGGTK

>CORE_REP|Org112_Gene4523#

MTTASAATGSNTDGTEEPHALVEQRGATLIVTMNRPRSKNALTGEMLSIMAEAWQRVDSDPEIRSCILTGAGGAFCAGADLKNMARSNPGDNMTAGSSFDPTRMPGLLKGYRLSKPLIAAVEGPAIAGGTEILQGTDIRIAGASAKFGVSEAKWSLFPMGGSAVRLPRQIPYTLAAEILLTGRHITAAEAKEFGLIGHVVPDGTALDKALEIAELVNNNGPLAVQAILKVMRDTEGMHEEEAFQIDAKVGLPVFRSEDAKEGPRAFAEKRKPNFQGR

>CORE_REP|Org216_Gene2382#

MSIDVPENEVGAEPRNRAAAKPKPTAKFRGPIMFRRDRKPGVRTADRNLLDTRADGDWVHTDPWRVLRIQAEFVEGFGALAELPHAVTVFGSARTPVDHPEYEAGYAIGAALARAGFAVITGGGPGAMEAANRGASEAGGYSIGLGIELPFEQSLNDWVDLGINFRYFFVRKTMFVKYSEAFVCLPGGFGTLDELFEALTLVQTGKITRFPIILFGSSYWAGLVDWMRGSLEGFGKISPGDIDLLHVTDHVDEVVDIIARAAEQRAELDTYGTEDQW

>CORE_REP|Org4_Gene5772#

MTGAAQWARGGMSIGSVLDLLRPDFPDVTISKIRFLEAEGLIRPERTPSGYRRFSVADCERLRFVLTAQRDQYLPLKVIKEQLEAIDSGAASLGVREARARAHSGRAGAAEPTATGSASGHPAASGGNSNGAAAPRRLGVVPSEISPDDLRFDHEIRLTRADLLAKAEIDDAFLNDLIRANLITPGAAGFFDGDAVTLAKTAKAMAEFGLEARHLRAFKLAADREAALVAQIAAPIAKSRDAGARARAEETVRELAALSLTLHACLVKSSVRTSLGG

>CORE_REP|Org42_Gene423#

MTVHSSSADRVLVRKQGPVTIVSINRPEVRNAVDRATAEDLAAAFREFDRDPDAAVAILTGEGGTFCAGADLKAVAAGDPNRFASDGDAPMGVSRMRLSKPVIAALSGHAVAGGLELALWADLRVADENTVLGVFCRRWGVPLIDGGTVRLPRVIGLGHAMDLILTGRPVSAHEAQGMGLVNRVAPAGRSLATAVQLAEQLAAFPQTCMRQDRLSALEQEGLGETEALANEYEHGVVSISTDTLAGATRFAGGAGRHGSFTDLGNPEDAPERSTQS

>CORE_REP|Org129_Gene1057#

MSSELQVDVSAGVAVLTLNRPAQQNAMTPTMAVELGTALRRCDTEDAIRAVVITGTPPAFCAGADLSARVGDVRGTIDPPPWQIRKPVIAAVNGHAVGIGLSLALQCDLRYMATDAVYGLNQVRRGAMADGYAHWTLPRLAGMANAADIMLTGRTFDGEEARQMGVANSSLPAGEVLPTALAVAHDLAAGSAPLPTALTKRLLWEGLGMSPEAVGRLESELHGFVGKSVDAAEGMAAFRDRRQPQWKGSISAEWPAGELSSPGERRGLDGTAEEPA

>CORE_REP|Org37_Gene6251#

MHVSETPGTETGTEITETGGTETPFVRYEVRDGFAVLTLDSPHNRNALSSKLVRELLDGLRKAGADEQARGVILTHTGNTFCAGADLKEALDADPAAAADIRTGWMIDVLRGIVELHKPVVAQVDGNVRAGGMGIVGACDIAVAGPSSSFALTEARLGLAPFMISLTLLPRLTSRAAARYYQTGETFDAAEAERIGLITVAAADAAAEVARLCGELRKGSPQGLAESKRLVNASIVAEFDRTADELAKRSGSFFGTPEVIEGMTAFFQRRPPSWAE

>CORE_REP|Org102_Gene2724#

MVASSMVPRTTGGAVRARPVGWLIVESTVTRVAFEDVLIAYRDSGAERAANTVPVVLVHGMGGDGHTWDRFARQLARRGRRVIVPDLRGHGRSAHADSYLFAEFGGDLLRLCDRLGLESADFVGHSLGGYAVSWLAMRRPELVRRLVIEEQPLPLRSGDEQVTLTRRLPSVPELWHATTSLLRHPRAVLAFDRSMTRTALEQFRKPYPEWWEGLADIAAPTLFLRGGPGGMVDPDKVEQLRVSIPDCTVHTFRCGHSIHRDRYHEFEAAVLPFLRH

>CORE_REP|Org109_Gene5232#

MNASQSDSVGGDGRATGGREFETILLERKGRVGWITLNRPKALNALNAQVLDDVIAALDELEHDDEIGVIVITGSERAFAAGADIKEMQPKSYMDMFMDDFFARWDRLAQFRKPTIAAVAGYALGGGCELAMICDILLAADTAKFGQPEIKLGVIPGIGGSQRLTRAIGKAKAMDLVLTGRNMDAEEAERAGLVSRIVPAAQLLDTALEVAETIASMSLPVAMIAKEAVNRSFETTLAEGLRFERRVFHSLFAIEDQKEGMSAFVEKRPAKFTNR

>CORE_REP|Org215_Gene1178#

MSFRRVIIGLLCLAGLAVVLDFGTAAYSEYRVSRLLREGSDLSADPEVTFRGFPFVAQAVDGTYDDIYIRARARRPDIPGEIQIESNLYGVRLPLSDLADGRVRMVPVDEVQASMHIEPIELGRLFRIPDLQVFGPPADKSDGSGGSGGTGMTTSGAIILTGTVPPRVDTPPGSSSTEKGQLVSVLADLRLDADGQIQIVATEIYHGEEATATPVAVLPAVPTEMILGWFTRTIDTRDLPFGIRPTKVEAVGGQIVVEGRGRDVTIDLDHLQRQQ

>CORE_REP|Org74_Gene3418#
[truncated: 113,678 more chars]
