# Supplementary material for: Design of a multi-epitope vaccine against six Nocardia species based on reverse vaccinology combined with immunoinformatics
Source: Front Immunol. 2023 Feb 2;14:1100188. doi: 10.3389/fimmu.2023.1100188 (PMC9952739; doi:10.3389/fimmu.2023.1100188)
Supplement: Supplementary file 10 [file Table_3.docx]

>CORE_REP|Org119_Gene7073#

MSSLATDSVDQCSSGESSAQPFVLSPAQTALWYAQRIRPDVPLTIAQYVEIHGDLDVGRLLYAIERFGAESEVGKLRLAEIDGIPHQIVDPARRPGWARVDLRGERDPHAAALRWMHEYTGSPIDLERDPLTANVVLRTGDSDYIWYSRAHHIVIDGYGAMNALTRTAEIYTALENRTEPVVSRAAPLAEIYADEVRYRETSRFRADRDYWLEQLAGAGEPMSLGGSTVTAATQDAGRRIAAGVLDDRAQAAMDAAVTTFGTANSALFVAALGAYVRSVTGNPDVVLSLPVSARTTVSLRRSAGVVSNVVPIRLRFGAETTLAEVVKATELQITGALRHQRYRHDDIRRDCGYSRDARGFFGPMVNIMLFHDELTFGSLVGSLNVLATGPVEDLSVNLYNGVGGRIHVDFEANPRLYGEAEVSVHHDRFLDFLTRFLGAAPDTHAETLTAITAAEHERVLHEWNATEAPRQPGTLAELFAERAAACPDAIALESGDDTADPSPVHPVTTLTYRELDERANRLARLLIERGAGPETVVGLCLRRSIDLVVGMYAIVKTGAAYLPLDPEHPADRLDQIVRQASPVCVLTAARDELAMPESAAALAIDTVELSGYRGAPITDAERTAALRADHLAYVIFTSGSTGKPKGVGVSHAAIVNRLRWMQHEYSLDRTDVVLQKTPATFDVSVWEFFWPLQIGARLVVAAHDGHRDPAYLARLIAEKGITTAHFVPSMLSVFVTDTDVRGCTALRQVFCSGEALPAATVRDFHAALPRPALHNLYGPTEAAVDVTYWPCPADPATVPIGSPVWNTQTYVLDSRLRPVPPGVVGELYLAGVQLARGYLGQPRLTADRFVANPFGAGVRMYRTGDLARWQLGTDRPGVLEYMGRSDFQVKIRGLRIELGEIEAALLDDARVARAVCVAHPGRNGDELVAYVVATPAAGRLDTTALLTELRRTLPAYMVPSALLELDELPLSANGKIDRKALPAPVGVRATGRSTAEPRTEVERVLARVFAEMLGTEVGVEDSFFDLGGNSLVAARAVARINAALGTGLTIRDLFEASTIAALTQRFATHPADVSSPKLVAAQRPERIPLSLAQQRLWILNRFAEHAAAYNMPLAVRIEGALDVEALRAGLVDVIERHESLRTTFPESAEGAVQLVHPAAEIPLTLDPIDAAGADVAELATEFAGYGFDLRSQAPIRVALYRTGPDQWVFLVVLHHICGDGWSIAPLARDLMTAVAARGAGAAPQWAPLPVQYADFALWQRELLGNESDPASALSGQLTHWRSALAGLPDQLDLPLDRPRPLRRSTTGGRVDFTISPEIRRAASELAAARGVSMFMVLHAALATLLSRLCASTDIAIGTPIAGRSDPALDELVGMFVNTLVLRTEIDPAAGFDRMLDVVRETDLNAFANADVPFERLVEVVNPERSAARHPLFQVMLSYDRDPDLRIELPGVRAEVLPIVSDIAKFDLQLVVHDDVTDGPLTAEFGYATDIFDRATVESFARRFVAVLNAVVAAPSMPIGDLSILDRREIANLVPIAGAPAEPFTTLARLLTDTAERVPDAVAVRYLGVDTTYRELDESSNRLARVLIEHGAGPEVVVAIALPRGLDAITAVWAVAKTGAAYVPIDPSYPGERIAHMIGDSGAILGLTDAACLAAMPEWPAPRGKHRKNYVDWLVLGSAELAAEAVHCSTAPITDADRHHSLCTVHPAYLIYTSGSTGKPKAVVVTHAGLASLANEQTHLFGVTDSARTLHFSSPSFDASVLELLLGFAAGATIVVAPAGMYGGAELATLLRTERVTHAFVTPAALATVPTDGLDELEAVIVGGEACSEELVETWSAEHRMHNMYGPSEATVAATATGPMVPGRPVPLGQPIRGMRLFVLDGRLHPVPPGTPGELYLSGPGLARGYHGRYGLTAQRFLANPHGRRGERMYRTGDLVVVETGGQVRFLGRADDQIKIRGFRIELREIDHVLRAHPGVNFALTVVHTDEHGQPRLASYVTVDHPVAAADLTETARQRLPGYMVPASVTVLAELPVTPAGKLDRKALPEPVFATGGSSRAPATELESRVAGVFGEILGRPVTGAEDSFFDVGGNSLLATRLAAALHAEFGVDLPVRVIFEAPTVAGVAERLTEAPRTQRLALAVQTTRPGRIPLSLPQQRLWFLNRYSPESSAYNIAFVIRIAGDLDVAALRAALTDLVERHEVLRTVFPEDSAGAQQVVLPTARALPAIEAIDTDEAGATAALGALAHRGFDLIRDTPLRMTLLRTGSERYLLGIVVHHIAADGWSLGPLTRDLAAAYVARHGGAAPAWTPLPVQYADFGLWQRACLGDEGEPGSLAAEQLAYWRSALADLPAELPLPYDRPRPAEPTQYAGAVPFTVPDPVQRALAELAKEQGVSMFMVLRSALAVLLRSVTGGRDIVIGTPVAGRTDTKLDELVGMFVNTLVLRSDVDPDRPFAGLLRADRDTELAAMAHADIPFERVVEELASGTTRGRHPLFQVALTVQDGPVPTLELPGLELRAEELDIALAKFDLELRVAHIGCDAGPGEPGRAFEFVYAAELFDEATIHTLADRFLRVLAAVTADPRVLVRDIDTRTERERRLLAPATGGPTTPQCTLAAYFTATAHMHPHRTAVRSGATTLTYAELDKRSNRLARALLARDIGIGDRVALGLTRSVESVLTVLAVVKTGAAFVPVDPNYPADRVRHMLADAGCWVGVTVGAHAERLRTAAADGPATDWLLLDDPAVRAELETYDDALVDDLDRMCTIEAADLAYLIYTSGSTGKPKGVAVTHAGLSNFADELRDRMRVDRESRTLHFASPSFDAAVLDLLLAVGSGAAMVLCPPDVYGGDELAALLERERITHTFMTPAALATIDHERWPLPHLRALMVGGEACAPDLVARWAPGRTMLNGYGPTETTIVATIATLTAEQPVTIGTLVRGARALVLDERLRPVPAGVPGDLYLGGHGVARGYFDRFGLTALRFVADPFGPAGARLYRTGDVVRWNDAGELCYLGRSDHQVKVRGFRIELGEITAALGEHPAVRFAHTEVRQIAGADRIVAFVQPADEHTGVDVEAVRDRLGAQLPAHMVPASITVLERIPLTPVGKLDSAALPEPQLAVAAATREPSTPSERLVARVMGELVGVDAVRADDSFFDIGGNSLLATQLVARLAAASNTRLEVRTVFAAPRVAELAAHLDSGPAGARSRPALVRQARPDRIPLSAAQRRLWFLNRFNGIGEAAADGADLSAGAYNVPVVLRMNGKLNVDALVVALHAVQDRHETLRTVFPEVGGEPTQRVLDLVTAAITLFVATVRPDEVDDAVRRFAAPGFDLAGVVPMRAALISVSPDGDRGVRNPAEVSDEHVLVLVVHHIAMDGQSLAPLALDVATAYRAACADRSPEWDELAVQYVDYTLWQQDTLGTEDDPDSVIRRQLDYWRHQLDGVPELLTLPADRRRPPVPSYRGGLVECEIDAFTHRDLHRVATSNNVSMFMVLHAALAVLLHRMSATDDITVGTPIAGRGHPALDRLIGMFVNTLVLRTRIDPDARFTDLLHTVRDVDLDAFAHADLPFERLVEVLNPARSQAHHPMFQVMLSVQNHPVGGLELPGLRIEAADVDTGIAKFDLQFTLTEAQTPERDPAGITLSVNYASDLFDEQTALRLGHRLARLLAAVAANPTTAVGDLELLDPAEWSGLAPVRGAEPDRPVTFPEVFAAAAAVDRAAIALRADGTQISYDALDRWTNRLARVLMRRGVGPETLVALGIPRSVESVATVLAVAKAGAAFVPVDPNYPAPRIAHMLSDSGAALGITLSAHRDELPGDVEWIVLDDPIFRGLVLDSPDGPIAAAERTAPLRIDNPAYVIYTSGSTGTPKGVVVTHGGLSNFAAETAQRFDVRPGCRVLHFATPSFDAAMLDLLLALGGAATLVITPPGVVGGEDLARVFIDEAITHAFITTSALGTVDPTGVTALRHVLVGGEALPPDLVTRWAPNRNLYNVYGPTETTIVTVISQPMTPGGPITIGGPIRGVSATILDGRLHPAPVGVTGELHLAGSALARGYLNRPGLTAQKFVANPFGKPGERMYRTGDLVRWWTGQGSPAAGRDHGGSREIEYVGRTDHQVKIRGFRIELGEIDAALAKHGGVEFATTIGHRTPAGSTALVSYVKARNGIGLTAAELTEHVAGLVPNYMVPQSIMLLDRVPLSPVGKLDRKALPEPVFSAADGYRAPATPTEVALCAAFAAVLGVETVGADDGFFELGGNSLLATKVVAQVRANGLDLPVQAMFGEATPAAIAARLDGSGAGIVAALGPVLPIRPNGKAAPLFCVHPAIGLAWCYSGLLAHLAPDRPVYGLQAPHVAGEDGFASIAEAAQQYVAHIKSIQPTGPYHLLGWSLGGLIAHEVAVQLQEAGDEVALLSMMDSYRLSDAWLEHAIPSVAEIIEEFGSDQLDAPLDPAMNLRDAAELLRARPGPFAALTVEHLERLYAGYTNGTLLAHGFRPRVFDGDLLFFTAAADEINRADPERTAAAWQPFVTGAIRDHELPCRHSAMTAPESLAAIGQVLRGALDGAAVLLPAGAQPAKNGVRRTKSGARKEKQR

>CORE_REP|Org15_Gene5536#

MADNEGTQTTETTGDANAEVEQQTTATEQSVTEPGGTAKSGGTDMSVAELREWLQRWVADATGQPVEQITVDRPMEEFGLASRDAIALGGDIEELTGVLLNPTIVYQHPTIAALAERVINGEPEAPEEAADDAFYTAGYQPGAAHDIAIVGLSTRLPGAGDTPESTWDFLINRGDGIRELPEGRWSEFLADPDIAAAVENGNTLGGYLDQDAIKGFDAEFFAMSPVEVERVDPQQRLMMELTWEALEHARIPANTLKGESVGVFIGTSTNDFQLVASLGLGKSDPDAPASADAYALTGGSTAIIANRVSYFYDFRGPSVAVDTACSSTLVAVHDAVRALRNGDADVALAGGVNMLLAPAITLGFDSIGAVAKDGHIKAFSSDADGMVRSEGAGMVVLKRLADAERDGDRILAVVKGTAVNSDGRSNGLPAPNPEAQVDVLRRAYRDAGIAPSTVDYIEAHGTGTPIGDPIEADALGRVVGRGREDDKPVLLGSAKTNFGHLESGAGAAALAKVILALQHNVIPPNIGYAGPSPFIPFDQAHLKVVDEPTEFPRYSGTATIGVSGFGFGGTNAHVVIQEYVPAASVESKEEAAQIASAETIEAELDNEATDVLAGAEAILEGSDPLAEPEPVAEVAEWTQERTEPLPVILPVSAYLPSRRRRAASDLADWLESEAGQAAPLEDVARSLAKRNHGRSRGVVLAKTHEEAVAGLRAIAAGKPGPGVFTADSPAAQGSMWVLAGFGSHHRKMGKQLYLENSIFARTVDEIDELVVDEAGYSVKEMILDDAQDWDVGTSQVGVFAIQLGLAALLRAHGAEPAGVVGHSQGEAAGAYISGGLPLEDAVRVICARSRLMGEGEQMITDDQVRNMALVEYSAEDIEKVLPEYPDLEVAVYAAPTNTVIGGPPDQVHAIVARAEAEGKFARVLQTRGAGHTSQMDPLLGELAAELAGIEPTKVTTDLYSTVHKATVYKAGSDPVHDVDYWVTNMRGSVYFTNAIRRAVDAGITTYLELAPNSVALMQVMGTTFAAGVHNAALIPTLKRKEDEAAGVISALAQLYVQGHPVDLVSLLPAGDYADVPRTAFLRKEYWPKVSIATGSGSGRAPGAHVALPDGRHAWEVAASAVTDLAGLVNAAAAQVLSEVALGATIAHSPLPASGTLTTTLTPHPGGASVQVHVREDNVFRLLFDAVVTASAPSTNGTSAPAVQPAPAPAETDSGTADLVVAESFGERWDPNGTQTVEERLATIAAESMGYAVEDLPMEIPLMELGLDSLMAMRIKNRVEYEFDIPSLQVSAVRDASLNEVGKVLRYAIEHRDEVAAMAEKQATEGGSLTVDDNFVAAARAAMEAGEDPAAAVTQQVEAAEPKPVESATPAGSADAVAEAADVDQKASATAGSASQAAEPKVEAADAKDGAADAKTGSGKGTAPAQAAAVFGGGQVAGAKEPEADVPPRDAAERLTFAAWAMVTGKSAGGIFNTLPILDEDTADKLAARLSDRVGSTIDVDDVLDCETIEQLSDIVRRHQDSATEVEGFIRPLRPRPEGSTAIPVFVFHPSGGNTLVYEPLLKRLPEGTPMYGFERIEGSIPERAREYAAEIRKIFPSGPYALYGWSLGAVFALQVAQIMRAEGDDVRLVGLIDLALPVEDEDPSPEGRRARIERFQAFAQKTYGIEGQLDDEMLQELADASDEEQLEIIMGLLKFADVKIPGGVMEHQRTSWLDSRDLQKAQPSHYEGDVTLYLADRYHDGMIELEPRFAERKPNGGWDDYIPNLEVIHIPGDHLQIIDEPRVAQIGADLTRKLAAISTEADDAPGKGEQ

>CORE_REP|Org214_Gene5614#

MTVSSELSSAAWAAGLPQALRGELATLEEAYFRHVDAGDVDCAITGITQIFRRHMELAMHRPAGRALVRVYHPDDSSGLGAAVQVVTDDMSLLVESVTASLSRLGVSVSEVIHPIFEVERDADGRLEQAAPHEVDGNGTAGLRESWMHLQLHPATSRAQLSRIESGLPNVIADVRQVIGDTEAIKDVQSKLAEDLELAAKSGKAPFSDTDLIDTANLLRWLASGNFTVLGYARYRLSSDQEQQSSNALPGTCLGVLRPDVGTDFQVPINALDRPLLILTQGLVPATVHRSVYPYFIGVADFDESGTIVGEHLFIGVFTVTAVHENVLDIPVIERRVRSVIEESGFDLDSFSGQAMLEVIQSFPRTELFSSDDDTMRKTAVAVLNIGLRRQVRLFLRADGYGRFVACMVYLPRDRYTTRVRLEMQEILVRELGGVDIDYSARVAESDLASVYFTVRMPAAEHGAPRYAAAPAADTSEANRLRIQGLLAEASRTWEDHLNDEVSTSTMLDPAVVQRYAVAFPDAYKEDFEADRALADIVRLEHLRDGGIDQYLYRNAGSDPGSWRFSLYVGGAGISLSQVLPVLQSLGVEVVDERPYQLELEPQPGGDPSSGGERWIYDFGLLARPELLRSALDRDLDAELLESSKRAAVLEAEVRGLRERFTEAFEAAWYGRAEADGLNELVLRARLPWRAVSILRAYAKYLQQAGFPYSQANISRVLLTYPDVARLFVDLFGARFDPDTVSAEHAAELETQVRGRIDEVVSLDADRILRAILNLIRATLRTNYYVTDAEGMPRDYLSMKVEPREISELPKPRPQFEIFVYSPRVEGVHLRFGPVARGGLRWSDRLEDFRTEVLGLVKAQAVKNAVIVPVGAKGGFVVKQPPAATGDPVADRQALGAEGVACYRTFISGLLDVTDNVDRATGKVLPPARVVRRDGDDTYLVVAADKGTATFSDIANDVAKRYGFWLGDAFASGGSAGYDHKAMGITAKGAWESVKRHFLEMDIDTQTEDFTVVGIGDMSGDVFGNGMLLSEHIRLLAAFDHRHIFLDPNPDTAASYAERQRMFQLPRSSWADYDAKLISAGGGVWDRTVKSVPISPQARKALGLGDDVESLSPPELVRAILLAPAQLLWNGGIGTYIKSSTESNADVGDKSNDPVRVNGNQLRVKVIGEGGNLGATALGRIEFCRNGGKMNTDALDNSAGVDCSDHEVNIKVLLDGVVSSGELAEPDRNPLLASMTDEVAQIVLEDNVAQNFLMGISRTDAPQMLNVHMRLIDDLEERRGLDRELEALPSEAQMQRMLEEGVGLTSPELANLMAHVKLSLKADLLQTDLPDSAYFTTRLPDYFPTPLRDRFGAAIKKHRLRREILTTMIVNEMVDYGGITYAHRLSEETGATATDSVRAFAAATEIFGLPEMWARIRAADATTSVRDLLELETKRTLDRASRWFLSNRPQPIAVGAEINRYCRDVQELAPKVPGWLRGHHVSTLTDQSAELIARGAPTDLATEVFGLLNLFPLLDIVDIADITDRDGDEVGALYYALNEHLKIDWLLQAVSHLERGDRWHSLARLALRDDMYASLRSLTLDVLSAGDPEESADEKIAYWESKNQSRLGRARAALSELFESGTHDLATLSVAARQVRSMVSGVGAQSEVAAR

>CORE_REP|Org108_Gene3171#

MARQARAEITRDSVLAGAADVFLRLGYANASLSEIIAQSNVTKGALYFHFGSKEELARAVVDQGNERLVSSCQGFFDPRVPALEACIGITYVVADLSMNDPMVGAMLKLTHQIGDYRGAAGDNIAKSWGDTYRLLAERAIAQGDLMPDLDPETIGLLLHGVTTGVHIVAVGTEAIDQMATRMERAWYFLLPAIVPPGEALLLPRVRRPSPAPLRAVILRTGAQPGGPQGGREHAATEERQNGRAGRFARSLQELSMSAPRPPLAGLAAAAGADIALRTVSDLVGTSQVELVAPAAARAFVAGTVATSKPVVVVTATGREADDLTVELTEILGEAVAQFPSWETLPHERLSPGADTVGRRLAVLRRLAHPEDPVFPEPLRVVVTTVRSLMQPMASGLGDIEPIVLRVGTETDFDELLTRLVEFAYTRVDMVGKRGEFAVRGGILDVFPPTADHPVRVEMWGDEVSELRPFSVADQRSLPELSIDVVVAPPCRELLLTEAVRDRAAEVAAANSADAALVEMLEKLAQGIPVDGMEALLPVLRPGALSLLTEELPEGTHLLLCDPEKIRTRAADLMRTGAEFLEASWTAASFGGDAPLGAHGLDLAASAYRSLPEIHDSATEHDLPWWTLSPLTSGDSSEVVLPVQAAPAARGSDELVATIFASLRAHVTTGGRAVVVVAGHGTAQRVLERLADADVPAAALDPGAEPETGVVGVLCGSLHDGVVFEDAGLVVVAESDLTGNRVTAPTEGKRLPAKRRNQVDPLALNAGDMVVHDQHGIGRFVEMIERTVGGARREYLVIEYAPGKRGQPGDRLFVPMESLDQLSRYVGGEMPSLSKLGGSDWANTKRKARKAVREIAGELVQLYAARQAAPGHAFGPDTPWQQEMEDAFAFTETVDQMTAITDVKADMEKPVPMDRVVCGDVGYGKTEIAVRAAFKAVQDGKQVVVLVPTTLLAQQHLQTFTERVAGFPVTVKGLSRFTDPAESREVLEGMASGEVDIVVGTHRLLQTGVRWKDLGLVIVDEEQRFGVEHKEHIKALRTHVDVLTMSATPIPRTLEMSLAGIREMSTILTPPEERHPVLTYVGAYSDKQVTAAIRRELLRDGQVFYVHNRVSSIDKAAKRIRDLVPEARVVVAHGQMNEDTLESTVQGFWQREFDVLVCTTIIETGLDISNANTLIVERADTLGLSQLHQLRGRVGRSRERGYAYFLYPPEKPLTETAYDRLATIAQNSDLGAGMAVAMKDLEIRGAGNVLGAEQSGHVAGVGFDLYVRLVGEAVEAYRAAADGKPITTEETKEVRIDLPVDAHIPPDYITSDRLRLEAYRKLAAAHDDSTLAAVVEELVDRYGPLPVEVGRLVSVAKLRLLAREYGVTEIAVTGTTVKISPLNLPDSKQLRLKRIYPSATYKAASGVVGVPLPRVQDSVGADRLRDVPLLQYLADLLLALDGKAQGAVDLTVATEVSVAR

>CORE_REP|Org42_Gene3301#

MGGAVSGGGVKVGEEGRAAARGELRAAGGVGVVGGERGSDDDGAVQARLDGRLVPAAVGCWVVTVLVLGAGWRVGVMVAVGAAVAAIGLWVGLMWAVAHRRERWRAVAVVALGAVLLGAGFAVAAAWREHRVQTHPLRAVQAGMSVRVVVTPVDDPKPVRGASFGGERTWLVRASLREYQHDSTVVRGGGAVVILATGSAWAKLPPGQPVEFRARPSPPRLRDLTVVTLRALGDPSLAGPLPWWQRLAGSVRADLVAASAAALPAGAAGSLPALVVGDTSALSDEVRRDFETAGLQHLTVVSGANFTILLTVVLFLTRVLTLGPRTTVCVAAGALVMFVVIARPDPSVLRAAAMGGVTLLALLTGRRRQALPALCAAVIGLLAVWPELAMSAGFALSVLATGALILLAPSWSDWLRAKGMWRLPAEILAVSAAAFVVTTPIVVALTGKVSLVAVVANVLVAPVIAPITVIGAAGAVLATAWMPLAELALRCAAPPMWWLLAVAEYCAAVPGATVTVPAGSTGGLIACAVVAAGIWLLRSAIVRRLLAAVLISAVAVLIPVRLWFPGWPPDGWVLAACDVGQGDGLALSAGPGSAVVVDVGPDPRTIRTCLNRLGITRIPLLVLTHPHADHIAGLDGALDGRDIGAVAVGPGELPGYSTDSAAVRQEPGSPLSPQPAPPTHCGSPAGLPERSGTAAYSPPPPSNTADWPPAGLPGVGMRPALGAEPGSQPTTATSASGADRHPPVVDRTDTGPAELAKTVHRVGIPVVELTAGCRLTIGDLTLDVLAPQAPRSRRTAALDLDTANDRSIVLAAHTAAGRILLTGDIEAATQRSLLASGAPIRADILKVPHHGSRTTTTEFLRAVHPRLALISAGATNTFGHPHPAILADLEALGTTIARTDRDGIITVRPNGPALEVRTTGPASAPRSRRPPRRRLPARRERDARPAPGAHQGGRAPPASASVCQPGGAALSPADRPQREVVLDRRRRSCRGSRGRALPTARHRSGFHLVDRHQPSRPGAPIASGGVRAQPGRPAPPIPEPPNAHQSLSDHDRRIGGVSERPAAVHLVLGDEELLIERAIASVTAQVRAGAPDPDGVPVDRLRAGEASTAELAELLSPSLFAEDRVIILESAAEAGKDAVAVITEAAADPPDGVVLMVVHSGGGRAKALAPALHKAGAVVHNCAKLSKASERAEFVRAEFRAAGARVSGEVVQAVIEAVGSELRELAAAASQLAADTGGKIDVAAVRRYYSGKAEVTGFDVAELAVTGDRPGAMEALRWANDRGVPHVLLADALADSVHTIAKVGSAGRGDPFKLAGPLGMPPWKVKKAQAQSRGWTPATIGSALQVVATLNADVKGGAADSAFALEHALMQILDLHGR

>CORE_REP|Org107_Gene6262#

MATEGFVRRPRIAPPRAPGGEVALTPPPEVTRALPAPLMMKLMPVVMVVAVIGMIAMMAMMGRNLLANPLSMMFPMMMLMSMVGMMAGFRGGTGKRAVELNEERKDYFRYLDQVRKDVRRTGNKQLETLVWSHPEPADLPSLIGTRRMWERRPNDPDFGHVRVGMGSHRLATKLARPETGPLEDLEPVSTVALRRFVRTHSVVHGLPTAVSLRAFPAINISGSPEDSRMLVRSMLMELVTFHGPDHLAVAIVCADPDGAWGWAKWLPHLQHPTQRDGMGSARMMYTSLGELETALAAELMERGRFMRNPQPTQGRLHLVVIIDDGYVNGNERLISESGLDSVTVLDLTAPEGGLAARRGLQLIASDGDVSARSAAGVEKFATADMVSPAEAEAFSRTLSRYRLATAAQIVSLGEGSTADPGLMALLKIPDAAQIDPARVWRPRTARERLRVPIGITPDGTPVEIDIKESAENGMGPHGLCIGATGSGKSEFLRTLVLSLVTTHSPDALNLVLVDFKGGATFLGLDSLPHVAAVITNLEEELSLVDRMKDALAGEMNRRQELLRSAGNYANVTDYEKARAAGVPLDPLPALFVVVDEFSELLSQKPDFAELFVMIGRLGRSLHVHLLLASQRLEENKLRGLESHLSYRIGLRTFSANESRAVLGITDAYHLPSVPGAGYLKSDASDPLRFNASYVSGPYVAPQGTVTGEDGTPVGGQRLALFTAAPVEMPAPPEEEEASPLDLPPSPTNPMLELPPPPSALGLPGAPGSDEGIPDSLLDVVVKRLTGHGRPAHEVWLPPLDESPTVDMLLPDPDWRSPVNRHGQLWMPIGVIDKPYEQRRDVLTISLAGAQGNVAVVGGPQSGKSTTLRAIIMAAAATHTPQHVQFYCLDFGGGSMAGLVGLPHVGSVAGRLDSDRVRRTIAELTSLMRQREERFAELGIESMAEFRRRKFAAAAHVPEGAASSGNPLADDRFGDVFLVIDGWAVIREEFDVLESQINAIAAQGLSYGIHVIIGASRWAEIRPVVKDQIGTRLELRLGDPTDSEMGRRTAFQVPVGRPGRGLTPEQLHMLIALPRLDSDSDPSTLADGVSRARQELAELHAGRHAPEVRMLPMQFSRDELLATTRAQGIELSPTKVVVGLGESELQPLVLDFQTEPHFMAFADVESGKTTLLRNIVMGVVENSDPEQAKIIMIDYRRTMLGVVEGEHLAGYSTSSQTCGPMIQEVAEFLSKRIPGSDITPQQLRDRSWWEGPEIYIVVDDYDMVATGGINPFAPLIEYMPQARDIGMHFVVTRRMGGVSRALYDPIIGGLKNMSVDTLIMSGSRDEGKIIGEIRPSKLPPGRGTLASRSKGQEMVQIAYLPPV

>CORE_REP|Org24_Gene2063#

MSAEPCRARHPKEGRTVILLLSTSDTDLLSARASGAEYRLANPARLLPEDLPALLAGADLVIVRILGGVRAWEEGLETLRASGVPLVALGGEIAPDAELMEQSTVPGGVAADAHNYLAAGGPLNLRQLHNFLSDTVLLTGHGFEPPVEMPRWGELERTARPVAADAPTVAVVYYRAQQLAGNTAYVDALCTAIEAAGARPLPLYCASLRAAEPELLARLREADALVVTVLAAGGTKPATASAGGEDEAWDVGALADLDVPILQGLCLTSSRAQWEDNDDGLSPLDVATQVAVPEFDGRIITVPFSFKEFDADGLSAYVPDPERAARVAGIAVRYARLRHIPNADKRVVLMLSAYPTKHARIGNAVGLDTPASAIRLLTEMRAAGYDLGAPGEIPGLEQGDGDALIHALIAAGGQDPDWLTAEQLEGNPIRIGADTYTAWFDTLPDDLRENVIEAWGPPPGELYVDRSADPKGEIVIAALRFGNVVLIVQPPRGFGENPVAIYHDPDLPPSHHYLAAYRWLAAPEGFAADAMVHLGKHGNLEWLPGKTLGMSASCGTDAALGDLPLIYPFLVNDPGEGTQAKRRAHATLVDHLIPPMARAETYGDISRLEQLLDEHANISALDPAKLPAIRQQIWTLMRAAKMDHDLGLEERPDEDVFDDMLLHVDGWLCEIKDVQIRDGLHVLGQAPAGETEVDLVLAMLRARQLWGGEVSVPGLREALGLSESGDESRNRVDSFEARARALVAALQAADWSVDAIDAIVDAQITAGGTGDNAGATPALADTAASGDAAAPGAALAGDTEKRGDSVAAEAGRSAAGGTTVVPGNAVAPGGAAASAGSTAPAVGPDAVRAVLRFAATEVVPRLRQTEVEIQRVLHALAGGFIPAGPSGSPLRGLINVLPTGRNFYSVDPKAVPSRLAWETGQAMADSLLERYLADHGEYPRSVGLSVWGTSAMRTAGDDIAEVLALLGVRPVWDEASRRVTTLEPIALDELGRPRIDVTVRISGFFRDAFPHVLALLDDAVRLVAGLDEPAESNYVRAHAQSDLAAHGDERRATTRIFGSKPGTYGAGLLQLIDSKSWRTDDDLAQVYTTWGGYAYGRDLDGAPAADDMRSAYRRIAVAAKNTDTREHDIADSDDYFQYHGGMVAAVRALTGKNPEAYIGDSTRPDSVRTRTLSEETTRVFRARVVNPRWLDAMRRHGYKGAFEMAATVDYLFGYDATTNVVADWMYEKLADSYVFDEVNRKFMEQSNPWALHGIAERLLEAAERNLWERPEDETLARLKQIYLETEGELE

>CORE_REP|Org134_Gene4052#

MDPRARSARAGHEAGSAVSDTASWRPELASDSVEPAADAEEAGTEPAQPGTDSAPTGGPAVDAAPGPGTSTGPDASAGSDTTTGPGAAARPATSGDGAAPSADHPEELDTQENNPIAIEDELEEMGLEGAREIGHGGFGVVYRCVQRALDRVVAVKVLSSDIDAESRERFLREEHAMGRLSGHPNIVDILQVDVTATGRPFIVMPYATRGSLEVVVRDNGPLGWSDTLRAGVKLAGAIESAHRAGILHRDVKPANILLSSYGEPQLTDFGIARVPGGFRTSSSMITGSPAFTAPEVLKGDEPTVRSDVYGLGATLFALLTGHAAFERQAGEKVVAQFLRITTQPVPDLREQDIPADVAAAIEQAMAQNPRDRPASAYEFGEMLRAIQRTHGQMADEMALLDTEDEAEAAAAPSGNRTGPAVTARRSWPLNLSPPTPRPGYDPAPTTTFPPTAATKFRPPTPAREPVQRTRLLDILRTGGRRRLALIHAPAGFGKSTLAAQWRGELTADGVAVAWIGIDSDDDNEIWFLAHLIEAIRRVRPDIGTGLDQVLEEQPADAVAYAITTLIDDVHAGGATVVVVVDDWHRITDPGTRRVMDSLLDNGCHHLRFVVTSRDQSGLPISRMRVRDELVGIGSAELRLTREETRQILVDRNRFTLDDAQIDELHRATDGWPAAVQLISLALRGNPDPDPLIQHLAEGGHGVREYLAENVIDALEPRMVDFLTAISIAEKVSGSLAAALSDDPEAEHLLEQAEQRELFVRRVEYDPEWFRVQPLFAEHLRARLERTDPARVKVLHRKAARWYAEHQLLRKSVDHAVSATDLKMALDLLESGGMDLIDGSRLATLLGTVSKLPVQQVASRSKLLMAVARANVNLQQSGAARSALGRLSSVLSRGSSGDADVVRQRCQAAVLAAADQVARDHTEGVMDQIGDCLDHPDELPAWTVSTAANLTSFVRLCEFDFDGARSIQDWAAEYHERSKDPLGSVFGLCSRGAVAFEQLDIATAARCFQQAWDTARARSGQRSHAVRVAAALLGELHYRRGELDAADRLLDESHELVARVGPIDFLISTFVIGARVKAVRGDMATAASRLAEGQRIAVEQDLPRLAAQVRAERVRLGPAAETSGPQTATERWNGNVIRDTGSTGHRLTGTAALTAEAEEIAAIRELLADGGIDDQDRAVRRARALYGRTHELHRPRAQLDTSLLLAECLAAAGWVGEAAAQLVPAVTTCAELDWTRPLLDAGPGVVAILRVLRNDLPSELPIRFVDELLA

>CORE_REP|Org45_Gene3532#

MHIHRAERADTLVDALAQLLARPLDDPFAAEVVAVPAKGIERWVIQRLATVLGSATGGDGIAANIEFPDPASLVGAVLAEATGLTPDTDPWAPERLVWTLLPVLDAVLAEPWCGVLSRHLGRGDAAGHKVGRRYATAARIADLFDGYGTQRPGMIAAWADGLDTDGTGRELPEDLRWQPRLWRTLREAVGVPSPAERLSAACAHLRAEPGVVSLPRRISLFGVTRLTTDQLEVLSALSAGREVHLWLVHPSPTLWTELASNGADAQADSADRSGSNAAGDTSASVRAVSDRERVDRADGSSPSSEMPDSLVRVRHPLLAALGRDVRELQQRLHAYDHTDTYHPPAAISPGTTASPGRAERSGARTVEAASDTPELGRPVDSAAGHVIEPTADCAEGGATEFSDVAGSDRGDRAVAAGFVAAATPAVGTGESATGRRSVERGAVAGASSVRGVASGRTLLSALQAAIREDRWPVAVEPELAGDGTVGVHACHGPARQVEVLRDCLLHIFAADHTLQPRDVVVMCPDVESYAPLVRAAFGQWSLDTAEAGHPGHALRVRLADRAQRAVNPLLGVIGTLLELADGRVTVTEVLDLAAAEPVRLRCGFDDDDLERLREWAAETGARWGIGQRQRQAFGLGDFAQNTLNAAVDRILLGVAAGESGADWLDLALPLDDVDSGDIDLAGRFAEFVDRLAVCLRDLRGPTLAEPAGTARPAGEWAAVLGRALDLLTDVTRAQAWTGVQARRELAAALEHAGDVPLRLPDIAALLATRLAGRASRANFRTGELTVCTMVPMRSVPHRVVVLLGLDDEVFPRAGGIDGDDVLARHRCPGDRDPRSEDRQLLLDAIMAAGERLVLLHTGSDPVTGAHRPPAIPLAEVLDTVRAHVGRAAMARIVRRHPLQPFDAANFRADDPFSFDPVALAGAVAARRPQRSRPVFLAAPLPSAAPGDVELTDLVAFAEHPVRAFLWQRLGIRVPEEEEEIDDRLPIALDGLTKWSMGERMLAARLNGVEADMLRAAEWRRGTLPPARMGAAVLDVIEGTVDQLVRVARPMHEIPGRTIDVAVDLGDGRRLTGTVADVHDDALLRATYSRLAPKHRLAAWVRLLALAASGSHQSWRALTLGRGQFRNPVWQSTLTAPDAATARAILRQLVHLRDAGLTEPLPIAPSATAVYADRRCKGASVDDATLSAEQDFDSAYGERTDRYLRQVWGPALRFPDLTRAPAEHGGDEPTRFGELARMLWNPLLANENQGRP

>CORE_REP|Org176_Gene3205#

MSTASFVHLHNHTEFSMLDGMAKIGPLFAEAERLGMPAVGMTDHGNMFGAAEFFTHASKTGITPIIGIEAYVAPESRLSTRRVFWGEPGQRADDVSGSGAYLHMTMFARDATGLRNLFELSSLASIEGQLGKWPRMDAELIAAHADGIIATTGCPSGEVQTRLRLGQFDDAYEAAGRWREIFGPDNFFLELMDHGLSIERRVREDLLTIGDKTGIPPLATNDCHYVLPGQAAAHEAMLCVQTGKTLSDPTRFRFGGNGYHLKSAAEMRALWDDEVPGACDATLAIAERIEPYDEIWRFRDRMPVFPVPDGHTEDSWLRHEVDAGLRRRFPGTVPDTYRDRAAYELDIIRDKGFPAYFLVVGDLVAHARAVGIRVGPGRGSAAGSLVAYALAITNIDPIEHGLLFERFLNPERPSAPDIDIDFDDRRRGEMIRYATDRWGADRVAQVITFGTIKTKAAIKDAARVNYGQAGFAVADRITKALPPAIAAKDISVAGIMDPAHERYAEAAEVRALIESDPDIRRIYDTAAGLEGMVRGAGVHACAVIMSSEPLIDVVPLWRRPQDGALITGWDYPSCEAIGLLKMDFLGLRTLTVIGDCLDNIRANRGLDIDLDTLTHTDPNTYAMLARGDNLGVFQMDSTGMRELLLRMAPTEFNDLVASNALYRPGPMGVGAHWAYADRKNGREAITPIHPELDEPLRDILGETYALIVYQEQIMQIAQHVAGYSLGQADLLRRAMGKKKPEVLAMEFENFRNGMRGNGYSDAAVTALWEAVLPFAGYAYNKSHAAGYSLIMYWTAYLKANHPAEFMAALLTSIGDDKDKSALYLADCRKRGIRVRPPDVNSSAATFTSVGTDIRFGLGAIRNVGGPVVEAITRARDTGGPFTTFSDYLDRVDATACSKKVTESLIKAGAFDSLGHRRKGLHRIHDDAIEAALGVKKAAARGQFDLFAADDAEDETASVFHIAVPDEEWEKPQLLAFEREMLGLYVSGHPLDDVAGALAAAVDAPIATVLAGSVPHGRTVALGGMIAQVERRITKKGDPWAVVRLEDLDASTEILFFPAAYARAATTLIEDAVVLVTARVDIRDGRRSLIADALTVPELARAGGTPLTLSLRTDTCTRDNVRALREILQRHPGNTEVQILYHARTGPARYGLDPALRVHLSSGLLGDLKALLGPAAVAAGSTVTAVNGAYGPGTSSVRGGRITSTSAIGPA

>CORE_REP|Org80_Gene4825#

MGWSNGPPSWSELERVLSGRPGRHDPDSMYPGDGGDSPAWSRKRGEYLADGDDVRPLGPVVPYAELHAHSAYSFLDGASHPEELVEEAARLGLEAIALTDHNGFYGAVRFFEAAREWDIATVYGAELTLAATESRSPVDPPPHPSRPARAGLRSLPVDAESASAEPSPRDTAAPRPHSGAPDSAARTGEPDPPGVHLLVLARGQEGYRRLSREIAAAHMAAGEKGILRYDLDTLTAAAGGHWQILTGCRKGALRQALEQDLRAGSEPVRAEAALRDLVERFGAERVSVELTHHGLPADDERNAHLIALADRLGLPVVATTGAHFASPAQRHRAMALAAIRSRSSLDEMAGWLAPTGGAHLRSGAEMARLFADCPQAVANAVALARECAFDLKLIAPELPPFPVPEGHDENSWLRELTLQGAAERYGAPQENPAAYRQIEHELAVITGMKFPGYFLVVHDIVTFCKNNGILCQGRGSAANSAVCYAIGITNVDPVRNNLLFERFLSPARDGPPDIDIDIESDRREEAIQHVYHAYGREYAAQVANVITYRGKSAVRDAARALGFSTGQQDAWSKQVSRWTGVGAETGTDIPERVLELAADIEGLPRHLGIHSGGMVICDRPIADVCPVEWARMPGRSVLQWDKDDCAAAGLVKFDLLGLGMLSALHYMIDLVREHEGVTVELHKLDLAETAVYEMLSKADSIGVFQVESRAQMATLPRLKPREFYDLVVEVALIRPGPIQGGSVHPYIRRRNGQEDWDFDHPSLQKTLARTYGVPLFQEQLMQIAVDVAGFTAAEADQLRRAMGSKRSPERMERLKARLYQGMRDLHGITGEVADRIYEKLYAFANFGFPESHSQSFAALVFYSAWFKLHHPAAFCAGLLRAQPMGFYSPQSLVADARRHGVVVHGPDINASRAEPTLEQRGTQVRLGLAAVRYIGAELAEKIVAAREESGPYTSLLDLTGRVELTVRQAESLATAGALDSVTTVASTRNGSPSGAAIPVRAATNRTPPASIPARSEPPPGGAAPGASSVRRTALWAAGAAAAERSDRLPGLVAAAEAPALPGMSALELAAADVWATGISPGSYPTEFLRAELDAMGVIPADRLLSVPDGSRVLVGGAVTHRQRPATAAGVTFLNLEDETGMVNIVCSVGLWTRYRRLAQTARALIIRGRIQNAEGAVSVYAETLRQLDLRMAGKSRDFR

>CORE_REP|Org31_Gene4357#

MFTKVLVANRGEIAIRAFRAAYELGVGTVAVFPYEDRNSVHRLKAAESYQIGEQGHPVRAYLSIDAIIDAAKSAGADAVYPGYGFLSENPDLAAACAREGITFIGPSAEVLELAGNKARAIAAAKAAGLPVLRSSVPTADVDELLAAAQELEYPIFVKAVAGGGGRGMRRVAEPAQLRESIEAASREAESAFGDPTVFLEQAVVNPRHIEVQILADQHGNVMHLFERDCSVQRRHQKVIELAPAPNLDLALRDRICADAVAFAKQIGYSCAGTVEFLLDERGNHVFIEMNPRIQVEHTVTEEITDVDLVQSQLRIAAGETLEQLGLSQDKITIRGAALQCRITTEDPANGFRPDTGRITAYRTPGGAGIRLDGGANLGAEIGAYFDSMLVKLTCRGRDFPAAVARAGRALAEFRIRGVTTNIPFLQAVLDDPDFKTGRVTTSFIDERPQLLTLRGSADRGTKILNYLADITVNKPHGERPTTVYPHDKLPPIDLTVPPPDGSRQRLLRLGPEGFARDLRAQKAVGVTDTTFRDAHQSLLATRVRTNGLLGVAGHVARLTPELLSIEAWGGATYDVALRFLYEDPWERLALLREAVPNICLQMLLRGRNTVGYTPYPEQVTRAFVSEATATGIDIFRIFDALNNVDQMRPAIDAVRETGTAIAEVAMSYTGDLSNPDETLYTLDYYLKLAEQIVDAGAHVLAIKDMAGLLRAPAAATLVKALRSNFDLPVHVHTHDTPGGQLATYLAAWQAGADAVDGASAAMAGTTSQPALSAIVAAAAHSEHDTGLNLQNVCDLEPYWEALRKVYAPFESGLPAPTGRVYTHEIPGGQLSNLRQQAIALGLGDRFEEVEAKYAAADRLLGRLVKVTPSSKVVGDLALALVGTGVDIEDFAADPGRFDIPDSVIGFLRGELGTPAGGWPEPFRSRALAGRGPAKPETPLTPADEAGLAGSSEQRRTTLNRLLFPGPTSEFLAHREKYGDTMGLSANQFFYGLRHGEEHRVQLEKGVTLLIGLEAIAEPDERGMRTVMCILNGQLRPVAVRDRSIASDVPAAEKADKGNAGHIAAPFAGVVTLAVSEGDAVAAGDTIGTIEAMKMEAAITAPRAGTVGRVAIGQVQQVEGGDLLIELTMGESGAGDQLRSVRNDQSE

>CORE_REP|Org63_Gene886#

MRGSQIALRTVPDAATAVLTKPPAAPVVTRNDFRTARLIALVAGLLGALFALATPFLPVTQTTAVLNWPQGGTLGNVQAPLMSQVPIDLKATIPCETIAQLPERGGMLLATAPPQGDRAALEAMFVRVSETSVDVVDRNAVVVSADRSRMGECAALSISSDSERTYAVFTGLTKQVERPVEGGAPGATELATVPVEGQLGGDLRPQVVGVFSDLKGAAPAGLAFDMTVDTRFSSSPTAIKLVAMIAAVLCTLIALAALARLDGSDGRGHRRFLPANWLKPTWADGAVAGTLLLWHFAGANTSDDGYILSMVRVAPHAGYMANYFRWYGVPEAPFGWYYYVIQVFSEISTASPWVRLPALACAILCWLVISREVVPRLGRGVRTSKVALWTGGLVFLAFWLPFDNGLRSEPIVALGALLTWVSIERAIATGRLLPAAVAILVAAFTLAAAPTGLMCVAALLAGIRPLVRIVVRKHRQFAALGAGRWGSTLPLLAPIAAAGVLVLTVVYSDQTFAGIQEANRVRQVTGPNLAWYEDYLRYYYLFVETVDGSLSRRFAFLVMLLCLFTTMLVLLRRRQVPGIASGPTWRLMGVVFGTIFFMMFNPTKWTHHFGAYAGIAGSLAAVTAVAVSASALRARKNRAIFLAGLLFVLAVAFSGINGYWYVSSFGVPWFDKRISLQGYQSNTVMLMLFGLALALVGWYALREDYTKPQPSAKTARGRRIRRFAAIPLTVVAALMVALEVLSLVKGAVSQYPAYSLARSNIDALGGSTCGLANDVLVEPDPNGGRLEPIIDPARPLTDPLAGVDSVGFDPNGVPNDLSADSVEVKPGTGNTSTQSVGAAFAEGQSAGTGGGQGALGVNGSTVALPFGLDPASTPILGSYQNGMQQPANVTSSWYQLPARSADKPLVVISAAGRILSFDDTGAMKYGQSLTVDYGKHLPDGTVQKLGTYLPRDIGPFPSWRNLRVPLDEIAPDADAVRIVANDPILIGDQWLAFTPPRMPKLQTLNSLLGSQQPILLDWAVGLQFPCQRPFDHENGVAEVPGYRILPDRPLAISSTNTWQAEEFGGPLGFAQMLAKSTTVPTYLKHDWARDWGSLERYDQYDRNAVPAKLDTGTTTRSGLWSPGNLRVF

>CORE_REP|Org113_Gene3340#

MPRRNDLQHILVIGSGPIVIGQACEFDYSGTQACRVLRSEGLRVSLVNSNPATIMTDPEFADSTYVEPITWEFVEKVIVAEKAKGTPVDALLATLGGQTALNTAVALHENGILEKYDVELIGADFEAIQRGEDRQKFKDIVAKVGGESARSKVCYTMDEVRETVAELGFPVVVRPSFTMGGLGSGMAYNDDDLDRIAGGGLAASPTANVLIEESILGWKEYELELMRDGRDNVVIVCSIENVDPMGVHTGDSVTVAPAMTLTDREYQKMRDLGIAILREVGVDTGGCNIQFAVDPRDGRLIVIEMNPRVSRSSALASKATGFPIAKIAAKLAIGYTLDEIVNDITKETPACFEPTLDYVVVKAPRFAFEKFPGADPTLTTTMKSVGEAMSLGRNFSEALGKVLRSLETKAAGFWTQPDGRWTDVAEVLADLRVPIEGRLYQVERALRLGASVEEVAEASGIDPWFVAEIAGLVELRGEIAQAPVLDEPLLRFAKHNGLSDRQIAALRPELAGEDGVRELRHRLGIRPVYKTVDTCAAEFEAKTPYHYSTYELDPAAESEVAPQPDREKVLILGSGPNRIGQGIEFDYSCVHAAQTLSEAGYETVMVNCNPETVSTDYDTADRLYFEPLTFEDVLEVYHSESESGRVAGVIVQLGGQTPLGLAQRLTDAGVPVVGTSAAAIDLAEDRGEFGQVLVAAGLPAPKYGTATTFAQAKEIAARIGYPVLVRPSYVLGGRGMEIVYDESSLEGYISRATELSPEHPVLVDRFLEDAIEIDVDALCDGEEVYLGGVMEHIEEAGIHSGDSACALPPITLGRSDIESVRRSTAALAQGIGVKGLLNVQYALKDDVLYVLEANPRASRTVPFVSKATGVQLAKAAARVMLGTSIAQLRKEGILPAEGDGGHAPMDAPVAVKEAVLQFHRFRRPDGTGVDSLLSPEMKSTGEVMGIDTDFGTAFAKSQSAAYGSLPTEGTVFVSIANRDKRAMVFPVKRLHDLGFRILATEGTAEMLRRNGIPCERVRKHSDPEFPAGSAGAADEAPVPSVVDQIKDGEIDIVFNTPYGNSGPRVDGYEIRTAAVGANIPCITTVQGAAAAVQGIEATIHGGIGVRSLQELHAVLRGHEER

>CORE_REP|Org23_Gene2846#

MRGAEGGEVLDYHALNAMLNLYGPNGEIQFDKDREAAHQYFLQHVNQNTVFFHNLDEKLDYLIDENYYEREVLDQYSREFIKSLFQQAYAKKFRFPTFLGAFKYYTSYTLKTFDGKRYLERFEDRVCMVALTLAAGDEQLARELVEEIIDGRFQPATPTFLNSGKKQRGEPVSCFPAGTPVDTIDGPRAIETLRPGEKVLSHDGSYRAVEALIENPNDQALVEISHFGHKEPIRCTPEHPILVWTTREVDTLIDGDGADPFNGFVWLAARDVQPTDFIVTTAPLGERDRRVFDLMEYTGEGVYEEVDGQIRKVNTDAKHRNKQRHNQRFVPVNRFVEESYELGLILGWYVAEGHVSKRSTDVSVPNGVHFTLGAHEVEYHVELGMAFKQVFGVDLSLHTNHSDQSTRMVCNSKIVASLLLSLAGTGYNAKRLSADVLTADEDFQRGLLAGLFRGDGCSTSGGMMLDLVNPELIDQVQLILRRLGIMSVVRSYINQAGNVTGQVFVPGLPGNNEDFIFDIGKNLHSYVGRKGVKRTTYQVVHGRHVYGVRSIGRTTETPEKVYNLHVEGTHTYTIRGTVVHNCFLLRIEDNMESIGRSINSALQLSKRGGGVALLLSNIREHGAPIKKIENQSSGVIPIMKLLEDSFSYANQLGARQGAGAVYLHAHHPDIYRFLDTKRENADEKIRIKTLSLGVVIPDITFELAKKNEDMYLFSPYDVERIYGKPFADIDVTEKYYEMVDDKRIRKSKIKAREFFQTIAELQFESGYPYIMFEDTVNRANPIAGKITHSNLCSEILQVSTPSEFNDDLSYSKVGKDISCNLGSLNIAKTMDSPDFAKTIETAIRALTAVSDQTHIYSVPSIEQGNNQSHAIGLGQMNLHGFLARERIHYGSEEGIDFTNIYFYTVVYHALRASNRIAIERGSYFGGFPESKYASGEYFDKYTDQVWEPKTDRVRQLFDEAGVHIPTQDDWRELKASVMEHGIYNQNLQAVPPTGSISYINHSTSSIHPVASKIEIRKEGKIGRVYYPAPYMTNDNLEYYEDAYEIGYEKIIDTYAAATQHVDQGLSLTLFFKDTATTRDLNRAQIYAWRKGIKTLYYIRLRQMALEGTEVEGCVSCML

>CORE_REP|Org19_Gene3335#

MADETSTRNAYPRVDFTAADAGSRQGASVSFPELERRVLDYWAADDTFRASIDNRAEGCDEFVFYDGPPFANGLPHYGHLLTGYVKDLVPRFQTMRGKRVERRFGWDTHGLPAEIEAEKQLGITDKSQIDAMGLAEFNAACKSSVLRYTNEWRDYVTRQARWVDFDNDYKTLDLDFMESVMWAFKSLYDKGLVYQGFRVLPYSWYEQTPLSNQEARLDDAYRMRQDPAVTVDMLLEVAADHPLHELDGANALIWTTTPWTLPSNLAIAVHPDITYAHVRGKDGKRYVLATERVSHYARELGTADDGLEVLSEHSGAALAGLRYRPPFDFFLGHPHAHRVLNADYVTTDSGTGVVHLAPAFGEEDMDVASANGIEVVQPLDAGGKFTSMVPPYEGLMVFDANPVIIKDLKAAGKLLRHETIEHSYPHSWRSGQPLIYMAVPSWFVAVTKFRDRMVELNQQITWVPEHIRDGQFGKWLENARDWNISRNRYWGAPIPVWVSDDPAYPRVDVYGSLDELERDFGVRPADLHRPGIDELVRPNPDDPTGKSMMRRTPEVLDCWFESGSMPYAQVHYPFENKEWFDGGAVTSDTAGTSVARAHSPGDFIVEYNGQTRGWFYNLHVLSTALFDRPAFKSVVAHGIVLGDDGLKMSKSKGNYPDVNEVFDRDGSDAMRWFLMASPVLRGGNLIVTERGIREGVSHALRPLWNAWTFLQLYASKPGVWRTDSAHVLDRYILAKLAAARDVMTEALEVYDIATACDELRSFADALTNWYVRRSRSRFWEEDRDAIDTLHTVLEVTCRLAAPLLPLITEVIWRGLTGERSVHLTDWPGETELPRDPELVSAMDEVRSVCSTVLSLRKAQNLRVRLPLSEVTIAAPDAERLRPFADLVADEVNVKKVDLTTDVAAHGRFELVVNARAAGPRLGKDVQHVIKAVKSGQWREDDGVVKALVPGHDDGIALLPEEYTQRLVAAEPESTAALPGNAGLVVLNSEVTEELEAEGWARDLIRDLQETRKSLGLDVSDRITVVLEVPQERAEWARTHRDLIAGEILATALDFGAAGAGAADVVGGVRAAIAKA

>CORE_REP|Org53_Gene3008#

MVRPPSARSAVPGVGRLGLLDPTAAASLRELGWDNVESIPVLWALSRAPDADLALNTLMRLREALGSDWQRLDSAIRTDTSLRGRLFALLGSSTALGDHLVAEPAAWEVLRRGDLPDRDELLADLLAAVQATPEAGPHAGPMLFRAGIAGPEAVALLRCRYRDQLMLLAALDLAATVENEPVLPYRVVGRHLTDLADAALTAALAVAVARVCKDQPCPVRLAVIAMGKCGARELNYVSDVDVVFVAEPADATATRLAAEMMSVGSQAFFEVDAALRPEGKQGALVRTLDSHLTYYKRWARTWEFQALLKNRPMTGDLELGREYRDAVMPMVWTASERPDFVPEVQGMRRRVEDLVPAELRERELKLGRGSLRDVEFAVQLLQLVHGRVDENLHVASTVDALSALAAGGYVGRDDAANLTASYEFLRLLEHRLQLQRLKRTHTLPADDDEEGMRWLARAAHIRPDGRQDAMGVLRSEIRRNAVRVRRLHAKLFYRPLLEAVVRMDPDALRLSPDAAVRQLAALGYAAPENAFGHLKALTGGVSRKGRIQALLLPTLLEWLGETPNPDAGLLAYRRVSEALDEQTWFLRELRDEGAVAQRLMIVLGSSEFLPDLLINAPETIRMFADGPHGPLLLGPQPEEVARGILTAAARYDDPNRAVAAARSLRRHELARVASADLLGMLEVPQVCRALSSVWVAVLDAALAAVIRAGEAESGEPAPAAFAVIGMGRLGGMELGYGSDADVLFVCEPRPGVDETKAVKWANTVAERVQRLLGAPSTDPPLHVDAGLRPEGRSGALVRTLSAYQAYYGQWAQSWEVQALLRAHQVAGDQELGVRFLHAVDKVRYPAGGVSEDAVREIRRIKARVDSERLPRGADPATHTKLGRGGLADIEWTVQLLQLRHAHEVESLHNTATLETLAAIEKAELLAAEDVALLRDSWLLATKARNALVLVRGKPSDQLPGPGRLLSAVATVAGWPNNDGGSEFLDHYLRITRRARAVVERVFGS

>CORE_REP|Org144_Gene5995#

MKFSAKDLVGSVQRLMATAQNGLEVIRFGGLTHDVESSPFEVVERRRMYRLRHYFPDDTTPDRPVVLLVPPLMVNADIWDVNAEDGAVGILHRGGIDCWVVDFGSPAKEEGGWERDLADHVLAVSSAIDAVTEATGSSVHLMGYSQGGMFAYQVAAYRYGKGVQSIVTFGSPVDIVAGMPFGLPYGMVSDVADFLADHVVTRLPITDSMVRIGFQMLDPMKTAKARIDFLRQLHDREALLPKERQRRFLNSDGWVGYAGPAAADLLKQFVAHNRLMLGGFVIRDHPVSLAELKCPILAFVGEVDDIGQPGAVRGIVRAAPNAEVYEATLVAGHFGLVAGSTATNHTWPLVRQWVDWLERDTLLPPEIHPMVDQVETNRPRSAATRVVHTAASLAEAGAGVGKALEGIANNTVRGSVELAGEAARALPRLTRLGMIQPHTRISLGRLIAEQGRRAPLKDLFLFDDRVHTNAAVNVRIDNVVRGLISVGIRPAMRVGVIMETRPSALATVAALSRLGAVAVLLAPGSELVRALELTGVDTVVADPENLRHAAETGARVLVLGGGDARQLDLPANGRVIDLEQIDPAQVKLPGWYRPDPGLARELAFVLVTGTGDRLETKYITNHRWALSAFGTATTADLNRRDTVYCLAPLHHSSGLLVSLGGAIAGGSRIALARSLDPARFAEEVHRYGVTVVTYTWTMMRDILDAEVFPTGPYSPTPSASPTANPQWHPIRLFIGSGMPAGLWRRTAEQFEPARVVEFYASIEGDVVLANVKGAKRGCKGRPVPGTARVELVAYDPVTEEIQTDEAGYARRCADNEVGLLIGKATEGVDISAGGLRGVFAPGDSWMPTENLFRRDADGDYWLIDRKDTVIHTRRGPVFGQPIVDVLNDITAVDMEVAYGLAVGDHCIAVAAVCVRKGLRLEPKDVTEALRALDPDQRPDVVYVVDEIARSASYRPSTRAVQAAGRPEPGPDTWWYNRASDAYEILTEQDAAAVLGDR

>CORE_REP|Org163_Gene5077#

MSTPVRGADLTDLIHSAAPAKSAGTNPAPAPAASRDPNGSHPPQPGGRVRVIREGVASYLPDIDPEETSEWLESFDEMLDREGPGRARYLMLRLLERAGERRVAIPSLTSTDYVNTIPTENEPWFPGDEEVERRFRAWIRWNAAIMVHRAQRPGIGVGGHISTYASSAALYEVGFNHFFRGKDHSGGGDSIFIQGHASPGIYARAFLEGRLSSDQLDGFRQEYSHGGPGHGLPSYPHPRLLNNFWEFPTVSMGLGPMNAIYQARFNHYLHDRGIKDTSDQHVWAFLGDGEMDEPESRGLAHVAAMEGLDNLTFVVNCNLQRLDGPVRGNGKIIQELESFFRGAGWNVIKVIWGREWDALLGADRDGALVNLMNSTPDGDYQTYKANDGAYVRDHFFGRDPRTKALVQDLSDQEIWNLKRGGHDYRKVYAAYAAAMAHKGQPTVILAKTIKGYTLGKHFEGRNATHQMKKLTLQDLKDFRDLQRIPISDAELEKDPYLPPYYHPGMEAREVQYMLDRRKALGGFLPERRAASKPLKLPGDEAYRSVRKGSGKQNVATTMALVRLMKELLRDKEIGKRIVPIIPDEARTFGMDSWFPSLKIYNRNGQLYTSVDAELMLAYKESAVGQILHEGINEAGSTASFTAAGTSYATHGEPMIPLYIFYSMFGFQRTGDGLWAAADQLARGFVLGATAGRTTLTGEGLQHNDGHSLLLASTNPAVVTYDPAFAFEIAHIVRDGLRRMYGGGTPPQGAAPLPGTHPHGSAGEFGGEDVFYYITLYNEPYPQPAEPEGLDVAGLLKGIYLYKRGGEGAVRAQILVSGVTVPDGLRAQALLAQEWGVQADVWSVTSWGELRKEALDKEIAALRNPGADPGVPYVTEALSRADGPYVAATDWMRAVPDQVRKWVPGDFTTLGTDGFGFSDTRPAARRVFNVDAQSIVVAALAGLGRTGGIDPAKAVEAAAKYRIDDVDAAPKPAASAEEELA

>CORE_REP|Org125_Gene1111#

MEIQAVTSPYDDGPNGGRPPRGPQSGPGGQPPRPAGGNPPGARPLPPRRQAPPPGPGGPRGGQPGGPPNPAGGPPRRPGPPPGGDRTPPMRGPAGGPPRTGGQPTVRGGQPNPAGGPPRRSANPAPRPGAGATQKIAKPGEQKPQATQKIAAGTLGEAMAQRGPRSTAANRSAPGGAGTRSGSGPGTGGRRAVAGGTPPSGPPPRKGNGGGDGPGGSAGKGPKTKKKSPWRIVRRVIYVLVALAIVVPSAVFLIAYTTVSIPQPGDLKTPQVATILASDGTTQISKIVPPEGNRTDVTIDQIPPHVRNAVIAAEDRDFYSNPGFSISGFARAARDNLMGKDTAGGGSTITQQYVKNAMVGNQHSLSRKMRELVISAKMARQWSKDDILTAYLNTIPFGRGTFGIDAAAKAYFGKSVEQLTVEEGAMLAATINQPYGLDPENNPKGAEQRWNYVLDGMVKAGSVPAAERAKMVYPKVLPSSANNDDSESKTAGPNGLIKRQVLSELSEAGISDTQLNTEGLQITTTIDQKAQQAAIDSVHKNMQGERDEVRTAVVSVDPKSGAVRAYYGGDNATGWDFANAGLQSGSTFKVFGLAENLELGKPLSTMYDSSDLTVNGIKITNAEGETCGTCTIAEALKRSLNTSFYRMELDMPDGPAKIAAMAHRMGIPDTIPGVGQTLTEPDGSGPNNGIILGQYQVRPLDMASAYATIAASGVYHKPHFVQKVVTADGQVLLDRGQVAGEQRISAAVADNLASAMQPIAASSRNHGLAGGRPSGSKTGTTQLGDTGQNKDAWMIGFTPSLSTAVWVGTADGVALKTPGGSIMYGSGLPSDIWKDTMDGALEGTPKENFPKPAAIGGQAGVPSYSAPYTAPTTTQQEYQPPVVVKPSQVEILPGITIPVPGIQPNPRSQPQQNQPQSQDTGPLPGQPVAPADGSSPSTSNSGDTSGNSRSQRPGAGVGNSTDGTGDGYTNSHR

>CORE_REP|Org30_Gene1262#

MTEPRIESEQDATRPLREDIRFLGGVLGDTIRDHEGPEVFDLIERVRIEAFRVRREEVGRSAVADMLDAVDIAVALPLIRAFSYFVLLANLAEDLQRDRRRAAHEAAGEPPQDSSLAATYRKLDAAALPGAEVADLLTDALVSPVITAHPTETRRRTVFDVQTRITELMRRRQHYPDRERAALELEIRRQVLTLWRTALIRLARLRIQDEIAVGLRYYELTLFDVIPAINAEVRAALRSRWPDADLLPRPMLRPGSWIGGDRDGNPFVTAEVVRTAAGQAAGVAFGRYLRELVELEKTLSQSGRLVQVSDAVAELATAGYADPATHADEPYRRALHRVRDRLISTAERALGTDVGSTGLLGLGLGPASGVGLWVGSVTRAAAVGPAHGGGPVADAAEIGGQKTSVSAAPAYPGPQSLLDDLDAIDASLRASGDGLLADDRLAALRHAVETFGFHLQGLDMRQNSEVHEQVVAELLAWAGVHPDYASLPEHERVRILSAELSTRRPLLGPHARLSELATKELGIIRAAAEVVATFGEPAIPNYIISMCTSVSDMLEAALLLKEGGLLDPGASDSPPRCAAGIVPLFETIEDLGAGAATLSAALEVPVYRELVAAKGMRQEVMLGYSDSNKDGGYLAANWALYRAELDLVEVARKTGIRLRLFHGRGGTVGRGGGRSYDAILAQPAGAVHGSLRLTEQGEVIAAKYAESGSAHRNLESLIAGTLESTLLDVEGLGDDAEPSYQLMDDLAARARAAYTRLVHDTPGFVEYFRESTPVAEVGDLNIGSRPASRKPTNSVADLRAIPWVMAWSQARVMLPGWYGTGSALEEWIDGDPQRLATLSGLYRRWPFFRTVLSNLAQVMAKSDLEIAARYAELVEDTALREQIFGMIGEEHARTIRMHAAITGNDQLLSDNPSLAESIHNRFPYLEPLNQMQVQLLRRLRGGDDSELVKRGILLTMNGLATALRNSG

>CORE_REP|Org134_Gene4320#

MDPSTAWSIRPTWEYVVSRTFADRHIGPDRAELDRMLPVVGVGSLDDLATAAIPAGILDDSVLAALPAAVSEHEALAELAALAHSNTVATSMIGLGYYDTLTPPVLVRNLLENPAWYTAYTPYQPEISQGRLEALLNFQTMVSDLTGMEVANASMLDEATAAAEAMTLLRRANRSASARLLIDTDLFPQTRTILYTRAEPLGIEIVEADLSGGQVPDGEFFGVLAQVPGASGRIVDVAPIVEAAHERGALVAVGADLLALTLITPPGELGADACFGTTQRFGVPMGFGGPHAGYLSVHAKHARQLPGRLVGVSVDADGAPAYRLALQTREQHIRREKATSNICTAQVLLAIVAAMYASYHGADGLRAIARRVHGHATAIAAGLDGAVVHERFFDTVLAHVPGGAEAVVGKAKSRGINLRLVDADHVGIACDEATTDAHVAAVLESFGTALPSEKRDAAPVSIENRTSEFLTHPAFTRYRTETAMLRYLRRLSDKDIALDRSMIPLGSCTMKLNSTAEMEAITWPGFARVHPYAPVEDAPGLLRLIADLEGWLSSITGYDSVSLQPNAGSQGEYAGLLAIRRYHLDRGDTHRDTCLIPSSAHGTNAASAAMAGLRVEVVACRDNGDVDLDDLRAKIADHADRLACIMITYPSTHGVYEHEVAELCALVHDAGGQVYIDGANLNALVGLARPGRFGGDVSHLNLHKTFCIPHGGGGPGVGPVAVRSHLEQYLPGDPLESGSHAVSAAKYGSASILPITWAYIRMMGADGLRRATLSAIASANYIARRLDEHFPVLYTGENGMVAHECILDVREITKQTGVTVDDVAKRLADYGFHAPTMSFPVAGTLMVEPTESEDLAELDDFIEAMIAIRREIDQVGAGVWPVTDNPLRGAPHTAASLVGEWDHPYSREIAVYPRGLDHSRAKVWPPVRRIDGAFGDRNLVCSCPPLDAYTD

>CORE_REP|Org134_Gene3863#

MDSTLAPETHGGHRPEPEPRSGNSPQAQHAPQAQESPQAPDTAAGGRAAAQGGLRAKQQRLTAEQQQRRTEAGHRRAEAEQRKSEETERLIDGLNPQQRAAVVHTGAPLLIVAGAGSGKTAVLTRRIAYLLAARGATPGQILAITFTNKAAAEMRERVIGLVGPRANNMWVSTFHSSCVRILRMQSALLPGLNSNFSIYDADDSRRLLTMISRDQEIDTKKYSARLLATAISNLKNELISPEQATADAESDDAELPGLVARVYTEYQRRLRAANALDFDDLIGETVALLQSHPQVAEYYRRRFRHVLVDEYQDTNHAQYILVRELVGHHASRPAADTAGADDAAAGAAAAAGSDDDWAEPDEHRVPPSELCVVGDADQSIYAFRGATIRNIEEFERDFPDAETILLEQNYRSTQHILSAANAVIARNEGRREKRLWTDSGEGDLITGYVADNEHDEASFVAREIDRLVDAGEATYGDVAVFYRTNNNSRALEEIFIRMGLPYKVVGGVRFYERKEVRDVVAYLRVLENPDDAVSLRRILNTPRRGIGDRAEACVAVHAEQRDIGFAAALRDAADGNVALLNTRAQRAISGFLELLEEIRAAGARPDADFPDVGNVVEAVLDRTGYRAELEASDDPQDGARLDNLNELVSVAREFSSEANNNAEAARAEGMLPEAADGEPEPGSLAAFLERVSLVADTDQIPDEGAGVVTMMTLHTAKGLEFPVVFVTGWEDGQFPHMRALGDPAELAEERRLAYVGITRARRRLYLSRAVVRSGWGQPVSNPESRFLKEIPGHLIDWKRLEPKSSGGSRTGRRRGDEDFERDWTEGWSEPRPGVRERRPAPRAGGVKRNNVDLVLAVGDRVSDDKYGLGRVVAADGVGPLATVTIDFGTAGKIRLIPQFSRTLVKL

>CORE_REP|Org163_Gene5199#

MKFACAAPVGWVFVQTHEIRRRFLDHFLRAGHTEVPSASLILADPNLLFVNAGMVQFKPYFLGQEEPPYPRATSVQKCVRTGDIEEVGVTTRHNTFFQMAGNFSFGDYFKEGAITLAWELISKPQDEGGYGFDPERIWVTVYQDDPETAEIWKRVAGMPEERIQFRDGKDNYWDMGVPGPGGPCSEIYYDRGPEHGRDGGPVADEDRYLEIWNLVFMQDVRGELSPKLGHPPVGSLPKKNIDTGMGVERIALLLQGVDNVYETDLLRPIIDKAEELTGRSYGFQHEDDVRFRVIADHARTAAMLIADGVNPGNDGRGYVLRRLLRRIVRSARLLGAEKPVMGEFMKIVSDLMAPSYPELATDFRRIETVAVGEETAFLKTLNTGSTLFDNTAAAVKAEGGSTIAGSDAFTLHDTYGFPIDLTLEMAAEAGLSVDEEGFRSLMAEQRKRAKEDAQARKHAHADLTIYKELVDRGATEFTGFDELTSEATVLALIADGVRVPTATVGQDVEVILDRSPLYAESGGQIADRGSITASSGLKLRVNDVQKIAKKLWVHKTTVEHGQVTEGDIVLAQADPAWRRGATQGHSGTHMVHAALRRVLGPNAVQAGSLNKPGYLRFDFNWQGQLSEQQKADIEAVSNDAVGADFPVNTFVTDLPKAKQMGALALFGENYGNEVRVVEIGGPFSMELCGGTHVQHSSQIGPITVLGESSVGSGVRRVEAFVGLDSYKYLAKERALLAGVASALKVPSEEVPGRVEQLVERLKVAEKELERTKMAAVLSSAGKFVEEAERIGRLLLVAVAAPEGVPAGDLRTLATDIRGRFGSEPAVVVLLGNADGKVPFVVAVNKPAQEFGVKAGDLVGSFGPSIAGRGGGKPEMAQGAGSDPSGIPAGLAAVRARVAELAG

>CORE_REP|Org44_Gene5774#

MDKTDPLTQKSRQALHDAQTKAVRFGHTEVDGEHLLLALLDDPDGLVPRLLAQAQADPDTLRTALETELGRRPKVSGPGAAPGQIFLTQRLVRLLDTAEREAKRLKDEYVSVEHLVIALIEEGTTTAAGRLLHEHGLTRDRFLQALTAIRGNQRVTSAMPEVAYEALDKYGRDLVADAAAGKLDPVIGRDAEIRRVVQILSRKTKNNPVLIGDPGVGKTAIVEGLAQRIHRGDVPEGLRDKTVFALDMGSLVAGAKYRGEFEERLKAVLNEVKAAEGRILLFVDELHTVVGAGAAEGAMDAGNMLKPMLARGELHMIGATTVDEYRKHIEKDAALERRFQPVLVDEPDEADAISILRGLRERLEIFHGVKIQDSALVAAVTLSHRYISDRFLPDKAIDLVDEACAMLRTEIDSMPAELDELTRRVMRLEIEEAALAKETDPASQSRLTELRKELADLRAEADAMRAQWEAERAALRKVQSLRQEIDQVRHDAELAERDYDLNRAAELRHGRLPELERRLDAEEQQLTAKQGRQRLLREVVTADEIAAIVSRWTGIPVSRLQEGERDKLLRLDEILHQRVVGQDEAVQLVADAIIRARSGIKDPRRPIGSFVFLGPTGVGKTELAKTLAAALFDTADNMVRLDMSEYQERHTVSRLVGAPPGYVGYEEGGQLTEAVRRKPYSVVLFDEIEKAHTDVFNTLLQVLDDGRLTDAQGRTVDFRNTVIIMTSNIGSEYLLEGATAGGEIKPEARERVMAALRGHFRPEFLNRIDDIVLFKPLTEAEIERIVELMTDELRGRLAERRMTLHLSDPARHFIAQQGFDPVYGARPLRRFIAREVETRIGRALLGGDVHDGATIHIGLSDGGLTVSFDNPNTGPSQDPADRVAAGTGS

>CORE_REP|Org114_Gene3624#

MIEDVTDLPNPSLPSVPQGPPAAGPGGAAGSSPVAPSASAMRRALRRARDGATLNLDEAVVLLHARDADLDDLCATAARVRDAGLRDSGYAGDVLPITYSRKVFIPLTRLCRDKCHYCTFVTVPGKLRAAGHGMYLEPDEVLDIARRGAELGCKEALFTLGDRPEERWPEARQWLDERGYDSTLDYVRAMSIRVLEETGLLPHLNPGVMSWAELSRLKPVAPSMGMMLETTSTRLFTEKGQAHYGSPDKDPAVRLRALTDAGRLSVPFTTGILVGIGENLTERAESILAIRKAHKAFGHVQEVIVQNFLAKSDTAMRDTPDADLQEFRATIAVTRILLGPKMRVQAPPNLVSLDECRALLAAGVDDWGGVSPLTPDHVNPERPWPNLEVLAQVTADAGYVLTERVTAHPKYVLAGHPWIDPRVSAHVAALADPATGLARPDTKPTGLPWQEPDHDWESVGRIDLNTAIDSEGRNTESRSDSALSDDGLGAFGDWETIREQVHELAAAAPERFDADVMAALRAAERDPAGLTDDQYLALATADGAALEAVTAFADQLRRDTVGDDVTYIVNRNINFTNICYTGCRFCAFAQRKGDADAFTLSSEEVADRAWEAWVEGATEICMQGGIDPELPVTGYADLVRAIKNRVPEMHVHAFSPMEVVNGASRGGQSIHDWLSALKEAGLDTIPGTAAEILDDEVRWILTKGKLPTSAWIEVITTAHRLGIRSSSTMMYGHVDNPKHWVGHLRVLRGIQDETGGFTEFVLLPFVHQSAPLYLAGASRPGPTVRDNRAAHALARIMLHGRIANIQTSWVKLGTTGTQLMLNGGANDLGGTLMEETISRMAGSEHGSAKTVAELTEIATGIGRPARQRTTTYGTPPRRSPVSLPVS

>CORE_REP|Org118_Gene5430#

MNTTVEPTQRKTVVVVGHGMVGHRFVEALRSRDEAGRWQIVVLSEESQAAYDRVGLSSYVGAWEKSALALPGNEYAGDALVDLRLGVRADEIDRAARKVTTSSGDVIGYDALVLATGSYAFVPPVPGHDRPECFVYRTLEDLDGIRAAAQNAGPGAVGVVVGGGLLGLEAANALRLMGMTPHVVEFAPRLMPVQVDEGGGAILEKLVTDLGLHVHTGVGTSAIEPAEDGAGLRVSLSDESVIDASLVVFSAGVRPRDQIARDAGLEIGPRGGALTDLGMLTSDPNIYAVGEVAAVEGTCYGLVAPGYTTAEIVADRLLGGAGEFPGADLSTKLKLLGVDVASFGDAHATTEGALSVVLHDAAKGTYAKLVISDDAKTLLGGILVGDASQYAALRPLVGSELPAEPAALISPAGAELGADALPDEAQICSCNNVSKGAIVGAIHEGACDIAGVKSCTSAGTSCGGCVPMIKKLLEQSGVEMSKALCEHFTQSRSELFQIVQVTGIRTFSELIAKHGTGIGCDICKPTVASILASTSSDHILDGEQSALQDTNDHFLANLQKNGTYSVVPRMPGGEVTPEQLIEIGQIAKDFGLYVKVTGGQRIDLFGARVEQLPQIWQRLVDKGMESGHAYGKSLRTVKSCVGSTWCRYGQQDSVGMAVLLEKRYRGLRSPHKLKLAVSGCARECAEARGKDVGVIATENGWNLYVGGNGGLTPKHAVLLAGDLDDETLIRYIDRYLMFYIRTADRLQRTAPWQESLEGGIEHLKQVVCEDSLGIAAELEESMARHVAGYKDEWAAVLEDPAKLSRFVTFVNAPEEADPTIAFDESGERKTPVLLGLPDVPGGGSLRNHGGHRAGRDGHGMPELPAMPAATATPGK

>CORE_REP|Org198_Gene7432#

MRFGRVRGVKALRRFTVRAHLPERLAALGELATNLRWSWHPPTQDLFAELDPQRWLEMGHDPVRMLGEVPAARVDELAADPDYVRRVDAAAADLRDYLAAPSWFERRAGEEGVRGIAYFSMEFGVTEVLPNYSGGLGILAGDHLKAASDLGLPLIGVGLLYRSGYFRQTLSADGWQTEHYPDLDPQGLPLRLLTSEQSESETAPVLIHVAMPDQRVLRARVWIAQVGRVPLLLLDSDIAENDPELRAVTDRLYGGDQEHRIRQEILAGIGGVRAVRAYTAANGLPDPDVFHMNEGHAGFLGVERIREFVAAGKDYDTALAAVRAGTVFTTHTPVPAGIDRFPMPMVRRYFGGAHGESESAMLPGLSVDRIVALGREADPSVFNMAHMGLRLAQRANGVSKLHGEVSRAMFAGLWPGFDAAEVPIGSVTNGVHAPTWAAREWFDKAREHIGAELVEEARGWERLRDVDLGELWSTRNALRAILVAEVRRRVRASWLDRGAAEAELGWVDSVFDPDVLTVGFARRVPTYKRLTLMLRDPQRLRAQLLDPQRPMQLVVAGKSHPADDGGKALIQQVVRFADDPAVRHRIVFLPDYDMSMARYLYWGCDVWLNNPLRPLEACGTSGMKSALNGGLNLSIRDGWWDEMYDGENGWAIPTADGVSDEHRRDDLEAAALYDLFERTVAPRFYDRDAAGMPVRWVEMVRHTLQTLGPKVLASRMVRDYAVEYYAPAANAYQQATADDFAVARTIADYRRRVEAAWPSVKVIQVDSAGLPDTPIIGARLSLTARIDLGGLAVDDVVVQAMLGRVSPSDDLSDVVTIPMTHQGSDSGVAHFVVDTPVPLSGAVGYTVRVLPHNELLAGDAELGLVAAPNA

>CORE_REP|Org138_Gene4495#

MIDHDSRIPTATTAPRSVVALVDSTTQVERELVGSWLAEGGINQEFGTEAPVTQIDLDPTAIATRLVDRHDDPLVVPVRVLWLPPERDGVRRTTFTDLITLSNPRKPNRLMQRRLIGKAPDRHLVLTGQPARLSELRANNPGAAGAAEAFARAIVRAGIVALERAERAVIGDRYKVPRLVAEEILDSPEFLRRLDDIAAHTGTSPREMHRRAEKALRELVAAQSRLVSDLFTQAMRPVHASTWKVDDDPVGFERLRSLNRRYPLVFLPSHRSYVDAFVLGDVLARNDFPPNHVIGGANLGFWPMGPIARRTGTVFIRRSFGDDEVYKAVVEEYFAYLLAKRFNLEWYFEGGRTRTGKLRPPRYGLLNYLAAALRSGRVDDVMLVPVSITYERLNEIGAIADEQTGGKKQPEGLAWLARYVRNQQHSAGRVYVRFGEPLSARERLTAHGDPLVEPADPGPAVSNPRAAASNTTSIPSPQAADTAPTTSNPAAAKADPEPETSNSTPATSNPEAVSNPTPAISASAAGGNGAVPESESVVEEQERRAVQRLAFDVAVGINAVTPITVNALTTLVLLGVHERALTRDELRAAIAPVLGYIEYRDLPRGELDTLRDDHGLAVVLEQLAIAKVVTVYRGGLEPVYSIGAGAHLEAAFYRNSAVHWFVNRAILELAVLTAVEAPEGDQLRVGWEAAYRLRDLLKFEFFFPERAEFTSELTAEMLHVDPQWHRRTAAGTVGTEILAQLAGSGFMMAHRVLRSFFDAQLVVAERLAAHDPATAVDRKAIIDECLNVGRQMLLQQRLQSPESVSSELFSSALKLADNHGLLTPDPADPAELAARRTRFAGELRAIGGRITRAATLDPSNRLETL

>CORE_REP|Org144_Gene202#

MTDTTLPPFGGSGGDRIDPVDIQQEMQNSYIDYAMSVIVGRALPEVRDGLKPVHRRVLYAMYDNGYRPDRGYVKSARPVAETMGNYHPHGDASIYDTLVRMAQPWSLRYPLVDGQGNFGSRGNDGAAAMRYTECRLTPLAMEMLREIDHETVDFIPNYDGRSQEPTVLPSRVPALLMNGSNGIAVGMATNIPPHNLTELAEAIYWALDNHDADEEATLAACMERVKGPDFPTHGLIVGSQGIHDAYTTGRGSIRMRGVVEIEEDNKGRTTLVITELPYQVNTDNFINSIAEQVRDGKIAGISDIHDESSDRAGMRIVVTVKRDAVAKVVLNNLYKHTQLQTSFGANMLSIVDGVPRTLRLDQMIRLYVKHQLDVIVRRTKYLLRKAEERAHILRGLVKALDALDEVIALIRRSANTDTARTGLMQLLDIDEIQATAILDMQLRRLSALERQKIIDELAKIELEIADLKDILAKEERQRAIVRDELAEIVEKYGDDRRTRIIAADGDVADEDLIAREDVVVTITETGYAKRTKTDLYRSQKRGGKGVQGAGLKQDDLVKHFFISSTHDWLLFFTNKGRVYRAKAYELPEANRTARGQHVANLLAFQPDEKIAQIIQIKNYEVAPYLVLATKNGLVKKSKLSDFDSNRSGGIVAVNLRDEDELVGAVLCSADDDLLLVSALGQSIRFSATDEALRPMGRATSGVQGMRFNASDELLSLNVVRPDTYLLVATAGGYAKRTAIEEYTPQGRGGKGVLTVQYDPKRGTLVGALIVEDDDELYAITSGGGVIRTVAKQVRKAGRQTKGVRLMNLGEGDTLLAIARNADEPDPDLLAGDTSDTGSSE

>CORE_REP|Org158_Gene3705#

MTQHLEQANAGQSNNDASATPPTPNSMPQRQGDPTSQRQSDPTSQRQGDPAAQRQGDTTAAQRPGDTTAQRPSLPVAQRQGGAPAAVPTSASRRVRARLARRMTGQRGIAAVKPVLEPLATVHRELYPKANLQLLQRAFDVADEKHAHQFRKSGDPYITHPLAVANILAELGMDTTTLVAALLHDTVEDTGYSLDELTNEFGQEVAHLVDGVTKLDKVNLGAAAEAETIRKMIIAMARDPRVLVIKVADRLHNMRTMRFLPPEKQAKKARETLEVIAPLAHRLGMATVKWELEDLAFAILHPKKYDEIVRLVADRAPSRDTYLAKVRAEIVNTLAASRINAIVEGRPKHYWSIYQKMIVKGKDFDDIHDLVGIRILCDEVRDCYAAVGVVHSLWQPMAGRFKDYIAQPRYGVYQSLHTTVVGPDGKPLEVQIRTQDMHRTAEFGIAAHWRYKETKGKHSNDSTEVDDMAWMRQLLDWQREAADPAEFLESLRFDLKSPEIFVFTPKGDVITLPQKSTPVDFAYAVHTEVGHRCIGARVNGRLVALERQLENGEVVEIFTSKAQNAGPSRDWQNFVVSPRAKAKIRQWFAKERREEALEAGKEAISKEVRRSGLPLQRLMSADAMSALAHELHYPDISALYAAVGESQVSAHHVVQRLMAQLGGVGDVENELAERSTPSTVPARQRGTGDAGVEIPGASGTVAKLAKCCTPVPGDEIMGFVTRGGAVSVHRTDCTNADSLRSEPERIIEVKWAPSPSSVFLVAIQIEALDRTRLLSDVTKVLADEKVNILSASVMTSGDRVAISKFTFEMGDPKHLGHLLNVVRNVEGVYDVYRVTSAA

>CORE_REP|Org102_Gene5670#

MNAANRPLRVGIVGAGPAGIYAADALMKSDAPAGFDGVSIDLYERMPAPFGLIRYGVAPDHPRIKGIITALHKVLDKPQVRLLGNIDYGVDITLDDLRGFYDAVIFSTGANADRALDIPGIDLDGSYGAADFVSWYDGHPDVPRTWPLDAQKVAVLGVGNVALDVARVLAKTGDELLPTEIPPNVYEGLKANQALEVHVFGRRGPAQAKFTPLELRELDHSPTIEVIVDPSDIDYDEGSEAARRHSKQVDMICNTLEQWAIRDVGDRPHKLFLHFFESPAEILGSGGKVVGLRTERTQLDGTGNCKGTGEYKDWDIQAVYRAVGYLSQNIPALPFDEQAGTVPNEAGRVLVDEGADGAARYLPQTYVTGWIKRGPVGLIGHTKGDANETIACLLDDVKDFTPAANPDPEAVTAFLEDKGIPFTTWAGWYRLDAHERALGEPEGRERVKVVEREDMLRASGVAVAPAVETAAILGALAGVHDPEINRPITELDMVAGVDIAPGNRVTVRVLLTVAGCPMRARLTRDIEAAVLSVPGTASVTVDFGVMTDTQRATLRQRLRGGDTPVIPFAQPGNRTRVYALASGKGGVGKSSVTVNLATVLARRGLRVGILDADIHGHSIPSMMGSTATPTQVDRMIMPPTAHGVRLISIAMFVSDNEPVVWRGPMLHRVLNQFLADVYWSDLDVLLIDLPPGTGDIAISLAQLLPTAEMIVVTTPQHTAARIAERAGAVATQTGQRVAGVIENMSWYEGPDGTRHLLFGSGGAEDVSARLTDILGADCPVLARIPLDPDVCAAGDEGIPMVLTHPESAAAQAISVLANRLDARRTRLAGQRLAVAPV

>CORE_REP|Org124_Gene1722#

MTAAQNLPVLFLTDPIVLPGMVVPIELDESAQAAIDAARAAKTDQVLVAPRLDEGYAAYGVVATIEQVGRLRGGAPAAVLKAERRAKIGHGVTGPGAALWVEAEPVEDVPADGRTKELAAEYKKLVVSVLQRREAWQVIDAVNQLSDPSAIADTAGYATYLTSEQKRELLETPEPAKRLATLIEWTKAHIAETEVSEKISEEVREGMEKSQREFLLRQQLNAIRKELGEDEPDGADDYRTRVEQADLPDSVREAALREVGRLERASDQSPESGWIRTWLDTVLELPWTVKTTDSTDVSAARAVLDADHHGLDEVKDRMVEYLAVRARRAARGLEVVGGRGSGAVMVLVGPPGVGKTSLGESVARALGRKFVRVALGGVRDEAEIRGHRRTYVGALPGRIVRAMKEAGSMNPVVLLDEIDKVGSDFRGDPAAALLEVLDPAQNHTFRDHYLDLDLDLSDVLFIATANVMETIPGPLLDRMELITVDGYTEDDKVAIARDFLVPRQLERNALTAEEVTVTEAALREIAADYTREAGVRQMERLIAKVLRKAATKLSEGGVADDDTVISLGLGYDPELGYDDPVHAGSDVASETAVGGADLPAGESRSTAAVSGESLTIDVGDLKDYLGRPRFTPDSVERTAVPGVATGLAVTGLGGDVLYIETNAVDGERSLTLTGQLGDVMKESAQIALTYVRSHLEEIGIEPSVLDRNIHVHFPAGAVPKDGPSAGVTMVTALVSLALGRQVRSDVGMTGEVTLNGRVLPIGGVKQKLLAAQRAGLKTVFIPARNEPDLDDVPAEVLAALDVRPVADVADILAYAIEPVEEPALDGRPLAATA

>CORE_REP|Org97_Gene925#

MGRAQDRARIFWYARPCGSLATSCHGAPPTAHRACDGGPIRLGRVPISQTLARLAGACVLAAVLVAGLLFPLAGGFGYMSNRAADAVDNVSAELVAGTAPAVSTMVDATGAPIAWLYEQRRFEVPSDKIANDMKLAIVSIEDKRFAEHGGVDWQGTLRAFLTNTSSGEVQQGASTIDQQYVKNFQLLVVAKTDAERRAAIETTPARKLREIRMALTLEKELTKDEILTRYLNLVPFGNGSYGIQDAAQTYFGVDAKDLKVAQAAMLAGMVQSSSKLNPYTNPKGVLERRNTVLDTLIQNIPSRADEFRAAKEQPLGVLPEPKGLPRGCIAAGDRGYFCDYALQYLANAGISKDQMDKGGYLIRTTLDPAVQNSVKAAVTANTDPNLENIAEVTSIIAPGQDSHHILAMTSSRTYGLDQGAHQTVQPQPYSMVGDGAGSIFKIFTTAAAMEKGLGTSAQLDVPSFFAAKGMGNGGAAGCPPATYCVKNAGNYRSPMSVTEALAQSPNTAFVKLIQDVGVTPTVDMAVRLGMRSYAEAGTSGHGNQSLADMIKQQNLGSFTLGPVAINPLELSNVAATLASGGKWCPPSPIAEVIDRDGKQVPLTQQACEQVVEPGLANTLANALSQDAVGGTAAGSARAVGWNAPVSAKTGTTETHRSSAFLGFTNSMAGAAYIYGDSPTPGEICSFPLRTCGDGNLYGGNEPARSWFGGIKPVLDKFPPPALPPLDDKYVRGSNNAQIPDVNGMSESEARSVLIGAGFQVSTVTTPGSAAKGTVTATTPNGSAIPGSVITVLVSDGTQREIPKPGPPPAPPVLPGLPQIPRLPPIPIPIPR

>CORE_REP|Org109_Gene6036#

MAGGASRTHTFHTSRACCGVRLVNPADSALRAQPRTGLSYGTSLLNRVLVGGPDGDPRLTHVVELPARAADRSAWPAWASPEVIAAIRDTGIDAPWRHQVDTADSAFHGRNVVVSTGTASGKSLGYQLPVLTALQADPKATALYLSPTKALGADQLRTVGALTHEGPLRDVHPATYDGDTPAEIRQWVRANARWVFTNPDMLHLGILRSHQRWARVLRKLRYVVIDECHAYRGVFGSHVALVLRRLRRIAARYGADPVFVLCSATSAEPAAAASRLIGAPCVAVTRDGSPQGPRTVALWEPPLLTAMTGENGAPVRRAATAEAARIMADLVGEGARTLTFVRSRRAAELTAMEAKRLLAEVDPDLAARVAPYRGGYLAEDRRALEAALSDGSLLGAATTNALELGVDIAGLDAVVISGFPGTVASFWQQAGRAGRRTQGSLVLLVARDDPLDTYLVHHPEALLDKPVEATITDPRNPYVLGPQLLCAALELPLTDAEVDDFGAREVLGDLSAQGLIRRRGAEDRARWYVTAETQPHDAVDVRGGIGAPVAIVDGETGRLLGTADAGRAQATLHQGAVHLHQGETYVVDELDPADGVAFVHAAEPGWTTSARQVTSIAVDAVTEYRAHGHVTAGLAQVRVTSQVIGYLRTLVTGEVLDLVELDLPPQTLPTRAVMYTVTPELLALAGIAPQDVPGALHAAEHAAIGLLPLVATCDRWDIGGVSTAEHPDTGLPTVFVYDGQAGGAGFAERGFAQLRQWLSATRLAIESCGCAAGCPSCVQSPKCGNGNHPLDKVAAARLLTAVLAELSADEDATLDS

>CORE_REP|Org101_Gene3463#

MTPPPMTPSDATETHVTDPLSLRRTQARPTTIRSTYRLQLRPDALTFADARAIAEYLQQLGISHLYLSPILTATKGSTHGYDVTDPTTVSAALGGPAGLKALSDEVRSRGMGLLVDLVPNHVGVADPRQNPWWWDVLRNGRESPFAHYFDIDWSAGNGAGGRLALPVLQSENDPAALTVDRSGAEPMLALHDLRFPIAPGTDGDNALRIHDKQHYRLVSWKAGVCTYRRYFTVGGLAAIRQEDPEVFEITHRELAAWCAHDIIDGVRIDHPDGLADPGAYLIRLRQIIGPNRLLLVEKVLSNREPLDATLPIDGTTGYDALADVGGVLIDPEGEPALTELSCRFAGHGSDRAWISETEHRIKRAVAETVLTADVRRLVAAIKRDARAESVDTMALTNATIEVLAFMPVYRTDYAPLAGMTAAVITEVERRNSELTNPLAVLTAALAMGGEAVTRFHQVGGAILAKAVEDTMFYQAARLVSLQEMGGNPARFGRSLIEFHLANIERAQRWPATMTTLSTHDTKRGEDVRARIGVLSQAAKDWARSVTAWQETAPGPDGATTLFLLQNMFGVWPPDGRPAAAIPGFRERLHQFAEKAIREAGTKTSWEEPDAAFEDEVHTWVDTVIDGPVGTELGDLVHRLAPHAWSDALAQKLLQLCGPGIPDLYQGCELWEDSLVDPDNRRPVDFALRAGMLQSLTGTPELDVTGAVKMWIVAYALWLRRERPDCFVGGTYAPLFGTGGEAQRLIAYTRGRPAEAPEIIVAATRHSVGLAETGWGDTALELPEGTWIDRLTGHTFQGRIRIERLFARLPVALLVR

>CORE_REP|Org106_Gene483#

MASRRAFDGGAGRPPVEVELSNLDKVLYPATGTTKGEVIAYYTAIAEAMLPHIAGRPVTRKRWPNGVEAPSFFEKNLAEHAPPWLERRVLEHSDRRVAYPLIDSEAGLAWIGQQASLEVHVPQWRFDGAAMGPATRIVFDLDPGPGVGLPECAEVALAVRDMIEDIGMHAFPVTSGSKGIHLYVPLDRVLSPGGASTVAKQVATNLEKLRPDLVTATMAKAVRRGKVFLDWSQNNPAKTTIAPYSLRGRAEPNAAAPRTWAEIEDAATLRHLRFDEVLARWRDDGDLLADLDPPLSRRGADALPGGADALAKYRSMRDPARTPEPVPAEPPSRGADNRFVVQEHHARRLHWDVRLERDGVLASWAVPKGPPTTPKQNRLAVHTEDHPLEYLHFHGVIPKGEYGAGEMTIWDTGTYETEKWRDDEVIVRFHGSKLTGRYALIQTNGNQWLMHLMREQPADAETPVGAETPVGAEDSGGPAVDAEIRDETAGDADAAGGRRSRTTAGAEVGRRGATPVPRGLSPMLAVSGEVGPLDGEQWCFETKWDGFRLIAEIDAGAVTLRSRAGNVVTDRYPRIAAVLAEELAGHRAVLDGEAVVFDEHNVAQVALLQADPARAEFVAFDVLYLDGTSLLRKRFADRRRVLEALGAAAPSLRVPPALDGPGAEALRYSEEHGLEGVIAKRWDSVYLPGKRGHSWVKQRNWRIQPVVVGGYRRSGARDFKSLLVGIPHDGELIYVGRVGTGFGEQDMTALARRLRGLERKTSPFANALTAEERKEAVWVRPTITGRVRFMNWTDTGRLWHPAWLGEDD

>CORE_REP|Org150_Gene1805#

MNRKTVFRNLAIVAGILLVIYLVSYFSNDTRGWKNVDTSVALTQLADKQNVKQVQIDDKEQQLRITLKQGNDATGGQNQIMAKYPGGSEVSAQILRDVQQSGAPFNTAVKQDSWFTQVLLFVLPMVILLGLFVFVMARMQGGGRGGMMGFGKSKAKQLSKDMPKTTFADVAGADEAVEELYEIKDFLQNPARYQALGAKIPKGVLLYGPPGTGKTLLARAVAGEAGVPFFTISGSDFVEMFVGVGASRVRDLFDQAKQNSPCIIFVDEIDAVGRQRGAGLGGGHDEREQTLNQLLVEMDGFGDRTGVIIIAATNRPDILDPALLRPGRFDRQIPVGNPDLAGRRAILRVHSQGKPIAPDADLDGLAKRTVGMSGADLANVINEAALLTAREHGNVITGPALEESVDRVIGGPRRKSRIISEHEKKITAYHEGGHTLAAWAMPDIEPVYKVTILARGRTGGHAMTVPEDDKGLMTRSEMIARLVMAMGGRAAEELVFHEPTTGASSDIDQATKIARAMVTEYGMSARLGAVRYGQEQGDPFLGRTMGTGSDYSHEVAREIDEEVRNLIEAAHTEAWAILNEYRDELDALAIALLERETLHRKDLEQVLATVDKRPRITAFNDFGERVPSDRPPVKTPRELAAERGESWPEEAETRKPNQQPAPQPVPANGYPQEGHRNPAPAPTGYSYPAPQPGNYPRPAGGPGYPRQSTHGSRPDYGAPAGWSAPGWPPQEDPSAGGSPQQPEYGGAPQGGGSQGYGEPQRYGEPQRYGGGHRRPEWPGDQDGSTQNGTQDHGDWDGPNSHR

>CORE_REP|Org121_Gene5397#

MSSATRANSKQAARHAADSHDAIRVVGARVNNLKDVSIELPKRRLTVFTGVSGSGKSSLVFSTIAAESQRLINETYSSFVQGFMPTLARPEVDVLDGLTTVITVDQQRMGSDPRSTVGTATDANAMLRILFSRLGKPHIGSPQAYSFNVASISGAGAVNIERAGRTIRERRSFSITGGMCPRCEGRGSVSDIDLTQLYDDSKSLAEGAFTIPGWKSDSFWTVRVYAESGFVDPNKPIRKYTKRELNDFLYKEPVKVKVDGVNLTYEGLIPKIQKSFLSKDKESMQPHIRAFVERAVTFTTCPDCAGTRLSEEARSSKIKRKNIADLCAMEIRDLAEWVRALKEPSVQPLLDSLVATLDSFVEIGLGYLSLERPSGTLSGGEAQRVKMIRHLGSALTDVTYVFDEPTIGLHPHDIQRMNDLLRQLRDKGNTVLVVEHKPETIAIADHVVDLGPGAGSGGGTICYEGSVEGLRASGTITGRHFDDRAALKESVRKPTGALEIRNATRHNLRGVDVDIPLGVLCVVTGVAGSGKSSLVHGSIPASEGVVAVDQTPIRGSRRSNPATYTGLLEPIRKAFAKANDVKPALFSANSEGACPNCNGAGVIYTDLGMMAGTASTCDVCEGKRFDASVLDYHLGGRDISEVLAMSVREAEEFFGSGEARISAAHAILARLVDVGLGYLTIGQPLTTLSGGERQRLKLATHMADKGGVYVLDEPTTGLHLADVENLLNLLDRLVESGKSVIVIEHHQAVMAHADWIIDLGPGAGHDGGKIVFEGTPAELVAARSTLTGEHLADYVGA

>CORE_REP|Org141_Gene5255#

MVSGSLLLCGIDLTVLHVAVPSVSRDLRPSAAQLLWIVDVYSLALAAMLVTCGTLGDRVGRRRMVLSGFLTFGLASAACALSTSTAQLIAARAALGVGAAMIMASTVAIIRVVFTDGRERAFAIGVWTSAHSVGATIGPLVGGLVAERWGWNAVFLVNIPVIIVILAVGARVIPESKNPAPRRWDLASVALSIAGLASVVYALKQAGEHAGVSTAILVTALSGAALLYAFVHRQRRLAEPLLDLSLFADRRFATAAVCVIGCFGSYVALLFFLTQWLQQVGGYSPLHAGLALMPLAAANAVGAVTAPRTASRWGNRGALTAALLLFALAYAVIAAVGDTAHYGTILPALLAAGYGAGIVMTLGADAIMSAAQPERSGEAAAIQETSFELGAGLGVAVLGTVMTVVYRTGMPHVPGLGPDERVIVGESFTAAQDLTAHLPSATADAVLDAARQSYDHGFTTIAVIATVTLVITAAMAAVLLRCKQNEPRNYRFQGGQASVPDTTHPPVTVLGLGAMGQAFVATLLKGGRTVTIWNRTPGKDAELVTAGARTTAAVDEAVTASPVIIAVLLDHRSVHSTLDPIADQLAGRQLINVTSTTAEESRELAFWAAGHGIEYLDGGIMAAPSMIGQPGASILYSGSRAVFDDHRGTLDLLASAEYFGTDAGMASMLDFSLLSAMYGMYGGFFNGVAMTRSVGVSAEAYAERAAAWVKAMTDYLPMLGKLIDARDYENGVQDIAFHKAAVDAIVRATRDAGAAPDFLAPLQHLIDRQIAEGNSALAFEHTVEEIV

>CORE_REP|Org45_Gene1385#

MATIEYLRTDPALPPVGIVDRSPLTPAKKGIFLAIAVLGAIAWAIVAFMRGENVNAVWIVVAAVSTYILAYQFYARLIQWKITKPRDDVATPAEAMENGKDFMPMDRRVLFGHHFAAIAGAGPLVGPVLAAQMGYLPGTIWIIVGVVLAGAVQDYLVLWASSKRRGRSLGQMARDELGPVGGVAAIVGVLVIMMILLAVLGIVVVKALAATENPVTGALEGGSPWGVFSIAMTIPIALFMGIYLRFVRPGKVGEVSVIGFTLLMLAIVSGNWVAESGWGRDWFTLSAATIGWLLIFYGFFASVLPVWLLLAPRDYLSTFMKIGTIFLLAAGILITMPVLKAPAVSEFASNSDGPAFAGSLFPFLFITIACGALSGFHALVSSGTTPKLLEKQSQARMIGYGGMLMESFVAVMAIVTASIIDQHLYFAMNASGALTGNTPETAAAYVNSLGLQGDPITAAQLTQAAEDIGEKKIVSLTGGAPTLAVGMSEVLHQFLGGAGWKAFWYHFAIMFEALFILTTIDAGTRVARFMVSDSLGNLGGPFTRFKDASWRPGAWLCSAIVVAAWGSVLLMGVTDPLGGIYTLFPLFGIANQLLAAIALTVVTVIVVKKGLLKWAWIPALPLAWDLIVTMTASWQKIFSADPKIGYWKSHDNAKIKLDAYEAARDSGQLPAGVADAAALEKQISDLEKIVRNTFIQGTLSIIFAVLVLIVAVVGVLVCVRAIRRGGSETTESPEEPSKIFGPSSFLATKAEKEVQQEWDELIASGQIRAPGARSGVHAH

>CORE_REP|Org87_Gene3703#

MTQTSERKSSAVEVEPGVFESVALIDNGSYGTRTVRFETGRLARQAAGSVVAYLDDETMLLSATTAGKTPKDQFDFFPLTVDVEERMYAAGRIPGSFFRREGRPSTDAILTCRLIDRPLRPSFVDGLRNEVQVVVTVLSLDPKDLYDVVAINAASASTQIAGLPFSGPVGGVRVALIPDQGANSAGGGQWVAFPTVEQLEGAVFDMVVAGRVVESGDVAIMMVEAEATEKVIELVEGGAQAPTEAVVAEGLEAAKPFIARLCRAQQDLAELAAKPTEEFPLFPPYGPDVYEAVEGAAEAELGEALSIAGKQEREEKIDEIKLAVLDRLAEQFAGREKELGAAFRSVTKKLVRQRILTDGFRIDGRGLADIRALSAEVAVVPRAHGSALFERGETQILGVTTLDMVKMAQQVDSLGPETSKRYMHHYNFPPFSTGETGRVGSPKRREIGHGALAERALIPVLPSQEDFPYAIRQVSEALGSNGSTSMGSVCASTLSLLNAGVPLKAPVAGIAMGLVSDTVTNDKGEQEVRYVALTDILGAEDAFGDMDFKVAGTREFVTALQLDTKLDGIPSQVLAGALSQAHDARTTILDVMAEAIATPDEMSPYAPRVTAIKIPVDKIGEVIGPKGKVINQITEDTGANISIEDDGTVFVGATDGPSAQAAIDAINAIANPQLPKVGERFLGTVVKTTAFGAFVSLLPGRDGLVHISKLGNGKRVAKVEDVVNVGDKLRVEIADIDNRGKISLVPVDENADEPAADAVDAGTE

>CORE_REP|Org125_Gene2067#

MATLSDRLDHVLGVKAAEPLADAFDMHIVEDLLRHYPLRYATQGQPLTEEAPEEGAHITVVGRVRKTELRPMRQRRGKLLKVELDTGSAKPVEITFFNGDKVSYLVKQGVRAMMSGTVHWWRPDRWNLSHPSYLILPETAESVDSLTSVRGGGALRGLAESAKGAGGVDISFFEREYIPVYPATAKVQSWDILACVRQVLDQLDPIDDPLPADLREDHELLPVSDALRLIHLPEHKSDIDQARQRLRFDEALALQLVLAQRRHDAAGRTARPCPPRSDGIAAEFEQRLPFELTAGQNKVIAEISGDLSRPHPMHRLLQGEVGSGKTIVALHAMLQVVDAGLQCALLAPTEVLAAQHYRSLRSMLGDLGAAGELGAADRATKVVLLTGSMSASAKKAALLDVVTGTAGIVIGTHALIQDAVEFFDLGMVIVDEQHRFGVEQRDALRAKAKDGITPHLLVMTATPIPRTIAMTTLGDLETSTLTELPRGRSPITTRVVPARMKPAWVERAWERIREEVAAGRQAYVVCSRIGDEEDDGAPKKGAKSKGKSRKQADEGTGEAPATHAAIDVFDTLRTGPLADLRLGLLHGRLPTDDKDRVMRSFNDGDIDVLVCTTVVEVGVDVPNATVMVIVDADRFGVSQLHQLRGRVGRGKHPGLCLLITETSPMGTAMARLEAVAGTLDGFELSVLDLRQRREGDVLGSAQSGTARSLKLLSLLDDLDVITTAQVLAREVVDADPGLTDHPGLANMMHAAVDSERLEYLAKS

>CORE_REP|Org113_Gene4327#

MERVTTTELRESAEGLLRELAGPEARLREDQWTAIEALVVHRRRALVVQRTGWGKSAVYFIAARLLRTQGRGPTVIVSPLLALMRNQVASARRAGVVAETINSGNVTDWDEIHARVASGEVDVLLVSPERLNNPDFRDSVLPKLAADAGLVVVDEAHCISDWGHDFRPDYRRIRTLIADLGEDIPVLATTATANDRVVTDVATQIGTDTLVLRGTLDRESLYLSVVRIPDAVQRTTWLSRQLAELPGSGIIYTLTVAAAHDLADVLTDHGYRVAAYTGRTDPGERETLEQALLDNEVKALVATSALGMGFDKPDLGFVVHIGAPSSPIAYYQQVGRAGRGLGTAAAATPTDTRGTAPSTNAHTMPDTGQPGTPADTQGTAPDTNVRAVPDAAPPGASVDPHADTSGTNVRAEVILLPGPEDRQIWNYFASVAFPREPVVRSVLAALDYERPLSTVALEPLVELSRSRLEMVLKVLDVDGAVHRVRGGWLATGQEWAYDTERYERLDRARAAEQQAMLDYQSTSECRMNFLRHQLDDPGLAADAPGCGRCDNCTGHRFDVEVGADEVAAIRARLDRPGIDLAPRKQWPTGLSKLGIPLSGKISDGPETGRVLGRLSDLGWGQRLRTLLDAPDGPAPDHIVDACIAVLREWDWTQRPGAIMALQSATHPKLAADLSARLAQIGRLTDLGVLHTRPDRPPVSAANSAHRVAALFDSWEPPDLTGLDTPVFLVDTHTDTGWTLTLAARTLRLAGAPAVLPLALATPT

>CORE_REP|Org98_Gene6070#

MSTDTDATPISDIPPGSAWPERLSSISRHDLPASIVVFLVALPLSLGIAIASDAPIAAGLIAAAVGGIVVGFLGGSPMQVSGPAAGLTVVVAEVIHQFGWQTTCFITAAAGLLQIVFGVSRIARAALAIAPVVVHAMLAGIGVTIALQQVHVLLGGASRSSAFENITELPGQLLTPHGDDFVIGLIVIGIIVAWRRVPDKVRLIPGPLVAVLVGTVLSLVLPGNPDRIKLDSSLFDAIGLPALPSGNWGGVVPAVLTIALIASVESLLSAVAVDKLHTGGRTNFDRELLAQGAANMTSGMLGGLPVTGVIVRSSTNVAAGARTRASTILHGVWILVFSIALVGVVQQVPKSALAGLLIVVGVQLVKLAHIQLAHRTGDLAVYAVTMVSVVFLNLLEGVLLGLAMAFAMLLWRVVKVSVRATPVAGTDRWIVRVDGTCTFLALPKLTKELATVPAGTDVLVELTVDFLDHAGYEAIHDWARQHESTGGNVEFVEIGTARMEHAMTRPPRRGRARGILDEVLGPWRERNGDAVAAGVAAYHRSHAHVMRPHLDQLRDRQDPHSLFLTCADSRIVPNVITNSGPGDLFTVRNVGNLHPADGSDASVEAALSFAVDNLKVHNVVVCGHSSCGAMKTLLAGTSAGPGLDTWLAHARPSLDAYRAGHPVRAVAAAAGYDETAQLSMVNVAVQLEILQRHPVIRQAAAARGLTVSGLFFDISTACVLEVTTDSISEIGRGPLKGAIDADRGKSGSTGIRPTRSPAQSRTA

>CORE_REP|Org77_Gene3469#

MTIATTDEHKAVQESMSGWAAAVRPIATMRDDTTGFWRMYWGQLTDLGIFRVAVDEQAGGAGGSITDLAVLVEQAAHDLVGGPVLTTALAGVVTGGRLDEQQPCGVALDTVVESASSLSAVDRPRQGDGGELVLNGVWETVLGAAPGAAVLLPVRIADGSRWCLIPAEAPGVTIEPLPALDPSTPLARVRCAEVQVPAADVFAPDFAVEDLIVALTTAELAGVAGWCLETAVEYAKVREQFGKPIGSFQAVKHICAWMLCRTELIRSVAADAAAAADDALANPAGSELPIAAAIAAAISLDAAVDTAKDCIQVLGGIGFTWEHDAHFYLRRATALRQLLGGGGYWRARVTELTRAGARRTTGADRILADVATETASSGEGVGAAVGVAAAVGNAAAVSLELGAAATGVAAEVAAIAALPVDQQRKAMVEAGLVMPHWPEPYGRAADPMTGLLISEELARAGLETPDLAIGGWAVPTLLQHGTPEQMERFVWPTLHGDVVWCQLFSEPGAGSDLAALRTTATKVDGGWMLRGQKVWTSLADTANWGICLARTDPAAPKHKGISYFLVDMRSAGIEIRPLVQITGEAKFSEVFLDDVFVPDECVVGALGNGWKISRATLSAERIAMGGKGIGQVLEELVGKLPATGPGTELINDRLGRFVADATAGLLLEQRGAALILAGADAGPQSSVRKLVGVRHRQEVAEFAVEVAGPAGALDNEATKEFLLTRCLSIAGGTEQILLTLAGERILGLPRDGS

>CORE_REP|Org45_Gene2052#

MSTVEGRTRGALLARQAGGALGAAVLAAVVAAAVAGVGLVAFASVEWPAFNSSNVTRALTTVGQVVAVAMLVAAIWLVRARKWPWVAKVLSWGGISAFVTVTLGMPLGATKLYLFGLSVDQEFRTQYLTRLTDSAALRDMNYVDLPPFYPAGWFWAGGRFANLFGLDGWEAFKPWAIIALAVAAALALVLWSELIRADWAIAVAAATTAVTVAYAAPEAYSAVLVVLLPPVLVLAWGALHRPAEYGGAEYQTDAARATGSKAADSGAVSTDAAGAKVAGAKAVGSGAGSSETAGTSATGSGAASPGVASTSTASTEAASTSTASTGATASGMVGAVPTAGGWGAVLGTGLFLGLAATFYTLYFAAAVFAVCLMAVAAAGIALWQRHFALRVHRRTKREVPGVWRLLWPILLRLIAIGVVAGLLALVVWLPFLLRVLDEGFPSSGTAFHYLPEGGARLPLPMFEFSLLGGLCLIGVIWLVLRVGSSRRAQALAVGVVAVYLWCLLSMLVTAAGTTLLSFRLEPVLMVLLAAAGAFGFVEGARAIYQVLNEPERFRAVVAAVAVLGAIGFGQQIPEILAPEITTAYTDTDGDGVRADKRPPSAVSYYDEIDAALREQIGRPRDETVVLTADLSFLSIYPYFGFQALTSHYANPLADFPARADEIKRWSTLETPEELLDALSTAPWRAPDAFLFRQSGDNYTLRLAEDVYPNQPNVKRYQVTFPATLFDDPRFTVTDIGPFTLVVVEH

>CORE_REP|Org97_Gene3126#

MIVSVSETETVPQQPRLPAAPPAATPPPTTAAAAQLPADRYLNRELSWLDFNARVLALAEDPSEPLLERAKFLAIFSSNLDEFYMVRVAGLKRRAEAGLSVRSADGLSPTEQLTLIAERTQELAGRHARVFLDQVRPALADEGIAIIGWADLDDDERRRLSGYFLDQVFPVLTPLAVDPAHPFPYISGLSLNLAVTVKDSETGGEHFARVKVPDNVDRFVRVRRTLNETPGARRDSDITAVPRLAAFLPMEDLIAAHLDQLFPGMEVVEHHSFRITRNADFEVDEDRDEDLLQALERELARRRFGSPVRLEVSDDMTEHMLELLLRELDVDPGDVIQVPGLLDLSCLWQVYGVDRPLLKDAPYVPATPPAFGERETPRNVFAALREGDVLVHHPYDSFSTSVQRFIEQAAADPQVLAIKQTLYRTSGDSPIVNALIDAAEAGKQVVALVEIKARFDEQANIKWARALEQAGVHVVYGLIGLKTHCKTCLVVRREGATIRRYCHIGTGNYNPKTARLYEDVGLLTAAPEIGADLTDLFNSLTGYSRKANYRNLLVAPSSVRSGIVERIRRETELAAQGVPARIRLKANAIVDEQIIDALYRASQAGVPVQIVVRGICGLRPGVPGMSDNIEVRSILGRFLEHSRILHFQAQDEYWIGSADMMHRNLDRRVEVMAQVKDPKLRERLAVVFDSALDPATRCWVLQPDGSWAAQPSPDADSRGVQVRDHQEFLMRLRRPDQQ

>CORE_REP|Org46_Gene6654#

MASSCSPPNKPLPGDRAGHHGVAVTGRTTRVAPQAATARAASRSSGASTSERQETVAYRADLDGLRGVAIGLVVIFHVWFGRVSGGVDVFLVLSGFFFTGLLLRRADSTGSPGVGTTLRRTVRRLLPAMVVVLAAVVVASVIVRPYTQWWELSAQTLSSLLYVQNWRLALTWSDYLAADPSVSPLQHLWSMSVQGQFYLAALATVAVAAWTTRRSMRSAALRPVLAVTVGVLGVVSFWYAWRGGQTQQGWNYYDSIARCWELLAGALLAAIAPLLSPPRMARAGLAALGLFGVVGCGWLILDGANRFPGPAALLPVAAAAGVIVSGNNLPLDQRPWPNRILATPTARWLGDIAYPLYLWHWPILIFYLTERGQPHAGVAGGIVIVTLSIVLAWVTHRWVEEPLRLRSRPRAEAAGAEGTTISRRVAGVAVVALGAVVIAAAGGWLTVMARINPPHAVGALDPRLYPGAEALASGAAVPQAPMRPTVFEAPGELPPPTVDGCIADWDTREVITCTYGVPDAERTLAVVGSSHAEHWLPALQVLAGEYSFRIQVYLKMGCPLTLAEDAMYKGEPIPDCRDWSREVIDRLGADRPDWVFTTGTRPREDIGDETPPEYLDVWSALSERGLNVIAIRDTPWLRREKVRYMAIDCLAKGGDRIGCGMRRQDALDEVNPALEPASRYPNVFPVDLSDAVCEPTVCAVIEGNVLIYHDEHHFTVSYSRSLADALGRRLQPLLGWW

>CORE_REP|Org7_Gene5304#

MALDSGTGTISDLGGVAAPIAKTVPTERAHHGDVFVDEYEWLRDKENPEVISYLEAENAYTEAQTAHLAGLRDSIFDEIKSRTQETDLSVPTRMGDYWYYSRSFEGKQYGVHCRCPIAADAEGIDAWTPPQLEAGTEVPGEQILLDSNVLAEGHDFFALGAYSISHDGNLLAYSVDTNGDERYVLRFKDLRTGDLLPDEVAETAPGATWSLDGTHVFYQTVDESWRPDTVWRHRLGTAPDADVKVFHEPDERYWVSVVSTRSEKFLMIWVGSKITTEGWVLESDNPEGEFRVILPRREGVEYSAEHAVVGGEDRFLILHNDVVDGVKAENFVLADAPVADPSNLTLLIGHRDDVRLEDVDAFADHLVLSYRREALTRVTVWPLTESGYGERKELDFDLELFSVGAGANPEWAQPTLRIGLSSFITPVQVFDYVPATGDLLLRKEQPVLGGYDANDYEQHRDWAVAEDGTRIPISLVWKKGGLAHSRLASAPGVPLDAPKPLLLYGYGSYEASMDPSFSVSRLSLLDRGMVFAVAHVRGGGEMGRLWYENGKTLTKKNTFTDFVSCARHLIDTGVTAADRLIADGGSAGGLLMGAVANLAPELFTGILANVPFVDPLTSILDPSLPLTVIEWDEWGNPLADKDVYDYMKSYAPYENIEAKDYPAILAITSINDTRVLYVEPAKWVAKLRATKTGDAQLLLKTEMSAGHGGVSGRYEKWKEVAFEYAWVLDRVGLAGA

>CORE_REP|Org103_Gene2295#

MTTSAIHHTRDSGGIVTLTIDDPNQRVNTMNSLFVESLAAELDAIENDADVTGVILTSAKKTFFAGGDLNDLRAARRDRIDEFAAFVQRNSVLLRRLEKLSVPVVAAINGSALGGGLELALAAHHRIVVDAPGVTLGLPEVTLGLLPGAGGVVRTVRLLGVQAALRDVLLSGKKHPVAGALELGLVDATVATIEELIPAATAWIREHAGARQPWDTEGFRIPGGAPGERGAPLHATLPALAATLRAQTKGAPAPAQANILAAAVEGAQVDVDNALAIEARYFLDLAIGQIAKNMIQANFFDMQVVNGPRGRDTAREPWLPRKAIVLGAGMMGAGIAYQCAVSGIDVVLKDVTPEAAERGKGYSLRVLDKRVRAGQISAATRDEVLARITPTADVAAAAGADLVIEAVFEDPALKADVLREIEPLLAPDALIGSNTSTLPITGLAENVSAPDRFIGLHFFSPVDRMPLLEVIKGGRTSSETVSRALDLARTIGKTPIVVNDSRGFFTSRVIGTFVNEALAMLGEGVPAPVIEQATTQAGYPAPALQLADELNLELLRRVRDASRVAAEAAGGSWDPHPAEAVLDRMLGEFGRAGRLAGSGFYEYEDGARTRLWPGLRAAFGSPRADLPFTDLKERMLFVEAIESVKCLDEGVLESVPDANIGSLLGIGYPGWTGGVLQYIDGYPGGVAGFVRRAEELAAAYGARFAPPSSLVAVARDGGTLADAHRERQPALS

>CORE_REP|Org101_Gene3640#

MAFATEIPTEGYEDRAAGATPLAHSEHRPVGEIERTEGQFQVVSDHQPAGDQPAAIAELERRITAGERDVVLLGATGTGKSATTAWLIERLQRPTLVMAPNKTLAAQLANELREMLPNNAVEYFVSYYDYYQPEAYIAQTDTYIEKDSSINDDVERLRHSATSSLLSRRDVVVVASVSCIYGLGTPQSYLDRSIQLEVGTEVDRDALLRLLVDVQYTRNDMAFTRGSFRVRGDTVEIIPSYEELAVRIEFFGDEIEALYYLHPLTGDVVRQVEMLRIFPATHYVAGPERMERAVRDIEAELEERLAELERQGKLLEAQRLRMRTQYDLEMIRQVGFCSGIENYSRHIDGRPAGSAPATLLDYFPDDFLLVIDESHVTVPQIGGMYEGDMSRKRNLVEFGFRLPSAVDNRPLTWEEFADRIGQAVYLSATPGPYELGQVGGEVVEQVIRPTGLVDPQVVVKPTKGQIDDLVHEIRVRTERDERVLVTTLTKKMAEDLTDYLLGLGVRVRYLHSEIDTLRRVELLRQLRLGEYDVLVGINLLREGLDLPEVSLVAILDADKEGFLRSSTSLIQTIGRAARNVSGEVHMYADKITDSMQHAIEETDRRRAKQVAYNTEMGIDPKPLRKKIADILDQVYKEADETEVEVGGSGRNASRGRRAQGEPGRAVSAGVYEGRDIKSMPRAELADLVKELTAQMMNAARELQFELAGRLRDEIADLKKELRGMDAAGLS

>CORE_REP|Org81_Gene2367#

MTERIQVGGLQVAKVLHDFVENEALPGTGVDSAAFWSGAEAVINDLAPRNRALLTERDDIQAKIDEWHRANPGTGYDKAAYKQFLTEIGYLRPEPADFQIGTENVDAEIATTAGPQLVVPVSNARFAINAANARWGSLYDALYGTDAISEENGAEKGTGYNKVRGDKVIEWARNFLDDAVPLITGSHVGSTKYSIEDGELVVGLEDGTDIGLADPSALVGYLGDPANPTSVLLKHHGLHIEIQIDPSSPIGSTDTAGVKDVVLESAVTTIMDFEDSVAAVDAEDKVLCYHNWLGLMKGDLAEEVSKGGKTFTRTMNPDRVYTALDGSQLVLHGRSLLFVRNVGHLMTSDAILDADGNEVPEGIMDGLLTVLIAKHALNGDTKLKNTRTGSIYIVKPKMHGPDEVAFTNELFDRIEQVVGLPANTLKVGIMDEERRTTVNLKACIHAAKDRVVFINTGFLDRTGDEIHTSMEAGPMVRKAEMKGQQWILSYEDFNVDTGIATGLPGKAQIGKGMWAMPDLMADMLVQKVGHPKAGANTAWVPSPTAATLHATHYHLVDVVKRQAEIAKGGARASVDQILEIPLAADTNWSAEERQQELDNNSQGILGYVVRWIDQGVGCSKVPDIKDVGLMEDRATLRISSQLVANWLRHGVVSEDEVIASLERMAPVVDRQNAGDPSYRPMAPDFASSIAFQAAKELVLEGTKQPNGYTEPILHRRRREAKEYNAKFGA

>CORE_REP|Org79_Gene341#

MAQEVLKDLNKVRNIGIMAHIDAGKTTTTERILFYTGINRKVGETHDGGATTDWMEQEKERGITITSAAVTCFWNNNQINIIDTPGHVDFTVEVERSLRVLDGAVAVFDGKEGVEPQSEQVWRQAAKYDVPRICFVNKMDKLGADFYYTVGTIVDRLGAKPLVMQLPIGAEDDFDGVVDLIDMKALLWPGKVETGTPPQIQEIPEDLKEKAEEYREKLLETVAESDEELMEKYFGGEELTKEEIQAAIRKLTIASEVYPVFCGTAYRNKGIEPILDAVVSYLPSPIDIGEVHGTSVDGEEDLTRKPSVEEPFSALAFKIAVHPFFGKLTYVRVYSGQAIPGEQMLNSTKSKKERVGKLFQMHANKENPVEHADAGNIYAFIGLKETTTGDTLCNPDHPIILESMDFPDPVIQVAIEPKTKADQEKLGTAIQKLAEEDPTFTVQLDEETGQTVIGGMGELHLDVLVDRMKREFKVEANIGSPQVAYRETIRKKVESLDYTHKKQTGGSGQFAKVIVTIEPYSPDPEELEEGESASYKFENAVTGGRVPKEYIPSVDAGIQDAMQYGFLAGFPLVNIKATLEDGAYHDVDSSEMAFKLAGSQVLKEAVAKAKPVLLEPVMAVEVVTPEEYMGTVNGDISSRRGQVFAMEDRSGAKVVKAKVPLSEMFGYIGDLRSSTAGRANFTMVFDSYAEVPQSVAQEIIDERNGNK

>CORE_REP|Org105_Gene3911#

MLAVTRSDCHRFTKLCDMADLAPSTVYIASPEGDTGKSTVALGVLQMLCATTARVGVFRPITRSTDEPDYILELLLEHSTADIEYAQAIGVTYEQVHADPDAAISEIVMRFHEVAKVCDAVVVVGSDYTDVASPSELRYNARIAVNLGAPVLLVVRGSERSPDEVKQLAELCSSELSAEHAQLVAIIANRCAPDQLDQVCAALSGFAVPSWTLPEVPLLIAPTMAELCAAIDGEMYSGDPELLHREAMKIMVGGMTAEHILERLEDGEVVIAPGDRSDVLLSVVNAHEAEGFPSLSGIIMNGGLLPHPAIARLMTGLKPRLPILTTSLGTYDTAGAAHRTRGRMSADNPRKVDTALALMEQHVDAGEFLRRLEVPRSTVVTPQMFEYQLIERARADRKRIVLPEGDDDRILRAAGRVLQRKIADLIILGDENAIRARAAELGVDIADAEVLDPRTSEHLEDFAREYTELRKHKGMTLERARETVTDISYFGTMMVHKGIADGMVSGAAHTTAHTIRPSFEIIKTVPGVSTVSSVFLMCLADRVLAYGDCAVVPDPSSEQLADIAISSAATAERFGIDPRVAMLSYSTGESGSGADVDKVRVATKLVRERAPQLLVEGPIQYDAAIEPTVADAKLPDSEVAGRATVFIFPDLNTGNNTYKAVQRSAGAIAIGPVLQGLRKPVNDLSRGALVADIVNTVAITAIQAQGE

>CORE_REP|Org163_Gene4874#

MRSATVQRDQARTRNAKESAAVTTSAPISPVALVRVPAGTTAGAAVREAGLPTKGPETVVVVRVDGELKDLSWTPDTDVDVEPVAANTDDGRNVIRHSAAHVLAQAVQQEFPGAKLGIGPYIKDGFYYDFRVERPFTPEDLAKLESRMKKIVKGAQRFSRRVVEVEDARVELAGEPFKLELISDKSGIDDPEVMEVGGKELTIYDNLDPRTGEKIWGDLCRGPHIPTTKFIPAFKLTRSSAAYWRGDQSREDLQRVYGTAWESQEALDEHLHLLAEAERRDHRKLGLELDLFSFPDELGSGLPVFHPKGGIIRKELEEYSRRRHVAAGYEFVNTPHITKGHLFEVSGHLDWYRDGMFPAMHLDAEFNEDGTVRKPGQDYYVKPMNCPMHNLIFRARGRSYRELPLRLFEFGSVYRYEKSGVVHGLTRVRGMTQDDAHIYCTKEQMHSELTDTLRFVLDLLKDYGLDDFYLELSTKDPKKFVGSEEIWEEATETLSKVASASGLELVPDPGGAAFYGPKISVQAKDALGRTWQMSTIQLDFNLPERFDLEYTASDGTKQRPVMIHRALFGSIERFFGVLTEHYAGAFPAWLSPVQVVGIPVAEAFAPHLDRVIERLQDEGVRAQVDRSDDRMQKKIFNNTAQKVPFMLLAGERDVNANAVSFRFRDGTQVNGVPVDDAVATIVAWLANRENASPTADGFEIRSSKGGA

>CORE_REP|Org101_Gene6880#

MRELVTDVIDVGADFPVARKALWDLFLEPQTYPRLFAGIGACELVEESPDSRIVRVRIGTAESGIRTHQLRLTVRRWYESFELQCPGTGSFVSVRLRGDEERTKIVVTVFAPGRLHPGIAEGSNAAVMSWVNAGLRRAVDVIRGARTSTVVNAENSPLRRQVSVAKQMVTVGVGRTSSLATGVKQARSLAKWGFNLAGGYATAAAYAPDRIAIIDDSGTRTFAEMHTRTSALAGALAALDLGFGDTIGLLARNHAGMVECMVAAGKLGVDVALLNVGLSGRQIEDIVQRHRLAALFVDGDLEQLVHYLHADLPRFNTDGRPPVPGRATLDDLIAEGERPFRLPNRAGRLIVLTSGTSGTPKGARRPEPKGFGTIAALLSRIPLPMAEPMLIPAPLFHTWGLAGLQISTPLRATVVLPERFDAEDCLRLVAEHRVASMIVVPTMVHRILDLPAAVRDRYDTASLRAVVSCGAPLAGATVLQFMDVYGDILYNVYGSTEVSWATIATPDDLRTAPTTAGRPPLGTRIAVLGPDQRPVPLGVTGHIFVGNHMLFDGYVNSAPPTEADGMLDTGDLGYLDVAGRLFIAGRDDEMIISGGENVFPRPVEEALAHLPQVSEVAVVGVPDQEFGQRLAAFVVKREGAGLDSDMIRSYIRHRLSRFSVPRDVTFLPTLPRGETGKIIKHLLTGGPPAEGSAQSPLGGPHLVT

>CORE_REP|Org63_Gene3321#

MTDTDLLATPGVESNPTGDRESESGQISKMIENTDVARSGLTGMLLPQLRALAGELGIRGTSGMRKGDLIAAIKENQAAGKSAKVEKPAKSEAKSAATAKADAPAKNAAPAESAPAKAEQATLDVTPAASAAPTEAPAPAKTAPADSAPAAESASSKATEATETTEESGRESGQRGRGRQRRGRDQARSASAETAPAETRADEPKQDSEQGGERRRERGQGERQSERGQGERGERGQGERGERGQGERGERGERGQGERGERGERGQGERGQSQNGSAGGRGGDDEEGGRGRRGRRFRERRRGRDRDGGGGEARELEIREDDVLQPVAGILDVLDNYAFVRTSGYLAGPNDVYVSMNLVRKNGLRRGDAITGAVRAPRDGEQANQRQKFDPLVRLDTVNGGDVEAAKRRPEFGKLTPLYPNQRLRLETQPNKLTTRVIDLIMPIGKGQRALIVSPPKAGKTTILQDIANAIATNNPEVYLMVVLVDERPEEVTDMQRSVRGEVISSTFDRPPSDHTSVAELAIERAKRLVEMGRDVVVLLDSITRLGRAYNNSSPASGRILSGGVDSTALYPPKRFLGAARNIENGGSLTIIATAMVETGSTGDTVIFEEFKGTGNAELKLDRKIAERRVFPAVDVNPSGTRKDELLLSPDEAAVLHKLRRVLSGLDSHQAIDLLIDRLKKSKNNLEFLMQVSKTAPGALDE

>CORE_REP|Org51_Gene2871#

MWSGARKSCRPCGRPDPSDRRENRPVSSYPHLFEPLDLGFTTLRNRVVMGSMHTGLEDRAWDIDKLAAYFAERARGGVGLIITGGYAPNRTGWLLPFGAKLTTTTEAYRHRTVTRAVHAHGAKIALQILHAGRYSYLPGSVSASSIKAPINPFRPRKLSARGIEQTIRDYVRCAELARLAGYDGCEIMGGEGYFLNQFLAPRTNKRTDEWGGSAANRRRLPLEIVRRIRAAVGPEFILIFRLSMAELVEGGQTFAEIRELARELERAGATIINTDIGWHEARVPTIVTSVPRAAFVEFTAKIAREVSIPVCASNRINMPEVAEEILTRGDAQLISLARPLLADPDWVAKAAGGREDEINTCIACNQACLDHAFQRKTVSCLLNPRAGHETDLVLAPTRRTKHIAVVGAGPAGLAAAVNLAERGHRVDLFEAEDRIGGQFDIARRIPGKEEFEETLRYFDRMIAKTGVRLHLNTRATAEDLLAARYDEVVLATGVRPRVPDIPGIDHPMVLTYAELVREAKPVGRRVAVIGAGGIGFDVGEFLTVDGHPTLKLDEWKQEWGVDADDERAPGQLRAPRPAPAAREVVLLQRKDSPFGRSLGKTTGWVHRAALRAKGVEQVGGVNYERIDDEGLHISFGERRARPRVIRVDNVVVCAGQESVRELAEPLRAAGVRVHLIGGAELAAELDAKRAIDQGTRLAARL

>CORE_REP|Org9_Gene5213#

MTTPRRPRRTASRPAGAPDPAEQRRAAEAAAERAESARPAEPAAEPAAEPAADRPGGSAGEPTAPAGTDQPTAPAKAERPARGDRGDARTQRQDKPARGDSGEGRSAGGRRSGRGGRQGRGRAEQLPAQPPVVAAQDRLGAPPKLPKNGLRVFALGGIGEIGRNMTVFEYGGKLLIVDCGVLFPEDQQPGVDLILPDFRPIEDRMDDIVAIVLTHGHEDHIGAVPFLLRNRSDIPVLGAKFTLALVAAKCREHRLHPKLIEVTEGETTSHGPFECEYFAVNHSIPDALAVAIRTPAGVALHTGDIKLDQLPLDGRLTDLAGFSRLGDEGVDLFLVDSTNAEVPGFVTPEREIGGVLDTVIGKARGRVIVASFASHVHRIQQVVDVAQKYGRRVCFVGRSMVRNMQIAQDLGYLTVPDGVVVDLDVAATLPGDRLVLISTGSQGEPLSALSRMARGDHRQINIRPDDLVVLASSLIPGNENSVFAVVNGLARLGASVITQQNAKVHVSGHASAGELLYLYNAVRPTNAMPVHGEWRHLRANAALAVATGVPEERVVLAEDGVVVDLVDGIASIVGRVPVGHVYVDGLSVGDVGESTLSDRLVLGEGGFISITVAIDETTGKAVSAPELSGRGFSDDPTALAEAAELVEAELLRLAGEGITDTHRIAQGVRRVVGRWVADTYRRRPMIVPTVIGV

>CORE_REP|Org141_Gene5357#

MDESAGARPAPRRPRGSCARRFRAGVGCDHRRRYHLGVTEHVEQLEFQAETHQLLELMIHSVYSNKDTFLRELISNASDALDKLRLESYKDKDLHVDTSDLHIELEVDTDGRVLTVRDNGIGMSRAEVVDLIGTLAKSGTAQLRKQLSEAKSEAAAEELIGQFGIGFYSTFMVADKVTLTTRRAGETEATRWVAEAGSSTYSIETVEDAPQGTAVTLQLKAADEDDHLFDYTQEWKLREIVKKYSDFIAWPIRMQVERTVTEGEGEDAQEQTVVEEQTLNSMKALWTRPKSEVSDEEYHEFYKHVSHAWDEPLEIIPLKAEGTFEYQALLFIPSQAPFDLFTREHKRGVQLYVKRVFIMDNCEELMPEYLRFVKGVVDAQDLSLNVSREILQQDRQIQMIRKRLVKKVLSTVKDVQGAEDQDNYQTFWREFGRVLKEGLLSDFDNRDTILAVSSFASTASESDLATLAQYVERMKDGQGSIYYMTGESRQQVESSPHLEAFRAKGLEVLILTDPVDEMWVGSVPEFDGKPFVSIAKGEVDLETEEEKKESEQLREQQDKDYAELLGWLGKTLADSVKEVRLTNRLTTSPACLVGDVFDFTPMLERMYRASGQALPETKRILELNPTHPLVTGLREAYDTRKQDADEGKVPELGETAELLYGTAVLAEGGELKDPAKFAHILTDRLTRTL

>CORE_REP|Org110_Gene6706#

MSSALDPSASPDLESNRPAATYAVVIALSAAIAAVVAALVVGLSAAQALSLLGIPDPGALTTYGLPAVRALADLSAALTVGSLLFAAFLVPPQASGLLDVGGYRAVRRASNFALLWACCAALLIPLTVSDTTGQPVRDTLDPVGLWRAIDQIELAGAWRTTVLFALIVAVGARLALRWGWTPVLFGAAIATMMPLALTGHSSSGGAHDVATNSLILHLVSAAVWVGGLFALLAHARRGGAHTDLAARRFSLTATFAFATIGVSGVINSWVRVPWDELFTSTYGRLVLAKAAALVLLGLFGYAQRRAALPALAADPKDRGALIRFAGVEVLVFAATMGLAVGLGRTPPPPPTSIPTPAEVELGYNLAGPPTVARMLFDWRFDLIFGTLAIILAIGYLLGVRRLRARGDAWPIGRTIAWLSGCVVLLLATSSGVGRYAPAMFSVHMGAHMALSMLAPILFALGGVVTLALRALPPAGRGGAPGPREWILAAVHNPVSRFLTHPIVASVIFVGGFYALYLGGIFDTFADSHGAHLLMNLHFLLSGYLFYWVVIGIDPKPRQVEPLTKLAMVFGSLPFHAFFGIALMSMTTVLGGWFYRGLGLGWNGDLLGDQRTGGSLAWASGEVPLVVVMLALLIQWSRSDKRLAQRTDRAADRDHDADLAAHNAMFAELAKRDRGPQKSAGDPAQP

>CORE_REP|Org158_Gene1247#

MIGQILEGRYRIDAPIARGGMSMVFRGEDTRLDRPVAIKVMDPKFAADPQFLTRFELEARAVAKLKHPALVAVYDQGVDGDHPFLIMELVEGGTLRELLRERGPMPPHAVRAVIEPVMQAIGVAHSSGLVHRDIKPENVLISDSGEVKIADFGLVRAVAAANITSASVILGTAAYLSPEQVTSGHADARSDVYAAGVLIFEMLTGRTPFTGDNSLSIALQRVENDVPSPSHHISGVPPEFDELVAHATAREPAHRFADGNEMAAEIRRIAQVLQLPAYRVPAPQESAEHLSARYRVGPTPAPAAPAESRSRPAPVGAADMTTRLPAEPPTTRVPQAAAPPAHQHTRVMTAARELPPDYAQSAAHAPPPTLPPHGDQPPRNGYLADRGRSRRTAVLWLGAVVVLALLLGIGGWWLGVGRYEAVPAIAGMDRERAVATLQAAGFDTEVRDKASDTIPMGNVVGTDPSAGTKVVKGSTVAVLISSGKPKVPDIRPGQDVQSVKQAIRDAGLTPVDAGEVSSTAAEGTVAKVDPDPGTILPMGADVKVYTSKGSAPVELPNVRGKTEEEARAALDAVGIEVTGTRVEFDSKVKAGEVAGTDPAAGTTIDSSQGVVLLISNAVEVPGLLGSSVGDARAKLEALGLGVSVRQLAPSDSSIVISQSSVPGAKVEPGSTITLVALP

>CORE_REP|Org109_Gene6329#

MSPPLPWAPSPCILRSSARRSATAYCGRGGYAEYLPRFDTVRRATSSTGGALAVSSAPITARSRRFPRRRQESATGLDRTVEFPSVKVGAGKRAAWRTLLPAVAVLTLISSCAANPPPPIESTDSPKTTPVKPAETTVVVALDTLGTAFNPHLRSDQSPATSAIASLVLPSPFRPVLDPARPGATAWVPDSSLLISAEVTAQEPFTITYKLRNEASWSDGAPIAAEDFRYLWQEMISEPGVVDPAGYRLISDVNSSAGGKTVTVVMSQPYPGWHELFSDLLPSHLLKDAPGGFARGMNGQVNGVRVSGGPFGIRSADPGRDEMLLERNDRFWGTPAMPGQILLRRGGTTAQLAGSLRTGDVQMALVHGGVATQAQLGAIPSVRTAIMPQSRVLQLVLNGRKGELSDPRVRSGVLALLDPALLATVGAQTGNWVEPARAQVLAPSDPGYAPTAPPRPSAEEAFALLAAAGYGRAPEPPPATSPTSPAPQPRTVGKDGKPLVVRIGAVDRDATALAVANTAADQLRSAGIDATVRSVAADELYGKELIEGTVDAIVGWEVAGSDPATVLASRYGCPPPALPGATGPAQAIAEAAQRAPSNLAGVCDPALQPAIDEALRGGDVARVLAEAEPKLWAMATVLPIVQDNAVAASGPRVDGASLSGAIQVGVFGDASMWRRIP

>CORE_REP|Org24_Gene1345#

MCGLLGYLTVDTSGAPEGTTAEAIAAQLHEALVCQRHRGPDERGTWHDEHMVFGFNRLSIIDIEHSHQPLRWGPPENRQRYAMTFNGEIYNYLELREQLTAEHGAEFGADPMFATEGDTETIAAAFHYWGPEAAARLRGMFAFAIWDTETRKLFIARDPFGIKPLFLATGPGGTAFSSEKKSLLDLLPQLGLSDALDPRALEHYTVLQYVPEPETLHRDVRRLESGCYAWVEPGQAPKITRYFDPRFRVVPFAKPGEVTAQPPTTRPRPAAQRPNTAEYRYREIAEALEDSVAKHMRADVTVGAFLSGGIDSTAIAALAIRHNPNLLTFTSAFEREGYSEADVAAETAAAIGAKHYIRTVSPEEFAASIPEIVWYLDEPVADPALVPLYFVAKEARKHVKVVLSGEGSDELFGGYTIYREPLSLKPFEYLPKPLRRLAGRLSERIPDGTRGKSLLHRGSLTLEDRYYGNARSFNDAQLRSVLRDFRPEWTHRDVTDPIWAMQGRDWDPVARMQHLDLFTWLRGDILVKADKMTMANSLELRVPFLDPEVFAVAEKIPVDQKITKDTTKYALRRALEDIVPPHVLHRAKLGFPVPLRHWLRGPELYDWARQQIIDSATDHLLDKTAVLGMLDAHRAGTSDHSRRLWTLLVFMIWHGIFVEQRIKPEIQEPTYPVSL

>CORE_REP|Org144_Gene6421#

MVSHQNDVGTGHGAATGSAQSDTGAAKLEKVVIRFAGDSGDGMQLTGDRFTHEAAAFGNDLATQPNFPAEIRAPQGTLPGVSSFQIQIADYDILTAGDQPDVLVAMNPAALKANLADLPRGATLILNTDEFTKRTLAKVGYRADPLDDDTLSDFVVHRVPMTSLTMGATESTGVGKKDGQRAKNMFALGLLSWMYGRPIGGTEQFMREKFAARPEIAEANVLAFRAGWNYGETTESFATTYEIAPAKLPPGTYRQITGNTALAYGLVAAGQLAGLPVFLGTYPITPASDILHELSKHKNFGVTTFQAEDEIAGIGAALGASLGGSLGVTSTSGPGLALKSETIGLAVMTELPLLIIDVQRGGPSTGLPTKTEQADLLQALYGRNGESPVAVLAPRSPADCFATAVEAARIALTYRTPVLLLSDGSIANGSEPWSIPNVTELAPIDPAFEPAGAETDPFLPYARDPETLARPLAVPGTKGRAHRIGGLEKADGSGNISYDPANHELMVRLRQAKIDGIGVPDLEVDDPDGRAELLLIGWGSSYGPIGEACRRARRRGVPVAQAHLRHLNPLPANLGAVLRRYRTVVAPEMNGGQLALLLRGKYLVDVRPWTKVAGTAFSAQELVGVIDAALDGSLEEMEHDKAFAARARATYTTQPDSSANRAQPGDVRPTGGNE

>CORE_REP|Org113_Gene5495#

MPEVERHKYDVVVIGAGGAGLRAVIEAREHGLSVAVVCKSLFGKAHTVMAEGGCAASMGNANEKDNWQVHFRDTMRGGKFLNNWRMAELHAQEAPDRVWELETYGALFDRTPDGRISQRNFGGHTYPRLAHVGDRTGLELIRTMQQKIVSLQQEDYAESGDYEARIKVFAECTITDLLKDTSGIGQGPEEAAGVTTEGPRSRSNDTGAISGAFGYWRESGRFVLFESPAVVLATGGVGKSYKTTSNSWEYTGDGHALALRAGASLINMEFLQFHPTGMVWPPSVKGILVTEGVRGDGGVLKNTEGKRFMFDYIPAVFKGQYAETEEEADQWLRDNDSARRTPDLLPRDEVARAINEEVKAGRGTEHGGVYLDIASRLPREEILKRLPSMHHQFKELADVDITSEPMEVGPTCHYVMGGIEVDPDTGAATVPGLFAAGECSGGMHGSNRLGGNSLSDLLVFGRRAGLGAATYVEQLEKRPAISNSDIDAAAKLALSPFDPPASGTGENPYTLHTDLQQTMNDLVGIIRKEHELEQANGHLQELRERYGNVTVEGHRQFNPGWHLAIDLRNMLLVSECVAQAALLRTESRGGHTRDDHPQMDANWRNRLLVCRVDPADADRTVPSVVVTSEDQKPMRSDLLALFELSELEKYYTPAEVAAHPAAAESSAKGDE

>CORE_REP|Org5_Gene139#

MSLSISPDEMLVAGDEHEEGAAAQSPNRARDRDAARRFSLLSRSVFVGGVIVTLLLFGIGAWQRRWIADDGLIVLRTVRNLMAGNGPVFNAGERVETNTSAAWTYVIWFFGWISDARLEYVSLVVALTLSLLAIVFAMVGSARLWRPVTGAAPTLLLPAGALVYIAVPPARDYATSGLENCLVIFWLGVLWWLLLRWSQDERPRLLNLLLAGFWAGLCWVIRPEMTVIGGLALVVLFFSRMPRTRLRPLFTRALLVLVGGLVPVGYQIWRMGYYGLPYPNTAVAKEAGGAKWQQGLKYLWDLVGPYYLWIPLLVLIVVAVALLVRARRGRAVADAGKAPATGRLTRLQRWLRSPAAVVTVLVGGGLLLVIFNIRVGGDFMHGRMLLPQLFCLMLPVSVLPVRLPVASAGSDRPGWLRWSFALPLIAWAGTVGWALFAANTTANTAGGQISASGIVDERIYYVLNSGHDHPVLAEDYLDYPRMRAMVQDIAANPNGGLLINSPSYMMWYVAPPPLPIPPAGYGHTVYFLNLGMTSMNVPLSVRVIDQEGLAYPLAAHTDRLVDGRIGHDKNLYPDWVVVDTGMVDQHPWMPWFLDEKWVIQARTALTCPATQDLLASYRAPLTLDRFKHNLIQSLHFAKYRIDRVPKYEIQRCHLVDPTTPPPVPN

>CORE_REP|Org112_Gene6280#

MSRGRDSGRDRAENGRGRGRFGGGRGNSGGGRASSGKRAASARSAAGPARARKPSRPRPAPGLDASTRFRFGVGRIVMLVALLVAALQLLWIQSVSAPRLSAEAASQRTVHQIDAATRGPILDRNGKSLAFTVNAKALTFQPVRVRKDLQEAHDENSAKPEPDQRMQAIAKYIHDKLGTAAPEQDLLKKLRSDEPFVYLVRNVDPRVAADISLKFPEVGTERQDLREYPGGSLAANVIGATGWDGHGQIGLESALDAILAGTDGSHTYDRGSDGAVIPGSWRDRQPAVNGYGVELTLDSDLQYYVQQQTQQAKELSGAQAASAVVLDARTGQVLAMANDSTFNPALGPQHWSSSSLGNPSVQEVYEPGSVNKIVTAAAAIEYGLTTPDEVLQVPGNIFMGGVTVNDAWQHGVMPFTTTGIFGKSSNVGTLMLAQRIGEDRYYDMLQKFGLGQRTGVGLPGESAGVVPSREQWSGSTFANLPIGQGLSMTTLQMTAMYQAIANDGVRVPPRIVKSKIDPDGNRTEEEPPEGVRVVSPETAATLREMFQAVVQRDPMGVQMGTGVPAAVEGYQVAGKTGTAQQIDPGCRCYSTSSYWITFAGMAPADNPRYVIGLMLDAPVRSSDGSGGQSAAPLFHAIASWALQRDRVPPSPPAKPLILQAS

>CORE_REP|Org101_Gene1761#

MIDILTPAANGIQGRVCPVRHDNDFEDFMEQSATQAVTETKDRATAVDAPASLRSDHRAPDRLVLQRGIFTGPSAKVSDELYAVVKGRAHRERQALRLEKGAAAHTNTYFGRFAASYWQRWTTVTEVRVTMVLDVVKKAKLRLVASDIAGHRRIIDTAQVTASGPVTLSATLDQYVDGGAIWLEFDAVGGDLGITEVSWTSAAPDHIRPVAIAICTFNRAEDCAHTVAALASDAVVLGAIDAVYVVDQGTDLVQNRPLYQEVAPTFGDKLRYIRQPNLGGAGGFTRGLYEVSAANEHADVILMDDDILCEPETVLRLNAFANMTVEPTLVGAQMLFLLNPDYLNVGAEEVHLQDLRHGQKVPKALRNTSMLKRNQERRVDAGYNAWWTCLIPAEVVAEIGLPVPIFFQWDDVEYGIRARESGFVTVTLPNAAVWHADFYWKDYDDWARYFSTRNSLIVGALHTDLDGKAITRKLFRELSEQLVAMQYGLVHTTLQGIEDFLQGPKVLQDGGIAALAAARTSRADYAETKKHPASTPPVRSGDIQLRRATGEPSRPLLVLIKRAINQWFGRTQHGVIGVTREDAYWWHVSLFDHVVVTDASQSGVRVRQRDKARARQLLRRTFHVLRRLRRELPTLQQQYRAAVPDLTSRANWERLYGITPE

>CORE_REP|Org8_Gene457#

MSRTRGTWTSVVAAILLVAGMATACSSDDTDEAADVCATTPNGTLVAASPTGPTGSKDISTNPELSTGYRSGMVAARTKTFAVATANTLASKAACEVLRDGGTAADALITAQTMLGLVEPQSSGIGGGAFLMYYDAASKSVEAYDGREVAPAAATENYLRWVSDTDRTEPKPNTRASGRSIGVPGVLRMLEMVHREHGKTGWRELFDPAIGLADRGFSISPRLAAQVAEQAKNLALDEAAKAYFLNPDGTPKPADTLLTNPAMAKTLGAIASEGAQAFYTGAIAQDIVAAATSTSGGRTPSLITTADLAGYQAKKRTALCTDYRNHQICGMPNPSSGGSTVAATLGILENFDLAALPPDNLGAGSDTARNGGKPKAEAVHLIAEAERLAYADRNKYVADTDFVPLPGNSLQTLLNKDYLKQRSALIDRNRSMGTAQPGDFGPVPLGVGPQPPEHGTSHISVVDQYGNAAAMTTTVESEFGSFHLVDGFVLNNQLTDFSADPLGTDGAPVANRLQPNKRPRSSMSPTLVFDKAPDGARGNLTHVAGSPGGSVIIQFVVKTLVGMLDWGLDPQQAVSALSFGAGNSPATGVGGEHPSINTADNGDHDALVLRLRELGHQVSVAPQSSGLSALTRDGTAWVGGADPRREGAVLGDNR

>CORE_REP|Org15_Gene5535#

MSPCCVCLGGEGMTDETFDDYLDETGNIAIPEGRTLVDYVEKHTRNDANDLAYRYIDYSRERDGEYQDLTWKEFGVRLRAVAARLQQVTKPGDRVAILAPQGLDYVISFFAAIYAGTIAVPLFDPDEPGHTDRLHAVLGDCTPSAILTASSSAAGVRQFFRPLPAAQRPRIIAVDAVPDTLGESWVRPDLAVDDIAYLQYTSGSTRTPAGVEITHRAVGTNLLQMVHAINLDWNSRGVTWLPLYHDMGLLCVILPAIGGKYITIMSPSAFVRRPGRWISELAAVSDGAGTFAAAPNFAFEHAAARGLPKNGETLDLSNVIGLINGSEPVTTSSMKKFNEAFAPYGLPKTAIKPCYGMAEATLFVSATRAEDEAKVIYVDRNELNAGRVVKVDHSAPNAIAQVSCGYVALSQWAAIVDSESIDSPEGAQELPEGRVGEIWLHGNNIGIGYWGREEETRQTFKNLLTNRQAEGSHAAGAPDDAIWLRTGDYGVYVDGELYITGRVKDLVIVDGRNHYPQDLEFSAQEASKMLRPGFIAAFSVPANQLPAEVFAADSHAGLKYDADDASEQLVIVAERGPGAHKADSQPIADAVRGALSQRHGVTVRDVLLVPAGSIPRTSSGKLARRACRAAYLEGTLRGGYQQQAFPDAPDEE

>CORE_REP|Org140_Gene4746#

MCCPNRTCRRADAGGAAVTSIQIAQRGTGLLRAFNEAGVLSAADVHVAVRLGRLGREESEAALFAAALAVRAVRSGSVCLELARMREIGIDADETWDTTVDPASLPWPEFDDVLAALRVSPLVIGGAAGPLRPLRLVEDRRAGGPLLYLDRYYQQEQTIRRVLTERSDRHPVVVPAVVRRELDRLFATPATEAGSTAPDRQRLAAALAATHWTTVVAGGPGTGKTHTIARIIALLDAHQRANPKAPALRVALAAPTGKAAARLQEAVRDQAADLGLPELSASTLHRLLGWQRGRGTRFRYHEFNRLPYDVIVVDETSMVSLTMMSRLMAALRPDTRLVLVGDPDQLASVDAGAVLADLVAGPVVGAPNPVLDQIIGRAAEPSADPEALTELEQTRLRGGIVRLTRGRRFGGRIADLAVAVRAGDSETALGLLREGGDALSLCEPEDVAAVRADVITAARRVTEAALAGDAAAALTALESHRLLCAHRQGPFGVERWDRMAGEWAAAAGAGPESGQNTWYPGQPLLVTANDHEARIYNGDTGVVIRQPDGSLRVALQRGSEPYLVHPTQFPSVVTVFAMTIHRSQGSQYDAVTIVLPEPESTLLTRELLYTAITRARGHVRIIGTDAAIRAATARRVLRASGLSHRVE

>CORE_REP|Org163_Gene4912#

MIWIRCAIRRELVQVGVLSRVDTPDDLRRLTVPQVRELAEEIREFLVRKVAATGGHLGPNLGVVELTIALHRIFDSPADPLIFDTGHQAYVHKILTGRKEQFDSLRKQGGLSGYPSRAESAHDWVESSHASAALSYADGLAKAFALSGQDRHVVAVVGDGALTGGMCWEALNNIAAAPDRPVVVVVNDNGRSYAPTIGGLAERLTALRTQPAYEHALDAGKRILKSIPRVGESAYSMVHAVKAGIKDAVSPQELFSDLGLKYVGPVDGHDVVALEAALRRAKDFGGPVVVHAVTQKGRGYAPAENHVADQMHACDPIDPLTGVPVGGPKARGWTSVFSEELIAQAERRADIVAITAAMPGPTGLAAFGERFPDRMFDVGIAEQHAMASAAGLALGGMHPVVAIYSTFLNRAFDQLLMDVALLKQPVTVVLDRAGVTGSDGASHNGMWDLSVLGIIPGIRVAAPRDAATLREELAEALAVNDGPTALRFPKGSVAEDISAVERLDGIDVLRTAEPEGGSVQAVHGDVLLVAVGSFAAAALEAANLLDSEGISVTVIDPRWVLPVSDTLLKLAENYRLVVTLEDGGLHGGIGSTVSARLRNSGLDVPTRDLGVPQQFLDHASRGELHTELGLTGPDIARRISGWLAAR

>CORE_REP|Org105_Gene4310#

MTTPKNLSSRYELGEIIGFGGMSEVHKARDLRLSRDVAIKVLRADLARDPTFYLRFKREAQNAAALNHPAIVAVYDTGEAEVDGGPLPYIVMEYVDGETLRDIVRGKGPLPPRRAMEIIADVCAALDFSHKAGIVHRDMKPANIMINRSGAVKVMDFGIARAIADAANPMTQTAAVIGTAQYLSPEQARGESVDARSDVYSVGCVLFEILTGEPPFTGDSPVAVAYQHVREDPRLPSLVHEGVPRELDSVVLKAMSKNPANRYQTAAEMRADLIRVLGGQKPSAPMVMTDEDRTTILGSEEPAPRSYHTVDNHDRSAYRDNDDTGEPEPVDPPSQRRTAYLTLGAVAAVIVAIALFWVLIGPGSKPDQVAVPDLSNSSVQQAEQKLEDLGFHVAIQEKPDARVAPGNVIATQPLGGSRVDEGSTITLQVSTGPAQVQVPRLTGLTRQEAEQKLNAIGLRLDPQVDKEASSTAELDKVIGQNPAEGASVEVDRAVKVTIGSGPEQVRVPNVVGQDIEVAEPNLVEGAQFKVVVQEVASSRPKGEVIATSPAGGSTAEKGSTVTVQVSLGAEFTMPSLVGLNASHAVDRLRQAGWAGSTTQIVQNTQVTLDSANVGKVLNQQPAAGSSVGRNSTIVIYTGVLPLGPP

>CORE_REP|Org136_Gene4665#

MTSPQEKAAAARKAAEEAARIAAEAAAAAEAAEAEAATAESGGAPAASGATAAESGAGSSGSGGAAAGSGGATDAVGGAAAGSGGAAESARGTTASSDVAASAGGAAGASGGPVGPATESGGADSGSDDDAPEGGGSSAAQEIAAGYAVEGAALELGTVVVDGTVDRTARVRIPLRTMNRHGLVAGATGTGKTKTLQGIAEQLSRAGVPVVLADVKGDLSGLSRPGEQNEKLAQRAVETAATDWAPTGFPTEFVSLGTGGLGVPIRATITSFGPILLSKVLGLNETQESTLGLIFHWADKQGLGLLDLKDLRAVIQHLTSPEGKADLQGIGGVSASTAGVILRSLVNLEADGGDTFFGEPELDPADLLRTEGGQGVITLFELGAQAARPAMFSTFLMWVLADLFQTLPEVGDVDKPELVFIFDEAHLLFADASKAFLDQVEQTVKLIRSKGVGVFFCTQLPTDIPNAVLSQLGARIQHALRAFTPDDQKALSKTVRTYPKTDTYDLEQALTSLGIGEAIVTVLSERGAPTPVAWTRIQPPRSLMDTIGADAIKSRALASALHGKYGQTVDRESAYEMLAANVAAAEPEAEPQPVPGRSPSAEEDSAAERIMKNPAVKSFLRSAATVAGREITRTLFGTRKRR

>CORE_REP|Org105_Gene5106#

MYRTGHADAIYVAAGPNSSVSAAVMKISGFHAMTGNRQAQRAFDAGILSLGLSIDGQESTRDLEYAKLAFQRATEWDPTMCDAWLGRAAAGEVTDEVIRNLHRTSTSTLYREQRRLGLAPRALAGRFVSGLYIDYPLASYTEIWLAYAANLIGSKQYDEAERVLDELAEYRAGMLSDPDREIDDRISAYIRGVLHFNTQRWPDVMSVLAGSAEWEDPYLATGAHVMVGSACAQLGLFGEAIRRMEQAENGPIPAARTTAMFCRGLCLRETGSEDEAQALFEQVYSQAPDFTANTEAMRDKSYRITITTKESIDARTDRWDPASAPSVEQLQTADAEDRAKKILTEARAELDRQIGLTAVKTQVAKLQATAQLAKIRAEKGMASVPRGNHLAFTGPPGTGKTTIARVVAKIYCGVGLLKTDKVVEAKRMDFVGQHLGSTAIKTDKLIDTAMDGVLFIDEAYTLIQTGLSGGDAFGREAVDTLLARMENDRDRLVVIIAGYDGEIDRLLAANDGLASRFAKRLQFPSYTPPELGQIGKLIASSRDSELSEDAVRLLEQACERLYNSERTDQSGQPRRGIDLAGNGRFVRNVIEAAEEEREFRLANDESLDLTAVDESVLMRIEAPDMEAALAGVLSSLGVS

>CORE_REP|Org12_Gene4561#

MSPAVLVSAPADLSAFGHDPWWLVVVKSVGIFIFLLLIPLLAVVIERKVVAWMQMRVGPNRVGPRGSLQSIADGVKMLLKEDIVPAMVDKPIYILAPIVALIPAVMAFAVIPLGPEVSIFGTRTPLQLTDMPVGVLYILAMTSIGVYGIVLAGWSSGSTYPLLGGLRSTAQVISYEIAMAACFGAVFLLAGTMSTSGIVERQWGTWNVWLLLPSFVIYAVAMVGETNRAPFDLPEAEGELVGGFHTEYSSLKFAMFMMAEYINMGTVSALATTLFFGGWHAPFPLNLWDGANSGWWPLLWFTLKLWTFLFVFIWLRGTLPRLRYDQFMNLGWKLLIPVSLLWVMIVATLKVVQDNGHDVQTTGLVTAGVIISVGLLAMMLRAGRAGDNPTPETAGPQQFSDFPVPPMPETAPTATKAGLLDPIGGFWVTFVTMFKKKNTEFYPEEKVPTAPRYHGRHQLNRHPDGLEKCIGCELCAWACPADAIYVEGADNTDSERYSPGERYGRVYQINYLRCIGCGLCIEACPTRALTMTNEYELADDNRADLIYEKDRLLAPLGDGMIPPPHAAYPGATEEDYYLGAVPAAPGTDRDTGLGSTGNAVARPSGSRGSDLVGPDPADPAVDAAGRQPAATGAQGGAQ

>CORE_REP|Org15_Gene5181#

MGTGNLLWLLPALPAAGALILLLAGHLSDRWGHWLGCATAVASFGVAVWAFAEMLGRAGADRAVSHNFFSWIPVAGLQAEFSLQLDQLTMCFVLLITGVGSLIHIYSVGYMSHDPARRRFFAYLNLFLAAMLILVMADNYLVLYLGWEGVGLASYLLIGFWHEKPSAAAAAKKAFVVNRVGDMGLAIALFLMFATFGSVDFGHVFAGVPQASDGTLTALGLLLLLGACGKSAQVPLQSWLGDAMEGPTPVSALIHAATMVTAGVYLIARSNAIFDAAPAARAGVLVVGAVTLLFGAVIGCAKDDIKKALAASTMSQIGYMVLAAGLGPAGYAVAIMHLLTHGFFKAGLFLGAGSVMHAMNDETDMRRYGGLRRYLPITFVTFGLGYLAIIGVPPFAGFFSKDRIIEAAFGYGGANGITLGAAALLGAGITAFYMTRVMLLTFFGEKRWTASKSGMEPHPHEAPAVMTGPMIVLAIGSVFSGGVFVFGSSLQNWLAPVVGTEHAESAVPAWAVTVAALVVVAIGVAVAYRQYAWRPVPLTAPQDVTPLTAAARRDLYGDAFNEAALMRPGTHLTRSLVFLDNRGIDGIVNTTAAVIGGLSARIRRVQTGFVRSYALSMFTGAALVVAALLAVRLL

>CORE_REP|Org216_Gene3071#

MPELRSRTVTHGRNMAGARALMRASGVPAADIGAKPVVAVANSFTEFVPGHTHLQPVGRIVGDAIRRAGGIPREFNTIAVDDGIAMGHQGMLYSLPSRDLIADSIEYMVQAHCADALVCISNCDKITPGMLLAAMRLDIPTVFVSGGPMEGGRATLADGTVRRLDLITAMSEAVNDDTSDADLATIEENACPTCGSCAGMFTANSMNCLVEALGLALPGNGTTLATHTARRDLYEAAGETIMAITRRYYDRDDASVLPRAIASRAAFDNAMALDLAMGGSTNTVLHLLAAAHEAGLDYTLADIEKRSRAVPCLCKVAPNGSHLMEDVHRAGGIPAILGELRRGGHLHTTVRAVHSESLDGWLAEWDVRGPNPAQAAVDLFHAAPGGVRSATAFSQSARWASLDLDAESGCIRDVAHAYSEDGGLAVLRGNLAVDGAVVKSAGVPADLHVFTGEAVVAESQEEAVTAVLSGRVRPGTVLVIRYEGPRGGPGMQEMLYPTAYLKGRGLAGSVAVVTDGRFSGGSSGLSIGHVVSPEAAAGGTIAAVADGDRITIDIPSRTLRLEVDDAEIARRLAHRRRTGYRPRSRHRPLSTALRAYALLAQSADKGGVRRLPPDELGGPEAAFDTQTRAG

>CORE_REP|Org207_Gene4008#

MAKAVGIDLGTTNSVIATVEGGQPTVIPNSEGSRTTPSVVAFTDQGERLVGQLARRQAILNPKGTVASAKRFIGRRFEEVATERDTVSYEVVSGSNGAARFDVRGKQVAPEEISAAVLRKLVDDASKYLGEKVTEAVITVPAYFNDAQRQATKDAGKIAGLNVLRIINEPTAAALAYGLDKKKNETVLVFDLGGGTFDVSLLDVGDGVVEVRATAGDTHLGGDDFDRRVVDWLAEEFRKDYGIDLRTDPQALQRLYEAAEKAKVELSSVSQTTINLPFITADAGGPKHLNTTLMRSKFDQLTGDLVERCMGPVQQAMADAKVTANDIDEVILVGGSTRIPAVQQLVRRLTGGKDPNMTVNPDEVVALGAALQSAVIKGEMSDVLLLDVTPLSLGVETLGGVMTKVIERNTTIPARRSEVFSTAEDNQNAVDVVVLQGERERAADNRVLGRFRLENIRPAPRGVPQVEVTFDIDANGILNVSAKDKDTGAEQTITISESSNLDQSEVERMVADAERHRGEDAKIRERVDARNTLDTIAYQVEKRLSELGEAAPAHDKARAEMLIGDARQAIKDDTVGIERLRELTSELQQLFYGLDTAAGGTAGNQAGGGARRDGGGGDDVIDAEFTSE

>CORE_REP|Org65_Gene1884#

MCGIVGYVGYRDALGVVVDALRRMEYRGYDSAGVAILDGAGAIAVERKAGRLANLEAELGEAGAGAFAGSTGMGHTRWATHGAPTDRNAHPHRDEAGAVAVVHNGIIENFAPLRRELEDAGVELRSDTDTEVAVHLVSRAYAEGPTAGDFEASALAVLRRLEGAFTLVFTHADHPDKIIAARRSTPLVVGVGKGEMFIASDVTAFIEHTREAVELGQDQAVVITADSYRVTDFAGNDAGSRTRPFTIDWDLAAAEKGGHDYFMLKEIEEQPAAVAETLMGHFDTGQGGSGRIVLDEQRLADQELRDVDKVFVVACGSAYHSGLLAKYAIEHWTRLPVEVELASEFRYRDPVLDRSTLVVAISQSGETADTLEAVRHAKEQKARVLAICNTNGAQIPRESDAVLYTRAGPEIGVASTKAFLAQVTANYLVGLALAQARGTKYPDEVAREFAELEAMPKLVARVLETAPQVRAIARELAKVPTVLFLGRHVGYPVALEGALKLKELAYMHAEGFAAGELKHGPIALIEDGLPVIVVMPSPKGRAVLHSKLLSNIREIQARGARTIVIAEEGDDTVRPFADDLIEIPSAPTLFQPLLSTVPLQIFAAEVAQARGYDVDKPRNLAKSVTVE

>CORE_REP|Org9_Gene416#

MVDTDHMPAPAMAPAAAPTGHARVLAWVAEVADLTAPEDVVWCDGSRQEWDRLTARLVDKGTFVALSGKPNSFWCVSDPEDVARVEDRTFICSRDKRDAGPTNNWVDPVDMRTVMTEHYRGAMAGRTMYVIAFCMGPLDAEDPKYGVQITDSEYVAVSMQIMTRSGAPVWNQLGQDAEFVQCLHSVGAPLSPGQADVAWPCDHTKYIAHFPEDRTIWSYGSGYGGNALLGKKCFALRIASVLARDEGWLAEHMLILKLTSPQGRTHYVAAAFPSSCGKTNLAMLEPALEGWKAETVGDDIAWLRLGPDGRLYAVNPEAGFFGVAPGTGAKTNPNAIATIEQGNSIFTNTALTDDGDVWWEGLTDTPPQHLTDWRGNDWTPESATGPAAHPNSRYCTPIEQCPSVAPEWDDPAGVPLSAIFFGGRRATTIPLIAESFDWTHGVFTASVLSSETTAAAAGQVGVVRRDPMAMLPFLGYHVGDYFAHWLRLGEAADPGKLPKIFQVNWFRRDADGRFLWPGFGDNVRVLKWALERIEGTAAADATAIGYVPVPSSLDLSGFSEAGKRSARAALEVHHAEWADEVASIEDWYASIGADTLPGPLGDQLAALKNRLAHTPARPTGTTAGAS

>CORE_REP|Org5_Gene6313#

MNNSSVDSVPSGGDDRPEGADERNHEGPADAATGYRDGSAAPAASESGAAEVSEPTFADLGIDDRLLAAIADVGYESPSPIQAATIPPLLSGADVVGLAQTGTGKTAAFAIPILMGLDKRPKPPQALVLAPTRELAIQVAEAFGRYSAHLPGIHVLPIYGGQNYAVQLQGLRRGAQVVVGTPGRVIDHLERGTLDLTQLRYLVLDEADEMLKMGFQEDVERILRDTPAEKQVALFSATMPSVIRKISKQYLKDPVEITVKSKTSTNTNITQRWVHVSYQRKLDALTRILEVEPFEAMIIFVRTKQATEELAEKLRARGYSAAAINGDIAQNQRERTIGQLKSGTLDILVATDVAARGLDVDRISHVVNYDIPHDTESYVHRIGRTGRAGRTGEALLFVAPRERRLLDAIERATRQPLTEMQLPSVDDVNAQRVVKFHDAITENLASPNLALFRKLIEDYEAEHNIPLADIAAALAIGGHDGENFFMEPEAEPIRPPRRERAPREERERRSEGPQRHRATGADMATYRIAVGKRHRVVPGAIVGAIANEGGLRRSDFGHISIRPDHSLVELPADLPSETLDALRRTRISGVLIQLQLDQGPPSHRPIGRGPRREGGRKHDRRKPRS

>CORE_REP|Org13_Gene1651#

MHRQVRSSDGRGTHHHARRGCRPRGDSPLSAAPVEIQPVPQAERPFPARMGPKGAAMWKIVTTTDPKLLGVMYIFTAISFFLIGGLMALLMRAELARPGLQFLSNEQYNQLFTMHGTLMLLFYATPIVFGFANCVLPLQIGAPDVAFPRLNALSYWLYLFGATVATAGFITPGGAADFGWTAYVPLSLAVHSPGVGADLWVMGVAVSGVGTILGAVNMITTVVCLRAPGMTLFRMPIFTWNILVTSILVLEAFPILTAALMGLEVDRHLGGHIYDPATGGPILYQHLFWFFGHPEVYIVAIPFFGIITEILPVFSRKPVFGYTALVYATIAIAALSSAVWAHHMFATGAVLLPFFSLMSFFIAVPTGVKFFNWIGTMWKGHLTFETPMLWSLGFVTTFLFGGLSGVLLASPPLDFHITDTYFIVAHFHYVLFGTIVFATFGGIYFWFPKFTGRFLDERLGRLHFWTTFLGFHTTFLVQHWLGAEGMPRRYADYLPADGFTTLNTISTIGSFILGFSMITFVWNVFKSYRYGQVVTADDPWGYGNSLEWATSSPPPRHNFYELPRIRSERPAFELHYPHMIERMRAESHTGWGSAGRSHAAALTEAPAAGPQESNRADESDAE

>CORE_REP|Org210_Gene6389#

MPAPAAGRSRGDGPVGHYKSNVRDLEFNLFEVYGLETVLDSGAFAELDGETARTMLAEAARLAEGPVAESYAETDRHPPIFDSDSHSVRIPEPFKRSVRAWQDAQWWRVAKSEAIGGVPAPSMLGWAINELVLGAQPAAYMYLSGPMMADVLAGIGTEQQRRWAAQAVERNWGATMVLTEPDAGSDVGAGRTTATEQQDGSWHLEGVKRFITSADSDDLFDNIMHLVLARPAGAGPGTKGLSLFIVPKFHFDHDTCEPGERNGVFVTNVEHKMGLKASATCELTFGGHGIPAVGYLVGGVHNGIAQMFEVIEEARMMVGTKAIATLSTGYLNALDYAKTRVQGSDPARAADKAAPKVTIIHHPDVRRSLMMQKAYAEGLRSIYLYTAGHQDPVVARHISGADDDLAARVNDLLLPIVKGVGSERAYQYLTESLQTFGGSGYLQDYPIEQYIRDAKIDSVYEGTTAIQAQDFFFRKIARDNGIALAHVLGQVRATAESDADRGRLKNEKVLLSHALEDVQEIIGRLTTHLLEAAVEPREVYKIGLNAVRLLLAVGDLLVAWRLIVGAETALTAIEAGQNTAFYAGKVAVASFFANSALPHLTAELAVVSATDATVMDLEEAGF

>CORE_REP|Org4_Gene3693#

MRTRSVPWLRAVSVPPPRSPEGSMSNLINLEQVSKSFGITPLLDNVSLGVHAGERIGVVGLNGGGKTTLLEVLTGLEPPDSGRVSRVGGLRLAVVTQRGVLPAGATVGSVVLAGLADDLNGVAGGPDEVAEHEWAANPRIRSVLEGIGIAGLGLQTSIDNLSGGERRRVALAAALVRDLDLLVLDEPTNHLDVEGVQWLAAHLLERRSALVVVTHDRWFLDTVATDTWEVVGGKVESYEGGYGDWIFARAERARQADASEARRSNLARKELAWLRRGAKARTSKPRYRVEAAEALIADVPPPRDSVSLAAFARKRLGRVVIELEDTTLTTPDGRELVRDLTWRLAPGERVGLVGVNGSGKTTLLRTLAGDTEPAAGKRIQGQTVQIGWLRQELDDLPTDMRVLEAVQQVAQRIMLGDKEISAGQLAERLGFSPARQRTPVGDLSGGERRRLQLTRILMAEPNVLLLDEPTNDLDIDTLQQLEDLLDNWAGTLVVISHDRYLIERICDTTWALFGDGKLTNLPGGIDEYLKKRAAQGQSATRAADKPTGPVTDAAAQRAARKELSRLERAIEKFDEREQRLHTALADAAIDPDKLVTLNAELKQVVADKEAAEERWMELAEDV

>CORE_REP|Org157_Gene2630#

MPIASDPSPGAETVPQYAAWRKGVAGVLAKARKVDVADLPDEPEQLLTQTTYDGLAVAPLYTRRDERPEPPLPGVFPFVRGRDATRDVHRGWDVCADITETDAAAANREILAGLENGLSAVRVGVGEHGVPVAELPTALRGLLFELAPLSLAAGAALPEAAAQLYAVLDDYRVDDRAAIRIGLGAAPLTSRFAGAADVDSDRAVELAKQAVARTETVRAITVDGTVFHNAGASDAQELGAVVAAGLEYLRALTGAGVDIADALGQLEFRLAATDDQFATIAKFRAARTVWARVAHVCGAPDFGGAPQHAVTSAAMMSKRDPWVNMLRTTLAAFGAGVGGADSLTVLPFDCALPPGELGVSKSFSDRMARNTQLLLLEESHLGHVQDPGAGSWYIEDYTAKLAAKAWEFMQELEKAGGYRAALDAGLLGQRIAETKATRDADVAHRRTAVTGVNEFPNLAEKPLSEQARTPGEIARYGAAFEALRDRSDAYLEANGARPKALLVPLGTVAEHNVRVTFIANLLASGGIASVNPGPLEVSAVEAAAKEAGAPIAVLCGSDARYGAEAGAAVAALRAAGVETVLLAGAEKAVADLDAAQRPDGFLTAKIDAVAQLSGLLEKVGA

>CORE_REP|Org102_Gene5286#

MRRNLLRLATAGSVDDGKSTLIGRLLYDSKALFSDQLSAIEKFSTARGDQAPDLSLVTDGLRAEREQGITIDVAYRYFATPRRKFVIADTPGHVQYTRNMVTGASTADLALILVDARKGVSEQTRRHAFLSSLLGVGHLVLCVNKMDLVDFSRHRFDEIREEFADFATKLEVRDLSFLPLSALHGDNVVEPSPHTPWFPGPPLLRHLEDVHIASDRNLIDARLPVQYVIRPSGETRRSYAGTIAGGVFKPGDEITVLPSGRATRVGQIWGPGGSKVEEACAGMAVSLTLDDELDIGRGDMLARPGNQPHQDRELDAMVCWFSDDTALRPGDRYAIRTAAQTAQVRVRALDYRLDVNTLHRVEDAPRLALNDIGRVTLHSTQPILFDPYRANRATGSFILIDERTDQTVAAGMITGRTPVQPQARGPVTWHRSAVERTQRLSQGGTLWLTGLSGSGKSTIAVELERQLIAAGRPAYLLDGDNLRHGINGDLGFGDDERRENIRRVAEIAALFADSGTIAIVSLISPFAAERENARKIHADKGLEFHEIFVDTPLATCEDRDPKGLYAKARAGEITRFTGIDSPYERPEHADLVVTPADGTPTAIAELIRRELGIAEHR

>CORE_REP|Org10_Gene3860#

MTESDRTESTAPGCDPAELRTLFLFEQLDDEQLAWLCADGRIELIEPGPVYRQGDPATCFYVLIEGELRLTKLAGGMEIELNRTDHRGVYAGAWTAYLGEQAEPTYNSSLYVTRPSRFFVLDAEIFARMMHAWFPMAVHLLEGAFFGNRNAHQRVAERERLMALGSLSAGLTHELNNPAAAAVRATSGLRERVAGMRHKLGMLAEGRFAPEVLVTLVRLQEEAAEQVAKAPALTPLEAADREDALGDWLDEHGIADGWELAPNFVQAGFDVDWLERVHGTLEGCSETVFEGAIRWLNYTIETELLMNEIADSTARISTLVGAAKQYSQMDRAPFQVVDIHELLDSTLVMLNRKLGDGVRVVKDYDRTLPALPCFAAELNQVWTNLIDNAVYAMGGEGTLTLRTYRENDCAVVEVGDTGPGMPEEVRRRVFEPFFTTKPVGEGTGLGLDISFRIVVNKHDGDIRVESAPGDTRFVVRLPLHRDIPARNPAPTPRNRQTETMTAIEGIDPSVPPSGPGCVECEASGGWWVHLRRCAQCGHIGCCDTSPSQHATAHHRQTGHPFIQSYEPGEDWYWDFRTEEMFTEGPELAAPHSHPAAQGVPGPSGRVPADWREHIH

>CORE_REP|Org49_Gene2014#

MRPSTLPPRTTGRRPPARNRPGRRALTGSRATPPGTARICPWRCSTARRIRLPIVAVSESARRRIIEWGRRALGLDVPPSDHTAAALHSVETDVPGLDKRETLQLARIRLLGATGAVIMAISALGVGAQPVRQNPTSGLRIIGFFARAHTSTLAMCMIGTVLVVMAWLLLGRFAIGGWGGNPRHRLSRSQMDRTLLLWIIPLSVAPPMFSNDVYSYLAQSEIAVRGLDPYEVGPADGLGLNNVLTNNVPNIWRETPAPYGPLFLWMGKGIAVVTGDNIIAGVWLHRLLVLGALALIVWALPRLARRCGVAAVSALWLGAANPLVLLHLVGGVHNDALMLGLMLAGLEICLRAIEDAYPFDQRAWAILLGGAGLIALSSTIKIVSLLALGFVGMALARRLGGGFRMVVKVGLILGVVAGVTILFVTTASGLGFGWLYTLNTASAVRSYLSLPTAIGIATGFGGVLLGLGDHTTAVLSITRPIAATLAAVVIVRMLFATWTGRLHAVGALGVSLGALVLLFPVVQPWYLLWAIVPLAAWANRPAFRVPAIALSVVVSLLVMPRGADFYVFQIVQSAIATVIVGLAFIFLTRNALPWRNQPGVSAPSQEATAYGVRS

>CORE_REP|Org46_Gene2776#

MRTLYPAIEPYESGMLAVGDGQSVYWEVSGNPDGKPVVFLHGGPGGGTAPFHRRFFDPAAYRIVLFDQRGCGRSTPHLADGASLEHNTTGHLIADIEALREHLAVERWQVFGGSWGSTLALAYAQRHPERVTELVLRGIFLLRRKEIDWYYNGAAGYVYPDEWEKFLAPVPEDERGQDLVEVYHRLLHSPDEDLARAAAIAWSTWEGATSSLLPHPDRVAETAEPRFALAFARIENHYFRHGGFLDEGQLLRDIAAITHIPAVIVQGRHDIVCPAVSAWELHRAWPGSVLHIVDDAGHAANEPGITHHLVEATDRFSRVGVSTVTTAADALIGALRDDIDRLSAAEPEVRADAEDSVHQMRVATRRLRSVLRSYGTLLAKKPAAAMNAELKWLAGLLGEARDAEVRADRFAALLAEHGEQAQPADLDAVTARLVNAERDRYRAAHDEVLAALDGKRYRELHDELARWRTAPPLRHSRAEAPATDVFGEVLRRDLDRVASLVRAEPTVDPHERVELLHDIRKSAKRLRYSCEAAEHVIGDEAAERGRRAKKLQTVLGDHRDAVESHSAIVRRAAEAAAADEDAGLYDILAAAEDAAAGRELSRYPATAAALLG

>CORE_REP|Org103_Gene5940#

MYSWDSSNAVSPVSSDNADSATSAAPGPFASADAAAHVRRDARPLVGRSERARNAAVRPGFGLALVMDAVTVVPTPANEPVHSYAPGSPERERLLARLSEISAETLDVPLVVGGKHRPGIGERHDIRAPHRHDLVLGTYTDTTHSEAQAAIDAALAAAPDWRSLPFDDRAAVFLRAADLLAGPWRETLAAATMLGQSKSVAQAEIDAPCELVDFWRFNVAFAREILAKQPQSSPGVWNRMEYRPLEGFVYAITPFNFTAIAGNLPTAPALMGNTVVWKPSPTQTLSAYYTMRLLEAAGLPPGVINMVTGDGVQLSEIALADPRLAGIHFTGSTATFQYLWQEVGANIGRYHGYPRLVGETGGKDFVLAHPSADPAALSTALIRGAYEYQGQKCSAASRAYIARSVWREMGEQFLATVEELRYGDVADLSNFGGALIDRRAYDKNVAAIERARSAGVTVAVGGTYDDTDGWFVRPTVLVCDDPADESFRTEYFGPILSVHVYDDGEPGAYSAILAEVESAAPYALTGAVFAQDRKAIEQACTALRFAAGNFYINDKPTGAVVGQQPFGGARASGTDDKAGSPLNLLRWVAPRTVKETFAPPTDHRYPHMRT

>CORE_REP|Org118_Gene1593#

MSPADAFVSGTRTITAPSKPAPADQPAWNKQKNSSMPTFRYRPFAEEVPGGSPAFDAAPIPFDRTWPDRVVDRAPGWCAVDLRDGNQALIDPMSPARKRRMFDLLVRMGYKEIEVGFPSASQTDFDFVREIIEDGAIPDDVSIQVLTQCRPELIERTFEACQGAANVIVHFYNSTSILQRRVVFRADRDAVKKIATDAAKLCLEIEQRYPDTNWRYEYSPESYTGTELEYAREVCDAVSEIIAPTPEKPLIINLPATVEMATPNVYADSIEWMSRNLARRDSIVLSLHPHNDRGTAVAAAELGYQAGADRIEGCLFGNGERTGNVCLVTLGMNLFSRGVDPQIDFSDIDEIRRTVEYCNQLPVHERHPYGGDLVYTAFSGSHQDAINKGLDAMKDTADASDSDVDDIVWEVPYLPIDPKDVGRTYEAVIRVNSQSGKGGVAYIMKTDHGLVLPRRLQIEFSQAIQKITDGEGGEVTPKEMWDVFAEEYLNPVLPLERIRQKMTAAETDSGTDTITAVVKVDGAEQEIVGSGNGPLASFVDALATIGYDVRVLDYSEHAMSSGDDAQAAAYVECAIGDKVTWGVGIATSITTASLRAVVSAVNRALRAR

>CORE_REP|Org4_Gene5021#

MSESENGSRPVALIDRDWEEQHMSPIENSDSAAWRELEAVRAEAAALRRQLADSPDRARELEARIDSLTIRNTKLMDTLKEARQQLVALREEVDRLGQPPSGYGILIGVYDDQTVDVFTSGRKMRLTCSPNIETSTLEYGQTVRLNEALTVVEAGVYDAVGEIGTLREILDDGRRALVVGHADEERVVWLAGPLAKVAEMDDLEDPDSPIRKLRPGDSLLVDTKAGFAFERIPKAEVEDLVLEEVPDVDYGDIGGLGRQIEQIRDAVELPFLHKDLFREYALRPPKGVLLYGPPGCGKTLIAKAVANSLAKKIAEARGEDAKEAKSFFLNIKGPELLNKFVGETERHIRIIFQRAREKASEGTPVIVFFDEMDSIFRTRGSGVSSDVETTVVPQLLSEIDGVEGLENVIVIGASNREDMIDPAILRPGRLDVKIKIERPDAESAQDIFSKYLVEDLPLHADDVAEFGGDKAMCIRAMIDRVVERMYAESEDNRFLEVTYANGDKEVLYFKDFNSGAMIQNIVDRSKKYAIKSVLDTGNPGLRIQHLYDSIVDEFSENEDLPNTTNPDDWARISGKKGERIVYIRTLVTGKNASASRAIDTESNTGQYL

>CORE_REP|Org5_Gene5463#

MTSTSRYGNALQASAQQRLVCAPVITRLSHLFLRTLRDDPADAEVPSHKLLVRAGYVRRIAPGVYSWLPLGLKVLRRIEDVVREEMNGIGGQEISLPALLPRDPYETTNRWTEYGDALFRLRDRKGADMLLGPTHEELFALTVKGEYNSYKDLPVTLYQIQTKYRDEERPRAGILRGREFIMKDSYSFDLDEDGLAASYAAHRGAYQRIFARLGVEYVIVAATSGAMGGSASEEFLATSPIGEDTYVTCLESGYAANVEAVVTPAPAEIPVEGRPEAVVHDTPGTPTIASLVEWANGAGIAEGYGRPVTAADTLKNVMVKLRHPDGKTEVVGIGVPGDREVDDKRLGASLEPAEVELLTDEDFTANPFLIKGYIGPKALLENGVRYLVDPRVVTGTSWITGADASGKHVVGLVAGRDFTPDGTIEAAEVREGDPSPDGRGVLHAARGIEIAHIFQLGYKYTDAFEVDVLGENGKPVRLVQGSYGVGISRMVAVVAEQMHDEKGLRWPSEIAPFDVHVVVANKDEAARAGAEQVVAGLDAQGLDILFDDRTASPGVKFKDAELLGMPWIVVIGRGWADGKVELRNRFTGEAEDIPADSAVESVLAKIRA

>CORE_REP|Org195_Gene4993#

MTSSTDAGPTDQPTPESQPGHRARPERPASERRVRPDRPRPEATSTGPRVRTGKPVRRKAEGQWALGYREPLNPNEQSKKDDNPLNVRARIENIYSKTGFERIDKGDLRGRFRWWGLYTQREQGYDGSWTGDENIDLLEAKYFMMRVRCDGGALNVAQLRTLGQISTEFARDTADLSDRENVQYHWIEVENVPEIWKRIEAVGLKTTEACGDCPRVVLGSPLAGESLNEIIDPTPAIDEIVRRYIGKKEYSNLPRKFKTAISGQQDVVHEINDVAFVGVVHPEHGPGLDLWVGGGLSTNPMLAKRVGVWVPLDEVPDVWEAVVSVFRDYGYRRLRTKARLKFLIKDWGIEKFRQVLEDEYLKRKLIDGPAPEQPTKPIDHVGVQRLRNGLNAVGFSPIAGRVSGTVLTEVAAAVERIGSDRIRFTPYQKLIVLDVPDDKVDALIDELEPLGLQARPSLWRRNLMACTGIEFCKLSFAETRKRSQALVPELEERLADLNAQLDVPITININGCPNSCARSQIADIGFKGQLVDDGDGNQVEGFQVHLGGSLGFDSAFGRKLRQHKVTTQELGDYVERVVRNFVKHRADGERFAQWAVRADEADLR

>CORE_REP|Org41_Gene2160#

MSESSERNSGAVAARPVQEHRYDVVIVGAGGAGMRAAIEAGPRVRTAVLTKLYPTRSHTGAAQGGMCAALANVEEDNWEWHTFDTVKGGDYLVDQDAAEIMAKEAIDAVLDLEKMGLPFNRTPEGKIDQRRFGGHTRDHGKAPVRRACYAADRTGHMILQTLYQNCVKHDVQFFNEFYVLDLVLTETDRGPVATGVVAYELATGDLHVFHAKSIVFATGGSGRMYKTTSNAHTLTGDGMAIVFRKGLPLEDMEFHQFHPTGLAGLGILISEAVRGEGGILRNADGERFMERYAPTIKDLAPRDIVARSMVLEVLEGRGAGPNKDYVYIDVTHLGEDVLEEKLPDITEFSRTYLGVDPVKELVPVFPTCHYVMGGIPTRIRGEVLRNNDDIVPGLYAAGECACVSVHGANRLGTNSLLDINVFGRRAGIAAAEYAERTDFVEMPENPAQMVQDWLALILSDHGNERVADIRTELQRSMDNNASVFRTEDTLKQALTDIHALKERYSRITVQDKGKRYNSDLLEAVELGFLLELAEVTVVGALNRKESRGGHAREDYPDRDDVNFMRHTMAYKEGTDLLSDIRLDFKPVVQTRYEPMERKY

>CORE_REP|Org101_Gene1042#

MALALSTGECQVGTVRPGSIDTLGRPSRAPNLASVMGRSNLRSSCVSAIYMEDLNNAMAKTIAYDEEARRGLERGLNALADAVKVTLGPKGRNVVLEKKWGAPTITNDGVSIAKEIELEDPYEKIGAELVKEVAKKTDDVAGDGTTTATVLAQALVREGLRNVAAGANPLGLKRGIEKAVEAVTAKLLDTAKEIDTKEQIAATAGISAGDSSIGELIAEAMDKVGKEGVITVEESNTFGLQLELTEGMRFDKGYISGYFVTDPERQEAVLEDPYILLVGSKVSTVKDLLPLLEKVIQAGKPLLIIAEDVEGEALSTLVVNKIRGTFKSVAVKAPGFGDRRKAQLADIGILTGGEVITEEVGLSLETAGIELLGQARKVVITKDETTIVEGAGDAEAIKGRVAQIRAEIENSDSDYDREKLQERLAKLAGGVAVIKAGAATEVELKERKHRIEDAVRNAKAAVEEGIVAGGGVALLQSAPALDDLTLTGDEATGANIVRVALSAPLKQIAFNAGLEPGVVAEKVSNLPAGHGLNADSGAYEDLLAAGVADPVKVTRSALQNAASIAALFLTTEAVVADKPEKAAAPAGDPTGGMGGMDF

>CORE_REP|Org113_Gene3852#

MPSDNSDFTINRRGLLALGGMAAAATATALGAPRAWAAPSATADADVIVVGAGLAGLVATSELAAAGRRVLLLDQEPEQSFGGQAHWSLGGLFFIDSAEQRLAGIKDSFDLARGDWFRTAGWDRGPDDTLGEDYWGKRWAENYLQFAAGEKQAWLRGLGMNWVPVVGWAERGQADGGIGNSVPRFHITMGTGPGVVEPFEKLVRDAAGKNVTFAFRHQVDELVVTGGAISGVRGTVLEPSGAARGTPSSRIKVGEFEFRAAQVIVTSGGIGANHELVRRNWPARLGKAPARMITGVPAHVDGRMLAIGESAGARLVNRDRMWHYTEGLKNYAPIWPGHGIRVLGAPSSMWFDAEGRQLPAPGIPSVDTLGTLDLIMRTGYDYSWFVLNKKIISKEFTLSGSEQNPELTNKDLAAYLANRALTDTPAPVKAFMDKGQDFVVADTLTELVAGMNKLTGADLIRLDSLRDQIAYRDNELGNPNSTDPRIVAIRRSRDYIGDNLFRTTDPHPILAPDAGPLIAIRMNILTRKTLGGLQTDLSGRVLDARGEPIRGLYAAGEVAGFGGGGVHGYRSLEGTFLGGCLFSGRQTGRAAAKESA

>CORE_REP|Org138_Gene6165#

MTQTDLHVAPSTLPAFTPLTTREAVSPRCDTPLPRHETALSPHDPALLSDREGALSELGGALSDCASAVSDRSGVNPGRDGAPPERAALSAQGGDLSDREGAGSGQVRPRAESGGAPPEREGFARGREPVTEYVAAQEVPVQQSASTHDEDRAPYVPLRDIVDPHRVGPTLEWLDDHAPHVIADELARMDAVTAGMVFRLLDKDRALDVFEELEPVDQQQILSGLRDERFRELVEEMDPDDRARMLREAPAKVAKKVLAGLSPRERRMTAQLLGYPEGSVGYYMTPEVVALPRNLPVAQALQWVRTKGGNAETVYTLPVVDGGRRIIGVVELRDLVLSSPDAMIADLVAAEPVFVRATDSAEKAARLMQGANLINLPVVDSEDRLVGLLTIDDAIEVIEAADSEDVARQAGAAPWEGHYMAAGVFQLARYRAMWLTLLLVAATLTVSVTDLFEGTLEQAAHLALFIPLIIGAGGNAGAQAATSCVRAVAVGEVRGSDLFRVVWRECRVGLLLGTMLALIGVVIGGLFVGMEIAAVVGITLVLICAWAATIGGTMPLLAKKLRIDPAVISAPMVTTLVDATGLIIYFTTAKLVLGI

>CORE_REP|Org98_Gene7027#

MLTEIRIDGLGVIATATAQFHAGLTCLTGETGAGKTMVVTSLHLLSGARADAGRVRLGAPRAVVEGRFTVDDVNDAARAEVAQVLEAAAAEPDDDGSVIAIRTVGSDGRSRAHLGGRGVPASVLADFTASLLTVHGQNDQLRLQRPDQQLSALDQFAGDAVGTALRKYQVLRRSWLDARTELLERTARSRELALEADRLKHSLNEIDAIAPEPGEDVRIVDEVRRLSDLDSLRDAAATAHGALAGPADTPEDGSGALEALGTARARIEAADDPALVALAPRLADAIAVVIDVTTELSGYLSDLPSDPGALDSLLTRQAELKTLTRKYAPDIDGVLAWAQEARTRLGSLDVSEEALAKLAAEVDTAADRVREAAKKLSGVRAKAAGKLAAAVSAELGGLAMGKARLEVEVRPLLAGAQDTAPLTVDGQELHAGHTGIDEAEFRLSAHSGAQSLPLSKSASGGELSRVMLALEVVLASSDHGATMVFDEVDAGVGGRAAVEIGRRLARLARTHQVIVVTHLPQVAAFADTHLVVDKSDDGKGAVNSGVRALTNDERVVELARMLAGLDDTETGRAHAEELLATARAEKAGAEAATR

>CORE_REP|Org105_Gene5302#

MNAHRVLRSPLGKIPIFRTDPMSTWGFLLTGSPSWVNRRYWHWLHSEPMTSVQQQPTPGPAGAPDIHTTAGKLADLRNRLEEAKHPMGEAAVDKVHAKGKMTARERILALLDEGSFVELDALARHRSVNFGLENNRPLGDGVVTGYGTIDGRDVCIFSQDVTVFGGSLGEVYGEKIVKVMDLALKTGRPLIGINEGAGARIQEGVVSLGLYGEIFHRNIQASGVIPQISLIMGPAAGGHVYSPALTDFVVMVDQTSQMFVTGPDVIKTVTGEEVTMEELGGANTHMTKSGVAHYVASGEQDALDYVKDLLSYLPSNNRAEAPRFPATDPIDGAIEDSLTEEDLELDTLIPDSPNQPYDMHEVIRRLLDDDEFLEVQAERAMNIIVGFGRVDGRSVGIVANQPTQFAGCLDIDASEKAARFVRTCDAFNIPIITLVDVPGFLPGTGQEYNGIIRRGAKLLYAYGEATVGKITIITRKAYGGAYDVMGSKHMGADVNLAWPTAQIAVMGASGAVGFVYRKQLAEAAKEGADVDALRLELQNEYEDTLVNPYVAAERGYVDAVIPPSHTRGQIVSALRLLERKMVTLPPKKHGNIPL

>CORE_REP|Org145_Gene4689#

MGGSCACPVLTLPVRNHCRSERCRFVRLPVRETGRAESAQHCRRTARRSQPVITATDLEVRAGVRTLLSAPGPALRVQAGDRIGLVGRNGAGKTTTLRILAGEGEPYAGKILRSTEIGYLPQDPREGDLDVLARDRVLSARGLDTLIRDMEKQQALMAEVADEAEREKAVRKYGRLEERFSALGGYVAESEAARICHSLGLPDRVLGQPLRTLSGGQRRRIELARILFSASDGSGGRSDRILLLDEPTNHLDADSITWLRGFLQNHDGGLIVISHDVELLEAVVNKVWFLDAVRGEVDVYNMGWKKYLDARATDEQRRRRERANAEKKASALKAQAAKLGAKATKAVAAQNMVKRAERLLDELDEVRVADKVARIKFPEPAPCGKTPLMAENLTKVYGSLEIFTGVDLAIDRGSRVVVLGLNGAGKTTLLRLLAGVEQPTAGQLVPGHGLKVGYFAQEHDTLDDQATVWENIRHAAPDAGEQDLRGLLGAFMFSGPQLDQPAGTLSGGEKTRLALAGLVSSAANVLLLDEPTNNLDPISREQVLDALRTYAGAVVLVTHDPGAAEALSPERVILLPDGTEDHWSAEYLELIQLA

>CORE_REP|Org119_Gene804#

MTKKPESQFLGPEQRRTAWERFGKDHFDVVVIGGGVVGAGIALDAATRGLQVALVEARDLASGTSSRSSKMFHGGLRYLEQLEFGLVREALRERELALSTLAPHLVKPLRFLYPLTHRAWERPYVAAGLVLYDTMGGAKSVPGQRHLSRMGALRLSPGLKRSALIGGVSYYDTVVDDARHTMTVARTAAHYGAVIRTSTQVVGFLREADRVVGVRVRDSEDGRTAEVRAHVVINATGVWTDEVQALAHQRGRFHVRASKGVHIVVPRDRIVSDAAIILRTPTSVLFIIPWGTHWIVGTTDTDWNLDLAHPAATKADIDYLLDRVNEVLVTPLTHDDIDGVYAGLRPLLAGESDETSKLSREHAVARVAPGLVGIAGGKYTTYRVMAYDAVDEAAQDIPARVSPSITEKVPLLGADGYFALVNQTVQLAEAYGVHPYRVKHLLDRYGSLIDEVMAMADGKPELLQPITDAPSYLQVEAVYAAAAEGALHLDDILARRTRISIEYSHRGADCAEEVAQLVAPVLGWDDAEIDREVTTYRARVEAEIRSQTQPDDASADALRIAAPEPRPEILEPVPADGSASKAVRQPNS

>CORE_REP|Org216_Gene3082#

MLIRLLRTYLSPYRAQLAGVVALQLVSVIAMLYLPSLNADLIDNGVTKGDIDYIWHTGLWMLAVTAVQIVASASSVFLGAQAAMSAGRDLRAALVHRVGTFSAREVGLFGAPSLITRNTNDVQQVQLLVVMSVTVLVMAPIMCVGGIIMALREDLKLSWLLLIAVPALALAMGLVVARLVPGFREMQARIDVVNRVLREQITGIRVVRAFVRERQETWRFGLANTDLTEASLRVGRLMALMFPVVMLISNVTTVAVIWFGGHLIDDGELQIGSLTAMLSYIMQILMAVMMASFLAMMAPRAAVSADRIGAVLTTESSVVPPEFPKPFAGDPGRVEFAAAEFAFPGAEKPVLRGIRFTVEPGTTTAIVGSTGAGKTTLLNLIPRLIDVTAGAVYVGGTDVRELDMELLREQIGLVPQKAYLFSGTVASNLRYGRPEATDEELWRALEIAQAADFVRDMPQGLETPVAQGGTTVSGGQRQRLAIARALVRRPRVYLFDDSFSALDVATDARLREALRPETRDASVIIVAQRVSTIRDADQIIVLEDGEMAGIGTHEQLLRDCAEYQEIVASQLSAQEEVR

>CORE_REP|Org128_Gene3333#

MSATPFRGWWRANCDLNPISVSASRGRVRPVSYTHGVWDAPLLGETIGANLDRTVAIHGDRDALVDRVTGVRWSYREFAAEVDAVALGLLEAGIGKGDRVGIWSPNRAEWTLVQFATAKIGAILVNINPAYRSEEVRYVITQAGIRMLISAREHKSSNYAEIIGRVRPECPDLEQVVLFDSAAWEALVAAGRAADPSRLAEAGTRLTADDPINIQYTSGTTGFPKGATLSHHNILNNGYFVGELCGYTEADRICIPVPFYHCFGMVMGNLAATSHGAAMVIPAASFEPRATLAAVAEERCTSLYGVPTMFIAELAHPNFESFDLSSLRTGIMAGSPCPVEVMKQVIERMGMAEVSICYGMTETSPVSTQTRRDDTITQRTATVGRVGPHLEIKIVDPDTGSTVPRGEPGELCTRGYSVMLGYWNDPDKTGEAIDAARWMHTGDLATMDDDGYVAITGRIKDMVIRGGENIYPREIEEFLYTHPDILDAQVVGIPDPKYGEELVAWIRVREGAATVDAPTLAQFCDGRLAHYKIPRYVHVVDEFPMTVTGKVRKVDIRATSVRLFGVPELDQQQTGEQ

>CORE_REP|Org5_Gene6751#

MSSSSLHVRVGVCNLGLVSSDITATAAWRKLHDHHSAIADRHLREFFADDPDRGRELIVEAGELRVDYSKHRITRETLDLLLDLAATAGVARRRDAMFAGEHINTSEDRAVGHVALRLPAGASMMIDGADAGVAVHEVLRRMGDFTDGVRSGQWRGATGERITTVVNIGIGGSDLGPAMLFQALRHYADAGISARFVSNIDPADLTAKLDGLDPARTLFVVASKTFSTLETLTNATAARRWLVAALGEDAVAEHFVAVSTHAQRVADFGIDTANMFEFWDWVGGRYSVDSAIGLSIMVVIGRERFAEFLAGMHSIDEHFVSAPPERNAPILLGLLGVWYSNFFGAQSRAVLPYSNDLARFPAYLQQLTMESNGKSVRLDGSPVTTSTGEIFWGEPGTNGQHAFYQLLHQGTRLVPADFIGFARPTDDLATRDGSGSMHDILMSNLFAQTKVLAFGRTAAEIEAEDGDAPGFDPALVPHRVMPGNRPSTTILAPQLTPSVVGQLIALYEHQVFVEGTIWGIDSFDQWGVELGKQQALALEPLLTAAEDPAPQSDSSTDALIRWYRGNR

>CORE_REP|Org105_Gene607#

MAGAARAPATLVPNARAVSYAICHISRRFGVGAIDTGQLIAEHYRLVERIGSGGTGVVWRAIDERLQRSVAVKQIHIKPSLPEAERDVLRQRAIREARNAARFQHPNAIVVFDITEHNGDPCLVMEYLKSRSLAQVLSAQGAVPLNQVARIGEQVASALIAAHQAGIVHRDVKPGNVLLDDHGTVKITDFGISRAAGDVTLTETGLICGTAAYLAPEVARGADPTPAADVFALGATLFHALEGEPPYGASSNPLAVLYAAANGQVSEPRNAGPATDFLLQLLSPQPEDRPTMRMARDQLAAFADAGADAVPAGFVPASEAYGRRNGDGATEVVATRALRSRAAAAGPATERQPAPRRSSVAVDTAAHRPVEPARDHPDTAAHPRTTTQPRPASPAAGKRRAVLIGALVGAVVAVSALLVSAFNSADDDSSPQASASSVATAPSGDASTSAAVPLLGQTPSVGGKVTDIGAAGQLVERFYSDPESSWSLLTPAAQKVYTDQQGFRQYWSADGRTIQSFGRIYAPNGVNADGSVDMRVTGLTYGGQSKNPDLRIIDAGGGRLLIDSDTR

>CORE_REP|Org113_Gene5713#

MRPEVVVVRWIFMLDRARVTKGDKVILDDVSLTVLPGAKIGVVGPNGAGKSTVLRVMAGLELTAGGEAVLAPGITVGILAQEPELDETATVRGNVEAAVAGTQALLARYTEIAERLADDADEELLAELGALQEQLDRRGAWDLDSRLDQAMDALRCPPPDAGVTTLSGGERRRVALCRLLLQRPDLLLLDEPTNHLDAESVQWLEQHLSGYPGTVVAVTHDRYFLDNLAEWILELDRGHAHPYRGNYGIYLDTKATRLRVEGRKDAERLRRLRRELEWIRSGPAARQAKGAARLRRYEEMAAAADGARVRTFDEIRIPPGPRLGGLVVEADHVDKSFGDHTVIRDLSFSLPRNGIVGVLGPNGAGKTTLFRLLIGELTPDAGKIRIGDSVEISYVDQNRVRIDPGRTAWDVVSGGHAVIGVGTMEVPSRAYLAAFGFRGVDQQKPSRLFSGGERNRLNLALTLKQGGNVLLLDEPANDLDTETLDSLENAIDEFAGCVIVTAHDRWFLDRLATHILAWEGTAADPGRWFWFEGNFAAYEQNKLARLGPDAARPHRLTHRRLTRD

>CORE_REP|Org5_Gene1415#

MVAPSSSRKPQKVNDMTAAADPQAFAARITADLTGPGGPFEMGVEEVLGAPIPLMRNRRRSMADLFHAAAAWGDHDYLVTADRRLTFAEHGAAATALARGLAERYGVGKADRIGILAANTPEWVMTFWAAQLLGAIPVGYNAWWAPREIAYGLEHTQPTVVVADAKRAALLTDTGIPVLTMESDLPALVADHPGPAPEAAIDEDDPAVILYTSGTSGRPKGVVHSHRNLLAVCDYHRFTDAMMVAFRGQQLGSGPSPRRFLLTSPLFHIASLHNLIVPRLATGATVVMHTGSFDPDRVLALMERQRVTNWGAVPTMVSRMLDCDLSRYDLSSLVAFSLNAAPSSPAFHQRLRSELPMAEVALTTSYGLTESGTAATVATPPVLAAFPDTVGMPIIGVSVEIRDPDNKPLPDGEEGEICVRSPYVMLGYWNDPAATAQAIDAERWLHTGDFGILEQGRLRLSGRRSDLILRGGENVYPIEIENALDEHPEVLESAVLGVPHDDLGQEVAAVVVVTDPGAVTEEQLRAFTAERLAYFKVPARWVITAQPLPRNATGKVVRREIEI

>CORE_REP|Org52_Gene3143#

MSPALAAGLQIASVVAVLALVYVPLGDYMARVYTSSSDLRAESWLYRLARVDPRAEQTWYGYAGSVLGFSLAGVLVLYVLQRIQGVLPLSHGLAGVSPAVAFNTAVSFVTNTNWQSYVPETTMSPLTQSAGLAVQNFVSAAVGMAVAVALIRGLVRVGRGGEVGNFWVDLTRGTLRILLPLAFVIALILLSQGVIQSYRSGFTGVGLDGRPVTTALAPVASQEAIKELGTNGGGVLAANSAHPFENPTPLSNVVQILAILLIPVALTRTFGTMIGNRRQGLTVLAVMAGIYAVILGVTTAAESGARGAAATAAGAMLEGKEVRFGIPGSVLFAVSTTGTSTGAVNSAHDSMSPLGGGAVLVNMLLGEIAPGGVGSGLYGILVLAVIAVFVGGLLVGRTPEFLGKKLRRREITLAALAVLVMPALVLIGTAITVILPDTAAALGNSGDPGTPGAVHGFSEVLYAYASASNNNGSAFGGLTVTSDWFQSSLGLCMLFGRFLPILFVLALAGSLAAQPRTPATAGTLPTAGAGFAGLLTGTVVLVAALTFFPVLALGPIAEALQ

>CORE_REP|Org202_Gene4973#

MRVRRPGRLDPVSTTLHARGLSAGHGERTLFDDLDLTIAPGDVIGLVGVNGAGKSTLLRMLAARETPTGTITLSPPDATVGYLAQEPERVPGETVLDFLGRRTGVTAAQRAMDAAAERLAEGGTDEYSPALERWLALGGADLEARAQEVAADLGLAESLADGLGTPMTALSGGQAARAGLASVLLSRYDILLLDEPTNDLDLDGLARLEDFVRGVRVPLVVISHDREFLARTVNRIVELDLAQQQVGLYDGGYEAYLAEREIARRHAREAFEEYADTRAALETRAQMQRNWLEHGVRNARRKARDPRKLDSDKAGRKMRAEATEKQAAKARQTQRRIERLEVVEEPRKEWELRMTIAAAPRSGAVVATATDAVVTRGDFRLGPVTTQIDWADRIVLTGANGAGKSTLLGLLLGRIAPDSGSAALGSGVEIGEVDQARSLFRGTTPLAERFGREMPDWPDAEIRTLLAKFGLRGPHVLRACDTLSPGERTRAALALLQARGVNLLVLDEPTNHLDLPAIEQLEQAVDSFTGTLLLVTHDRRMLDSVRATRRWHLRDGLLHED

>CORE_REP|Org129_Gene4552#

MCAHERSLVPVQPSPQVLRPAVALSTPVQAVAELTGARLNSGDPAGIEITGIEQRSNAVQPGDLFAGLAGAKAHGARFAADAVERGAVAVFTDAAGAELIGELDVPVLVHDDPRAVLGELSAAVYGHPSRRLQVIGITGTSGKTTTSYLVEAGLTAAGLSTALIGTIETRIARRVDPNPEETAAGASAAVEYRRVPSALTTPEAPQLHAMFALMVEQGVRAVVMEVSSHALALGRVDGVHFSVGAFTNLSQDHLDFHADFEDYFAAKRRLFVPDPGAPQRQVAADTCVICVDDAWGRRLAREAGGRARVVTVATADCPTGETEPEWTVTGAAALADGGQQFTAIGPAGEISARLRLPGRYNIANGLLAIAVCAAAGVDAAVAAAALGEVDVPGRMQRVNAGQDFLALVDYAHKPAAVESVIATLRRHLRDSGGRLAVVVGAGGDRDAGKRPLMGATAARGADLLVITDDNPRTEDPAEIRAAIRAGALGIAEAERGEVREIGDRAAAIAAAVDWARRGDVVLVAGKGHETGQEIAGVKYPFDDREVLGQALERKTKDLTVS

>CORE_REP|Org4_Gene1527#

MGGVSDMATQLGNKVSAQADSADERYRAAAFLKRSINKVFPTHWSFLLGEIALYSFIILLLSGVYLTLYFDPSMTEVVYDGSYQPLRGVTMSRAYETALNISFEVRGGLFVRQVHHWAALLFAASIIVHLFRVFFTGAFRKPREANWVIGSLLLILAMFEGYFGYSLPDDLLSGTGLRAAFSSITLGMPIIGTWLHWLMFGGDYPGTIIIPRLFIAHVLLFPGIMLALIAAHIALVWYQKHTQYPGPGRTEKNVIGARIVPVFSLDQGAFFAFTLGIVAIMSGVFQINAIWTMGPYNPAQISAGSQPDFYMMWTDGMMRLIPPWELYLGRYTVPAPVWGALLMGVVFTVLITYPWIEKRLTRDTAAHHNLLQRPRDVPVRTAIGAMAIAFYVVLTLSCVNDIVAYKFDISLNATTWTGRIGLLLLPPLAYFVAYRVCLGLQRSDRAVLEHGVETGVIKRLPHGEYIEIHQPLGPVDEHGHPIPLAYQGAPVPKKMSKLGLAGKPGTGSFLRADPWQESERNHETDHAEEHKQLAVLRDYQERDQRNGNGSHGNGSSDS

>CORE_REP|Org18_Gene2143#

MLSHPERLPLLTASTDGAVSSGPRGLPAEVSRRRTFAVISHPDAGKSTLTEALALHAKMISEAGAIHGKAGRKSTVSDWMEMEKARGISVSSTALQFNYRAAGSDIDNVINLVDTPGHSDFSEDTYRVLTAVDAAVMLIDAAKGLEPQTLKLFQVCRHRGIPVITVINKWDRPGRAPLELLDEIDERIGLTPTPLFLPVGIAGDFRGLLRRGPDGEAVEYIHFTRTAGGATIAPEESLTPEQAQAREGEAWETAAEESELLSATGQDHDQELFLAGQTSPVIYASAMLNFGVRQLLETLVALAPAPAGRRDVDGGMRETSDPFSAVVFKVQAGMDTAHRDRLAFMRIVSGEFERGMVVTHAQTGRPFATKYALTVFGRERATVDTAYPGDVVGLVNATALAPGHTLFVDKKVEFPPIPSFAPEHFAVLRAQSAGKYKQFRKAIDQLDSEGVVQVLRNDARGDASPVLAAVGPMQFEVVTARMQAEYNVETQMDHLPYTLARRTDAASAEELGRQRGVEVFTRSDGVLLALFSDKWRLQYIEKEHPGLTLEPLVATAD

>CORE_REP|Org215_Gene1101#

MTPPPQTTPLALHDAARAYGDAPAVVDGAVRLSWAELLDSVRETARALLARGIGSGDRIGIWAPNTHHWVTAVLATHYVGAVIVPLNTRYVAEEAADVLARVDAKALFIAGPFLGRDRLAELRAAAPDLKIGTTIVIPGDTGPGGTDDAGTAVTDDAAAIGTGGVGVSAAGDAAARTGDDTLTWRNLAALAEQVSAADAIARAESVSPDDLSDILFTSGTTGRSKGTLIAHRQALAGARAWSECATLNSTDRYLVVPPFFHNFGYKAGILACLVTGATIVPQATFDVPETMRLVQDHRITVLTGPPTIYQTILEHPARRDADLSSLRVAVTGAATVPVVLIERMRTELEFDVVLTAYGLSESGGFGTMCRPEDDAETIANTCGRAIGDFEVALADNGEVLIRGSQVMLGYLDDPVATADTIDCDGWLHTGDVGTLDGRGYLKITDRLKDMYICGGFNVYPAEVEQALARLDGVAETAVIGVPDERMGEVGKAFVVRKAGSGLTADDVVAHAKTLLANFKVPRYVEFRDQLPYSAAGKVLKRQLRDDTGRAENEERA

>CORE_REP|Org1_Gene1663#

MDFLLHPCASAVGDGHVGVGTGFAVRGTRGQRLIAGPAGGNWQDRGMGLRRGCGVLVAALAVVAAGCTIERGEQAGPMPAAPAGLERFYEQAVPWGPCAGFTDDQVRLPPNAQCARIEVPVDYADPAGPTAQIALSRIPASGAKIGSLLLNPGGPGVSGLDTVAIANQTPLSERFDRVGFDPRGVGASTPAITCLTPPEADAERAERPEDNTPAGIAAAEADNRDYAAKCVQRSGAELLEHVGTREVVQDMDVIRAVLGDPKLTYLGYSYGTKLGSLYAEKFPDRVRALVLDGAVDSSQDPVQESLRQAAGFQRAFDAYAADCARTPDCPLGTDPAQAVARFRELVDPLWERPAATTDPRGLSYNDAITGVTQTLYTDDLWQVLTLGLQELRDGRGDTLLQLADLYDGRRDDGTYRNTQDAFNAIRCVDDPRVTDPAVAARQDTEYRKAAPFLDDGRGTGAAPLELCAAWPVPNSGEPHSISVQGLPTTVVVSTTEDPATPYQAGVDLAAQLGAALVTFRGNRHTAALVAGNECLDSAVIAYLVDLTIPPAGLTC

>CORE_REP|Org24_Gene6553#

MVPSRRRSPARCSTPDWRGAERYERARWTGAGMLTNGALIADRYRLHRLIATGGMGQVWEALDTRLDRRVAVKVLKAEFSADPTFRHRFRTEAKTTAQLNHPGIAGIYDYGETMDPAGGETAYLVMELVSGEPLNAVLNRLGRLSVAQGLDMLEQTGRALQVAHAAGVVHRDVKPGNILVTPTGQVKITDFGIAKAVDASPVTKTGMVMGTAQYIAPEQATGEDATAASDVYSLGVVGYEALAGQRPFTGDGALTVAMKHVRETPPPLPPDLPPNVRELIEITMAKEPGQRYTSGGEFADAVAAVRAGRRPPPPSGLAGPMTSGATRVLPPGPTVILPTAARGDAATVRYPTPPHAQRAQQPPVATAMMQGPNTPPPGTPPIGGRTAEQGGGRFTNSQKALAGLGVGALVVGAAAAFVLLSGDPPDSTPPTKTSAVVVPPPVPTTTTTTEPPTTTRYVPPPPTVPPTTEEPLPTTTEPPPTTTTQPPTTTPQQPSTTKPAPTTTVKPSKTTIEPPFEIPSWPPTVPGGAGGAFGTTRPAHSSTPAPAAQQGLP

>CORE_REP|Org175_Gene4706#

MTDQPSNDNDSSDYALPDPSGPAPAPNGSVPNGSVANGAGPIDTEVAEPEPIQAQPIGAGGGNSGFVVVANRLPVDLEKLPDGSTRWKRSPGGLVTALEPVLRSNKGAWVGWAGVPDVDVDPIIEDGLELHPVPLTAQEVEDYYEGFSNGTLWPLYHDVIVRPVYDRKWWAAYVQVNRRFAEATAKVAAEGATVWVQDYQLQLVPKMLRMLRPDLTIGFFLHIPFPPVELFMQMPWRTEIIEGLLGADLIGFHLPGGAQNFLYLARRLAGQPTSRGNVGVRSKLGVVQVGFRNVRVGAFPISIASAELDEHSRRRSVRERAAKIRAELGNPKNILLGVDRLDYTKGIDIRLNALEELLMEGRVDPSDTVMVQLATPSRERVQSYIKMRGDIERQVGRINGEFARVGYPVVHYLHRPIPREELIAFFVAADAMLVTPLRDGMNLVAKEYVACHSGLNGALVLSEFTGAAAELRQSYLCNPHDLDSVKDAIASALTDDRDTKRRRMRSLRRQVLTHDVDRWARAFLDALAHDQVAGSALLSDEEDMYSEPHR

>CORE_REP|Org13_Gene6328#

MTEYPWLTTLWVLPLAGAVVVLAVPAGRRTVARVTGLVLSLATLAVAIVVAVRFDPGGPQYQLVESHRWIPAFGAGYTLGVDGIALVLLLLTAALVPLLILAGWKDDREAGGGRRVAHIYVALTLIVESMVLISFVSLDILLFYVFFEVMLIPMYFLIGGFGPRTSGGESAAAELALRQQRSRAAVKFLLYNLFGGLIMLAAVIGLYVLTARAHLGGAGGTFDFRAVTAAANSGQLGAGPAVLNALFLGFMFAFAVKAPLWPLHTWLPGAAVSATPASAVLMMAVVDKVGTFGMLRYCLLLFPAASTTYAPMISVLAVIGILYGALLAIGQTDVMRLIAYTSISHFGFIILGIFAMTNQGGSGATLYMVNHGISTAALFLIAGFLVSRRGTRVIAEFGGVQKVAPVLAGTFLIAGLATLSLPGLAPFVSEFLVLAGTFTRYPVAAVFASGALVLAALYVLWMYQRMMTGPVRKGNERLQDLLPRELLVVVPLLAALLVLGAYPKPVLDRINPAVAGTLTTIGKHDPAPTVAPDAATVPAATTPSGGNHR

>CORE_REP|Org33_Gene4362#

MHATPPAWQHGWCMRKSETFDITSTSGANDDADQAEFTSDIDLTDGDVTDENDDYAREGDVLAERAARMKRNGWTAEPTAGELQLEERSSLRRVAGLSTELTDITEVEYRQLRLERVVLVGVWTSGTAAQAEASMAELAALAETAGSQVLEALIQRRDKPDPATYIGSGKADELRAVVLETGADTVICDGELTPAQLTALEKVVKVKVIDRTALILDIFAQHATSSEGKAQVSLAQMEYMLPRLRGWGESMSRQAGGRAGSNGGVGLRGPGETKIETDRRRIRERMAKLRREIREMKTARETKRARRASSGIPQVAIVGYTNAGKSSLMNALTGSGVLVQDALFATLDPTTRRAELDDGREVVFTDTVGFVRHLPTQLVEAFRSTLEEVTGADLLLHVVDGSDPDPAGQIKAVREVIADVIKESGAAAPPELLVVNKLDAISPMRRTELRGLLPDAEFVSAHTGAGVDGLRARLNEVLGGLDVEVGVLLPYTRGDLLARVHADGRILESAHEEGGTRLRARVPHALAAALSEYAHAGAAGEPVGAERT

>CORE_REP|Org118_Gene6962#

MQVAAHSAAGRAPGSATDPQSPRTYEVRTYGCQMNVHDSERLSGLLEDAGYTKATGGQTADLVVFNTCAVRENADNKLYGTLGHLAPIKAERPGMQIAVGGCLAQKDRDTVVRKAPWVDVVFGTHNIGSLPVLLERARHNEQAQVEILESLEAFPSTLPARRESAYAGWVSISVGCNNTCTFCIVPSLRGKEVDRRPGDVLAEVQALVDQGVLEVTLLGQNVNSYGVNFAEPALPQGHPADFAPEHRDRGAFAKLLRACGSIDGLERVRFTSPHPAEFTDDVIEAMAQTPNVCPQLHMPLQSGSDRVLKAMRRSYRKDRYLGIIEKVRAAMPHAAITTDIIVGFPGETEEDFQETLDVVRQARFTSAFTFQYSIRPGTPAATMADQVPKAVVQERYDRLIELQEQISLEANRALIGTEVELLVAEGAGKKNAATARMSGRARDGRLVHFRPGDAAIRPGDIVTVDITEAAPHHLIADGPVHTHRRTRAGDAHERGVLPKTAPIGVGLGLPRIGAPETPPAAAGCETLPAAAGCETPPAAAGCDTGCGA

>CORE_REP|Org113_Gene4117#

MTAQAWILIAAIVAVLLVAFVAGFVLYKRRRVSIAPAAEQDKELTDRSGGYTASGGFSFSQGGAGSGTLTPPRPEPVPIERTDDEGQPHIGDDAAVPRDSARRTITDVRLPEPETLTDQQSGGATATAPVAEPETDGAAAPAEPATEIETPVDSAPADTTPTETALTETAPAETAPTEAAPTDTAPAETALPDTAPAETTPPKAAPDDTIAPAPQQPVTTDGGPATAPADAAPVAEPAIAEIEPTAGRLTKLRGRLSRSQNAVGKSLLGLLGGGDLDEDSWEEIEDTLVMADLGTSVTTTVVERLREELAARSVRTSEQARQVLRDVLVEALRPELDRSIRALPHADHPSILLVVGVNGTGKTTTTGKLARVLVADGRRVLLGAADTFRAAAADQLQTWGERVGADTVRGKEGADPASVAFDAVTAGISEGVDAVLVDTAGRLHTKTGLMDELGKVKRVVEKKAEVDEVLLVLDATVGQNGLTQARVFAEVVDITGVVLTKLDGTAKGGIVFQVQHELGVPVKLVGLGEGADDLAPFEPGAFVDALLG

>CORE_REP|Org138_Gene2201#

MIEMTLREIADVVGGTLHDCPDPEVTVTGAVEFDSRRIGSGDLFLALPGARVDGHDYARQAVAAGAVAVLAARPVGVPAIVVTPPSAAPAARDGDTGADGDAAPEDRRTSRALALAADTDGSGAAVLAALAKLARTSVDRLVAAGGLTVVGVTGSSGKTSTKDLLAAVLSPLGPVVAPPGSFNNELGHPWTALRADADTRFLVLELSARGRGHIRALTEVAPPGIGVVLNVGTAHLGEFGSREAIAETKGELVEALPATGLAVLNADDPLVSAMAARTAARVVQVGQAAGADLRATDVTLDEQARARFTLRRGDESVDVTLAVHGEHQVGNALAAAAVALECGADLATAAAALGAAQAVSERRMDVRTRADGVTVINDSYNANPDSVRAALKALVTMAKSGDTARRSWAVIGEMGELGEESVLEHDRIGRLAVRLDVDRFIVVGAGRPVRALFQGAVQEGSWGEEAVHVPDIAAAVELLDSELAPGDVVLVKASKSVGLWAVAEHLTAAAGPDARPAAGSDSRPAADSKSRPAADSIARPAAAEEAAR

>CORE_REP|Org162_Gene5667#

MGRENADPIPPGVPPVSQAGRPVVLIADKLAQSTVDALGDGVEVRWVDGPNRAELLAAVPEADALLVRSATTVDAEVLEAGKKLQIVARAGVGLDNVDVPAATERGVMVVNAPTSNIHTAAEHAVTLLLAAARQIPAADATLREHTWQRSKFNGVEILGKTVGVIGLGRIGQLFAQRLAAFETKIIAYDPYTSPARAAQLGIELVSLDEVLERADFISIHLPKTPETKGMLNAETIAKTKKGVIIVNAARGGLIDEQALADAITSGHVRAAGIDVFETEPCTDSPLFDLPQVVVTPHLGASTAEAQDRAGTDVAKSVQLALAGEFVPGAVNVTGGSVTDNVAPWLEIVRKQGALLGALADELPVSLEVQVRGELAADDVAVLELSALRGVFSALIEDAVTFVNAPSLAKDRGLEAAVTTHTESPTHRSLVDLRAVFGDGRTLNVAGTLTEPQQVQKIVNINGRNYDMRAEGLNLAVLNYEDRPGQLGRLAGKLGEAGIDILAAQLTQDLDKEGATVVLRVNQEVPAEVQASIAEAVGAAKVAQVDLS

>CORE_REP|Org102_Gene2110#

MPGERSGGEKNDFRRTIGWCSVSTPNPSSSGTADSAASGQAKAAAARAAVEHDVPEQMRIRQEKRERLLAEGREAYPVVVERTHALAEIRTAYPDLAPDTQTGLMVGIVGRVIFMRNTGKLCFATLQEGDGTKLQAMISLNGVGAESLAAWKADVDLGDFVSVHGEVIASRSGELSVMADSWAMAAKALRPLPVAHKEMNEESRVRQRYVDLIVRPEAREMARTRVAAVRALRNALERRGFLEVETPMLQTLHGGAAARPFVTHSNALDLDLYLRIAPELFLKRCVVGGLEKVFEINRNFRNEGADSTHSPEFAMLETYEAYGTYDDSATMMRELIQEVAQEVYGTQVVTLADGTEYDLSGEWTTVEMYPSLSESIGVEVTPETTVEELLALADRVGLEIPEGKGYGHGKLVEELWEHVYGDKLYAPTFVRDFPVETSPLTRQHRSKHGVTEKWDLYVRGFELATGYSELVDPVIQRERFVDQARLAAAGDDEAMRLDEDFLAAMEHGMPPTTGTGMGIDRLLMALTGLGIRETILFPIVRPSAR

>CORE_REP|Org150_Gene4572#

MSTTTRSETSPIRSRLDTTSQTFGTNREAQLRNLAELDDQLDLARAGGGERYIRRHHERGRLLARERIELLLDRDAHFLELSSLAAWGTEFTTGASVVTGIGVVAGVEVAIIAHDPTVRSGAMNPWSLKKTLRALEIARTNRLPVINLVESGGADLPNQANLFVQAGQIFHDLSDLSARGIPTIALVFGNSTAGGAYVPGMCDHAVLVDQQAKVFLGGPPLVKMATGEDADDEDLGGAAMHSRVSGLADHFAVDEHDAIRIGRRIVSELNWRKQGPGPTLPADPPLYDPEELLGIAPADFRVPFDPREVIARVVDGSRFGEYKPEYGTSLVTGWASIHGFPVGILANANGVLFSEEAEKATEFILLANQTDTPLVFLQNTTGYMVGTTYEQRGIIKDGAKMINAVTNSKVPHFTINMAASFGAGNYGMSGRAYSPRFMFAWVGAKLAVMGAAQLAGVLSIVGKAAAANSGREFDEQADTRRRQEIEAQIAAESHSFFISGKVYDDGVLDPRDTRTVLGIALSAAHSGPVEGRRGYGVFRM

>CORE_REP|Org119_Gene7095#

MLVTYGSPVLVQRLVARPGGDGVEPSWRGRTQSIRRPPAPEPHHLTDARVRLPYSGSDAGGGVPESTRVHPKPGCCADGGSTPFSLVRAETTTGVQQVRSGGILASSRWRPSRVRLAASLAAISALALTGCGTDVDDITVGPGKGWPAAFHDGRNAGTSPVTGAKKIALSWSRPIGGPIAEPVTIGPDGQFFLTTLSRDCMLFSGQMATGRKRFCSKLGPSAISAPSVVDGATNVYVGDDDAVNSYNYLGQPRWRTPIGGTPVSTQFTGDGRLLVVTQSGQVDVLSRQTGERTVPTTQLLGEPDFLEYPNLTRPAAGQGLDDCRTGGPQCPVANISAVDAASGRFFVTVWKPGHPAAALVALRYADNKIQQEWSAELLSGGSGTSPVLSSDGKTLYVGDNSKRLIAVDTADGRTKWVHQLEWAPQGGFSVSDAGLIIPAGDDGYLLALRDTGDAAETVWERKDLALRGTPVQTAGGTGYTTAAIGDGLNLITFDTKTGATIDSDVLPGAQGSTTGTSIGPKGEVLVATRIGELFTFEPER

>CORE_REP|Org210_Gene4354#

MSRRVTDNGRSDALEGGSLRDHGERREGRDGRSRSAAGARHVHTLIVGSGFSGLGLAIRLSRQGRDDYLVLERGNDVGGTWRDNTYPGAACDVPSQLYSYSFALNPNWSRSFSKQPEIQSYIQGVADRHGVRDKHIFDCEMTGARWNEEQARWEVQTSKGAFTADILVSAVGALCEPNLPDIKGINDFRGRIFHSARWDHDADLTGERVAVIGTGASAIQIVPSIAPKVAHLDVYQRTAPWLLPRIDRPYTLPERLAFKYVPGVQKLSRAAIYAARETQVVGLAKFPPAMLALEGLAWLKLRLEVPDAQLREKVTPNFRIGCKRMLISNEYYPALGRDNVDVVTDGIREIRANSIVTADGTEREIDALIVATGFHVTDSPVYETISGRDGRTLTELFDEIGQQGYKGAAIHNFPNMFFLLGPNVGLGHTSMVYMIESQINYIADAIATFDQRGLRTVEVRKDAQDSYNRDLQDRMSNSVWLNGGCASWYLDKHGNNTTLWPDFTFRFRKLTEKFDVAAYDTTRSTDGAAGPDLKVVAAQ

>CORE_REP|Org24_Gene2768#

MERTAHGDNRTAHGGRGQLTVTGAPTTVTTGNPLRDERDSRVPRIAGPCSMVIFGVTGDLSRRKLLPAIYDLANRGLLPPGFALVGFARRDMSDDEFADLVHESIKSSARTTFREEVWQQLREGLRFVQGTFEDDGAFHRLATTLKDLDRDRGTGGNHAFYLAIPPTEFPVVLDQLSKNGLAQPAPGAGDPAPWRRVVIEKPFGHDLDSAQELNALVNRVFPEQTVFRIDHYLGKETVQNILALRFANQLFDPIWNANYVDHVQITMAEDIGLGGRAGYYDGIGAARDVIQNHLLQLLALTAMEEPVSFQPKQLQIEKIKVLSATKLVEPLDETTARGQYTAGWQGSEPVAGLLQEEGFDPDSTTETYAAITLAVETRRWAGVPFYLRTGKRLGRRVTEIAVVFKRAPHLPFDQTMTEELGQNALVIRVQPDEGITMRFGSKVPGSSMEVRDVNMDFSYGEAFTEDSPEAYERLILDVLLGVPSLFPVNEEVELSWRILDPVLERWAADGRPEPYEAGTWGPESADEMLARSGREWRRP

>CORE_REP|Org218_Gene4924#

MTITQDPRASAAVSDNYAAHQALVGELRERLAATALGGPEKARQRHIARGKLLPRQRVDQLLDPGSPFLELSPLAANGMYDDECPGAGVITGIGRVSGRECVIVANDATVKGGTYYPLSVKKHLRAQEVALQNHLPCVYLVDSGGAYLPHQDEVFPDREHFGRIFYNQANMSAKGIAQIAAVMGSCTAGGAYVPAMSDEAVIVRDQGTIFLGGPPLVKAATGEVVSAEELGGGALHSRTSGVTDHLADDDQDALRIVRRIVSTLGPRPESPWEVRTPIEPAAPVEELYEVVPVDLRTPYDVREVITRIVDGDPDGGSGFHEFKAEYGKTLVTGFAHIHGHPVGIVANNGVLFSESAMKGAHFIELCDKRKIPLLFLQNITGFMVGRDYEAGGIAKHGAKMVTAVACARVPKLTVVIGGSYGAGNYSMCGRAYSPRFLWMWPNARISVMGGEQAASVLSTVRGDQLDSSGKPWTEEDQEAFKAPIRDQYERQGNPYYSTARIWDDGVIDPADTRTVLGLALSVCAQAPLEPVSYGVFRM

>CORE_REP|Org152_Gene4600#

MDPAARKPIKRALVSVYDKTGLIELASGLHAAGVELVSTGSTAGKIADAGIPVTKVEDLTGFPETLDGRVKTLHPRVHAGILADTRREEHVDQLVELGVEAFQLVVVNLYPFTQTVASGATVDECVEQIDIGGPSMVRAAAKNHPSVAVVVDTRDYDDVLVSVRDGGFTLARRTELAAKAFQHTATYDVAVASWMTSVAVPAAAPATASEGAASEGAESEGAQRFPEWIGATWTRQSVLRYGENPHQAAALYTDAAGPAGLAQAQQLHGKEMSYNNYTDADAAWRAAYDFEAPAVAIIKHANPCGIAVGADIAEAHRKAHACDPVSAYGGVIAANREVSVEMAEQVAEIFTEVIVAPGYADGAVDVLRRKKNVRILIAEAPRRKGAELRPVSGGVLLQDRDILDAAGDAPANWQLVAGDAASAETLADLEFAWRACRAVKSNAILLAHDGASVGVGMGQVNRVDAVQLAVQRAGDRAKGSVAASDAFFPFSDGPQQLVAAGITAIVQPGGSIRDKDTIDLCREAGVTLYFTGARHFAH

>CORE_REP|Org15_Gene6278#

MSDPRASGVRGRESADSTEQLDTGDRVHVTKSTGARVETAVPSNGNESSPDTYWRRAGRFRHRISRRLSAVPLRVTLALALVSLTGLGLLISGVAVTSAMRNVLMDNVDRQLFGAAHDWAGPDAPPPQRLPGPVGRERPPGLFYVRIEDPSGKVRSLFPTGPSVPDFPADLGKHPRTIGSVGNPDEHWRAERVTTPGGSSWVAIRLSETENIIDRLIGLQVAVGLMVLAVLAIVAQFVIRRSLRPLGEVEKTAAAIASGDLHRRVPVQGTNTEVDRLSQSLNGMLSQIQSAFAATEASEESARRSEARMRRFVADASHELRTPLTTIKGFAELYRQGALADPDMFMDRIERESKRMSLLVEDLLMLARLDAQRPVERRPVDLLALASDAVHNARAVDAAQRPEEPRRPIDLEIRPGTGTLEVRGDEARLRQVLGNLVNNALLHTPPEAAVTVALTPAPDEVVIEVADTGPGLPTEDAERIFERFYRTDTSRSRDSGGTGLGLSIVQALVAAHGGTVSVRSAVGQGTTFAVRLPRSQE

>CORE_REP|Org142_Gene323#

MSAAGRSTNRYVLAIDQGTTSSRAIVYDAAGQLVSVAQREHRQLYPAAGHVEHDAAEVRRNVEGLIGRVITAAGIQARQVVGLGIANQRETTVLWDRHTGRPVRNAIVWQDTRTEDLVARLAQRPDADEVQVRCGLPVLNYFAAPRIRWLLDSDERLRARAERGEVLFGTMDSWLIWNLTGGANGGVHVTDVTNAGRTLLMNLHTLDWDERLLEFFEIPRAMLPEIRSNAEIYGYTAIEPAGIPIAAALGDQQAALFGQTCFAAGEAKCTYGTGSFLLSNTGTAPVRSGHGLLTTVAFRIGDEPAHYALEGSIASTGSLVQWLRDRLGLIESAPEIETLAATVTDNGGCYVVPAFSGLFAPRWHAEARGVIAGLTSYITRGHLARAVLEATAWQTREVVEAMNADTGQSLRELRVDGGMTSNNLLMQQISDALGVPVERPLFAETVSLGAAYAAGLAVGLWPDMEGLRRNRHTAARWLPALTAADRDREYRHWSRAAALSYHWTTETGSAPGDERLRPPYTVRTTASSDAPASDRP

>CORE_REP|Org2_Gene1014#

MTPAPLAVDVQEGSIRMSNPVSGERTAVRPSTGGDDPHKIAMLGLTFDDVLLLPAASDLIPSSVETSSQLTREIRLRTPLVSSAMDTVTEARMAISMARAGGMGVLHRNLAAADQAAQVETVKRSEAGMVTDPVTCRPSDTLAEVDAMCARFRISGLPVVDETGSLVGIITNRDMRFEVDQNRRVEEVMTKAPLITAQEGVTAEAALGLLRRHKIEKLPIVDGNGRLRGLITVKDFVKTDQYPNATKDRDGRLLVGAAVGVGEDAWSRAMTLADAGVDVLIVDTAHGHQAQVLQMVTKVKAEVGDRIQVVGGNVATRAGAAALVEAGADAVKVGVGPGSICTTRVVAGVGAPQITAILEAVAVCKPAGVPVIADGGIQFSGDIAKAIAAGASTVMLGSLLAGTAESPGELILVGGKQFKSYRGMGSLGAMQGRGQAKSFSKDRYFQDDVLAEDKLVPEGIEGRVPFRGPVNQVIHQLVGGLRAAMGYTGSQSIAHLQDAQFVQITAAGLKESHPHDITMTVEAPNYTGRG

>CORE_REP|Org45_Gene1567#

MSASDRTATPGASAPAFYITTAIAYPNGAPHIGHAYEYISSDALARFKRLDGFDVFFMTGTDEHGQKVQQAAKAAGVPEREYAAGNSDVFERMDKALDVSFDRFIRTTDEDHHAASIAIWERMAANGDIYLDTYSGWYSVRDEAFYTEEEITVLDDGTRVSTETRTPVEWTEESNYFFRLSSYQDRLLELYETRPDFIAPATRRNEIVSYVKAGLKDLSISRTTFDWGVPVPGDPEHVMYVWVDALTNYLTGAGFPNTDSAAFQKFWPADLHIIGKDITRFHCVYWPAFLMSAGIELPKRVFVHGFLYNKGEKMSKSVGNVVDPMELVDTYGLDAVRFFLLREISYGQDGSYSHEAIVGRINTDLANEYGNLAQRCLKMVARDFGPVAPTPGEFTEDDRALLDRANGLLEKVRAEFDQQQIHLGLEQLWLMLGETNRYFSAQAPWTLAKAGTPEGTAREGTILYVTMEVLRIVSILVQPVIPGSANKILDLLGQTGRTFADIATPIQPGIALPAPEVVFPKFVEPKA

>CORE_REP|Org16_Gene1684#

MRVAETQRPVLVVDFGAQYAQLIARRVRESSVYSEVVPHTATVEEIADRQPLAVILSGGPSSVYAEGAPQLDPRLFDLDIPVFGICYGFQAMAQALGGTVAHTGTREYGRTELNIDGGVLHGGLPTVQPVWMSHGDAVTDAPAGFEVTGTTAGAPVAAFENRARRLAGVQYHPEVLHSPHGQQVLSRFLHELAGIPASWTPANIADALVEQVREQIGDGHAICGLSGGVDSAVAAALVQRAIGDRLTCVFVDHGLLRAGEREQVQRDFVAATGAKLVTVDAVEKFLGELKGVTDPEEKRKIIGREFIRSFEDAVAEVVKSTGTEDGDGGPAVEYLVQGTLYPDVVESGGGSGTANIKSHHNVGGLPEDLEFELVEPLRLLFKDEVRAVGREVGLPEEIVARQPFPGPGLAIRIIGEVTPDRLETLRQADAIAREELTAAGLDAQIWQCPVVLLADVRSVGVQGDGRTYGHPIVLRPVSSEDAMTADWTRLPYEVLERISTRITNEVAEVNRVVLDVTSKPPGTIEWE

>CORE_REP|Org214_Gene7760#

MGCRTRPVQCDTVSRGRRGHRRRGAHPALAAADHATGRRDPGDPGADGRRGTHLRRRAHRARPGSAGRHRHRPARRRRAHAHRRRAGRPGRLPLRAARHRAPARPRNRPPRRAVHRDQPARRQGHRDRGRTRDRARHTARRTLALLRRSPDGNRRSHSRPARARHRDRGHRHHREDAAEFRRAVAADARLHVGRSGGGALRKREYDESDVRVRPGKSSRPRTKTRPQHNDAEPAMVVSVDRGRWGCVLGGDPGKQIVAMRARELGRTPIVVGDQVDVVGDLSGKPDTLARIVRVTDRRTVLRRTADDTDPFERIVVGNAEQLFIVVALADPPPRTGFVERCMVAAFAGGLRPVLCLTKHDLDAASEFAAAFEDLDLTIVYGGIEDPLEPVLELLHDRLTAFIGHSGVGKSTLVNRLVPDAYRAVGAVSGVGKGKHTSTQSVALPLPAGGWVIDTPGVRSFGLAHITPDDVVAAFSDLAAAIEDCPRGCTHLGPPADPECALDQLPGKERRVAAIRVLLNALNSNENY

>CORE_REP|Org85_Gene6549#

MTQHRDSDSTRGGGRATGGPVYARPGAPDALMSYQSRYDNWIGGQWVAPVKGQYFENPTPVTGENFCEVARSTAEDIELALDAAHAAAPAWGKTSAAERAAILNKIADRIEANLDAIALAEAWDNGKPIRETLAADIPLAVDHFRYFAGAIRAQEGSLSEIDAETVAYHFHEPLGVVGQIIPWNFPILMATWKLAPALAAGNAVVLKPAEQTPASIMFLWSIIGDLLPPGVVNIVNGFGVEAGKPLASSNRIAKIAFTGETTTGRLIMQYASQNLIPVTLELGGKSPNIFFSDVMSADDDFLDKALEGFTMFALNQGEVCTCPSRSLIQADIFDRFLELAALRTKAVRQGDPLDTETMIGAQASNDQLEKVLSYIEIGKGEGAQLVTGGERALLGGDLNGGYYVQPTMFTGRNAMRIFQEEIFGPVVSVTSFTDYDDAISIANDTLYGLGAGVWSRDGGTAYRAGRDIQAGRVWTNTYHQYPAHAAFGGYKQSGVGRENHKMMLDHYQQTKNLLVSYAPKAMGFF

>CORE_REP|Org151_Gene7020#

MVRKVAASVVDTQAKLDELVKILAIAAEPAGEAGIAKREKKSIPSVRQRVHMLLDPGTFIETSALARQPDQKDALYGDGLVTGRGLIGGRPVVVIAHDQTVYGGSVGITSARKFMRALQFAFDNACPVVTINDSGGARIQDAVGSIASFGDISRVLEKLSGYVPQVSIILGKCAAGSVYGPINTDVLIGTRDSYMFVTGPEVIKAVNGEDITAEALGGAKVQAERGTLHHVAETEEQAYEWARQYLSYMPTSCLEQPLIVNPGLEPEITATDRELDTIIPDSDRTGYDMHEILLRIFDDGEFHEIRAAFAPNLITGFARVDGVPVGVIANQPLVLGGSIDAACSDKSTYFIRLCDAFNIPLVFVVDTPGVLPGLEQEANGVIIRGGRVPRAIIEATVPIINLVVRKSYGGAYGMMAARQVGADISFAWPTARIAVIGAESAVDLIGKRQLAAVPEEQRAAAREFMVNHYNETIATPWIAAERGYIDAVIEPSRTRLEIRHALRLLREKPTVKPEFNPRKHAVYPM

>CORE_REP|Org5_Gene6966#

MSERSRATKKHPGRILFVGSGPGDPALLTVRAREVLRRAELAFTDPDVDKGVLAMIGTAVEPGPEGESTVDVRPALGEPAEVAKTLVHEARAGHDVVRLVSGDPMTTDAVIAEVNAVTRSHMAFEVLPGLPSATTVPAYAGIALGSSHTEVDVRGEVDWASVAAAPGPLVLHATSGHLAETASALVENGLAPQTPVAVTVRGTTRQQRTIEATLATLNSAASELVGPLVVTVGKEVEKRTKMSWWESRALYGWTVLVPRTKEQAGEMSEKLVMHGAIPMEVPTIAVEPPRSPAQMERAVKGLVDGRYQWVVFTSTNAVRAVWEKFGEFGLDARAFSGVKIACVGEATADKVRSFGINPELVPSGEQSSEGLLADFPPYDDVFDPVNRVLLPRADIATETLAEGLRDRGWEIDDVTAYRTVRASPPPAETREMIKTGGFDAVLFTSSSTVRNLVGIAGKPHARTIVACIGPKTAETAIEFGLRVDVQPETAQVGPLVDALAEHAAHLRAEGLLPPPRKKSRRSR

>CORE_REP|Org45_Gene2547#

MSARRIPGGAAPTRSRATWKTDPARTGGPAYLRRRRPGAGITEQSARAITLDEAVAAIRSGPQGPEIAAVFDFGGTVVHGFDPPSLTRRLLRRDRDPVAAGLLGSIRGARSEGEYERFLQHTMHAWAGLPEHRLEELGATLFQGTVYGHLYPEAWRLIREHETAGHTLVLVSALTRFQVRPAADELGIPTVLCTAMAAQDGVLTGHVEGKPLWRNGKADAVRRFALAEGIDLTRSWVYADSAADLPLLGVAGRPVAVNPDPRTVLEATEKEWPILHFRPRTTPRPTDYARTVAGFAALLGGALFGVAAKAHTKQRREMADSLLNHAAESTLRGTGVRVRVTGREYARAPRPAVFIFNHQSQFDMVVVAEVLGGGITGIAKKEITRNPIFGPLMRFVEVTFIDRADTAAAKAALAPVVQTLRGGLSIVIAPEGTRSRTPRVGAFKKGAFHIAIQAGVPIIPVVIRNAGEIAWRDSAVVRKGVVDVAVLPPIDVSGWDPAAMDADIERVRQLFVDTLLEWPTGD

>CORE_REP|Org80_Gene4558#

MWWTCSSDICGASSKWKGHRGCCTPFAVSVSCCGRRNERAVVSAADTPGSSDAAARPVGAGPPDRRAAAAGSRRRRSYSLRTRVAGAAAAGAILIITILSVITLQAIERVNVEQTDQQLTLASRLVLIDPVIAVGLVNLIGPNENLALTVRDDGELTATTAIELPDLPTGSHTVTVDGASYRVLTTTENQQAGRTVSLGIPNADAARATAQQQRWVLAGGLVAIAAAAGLGWLFGGRAVRPIVDLTRQVGARSGYRDPEHPPQPVDGSGVLEAEQLADAVNTMLSRVDQAQGETAAALETARDFAAVSAHELRTPLTAMRTDLEVLRTLDLDETQRAEILDDLHRSQGRVEATLSALERLASGDLTHERDHVDTDVGDLCDQAAHDAMRHFPGLTVRIDTDAELVTRGLPAGLRLAVDNALANSVKHGGATEALVSAHRAPDGHIIVSIDDNGRGIPVQERQAVFDRFYRGTQATKGGSGLGLALVAQQAQLHGGQAYFDDGTLGGVRLVLDLPARPARTG

>CORE_REP|Org103_Gene853#

MRQKGDVPDDARPGTLLRTRGGGLLSAAAEQRKEGGAEVRDRDVLDRVPTGLFIGGGWREADDGARFLVEDPATGARLADVADGGPAEMAAALDAAAEAQRSWAATPRRQRSDLLRAAYEEVLRRLDEFALLITLEMGKPLAESRGEVRYGAEFLRWFAEEAVRVGGRWGRSPEGATRMLTMKEPVGPTLMITPWNFPLAMATRKIAPAVAAGCTMVLKPAEQTPLTALAFAELLRTAGLPDGVLNVVTTTRAPETIAPLLRDQRLRKLTFTGSTAVGKRLLAQAADQVLRVSMELGGNAPFLVFEDADLDRAIEGAMVAKMRNVGQACTAANRLLVHEAVAEKFAAGFAAAMAAQKIGRGTEPGVQVGPLIDARARDKVARLVADAAGDGARVLTGGSAVDGPGYFYAPTVLTDIGDHPVAREELFGPVAPIATFGSVEEAVAMANATEYGLASYVFSENLSRVLEVVENLQFGMVGVNQGVVSNAAAPFGGIKHSGFGREGGIEGIEEYLATKYVGIAS

>CORE_REP|Org29_Gene4463#

MARTTSKRQAKSGANETVAPLGSSRRGADEPAPMRPPTPLTRTVSLRWRVTLLAASVVAIAVAVTSIAAYAMVARALYGDVDAQLRARAATMINGDIDSMAFQSLGVATLFSNNIGVGLIYPFSVSSPPSTPEGERTLDSLPVYIPPQPTKPPIGTEEIAVAKGEHTSSLRTYNNQRVLARRMDSGVTLVISQRLEPTREVLDRLAWLLFVVGGCGVLLAAAAGTAVGRTGLRPIARLTAATERVARTDDLTPIPVTGDDELARLTESFNTMLRALAESRDRQRRLVADAGHELRTPLTSLRTNMELLIAAGRPGAPRIPDEDMAELRMDVVAQIEELSTLVGDLVDLAREDAPETVYERVDLGEVAERALERARRRRGSIEFVAALRPWFVYGHEAGLERAILNVLDNAAKWSPAGAQVRVSMAEVGRGLLELSVDDAGPGIPPAERELVFERFYRTTASRSMPGSGLGLAIVKQVVTKHGGTITIDTSERGGALIRIVLPGEAGAPVATAEDEPDP

>CORE_REP|Org19_Gene2296#

MGTETVENRRHRVVVIGSGFGGLFACKHLEHDNVDVVLISKTSTHLFQPLLYQVATGILSTGEIAPATRIVLRKHHNTQVILGEVHDIDLVNKTVTSKLLNQDTVTSFDSLIVATGAQQSYFGNDRFATYAPGMKTIDDALELRARILGSFEEAELAKTQEERDRFLTFVVVGAGPTGVELAGQIAELADRTLVGTFRNIDPRDARVLLVEGAGAVLAPMGPKLGGKAQRRLEKMGVEIQLNAMVTDVDARGVTVKDKDGTERRIESACKVWSAGVQASELGKMLAERSKGTETDRAGRVVVEPDLTIKGYPNVFVVGDLMAVPGVPGQAQGAIQGATYAAKQIKAEVAGKQTPDQRKPFKYFNKGSMATVSRFNAVCQIGKLEFSGFLAWLIWLVLHLYYLIGYRSRTVTVFQWFVAFLGRNRGQMAATEQWVFARLALEAMNGNETDARDVQAEVGNTTPPAAPGEPSAKSAAATPDGEKASGTSESAGSGESTATSKSGASSESTTSGSSQPKAG

>CORE_REP|Org5_Gene6285#

MPQNRSASSPRPRGAGAPLAGGRVPRARGVRPVAGPPVNRPGRILAVAAAAVVFIVTGFGWHSVDSLVSGIERIGNLGLGGGHDGAVDILMVGIDSRTDAHGNPLSDQERAMLHAGDEVGTNTDTIVLIRVPNDGSSATAISVPRDSYVDIPGLGKGKINSAYGATKEAARQKLADQGLSDSQIEEKSTQAGRQALIKSVANLTGITVDHYAEVGLLGFVLLTDAVGGVQVCLNNPVDEPLSGADFPAGEQRLSGPQALSFVRQRHDLPRGDIDRIVRQQVFMASLVNQSLNAKILANPGKLRELSDAVGRTIVLDKGWDVVSFMHQLQDLSGGKVNFETIPVQNLDATTSDGESVVKVDPKAVKSFVAAAVGGKSDEHRDSDAVAPDTVTTDVYNSGSTSGLATQVAQALTGKGFHTGSVANWTGEPVRSSRVLAASTSDAKAKAVAEALGGLTVIADPELSQGAIRVVLADDYSGPGSDAGSLFDLSGTSQTSGAPTPVPPAPPIDAGQNGPKCVN

>CORE_REP|Org190_Gene4366#

MTCRVVLLRHVVDRQEVSVSALDVSRWQFGITTVYHFLFVPLTIGLAPLIAGMQTAWVITGKEHWYRLTKFFGKLFLINFALGVATGIVQEFQFGMNWSEYSRFVGDVFGAPLALEGLVAFFMESTFIGLWIFGWSRLPKLVHLATIWMVAIGVNASAYFIIAANSFMQHPVGARYNPETGRAELTSIVELLTNNTALAAFPHVVAGSFLTAATFVAGIAGWWMVRNARSGDEQKLTEARTMWRPAARASLVVIALSGVALIYTGDVQGKLMFEQQPMKMASAESLCHTATNPDFSVLTVGTHNNCDSVTHVIEVPYVLPWLAEGKFTGVTLDGVVDLQQAYNEKYGVGDYRPNLFVTYWSFRAMIGLAGGSALLAIAGLWVMRRGRVPDQRWFSWLSLLAIPTPFLANSAGWVFTEMGRQPWVVVPNPTGDPNLRLLVQDGVSNHSATTVWVSLITFTIVYGLLAVVWFYLMRRYVIEGPDKAAAPAPAKGPDDTGAPGAKPRTEEPAVEQLSFAY

>CORE_REP|Org4_Gene3225#

MTAYRTRGVMVGYQDRTGMVRELTHFIGGQHVAGTSGRFGDVYDPNLGQVQARVPLASKDEVAAVVANAEAAQRVWAAFNPQKRARVLMKFLTLVQDDMDNLAALLSAEHGKTIADAKGDIQRGLEVIEFATGIPHLLKGEYTESAGTGIDVYSMRQPLGVVAGITPFNFPAMIPLWKAGPALATGNAFVLKPSERDPSVPLRLAELFLEAGLPAGVFNVVNGDKEAVDALLHDPRIKAVGFVGSTPIAQYIYETATANGKRAQCFGGAKNHAIVMPDADLDDVADQLIGAGYGSAGERCMAISVAVPVGQETADRLLAKLTERVHKLNIGRSDDPGADYGPLVGKDGVDRVHNYVQIGIDEGAELVVDGRGVTVPGAEDGYFVGATLFDNVTPEMRIYKEEIFGPVLSVVRAKDYEEGLRLANEHEYGNGVAIFTRDGDTARDFAARVQVGMVGINVPIPVPIAYYTFGGWKRSGFGDLNQHGPDSIRFYTKTKTVTQRWPSGLKESNAFVIPTMD

>CORE_REP|Org12_Gene746#

MTGGNETAEVGTAADPVNHTGQAPAELPELLRRVHMVGIGGAGMSGIARILLARGGEVSGSDAKESRGVLALRARGAQVRIGHDASALDLLEGGPSAVVTTYAAIPKTNPELVEANRRGVPVLMRPTVLAELMRGHHTLLVSGTHGKTSTTSMLVVALQHCGFDPSFAVGGELNEAGTNAHHGTGGYFVAEADESDGSLLQYDPDVAVVTNIESDHLDFFGSDEAYVQVFDDFVARLVPGGLLVVCLDDPGSLALARRVADRVAAGELDIRVAGYGSAEAVGVPVPMQARLVAWEPRDVGGVATVQLGDEPAPRTLRLSVPGRHMALNALAALLAARDAGADVDEILQGLEGFGGVHRRFQFVGRENGVRVFDDYAHHPTEVRAVLGAAAELVRQEAADGARSRQGRVIVVFQPHLYSRTATFAEEFGAALSLADEVVVLDVYGAREKPLPGVNGALVAQAVTKPVHYQPDMSRVGRQAASLARAGDVVITMGAGDVTMLGGQILDGLRVRPSTGR

>CORE_REP|Org10_Gene3434#

MAIARAGLPRLMAMSAPTVELMDYADVVARYEPVLGMEVHVELSTATKMFCGCPTDFGAEPNTQVCPVCLGLPGSLPVVNEKAVESAIRIGLALNCSITPWGRFARKNYFYPDQPKNYQISQYDEPIATDGHLDVVLDDGSVFRVDIERAHMEEDTGKSVHVGGATGRIHGASHSLLDYNRAGVPLIEIVTKPITGAGERAPEVARAYVTALRDLLKSLGVSDVKMEQGSLRCDANVSLMPVGASEFGTRTETKNVNSLKSVEVAVRYEMRRQAAVLAAGGAIVQETRHFHESDGTTSPGRRKETAEDYRYFPEPDLEPIAPSPEWIEELRATIPEYPWLRRARIQQEWGVSDEVMRDVVNAGALDLIIATVEAGAPANEARSWWVAYLSEKAKERGVALDELPITPAQVAEVVKLVESKTVNSKVAKQVVDIVLAGEGEPAAVVEAKGLGMVSDDSALQAEVEKALAANPDIADKIRAGKVQAAGKIVGDVMKATRGQADAARVRELVLAACS

>CORE_REP|Org102_Gene2469#

MVAENTDSMSRDVFATGHPALYRRCMSREPSILIIGAGFAGLGMALELRRHGIGNFTLLEKAAELGGVWRENTYPNAACDVPSPLYSWSFEPKSDWPRRFSHQRDIHEYMRAVADKYRIPEHIEFGVEVTDAEFDERAGVWRVTTADGATRTADILIPAVGQLSRPAMPNLPGIESFTGAAFHSAQWDHSVDLTGKRVACIGTGASAIQYIPAIQPNVEHLTLFQRSAAWILPKFDTEYSALHHALFKYVPPVRLAERFAIWSFFEVLALALTDIPAIKSPVIAIADRHREKQVPDPELRAKLTPDYAAGCKRGLFSNEYFPALAQPNVTVETTAIEAVTPTGIRTADGVEHAVDVIVYGTGFKGTEFLAPMNIYGLGGRKLSDEWAAEGARAYLGMSVPHFPNMFMMYGPNTNVGSGSIIYMLEAQARYIRQAIGYLARRPGSFVSARPNVEQSWDDWLQHRLKDTPWNFCSSWYRNASGRITNNWPGATVLFRWKTRTFEPGDYEVAARA

>CORE_REP|Org184_Gene4905#

MSATAPAWVRFTFVVSTGPDPAQLPAQSDPAQLAPDERRTRLLAAAAVTLGSLGDVLTPLGELFAARGFQLYLVGGSVRDAILGRLGTDLDFTTDARPEQVQQMMRGWADHLWDTGGLAFGTVSAAKDDQQLEITTFRSDSYDRVSRNPEVTFGDTLEGDLVRRDFTVNAMAVKIGADGALEFVDPLGGMDALLAGVLDTPSAPQESFGDDPLRMLRAARFVSQLGFTLHPRVQTAITEMAGEIERITAERVRTELDKLIAGAHPIDGINIMCETGLAQIVLPEVPAMKLEIDEHHQHKDVYWHSLTVLEQAIDQEEGDPDLVLRWAALLHDIGKPDTKRNEPGGGVSFHHHEAVGAKMVRKRMRALKYPKQFTEDVARLVFLHLRFHGYGKGQWTDSAVRRYVTDAGDLLPRLHKLVRADCTTRNKRRAAALRATYDDLEHRIARLQEQEDLDRVRPDLDGNAIMELLDLKPGPDVGKAWKYLKELRLDRGPLTRDEAEAALLEWWKTQQ

>CORE_REP|Org142_Gene5069#

MLDEGRTQFYGGHVPAPLTTRQQVNGYRFLLRRLDHALVRRDVRMLHDPMRSQLRSLLVGAVLGLLVVAGAAILAFIRPQGAIGDAKIVMGKDSGALYVVVADNDGGNTLHPVLNLASARLISGSSESPASVKDDKLADMPRGPLLGIPGAPSALPGSAQGTSSEWSLCDTVELSITGSAASASGVDTAVLAARPDLSERIRRADPDEAVLVRRSDRTYLIYEGKRAQVDPENSAIARALSLSGERPRPAGAGLLGAATPVPPIAVPEIPNAGKPGPGALSDIPVGGVISVAATGRGERAELYVVLADGVQHISDFTADVIRTANSQGMSQIETVPPDALTGIAVLSQLPVDHFPAAAPTILSAEDAPVTCVSWSKTEQSDADAVDGPTDRASAALLVGARLPLPEGAQPVSLATADGSGDRVDQAYLRPSSGEFVHVTGMEPGSPRRGSLFYIADNGIRYGVPDIDTAMVLGLGDAPALAPWAIVGQLVPGPTLASTDALTRHDVLPQSN

>CORE_REP|Org154_Gene2389#

MAESAQPRLAAHPGGTGRTSVLTSYDPRTGEAVGEYPVQRTAEVARAVRAARAAEKWWGGLGFGGRKRWLLDWKRAIVRRSGELVELICTETGKPEADAAIEVVLAIENLDWAARNAARALGRRSLGRNWLTRNHKATVGYLPLGVVGVLGPWNNPVYTPMGSIAYAMAAGNAVVFKPHELTTGVGVWLAESWRALAPDQPVLQAVTGDDATGLALCRAEVDKVAYAGTEAGAREVIAGCAETMTPVVVERDDKGAMIVHVDAKLDDAAEAAVYGAMANAGQNPSGVQCAYVADSVYDSFLHLVIAQARRLRPGADRRASYGPMIMEAQADVVRRQVRDALARGGRAVVGGLESIREPYIEPIVLAEVPEESLAVTGEAIGPVLVVNRVASMEEAAERVNATGNAVAVSVFTRDVHSIEAFAERLRVGVVTINSATAYTGIPALPYGGVGEYGHGHSHGDEGLREFSRTLSIARKRYRGTVNLTTFDRHPRHLRAATAIFQLRHARRP

>CORE_REP|Org39_Gene5783#

MIDKRQQQRDDPGKTIPAVTRVLIIGAGLSGIGTAIRLRCAGIEDIVVLERATGPGGAWRDNVYPGAHCDIPSVLGSFSLARNPRGSHEYSSGADILAYIHDVIARHGLERRIWYGRTVIGLDFDEAAGTWRVRTSTSGGEEVITARSVVMAVGPLSNTSRPDIVGIDGYRGHKVYSARWDPSLDVTGLTVAVVGTGATAVQVIPELVNRARHVTVFQHTPRWVLPHPQYRVPAWNRSLFEKLPLTKDLTRTAYFWAHEAMRSGVVWPTGLTTALEQVAKLQLRRQIKDKWTRRQLTPNYRANCQQLLVSNAYLPALDRDHCKLLTFPIVRLTERGILTVDGVERCFDTIVFATGFDVPCKIGTPFPIRGRDAHLLREEWAEGACAYKSVHVSGYPNLHFTFGPNSCSGRNSALFFLEAQIDYIVESVRMLERWGLRYLDARKSAQDRFNAGIRQRFSGTTRNSRCASWHPTEDGFNPTIFPGSARQFRAQMDEFTLSDYHAVSLSE

>CORE_REP|Org104_Gene5315#

MTPITVSRRTTADGNGRVVLPIVTIGRVQFLPGHQPPYDLTYDDLFLVPNRTDVASRFDVDLSSVDGSGTTIPIVVANMTAVAGRRMAETVARRGGIVVLPQDLPLDAAADTIGYVKSRSLTADTPVSMEPEHSVAEALALMHKRAHGAVVVVEDGKPVGVVTEASCTDVDRFARLREVARTDFVSAPASTSPRALFDLLEAEHAQLAVLTTEDGALAGVMTRTGAVRAGIYQPNVDAEGKLRIAAAVGINGDVAAKAKSLVDSGADLLVIDTAHGHQEKMLEALRAVAGLGLGVPLAAGNVVSAQGTRDLAEAGADIVKVGVGPGAMCTTRMMTGVGRPQFSAVAECAAAAKEVGVHVWADGGVRHPRDVALALAAGASNVMIGSWFAGTYESPGDLRVDRDGNAYKESFGMASKRAVAARTATDSGFDRARKALFEEGISSSRMRLDPERPGVEDLIDHICSGVRSACTYAGARTLPEFHHRAVLGVQSAAGFAEGRPLPSGW

>CORE_REP|Org5_Gene2466#

MSTTDHNPSGATQHMPTTVTSPQVAVNDIGSAEDFLAAIDKTIKYFNDGDIVEGTIVKVDRDEVLLDIGYKTEGVIPSRELSIKHDVDPNEVVSVGDEVEALVLTKEDKEGRLILSKKRAQYERAWGTIEELKEKDEAVKGTVIEVVKGGLILDIGLRGFLPASLVEMRRVRDLQPYVGKEIEAKIIELDKNRNNVVLSRRAWLEQTQSEVRSEFLHQLQKGQVRKGVVSSIVNFGAFVDLGGVDGLVHVSELSWKHIDHPSEVVEVGMEVTVEVLDVDLDRERVSLSLKATQEDPWRQFARTHAIGQIVPGKVTKLVPFGAFVRVEEGIEGLVHISELAERHVEVPDQVVAVGDDAMVKVIDIDLERRRISLSLKQANEDYHAEFDPSKYGMADSYDEQGNYIFPEGFDPETNEWLEGFDKQREEWEGRYAEAERRHKMHTAQMEKMAADAAAEAANGGGAGNYSSESGAQASSSSSSSSESAGGSLASDAQLAALREKLSGNA

>CORE_REP|Org7_Gene2823#

MTAVADRSLGPELARTQTISTDTDVLVLGLTSSENGPAIVPEDLFGDVLTAEVRAELLDQLGAVGAKGKTEELTRVPAPAGLDGVTSVLAVGLGAAEKIDAEQIRRSAGVAARALSGTELVVTTLSGLDIGAAAEGFYLGAYTFTPFKSDKSAPKPDERPVARVELLVPEPEFGEQELFRAQLIAEAVATARDFVNTPPSHLYPAEFASRAQELAEAAGLQVEVLDEKALEAGGYGGVLGVGKGSSRPPRLVRITYAGGPKKVALVGKGITFDTGGISIKPAQNMDNMTSDMAGAAAVIATTLLAARLSLPVTVTATVPMAENMPSATAQRPGDVLTQYGGITVEVLNTDAEGRLILADAIVRASEDDPDYLIDVATLTGAQMVALGTRTPGVMGTDEFRDRVAAVSRAVGENGWAMPLPAELRADINSKIADLANVAPHRWGGMLSAGLFLKEFVPEGVQWAHLDVAGPAYNTGGPFGYIGKGGTGVPVRTLITVLEEIGAE

>CORE_REP|Org5_Gene928#

MVRNGRAAGWLRVAVAGAVLGAASGAGPAVMVGAGPASAVAPPAIDDGALGQAQAVNAKNGPPDETEKRAICAEPYLTGAVPRDPPLPQRILDLDRAWKFSRGAGQKVAVIDTGVNRHPRLPDLQPGGDFVTAGDGTEDCDGHGTLVAGLIAARPSPEDAFSGVAPEAQILAIRQLSLQYEAKNHRDDDTGKVAAGGYGDVLTMAAAVVRAVDMGATVINISEVSCSPAGSGTADGPLGAAVKYAADRNVVVVAAAGNLDQSACSVQNQTSGWNGVSTVISPAWFSPYVLSVASTDPDGATSPFSIHGPWVGVAAPGRTIISLDSKPGGTGLVDTEHGDEGPLTIDGTSFSAAFVSGLAALVRSRFPDLSAAQVIDRIERTAHNPGAGRDDRVGFGLIDPLAALTAQLPPPADRTGALPRAIAPPAPDPGPDPVPRRVAVIGSIALLALLVIGWAAALPYRRGRPGRGTGDPADGFVGTAETASPERISASSGPAGTDSPGGE

>CORE_REP|Org5_Gene949#

MPSKPTTRWQVSGYRFLVRRMEHALVRRDVRMLHDPMRSQSRAYAVGLVLGIVALAGCGVLALLKPQGSIGDNKILLGKDSGAVYAVIDGVVHPALNLSSARLAVGEPAKAVSIKESELAKKPRGALIGIPGAPSSLNFDGSGKGRAWSICDGLKNDGSQDLSTTVIAGDPSLGSKASRLGEGAALLVQGRDAAYLVYDNQRARVDMNDPKVTEALGIRGKTPRPISPGLLNAIPEVLPIEPPKIVDPGGMPTYSLNNHRIGDVVHVATKDQYYVVLRTGLQSISPLTADIIRNSNTAVSTDPEIDQSQAVQQNVSNELPVQKYPVKAPTIVEAKDQPVACMSWKPVAGASDKTDGSKRATLAVITGYSLPIPDNAQTTPLAQADGSGQNVDAFYSTPGSGFFVQTTGIETDSQRRDSMFFIADTGVRYGIKDANAQKALGMDAEKAKPELAPDQIVGLLAAGPTLGRQEAMVAHDGVAPDPAPAKQLVQSKQDQQAQQQSPN

>CORE_REP|Org5_Gene3985#

MTFELLRAAGVRAPVRAAVREMRNLPRSCRYGGGVHFTNGGSVIEQYPLSGWSRTAPTVARVLRSRDLDVVARAVREAGPRGVIARGLGRSYGDPAQNGGGLVVDMTVFDRVHTIDPDSGVVDVDAGVSLDALMRAALPHGLWVPVLPGTRQVTVGGAIASDIHGKNHHSQGSFGNHVLSMDLLAADGTVRTIGPDGADADLFWATVGGMGLTGIVVRARIRMKHTETAYFIVDCDRTTDLDETMRLLTDGSDEGYEYSVAVPDTISTGAKLGRAGFSRGNLATVDQLPPRLRRDPLHFAAPQLLTVPDIFPSGMVNNLTTRIAGELTYRVFGKQGRGMIQNITQFLHPLDVLGEWNRAYGRRGFLQYQFSMPYGAEDQLADAVRTIARSGHRSFLNVFKRMGPSSRAPLSWPHPGYMLSLDFTLAPGVNEFCADLDRRVLAAGGRLYFAKESRTTPEMIRAMYPRLEEWRTIRDAVDPERTFVSDMARRLRLVDDEPVLAR

>CORE_REP|Org162_Gene2884#

MPADISAPPSRGPAPTGGKTPTVIRLLVLATFVVILNETIMINAIPRLMHDLDVTERAAQWVSTAFMLTMAAVIPVTGWFLQRVSTRQAYAIAMGVFLAGTALSAVAPTFAVLLVGRIIQAGGTAVMMPLLMTTLMTVVPEQDRGRVMGNVTLAISVAPAMGPVISGLVLQAGSWRWLFVLVLPIAGTVTWLGLRRLDNIGEPQTGDIDWLSVAFAAFGFGGLVYGLSKFETDHVAVPALLVAAGLALIAVFAFRQLRLQRSGVPLLDLRILLSGTYTKALVLMSVAFLAMLGSMILLPLYLQNLRHLSPLETGLLVMPGGLAMGLLGPTVGRLFDRFGGRPLVIPGAVGVTVALAGFTQISMSMPYWQLLALHILLMISLAGLFTPVFTLGLGALPPHLYSHGSSMLGTLQQVAAAFGTALVVTVMSARMTQLMETGTEPVTAQLDGMRLAFAVSAALSVLVIVTAILLPSRAPAPEETGEDDASEAETAESAAPLLVKD

>CORE_REP|Org5_Gene2339#

MVEHTPRALRSPGPMLEFLRGRDVLVAGWGVSGRSLIEPLRDIGARPVVTDAGEKAMAEAAELGLDTATGAELLEPDALNRFALVITSPGWRPDSPVLVSAVTEGIPVWGDVEFAWWVDQARLYGPVRKWLVITGTNGKTTTTQMTHAILRAAGLASVACGNIGLPILDALRRTPGPQILAVELSSFQLHWAPSVRPEAGVVLNVAEDHLDWHGGLDAYAAAKARALTGRVGVVGLDDAVAAALARKSKARRTVGFRVGVPADGELGVVDGKLLDRAFTKAAILAEVGDISPPGPAGVADALAAAALTRAIDVAPQFVKEGLAEHKVGPHRAAFVRELSGVGFVDDSKATNPHAARSSILAHPQVIWIAGGLLKGAHIDDLVEEVADRLVAAVVFGKDAAVIAAAMARHAPDVPVVELGSGDDDRMSGELSTASLVAEIDGADAVMARAVRIAAGYAGRGDTVLLAPAAASLDMFADYTHRGRSFVAAVQALDERDIGSQQ

>CORE_REP|Org43_Gene4984#

MADVAVVGSGPNGLAAAVVLASAGLSVEVFEAAATAGGGCSTAELTLPGFHHDVCAGAHPMASASPFFRAFDLAAHGVELLAPPASYAHPLDGGRAGVAWRDLDRTVADLGPDGPAWRSFFEPLVRDWPGVVGVAMSDLRHLPPDLPTAVRFGLRLLEQGSPLWNLRFRGDVAPALLTGVATHAITSPRALPAVGAGVLLGTLAHAAGWVIPRGGSQAIADALIAELERLGGSVHTGHRVDSLDEFGGARAIVLDTSPAELLRLAQDRLPAGYARRLRRFRYGGAACKVDFALSGPVPWAAPDCAQAGTLHLVGSRAEAMAAEGAVASGRHAERPYVLAIQPGVVDDSRAPAGKYTFYTYAHVPHGSDLDVTDAVIAQVERFAPGFRDLILAHNTRTAAELPTHNANYVGGDISAGAMILPQVLFRPAPRWNPYATPLPGVYLCSSATPPGPGVHGMNGLNAARHVLRREFDITTDPLELLGTAVRTGARRLRPLTRSGGV

>CORE_REP|Org36_Gene4556#

MQRIIGIEVEYGISTPTEPTANPILTSTQAVLAYAAAEGVPRAKRTRWDYEVESPLRDARGFDLSRMNGPAPVIDADEVGAANMILTNGARLYVDHAHPEYSAPEVTDPLDAVIWDKAGERVMEAAARHASSVPGAPRLQLYKNNVDGKGASYGTHENYLMNRDTPFNSIIVGLTPFFVSRQVICGSGRVGIGQSGDHAGFQLSQRSDYIEVEVGLETTLKRGIINTRDEPHADADKYRRLHVIIGDANLAEMSTYLKVGTTALVLDLIESGEDLSDLQLARPVTAVHTISHDPTLRATVALADGRELTGLALQRLYHERVAKFVHREGNDDPRVADILDNWAMVLDLLERDPMECANLLDWPAKLRLLEGMRSREGLNWGAPKLHLMDLQYSDVRLDKGLYNRLVARGSMKRLVSEQQVLDAMTNPPTDTRAYFRGECLRRFGADIAAASWDSVIFDLGGDSLVRIPTLEPRRGTKAHVGKLLDGVDTAAELVEQLTT

>CORE_REP|Org3_Gene1380#

MTGPDETDGPDFAREAGNAEPEPQHGTGAPLGSGPSPVDLAEMALVEAELDRRWPETKIEPSLTRIATLMDLLGSPQQSYPAIHIAGTNGKTSVTRMIDALLTALHRRTGRITSPHLQLATERISIDNAPITPARYVEVYRELAPYIEMIDQQSAAAGGPAMSKFEVLTGMAYAAFAEAPVDVAVVETGMGGTWDATNVIDGQVAVITPIGLDHTEYLGPDLTAIAREKAGIIKRAPESLIPRDNVAVIAEQDPEAMDVLLRRAVEVDAAVAREGAEFRVLARKIAVGGQQLELQGLGGVYDEIFLPLHGEHQARNAVLALAAVEAFFGAGAQRQLDVDAVRAGFASVTSPGRLERMRSAPTIFIDAAHNPAGAKALAATLTSEFDFRKLVGVVAVLGDKDAAGILEALEPVFDEIVVTTNGSPRALDVDSLTDLAVQRFGDERVVPAYTLPDALETAIAIAEDVADTGEMVSGAGVIVTGSVVTAGAARALFGKEPA

>CORE_REP|Org10_Gene811#

MTVTEHRSPELASRQPADPSALLSPRRAKIVCTLGPAVATDDKVRALVETGMDIARLNFSHGDHPDHQANYDRVRSAAAATGRSVGILADLQGPKIRLGRFADGATVWATGDTVRITVEDCPGDHDRVSTTYIHLADDARPGDRLLVDDGKLALTVTAVDGPDVVCTVVEGGPVSNNKGLSLPGMDISVPALSDKDIDDLEFALRLGADLVALSFVRSAADIDRVHEVMDRVGRRVPVIAKIEKPEAVDNLEEIVLAFDALMVARGDLGVEVPLEQVPLVQKRAVQLARENARPVIVATQMLDSMIDNSRPTRAEASDVANAVLDGADAVMLSGETSVGKYPLETVATMARILAAVEQNSTAVPALTHVPRTKGGVLSFAARDIGERLNAKALVAFTQSGDTVRRLARLHTPLPLLAFTPVPEVRHQLALTWGTEAFLVDRVHTTDDMIRQVDTALLHLDRYQHGDLVVIVAGSPPNTVGSTNLIHVHRIGTDDYTSY

>CORE_REP|Org119_Gene5784#

MSAPAPPSAGAFPSPPGGFAPAPPPSTRRNVELLLLAGAAVITTAALFLVEASQEQSLTWDIAKYGAAYLGLFGVAHLAVRRFAPFADPLLLPIVALLNGLGLVLIHRLDLADQQTAVYNSWSMPSPDANKQILWTGLGMVVFVVLLIALRDYRTLARYSYTLGLVGLVALAMPALLPSRFSEINGSKNWIKVPGFNIQPAEFAKILLIIFFASVLVAKRDLFTAAGRHLLGMEFPRGRDLGPIVVVWIVCVGVLVFEKDLGTSLLIFGTVLVMLYIATERVGWLIIGGALLGLGFVFAYQTFGHVQVRTQTWLHPFDDYNNTGYQISQSLFGLATGGLAGTGLGSGRPNQVPFAKTDFIITTIGEELGLIGLTAVLVLFLVFIVRGLRTALAVRDSFGKLLAAGLAFTIAIQLFVVVGGVTKLIPLTGLTTPFMSYGGSSLLANYALLALLIKVSDAARAPAPARKSVPAAPIADATTELLRKPEGGRPEAGPAT

>CORE_REP|Org210_Gene726#

MPQQTAVVVLAAGAGTRMRSKTPKVLHSLAGRSMLEHALHAANEIDPTALITVIGHDREQVGAAVNSVAAELGREITSAVQEQQLGTGHAVQCALTALPADFAGDLLVTSADVPLLDGHTLSALLDEHRSYQPRSAVTVLTFVPEDPNGYGRIVRDADGGVLEIVEHADATPEQAAINEVNSGVYVFDVAVLRTMISRLTTANAQHELYLTDVLKLAREAGNPVHGARLVDAAKVTGVNDRVQMAQAARTLNRYILERHMRAGVTVIDPATTWVDASVRIGRDAVLRPGVQLLGNTVIGEDAEVGPDSTLTDVLVGEGAKVVRTHGEGATIAAAATIGPFAYLRPGTIVGESGKIGAFVETKNASIGAHSKVPHLTYVGDATIGEYSNIGASSVFVNYDGVKKHHTVVGSHVRTGSDTMFVAPVTVGDGAYSAAGTVLRRNVPPGALAVSGGAQKNIEGWVQRYRPGTAAAQAAAEAIAADDRASQATEQKDGNTE

>CORE_REP|Org52_Gene4515#

MTATAAATGRQPGTPVFTHHTRGRWLEHWEPDNPEFWEAGGKRTARKNLAFSVFAENLGFSVWVIWGTVVTSMGAAGFPFLAGLGQGNPVAVSNALLLTSTPTLVGAALRIPYTFAIPRFGGRAFTAFSAAMLLVPTLGLAWFVNQPGTPMWVFMVLAALAGFGGGNFSSSMANISFFFPEGKKGAALGINAAGGNLGVAQTQLVLPLLITLGTHLTAKDPAGYRFGITLSVLVWVPFILIATVGALRYMDSIATAKSDGKSYKLALTNRHTWVMSFLYIGTFGSFIGFSFAFPTLIKANFPSLAGIGWITTLGNLAFLGALVGSFSRPFGGWISDKVGGARITVFVFGGMAVAVAAIMAALELKSFPLYLIAFLVLFVLTGIGNGSTYRMIPSIFSAESKKYAAEHDIDPADAAASAKRQAGAAIGVIGAIGASGGYLLQQALRLSNINFGSMAPAFWAYAAAFLVMAGVTWFYYLRSSFAIGRFTSLAYANV

>CORE_REP|Org106_Gene2979#

MLENPAATTQFPVTQRAFGLAILVLSGLQLMVVLDGTVVIFALPRLQDQMGLSSAGSAWIVTAYGLTFAGLMLLGGRLGDAFGRKRMLIAGVGLFTVASLLCGLAHWQAMLIAARALQGAGAAIAAPVAFALVATTFAPGKARNQAIAIVGSMVGIGSVGGLVVGGALTQLSWRWIFLINVPIGALIILGAIYCLADTGHHRVALDARGAVLGTLACAAIVFGATEGPELGWSHPAVIGALIGGAILLVVFVIAERNVDDPLLPWSLFDSRDRVTTFVLILLAGGVLGAMTYFVAQFLQNVLGYGPLQAGVASIPFTVGIGIGGALASKLAMTVAPRWLLFGAALVLAVGLLFGSTLDGEVSYLPTLLPLLIVIGFGVGVAMVVTPLCVLVGVPPSDIGPLSAVGQMFMNLGTPMAIGILTPVAVSRTLSLGGTTGKVSAMTDAQIVALGEGYTLVLAVCAGVAAVIGLIALTLRFTPEQIARAQHAQEEAQRS

>CORE_REP|Org113_Gene4603#

MTTSELSASPTLTPDVRNGIDYKVADLSLAEFGRKEIRLAEHEMPGLMALRREYAEVQPLKGARISGSLHMTVQTAVLIETLVELGAQVRWASCNIFSTQDHAAAAVVVGPHGTVDEPKGTPVFAWKGESLEEYWWAAEQMLTWDGEPANMILDDGGDATMLVLRGAQFEKAGVVPPEDETHSTEYKVFLNLLRASLEANPGKWTAIAESVKGVTEETTTGVLRLYQFAAAGELAFPAINVNDSVTKSKFDNKYGTRHSLIDGINRGTDVLIGGKKVLICGYGDVGKGCAESLAGQGARVQVTEIDPINALQALMDGFDVVTVDQAIGNADIVITSTGNKDIIGLDDMKAMKDQAILGNIGHFDNEIDMAALESSGATRLNIKPQVDLWTFGESGKSIIVLSEGRLLNLGNATGHPSFVMSNSFSNQVIAQIELWTKPEEYDNEVYRLPKALDEKVARIHVEALGGTLTKLTKDQAEYIGVDVEGPYKPEHYRY

>CORE_REP|Org114_Gene3042#

MNTPLRRVAMAVMIMVVALLANATYVQVIKADSLRSDPRNVRVLMDEYSRQRGQISAQGTVLASSVATDDRYKYLRTYPTDPAAYAPVTGFYSMQYGSTGLEHAEDSVLNGSDNQLFGRHLVDLVSGRDPRGGNVVTTIDPVMQKVAYEQLTSKGYTGSVVAIEPSTGRILTMVSTPSYDPNQLSSHDGAAVTQTWNDLQQDPRSPMLNRAVSQTYPPGSTFKVVVTAAALSAGVAQPDDQFTAASRITLPDTATTLENYNGNHCGPGDSQTASLTTAFKLSCNTAFVELGIKVGSAKLKDEAAAFGIGQHEGIPIPVADSTVGTIPDGAALGQSSIGQRDVALTPLDNAVIAATIANGGVRMRPYLVDQLQGPDLSVLSTTKPMSVGQAVNAQVASQLTTLMMESEKNTQGGGRSPYTIASKTGTAEHGSDPRNTPPHAWYIAFAPAQNPKIAIAVIVENGGDRALAATGGSVAAPVARAVLDAGLAGG

>CORE_REP|Org103_Gene1246#

MFVGKVLAMEKMYAGGRLRALREQRKLSQSGLAKMLGLSVSYVNQLENDQRPLTVPVLMRLTTTFDLEVNFFAPETDARLLADLQGVFAENPEAGPLTSGELDDLLTRAPAAARLLIHQHRRLRAADDQLDQLTAGIDKPASAPRAAMPYEDVRDYFYDRRNHIPALDLAAEELFDRNGFTLGGLDLQLARLLRDQHDVAVRIRSEDPAKPGPKRIYDPATRTLTLARRLTAGQRAFQLATQLAFITQTEVIEELLAETAALPAESRRLLRIGLASYFAGALILPYGRFLDAAEKLRYDIDLLSAQFEVGFETVCHRLSTLQRRGRRGVPFFFVRTDRAGNISKRQSATAFHFSRVGGSCPLWVVHDAFATPGRIRTQIAQMPDGRTYLWLARTTGERSPGYRAPSRDFAVGLGCDLTFADKLVYSQGLPIHDPSEAVPIGAGCRVCERGDCAQRAFPQIGRPLAADENRPATTPYAPASPAVGDRADQW

>CORE_REP|Org12_Gene4594#

MRCPRSASKTTATIGIVDVVASVRAEGSVTLRSPTPRPARAHPASAPAAAHTPARTTARIPSPPACEPSRAAGAATGPHAVRDLFPALADDGPVYLDSAATTQKPLPVIEAIEGYHRHHTANSGRGTYPWATTLTRAIEGVRADTARFLHADPDEVVFTAGATAGLNAIALAWGLTTLADGDEILYSPRDHASNVYPWLQLRATLAHFGRRLRLVPYRTTALGEADIDDIAAKLGPRTRLLTLSHLHHVYGARNTLEELRDRIDARVAVCFDCSQSAGHIPIDVRELGADFAVLSAHKMFAAPGTGVLFCHRRVHDQLSPFLPGGNSGVSVRDSALLPARMPHRLEGGTHNIPGILALGAALRVLDSIGIDTIERHNRMLTRRLVDGMRALPGLRLLPGPGHAPCDTGYGIVSFTLDGITATDLGFVLAELGFLVRTGAHCVPADTAGENAEVVAAEADSVRVSTHVYTTAEEIDRFLGCLTTIATEVR

>CORE_REP|Org128_Gene2505#

MTSTTAASRESDAPQNQALTQEETIASLGTYGYGWADSDIAGASAKRGLSEDVVRDISAKKNEPEWMLDIRLKALRIFDRKPMPNWGSNLEGIDFDNIKYFVRSTEKQAESWEDLPEDIKNTYDKLGIPEAEKQRLVAGVAAQYESEVVYHQIREDLESQGVIFLDTDTGLREHPEIFQQYFGSVIPAGDNKFSALNTAVWSGGSFIYVPPGVHVDIPLQAYFRINTENMGQFERTLIIVDEGAYVHYVEGCTAPIYKSDSLHSAVVEIIVKEGGRCRYTTIQNWSNNVYNLVTKRAKAGAGATMEWIDGNIGSKVTMKYPAVWMTGEHAKGEVLSVAFAGEGQHQDTGAKMLHLAPHTSSTIVSKSVARGGGRASYRGLVQVNKGAHGSKSTVKCDALLVDTVSRSDTYPYVDIREDDVTMGHEATVSKVSEDQLFYLMSRGMTEDEAMAMVVRGFVEPIAKELPMEYALELNRLIELQMEGAVG

>CORE_REP|Org153_Gene2292#

MSKFIDRVVLHVRAGKGGHGCASVHREKFKPLGGPDGGNGGNGGDVILEVDPNVHTLLDFHFHPHAKASNGKPGEGGNRDGKQGTDLLLKVPDGTVVLGADGEVLMDLVGAGNRFIAARGGRGGLGNAALASKARKAPGFALLGEEGEERDIVLELKSVADVGLVGFPSAGKSSLVSVLSAAKPKIADYPFTTLVPNLGVVASGDTTFTVADVPGLIPGASEGRGLGLDFLRHLERCAVLAHVVDCATLEPGRDPVSDADALEAELAAYKPALSADADLGDLADRPRVVILNKTDVPDAAELAEMVTDEFTARGWPVFQISAVSRAGLRPLTFALADMVRKYREEHPKAAPKRPVIRPIAVDETGFSVIADPEEPGGFIVRGTRPERWVRQTQFDNDEAVGYLADRLARLGVEDELVRLGAEPGAPVTIGDVTFEWEPQISAGVDMVPTGRGTDIRLEQTDRVSAAERKHASRVRRGLVRDDEDEA

>CORE_REP|Org75_Gene2079#

MAVSLGKAGHTPAARESGYAAHRAGVDRLLASYRAIPPEATVRLAKKTSNLFRARAANPAPGLDVSGLTRVIAVDPEARTADVAGMTTYEDLVATTLPYGLAPLVVPQLKTITLGGAVTGLGIESTSFRNGLPHESVLEMDVLTGAGEILTVTPDGEHADLFRGFPNSYGTLGYTVRLKIELEPVPPYVALRHVRFHDLRELEAAIAAVVEDRSYDGERVDYLDGVVFTATESYLTLGRQTDEPGPVSDYTGMDIYYRSIQHDSPHPKRDRLTVHDYLWRWDTDWFWCSRAFGTQNPKIRRFWPKRYRRSSFYWKLVALDHKYHIGDKIEARKGNPPRERVVQDIEVPVERTADFVSWFLREIPIEPIWLCPLRLRAEGPAVSGAGAAGTRAWPLYPLERDRTYVNVGFWSAVPTVPGQPEGAANRAIERTVTEFDGHKSLYSDSYYDKDEFAALYGGDSYTELKKRYDPDQRLLDLYSKAVQRK

>CORE_REP|Org198_Gene7470#

MRGHNGTERQRGGIVSAHPAGPRHAEIPHAPSLPERPREPQQMIDLPANVWPRNASRDSDGVVRLAGVPVHELAAEFGTPLFVVDEDDFRSRCRDMVRAFGPNARVHYASKAFLCGEIARWIRDEGLSLDVCSGGELAIALHAGFPAERIALHGNNKSATELEAAVTAGVGHVVVDSLIEIERLEAIAGRAGVVQDVLVRVTVGVEAHTHEYISTAHEDQKFGFSIAGGDAMEALARVFEADNLRLVGLHSHIGSQIFEIDGFEIAARRMLRLLHDAIEKFGVERTAQISTLDLGGGLGISYLPNDDPPPLDDFAAKVRDLVAAEAASIGLPEPKIAVEPGRAIAGPGTVTLYEVGTTKDVSLDGGLRRRYISVDGGMSDNIRPALYQADYDCRLVSRTSEAAAVVARVVGKHCESGDIVIRDTWMPEDVGPGDLVAVAATGAYCYSMSSRYNQLTRPAVVAVRDGQPRLILRRETVADLLSLEV

>CORE_REP|Org81_Gene2965#

MKVTCSSPTTAIVSGPPTGLPRRVLSCAGGHRGEPCVRSRGEPCVRSLGGLCFGSGGESGFAASRESGVAAGDVSGATVSRGSGGAVRREFDAAGDRAAVERSAESVGVDRRGGPVGVRPADRHTADVPAADPARAATEPSGSVDHQRLRHHGDVDARPGMVDFAVNVQGTAPPEWLRQRLAGRLEELGRYPDSGEESATRAAVAARHGRRAEEVLLLAGAAEGFAMLPRLGARRAAVIHPSFTEPELALREAGVPVTRVVLEPPYTLDAGLVPEQADLVVVGNPTNPTSVLHPAEALLALRRPGRIVVVDEAFADAVPGEPETLSGIDLPDVLVLRSLTKTWALAGLRCGYALGAPQVLARLNQGRPHWPLGSLQLEAIAATSAPAAVVETRRKAEVIAADRAAMIPRLRTLGIDVHEPAAGPFLLIRVPDAELLRKRLADKGIAVRRGDTFPGLAAGFLRVAVRPPAEVDRLVAAIEDVGL

>CORE_REP|Org31_Gene5564#

MAKQVPTSRLARGTKLGAVAASSVIRTQRARLSMRGRSEAVRAKMAEESMIRTTEQVVMVLGTMKGVAMKLGQMMSVLDLDLVPDAHRERFQKRLAVLRNAAPSVSFESMRQVIEDDFGQPLDAVFAEFEAEPVAAASIGQVYRARLRDGRQVAVKVQYPGIDAAVRADLKNLAMFRRVLQSAMPWVTPAVLDELRLNMESELDYQAEANTQLQIAELYAGHPFIVVPRSLPELSTTRVLVTEYVAGKGFEEIRQLPDAERDRIGEIIYRFYVGSLFTFNEFCGDPHPGNVLLAEDGRVGFLDFGLFNRMDPGHVQFELTCLRAAAEDRAEDLRELMIERGVIDSPEEIGAEECLEYVLAASEWCLIDEELTITPELASGAFLLAVDPRASEFAGMKQQNLPPEHLFSRRADFLTFGMLGQLGCTANWHRISREWLYNEPPVTELGRAHHVWLAEHPPVAPKKSRAKSSKAGKSTKAARPQA

>CORE_REP|Org152_Gene795#

MSVLLVGISHRSAPVAVLEKVAITEDDRPKLTDKMLASSHVSEAMIVSTCNRVEIYAVVDAFHGGLGEISDMLTRHSGLPLPELTKHAYVRYAEAAAEHLFAVASGLDSMVVGEQQVLSQIRAAYAASDAQQAAGRTLHELAQHALRVGKRVHSETGIDRAGASVVSVALDRARAVLGAAELSREAAASVSENAVAPYDSALIGRTALVLGAGAMGGLAVAQLARAGVGRIIVVNRTLERAQRLARTATDMHGVTADAMEMSRMVEAMAVADVVVTCTGAVGAVVTLADVHRALAGRPHQHLVICDLGLPRDVEHAVAGLPGVTVIDMETLQRDPSAGAAADDTVAARGIVADELAKYLAGQRMAEVTPTVAALRQRAAEVVEAELLRLDSRLPSLAADDREEVARTVRRVVDKLLHAPTVRVKQLASTPGGDSYAEALRELFELKPGAAQAVAAPMEIAALGELADDFTAAHLGDEQGPNS

>CORE_REP|Org5_Gene6276#

MTVARSAESVHAVVKAYDVRGVVGEQIDAAFVRDVGAAFARLMRDSATRIAIGHDMRESSPELAAAFADGVLDQGLDVVHIGLASTDQLYFASGHLQCPGAMFTASHNPARYNGIKLCKANALPVGQETGLATIADELIEGVPAGPGPRGTATEQNLLEAYAEFLRGLVDLSGIRPLKVAVDAGNGMGGYTVPAVLGAVSQLTIEPLYFELDGSFPNHEANPLDPKNLVDLQKFVRETGADIGLAFDGDADRCFVVDERGEPVSPSAVTALVAERELAKEPGATIIHNLITSQSVPELVTELGGTPVRTRVGHSFIKQQMASTGAIFGGEHSAHYYFRDFWGADSGMLAALHVLAALGGSDGPEGGSLRNHEGPRGRRIEHSEKDDRTMSELSSSYSTYAASGEINSTVADAKDRTLAVVTAFEGRARSVDRLDGVTVRLPGNAWFNLRASNTEPLLRLNVEARSQEEVDALVTEILSIVRG

>CORE_REP|Org63_Gene113#

MDSVSQRLDLRPNRIAVLSVHTSPLAQPGTGDAGGMNVYVLQTAVELARRGTEVEIFTRATASNLPPVQEAAPGVLVRNVVAGPFEGLDKHDLPTQLCPFTAEVLRQEARHLPGYYDLVHSHYWLSGQVGWLARDRWRVPLVHTAHTLAAVKNAALAEGDCPEPATREIGEKQVIAESDRLVANTAEEARQLVELYGADPERIDVVPPGADLTLYRPGDKAAARAALGLSADEQIVAFVGRIQPLKAPDVLVRAAAEVLRADPERPLRVLIVGGPSGSGLDRPDALIELAAELGIAARVSFLPPQPPQRLVLVYRAADLVAVPSYNESFGLVAIEAQASGTPVLAADVGGLGTAVRHDVSGLLVPGHRTSDWANALRHLLDDPGRLHRMGERAVAHAANFSWAHTADGLLASYAAALAGFRDERSALGGRGLAHSLVRDDAYDRAAADRTNLAGERTAALLAESSQARSRALWRRRMGVRR

>CORE_REP|Org13_Gene5367#

MTIMAPAQADATTDPRDPLGRLQRFFDPGTVLPLHPRDKSGVLAAIGEVDGVRTVAYCSDATVMGGAMGVDGCKHIVDAIDTAIDSRIPVVGIWHSGGARLAEGVEALHAVGTVFEAMVRASGLVPQISVVVGFAAGGAAYGPALTDVVIMAPEGRVFVTGPDVVKSVTGENVDMATLGGPETHGKKSGVCHIVADDENDAMHRGRRLVSMFAEQGEFDLSAAAHGDVDLKAMLPESAKRAYDVKPVVRELLDKIPSPDGNDESSFEEMQGGYARSIVTGLGRLGGRTVGVLANNPLRMGGCLTSESAEKAARFVRLCNSFGIPLVVVTDVPGYLPGVGMEWEGVVRRGAKLLHAFAEARVPRVTLVTRKIYGGAYIAMNARSLGATAVYAWPESEVAVMGAKAAVGILHKKAIAKAPEEEREALIERLTAEHETIAGGVGRALSLGVVDEVIDPAKTRSTIAAALASAPAQPSHNKNIPL

>CORE_REP|Org127_Gene3048#

MSPSVTPSSNSDTLAGDGIWTDETDWEIADFEGDADAHEHVPMPTVAVVGRPNVGKSTLVNRILGRREAVVEDIPGVTRDRVSYEASWAGRRFLVQDTGGWEPDAKGLQQAVARQAELAMQTADAILLVVDATVGATATDEAAVKALRRSKTPVILVANKVDGEKAEADAAVLWSLGLGEPRMVSAAHGRGTGDLLDDVLAVLPETPREGSGGGGPRRVALVGKPNVGKSSLLNKLSGDERSVVHDVAGTTVDPVDSLVELGGKTWRFVDTAGLRRKVGTADGTEFYASLRTKAAIEAAEVAIMLIDASEPITEQDLRVIGMVADAGRALVLAFNKWDLVDEDRRYQLEREVERELVRVPWAQRVNISAHTGRAVQKLVPAMETALESWDQRIPTGRLNTWLKEVIAATPPPMRGGRLPRVLFATQATTRPPTFVLFTTAFLEAGYRRFLERRLREEFGFDGSPVRISVRVREKRDRSKK

>CORE_REP|Org19_Gene6498#

MGRPEEAAECYRGGRPIGLQTTLPNGWQTQADQAAPVHRYAEPVNDRSRVADPDELPATTSSARSTGGLTTRQRRLLLIAIALFVVSAIVSWAAHIWTGYIDLQVYRNGARTWLDGGDLYGPMPKVYGIGLPFTYPPLAALFFAPLALMPLAVAQWLVLLTSMASLAVTLWLVLVRIRPEMDRSTRVILLIGALAVLGLSEPVRQTYNFGQINLILMAAVALDALVRKPFWPRGMLIGITVAVKLIPAGYLLYFLLRRDWRACLTLIGSAIGAIALAYLLFPHDSTEYWFHTLIDTGRIGPPQYAGNQSLKGFAFRLGVSDGAATAIWIGLSLIAIGLAALWMKRLLDAGHQVSALLVNSAAVLLVSPVSWSHHWVWVAPALLVAGDLIARMPAADAPDTPGRAARRRRMWIAITAVITVLFMVGPQWVLPHNADRELRWAWWQQIIGSSYVLVTFAALVIAAIAYRPSGVRNAASAAE

>CORE_REP|Org169_Gene5623#

MFVSLVPNVLATRYASPQLVQLWSPENKIVLERRLWLEVLRAQTELGAAGTEAVTPEVLEDYERVLGEVDLASIAERERITRHDVKARIEEFNALAGHEQIHKGMTSRDLTENVEQLQIRLSLEHVYEHGVAVAARLAERAAEYQSLVMAGRSHNVAAQATTLGKRFAGAADEVLIALHRVRELIDRYPLRGIKGPMGTAQDMLDLFDGDAAKLAQLEQKVAGHLGFATVLTSVGQVYPRSLDHDVISALVQLGAGPSSFAHTVRLMAGHELVTEGFQPGQVGSSAMPHKMNTRSCERVNGLQVVLRGYGSMAAELAGAQWNEGDVFCSVVRRVALPDAFFAIDGMMETFLTVLGEFGAYPAVIERELNRYLPFLATTRILMAAVRAGVGRESAHEVIKEHAVAVALAMREQGREPDLLDRLAADDRMPLDRAGLEAALADRTAFIGAAEAQVGDVVAQVQKLIDANPEAARYTPSPIL

>CORE_REP|Org85_Gene7073#

MTLRLFDTDTRTTREFAPLVPGRASVYLCGATVQGEPHIGHVRSGVAFDVLRRWLLAHDYDVWFIRNVTDIEDKILHKAAEAGRPWWEWAATYERAFDNAYETLGVLPPSIEPRATGHITQMVDLMQRLIERGHAYASAGNVYFDVRSYPEYGSLSGHRLDDVHQGESAGEGKRDPRDFTLWKAAKPGEPTWPSPWGPGRPGWHLECSAMAEFYLGPEFDIHCGGMDLVFPHHENEIAQSKAAGDGFANYWLHNGWVTLGGEKMSKSLGNVLSVPNVLKQVRAVELRFYLGSAHYRSMLEYSDKALHDAAQTYQRIEAFVHRTADRAGDIPVGKWTDAFAAAIDDDLAVPKALAEIHRVVHEGNKALESGAVDSARDLAGQLRAMLGILGVDPLDPHWFTPSDSSAAIGALDVLVRAELDRRQQARAAKDWASADAARDRLQAAGIEVTDTPNGPEWALAAAQHLPPDAQQPGKAD

>CORE_REP|Org56_Gene2351#

MAERPRTLAEKVWDQHVVVRGAGEGAQREPDLIYIDLHLVHEVTSPQAFDGLRAAGRPVRRPDLTIATEDHNVPTVDIDKPIADPISRTQVETLRRNCEEFGVRLYPMGDIEQGIVHVVGPQLGLTQPGMTVVCGDSHTSTHGAFGALAMGIGTSEVEHVMATQTLSLRPFKTMAINIDGELPPGVTSKDVILAVIAKIGTGGGQGYVLEYRGEAVRAMSMEARMTMCNMSIEAGARAGMVAPDEVTYEFLKGREHAPTGADWDAAVAAWEALKTDPDAGFDAEVHLDASTLTPFVTWGTNPGQGAPLGDVVPNPEDFADENERAAAEKALTYMDLEPGTPLREVPVDTVFVGSCTNGRIEDLRAVADVLKGRKVADSVRMLIVPGSMRVRAQAESEGLGEIFTAAGAEWRQPGCSMCLGMNPDQLSPGQRCASTSNRNFEGRQGKGGRTHLVSPQVAAATAVRGRLSAPADLN

>CORE_REP|Org154_Gene4464#

MRCCCPHRPAGARHGCNVEDVQRRIMGIETEFGVTCTFHGHRRLSPDEVARYLFRRVVSWGRSSNVFLRNGARLYLDVGSHPEYATAECDSLHQLVTHDRAGERVLEELLIDAEQRLAEEGIGGDIYLFKNNTDSAGNSYGCHENFLVVRAGEFSRISDVLLPFLVTRQLICGAGKVLQTPKAATFCLSQRAEHIWEGVSSATTRSRPIINTRDEPHADAEKYRRLHVIVGDSNMSETTTMLKVGTAALVLEMIEAGVAFRDFALDNPIRAIREVSHDLTGRRPVRLAGGRQASALDIQREYYARAVEHLRNRDRDPQIDQVVDLWGRALDAVEAQDFAKVDTEIDWVIKRKLFQRYQDRYDMELSDPKIAQLDLAYHDIKRGRGVFDLLQRKGLAKRITEDEAVDAAVDTPPQTTRAKLRGDFITAAQEAGRDFTVDWVHLKLNDQAQRTVLCKDPFRSVDERVDRLIASM

>CORE_REP|Org112_Gene2784#

MVATNTRETAESADAADSADTTAARPVKKAAAKKAPAKKAAAKKTAAKKTAAKKTAKATKATKAAKKAAPKKAGEGADGAETENLDDESLEIDDLGDLEVDEEDLGDEELEVEDDEAEDEAEEAEAETEEEADEPTAKDKASGDFVWDEEESEALRQARKDAELTASADSVRAYLKQIGKVALLNAEEEVELAKRIEAGLYATEKIREYADKGEKLNVQLRRDLNWIMRDGNRAKNHLLEANLRLVVSLAKRYTGRGMAFLDLIQEGNLGLIRAVEKFDYTKGYKFSTYATWWIRQAITRAMADQARTIRIPVHMVEVINKLGRIQRELLQDLGREPTPEELAKEMDITPEKVLEIQQYAREPISLDQTIGDEGDSQLGDFIEDSEAVVAVDAVSFTLLQDQLQSVLETLSEREAGVVRLRFGLTDGQPRTLDEIGQVYGVTRERIRQIESKTMSKLRHPSRSQVLRDYLD

>CORE_REP|Org101_Gene365#

MVGNMADETQYRIEHDTMGEVRVPVDALWRAQTQRAVENFPISGRGLERAQIRALGLLKGACAKVNKDLGLLDPAKADAIIAAANEIAAGAHDDQFPIDVFQTGSGTSSNMNANEVIASIAKANGVTVHPNDDVNMSQSSNDTFPTAVHLAATEAVITDLVPALEHLRLALLDKSTEWRTVVKSGRTHLMDAVPVTLGQEFGGYTRQIAASIDRVMATLPRLGELPIGGTAVGSGLNAPDGFGGKVVAELVRATGIDALREARDHFEAQAARDGLVEASGAVRTVAVSLTKVANDIRWMGSGPLTGLGELQLPDLQPGSSIMPGKVNPVLPEAVTQVAAQVIGNDAAVAFGGANGAFELNVYIPVMARNLLESIRLLANVSRLFADKCVHGLVANVEHLRTLAESSPSIVTPLNSAIGYEEAAAVAKEALKNKKTIRQTVIDRGLLDEKLTEAELDRRLDVLSMAKVKDGK

>CORE_REP|Org6_Gene4755#

MPGMRHDRLPDGFGVRIDPRVRAYSGNRILIGGTPARVLRLAPEAAEMIGDGYLEVTGPKSAVVARRLLDSGVANPRPRLLPSTDDVTVVVPLHNNPEGLARMLAVLRGHHVIVVDDGSDQPVRIPETRGTRCRVTVLRHDTAHGPAAARNAGLRAATTEFVAFLDSDVVPRSGWLEVMLGHFSDPEVALVAPRIVALDAESNALARYEHTRSSLDLGRREAAVHSRGPVSYVPSAAMLVRRQALLAVGGFDESMRVAEDVDLCWRLERAGRRLRYEPAAHVAHDHRVAFRAWFGRKMFYGTGAAPLARRHGPVAVSPLSLPYWTALAAVLFATLTRWGLLGGLVALATALVRLRRVFAGLDNPTRIAALYLARGFFAGLWRIASAMCRHYWPITLLAVLVSRRVRRIAVTMAVADGLADWFTHRDAGGLDPVRYLVYKRLDDLAYGTGLWVGAARARSLDALRPAFSRR

>CORE_REP|Org26_Gene4804#

MMMSTRDSATKRRPDSGRSQDSAVGLNQPKRDWMGAAMRVMTTLTGSELAEKYNLRKPIERVTYEGTKTGFRTLGAATRAFNKVAGGGQPKRLATNEAKNKDYFDLTPTDEQQMIVETVREFAAEILRPAAHDADEAAAAPKDLLGRAAELGITLINVPEELEGAASERGAVTNSMVAEALAHGDMGLALPILAPSGVAVALSQWGTDAQQQTYLPAFTGENVPQASVVISEPRALFDPFALQTKAVRSPSGYRLSGVKSLVPAAADAELFIVGAELDGRPALFIVESDAQGLVVEADPSMGLRAAGLGRLILDNVAVGSDALLGDGDGKQHAEDYADAVRLARLGWASLAVGTGQAVLDYVIPYVNEREAFGEPISHRQAVAFMVANIAIELDGLRLVTLRGASRAEQGLSFAREAALAKKLATDKGMQFGLDGVQLLGGHGFTKEHPVERWYRDLRGIGVAEGVVLV

>CORE_REP|Org53_Gene4027#

MTAARSVSASSATSHSTGTGKTAAVSSDSKGARSSHSQASAKTFVIDTSVLLSDPWAFTRFGEHHVVLPLVVISELEGKRHHHELGWFAREALRNLDDLRLLHGRLDQQVPIGTEGGTLQVELNHTDPSVLPVGFRTETNDSRILACALNLAAEGRRVVMVSKDIPLRVKAGAVGLHADGYHAQDVVTSGWSGMVELDVASSQIDQLYAESVIDLDAARELPCHTGIRLLGGSSSALGRVTPDKRVQLVREREAFGLHGRSAEQRIALDLLLDESVGIVSLGGKAGTGKSALALTAGLEAVLERRTQRKVVVFRPLYAVGGQELGYLPGSESEKMGPWAQAVFDTLDGLASPEVMEEVLSRDMLEVLPLTHIRGRSLHDSFVIVDEAQSLERNVLLTVLSRLGSGSRVVLTHDVAQRDNLRVGRHDGVAAVIEKLKGHPLFAHITLTRSERSPIAALVTEMLEEYGPNA

>CORE_REP|Org67_Gene2964#

MTRIAIIGGGPAGYEAALVAAQHGAQVTLIDRDGIGGACVLWDCVPSKTFIASTGMRTDLRRARDLGITLDPSQAAVQLPEVNARVKALALAQSSDIRSKLLSAGVTLISGTASFTDPAPGRAPHRITVRPTGERAGERVIDAEVVLIATGASPRVLPGAEPDGERILTWRQLYDLRELPETLVVVGSGVTGAEFVSAYTELGVQVKLVSSRDRVLPGEDADAALVLEEALAERGVELVKHARADAVERTADGVVVKLSDGRTVAGTHALMTVGSTPNTGDLALDKVGIELDRGGYLRVDRVSRTAVSGIYAAGDCTGLLPLASVAAMQGRIAMYHALGEGVSPIRLKTVASAVFTRPEIATVGVSQTAIDNGEVPARTVMLPLNTNPRAKMSGLRRGFVKIFCRPATGVVIGGVVVAPIASELILPIALAVQNNLTVNDLAQTFSVYPSLTGSVTEAGRLLMRHDDLD

>CORE_REP|Org16_Gene180#

MAKTYVGARLRQLRTERGLSQISLAKKLEISASYLNQIEHDVRPLTVPVLLRISEVFGVDTSFFASQDDTRLIAELQEVVMDTELGIEADAQEIADMVSAHPSLARALVNMHRRYRNTTAQLAAATEDRFSDGSGSGAISRPHEEVRDFFYQRQNYIHELDTAAEELATRMRLHGGDLRRELTRRLTTGYGVQIVERIDLGEGVLHRYDPEARKLEIAPHLSGGQRVFKLATELAYLECGDLIDKLVEEGNFASEDTRTLAKLGLANYFAAATVLPYSHFHEVAEDFRYDIERLSAFFAQSYETICHRLSTLQRPKLRGVPFSFVRVDRAGNMSKRQSATGFHFSSAGGTCPLWNVYETFAYPGRIMTQIAQMPDGRKYLWIARTVERRATRYGQPSKTFAIGLGCELRHAGRVVYADGLDLTDPQATPIGAGCRVCERANCPQRAFPPLGKSLDISEHRSSISPYVLR

>CORE_REP|Org102_Gene1432#

MTTTLHPSSEPPRPVGEGTESAEVAGAIESNSAPAGAGADISAPPRDDSTEATTASDTGSMAATSSAFAMAPRHGDAAASGIERDAVERAANVASEPGAASTEFPRRPAGFLPALEGMRGMAALGVVVTHVAFQTGATSLPMVGRVLERFDMAVAVFFALSGFLLWRPHAAAARGLGTAPTAGRYLLHRAARILPAYWAVVCAVLILLPTAASTAGFRVWLANLGLVQVFVPLTLTDGLTQMWSLSVEMAFYLLLPLLAVAVAWLRGDRARWRVPVLLAFGTLCLTWNLIPVPTPDAINSDNWLPGYLPWFAAGMLLAELSDLAVPRLRRLAGNPWILWTIALVALLLSATDLGGLPGLTRGAPWQYVLKMAFGAIIGFTLLAPLVLRPDIRHRWLESRTAAMLGRWSYGVFLWHLAVLSIVFPVFAIVPFSGDFPQVLALTIALTLPLAAASYALIEEPVRRWARRFG

>CORE_REP|Org151_Gene6489#

MSVKSTVEQLSPTRVRINVEVPFEELKPDFDRAYKALAKQVKIPGFRPGKAPAKLLEARLGRGAVLEQVVNDVLPGRYSEAVTAGQVKVIGQPEIEITKIEDGEELAFTAEVDVRPEITLPAYDGIEVTVDAFTIGDEDIEEQLLSLRQRFGTLTGVERAVQDGDFVSIDLSATVDGEEVPEAATTGLSHEVGSGQLIEGLDEALIGLNSGESKEFTSTLVAGEHAGKEAVITVTVQSVKERELPAADDDFAQLASEFDTLDELKEDLRTRVERSKKVQQAGEIRDKVLETLLEQVEVPLPEAVVKAEIDAVTHDAVHGFDHDEAKLAEALEAQGSSREEFDKDAKESAEKSVKTQLLLDAIAEADNTQVGQEELTERILFQSQRYGLAPEQFIQQVQQAGQLGAIFADVRRGKALAGVVGKVKVTDSAGNAVDTAEMFGAPEDSAPEIEADGVVEVESDETAGAKAE

>CORE_REP|Org215_Gene5410#

MVTCRDSIVIGSCISHIGRLDEVHEVRRHRPVRRRRHRNRRRDRIRCTGGSAHRTAAGLRAGARAGSGCRLHHRAGRGGRRHRHQRHRRSVQRRRRRAGREPDQRIRCGGGAGAADRKSRWGGRADRRGRRRQRSGTVADPRGRAARARAGHLRATVVLRRIAARRARRGRGRGDRRRHRPARAGRLRDSRRHHRRAHRPAGRRWSAADRLGLRLLQRAALRALILGGTREARVLAETASGERGFEIVSSLAGRVRDPLLPVGQVRIGGFGGVDGLRTWLSDNRIEAVVDATHPFAAGITDHAAAAAASLGLPILHVRRPGWTQRPGDSWIRVPDLSAAATAVAGLGDRIFLTIGRQGVSAFAALRGHWFLIRAIDPPEGALPPRHELLLARGPFTVADETALLTAHRITALVTKDSGGAQTEAKLDAARARGLPVVVVDRPPLPTGARSVDSVAGAWDWLRAAQRLS

>CORE_REP|Org4_Gene3246#

MNAVSQGDSVSGGGRSAVGPNSAQMVWGRDQPIGVAVLGMGNVGTEVVRILREHAEDLRSRVGAPVVLRGVAVRDLATDRGIPTALLTTDADALVARDDVDLVVEVIGGIDPPRRLILAALNAGKSVVTANKALLADYTGELAAAAERNRADLYFEAAVAGAIPVVRPLIQSLAGDRVNRVVGIVNGTTNFILSAMDETGADYADTLAEATRLGYAEADPTADVEGFDAAAKAAILASLAFHTRVTAADVYREGISKISSEDLETASALNCTVKLLAICERVAAGPGEPSPEEGGKERVSVRVYPALVPRKHPLAAVSGAFNAVVVEAENAGRLMFYGQGAGGAPTASAVLGDLVMAARNKFYGGRAPGESVYAELPIAPIGDTPTRYHVNLQVEDRPGVLAAVAGEFAKHGVSISTVRQEGHGTGARLVVVTHHALESALADTVAALAEMESVTSITSVLRLEGTEE

>CORE_REP|Org102_Gene679#

MEGMRIADHVVDLIGNTPLVRLNSVVGPNSGLVAAKVEYLNPGGSSKDRIAVKMIDAAEQAGLLRPGGTIVEPTSGNTGVGLALVAQQRGYKCVFVCPDKVSEDKRNVLRAYGAEVVVCPTAVAPEDPQSYYNVSDRLVREIPGAWKPDQYSNPGGPDSHYETTGPEIWRDTEGKVTHFVAGVGTGGTITGTGRYLKEVSGGKVQIIGADPEGSVYSGGTGRPYLVEGVGEDFWPSAYDPAVPDEIIAVSDADSFDMTRRLAREEGLLVGGSCGMAVVAALRVAERDPDAVVVVLLPDGGRGYLSKIFNDDWMSSYGFLRSRLDGSAATEPLVGDVLRGKSGALPDLVHTHPQETLRDAIEILREYGVSQMPVVGAEPPVMAGEVAGSVSERDLLSAVFEGRAHLTDSVKQHMSPAFPLIGSGEPVSAATKALEETDALMVVEDGKPVGVITRHDLLGFLSTGALGH

>CORE_REP|Org13_Gene5085#

MEDHVTTPESVRLTSAVDPATAVSRLACSGHFSDYIVYERPGRWVFAAAPLGRVELDTDELRVSTSAGSARERWTGRPVDALERALDSLRVTCGPGASGTAYGWIAFEFCADALGAQRHLTERADLAHVIIPRIEVTVTESGVGVDGATAAEVEVIEQLLLSPAEPLPTPHPVDVRADTCGYRARVAAAVAEIAAGDYQKVILSRRVELPFRVDLPASYRLGRAHNTPARSFLLRLGGLAAAGFSPELVVSVDDEGVVTTEPLAGTRALGQGVAADLAARNDLESDPKEIVEHAVSVKTSFAEIASIAEPGTTTVADFMAVRERGSVQHLASTVRGRLARHRTRWDALDALFPAVTASGIPKRAAVDAVFRHDAARGLYSGAVVTLSESGSLEAALVLRAVYQDADAAWVRAGAGIVAQSRPDREFEETCEKLGSVAPYLVPAISHGPGSTPRSPMVTGRRSHRS

>CORE_REP|Org141_Gene3803#

MGRLFGTDGVRGLANESLTPELALRVSGAAAQILSRGKKRALAVVGRDPRASGEMLEAAVTAGLTAAGVDVLSVGVLPTPAVAYLTGLYDACLGVMISASHNPMPDNGIKIFAAGGHKLDDAIEDRIEAVMAEAPLRPTGAGIGRVLGASGARDHGLAIPDQYSVAGTHERYVEHLVEATGHELNGLTVVVDCAHGAASEVGPAAYREAGATVIAISADPDGLNINDGCGSTHLDQVRRAVREHGADLGLAHDGDADRCLAVDADGNVVDGDAILAILALAMRDAGELAENTLVATVMSNLGLHIAMREAGITMRTTAVGDRYVLEELRRGRFTLGGEQSGHVVFPAHGTTGDGILTGLKLMGRMASTGRTLADLASVVQTVPQILVNIPVADKAAVMAAADVLDAVADAERELGETGRVLLRPSGTEQLVRVMVEATDLAQAQRLADDLAERVAAVQRISAPSH

>CORE_REP|Org112_Gene6545#

MRTVPTATAPRPPRTPRRNPRPAPHRTRPTARTPPPWEPPAPAVVAAPTRIPTPTTAVVSRWRRSWPTCAPRATANRTHGTSGAGRRPRVPRGRRPAVSSGTSHSSWPPRIGTDILVGVTSGTRVARDWNGTFERTRVTSDKAVCGTDPAPARLTVGIVSAGRVGSALGAALERAGHVVFGVSGISDASVYRARTRLPDSEILPAEEVARRAELLLLAVPDSELAGLVSGLATADAVRPGTIVAHTSGANGIGVLAPLTARGALPLAIHPAMTFTGHDEDVSRLGNACFGITAADDIGYAIAQSLVIEMGGEPVRVAEEHRTLYHAALAHGSNHLVTLILDAVEALRAALAGPGLLGQQLVDDQPGGLAERVLAPLASAALDNALRRGPSALTGPVARGDVDAVAAHLNALESTDAELAAGYRALSLRTAQRARTNPALLELLASPSDRRDAAEGSDQAKEGN

>CORE_REP|Org162_Gene1548#

MIPCSTDVGRRRSGGTDVEVGNDRANDPTDAEQLAQRYRSLVEHSPDGVVVHERGILVYANPAIVRLLGADSTEDLVGQPVTRFVDPKSVPGMLARIGRLTEAGAASEPAEMTLVRTDGSLLDVETVSVLTAWHNRLAYQVVIHDLSAQRAAEAAQRRAEQHFTTVVSQLEEGVVVIDRQGRIESINPAALRIFGHEGEDLVGTPIYALPLTLLDANAMTLPPTRHPVARTLATGETVVGYVFGVDRPDGQRRWLSGSSRLLNPGDPQSSAVSSFNDITEFRASRRQLEYQATHDPLTGLANRALVLSRLAAALGTTEDLPVSTVLFIDLDGFKSINDTLGHAIGDTVLQIVAQRLQRGLRTDDIVGRIGGDEFLVLLSGRTLGEDLEALVARLRQTMAEPIIARGHRIQVDASIGITPLHPGDSRTPEAVLHDADVAMYRAKPPGHRDSSPVTRRPNNTHAS

>CORE_REP|Org215_Gene1707#

MNSLFLLALAIVLVPLGGIFAALDSSLNTISAARVDDMVRAERPGAARLAHIITDRPRYVNLMVLLRVLCEITATVLLAAVLLDWMDQLWALVVTAAVMVLVDYLVIGVGPRTLGRQHAYSLALAASLPLQAIGTLLGPVSRLLILIGNAITPGKGFRNGPFASEIELREVVDLAGERGVVADDERRMIQSVFELGDTAARAVMVPRTEMVWIESEKTVAQAMSLAVRSGHSRIPVIGENVDDIVGVVYLKDMVPYADRSRKVRVHEVMRAAVFVPDSKPLDDLLDEMQRRRNHMAVLVDEYGGIAGLVTIEDVLEEIVGEIVDEYDQNEVPDVEDLGNGKYRVSARLSVEDLGELFGMAIEEEDVDTVGGLLAHELGRVPLPGSKAVAHGLVLKGEGGSDARGRVRVHTVVVKRAAEKTGAEKSDAGRSTSERVDGESAGVNGVGGSGANEDGEADD

>CORE_REP|Org39_Gene6539#

MGGLHPFDGSRTPGGCQFDSACVSGPRRRRCRPAGGRGVRAVVGGGRATVGSRETRRRESRVTVTEFRARTAPSRWPRAGPSEVDLVRTLAVVGGGVIGLSVAWRAAESGWRVTLYDPAVGSGASWVAGGMLAPLSEGWPGEDAALEFGAASLTRWPDFAARLKSVTGAEVFTAAETLTVALDAADAADLRTIADWVNAKLAESGAEADPAVGSALRLLDRAGVRSVEPGLDRRVRAGLLSPAEPAVDNRSLVTALREACVAVGVEVRAEEVAALRELPHDRVVLATGASARLWPDLPVRPVKGEILRLRRRLSAPPPPNRVVRARVHGRPIYLVPRPDGLVLGATQYEAGFDTVVTVGGVRDLITDAEAIFPGVGEYEFAEATAGSRPGTPDNLPLIGYLDERVIAALGHGRNGILGVPVTADAVLALLADTELPAARAASPTRFRVPEPHFAGGKQ

>CORE_REP|Org101_Gene5725#

MTDQKPESFPLRRSVAASAMGNATEWFDYGVYAATATYLTDAFFPGELGTLGTMLGFAVSFVLRPLGGMVWGPLGDRIGRKAVLATTILLMAAATGAIGILPTHSSVGVFAPILLIGLRVVQGFSTGGEYGGAATYLAECASDKRRGFLGSFLEFGTLAGFVGGSATVLACQLAIGSDAMHDWGWRIPFLLAVPLGLVGWYLRSRLDESPVFTEVAEVAEQTDQEHRPGGLHGLRELVTTYRRELLTLGGLVVALNVVNYTLLTYQPTYLQKTIGISESGTTAMMLIGQTVMMVTLPFFGRLSDRVGRRPMWLFSLVGLAVLALPMYWLMGQGTAWAITGFIVLGLLYVPQLSTISSTFPAIFPTQVRYAGFALAYNVSTAAFGGTAPLVNEAAIESTGWSLFPAAYMIGASLIGLVAWCFLRETAGTSLRGTEVPDAGEDAPAIIPAGPAGAALAP

>CORE_REP|Org117_Gene5341#

MNRRVRDSGRDDGYGRRTALAREWESGVETLLVVGAGPKALAVAAKSHVLRQLGLSAPRVIAVEAHAVGGNWLASGGWTDGRHRLGTSPEKDIGFPYHSTWARGHNREINEAMMAFSWTSFLVEHGTYAEWIDRGRPSPQHHVWAKYLQWVARKIDLELVLGKVRTIRQRPTDGGAGWSVEVAGADGATTELEADGLMITGPGQSTKALAKHPRVLSIAEFWDLAGKRKLPISSRAAVIGGGETAGSALDELVRHEMLTISVISPMATIYTRGESYFENSLFSDPTKWNALSIQERRDVIRRTDRGVFSVRVQESLLGDNRVHHLQGRVTRIVGQGDGVAVTLRNEMRADQVHNFDLVVDATGGQPLWFLDLFDSESADLLELAVGGPLTQQRIESSIGYDLAVTGLGAKLYLPNMAALAQGPGFPNLSCLGELSDRVLRAEPARVRAGARQLAAQ

>CORE_REP|Org39_Gene2821#

MMLRVSVIGTGYLGATHAAGMAELGFDVLGVDNNAAKAAALAAGRVPFHEPGLPELLSKHVDQGCLSFGTSLAEAASFADVHFLCVGTPESPTGAADLTQLYTAIEGLVPHLTRNCLIVGKSTVPVGTAEALAARVDELAPRGIAVELAWNPEFLREGHAVYDTLHPNRLVFGVSTPDAEWALRQVYAAAIAEGAPVVVTNLPTAELVKVSANAFLATKISFINAMAELCEITGADVNLLADALGHDDRIGRKFLGAGLGYGGGCLPKDVRALIARAGELGVPESVRFLDSVDAINLRRRERVVRETLEILGDERPTGRVAVLGAAFKPLSDDVRDSPALDVAVRLHHAGIEVTVYDPEANRSASRIAPQLRYAPNATAAVLGADVVLHLTEWREFRELDPALLSSVARRRVLIDARNTLDPEPWLSAGWDFRALGRLVARRADAGRVDPLLESA

>CORE_REP|Org30_Gene3982#

MAVAIALFTRDLRVRDNPALTAAARSAEVLPVFVVDETICASDYLSPNKATFLAATLADLDDQLRRLGGCLLLRGGDTVREVCQLVRQYSVDEVHVAADVSGYSRRREDRLRARLTQLGCRLRVHDSGTTVAAPGHLLPSGGTDHFAVFTPYFRRWSSMGMRAPLPAPRRIHLPDGVGTDRVPTAAELRVGEVSPELAPGGESAGRRAARNWFRDGIAAYSDLHDDLAADATSRLSPYLHFGCLSAVELVHRSDSSSPGGAAFVRQLAWRDFHHQMLAARPSAAHSDYRSRNDRWSDDDALLAAWRAGRTGYPIVDAGMRQLAAQGWMHNRARLITASFLTKTLYVDWRAGAKHFMGLLADGDVANNQLNWQWMAGTGADTRPNRILNPIRQADRYDPEGDYVRRWVPELARLRGSDIHQPWRLTQDATTGYPARIVDHEHAAAEFRTLRQRDSH

>CORE_REP|Org4_Gene7917#

MPLQQFESFIRSAVGHIDLNEEVESEMSNAGTPKTAAEIQQDWDTNPRWKGVTRNYTAEQVSKLQGTVVEEATLARRGSEILWDLVNNEDYINSLGALTGNQAVQQVRAGLKAIYLSGWQVAGDANLSGHTYPDQSLYPANSVPSVVRRINNALLRADEIAKVEGDDSVKNWLAPIVADAEAGFGGALNAYELQKAMIAAGAAGVHWEDQLASEKKCGHLGGKVLIPTQQHIRTLTSARLAADVADVPSVIIARTDAEAATLITSDVDERDREFLDGTRTAEGFFGVKNGIEPCIARAKAYAPYADLIWMETGVPDLEVARKFAEAVRGEFPDQLLAYNCSPSFNWKAHLDDATIAKFQRELGAMGFKFQFITLAGFHSLNYGMFDLAYGYAREGMTAFVDLQEREFKAASERGFTAIKHQREVGAGYFDTIATTVDPNTSTAALKGSTEEGQFH

>CORE_REP|Org56_Gene4749#

MSSVDVLSRSKGRLDSYFGIARLGSTMKRELMAGTVTFLAMSYVLAVNPAVLGDHGQLGSRGIPTQAVFTATAVAAVVGTLVMGVWARYPIALAPGMGLNAFFAYSVVLGMGIDWQVALSGTLLSGIIFFVLAVTKIREKIIDAIPLQLKLAVGAGIGMFVAFLGFKNAGIVVSDPATFVHLGDFTKGTTLLALFGLLVTVVFLVLGWHGAVLYGIVCTTVVGIVSGLVHLPHQVVALPHGLDQTFGQAIVNLPHAFTGQMAIVVLTMLFVDFFDASGTLIGIANQAGLLGPDGKLPRAAQALAADSIGTAAGAIIGTSTTTAYVESTAGVSAGGRTGLTAVSTAGWFLAAMFFFPIFAVVADVPAVTAPALIVVGVLMSRALGDIDWSKLEFAIPAFITVIMMPLTYSIANGIAMGLTFYPVVMVARRRGREVHPVMWVLMAVFLAYFFFLAE

>CORE_REP|Org5_Gene7373#

MRPASPRDSSAVARASVTGARETPAAGISEVEPPAHSSQPLRERTFSAPDSSELANLSAPGDPVREVLLLCWRDTGHPQGGGSERYLERVGAQLAARGVKVTLRTARYRGAARRERIDGIEISRAGGRFSVYPRALAAIAAGRMGFGPLRGLRPDAVIDTQNGIPFFARVVSGAPSVVLVHHGHREQWPVAGRLVGRIGWWIESWLSPRVHRNDQYLTVSLPSAEELASLGVDAARIAVVRNGAEPVPGQSPTGAEPIRTPHPSIVVLSRLVPHKQIEDALEVVAGLRGRLPGLELDVIGDGWWADNLKTRARELGIADAVNFHGFVDEPRKHELLSRAWVQVLPSRKEGWGLAVIEAAQHGVPTIGYRSSRGLTDSIVDGVTGVLVDDVFQLTETTGELLADPEMRVVMGEKARTRAREFSWEQTGFGVGSVVAAAARGEFVSGLVAGRSAE

>CORE_REP|Org5_Gene1113#

MSTIGRSPAGREDDAAEGDSAGTGTPISASRQTTTGLSGRTSGSAAGPEAGTCRHTGLATGRQTEVAADQAAVESWLREHGDDLIGWRRHIHANPELSRAEHATTEFVESWLVKADLEPRILPTGNGLICDIGPSGPRLALRADMDALPLQEYTGRPFASTVPGVSHACGHDAHTAILLGTALALAELDELPVGVRLVFQHAEEVMPGGAIDMVAAGAMDDVSRVFALHCDPRLEVGRIGVRVGAITSAADTVELVLDSPGGHTSRPHLTSDLVYAIGTVITGLPGLLSRRIDPRTSTVMVWGAVSAGKAPNAIPQTGMLTGTVRTGDHATWSLLEPMVREIVDGLLAPTGVRYQLNYKRGVPPVVNDEFCTRMFEDAILGLGPDALSDTPQSGGGEDFSWYLEEVPGAMARLGVWSGEGPQLDIHQPTFDIDERALAAGVRVLTNLVLQAR

>CORE_REP|Org101_Gene2174#

MVGAWCWTASPKLSAVRVLVIGSGAREHALVLALRRDPAVTGIVAAPGNAGIAQHAQTRPVDPCSAEAVVALATDVAAELVVIGPEVPLVLGVADAVRAAGIACFGPSAAAARIEGSKAFAKDVMAAAGVRTAHSEIVDNPADLDAALDRFGPTWVVKDDGLAAGKGVVVTADRSAARDHGAELLEQGHPVLLESFLDGPEVSLFCLVDGETVVPLLPAQDHKRVGDGDTGPNTGGMGAYTPLPWLSPDAVTTIIEDVVKPVAAELVRRGSGFSGLLYAGLAMGVAGPAVVEFNCRFGDPETQAVLALLESPLGELLAATANGTLAEVEPPRWRDGSAITVVVAAENYPGRPRIGDVISGAGDGAIDDTAAVLHAGTALREDGALISAGGRVLNVVGVGADLAEARTNAYARITAIKLPGSHYRTDIGLAAVEDRIAVPDRASASSGQTRES

>CORE_REP|Org101_Gene3287#

MLTLADMDRQKEFVLRTLEERDIRFVRLWFTDVLGYLKSVAIAPAELEGAFEEGIGFDGSAVEGFARVSEADMVARPDPSTFQVLPWSTSKGHQHSARMFCDITMPDGSPSWADPRHVLRRQLNKAGDVGFSCYVHPEIEFFLLENGPQDGSQPIPADSGGFFDQAVHDSAPNFRRHAIDALESMGISVEFSHHEGAPGQQEIDLRYADALSMADNVMTFRYLIKEVAIDEGVRATFMPKPFAQYPGSAMHTHMSLFEGEANAFHDPDDPINLSVTARAFIAGILEHAPEISAISNQWVNSYKRLIHGGEAPTAASWGRSNRSALVRVPMYTPNKSSSRRIEIRSPDSACNPYLTFAVLLAAGLRGIEKGYTLPPEAEDDVWSLTAAERRAMGFRELPGTLDEALQAMERSELVAETLGEHVFDFFLRNKRREWADYRSQVTPYELKEYLGL

>CORE_REP|Org113_Gene5852#

MGTSGSDGGTATKTTVLITVTGPDKPGVTSVLLAALSRHGVSLLDVEQVVIRGRLTLGVLVTSPGDPEELQDQLEEAMATVGMEVEVEIGANSVSGAPLSTHAVVVLGSPVTARAFSTIARTLAAQGANIDSIRGIADYPVTGLELMVTAPTTVPDDAATLTTPVAAGPDPSTASSGGSRGRAAASGPDPSIAETRLRTALAEVAAKENVDVAVERAGLARRAKRLIVFDVDSTLIQGEVIEMLAAHAGVEDEVRKVTEAAMRGEIDFAESLRQRVATLTGLDETVIDRVAERIELTAGARTTIRTLRRLGFRCGVVSGGFRQVIEPLAHELELDFVHANTLEVVDGKLTGKVIGEIVDRPGKAVALRRFAAEAGVPMEQTVAVGDGANDIDMLNAAGLGIAFQAKPALREVADTALSHPFLDAVLFILGVTRDEVEAADARDGLLRRVPLS

>CORE_REP|Org116_Gene5304#

MCRASLLVQPAVLELIVERVSVCRRRHLVVETRSARPNLPDGFDVTDPDIYAERVPVEEFAELRRTAPIWWNPQPPEVGGFHDDGFWVVSKHADIKEVSRRSDVFSNFENTAIPRFNDDISREQIELQRIVLLNMDAPEHTKLRKIISRGFTPRAINGLRAELSAKAEQIVKAAAAAGSGDFVTQVACELPLQAIAELIGIPQEDRMKVFNWSNQMTGYDDPDNDADPVTASAEVLGYAYQMAAARKACPADDIVTTLIEADVDGDKLTEEEFGFFVIMLAVAGNETTRNAISHGMIAFLENPDQWELYKKERPATAADEIIRWATPVTSFQRTALVDTELGGVQIKKGQRLVLLYRSANFDEDVFENPYKFDIMRADNPHLSFGGTGAHFCIGANLARLEIDLIFNAIADHLPDITRLGDPKRLRSGWLNGIKEFPVDYKTAARCPVSH

>CORE_REP|Org24_Gene3427#

MSGPLGHAVGLNLVQNTSAASHDRAGGDPNFAPRAGGDEPYLVGLDLNGRRVVVVGGGTVAQRRLGLLIASGADVHVITRATTPAVEGMATSGQLTLTLRDYVDGDLDGAWYALACTDEPDTNAAVVAEAERRRVFCVRADAARFGTAVTPATARYDGLTLGVLAGGEHRRSAAVRTALLEALQSGVVTDESEPTAPGVALIGGGPGDPDLITVRGRRLLARADLVVADRLAPPELLAELGPHVEVVDAAKIPYGRAMAQEAINDALIDGAKAGKFVVRLKGGDPYVFGRGFEELEACAAAGVPVTVVPGVTSAISVPALAGIPVTHRGVTHEFVVVSGHVAPDHPDSLVDWPALARLRGTIVLLMAVERIEQFAAALLAGGRAADTPVTVVQEGSLRTQRTLRAQLSTVAERVRAEGIRPPAIIVIGPTAGFTAGTPDAEAVSIDQGAS

>CORE_REP|Org57_Gene3374#

MRLVRRTGCTETGYRSSHRLAPVIRSSYDFSVTATLPETTASPAGTTARRRRALVEGRLLVLAAIIMSALVLRVAVTAFSPLAEEIGHEIGYGTAVVGVFGMIPTLMFSLSGLLTPLMVRRLGLERTALAAMLMAGLGMLIRVLVSGTTELFVFSALALGGMGIGNVVIPPLVKRYFPDRLAIVSALYITMVQIGTVLPALVAVPVAEAHGWRISLGMWALLGFAAAVPWFGVLRDRRGRDTADTTALPADGHTTGKAWRSPVAWGMAGMFGMTSLTTYSMFTWLPTIFADAGASAAFGGTMVALFAVVGLIAALTAPTVAARMTNPFPVVIGCAVCFFVAFTALLIAPMSAPILWVIVLGLGPSTFPMALTLINLRTRTPAGSASLSGFTQGVGYAVACAGPVLFGMLHTATGGWAAPFAFLGVAVLVLLAGAWQACKPRMLEDTWSAR

>CORE_REP|Org16_Gene5537#

MKVAQTPSDANGAGDAKPVLSYPGGEYAMTVAQAVEGNDGIDLGKLLASTGYVTYDPGFTNTAPTKSAITYIDGEAGILRYRGYPIEQLAASSNFIEVSYLLIYGELPTQAQLEDFTDRIRRHTLLHEDLKRFFDGFPRNAHPMPVLSSAVNALSAYYQDSLDPRDPEQVELSTIRLLAKLPTIAAYSYKKSVGQPFLYPDNSLSLVENFLRMTFGFPAEPYEVDPEVAAALDMLLILHADHEQNCSTSTVRLVGSSDANLFTSVSGGINALWGPLHGGANQAVLEMLDDIKANINGGTVDAAVKDFIRKVKNKEDGVKLMGFGHRVYRNYDPRATIVKKTADQILGKLGVQDPLLDIAKALEEAALTDSYFVDRRLYPNVDFYTGVIYRAMGFPTRMFTVLFAMGRLPGWIAHWREMHSEPLKIGRPRQIYTGYGARDYGDIAGR

>CORE_REP|Org163_Gene2588#

MTTRIELARVDLRGRTPSVAELRAALPRGGVDVDSVLHQVRPVVEAIRDQGVSAALEFSERFDGVIPPTVRVPAAELEGALERLDPAVRAALEESIARARKVHADQRRTDKTTEVVPGGTVTERWVPVERVGLYVPGGNAVYPSSVVMNVVPAQTAGVGSLVVASPPQAQFGGLPHPTILAAAQLLGVDEVWAVGGAQGVALLSYGGVDTDGAQLEPVDLITGPGNIYVTAAKRLCRGLVGIDAEAGPTEIAILADATADPVHVAADLISQAEHDVLAASVLVTDSAQLADAVDAALTAQLTVVKHAHRVGEALRGKQSGTVLVDDIEQGLRVVNAYAAEHLEIQTTDAPAVAARVRSAGAVFVGAYAPVSLGDYCAGSNHVLPTAGCARHSSGLSVQTFLRGIHVVEYTEAALKDVAGHVVALANAEDLPAHGQAVQARFEALS

>CORE_REP|Org162_Gene4624#

MPCDLPSKQPKECRFVAIIEQVGAREILDSRGNPTVEVEIALDDGTLTRAAVPSGASTGEHEAVELRDGGDRYQGKGVQKAVEGVLDEIAPAVIGLDAVEQRTVDQTLLDLDGTPDKSRLGANALLGVSLAVARAAAESSGLELFRYVGGPNAHVLPVPMMNIVNGGAHADTGVDVQEFMIAPIGAPTFKESLRWGAEVYHSLKSVLKSQGLSTGLGDEGGFAPDVAGTRAALDLIASAIEKAGYKLGTDVALALDVAATEFYTAGEGYKFEGSVRSAAQMNEFYSELLSAYPIVSIEDPLSEDDWDGWVALTDAIGDKVQLVGDDLFVTNPERLEEGIAKGAANALLVKVNQIGTLTETLDAVDLAHRNGYKTMMSHRSGETEDTTIADLAVAVGSGQIKTGAPARSERVAKYNQLLRIEDALGDSARYAGDVAFPRFAFEG

>CORE_REP|Org151_Gene2264#

MTDPTVSAVDPSQWSFETKQVHAGQAPDATTGARALPIYQTTSYAFRDTDHAAALFGLAEPGNIYTRIMNPTQDVVEQRVAALEGGVAALLLASGQAAETYAILNLAAAGDHIVSSPHLYGGTYNLFHYTLPKLGIEVSFVDDPDDLEQWRAAIRPNTKAFYGETIANPSSAIFDIPGIAAVAHAAGLPLLVDNTVATPYLIQPLAHGADIVVHSATKYLGGHGSAIAGVIVDGGTFDWTVTDAQGQSRYPGFTTPDPSYHGAVFADLGAPAFALKARVQLLRDLGAAVSPFNAFLISQGLETLSLRVERHVANATAVAEFLRTHPDVISVSYAGLPTSPWYERAKQLAPKGAGAIVAFELRGGVDAGKKFVDGLVLHSHVANIGDVRSLVIHPASTTHSQLTPDEQLRAGVTAGLVRLAVGIEGIDDILADLRAGFTAAAT

>CORE_REP|Org105_Gene2713#

MINRGVVRMRNTKAVQAAEPEFVEVVIVGSGFGGLAAAKQLAKSGVPYVLISSTPEHLFQPLLYQVATGVLAADEIAPPIASILRRHEKADVRLGKVTAIDPDAAELVYETADGPRRIRYGSLIAATGANQSYFGRDDFAEKTFALKTIDDAKRLRAQIDHVFTQAKHADKETRERLLSFVVVGAGATGVEVAGQLAELAKRYYHQDVSVTLVEGAGEVLPPFGGGLSEYAKQSLTKGGVEVLLGTFVTDIEPGKVTVKDKQGVEHRIAAETVVWSAGVQASGFTKILAEATGAETDRAGRLLINPDLTVGGYADIYAIGDMTSLKGYPGQSPVAMQEGRHAADIIRRKKLPGTEFEYWDKGSMAVIRRRSAIAKVSDKIKFKGLIAWYMWLAVHLFYLVGFRNRFMAVMGWLVAFTGNGRPGFAEIDKDRPAVGHKPPIAA

>CORE_REP|Org1_Gene899#

MRDEPRSRPVSRSSRRCASDEGASIGDSTARHQGAANPPRRRSGKSNTERTTHVRDMVEIGMGRTARRTYELDDVDIVPSRRTRSSKQVSLAWQLDAYRFEIPLVAHPTDALVSPRSAVELGRLGGLGVINGEGLWARHADVETKIEQLTELAAQGRFDRAVALLQQLHAAPMQPDLLAAAVAEVRAAGVTVAVRVSPQNARTLTPALLQAGIDLLVVHGTIISAEHVGDGEPLNLKTFIAELDVPVVAGGVSDHRTALHLMRTGAAGVIVGYGSYPGATTTGEVLGIGVPMATAIADAAAARRDYLDETGGRYVHVIADGDIATSGQLAKAIACGADAAMLGVPLAVAAEAPGRGWYWPSAAAHPSVPRGSLLQVGDGWDLGAEDEADEAARPPLERVLFGPSDDPFGSLNLVGGLRRSMAKAGYSDLKEFQKVGLSVRA

>CORE_REP|Org2_Gene7412#

MAAANYGLSMGADAVPARHGEGSDADGLDPDGPGSRLTVTSEVGTLRTVLLHRPGDELRRLTPRNNDQLLFDGIPWVERAQQEHDAFTGVLRERGVEVLLLADLLAETLAVSGAARIQGISAAVDARRLGHSLADQLAAFLRGVRARDLANILMAGMTFDELPFGPDATSLVRRMHHGADFVIDPLPNLLFTRDSSFWVGPRVAITSLALPARIRETSLTDLIYAFHPRFLGVRRAYESHTAPIEGGDVLLLGPGVVAVGVGERTTPAGAEALARSLFDDDLAHTVLVVPIAQNRATMHLDTVCTMVDQDALVMYPAVRDSLCAFTIERDEDYSARNGDGRVSMSGPDPFLVAAAKAMGIDKLRVIDTGLDGVTAEREQWDDGNNTLALAPGVVAAYERNEMTNARLEDAGIEVLRIPGSELGSGRGGPRCLSCPLSRDDL

>CORE_REP|Org101_Gene6844#

MADPALRTDLDVPLHEFDPLVAELVGRELGRQQHGLEMIASENYAPLAVMQAQGTVLTNKYAEGYPGRRYYGGCEHVDELESLALTRLRALFGAEYANVQPHSGAQANAAVMHALLRPGDRILGLALDHGGHLTHGMKINFSGRLYDVAAYHVRAEDQLIDMAEVARLAREHRPKLIVAGWSAYPRHLDFAEFRRIADEVGAYLMVDMAHFAGLVAAGLHPSPVPHAHVVTSTTHKTLGGPRGGFILATAELGKKLDSAVFPGQQGGPLEHVIAAKAVAFKMAAEPAFRDRQERTLTGARLLADRLLAEDCRAAGIGLVSGGTDVHLVLVDLRAAELDGKQAEDLLHSVGITVNRNAVPFDPRPPIVSSGLRIGTPALAARGFDRAAFVEVADIIATALRVGRPWREFSVRVEVLTQKFPLYAGMRQHLPSATDAARELA

>CORE_REP|Org103_Gene5459#

MACDLRGTSRTRRWSRGRRPYDSGVSVPQAVLLAVLAAVVGLAVGGLLIPYVNARQAARRQADSGLTMSQVLDLIVLASESGIAVVDQYRDVVLVNPRAEELGLVRNRLLDERAWAAVEKVLATGESAEFDLTAKNPLPGRSRIAVRGVARPLSQEETGFTVLFADDDSEQARMEATRRDFVANVSHELKTPVGAMSLLAEALLESADDPEAVRHFGQRVLGESRRLGKMVTELIALSRLQGAEKLPELEVVDVDTVVMQAVDRSRTAAEAAGITVSTDRPSGLEVLGDETLLVTALSNLVENAIAYSPPGSHVSVSRSLRGKYVAMAVTDRGIGIAKEDQERVFERFFRSDKARSRATGGTGLGLAIVKHVAANHNGEITLWSKLGTGSTFTLRIPAHLEADSGDDDVDADGAAVSTKENGSRPSGPGRPNGVEARR

>CORE_REP|Org209_Gene2601#

MITAETNVFESLESNVRGYCRNWPTVFTTAKGAWLQDEDGKDYLDFFAGAGALNYGHNNPVLKQPLIDYIASDGITHGLDMSTAAKRKLLETLRDTVFAPRGLDYKVQFPGPTGANAVEAALKLARKVTGRETVLSFTNAFHGMTLGALSVTGNAAKRAGAGVPLVHAAHMPYDGYFDNTTADFQWMERVLDDTSSGFDRPAAVIVETVQGEGGINVARVEWLQHLAQLCAEREILLIVDDVQMGCGRTGPFFSFEVAGITPDIVTLSKSIGGYGLPLALVLFKPELDQWAPGEHNGTFRGNNPAFVTAQVALETFWSDGALEAATKAKGEKVATELATVAGHFPGLSTRGRGLVHGIAFEDPSQAGKVCQVAFERGLLVETSGSSDEVVKLLPPLTITDDELDQGLQILTGAIDTVCTGWGRLHHRAPAEGGDRR

>CORE_REP|Org94_Gene5589#

MRLRYWFRWLSAQGAPRLVLRTQARRGDPFARLVGGREGIEDPYPLIEQLRGDGGPVRTPLSWAAFDHELCRAILRDNRFGVRSPQSFTAFEPLKRLAARSPLPPNPVEPPSMLVIDPPEHTAMRKPVAAAFTPRAIGRLRDRVASVTTELLDALPSHGSVDLVAAYASQVPIAIISEMLGFPDADRQMFLGWGDRMTPLLDIGIPWRAHKRALLAMEVMNDYLDRHIARLRREPGDDILSALVTAGDLDDHELKASASLLMGAGFETTVNLIGNGVVQLLAHPDQLARLREEPDLWPNAVEEILRIDSPVQSTARTALTDVELDGALLRRGHTVVLSLAGANRDPKVFADPERFDVARPNAKDHLSFSSGIHVCLGASLARMEGVYALRALFERFPDLALAEPPHRRALFTLHGYERMPVHLGKRAAAREMSPLS

>CORE_REP|Org15_Gene2657#

MPAIVLIGAQWGDEGKGKATDLLGGRVQWVVRYQGGNNAGHTVVLPNGDNFALHLIPSGILTPGVTNVIGNGVVIDPGVLLDELAGLEQRSVDTSRLLLSADAHLIMPYHVAIDKVTERFLGNKKIGTTGRGIGPCYQDKVARVGVRVADVLDEKILTQKVEAALEFKNQVLVKIYNRRALDPQQVVDEVLNQAEGFKHRISDTRLLLNQALENGETVLLEGSQGTLLDVDHGTYPYVTSSNPTSGGAAVGAGVGPNKITTVLGILKAYTTRVGSGPFPTELFDQSGEYLAKTGGEVGVTTGRARRTGWFDAVIARYATRVNGITDYFLTKLDVLSSLDRVPICVAYEIDGERVEQMPTTQTEFHHAKPIYEEMPGWWEDISGARSFDDLPANARAYVERLEELSGARVSCIGVGPGRDETIVRHDILNEQLSSR

>CORE_REP|Org129_Gene4082#

MWDFRTEPDFQAKLDWMDTFVREECEPLDLLFPHIGQPYNTENAAARAILKPLQDRVREQGLWACHLGPDLGGQGYGQVKLALMNEILGRSMWAPTVFGTAAPDTGNAEILAMFGTAEQKSRYLQPLLDGDIVSCFSMTEPQAGADPKEFVCAARRDGDEWVISGEKWFSSNARYAAFFIVMAVTDQDASPYRRMSMFVVPAETPGIEIIRNVVVMPDREELDEGTHGYIRYNDVRVPADAILGGAGQGFEVAQARLGGGRVHHAMRTVGKCRRAFDMMAERVLSRHTQGELLADKQMVQQFIADSWIELAQFRLLVLQTAWIIDNEPHGTARTDIAMCKVAMAKIFADIISRAVQIHGSLGVTAELPLYEWWTSVPSLALADGPTEVHKATVAKQVLKGYRPAAGLFPSEHIPTRREAARARYADILKEHGLA

>CORE_REP|Org24_Gene2681#

MTATGHDSRLAGPHDSPLAEVAALDVARIRADFPILSRTVRDGKPLVYLDSGATAQRPTAVLDAERDFLVQRNAAVHRGAHQLAEEATDAYEGARADIARFVGVDADEIVFTKNATESLNLVTYSFADNRFPYRVGPGDEIVITELEHHANLVPWQELARRTGATLKWYGVTDDGRIDLDSLELSPATKVVAFTHQSNVTGAVAPVEELVRRAKAVGALVVLDACQSVPHMPVNFRELGIDYAAFSGHKMLGPSGVGVLYGRREILADTPPFITGGSMIETVFMEESTYAPPPQRFEAGVPMTSQVVGLGAAVRYLDAVGMEAVAAHEHALTEAALLGLGKLDGVRIIGPTENVNRGGAVAFVVDGVHAHDVGQILDDEGVAIRVGHHCAWPLHRRFGVAATARASFAVYNTLDEVDTLVAAVRKAQTFFGVA

>CORE_REP|Org100_Gene1857#

MDKLPGVSERFLVTGGNRLVGEVAVGGAKNSVLKLMAAALLAEGTTTITNCPDILDVPLMAEVLRGLGCEVTITDDAPGDRSVVTITTPAEPKYHADFPAVTQFRASVCVLGPLMARCKRAVVALPGGDAIGSRPLDMHQAGLRLLGATSEIEHGCVVARAEELRGARIRLDFPSVGATENILMAAVLAEGETVIDNAAREPDIVDLCNMLVQMGARISGAGTSVLTIQGVERLHPTEHRVIGDRIVAATWGIAAAMTMGDVRVTGVNPKHLALVLDKLRSAGARISFDVDGFRVVQPDRPRAVNFSTLPFPGFPTDLQPMAIGLAAIADGTSMITENIFEARFRFVEEMIRLGADARTDGHHAVVRGIPRLSSAPVWSSDIRAGAGLVLAGLVADGTTEVHDVFHIDRGYPNFVEQLQSLGGLVERVGGAE

>CORE_REP|Org185_Gene4863#

MLARIRHAADVAANTQVECVGPDRHGHTGAVSSPASTASHTDASVPVLRDFGGGPFGIYVHVPFCATRCGYCDFNTYTAGELGSSSSPQSWMTALRGELATAARQFAALPSATPEVATIFVGGGTPSLLGGDGLAEVLDAVRAEFTLAADAEITTESNPESTSPAFFERIRSAGYTRVSLGMQSAAQHVLAVLDRTHTPGRAVAAAKEARAAGFEHVNLDLIYGTPGERDSDLDASIDAVLEAGVDHVSAYSLIVEDGTALSRRVRRGELPAPDDDVLAARYERLDARLSAAGLTWYEVSNWAASDAARCRHNLGYWDGGDWLGAGPGAHSHLGGVRWWNVKHPARYADCVAEGGLPAAGWESLTDDERYLERIMLTVRLRTGLPMSDLHPGGKAKAMQIIADGRAALRDDHLVLTEQGRLLADGVVRDLVS

>CORE_REP|Org16_Gene2197#

MTGEKHTLATGDTQNAPENDRLIAVVGIGADGWPGLSPRVRDEIAAAQVLFGSRRQLDLIPADASIAQRRAWPTPLLPALPELLAAHRGSRICVLASGDPMFYGIGVTLANLLGPQAIRVYPQPSSATLACARLGWASAHTPVVSIVGRPLETVLPALADGRRLLVLSADEHSPAQVAELLRCNGFGESRLTVLEQLGGPAERVVAATAAQWSRPPGDPLNIVAVEAVRDPAAPRLTRLPGLPDASYGGDGQLTKAEVRTLSIAALAPAPGELLWDVGGGSGTIAIEWCRTHPDCRAITFERSAARRDQIAANAAALGVPAIVVRGEAPADLPAAGDPAPDAIFLGGGLTQDGLFATCWDRLRPGGRLVANAVTAESEALLLRWAATHGGELRKFQIYRGEPLGGFTAWRPHLPVAQWIAVKPADRSVSPE

>CORE_REP|Org49_Gene2684#

MPRQTQIGLMSHAELVSEHETQDANYAKLKTEKLTLDLTRGKPAPEQLDLSADLLSLPGADDYRDASGTDVRNYGGLHGLPELRAIFGELLNIPVENLLAGNNASLEIMHDMVVFAMLHGTADSERPWVQEPVRKFLCPAPGYDRHFAITQSLGFEMIPIPMRHDGPDVHAIAELVAADPTVKGLWAVPNYSNPTGVTFSEEVVRELVSMPTAAADFRLFWDNAYAVHPLTDTADPVLDVLGMAAAAGNPNRPFVFASTSKITFAGAGVSFVGASTANLGWYLKHAAKQSIGPDKVNQLRHLRFFKDAEGVRTHMQKHRAILEPKFALVLRILEDRLGASKVASWTEPKGGYFISLDVLEGTASRVVALAKDAGIALTPAGASFPYGRDPDDKNIRIAPSFPKESELEKAMDGLATCVLLAATEKLLADGK

>CORE_REP|Org176_Gene7174#

MPEARAAGPVTGVAALPRASDGVAQRSGHEPDAGGHFGVYGGRHVPEALMAVIEEVTAEYEKSRLDDSFLNELDRLQRDYTGRPSPVFECTRLAEHAGGARILLKREDLNHTGSHKINNVLGQALLAKRMGKARVIAETGAGQHGVATATACALLGLECVVYMGAVDTARQALNVARMRLLGAEVVSVTSGSQTLKDAINEALRDWVTNAEDTYYCFGTAAGPHPFPMLVRDFQRIVGMEARAQVQASTGRLPDAVVACVGGGSNAIGIFHAFLDDADVRLIGYEAAGDGVDTGRHAATFTGGTPGAFQGAYSYLLQDEDGQTIESHSISAGLDYPGVGPEHAYLKDVGRAEYRPITDTEAMDALLLLSRSEGIIPAIESAHAVAGALQLGKELGPDAIILVNLSGRGDKDMDTAARWFGLFDTEPQEADQ

>CORE_REP|Org10_Gene4638#

MSASVKPRLAGRQPSSCGGVLRVMTGLPKVGGKRARRRFSRHDRVVRVTVSTDQSPCPSATGAELLPPPDGTLAIVPVGDIRLESGAVIPDVHLGVQRWGELSPGLDNVVLVEHALTGDSHVVGPADDVHQLPGWWNGMVGPGAPMDTDEWCVIATNVLGGCKGSTGPGSTAPDGKPWGSRFPAISIRDQVTAEAALFDRIGIHRLAAVVGGSMGGMRVLEWMVGAPERVAAALVLAVGARATADQIGTQTTQIAAITADPDWQGGDYHDTGRAPTTGMGIARRIAHLTYRTEDELDHRFANHAQDGEDPFDGGRWAVQSYLEHQAEKLCRRFDPATYVLLTEAMNRHDVGRGRGGVAAALAATPVPCVVGGVDSDRLYPLHTQQELADLLPGCARLEVVHSRDGHDGFLTETAAIGKLLVETMRLARAHR

>CORE_REP|Org12_Gene1520#

MFEWSETDEMIRAAVRAFIDKEIRPNLDALDSGAMPPYPILRKLFGEFGIDVMGAEAIEKLLAKQRAAEAAPDAAQRDKKLRSGGDPFGEQQSLMAVLISELSGVSMGLVAAMGVSIGLGAATIMSRGTLAQKERWLADIVTLKKIAAWAITEPDSGSDAFGGMKTSVKRDGEDYILNGQKTFITNGPYADVVVVYAKLDEGDGGAGGRSAGSAPIDKRDRKVLTFVLDKGMEGFTQGKPFKKMGLHSSPTGELFFDNVRVGKDRLLGETEEHKGGDGRESARTSFVAERVGVGFMALGIINECHRLCVEYAKTRVLWGQEIGRFQLVQLKLAKMEIARINVQNMVFNTLERGRAGKPPTLAEASAIKLYCSETATEVAMEAVQLFGGNGYMQEYRVEQLARDAKSLMIYAGSNEIQVTHIAKGLLGR

>CORE_REP|Org78_Gene5482#

MALTPDQITAIDAAHVWHPYGGFPATTEPLVVASASGVRLTLADGRELVDGMSSWWAAVHGYRHPVLDAALVAQSQRMSHVMFGGLTHEPAARLTELLVQLTPEGLDKVFLCDSGSVSVEVAVKMCLQYWRSLGKPGKRRLLTWRGGYHGDTFTPMSVCDPEGGMHALWTDALAEQVFVGMPPAEYRPGYVAELEAALAAHADELAAVVVEPVVQGAGGMRFHDPRYLADLRRLCDAHDVLLVFDEIATGFGRTGELFAAEHAGVRPDVMCVGKALTGGYLTLAAALCTTRIAETISAAHGGLMHGPTFMGNPLACAVAVASVELLLARDWRGEVRGIEDGLRAGLAPVRDLPGVVDVRVLGAIGVVELDRPVDMRAATAAAVAAGVWLRPFRNLVYTMPPFISTAADVAAITRGIAAAVAAGDVPS

>CORE_REP|Org39_Gene1705#

MARIGDGGDLLKCSFCGKSQKQVKKLIAGPGVYICDECIDLCNEIIEEELAESSEVKLDELPKPTEIRDFLEQYVIGQDAAKRNLAVAVYNHYKRIQAGDKGRDPRGEPVELAKSNILMLGPTGCGKTYLAQTLAKMLNVPFAIADATALTEAGYVGEDVENILLKLIQAADYDVKRAETGIIYIDEVDKIARKSENPSITRDVSGEGVQQALLKILEGTQASVPPQGGRKHPHQEFIQIDTTNVLFIVAGAFAGLEKIVQDRIGKRGIGFGAEVRSKAEVDTTDHFAEVMPEDLIKFGLIPEFIGRLPVVASVTNLDKESLVRILAEPKNALVKQYVRLFEMDGVDLEFTQDALEAVADQAILRGTGARGLRAIMEEVLLPTMYDIPSRDDVAKVVVTADTVNDNVLPTIVPRKRQQGPERREKSA

>CORE_REP|Org118_Gene827#

MQLRGGGTIEQVSPTSQSPHQPPRILEQSLKLQNVLYEIRGPVHAHAARLEAEGHRILKLNIGNPAPFGFDAPDVIMRDMIAALPYAQGYSESKGILPARRAIVTRYELVPGFPEFDVDDVYLGNGVSELITMTMQALLDSGDEVLIPAPDYPLWTAMTSLAGGTAVHYLCDESNGWQPDVADIESKITDKTKALLVINPNNPTGAVYSSEVLQQLVDLARKHQLLLLADEIYDKILYDDTKHISLASLAPDLLCLTFNGLSKAYRVAGYRSGWLVITGPKEHAAGFLEGIDLLASTRLCPNVPAQHAIQVALGGYQSIEDLILPGGRLLEQRDVAWEKLNMIPGVSCVKPKGALYAFPRLDPEVHDIHDDGKLVLDLLLQEKILMVQGTGFNWPQHDHLRIVTLPWARDLAVAIERFGNFLSSYRQ

>CORE_REP|Org5_Gene1930#

MSEAKNTGPRRDTETLSHLDDEGRARMVDVSAKAKTARTAVAAGVLRTTPEVVALVRADDMPKADVLATARIAGIAGAKKTSELIPLCHQLALSSVHVRFDFTDDAITIEARAKTKGPTGVEMEALTAVAIAGLTLHDMVKAVDPAAVLDGVRLLTKDGGKHGHWERLGEPVDHATSGRSSHAHDHTRPGQAQTAAPSPGASADTPAGHGPSGRPHDSAHGAMEPTPAPLATAAHSGRPSEPVDDAAAHSGHAHGRGPGTHSGVARAGEGSRSAVVVVASTGAAAGTRVDTTGPVLMDWLAGLGFSVRGPLVYADAEIAAGLRDALEGAPGLVITTGGTGAAPSDATPEATLAVLDRELPGVAESIRQRGTAAFPLAALSRGVAGLSGATVIVNLPGSPGGVRDGIAVLEPLLDHLLAQVAGGGRHE

>CORE_REP|Org217_Gene187#

MVAEHGEVDLAAARPDSGPTAAAAPAGNGSGATANRSGGEAGVRRRPKDRKAQIVRAAARAFSERGYYPVGVDEIAAEVGISGPALYRHFANKYALLVAAAEEGARHLLQVAQAADDPALDPEPRLDAVIKAISEHTIDIRREAGLYRWERRYLEREDRLRIRRIYDELNDTIAAPIARLRPGADPADLRMLSAAVMSAVASIAAHRTALSGARLLPLLRDMCWAILRTELPPAPVDTEDEPAPRGLPVTSKREQLLTEAIRIFGRQGYHEASIEEIGAAVGINASSVYRYFSSKADLLAAAFHRTGDRVSVAITEALAEATSRPDAVRRIAARQAKLTFAMPEIMPVYYAEFSNLPQAEQHKLRAIQRQNVLEWANLLDGDPIEARFRVHAAIGQVIDVGRLIRFDSRPAQLARVTALMEAVLLG

>CORE_REP|Org105_Gene2202#

MPLITMDRFPGRERLHGKMDGVLRWITAGESHGPALVAILDGMVAGVEVTSDEISAQLARRRLGYGRGARMKFEADKVTIVGGVRHGRTMGGPVAIEVANSEWPKWTTVMSADPVDPAELADLARNAPLTRPRPGHADYSGMLKYGFDDARPVLERASARETAARVAAGTVARAFLRQAFGVEVVSHVISIGTAANTTGHVPTAADLDAIDASPVRAFDAEAEAAMIAEIEAAKKDGDTLGGVVEVVVEGLPVGLGSFTSGENRLDSRLAAALMGIQAIKGVEVGDGFETARRRGSQAHDEMKPGPDGVLRSTNRAGGLEGGMTNGEALRVRAAMKPISTVPRALSTVDMSSGEEAVAIHQRSDVCAVPAAGVVAESMVALVLAQAALEKFGGDSLTETCDNITSYVKRISSRPHVAPTDADSRAR

>CORE_REP|Org7_Gene3766#

MRERGGVVQNYSEPDYERLVVERPKTAGLGAPGSEFEVGHRTEFARDRARVLHSAALRRLADKTQVMGPRDGDTPRTRLTHSLEVAQIGRSIGEGLGCDPDLVDLAGLAHDIGHPPYGHNGEKALDHFADAHGGFEGNAQNLRILTRLEPKVLDPAGVSAGLNLTRASLDAALKYPWGRTGPGTKFGAYDIDAERLAWIRKGAPERRRSLECQIMDWADDVAYSVHDVEDGVIAGRIDLRALADPWEQEALASLGRHKHYSLSAEELVAAAQRLSELPVVAAVPAYDGTLASSVALKRLTSELVGRFATGAITATRETAGTGPLSRYGADLEVPLIAAAEVAVLKTVALHYVMSDRDHKLRQAGQRDQIQAVATRLLATAPNGLDPLLLPWWHAAADDTARVRVIVDQIASYTESRLERVAALLGV

>CORE_REP|Org66_Gene1451#

MLVTSTDSVSGANLAKDKPEPASGTLERDVQTLEKAIYEVKRVIVGQDRLVERLLVGVLARGHVLLEGVPGIAKTLAVETFARVVGGSFSRVQFTPDLVPTDLIGTRIYRQGREEFDTELGPVVANFVLADEINRAPAKVQSALLEVMAERHVSIGGKTYPMPDPFLVMATQNPIESEGVYPLPEAQRDRFLFKVVVDYPSVEEEREIIYRMGVTPPEAKQILGPEDLIRLQKVAANTFVHHALVDYVVRVIAATRKPLDYGMADVANWIAYGASPRASLGIIAAARAVALIRGRDYVVPQDVVEVIPDVLRHRLVLSYDALADEVSPEDVIRRVLQTVGLPQVAPQAVPAGAQAAPAGPPQQHQQQIPQPPAQPGQGQQGQGQPGQGQQGQGQPGQGQPGQQGQGAPNQGAQAPMAPAGTNQPK

>CORE_REP|Org102_Gene2841#

MPAIPGRQPAGTGPRGTMTILSNVSARSFDSSAMPTVTMIGGGQLARMTHQAAVALGQRLRVLAERPDDPAAQVTPEVVLGTHTDLAALRKAAVGSHAVTFDHEHVPTEHLEALIAEGVNVQPPPGALVYAQDKLAMRRKLAELGVPVPVFTAVASAADAVAFGDEHGWPVVLKAVRGGYDGRGVWMPADAAEATRIADDQLAHGVALLAEAKVDLKRELSAMVARSPFGQAAVWPVVETVQRNGQCAVVIAPAPELPEQRATEASALALRLASELGTTGAMAVELFETHAGELLVNELAMRPHNSGHWSMDGAVTGQFEQHLRAVLDYPLGDTAPLAPVTVMANILGAPEAPAMSMDERLHHLFARMPDARVHLYGKGERPDRKIGHINILGDDVAATREQAERAAHWMSHAVWTDGWDPHHE

>CORE_REP|Org144_Gene454#

MADISVRGRIALRAAAAASWASQKAGRGKGSMIGGLIALQIDKTIMDQLGRGKRTVLITGTNGKSTTTRMTTAALGTLGAVATQADGANMDAGIVAALSVHRGAPLAAIEVDELHLPHVTDSLNPAAVVLLNLSRDQLDRVGEINMIERKLRAGLARHPATVVIANCDDVLVTSIAYDHPNVVWVAAGSGWSMDATSCPRSGEPIVWEDAPAGARGGEAGKHWRSTGADFARPEPDWWLEGNDLVGPDGVRLPLELALPGRANRGNAAQAVAAAVALGAGAADAVTATGTVREIAGRYRTVQVGDHAARLLLAKNPAGWQEALSMIEPAAAGLVIAVNGQVPDGEDLSWLWDVRFEHFEGVQVVASGERATDLAVRLTYAGVEHTTVSNPVRAIASCPAGHVEVLANYTAFRDLNRDLDGRTA

>CORE_REP|Org14_Gene597#

MTAGTVGEVDTVREAVHEAARRARVASRTLAQLTTAQKDAALHAAADALLAAKDAVLAANAEDIAIAEAGGTAASLLDRLRLTEPRIDGIASGLRQVAGLPDPVGEVLRGSTLANGLEIRQVRVPLGVVGMVYEARPNVTVDAFGLALKSGNAALLRGSSSAARSNAALVEVMREALVAQGLAADAVQLLPSEDRSSVTHLIQARGLVDVVIPRGGAGLINAVVRDARVPTIETGTGNCHVYVHAAADLEMAESILLNSKTRRPSVCNTAETVLIDRAIAETAVPRLIDALERAQVTIHGDLPGLVPATEEDWADEYLSLDIALKVVDGLDAAVDHINEWGTGHTEAIVTADLKAAREFTARVDAAAVMVNASTAFTDGEQFGFGAEIGISTQKLHARGPMALPELTSTKWIVWGDGQIRPS

>CORE_REP|Org119_Gene4741#

MHELVGALRSYAWGSRTALAQLCGRPVPSAHPEAELWFGAHPADPAHVRIADHTTSLLDFVAADPIRELGPAAAEFGGKLPFLLKILAAEEPLSLQAHPSAAQARAGFERENRTNVALDSPMRNYRDDNHKPELVVALDRFEALAGFRNPRRTVELLRALQVPGLESYANLLAAQPDSDGLRTLFTTWITLPQPVLATLLPAVLDGCVRYLSGKGKREFTAEARTALELAEAYPGDAGVLAALLLNRLTLEPGQGLFLAAGNLHAYLRGLGVEIMANSDNVLRGGLTPKHVDVPELLRVLDFEPIDLPVVLPEPAGDGSVRYATPAPEFALRRFDLVAGSGQVPLTAAGPGIVLCTAGSVRLLQGTTELALQRGAAAWISATDTDIRAQAPDGDAQLFCACVGGVGAAGLPATLTGTDQHRG

>CORE_REP|Org109_Gene6011#

MFVRTLSVRDFRSWEYAELELSPGRTVFLGSNGNGKTNLLEAIGYLATLGSHRVATEAPLIRTGTERARIGATVVNAGRELRIDVELNQGSPNRAQINRSPVRRTREILGILQTVLFAPEDLALVRGDPGERRRFMDELCTTRLPRLAAVRGDYDRVLRQRSALLKTAGRQARSKADLSTLDVWDGHLAEHAAVLLAQRLRLVHDLAPYLARSYASIAPESRPASIVYRSAALPPEFLDPARPPRPEDTGELEAIVLRELAAARPKELERGVCLVGPHRDELDLMLGDSPAKGFASHGESWSFALALRLGAFELLRATGAEPVLLLDDVFAELDRRRRAALAAVAADAEQVLITAAVPEDVPAELAAVPLRVETAGGPDSRVSRIVAPGWSESGEPGNDDAEYPPLGREGDPLTGAPESRTP

>CORE_REP|Org132_Gene1794#

MPVENVAVAGEARIPAVNKGELFASFDVAAFEVPSAHDEAWRFTPLRRLRGLHDGTAVRDGRAGIEVAVSDGDARGASAVTQAAASGPDASTAAVDGSVIDGVTVETVGRDDARLGEGGVPTDRVAAQAYSGFEQATVVSVGAETEVDRPVVVRVTGPGADKTAFGHLQIRLGNFAAATVVIDQRGSGTYAENVEFVLGDSAKLTVVAVQDWDDDAVHVTAHHAKLGRDAVLRHTDVTLGGDLVRLTATVRYDGPGGDAELLGLYFADDGQHFEQRLLVDHAQPHCKSNVLYKGALQGDPSSAKPDAHTVWVGDVLIRAEAEGTDTYEANRNLVLTDGARADSVPNLEIETGEILGAGHASATGRFDDEQLFYLRARGIPEEAARRLVVRGFFHEIIQKIAVVEIRERLESAIETELAAIGA

>CORE_REP|Org25_Gene3927#

MKILMVSWEYPPVVVGGLGRHVHHLAIELAAAGHEVVVLARRPTGTDPSTHPTHSYIADGVLVVAVAEDPPFFDFGEDMLAWTLAMGHAMVRAGVALGKPGIGDGWTPDVVHAHDWLVAHPAIALAEYYDVPLVSTIHATEAGRHSGWVSGRVNRQVHSVEWWLANESDALITCSASMQDEVERLYGPDRIPLTVIRNGIDVGAWTFRPRPPRSGPPRLLYVGRLEYEKGVQDAIAALPRIRRAHPGTTLTIAGVGTQFEWLRERARGHRVARAVTFAGQLDHTELLGWLHGADAIVLPSRYEPFGIVALEAAAAGTPLVTSTAGGLGEAVIDGVTGASFEPADVDGLVQTVRAVLDDPAAAQERAYAARERLTADFAWDVVAAETAQVYTAAKRRVRSPLGRPVITMRPLPERDPGQPV

>CORE_REP|Org216_Gene4894#

MIDLRLLREDPNAVRASQRARGEDPALVDALLEADAARRAAVATADNLRAEQKAMSKQIGKAPKEERSALLARAQELSVKVKEAEAAQHAADADLDAAHRALSNVVLPAVPAGGEDDYVVLETVGTPPEFDFEPKDHLELGEALGLMDMERGAKVSGSRFYFLTGHGALLQLGLLQLAAQKAVANGFTMMIPPVLVRPEVMAGTGFLGRHAAEVYHLADDDMYLVGTSEVPLAGYHADEILDLSAGPKRYAGWSSCFRREAGSYGKDTRGIIRVHQFDKVEMFVYTTPDQAEAEHERLLAWEREMLAAIEVPYRIIDVAAGDLGSSAARKFDCEAWVPSQQTYRELTSTSNCTTFQARRLSVRYRDENGKPQIAATLNGTLATTRWIVAILENHQRADGSVRVPAALVPFVGTDVLRPPA

>CORE_REP|Org12_Gene4586#

MRTLDTSFSTRNGGFREVVVTAVEITTSIGADTESTWQALLSGASGIKVLTDEDITRHDLPNAIGGKLIHDPTADLDRVRKRRMCYVQQMSYAMGQRLWETAGAPEVDKDRLGVCIGTGLGGADVIVEANDTMREHGYRKVSPFAVPMSMPNGVSGVVGLDIGARASLVTPVSACASGNEALVHAWRSIVLGDADMVVAGGVEGYINPMAIAGFTMARALSSRVDEPERASRPFDRDRDGFVFGEAAALLLVESEEHARARGATPLARLLGAGLTADGYHMVAPDPEGLGCARAMRRAIETAGVSAADVDHVNAHATGTSIGDLAEAKGIAAAIGTHPAVYAPKSALGHSVGAVGALEAAISVLTLRDQVIPPTLNLDNQDPEIDLDIVHDKPRHTDVEFAMNNSFGFGGHNAAVLFGRY

>CORE_REP|Org19_Gene113#

MNTPSFVIVGASLAGAKAARALRDNGFDGSLTLIGDELWYPYERPPLSKDYLQGKVDRDTVFVHPPRWYTTHEVDLRLDTTATAIDRGNHLLTLGNGEQLPYDKLLLTTGASPRRLPLPGADAHGVYYLRSLDDSNRLRELLHTASRIALVGGGWIGLEVAAAARAAGVEVTVVERSPLPLQAVLGPDIAAVFADLHRDHGANLHLAATLAEITTCDGAATGLRLADGSRIDADAVIVGIGATPNTGLAGDSGLDVDNGIVVDPALRSSDPDIFAAGDVANAYHPFYSRHIRVEHWANALHQPDTAAATMLGRDASYQRLPYFYTDQYELGMEYTGYCEPDQHYQVVVRGDLQERRFIAFWLRDDRVAAGMNVNIWDVTEPIQTLIRTRTVVDPARLADPDVPLTDLTTPASSRTERDMN

>CORE_REP|Org15_Gene4950#

MSTEQLQQRWSSALMNNYGTPKVALVRGSGAVVYDAEGKRYVDFLGGIAVNSLGHAHPAILEAVAQQLATLGHVSNLYVSEPVLELAERLLAHFGDGTGPIGQGSEEAAGVTTEGPRSGRIGTARAFFCNSGTEANEAAFKIARLTGRHTIVACEEAFHGRTMGALALTGQPSKRAPFEPMPPGVVHVPYGDAAALAAAVDSDTAAVFLEPIMGESGVIVPPPDYLAEARRITSERGALLILDEVQTGICRTGPFFAHQAAGIVPDVMTLAKGLGGGLPIGAVLAQGPAAELLTPGLHGTTFGGNPVSAAAALAVLRTIDEQGLAAHVESVGKTLIDGIEELGHPLIDHVRGAGLLIGIQLTQDVSAKVEEAARAAGYLINPPKPNVIRLAPPLILTEAQAQGFLVDLPGILDAAFQESE

>CORE_REP|Org105_Gene3182#

MRVARARPSVYVESVSFAHAAAMTGPADRLRVAMLTREYPPEVYGGAGVHVTELVSELRALAEVTVHCMGAPRDDAVVHQPDTHLYAANPAIQMMSAQLRMADATGEVDVVHSHTWYTGLAGHLSATLYGIPHVLTAHSLEPRRPWKAEQLGGGYRLSSWSERNAVEHADAIIAVSAGMRRDVLDAYPAVDPARVHVVHNGIDASVWHPGPPEAGGEPFLWQLGVRTDRPIAAFVGRITRQKGVAHLLAAARDFDSEIQVVLCAGAADTPELAAEVASAVEELSRRRGNVFWVQDMLPTEQIRQVLAAATVFVCPSVYEPLGIVNLEAMACGTAVVASDVGGIPEVVADRNTGRLVHYDPAAPSEYERGLAEAVNELAADQVLASEYGAAGRARAVAEFDWSRIAAQTLEVYDRVRKP

>CORE_REP|Org5_Gene4499#

MDNRNGCEHPTSNTHRQAPDRARDGDGVTAPQYDSCSVTSMSSDDGARRPDHAARRAGLRDLLVENGVDALLVTDLVNIRYLTGFTGSNAALLVHSWDGSQVEDEGPRTVICTDGRYITQVGEQVPDVRAEIARASARRLVELAGEWQIGRVGFESHVVTVDQHRAFVEQGSGLQFVPAPGLVEQLRTVKDAYEVDRLRTACAAADAALAALLERGGIRPGRSEREVARELEWLMFEHGADGIAFETIVAAGANSAVPHHRPTSAVLAAGDFVKLDFGAVVGGYHSDMTRTLVLGEPSDWQREIYQLVYEAQRAGREALAPGVRCADVDAASRAVIEAAGYGDLFVHGLGHGVGLQIHEAPPVAKTATGTLLDGVAVTVEPGVYFPGRGGVRIEDTLVVRAGEPELLTHTGKDLTVVD

>CORE_REP|Org2_Gene6466#

MNPRPASISARPVDEYRDSIEQLLRPLAARAVEDVAVPHALGRQLADDVRAPVDLPVFRNSAMDGYAVRAASVAVAPVTLPLAGVVAAGNAGQTPLPPGAAMKVMTGAPIPPGADCVVPVEDARADEGTVTVERGRSAGEFVREPGTDVHAGDLLARAGTTLAPRHIAALAAVGLPAVAVVRPVLAAIITTGDELVPAGTELRPGQIYNSNGIALAAALTANGVTVVSVEHSTDDPAQFRKLLAAATGSADVVFTSGGVSKGDFEVVKDVLEPLGGQFGPVAVQPGGPQGRTVVDGVPVLSFPGNPVSTMVSFEVFARPILRRLAGLAPVPSYDLPLRNAVARSPQGKRQFLRGKLIHPESGDLPASQQLPEAVEVVSGPGSHLIASMAWADVLIDVPAAATTLPAGTVVRVWTL

>CORE_REP|Org198_Gene6103#

MSDPSRSEIVSPVTDAPNATTAPRADDAASLSAITHDEIFAGHLGGKLSVELSSPLETQRDLSIAYTPGVAQVSRAIAQDEELSKRYTWTERLVVVVSDGTAVLGLGDIGPRASLPVMEGKAALFKKFAGLNSIPIVVDTKDVDEIVDLLVKLRPSFGAVNLEDISAPRCFEIEKRVIEALDCPVMHDDQHGTAIVVLAALNGAAKVQGRGIEGLKVVVSGAGAAGVACTNILLAAGVRDVTVLDSKGIVSRERTDLNEVKAELATRTNPRGLNGGAAEALNGADVFLGLSAGLIAEELIASMAPESIVFAMSNPDPEIHPEVARKYAAIVATGRSDFPNQINNVLAFPGVFKGALDAGARRITEGMKIAAADAILSVVADELGPDKIVPSPLDPRVAPAVAEAVAAAARAEGVA

>CORE_REP|Org102_Gene1805#

MLGSTRLPRALPIRWAPVTVASPFDLIVVGSGFFGLTIAERTANLLGKRVLVVERRYHLGGNAYSEADPETGIEIHKYGAHLFHTSNKRVWDYVNQFTEFTGYQHRVFAMHKGQAYQFPMGLGLLSQFFGRYFTPDEARKLIAEQSAEIDTKDAANLEEKAISLIGRPLYEAFIRDYTAKQWQTDPKELPPGNITRLPVRYTFDNRYFNDTYEGLPKHGYTAWLAKMAESDLIEVRLDTDWFEVRDEIRAQNPDAPVVYTGPLDRYFDYAEGELGWRTIDFETEHLETGDFQGTSVMNYNDADVPYTRIIEPRHFHPERDYPTDKTVIMREYSRFAQTGDEPYYPINTPDDRAKLLAYRERAKTETAAAKVLFGGRLGTYQYLDMHMAIGSALNMFDNVLRPHLESGAPLVDTAE

>CORE_REP|Org186_Gene601#

MTNGNSTPSLRKDGGRDEDRAQSAVDDFAYGSGAPRHGFFNRLYTGTGAIDVIGKRRMWYAITALIVLISLASMLVRGFNFGIDFEGGSRIQFPAGDATTSEVETVYHNTLGTDPVSVQTVGSGSTATMLIRSEALSTEQADQLASALFTEFQPLGNDGQPSRAAISTSDVSETWGDQITRKALIALLVFLVIVSVYIAVRFERDMAIAAMAALVFDVGVTAGIYSLVGLEVTPATVIGILTILGFSLYDSVVVFDKVEENTRGILHLNRRTYAEQANLAVNQTLMRSINTALIGILPIVGLIVIAVWMLGVGTLKDLALVQLVGLLVGTYSSIFFATPLLVSLKERWGPVAAHTRKVLAKRSNVASARAAAEADRRVSVATGRPAAPGGPARPRPNAAPRPGARPSGKRHRRN

>CORE_REP|Org150_Gene6053#

MSTSTGSSPGTDTAAGNGPPGGGSLTEPFTAAIAEAEKLIAAADFINDEKDLAEGYDYLAGSIAACLQLSGTHGTSHPYFVSSTGPHAKMGLDNPDTLYYHANVEPGAEYLLTGVRGSTVDLSFQVLRGDYTATDVPNGDDAFDDRRLQIAADGSYQLRFGPPKADAGPNYFVLGEGASMLAVREVYGDWNAETKGSIRIERLDTVGTAAPALGVDQLRKRYRAAARALVQRIHTWFNFPKWFYLDLPVNTLTEPRLTPGGLSTQYSSVGHFDLDDDQALVITVPKSDAPYQGFQLGSRWYISLDYVNHQTSLNSAQAQVDPDGMIRMVVSARNPGITNWIETTGRRRGILQFRWQRVAREMTPADGPQFTLVPFDEVAAQLPHYELNRIDEQGWRARIADRQRGFADRMLG

>CORE_REP|Org88_Gene1874#

MQSWSDTPIPTVPGAGPPLRLYDTADRQVRPVTAGATASMYVCGITPYDATHLGHAATYLTFDLINRIWRDGGHEVHYVQNVTDVDDPLFERAARDGVDWRELGTSEIELYREDMAALRIVPPRDYIGAIESVDEVVEFVGKLVASGAAYTVDDAEFPDIYFRADATEQFGYESGYDRATMERLFAERGGDPDRPGKRDTIDALLWRAARPGEPSWPSPFGPGRPGWHIECAAIAVNRLGTEFDIQGGGSDLIYPHHEYSAAHAEALVAGRRFARHYVHAGLIGLDGEKMSKSKGNLVLVSTLRRAGVDPAAIRLGLLAGHYRQDRMWTDAVLDEAGARLDRWRCATALPAAGAATDTIARLRQHLADDLDTPKALAAVDNWADEALTYGGTDTDAPAAIATAVDALLGVRL

>CORE_REP|Org150_Gene4138#

MINLELPKKLRASANQAHQVAAEIFRPISRKYDLAEHEYPVELDTMAAMVEGLADSGTQDISGATGGRKAKSGEQAADSHSTELLGNSNGGNMSALLNALETCWGDVGLMLSIPYQGLGNAAIAAVATDEQLERFGKVWAAMAITEPSFGSDSAAVSTTAVLDGDEWVLNGTKIFVTAGSRATHIVVWASVDKSKGRAAIKSFVVPRDAKGLTVARLEHKLGIKASDTAELRLEDCRIPADNILGSPEVNVEKGFAGVMQTFDNTRPLVAAMAIGVGRAALEELRTILEESGVEISYDIPANNQSAAAAEFLRLESDWEAAYLLALRAAWMADNKKPNSLEASMSKAKAGRMGTDVTLKAVELAGAVGYSQRTLLEKWSRDSKILDIFEGTQQIQQLIVARRVLELSSAQLK

>CORE_REP|Org176_Gene2589#

MFIDLTTEQRRLRDELRAYFADLVTPEEEAEMAVNRHGDAYRAVVRRMGRDGWLGVGWPKEYGGQGFGPVEQQIFFNEAVRADVPLPLVTLLTVGPTLQQFGTAEQKQRFLPGILAGDIHFAIGYSEPEAGTDLAALRTSAVPDESGDWIVNGQKIFTTGAHEADYIWLACRTGSTESRHRGITILIVDTADPGYSWTPIITCDGAHHTNATYFDNVRVPANMLVGEENRGWKLITTQLNHERVSLGPSGKIEQLYDRVRDWAQPRGVLAELDVRRALGRIHAMVRLNELLNWQVAAASDGRRSQGPAGSRSATASGPGTPAAGVDGDQSRVIADASATKVYSTESLQEAGRLAEEIVGRYGDPADPATGELLTWLDRRTKQNLVVTFGGGVNEVMRELVASAGLRLPRVPR

>CORE_REP|Org49_Gene3373#

MKSAFPGPVGAQTVYLDHAATTPMFPVAVEAMTAVLGTAGNASSLHGSGRAARRLLEEARESIAANLGARPSEVIFTSGGTESDNLAVKGIYRARRDAEPRRRRILVSAVEHHAVLDAVEWLEQHEGADVTWLEVDSEGVVSPRTLRAALESYADEVALVSVMWANNEVGTVQPIVELSSVAQEFDVPMHSDAVQAAAQLPVDFAASGLSAASFAGHKVGGPHGIGVLLLGRTVPCVPIVHGGGHERDLRSGTSDVPAAVGLAAALRETVRGMASRTVELTRLSDRLIAGVRELVPDAVLNGATGERRLPGNVHFTFPGCEGDSLLMLLDAAGIECSTGSACNAGVAGPSHVLLAMGVEPALARGSLRFSLGHDSTDTDVDAVLAALPQVVERAKAAGLASVGSAPHAKGGY

>CORE_REP|Org140_Gene4764#

MVRGKGTSVPEQAFIYEAVRTPRGKQRGGALHSMKPVDLVSGLIDEVLARHGGLDPADVDDVVLGVVTPIGEQGSVIARTAALNSGLQETVPGTQINRFCASGLEAVNLASAKVASGFDDLVLAGGVESMSRVPMGTDGGALFADPATAFDHHIVPQGVSADLIATIEGFSREDVDAYAAESQNRAEKAWASGYFAKSIVPVKDINGVTILDHDEHRRPGSTVESLGKLKPAFAALADMAGFDDVALQKYPSVEKINHVHTGGNSSGIVDGSSLVLIGSEQAGERNGLTPRARVVTFAQIGSEPTIMLTGPTPATELALKKAGLSVDDIDVFELNEAFASVVLKWMKDLKIPHEKVNVNGGAIAMGHPLGATGAMIYGTCLDELERTGGRYGLITLCVGGGMGIATIIERL

>CORE_REP|Org50_Gene671#

MSGGQGRQKSARRWSMRTIAGPVIVVAVAALALTGCSASDATEAAQAAIDRNPITELIKPKLMSPVKDGEVGVSPGVPMTFKVEDGKFTNVSLVSPQGKTVNGKLAADGRSWETTEVLGYGKTYQLKADAIGLGGANSATLSFTTSSPGNQTKPYLIPGEGEVVGIGQPVAVQFDENIPDRKAAQDAIKITTEPPVEGAFYWVNNREVRWRPEHFWAPGTKVTIDVNVYGRDLGNGLYGQDNIHSFFTIGDAVIFTADDDTKQVTVEQNGQVIRTMPTSMGKDSTPTDNGIYIVADRHEKIIMDSSTYGVAVNSPDGYKTPVDFATRLSYSGIFFHSAPWSVGAQGYSNTSHGCLNLSPANAQWVFQNAKRGDITIVKNTVGGTLSGVDGLGDWNIPWPVWKAGNADDNR

>CORE_REP|Org144_Gene5981#

MSEKEPNDMGRPDEGHDGGKPSTEQVTGAGAHVAKTDGHALTEPTEAELDAMSRDELVKLGTERDGVDVAYRRERFPVPGTRAEKRAERAVAFWFAVSGIAAAALVGVFLFWPWEFKANKEDGHAAYSLFTPLVGITFGVSVLVIGIAVVLIRKLFIPAELSIQDRHDGPSPEVERRTLVAELSDALDSSTLARRKLITRTAGAGVGVLGIGALLVFVGGMVKNPWAKGDKSPLWVSGWTPDYEGETIYIRRDTGRPEDVVLVRPEDLDAGAMETVFPWKEKWRGDEHATLQSLRGIRNAVMLIRLRTEDAQKAIKRKGQESFNYGDYFAYSKICTHLGCPTSLFEQQTNKILCPCHQSQFLATEWGKPVFGPAARALPQLPITVNSEGFLVANGDFIEPLGPAFWERRS

>CORE_REP|Org163_Gene5468#

MLTGHFFGAVAGSMIFGGVLGDNGVGERFGRGGRAMDEERQQWTGVRRRPRRGRAFILVVTFVLATAVFLGYRGDLPAPNRPYPAEPVASVAFTPPAPLDPGLVTAAVRPALVNINASAERSGPGAAGSGIVLTADGEVLTSHHVVKGADTVIVTDVGNGKVYNAVVLGYDSEADIALLDLPAAAELPIATIGTSTGLRLREEVLAIGNAGGTGGTPTAVHGPLTDLDSAIVAVNAADLSRKALSGMLEVAAAVTPGQSGGALVDRNASVVGVIAAASGDGARTADQPANGYAVPIDAAMRVVQQIRSGTPTDTVHVGPTATLGVLISNALPVGTGARVDVALHGMPAYAAGLAAGDVITSLDDHVVTSAQSLRAALNTRKPNDTVRLGVTGAAGERTVRVVLVAGPPN

>CORE_REP|Org120_Gene4992#

MTTFAAASNANGRLVRNQGVTGPLGFRAAGIAAGIKASGKPDLALVFNEGPEYAAAGVFTSNKIKAAPVLWSQQVLTGKRLRAVILNSGGANACTGPGGFQDTHQTAEELAAALSNWGTETGAGEIAVCSTGLIGDRLPMDKVIPAITEIVHEMGGGLSGGLDAAHAIMTTDTVPKEAAFHHRDKWNVGGMAKGAGMLAPSLATMLVVLTTDAAVSADQLDQALRNATARTFDRLDVDGSCSTNDTVLLLANGASEVTPSQADLDAAVLAVCDDLAAQLMADAEGVTKRVLVTVAGAVNEDEAVAAARTVARDSLVKTALFGSDPNWGRVLAAVGMAPVTLDPNRISVSFNGNPVCIDGAGAPGARDVDLSGMDIEVRIELNVGDAQATIRTTDLSHGYVEENSAYSS

>CORE_REP|Org134_Gene3544#

MELKVQVYRHASSIPGGCPPPTTPVVAGSGIVNAQVETVVDLDAIAHNVRILREHAGDAAVMTVVKADGYNHGAVEVGRAALAAGAAELGVTTISEAVHLREAGITAPILCWLNNSGADYGAGIAADIEIGISSMSQLRAVEAAARRLGRTATLTLKVDTGLNRNGVSVTEYRDVLTALRPLVDEQVLRFRAIFSHLAHADQPHHPTIDVQRDRFVDAIATAKEYGLVPEVTHLANSAAALTRPDLAFDMVRPGIAMYGLSPVPELGDFGLRPAMTFQAEISLIKHVAAGEGVSYGHEWIAPHDTTVALIPAGYADGVSRRLGGRCEVWVRGARRPSIGRVCMDQMVIDLGDNLDGVAEGDTAILFGTGESGEPHAQDWADLLDTIHYEVVCSPRGRVVRRFRGGQQ

>CORE_REP|Org104_Gene2232#

MSPREGTTAMSAPIVIVGAGLAGLRTAEELRRAGYEGDLVLLGDEARLPYDRPPLSKQFVRGETDDTTLRPAEFFTDKRIELRLGTTATGVDTATRRVLLADGSALAYDHLVIATGLRPRTLPGLPTPAGVHVLRDHADATALRDESASATAALVIGAGFIGCEVAASFRARGLDVVLVEPQPTPLASVLGEQVGGLVARMHRAEGVDLRCGTGVRTLLSDDRGRVRGALLSDGAEVRADLVVLGVGSRPAVEWLADSGIALAEQAAGGGVLADEVGRTSVERVWAVGDVAAWRHETGAQQRVEHWTNAGEQAKLVACALLGAEPPTAARVPYFWSDQYDVKIQALGTPSADDDVSVAADDGRKFLAYYSRAGALTAVVGAGMTAQVMKARAKVAAGAPVADLLATT

>CORE_REP|Org25_Gene399#

MTEAVIVATARSPIGRAGKGSLTGMRPDDLAAQTIKAALDQIPECDAHAIEDLYLGAWEHTGEQSENIARRVAVQLGLDGVPGATVNRACASSVQTTRMAANAIKAGDGEIFVSAGVECVSRYPQHNGVGAGDERFHNPTFDRARARTAQFAETGAPWTDPRAESLLPDVYITMGQTAENVASYRGVTRAEQDEFAVRSQQLAEKAIADGFFAREITPVTLPDGTVVRVDDGPRAGTTVEKLAGLSPVFRDSGTVTAGNACPLNDGAAALVLMSDRKAAELGLTPLARVVATAASGLSPEIMGLGPVEASQRALAKAGLSIGDIDLVEINEAFAAQVIPSYRELGIELERLNVNGGGIALGHPFGATGARITTTLLHTLRERDKQFGLETMCVGGGQGMAIIFERLS

>CORE_REP|Org101_Gene7494#

MTTVPTHPVGSASVRPRSVIVSGARTPVGRLLGGLKDFSGSDLGGFAIKAALEKGGVAPEQVDYVIMGQVLTAGAGQIPARQAAVAAGIPMDVPALTLNKVCLSGINAIALADQLIRAGEYEIVVAGGQESMSQAPHLLEKSREGFKYGDVTLRDHMAYDGLYDIFTDQPMGALTEQRNDTEPVSREEQDAFAAASHQRAAEAWKNGLFDDEVVPVAVPQRKGDPVLVAADEGIRADTTAESLAKLRPAFRKDGTVTAGSASQISDGAAAVVVMSKAKAEELGLSWLAEIGAAGVVAGPDSTLQDQPANAIAKACAREGISPADLDLVEINEAFAAVGVASTRKLGIDPAKVNVNGGAIAIGHPLGMSGARILLHLVLELKRRGGGVGAAGLCGGGGQGDALIVRV

>CORE_REP|Org170_Gene222#

MSKIKVEGTVVELDGDEMTRIIWQFIKDKLIHPYLDVNLEYYDLGIEYRDKTDDQVTVDAANAIKKHGVGVKCATITPDEARVEEFGLKKMWRSPNGTIRNILGGTIFRAPIIISNVPRLVPGWTKPIIIGRHAFGDQYRATDFKVFQGGTVTLTFTPDDGSEPIVHEVVKMPEDGGVVMGMYNFKKSIEDFARASFNYGLQQNYPVYMSTKNTILKAYDGMFKDTFQEIFDAEFKSQFDAAGLTYEHRLIDDMVASSMKWEGGYVWACKNYDGDVQSDTVAQGFGSLGLMTSVLLTPDGQTCEAEAAHGTVTRHYRQHQQGKPTSTNPIASIFAWTRGLEHRGKLDNTPEVIGFAQTLEDVVIKTVEGGQMTKDLALLVGGDQGYLTTEEFLAALDANLARALR

>CORE_REP|Org101_Gene1143#

MSTPADDLVLVINSGSSSIKYQLLDPESSAVTASGMVERIGEENGGIEHHADGASTEHRGPIADHTAGLRLVFEMFADTGHDLAAAGVRAVGHRVVHGGEVFYRPTLIDDKVVAAISKLSSLAPLHNPANVAGIESARTLLPGVPQVAVFDTAFFHGLPDAAKTYAIDAKVAAAHGIRKYGFHGTSHEYVSGQVAELLGRDPAELNQIVFHLGNGASASAIRGGRPVDTTMGLTPLEGLVMGTRSGDLDPGIVAHLVRSADMDIDQIDTLLNRDSGIKGLSGVNDFRELQRLIDGGDSAARLAYDVYIHRLRRYLGAYLVDLGGVDAITFTAGVGENSPQVRADALAGLSRFGIEVDAAANTAKDRTARRISPPDAEVAVLVVPTNEELAIARAAHDVAEAEAPR

>CORE_REP|Org5_Gene5772#

MSGFEQPFDQALRAGERPLCDLTRGKLRAQDLNWCRINQVSAWSMLPECGRADPMASEEVGPMSSTERRRLEVLRAIVADYIANKEPIGSKTLVDKHNLGVSSATVRNDMAVLEAEGYITQPHTSSGRIPTDKGYRQFVDNIAEVKPLSSAEKRAIMGFLESGVDLDDVLRRGVRLLAQLTRQVAMVQYPTVSASTVRHLEVVALNPARLLLVVITDTGRVDQRLVELGAVIDDEDLAALRGMLGKAMDGKRLSAASSAVAELPEQAPGRLRDVLIRVSTVLVETLVEHPEERLVLGGTANLTRNAGDFGFPGSLRAVLEALEEQVVVLKLLAASQEPGTVTVQIGEETMVEQMRGTAVVSTGYGMPGTVLGGMGVVGPTRMDYPGTIASVAAVARYIGEVLAER

>CORE_REP|Org64_Gene5402#

MKLPALLSKNTETLPGRTGIARVDRNTRRLLRRVGPGDIAVVDEMDLDRITADRLVEAGVVAVVNTSPSISGRYPNLGPEVLVANDIMLVDTVSSDAFTKIKDGSKIRIHDGVVYADKLTKKEPEALVEGIELTEAAIAERMIEARNGLADHLEAFAGNTIEFVRTESALLIDGIGVPELELDMKQRHVVVVADGPDHAEDLKRLKPFIKEYAPIMVGVGRGADTLRKQGYRPDLIVGDPEEITSATMKCGAEVILPADTDGHAKGLERIQDLGIGATTFPSSGAPADLALLLAEHHGAALIVTVGAAASLDDFFDRGRRDSNPATFLTRLKVGTKLMDAKAVATLYRNRMSGVALAMVVLAALIAVIVVLLASNTGTEVLDWAVDTWNRFARWCQDLVGAGQR

>CORE_REP|Org45_Gene5718#

MRRSQQGGGDAAGGSAGFDSGDNERVSDVVKVLLLGSTGSIGTQALEVIAANPDRFEVVGLAARGGNTELLASQIAATGTGNVAVADPAAAAKLGVPLAGPHAAAQLVRDTDADVVLNALVGSLGLEPTLATLESGRRLALANKESLVAGGSLVTRAAAPGQIVPVDSEHSALAQCLRGGRAEEVERLVLTASGGPFRGWTTEMLESVDPAAAKAHPTWSMGLMNTLNSASLVNKGLELIETHLLFGIDYDRIDVTVHPQSIVHSMVTFTDGSTLAQASPPDMRLPIALALGWPDRVPGAATACDFSQAATWTFEPVDNTVFPAVELARRAGKAGGCVTAVYNAANEIAVQAFLDGVIRFPDIVRTVARVVESADRWSAEPSTLDEVLAADTWARDCARAFVRN

>CORE_REP|Org105_Gene3394#

MGKRSADRADIVVGMLNEQRIREDTPGVGHGLVFLDSAGSSLPPRVVTETVIAHLRREAAVGGYRAANERLGDLAAVKESIAALINASPAGIALSDSATRSWADFFYSVPLGPGDRILISGSDYASNAIAALQRARASGATVEHIPSDPTGQLDLDAFAGLVDERVKLVSLLHAPTNGGLVNPAAEATRIAHEAGALVLLDACQSAGQIPLDVAELGVDALSATGRKWLRGPRGTGFLYVRPELAARMEPARLDLHSAEWTAPDDYRLAPDAGRFEFWEHDVAARLGLGAAVDYLLELGPDEVYAAIAARAEYLRKGLAEITGVTVRDLGIRHSGIVSFTVDSVAPVQVRDRLAAEDITVTVSHRSSTLLDMAGRALDAVVRASPHCFVDFAELDRFLTAVAEL

>CORE_REP|Org138_Gene5469#

MLATTGARLRDAVPLAELTTLRVGGPAPVADCAGTEALVATVRALDAADIPVLLVAGGSNLLVADEGFPGVVVRIANAGVRILTGADDAVAAVAGNDARAAATRTDAETGAVVATPAAGGIGVTDMPGGGDVVRVIAEAGANWDAVVAETVVAGYGGLECLSGIPGSAGATPVQNVGAYGVEVASLLTRVQLLDRASGEITWVQPSELGFGYRTSVLKHSDHAVVLAVEFALRADGSSAPLRYRELAAALGAEEGESRPAAEVRAAVLRLRAGKGMVLDPADHDTWSAGSFFTNPVVPAARVDEVRAAIAARVGPDVAVPTYPAPDGVKFSAGWLIERAGFAKGFPDESAPARLSTKHTLALTNRGAAKASDVVALARTVRDGVAERFGIRLEPEPVTVGLTL

>CORE_REP|Org45_Gene3101#

MVSSSDLLSQGVFSTAAVVPLRELLLVLLVATVVTYLSTGGVRVGAIAFGAVAVPRERDVHVKPIPRMGGVGIYLGVLAAVLFAHQLPALRRGFDYPADIPAVVVAGTLIVLVGIIDDRWGLDWLTKLVGQVTAAGVMAVMGLSWVAIYNPFTNTTVVLDQLQGGLVTVGITVTMINAMNFVDGLDGLAAGLGLIAAAAVFVFTVGLLYEQGGSTDTYPPALLAAALAGGCLGFLAHNFQPARIFMGDSGSMLIGLMLAAVSTGASGRIPLQGYGTRDIVGLLSPLLLVGAVMFIPVLDLVLAIVRRVRAGVSFSTPDKMHLHHRLLQIGHSHRRVVLLIYLWVSVLAFGAVGSSLMDRRLVVLLFAGGLVFALVITAVPSMGELTLKAGRKPPRGPDAARG

>CORE_REP|Org33_Gene789#

MTAASSEPVIFDPYDYAFHEDPYPVYARLRAEAPLYHNPELDFWALSRHADVTAAFRDATRLSSANGVSLDPAAWGPHAHRTMSFLAMDDPRHMRMRKLVYKGFTPRRVAEMETRIREITLSYLEPALERGRLDWIDEFAGKLPMDVISELMGVPEPDRAEIRRLADLVVHREDGVLDVPDAAIDASLRLVGYYADMVKDRRADPTDDLTSALLDAEIDGDSLSDDEIIGFMFLMVVAGNETTTKLLGNAVYWAARNPAEYAKVAADPDRVPDWVEETLRYDTSSQMVARSATTDIDYHGGTIPAGAKVLLLIGSANRDSAAFDDADSYRIDRHDTSALASFGAGVHFCLGAHLARLEANVALREFATRVTEYTVVTEGIERVHSTNVRGFAHLPITVEVH

>CORE_REP|Org14_Gene2625#

MTYDVARVRGLIPSLGDGWIHLDPQAGMLVPDSVSRAVSTGFRTSAFSHTNRHAAARRSGAILDAAREAVADLVGGDPAGVVLGPDRAVLLAWLAESLSSRLGLGTGIVLSRLDDEANVAPWLRIANRYGAHVRWAEVEIETCEMPAWQFEELIGPTARLVAVTAASPIVGSAPAVRVAADRVHEVGGLLVADCFGAAPYALIDIDELNADVVALSAPAWGGPQIGALVFRDPAFLDRIPSMSLNPYAKGAERLEVGGHQYALLAGLTTSIDYLAGLDEQATGSRRERLEISITSLQDYHDQLFEHLMEVLDAVPDLTVIGRASTRIPTVSFTIAGMQAEKISAELADHRIGTVSGAHGGSRLLDALGVNDEGGAVTLGLAPYTTKFEIEQLGRALNSLEK

>CORE_REP|Org94_Gene5557#

MSERVVLAYSGGLDTSVAISWIGKETGAEVVAVAIDLGQGGEDMNVVRQRALDCGAVESIVIDARDEFAEQYCLPTIQANALYMGQYPLVSAISRPLIVKHLVEAAKFHGADTVAHGCTGKGNDQVRFEVGIGALAPDLNVIAPVRDYAWTREKAIAFAEENKLPINVTKKSPFSIDQNVWGRAVETGFLEDLWNAPTKDVYDYTADPTVNFEAPDELIITFDKGVPVAIDGRPVSVLEAIVELNHRAGRQGVGRLDMVEDRLVGIKSREIYEAPGAITLITAHQALEHVTIERELGRYKRQVEQRWGELAYDGLWFSPLKRALDAFVQDTQQHVSGDIRMVLHGGSAVVNGRRSEQSLYDFNLATYDEGDTFDQSLAKGFVQIHGLSSKVAARRDLNQK

>CORE_REP|Org114_Gene7015#

MTTASGGRPTGGLSGRAAIVGIGATDFSKDSGRSELRLAAEAVTAALADAGLTPADVDGLTTFTMDTNTQAAVARATGIPSLKFFSNIPFGGGAAAATVQQAAMAVATGVADVVVAYRAFNERSGNRFGQFATHLATGNPSSSGVDNAFSYTHGLGTPAAQVAMVARRYMHVYGATSADFGRVAVADRKHAAVNPAAHFYGKPITLEDHQSSRWIAEPLHLLDCCQETDGGVALVITSAERARDLPNKPAVILGAAQGSGADQYVMTSYYRDALTGLPEMGLVGDQLWSQSGLTPADMQAAILYDHFTPFVLMQLEELGFCPRGEAKDFIADGAIELGGRLPLNTHGGQLGEAYIHGMNGIAEGVRQIRGTSVNQVDGLENIIVTAGTGVPTSGLILSTN

>CORE_REP|Org19_Gene2810#

MSSPQSLPPLVEPAAELTRDEVARYSRHLIIPDVGMDGQKRLKNAKVLVIGAGGLGSPALLYLAAAGVGTLGIVEFDEVDASNLQRQIIHGESDIGRSKADSARDSILEINSGVEVVLHKIRLEPENAVDLFAQYDLIVDGTDNFATRYLVNDAAVLAGKPYVWGSIYRFEGQVSVFWEDAPDGPNGEKRGINYRDLYPEAPPPGMVPSCAEGGVLGVLCASIGSIMVTEAIKLITGIGETLLGRLMVYDALDMNYRTIKLRRDPERQPITELIDYEAFCGVVSEEGQAAAVGSTVTARELKDMLDAGKDVAIIDVREPVEWDIVRIDGATLIPKDRILSGEALAELPQNTPIVLHCKTGIRSAEALAALKRAGFSDATHLQGGIVAWANQVDPSLPVY

>CORE_REP|Org214_Gene3195#

MSSKNGGFSVRGVREWLEAPAASVAERGGKLNVLRGAVARVTTPLLPDDYLHLANPLWSARELRGRIVDVRKETADSATLVIKPGWGFDFKYEPGQYIGIGVLVDGRWHWRSYSLTCPPNWSDPGVGGKRVISIAVKAMPEGFLSSHLVSGVPVGTVVRLAAPQGGFVLPYPPPERVLFLTAGSGITPVMAMLRAMDRRDLVTDVVHLHSARTAQDVMFGAELRDLHDRYATTHPADSVGDRPPASFTSHLHLTGEQGKFALADLDTKFPDWRERQTWACGPAAMLDEIEHHWREAGLADQLHVERFEIERSAVGEGGTVSFGKTGRTVEVDGATSLLEAGESAGVQMPFGCRMGICQTCVVTLSSGYVRDLRNGDEHREGDKVQTCISAAAGDCTLDV

>CORE_REP|Org121_Gene3999#

MNSVSRSDSVGGGGRATGGRWNTPERRELRATVRSFAEREILPYMDEWERDGEIPRELHKKAGALGLLGIQFPESAGGSGGDGIDAMIVCEELHQAGASGGLFASLFTCGIAVPHMIAAGNPEQIERWVRPTLAGEKIGSLAITEPGGGSDVGHLTTTARRDGDHYIVNGAKTYITSACRADYVVTAVRTGGPGSQGISLLVVEKGTPGFTVSRKLDKMGWRASDTAELSYVDVRVPAANLVGAENSGFFQIAGAFVSERVGLAVQAYSSAQRCLDLTLDWVRSRETFGRPLISRQAVQNTVTEMARRIDVARVYTRDVAQRSANGETDLIAEVCFAKNTAVEAGEWVANQAVQLFGGLGYMRESEIERQYRDMRILGIGGGTTEILTGLAAKRLGYQS

>CORE_REP|Org14_Gene5086#

MHRLRHGDTGPAVAEVRSNLASLGFHPHPHGTDRSGGQGEYWKDSDAVFDRELDSAVRAFQQQRGLLVDGVVGPATYRALKEASYRLGARTLIYQLSAPLYGDDVATLQRRLQDLGFYVHRIDGYFGPHTHDALTAFQREIGLSADGICGPDTLRSLELLGARVTGGNPHRIAEEEVVHRAGPQLTGKRIVIDPGMGGEDKGLAVPTEFGDVYESEILWDLASRLEGRMAATGMETFLSRPWGANPTDAERAETSNTFDADLMISLRCAANLSSSAGGVASFHFGNSHGSVSMIGQVLAGFIQREIVARTSLQDCRTHARTWDLLRLTKMPTVQVDIGYLTNEYDASVLTNPRMRDVIAEAILISVKRLYLLGQDDQPTGTYTFAELLAEELAAADRM

>CORE_REP|Org12_Gene3677#

MSQKRTGCTEGAAETLVADRSAHFRLVRKCAVAYETVGRSSGRCFVTGYRLAIVGVPTAVPVSQEPGLKLGWPDRADKARLHYVSGKGGTGKSTVAGALALALAAGGRRVLLVEVEGRQSIAQLFDLPPLPPTETKIATADGGGEVMALTLDIEHAFLEYLDMFYNLGFAGRAMRRMGAIEFVTTIAPGLRDVILTGKIKECAVRTDKSGRRVYDEVVVDAPPTGRIAGFLDVTKAMAEVAKGGPIAGQAEGVAALLHSEQTIVHLVTLLEALPVQETSDAIAELTESDFRIGTVIVNRATEGFLPAPVRARVATGDVDLDAVRAGLAEAGITVDDNDFQGLIREAVEHSATLQAQDDSSAELAKVDISRLYLPALPDGMDLGGLYELAEHLSAQGVR

>CORE_REP|Org4_Gene2732#

MSAPSTPRRPYTPLRDAYVIDAVRTPVGKRGGALAAVHPADLGAAALRGLLDRNSIDPGNVDDVIVGCVDNVGPQAGNVGRTAWLAAGYPEEVPGVTVDRQCGSSQQAINFGAQAIMSGTAEVIVAGGLQNMSAIPISAAMYAGKEYGFDSPFVGAAGWDHRYGTAEVTQFRAAQLIAEKWGISREDMERWALRSHERARDAIKNGRFDREIVPVGDFWIDQGPRETTLEKMASLPPLAEGSPLTAAVASQISDGASATLLASEWAVEAYGLTPRARIHHVSARGADPIFMLTAPIPATKWALEKTGLTIEDIDVIEINEAFAPVVLAWLKETGADPEKVNVNGGAIALGHPLGATGAKLFATLLNELERRNGRYGLLTICEGGGTANVTIIERLPN

>CORE_REP|Org142_Gene4996#

MRRDSVRRPGERRGSRPVSEDRVIFSRPYRAAAEVENLRAVLDSDHSHGDGRFTKTATAKIKAITNSPHALLTTSCTHALELGALLLELGQDDEVIVPSFAFTSAATAVALRGATCVFVDIDPATGNIDPMSVADAVTDRTKAVLVMHYGGVAADMAPLLEIAGEHGLALIEDNAHGLGGTWRGRALGTIGTIGTQSFHDTKNVHCGEGGALLLSDEILMGRAEIIREKGTDRARFLRGQVDKYSWQDIGSSYLPSELNAAVLDAQLAEFDRIQTGRHRVWDAYASALPEWARRNDVRLMQVPGDREHTAHLFYLRLPSEDIRDTMIRHLADRGIVAPFHYVPLDSSPAGLKYGRTPVPCTHSAEFSATIVRLPLWPMLGDDQIQRVVDAVTAFAV

>CORE_REP|Org142_Gene4386#

MWDAIQYCLEYRPEEIPVAVDRLLPTDEAKDLIQLTRDVADKVLAPIVDEHERSETYPEGVFATLGEAGLLTLPYPEEWGGGGQPYEVYLQVLEEIAARWTAVAVAVSVHSLSIHPLMAFGTEEQKQRWLPEMLGGTTIGAYSLSEPQAGSDAAALACRATGVDGGYRITGSKAWITHGGIADFYNLFARTGEGSKGISCFLVDKDTEGLSFGKPEQKMGLHAVPTTSARYDDAFVPSERRIGNEGQGLQIAFSALDSGRLGIAAVAVGLAQAALDEAVAYAQERVTFGRKIIDHQGLGFLLADMAAAVDSARATYLDAARRRDAGLPYSRNAAVAKLVATDAAMKVTTDAVQVLGGYGYTRDFRLERYMREAKITQIFEGTNQIQRLVISRHLAG

>CORE_REP|Org51_Gene384#

MALSAGTRRTVACLLVGLGALLIVMALLIPTYTVDKLAKTPLDLEITTIANSQQGQDSLVLDSKSLTAPEGSAKVDSNVPLISQRFLTVEEPSNATEMTVQAGQTLRRTDRQGDTGLLTASIDRVTIDRKTGMPVDTEPNGSIAVTTNAAGESIADPVQHTGLQYRFPIGTEKKSYPYFDLNARATFDANFIEETEINNLKVYHFQQTVPVTSMWDVVQAPTNRLTLPAAKWGLEGGDTPVTMTRYYTNVRDLWIEPQTGTVVKGSEQLHLFYGRSPQQEDVTALKSTLVFDENTIESQIAIAKDNIDTLSLFGRVVPIILGIVGVIALIAGALLGIRGAKNPAPAGGGFGPRGGAGPSRGPAPTGGAAAAPGSGAVRRGEDDAPTEQINIKKNL

>CORE_REP|Org27_Gene1522#

MNPAFVIDAVRSPMGRARANGALADVHPVELLAQVVSALIARTRVDPGEVEDLLVGCVTQSAEQSGNIGRMAWLAAGLPEHVPAVTIERKCGSGQQALQFAAQGVMAGSYDIVIAAGVESMSRVPIGSNRQGADIYGPSVTERYAPGLVSQGVAAELVAQRWGIGRARLDEFAARSHELAHAADAAGAFRREIVPVTVPGTGAVVDRDETIRPGTTADKISGLAPAFRTDELAERFPELDWRVTAASSSQLTDGASAVLIASERAAGRMGWQPRARFHGFHACGDDPLLMLTAPIAATRTLLARTGLGLDDIDHVEVNEAFASVPLAWADELHADLDRLNPRGGAIALGHPLGATGCRLLTTMLHAMEDNGGRYGLQTVCEAGGMANALVLRRE

>CORE_REP|Org192_Gene1814#

MAMQQEWADKDYYGDLGVSSSASAADIKKAYRKLARENHPDSNPGDKKAEEKFKRVAEAYDVVGDEQKRKEYDQLKSMISSGGFGRFGRGGGSGFPGGFRGTETEFDLSDIFGSAAGGQAGDSGLGDIFGGFFGGRGGAGRNARPSRGADVETEITLDFREAAKGTTIPVELTGDAPCTTCHGSGSKDGKTHTCQMCSGSGYIRENSGAFGMARPCTNCGGTGEIIEDPCDTCGGTGTVRRTRSITVRIPAGVIDGQKVRLAGQGEAGPNGTPAGDLFVTVHVRNDEVFTRSGDDLEVTVPVAFSEVALGATITVPTLDNPVKVKVPAGTPNGRTLRVKGRGIPKRSGAGDLMVTVEVKVPKDLDPSATSALRAYAQAEKDSGFDPRAGWAGL

>CORE_REP|Org102_Gene3646#

MGGVISAARPGTRATALGWDTGEVMTAPLPLVFDAPRRGMPPRHLADLDAEERRAVMADLGLPKFRADQIARQYYGRLQADPEQMTDLPADMRAKVGEALFPPLLTPVRHIACDDGSTRKTLWKAGDGTLLESVLMRYPDRATLCISSQAGCGMACPFCATGQGGLNRNLSTAEIVDQVRAAAAALRDGEVAGGPGRLSNIVFMGMGEPLANYKRVVNAVRRITSPAPDGLGISQRNVVVSTVGLAPAIRKLADEDLSVTLAVSLHTPDDELRDTLVPVNNRWPVAEVLDAARYYADKSGRRVSIEYALIRDINDQPWRADMLGKKLHKALGSRVHVNVIPLNPTPGSKWDASPKPVEREFVRRVEAQGVPCTVRDTRGQEIAAACGQLAAEG

>CORE_REP|Org150_Gene5131#

MPSLDNAGSHTPREDSGATDAAASQAGTDAAAAHADTGAAASRAGTDPAASRVGTDAAAPRAGSDAAGGADTAVRVSGADPAVVVDDVRKSFGEVQALQGISFTAARASVLGILGPNGAGKTTTVKILSTLLRPDSGSASVAGHDVVADAAGVRRSIMMTGQYAALDENLSGRENLELFGRLMGLPKKDARRRADTLLEEFDLVGAGKRAVRHYSGGMRRRVDIACGLVVRPEVVFLDEPTTGLDPRSRQGVWDLVNALKEQGITVLLTTQYLEEADVLSDNIIVIDKGTVIAEGTADELKEKTGGSYCEVVPLDPTQLRKAVTALGELVPEALRHEFAGDRISIPAPDGASTLAEAVRRLDAAGLELADIALRRPSLDDVFLSITGHSGGHQ

>CORE_REP|Org113_Gene518#

MSAIPTVARLRPFGATIFAEMTELAVRHDAVNLGQGFPDTDGPAAMLEAARTAIADGVNQYPPGRGMPVLRRAIAADRRLRYGTDYDIDREVLVTVGATEALAAAVLGLVEPGAEVVLIEPYYDSYAAVVALAGATRRTAHLVPDGTGFALDLDSLRAAITPKTRMLLLNTPHNPTGAVFSRADLEAIADLAREHDLIVVSDEVYEHLVYDGNTHISIATLPGMYERTVVVSSAAKTFSVTGWKIGWACGPAPLIDGVIAAKQFLSFVGGGPFQPAVAYALEHEQAWVRDLRDSLSDKRIRLSEALGDAGFTVQRSDATYFVCADISPLTSADALTFCRELPERLGVAAVPLSVFADDRPSWDRLIRFAFCKKDETLDEAVRRLHAAHGAARS

>CORE_REP|Org155_Gene1979#

MELSSMKFRVAREDFAESVAWVARSLPSRPPVPVLGGVLLVADEDGLTVSGFDYEVSAQMRVAAEVAGPGQVLVSGRLLADITKALSNKPVDVSVDGTRVLISCGSAKFSLPTMPVEDYPQLPEVPQQSGELNAEVFAEAVAQVAVAAGRDDTLPMLTGIRVEIEGPQVVLAATDRFRLAVRHIEWQPARPDIETAVLIPARTLSESAKTLGATDAPVQLSLGTGAGADGLLGIVNAGRRTTTRLLDAEFPKFRQLLPKEHTSIATLQVATLTDAIKRVALVAERGAQVRLEFSGEGLLLSAGGDDAGRAEEWLEADFRGEPLTIAFNPGYLIDGLSALHSDRVTFGFTTPSRPAVLLPASEDEEPQPLDSGSFPALDSAYIYLLMPVRLPG

>CORE_REP|Org31_Gene5179#

MSERSERTEGTAGLGFSTRAVHAGFDPDPQTGAVNVPIYASSTFAQDGVGGLRGGFEYARTGNPTRSALEANLAALESGRYGRAFASGMAATDCAVRATLRPGDHIVIPDDAYGGTFRLIDKVFSQWGIEHSPAHVFDVDEMRAAIRPNTKLVWVETPTNPLLSIGDIPALADVAHAAGAKLVVDNTFATPYLQQPLLLGADIVTHSTTKYLGGHSDVVGGALITNDPELDAAFAFLQNGAGAVPGPFDAFLTMRGTKTLAVRMDRHCDNAETLVEFLAGHPAIAQVIYPGLPEHPGHQVAAKQMRRFGGMISVRLHGGADAAREFCSRTKVFTLAESLGGVESLIEHPAAMTHASTEGSALEVPADLVRLSVGIEDAADLLADVEQALAS

>CORE_REP|Org56_Gene2793#

MGRTLLITNDFPPRPGGIQSYVHSLALRMPPEDLVVYAPRWRGDSHLRFDAQQPFRVVRHPTTLMLPTPLVLRRAATLLRDERCDTVWFGAAAPLALMSPALRRFGADRILASTHGHEVGWSMLPGARQVLRVIGDHTDVVTYVSKYTRRRFAAAFGAQAALEYLPPGVDSTVFRPDPAARAELRARYGLGERPTVLCLSRLVPRKGQDMLILAMREIRRRIDGAVLVIAGGGPYEEKLRALVRALDLESDVVFTGRVPAAELAAHHTLADVFAMPSRTRGAGLDVEGLGIVYLEASATGVPVVAGNSGGAPETVLEGRTGTVVDGRDERAVAAAVVDILSDRDAAARMGAAGCEWVADQWRWDVLGGRLRGLLDPGAHSGVLGSDGSVR

>CORE_REP|Org194_Gene679#

MTQEVVERVEALLPTLRDRAQEAEDLRRLPEESVKDLQETGFFKLLQPRQWGGHAADPVVFYDTVRKIASACGSTGWVAGIIGVHNWHLALFDQRAQEEVWGEDTDVRISSSYAPMGAGLVTEDGSGYTVNGSWAWSSGSDHADWVVVGGPVIKNGKPVDFGSFLIPRTEYRIDDVWNVVGLRGTGSNTVVVENVFVPKHRFLSFRAMSDLKSPGLEQNTDPVYKMPWGTIHPTTISTPIVGMAYGALEAHVEHQGKRVRAAYAGENAKDDPFGKVRIAEAASDIDAAWRQLSGNVADEYAHLVAGREVPFDLRARARRDQVRATGRAISSIDKLFEASGATALANGTPLQRFWRDAHAGRVHAANDPERAYVMYGTHAFGLPIADTMV

>CORE_REP|Org101_Gene7468#

MNTSRGGQPAGWVSTGGRQPAEPAGWVSTGVAPQAGVHSRWPGLIAAYRDRIAGARDWEPVTLLEGGTPLVPAPHLSELTGCEVYLKVEGLNPTGSFKDRGMTVAITDAKYQGQKAVLCASTGNTSASAAAYATRADMTCAVLIPQGKIAMGKLAQAVMLGAKIIQVDGNFDDCLELARKVTSDFPSIGLVNSVNPARIEGQKTASFEICDVLGRAPDVHALPVGNAGNITAYWRGYREYYADGVTTQLPRMLGVQAAGAAPLVHGAPVSDPETIATAIRIGAPASWNAAVAAKEESGGAFRAATDEEILAAYRLVAASEGVFVEPASAASVAGLLAARTEGWLDSGLTVVCTVTGNGLKDPDTALLGMPQVQAIPVDPVAVAAELELA

>CORE_REP|Org15_Gene4571#

MDLFEYQAKELFVKHGVPSSEGRVTDSAEDARAIATEIGKPVMIKSQVKVGGRGKAGGVKYAATPDDAFTHASNILGLDIKGHVTKKILVAEAKDIAEEYYISFLLDRANRTYLAMCSVEGGMEIEEVAATKPDRLAKVPVDAVKGVDLAFARSIAEQGHLPADVLDAAAVTIQKLWEVFVAEDATLVEVNPLVRTPENEILALDGKVTLDENADFRHPDHAEFADRDATDPLELKAKENDLNYVKLDGEVGIIGNGAGLVMSTLDVVAYAGEKHNGVKPANFLDIGGGASAEVMANGLDVILNDAQVKSVFVNVFGGITACDAVANGIVKALEMLGSEANKPLVVRLDGNKVEEGRKILVEANHPLVTLAQTMDEGADKAAELAAAK

>CORE_REP|Org72_Gene4483#

MRPVAVISDAIAADTVSVPPEAATVTLADLPLRESLRGHTPYGAPQYDIAVRLNTNESPHPPSTAMIDDLLASIRAVAAELHRYPDRDALALRADLAAYVTRRTGVEVSADNIWAANGSTEILHQLLLAFGGPGRSALGVTPSYAMYRIAAECLGTAWLSVGEPADSAPDIEQMVTAITEYQPDIVFVTTPHNPTGALLAPSDLERLLRIAPGLVIVDEAYGEFSAAPSAIGLIDEYPAKLVVTRSLSKALAFAGARVGYLVATPAVIEAMLLVRLPYHLSTLGQTAARVALRHADEALARAAEVVAERQRVSRSLHEMGFRVRDSEANFLLFGPFPDPPRAWQRYREHDVLIRDVGIPRTLRVTIGSPEENDRFLAVSAGLLTGESH

>CORE_REP|Org210_Gene809#

MGTPVIVEAARTPIGKRNGWLAGLHAAEVLGAAQRGVLERAQLDPALVEQVIGGCVMQVGEQGNNVTRTAWLHAGLPWQVGATTVDCQCGSAQQANHLIAGQIAQGAIDIGVACGVESMSHVPLGANVGENAGPRRPASWDIDMPNQFEAAERIAKRRGITRDDIDEFGVRSQRLAAQAWAEGRFDREVLTIAGAPQVDKEGTLTGETLDVNRDQGLRETTRESLAKLKPVLEGGIHTAGTSSQISDGAAAVLLMDEQAAARAGLKPRARIVTQCLVGAEPEFHLDGPVQATTRLLEKSGMSIADIDLFEINEAFASVPLSWASVHKPDMDRVNVNGGAIAIGHPVGSTGSRLITTALHELERSDKSIAMVLMCAGGALATGTIIERL

>CORE_REP|Org215_Gene2838#

MRVAVVAGPDPGHAFPAIALCLRFLEAGDEPVLFTGPRWFDAAKQVGIGVRRLKGLAPRAEDDDADAGQRIHERAAFISTEILPDMSAMLPDLVVSDVLTAGGGMAAERMKVPWVELSPHPLYLPSKGLPPIGSGLAPGEGLSGRARDAVLRGMTARAIRQGEEQRERARAGIGLPPEDPGPAARLVATLPALEVPRPDWPDNAHLVGPLLWEPTANVLDLPPGDDPLVMVAPSTAHTGVSGMVDTVLEALDGAGVRVAISMLDTPPAELPPWATAGLGRQDELLRHAAVVIGGGGHGLLAKSLLAGVPVVTVPGGGDQWELANRAARHGSSLLVRPLTAEAVRTAVRRILDEPTFAERARKASADASVVSDPVPLCHEVAAAARTHR

>CORE_REP|Org6_Gene5593#

MSTPVPTRERRARRCFRNVWGMRLVAVGRVAMCVALGSALLAGCARFDDSASSPFTPEPTFSPADPRPPDQPPSSTTRPSGPCIDPDPSVVATCLDTTGGLVGVGHGALVAERRTGRILEVVDPDTPPVEVATVSVDGSGDGGLLDIALSPTYGEDGLIYAYITTGSDNRVVRLAEGGPPKDILTGIPKGATGNRGALEWATPDRLMVLTGDAGNPGLANSPGSLAGKLLRLDSPAPGSAAPQIVAAGIGTPGDLCRDGSDNVWFTDRTAVEDRLQRMDPSGAVSVAWTWPDRPGVAGCAVAADGVAVALTYAKALAIAPTDPNTLAVTTAPTLMVQDRYGQLGGATIGPDGTVWVGTVNKAEGTPGPNDDRVVRVPPPSGGGGGGPD

>CORE_REP|Org101_Gene5781#

MRRAARGGGDLSPAGPVGACTLTRMPLSAFVRTRNRTATPGSGSARVAPRRLAAVVALAATVVAAGCSKTDDASTIVRTTTNIAGAGVVGLERDTTRACPLPSAPDAANGSTRTVTHAAGVSEVPADPKRIVVLTTSALDATCAVGLWERVVGAVTIDGPSPQPAYLGTGVLKIPGVGTAAQPNPALIAAQHPDLIIGDIPTATASFDALQAIAPTVLVGANNSWQAEFTALAAGLGRKAAADAALEDYRTAATDTGNVLSSGQTQASVLRFTGDTNQIQGSNSFAGQILADAGVQRPQAQRGTTFDVRPDEFAGKLEGDLIYVLLAGADGKKHGEQVMRSAAFKDLGASTDKRVFAVEDTVWHGNGLTAARALLTDLTGTLNGFVTD

>CORE_REP|Org1_Gene5478#

MPGPVVVSDTSPTVPASTGLWSGIMNGFLLARPDGVLRASGVRAAFDAVNDARAALRTGGAAIVVGALPFDPARPAALVAPAEQVHTAGPWRPAALPPLPRVQVVSEFPSAGEHLARVTKLVEQLDDPDTELRKVVAARSVLAEADGVLEPETVAAQLAARHPGASVFAVDLTAAGRTGATLIGASPELLVARRGRTVTLHPLAGTAPRRADPDADAAQAAELLDSAKNREEHSYVIEWIRDVLTPLCTELRIPEGPRLVETHDVWHLATPIVGTLREPAPTALDLAVLLHPTPAVCGTPTAAALETITRIEGDRGFYGGAVGWCDADGDGTWVVAIRCAELAADGRSLRAYAGGGIVAASQPQAELDETTAKLRTFLGGLDCAVPTH

>CORE_REP|Org132_Gene4629#

MSDFLSTGTLPEEYRELALTVRDFANQVVAPVAAKHDAAHTFPYEVVSGMADMGLFGLPFPEEYGGMGGDYFALCLALEELGKIDQSVAITLEAGVSLGAMPIYRFGNEAQKQEWLPQLTSGRALAGFGLTEPGAGSDAGGTRTTAVRDGDDWIINGSKQFITNSGTDITRLVTVTAVTGESEGKKEISTILVPTDTPGFVAEPAYNKVGWHASDTHPLSFTDVRVPQSNLLGELGRGYANFLRILDEGRIAIAALSVGAAQGCVDESVRYAGEREAFGRAIGRNQAVAFKIARMEARAHAARTAYYDAAALMLAGKPFKKQAAIAKLVASEAAMDNARDATQIFGGYGFMNEYAVARHYRDSKILEIGEGTTEVQLMLIGRELGL

>CORE_REP|Org80_Gene5463#

MAGNPDFDLFKLEDFHDELRAAIRGLAEKEIAPYAKDVDGNARFPEEALTALNAAGFNAVHVPEAYGGQGADSVATCIVIEEVARVCGSSSLIPAVNKLGTMGLILNGSEELKQKVLGDLVNGKMASYCLSEREAGSDAASMRTRAKQDGDDWVINGSKCWITNGGKSEWYTVMAVTDPDKGANGISAFMVHKDDEGFVVGPLEHKLGIKGSPTAELYFENCRVPGDRIIGEPGTGFKTALQTLDHTRPTIGAQAVGLAQGALDAAIAYTKDRKQFGKAIADFQNTQFMLADMAMKVEAARLMVYTSAARAERGEQNLGFISAAAKCFASDVAMEVTTNAVQLFGGAGYTTDFPVERMMRDAKITQIYEGTNQIQRLVMSRALLKG

>CORE_REP|Org85_Gene5120#

MSFVETEEQQALRAAVAALAAKYNYRDYVLPKARANEPLTELWDEAGKLGFLGVNLPEEYGGGGAGLYELALVMEELSAQGAGLLLMVVSPAICGTIITKYGTDEQKQTWLPKLGDGSAKMVFGITEPDAGSNSHQITTTARRDGEDWILNGRKIYISGVDQAEAVLIVSRTEDHKTGKLKPALFIVPTDAEGFHKTPQEMDIIEPDHQFTLFLDDVRLPANALVGKEDAALMQLFAGLNPERVMGAAMAIGLGRYAIDRAVQYAKERTVWKTPIGAHQGISHPLAQVKIELELAKLMMRKAATLYDLGDEMGAAEAANMAKYAAAEASIKALDQAIQTHGGAGLTKEYGLAAMLAAARIGRIAPVSREMVLNFVAQYSLGLPKSY

>CORE_REP|Org103_Gene5508#

MSNTLRTLPMPTGSGVGDVLPHLREAMEGNGPAWLPIPTTDRREARRLADALRPGDPIDDDVALVVTTSGTTGVPKGAMLSSSALRASGTATHDRLGGPGTWLLALPTHHIAGLQVLMRSILAGTEPTVLDVSGGFLPEALAGAISGMRGERRYTSLVPTQLIKAIEEPEATAALADLDAVLVGGAATPAPVYERARELGINVVRTYGMSETCGGCVYDGVPLAGTLVRIEDGRVVLGGPMIAKGYRGQPDHPAFAEPGWFRTEDAGTYDNGVLQVTGRLDEAITTGGLLVIPQVVEAVLVTHPAISECVVLGLPDERLGQRVAVAVVPAEGARPTLEELREHVVRELDAIAAPRELAILDELPLHGPGKPNRNKLRELLLTRSHT

>CORE_REP|Org17_Gene4449#

MSTAITLGMPAAPAAVLAPRRKTRQLMVGTVGVGSDHPISVQSMTTTKTHDVNATLQQIAELTASGCDIVRVACPRQEDADALPMIAKKSQIPVIADIHFQPRYIFAAIDAGCAAVRVNPGNIKEFDGRVKEVAKAAGAAGIPIRIGVNAGSLDKRMLEKYGKATPEALVESALWEASLFEEHGFGDIKISVKHNDPVVMVEAYRQLAAQCDYPLHLGVTEAGPAFQGTIKSAVAFGALLSEGIGDTIRVSLSAPPAEEVKVGGQILQSLNLRPRKLEIVSCPSCGRAQVDVYSLANAVTAGLEGLEVPLRVAVMGCVVNGPGEAREADLGVASGNGKGQIFVKGEVIKTVPEHQIVETLIEEAMRIADEMGTDAETGDPVVTVG

>CORE_REP|Org135_Gene889#

MFTLNDDERAIRETARDFADEFLAPHALEWDEHMHFPIEVLRKSGSVGLGGIYVAEDVGGSALRRLDAVRIFEELATGCPAVAAYISIHNMAAWMIDAYGDDGQRFRWLPGMTSMEILGSYALTEPGVGSDAAALTTKAVRDGDDYILNGAKQFISGAGANDVYVMMVRTGEEGPRGISALIVPADTPGLTVGPNEKKMGWKAQPTRQVILTDARVPVANRLGAEGDGFRIAMNGLNGGRLNIAACSIGGAQAALDKTVPYLAQRQAFGAPLLKNQALQFDLADMRTQLEAARTLLWRAADALDADADDKVELCAMAKRFATDAGFEVANKALQLHGGYGYLAEYGLEKIVRDLRVHQILEGTNEIMRVVVARSMTSAARGAGAA

>CORE_REP|Org210_Gene5906#

MRDRACTKRFPEEQVNFKRSGALLGVLAAAGTLTLTACGSDDNSAATGNTTKVDVACGGKKALKASGSSAQKNAMDRFIAAYEQNCDGAKLDYTSSGSGAGVNEFVGGQTDFGGSDSALDPKKDEPKKAADRCGAPAWNLPTVFGPIAITHNLDGVTNLTLDGPTAAKIFNGTITKWDDPAIKGLNQGVNLPSDEIHVIFRSDESGTTDNFQRYLDAASNGAWGKGAGKAFAGGVGEGAKGNEGTSAAIKSTKGSITYNEWSFARSQNLSTAQIITDAAVKPVALNTESAGKAIAAAKIVGQGNDLIIDTNSFYKPTDPGAYPIMLATYEIVCSKYADADTGKAVKAFLTSAITNGQNGLEDSGYVPIPDAFKTKLTTAINAIS

>CORE_REP|Org144_Gene3837#

MLTHPVGSTPCYSPEPTSPFLVIYPERVRENYRALHAAMPAARIRFAVKASPVPELIQVLDEEGAEFDVASIGEIELCLELGVEPATLCYGNPIKKAADIARAYALGVRRYAFDTEDDLLRITEHAPGSQVECRFLASAPESRTPFGTKFGCAPAEALRLLVRARDLGLVVAGPYFHVGSQQLDPNAWRIGIEQAGRIVEALADKDIHVTSVNIGGGLPIAYADPAPALDEIATVVGTAAAEYLPATAALVVEPGRALVGSAGVIHAEVVGVRIAPDGRRWVYLDIGRYNGMAETENEYIAYRFVTDRDGDPVDEAVVAGPTCDGDDVLYQRTRVLLPTTLQAGDRVTILDTGAYTASYSSVSFNGFPPLTVHVSGAEPTRPAG

>CORE_REP|Org105_Gene5334#

MLCDTGHNPDSDVQEAVKEMLGKPSYPVQLIQPDGRRVLDREHAAVVADVGPDRLRDLYEDLVVTRRIDTEATALQRQGQLGLWAPLLGQEAAQVGSARALRPDDYVFCSYRESAVAYCRGVDPARLTRMWRGVAHSCWDPDAVNMTNPAIVVGAQGLHATGYAYAAHLEGADIATIAYFGDGATSQGDIAEALGFAASWSAPVVFFCQNNHWAISEPVRLQSATPIAQRALGYGIPSVQVDGNDVLAVLAVTRQALARAHAGGGPSFIEAITYRMGPHTTADDPTRYRSDAETEEWKRRDPIDRVHRLLDRENLLDEQFEQRVRDKADEIATVVRTATIDMPDPDPMELFDHVYSTEHPLIAEQRRAYAQHLAAHAPTEGVPS

>CORE_REP|Org112_Gene5694#

MSVVTRVTDLIGHTPLFELAATDTGTRLYLKLENLNPTGAAKIRMARAMVDDAEHRGLLSPGGHIIESTSGNTGLGLAVVAAERGYRFTAVVDHHACRDKLRAMKAMGAELVFVAEEGDDSLSTSAREELAEKMAREECEAALDAGTAPNAYFTEQHNNDANALGYYALADELLDELGRVDVLISAVGTGGSMFGTARRLRERGIEPLLYGVEPVGSIAFGGPAGPYWQSGTGTPEGADPGKIVAEDLGLLKEGVKVSDVEAFATARVLAAKLGLMIGGSAGGSVFAALRRLDDFPAGSTVVTIVCDGGEKYLDTVFDDEWMSDRDLLDAETERAVAALLDRLPSAARRDTASAADAARTPRATHTILLPADEREPVSGVAAK

>CORE_REP|Org105_Gene3199#

MAEVPGAERSLTTLSRVTLDLHADPIALTAALVDIPSVSRDELAVTDAVEAALRTQTTGFEIVRDGNVVLARTDRGLPTRVVLAGHLDTVPIADNVPSRMDTEGGEPVMYGCGTVDMKSGDAVFLHLAATIAEPAHDLTLIFYDCEEIAAEFNGLARIERERPEWLDGDLAILGEPSGGWVEAGCQGTLRARLTTAGTRAHSARAWLGDNAIHRLAPVLGRLSEYRAREVDIDGCVYREGLSAVRVAGGVAGNVVPDAAEVDVNFRFAPDRSVAQATDHVREVFAGLELDFQVTDAAPGALPGLTAPAAKDLITRVHAHGAAGVRAKYGWTDVSRFAARGVPAVNFGPGDPNLAHKRDERVPLAQITQVTAMLRSYLTGASS

>CORE_REP|Org6_Gene3417#

MTTSPAVPGGQATVPSDFVSGLEGVVAFTTDIAEPDKDGGALRYRGVDIEDLVGSRVTFGDVWALLVDGEFGHGLPPAEPFPLPVHTGDVRVDVQAGLAMLAPIWGYQPLLDIDDQTARENLARASVMALSYVAQSARGIYQPAVPQKKIDECNTVTERFMTRWKGDPDPRHIEAIDAYWVSAAEHGMNASTFTARVIASTGADVAASLSGAIGAMSGPLHGGAPARVLPMIEEVEKTGDARALVKGILDRKEKLMGFGHRVYRAEDPRARVLRATAQRLGAPRYEVAAALEQAALAELRERRPDRAIETNVEFWAAVILDFAEVPAHMMPAMFTCGRTAGWCAHILEQKQLGKLVRPAAIYTGPGPRKPAEVAGWSDISHL

>CORE_REP|Org105_Gene4411#

MLVRRGPRVSDEEAGVVAIRLLTGRSGERSSRMRSARRPGRTTRSGSWVKRAAIGVAAAMLVPMGVSIAGPAAPASAAFNPAGFDFWVDSGMGPIKSRIFRAKDGNTNRVVYALDGLRAPETLSGWEIDTNVAQLLTDWNINVVMPVGGMSSFYADWNAPSSFAGIPPGTGSSSGSGALNALAAGPGKSYRYQWETFLTQNLRWALRDRLGFNPNRNGVFGLSMGGSAALTLAAYHPDQFSFAGSYSGYLNISAPGMREAIRLAMLDAGGYNVDSMAPPWGPQWLRMDPFVFAPLLRDNNTRLWVSAGSGLPGPADGPTAGTVNGMALEALALANTRAFQLRMATLGANNVVYSFPNVGIHAWSYWAEEVARMTPDLSAHIG

>CORE_REP|Org105_Gene3985#

MDFSLTDEQQLLRDSVAGFLTARYELEKSRSAAKSVAGWQPEIWRGFAEELGILGATLAERVDGMGGGPTELMVIAEELGHALVVEPFVDTVVVGGGLLSRAGGEQADAVLRDIVAGSARIAFAALEPSAGESAHDISLTARRDGDEWVLDGSKIVVTSAPLATHLIISARTSGERRDRDGVSLFLTEFDTSAPSAGLEVHSYRTIDDRQAADLTFTGFRLPATALLGAEGEATANIEATLEEAIAAVAAESVGLMRKVVADTVEYSKQRQQFGQPIGQFQVLQHRMVDMYMELEQATAAAYLAAFALSASPSERARAISATKVTIARAARFIGQQSVQLHGAMGMTEELAIGHYFKRLTAIENEFGSSAYHLHRYARLTRP

>CORE_REP|Org43_Gene3725#

MAQNENIAALAAAGVSVWLDDLSRDRIRSGNLAELVRTRGVVGVTTNPTIFQGALSKGHAYDAQLKELAAQGADADADAAIRTITTDDVREACDVLAPLFEATGGLDGRVSIEVDPRFAFDADKTVAQAVDLWKTVDRPNLFIKIPATEEGLPAITAVIAEGISVNVTLIFSVQRYRAVMGAYLDGLRKARVAGHDLAKIHSVASFFVSRVDTEIDKRLAAIGTPEALELRGKAGIANARLAYAEYQDVFDGGAHTSTYQHLAAAGARRQRPLWASTGVKNPDYPDTMYVTELVAPNTVNTLPEKTLEAVADHGEIRGDTVSGTAAEAAEVFERLRAVGIDLDDVFAVLEREGVEKFEASWAELLSATAEELRAAASGSEGN

>CORE_REP|Org168_Gene2393#

MARAAWSDDEVEAVRELARNFFEKEVVPHEEKFVEQGHPDRHLYHRAGELGLLCTAIPAEYGGGGGTFAHEAAIIEEQTLAGDGALGMPVHSSIIAPYLAEFGSEELKRRVLPKAASGEMVLSIGMTEPGTGSDLQNIKTRAVREGDEYVITGSKIFITNGWLCDGIIIAAKTDPTKGAAGVSLIFAEVGDDTPGFTRGRILSKIGGKGQDTAELFFDGLRVPASNLLGEAEGQGFYQMMQLLAQERLVTAVIAVPMMEKAVQLTVEYTKGREAFGKPLYAMQNTKFELAECATIARVARTFLDDAIVKHLRGELDIPTAAMTKYWITDQLGGVVDRCLQLFGGYGYMTEYPISQLYTGARVLRILAGSNEVMKDLIARSL

>CORE_REP|Org63_Gene1320#

MTGDTGGSRAGTDAALVLEDGRVFRGQAYGAVGQTLGEAVFCTAMTGYQETLTDPSYHRQIVVAAAPQIGNTGWNDEDDESAKIWVAGYVVRDPARRASNWRATTTLPDELERQRIVGIAGIDTRALVRHLRTRGSMKAGIFSGDALAGPDELVARVNGQPSMLGADLAGEVSTDALYTIEPDGEHRCTVVAVDLGIKTNTPRMFAQRGMRVHVVSSSTPLEQILELKPDGVFLSNGPGDPATADAAVELTRGVLGKGLPLFGICFGNQILGRALGRDTYKMKFGHRGINIPVVEHETGRISITAQNHGFALEGERGERFDTPFGTAEVSHVCANDGTVEGVRLVDGRAFSVQYHPEAAAGPHDAAYLFDRFAGLMEGA

>CORE_REP|Org101_Gene2264#

MTRAADIDPRAHPSPGHHPSSNRCAMHADLDDQRTMSVSPLRSPAEVRRVHPITDELAGTVRKGRAATVDVLNGADDRLMVIVGPCSVHDPAAALDYARRLAAKAAELDDRLHVVMRVYFEKPRTTLGWKGLINDPHLDGSFDVNTGLGIGRKVLVDITALGLPVACEFLDPITPQYIADLVSYGAIGARTAASQVHRQLSSALSMPVGIKNGTDGDVQVAVDGVRAAAASHVFPGTDLDGRAALIRTTGNPDCHVILRGGSTGPNYDAASVAEACLRLEKAALPQRLVVDASHGNSNKDHNKQVDVVTDIAERLAAGEPGVVGVMLESFLVAGRQDLTLGKAADLTYGQSITDACLDWETTASQLDRLADAVAQRRNR

>CORE_REP|Org105_Gene3211#

MSTGLSSTGHAARVRVSSLLPDFPWDTIAGAKAKAAAHPGGIVDLSVGTPVDPVDPLIRAALNSVAEVPGYPTTHGTTALREAAVAALRRRYGITGIDQAAVLPVIGTKELIAGLPRLLGFGAGDLVVIPEVAYPTYEVGGLLAGTRIARADGLTQLGPESPALIYLNSPSNPTGKVLGVEHLRKVVAFARERGAIVVSDECYLGLSWEGRAVSVLDPEVCDGDHTGLLAVHSLSKTSNLASYRAGFVTGDAELIAELLEVRKHSGMMVPLPIQAAMTAALGDDAHENQQRERYRARRETLRTALLAAGFRIDHSEAGLYLWSSRDEPCRDTLDWLAERGILAAPGDFYGPAGAKHVRIALTATDERIAEAASRLGAG

>CORE_REP|Org105_Gene4449#

MPDFTESAFKATKATSIYFRYLRNSPMPAVPEARPRRRKVSRVRLLVTGGAGFIGANFVQQTVTERPEVTVTVLDALTYAGNRASLEPVADRIDFVHGDISDLDLVDELVSGVDAVVHFAAESHNDNSLTEPWPFVQTNIVGTYSLLQAVRRHDVRYHHVSTDEVYGDLDAADPAFTEQTAYNPSSPYSATKAASDLLVRAWTRSFGVRATLSNCSNNYGPYQHVEKFIPRQITNLIDGVRPRLYGAGHQIRDWIHVDDHNRAVWDVLERGRIGQTYLIGADGELDNKTVVRLILEAFGRDPDDFDHVTDRPGHDQRYAIDASLLRDELGWRPRYADFRAGLADTIAWYRANEDWWRPHKESTERAYAAAGEKTISPN

>CORE_REP|Org1_Gene658#

MRMTTAFPTIPDDLKPADGRFGCGPSKVRPEQLESLVRVGGSVFGTSHRQKPVKDVVARVRSGLRELFSLPDDYEVVLGNGGTTAFWDAAAFGLIRERSLHLTNGEFSSKFAAVAKGNPFIGDPIVVSAEPGSAPEPVADPAADLIGWAHNETSTGVAIPVQRPAGSEHALIAIDATSGAGGLPVTITDADVYYFAPQKCFAADGGLWVALMSPAALARVEEIKSSGRWTPEFLSLPVAIDNSTKEQTYNTPAIATLLLFADQIEWLNGNGGLDWAVKRTADSSSRLYQWAESSEYATPYVTDPAHRSQVVGTIDFADSVDAAQVAKILRANGIVDTEPYRKLGRNQLRIGMFPAIDPDDVSQLTRSIDWVVEKLS

>CORE_REP|Org161_Gene4102#

MEIVHPDVSADLAELDATLKTVESVLDIEELRRRIDELEHQAADPDLWNDQDHAQRVTSELSHAQGELRRVEDLRRRLEDLPVLYELAEGEEGEARTAALEEADAERAALHSDVEAMEVRTLLSGEYDKREALVNIRSGAGGVDAADWAEMLMRMYIRWADRHGYPVEVYDTSYAEEAGIKSATFAVKTPYAYGTLSVEMGTHRLVRISPFDNQGRRQTSFAEVEVLPVVETTDHIEVPETEIRVDVYRSSGPGGQSVNTTDSAVRITHIPTGIVVTCQNEKSQLQNKISAMRVLQAKLLERKRQEERAEMDALKTNEGASWGNQMRSYVLHPYQMVKDLRTNYEVNNPSAVLNGDIDGFIESGIRWRMRESQAS

>CORE_REP|Org30_Gene3953#

MSSHTDSRFAGDVYADRLERAVQLMRAAHLDALLITPGPDLRYLIGSAADSFERLTCLVIPADKSTPSVVIPKLELASLDGSAVSDLGLQVADWVDGIDPYQIVKSALHVGSRVAVTDSMPALHLLPLAESFSGLPVSATPVLRELRMIKDAAEIEALREAGAAIDRVHARMGEWLLPGRTEAEVAADIREAIVAEGHTEAEFVIVGSGPNGAIPHHMQSERRLQQGDVVVIDIGGPVPTGYNSDCTRTYVLGEPRSEVATRYAELEDAQAAAVAAVRPGVSAESVDAAARDPLKAAGLGAAFVHRTGHGIGLSVHEEPYIVEGNELELRPGMAFSVEPGIYFRGDWGARIEDIVVVTEDGCESMNQRPHGLTVL

>CORE_REP|Org1_Gene1712#

MCGDQEGVSVAHKASEEIGARPVRGRQGRILRRAGLAVSLGITAMLVSGCSIDNVWLRFGWPSGVTPQATRMRELWTWSIIAALAMGVLVWGLTFWTVVFHRKKKDSPEFPRQTGYNVPLELTYTAIPFVIIAVLFYFTVVVQNYVHEKVADPDVTVDVTAFQWNWKFGYREVDFKDGGYQFNGIDTAREEAAQAQLKEYEERVDTEHGHPQPGPVHGKPENDILSYLHYDTVETVGTSTEIPVLVLPTGKVIEFQLAAADVIHAFWVPEFLFKRDVMPNPKENHSDNVFQITEIEKEGAFVGRCAEMCGTYHSMMNFEVRAVSPEKFTRYLDERRAGKTNAEALAAIGESPVATSTRPFNTDRTVKSAAAPEAE

>CORE_REP|Org1_Gene3714#

MKIGMVCPYSFDVPGGVQAHVVELARVFLERGHKVSVLAPASEGTPLPDFVVSAGRAVAIPYNGSVARLSFGPMAYTRIRRWIDGNDFDVLHIHEPNAPSLSMLALKIAEGPIVATFHTSTTRSLVLSTFQGVLRPYHEKISGRIAVSELARRWQVEALGSDAVEIPNGVDVPAFARAPMLPGYPRPGGTVLFLGRYDEPRKGMQVLLAALPELVARHPDVEILVVGRGDEQRLRREAGRHARHLRFLGQVSDAEKASAMRSADVYVAPNLGGESFGIILIEAMAAGTAVVASELDAFRRVLRDGTAGMLVPVGDDVALAGALDTLLTDTERREALVRRANQVVGEYDWPVVAEQILRVYETVTVGDTRVRAAG

>CORE_REP|Org45_Gene5914#

MRVLAAMSGGVDSAVAAARAVDAGHEVVGVHLALSTAPGTLRTGSRGCCSKEDAGDARRAADVLGIPFYVWDFADRFKEDVIDDFVASYAAGETPNPCLRCNEKIKFSALADRAVALGFDAVVTGHYARLADGVLRRAVDADKDQSYVLAVLTAEQLARAMFPVGDTPKPLIREEAATRGLAVANKPDSHDICFIPSGDTRAFLGAKIGVRPGAVLDADGTKLADHEGVHGFTIGQRKGLGLPGPAADGKPRYVTDIDPETGTVRVGSAADLEVWTVLAERAVWTSGAVPDGPIECVAQVRAHGGTAPAVAEPADGGLVVRLRQPLTGVARGQAVVLYRPDAERGDQVLGSGTISGTERERHSYATELVSDATA

>CORE_REP|Org46_Gene6162#

MVDLINLVQTLTSPHPLDRYLELVRPTLTVRDMRAEITHVRRSAPGSVTLTLRPPRQWKGHVAGQYVQIGVVIDGVRHVRCYSPVNPEGGRDRRIQLTVKAHPDGLVSQYLYRHAAAGMVVDLTPADGVFRLPEPRPERVLLISGGSGITPVLSMLRTLAAEDHPGEVVFLHYAKSPAVLPHRAELDAIARRHRNFRIELRYPHRIQDVAPRVDPDAPWVDLAPVKGGGYFDYDELERVAPWFAAAQTYVCGPQSLMDAVRTIYQAEQLEDRLHTEEFTIALAPVDAAEAHGTVNFSASGVSARNDGATLLEQAESAGLSPEYGCRMGICFSCTAVRRSGCTRNLRTGETDSDPDQPIQLCINAPVGDVEVDI

>CORE_REP|Org102_Gene5145#

MADKLLLVTDEKLLQGPIHAVHVELGATFAPFGGWEMPVSYAGTVGEHTAVRTAVGLFDVSHLGKATVKGAGAAAFVNSALSNDLGRIRPGKAQYTLCCTDEGGVIDDLIAYYVSDDEIFLVPNAANTAAVVAELAKASPDGVTVTDEHREYAVFAVQGPKSVEVLTALGLPTEMEYMAYADAEWEGRPVRVCRTGYTGEHGYELLPRWADAEALFRALVAQVRAAGGQPAGLGARDTLRTEMGYPLHGHELSLEISPVQARAGWAVGWKKPEFWGKAALEQEKAAGPKRMLLGLKALDRGVLRQGQAVMRGDERVGETTSGTFSPTLKIGIALALLDTAAGLAEGDEVEVDVRGRRLRCEVVRPPFVQAKTA

>CORE_REP|Org5_Gene2511#

MIVHNHHMADTSSGPVLRVAVAGASGYAGGEVLRVLLGHPEYRSGRLEIGALTAGSNAGTTLGALQPHLLPLADRVLEETTAQVLAGHDIVFLGLPHGQSAAIAEQLPESTVIIDCGADFRLTDPEAWETYYQTPHAGSWPYGLPELPGARERLRGATRIAVPGCYPTVSSLALAPAVGAGIVEPEVTVVAVSGTSGAGRKLDVGLLGSEVMGSVRAYSIAGAHRHTPEIAQNLTAAAAATGADIGDVTVSFTPVLAPMPRGILATCTARLRPDAAGAVADPAAVRAIYDKAYGDEPFIHLLPEGVLPQTGSVVGSNAITLQVAVDTAARTLVVIGAVDNLTKGTAGAAVQSMNLALGFDEAAGLSTVGVAP

>CORE_REP|Org3_Gene1213#

MVSSVRVGGGRHLTSHDKYLILGDMSVIRSAGLRGFRATVAELGGDAEEFAIACGLPVAALDTDDMLVPDQAVSAVLELAAHRLDCPDLGLRMSARQDLAMLGPLALAIRSSPALADVLECSSRYLFVHARSLSLVLEPDPYGDRGVAALRYGVRAAAAIPIQGTDLGLAFVHRTIQRLIGDRYGLRSVELPYRPPAPLSVYEEFFGAPVRAGRRDALLRVPSSLAARQLSGGDENLHRLAMEFLAQQTAATGSSAVPTVRAAVKQLLGTTPPEIGVVAGLLTMHPRTLQRRLSAEGTTFAAVLDDVRRSETRRYLTTTDIAMSQIASLVGLTEQATLTRCCRRWWGHPPTAIRKDPALAREQTSVPATALT

>CORE_REP|Org102_Gene1037#

MNLNSAVTLNTEVVQDIEADMVDSGEAFDPAAHAAAMVGHHYRVADYYEVGREKVREYARAVQDYHPVHWDEDAAREYGYDGLVAPLTFISLVGILAQRKLFEQVVTGYDLSQIMQTDQILEFHRPIKAGDRLSCIVYLHSFRQAFGGDIIVTKNDVVAQNDELVLTTYTTLIGRSGGDIDPNLSDAVRNVLMHGIGPDERPDHQAHADAANQVHAAPVPVQQTQGDVPAKHAIRFDDVTVGQELPTRIVRLTRGDLVNYAGVSGDANPIHWSDDVCKLVGLENVVAHGMLTMGLGGGFVTSWLGNPGAVKEYNVRFTSPVYVPVDRAAEIEYTGKVKSMDPETRTAVVAIVAKSQGRKIFGRATATVQLA

>CORE_REP|Org198_Gene7039#

MSHDGAERQRGTVVRQILFAAAIALAVSILLTPLLIKMFAKQGFGQEIRVDGPASHQAKRGTPTMGGVAIIVGMWAGYLGSHLIGIGYNADGPSASGLLVLGLATALGGVGFVDDFIKIRKQRNLGLTAAGKYLGQLTSAVVFGVLALQFRGASGLTPASRHLSYVRDISTVTMGVVVFLVFVCLVVVAWSNAVNLTDGLDGLAAGSMSLVLGGYVVITFWQYYHACETKPETGCYNVRDPLDLALVCAAGAAACVGFLWWNAAPAKIFMGDTGSLALGGLLAGLSITTRTELLMIVIGALFVAETLSVVLQVAVYRTTRNRLFKMAPFHHHFELSKWAETTVIIRFWLLAAIASAVGLGLFYSEYLSAVG

>CORE_REP|Org103_Gene5490#

MSPDHDQPAERREPLEADNPAADTTADLTVALPAADATAAPTADSAVPVPSATEAEPGFDPDRPAPTVAEQAAPPDAPAAEAPAAEQAVAPDAPAAAQSAPTSAEETEAGFPTIDAPFSEAEARGSAAADADSPAVDRAARATVAHTETRAPQADSLDADDEVETIVHVLRHGEVHNPNGILYGRLPGFGLSVTGRAQAGAVARALADHDIALVIASPLQRAQETAEPIAAQHGLLVRTDENLIEAGNTFEGLRVSVGDGALRKPRHWWKLRDPFTPSWGEPYLQIAHRMLAAVNKARVEAAGHEAVLVSHQLPVWTLRRFLQGQRLWHDPRTRQCSLASLTSLVYRGDTLVDIVYSEPAGGSDPTVHGA

>CORE_REP|Org167_Gene3195#

MYALLLRVMFLVPPERIHHLAFAAMRVAARFAPIRALVRRLAVVDDPILHSTVFGVPFRAPLGLAAGFDKNAEGVDVWGPFGWGFAEIGTVTAQAQPGNPAPRLFRLPADRALINRMGFNNHGAARAAEQLRARTATVPIGANIGKTKVVEPAGAAADYATSAALLGPLADFVVVNVSSPNTPGLRDLQAVESLRPLLRTVLETVTDGGRSVPVLVKIAPDLSDEDIDAVADLAVELGLAGIVATNTTIRRDGLHTDPEDVAAMGAGGLSGAPVADRSLEVLRRLYRRVGDRLALISVGGIETPEQAYQRVLAGASLLQGYTGFIYGGPFWTRKIHRGLAELLRRDGYTSLAEAVGAEHRGPQPQADTA

>CORE_REP|Org159_Gene5055#

MFLVSLTLGIVGLPNVGKSTLFNALTKNDVLAANYPFATIEPNVGVVPLPDPRLNKLAEIFSSERIVPATVSFVDIAGIVKGASEGAGLGNKFLANIREADAICQVVRVFADDDVVHVDGRVDPSADIEVIETELILADLQTLEKAVVRLEKEAKVKKDRKPVADAAKAAQEILDSGTTLFAAADKVDTELLKELSLLTTKPFLYVFNADESVLTDEAKVAELKASVAPADSVFLDAKVEAELLELDEESAIELLESIGQTEPGLHALARAGFHTLGLQTYLTAGPKEARAWTIHQGDTAPKAAGVIHTDFERGFIKAEVVAYNDLLEAGSMAAAKAAGKVRMEGKDYVMADGDVVEFRSGVASPSKNK

>CORE_REP|Org22_Gene5576#

MDLQEFWFVLIGVLFTGYFVLEGFDFGVGMLMPVLGRGAVSDRGARGPSVVTQAAASGPDAHAADTRRRVVLNTIGPVWDGNEVWLITAGGAMFAAFPEWYASLFSGFYFPLLLLLVALILRICAIEYRGKIDDPVWRARCDLGIGIGSWVPALAWGWVFANIVRGVPLDADHQMTGSFLDLLSPYALLGALTTGLLFALHGAVFLSLKTGGEVREDAMRTGRLLLAPTALVVGGFGLWTQLAYGADWTWIPLGLAVLGLAVAAVAHFAERDGWAFTGTALVIVAASALLFGSLFPDVLPSTIDPAFSLNVDNASSTPYTLKVMSWAAVIVTPVVLLYQGWTYWVFRKRITVEQIPPGIGLSRQPVVEE

>CORE_REP|Org162_Gene5526#

MNRHDGAPAPGGATPPAGAGQQPASSADHRNAAPHPGGTTPHRSSERQLADAAQLTGAATARRAVPLLRDVTLRDGLQLTGKVLPTEHKVEIVRRLLGLGVPELEIGSLARPDLVPPMANSLEVVAALSPEELRRCWLWVATPRHVEKAAAAGARNFQYCFSVSDAHNRANIGRATEDSVAAMPAAVELARAVGGRIQLCLATAFTCPFDGPVDPERVLAIAADPRTAGADDVVLADTLGQAHPGQVAALVAAVRARNPQRRIVFHGHDTWGLGVANSLAAAAAGADVVDGSLGGLGGCPFAPGASGNTSSEDLLFATRPDWFTPAVLGELVRMSEALLTELGEPNRSRTVEGARSKAQAFEWVIRG

>CORE_REP|Org189_Gene3692#

MTAAAQFTRIPHPAPLPEQRVQEILTAPGFGRYFTDHMVSIDYTEAEGWTNARVEPYGPLSMDPATMVFHYGQAIFEGLKAYRQPDGGVSCFRIDANAARFRRSARRMAMAELPDELFIESVRQLLEVDERWVPAAGGEESLYLRPFMFATESGLGVKPAAAYKYLLLGSPAGAYFPRGVKPVRVWLSTDYVRAAPGGTGEAKVAGNYAASLLAQAEATEKGCDQVVWLDACERRYVEEMGTNNLFFVFGSGSDARLVTPELSGSLLPGITRDSLLTLAADSGYQVEERKISVEEWRKGAESGEITEVFACGTAAVITPVGWVRSGDEEFAIGGGEPGEVTMALRETLTGIQRGTFADIHQWMRRL

>CORE_REP|Org126_Gene4339#

MRYGTARAAGLLIGFAADRIFGDPRRGHPVALFGTAAAAVESAGYRDARAAGVVHEIVLVGGVVALSAGAEIAARPSGITRGSGVVSAWRNRHGGVAGGRRGGCAGVRTAVVQGRPGRHAGLGTIVITAVGTWIALGGTTLARTGREMADRLEAGESTGAREVLPSLCGRDPEALDADGLARAAVESIAENTSDATVAPLVWGAVAGVPGLLGYRAINTLDAMVGYRNERYSNFGWAAARVDDLANLLPARVSGVLTVMLAPLVGGRPDDAWRAWRRDAGAHPSPNAGVAEATMAGALGVALGGRTEYRHGTEMRPVLGDGRTPRVPDLRRAVRLSNGVQLTAAVVAAVTAYALGRRRSARAELPE

>CORE_REP|Org163_Gene5601#

MDSYQGEGSTVTQPSAIDDILAEYAGLETQLADPSLHNDAGAARRVGKRFAELAPVMATYRKLETVRGDLSAAQELAADDAAFAAEIPDLERQVEELEQALADLLAPRDPHDGDDVVLEVKSGEGGEESALFASDLARMYVRYAERHGWKVEILDVALSDLGGYKEATLSIKSRDASRDGVWSRFKFEGGVHRVQRVPVTESQGRIHTSAAGVLIYPEPDEIEEVQIDESDLRIDVYRSSGKGGQGVNTTDSAVRITHLPSGIVVTCQNERSQLQNKARAMQVLAARLQALAEDQADQEAAAGRASQIRTVDRSERIRTYNFPENRITDHRIGFKAHNLDAVLDGDMDALLDALGKADREARMAAE

>CORE_REP|Org102_Gene3743#

MSEAVDADNSTATSADPSPVMNDSGVAPVVAGIGAEEPSSPVDLSAAVDVLDTARSVTVLCHVQPDADTIGSGLALAQVLHRRGVPVRVSFAEPAEVPVSMRSLPGIELLVPPDRVPAEVDVLVAVDCGSVGRLGTLRDRLAGARVSLVLDHHRSNTRFGTVNVIDESAESTAGLVVRVLDAWGESIDRPIAHCLFAGLVTDTGSFKWPRPGSHTLAERLLATGIDGAAITRTLMDTHPFAWLPMLSKVLGSARLEPAAAGGAGLVYAFVRRDDTAGVRSEEVESVVDIVRTTAEADIAAVFKESRTTPDLWTVSLRSHARPDGTPGVDVARIATTLGGGGHRYAAGYTTSGTPDQLVATLLAELG

>CORE_REP|Org145_Gene4685#

MNTVQVARPPRARGAVALRRSPPEGISAVTQAPVQTDILEIAREQVLERGEGLTQDQTLAVLRLGDDRLEELLGLAHEVRMKWCGPEVEVEGIISLKTGGCPEDCHFCSQSGLFQSPVRAAWLDIPSLVEAAKQTAKTGATEFCIVAAVRGPDARLMAQVAAGVEAIRNEVDIQVACSLGMLTQEQVDQLAAMGVHRYNHNLETAKSHFPNVVTTHTWEERWDTLRMVREAGMEVCCGGILGMGETLEQRAEFAAQLAELEPDEVPLNFLNPRPGTPFGDLEVLPAAEALKAVAAFRLALPRTILRFAGGREITLGDLGAKQGILGGINAVIVGNYLTTLGRPAESDLDLLGELKMPIKALNETL

>CORE_REP|Org144_Gene6103#

MAARIAQTSGAEHTAILGLGVYRPARVVTNDEVAGPINSSDEWIRTRSGIKTRRFASAVETVQSMSVAAARGALESAGVDADQVDCVIVATSTHLLLTPAAAPRIATELGMNGSAAFDVSAGCAGFCHALALASDLVRCGTAGHVLVIGVEKLTDTINPTDRSTAFLFADGAGAVVVGPSDVPGIGPTVWGSDGTQAHAIRQDKDWVEFFREIEEKGTDAVRPYLAMEGTAVFRWAAHSLEKVCRDAVDRAGLSTDDLNAMIPHQANGRIIEIMARVLELPENCALANDIEETGNTSAASIPLAMESLLRKGESQPGDTALLIAFGAGLSYAAQVVTLPRFAAPAAPITADTSDIESVDAEAATV

>CORE_REP|Org106_Gene362#

MNAPTPRLRTALTDLVGIEHPVVQTGMGWVAGPSLVSATANAGGLGILASATMTYEELEAAIAKTKAQTDRSFGVNIRADASDANERIDLLIRERVKVASFALAPKKDLIAKLKDAGVVVIPSIGAAKHAVKVASWGADAVIVQGGEGGGHTGPVATTLLLPSVLDAVDIPVVAAGGFFDGRGLAAALAYGAAGVAMGTRFLLTQDSSVPDAVKQEYLNRHLQDTVVSLKVDGMPHRVLNTELVQRLEHSGRWRGFAAAVSNAARFKSMTGMKWSTIVKDGLAMRKTKDLTWSQVIMAANTPMLLRAGLVEGNTQAGVLAAGQVTGIIDDLPTCKELIERIVTEAEERLDALASLRASAQPDASG

>CORE_REP|Org216_Gene3268#

MLRRSLLRGLLKCKCVRCRRRHSLVRHRAVRPGRCGGAAARRCGMVVLNPEGPSAQARLLTAACRGVVRPVLRAAPITRATIPVGALAIDGLARLRPHPRGIEREQVTMPGFAMEIIRPAGAARAMRHGALLYLHGGGFAVCGLETHRPVAASLARRTGLPVVNVAYRQLPVRSITESIDDCLAAYRWLLRHGAEPDRIVFAGDSAGGYLTFATALRALECGLPAPAGLVGLSPLLDLDYAAKRDYVNVARDPYIPLSALAAVVRLGAEREGRLDPLLSPVNGALAHLPPVLLVAAEDEVLRFDAELMAARLDAAGVPNSVELWRGQVHAFMSIAPGLPESRAALGRVARFVRGRLADSQRARTA

>CORE_REP|Org81_Gene5027#

MRGTNTAEPEVLIERRDGLGLITLNRPKAINALNHPMALAILDALREWAADDEVRTVVLTGAGERGLCAGGDIVAIHNDAKNAVAQADTAAADSPSGRFWRDEYMLNALIGRYPKPYVAVMDGIVMGGGVGLSGHASHRIVTERSKIGMPETGIGFIPDVGGTYLLSHAPGEIGTHVALTTARMSAGDAIAAGFADYFVPAEQLPALLEALRDNDADTAIAKFAQAAPESELMAQRDWIDACYSADSVEEIVARLRTHDAPEAAKAAADVLTKSPVALKVTLRSLRNARAATHLEEVLNEEYRVSVASLSTHDLVEGIRAQVVDKDRNPQWNPATLADVSVAEVDTYFAELGDKELGLTAPEGK

>CORE_REP|Org152_Gene6790#

MSGFPRERRKPRQDETGSGTAGATPRDEPSKACGECPGNQRRECDPQVTTIRELRSRIRAASSVRKITKAQELVAASRLTKARARVAAAEPYAREITRVLTELASASTLTHPLLTERPAPRRAAVLVITSDRGMCGSYNARVLERTEELLTTLRTAGKEPVLYVMGAKGLTYFGFRRRPVDGSWTGFSHSPTYADAADACRHLVDAFMAGADGDVSTPDGTGSMAGVDELHIVHTRFVSMLSQVPEVRRLAPIQVTFADESFEMGPDSFSDSPTAEVHAQYEFEPDADRLLSALLPKYINARIYASLLDAAASESAARRTAMKAASDKATNVVDSLTRSANSLRQAQITQEITEIVGGAEALA

>CORE_REP|Org210_Gene1197#

MNDADHSDDTSASGKSGDSEEPTLVALGGGHGLYATLTAARRLTERITAVVTVADDGGSSGRLRAELGMLPPGDLRMALAALAEDPDGVWARTAQHRFGGTGALAGHSVGNLVLAGLAEVLGDPVAALDEMAAILRCVGRVLPMSPTALTIEADVSGLEADPRVSRCIRGQVAVATTPGKVRRVRLIPSDPPASPEATSAIEHADVVVLGPGSWFTSVIPHMLVPDLREALMDTHAVKVLVLNLAAEPGETTGFSAERHLHVLSQHAPDFAVDHVLVDSGSVPEGREREHVARAAEQLRARVTFADVAEAGTDRHHPGKLAAALDQVIRQPRPELAGLRVEGRHPVQQVRSVLGGKERVSWR

>CORE_REP|Org169_Gene6025#

MHSSGRRRTGRWTGADPGRAARPKRSSYYTGPVSFDNVRGRSTPGRPSRTPSGTPSVVGQLGRRPDGTIPFSVEFNPPRDAAAEARLWRAAREFERMHPAFVSMTYGAGGSTRDRTARITGQLARETTLLTVAHLTAVGHSVAELRSIVGSYADAGIRNMLVLRGDPPGDPLGEWRKHPDGVEYAEELVRMVCELGDFHVGVASFPQGHYRSPDLEHDTRYLVSKLRAGAEYSITQMFFDVEHYLRLRDRVAAYDAEQGAKPIIPELMPITSLRTVQRAEELSGRPLPARVMQRLEQAAGNDPEANRNAVRAVGIEIATEIGQRLIDEGAPCLHFITLNFAKATTEVLTNLGYTVTPAAVSA

>CORE_REP|Org5_Gene5551#

MLRVAWVVVSRCRADCVPRSCLPSSVGASVRRCVAGPLAAARWLARGGYVGDGGGMEELPGDLRDLLDQYERHLRLGRNRSAHTVRAYLGDARALLNHLCDRSPDASVGEIDLPLLRSWLAELAAGGAARTTMARRASAARTFTAWLTHTGQLVADPGPRLGSARAHRVLPAVLGRDQADAAMTAAESGAAQQDPMALRDRLIVEMLYATGIRVSELCGLDIDDVDRARRLVRVLGKGNKERSVPFGGPADRSLEAWLNFGRPHLATAESGRALLLGRRGRRLDQRQARTVVHDVVSAVPGAPDLGPHGLRHTAATHLLEGGADLRVVQELLGHASMATTQLYTHVSIERLRHVHDQAHPRA

>CORE_REP|Org99_Gene2641#

MSQAKKGGLGRGLAALIPTGPDTIPNGLTTPTAPPGTAKPKGLVTPNGLGTAAANVIIGVDPAGAKPASPLRSDTQAEELTSPSGAVYREIPPDQIEPNPKQPRQVFEEDALAELVHSIREFGLMQPIVVRRLEPGVDKYQLVMGERRWRACQEAGLEAIPAIVRETADDALLRDALLENIHRVQLNPLEEAAAYQQLLEEFGVTHEELAARIGRSRPVVTNMIRLLKLPIPVQRRVAAGVLSAGHARALLGLEAGPDAQEALAARIVAEGMSVRATEEAVTLANREPDSAATPPAPKRKPIHMPGLQDVAEKLSNSFDTRVTVSLGKRKGKIVVEFGSVEDLERIVGLMQQQQLQMSSE

>CORE_REP|Org4_Gene703#

MGVDLPRVAEPGKIAAMRIGVLTGGGDCPGLNAVIRAVVRTANGRYGDAIVGFEDGWRGLLEDRKIQIHNDDRTDRLLAKGGTILGTARTNPDVLRAGLGRIKRTLDDNGIDALIPIGGEGTLTAASWLSDEGVPVVGVPKTIDNDIDCTDVTFGHDTALSIASEAIDRLHTTAESHQRVMLVEVMGRHAGWIAVNAGMAAGAHLTLVPEVPFDVDQVCTMIKRRFQRGDKHFICVVAEGSHPAEDSGFALRAGGIDEFGHERFTGVAQQLGAEIERRIGKEVRTTVLGHVQRGGSPTPYDRVLATRFGLHAAEAVHAGQFGQMVALHGSAIELVPLSEATKQLKRVPSERYQEAEAFFG

>CORE_REP|Org5_Gene6478#

MLWERRACVSGRGARGHVHARKLVHMSSAIPLNVGIARSADASGVGAGKRVLLAEPRGYCAGVDRAVETVERTLEKHGAPIYVRKEIVHNRHVVETLRDRGVVFVDETDEVPEGSVVVFSAHGVSPAVHESAAARNLHTIDATCPLVTKVHQEAKRFARDDFDILLIGHEGHEEVEGTAGEAPDNVQLVDGPDAVDGVHVRDEDKVIWLSQTTLSVDETMETVQRLRARFPKLQDPPSDDICYATQNRQVAVKAMAPECDLVIVVGSRNSSNSRRLVEVALNAGAAASYLVDFAREIDPSWFEGVRTVGVTSGASVPEILVRGVLDLLAEHGYGEVQPVTTANETLVFALPRELRTSARR

>CORE_REP|Org31_Gene4493#

MPGGHNGQVTLVEMGIPAVRSGRPSLDGRPDTPVLLDRFGRVARDLRVSITEKCSLRCTYCMPEEGLPAIPQDELLTVAEIVRLVRLAVRELGVQEVRFTGGEPLMRRDLEQIIAGCHEQVPHVPLAMTTNGVGLEHRARGLAAAGLHRVNVSLDTVDRAGFATLTRRDRLGSALAGIRAARDAGLAPVKINAVLMRETLSGAADLLQWCLDEQCELRFIEEMPLDADHEWARANMVTAAELLEVLGTRFALTAAGRADPSAPAETWLVNGGPATVGIIASVTRKFCDTCDRTRLTADGMLRSCLFSDQEYDLRRVLRSGADDHELATLWRGAMWNKWAGHGIDAEGFVPPERTMGAIGG

>CORE_REP|Org4_Gene2114#

MAAHRRQGTLRLDTKRLVGGALAAGVLATTTVYGAGPVGADPVALPATAADAVQRMVDLSRQSEQLNEQALNAQSDLDTKLGLQREADAKLAASTDQVNRARDEVRKYQPIIDRTAIAAYQGARTNRLFAVLVSDSPQQLLDQMSTLDVLAAQTSDQLALYKKATDAAEGAEADARRASDEARAAADKAETVRGELERKRSDLSGAIVQVVQAWTGLSTKDKSALAGPAFPPGFDRDTLLQGLVPGSGTSALAASLTRIGDPYVWGATGPHQFDCSGLVQWAFKQVGKDVPRTSSQQASYGTPVAQNDLQPGDVVFFYNDISHVGIYAGNGLMVHASTFGVPVAVAPISTTPYHSARRY

>CORE_REP|Org134_Gene157#

MHDRGGTVGRTADALLLLSFGGPERPEDVMPFLENVTRGRGVPRERLAEVADHYLHFGGVSPINELNRQIIAAVEDELSAAGTDLPVYFGNRNWHPMVEDTLAQMTADGVRSALVFPTSAWGGYSGCLQYQEDIVRARGAVGDGAPELTKLRQYFDHPLFIESFADAIRAAVQQIPADRRDRIRLVFTAHSIPISADVSAGPPADGGRLYSRQVSDAARLCVAATGFTDYDLVWQSRSGPPQVPWLDPDIVDHLENLSGKGVDAVVVCPVGFVSDHLEVIWDLDNEAAEKAAELGMAFARAGTPGTDPRFAQLVVELIREQMDDAPARCLGSVPGYGGTVNGQACAVDCCKPPARPGR

>CORE_REP|Org210_Gene2958#

MLECSREQAEVLVPERSKDARTGFIAGRFGEFPARWGQGLSDQLTRIARSPFGPSEEEVRANLAGEASADAALALHDAELAESYGAEADSELPPHARPPRDLTAAAFFDVDNTMVQGASIVHFARGLAARKYFKTSDLVDMAWKQVKFRVTGKESQGDMASGKEKALSFIAGRSTAELAALGEEIYDEIIADKIWPGTRALAQMHLDAGQQVWLVTATPVELAQVIAKRLGLTGALGTVAESVDGVFTGRLVGDILHGLGKAHAVRTLAIREGLNLKRCTAYSDSHNDVPMLSLAGTAVAINPDSDLREVAKNRGWEIRDFRTGRKAAKIGVPTALALGAAGGAAAAVLTRRREQHG

>CORE_REP|Org38_Gene4207#

MVREQTLSLETDGTQQTATLAGQRFRVRDHYEVGREKIREFARAVQNHHGAHQQESDALRLGHENVIAPPTFASVIGSAGTRSLLESVLTEYDLSQILQTDQVFQAYRPIQAGDRLSSEILIESIRQFGDNDFVVVRSALLNQHGELALIGSTTIVARRGVDVDPSVADVVDNIMMHGQVVEFPSAGSEAADGSALIPLGTGTLPGPAPDRVPAPVHTLPDFDRLSVGDQLAPGIFRLTRGDLANYAGVSGDANPIHFSDHAAELVGLPTVVAHGMLTMGLASGYLTAWLGDPTAIEKFSVRFAGFVPVAPNAASTVEFTGRIKALDPRTRTATIVLGGTSEERKLFGRAIAEVRFS

>CORE_REP|Org81_Gene3324#

MAALGAMMPLTERITDGPSGFGGVEATPEWAREIKRLARERNATILAHNYQLPEIQDVADHVGDSLALSRIAAEAPEDTIVFCGVHFMAETAKILSPEKTVLIPDQRAGCSLADSITADELRAWKAEHPGALVVSYVNTTAAVKALTDICCTSSNAVDVVASIDADREVLFLPDQFLGAHVKRVTGRENMHIWMGECHVHAGINGDELNEQARTHPDAELFVHPECGCATSALYLAGAGEFPADRVHILSTGGMIDAAKAAAARSSRSGESGTVNQVLVATEVGMLHQLRKAAPGVDFQAVNDRASCKYMKMITPAALLRSLVENRDEVHVDPETAALARNSVQRMIEIGNPGGGE

>CORE_REP|Org15_Gene6315#

MGVSVQVPEWTTELAPATAARIVELLERAGAADGVAPISEQAVLSVTASGTVESATAPAGPAATDSGSAAPASPETDPAAADHDHGDVEPPTGRTRHLPVERDGELVAYANLVPAHGDHPAMAEAVVDPRARGRGIGATLVAAALQAGGPGARIWAHGNLAPARAVAGRLGLTIARELWQMRRPLTQAPDGDQATVELPELEVPADIVLRTYAGPADDAEILRVNNAAFDWHPEQGGWTEAEIAVRRAAPWFDPKGLFIAADPADPSHILGFHWTKVHEPDETSDAVGEVYVVAIDPAAQGRGLGRVLTLAGLHYLRDRGLGAVILYTEADNTAAVHTYTRLGFETAHIDAAYTAR

>CORE_REP|Org207_Gene1186#

MIVAQEQGNVGAAATGTPLRIGTRGSLLAMTQAGTVRDALIAAGRPAELVVVKTPGDMSSDPVQKIGVGVFTSALRDELAAGTIDLAVHSYKDLPTAPDPRFVIAAIPPREDPRDALVARDGLVLGELPAGAKVGTSAPRRVAQLRALGLGLDIVPLRGNLDSRLARVTDGELDAVVVARAGLSRIGRTAVITEALEPVQMLPAPAQGALAVECRSEDAALIEALAELDDAATRAAVVAERALLAELEAGCTAPVGALAEVVESLDDDGRIVEELSLRGCAAAVDGSEVLRASVVGDPERAAELGRALARELLELGARELLVEVAATEPGARSGTGAVRPPETDLSNPSPMENPQ

>CORE_REP|Org129_Gene2372#

MSTPTRRRLPDAPFLAAATGAGPGRRPVWFMRQAGRSLPEYRELRAGIGMLESCFDPELVCEITLQPIRRHGVDAAILFSDIVVPLKAAGIELDIVPGVGPVIANPVRSVDDVRALPRLRREEVGAITDGVRLLLDELGETPLIGFAGAPFTLASYLVEGGPSRNHERTKALMLGDPQTWHALLGVLTDITVEFLRAQIAAGVDAVQLFDSWAGALSLAQYREFVLPHSERVFAEIGEAGVPRIHFGVGTGELLGAMGEAGADVVGVDWRVPLTAAARRVGPGKALQGNLDPAVLFAGSSVVEREIRRIAHEADEAITLGATGHIFNLGHGVLPDTDPGAITAAVELIHSLPTPL

>CORE_REP|Org125_Gene5591#

MTVHDEVTAAGQSDSTGPRSGNEVPPGDTSSMPSESSNKPAGRDTGGDSRRGETVFRSLATAAGATIVAAIALIALFLLIRAVPSVAANKANFFTSAEFNVTNADNMHFGIRDLFMVTVLSSLLALLIAVPLGVGIALFLTQYAPKVLSRPFAMLVDLLAAVPSIVFGLWGFLVLAEKLAPFEQFLNDKLGWFFLFKDGNVSISGGGTIFTAGVVLAVMILPIITSVSREVFHLTPRAHIEAAQALGATKWEVVRMTVLPYGRSGVIAGSMLGLGRALGETIAVLIVLRTAASPGHWSLFDGGYTFASKIASAASEFSQALPTGAYIAAGFVLFALTFVVNALARIAAGGKVNG

>CORE_REP|Org139_Gene5133#

MGYRCRFGTVVRIRSAGVDQRSVPEAAIIRQALDEIGRVIVGKQQAVQSIMAAVLAGGHVLIEDLPGLGKTTIARTFAAVLGLETTRVQFTPDLLPADLVGATVYNAAAGRFEYRPGPIVTNVLIADEINRTPPKTQSALLEAMAEGQVSTDGVTRPLPAPFLVIATQNPIEHEGTYPLPEAQLDRFAMCLGLGYSTAAQEKALLRQRMSAAGGVRPRQIADAATVDRLRAATSRVDVDDDILDYIVALVRATRTHVQVEVGASPRAELDLLQVARAHALLQGRDFVIPEDVKTVAPEVVSHRVSLRPEAWMRRIRGRTIVAEVLSRTPAPRLREAAASAAGALGLAGAVDSR

>CORE_REP|Org5_Gene5803#

MSRRSSSRFRFSARRGRIAVALTALAAVGLTAACSGGSSDTPGGATPGGGAGNLTLFAYSVVKPGYDKVIAEFNKTDPGKGAQIQQSYGASGDQSRKVKDGAQADVVSFSVEPDITRLVDAGIVDSNWNADANKGVPFGSVVVMAVRKGNPKGIHDWNDLLKPGVEVVTPNPFSSGSAKWNLLAPYAAESNGGQNPQAGLDYLGKLISKDHIKVQPKSGREATETFLQGTGDVLLSYENEAIFSERSGDPIEHVIPPTTFKIENPVAVTKNAKNPAAAVAFKDFLYSQAGQKAWAEAGFRPVDPQVAEQYAKDFPKPQKLWSIADLGGWKQVDKQLFTPDTGSVAVLYDKATK

>CORE_REP|Org64_Gene326#

MLISQRPTLTEEVIAENRSKFTIEPLEPGFGYTLGNSLRRTLLSSIPGAAVTSIRIDGVLHEFTTVPGVKEDVTDIILNLKGLVVSSEEDEPVTMYVRKQGPGTVTAGDIVPPAGVVVHNPDMHIATLNDKGKLEIELVVERGRGYVPAVQNKASGAEIGRIPVDSIYSPVLKVTYKVEATRVEQRTDFDRLILDVETKNSISARDALASAGKTLVELFGLARELNVEAEGIEIGPSPAEADHIASFGLPIEDLDLTVRSYNCLKREGVHTVGELVARTESDLLDIRNFGQKSIDEVKVKLHALGLSLKDSPASFDPSSVVGYDASTGTWSDSGTFSDNDGGEQDYAETEQL

>CORE_REP|Org114_Gene7067#

MSGESGRLHDAKTREILLVAHPGRAELTETAHRVAKIFEQFGIGLRVLADEAYSTRFDTDEQGRPDGYPVRVMEHGPEAAIGCEMVLALGGDGTFLRAAELAREANVPVLGINLGRIGFLTEAEAENLDEALAQVVRRDYRIERRMTIDVTVRVDDTITERGWALNEASIENSARMGVLEVVLEVDGRPVSSFGCDGVLVATPTGSTAYAFSAGGPVVWPELEALLVIPSNAHALFARPLVTSPDSRIAVETVATGHDAIVFLDGRRTLALPRGGRVEAVRGTEPVLLVRLDSAPFADRMVRKFQLPVTGWRGRSSARMGAEEEARVPTQRPRAGDVEHRRTESTSADRDQD

>CORE_REP|Org144_Gene5066#

MSTSAVSEASERAIDAACDESTVVAGVDLGDTDLAATVRAGLEEVEKLLVAELSDGEEFLQEAALHLAKAGGKRFRPLFTLLTGQLGPRASDPALVTAGTVVELVHLATLYHDDVMDEATVRRGAPSVNSRWGNNIAILAGDYLFAHASRLTSTLGPDAVRIIAETFAELVTGQMRETMGARETQDPVEHYLRVVWEKTGSLIAAAGRFGGTFSGASLDHVERLARLGDAVGTAFQISDDIIDISSATEQSGKTPGTDLREGVHTLPVLYALRDEGADGDRLRKLLAQPLSTDAEVEEALELLGRSHGMVLAKEKLHGYADLAHAELSALPQGPANDALERLVRYTIERVG

>CORE_REP|Org13_Gene6761#

MRFGRSAAPVSHSALAPESAPRGRRGAQRGWRTRLLAAGAAMALPIAAGIMAPAAIAAPVHAPVHQTPAGGYDELMVPSSMGPIKVQVQWARNGGNAALLLLDGLRARDDRNAWSFETNAQQMFGNDNVTLVMPVGGQSSWYADWQGPSNTNGQKFTYKWETFLTKELPDFLSNYGVSRTNYAVAGLSMSGPAALRLAAFHRDQFKYAASFSGPLNWNAPGMREAIRVMMLDAGRFNVDSMAAPWSPQWLRSDPMVFAPQLRGLPMYISAASGLPGQYDHPNGLVGAFNTGNAMGIELISMVSTHSFKARLDSLGIPAAYDFPPTGTHAWLYWQDELAKARTGILAALNA

>CORE_REP|Org35_Gene172#

MTEHHEAPGGASKALRSSDGPEGGSSRNHEAANRTHKDIPQATVARLATYLRVLAMLADDGVLIVSSEELAVAAGVNSAKLRKDLSFLGPNGVRGVGYDVAKLRTRIEDVLGLSEGHRVVLVGAGNLGRALVGYGGFRRRGFTVVGLFDNDPALIGRTVAGLRVRDAAELDAVIATLEPTIAVIAVPDDAAQQVCDALVAAGLQSILSFAPCELVVPATVEVRRVDLAVEMQMLSFERVRNAESDRWPSDGHAGGSERNHGGPREATDSIQPAAVGERGDSIPVAHPLGSGRVARRAANTHPQPAHPGRAHPAAATQHPATPQHPATPQHPATSHSATEPTSKGSVVTP

>CORE_REP|Org4_Gene1586#

MSEPTGQDTPIETGAAAQVDSASATPGDPAAEPVVGGSDSSAVTPAGTGPDARIVAVVVTHKRRELLAESLKVIASQSRPVDHLIVIDNANEAEVAELVRDQPIESTYLGSAHNLGGAGGFALGMLHALSMGADWVWLADDDGRPDGAEVLATLLDCARRHGLVEVSPVVCDIDEPDRLAFPLRRGVVWRRLRSELGDEDFLPGIASLFNGALISAKAVDVIGVPDLRLFVRGDEVEVHRRLVRSGLPFGTCLQTAYLHPNGAAEFKPILGGRMHTQYPDDPVKRYFTYRNRGYLMSQPGMRKLLPQEWIRFSWFFLVTRRDPAGLREWFHLRSLGRHEQFGKPDPRG

>CORE_REP|Org37_Gene1714#

MTAPTPATSRRREAPDRNLALELVRVTEAGAMAAGRWVGRGDKEGGDGAAVDAMRQLVATVSMRGTVVIGEGEKDEAPMLYNGEAVGDGTGPEVDFAVDPIDGTTLMSKGSPGAIAVLAVAERGAMFDPSAVFYMEKIAVGPEAADVIDLSVPIAENLRRVAKAKNSLVSDLTVCVLDRPRHARHIQEVRDAGARIRLISDGDVAGAIACARPESGTDMLVGIGGTPEGIIAAAALRCMGGALQGKLAPTDDAEKQKALDAGHDLDRILTTEDLVSGENVFFSATGVTDGDLLRGVRYYSGGASTQSIVMRSKSGTVRIIDAYHRLTKLHEYASVDFVGDETAIPPLP

>CORE_REP|Org15_Gene485#

MTSYAPAEALAIEADELVKVFGEQRAVDGVSLAVPQGAVYGVLGPNGAGKTTTIRMLATLLRPDGGRARIFGHDVVAEPTAVRSLIGVTGQYASVDEKLSATENLIIFSRLLGLSRSEAKRRAAELLEEFGLTEAATKALENFSGGMRRRLDLAASLIATPPLLFLDEPTTGLDPRTRAQMWETIRRLVREGATVLLTTQYLDEADQLADRIAVIDHGRVIADGTSDELKGSVGQSALQITVADRDVIERARTLIGEFLSRADGKLVEASISPEAGRVTAPLSDPSVTADLLIRLRDNDIRVDEITVSKPSLDEVFFALTGHAAESDAAESDSAESDSAGSNSEGTAA

>CORE_REP|Org101_Gene2563#

MIIMGIESSCDETGVGIVRRHADGSCELLADEVASSVDQHARFGGVVPEIASRAHLEAIVPAMRRALAVAGIAKPDALAVTIGPGLAGALLVGVAAAKAYAAAWDVPFYALNHLGGHVAVDTLEHGPMPPCVALLVSGGHTHLLHVTDLAEPIVELGSTVDDAAGEAFDKVARLLGLGFPGGPALDAAAAQGDPGAIAFPRGMTGPRDARYDFSFSGLKTAVARYVEAAQRSGLTAADLPIPNIAASFQEAVADVLTMKAVRAAQDVGVDTLVLGGGATANSRIRSMAEERCAAAGLTLRVPKPRLCTDNGVMIAALGAHVIAGGAPPSALTVATDPGLPVSVSRVS

>CORE_REP|Org116_Gene5513#

MATAPGAVARSVTWVTTRSYAVRVSARRPALGRSHPFAVLVGPIRLAVIQRRRRDRSPVTGESDPSGTTTGRLVLAATPMGDIGDASQRLRDALTTADVVAAEDTRRTRALAKALGVEITGRVVSFYDHVENARIPALLDDIAAGRTVLLVTDAGMPSVSDPGYRMVAACVDRDLPVTCLPGPSAVTTALALSALPVERFCFDGFPPRKSGARRAWLRTLRTEPRACVFFEAPHRLADCLADAVEVLGPDRRAAVCRELTKTYEEVVRGTLADLATWAVDGARGEITVVLAGATPTATDPTTLVPEVEALVEDGLRLKDACAQVSSATGVSRRELYDAVLSARTEG

>CORE_REP|Org114_Gene2607#

MPIATPEIYAEMIARAKENSFAFPAINCTSSETVNAAIKGFADAGSDGIIQFSTGGAEFGSGLGVKDMVTGAVALAEFATVIAAKYDVTIALHTDHCPKDKLDTFVRPLLAISADRVKSGQNPLFQSHMWDGSAIPIDENLEIAKELLKQAHAANIILEVEIGVVGGEEDGVENAINDKLYTSPEDFEKTIDALGAGENGKYLLAATFGNVHGVYKPGNVKLKPEVLAEGQRVAAAKLGLGADAQPFDFVFHGGSGSLKSEIEDSLRYGVVKMNVDTDTQYAFTRPVAAHMFSNYDGVLKVDGEVGNKKVYDPRSYLKKAEANMAARVVEACNDLKSAGRSISAK

>CORE_REP|Org30_Gene3136#

MSSPASSPVAAGERKQRVLSGIQPTSSSFHLGNYLGALQYWVTMQDDYDALYFIPNMHAITVPQEPKELRLRTRRSVAQLLAIGIDPERSTLFVQSQVPEHAELTWVLSCLTGFGEASRMTQFKDKSVKQGAENATVGLFTYPVLMAADILLYRPHQVPVGEDQRQHLELTRNLAQRFNTRFKKTFVVPEPHIVKGTAKIYDLQDPTAKMSKSANTDAGLINLLDDPKVTAKKIRSAVTDTEREIRYDPDAKPGVSNLLVILGSLTDTPIVTLEKEYEGKGYGDLKSDVADALVEFVTPLQAKVEEYMADQGELDRILAAGAERAREIAGNTLAQVYDRVGFLTR

>CORE_REP|Org16_Gene3362#

MGVRVGVVGATGQVGAVMRKLLEERDFPADEVRFFASARSAGKKLPWRGGEIVVEDTETADPSGLDIALFSAGATMSRVQAPRFAAAGVTVIDNSSAWRKDPEVPLVVSEVNPEQTRNLVKGIIANPNCTTMAAMPVLKPLHDEAGLQRLIVSSYQAVSGSGLAGVEELATQARAVIGDAEKLTHDGSALQFPAPNKYVAPIAFNVLPLAGSLVDDGSGETDEDQKLRNESRKILGLPDLLVSGTCVRVPVFTGHSLSINAEFARPLSVERAKQLLADAAGVKLVDVPTPLEAAGKDESLVGRIRQDPGVPEGRGLALFVSGDNLRKGAALNTIQIAEVLLAQR

>CORE_REP|Org113_Gene7273#

MNILRRLTFLLRPSWAILAVVVVAFAYLCFTVLAPWQLGKNTSTSHRNDLIAASVKADPVPAAGLLESADGAAPAGTNPADTEWRRVIVTGSYVPGSTVVERLQHLDDQPAYGVLAAFRLDDGRIVLIDRGLVAAADGTRLPAIAEPPAGPQRLEGRVRRSEGTIPGKDPMVGDGLRQVYSVDTTQMSSVLGMRLTPFATGEQGGYLQLDSGQPGAFTPEPLPQLDAGPYLSYGLQWIAFGVMAPLGLGYFVYAEIRERRRDRAAASAEPTDPTTSAPVPSTAPDTGTAHTAATEPTAAAPTASATMTETPPAEMTNSPSATDETKKPHRTTADRLADRYGNRR

>CORE_REP|Org34_Gene2552#

MQLGMIGLGRMGANIVRRIVADGHTAVGYERHAPHIEELGAELGASFSGTTDLAEFVSRLETPRVVWVMIPAGATGAVIDQVAELLEPGDIIIDGGNIRYHEDIQRAERLAPKGIHYVDIGTSGGVFGRTRGFCLMIGGEAGPVRYLDPLLRSIAPGVDAAPRTPGRTGEPSPAEQGYLHCGPAGAGHFVKMVHNGIEYGAMAAYAEGLNILHKADYGAGYDSGAHSAEETPLEHPEYYRYDIDIPEVTEVWRRGSVVASWLLDLTAAALHADPNLDSFGGRVSDSGEGRWTIDAAIDIGVPVPVLSAALFQRFSSRGESHYADKMLSAMRKAFGGHNELPQG

>CORE_REP|Org7_Gene7659#

MNIEIEALRAIVADKGISIETVISAIESALLTAYRHTEGHQPNARIDINQKTGTVRVMARELDADGNVISEWDDTPEGFGRIAATTARQVVLQRLRDAENEKSFGEFSTHEGDIVGGVVQRDARANARGTIVVRIGSELHGAEGLIPPAEQVPGETYEHGDRIKCYVVGVSRGPRGPQITLSRTHPNLVRRLFALEVPEIADGSVEIVAVAREAGHRSKIAVRSTVSGVNAKGACIGPMGQRVRNVMSELAGEKIDIIDFAEDPATFVGNALSPSKVVSVTIVDPEARAARVVVPDFQLSLAIGKEGQNARLAARLTGWRIDIRSDAAPDMGGGTVRTEAHRS

>CORE_REP|Org127_Gene3382#

MSNSGAKKLTPEQLRKLYRPGELTVVHEPAGVSAVTSALRGVGRTVALVPTMGALHEGHLELVRRAKRTNQVVVVSIFVNPLQFGENEDFDKYPRTLDSDVALLREEGVALVFAPSVAQMYPDGPRTSVHPGPLGAELEGASRPTHFAGMLTVVAKLLQIVRPHEAFFGEKDYQQLTLIRQMVRDLNFDVDIVAVPTVRESDGLALSSRNRYLDEQQRELAITLSAALAAGRHAAGRGPDAVLAAARSVLDGATGVDVDYLELRGSDLGPIPSSGNARLLVAARIGATRLIDNVPVSVPPAVSDAASAGPSVVTQAAASGPVAPADGHNAVPDFQPAQADA

>CORE_REP|Org4_Gene4426#

MPEAMHSVRGETVRIGQQPGGNGAHGSAPTVLTERFTGPGLPSRVLRRVLAYIALTKPRVIELLLVATIPTMLLADRGTIDIRLILVTLFGGWMGAASANTLNCVADADIDKVMKRTAKRPLAREAVPTSHAFVFGVVLGLASFAWLWWQANLLSGALVVATILFYVFVYTLGLKRRTSQNVVWGGAAGCMPALVGWSAATGGIGWPAIALFGVIFFWTPPHTWALAMRYKEDYRAAGVPMLPVVATEQAVTKQIVIYTWLTVLTTLALVPATGVVYAAVALVAGAWFLLMAHQLYAGVRRGESVKPLRLFLQSNNYLAVVFCGLAVDSVLGWDTVGSFFG

>CORE_REP|Org195_Gene950#

MRSIWKGSIAFGLVNVPVKVYTATEDHDIRFHQVHAKDGGRIKYDRVCTVCGQSVQYTDIDKAYESPDGDKVVLNDEDFAKLPVAEKHEIPVLQFVPSDQIDPVLFEKSYYLEPDSSTPKAYVLLARTLEEIERTALVYFTLRQKTRLAALRVREGILVLQTLLWPDEVRSVEFESLDGVAEPRSQEIKMAETLVEAMSDDFDPDQFTDEYQIELKRLLDEAIASGTGKVPEQPEPVPSGMDAEVVDLVAALQRSLEASGRRTASGDGAAAEPKKTAKKAPAKSTASKSGAAKTTASKSGASKSASKSAAKSATSKSAAKSAAKKAPAKAAKKTAARKGA

>CORE_REP|Org46_Gene4582#

MSERSERTSDTGTRVRDLIIVGSGPAGYTAAVYAGRAELQPLQFEGTQFGGALMTTTEVENFPGFREGIMGPDLMEEMREQAKRFGAEIRTEDVDAIDLTGPVKKVVVGGETFEAYAVILAMGSAARYLNVPGEQRLLGRGVSACATCDGFFFKGQDIVVVGGGDSAMEEATFLTKFASSVTIVHRREEFRASRIMLERAKANEKIKFVLNAEVAEVHGDSSVTHLTLRDTRTGETSDLPATGLFVAIGHDPRSELVKGQVALDDEGYVLVQHPTTATDIPGVFAAGDLVDHTYRQAITAAGTGCRAAIDAERWLADQGDITSNTLDHAGESVAVPAN

>CORE_REP|Org119_Gene6972#

MTAQERALVVDHEGAQRANHDEAERERGAVMSEEPTGADLAEAVVKGPPKTLAGGLFKAIRPRQWVKNVLVLAAPLAAGTANEIDVLAHVGIAFVVFCMAASGIYLVNDALDVEADRAHPTKRFRPIAAGVVPVNLAYALSLVLLVGSIAGSFLASWHLAVVMAVYIGIQLAYCFGLKHQAVLDICIVSSGFLLRAVAGGAAANIDLSQWFLLVMAFGSLFMAAGKRYAELQIALATGAKIRRSLEYYTPTYLRFVWTLAATAVVVFYGLWAFQQDSLKDTNWYAISMIPFTIAILRYAVDVDGGQAGEPEEIALGDRVLQLLAIALIGAVGVAVYLT

>CORE_REP|Org19_Gene2534#

MAVEMFYDDDADLSIIQGRKVAVIGYGSQGHAHSLSLRDSGVEVRIGLKEGSKSRAKAEEAGLTVGTPAEVSEWADVIMLLAPDTAQAKIFTEDIEPNLKDGDALFFGHGLNIHFGLIKAPAGVTIGMVAPKGPGHLVRRQFVDGKGVPALIAIDQDPKGEGQALALSYAKGIGGTRAGVIKTTFKEETETDLFGEQAVLCGGTEELVKTGFEVMVEAGYAPEMAYFEVLHELKLIVDLMYEGGIARMNYSVSDTAEFGGYLSGPRVIDADTKERMKAILKDIQDGTFVKRLVANVEGGNKELESLRKQNAEHPIEVTGKKLRELMSWVDRPITETA

>CORE_REP|Org201_Gene1319#

MGDAIVAEGLVKRYGQQVALDGLDLTVPEGTVTALLGPNGAGKTTTVRVLTTLLIPDGGRATVAGIDVLRDPRALRRRIGASGQYAAVDEYLTGFENLEMVGRLYHMGVQRSKERARELLDRFRLSDAADRPVKGYSGGMRRRLDLAGALVAAPPVLFLDEPTTGLDPRARLDLWDVIEELVAGGTTLLLTTQYMEEADRLADSIAVIDRGKVIAKGTADELKTMVGGDRIELTVDHVDNLAIAQQALAGLADGEIHLEPGLRRIIVPVSNGSQALVEAVGRLNDHSVKIHDVGLRRPSLDDVFLTLTGHEAEELINADDAADGLGALEATEGKTR

>CORE_REP|Org190_Gene3432#

MGATVVGSRSNVEVVCESVPVTGDQQAPVCVLGTGLIGGSLLRAAVGAGFRAWGYNRSAAGAEAARADGFDVTGDLPAVLRRAAETDALIVVAVPMPAVDQILSAVSTFAPDCAVTDVVSVKAPVAAAARRHGLGARFVGGHPMAGTSQSGWAATDPALFRGAAWAVGVDPGTHPQPWTRVVRLALACGSVVVPVVAEEHDRAVARISHLPHVLAEALAVAGAGGGELALGLAAGSFRDGTRVAATAPDLVRAICEPNAAALLEVLDETLTVLNAARDLLEEDGSLADLTEAGHDARQRYETTERWEITDIRPGDQDWLERLREAGQRGGVITRLD

>CORE_REP|Org43_Gene3438#

MTRAAVLGAGSWGTAFAKVLADAGTEVTIWARRPEIAEALATEHRNPAYLPDVQLPAVSATHDAAAALDGAQLVVLAVPSQSLRANLTGWRPALRAAIDEHDATLLSLAKGIETGTLLRMSQVIAEVTGAEERRIAVLSGPNLAREIAAGQPAATVIACSDAARAEAVQQASYTGYFRPYTNTDVIGCEIGGACKNVIALACGIAAGMGLGDNSIASLITRGLAEIMRLAVTLGAEPVTLAGLAGVGDLVATCTSPLSRNRSFGHVLGAGGSMEAAQQATHGQVAEGVKSCTSVRALAAAHEVEMPLTDAVHRVCHEGISVREAVGSLLGRRIKPE

>CORE_REP|Org5_Gene2060#

MDFCGVRTKAVRKLHRRRHLLRALLRCFVRDVIALFAPGQGSQTPGMLAPWLDLPGARDRIELWSKAAGLDLLQLGTTATAEEITDTAVTQPLVVAAALLAFAEIPHGSVPADTIVAGHSVGEFAAAAVAGVISPDDAVKLAAIRGAEMAKACALVPTGMSAVLGGDEAAVLDRLAELDLTPANRNAAGQIVAAGRLDALAELAANPPEKARVRALPVAGAFHTSFMAPAQDAVAEAISQMVVPDEPIRTLLSNFDGKPVTSGKDAMEKLAAQVTRPVRWDLCTETVRVAGVSAVAELPPAGTLVGIAKRELKGTPNLALKTPENIPAFVELSATG

>CORE_REP|Org134_Gene5162#

MGRVRAIRLNGFGGPEVMEWAETPDPQAGPGEVLIDVAAAGVNRADVMQRKGHYPPPPGASEVPGLECSGVIAAVGDGVRGWSVGDRVCALLSGGGYAERAVAPAGQLLPIPDGLDLGAAAGLPEVAATVWSNLVMTAGLHAGQLVLIHGGGSGIGTHAIQVAKRLGARVAVTAGSAGKLERCRELGADILINYREEDFVAVIRAEQGSGGPGADIILDNMGAAYLARNVEALATYGQLVVIGLQGGVDAELNLAALLGKRAAVRATNLRGRPANGVGSKAEIIAEVREHVWPLVTEGAVVPVIHAELPINEVGDAHALLDSADTVGKVVLHIGDY

>CORE_REP|Org100_Gene3243#

MKLAVIPGDGIGPEVIAEALKVLDVVVPGVEKTEYDLGAKRYHATGEILPDSVLPELREHDAILLGAIGDPSVPSGVLERGLLLRTRFELDHHVNLRPSRLFTGVRSPLAGAPDIDFVVVREGTEGPYTGTGGAIRVRTPHEVATEVSTNTRFGIERVVRYAFAKAQARRKHLTLVHKTNVLTFAGSLWQRTVDEVGAEFPEVTVAYQHIDAATIHMVTDPGRFDVIVTDNLFGDIITDLAAAVSGGIGLAASGNIDASGTNPSMFEPVHGSAPDIAGQSKADPTAAILSVSLLLNHLGDTEAAARIDAAVAKDLAARSGTASTVEIGDRIAAAV

>CORE_REP|Org140_Gene4760#

MKPRQGALVLSTAFTETFGIRHPIVQGGMQWVGRAELVAAVANAGALGMITALTQPTPEDLAKEIVRTRELTDQPFGVNLTILPAITPPPYDEYRQVIIDSGVKIVETAGSNPAPHLPDFHAAGIKVLHKCTSVRHAVKAQDAGVDAISIDGFECAGHPGEDDVPGLVLIAAAAEHLTIPMIASGGFADGRGLVAALALGADGINMGTRFMCTQEAPIHRAVKEAIVAGRETDTELIFRPLRNTARVARNAVSLEVVDILNKGGKFEDVRDLVAGTRGRKVLETGDLDGGIWTAGTVQGLIHDIPTVGELVDRIVADAETVITERLSGRLARVDV

>CORE_REP|Org144_Gene4159#

MTVRPDDPAPNPHATEAEVEAARKDTKLAQVLYHDWEAETYDDKWSISYDERCIEYARGRFDAAVGPAPLPYERALELGCGTGFFLLNLMQGGVAKTGSVTDLSPGMVKVALRNAQNLGLDVDGRVADAETIPYEDNTFDLVCGHAVLHHIPDVELALKECLRVLKPGGRFVFAGEPTTAGNFYARWLGRITWKATTTVTKLPQLAGWRRPQTELDESSRAAALEAVVDLHTFDPSDLEAMASSAGAVEVKASTEEFAAALWGWPVRTFEAAVPDEKLTMGYRMAMYKAWLRLSWLDENVMRRVVPRQFFYNAMITGVKPWGMEAGAAGANSSTE

>CORE_REP|Org5_Gene3382#

MASLSVRPGSVGAMPTPSASDRPLLLLDGASLWFRAFYAIPEKITAPDGRPVNALRGFTDMVAALITRHRPGRLVVCLDLDWRPDFRVALVPSYKAHRLDTAAGAAAGAEEVPDTLTPQVGMIADVLAAAGIATAGAAGLEADDVIGTLATRERDDEVVVVSGDRDLLQLVRDEAPLVRVFYVGRGLAKAELLGPAEVAAKYGVPQENAGPAYADMATLRGDSSDGLPGVAGIGDKSAATLISRFGSLEALVAAVDDPDSNLARGVRAKLVAAQEYLKAAAPVVRVVRDAEVELSGPDTLPTAPADPDRLDALATAYNAESPFKRLTAALAANV

>CORE_REP|Org49_Gene2429#

MNAGLGPAARYLLALCARGAHPRANRKTGEDTALRTTTYRRTGLLALAATAAAGLVLAAPAQAAPLWPGGPDIPGVPSAVMPQPEPGDTPNSPNAKAPVPPANFAAPNISPGDGEVVGVAQPIIINFKEPVTDHETAEKAIRITSTNKVSGHFYWFGDKQVRWRPESFWPAGSQITVEAGGTHVAYEIGDEFIATADDSTHEITVTRNGEVVRVMPTSMGKPGHETPNGTYITSERNRKMIMDSSTYGVPVTDPEGYKLEVEYAVRMSNSGIFVHSAPWSVAQQGVSNASHGCLNVSPADAQWFFENVKKGDPVVVVNTNGGTLNPGDGYGDWN

>CORE_REP|Org1_Gene1864#

MRVPIPPSRRHGAPDLPPATEDNPAAAAQGTPIYSLSTSVRQRLRGFLGSKRRLPRKDSSQMTVLDSILDGVRADVAAREALLDFQSIKAAAAKAPAPLDARAALLEDGIGVIAEVKRASPSKGALADIPDPASLAKAYEDGGARVISVLTEGRRFGGSLDDLDAVRATVNIPILRKDFVVGPYQIHEARAHGADVILLIVAALEQDVLASLIDRTESLGMTALVEVHTEEEADRALEAGASVIGVNARNLKTLEVDRDVFARIAPGLPTEVIRVAESGIRGTADLLAYAGAGADAVLVGEGLVTSGDPRAAVSELVTAGTHPSCPKPARRGR

>CORE_REP|Org49_Gene4483#

MPIDETLAGYSNLAFKSAFVVYLLVLAMLIVQYASARKKLTAERELVTVGGSGSGSGSGSGDVLAANVPGKLAEKPAPTLAERFGNMAFAVLFVAIGLHLASIVLRGFAVHRFPLGNMYEFITMACAAAMVTGLVFMSDRRFRAMWVFLIVPVLILMYLAGNVLYAEAAPVVPALKSFWLPIHVTIVSIGSGIFLLSGVASLLFLFRMRQPDGQESDNLLGTLARRLPDARTLDRLAYKTTIVAFPLFGTGVILGAIWAEAAWGRFWGWDPKETVSFITWVVYAAYLHARATSGWRDTKAAWINIAGFTAMLFNLFIINIVVSGLHSYAGLN

>CORE_REP|Org170_Gene2897#

MSVSARRGSRSRRSRPVGCLVLLALAVLVVIVVVLAWYLLAGRLKEPEPGPKPPEERPTSQPASCPDVQMIAVPGTWESASNDDPYNPTANPASLMLNVTGPLREQFPAERVDIYTVPYVAQFSNPIAFPPDGQQSYNNSRSEGTRRMVDMLTDRHAECPLTTYVFAGFSQGAVIAGDIAAQVGAGNGPIPQDLLLGVTLIADGRRTGESGPPNAIPIGPVPPGVGAEVALAGLNVPGITMTGPRPGGFGAVADRTYTICAPTDLICDAPRDALRPTNIVGSLTTLIGAIGNPVHALYNGFVVDPNGTTATRWTANWASGLIEAAPRPPHS

>CORE_REP|Org2_Gene5546#

MTVLYHRDVLYRAFLRLVDKLPLPSLRVQRILAIAVILTQAGISVTGAVVRVTASGLGCPTWPQCFPGSFTPVGVSEVPVLHQAVEFGNRLLTFVVSLCAALIVLAVVRARRRRDVLVYAWLMPGGTLLQGIIGGITVRTGLLWWTVAIHLLASMLMVWLSVVLYAKICEPDDGIATVQAPAPLRWLTGLSAVAMSGVLIAGTLVTGAGPHAGDKSIERQVERLQVEIVTLVHLHSQLLVGYLALLIGLAFGLFAVGITPAVRKRLFVVLAIVCAQALIGVVQYFTDVPAVLVVFHVGGAAACVAATAALWAALHTREPVPAAVTVEQSV

>CORE_REP|Org130_Gene3075#

MNEKLSDILSKMLDLHTTADLLSYDFVQQAVLAAALLGLLAGVIGPLIVNRQMSFAVHGTSELSLTGAAAALLVGIGVGAGAIAGSVIAAVMFGLLGSKARERDSVIAVVMSFGLGLSVLFLWLGPSRAGSKFSLLTGQVVSVGGTGLTSLALCTVGVLAVLAFIYRPLLFASTDPEVAVARGVPVRALSVVFAVLLGVTAAFGVQIVGALLVLSLLITPAAAAAQLTASPLRATLLSVLFAEIAAVGGILLSLAPGVPVSTFITTISFVIYLACRFIGRPVVNARRRTALRTPAPAATAGPDPAPHIDASNDPDSRRNRAEEQLPVRG

>CORE_REP|Org14_Gene5873#

MRIPFDPRRSRRRTNPDGTMSLVEHLQELRSRLLKSLLAVALTTILGFLWYSHSFLGIESLGDLLRGPYCSLPPEHRAQLTTDGTCRLLATAPFEQFMLRFKVAFTAGVVMACPIWLYQLWAFVTPGLYAKERKYAISFVASGVVLFVTGAVLAYWVVAHALSFLMGIGSNVQITALSGSQYFGFIIKLLIIFGVSFETPLLIIGLNMVGVLTYERLKKWRRGMIFGLFVFAAIVTPQDPFSMLALAAALTVLFEVAVQIARLNDRRRARRGDNWGALSDDEASPLAGPDDLDGVSPVEPARPVTATGPVSAEPAPKTPRPVSDYSDTL

>CORE_REP|Org1_Gene5346#

MLDSMIEVRGLTKHYGRTAAVEDLTFTVKPGQVTGFLGPNGAGKSTTMRMILGLDTPTAGTALIDGKPYHQLKQPLRTVGALLDAKWVHPNRSARAHLEWLAASNGIARSRVEEVLRLVGLSEVAGKNAGGYSLGMSQRLGLAGALLGDPKVLLFDEPVNGLDPEGILWIRRFMQRLASEGRTVLVSSHLLSEMAQTAEHLIVIGRGKLIADTPTKEFIERASEQTVRVRSPQLDQLRSLLTSNGMTVREDGTGAEGPALLVAGVTSDAVGKLAGANDITLFELSPQRASLEEAFMRMTGGAVQYHGEGAEAVGVPGPGGPYTAMGGAL

>CORE_REP|Org4_Gene4494#

MTAAPEPPYRVATSPHAPPSFQAGELTDPRLTAALKTLELTVRRRLDGVLHGDHLGLIPGPGSEPGEARTYQPGDDVRQMDWSVTARTTHPHVRQMIADRELETWMVVDLSASLDFGTALCQKRDLAIAAAAAITHLTSGGGNRIGAVVATGERLVRVPARSGRVHAQSLLRSIATTPHARDGVRGDLRGGIESLRRPQRKRGLAVIISDFLGEIDWQRSLRAISARHDLLAVEIIDPRDLALPDIGDVVLHDPETGRTREFSVTPTLRADFAAAAQRHREQVEQALRSCGAPVLTLHTDRDWIADVVRFVSTRRHSLGAPSGRVPRQ

>CORE_REP|Org25_Gene4331#

MTLSDSSPANGSAPTAATVRHFLRDDDLTPSEQAEVLALAAELKGAPFARRPLEGPRGVGVIFEKNSTRTRFSFELGIAQLGGHAVVVDGRDTQLGREETLGDTGRVLSRYVDAIVWRTFEQTRLDEMAATATVPVVNALSDEFHPCQVLADLLTLTEQLGPLSGRKLAYFGDGANNMAHSLLLGGVTAGLHVTIAAPEGFAPLPWVVEAARARAAETGASVTLTDDPRIAAEGAHALVTDTWTSMGQENDGLDRVGPFRRFQINAGLLAKAQQDAVVLHCLPAHRGEEITDEVLDGPRSVVWDEAENRLHAQKALLVWLLDRQNGRR

>CORE_REP|Org176_Gene596#

MRSARGILVAVLVATALASGCTSDSSAPAPTKSPVYTDPPLPAKAVPVFTDSPVPLPPPGTPRCGDPTASLRPSGAGAAARGPTIDAIRARGRLLVGLDTGSNLFSYRDPVSGAIVGFDADIAREVARDLLGSPDLIEFRSLGSAEREAALQNRTVDLVAKTMTINCERREKVAFSTVYLHANQRVLAVKNSGIRSLADLAGRRVCIVSGTTSLEHIRRDQPAATILTVPSWADCLVVLQQRQVDAVSTDDAVLAGLAAQDPYTELVGGSISEEPYGIGIPKGNDDLVRFVNGTLERIRNDGTWVGLYQRYLPSLGPVPAPPAPTYQD

>CORE_REP|Org102_Gene1947#

MSERSDRPEDTTERPRDTADHAGPADTAGSSAAVAPSAGTGEFAAADGAEVASSSGVGEHVVAAASSQAADITAERIAAAARVEQAAAQGQRSSESTVRIGLVLPDVMGTYGDGGNAVVLRQRLRMRGYDAEIVEISLSEPVPDSLDIYTLGGAEDSAQRLATRHLQRYPGLQTAAGRGVPVLAICAAIQVLGHWYETSSGERVDGVGLIDVTTSPQAERAIGEVVTNPILAGLSQPLTGFENHRGGTKLGGAATGLARVTRGVGNGVGDGLEGVVQGSVIGTYMHGPALARNPELADYLLAKALGVDSLPPLDLPEVEQLRRERLRA

>CORE_REP|Org81_Gene4684#

MAMTAEVKDELSRLTVSQVSSRKAELSALLRFAGGLHIVGGRVIVEAEVDMGSIARRLRREIFELYGYGSDVHVLGAGGLRKTSRYVVRVSKEGEALARQTGLLDVRGRPVRGLPAQVVGGSISDAEAAWRGAFLAHGSLTEPGRSSALEVSCPGPEAALALVGAARRMGISAKAREVRGTDRVVVRDGEAIGALLTRMGAQDTRLTWEERRMRREVRATANRLANFDDANLRRSARAAVAAAARVERALEILGDDVPDHLAAAGKLRVLHRQASLEELGQLADPPMTKDAVAGRIRRLLSMADRRAKELGVPDTESAVTAELLEDA

>CORE_REP|Org8_Gene4316#

MAGNSQRRGAIRKGGTKKGAVVGSGGKRRRGLEGRGATPPAEARTKHPAAKRAAAAAKAAAAGRGGPRGGGSGRPAGRKNDDGPEMVLGRNPVVECLRAGVPAAALYVAVGTENDERLTESVKLAADAGISILEVPRTDLDRLSANGMHQGLALQVPPYRYSHPDDLLDQVRNSAEPALLVALDNISDPRNLGAVIRSVAAFGGQGVLIPQRRSASVTAVAWRTSAGAAARLPVARATNLTRTLKDWAAQGIQVVGLDAGGDTTLDDFDGREPTVVVVGSEGKGLSRLVRENCDAILGIPMAGPVESLNASVAAGVVLAEIARQRRL

>CORE_REP|Org215_Gene5280#

MDNSPGTPSRRAALGRRSATEESRHPLIGRSRGEAGEPRVGLRAVIREAVETLADAGVHSPHTDAELLAAHVLGVDRMRLMMVPLLTPEQLADFRALVARRAERVPLQHLTGTAAMGEIDLAVGPGVFIPRPETELLFAWALAQLEAVGHEHRPVVVDLCTGSGALALAIAHARPDAQVHAVELDPAALDWARRNALHRADQGDTPIDLHAGDVTAPDLLSHLNGTVDVVVANPPYIPESARLDPEVADHDPRRALFGGPDGLSVIRPMIGTIARLLRVDGVTAVEHDDTNGSDTAALFHSHGGFDAIVEHPDLAGKPRFVAARRTG

>CORE_REP|Org121_Gene3069#

MTASWIDNQKNLMLFSGRAHPELAEQVAKELDVHVTPQTARDFANGEIFVRFEESVRGSDAFVLQSFPAPLNQWLMEQLIMIDALKRGSAKRITAVLPFYPYARQDKKHRGREPISARLVADLLKTAGADRIITVDLHTDQIQGFFDGPVDHMHAQLQLAEYVRTNYSLDNITVVSPDSGRVRVAEKWADSLGGSPLAFIHKTRDPLVPNQVKSNRVVGEVEGRTCILIDDMIDTGGTIAGAVKVLKDAGAGDVVIAATHGVLSNPAAERLAACGAKEVVVTNTLPITEEKKFPQLTVLSIAPLLARTIREVFENGSVTGLFNGNA

>CORE_REP|Org82_Gene6136#

MPNRYRPTSSDRYRTGGTGRHPGPCSTTEDSALSQHRVGPSSITVQSLLGEGGEAGRPKRHRAEPSATERVKAAATAAVAAGALIGAASQAAPALAYASPLLPGSHDSDEDEQAAPTVVKGSSILPVAEAKAAAEPVAEVAPEPAAVAGQIAAPVAAPFGIPNLPPEIAGPLAQAEEVLKGVQQQVAPAPQASAVRPVAGAVSSGFGSRWGAMHYGIDFADALGAPIHSVSNGTVIEAGPASGFGLWVRVLQDDGTTAVYGHVNEMFVHAGQRVNAGDVIATVGNRGQSTGPHLHLEIWDQAGTKIDPMPYLAAKGVPLGWGPSAH

>CORE_REP|Org174_Gene27#

MIGFLLRRAANYVVLLLLASFLTFAVAGLTFRPLDSLEQRNPRPPQAVIDAKAEQLHLDEPIPQRYLTWVSGAVRGDFGTTLAGQPVSEELGRRIGVSLRLLVIGSVLGTVLGVLIGAAGAIRQYRFSDYFTTIVSLVLLSTPIFLLATLLKYGALEINSLTGQRIFLYTGETSAHRIEGLWPQLLDRLQHLVLPTLALALGGMAGYSRYQRNAMLDVLQSDFIRTARAKGLTRGRALYKHGLRTALIPMATLFAYSLGGLITGATFTEKIFGWHGVGEWLVDAVNAQDIYVVVTVTVFTGLVVLVSGLLSDIVYAILDPRVRVG

>CORE_REP|Org138_Gene6180#

MTDRIVAPTGEDPARKVKKLYRSDVRRARRLLRGQFRVTPVFHTEVQGPHGPVPVTLKLEYLQHGGTFKVRGSLNALLGARAGADSVVLASGGNAGIAAALASAVRGLSCTVVVPESAPHTKVAAMWSHGAEVLWHGTTYAEAYRFATELAVERGALQLHAYDQPAIVAGAGVVGLEIEDQVRGRPPVLVAVGGGGLVSGIAVALGPRGRVIGVEPHGAPTLHAALAAGRPVEVEVSSVASDSLGASRIGAIAMEVAQRYGVESLLVSDDAIVMAREYLWREFRIVVEPAGATALAAIQSGVYVPKPYERPVIVLCGANTDLATL

>CORE_REP|Org108_Gene1873#

MEQRTVGRSGLRVSRIGLATHTWGTRTDADQAAVQLMAFVEAGGTLVDTSPVYAGGAAQRILADLLGDLVSRDDLVLSGCAGLHPRPVPPAADGTPPMPQVPGIGVDTSRRTLLRQLDRTLLELGTDHLDIWHIAAWDPRTPLEEVAATVELALRSGRVRYAGVRGFTAWQLASLAAMAPITVTQTPYSLLARTAEDDTVPAAAHHGVGMIATAPLAGGILTGKYRDGVPADSRGADEATAAEIRGRLDERATRVVDALVTAADGLATSPLAVALAWIRDRPGVASMFVGARDIGQLTGVLAAETLELPRAIAAALDDVSARTD

>CORE_REP|Org82_Gene6175#

MRAAQVSKLEGPEAVQIVDIPEPAAFPGGVVIDVHAAGVAFPDVLMTRGLYQMKPELPFVVGGEVAGIVREAPEDAHVRPGDRVVALTMLGNAMAETAVTPTQMVFRLPDNVSLEAGAGILFNDLTVHFCLRTRGRLAEGETVLVHGGAGGIGTSTLRMAAALGAGRVIAVVSTEAKAEVARANGATDVVLTDGWLAAVKELTGGRGVDIVLDPVGGDRFTDSIRSLASAGRLLVVGFTAGEIPTVKVNRLLLKNVEVTGAAWGEWVMTHPGYLQEQWAEVEPLLASGKIAPPEPVLYPLDKAAEAVASLDNRTATGKVVVTLR

>CORE_REP|Org18_Gene4392#

MSIAVSASIATDHLMRFPGRFADVLLADQLDHVSLSFLVDDLVIRRGGVGGNIAYAMGLLGRNPLLLGAVGADFSEYRQWLEAHGVDCSAVRISDSAHTARFVCTTDEDMAQIASFYPGAMSEARDISIAGLVEENRTLDLVLVGANDPEAMLRHTAECRELDIPFAADPSQQLARLDGDQTVQLIDGAAYLFTNEYEWGLLKQKSGLTEEEVASRVGIRVTTLGKNGVVVVDRDGSEVRVGVVPENAKVDPTGVGDAFRAGFLTGHTAGLSLERAAQLGSLVAVLVLETVGTQEWSLDPDDALKRLTQAYGPEAAAELEPLLR

>CORE_REP|Org112_Gene5835#

MTGRAGTVTHPTGTVASSIAVVTAASALTVTGAAAGRRAVGGAETPGDRGRPALIAVAHGSRDPRSAATMHAVVSDVAAARPDLDVRLAFLDLSTPSVEQVVDAVAADGHTHAVVVPLLLGKAFHARVDLPGLLAAAGARQRRLRLTQADVLGPDPRLIEALRDRVLESLTAGSSGEASHAARIDTRSLAVPRVDVDACAASPLTSGGHLGVAVAAVGSSSAAANARTAAVARQLAARTGWDTEICFATTEPTVTTALSRLRDRGAGQLLVAPWFLAPGLLTDRLANAASDIAHTAVIGAHPLLTQVVLDRFDTAAALPHALTA

>CORE_REP|Org158_Gene2483#

MTDRIAAVDCGTNSIRLLIADVAPAGEASATPHLTDVHREMRIVRLGQGVDATGSLHPEAIERTRAALHDYVDLMLDAGVSRVRMVATSATRDASNREDFFAMTREELGRVVPGAQAEVITGDEEARLSFAGAVGELSSADGPFVVVDLGGGSTEVVLGDSSGVQAAYSADIGCVRITERCLRGDPPTPEEVASGRFFASERLAQAFGVVPVERARTWVGVAGTMTTLAAVALDLPEYDSEKVHLTRLTLPQVRAVCDRLIGMTHDERAALGPMHPGRVDVIGGGAVITEVLADELARRAGIDALIVSEHDILDGIALSVAPRR

>CORE_REP|Org151_Gene5995#

MTDTVARYESLIATLGNTPLVGLRTLSPQWDGENHVRLWAKLEDRNPTGSIKDRPALRMIEQAERDGLLRPGCTILEPTSGNTGISLAMAAKLKGYRLVCVMPENTSVERRQLLTMFGAQIIDSPAAGGSNQAVARAKQLAAEHPDWVMLYQYGNPANALAHYETTGPEILADLPEITHFVAGLGTTGTLMGTGRFLREKVPSIEIVAAEPRYGELVYGLRNIDEGFIPELYDESVLTTRFSVGPFDAVKRTRELVSEEGIFAGISTGAILHAALGVARKAAKAGTRADIAFVVADGGWKYLSTGAYDGTLEEAEERLDGQLWA

>CORE_REP|Org60_Gene919#

MTAPTLEVHLTDDDLTAALRADARRGLTADPKWLPPKWFYDARGSELFEQITELPEYYPTRTERALLERVVGEIARAAQAQVLVELGAGSAAKTRLLLSALTAEGPLKTYVPQDVSATALRATAAEVAREFPGLAVHGVVSDFTDTLHNLPRGGRRMIAFLGGTIGNLVPAERAEFLTDVHDVLEPGEHLLLGAGLVIDPAILVPAYDDAAGVTAEFNRNVLHVLNARLAADFDPDDFRHVAVWDAENEWIEMRLEATADMRVDVADLGLVLDFARGEQLRTEISAKFRLEGLDTELSAAGFTLDKAWTDPDNRFTLVLATRG

>CORE_REP|Org170_Gene998#

MGTVLSVVPSPVTVRAPSKVNLHLGVGDLRPDGYHDLTTVFQALSLSDDLEIAPAASLTVRVTGEGAGEVPTDRTNLVWKAAVRLAHLAGRAPLVEISISKGIPVAGGMAGGSADAAATLVGLNELWDLGLSREELTAVAAELGSDVPFSLHGGTALGTGRGERLLPVLSRNTFHWVIALAKGGLSTPAVYHELDRLREIGDPPRLGAPQELMQALASGDPKQLAPLLGNDLQAAAVSLKPELRRTLRAGVSAGALAGLVSGSGPTCAFLCESEESAVQVAAELAGAGVSRSVRTATGPVPGARVVGGEGPHPQPWREGGNIG

>CORE_REP|Org114_Gene5945#

MARRARVDAELVRRGLARSREHAVELISAGRVLINGTVATKPATGVETATPLLVREEPDEVRWASRGAHKLLGALAAFEPQGVTVAGKRCLDAGASTGGFTDVLLSKGAAAVVAADVGYGQLVWRLRSDDRVEVHDRTNVRALTPELIGGTVELVVADLSFISLGLVLPALALCCAPGADLLPMVKPQFEVGKERVGSGGVVRDPALRAEAVRAVAAAAARLGLRTHGVVASPLPGPSGNVEYFLWLRKELSGADHSTGSITGAAADSSASAHPGVQSVPEDGAGTGLPAAPGAAAVGAAAYDAVEEERVAALIQRAVEEGPQ

>CORE_REP|Org105_Gene1446#

MRYAAPPLECAQRHARSACGDLGGEHVIDGGERVTGLKTGLEAVLARAHELPSPPARGAVTLRARGVSVDRRGGGAKARRVLAEVDFEVAAGEVVALVGPNGAGKSTLLAALAGELDPTEGSVELDGRPLTQWTPLDMARRRAVLPQSHTVGFPFSAGAVVAMGRAPWQRTALRERDQEIIAASMAATDVTHLAEQAFPTLSGGERARVALARVLAQDTATLLLDEPTAALDLGHQETVLRLADERAAAGAAVVIVLHDLGVAAAYADRVAVLDAGRIAADGPPRDVLTTELLTRVYQYPVEVLDHPVTGAQLVLPVRGGGGE

>CORE_REP|Org114_Gene686#

MRAIQVSEHGGPEVLRYTEVPDPVIGPKQLLVDTEAIGINFIDTYIRTGRYPQNVPYVPGAEGTGVVAAVGAEVTEFQAGDRVAWAAAPGSYAERVAVDEAVAIPVPEGIDVPVAASALLQGMTAHYLVESIYKPEPGEAVLVHAGAGGVGLIITQLLAKRGVRVITTVSSDEKEKLSREAGAAQVLRYGDELASRVRELTGGVGVAAVYDGVGASTFEASLASLRVRGMLALFGAASGPVPPFDLQRLNALGSLFVTRPTLAHYTRDRAELLWRARDVMNAIADGTLRIRVGATYPLAEAERAHRDLEGRKTTGSIVLLPR

>CORE_REP|Org113_Gene7331#

MNTRMSVYRSASNSSTHGSEEGVPGRPTGGLLLVHAHPDDESVTTGGTIAYYRRRGVPVTVVTCTLGEEGRVIGERYAQLVSSTADQLGGYRIAELTRALAALDAGEPWFLGGPGRWRDTGVVGTSTGPGLPDPLHSRAFASAGDEAVHELVRVLLAIRPQVVIGYDPHGGYGHPDHIRAHQITMAAVDATAGLGWATPKLYWAVTDATVLHRHLRSLSHRSAELPPGWRLPVTGELASVPSSSVTTTIDVSEVMPAKLAALRSHSTQISVSPTGREFALSNKIAQPILPEEHFILVRGRLDAGCPARHERDLLAGLSANTK

>CORE_REP|Org113_Gene6136#

MTADAGSDATQAVPPATSCFRTAVVPAAGLGTRFLPATKTVPKELLPVVDTPGIELVAAEAAESGAQRLVIVTSPGKDGVVAHFVEDLVLESTLAERGKFHLLEKVRKAPGLLDVSSVVQEEPLGLGHAVSQAEQVLDDDEDAIAVLLPDDLVLPCGVLDVMTRVRRKRGGSVLCAIDVPKQEVSAYGVFDVVPVPDATNPDVLRVVGMVEKPKLADAPSTFAAAGRYLLDRAIFDALRRIEPGAGGELQLTDAISLLIAEGHPVHVVVHRGSRHDLGNPGGYLRAAVDFALERDEYGPALREWLQRRLAPDWNPQLTSPQ

>CORE_REP|Org112_Gene2009#

MSDKFEKPESAAATAAAAAAGTEPEPIKAIPLRRPGRWIAAAIILALLGLFLYGAATNPAYHWDTYANYLFDKRILEGALVTLELTVLAMVLGVVLGVVLAIMRLSPNPVLRSVSWVYLWIFRGTPVYVQLVFWGLFPGLYQTITIGVPFGPSFADFNVLDWRAPFLFAVIGLGLNEAAYMAEIVRAGVNSVGEGQREASVALGMSWSQTMRRTVLPQAMRVIIPPTGNELISMLKTTSLVTAIPLTTDLYGRARDIYGVNFQPVPLLLVAATWYLVVTSVLMVGQFYLERYYSRGSSRQLTGKQLRAMASQQHVVEEGK

>CORE_REP|Org215_Gene3040#

MAEVLVLVEHAEGAPKKVTTELLTAARSLGTPAAVVVAAPGTADKLGDALAAAGAEKIYVAESDDAEGFLVTPKVDVLAGLSESASPAAILVAATAEGKEVSGRLAARIGSGLLVDVIAVNGDGSAVHSIFGGAFTVDAKATGDVPVISVRPGAIEAAPQNGAGEKVAVEVPAQEEGVVKVTAREPIVGGDRPELTEAAIVVSGGRGVGSADNFGKVVEPLADALGAAVGASRAAVDSGYYPGQFQVGQTGKTVSPQLYIALGISGAIQHRAGMQTSKTIVAVNKDEEAPIFEISDYGIVGDLFNVAPQLTEAVKAHKG

>CORE_REP|Org24_Gene597#

MATARRGRRRSGRSHRVAARSARRARLTGVTLPSVTPVTGRLVVTGARGQLGRALLDLAPDARGYTHADLDITDLDAVRAALRCGDVVINCAAYTAVDRAETDIDAACAVNARGPMALAVACGEVGARLIHVSTDYVFPGTGSRPYETADPTGPTSVYGKSKLAGERAVADLLPETGHIVRTAWVYTGTGSDFVATMRRLERERETVDVVDDQIGSPTYAPDLAAALVELAEQPDAPRILHAANAGQASWFDLARAVFAGVGADPDRVRPCSTSAFPRPAPRPAYSVLSTASWTAAGLSPLRPWQDALNDALAAASD

>CORE_REP|Org172_Gene3028#

MVRRYTNALKTALLLGVLTALILTIGYALGGSTGLIVATVLSLVMNGAAYFYSDTIALHAMAARPVNEAQAPELHAMVRELATSAGQPMPRLYVSPIAQPNAFATGRSPRHAAVCVTDGILRLLTPRELRAVLGHELSHVYNRDILTSSVAAALAGILTSLANLALFLPIGSSTEDDDGPHPVAALLMLILAPVAAGLIQLAISRSREYQADVDGADLSGDPLALASALQKIDQWTRRLPLPADAPHAAYAHLMIAHPLSDDGVAALFSTHPPTAERIRRLRQLASQVAAGPSGPARPAPRSSWAGALAPTMRSLAR

>CORE_REP|Org158_Gene6059#

MASGTDGTLLCRYNRPRRDHLVPSNEQRRAAAKRKLERQLANRAARARKRKQLTIAASALGVVVAVAAGVGIYYLTRGDDDTATTATTSETPEASLASAPPSATPKPELVNCTYRDSGEAARPVDKPRADGIRTTGDDATLSVSMQTSQGPIGLTLNNAESPCTTNSFASLASQKYFDGTSCHRLSTSGLKILQCGDPTGTGMGGPGYAFDNEYPTDQYAPGDPTAQSVPVKYQRGVIAMANSGPSPDGTGTNGSQFFLVFGDSQLPPQYTIFGTIDETGLETLDKIAAAGDDGSMEPSPGGGKPNLQVTLESVQID

>CORE_REP|Org4_Gene1651#

MTEMIVNEASNVTQRALGISDFDTLAALESESIHVFREVAGEFERPVILFSGGKDSTVLLHLALKAFWPAPLPFALLHVDTGHNLPEVLEFRDRIVERYGLRLHVAKVEDYLADGRLTERPDGIRNPLQTVPLLDAISEHRFDAVFGGGRRDEERSRAKERIFSLRNAFGQWDPKRQRPELWNLYNGRHAPGEHVRVFPLSNWTELDIWRYIAREDIDLASIYYAHQRPVYQRDGMWMTPGVWGGPREGEALQTLSVRYRTVGDGSSTGAVLSDAADNEAILAEVAASRLTERGATRGDDRVSEAAMEDRKREGYF

>CORE_REP|Org113_Gene1830#

MELRQLRYFVTVVEEAGFTRAAQRLHLAQPGLSAQIRQLERELGQPLLDRSGRTVTLTAAGAAVLPHARAALAAAQQISHTADEFTGLLRGQVRIGLISGAATEEFDVATVLSAFHHDHPQIGISLTEDTTDRMLAAVARGALDIALVGLTGAPLDEGFGVDIVFETRLQAAVARENREFGDRIALADLRERSLICLPRGTGIRGVLEHACAAAGFEPRVDFEAAAPPLLIQLAAGGLGIAVVPALEPDQAAAAGVRMADITDPDLIGHLALVWRADRPLAPAAKVVLGQLRIALGRWKARHPISPGDGRGRNGSA

>CORE_REP|Org19_Gene6366#

MSALITPRDGRSCVVMGVVNVTSDSFSDGGRYLDPAVAVAHGVRLYEAGADIIDVGGESTRPGAVRIDPETEAQRVVPVIRGLVEAGVPTSVDTMRASVAAAAIDAGVSVVNDVSGGRADAEMVKVVAAAEIPWILMHWRANADHRHIGPADHYDDVVREVLAELSSQVDLAMAAGVHPSRLVLDPGLGFAKNAEHNWALLGALPELTAQGLPILVGASRKRFLGSLLGDESGPRPPDGREVATATISALAAQHGAWGVRVHDVRSSLDAIAVADAWRRAAESAERRAAESAERWAAESVERRAAEAGSHNQGSE

>CORE_REP|Org17_Gene6792#

MPPRTNPPIIRSLPVTTPDTTSTTGTARVKRGMAEMLKGGVIMDVVTADQAKIAEDAGAVAVMALERVPADIRAQGGVARMSDPDLIDGIVNAVSIPVMAKARIGHFVEAQILQSLGVDYIDESEVLTPADYANHIDKWQFTVPFVCGATNLGEALRRITEGAAMIRSKGEAGTGDVSNATTHMRKIRAEIRHLQSLPEDELFVAAKELQAPYELVREIAETGKLPVVLFTAGGIATPADAAMMMQLGAEGVFVGSGIFKSGNPAERAAAIVKATTFYDDPDVLAKVSRGLGEAMVGINVEEIPEPHRLAERGW

>CORE_REP|Org151_Gene1532#

MDWVRPRPLGSAAVSDSDSASAGLAVVTVTYSPGEHLEHFITTLADATTEKPQVILADNGSTDGVPELVAEANSHVRLLRTGGNIGYGGAINRAVAEIDPAIEFIVIANPDIRWGTDAIDQLLAAAQRWPRAGAVGPLVLEPDGSVYPSARRVPGLLDGAGHAILGTVWKTNPWTRRYRQENEEISERAVGWLSGSCLLVRRAAFDSIDGFDSRYFMYMEDVDFGDRMGKAGWHNVFVPSAEVTHAKGHAAGRHPEKMLPAHHASAYRFQADRHPHWWQLPLRLALRAGLAVRSRIAVRSALRQQAREAGHPV

>CORE_REP|Org9_Gene2292#

MHSAYARRMVDRVLVTLDGVVRDADEPLLFADDIGVLRGDGVFETVLVRDGDACAIEFHLGRLRRSAQALDLPEPELSRWREAVQTAAKEWGSEREGMMRLVLTRGRDTELGAPSSVTSGDLAAAVPVPTAYVLVVPVPERVAKARAEGVSVVTLARGISIDLAQAAPWQLLGAKTLSYATNMAALRFAHRMGADDVIFTSTENRVLEGPRSTVVIARDKELITPPAKNGVLPGVTQRALFTEAKKAGWECRYAPLFTADLLTCDSIWMLSSVTLAARVNSLDGLRMSAPDNAEEIIELVDRGVQRGGAIGDW

>CORE_REP|Org1_Gene4747#

MVMFSPPAAPLPTLCGKPVATDRALVMAIVNRTPDSFYDRGATFTDEAAMAAVDRAVAEGADLVDIGGVKAGPGSEVDAAVDIGGVKAGPGSEVDAAEETRRVVPFVAAIRAAYPDLLISVDTWRSEVARAAVAEGADLINDTWAGADPELVRVAAEHGAGIVCSHTGGAVPRTRPHRVRYADVVAEVTETVVAAAERAAAAGVRTDSILIDPTHDFGKNTYHGLELLRGLDVLVNSGWPVLMALSNKDFIGETLGVGLSERLEGTLAATAWSAAAGARVFRVHEVAHTRRVVDMIAAIQGIRPPARTLRGLV

>CORE_REP|Org84_Gene3006#

MTTETETTGVTDIGGTDIEFTKGHGTENDFVVLPDEDVRLDLTPARVAALCDRQRGLGADGVLRVARAGALLRAGVLDALPAGVSDTDWFMDYRNADGSIAEMCGNGVRVFAHYLAATGRAEGTEHVVGSRAGARPVTVHAAGPTHGEVTVAMGEVRALGASTATVAGWGYSGLGIDVGNPHLACVDPTLTAEALAKLDLTVSPGYDPDLFPHGVNVEILTPLDEQRAVDMRVYERGVGETRSCGTGTVAAAAAALSAEGFELATGSGAVTVRVPGGAVRVGLEAGSAWLRGPSVLVATGRLTADWWQTLG

>CORE_REP|Org46_Gene4697#

MTSEVLHRLSALDKAHVLADALPWLQKFRDKVVVVKYGGNAMVDEHLKQAFAADMAFLRTVGVHPVVVHGGGPQISAMLKKLGLQGEFRGGFRVTTPEVMDVVRMVLFGQVGRELVGLINSHGPFAVGISGEDAGLFTATRRTVEVDGEPTDIGLVGDVTEVNPDAVLDLIGAGRIPVVSTIAPDADGVVHNINADTAAAALAEGIGAEKLVVLTDVEGLYTNWPDRSSLTSRIDTAALAELLPRLDAGMVPKMEACLRAVSAGVPTAHVIDGRVPHAVLLELFTGEGIGTMVTPAPLIPSGSGAPDGTKQ

>CORE_REP|Org152_Gene1314#

MGEKRTPVVDALGGLLIVDKDGGWTSHDVVAKARRLLRTKKVGHAGTLDPMATGVLVLGVERATKMLGLLTLTTKAYTATIRLGQSTVTDDAEGEVTATTAAGHLTDAEIASGVAALTGDIQQVPATVSAIKVDGERAYARARAGEDVQLAARPVTVSRFDVLARREVDGADGQFVDLDVEVECSSGTYVRALARDLGARLGVGGHLTALRRTRVGPFTLEHARTLAELTAAAEAEEPLLSLDVDAAARTAFPVRAIDERQAEDLRNGRWLEPVGLSGVYAAIDPSGRAIALLQESGKRASSVMVVRPANL

>CORE_REP|Org122_Gene986#

MARMSEPSPYVEFDRKQWRTLRKSTPLVLTEEELIGLRGLGEQIDLEEVAEVYLPLARLIHLQVAARQRLFAATATFLGEKHPDRQVPFVIGVAGSVAVGKSTTARVLQALLARWEHHPRVDLVTTDGFLYPTAELTRRGIMHRKGFPESYDRRKLLRFVTEVKSGAEEVCAPVYSHISYDIVPGKLHCVRQPDILIVEGLNVLQTGPRLMVSDLFDFSIYVDARIEDIEKWYVQRFLALRKTAFADPDAHFHHYASLTDEQATLAAQEIWNSTNRPNLVENILPTRPRATLVLRKDADHSINRLRLRKL

>CORE_REP|Org204_Gene817#

MRETRTMPVPDGLDGMRVDAGLSRLLGLSRTAVAALAEEGSVQLDGVAAGKSDRLTAGAWLEVVFPEPKRELTIEAEPVEGMKILYADDDIVAVDKPVGVAAHTGVGWSGPTVVGGLAAAGYRISTSGAHERQGIVHRLDVGTSGVMVVAQSEHAYTVLKRAFKQRTVDKRYHALVQGHPDPSSGTIDAPIGRARGNDWKFAVTADGRPSVTHYDTVEAFQAASLLDIHLETGRTHQIRVHFSAIRHPCCGDLTYGADPRLAERLGLQRQWLHARSLGFQHPADGRYLEITSEYPADLTHALDVLRNA

>CORE_REP|Org1_Gene1776#

MTHKIGTDEGLSKTSTPGRHRGRRKVDQMSTLHKFKAYFGMVPLEDYEDDYVDDRAPRASERGGARGPRPYSERAGYGADRYGEDRYSADRFGPERFGAERFGPDRFGADRFDEDADYPEPAYKSYKSGYPVARRDDYPEDAYGEDRYEAPRRPTRIDAAPSSGRFRAGGGAPMLRGATRGALAVDPEAEERRLEERMRPEPVVARRPGIFEDGGPLSKITTLRPRDYSEARIIGERFREGNPVIMDLVELSNADAKRLVDFAAGLAFALRGSFDKVATKVFLLSPADVDVSAEERRRIAETGFYNQK

>CORE_REP|Org12_Gene4391#

MSIFLNKDSKVIVQGITGGEGTKHTALMLKAGTQVVGGVNARKAGTTVSHTAKDGSAVELPVFGTVAEAIKETGADVSIAFVPPKFAKDAIIEAIDAEIPLLVVITEGIPVQDTAYAWAYNLEKGGAEGPKTRIIGPNCPGIITPGESLVGITPANITGKGPIGLVSKSGTLTYQMMYELRDFGFSTSIGIGGDPVIGTTHIDAIEAFEKDPETKLLVMIGEIGGDAEERAAAYIKENVTKPVVGYVAGFTAPEGKTMGHAGAIVSGSSGTAAAKKEALEAAGVKVGKTPSETAALAREILEKASITA

>CORE_REP|Org140_Gene5470#

MPSSKHCPGLRWNTAPDLGRQRCRWVPYGGRVRVSPSSAAGIGPSATAAAADPVQAADGAPKSKTRTRPAETRLGLVRRARRMNRKLALAFPDAHCELDFTTPLELAVATILSAQCTDVRVNLTTPALFAKYPDARAYAEANRAELEEYIRPTGFYRNKANALIGLGQALLENFDGELPHTMDELVKLPGIGRKTANVILGNAFGVPGITVDTHFGRLVRRWGWTAEEDPVKVEQAVGELIERKEWTLLSHRVIFHGRRVCHSRKPACGVCLLAKDCPSFGIGPTDPDAAAELVKGPEAEHLLELVGR

>CORE_REP|Org35_Gene2401#

MRNHPGPGSAGDSSRLRASGTVGVAMVTPFSAEGKLDVDAGVALAARLVDRGVDLLAISGTTGESPTTTESEKADLLRAVVDAVGSRATVIAGAGTYDTAHSVELARNAQRAGAHGLLVVTPYYSRPTQEGLIAHFTAVADATDLPVTLYDIPPRSIVPIASDTIRRLAEHPRIVAVKDAKGDLNAGAELIATTGLAFYSGDDTLNLPWLSIGATGFISVIGHLVPERLRELVDAYTAGDVVRAREINAGLVPLNAAMARLGGVAMSKAGLRLLGIDVGEPRLPQLMPGPDQLDLLSADLRAAGVLG

>CORE_REP|Org6_Gene5554#

MTLPYVKLLVGNLVYAVSRKPLRATPGRTPTSNVDGMSEAVIPAAVGDDEVMTADTGGEQTLPLTGERTVPGIAEENYWFRRHEVVYARLLSRCAGKTVLEAGSGEGYGADMIAGVAAAVVGLDYDASAAAHVRGRYPRVRMIRGNLAALPLPDAAVDVVVNFQVIEHLWDQSQFLRECLRVLRPGGELLISTPNRITFSPGRDTPLNPFHTRELNAAELDELLVEAGFRVESMTGVHHGATLRALDTKHGGSFIDAQIQRALAGQPWPAELTADVAAVTIDDFDLRADDIDASLDLVAVAVKPGS

>CORE_REP|Org102_Gene1292#

MITVFICSERVAPAIRAAAPRRVSGVELLPLTPNGKTPVRVLTIAGTDSGGGAGIQADSRTMALCGVHACVAVAAVTVQNTVGVSGFHEIPPQIVADQVRTVVTDIGIGAAKTGMLASTTIIEAVAGVCREVGIGGGGDIPLVVDPVAASMHGDPLLHAEALDAVRNTLFPLATVVTPNLDEVRLLTGVEVVDDRSARRAAEALHALGPRWAIVKGGHLRSSAYSTDLLFDGENCYELTAERIATGNDHGGGDTLAAALACALAHGYPVPDAFAFAKEWTRRCLEAAYDLGAGHGPVSPLWRLHEL

>CORE_REP|Org127_Gene4988#

MTDNSERICAGRTVIVTGAGRGIGRAHALAFAAAGANVVVNDLGAELDGAPSADSPAAQVVEEIVQAGGRAVVNGDDVADWAGAKRLIGQAVETFGGLDVVVNNAGIVRDRMLVNLAEDEWDAVIRVHLKGHFATMRHAIEYWRAESKAGRARDARIINTSSGAGLQGSVGQGNYAAAKAGIAALTITAAAEFGRYGVTVNAIAPSARTRMTETVFADMMARPDDGFDAMAPENVSPLVVWLGSPDSAGVTGRMFEVEGGKVALADGWRHGVAEDRGARWQPSELGPVVRELIAKATDPEPVYGA

>CORE_REP|Org86_Gene5258#

MHHRGQDVVQSPARRSHGQAGRGDLDGHPRGRVNRGDRGIHDVEPGGWGKVQGVSGAAGTPVADAVAARAAELSANLDALLARIEAACRASGRAPDSVRMLPVTKFFPARDVAILHDLGLREFGESREQEASAKVAELSGLDGIAWHMIGRLQRNKAKVVARWAHTVHSVDSERLATALDRGACAALAAGERAEPVRVLVQVSLDADPARGGVVPAELDALADRIAAAEGLQLAGLMAIPPLDAEPDSAFALLETLHTRILARHPGARELSAGMSGDLESAIAHGSTCVRVGTALMGARPITSG

>CORE_REP|Org81_Gene2585#

MNVGAPDRARWVGENPEYKETATVQRIGVIGGGTMGAGIAEVAARAGGSVLVLERDTEAADAAVARIEKSLGRAVKSGRLEQAAADQARARITLTTAIDDFADRELVIEAAPEIESLKTDFFTKLDGIVSPETILATNTSSIPVIRLANATANPGRVVGVHFFNPVPVLPLVEIVVTLKTDREVADRVTAYARDILGKRTIESKDQAGFIVNALLIPYLCSAIRMYETGFASAEDIDEGMVSGCAHPMGPLRLTDTVGLDVTLAVAESLYAEFGEPQYAPPVLLRRMVDAGYLGRKTGRGFYTY

>CORE_REP|Org47_Gene599#

MTDSSNSAGTEPAPGSGATAELSKPAAAGSRGPNWSWLRVTGSVGPLGIATAVLWLSIIVLLPLAALTVSAFDEGWAGFWDAVTSPVALASLRVTVFVSVIVALINVVMGTLIAWVLVRDDFPGKGIVNALIDLPFALPTIVASIVLLSLYGPESPIDIHLNATQPGLVVALAFVTLPFVVRSVQPVLIEVDKEVEQAALSLGADNWTTFRRIVLPTLTPAIISGGGLAFARAIGEYGSVVLIGGNIPRETQMASQYIQQQIEIDRPVAAAAVSVALLVIAFVSLLVLRLFAERSARKEQEAR

>CORE_REP|Org66_Gene2436#

MAAAVSVHPRRHDLRRFERSAAQHHRRASARAPSGGSAVSGPLSVAPQPIPGHGLLTGRVAVITAAAGTGIGSATARRLLAEGADVVISDWHERRLGETEVELKGEFPERRVAAIACDVQSTTQVDELVRGAAAALGRIDIMVNNAGLGGETPVVDMTDEQWDRVLDITLNGTFRCTRAALNYFRAAGHGGVIVNNASVLGWRAQYGQAHYAAAKAGVMALTRCSAIEAAELGVRINAVAPSIARHAFLDKVSSSELLDRLSEREAFGRAAEPWEVAATIAMLASDYTTYLTGEVVSISSQRA

>CORE_REP|Org113_Gene136#

MRGIILAGGTGSRLHPITRGVSKQLVPVYDKPMVYYPLSTLMLAGVRDVLVITTPEDAESFRRLLGDGTQFGMSIDYVVQPEPDGLARAFVLGADHIGTDCAALVLGDNIFHGPGLGTRLRRFDGLDGGTVFAYRVSDPSAYGVIEFVGGKAVSIEEKPKLPRSSYAVPGLYFYDNDVVEIARGLRPSARGEYEITDINRTYLEQGRLRVETLARGTAWLDTGTFDSLLDAANYVRTIEERQGLKIGVPEEVAWRMGFIDDEQLSRLAEPLVRSGYGTYLMDLLTRGKNDGTTADEYRDEQDD

>CORE_REP|Org191_Gene1599#

MTTLTKFDQPVKAPTFRDISTSRKVKNHIATAVVSICFAVALIPLGWVLWMVVSEGIGAVLSSTWWMNSQKGILPDQSGGGVYHAIYGTIIQSAVAAIIAVPLGIMAAVYLVEYGRGRLAKVTTFMVDILAGVPSIVAALFIFALWIATLGFPQSAFAVSLALVLLMLPVVVRSTEEMLKLVPDELREASYALGIPKWKTIVRIVVPTALPGMISGILLSLARVMGETAPVLVLVGYAKSINTNLFDGNMASLPLLIYQELANPEAAGRERVWGAALTLILLIALLYAAAAVVNKLLTRNR

>CORE_REP|Org203_Gene471#

MRASFLFGEVVEGLRRNVTMTIAMILTTAVSLTMLGGGLLAVRIADKTEQYFLDRLEVRLYLTEDVSATDPDCSLEPCSSLMADLKATEGVESVQFLNRDDAIREAKEKTFKDQPELAEYVADTPLPASLRVKMVDAQLYPTIYESFYDRPGVGMVRNDKDIVDRLVSLFDGLRNAAFGLAILQAVAALLLIANMVQIAAFTRRTEVGIMRLVGATRWYTQLPFLLEAVVAALAGSLLAVAGLFIARPLVVDRALGDLFASKVFPRITGDDIAMTALIIAPIGVAFAAVTAYATLRYYVRE

>CORE_REP|Org17_Gene4533#

MSANASVLYDVPGPKARRRHALYSVLVLAVLVVLGWLVWRAFDEKGQLTAEKWKPFVESEVWQTYILPGLRGTVVAAALAIVFAMVIGVVFGLLRLSDHRVVRWVAGVIVEVARAIPVLILMIFLYNWFAKDNLFASDQLALAAVVIALTVYNGSVIAEIVRSGIRSLPRGQTEAAQALGLRKGQMMRIILLPQAVTAMLPALISQMVVALKDTALGYQITYQEIVRQGQQLGAAEQNTVPALIVVAVIMIALNWALTVLATRVEQRLRSRRRGRTVLGVNSVLTDAAPGVDLSLSRTAAP

>CORE_REP|Org120_Gene2356#

MIGPMKIRKAVIPAAGIGSRLLPLTKAIPKEMLPVGDKPVIEHTVRELVSSGITDITIVVSSGKSLIQDHFRPNPALVAQLRADGKTAYADAVEEVGELSRLGHITYLDQHGPYGNGTPVLNAARNLGDEPMLVLWPDDVFVADVPRAQQLINAYEQTGAPVLALMPMDPTESQRYGVPVVADDQGHGLLRITGLREKPKPEDAPSNYAAIGGYVVTPGVIEELRTQTRAWYEHRTGEVYLTDAINVHAADNPVYGQVIRGRWYDTGNPADYLVAQFASALANPQYGPLLRTLAEDTAS

>CORE_REP|Org109_Gene7387#

MRAAEEVVRPRPIGCVNVDTVSLSGKELAAAVNADTKARAAALTDRGTTPRLALIVANDDPASAWYVNSLRKAAERLGIACDTVDLGPEAGVAQIRAELTARGADAATDAIMLQTPLPAGVTLDDVSSAIVASKDVDGVSPLSLGLLAAGLDGFVPATSEAVVELLEHHEIPLAGRHVAVVGRSNVVGKPLAQLLLAKDATVTVCHSRTADLAAVTAAADVVVAAAGRIGLISGKHVREGAVVIDVGTNEAPDGKIVGDVDADSVRGKAAGLSPVPGGVGPVTTALLMRHVVIAAESR

>CORE_REP|Org49_Gene5817#

MSVGPRGHSDVPATQYEEESVKHIHAGKVRDLYEDGDELILVASDRVSVYDVVLPTPIPEKGALLTQLSNWWFRFFADVPNHLISTTDVPAEFAGRAVRAKKLSMVKVECIARGYLTGSGLAEYRRTGSVSGVALPPGLVEGDKLPEPIFTPTTKADEGHDEFITFDDVVNQEGREVAERLRDLTLDVYARGAEHAASRGVIIADTKLEWGWDGDVLTLGDEVLTSDSSRFWPADEYAPGRPQPSFDKQFVRDWSTSTGWNKEYPGPEIPADIVAATRAKYQQAYELITGETWTGVS

>CORE_REP|Org25_Gene1095#

MNSAGAAVNSEDARLLVERVGPLATIQDLGRPGWFDSGVGVSGAADRGALRLANRLVGNPEGHAGIEVLLGGLTIRTRRHTTLSVTGAPAPARVDGRPVGHASVLELEPDQELSLGIAATGLRSYVGVRGGVDVTPVLGSRSRDTMSGIGPAPLRPGIELPIGPAPRSFPTVDLAPVPDLPAVLDVRAIPGPRDDWFTDADVLFAGRWTVSADVDRIGVRLRREAGPVLERRLGQELPTEGMALGAVQVPPSGQPVVFLADHPITGGYPVIAVLADADVDVMAQARPGQTLRFRRV

>CORE_REP|Org159_Gene5771#

MMTQAPAGREKTRVITLTEPPKNSRLFAKAALGAVPLLSARKPTLPDRAVRLDGLRVDPDHLAAYCRATGLRFGDALPLTYPFILTFPLAMQLVVARDFPFVAVGAVHAQNVIERTRDISVSEPLDIRTHIENLREHPKGLLVDAISDVKVGRELVWHQVTTFLHQQRTSLSGGPKQEPKPDEVPPPPLRTLRVDQKTITRYAAASGDHNPIHTSALGAKAFGFPRSIAHGMWSAATVLGAVEGRIPEQTTYSVKFGKPILLPSAVNLYADQVEGGWDLALRHPKKGYPHLTATLR

>CORE_REP|Org151_Gene2073#

MRDKRMSLDEVVGELRSGMTIGIGGWGSRRKPMALVRAILRSDLTDLTVVSYGGPDLGLLCSAGKVRKAYYGFVSLDSPPFYDPWFAHARTSGALVAREMDEGMLKCGLEAAAARLPFLPIRAGLGSAVPDFWDGELRTVASPYPDADGRTETLIAMPALNLDAALVHLNLGDKHGNAAYTGVDPYFDDLYCLAAERRYVSVERIVDTDELVKTVPLQALLLNRMMVDGVVEAPGGAHFTLAGDSYGRDEKFQKHYVQSAKTPETWQQFVDKYLAVSEDEYQAAVREFAQEAQK

>CORE_REP|Org150_Gene6394#

MSTTSEYRVGDAMRQKRLGRKLFNAASGPVEGIAGLGDQLSFHLHGIAWIPRTLRRYRREMIRLVAEVSLGTGALAVIGGTIVIVGFLTAAAGYEVGQQGSNSLGRVGIEALSGFISAFFNTREAIPVVAGVALTATVGAGFTAQLGAMRVSEEIDALEVMSVPAVPYLVTTRILAGLIAIVPLYAIALFMGYASTQFVSIVLSGQSEGTYTHYFNVFLVPSDVIWSLVKVIAFALVVMSVHCYHGYHASGGPAGVGVAVGRAVRASLISIMIIDLIIGIAVYGGIHATVRVSG

>CORE_REP|Org138_Gene2183#

MSSSPPSAPDPSGPGIGSAQENQATKIRKRRRGKRRFAGGLVLLMGLVGAGFTASALTPDAQVATANEDQSALLREGKQIYDTSCVTCHGVNLQGVEDRGPSLIGVGEAAVYFQVSTGRMPAVRNEAQIMRKPPKFDARQTDALGAYIAANGGGPTVVRDADGEIAQESLIGGADLGRGGELFRMNCASCHNFTGKGGALSSGKFAPPLEPANEQQIYTAMLTGPQNMPKFSDRQLTPEEKRDIVAYVKDRTETQSEGGYGLGGFGPATEGLAAWIVGITLLVGSAMWIGSRS

>CORE_REP|Org128_Gene4969#

MRLPRSRVGGHPIHKVDAAREHATLPESSLPIGVSADYELPGAARSDVRTEVEVRPEAEVGPQARAVVANGADFDDTESVAGDAESVAGDAESVAGDAADDALSGTAAFDATGDRTMMPSWDELVREHADRVYRLAYRLTGDPQDAEDLTQETFIRVFRSLQNYQPGTFEGWLHRITTNLFLDMVRRRNRIRMEALPEDYDRVPSEGPGPEQVYHDARLDPDLQRALDALAPEFRAAVVLCDIEGLSYEEIGATLGVKLGTVRSRIHRGRQALREYLAHNGSQQRFAAEEKVG

>CORE_REP|Org127_Gene749#

MAVVTMKQLLDSGAHFGHQTRRWNPKMKRFIFTDRNGIYIIDLQQTLTYIDKAYEFVKETVAHGGTVLFVGTKKQAQESIAAEATRVGMPYVNQRWLGGMLTNFSTVHKRLQRLKELEAMEQTGGFEGRTKKEILMLTREKNKLERTLGGIRDMAKVPSAIWVVDTNKEHIAVGEARKLNIPVIAILDTNCDPDLVDYPIPGNDDAIRSAALLTKVVASAVAEGVQARASRASGDVKPEAGAGEPLAEWEQELLAQATPAAEGGEAAAEAPAETAAEAELKEEPATKTPADF

>CORE_REP|Org184_Gene952#

MFASGIRSSGIHALSVSSDVTPWLRSSGLEIVLLILGAVLFSRFATFVRDRVTSKIDAGFQSSDALVRTEAAKHRHALAQVVTWVVLTIVYVLVGMEVLQRLGFAVTGLVAPAAVLGAALGFGAQRIVQDILAGFFLITERQYGFGDVVRINVTGAADPAEGTVEDVTLRITTLRDADGQVIIVPNGQIVKVTNLSKDWARAAIDVPVSASADITRVNEILHKVGEEAYRDRRLEPLLLDEPTVMGVEDLTVDQMNIRMVARTLPGKQFEVGRELRVRVAAALRREGISETA

>CORE_REP|Org47_Gene2557#

MATAAQWIEGARPRTLPNAIAPVIAGTGAAASIDGLVWWKAILALLVSLALIIGVNYANDYSDGIRGTDDERVGPLRLVGSGLASPAAVRTAAIVSLGVGAIFGLILVALTAWWLILIGAACLAGAWFYTGGSKPYGYRGFGEIAVFVFFGLIGVLGTQFVQAERVDWVGAVVAVAVGAFSSAVLVANNLRDIPTDTESGKVTLAVKLGDPRTRTLHLVLLAVPFIATLLLVARSPFALVGLLAIPLAVRANAPVRSGRGGLELIPALRDSGLALLAWSVLTAAALGLAAL

>CORE_REP|Org214_Gene5800#

MTRIAVIGGGRIGEALIAGLLESGRLAKDLVVVEPVTERAAQIAEQFSVRVTDSVADAAVGADLLVVAVKPADVDAVMTALGKAALSDNASVGNDRDQILVSLAAGVPTARLEAKLPAGFPVVRVMPNTPMLVGQGMSVIAPGRYARAQQLELVTDVLGAVGKVVTVAEAQMDAVTAVSGSGPAYFFLIVEAMVDAGVGLGLTREVATELVVQTMIGSAALLQESEQSAAELRAAVTSPAGTTAAAVRELERGGVRSAFLEALHAAKQRSAEQGGVSDGPVGVGTGVGAGA

>CORE_REP|Org5_Gene1239#

MTKVTGTAEPGPGIRHTVKVGSMYAASREASSRAREALSAALTGSEAVAATTGSELFAVVAVLDDQRSLRVALADKSVASSVRADLAERVFGGKISAATQAVLTTAVAQDWSRTRDLVDTLVLLGQEALLRAAADRGRIDAVEDELFRLGRTVEDNPDLEQALTDRGKPAQAKRDLLARLLTGKVEDVTMQLAEQAVGRAHGDVGVAFDQLSDLAASLRKQIVAHVRSATALTQQQRDQLAASLQRIYDKPVTIHVQVDPTLLAGVVVHIGDDVIDGSAIGRLQRLRQALA

>CORE_REP|Org113_Gene5858#

MSAAQDNSVSDTSAETTAYGAAPATSRRKTRAHHLQQWKAAGEKWSMLTAYDYSTAKLFEEAGIPVLLVGDSAANVVYGYDTTVPITVDELIPLVRGVVRGAPNALVVADLPFGSYEGSPEQALASATRFMKEGGAHAVKLEGGERVSEHIARLTASGIPVVAHIGFTPQSVNGLGGFRVQGRGDGAEQLVADAIAVQEAGAIAVVIEMVPAEIAGRLTHKLTIPVVGIGAGNDCDAQVLVWQDMAGYTSGKTAKFVKRFGRVGDELRSAAAAYAEEVARGTFPGPEHSF

>CORE_REP|Org102_Gene5292#

MPDNPAPANLTPAEPTAADRAPADSAPLNLAAGAPAPGNPAQPVAFVTGAARGIGAAIAQRLAADGATVAVVDLDENSCAAAVDTIVAAGGKAIAVACDVTAEDQVDAAVDRVAAELGSLDILVNNAGVLRDNLLFKMSVAEWDTVMSVHLRGAFLCSRAAQRHMVAQRSGKIVNTSSVSALGNRGQANYSAAKMGIQGFTRTLAMELGPYGINVNAVAPGFIVTEMTAATAARLGVSSEELQAKTAEITPLRRVGQPADIADVVAFLASENAAFVTGQTIYVDGGRRL

>CORE_REP|Org117_Gene5952#

MPLLRVAVPNKGSLSESALTLLTEAGYRLPRVRNKELNCFDPENEIEFFFQRPRDIAVYVGAGTLDLGITGKDLLDDAAAPAESVLDLGFARSTFYFAARPDGPKSVADLAGRSVATSYPELVRKHLAQAGVSANVVVLQGAVENAVALGLADAIADVVETGTSLENAGLVTFGEPLMRSEAVLIRSTTAEWTDERAEAVTVMLDRLNGVLTARRYVMVDYDCPRAVLDAACALTPGIESPTVSPLADPDWVAVRSLVERKSINRTMDDLKKLGASAILATELAACRL

>CORE_REP|Org1_Gene3177#

MTAGDPMRLHPGHALSSFTEHLRALAPELLGPNRFAALDGATGSSGGTGAKDIAPHGTTIVAVSYRGGVLIAGDRRATQGNLLASRDMDKVYITDTFSAAGIAGTAGMAVELVRLFAVELEHYEKIEGVPLTFDGKANKLSKMVRDNLPAALQGLAVVPVLVGYDERAGDPDRAGRIVSYDVVGGRSEERFGYTAVGSGSMFAKTSLKKLYAKGIDQARALRIALESLYDASDDDTATGGPDLLRGIYPTAVVIDAEGALEVPESRLEEIARGIVADRTAAQEGSAGA

>CORE_REP|Org112_Gene446#

MSVRTRKPLVPGTQSPIREVPKSIERPEYVWKKTVNEGHEPWVQTPETIEKMRIASKIAAQALAEAGKAVAPGVTTDQLDAIAHEYLCDHGAYPSTLGYKGFPKSCCTSLNEVICHGIPDSTVIEDGDIVNIDVTAYIHGVHGDTNATFLAGDVDEEVRLLVERTEEATMRAIKAVRPGRALNVIGRVIESYANRFGYGVVRDFTGHGVGPTFHSGLVILHYDQPAVEAEIEPGMTFTIEPMINLGGIDYEIWDDGWTVVTKDRKWTAQFEHTLVVTDTGAEILTLP

>CORE_REP|Org144_Gene5001#

MGSRYTPPALRPFRLIGAAAQGPVRANQRAGHQAITFVAAIAAIPFALKHYRKEVLRLTADVGWGNGSLIVGGGTVGVVVILCGFGGITVGMESYTALNLLTMNPLTGAISGFATTREIGPILATLAFAIQAGCRFTAQLGAMRIAEEIDALESIAIRPLPYLVSTRMIAATLTIVPLYSVGLAVAYLMTKLSVLFLGGTSAGTYDHYFFQFLNGADVFFSVLKVMVFVLLSTFLQCYYGYVATGGPEGVGQAAGRAIKMVIVVMVFANLFLTLAIWGIDPGFRISG

>CORE_REP|Org17_Gene5165#

MMALDPQLDPGELRTLIRTALDEDLRYGPDITSAATVPAEATVKAAMVSRQPGTVAGIDVGLLVLDEVIGAGNYEVTDRVADGTRVGPGDAVLTVVAPTRQLLTAERTMLNLVTHMSGIATATAAWVDAVEGTECRIRDSRKTLPGLRALQKYAVRVGGGVNHRMGLGDAALIKDNHVVAAGSVVAALRAVRELDPDIECEVEVDSLDQLDAVLAEDVELVLLDNFPLWATQAAVQRRNSRSPRTKLESSGGLSLESAADYARTGVDYLAVGALTHSVRVLDLGLDM

>CORE_REP|Org1_Gene1643#

MAKGAWSGFAGEAAACGVRSRLGCRGGRWPHPSIVCRSSPPLNVEPSIQAKLLQLAAVDAELTRIAHRRTVLPEQQEVARLEARRNEHKDAAVKVEIVLDDLDRDIKKLEGEIEAVRKREERDRGMLTSGSVGAKQLSEIQHELGSLERRRGVLEDELLEVMERREASASDHDHAGAQLTRTEQELADAQRQRDEALADLDVAQARCENDRGELVGLFPDELLAVYDRQRAQRGVGAALLQARRCGACRIELDRGEIARIAKTAADEVVRCPECGAILVRTKESGL

>CORE_REP|Org176_Gene4330#

MPLTPADVHNVAFSKPPIGKRGYNEDEVDAFLDLVEQELSRLIEENADLRQRVAELDAELADAKKNRGPGVVNAVKPPVPQAPPPQPEPIKPPVPAAPPAPMPAAPVAKDAPGADANLQAAKVLSLAQEMADRLTSDAKAEAESLLSNARANSERLVGDARTRSEAMIADARQKSDAMLSDAQTRSDSQLRQAKEKADALQADAERKHTEIMATITQQRSVLESRIEQLKTFEREYRVRLKSYLESQLEELENRGSAVPVDGGEAFADANTANNLAPASFAKGGK

>CORE_REP|Org162_Gene2632#

MLRAAQSGWRRPRFARRATLRRSLRLLGSFKFEQTDPAVFYGGVAADTADLVGDFFRDLTGRSLRGTVVLDVGGGPGYFADEFAKAGARYIPVEPDPSEMHAAGLSVPGAVRGSGMALPFRDDAVDICVSSNVAEHVPQPWVMADEMLRVTKPGGLMVLSYTVWLGPFGGHETGPWHYLGGEYAARRYRRKHGREPKNRFGRSLFAVRAADGLRWARSAPPDIEILAVFPRYHPRWAWWLVRIPGLRELLVSNLVVVAGKRNSTLEAAATPESAQSARAFGFAPR

>CORE_REP|Org3_Gene3438#

MTHDQESGAGSVQTFGDRLQHAMRQFGPLCVGIDPHPGLLDQWGLTDDVDGLEAFAEICVEAFDGCVALVKPQVAFFEVYGAGGIGVLERTIEVLRDSGTLVLADAKRGDIGSTMDAYARAWLGDGPLASDAVTVSPYLGFGSLDPALELAQANHRGVFVLAATSNPEGAELQRITAGDGRSIAQTIVDAAAARNTGDSFGSVGVVVGATLTEAPDLSALNGPILMPGVGAQGGGAESVRGLVPEHLLHGVVPNASREVLREGPSVPALRAKLAAMQEEFGFLQA

>CORE_REP|Org5_Gene2415#

MSAAADRAALLATASEVLDTATPRFVEGVGAPSAVQKGRGDFATALDLELERTLSQQLLERTGIPVHGEEFGGPELSSGTAWVLDPIDGTFNYSAGHPLSGMLLALVEDGQPVLGLTWVPLLEQRYAAAVGGPLLLNGKPLPPLESGRLADAMIGFGAFNIDAHGRIPGRFRFDLLGALSRLSSRMRMHGSTGIDLAYTASGILGGAVVFGHHPWDNAAGVALVRAAGGVVTDLRGEPWSITSRSVLAAAPGVHEELLEMIDSAVDRAAERDDTAGRDENSEGTQ

>CORE_REP|Org4_Gene3472#

MVRPSAGGRCLPAARAAVVGTASGPCNTVVDVIVALIDSGLGLLPTSAWLRKLRPDVDLLLQLDPDGAPWGPKPEQWTIDRVVRAAGMSIELGAEVIVIPCNTASVTALEHVRAEVGPDVPVIGTVPAIKPAAAVCRSVAVWATAATTASRYQADLIAKFGGNADVVGVACHGLADAIDRGDLVGARESIARAVAETPDDVEGVVLGCTHYPLVIDAIVAALPDGVRLFDSAQAVAAQTIRRMDALGRPTPGNGAVLVRNSGRPGELPASAAAFESGRILGAQG

>CORE_REP|Org1_Gene1097#

MHGAPARPGGPRGFSGDRFVSEISSWQHVGMRLTVLGCSGSVSGPDSPASGYLLTGPDMTPVVIDFGPGVLGALQRYADPGEVDIFLTHLHADHCLDLPGLLVWRRYHPTPPVGRAIVRGPSDSALRIGNASAEVGGECDDWSDVIDLRPWQEGETVEFGPGHTIAARRMFHPPESYGLRITTNAGRTFVYTGDTAMCDAVQELAQGADVLMAEASWTHDPANRPPGIHLSGTEAGRIAARAGVGELLLTHIPPWTSREDVIAEAKAEFTGPVHAVAPGEVFDL

>CORE_REP|Org102_Gene1101#

MNVTVLAGVGESFQQTASSGPLLLALGACVLAGLVSFASPCVVPLVPGYLSYLAGLVGAEAPPVSVESAKREAARGGSTALAEKTAKSRARLRVAGAAGLFVAGFTVVFVLATATVFGAIQVLNVNRELLQRVGGVVTIVMGLAFIGLIPALQKDTRMEPRRLSGIVGAPLLGAVFALGWTPCLGPTLSGVMAVSAGTDGTTAARGVALIVAYCLGLGLPFVILAFGSATALRGVGWLRRNSRTIQVIGGLLLVAVGIALVTGAWDQFVSWVRDAFVSNVTLPI

>CORE_REP|Org43_Gene3246#

MSRVSIDTHQAWVEFPIFDAKSRSLKKAFLGKAGGAIGRNQSDVVVVEALRDINLSLREGDRIGLVGHNGAGKSTLLRLLSGIYEPSRGSARIRGRVAPVFDLGVGMDPEISGYENIIIRGLFLGQTRKQMMSKIDEIADFTELGEYLHMPLRTYSTGMRVRLAMGVVTSIDPEILLLDEGIGAVDAEFMKKARLRLQELVARSGILVFASHSNEFLAQLCDSALWIDHGQIRLRGGIEEVVRAYEGPDAGNHVATVLREMAAERAGRAEGSADERELEQNAT

>CORE_REP|Org89_Gene2183#

MSDRTAGMTEPAARTRERTLSVTTLAAGEFHAPSLTDFFPPAVLFEGTPFELDRLMLVRLLMTAVLVAVMLLAFRSPRIIPRGLQNVAEIGLVFVKEQIAEEVLGKETGRKFFPLIATIFFTVLFLNFSGIVPLLNISSNARIGMPLVLAVVAYIAFNYVGIKKYGFFTYMRSSIVVPNVPPALHVLLIPIEFVSTFILRPFTLTVRLMANMLAGHIMLVLFFSATWYFLFDAAAWMKVFSPFSLLAGLGFTLFEMLVIFLQAYVFALLTAVYIGLAEHADSH

>CORE_REP|Org5_Gene5557#

MRLRDNLFGVADESESVAVPGADDEPKDESASRRRRRGGKKRKQRPFWQELPILIVVAGVIAALVVNFIGRPYVIPSQSMEPTLHGCTGCVGDRIYVEKLSYDFGDPKPGDVVVFKGPSESWNKGYHSQRSSNVVKRGFQNFFSFFGLVPPDENDLVKRVIAVGGQTVQCCDAQGRVMVDGKPLDEPYANYKYPYQPGLPFATKVAGSLIVDPHGREFGPIKVPDGNLWMMGDNRNESLDSRGHVDDEYSGTVPIDDVRGRAVFKIWPPSRIGPVRSQNPQSN

>CORE_REP|Org128_Gene3051#

MRSGTTTVRAGRSSLRGVQRPPLDVDILRHAVAEQPDLSFFSRIDVVESTGSTNADLIGEAGDPSSDRRVLVAEYQDRGRGRHERSWVSPPRAQIAMSILVRLGGIEPAVLGWLPLLTGVAVVDAVRETTGLDANLKWPNDVLIGGRKVAGILAEVASGAGAPAVVVGVGLNVSLTEDELPVPHAVSLTLAGAENADRNELVLALLRAFARHFTEWRTENWNVAALADAYRARCATLGADVRAELPGGEVITGVATDIDAYGRLIIGDRSVSAGDVTHLRPA

>CORE_REP|Org194_Gene3604#

MSNPGTGWEMAGPAWLFCPADRPERYAKAAAAADVVIIDLEDGVAEADKAAAREALIATPLDPDRTVVRVNAAGTVEHMLDLDAVARTGYRRLMLPKCESAEQITTLADYEVIALVESPLGALAVGRAVMARNAIGVMWGAEDLVAGLGGNSSRHADGSYRDVARHVRSQSLLAAKAYGKFALDSVYLDIPDLDGLAAEALDAVAVGFDAKVAIHPSQVPVIRRAYAPTGAEIDWARRLLAEVPNHRGVFTFEGRMVDAPVLRHAERIVRRAQGADSANVGS

>CORE_REP|Org102_Gene4695#

MPAVSERQRGRGGDAARNLAIPAGIGGYAAIILSDMAAGKGGKPSKEAKAAAKAARKQASKERRQQLWQAFQMQRKEDKLLLPLMIGALVGVTALFLIIGLIFDLQWFLLPIGVLLGALAAFIIFGRRVQKNVYAKAEGQAGAAAWVLDNLQGKWRVTPGVAATTQLDAVHRVIGLPGVILVAEGSPGRVKSLLAQEKKKVARLVGDTPIYDIVIGNDEGQVALKDLQRFLTKLPRNIDAKRMELIEGRLSALATRGGPALPKGPMPTGAKMKGMQRTIRRR

>CORE_REP|Org97_Gene4529#

MGNLREQIIAELGVAAEIEPKVEVRRRVEFLADYLSSTPATGFVLGISGGQDSSLTGKLCQLAVDELRARGQEATFVAVRLPYGAQADADDAQRALDFIGPDHVVEVNVKPGADAVAAATAEGVRELLGHETELRDFVRGNIKARERMIIQYAIAGQLNLVVVGTDHAAEAVTGFFTKHGDGGVDITPLTGLTKRQGAALLQELGAPPSLWEKVPTADLEDDRPALPDEEALGLKYAQIDDYLEGKDVAPEVAERVETIYCNTRHKRTVPVSPLDSWWKN

>CORE_REP|Org160_Gene3260#

MRINRALRLGVGAIALALTAATTAGCGSGDDKTALDHAKEGKLTIGIKFDQPGLGQRNTDGTYSGFDVEVARFVAAKLGVQPDGITFKEAPSAQRETLIENGQVDFIVATYSITDQRKEKVDFAGPYYVAGQSLLVNADNTDITGPETIAGKTVCSVKGSTPAQNIEKNFPDTQLQTYDTYSLCLEGLNSGAVDAMTTDDIILAGYAAQTPGRYKLVGKPFTTENYGIGLKKGDQESRDKINDAIEAMITEGAWDKAFQDSVGRAANYPTPPAPQVDRY

>CORE_REP|Org38_Gene1209#

MAIRKYKPTTPGRRGSSVSDFAEITRSTPEKSLIRPLHSKGGRNAHGRITTRHRGGGHKRAYRLIDFRRLDKDGIPAKVAHIEYDPNRTANIALLHYVDGEKRYIIAPKGVVQGTPIESGPTADIKPGNNLPLRNIPTGTTIHNVELRPGGGAKMARSAGSSIQLLGKEGTYATLRMPSGEIRRVDVRCRATVGEVGNAEQSNINWGKAGRMRWKGRRPTVRGVVMNPVDHPHGGGEGKTSGGRHPVSPWGQPEGRTRKPNRPSDKLIVRRRKSGKNKR

>CORE_REP|Org5_Gene5353#

MTDAADATPGAPGTGNTAEPDATTAAGASARTDAAPPMISMRNVDKHFGDLHVLRDVNLEVPRGQVVIVLGPSGSGKSTLCRTINRLEPIDSGTIAVDGVELPAEGRALAKLRADVGMVFQSFNLFAHKTILDNVLLGPVKVRRVDKKRARARAMELLERVGIADQADKYPAQLSGGQQQRVAIARALAMDPKVMLFDEPTSALDPEMVNEVLDVMVALAKEGMTMLVVTHEMGFARRAGDRVLFMADGRIVEDAPPETFFTAPASERARDFLGKILSH

>CORE_REP|Org19_Gene6391#

MTSRPRPCSTRFSPRPPDRIALTLNSRRVVALVPAAGRGVRLGESTPKAFVPVGGSPMLVHAVDGLITSGVVDRIVIMAPIEMIDAARELLAARAHAASSIPVDVVAGGVERTDSVRAGLAAAPEATHILVHDAARALTPPSLIARVVGALDAGHRAVVPGIPVADTIKAVDAAGDVTGTPDRSGLRAIQTPQGFDAALLREAYAVDLPATDDAGLVEAMGATVSVVPGDPLAFKITGPLDLRLANALVADDSAAAGGASGAASAETAITAATRAAVTG

>CORE_REP|Org19_Gene2600#

MRNPLATPTGCGCPARHRRRREQHCAARSRRRPGAGVPQDTGGVVTSAERPPAATRVLVVDDEPQILRALRINLSVRGYEVITAATGAAALRAAAEKHPDVVVLDLGLPDIDGVEVLAGIRGWSSMPVIVLSARTDSSDKVQALDTGADDYVTKPFGMDELLARLRAAVRRSASTAEESAPIVETSSFTVDLAAKKVIRGGRDVHLTPTEWGVLEMLVRNQGKLVGRRELLREVWGPTYATETHYLRVYLAQLRRKLEDDPSQPKHLLTEAGMGYRFQA

>CORE_REP|Org31_Gene3788#

MGGLLEGKTILVTGIITDSSIAFHAAAVAQEQGAKVIITGIPERLRLIDRIAKRLPQEVPPAIPLDVTSEENLAELADKLRELAPEGIDGVLHSIAFAPRTLMGPEALPFLDGPGPDAAKAFEISAWSYASLARAVLPVMNERGSIVGMDFDPRTAMPFYNWMGVAKAALESVNRYVAREVGAAKKIRSNLIAAGPIKTLAAKAIAGTATDDAAKLNQLNEYWDGASPIGWDVDDPTVVAKSIVAMLSDWLPGTTASIIYVDGGASHNTWFPEDMSIN

>CORE_REP|Org127_Gene922#

MPGKRPAPEPPEPLSPLIDAHTHLDACGAEDAESVAAMVDRAAAVGVGRVVTIADDLDAARFAVDAAHWDPRVYAAVALHPTRANALDDAARAELEKLAADPRVVAVGETGLDYYWPGKLDGCADIEDQVEGFRWHIDLAKRLGKPLMIHNREADHDVLAVLLDEGAPDTVIFHCFSSDANMALACVAEGYLLSFSGTVSFRNAHELREAAKLVPDEQILVETDAPFLTPHPFRGAPNEPYCLPYTVRALAELREQDPAALAEITTANAERVYRLQRG

>CORE_REP|Org120_Gene7219#

MGAVILRRDSSTATRPNPAGSVATSTVRPPSQHPSGARPPAIPAELVPNHVALVMDGNGRWAQERGLPRTAGHERGEAVLMDTVEGCIEMGVKWLSAYAFSTENWRRSPDEVRFLMGFNRDVIRRRRDEMNEMGVRVRWAGRRPRLWRSVINELEIAEEMTKHNTVMTLTMCVNYGGRAEIADAAREIARRVAAGEIDPEKVTEATVARFLDEPDMPDVDLFLRPSGEFRSSNFLIWQSAYAEFVYQDTLFPDFDRRNLWAACLEYASRDRRFGGTK

>CORE_REP|Org112_Gene4523#

MTTASAATGSNTDGTEEPHALVEQRGATLIVTMNRPRSKNALTGEMLSIMAEAWQRVDSDPEIRSCILTGAGGAFCAGADLKNMARSNPGDNMTAGSSFDPTRMPGLLKGYRLSKPLIAAVEGPAIAGGTEILQGTDIRIAGASAKFGVSEAKWSLFPMGGSAVRLPRQIPYTLAAEILLTGRHITAAEAKEFGLIGHVVPDGTALDKALEIAELVNNNGPLAVQAILKVMRDTEGMHEEEAFQIDAKVGLPVFRSEDAKEGPRAFAEKRKPNFQGR

>CORE_REP|Org4_Gene5772#

MTGAAQWARGGMSIGSVLDLLRPDFPDVTISKIRFLEAEGLIRPERTPSGYRRFSVADCERLRFVLTAQRDQYLPLKVIKEQLEAIDSGAASLGVREARARAHSGRAGAAEPTATGSASGHPAASGGNSNGAAAPRRLGVVPSEISPDDLRFDHEIRLTRADLLAKAEIDDAFLNDLIRANLITPGAAGFFDGDAVTLAKTAKAMAEFGLEARHLRAFKLAADREAALVAQIAAPIAKSRDAGARARAEETVRELAALSLTLHACLVKSSVRTSLGG

>CORE_REP|Org42_Gene423#

MTVHSSSADRVLVRKQGPVTIVSINRPEVRNAVDRATAEDLAAAFREFDRDPDAAVAILTGEGGTFCAGADLKAVAAGDPNRFASDGDAPMGVSRMRLSKPVIAALSGHAVAGGLELALWADLRVADENTVLGVFCRRWGVPLIDGGTVRLPRVIGLGHAMDLILTGRPVSAHEAQGMGLVNRVAPAGRSLATAVQLAEQLAAFPQTCMRQDRLSALEQEGLGETEALANEYEHGVVSISTDTLAGATRFAGGAGRHGSFTDLGNPEDAPERSTQS

>CORE_REP|Org129_Gene1057#

MSSELQVDVSAGVAVLTLNRPAQQNAMTPTMAVELGTALRRCDTEDAIRAVVITGTPPAFCAGADLSARVGDVRGTIDPPPWQIRKPVIAAVNGHAVGIGLSLALQCDLRYMATDAVYGLNQVRRGAMADGYAHWTLPRLAGMANAADIMLTGRTFDGEEARQMGVANSSLPAGEVLPTALAVAHDLAAGSAPLPTALTKRLLWEGLGMSPEAVGRLESELHGFVGKSVDAAEGMAAFRDRRQPQWKGSISAEWPAGELSSPGERRGLDGTAEEPA

>CORE_REP|Org37_Gene6251#

MHVSETPGTETGTEITETGGTETPFVRYEVRDGFAVLTLDSPHNRNALSSKLVRELLDGLRKAGADEQARGVILTHTGNTFCAGADLKEALDADPAAAADIRTGWMIDVLRGIVELHKPVVAQVDGNVRAGGMGIVGACDIAVAGPSSSFALTEARLGLAPFMISLTLLPRLTSRAAARYYQTGETFDAAEAERIGLITVAAADAAAEVARLCGELRKGSPQGLAESKRLVNASIVAEFDRTADELAKRSGSFFGTPEVIEGMTAFFQRRPPSWAE

>CORE_REP|Org109_Gene5232#

MNASQSDSVGGDGRATGGREFETILLERKGRVGWITLNRPKALNALNAQVLDDVIAALDELEHDDEIGVIVITGSERAFAAGADIKEMQPKSYMDMFMDDFFARWDRLAQFRKPTIAAVAGYALGGGCELAMICDILLAADTAKFGQPEIKLGVIPGIGGSQRLTRAIGKAKAMDLVLTGRNMDAEEAERAGLVSRIVPAAQLLDTALEVAETIASMSLPVAMIAKEAVNRSFETTLAEGLRFERRVFHSLFAIEDQKEGMSAFVEKRPAKFTNR

>CORE_REP|Org74_Gene3418#

MANYTAADVKRLRELTGSGMMDCKNALAETDGDFDKAVELLRIKGAKDVGKRAERTTAEGLVAAKDGVMIEINSETDFVAKNDEFQALANQIVTAAAAAKTADLDSLKALDLGDGRTADAALQELAAKIGEKLELRRVVSLDGPVATYLHKRASDLPPAVGVLVEYQGAGDAAAEAARAAAMQVAALKAKYVTRDEVPADIVENERRIAEQTAREEGKPEAALPKITEGRVNGFFKDVVLLEQPSVTDNKKTVKQQLDEAGVTVTRFARFEVGQA

>CORE_REP|Org75_Gene2980#

MFRRAHADHQNLIPLLPRSNVSPMRDLDNSAPRADRPASEAEHRAALRRVAADLAAMRVDYGGVPSGSGEDVDLDEAWLAGGWEPLLRNWIEQATAVDIAEPNAMVLATVAVVDGVPRPASRTVLCKGLSPEGVTFYTNYDSAKGTQLAAVPYAAATFVWPALGRQVHLRGPVERTSAEQTAVYWRSRPRDSQLGAWASQQSRPIDSRAALDRALAEVTARFAGVEEIPVPPHWGGYLLRPEQVEFWQGRRGRLHNRLLVRVAGERMTVERLQP

>CORE_REP|Org163_Gene2356#

MTTLEIKDLHVEVANPDESGEPIKILKGVNLTVRSGETHAIMGPNGSGKSTLSYAIAGHPKYTVTSGSITLDGEDVLEMSVDERARAGLFLAMQYPVEVPGVSMSNFLRTAATAVRGEAPKLRHWVKEVKESMSELEIDAAFADRSVNEGFSGGEKKRHEILQLGLLKPKIAILDETDSGLDVDALRIVSEGVNRYKERENGGVLLITHYTRILRYIQPEFVHVFVGGRIVAEGGAELAEELDANGYVRFTQHSSTPSASPTAVPQSATAGA

>CORE_REP|Org38_Gene1444#

MTVHTHPGGSAASPSPGVLEKTPPAKPAPVPEGSVMVTVKVARFNPEDDKGAHWESFQVPVLPTDRFLNVLIYIKSYLDGTLTFRRSCAHGVCGSDAMRINGVNRLACKVLMKDMLPKGDKTLTITVEPIRGLPVEKDLVVNMEPFFDAFRAVKPYLMTSGNEPTRERIQSQADRARFDDTTKCILCACCTTSCPVYWSDGSYFGPAAIVNAHRFIFDSRDEGARERLDILNDVEGVWRCRTTFNCTDACPRGIEVTKAIQEVKRALLFAR

>CORE_REP|Org103_Gene1084#

MTDLTAAFAASVACGAMSSDLLGKSALVSGASRGIGKAVAAELLRRGANVLITARKPEPLAEAAAELRALGHQGEVATIAGNSGDAQARAEAVGRAVTEFGSLDILINNTGINPVFGALMDADLDAVRKIFDVNVVAALGYAQEAYKAWMGEHGGAIVNVASVAGLRSTGVIAAYGASKAALIRLTEELAWQLGPKIRVNAVAPGVVKTKFADALYSADEERAASVYPMKRLGSPEDVARLIGFLASDEAAWITGETVRVDGGLLATGGI

>CORE_REP|Org127_Gene2324#

MGQKINPHGFRLGITTDWKSRWYADKQYKEYVKEDVAIRKLLSTGMERAGISKVEIERTRDRVRVDIHTARPGIVIGRRGAEADRIRAELEKLTGKQVQLNILEVKNPESDAQLVAQGVAEQLSNRVAFRRAMRKAIQSAMRSPNVKGIRVQCSGRLGGAEMSRSEFYREGRVPLHTLRADIDYGLYEARTTFGRIGVKVWIYKGDIVGGKREVTASAAPSGERRERRERPSRPRRSGSSGTTATSTEAGRAATAVAEPAEAPSVSKEG

>CORE_REP|Org127_Gene2533#

MKLSAPSRISLRVIALAYLIVLLVAPLVIILWRSFEHGIGAFLDSITTPAAISAFNLSLLIVVIVVPLNVVFGVITAIALVRGNFRGRTLIQGVVDLPFAVSPVVVGVSLIMLWGVGGWFGGLDSFGFRVIFGLPGMVIATLFVTLPFVVSEVVPVLHEIGDDQEQAAATLGASRWQTFWRITLPAIRWGLTYGIVLTVARALGEFGAVIMVSSGLPGISQTLTLLVHGRYINDHNTFGAYCAATLLMAMALVTLVLMTLLERKRGTAK

>CORE_REP|Org151_Gene5632#

MPVGRRVARVELPVQVRGLPYRIYEARLTKQLAGKQHPRHVAVMCDGNRRWARENGFADVSHGHRVGAVKIAELVGWCQAEGIEMVTVYLLSTENLQRDPDELETLFEVITDVVEELSAPEQNWSVRVVGSLDGFPELIAKRIRTAAERTEDRNGVHVNVAIGYGGRQEITDAVRSLVRQEIAAGETGEDLVQSITVNAIGQHLYTSGQPDPDLVIRTSGEQRLSGFLLWQSAYSEIWFTEAYWPEFRRVDFLRALRDYAARHRRFGI

>CORE_REP|Org77_Gene3533#

MALEIDLSGRVVLVTGGVRGVGAGVSRALLAAGATVLACARRPGDAPVEYEGRQAEFLPCDVRDGDAVRELIDTVIARHGRLDHLVNNAGGAPFALAADASAKFHAKIVELNLLAPLLVSQLANAVMQAQPDGGTIVNISSVSAHRPSPGTAAYGAAKAGVDSLTASLAVEWAPKVRVNSVVVGPVETELSLLHYGDADGVAAVGATIPLGRLARPEDVGRCVAFLASPLAGYVSGATLEVHGGGERPAFLDAATVNTAAPNGAPKP

>CORE_REP|Org158_Gene1249#

MRRGGVDAETADPVDDSVDPDETGRRADAARWARLRRGRREPWWIRVRESRRARIGVAAGVVVALALGLVAYFSPLLAVRTVRVDGLSVVTEAQVLDALDMPAGRSMLRIDTTELAQRVARLPKVHSVRVQRVFPSSVRVTVVERTPVLFFEAPDGAHLIDSESVEFAIEPAPIGVPKLVTETPGGNDPATRAAVTVINAVPVSLRLEVGEIVARSVSDIQLELRDGRRVLWGGAGDSERKAAVVIPLLTRDGEVFDISSPNLVTVK

>CORE_REP|Org147_Gene1577#

MNNLHRELAPITSEAWSAIEEEATRTFKRHIAGRRVVDLSGPHGTDYSAVGLGRTAPIAAPDDGVQARQRLVAPLVELRVPFTLSREELDNIERGAEDADLDPVKDAAKRIAWAEDRAIFEGYPAANITGIRASASNEPVAVPSDPRLVPEAVAQALSELRLAGVDGPYSVLLSADLYTAVSETSDHGHPIRTHIERLIPEGEIIWAPAIDGAFVLTTRGGDFDLQIGQDLSIGYLSHDAESVQLYFQQSLTFLVYTAEAAVALQA

>CORE_REP|Org176_Gene1764#

MTLPYYASAEQIMRDKTELARKGIGRGRSVVVLTYDKGVLFVAENPSTTLHKVSELYDRVGFAAVGKYNEFENLRRAGILQADIRGYQYDRRDVTGRALANAYAQALGSIFTDQLKPYEVEICVAEVGYPEQAPQSVLYRITFDGSIVDEREFVVMGGTTEPIVTALKSSYQPGLDLASAIGVAVRALQAGVPEGAEKEKRALGVSSLEVATLEQARPRRAFRRVAGAALERLLNPAQEAGASAAEEGASGPKDEEPAAKEAPPAE

>CORE_REP|Org195_Gene78#

MLLTIDVRNTSIELGLFSGSGAHSKLVRHWRMHTNPLLTADEFAMQVRGLVGAQLDEVIGVSALSTVPPVLRELRAMLGQYWAHVPHVLVEPGVRTGIPLLVDNPKEVGADRIVNCLAAYQRYSAPAIVVDFGTAICVDLVSKKGEFLGGIIAPGVEISTEALVERSALRRAELTRPRSVLGKNSMECMQSGAVFGFAGLVDGLIDRIRDEFDAFAGDDVAIVATGATAPLIVPESETIDDHDPHLTLTGLRLVYERNQRRKGSV

>CORE_REP|Org19_Gene2707#

MRGRLLHFEHTFDTRLRRAALRGPARLVAQTGSTQEAVIEEQARVIGSILPAGVAAAELLRYQEDLKPHPGEEHLIAQSVEKRRRDFIGARHCARLALAQLGEPPVAIGKGERGMPLFPRGVVGSLTHCDGYRAAVLAHRLRWRSVGIDAEPHDTLPEGVLDSVSLPAERDWLRGAIPATGLHMDRLLFCAKEATYKAWFPLTQRWLGFEDAHITFTVDDTGTAGTFHSALLVPGQTVDGGAPLTAFDGRWTITDGLILTAIVAG

>CORE_REP|Org63_Gene4595#

MILDRFRIDDQVAIVTGAGRGLGAAIAVAFAEAGADVVIAARTESQLEEVAERVAAAGRQAHVVPADLSDADATAALAASAVERFGRLDIVVNNVGGALPCPLLDTTPQALAQAFDFNVVNAHALVRAAVPRMLETAGGGSILNITSTMGRLPGRAFAAYGTAKAALAHYTKLAALDLNPRIRVNAIAPGSILTSALEIVASNDAMRTELEAKTPLHRIGEPEDIAAAALYLVSPAGKYLTGKILEPDGGLIIPNLDLPIPDLT

>CORE_REP|Org21_Gene1896#

MTKPDLSTNRFPAGTFTPAPRPNTRATMLAAQTRLELILLLRNGEQLLLTMLIPITLLVGLTLLPFGDLGTDRVDQIVPAVMMVAVMSTAFTGQAIAVGFDRRYGALKRLGATALPKWGIVAGKSAAVLIVVVLQALLLGLIGAALGWRPEPAGLLLGAVVIALGTATFATMGLLLGGTLKAEVVLALANVLWFVMLGVATVVFASDDLPTAVSVLARLIPSGAMAVALEDALRNSIDWFGVAVLAAWGLICGFAATRFFRFH

>CORE_REP|Org15_Gene4889#

MSDGTGLLADKVVVISGVGPGLGRSLCVQAAAAGAKVVLAARTESRLREVADEIDGAGGTSLIVPTDITDDAAVANLVERTVATFGRVDALINNAFAMPSMKSLARTDFQQISDSLELTVLGTLRATQAFTDELAKTRGAVVMINSSVLRHSEPRYGSYKVAKSALLAMSQTLATELGAKGIRVNSVAPGYIWADRLKWYFGEVAKKYGITVEQVYEQTASRSDLKRLPEPDEIARAVVFLASEWASAITGQTLDVNCGEYHA

>CORE_REP|Org37_Gene1288#

MPAPGRRSRRGPASARRIRGHGPAAPGHDGNGEPVSGRADSDGDTPIRVLLVDDEQLVRSGFRLLLDIEDDITVVGEAANGAEAVRKARALRPDVVLMDIRMPTMDGIQATREIAATTGLQDVRILILTTYDTDAYVFEGLQAGASGFLLKDAGPAELLHAIRVVAAGEALLAPRITRRLIAQFTARRAADRAAEQRLAVLTDREREVLALVGQGMSNAEIGAELFLSPATARTHVSRAMVKLGARDRAQLVVIAYRTGLVAP

>CORE_REP|Org10_Gene6258#

MLAARPGVGDRGAPTKLLEVSLVLLPAVDVANGEAVRLVQGEAGSETSYGSPRDAALAWQEAGAEWVHLVDLDAAFGRGSNRELLAKVVGELDVKVELSGGIRDDDSLEAALATGCARVNLGTAALEDPQWCARAIAKHGERIAVGLDVRIIDGDYRLRGRGWVSDGGDLWEVLERLERDGCSRYVVTDVTKDGTLTGPNLELLSEVCAATEAPVIASGGVSAIEDLVAIAGLVPEGVEGAIVGKALYAGRFTLPEALAAVR

>CORE_REP|Org5_Gene5214#

MFEPKGAPPIPLGTHGVRLYHRPVRSGTIGRMARLDYQALNSTIRYLMFSVFQVEPGVLGDQRDTVVKQAREFFDSLEERGVVVRGIYDVAGLRADADFMIWWHAEHIEDLQAAYADFRRTTDLGRASTPVWSNAAIHRPAEFNKSHIPAFLAGEEPGNYICVYPFVRSYDWYLLPDEERRRMLADHGKQARGYPDVRANTVASFALGDYEWILAFEAPELHRIVDLMRDLRATDARNHVREEIPFFSGPRVDVEKLVNALP

>CORE_REP|Org58_Gene1395#

MPNIVVLIKQVPDTWSERKLSDGDFTLDREAADAVLDEINERAVEEALLIKEAQGGEVTVLSAGPDRATEAIRKALSMGADKAIHINDPAIHGSDAVQTAWVLASALGQVEGVELVIAGNEATDGRAGAVPAIIAEYLGLPQLTHLRKLTVDGDKITGERETDEGVFKLEATLPAIVSVTEKINEPRFPSFKGIMAAKKKEVQVFTLADLGVDPSTVGVANAGSTVTSSTPKPPRTAGEKVVDEGDGGTKIAQYLVGQKII

>CORE_REP|Org19_Gene685#

MADRSGTGPLPAEPLRIADREFGSRLIMGTGGAENLAVLEEALVASGTELTTVAMRRIDAAGGTGVLDLLKRLDIAPLPNTAGCRTAAEAVLTAQLAREALETDWVKLEVIADERTLLPDAIELVSAAEQLVDDGFVVLPYTTDDPVLARRLEDAGCAAVMPLGSPIGTGLGIGNPHNIEMIVEAAGVPVVLDAGIGTASDAALAMELGCSAVLLATAVTRARHPARMARAMAAAVQAGHLARHAGRIPKRFWAQASSPAV

>CORE_REP|Org139_Gene5112#

MAPPPTTARVGIGSSSCSHTHGSAATVEGVQPSHPNRPLPPRERLASARLYLCTDARREKGDLAKFAEAALAGGVDIIQLRDKGSPGEQKFGPLEAKAELGALAELKAAARRHGALVAVNDRADLALAAGTDVLHLGQGDLPPWYARRILGPDVVIGRSTHNRAQAGLAAIDEHIDYFCTGPVWATPTKPNRQAAGIDLVRSTAEAHPTRPWFAIGGIDTQNLPEVLAAGADRVVVVRAITEARDPEAAARELKAALLANV

>CORE_REP|Org71_Gene936#

MADGRAAGHPDVEGSTRTRCGLRPFGADQTGERVTTAATETTTAVLVVDDQELVRGGLRRILRRRDGFVLTECADGDEVVPAISAEPPDVILMDLRMKRVGGIEATRLVRMRADAPPVLVLTTFDDDQLLSGALRAGAAGFILKDSPAEDLIRAVRTVAAGGAWLDPAVTGRVLSAYRTVRPATPTDARLAELTAREYEVLELIGRGRVNSEIARELGISEVTVKSHVGHIFGKLDLRDRAAAIVFAFDHGVVSPGQSTV

>CORE_REP|Org210_Gene6345#

MICRSGSREVTAGKDEADAPAAGDAADHASLLEALGVEVRPDLLRLALTHRSYAYENGGLPTNERLEFLGDSVLGLSITERLYLEHPEKSEGELAKLRASVVNMHALAEVARGLGAGGLGRHLLLGKGEELTGGRDKASILADGMESLLGAVHLQHGIEVARGVVLRLFAELLERGPRMGAGLDWKTSLQELTAERGIGVPSYEITSTGPDHDKEFTATAVIGGQAYGQGVGRSKKEAEQKAAGAAWQALTADGSAEAGD

>CORE_REP|Org93_Gene2247#

MINAVGNPQAILLLGGTSEIGLAICAEYLKKAPARVILATLPGDPLRADAVAQMEAAGASEVEVIDFDALDTDSHPKVIDAAWARGDVDVAIVAFGLLGEAEELWRNQRKAVQIAEINYTAAVSVGVLVGEKMQAQGYGRIIAMSSAAGERVRRSNFVYGSTKAGLDGFYLGLGEALRPFGPRVLVVRPGQVRTRTTIEHWKATGAKEAPFTVDKEEVAALAVSASDKGKELIWAPGVFRYVMMVLRHIPRPIFRRLPI

>CORE_REP|Org198_Gene6479#

MGGELVELLLLTSDPDPESVLPSLALLAHNVRPAPTEVSSLLEAGTADVALVDARTDLAAARGLCRLLGSTGSSVPVVAVLTEGGLVAVNADWGLDDILLPGTGPAELDARLRLLVGRNGGVASPENTGKITLGELVIDEGTYTARLRGRPLDLTYKEFELLKYLAQHAGRVFTRAQLLQEVWGYDFFGGTRTVDVHVRRLRAKLGSEYESLIGTVRNVGYKAVRPSRSASKGESAPLPEDDAEGTDDAPLAPVNGSVQ

>CORE_REP|Org3_Gene4843#

MSGTGNAGNTGARIDPRGRAVNVGGVAKTHSVRASLDKQPNEVAAMFDEVAQRYDSTNILISGGQDWYWRRETRKAIAPRPGERVLDLAAGTGVSTAEFAKSGAWCVAADFSQGMLAAGRFRNVPMVAGDAMALPFADNSFDAVTISYGLRNVADPDLALREMLRVTKPGGRLVICEFSTPVVPAFRGVYKLYLTKVLPQIAKVASSNPEAYAYLAESIPDWSNQRQLALRIADAGWSGVKWRNLSLGAVALHRAYKLG

>CORE_REP|Org187_Gene133#

MAKRIDVKDLNIYYGKFHAVADVSLTVLPRSVTAFIGPSGCGKSTVLRSLNRMHEVTPNARVEGVVALDGEDIYGENVDPVGVRRTIGMVFQRPNPFPTMSIRDNVVAGLRLQGVRNKSELDEVAERSLRGANLWNEVKDRLDKPGGGLSGGQQQRLCIARAIAVSPDVLLMDEPCSALDPISTLAIEDLITELKKDYTIVIVTHNMQQAARVSDQTGFFNLEAQGKPGKLIEIDDTEKIFSNPGQKATEDYISGRFG

>CORE_REP|Org173_Gene2555#

MAEFVTVDELDGDTERRVAVLRIARAPMNLLNIQVVRELAGAAHAIGEDPRIGAVVVYGDERVFCAGDDLAELAALSAEQAAAVAADLQHALGCLARLPQPTVAAISGYALGSGLELALGADRRVIGDNVKLGLPQIKAGLIPLAGIRRLTALIGPGAAKDLVYTGRFVEPDEARALGLVDEVVAPDDVYTAAVEWARGFLDGPARALAAAKAVFEAGPHGHDRARIAWSELFGTEDRLIGTRSYLADGPGSAEFVGR

>CORE_REP|Org83_Gene1201#

MRLGRVASPDGVAFVSIEGDGGDSVAKEIAEHPFGTPTFTGRSWPLADVRLLAPILASKVICIGKNYAAHAAEMGGEAPADPVIFMKPSTSIIGPNASIILPPSSSQVDYEGELAIVIGRPCKDVPAARALDVILGYTVANDVTARDQQRHDGQWTRAKGYDTFCPLGPWIETQLDPSDLEIVTELDGEVRQRSRTSLLLHDIPKLIEWVTTVMTLLPGDVILTGTPEGVGPMTAGQNVSVTVSGIGTLTNPVAAKR

>CORE_REP|Org41_Gene2931#

MGINRHTESTGITVVTVDYPPVNAIPTDGWFAIADAVRAAGRDPETKVVVLRAENRGFNAGVDIKEIQSKPGHQALIDANHGCFEAFGAVYDCPVPVIAVVQGFCLGGGIGLVGNADVVIASDDATFGLPEVDRGALGAATHLARLVPQHLMRALFYTASTITAQQLHHHGSVYQVVPRAELDAAAMEVAKNIAAKDGRVIRAAKRALNGIDVQDVHRSYRYEQGFTFELNLAGVADEIRARFDDDLAARKAGNQEQ

>CORE_REP|Org117_Gene417#

MTVAVRVIPCLDVDAGRVVKGVNFQNLRDAGDPVELAATYDAQGADELTFLDVTASTGDRGTMIDVVTRTAEQIFIPLTVGGGVRTVEDVDRLLRAGADKVSVNTAAIARPEVLREMSERFGSQCIVLSVDARTVPDGQPDTPSGWEVTTHGGKRGTGIDAVEWAERGAELGVGEILLNSMDADGTKTGFDLPMIRAVRAAVSIPVIASGGAGAVEHFAPAVQAGADAVLAASVFHFGDLTIGQVKDSLRDAHLVVR

>CORE_REP|Org140_Gene718#

MSERSEGTVDAATSTRTAVVTGASSGIGAATARELAKQGYHVYVGARRLDRLQRLAEEIGGTALELDVTSDDSVRAFTDAIDRVDVLVNNAGGAKGLATVAEADLDDWRWMWETNVLGTLRLTKALLPKLIDSGDGLIVTITSVAAFHAYDNGSGYTSAKHAQAVLHRTLRGELLGKPVRLTEIAPGAVETEFSLVRFSGDEERAAKVYEGIDPLVAQDIAEIVAFTASRPPHVNLDQIIVKPRDQADPGRFARRTS

>CORE_REP|Org114_Gene5983#

MLELTDVTKEYRVGEQTVRALDGISLRIEPGEFTAIIGPSGSGKSTLLHMLGALDSPDSGSIRFQDAEIGGLDDDRQSEFRRHRVGFVFQFFNLLPTLSAWENVAIPKLLDGTGLRKAKPRALELLELVGLADRAEHRPAELSGGQMQRVAVARALIMDPPLILADEPTGNLDSKTGASILELLGDITRQGNSVVMVTHDMGAVRYCDRLITLRDGKIGSNELVEHTENGEVRTVPVELTASLSEDGSEPAQAVRP

>CORE_REP|Org218_Gene4719#

MRSVVRHLFLAVAIMALAMVGSGAANTMMIDRSPPGADIGTVASITPSAGRTVGIAHPVTIRFDADVADRAAAERAVSITADRPLPGTFAWAGDRQLTWSPTGYLPANATIGVRFGDQVRTEFATNAGVVADADMSAHTFTVSIGGQVVRVMPASMGKPGWETPTGTFPVLEKFRHIVFDSRTIGIPLDSPEGYLIDGEYGVRLTWSGVFVHSAPWSVDSQGYANVSHGCINLAPGDAAWYYENVGIGDPVTTHW

>CORE_REP|Org148_Gene2575#

MGWRSGRELDAGTVLVLGGRSEIGLEVARRIAPGRTVVLAARRSDELAEQVAAVEAAGAAAVHCVEFDADDTASHPALLEKIAAEHGPLAVVVVAFGILGDQARAERDPAHALAVVHTDFTAQVSVLTVLANMLRAQGSGQLVVFSSIAGVRVRRANYVYGSAKAGLDGFASGLADALHGTGVHLLLVRSGFVIGRMTEGMDPAPFSSTPGQVAEAVVKGLRRRAGQIAVPGVLRLVFIAFRMLPQPIWRRMPR

>CORE_REP|Org102_Gene2417#

MSGPILREVPVASPVHRLWAGTKMIAAFAVSVLLMFIPSWPVLGICVAFLAAVWITARLPLGTLPRFPWWFWAGLLLGALINLPIGSAAVLRYLNVAVFGLILLSASFLIAWTTPMGEIAPALAKLGAPLRRIGWTRRGVRHAVPVDEWAVVVALTLRGLPLLLEEMRILRASRKLRPSDGMLQRAANPLVDILTAALAVSTRRAGELGEAITARGGTGELTARPSTPGRRDIYALAIVGVVCAGAILVDVLV

>CORE_REP|Org1_Gene619#

MGPRAGPGEQGGRPLRVTVVVPTYNERENLPVAVARLTALPVSDLHILVVDDNSPDGTGEVADKLAADLPDVVGVLHRTEKDGLGRAYVAGITRALDEGADVVIQMDADLSHPAEVIPAMLDKLRDTDAGVVLGSRYVPGGSTAEEWKWYRKALSAWANFYVNLILRLGVKDATAGFKAWKAETLRAIDVASIRSNGYSFQVEMNYRTVKKGITIAEVPIRFEERTKGASKMSLKVQLESALMPWKLLFGRAV

>CORE_REP|Org195_Gene1079#

MSARGHAFGIDIGGSGVKGAVVDLATGELVHERIKIATPHPATPFAVAEAVAELVAQAGWSGPVGLTLPAVVIGGVARTAANIDKSWIGTDARALFSAALGGRSVTVLNDADAAGMAEDRYGAARNFTGLVILLTFGTGIGSALLYHGTLVPNTELGHLEIQGMESEHRAAASIKERDGLSYEQWAAQVSLVLTTLENLFFPIVFVAGGGISRDAEQWIPLLTNQTPVIPARLRNTAGIVGAAMAIAGGIAP

>CORE_REP|Org35_Gene4389#

MSNSVEDSRKSESQNAERASRSVLVTGGNRGIGLAVAQRLLADGHKVAVTHRGSGVPDGLFGVKCDVTDSESVDRAFSEVEAHQGPVEVLVANAGITDDTLLMRMTEEQFTRVIDANLTGAFRCAKRANRAMLRARWGRMIFLGSVVGLGGGPGQINYASSKAGVIGLARSVTRELGSRNITANVVAPGFIETDMTAELPEEMRETAKKFIPLQRLGAPEEVAAVISFLASEDSRYVSGAVIPVDGGMGMGH

>CORE_REP|Org2_Gene6607#

MNSLTPAVSLLTSNNDGVNTTSSSVPAASVLVAEDDPHVRSTLDQLLRFEGYQVYLAADGQEALELLAQQRPDLAVVDVEMPRLDGLSLCRLLRRRGDRLPILVLTARQQIGDRVAGLDAGADDYLPKPFATDELLARLRALLRRSTFDEDDDTVLAVGDLTLNTATRQVHRGDRPIELTKTEFDVLELLLRNARIVLSRSRIYEHIWGFDFDTESRSLDVYIGYLRRKTEENGEPRLIHTVRNVGYSVRPA

>CORE_REP|Org13_Gene1770#

MLDDRIRGGRDVVESGIGDDGIPGAELVVAMDGPSGTGKSSVSRRLAARLGARYLDTGAMYRVATLRVLRSGVELTDPAEIAAAVKDLPLTIGTDPGHELIELDGEDVSAEIRGDAVTKAVSAVSAVAEVRTQLVALQREIAAAAGRIVVEGRDIGTVVLPDADAKIYLSASAQARAARRNQQNIAEGRGDDYDAVLADVQRRDNLDSTRAVSPLRPAADAVQVDTSELSMDQVIDELYRVVAQQITVGERR

>CORE_REP|Org45_Gene4290#

MSGHSKWATTKHKKAALDAKRGKLFAKLIKNIEVAARTGGGDPDGNPTLYDAIQKARKNSVPLDNIERARKRGGGEEAGGADWQTIMYEGYGPSGVAVLVECLTDNRNRAAGEVRVAVTRNGGNMADPGSVSYLFSRKGVVTLEKNGLSEDDVLMAVLDAGAEEVNDLGEEFEIISEPSDLVAVRSALQGAGIDYNSAESGFQPSVSVPADADLAKKVFKLVDALEDCDDVQNVYTNIDVSDEVLAQLDAE

>CORE_REP|Org113_Gene6275#

MTSMWGAPLRSRWRGSRRRDPEQARFLTVASLRWVLANRAYTPWYLVRYYRLFKFRMANPHIVLRGMVFLGRRVEIHATPELGRMEIGKWVHIGDGNALRCHEGSLRIGDKVVFGKDNVVNTYLDIEIGESTLVADWCYICDFDHRMDDVNMPIKDQGIVKSPVRIGPDTWIAAKVTVLRETAVGRGCVLGAHAVVKGDIPDYSIAVGAPARVVKNRKKAWDDAAEERARHAAALADIARKKAGVGAGSDD

>CORE_REP|Org166_Gene5029#

MTVGYDAKFRVWRGDETGGALQDFTVEVNEGEVVLDIIHRLQATQAPDLAVRWNCKAGKCGSCSAEVNGRPRLLCMTRMSTFTRDELVTVTPMRTFPVVRDLVTDVSFNYEKAREIPSFTPPPELKPGDYRMKQVDVERSQEFRKCIECFLCQNTCHVVRDHEENKQAFAGPRFLMRIAELEMHPLDVADRRQLAQDDQGLGYCNITKCCTEVCPENIKITDNALIPLKERVADRKYDPLVWLGNKLFRR

>CORE_REP|Org1_Gene4194#

MSMYRVFEALDELVAIVEEARGIPPTRSCIVPRGDVLDLLDDVRDALPGELDDAQDVLDHRDKIVSDARTAAETTVTSADEQARDTIDSAREEADRILADAKAHADRMVAEASAHADHLVTTAQAEAERIVAEAKAEYETVTGRARAEADRMIESGKASYERSVAEGEAEQARLVAQTEVVRAAHAESARIIDTAQAEADRMRDECDHYVDSTLAQFEETLNSTLRTVGRGRHQLRSGAGAPDYATDYRR

>CORE_REP|Org5_Gene560#

MSRMNGVAGDRIPEARVLVVDDEPMIVELLSVSLRYQGFEVAAAGNGAEGLDRAKQFRPDALIVDVMMPGMDGFGLLRRLRADGIDAPVLFLTARDEVDDKITGLTLGADDYVTKPFSLEEVVARLRVILRRSGHVVEETKSSRIRFEDIELDDDTHEVWKAGEPVALSPTEFTLLRYFMVNAGTVLSKPRILDHVWRYDFGGEVGVVETYVSYLRKKVDTGPDRLIHTLRGVGYVMRAPSRSRSSAK

>CORE_REP|Org23_Gene555#

MTYTLVLLRHGESEWNALNLFTGWVDVHLTDKGIAEGKRAGELLAEHGILPDIVYTSLLRRAISTANIALDAADRHWIPVVRDWRLNERHYGELQGKNKAQIRDKYGDEQFMLWRRSYDTPPPPIEADNEYSQTGDPRYAGIEVPRTECLLDVVNRMVPYWESTISKDLLSGKTVLVAAHGNSLRALVKHLDQISDDDIAGLNIPTGIPLRYELDENLRPVRPREYLDPEAAAAGAAAVAGQGGGA

>CORE_REP|Org63_Gene1785#

MKLDVVTIFPEYLEPLRTALLGKAIDKGLISLTVHDLRRWTHDVHKSVDDAPYGGGPGMVMKPTVWGDALDEVCPDDALLVVPTPAGVPFTQDTAHRWAAEEHLVFACGRYEGIDQRVFDDAARRVRVEEVSIGDYVLIGGEAAVLVMTEAVVRLLPGVLGNQQSHQEDSFSDGLLEGPSYTRPVSWRGLDVPPILLSGDHAKVAAWRREQALARTRERRPDLLVHRNLVDRNLVDRDGEAGPQH

>CORE_REP|Org127_Gene78#

MTDPDTAPDRKGYRRVLLKLGGEMFGGGEVGLDPDVVQTVAEQIAEVVSTGVQVAVVIGGGNFFRGAELEERGMERARSDYMGMLGTVMNSLALQDFLQKQGVDTRVQTAITMGQVAEPYLPLRAKRHLEKGRVVIFGAGMGMPYFSTDTTAAQRALEIGAEVVLMAKAVDGVFTADPKVEAEAIMFSEITHKEVLERGLKVADATAFSLCMDNQMPMLVFNLLIKGNIARAVAGEKIGTLVRS

>CORE_REP|Org31_Gene655#

MVPLPDAYRTSELVSTPKVLVVDDDEDVLASVERGLRLSGFHVLVARDGAQALRSVSEHAPDAIVLDMNMPVLDGAGVVTALRAMGNEVPICVLSARASVDERISGLESGADDYLVKPFVLAELVARIRALLRRRTDTPPAATPGAITVGPLEVDIAGYRAVLHGNEIELTKREFELLSTLARNVGVVLSRERLLELVWGYDFAADTNVVDVFVGYLRRKLEVDGAPRLLHTIRGVGFVLRAPK

>CORE_REP|Org195_Gene709#

MFYWLLKYVLLGPFIHLYNRPTVEGVENIPADGPAIMAGNHLSFADWLFAPLLSPRRINYLAKAEYFTTPGIKGRFQKFFFSASGQYPIDRSGADAAEDALNAARKLLDQGRLVGLYPEGTRSPDGRLYKGKTGLARLALETGVPVIPVAVIGTDEVSPPGPFRWRRRKVTVKFGEPIDFSRYEGMGGNRFVERAVTDEVMYELMQLTGQEYVDVYAHSLKKDVPSGSRPEAVSVRIPDTAAG

>CORE_REP|Org51_Gene5136#

MNTLLVTGTSTGVGKTVVTAALTALARAEGLAVAVCKPAQTGVAPGEPGDLAEVRRLAGPVPTLELARYPEPLAPDTAARRCGAPLLTLDETATAVRGLDAELTVVEGAGGLLVRVGEFTLLDLARELDAPVLVVAAAGLGTLNHTELTIRALDAAGVRCAGVVIGAWPAEPDLASLCNREDLPRLTGVPIVGAVPAGVGAWDHDRFTAAVPGWFAPGWSPRLPFNSDSPPSTWGFDTSQSGT

>CORE_REP|Org112_Gene4447#

MTIKLAESELRAIAEKGAAELGPDATALELLRWTEDTFGSDYIVASNMQDAVLVQLAAQVRADGGAVDRGPVDVLFLDTGYHFAETIGTRDAVEMVYGVNIVNVTPEHTVAEQDQLLGKDLFARDAGECCRLRKVVPLRGSLSGYNAWVTGIRRVEAPTRANAPLISFDEAFGLVKINPIAAWSDEEMAAYIEANGILVNPLVEEGYPSIGCAPCTRKPEPGSDPRSGRWAGLAKTECGLHAS

>CORE_REP|Org114_Gene4108#

MSANLMIVEDDDRVRVALRLAMEDEGYDVAEAEEAEVALRQLRDNGAPDFMIVDLMLGGMDGFTCIREIRRDHDVPIIVVSARDDTHDVVAALEAGADDFVTKPFEVKEITARMRAVARRARFAEQAAAEEDPDSELGTMVLDEQAGNPLVLSTESGIVRRGDEEIHLTLTEYRLLCELAGSAGRVLSRGTLLERVWDRGFFGDERIVDVHIRRLRTKIERDASDPQLIVTVRGLGYRLDVQR

>CORE_REP|Org152_Gene6409#

MTEQRREQQAPRVNAGRPSVPGSHRGIRALRSGGPLRRTAPRRVLSVLLASVCLVAALAGCSTAGKDAVAQGGTFDFVSPGGETDIFYDPPSSRGTIGTLSGPDLMTEGKTTAVSDFPDQVVVLNIWGQWCGPCRAEAPALEQVYEATRDSGVAFLGINVRDNQKDKARDFVTDYNVGYPSIYDPSMRTLLALGGKFPTSVIPTTLILDRQHRVAAVFLRSLLAEDLQPVVERIAAEGRS

>CORE_REP|Org5_Gene1107#

MPLRNGSLSECGNLPRVPMSNPDRPYRSLMRTHTVDHPLVATLLTTMRDARTSNAGFRAALADLTGFLVYEALREVPIRTFDIDTPVATTRGARLAEPPLLVPVLRAGLGMVDAASALIPESRVGFVGMARDEHTHRPVPYMESLPADLSGLPVFVLDPMLATGGSMCHTLRLLVDRGATDITAVCVVAAPEGIAALENSGFPVRLVTAAVDDRLNDDAFIVPGLGDAGDRQFGPRLD

>CORE_REP|Org48_Gene2317#

MAKRSKAYLAAAEKVDRTKLYSPLAAARLAKETATTKTDATVEVAVRLGVDPRKADQMVRGTVNLPHGTGKTARVIVFAAGEKAAEAEAAGADAVGAEDLIERIQGGWLDFDAAIATPDQMAKVGRIARVLGPRGLMPNPKTGTVTTDVAKAVSDIKGGKINFRVDKQANLHFVIGKASFDEAKLVENYGAALDEILRAKPSTAKGRYVKKVTVSTNTGPGIPVDPNRTRNLLDEDA

>CORE_REP|Org12_Gene5846#

MSTDTVKSEKATNLTLPVKEPGGKTNGTVELPAEIFDATANIALMHQVVVAQQAAARQGTHATKTRGDVRGGGKKPYRQKGTGRARQGSTRAPQFTGGGTVHGPQPRDYSQRLPKKMKAAALRGALSDRARNERIHVISELVAGQTPSTKAAKNFLAELSDRKKVLVVVGREDVTAWKSVANLQGVHPIAPDQLNTYDVLLSDDVVFSVEALNAFVHGPTESAQESQPGSAAQEESK

>CORE_REP|Org38_Gene681#

MDRRSRALSSPTPPPSPAADPVASPTARWTPTATPATVVVLASGTGSLLRALLDAATAADFPARIVAVGVDRPCPATAHAEAAGVPHFRVALNDFPDRAAWDAALTDAVAGYAPDLVVSAGFMKILGARFLGRFGGRIINTHPALLPAFAGAHGVRDALAYGVRVTGSTVHLVDAGVDTGPILAQEAVPVLPDDDEASLHERIKVVERRLLAQVVAAAATRGIVSDGRKAVIPDERV

>CORE_REP|Org37_Gene4737#

MARRDRFGYATGVSNRPAGGSGPDHQASEPPEPARESADAAVGTETDRILTVPNVLSVLRLIGVPVFLWLLLVLHADGWAFALLVASGVTDYLDGKLARLLDQYSRLGALLDPLVDRLYLIATVLGLLVRGILPWPFVVVLIGRDLILSATLPIYRRRNLDPPEVIYLGKAATFALMSALPWLLAGQMDWAAAGFGRAFGWALLWWGTAVYVWTGLLYLRKAFAVARTIPVAPRATS

>CORE_REP|Org142_Gene4260#

MSHGPDVSSTEYATAAPAAPLPFSAQIRSATARQHEEAENSSFISDMLGGELGVESYLRYTGQLWFVYRALEGRWDSLAEDPIAGPFIRPELARTAELERDLAHLAGPGWRDGLEPLPATAAYAARIDECARDWPGGYIAHHYTRYLGDLSGGQVIRGTAEKLWNLPHRGDGVRFYVFDAVGNPAAFKREYRALLDRMPLDDLERRRVLDEGQRAFAMNTAVFEELAEEFPTRRPS

>CORE_REP|Org102_Gene4053#

MHDEGVKQVKDLVDTTEMYLRTIYDLEEEGVTPLRARIAERLEQSGPTVSQTVARMERDGLLTVAGDRHLELTEKGRAMAVAVMRKHRLAERLLVDIIGLDWQNVHAEACRWEHVMSEEVERRLVEVLNHPTTSPYGNPIPGLDELGVTGTSNAEEKLVRLSDLPSGQSAAVVVRRLSEHIQTDPEIINQLREAGVVPDARVNVETKPGAVVILVPGHAGFELSDEMAHAVQVKLV

>CORE_REP|Org102_Gene3540#

MNSRAVCYPDMGAKPLSEVVDSGWAEALEPVADRIAAMGEFLRAELAAGRGYLPKGENVLRAFQRPFDKVRVLIVGQDPYPTPGHPMGLSFSVAPDVSPVPRSLANIFSEYSKDLGHPTPSCGDLSPWSDQGVLMLNRVLTVTPGQPASHRGKGWEAVTEQAIRALVARDQPLVAILWGRDAATLKPMLGSTPYIESAHPSPLSASRGFFGSRPFSRTNELLGTLGAAPVDWRLP

>CORE_REP|Org103_Gene3922#

MVERARSRDRMVAVSTVYDCADPDSRAAGLTAATSALKSGRLVVMPTDTLYGLAADAFDSTAVAELLAAKRRGRDMPVPVLVGSWNTIDGLVFSVRPQARDLIRAFWPGGLSLVVQQAPSLAWDLGDTRGTVMLRMPLHPVALDLLRDVGPLAVSSANVSGQPPATTAAEAREQLGSLVSVYLDGGPAAHAVASTIVDLTADRPRILREGAVSAAEIAEVLGMSPDELTGTAAR

>CORE_REP|Org206_Gene4230#

MTGVVSQAGTSNVALVSDKLVVWMDCEMTGLRLDSDKLIEVSALVTDSDLNILGDGVDIVIHADDAALAAMPPVVAEMHARSGLTDEVRRSTVTVAEAEQQVLDYIRQYVPTPRTVPLAGNSIATDRAFIARDMPALDAHLHYRMIDVSSIKELCRRWYPRIYFGQPEKGLTHRALADIKESIRELEYYRRTAFVAPPGPSTAEIAAVAAQLGGTAQTAESPQVEGTTQAD

>CORE_REP|Org121_Gene2168#

MLSFFGRDTFAKATAPLGKALVSTGLTPDAVTVIGTTASILAAVTLFPTGHLFWGTMVIWFFVMFDMLDGAMARARGGGTKYGAVLDATCDRVADGAIFGGLAWWAVYHEHHQPLFAATLVVLVTSQVISYAKARAEASGLSADGGIIERPDRLIIVLVGAGFTGIGGYWGLDWLTYAVHVAMWILAVLSIVTVFQRVLAVRRSPGARDVITPARPENPTAGTANATELPS

>CORE_REP|Org5_Gene6261#

MVWRTARFGPCNLHAMAALIAVEGLDGAGKRTLIDAVVATLRDRGVRVGTLAFPRYGRSVHADLAAEALRGRHGDVAGSVNAMALLFALDRADARDELSKLLADNDIVLLDRYVASNAAYSAARMDEDADGEMVSWVAELEFARFRLPVPDAQVLLDVPAELAAERARRRGELDTARALDAYERDISLQERTAEVYRVLAASHWHGPWWVHAPQDDPARLTARLSELIGR

>CORE_REP|Org44_Gene3372#

MTSVLIVEDEESLADPLAFLLRKEGFEVTVVGDGPSALAEFDRSGADIVLLDLMLPGMSGTDVCKQLRTRSGVPVIMVTARDSEIDKVVGLELGADDYVTKPYSARELIARIRAVLRRGAGDELDGNGESGVLEAGPVRMDVDRHTVMVNGKPVTLPLKEFDLLEYLLRNSGRVLTRGQLIDRVWGADYVGDTKTLDVHVKRLRSKIEADPAKPEHLVTVRGLGYKLEA

>CORE_REP|Org96_Gene3994#

MITMRNVTKSYKTSTRPALDNITVDVDKGEFVFIIGPSGSGKSTFMRLLLKEESPTAGEIRVADFRVDRLPGRKVPKLRQRMGCVFQDFRLLQQKTVQENVAFALEVIGKRRQVIERTVPEVLDMVGLGGKADRLPSELSGGEQQRVAIARAFVNRPLVLLADEPTGNLDPDTSGEIMLLLERINRTGTTVLMATHDNHIVDAMRRRVVELDHGRLVRDEATGVYGVGR

>CORE_REP|Org125_Gene428#

MTEKKQGPATDAKTRPAAGILGTKLGMTQVFDEKNRVVPVTVVKAGPNVVTQIRTEERDGYSAVQLAFGAIDPRKVTKPVAGQFAKAGVTPRRHVAEIRVADAAAFEVGQELSADVFEEGTYVDVTGTSKGKGYAGVMKRHGFRGQGAAHGAQAVHRRPGSIGGCSTPGRVFKGMRMAGRMGNDRVTTQNLSVHKVDAENGLLLIKGAIPGRKGGIVIVKSAAKGGARA

>CORE_REP|Org82_Gene1831#

MPDGGAAVIPEQRVLHVPDPAERENLATFLTRAVRLDPAAVVRLRRRGENYVSAWVATGFEALAMRTVVAELGVDDVTVGADVVLAGSASGSPIDLGYSMDSAWRGALPPTDGFSHVEDVPARTLVELAEQGAQLAKEHGSGHGPPASLLDQAVLTVSGAGTRVQVPMRVVFALTAMDFIPHSGDKADPRRIPPAEIVRVRATRTWLRLDARYGSVARRIGGGIPLLPQ

>CORE_REP|Org102_Gene5430#

MNTTSRGDSVDGGGRVTNGRTKRLALLDYGSGNLHSAERALVRAGAEVTVTADPDIALNADGLVVPGVGAFAACMAGLQEVRGERIIGKRLAGGRPVLGICVGMQILFERGVEHGIETAGCAEWPGTVERLDAPVLPHMGWNTVRAPEDSVLFAGMDADTRFYFVHSYAAQKWEWNGDGTIAPAKLTWAEHGVPFLAAVENGPLSATQFHPEKSGDAGAQLLRNWVRSL

>CORE_REP|Org17_Gene4534#

MFDLISRYDNQLWDAFWVTIKLTVFSAVGALIIGTLVAAMRVSPIPVARWIGTAYVTVFRNTPLTLILVFCSLGLYTTLGIKLASESSGLDFLPTNNFRFAVLGLSVYTASFVCESLRAGINTVPLGQSEAGRSLGLTFGQNLRHVVLPQAFRSVIAPLGSVLIALTKNSTIASAIGVAEAAALMASMNETEADILAIGAIFALGFVILTLPTGLLFGWLAKRFEVKR

>CORE_REP|Org3_Gene3830#

MMPAGSGSASRSRTGSHPAPDAGFVLDVRNLGRGAGSMRELHRTVTTTERLGLDLIAVPAGAEVELDLRLQAVSEGVLITGTVSAPLTGECSRCLEPFDDEIELSLTELFAYPDSATEQTTSEDEVYRMADDLIDLEPVIVDALGVELPLQPLCTPDCAGLCPECGVRMAIAGSDHGHEILDPRWAGLAKFAADSPSTGSPDAGAATPDRSAEQADTADRADTQTEEK

>CORE_REP|Org10_Gene2540#

MSANHNAVDQVPNGLDRAAFDDDAQPAVAEPPLRALATGRPFDQPRAEAAIRELLIAVGEDPDRPGLIDTPARVARAYREMFAGLYVEPDRVLDTTFDEGHQELVLVRDIPMYSTCEHHLVSFHGVAHVGYIPGPAGRVTGLSKLARLVDLYAKRPQVQERLTSQIADAVMRKLEPRGAIVVVEAEHLCMAMRGIRKPGASTTTSAVRGLLQTNAASRAEALDLILRK

>CORE_REP|Org2_Gene5611#

MSTPTYARPAVPMIAPSILSADFAHLADEARAVEGADWLHVDVMDAHFVPNLTLGLPVVESLLKATDIPLDCHLMIEDPGRWAPPYAEAGAYNVTFHAEATDDPIAVARDIRAAGAKAGLSVKPNTPIEPYLEILREFDTLLVMSVEPGFGGQSFIADVLDKARTVRRLVDAGELRLLVEIDGGINANTIEAAAEAGIDCFVAGSAVYNTADPAATVQALRKQAAAHR

>CORE_REP|Org2_Gene5880#

MLVVSLITEGRPVDSQGRPFLRRRPQPWLIMLGVLLLICTIVWVKALTTTERDTSAMACNSPSPATDPGAGQPVALGQRVGNGRLQDVEPAPLAAAKVRVLNANNQRGQAAHVAAQFGDLGFASAPGTQFGNDSVYVNGDLECTGQIRFGVNGRPAAASVQLVVPCAELIEDQRSDDTVDMVLGSLFRDIRPSNDAEEVLRSLKNPAPGGPPQIDIKLLDAARQARC

>CORE_REP|Org134_Gene5164#

MVESAREVREPAARQGRDIVAEYTLPDLDYDYSALEPHISGQINEIHHSKHHAAYVAGVNTALEKLEAAREAGDHGAIFLNEKNLAFHLGGHVNHSIWWKNLSPNGGDKPVGELAAAIDDQFGSFDKFRAQFTAAANGLQGSGWAVLGYDTLGQKLLTFQLYDQQANVPLGIIPLLQVDMWEHAFYLQYKNVKADYVTAFWNVVNWADVQERFARAVSEGKGLIFG

>CORE_REP|Org45_Gene5167#

MSNMFDPRAAGITFAAAGSSLPQARYILPSYTEQTSFGVKETNPYTKLFEERIIFLGAPIDDTSANDIMAQLLVLESLDPDRDITMYINSPGGSPVSLMAIYDTMQYVRADVATVCLGQAASAAAVLLAAGAPGKRAALPNARVLIHQPYTQGGFQGQVSDLEIQAAEIERTRSLLDTILARHTGKEAEIIRRDTDRDKILTADEAKDYGIVDTVFEYRKLSAQR

>CORE_REP|Org7_Gene3709#

MTAVLLAEDDEAIAAPLSRALGREGYSVTVERFGPAVLERALEGHHDLLILDLGLPGMDGLEVCRQVRASGADIAVLMLTARTDEVDFVVGLDAGADDYVGKPFRLAELLARVRALLRRSGIGDDTVEVGGIRLEPAARRVLVNGAEIGLANKEYELLKVLIDRAGQVVPRETILREVWGDAELRGSKTLDMHMSWLRRKIGDEGPMAERRIVTVRGVGFRLNTD

>CORE_REP|Org19_Gene1545#

MSETVPTAYTRAQVAAMIDHTLLAPEATAADVANTIAEARELGVYAVCLSPSMLPVTAPGLVVATVAGFPSGKHHSLVKGTEARFAVEQGAAEVDMVIDIGAAVAGDYNAVLADIVTVREAVEDRALLKVIIESAALSDAAIVEVCRAAERAGADFVKTSTGFHPAGGAEVRAVRLMRETVGGRLGIKASGGIRTAAAAAELIAAGATRLGLSRSADVLAGFPE

>CORE_REP|Org216_Gene4990#

MYGSLSTHVLRCDGAPVPIALAALPTTQTAAIPDPAVVDELLPVLAADSLPRLIVLGEDAALAAVLTHLMKTERLHVEIGYVPIDRTYGARAYEIGSGNSAAKRAIEGKATATPLIRDDTGKVLVGRATITGPEGAKLDGEAYVDDTLLFTGRVTAMQISPTLHMPGVRAAAQRGVLRKRRWVEGRAAQLGTPGAVVTRDGIRTTRPVPRSTFYRHHEPWLLVR

>CORE_REP|Org1_Gene1379#

MRRPLSSALMHETGSTGRKLGVMGGTFDPIHHGHLVAASEVANRFDLDEVIFVPTGQPWQKAHKKVSPAEDRYLMTVIATASNPSFTVSRADIDRGKVTYTVDTLREMKAQYPDAQLYFITGADALANILSWQDWAELFELAKFVGVSRPGYELNTDHLEEHLRDLPPDAVTMLEIPALAISSSECRRRAAENRPVWYLVPDGVVQYISKRQLYVPDAAEGS

>CORE_REP|Org38_Gene1267#

MPGRQRRDGGNGPAGQNGAAGGGDNRDNRGGGRDRRGGGDNRRDNAAEKNQLERVVAINRVSKVVKGGRRFSFTALVIVGDGNGLVGVGYGKAKEVPAAIQKGVEEARKGFFRVPMIGSTITHPVQGEAAAGVVMLRPASPGTGVIAGGAARAVLECAGIHDILAKSLGSDNAINVVHATVAALKMLQRPEEVAARRGLPLEDVAPAGMLRARAQAAGGAR

>CORE_REP|Org101_Gene6186#

MPNPLPAAMRWTGRLDVWHTISLSVTGPTTVHPMNSRGPLGGSTSDAHPGVPVSTQSSNLVVRRARTSDVPEIKRLVDVYAGRILLEKNLVTLYEAVQEFWVAVLDGRVVGCGALHVLWADLGEVRTVAVHPDVKGSGVGRLVVEQLIAVARELELRRVFVLTFEVDFFARHGFVEIDGTPVTAEVYAEMCRSYDTGVAEFLDLSYVKPNTLGNTRMLLTL

>CORE_REP|Org4_Gene7730#

MAVGQRAGMRRQVSVTSNQAGIAMTSATAGLNLSDSVYERLLRERIIFLGTQVDDDIANKICAQILLLTAEDPTKDISLYINSPGGSVTAGMAIYDTMQFAECDIRTVGMGLAASMGQFLLTAGTKGKRLALPHARIMMHQPSAGIGGSAADIAIMAEQFAHTKRELNELQAQHTGKSVEQVTADADRDRWFTAKEALEYGFIDRVVSHANQTGGVSDN

>CORE_REP|Org4_Gene7848#

MILLGSRVAADRDSPGCPDGPRGMIRDPDLGRTCAAAYTRPVACGSYARNPAVNPQWSGRKIHRNRWQARVRRVTDHARRVGNPACVYIQVKEAEVIQQESRLRVADNTGAKEILCIRVLGGSSRRYAGIGDIIVATVKDAIPGGNVKRGDVVKAVVVRTVKERRRPDGSYIKFDENAAVLIKADNDPRGTRIFGPVGRELRDKKFMKIVSLAPEVL

>CORE_REP|Org217_Gene444#

MTDTTLRHRTARVERVTKESSIVVELDLDGTGRTDISTGVPFYDHMLTALGAHASFDLSVRAEGDIQIEAHHTVEDTAIVFGQALGKALGDKAGIRRFGDAFIPMDETLAHAAVDVSGRPYCVHTGEPEHLLHAVIPGSPVRGTGEPGAPYSTVLNRHVFESIALNARIALHVRVLYGRDQHHVTEAEFKAVARALRAAVEFDPRVSGVPSTKGTL

>CORE_REP|Org6_Gene3761#

MMLVCTMVDMPRSNPISAWKSLREGNERFVSGNLLHPSQGAADRAKLVAGQHPSAILFGCGDSRVAAELIFDQGLGDMFVVRTAGHVIDSSVLGSIEYGVAVLDVPLIVVLGHDSCGAVKATIDALDGGEVPGGFIRSVVERVAPSILVGRREGLSSVDEMEARHVVETSKLLMQRSMIISEKVESGQLAIACVTYKLAEGKVQLERVVGNIGEVV

>CORE_REP|Org107_Gene36#

MRVLVVDNYDSFVFNLVQYLGQLGAEAVVWRNDDPNLADVDAVVAQFDGILISPGPGTPDRAGASIELVRACARHAVPLLGVCLGHQAIGAAFGATVTRAPELLHGKTSSVFHVGAGVLAGLPDPFTATRYHSLTVLEETLPAEIEVLGRTESGIVMAMRHRTLPIQGVQFHPESVLTQGGHRMLANWLGICGERPAEGLVEMLEAEVAALVLQ

>CORE_REP|Org4_Gene5090#

MMRMSDVRASYLTDGAEIYRRSFAMIRDEADLSGFPPDVAQVVVRMIHGCGQVDLAADVAFSPAVVAAARTALRAGAPILCDANMVASGVTRKRLPADNEVICTLTDPRVPELAAAMGNTRSAAALELWRDRLDGAVVAIGNAPTALFHLLDLLDAGAPRPAAVLGIPVGFIGAAESKDALAAFGGVEFLTVRGRRGGSAITASALNAIASEQE

>CORE_REP|Org13_Gene5664#

MVEEAVTQQDPGRIVDTGAGDPDRLQRLWTPYRMSYITGAVAADRDTRSAPAEADAPAGAEEKTSAGLKHPFTEIPKMSDEDGLVIARGEWVFAVLNLYPYNPGHMMVVPYRRVADLEDLTPEESAELMAFTQRAIRVMKKVSRPEGFNVGLNLGGVAGGSLADHLHQHIVPRWGGDANFITVVGGVKVMPQLLRETRALLAGAWHEAKEVE

>CORE_REP|Org8_Gene2951#

MSLAEPTRIEVAAVASVAEPRRLSVACSSSAWGLLLAASAGWLASFTLTVERFKLFVDSDYRPSCSINPILSCGSVMATPQAAVLGFPNPIIGVVAFSVVIPLALLSVARIALPQWIWIGLWVGTACGVGFVCWLIFQTLYRIHALCPYCMVVWAVVTPMLAVLSNQLSEALPRPIRAIVDWRWTVVALFCTAVVLQVFLAFQDYWLSLI

>CORE_REP|Org210_Gene195#

MGTNAGGAGAKQDAPAAAKRVVVAEDEALIRMDLVEMLSEEGYQVVGEAGDGQQAVDLAVELRPDLVIMDVKMPRRDGIDAAGEIASKRVAPVVILTAFSQRDLVERARDAGAMAYLVKPFTKSDLVPAIELAASRFHEISALEQEVANLSDRLETRKLVERAKGVLMQTQGLSEPQAFKWIQRTAMDRRTTMKAVAEVVLENLAPKS

>CORE_REP|Org39_Gene4005#

MFTGIVEELGEIVAVEERGDASRITVRGPVVTSDAGHGDSIAVNGVCLTVVDKVVEGDTFTVDVMGETLNRSSIGGLTVGSRVNLERAAAVDSRLGGHIVQGHVDGTGTVVSRDPQENWEVVRISLPNSLARYVVEKGSITVDGISLTVSALGVAAAPAADGNTDWFEVSLIPTTRELTTLGTAPVGTTVNLEVDIIAKYVERLVARG

>CORE_REP|Org105_Gene4460#

MMETMTHSDDASDPVVVIGPEGRPLLLPSGKDSEAAYTAQVVSNGDEKSNDSDERDDSGESLADMVEQPAKVMRIGTMIKQLLEEVRAAPLDDASRARLREIHKSSIRELEQGLAPELREELERLTLPFTDESVPSDAELRIAQAQLVGWLEGLFHGIQTALFAQQMAARAQLEQMRQGALPPGVHASDQRAAQHGHTMGGTGQYL

>CORE_REP|Org1_Gene1099#

MTMARVLVASRNAKKLAELRRILDDAGVAGVQIVGLDDVPPYDEAPETGATFEENALAKARDGAAATGLPCVADDSGLAVDALNGMPGVLSARWSGTHGDDAANNALLLAQLRDVPDERRGARFVSACALVVPGGTETVVRGEWPGTIGRKPMGEGGFGYDPLFVPDGGDVTAAQLTPAAKDAASHRGRALRHLLPALAALADRTE

>CORE_REP|Org112_Gene424#

MTTAVGTPGSAITQRVHSLNRPNMVSVGTIIWLSSELMFFAGLFAMYFVARAQANPELGWPTEPTELNMALAVPVTAVLVASSFTCQMGVFAAEKGDVFGLRRWYFITLLMGTFFVAGQGYEYYHLVHEGTSISSSVYGSVFYITTGFHGLHVIGGLIAFVFLLVRTTLSKFTPAQATAAIVVSYYWHFVDIVWIGLFATIYFVR

>CORE_REP|Org39_Gene3985#

MVEHTRKGRLVVLVGPSAVGKSTVVRCVRERLPELVFSVSATTRAPRPGEVDGRDYRFVTREQFDAMVAADELLEWADIHGGLQRSGTPAAPVREALAAGRSVLLEVDLEGARSVRRAMPEALLVFLAPPSWDELVSRLTSRGTESPEVIERRLQTAKTELAACDEFDIVIVNDEVTAACERLVSLFVSTNSRSASKHGSSAP

>CORE_REP|Org5_Gene3436#

MSNLVSVSSVHHETAITTKRPEETGEGLSSGWGWLPYDGRMTTVLLNTSAGDITLELDEEKAPKTVANFVDYVKAGHYSGTIFHRVIPGFMVQGGGLTADMQQKPAPNKVENEAKNGLRNDKYTVAMARTSDPHSASAQFFINTSNNDFLNYPGQDGWGYTVFGRVVNGSEVVDAIEGVSTGSQSGHQDVPKDTITIESAQLV

>CORE_REP|Org114_Gene3493#

MSDANLLEATVRTEFGKGAARRTRRDGNVPAVLYGHNSDPQHLAVNAQAFAAILREHGTNAILSLDIAGKKQLAMTKSVVVHPIRRYIEHADLLIIKRGERVVADVHVTLVGEAAAGTLVTQEATVLSVEADAMKLPESIEVSIEGIEAGTQITAGGIELPKGVTLAGDAEALIVNIIAAPAAEATEGEGEAEGEAAAEAAE

>CORE_REP|Org208_Gene705#

MEAFTVHKGIGVPLRRSNVDTDQIIPAVYLKRVTRTGFEDALFAAWRSDPDFILNTEPFNRGTVLVAGPDFGTGSSREHAVWALSDYGFRVVISSRFADIFRGNAGKGGLLAAQMSQNDVEMLWKMIEEQPGLELVVDLRDRTVTAGTVVLPFDIDDYTRWRLLEGLDDIGLTLRRSDVIAEFEKARPTWKPTTLPAPISQA

>CORE_REP|Org76_Gene2857#

MYEGPVQDLIDELGKLPGVGPKSAQRIAFHLLQVEPPEIDRLQAVLQKVRDGVRFCAMCGTVADGELCRICADPRRDRTMICVVEEPKDVQAIERTREFRGRYHVLGGALDPLSGVGPDQLRIRELLARIGNQEDGVDVTEVIIATDPNTEGEATATYLVRMLRDFPGLSVTRLASGLPMGGDLEFADELTLGRALSGRRAL

>CORE_REP|Org90_Gene2635#

MTRIVAGTAGGRRLRVPPAGTRPTSDRVREALFSAIDARLDLEGARVLDLYAGSGALGLEALSRGAARAMLIESDRKAAAVVRGNIADLGLPGAELRVGSVASVLAQPAPVEFDLVFSDPPYDLDTATVVADLTALAGNGWLAPDALVVVERSSRSPEIDWPAGYSARKPRRYGETRIELAEFDGAPAEPGPRPASV

>CORE_REP|Org4_Gene4376#

MFVRFREFAVTIQPVRLFGDPILRARAAEVTAFDRELQQLVTDLTDTMHDDGGVGMAAPQIGVGLRVFVYDTHDAAGHLVNPQWEVIGDEEQVGPEGCLSIPGVRHDTRRALRVRASGVDMHGAPVEFIAEGLLARCVQHETDHLDGVLFIDRLDPAERKDAMRTIRESDWFSAGVTARPSRSVSGAGQAGPFGRGR

>CORE_REP|Org8_Gene5086#

MAILPIVIVGDPVLHNPTEKVTQSPEELAELIADMYETLDAAHGVGLAANQVGVPLRLFVYDCPDVRADGTATRRRGAVINPVLETSAIPETMPDPDDDEEGCLSVPGEQYPTGRATWAKVTGTDEHGNPVEIEGENFFARMLQHEVGHLDGFLYVDVLIGRNARAAKKAIKRNGWGTPGLSWIPGTVPDPFGHDD

>CORE_REP|Org46_Gene581#

MSSTESSAGPALVVGLGNPGPEYERTRHNVGFLVADVLAERVGGRFGVHKKSGADLLQARLDGRQVLIAKPRSFMNVSGRPVAALAKFFSVPPTEVIVVHDELDLPFGAIRLKRGGGEGGHNGLRSVSSALTTKDYLRTRIGIGRPPGRQDPADYVLKPFSAPERKEVPVIVEQAADAVELLLRVGLETAQNQLH

>CORE_REP|Org85_Gene5178#

MIDEATTNESALGTTAGSDRDRLAALVRELAVVHGRVTLSSGKEADYYVDLRRATLHHDAGPLIGKLLRELVADWDFDAVGGLTMGADPVAFAVMHAPGRPIDAFVVRKAAKAHGMQRQIEGPDIVGKRVLVVEDTTTTGNSPLTAVRALRDAGATVVGVATVVDRETGADQVIAAEGLEYRSILGLKDLALS

>CORE_REP|Org152_Gene5548#

MTVEIRELSVPGAWEFTPRLHGDARGVFLEQFKASEFEKAVGRPFDLQQVNVSTSAAGVLRGIHYTANPPGQAKYVTCVRGAFLDVVVDLRPDSPTFGRWDAVVIDDVTRRSVFLAEGLGHALLSLADDSTVTYLCSLEYTPEFDAEVDAFDPAIGIEWPTMGRDGQPLTVIRSAKDAAAPPLSDARLLY

>CORE_REP|Org12_Gene5806#

MTTAENTEKIAPRLKLRYRAEIKDALNNEFNYANVMQIPGVVKVVVNMGVGDAARDAKLINGAVNDLALITGQKPEIRKARKSIAQFKLREGMPIGARVTLRGDRMWEFLDRLVSIALPRIRDFRGLSPKQFDGNGNYTFGLSEQSMFHEIDVDSIDRPRGMDITVVTTATNDEEGRALLKHLGFPFKEN

>CORE_REP|Org188_Gene4603#

MVVIEEALFDAEEKMEKAVSVAKDDLGTIRTGRANPGMFARVVVDYYGSPTPVTQMSSITVPEPRMVVIKPYEAGQLGAIETAIRNSDLGVNPTNNGDILRITIPQLTEERRRELVKQAKGKGEDAKVAIRNVRRKAMDELARIQKDGEAGEDEVGRAEKELDKTTAKYVSQIDELVKHKEAELLEV

>CORE_REP|Org68_Gene229#

MADTSDFKNGLVLKIDGQLQQIVEFQHVKPGKGPAFVRTKLKNVVSGKIVDKTFNAGVKVETATVDRRDMTYLYHDGSDYVFMDGETFDQISISEATIGSSARFLLENMAVQVAMHEGAPLYVELPVSVELEVTHTDIGLQGDRSTGGTKPATLETGAEVQVPLFINTGDKLRIDSRDGSYLGRVNA

>CORE_REP|Org5_Gene1146#

MDGIPTAGTTSEDGAVTGEAVNGPQVGLIMGSDSDWPTMEAAAEALAEFGIRFEVGVVSAHRTPQRMLDYAREAAGRGIKVVIAGAGGAAHLPGMVASATPLPVIGVPVPLKYLDGMDSLLSIVQMPAGVPVATVSIGGARNAGLLAVRILAATDPELRTRMEQFQADLEKLVLDKDEALRTRLLG

>CORE_REP|Org15_Gene512#

MAKPEKVTAVAEITEQFKSATATVVTEYRGLSVGSLTQLRRALGSNATYSVAKNTLVKRAAAEAGVEGLDDLFVGPTAITFITGEPVDAAKALKTFAKDNKALVIKGGYMDGAPLSVSEVERIADLESREVLLAKLAGAMKGNLTKAAGLFAAPAGQVARLFAALEDKQRAAGGAEAAPAADAE

>CORE_REP|Org129_Gene4267#

MPKPKKGARFGGSASHQKAIFANLATALFEHGRITTTEAKAKALRPYAEKLVTKAKAGSLADRREVLKVIRNKDVVHELFANIGPSFEGREGGYTRIIKTVPRKGDNAPMAIIELVREKTVTSEADRARRVAAQQAKTEAAEAEEATEAKADEAKADEAAEAKADEAAEAESTEAPAEDKAE

>CORE_REP|Org99_Gene3320#

MRVVLLGPPGAGKGTQAVLLSEKLGVPHISTGDLFRANISQQTPLGREAQKYMDAGDLVPSDVTNRMVEARVNEPDAANGFVLDGYPRTVDQADALEKILGDMNSKLDAVLCFVVPEDTVVERMLARGRNDDTEDVIRNRMRVYREETEPLLDHYDGLVVTVDGVGEVDEVNERALRALGR

>CORE_REP|Org113_Gene4110#

MDLVVGRVAKSHGVRGELVVEVRTDEPELRFAPGATLRGRAPKSDVVRTYTVESAREHSGRLLVRLEGVGDRGAADALRGTLFLVDSADLPPSEDPDEFYDHELEGLSVRLADGTEIGTVNEVLHSAAGELLSIRASEGAGGRGGREILVPFVTAIVPTVSIAEGVIVIDPPEGLLDEEHQ

>CORE_REP|Org170_Gene1954#

MSRIGKQPIAIPSGVEVTINGQDIAVKGPKGQLSLTVSEPITVTKGEDGQLQVARPDDERRSRALHGLTRTLVANMIEGVTKGYEKKLEIAGVGYRVALKGQNLEFALGYSHPVVAEPPQGITFAVESPTKFSVAGIDKQLVGEVAANIRKYRKPEPYKGKGIRYAGENVRRKVGKTGK

>CORE_REP|Org105_Gene4498#

MSGARPGPEPNIAGRVRPHPADARVVRPAEADMIRAAIEAARAADPRDVPVGAVVFDADGRELARAANAREALGDPTAHAEVLALRRAAAVHGDGWRLEGATLAVTLEPCTMCAGALVLARVGRLIFGAWEPKTGAVGSLWDVVRDRRLNHRPEVRGGILEPECAALLDEFFHSQR

>CORE_REP|Org113_Gene3342#

MERVLWVIGLAALFALLLWLMYRSWVKRARKQASSVGELPGVPADLGAQVLEPTTGLYTGTTLAPSWQNRIVVGDLGFRATAELTRFERGILLERDGAEVIWIPQESITAVRTERGHAGKVMTDNGVLVIRWKLPTGTEVDTGFRGDDKTVYPAWTAVSGAGTSGTATEDEVNE

>CORE_REP|Org16_Gene3452#

MGRASARCAVVARRHRRRGCLAASRRICGAASRRSRFTQSGERVSTDRIELRGLRAYGHHGCFDFERRDGQEFLVDITLWLDFAKAAATDELSATVDYGELAQRAVAIVSGPPRNLIETVVAEIADDVMRDERVTAAEVVVHKPSAPIPHTFADVRVVATRRRGLPPTPEVAG

>CORE_REP|Org4_Gene621#

MSVSGNDDRSSTAPGALVYFSSASENTHRFVEKLGIPATRIPLHTADSLRVDEPYVLIVPTYGGGRHVLDAQRPNSISGHRSDKDFVPRQVAKFLNDPHNRALLRGVVAAGNTNFGDTYCYAGEVISRKCGVPYLYRFELMGTAEDVERVREGLGLFWQQQRQHRPEKRLA

>CORE_REP|Org5_Gene6165#

MSRPLSHPWTTVLGMTDKKLGPGDPAPDFTLPDADGKPVSLSDYRGRKVIVYFYPAASTPGCTKQACDFRDNLAELNEAGLDVIGISPDKPAKLAKFRDNENLTFPLLSDPDREVLQAWGAFGEKTMYGKRITGVIRSTFLVDENGKIEVAQYNVRATGHVAKLRRDLSV

>CORE_REP|Org105_Gene5423#

MVGVSAPSDRSAQHRPRHNRPALIALVVVAAVACLALGWWQWERFESSSGTGQNLGYALQWPLFAAFAVFAYFRFVRLESEAEEEQAAPAEVAPKPAKAKKAAPREIPAGILPERPKVRVDRPDAGPERGSDPALAEYNKYLAQLHADEIGERVRAAGLDTSDTERSAG

>CORE_REP|Org45_Gene5877#

MTTTTHTLSVLVEDKPGVLARVASLFSRRGFNIQSLAVGGTEIPEISRMTIVVTVEDLPLEQVTKQLNKLVNVIKIVEQDQESSVARELILVKVRADASVRTQVIEAVNLFRAKVIDVSPDALTIEATGTRSKLDALLRMMEPYGIREIVQSGVVAVGRGPKSITATR

>CORE_REP|Org5_Gene452#

MTDLTALRTGIGSDVHPIEPGRPCWMAGLLFDGDDGCAGHSDGDVAAHALCDALLSAACLGDVGAVFGTGRPEWEGVSGAAMLTEVRRLLDEQGYRIVNAAVQVIGNRPKIGPRRIEAQQVLGDLLGAPVAVSGTTSDGLGLTGRGEGIAAVATALLLHDGRCSTS

>CORE_REP|Org19_Gene4505#

MRMEQMYQEVILDHYKHPHHRGLREPFGAEVHHVNPTCGDEVTLRVHIDDNGDVADVSYDGQGCSISQAATSILTDQVIGQPVQQALKVVDSYSEMISSRGTVEGDEDMIGDGIALAGVAKYPARVKCALLGWMAFKDAVVRITSAEETKRTMGNGSAGNGKRHD

>CORE_REP|Org1_Gene884#

MNEDERTVVAKHQRTLPTVADTEALGRELAAQLAAGDLVVLDGPLGAGKTALTRGIAAGLGVQGRVSSPTFIIARQHRAGPRDGAPPVPMVHVDAYRLGGDLDELDALDLDTDLHQAVVVVEWGRGVVEHLTDRHLWVRLTREPDSEVRTAVWEWVDRQPSPPSR

>CORE_REP|Org5_Gene1043#

MAGRASGYGLGVLKLVFFEPRIPPNTGNAIRLAAGTGCELHLIEPLGFDLSEPKLRRAGLDYHDLAVVTVHENLTAAWKSLRPERVFAFTTHATTRSTDISYRAGDVLLFGPEPTGLPEEVLTDPHITDQLRIPMLPGRRSMNLSNAAAVAVYEAWRQLGFPGAV

>CORE_REP|Org36_Gene539#

MTETNVTWLTPESHDRLKSELDALIANRPVIAAEINERREEGDLKENGGYHAAREEQGQQEARIRQLQELLNNAKVGVAPTKSGVALPGSVVKVYYDGDESDTETFLIATREEGLSDSKLETYSPNSPLGGALIDAKVGETREYTLPNGNTMKVTLISAEPYHS

>CORE_REP|Org22_Gene3343#

MHTDGADPREQLRAAGLRVTAPRVAVLDAVAARPHSDADTVAVTVRQQLGSVSTQAVYDVLHACVRAGILRRIEPAGSAALYEARTGDNHHHLVCRNCGTVVDVDCVVGSAPCLQPDDEHGFAIDEAEVVFWGLCPNCRPDRRPTPRQSRVSPPGSVDQPRRLL

>CORE_REP|Org140_Gene5#

MIFKVGDTVVYPHHGAALIEAIETRTIKGEQKEYLVLKVAQGDLTVRVPAENAEYVGVRDVVGQEGLDRVFQVLRAPHTEEPTNWSRRYKANLEKLASGDVNKVAEVVRDLWRREQDRGLSAGEKRMLAKARQILVGELALAEGTDDGKAETLLDEVLAAAS

>CORE_REP|Org120_Gene7541#

MATPALAKSKENRHLGKKDAPKKKKLAGIIKLQIQAGQANPAPPVGPALGQHGVNIMEFCKAYNAATESQRGNVIPVEISVYEDRSFDFKLKTPPAAKLLLKAAGVQKGSAEPHRNKVAKVTMDQVREIAKTKQEDLNANDIDQAAKIIAGTARSMGITVEG

>CORE_REP|Org101_Gene2781#

MPAMRVYLGADHAGFELKNLVKDHLEKAGHEVVDCGALEYDALDDYPAFCIEAARRTVADPGSLGLVFGGSGNGEQIAANKVPGARCALTWSVETAKLARQHNNAQLAGIGGRMHSTEEALAIVDAFVTTPWSEEERHQRRIDILAEYEKSGDAPAVPAY

>CORE_REP|Org210_Gene142#

MSGTGVPEFALADAKELTVGIVASRWHTTICDTLLANAERVAREAGVEQITVVRCAGAMELPVVAQALARTHDAVVALGVVIRGETPHFEYVCDAVTAGLTRVSLDEGTPVTNGVLTVNTEEQALDRAGLPDSAENKGEQAGAAALDAALTLRALRREV

>CORE_REP|Org141_Gene5144#

MGLSGPTAGWEPEGGPRYAVAVTEQTLILIKPDGVARGLVGEVLNRIERKGLKIAALELKQVSDELAGEHYAEHAGKPFYGSLIEFITSGPVVAAILEGPRAIAAFRQIAGGTDPVEKAATGSIRGDLALETQENLVHGSDSPESAKREIALWFPEFPA

>CORE_REP|Org5_Gene6906#

MSETTESAAPEQRADAGSRPLRRTRITIVTIATAVSVLAVLLVLAAWRDDMLITSDKGVTSAEVLSAGRLRSAVTYVTPDGVTHNPKVGVLYPTKLTAGERINVEYSRSDPELVRVAGRDARVAIIPALSVIVVVWAVALPAFWLVRRIARRRSVRAGE

>CORE_REP|Org39_Gene6837#

MHCPYCRHPDSRVVDSREAEEGTAIRRRRACPQCGRRFSTVETAILSVVKRSGVSEAFSREKVIRGVRRACQGREVDDDALNLLAQQVEDAVRAKGSPEVPSHEVGLAILGPLRDLDEVAYLRFASVYRSFSSAEDFEREIAELRKHREEQSVGATAD

>CORE_REP|Org147_Gene1284#

MPDTAAPKPPILVLNGPNLNMLGVRQPEIYGSETLADVVELCTRTAAGLGREVRAFQSNSEGALIDQIHQARGTVSGIVINPGGLTHTSVALRDALVIPEVPIVEVHVSNVHAREEFRHHSFISPIATAVIAGMGVFGYAAAIEFLARRSSTDAARR

>CORE_REP|Org22_Gene1736#

MPRKGPAPKRPLINDPVYGSPLVTQLVNKILLDGKKSTAERIVYGALEQAREKTGTDPVVTLKRALDNVKPSLEVKPRRVGGATYQVPVEVRPGRANTLALRWLVNFSRARREKTMVERLANELLDASNGLGASVKRREDTHKMAESNRAFAHYRW

>CORE_REP|Org5_Gene5213#

MSSQSDSSATSSLPAPKIELSPQEWRSRLTPQEYAVLREAGTERAFTGEYTDTETTGVYSCRACGAELFSSSEKFHSHCGWPSFFDPADSDAVILRSDDSLGMHRVEVLCANCHSHLGHVFEGEGYPTPTDKRYCINSISLRLHPSNNADDATH

>CORE_REP|Org46_Gene1359#

MMSTVALKRSWAQDLDTATLYQLLKLRVEVFVVEQKCAYPELDGKDLLPETRHFWLDDEGEVIATLRLCEEHHDGVKSFRIGRLCTAVPARGHGYTTRLLQAALAEVGSATVRLSAQSYLIDLYSKHGFKVDGAEFEEDGIAHVPMRRGGE

>CORE_REP|Org85_Gene5823#

MTAYRTIVVGTDGSDSSYVAVEKAAALAGVSAATLVIACAYYPTDDRDVAAAADVLKEEAYQVRGSAPTNEILRTARDKAAAAGAVDIVERAVVGEPVESLLTLTKEVDADLLVVGNRGLNTLTGRLLGSVPSDVARKSRSDVLIVHTVR

>CORE_REP|Org12_Gene5784#

MTPIKLHHLRPAPGAKTEKIRVGRGEGSKGKTAGRGTKGTKARKNVPASFEGGQMPIHMRLPKLKGFTNPFRVEYQVVNVGDIARLFPQGGTIGKDELVAAGAVRKNQLVKVLGEGEIGVAVQVSADKFSGSAKEKITAAGGTATELG

>CORE_REP|Org31_Gene710#

MSEAKSKLTQSTTETQNPHARATAKHVRVTPMKARRVVDLVRGKRVEDALAILKFAPQAASEPVAKVVASAAANAENNLGLNPDTLVISTAYVDEGATMKRFQPRAQGRAFRIRKRTSHITIEVESIPAAGGAATRNRRKGGAK

>CORE_REP|Org68_Gene3456#

MFLGTYTPRLDDKGRLTLPAKFRDDLAGGLMVTKGQDHSLAVYPKEEFTALARRAAAASRSNPQARAFVRALAAGTDEQRPDAQGRITLSADHRRYANLSRDCVVIGSVDFLEIWDKQAWESYLAEHEEDYAQARDESLGGIF

>CORE_REP|Org5_Gene4599#

MTAMTILYTAEALATGDGRNGHARTSDGKLDLTLAMPPEMGGSGAGTNPEQLFAAGYAACFHSALRLVGGQEKADISDSAVGARVGIGPNETGGFGLEVTLEISLPNLPREQAQALADKAHQVCPYSNATRGNIDVHVTLAED

>CORE_REP|Org151_Gene30#

MLRTMMKSKIHRATVTHADLHYVGSVTVDQDLLDAADLLEGEQVCIVDIDNGARLETYVIAGERGSGVIGINGAAAHLVHPGDLVILIAYGQMNEQEIAEYDPKVVFVDERNRPVELGSDPAHAPEGSGLTSPRSLSFAG

>CORE_REP|Org11_Gene1425#

MLMPRKVKHRKQHHPSRSGMAKGGTSVAFGEFGIQALEPAYVTNRQIESARIAMTRHIKRGGKIWINIYPDRPLTKKPAETRMGSGKGSPEWWVANVKPGRVMFEMSYPNEEIAREALRRAMHKLPMKCRIVTREEQF

>CORE_REP|Org169_Gene15#

MPPKSRASGPKKSQKARRRDKKNVPHGHAHIKSTFNNTIVSITDPEGNVISWASSGHVGFKGSRKSTPFAAQLAAENAARKAQENGVKKVDVFVKGPGSGRETAIRSLQAAGLEVGTISDVTPQPHNGCRPPKRRRV

>CORE_REP|Org143_Gene2339#

MTEIDWKLLRDKAIGVMRNAYAPYSRFPVGAAALCADGRIVSGCNVENVSYGLGLCAECVLVGNFQSGGGGRLLAVSVTDSRGEILMPCGRCRQVLYEFGGAELLVDHPRGPIRLGELLPDAFGPDDLDAVQGPRSP

>CORE_REP|Org4_Gene7830#

MIMAQTENQIAKRKPRGKDVSTTRRLSKTRRHFRLRKKVVGTTERPRLVVNRSSRHLHAQLVDDSVGKTIAAASSIEADVRALDGDKSAKSKKVGELLAERAKAAGVDAVVFDRGGHDYHGRIAALADAAREAGLKF

>CORE_REP|Org14_Gene446#

MDSAAARNLSASEDSSASEEIVAVVEGASGAAVEGASGAVVEGAPEGAAVDGLTRRELDILDFERKWWKYAGAKEEAIRELFAMSATRYYQVLNAVVDKPEALAADPMLVKRLRRLRASRQKSRAARRLGFQV

>CORE_REP|Org105_Gene256#

MKPGRDRVGVTFRPLDPKKNSEALHSNKKGATKVAALTQEQIVEELGKIIEEVTGIEPSEVTIEKSFVDDLDIDSLSMVEIAVQTEDKYGVKIPDEDLASLKTVGDAVSYIQKLEAENADAAAELKAKFDNAE

>CORE_REP|Org59_Gene5282#

MTMTDPIADFLTRLRNANSAYHDQVKAPHSKLKANIAEILKREGYIADYRTEDAQVGKTLVVDLKYGPSRERSLAGVRRVSKPGLRVYAKSTNLPKVLGGLGVAIISTSQGLLTDKQAAKQGVGGEVLAYVW

>CORE_REP|Org189_Gene129#

MANVDELLETFGNMTLLELSDFVKKFEEKFEVTAAAPVAVAAVGGAAAGGAAEAESEQDEFDVILEGAGDKKIQVIKVVREIVSGLGLKEAKDLVEGAPKPILEKVAKDAADAAKEKLEAAGASVSVK

>CORE_REP|Org5_Gene4391#

MSETPTTEQQATEQPAAEPELTKEQIALLEDLEEAMRDVVDPELGINVVDLGLVYGMRLEEEVAVLDMTLTSAACPLTDVIEDQSRNALVRSGLVEDLKINWVWMPPWGPDKITEDGREQLRALGFTV

>CORE_REP|Org57_Gene1684#

MPTINQLVRKGRRDKVAKTKTAALKGSPQRRGVCTRVYTTTPKKPNSALRKVARVRLTSAVEVTAYIPGEGHNLQEHSMVLVRGGRVKDLPGVRYKIIRGSLDTQGVKNRKQARSRYGAKKEKS

>CORE_REP|Org198_Gene78#

MARLMGVDLPREKRMEIALTYIYGIGRTRSKEILAATGVSPDLRSKDLSDDQVTQLRDYIESSPELKVEGDLRREVQADIRRKIEIGCYQGLRHRRGLPVRGQRTKTNARTRKGPKRTVAGKKK

>CORE_REP|Org169_Gene424#

MAKKVTVSLIDDVDGESIADETIEFAIDGVSYEIDLSAANAAKLRDGLDVWVANARRVSGRRRSKPVGATTGAPKGRVSMDREQSAAIREWARRKGHKVSARGRISADVVESYHKEVGRNN

>CORE_REP|Org38_Gene3295#

MTVQNETATETHGVVLTDAAASKAKALLDQEGRDDLALRIAVQPGGCAGLRYQLFFDDRSLDGDLTVDFDGVKLAVDRMSAPYVQGASIDFVDTIEKQGFTIDNPNATGSCACGDSFN

>CORE_REP|Org5_Gene3354#

MSPRTSAGDTPTGRDNRLRDICGRLKEWRPVKTFESLFAELQDRAANRPEGSATVAALDAGVHAQGKKVIEEAGEVWIAAEYETDDQLAEEISQLLYWVQVLMVGRGLKLEDVYRHL

>CORE_REP|Org38_Gene3810#

MSDSAKTVAGKTVTVTDASFADDVLLSEKPVLVDFWAAWCGPCKMVAPVLEEIAGTHADKLTIAKIDVDANPETARDYKILSLPTMMLFRGGKPVKQIVGAKGKAALLRELDDVI

>CORE_REP|Org4_Gene1773#

MEMNTLDFVDEKSLRSDVPDFRPGDTLNVHVKVIEGSKERIQVFKGVVIRRQGGGIRETFTVRKVSFGVGVERTFPVHTPNIDHIEVVTRGDVRRAKLYYLRDLRGKAAKIKEKR

>CORE_REP|Org4_Gene3250#

MRLLTRADLPPKSAPVHVPAIPRVSDPAANIERTPMKAGIHPTYVDTTVVCGCGNTFQTRSTKESGHITVEVCSQCHPFYTGKQKILDTGGRVARFEARYGKRAGKKADSDAK

>CORE_REP|Org79_Gene2198#

MKLITAIVKPFTLEDVKTGLEQAGVLGMTVSEVQGYGRQKGHTEVYRGAEYSVDFVPKVRVEVVVDDASVDKVVEVIVEASRTGKIGDGKVWVTPVETIIRVRTGERGTDAL

>CORE_REP|Org56_Gene4690#

MADRVLRGSRLGAVSYETDRDHDLAPRRVARYRTDNGEEFDVPFADDAEIPPTWLCRNGQEGILIEGTTQEPKKVKPPRTHWDMLLERRSKEELEELLQERLELLKTRRGR

>CORE_REP|Org102_Gene4428#

MALPQLTDEQRAAALEKAAAARRARAELKERLKRGGTDLKTVLQDAEKDEVLGKMKVSALLEALPKVGKVKAAEIMSELEIAPTRRLRGLGDRQRKALLARFDFSAE

>CORE_REP|Org100_Gene1403#

MATYAIVKTGGKQYKVAVGDLVKVEKIEGEPGAAVELSPVLVVDGAELTTEAEALAKRSVTAELVEQTKGPKIRIHKFKNKTGYHKRQGHRQPLTVLKVTGIK

>CORE_REP|Org162_Gene6068#

MALRARTGNYRRNSVALTTEQKKAILAEYGVHEKDTGSPEAQIALLSKRIADITEHLKKHKHDHHTRHGLMALIGRRKRLSKYLQDKDIERYRALIERLGLRR

>CORE_REP|Org59_Gene5336#

MTTIADPRDILLAPVISEKSYGLIEEGTYTFLVHPDSNKTQIKIAVEKVFGVKVTSVNTANRQGKRKRTRFGYGKRKNTKRALVTISADSKPIEIFGGPVA

>CORE_REP|Org158_Gene5032#

MRSVRHYEVMVILDPSLDERTVGPNLNNMLNVVTAEGGKVDNVDIWGRRRLAYEIAKQSEGIYAVVNLTANSDTVSELDRQLGLNESVLRTKVLRLDKK

>CORE_REP|Org107_Gene612#

MPRSLKKGPFVDDHLLAKVDVQNEKGTKQVIKTWSRRSTIIPDFIGHTFAVHDGRKHVPVFISDNMVGHKLGEFAPTRTFKSHVKEDRKSKRR

>CORE_REP|Org26_Gene4485#

MAHKKGASSSRNGRDSNSKRLGVKRFGGQTVKAGEILVRQRGTHFHPGVNVGRGGDDTLFALEAGAVAFGTKRGRKTVNIVVPEPVEA

>CORE_REP|Org4_Gene7783#

MSVTGIAEDMAKKDGAIEVEGRVVEPLPNAMFRIELENGHKVLAHISGKMRQHYIRILPEDRVVVELSPYDLSRGRIVYRYK

>CORE_REP|Org73_Gene4579#

MATGTPAAELRELTEEELVSRLRESKEELFNLRFQMATGQLDNNRRLRVVRHEIARIYTVMRERELGLATGPAGKGDAA
