# Supplementary material for: Design of a multi-epitope vaccine against six Nocardia species based on reverse vaccinology combined with immunoinformatics
Source: Front Immunol. 2023 Feb 2;14:1100188. doi: 10.3389/fimmu.2023.1100188 (PMC9952739; doi:10.3389/fimmu.2023.1100188)
Supplement: Supplementary file 11 [file Table_4.docx]

>CORE_REP|Org119_Gene7073#

MSSLATDSVDQCSSGESSAQPFVLSPAQTALWYAQRIRPDVPLTIAQYVEIHGDLDVGRLLYAIERFGAESEVGKLRLAEIDGIPHQIVDPARRPGWARVDLRGERDPHAAALRWMHEYTGSPIDLERDPLTANVVLRTGDSDYIWYSRAHHIVIDGYGAMNALTRTAEIYTALENRTEPVVSRAAPLAEIYADEVRYRETSRFRADRDYWLEQLAGAGEPMSLGGSTVTAATQDAGRRIAAGVLDDRAQAAMDAAVTTFGTANSALFVAALGAYVRSVTGNPDVVLSLPVSARTTVSLRRSAGVVSNVVPIRLRFGAETTLAEVVKATELQITGALRHQRYRHDDIRRDCGYSRDARGFFGPMVNIMLFHDELTFGSLVGSLNVLATGPVEDLSVNLYNGVGGRIHVDFEANPRLYGEAEVSVHHDRFLDFLTRFLGAAPDTHAETLTAITAAEHERVLHEWNATEAPRQPGTLAELFAERAAACPDAIALESGDDTADPSPVHPVTTLTYRELDERANRLARLLIERGAGPETVVGLCLRRSIDLVVGMYAIVKTGAAYLPLDPEHPADRLDQIVRQASPVCVLTAARDELAMPESAAALAIDTVELSGYRGAPITDAERTAALRADHLAYVIFTSGSTGKPKGVGVSHAAIVNRLRWMQHEYSLDRTDVVLQKTPATFDVSVWEFFWPLQIGARLVVAAHDGHRDPAYLARLIAEKGITTAHFVPSMLSVFVTDTDVRGCTALRQVFCSGEALPAATVRDFHAALPRPALHNLYGPTEAAVDVTYWPCPADPATVPIGSPVWNTQTYVLDSRLRPVPPGVVGELYLAGVQLARGYLGQPRLTADRFVANPFGAGVRMYRTGDLARWQLGTDRPGVLEYMGRSDFQVKIRGLRIELGEIEAALLDDARVARAVCVAHPGRNGDELVAYVVATPAAGRLDTTALLTELRRTLPAYMVPSALLELDELPLSANGKIDRKALPAPVGVRATGRSTAEPRTEVERVLARVFAEMLGTEVGVEDSFFDLGGNSLVAARAVARINAALGTGLTIRDLFEASTIAALTQRFATHPADVSSPKLVAAQRPERIPLSLAQQRLWILNRFAEHAAAYNMPLAVRIEGALDVEALRAGLVDVIERHESLRTTFPESAEGAVQLVHPAAEIPLTLDPIDAAGADVAELATEFAGYGFDLRSQAPIRVALYRTGPDQWVFLVVLHHICGDGWSIAPLARDLMTAVAARGAGAAPQWAPLPVQYADFALWQRELLGNESDPASALSGQLTHWRSALAGLPDQLDLPLDRPRPLRRSTTGGRVDFTISPEIRRAASELAAARGVSMFMVLHAALATLLSRLCASTDIAIGTPIAGRSDPALDELVGMFVNTLVLRTEIDPAAGFDRMLDVVRETDLNAFANADVPFERLVEVVNPERSAARHPLFQVMLSYDRDPDLRIELPGVRAEVLPIVSDIAKFDLQLVVHDDVTDGPLTAEFGYATDIFDRATVESFARRFVAVLNAVVAAPSMPIGDLSILDRREIANLVPIAGAPAEPFTTLARLLTDTAERVPDAVAVRYLGVDTTYRELDESSNRLARVLIEHGAGPEVVVAIALPRGLDAITAVWAVAKTGAAYVPIDPSYPGERIAHMIGDSGAILGLTDAACLAAMPEWPAPRGKHRKNYVDWLVLGSAELAAEAVHCSTAPITDADRHHSLCTVHPAYLIYTSGSTGKPKAVVVTHAGLASLANEQTHLFGVTDSARTLHFSSPSFDASVLELLLGFAAGATIVVAPAGMYGGAELATLLRTERVTHAFVTPAALATVPTDGLDELEAVIVGGEACSEELVETWSAEHRMHNMYGPSEATVAATATGPMVPGRPVPLGQPIRGMRLFVLDGRLHPVPPGTPGELYLSGPGLARGYHGRYGLTAQRFLANPHGRRGERMYRTGDLVVVETGGQVRFLGRADDQIKIRGFRIELREIDHVLRAHPGVNFALTVVHTDEHGQPRLASYVTVDHPVAAADLTETARQRLPGYMVPASVTVLAELPVTPAGKLDRKALPEPVFATGGSSRAPATELESRVAGVFGEILGRPVTGAEDSFFDVGGNSLLATRLAAALHAEFGVDLPVRVIFEAPTVAGVAERLTEAPRTQRLALAVQTTRPGRIPLSLPQQRLWFLNRYSPESSAYNIAFVIRIAGDLDVAALRAALTDLVERHEVLRTVFPEDSAGAQQVVLPTARALPAIEAIDTDEAGATAALGALAHRGFDLIRDTPLRMTLLRTGSERYLLGIVVHHIAADGWSLGPLTRDLAAAYVARHGGAAPAWTPLPVQYADFGLWQRACLGDEGEPGSLAAEQLAYWRSALADLPAELPLPYDRPRPAEPTQYAGAVPFTVPDPVQRALAELAKEQGVSMFMVLRSALAVLLRSVTGGRDIVIGTPVAGRTDTKLDELVGMFVNTLVLRSDVDPDRPFAGLLRADRDTELAAMAHADIPFERVVEELASGTTRGRHPLFQVALTVQDGPVPTLELPGLELRAEELDIALAKFDLELRVAHIGCDAGPGEPGRAFEFVYAAELFDEATIHTLADRFLRVLAAVTADPRVLVRDIDTRTERERRLLAPATGGPTTPQCTLAAYFTATAHMHPHRTAVRSGATTLTYAELDKRSNRLARALLARDIGIGDRVALGLTRSVESVLTVLAVVKTGAAFVPVDPNYPADRVRHMLADAGCWVGVTVGAHAERLRTAAADGPATDWLLLDDPAVRAELETYDDALVDDLDRMCTIEAADLAYLIYTSGSTGKPKGVAVTHAGLSNFADELRDRMRVDRESRTLHFASPSFDAAVLDLLLAVGSGAAMVLCPPDVYGGDELAALLERERITHTFMTPAALATIDHERWPLPHLRALMVGGEACAPDLVARWAPGRTMLNGYGPTETTIVATIATLTAEQPVTIGTLVRGARALVLDERLRPVPAGVPGDLYLGGHGVARGYFDRFGLTALRFVADPFGPAGARLYRTGDVVRWNDAGELCYLGRSDHQVKVRGFRIELGEITAALGEHPAVRFAHTEVRQIAGADRIVAFVQPADEHTGVDVEAVRDRLGAQLPAHMVPASITVLERIPLTPVGKLDSAALPEPQLAVAAATREPSTPSERLVARVMGELVGVDAVRADDSFFDIGGNSLLATQLVARLAAASNTRLEVRTVFAAPRVAELAAHLDSGPAGARSRPALVRQARPDRIPLSAAQRRLWFLNRFNGIGEAAADGADLSAGAYNVPVVLRMNGKLNVDALVVALHAVQDRHETLRTVFPEVGGEPTQRVLDLVTAAITLFVATVRPDEVDDAVRRFAAPGFDLAGVVPMRAALISVSPDGDRGVRNPAEVSDEHVLVLVVHHIAMDGQSLAPLALDVATAYRAACADRSPEWDELAVQYVDYTLWQQDTLGTEDDPDSVIRRQLDYWRHQLDGVPELLTLPADRRRPPVPSYRGGLVECEIDAFTHRDLHRVATSNNVSMFMVLHAALAVLLHRMSATDDITVGTPIAGRGHPALDRLIGMFVNTLVLRTRIDPDARFTDLLHTVRDVDLDAFAHADLPFERLVEVLNPARSQAHHPMFQVMLSVQNHPVGGLELPGLRIEAADVDTGIAKFDLQFTLTEAQTPERDPAGITLSVNYASDLFDEQTALRLGHRLARLLAAVAANPTTAVGDLELLDPAEWSGLAPVRGAEPDRPVTFPEVFAAAAAVDRAAIALRADGTQISYDALDRWTNRLARVLMRRGVGPETLVALGIPRSVESVATVLAVAKAGAAFVPVDPNYPAPRIAHMLSDSGAALGITLSAHRDELPGDVEWIVLDDPIFRGLVLDSPDGPIAAAERTAPLRIDNPAYVIYTSGSTGTPKGVVVTHGGLSNFAAETAQRFDVRPGCRVLHFATPSFDAAMLDLLLALGGAATLVITPPGVVGGEDLARVFIDEAITHAFITTSALGTVDPTGVTALRHVLVGGEALPPDLVTRWAPNRNLYNVYGPTETTIVTVISQPMTPGGPITIGGPIRGVSATILDGRLHPAPVGVTGELHLAGSALARGYLNRPGLTAQKFVANPFGKPGERMYRTGDLVRWWTGQGSPAAGRDHGGSREIEYVGRTDHQVKIRGFRIELGEIDAALAKHGGVEFATTIGHRTPAGSTALVSYVKARNGIGLTAAELTEHVAGLVPNYMVPQSIMLLDRVPLSPVGKLDRKALPEPVFSAADGYRAPATPTEVALCAAFAAVLGVETVGADDGFFELGGNSLLATKVVAQVRANGLDLPVQAMFGEATPAAIAARLDGSGAGIVAALGPVLPIRPNGKAAPLFCVHPAIGLAWCYSGLLAHLAPDRPVYGLQAPHVAGEDGFASIAEAAQQYVAHIKSIQPTGPYHLLGWSLGGLIAHEVAVQLQEAGDEVALLSMMDSYRLSDAWLEHAIPSVAEIIEEFGSDQLDAPLDPAMNLRDAAELLRARPGPFAALTVEHLERLYAGYTNGTLLAHGFRPRVFDGDLLFFTAAADEINRADPERTAAAWQPFVTGAIRDHELPCRHSAMTAPESLAAIGQVLRGALDGAAVLLPAGAQPAKNGVRRTKSGARKEKQR

>CORE_REP|Org15_Gene5536#

MADNEGTQTTETTGDANAEVEQQTTATEQSVTEPGGTAKSGGTDMSVAELREWLQRWVADATGQPVEQITVDRPMEEFGLASRDAIALGGDIEELTGVLLNPTIVYQHPTIAALAERVINGEPEAPEEAADDAFYTAGYQPGAAHDIAIVGLSTRLPGAGDTPESTWDFLINRGDGIRELPEGRWSEFLADPDIAAAVENGNTLGGYLDQDAIKGFDAEFFAMSPVEVERVDPQQRLMMELTWEALEHARIPANTLKGESVGVFIGTSTNDFQLVASLGLGKSDPDAPASADAYALTGGSTAIIANRVSYFYDFRGPSVAVDTACSSTLVAVHDAVRALRNGDADVALAGGVNMLLAPAITLGFDSIGAVAKDGHIKAFSSDADGMVRSEGAGMVVLKRLADAERDGDRILAVVKGTAVNSDGRSNGLPAPNPEAQVDVLRRAYRDAGIAPSTVDYIEAHGTGTPIGDPIEADALGRVVGRGREDDKPVLLGSAKTNFGHLESGAGAAALAKVILALQHNVIPPNIGYAGPSPFIPFDQAHLKVVDEPTEFPRYSGTATIGVSGFGFGGTNAHVVIQEYVPAASVESKEEAAQIASAETIEAELDNEATDVLAGAEAILEGSDPLAEPEPVAEVAEWTQERTEPLPVILPVSAYLPSRRRRAASDLADWLESEAGQAAPLEDVARSLAKRNHGRSRGVVLAKTHEEAVAGLRAIAAGKPGPGVFTADSPAAQGSMWVLAGFGSHHRKMGKQLYLENSIFARTVDEIDELVVDEAGYSVKEMILDDAQDWDVGTSQVGVFAIQLGLAALLRAHGAEPAGVVGHSQGEAAGAYISGGLPLEDAVRVICARSRLMGEGEQMITDDQVRNMALVEYSAEDIEKVLPEYPDLEVAVYAAPTNTVIGGPPDQVHAIVARAEAEGKFARVLQTRGAGHTSQMDPLLGELAAELAGIEPTKVTTDLYSTVHKATVYKAGSDPVHDVDYWVTNMRGSVYFTNAIRRAVDAGITTYLELAPNSVALMQVMGTTFAAGVHNAALIPTLKRKEDEAAGVISALAQLYVQGHPVDLVSLLPAGDYADVPRTAFLRKEYWPKVSIATGSGSGRAPGAHVALPDGRHAWEVAASAVTDLAGLVNAAAAQVLSEVALGATIAHSPLPASGTLTTTLTPHPGGASVQVHVREDNVFRLLFDAVVTASAPSTNGTSAPAVQPAPAPAETDSGTADLVVAESFGERWDPNGTQTVEERLATIAAESMGYAVEDLPMEIPLMELGLDSLMAMRIKNRVEYEFDIPSLQVSAVRDASLNEVGKVLRYAIEHRDEVAAMAEKQATEGGSLTVDDNFVAAARAAMEAGEDPAAAVTQQVEAAEPKPVESATPAGSADAVAEAADVDQKASATAGSASQAAEPKVEAADAKDGAADAKTGSGKGTAPAQAAAVFGGGQVAGAKEPEADVPPRDAAERLTFAAWAMVTGKSAGGIFNTLPILDEDTADKLAARLSDRVGSTIDVDDVLDCETIEQLSDIVRRHQDSATEVEGFIRPLRPRPEGSTAIPVFVFHPSGGNTLVYEPLLKRLPEGTPMYGFERIEGSIPERAREYAAEIRKIFPSGPYALYGWSLGAVFALQVAQIMRAEGDDVRLVGLIDLALPVEDEDPSPEGRRARIERFQAFAQKTYGIEGQLDDEMLQELADASDEEQLEIIMGLLKFADVKIPGGVMEHQRTSWLDSRDLQKAQPSHYEGDVTLYLADRYHDGMIELEPRFAERKPNGGWDDYIPNLEVIHIPGDHLQIIDEPRVAQIGADLTRKLAAISTEADDAPGKGEQ

>CORE_REP|Org107_Gene6262#

MATEGFVRRPRIAPPRAPGGEVALTPPPEVTRALPAPLMMKLMPVVMVVAVIGMIAMMAMMGRNLLANPLSMMFPMMMLMSMVGMMAGFRGGTGKRAVELNEERKDYFRYLDQVRKDVRRTGNKQLETLVWSHPEPADLPSLIGTRRMWERRPNDPDFGHVRVGMGSHRLATKLARPETGPLEDLEPVSTVALRRFVRTHSVVHGLPTAVSLRAFPAINISGSPEDSRMLVRSMLMELVTFHGPDHLAVAIVCADPDGAWGWAKWLPHLQHPTQRDGMGSARMMYTSLGELETALAAELMERGRFMRNPQPTQGRLHLVVIIDDGYVNGNERLISESGLDSVTVLDLTAPEGGLAARRGLQLIASDGDVSARSAAGVEKFATADMVSPAEAEAFSRTLSRYRLATAAQIVSLGEGSTADPGLMALLKIPDAAQIDPARVWRPRTARERLRVPIGITPDGTPVEIDIKESAENGMGPHGLCIGATGSGKSEFLRTLVLSLVTTHSPDALNLVLVDFKGGATFLGLDSLPHVAAVITNLEEELSLVDRMKDALAGEMNRRQELLRSAGNYANVTDYEKARAAGVPLDPLPALFVVVDEFSELLSQKPDFAELFVMIGRLGRSLHVHLLLASQRLEENKLRGLESHLSYRIGLRTFSANESRAVLGITDAYHLPSVPGAGYLKSDASDPLRFNASYVSGPYVAPQGTVTGEDGTPVGGQRLALFTAAPVEMPAPPEEEEASPLDLPPSPTNPMLELPPPPSALGLPGAPGSDEGIPDSLLDVVVKRLTGHGRPAHEVWLPPLDESPTVDMLLPDPDWRSPVNRHGQLWMPIGVIDKPYEQRRDVLTISLAGAQGNVAVVGGPQSGKSTTLRAIIMAAAATHTPQHVQFYCLDFGGGSMAGLVGLPHVGSVAGRLDSDRVRRTIAELTSLMRQREERFAELGIESMAEFRRRKFAAAAHVPEGAASSGNPLADDRFGDVFLVIDGWAVIREEFDVLESQINAIAAQGLSYGIHVIIGASRWAEIRPVVKDQIGTRLELRLGDPTDSEMGRRTAFQVPVGRPGRGLTPEQLHMLIALPRLDSDSDPSTLADGVSRARQELAELHAGRHAPEVRMLPMQFSRDELLATTRAQGIELSPTKVVVGLGESELQPLVLDFQTEPHFMAFADVESGKTTLLRNIVMGVVENSDPEQAKIIMIDYRRTMLGVVEGEHLAGYSTSSQTCGPMIQEVAEFLSKRIPGSDITPQQLRDRSWWEGPEIYIVVDDYDMVATGGINPFAPLIEYMPQARDIGMHFVVTRRMGGVSRALYDPIIGGLKNMSVDTLIMSGSRDEGKIIGEIRPSKLPPGRGTLASRSKGQEMVQIAYLPPV

>CORE_REP|Org134_Gene4052#

MDPRARSARAGHEAGSAVSDTASWRPELASDSVEPAADAEEAGTEPAQPGTDSAPTGGPAVDAAPGPGTSTGPDASAGSDTTTGPGAAARPATSGDGAAPSADHPEELDTQENNPIAIEDELEEMGLEGAREIGHGGFGVVYRCVQRALDRVVAVKVLSSDIDAESRERFLREEHAMGRLSGHPNIVDILQVDVTATGRPFIVMPYATRGSLEVVVRDNGPLGWSDTLRAGVKLAGAIESAHRAGILHRDVKPANILLSSYGEPQLTDFGIARVPGGFRTSSSMITGSPAFTAPEVLKGDEPTVRSDVYGLGATLFALLTGHAAFERQAGEKVVAQFLRITTQPVPDLREQDIPADVAAAIEQAMAQNPRDRPASAYEFGEMLRAIQRTHGQMADEMALLDTEDEAEAAAAPSGNRTGPAVTARRSWPLNLSPPTPRPGYDPAPTTTFPPTAATKFRPPTPAREPVQRTRLLDILRTGGRRRLALIHAPAGFGKSTLAAQWRGELTADGVAVAWIGIDSDDDNEIWFLAHLIEAIRRVRPDIGTGLDQVLEEQPADAVAYAITTLIDDVHAGGATVVVVVDDWHRITDPGTRRVMDSLLDNGCHHLRFVVTSRDQSGLPISRMRVRDELVGIGSAELRLTREETRQILVDRNRFTLDDAQIDELHRATDGWPAAVQLISLALRGNPDPDPLIQHLAEGGHGVREYLAENVIDALEPRMVDFLTAISIAEKVSGSLAAALSDDPEAEHLLEQAEQRELFVRRVEYDPEWFRVQPLFAEHLRARLERTDPARVKVLHRKAARWYAEHQLLRKSVDHAVSATDLKMALDLLESGGMDLIDGSRLATLLGTVSKLPVQQVASRSKLLMAVARANVNLQQSGAARSALGRLSSVLSRGSSGDADVVRQRCQAAVLAAADQVARDHTEGVMDQIGDCLDHPDELPAWTVSTAANLTSFVRLCEFDFDGARSIQDWAAEYHERSKDPLGSVFGLCSRGAVAFEQLDIATAARCFQQAWDTARARSGQRSHAVRVAAALLGELHYRRGELDAADRLLDESHELVARVGPIDFLISTFVIGARVKAVRGDMATAASRLAEGQRIAVEQDLPRLAAQVRAERVRLGPAAETSGPQTATERWNGNVIRDTGSTGHRLTGTAALTAEAEEIAAIRELLADGGIDDQDRAVRRARALYGRTHELHRPRAQLDTSLLLAECLAAAGWVGEAAAQLVPAVTTCAELDWTRPLLDAGPGVVAILRVLRNDLPSELPIRFVDELLA

>CORE_REP|Org113_Gene3340#

MPRRNDLQHILVIGSGPIVIGQACEFDYSGTQACRVLRSEGLRVSLVNSNPATIMTDPEFADSTYVEPITWEFVEKVIVAEKAKGTPVDALLATLGGQTALNTAVALHENGILEKYDVELIGADFEAIQRGEDRQKFKDIVAKVGGESARSKVCYTMDEVRETVAELGFPVVVRPSFTMGGLGSGMAYNDDDLDRIAGGGLAASPTANVLIEESILGWKEYELELMRDGRDNVVIVCSIENVDPMGVHTGDSVTVAPAMTLTDREYQKMRDLGIAILREVGVDTGGCNIQFAVDPRDGRLIVIEMNPRVSRSSALASKATGFPIAKIAAKLAIGYTLDEIVNDITKETPACFEPTLDYVVVKAPRFAFEKFPGADPTLTTTMKSVGEAMSLGRNFSEALGKVLRSLETKAAGFWTQPDGRWTDVAEVLADLRVPIEGRLYQVERALRLGASVEEVAEASGIDPWFVAEIAGLVELRGEIAQAPVLDEPLLRFAKHNGLSDRQIAALRPELAGEDGVRELRHRLGIRPVYKTVDTCAAEFEAKTPYHYSTYELDPAAESEVAPQPDREKVLILGSGPNRIGQGIEFDYSCVHAAQTLSEAGYETVMVNCNPETVSTDYDTADRLYFEPLTFEDVLEVYHSESESGRVAGVIVQLGGQTPLGLAQRLTDAGVPVVGTSAAAIDLAEDRGEFGQVLVAAGLPAPKYGTATTFAQAKEIAARIGYPVLVRPSYVLGGRGMEIVYDESSLEGYISRATELSPEHPVLVDRFLEDAIEIDVDALCDGEEVYLGGVMEHIEEAGIHSGDSACALPPITLGRSDIESVRRSTAALAQGIGVKGLLNVQYALKDDVLYVLEANPRASRTVPFVSKATGVQLAKAAARVMLGTSIAQLRKEGILPAEGDGGHAPMDAPVAVKEAVLQFHRFRRPDGTGVDSLLSPEMKSTGEVMGIDTDFGTAFAKSQSAAYGSLPTEGTVFVSIANRDKRAMVFPVKRLHDLGFRILATEGTAEMLRRNGIPCERVRKHSDPEFPAGSAGAADEAPVPSVVDQIKDGEIDIVFNTPYGNSGPRVDGYEIRTAAVGANIPCITTVQGAAAAVQGIEATIHGGIGVRSLQELHAVLRGHEER

>CORE_REP|Org44_Gene5774#

MDKTDPLTQKSRQALHDAQTKAVRFGHTEVDGEHLLLALLDDPDGLVPRLLAQAQADPDTLRTALETELGRRPKVSGPGAAPGQIFLTQRLVRLLDTAEREAKRLKDEYVSVEHLVIALIEEGTTTAAGRLLHEHGLTRDRFLQALTAIRGNQRVTSAMPEVAYEALDKYGRDLVADAAAGKLDPVIGRDAEIRRVVQILSRKTKNNPVLIGDPGVGKTAIVEGLAQRIHRGDVPEGLRDKTVFALDMGSLVAGAKYRGEFEERLKAVLNEVKAAEGRILLFVDELHTVVGAGAAEGAMDAGNMLKPMLARGELHMIGATTVDEYRKHIEKDAALERRFQPVLVDEPDEADAISILRGLRERLEIFHGVKIQDSALVAAVTLSHRYISDRFLPDKAIDLVDEACAMLRTEIDSMPAELDELTRRVMRLEIEEAALAKETDPASQSRLTELRKELADLRAEADAMRAQWEAERAALRKVQSLRQEIDQVRHDAELAERDYDLNRAAELRHGRLPELERRLDAEEQQLTAKQGRQRLLREVVTADEIAAIVSRWTGIPVSRLQEGERDKLLRLDEILHQRVVGQDEAVQLVADAIIRARSGIKDPRRPIGSFVFLGPTGVGKTELAKTLAAALFDTADNMVRLDMSEYQERHTVSRLVGAPPGYVGYEEGGQLTEAVRRKPYSVVLFDEIEKAHTDVFNTLLQVLDDGRLTDAQGRTVDFRNTVIIMTSNIGSEYLLEGATAGGEIKPEARERVMAALRGHFRPEFLNRIDDIVLFKPLTEAEIERIVELMTDELRGRLAERRMTLHLSDPARHFIAQQGFDPVYGARPLRRFIAREVETRIGRALLGGDVHDGATIHIGLSDGGLTVSFDNPNTGPSQDPADRVAAGTGS

>CORE_REP|Org158_Gene3705#

MTQHLEQANAGQSNNDASATPPTPNSMPQRQGDPTSQRQSDPTSQRQGDPAAQRQGDTTAAQRPGDTTAQRPSLPVAQRQGGAPAAVPTSASRRVRARLARRMTGQRGIAAVKPVLEPLATVHRELYPKANLQLLQRAFDVADEKHAHQFRKSGDPYITHPLAVANILAELGMDTTTLVAALLHDTVEDTGYSLDELTNEFGQEVAHLVDGVTKLDKVNLGAAAEAETIRKMIIAMARDPRVLVIKVADRLHNMRTMRFLPPEKQAKKARETLEVIAPLAHRLGMATVKWELEDLAFAILHPKKYDEIVRLVADRAPSRDTYLAKVRAEIVNTLAASRINAIVEGRPKHYWSIYQKMIVKGKDFDDIHDLVGIRILCDEVRDCYAAVGVVHSLWQPMAGRFKDYIAQPRYGVYQSLHTTVVGPDGKPLEVQIRTQDMHRTAEFGIAAHWRYKETKGKHSNDSTEVDDMAWMRQLLDWQREAADPAEFLESLRFDLKSPEIFVFTPKGDVITLPQKSTPVDFAYAVHTEVGHRCIGARVNGRLVALERQLENGEVVEIFTSKAQNAGPSRDWQNFVVSPRAKAKIRQWFAKERREEALEAGKEAISKEVRRSGLPLQRLMSADAMSALAHELHYPDISALYAAVGESQVSAHHVVQRLMAQLGGVGDVENELAERSTPSTVPARQRGTGDAGVEIPGASGTVAKLAKCCTPVPGDEIMGFVTRGGAVSVHRTDCTNADSLRSEPERIIEVKWAPSPSSVFLVAIQIEALDRTRLLSDVTKVLADEKVNILSASVMTSGDRVAISKFTFEMGDPKHLGHLLNVVRNVEGVYDVYRVTSAA

>CORE_REP|Org46_Gene6654#

MASSCSPPNKPLPGDRAGHHGVAVTGRTTRVAPQAATARAASRSSGASTSERQETVAYRADLDGLRGVAIGLVVIFHVWFGRVSGGVDVFLVLSGFFFTGLLLRRADSTGSPGVGTTLRRTVRRLLPAMVVVLAAVVVASVIVRPYTQWWELSAQTLSSLLYVQNWRLALTWSDYLAADPSVSPLQHLWSMSVQGQFYLAALATVAVAAWTTRRSMRSAALRPVLAVTVGVLGVVSFWYAWRGGQTQQGWNYYDSIARCWELLAGALLAAIAPLLSPPRMARAGLAALGLFGVVGCGWLILDGANRFPGPAALLPVAAAAGVIVSGNNLPLDQRPWPNRILATPTARWLGDIAYPLYLWHWPILIFYLTERGQPHAGVAGGIVIVTLSIVLAWVTHRWVEEPLRLRSRPRAEAAGAEGTTISRRVAGVAVVALGAVVIAAAGGWLTVMARINPPHAVGALDPRLYPGAEALASGAAVPQAPMRPTVFEAPGELPPPTVDGCIADWDTREVITCTYGVPDAERTLAVVGSSHAEHWLPALQVLAGEYSFRIQVYLKMGCPLTLAEDAMYKGEPIPDCRDWSREVIDRLGADRPDWVFTTGTRPREDIGDETPPEYLDVWSALSERGLNVIAIRDTPWLRREKVRYMAIDCLAKGGDRIGCGMRRQDALDEVNPALEPASRYPNVFPVDLSDAVCEPTVCAVIEGNVLIYHDEHHFTVSYSRSLADALGRRLQPLLGWW

>CORE_REP|Org103_Gene2295#

MTTSAIHHTRDSGGIVTLTIDDPNQRVNTMNSLFVESLAAELDAIENDADVTGVILTSAKKTFFAGGDLNDLRAARRDRIDEFAAFVQRNSVLLRRLEKLSVPVVAAINGSALGGGLELALAAHHRIVVDAPGVTLGLPEVTLGLLPGAGGVVRTVRLLGVQAALRDVLLSGKKHPVAGALELGLVDATVATIEELIPAATAWIREHAGARQPWDTEGFRIPGGAPGERGAPLHATLPALAATLRAQTKGAPAPAQANILAAAVEGAQVDVDNALAIEARYFLDLAIGQIAKNMIQANFFDMQVVNGPRGRDTAREPWLPRKAIVLGAGMMGAGIAYQCAVSGIDVVLKDVTPEAAERGKGYSLRVLDKRVRAGQISAATRDEVLARITPTADVAAAAGADLVIEAVFEDPALKADVLREIEPLLAPDALIGSNTSTLPITGLAENVSAPDRFIGLHFFSPVDRMPLLEVIKGGRTSSETVSRALDLARTIGKTPIVVNDSRGFFTSRVIGTFVNEALAMLGEGVPAPVIEQATTQAGYPAPALQLADELNLELLRRVRDASRVAAEAAGGSWDPHPAEAVLDRMLGEFGRAGRLAGSGFYEYEDGARTRLWPGLRAAFGSPRADLPFTDLKERMLFVEAIESVKCLDEGVLESVPDANIGSLLGIGYPGWTGGVLQYIDGYPGGVAGFVRRAEELAAAYGARFAPPSSLVAVARDGGTLADAHRERQPALS

>CORE_REP|Org158_Gene1247#

MIGQILEGRYRIDAPIARGGMSMVFRGEDTRLDRPVAIKVMDPKFAADPQFLTRFELEARAVAKLKHPALVAVYDQGVDGDHPFLIMELVEGGTLRELLRERGPMPPHAVRAVIEPVMQAIGVAHSSGLVHRDIKPENVLISDSGEVKIADFGLVRAVAAANITSASVILGTAAYLSPEQVTSGHADARSDVYAAGVLIFEMLTGRTPFTGDNSLSIALQRVENDVPSPSHHISGVPPEFDELVAHATAREPAHRFADGNEMAAEIRRIAQVLQLPAYRVPAPQESAEHLSARYRVGPTPAPAAPAESRSRPAPVGAADMTTRLPAEPPTTRVPQAAAPPAHQHTRVMTAARELPPDYAQSAAHAPPPTLPPHGDQPPRNGYLADRGRSRRTAVLWLGAVVVLALLLGIGGWWLGVGRYEAVPAIAGMDRERAVATLQAAGFDTEVRDKASDTIPMGNVVGTDPSAGTKVVKGSTVAVLISSGKPKVPDIRPGQDVQSVKQAIRDAGLTPVDAGEVSSTAAEGTVAKVDPDPGTILPMGADVKVYTSKGSAPVELPNVRGKTEEEARAALDAVGIEVTGTRVEFDSKVKAGEVAGTDPAAGTTIDSSQGVVLLISNAVEVPGLLGSSVGDARAKLEALGLGVSVRQLAPSDSSIVISQSSVPGAKVEPGSTITLVALP

>CORE_REP|Org24_Gene1345#

MCGLLGYLTVDTSGAPEGTTAEAIAAQLHEALVCQRHRGPDERGTWHDEHMVFGFNRLSIIDIEHSHQPLRWGPPENRQRYAMTFNGEIYNYLELREQLTAEHGAEFGADPMFATEGDTETIAAAFHYWGPEAAARLRGMFAFAIWDTETRKLFIARDPFGIKPLFLATGPGGTAFSSEKKSLLDLLPQLGLSDALDPRALEHYTVLQYVPEPETLHRDVRRLESGCYAWVEPGQAPKITRYFDPRFRVVPFAKPGEVTAQPPTTRPRPAAQRPNTAEYRYREIAEALEDSVAKHMRADVTVGAFLSGGIDSTAIAALAIRHNPNLLTFTSAFEREGYSEADVAAETAAAIGAKHYIRTVSPEEFAASIPEIVWYLDEPVADPALVPLYFVAKEARKHVKVVLSGEGSDELFGGYTIYREPLSLKPFEYLPKPLRRLAGRLSERIPDGTRGKSLLHRGSLTLEDRYYGNARSFNDAQLRSVLRDFRPEWTHRDVTDPIWAMQGRDWDPVARMQHLDLFTWLRGDILVKADKMTMANSLELRVPFLDPEVFAVAEKIPVDQKITKDTTKYALRRALEDIVPPHVLHRAKLGFPVPLRHWLRGPELYDWARQQIIDSATDHLLDKTAVLGMLDAHRAGTSDHSRRLWTLLVFMIWHGIFVEQRIKPEIQEPTYPVSL

>CORE_REP|Org8_Gene457#

MSRTRGTWTSVVAAILLVAGMATACSSDDTDEAADVCATTPNGTLVAASPTGPTGSKDISTNPELSTGYRSGMVAARTKTFAVATANTLASKAACEVLRDGGTAADALITAQTMLGLVEPQSSGIGGGAFLMYYDAASKSVEAYDGREVAPAAATENYLRWVSDTDRTEPKPNTRASGRSIGVPGVLRMLEMVHREHGKTGWRELFDPAIGLADRGFSISPRLAAQVAEQAKNLALDEAAKAYFLNPDGTPKPADTLLTNPAMAKTLGAIASEGAQAFYTGAIAQDIVAAATSTSGGRTPSLITTADLAGYQAKKRTALCTDYRNHQICGMPNPSSGGSTVAATLGILENFDLAALPPDNLGAGSDTARNGGKPKAEAVHLIAEAERLAYADRNKYVADTDFVPLPGNSLQTLLNKDYLKQRSALIDRNRSMGTAQPGDFGPVPLGVGPQPPEHGTSHISVVDQYGNAAAMTTTVESEFGSFHLVDGFVLNNQLTDFSADPLGTDGAPVANRLQPNKRPRSSMSPTLVFDKAPDGARGNLTHVAGSPGGSVIIQFVVKTLVGMLDWGLDPQQAVSALSFGAGNSPATGVGGEHPSINTADNGDHDALVLRLRELGHQVSVAPQSSGLSALTRDGTAWVGGADPRREGAVLGDNR

>CORE_REP|Org15_Gene5535#

MSPCCVCLGGEGMTDETFDDYLDETGNIAIPEGRTLVDYVEKHTRNDANDLAYRYIDYSRERDGEYQDLTWKEFGVRLRAVAARLQQVTKPGDRVAILAPQGLDYVISFFAAIYAGTIAVPLFDPDEPGHTDRLHAVLGDCTPSAILTASSSAAGVRQFFRPLPAAQRPRIIAVDAVPDTLGESWVRPDLAVDDIAYLQYTSGSTRTPAGVEITHRAVGTNLLQMVHAINLDWNSRGVTWLPLYHDMGLLCVILPAIGGKYITIMSPSAFVRRPGRWISELAAVSDGAGTFAAAPNFAFEHAAARGLPKNGETLDLSNVIGLINGSEPVTTSSMKKFNEAFAPYGLPKTAIKPCYGMAEATLFVSATRAEDEAKVIYVDRNELNAGRVVKVDHSAPNAIAQVSCGYVALSQWAAIVDSESIDSPEGAQELPEGRVGEIWLHGNNIGIGYWGREEETRQTFKNLLTNRQAEGSHAAGAPDDAIWLRTGDYGVYVDGELYITGRVKDLVIVDGRNHYPQDLEFSAQEASKMLRPGFIAAFSVPANQLPAEVFAADSHAGLKYDADDASEQLVIVAERGPGAHKADSQPIADAVRGALSQRHGVTVRDVLLVPAGSIPRTSSGKLARRACRAAYLEGTLRGGYQQQAFPDAPDEE

>CORE_REP|Org105_Gene4310#

MTTPKNLSSRYELGEIIGFGGMSEVHKARDLRLSRDVAIKVLRADLARDPTFYLRFKREAQNAAALNHPAIVAVYDTGEAEVDGGPLPYIVMEYVDGETLRDIVRGKGPLPPRRAMEIIADVCAALDFSHKAGIVHRDMKPANIMINRSGAVKVMDFGIARAIADAANPMTQTAAVIGTAQYLSPEQARGESVDARSDVYSVGCVLFEILTGEPPFTGDSPVAVAYQHVREDPRLPSLVHEGVPRELDSVVLKAMSKNPANRYQTAAEMRADLIRVLGGQKPSAPMVMTDEDRTTILGSEEPAPRSYHTVDNHDRSAYRDNDDTGEPEPVDPPSQRRTAYLTLGAVAAVIVAIALFWVLIGPGSKPDQVAVPDLSNSSVQQAEQKLEDLGFHVAIQEKPDARVAPGNVIATQPLGGSRVDEGSTITLQVSTGPAQVQVPRLTGLTRQEAEQKLNAIGLRLDPQVDKEASSTAELDKVIGQNPAEGASVEVDRAVKVTIGSGPEQVRVPNVVGQDIEVAEPNLVEGAQFKVVVQEVASSRPKGEVIATSPAGGSTAEKGSTVTVQVSLGAEFTMPSLVGLNASHAVDRLRQAGWAGSTTQIVQNTQVTLDSANVGKVLNQQPAAGSSVGRNSTIVIYTGVLPLGPP

>CORE_REP|Org105_Gene5106#

MYRTGHADAIYVAAGPNSSVSAAVMKISGFHAMTGNRQAQRAFDAGILSLGLSIDGQESTRDLEYAKLAFQRATEWDPTMCDAWLGRAAAGEVTDEVIRNLHRTSTSTLYREQRRLGLAPRALAGRFVSGLYIDYPLASYTEIWLAYAANLIGSKQYDEAERVLDELAEYRAGMLSDPDREIDDRISAYIRGVLHFNTQRWPDVMSVLAGSAEWEDPYLATGAHVMVGSACAQLGLFGEAIRRMEQAENGPIPAARTTAMFCRGLCLRETGSEDEAQALFEQVYSQAPDFTANTEAMRDKSYRITITTKESIDARTDRWDPASAPSVEQLQTADAEDRAKKILTEARAELDRQIGLTAVKTQVAKLQATAQLAKIRAEKGMASVPRGNHLAFTGPPGTGKTTIARVVAKIYCGVGLLKTDKVVEAKRMDFVGQHLGSTAIKTDKLIDTAMDGVLFIDEAYTLIQTGLSGGDAFGREAVDTLLARMENDRDRLVVIIAGYDGEIDRLLAANDGLASRFAKRLQFPSYTPPELGQIGKLIASSRDSELSEDAVRLLEQACERLYNSERTDQSGQPRRGIDLAGNGRFVRNVIEAAEEEREFRLANDESLDLTAVDESVLMRIEAPDMEAALAGVLSSLGVS

>CORE_REP|Org101_Gene1042#

MALALSTGECQVGTVRPGSIDTLGRPSRAPNLASVMGRSNLRSSCVSAIYMEDLNNAMAKTIAYDEEARRGLERGLNALADAVKVTLGPKGRNVVLEKKWGAPTITNDGVSIAKEIELEDPYEKIGAELVKEVAKKTDDVAGDGTTTATVLAQALVREGLRNVAAGANPLGLKRGIEKAVEAVTAKLLDTAKEIDTKEQIAATAGISAGDSSIGELIAEAMDKVGKEGVITVEESNTFGLQLELTEGMRFDKGYISGYFVTDPERQEAVLEDPYILLVGSKVSTVKDLLPLLEKVIQAGKPLLIIAEDVEGEALSTLVVNKIRGTFKSVAVKAPGFGDRRKAQLADIGILTGGEVITEEVGLSLETAGIELLGQARKVVITKDETTIVEGAGDAEAIKGRVAQIRAEIENSDSDYDREKLQERLAKLAGGVAVIKAGAATEVELKERKHRIEDAVRNAKAAVEEGIVAGGGVALLQSAPALDDLTLTGDEATGANIVRVALSAPLKQIAFNAGLEPGVVAEKVSNLPAGHGLNADSGAYEDLLAAGVADPVKVTRSALQNAASIAALFLTTEAVVADKPEKAAAPAGDPTGGMGGMDF

>CORE_REP|Org98_Gene7027#

MLTEIRIDGLGVIATATAQFHAGLTCLTGETGAGKTMVVTSLHLLSGARADAGRVRLGAPRAVVEGRFTVDDVNDAARAEVAQVLEAAAAEPDDDGSVIAIRTVGSDGRSRAHLGGRGVPASVLADFTASLLTVHGQNDQLRLQRPDQQLSALDQFAGDAVGTALRKYQVLRRSWLDARTELLERTARSRELALEADRLKHSLNEIDAIAPEPGEDVRIVDEVRRLSDLDSLRDAAATAHGALAGPADTPEDGSGALEALGTARARIEAADDPALVALAPRLADAIAVVIDVTTELSGYLSDLPSDPGALDSLLTRQAELKTLTRKYAPDIDGVLAWAQEARTRLGSLDVSEEALAKLAAEVDTAADRVREAAKKLSGVRAKAAGKLAAAVSAELGGLAMGKARLEVEVRPLLAGAQDTAPLTVDGQELHAGHTGIDEAEFRLSAHSGAQSLPLSKSASGGELSRVMLALEVVLASSDHGATMVFDEVDAGVGGRAAVEIGRRLARLARTHQVIVVTHLPQVAAFADTHLVVDKSDDGKGAVNSGVRALTNDERVVELARMLAGLDDTETGRAHAEELLATARAEKAGAEAATR

>CORE_REP|Org216_Gene3082#

MLIRLLRTYLSPYRAQLAGVVALQLVSVIAMLYLPSLNADLIDNGVTKGDIDYIWHTGLWMLAVTAVQIVASASSVFLGAQAAMSAGRDLRAALVHRVGTFSAREVGLFGAPSLITRNTNDVQQVQLLVVMSVTVLVMAPIMCVGGIIMALREDLKLSWLLLIAVPALALAMGLVVARLVPGFREMQARIDVVNRVLREQITGIRVVRAFVRERQETWRFGLANTDLTEASLRVGRLMALMFPVVMLISNVTTVAVIWFGGHLIDDGELQIGSLTAMLSYIMQILMAVMMASFLAMMAPRAAVSADRIGAVLTTESSVVPPEFPKPFAGDPGRVEFAAAEFAFPGAEKPVLRGIRFTVEPGTTTAIVGSTGAGKTTLLNLIPRLIDVTAGAVYVGGTDVRELDMELLREQIGLVPQKAYLFSGTVASNLRYGRPEATDEELWRALEIAQAADFVRDMPQGLETPVAQGGTTVSGGQRQRLAIARALVRRPRVYLFDDSFSALDVATDARLREALRPETRDASVIIVAQRVSTIRDADQIIVLEDGEMAGIGTHEQLLRDCAEYQEIVASQLSAQEEVR

>CORE_REP|Org128_Gene3333#

MSATPFRGWWRANCDLNPISVSASRGRVRPVSYTHGVWDAPLLGETIGANLDRTVAIHGDRDALVDRVTGVRWSYREFAAEVDAVALGLLEAGIGKGDRVGIWSPNRAEWTLVQFATAKIGAILVNINPAYRSEEVRYVITQAGIRMLISAREHKSSNYAEIIGRVRPECPDLEQVVLFDSAAWEALVAAGRAADPSRLAEAGTRLTADDPINIQYTSGTTGFPKGATLSHHNILNNGYFVGELCGYTEADRICIPVPFYHCFGMVMGNLAATSHGAAMVIPAASFEPRATLAAVAEERCTSLYGVPTMFIAELAHPNFESFDLSSLRTGIMAGSPCPVEVMKQVIERMGMAEVSICYGMTETSPVSTQTRRDDTITQRTATVGRVGPHLEIKIVDPDTGSTVPRGEPGELCTRGYSVMLGYWNDPDKTGEAIDAARWMHTGDLATMDDDGYVAITGRIKDMVIRGGENIYPREIEEFLYTHPDILDAQVVGIPDPKYGEELVAWIRVREGAATVDAPTLAQFCDGRLAHYKIPRYVHVVDEFPMTVTGKVRKVDIRATSVRLFGVPELDQQQTGEQ

>CORE_REP|Org5_Gene6751#

MSSSSLHVRVGVCNLGLVSSDITATAAWRKLHDHHSAIADRHLREFFADDPDRGRELIVEAGELRVDYSKHRITRETLDLLLDLAATAGVARRRDAMFAGEHINTSEDRAVGHVALRLPAGASMMIDGADAGVAVHEVLRRMGDFTDGVRSGQWRGATGERITTVVNIGIGGSDLGPAMLFQALRHYADAGISARFVSNIDPADLTAKLDGLDPARTLFVVASKTFSTLETLTNATAARRWLVAALGEDAVAEHFVAVSTHAQRVADFGIDTANMFEFWDWVGGRYSVDSAIGLSIMVVIGRERFAEFLAGMHSIDEHFVSAPPERNAPILLGLLGVWYSNFFGAQSRAVLPYSNDLARFPAYLQQLTMESNGKSVRLDGSPVTTSTGEIFWGEPGTNGQHAFYQLLHQGTRLVPADFIGFARPTDDLATRDGSGSMHDILMSNLFAQTKVLAFGRTAAEIEAEDGDAPGFDPALVPHRVMPGNRPSTTILAPQLTPSVVGQLIALYEHQVFVEGTIWGIDSFDQWGVELGKQQALALEPLLTAAEDPAPQSDSSTDALIRWYRGNR

>CORE_REP|Org215_Gene1101#

MTPPPQTTPLALHDAARAYGDAPAVVDGAVRLSWAELLDSVRETARALLARGIGSGDRIGIWAPNTHHWVTAVLATHYVGAVIVPLNTRYVAEEAADVLARVDAKALFIAGPFLGRDRLAELRAAAPDLKIGTTIVIPGDTGPGGTDDAGTAVTDDAAAIGTGGVGVSAAGDAAARTGDDTLTWRNLAALAEQVSAADAIARAESVSPDDLSDILFTSGTTGRSKGTLIAHRQALAGARAWSECATLNSTDRYLVVPPFFHNFGYKAGILACLVTGATIVPQATFDVPETMRLVQDHRITVLTGPPTIYQTILEHPARRDADLSSLRVAVTGAATVPVVLIERMRTELEFDVVLTAYGLSESGGFGTMCRPEDDAETIANTCGRAIGDFEVALADNGEVLIRGSQVMLGYLDDPVATADTIDCDGWLHTGDVGTLDGRGYLKITDRLKDMYICGGFNVYPAEVEQALARLDGVAETAVIGVPDERMGEVGKAFVVRKAGSGLTADDVVAHAKTLLANFKVPRYVEFRDQLPYSAAGKVLKRQLRDDTGRAENEERA

>CORE_REP|Org24_Gene6553#

MVPSRRRSPARCSTPDWRGAERYERARWTGAGMLTNGALIADRYRLHRLIATGGMGQVWEALDTRLDRRVAVKVLKAEFSADPTFRHRFRTEAKTTAQLNHPGIAGIYDYGETMDPAGGETAYLVMELVSGEPLNAVLNRLGRLSVAQGLDMLEQTGRALQVAHAAGVVHRDVKPGNILVTPTGQVKITDFGIAKAVDASPVTKTGMVMGTAQYIAPEQATGEDATAASDVYSLGVVGYEALAGQRPFTGDGALTVAMKHVRETPPPLPPDLPPNVRELIEITMAKEPGQRYTSGGEFADAVAAVRAGRRPPPPSGLAGPMTSGATRVLPPGPTVILPTAARGDAATVRYPTPPHAQRAQQPPVATAMMQGPNTPPPGTPPIGGRTAEQGGGRFTNSQKALAGLGVGALVVGAAAAFVLLSGDPPDSTPPTKTSAVVVPPPVPTTTTTTEPPTTTRYVPPPPTVPPTTEEPLPTTTEPPPTTTTQPPTTTPQQPSTTKPAPTTTVKPSKTTIEPPFEIPSWPPTVPGGAGGAFGTTRPAHSSTPAPAAQQGLP

>CORE_REP|Org15_Gene6278#

MSDPRASGVRGRESADSTEQLDTGDRVHVTKSTGARVETAVPSNGNESSPDTYWRRAGRFRHRISRRLSAVPLRVTLALALVSLTGLGLLISGVAVTSAMRNVLMDNVDRQLFGAAHDWAGPDAPPPQRLPGPVGRERPPGLFYVRIEDPSGKVRSLFPTGPSVPDFPADLGKHPRTIGSVGNPDEHWRAERVTTPGGSSWVAIRLSETENIIDRLIGLQVAVGLMVLAVLAIVAQFVIRRSLRPLGEVEKTAAAIASGDLHRRVPVQGTNTEVDRLSQSLNGMLSQIQSAFAATEASEESARRSEARMRRFVADASHELRTPLTTIKGFAELYRQGALADPDMFMDRIERESKRMSLLVEDLLMLARLDAQRPVERRPVDLLALASDAVHNARAVDAAQRPEEPRRPIDLEIRPGTGTLEVRGDEARLRQVLGNLVNNALLHTPPEAAVTVALTPAPDEVVIEVADTGPGLPTEDAERIFERFYRTDTSRSRDSGGTGLGLSIVQALVAAHGGTVSVRSAVGQGTTFAVRLPRSQE

>CORE_REP|Org29_Gene4463#

MARTTSKRQAKSGANETVAPLGSSRRGADEPAPMRPPTPLTRTVSLRWRVTLLAASVVAIAVAVTSIAAYAMVARALYGDVDAQLRARAATMINGDIDSMAFQSLGVATLFSNNIGVGLIYPFSVSSPPSTPEGERTLDSLPVYIPPQPTKPPIGTEEIAVAKGEHTSSLRTYNNQRVLARRMDSGVTLVISQRLEPTREVLDRLAWLLFVVGGCGVLLAAAAGTAVGRTGLRPIARLTAATERVARTDDLTPIPVTGDDELARLTESFNTMLRALAESRDRQRRLVADAGHELRTPLTSLRTNMELLIAAGRPGAPRIPDEDMAELRMDVVAQIEELSTLVGDLVDLAREDAPETVYERVDLGEVAERALERARRRRGSIEFVAALRPWFVYGHEAGLERAILNVLDNAAKWSPAGAQVRVSMAEVGRGLLELSVDDAGPGIPPAERELVFERFYRTTASRSMPGSGLGLAIVKQVVTKHGGTITIDTSERGGALIRIVLPGEAGAPVATAEDEPDP

>CORE_REP|Org142_Gene5069#

MLDEGRTQFYGGHVPAPLTTRQQVNGYRFLLRRLDHALVRRDVRMLHDPMRSQLRSLLVGAVLGLLVVAGAAILAFIRPQGAIGDAKIVMGKDSGALYVVVADNDGGNTLHPVLNLASARLISGSSESPASVKDDKLADMPRGPLLGIPGAPSALPGSAQGTSSEWSLCDTVELSITGSAASASGVDTAVLAARPDLSERIRRADPDEAVLVRRSDRTYLIYEGKRAQVDPENSAIARALSLSGERPRPAGAGLLGAATPVPPIAVPEIPNAGKPGPGALSDIPVGGVISVAATGRGERAELYVVLADGVQHISDFTADVIRTANSQGMSQIETVPPDALTGIAVLSQLPVDHFPAAAPTILSAEDAPVTCVSWSKTEQSDADAVDGPTDRASAALLVGARLPLPEGAQPVSLATADGSGDRVDQAYLRPSSGEFVHVTGMEPGSPRRGSLFYIADNGIRYGVPDIDTAMVLGLGDAPALAPWAIVGQLVPGPTLASTDALTRHDVLPQSN

>CORE_REP|Org5_Gene928#

MVRNGRAAGWLRVAVAGAVLGAASGAGPAVMVGAGPASAVAPPAIDDGALGQAQAVNAKNGPPDETEKRAICAEPYLTGAVPRDPPLPQRILDLDRAWKFSRGAGQKVAVIDTGVNRHPRLPDLQPGGDFVTAGDGTEDCDGHGTLVAGLIAARPSPEDAFSGVAPEAQILAIRQLSLQYEAKNHRDDDTGKVAAGGYGDVLTMAAAVVRAVDMGATVINISEVSCSPAGSGTADGPLGAAVKYAADRNVVVVAAAGNLDQSACSVQNQTSGWNGVSTVISPAWFSPYVLSVASTDPDGATSPFSIHGPWVGVAAPGRTIISLDSKPGGTGLVDTEHGDEGPLTIDGTSFSAAFVSGLAALVRSRFPDLSAAQVIDRIERTAHNPGAGRDDRVGFGLIDPLAALTAQLPPPADRTGALPRAIAPPAPDPGPDPVPRRVAVIGSIALLALLVIGWAAALPYRRGRPGRGTGDPADGFVGTAETASPERISASSGPAGTDSPGGE

>CORE_REP|Org5_Gene949#

MPSKPTTRWQVSGYRFLVRRMEHALVRRDVRMLHDPMRSQSRAYAVGLVLGIVALAGCGVLALLKPQGSIGDNKILLGKDSGAVYAVIDGVVHPALNLSSARLAVGEPAKAVSIKESELAKKPRGALIGIPGAPSSLNFDGSGKGRAWSICDGLKNDGSQDLSTTVIAGDPSLGSKASRLGEGAALLVQGRDAAYLVYDNQRARVDMNDPKVTEALGIRGKTPRPISPGLLNAIPEVLPIEPPKIVDPGGMPTYSLNNHRIGDVVHVATKDQYYVVLRTGLQSISPLTADIIRNSNTAVSTDPEIDQSQAVQQNVSNELPVQKYPVKAPTIVEAKDQPVACMSWKPVAGASDKTDGSKRATLAVITGYSLPIPDNAQTTPLAQADGSGQNVDAFYSTPGSGFFVQTTGIETDSQRRDSMFFIADTGVRYGIKDANAQKALGMDAEKAKPELAPDQIVGLLAAGPTLGRQEAMVAHDGVAPDPAPAKQLVQSKQDQQAQQQSPN

>CORE_REP|Org210_Gene726#

MPQQTAVVVLAAGAGTRMRSKTPKVLHSLAGRSMLEHALHAANEIDPTALITVIGHDREQVGAAVNSVAAELGREITSAVQEQQLGTGHAVQCALTALPADFAGDLLVTSADVPLLDGHTLSALLDEHRSYQPRSAVTVLTFVPEDPNGYGRIVRDADGGVLEIVEHADATPEQAAINEVNSGVYVFDVAVLRTMISRLTTANAQHELYLTDVLKLAREAGNPVHGARLVDAAKVTGVNDRVQMAQAARTLNRYILERHMRAGVTVIDPATTWVDASVRIGRDAVLRPGVQLLGNTVIGEDAEVGPDSTLTDVLVGEGAKVVRTHGEGATIAAAATIGPFAYLRPGTIVGESGKIGAFVETKNASIGAHSKVPHLTYVGDATIGEYSNIGASSVFVNYDGVKKHHTVVGSHVRTGSDTMFVAPVTVGDGAYSAAGTVLRRNVPPGALAVSGGAQKNIEGWVQRYRPGTAAAQAAAEAIAADDRASQATEQKDGNTE

>CORE_REP|Org5_Gene6276#

MTVARSAESVHAVVKAYDVRGVVGEQIDAAFVRDVGAAFARLMRDSATRIAIGHDMRESSPELAAAFADGVLDQGLDVVHIGLASTDQLYFASGHLQCPGAMFTASHNPARYNGIKLCKANALPVGQETGLATIADELIEGVPAGPGPRGTATEQNLLEAYAEFLRGLVDLSGIRPLKVAVDAGNGMGGYTVPAVLGAVSQLTIEPLYFELDGSFPNHEANPLDPKNLVDLQKFVRETGADIGLAFDGDADRCFVVDERGEPVSPSAVTALVAERELAKEPGATIIHNLITSQSVPELVTELGGTPVRTRVGHSFIKQQMASTGAIFGGEHSAHYYFRDFWGADSGMLAALHVLAALGGSDGPEGGSLRNHEGPRGRRIEHSEKDDRTMSELSSSYSTYAASGEINSTVADAKDRTLAVVTAFEGRARSVDRLDGVTVRLPGNAWFNLRASNTEPLLRLNVEARSQEEVDALVTEILSIVRG

>CORE_REP|Org112_Gene2784#

MVATNTRETAESADAADSADTTAARPVKKAAAKKAPAKKAAAKKTAAKKTAAKKTAKATKATKAAKKAAPKKAGEGADGAETENLDDESLEIDDLGDLEVDEEDLGDEELEVEDDEAEDEAEEAEAETEEEADEPTAKDKASGDFVWDEEESEALRQARKDAELTASADSVRAYLKQIGKVALLNAEEEVELAKRIEAGLYATEKIREYADKGEKLNVQLRRDLNWIMRDGNRAKNHLLEANLRLVVSLAKRYTGRGMAFLDLIQEGNLGLIRAVEKFDYTKGYKFSTYATWWIRQAITRAMADQARTIRIPVHMVEVINKLGRIQRELLQDLGREPTPEELAKEMDITPEKVLEIQQYAREPISLDQTIGDEGDSQLGDFIEDSEAVVAVDAVSFTLLQDQLQSVLETLSEREAGVVRLRFGLTDGQPRTLDEIGQVYGVTRERIRQIESKTMSKLRHPSRSQVLRDYLD

>CORE_REP|Org13_Gene5085#

MEDHVTTPESVRLTSAVDPATAVSRLACSGHFSDYIVYERPGRWVFAAAPLGRVELDTDELRVSTSAGSARERWTGRPVDALERALDSLRVTCGPGASGTAYGWIAFEFCADALGAQRHLTERADLAHVIIPRIEVTVTESGVGVDGATAAEVEVIEQLLLSPAEPLPTPHPVDVRADTCGYRARVAAAVAEIAAGDYQKVILSRRVELPFRVDLPASYRLGRAHNTPARSFLLRLGGLAAAGFSPELVVSVDDEGVVTTEPLAGTRALGQGVAADLAARNDLESDPKEIVEHAVSVKTSFAEIASIAEPGTTTVADFMAVRERGSVQHLASTVRGRLARHRTRWDALDALFPAVTASGIPKRAAVDAVFRHDAARGLYSGAVVTLSESGSLEAALVLRAVYQDADAAWVRAGAGIVAQSRPDREFEETCEKLGSVAPYLVPAISHGPGSTPRSPMVTGRRSHRS

>CORE_REP|Org215_Gene1707#

MNSLFLLALAIVLVPLGGIFAALDSSLNTISAARVDDMVRAERPGAARLAHIITDRPRYVNLMVLLRVLCEITATVLLAAVLLDWMDQLWALVVTAAVMVLVDYLVIGVGPRTLGRQHAYSLALAASLPLQAIGTLLGPVSRLLILIGNAITPGKGFRNGPFASEIELREVVDLAGERGVVADDERRMIQSVFELGDTAARAVMVPRTEMVWIESEKTVAQAMSLAVRSGHSRIPVIGENVDDIVGVVYLKDMVPYADRSRKVRVHEVMRAAVFVPDSKPLDDLLDEMQRRRNHMAVLVDEYGGIAGLVTIEDVLEEIVGEIVDEYDQNEVPDVEDLGNGKYRVSARLSVEDLGELFGMAIEEEDVDTVGGLLAHELGRVPLPGSKAVAHGLVLKGEGGSDARGRVRVHTVVVKRAAEKTGAEKSDAGRSTSERVDGESAGVNGVGGSGANEDGEADD

>CORE_REP|Org117_Gene5341#

MNRRVRDSGRDDGYGRRTALAREWESGVETLLVVGAGPKALAVAAKSHVLRQLGLSAPRVIAVEAHAVGGNWLASGGWTDGRHRLGTSPEKDIGFPYHSTWARGHNREINEAMMAFSWTSFLVEHGTYAEWIDRGRPSPQHHVWAKYLQWVARKIDLELVLGKVRTIRQRPTDGGAGWSVEVAGADGATTELEADGLMITGPGQSTKALAKHPRVLSIAEFWDLAGKRKLPISSRAAVIGGGETAGSALDELVRHEMLTISVISPMATIYTRGESYFENSLFSDPTKWNALSIQERRDVIRRTDRGVFSVRVQESLLGDNRVHHLQGRVTRIVGQGDGVAVTLRNEMRADQVHNFDLVVDATGGQPLWFLDLFDSESADLLELAVGGPLTQQRIESSIGYDLAVTGLGAKLYLPNMAALAQGPGFPNLSCLGELSDRVLRAEPARVRAGARQLAAQ

>CORE_REP|Org4_Gene7917#

MPLQQFESFIRSAVGHIDLNEEVESEMSNAGTPKTAAEIQQDWDTNPRWKGVTRNYTAEQVSKLQGTVVEEATLARRGSEILWDLVNNEDYINSLGALTGNQAVQQVRAGLKAIYLSGWQVAGDANLSGHTYPDQSLYPANSVPSVVRRINNALLRADEIAKVEGDDSVKNWLAPIVADAEAGFGGALNAYELQKAMIAAGAAGVHWEDQLASEKKCGHLGGKVLIPTQQHIRTLTSARLAADVADVPSVIIARTDAEAATLITSDVDERDREFLDGTRTAEGFFGVKNGIEPCIARAKAYAPYADLIWMETGVPDLEVARKFAEAVRGEFPDQLLAYNCSPSFNWKAHLDDATIAKFQRELGAMGFKFQFITLAGFHSLNYGMFDLAYGYAREGMTAFVDLQEREFKAASERGFTAIKHQREVGAGYFDTIATTVDPNTSTAALKGSTEEGQFH

>CORE_REP|Org101_Gene2174#

MVGAWCWTASPKLSAVRVLVIGSGAREHALVLALRRDPAVTGIVAAPGNAGIAQHAQTRPVDPCSAEAVVALATDVAAELVVIGPEVPLVLGVADAVRAAGIACFGPSAAAARIEGSKAFAKDVMAAAGVRTAHSEIVDNPADLDAALDRFGPTWVVKDDGLAAGKGVVVTADRSAARDHGAELLEQGHPVLLESFLDGPEVSLFCLVDGETVVPLLPAQDHKRVGDGDTGPNTGGMGAYTPLPWLSPDAVTTIIEDVVKPVAAELVRRGSGFSGLLYAGLAMGVAGPAVVEFNCRFGDPETQAVLALLESPLGELLAATANGTLAEVEPPRWRDGSAITVVVAAENYPGRPRIGDVISGAGDGAIDDTAAVLHAGTALREDGALISAGGRVLNVVGVGADLAEARTNAYARITAIKLPGSHYRTDIGLAAVEDRIAVPDRASASSGQTRES

>CORE_REP|Org103_Gene5459#

MACDLRGTSRTRRWSRGRRPYDSGVSVPQAVLLAVLAAVVGLAVGGLLIPYVNARQAARRQADSGLTMSQVLDLIVLASESGIAVVDQYRDVVLVNPRAEELGLVRNRLLDERAWAAVEKVLATGESAEFDLTAKNPLPGRSRIAVRGVARPLSQEETGFTVLFADDDSEQARMEATRRDFVANVSHELKTPVGAMSLLAEALLESADDPEAVRHFGQRVLGESRRLGKMVTELIALSRLQGAEKLPELEVVDVDTVVMQAVDRSRTAAEAAGITVSTDRPSGLEVLGDETLLVTALSNLVENAIAYSPPGSHVSVSRSLRGKYVAMAVTDRGIGIAKEDQERVFERFFRSDKARSRATGGTGLGLAIVKHVAANHNGEITLWSKLGTGSTFTLRIPAHLEADSGDDDVDADGAAVSTKENGSRPSGPGRPNGVEARR

>CORE_REP|Org209_Gene2601#

MITAETNVFESLESNVRGYCRNWPTVFTTAKGAWLQDEDGKDYLDFFAGAGALNYGHNNPVLKQPLIDYIASDGITHGLDMSTAAKRKLLETLRDTVFAPRGLDYKVQFPGPTGANAVEAALKLARKVTGRETVLSFTNAFHGMTLGALSVTGNAAKRAGAGVPLVHAAHMPYDGYFDNTTADFQWMERVLDDTSSGFDRPAAVIVETVQGEGGINVARVEWLQHLAQLCAEREILLIVDDVQMGCGRTGPFFSFEVAGITPDIVTLSKSIGGYGLPLALVLFKPELDQWAPGEHNGTFRGNNPAFVTAQVALETFWSDGALEAATKAKGEKVATELATVAGHFPGLSTRGRGLVHGIAFEDPSQAGKVCQVAFERGLLVETSGSSDEVVKLLPPLTITDDELDQGLQILTGAIDTVCTGWGRLHHRAPAEGGDRR

>CORE_REP|Org12_Gene4586#

MRTLDTSFSTRNGGFREVVVTAVEITTSIGADTESTWQALLSGASGIKVLTDEDITRHDLPNAIGGKLIHDPTADLDRVRKRRMCYVQQMSYAMGQRLWETAGAPEVDKDRLGVCIGTGLGGADVIVEANDTMREHGYRKVSPFAVPMSMPNGVSGVVGLDIGARASLVTPVSACASGNEALVHAWRSIVLGDADMVVAGGVEGYINPMAIAGFTMARALSSRVDEPERASRPFDRDRDGFVFGEAAALLLVESEEHARARGATPLARLLGAGLTADGYHMVAPDPEGLGCARAMRRAIETAGVSAADVDHVNAHATGTSIGDLAEAKGIAAAIGTHPAVYAPKSALGHSVGAVGALEAAISVLTLRDQVIPPTLNLDNQDPEIDLDIVHDKPRHTDVEFAMNNSFGFGGHNAAVLFGRY

>CORE_REP|Org15_Gene4950#

MSTEQLQQRWSSALMNNYGTPKVALVRGSGAVVYDAEGKRYVDFLGGIAVNSLGHAHPAILEAVAQQLATLGHVSNLYVSEPVLELAERLLAHFGDGTGPIGQGSEEAAGVTTEGPRSGRIGTARAFFCNSGTEANEAAFKIARLTGRHTIVACEEAFHGRTMGALALTGQPSKRAPFEPMPPGVVHVPYGDAAALAAAVDSDTAAVFLEPIMGESGVIVPPPDYLAEARRITSERGALLILDEVQTGICRTGPFFAHQAAGIVPDVMTLAKGLGGGLPIGAVLAQGPAAELLTPGLHGTTFGGNPVSAAAALAVLRTIDEQGLAAHVESVGKTLIDGIEELGHPLIDHVRGAGLLIGIQLTQDVSAKVEEAARAAGYLINPPKPNVIRLAPPLILTEAQAQGFLVDLPGILDAAFQESE

>CORE_REP|Org102_Gene1805#

MLGSTRLPRALPIRWAPVTVASPFDLIVVGSGFFGLTIAERTANLLGKRVLVVERRYHLGGNAYSEADPETGIEIHKYGAHLFHTSNKRVWDYVNQFTEFTGYQHRVFAMHKGQAYQFPMGLGLLSQFFGRYFTPDEARKLIAEQSAEIDTKDAANLEEKAISLIGRPLYEAFIRDYTAKQWQTDPKELPPGNITRLPVRYTFDNRYFNDTYEGLPKHGYTAWLAKMAESDLIEVRLDTDWFEVRDEIRAQNPDAPVVYTGPLDRYFDYAEGELGWRTIDFETEHLETGDFQGTSVMNYNDADVPYTRIIEPRHFHPERDYPTDKTVIMREYSRFAQTGDEPYYPINTPDDRAKLLAYRERAKTETAAAKVLFGGRLGTYQYLDMHMAIGSALNMFDNVLRPHLESGAPLVDTAE

>CORE_REP|Org142_Gene4996#

MRRDSVRRPGERRGSRPVSEDRVIFSRPYRAAAEVENLRAVLDSDHSHGDGRFTKTATAKIKAITNSPHALLTTSCTHALELGALLLELGQDDEVIVPSFAFTSAATAVALRGATCVFVDIDPATGNIDPMSVADAVTDRTKAVLVMHYGGVAADMAPLLEIAGEHGLALIEDNAHGLGGTWRGRALGTIGTIGTQSFHDTKNVHCGEGGALLLSDEILMGRAEIIREKGTDRARFLRGQVDKYSWQDIGSSYLPSELNAAVLDAQLAEFDRIQTGRHRVWDAYASALPEWARRNDVRLMQVPGDREHTAHLFYLRLPSEDIRDTMIRHLADRGIVAPFHYVPLDSSPAGLKYGRTPVPCTHSAEFSATIVRLPLWPMLGDDQIQRVVDAVTAFAV

>CORE_REP|Org142_Gene4386#

MWDAIQYCLEYRPEEIPVAVDRLLPTDEAKDLIQLTRDVADKVLAPIVDEHERSETYPEGVFATLGEAGLLTLPYPEEWGGGGQPYEVYLQVLEEIAARWTAVAVAVSVHSLSIHPLMAFGTEEQKQRWLPEMLGGTTIGAYSLSEPQAGSDAAALACRATGVDGGYRITGSKAWITHGGIADFYNLFARTGEGSKGISCFLVDKDTEGLSFGKPEQKMGLHAVPTTSARYDDAFVPSERRIGNEGQGLQIAFSALDSGRLGIAAVAVGLAQAALDEAVAYAQERVTFGRKIIDHQGLGFLLADMAAAVDSARATYLDAARRRDAGLPYSRNAAVAKLVATDAAMKVTTDAVQVLGGYGYTRDFRLERYMREAKITQIFEGTNQIQRLVISRHLAG

>CORE_REP|Org150_Gene5131#

MPSLDNAGSHTPREDSGATDAAASQAGTDAAAAHADTGAAASRAGTDPAASRVGTDAAAPRAGSDAAGGADTAVRVSGADPAVVVDDVRKSFGEVQALQGISFTAARASVLGILGPNGAGKTTTVKILSTLLRPDSGSASVAGHDVVADAAGVRRSIMMTGQYAALDENLSGRENLELFGRLMGLPKKDARRRADTLLEEFDLVGAGKRAVRHYSGGMRRRVDIACGLVVRPEVVFLDEPTTGLDPRSRQGVWDLVNALKEQGITVLLTTQYLEEADVLSDNIIVIDKGTVIAEGTADELKEKTGGSYCEVVPLDPTQLRKAVTALGELVPEALRHEFAGDRISIPAPDGASTLAEAVRRLDAAGLELADIALRRPSLDDVFLSITGHSGGHQ

>CORE_REP|Org1_Gene5478#

MPGPVVVSDTSPTVPASTGLWSGIMNGFLLARPDGVLRASGVRAAFDAVNDARAALRTGGAAIVVGALPFDPARPAALVAPAEQVHTAGPWRPAALPPLPRVQVVSEFPSAGEHLARVTKLVEQLDDPDTELRKVVAARSVLAEADGVLEPETVAAQLAARHPGASVFAVDLTAAGRTGATLIGASPELLVARRGRTVTLHPLAGTAPRRADPDADAAQAAELLDSAKNREEHSYVIEWIRDVLTPLCTELRIPEGPRLVETHDVWHLATPIVGTLREPAPTALDLAVLLHPTPAVCGTPTAAALETITRIEGDRGFYGGAVGWCDADGDGTWVVAIRCAELAADGRSLRAYAGGGIVAASQPQAELDETTAKLRTFLGGLDCAVPTH

>CORE_REP|Org132_Gene4629#

MSDFLSTGTLPEEYRELALTVRDFANQVVAPVAAKHDAAHTFPYEVVSGMADMGLFGLPFPEEYGGMGGDYFALCLALEELGKIDQSVAITLEAGVSLGAMPIYRFGNEAQKQEWLPQLTSGRALAGFGLTEPGAGSDAGGTRTTAVRDGDDWIINGSKQFITNSGTDITRLVTVTAVTGESEGKKEISTILVPTDTPGFVAEPAYNKVGWHASDTHPLSFTDVRVPQSNLLGELGRGYANFLRILDEGRIAIAALSVGAAQGCVDESVRYAGEREAFGRAIGRNQAVAFKIARMEARAHAARTAYYDAAALMLAGKPFKKQAAIAKLVASEAAMDNARDATQIFGGYGFMNEYAVARHYRDSKILEIGEGTTEVQLMLIGRELGL

>CORE_REP|Org80_Gene5463#

MAGNPDFDLFKLEDFHDELRAAIRGLAEKEIAPYAKDVDGNARFPEEALTALNAAGFNAVHVPEAYGGQGADSVATCIVIEEVARVCGSSSLIPAVNKLGTMGLILNGSEELKQKVLGDLVNGKMASYCLSEREAGSDAASMRTRAKQDGDDWVINGSKCWITNGGKSEWYTVMAVTDPDKGANGISAFMVHKDDEGFVVGPLEHKLGIKGSPTAELYFENCRVPGDRIIGEPGTGFKTALQTLDHTRPTIGAQAVGLAQGALDAAIAYTKDRKQFGKAIADFQNTQFMLADMAMKVEAARLMVYTSAARAERGEQNLGFISAAAKCFASDVAMEVTTNAVQLFGGAGYTTDFPVERMMRDAKITQIYEGTNQIQRLVMSRALLKG

>CORE_REP|Org105_Gene4411#

MLVRRGPRVSDEEAGVVAIRLLTGRSGERSSRMRSARRPGRTTRSGSWVKRAAIGVAAAMLVPMGVSIAGPAAPASAAFNPAGFDFWVDSGMGPIKSRIFRAKDGNTNRVVYALDGLRAPETLSGWEIDTNVAQLLTDWNINVVMPVGGMSSFYADWNAPSSFAGIPPGTGSSSGSGALNALAAGPGKSYRYQWETFLTQNLRWALRDRLGFNPNRNGVFGLSMGGSAALTLAAYHPDQFSFAGSYSGYLNISAPGMREAIRLAMLDAGGYNVDSMAPPWGPQWLRMDPFVFAPLLRDNNTRLWVSAGSGLPGPADGPTAGTVNGMALEALALANTRAFQLRMATLGANNVVYSFPNVGIHAWSYWAEEVARMTPDLSAHIG

>CORE_REP|Org63_Gene1320#

MTGDTGGSRAGTDAALVLEDGRVFRGQAYGAVGQTLGEAVFCTAMTGYQETLTDPSYHRQIVVAAAPQIGNTGWNDEDDESAKIWVAGYVVRDPARRASNWRATTTLPDELERQRIVGIAGIDTRALVRHLRTRGSMKAGIFSGDALAGPDELVARVNGQPSMLGADLAGEVSTDALYTIEPDGEHRCTVVAVDLGIKTNTPRMFAQRGMRVHVVSSSTPLEQILELKPDGVFLSNGPGDPATADAAVELTRGVLGKGLPLFGICFGNQILGRALGRDTYKMKFGHRGINIPVVEHETGRISITAQNHGFALEGERGERFDTPFGTAEVSHVCANDGTVEGVRLVDGRAFSVQYHPEAAAGPHDAAYLFDRFAGLMEGA

>CORE_REP|Org105_Gene4449#

MPDFTESAFKATKATSIYFRYLRNSPMPAVPEARPRRRKVSRVRLLVTGGAGFIGANFVQQTVTERPEVTVTVLDALTYAGNRASLEPVADRIDFVHGDISDLDLVDELVSGVDAVVHFAAESHNDNSLTEPWPFVQTNIVGTYSLLQAVRRHDVRYHHVSTDEVYGDLDAADPAFTEQTAYNPSSPYSATKAASDLLVRAWTRSFGVRATLSNCSNNYGPYQHVEKFIPRQITNLIDGVRPRLYGAGHQIRDWIHVDDHNRAVWDVLERGRIGQTYLIGADGELDNKTVVRLILEAFGRDPDDFDHVTDRPGHDQRYAIDASLLRDELGWRPRYADFRAGLADTIAWYRANEDWWRPHKESTERAYAAAGEKTISPN

>CORE_REP|Org145_Gene4685#

MNTVQVARPPRARGAVALRRSPPEGISAVTQAPVQTDILEIAREQVLERGEGLTQDQTLAVLRLGDDRLEELLGLAHEVRMKWCGPEVEVEGIISLKTGGCPEDCHFCSQSGLFQSPVRAAWLDIPSLVEAAKQTAKTGATEFCIVAAVRGPDARLMAQVAAGVEAIRNEVDIQVACSLGMLTQEQVDQLAAMGVHRYNHNLETAKSHFPNVVTTHTWEERWDTLRMVREAGMEVCCGGILGMGETLEQRAEFAAQLAELEPDEVPLNFLNPRPGTPFGDLEVLPAAEALKAVAAFRLALPRTILRFAGGREITLGDLGAKQGILGGINAVIVGNYLTTLGRPAESDLDLLGELKMPIKALNETL

>CORE_REP|Org216_Gene3268#

MLRRSLLRGLLKCKCVRCRRRHSLVRHRAVRPGRCGGAAARRCGMVVLNPEGPSAQARLLTAACRGVVRPVLRAAPITRATIPVGALAIDGLARLRPHPRGIEREQVTMPGFAMEIIRPAGAARAMRHGALLYLHGGGFAVCGLETHRPVAASLARRTGLPVVNVAYRQLPVRSITESIDDCLAAYRWLLRHGAEPDRIVFAGDSAGGYLTFATALRALECGLPAPAGLVGLSPLLDLDYAAKRDYVNVARDPYIPLSALAAVVRLGAEREGRLDPLLSPVNGALAHLPPVLLVAAEDEVLRFDAELMAARLDAAGVPNSVELWRGQVHAFMSIAPGLPESRAALGRVARFVRGRLADSQRARTA

>CORE_REP|Org4_Gene2114#

MAAHRRQGTLRLDTKRLVGGALAAGVLATTTVYGAGPVGADPVALPATAADAVQRMVDLSRQSEQLNEQALNAQSDLDTKLGLQREADAKLAASTDQVNRARDEVRKYQPIIDRTAIAAYQGARTNRLFAVLVSDSPQQLLDQMSTLDVLAAQTSDQLALYKKATDAAEGAEADARRASDEARAAADKAETVRGELERKRSDLSGAIVQVVQAWTGLSTKDKSALAGPAFPPGFDRDTLLQGLVPGSGTSALAASLTRIGDPYVWGATGPHQFDCSGLVQWAFKQVGKDVPRTSSQQASYGTPVAQNDLQPGDVVFFYNDISHVGIYAGNGLMVHASTFGVPVAVAPISTTPYHSARRY

>CORE_REP|Org13_Gene6761#

MRFGRSAAPVSHSALAPESAPRGRRGAQRGWRTRLLAAGAAMALPIAAGIMAPAAIAAPVHAPVHQTPAGGYDELMVPSSMGPIKVQVQWARNGGNAALLLLDGLRARDDRNAWSFETNAQQMFGNDNVTLVMPVGGQSSWYADWQGPSNTNGQKFTYKWETFLTKELPDFLSNYGVSRTNYAVAGLSMSGPAALRLAAFHRDQFKYAASFSGPLNWNAPGMREAIRVMMLDAGRFNVDSMAAPWSPQWLRSDPMVFAPQLRGLPMYISAASGLPGQYDHPNGLVGAFNTGNAMGIELISMVSTHSFKARLDSLGIPAAYDFPPTGTHAWLYWQDELAKARTGILAALNA

>CORE_REP|Org15_Gene485#

MTSYAPAEALAIEADELVKVFGEQRAVDGVSLAVPQGAVYGVLGPNGAGKTTTIRMLATLLRPDGGRARIFGHDVVAEPTAVRSLIGVTGQYASVDEKLSATENLIIFSRLLGLSRSEAKRRAAELLEEFGLTEAATKALENFSGGMRRRLDLAASLIATPPLLFLDEPTTGLDPRTRAQMWETIRRLVREGATVLLTTQYLDEADQLADRIAVIDHGRVIADGTSDELKGSVGQSALQITVADRDVIERARTLIGEFLSRADGKLVEASISPEAGRVTAPLSDPSVTADLLIRLRDNDIRVDEITVSKPSLDEVFFALTGHAAESDAAESDSAESDSAGSNSEGTAA

>CORE_REP|Org34_Gene2552#

MQLGMIGLGRMGANIVRRIVADGHTAVGYERHAPHIEELGAELGASFSGTTDLAEFVSRLETPRVVWVMIPAGATGAVIDQVAELLEPGDIIIDGGNIRYHEDIQRAERLAPKGIHYVDIGTSGGVFGRTRGFCLMIGGEAGPVRYLDPLLRSIAPGVDAAPRTPGRTGEPSPAEQGYLHCGPAGAGHFVKMVHNGIEYGAMAAYAEGLNILHKADYGAGYDSGAHSAEETPLEHPEYYRYDIDIPEVTEVWRRGSVVASWLLDLTAAALHADPNLDSFGGRVSDSGEGRWTIDAAIDIGVPVPVLSAALFQRFSSRGESHYADKMLSAMRKAFGGHNELPQG

>CORE_REP|Org127_Gene3382#

MSNSGAKKLTPEQLRKLYRPGELTVVHEPAGVSAVTSALRGVGRTVALVPTMGALHEGHLELVRRAKRTNQVVVVSIFVNPLQFGENEDFDKYPRTLDSDVALLREEGVALVFAPSVAQMYPDGPRTSVHPGPLGAELEGASRPTHFAGMLTVVAKLLQIVRPHEAFFGEKDYQQLTLIRQMVRDLNFDVDIVAVPTVRESDGLALSSRNRYLDEQQRELAITLSAALAAGRHAAGRGPDAVLAAARSVLDGATGVDVDYLELRGSDLGPIPSSGNARLLVAARIGATRLIDNVPVSVPPAVSDAASAGPSVVTQAAASGPVAPADGHNAVPDFQPAQADA

>CORE_REP|Org201_Gene1319#

MGDAIVAEGLVKRYGQQVALDGLDLTVPEGTVTALLGPNGAGKTTTVRVLTTLLIPDGGRATVAGIDVLRDPRALRRRIGASGQYAAVDEYLTGFENLEMVGRLYHMGVQRSKERARELLDRFRLSDAADRPVKGYSGGMRRRLDLAGALVAAPPVLFLDEPTTGLDPRARLDLWDVIEELVAGGTTLLLTTQYMEEADRLADSIAVIDRGKVIAKGTADELKTMVGGDRIELTVDHVDNLAIAQQALAGLADGEIHLEPGLRRIIVPVSNGSQALVEAVGRLNDHSVKIHDVGLRRPSLDDVFLTLTGHEAEELINADDAADGLGALEATEGKTR

>CORE_REP|Org134_Gene5162#

MGRVRAIRLNGFGGPEVMEWAETPDPQAGPGEVLIDVAAAGVNRADVMQRKGHYPPPPGASEVPGLECSGVIAAVGDGVRGWSVGDRVCALLSGGGYAERAVAPAGQLLPIPDGLDLGAAAGLPEVAATVWSNLVMTAGLHAGQLVLIHGGGSGIGTHAIQVAKRLGARVAVTAGSAGKLERCRELGADILINYREEDFVAVIRAEQGSGGPGADIILDNMGAAYLARNVEALATYGQLVVIGLQGGVDAELNLAALLGKRAAVRATNLRGRPANGVGSKAEIIAEVREHVWPLVTEGAVVPVIHAELPINEVGDAHALLDSADTVGKVVLHIGDY

>CORE_REP|Org1_Gene5346#

MLDSMIEVRGLTKHYGRTAAVEDLTFTVKPGQVTGFLGPNGAGKSTTMRMILGLDTPTAGTALIDGKPYHQLKQPLRTVGALLDAKWVHPNRSARAHLEWLAASNGIARSRVEEVLRLVGLSEVAGKNAGGYSLGMSQRLGLAGALLGDPKVLLFDEPVNGLDPEGILWIRRFMQRLASEGRTVLVSSHLLSEMAQTAEHLIVIGRGKLIADTPTKEFIERASEQTVRVRSPQLDQLRSLLTSNGMTVREDGTGAEGPALLVAGVTSDAVGKLAGANDITLFELSPQRASLEEAFMRMTGGAVQYHGEGAEAVGVPGPGGPYTAMGGAL

>CORE_REP|Org105_Gene1446#

MRYAAPPLECAQRHARSACGDLGGEHVIDGGERVTGLKTGLEAVLARAHELPSPPARGAVTLRARGVSVDRRGGGAKARRVLAEVDFEVAAGEVVALVGPNGAGKSTLLAALAGELDPTEGSVELDGRPLTQWTPLDMARRRAVLPQSHTVGFPFSAGAVVAMGRAPWQRTALRERDQEIIAASMAATDVTHLAEQAFPTLSGGERARVALARVLAQDTATLLLDEPTAALDLGHQETVLRLADERAAAGAAVVIVLHDLGVAAAYADRVAVLDAGRIAADGPPRDVLTTELLTRVYQYPVEVLDHPVTGAQLVLPVRGGGGE

>CORE_REP|Org113_Gene6136#

MTADAGSDATQAVPPATSCFRTAVVPAAGLGTRFLPATKTVPKELLPVVDTPGIELVAAEAAESGAQRLVIVTSPGKDGVVAHFVEDLVLESTLAERGKFHLLEKVRKAPGLLDVSSVVQEEPLGLGHAVSQAEQVLDDDEDAIAVLLPDDLVLPCGVLDVMTRVRRKRGGSVLCAIDVPKQEVSAYGVFDVVPVPDATNPDVLRVVGMVEKPKLADAPSTFAAAGRYLLDRAIFDALRRIEPGAGGELQLTDAISLLIAEGHPVHVVVHRGSRHDLGNPGGYLRAAVDFALERDEYGPALREWLQRRLAPDWNPQLTSPQ

>CORE_REP|Org24_Gene597#

MATARRGRRRSGRSHRVAARSARRARLTGVTLPSVTPVTGRLVVTGARGQLGRALLDLAPDARGYTHADLDITDLDAVRAALRCGDVVINCAAYTAVDRAETDIDAACAVNARGPMALAVACGEVGARLIHVSTDYVFPGTGSRPYETADPTGPTSVYGKSKLAGERAVADLLPETGHIVRTAWVYTGTGSDFVATMRRLERERETVDVVDDQIGSPTYAPDLAAALVELAEQPDAPRILHAANAGQASWFDLARAVFAGVGADPDRVRPCSTSAFPRPAPRPAYSVLSTASWTAAGLSPLRPWQDALNDALAAASD

>CORE_REP|Org127_Gene4988#

MTDNSERICAGRTVIVTGAGRGIGRAHALAFAAAGANVVVNDLGAELDGAPSADSPAAQVVEEIVQAGGRAVVNGDDVADWAGAKRLIGQAVETFGGLDVVVNNAGIVRDRMLVNLAEDEWDAVIRVHLKGHFATMRHAIEYWRAESKAGRARDARIINTSSGAGLQGSVGQGNYAAAKAGIAALTITAAAEFGRYGVTVNAIAPSARTRMTETVFADMMARPDDGFDAMAPENVSPLVVWLGSPDSAGVTGRMFEVEGGKVALADGWRHGVAEDRGARWQPSELGPVVRELIAKATDPEPVYGA

>CORE_REP|Org81_Gene2585#

MNVGAPDRARWVGENPEYKETATVQRIGVIGGGTMGAGIAEVAARAGGSVLVLERDTEAADAAVARIEKSLGRAVKSGRLEQAAADQARARITLTTAIDDFADRELVIEAAPEIESLKTDFFTKLDGIVSPETILATNTSSIPVIRLANATANPGRVVGVHFFNPVPVLPLVEIVVTLKTDREVADRVTAYARDILGKRTIESKDQAGFIVNALLIPYLCSAIRMYETGFASAEDIDEGMVSGCAHPMGPLRLTDTVGLDVTLAVAESLYAEFGEPQYAPPVLLRRMVDAGYLGRKTGRGFYTY

>CORE_REP|Org66_Gene2436#

MAAAVSVHPRRHDLRRFERSAAQHHRRASARAPSGGSAVSGPLSVAPQPIPGHGLLTGRVAVITAAAGTGIGSATARRLLAEGADVVISDWHERRLGETEVELKGEFPERRVAAIACDVQSTTQVDELVRGAAAALGRIDIMVNNAGLGGETPVVDMTDEQWDRVLDITLNGTFRCTRAALNYFRAAGHGGVIVNNASVLGWRAQYGQAHYAAAKAGVMALTRCSAIEAAELGVRINAVAPSIARHAFLDKVSSSELLDRLSEREAFGRAAEPWEVAATIAMLASDYTTYLTGEVVSISSQRA

>CORE_REP|Org113_Gene136#

MRGIILAGGTGSRLHPITRGVSKQLVPVYDKPMVYYPLSTLMLAGVRDVLVITTPEDAESFRRLLGDGTQFGMSIDYVVQPEPDGLARAFVLGADHIGTDCAALVLGDNIFHGPGLGTRLRRFDGLDGGTVFAYRVSDPSAYGVIEFVGGKAVSIEEKPKLPRSSYAVPGLYFYDNDVVEIARGLRPSARGEYEITDINRTYLEQGRLRVETLARGTAWLDTGTFDSLLDAANYVRTIEERQGLKIGVPEEVAWRMGFIDDEQLSRLAEPLVRSGYGTYLMDLLTRGKNDGTTADEYRDEQDD

>CORE_REP|Org120_Gene2356#

MIGPMKIRKAVIPAAGIGSRLLPLTKAIPKEMLPVGDKPVIEHTVRELVSSGITDITIVVSSGKSLIQDHFRPNPALVAQLRADGKTAYADAVEEVGELSRLGHITYLDQHGPYGNGTPVLNAARNLGDEPMLVLWPDDVFVADVPRAQQLINAYEQTGAPVLALMPMDPTESQRYGVPVVADDQGHGLLRITGLREKPKPEDAPSNYAAIGGYVVTPGVIEELRTQTRAWYEHRTGEVYLTDAINVHAADNPVYGQVIRGRWYDTGNPADYLVAQFASALANPQYGPLLRTLAEDTAS

>CORE_REP|Org49_Gene5817#

MSVGPRGHSDVPATQYEEESVKHIHAGKVRDLYEDGDELILVASDRVSVYDVVLPTPIPEKGALLTQLSNWWFRFFADVPNHLISTTDVPAEFAGRAVRAKKLSMVKVECIARGYLTGSGLAEYRRTGSVSGVALPPGLVEGDKLPEPIFTPTTKADEGHDEFITFDDVVNQEGREVAERLRDLTLDVYARGAEHAASRGVIIADTKLEWGWDGDVLTLGDEVLTSDSSRFWPADEYAPGRPQPSFDKQFVRDWSTSTGWNKEYPGPEIPADIVAATRAKYQQAYELITGETWTGVS

>CORE_REP|Org128_Gene4969#

MRLPRSRVGGHPIHKVDAAREHATLPESSLPIGVSADYELPGAARSDVRTEVEVRPEAEVGPQARAVVANGADFDDTESVAGDAESVAGDAESVAGDAADDALSGTAAFDATGDRTMMPSWDELVREHADRVYRLAYRLTGDPQDAEDLTQETFIRVFRSLQNYQPGTFEGWLHRITTNLFLDMVRRRNRIRMEALPEDYDRVPSEGPGPEQVYHDARLDPDLQRALDALAPEFRAAVVLCDIEGLSYEEIGATLGVKLGTVRSRIHRGRQALREYLAHNGSQQRFAAEEKVG

>CORE_REP|Org102_Gene5292#

MPDNPAPANLTPAEPTAADRAPADSAPLNLAAGAPAPGNPAQPVAFVTGAARGIGAAIAQRLAADGATVAVVDLDENSCAAAVDTIVAAGGKAIAVACDVTAEDQVDAAVDRVAAELGSLDILVNNAGVLRDNLLFKMSVAEWDTVMSVHLRGAFLCSRAAQRHMVAQRSGKIVNTSSVSALGNRGQANYSAAKMGIQGFTRTLAMELGPYGINVNAVAPGFIVTEMTAATAARLGVSSEELQAKTAEITPLRRVGQPADIADVVAFLASENAAFVTGQTIYVDGGRRL

>CORE_REP|Org43_Gene3246#

MSRVSIDTHQAWVEFPIFDAKSRSLKKAFLGKAGGAIGRNQSDVVVVEALRDINLSLREGDRIGLVGHNGAGKSTLLRLLSGIYEPSRGSARIRGRVAPVFDLGVGMDPEISGYENIIIRGLFLGQTRKQMMSKIDEIADFTELGEYLHMPLRTYSTGMRVRLAMGVVTSIDPEILLLDEGIGAVDAEFMKKARLRLQELVARSGILVFASHSNEFLAQLCDSALWIDHGQIRLRGGIEEVVRAYEGPDAGNHVATVLREMAAERAGRAEGSADERELEQNAT

>CORE_REP|Org5_Gene5353#

MTDAADATPGAPGTGNTAEPDATTAAGASARTDAAPPMISMRNVDKHFGDLHVLRDVNLEVPRGQVVIVLGPSGSGKSTLCRTINRLEPIDSGTIAVDGVELPAEGRALAKLRADVGMVFQSFNLFAHKTILDNVLLGPVKVRRVDKKRARARAMELLERVGIADQADKYPAQLSGGQQQRVAIARALAMDPKVMLFDEPTSALDPEMVNEVLDVMVALAKEGMTMLVVTHEMGFARRAGDRVLFMADGRIVEDAPPETFFTAPASERARDFLGKILSH

>CORE_REP|Org19_Gene2600#

MRNPLATPTGCGCPARHRRRREQHCAARSRRRPGAGVPQDTGGVVTSAERPPAATRVLVVDDEPQILRALRINLSVRGYEVITAATGAAALRAAAEKHPDVVVLDLGLPDIDGVEVLAGIRGWSSMPVIVLSARTDSSDKVQALDTGADDYVTKPFGMDELLARLRAAVRRSASTAEESAPIVETSSFTVDLAAKKVIRGGRDVHLTPTEWGVLEMLVRNQGKLVGRRELLREVWGPTYATETHYLRVYLAQLRRKLEDDPSQPKHLLTEAGMGYRFQA

>CORE_REP|Org120_Gene7219#

MGAVILRRDSSTATRPNPAGSVATSTVRPPSQHPSGARPPAIPAELVPNHVALVMDGNGRWAQERGLPRTAGHERGEAVLMDTVEGCIEMGVKWLSAYAFSTENWRRSPDEVRFLMGFNRDVIRRRRDEMNEMGVRVRWAGRRPRLWRSVINELEIAEEMTKHNTVMTLTMCVNYGGRAEIADAAREIARRVAAGEIDPEKVTEATVARFLDEPDMPDVDLFLRPSGEFRSSNFLIWQSAYAEFVYQDTLFPDFDRRNLWAACLEYASRDRRFGGTK

>CORE_REP|Org103_Gene1084#

MTDLTAAFAASVACGAMSSDLLGKSALVSGASRGIGKAVAAELLRRGANVLITARKPEPLAEAAAELRALGHQGEVATIAGNSGDAQARAEAVGRAVTEFGSLDILINNTGINPVFGALMDADLDAVRKIFDVNVVAALGYAQEAYKAWMGEHGGAIVNVASVAGLRSTGVIAAYGASKAALIRLTEELAWQLGPKIRVNAVAPGVVKTKFADALYSADEERAASVYPMKRLGSPEDVARLIGFLASDEAAWITGETVRVDGGLLATGGI

>CORE_REP|Org151_Gene5632#

MPVGRRVARVELPVQVRGLPYRIYEARLTKQLAGKQHPRHVAVMCDGNRRWARENGFADVSHGHRVGAVKIAELVGWCQAEGIEMVTVYLLSTENLQRDPDELETLFEVITDVVEELSAPEQNWSVRVVGSLDGFPELIAKRIRTAAERTEDRNGVHVNVAIGYGGRQEITDAVRSLVRQEIAAGETGEDLVQSITVNAIGQHLYTSGQPDPDLVIRTSGEQRLSGFLLWQSAYSEIWFTEAYWPEFRRVDFLRALRDYAARHRRFGI

>CORE_REP|Org77_Gene3533#

MALEIDLSGRVVLVTGGVRGVGAGVSRALLAAGATVLACARRPGDAPVEYEGRQAEFLPCDVRDGDAVRELIDTVIARHGRLDHLVNNAGGAPFALAADASAKFHAKIVELNLLAPLLVSQLANAVMQAQPDGGTIVNISSVSAHRPSPGTAAYGAAKAGVDSLTASLAVEWAPKVRVNSVVVGPVETELSLLHYGDADGVAAVGATIPLGRLARPEDVGRCVAFLASPLAGYVSGATLEVHGGGERPAFLDAATVNTAAPNGAPKP

>CORE_REP|Org63_Gene4595#

MILDRFRIDDQVAIVTGAGRGLGAAIAVAFAEAGADVVIAARTESQLEEVAERVAAAGRQAHVVPADLSDADATAALAASAVERFGRLDIVVNNVGGALPCPLLDTTPQALAQAFDFNVVNAHALVRAAVPRMLETAGGGSILNITSTMGRLPGRAFAAYGTAKAALAHYTKLAALDLNPRIRVNAIAPGSILTSALEIVASNDAMRTELEAKTPLHRIGEPEDIAAAALYLVSPAGKYLTGKILEPDGGLIIPNLDLPIPDLT

>CORE_REP|Org15_Gene4889#

MSDGTGLLADKVVVISGVGPGLGRSLCVQAAAAGAKVVLAARTESRLREVADEIDGAGGTSLIVPTDITDDAAVANLVERTVATFGRVDALINNAFAMPSMKSLARTDFQQISDSLELTVLGTLRATQAFTDELAKTRGAVVMINSSVLRHSEPRYGSYKVAKSALLAMSQTLATELGAKGIRVNSVAPGYIWADRLKWYFGEVAKKYGITVEQVYEQTASRSDLKRLPEPDEIARAVVFLASEWASAITGQTLDVNCGEYHA

>CORE_REP|Org37_Gene1288#

MPAPGRRSRRGPASARRIRGHGPAAPGHDGNGEPVSGRADSDGDTPIRVLLVDDEQLVRSGFRLLLDIEDDITVVGEAANGAEAVRKARALRPDVVLMDIRMPTMDGIQATREIAATTGLQDVRILILTTYDTDAYVFEGLQAGASGFLLKDAGPAELLHAIRVVAAGEALLAPRITRRLIAQFTARRAADRAAEQRLAVLTDREREVLALVGQGMSNAEIGAELFLSPATARTHVSRAMVKLGARDRAQLVVIAYRTGLVAP

>CORE_REP|Org117_Gene417#

MTVAVRVIPCLDVDAGRVVKGVNFQNLRDAGDPVELAATYDAQGADELTFLDVTASTGDRGTMIDVVTRTAEQIFIPLTVGGGVRTVEDVDRLLRAGADKVSVNTAAIARPEVLREMSERFGSQCIVLSVDARTVPDGQPDTPSGWEVTTHGGKRGTGIDAVEWAERGAELGVGEILLNSMDADGTKTGFDLPMIRAVRAAVSIPVIASGGAGAVEHFAPAVQAGADAVLAASVFHFGDLTIGQVKDSLRDAHLVVR

>CORE_REP|Org114_Gene5983#

MLELTDVTKEYRVGEQTVRALDGISLRIEPGEFTAIIGPSGSGKSTLLHMLGALDSPDSGSIRFQDAEIGGLDDDRQSEFRRHRVGFVFQFFNLLPTLSAWENVAIPKLLDGTGLRKAKPRALELLELVGLADRAEHRPAELSGGQMQRVAVARALIMDPPLILADEPTGNLDSKTGASILELLGDITRQGNSVVMVTHDMGAVRYCDRLITLRDGKIGSNELVEHTENGEVRTVPVELTASLSEDGSEPAQAVRP

>CORE_REP|Org35_Gene4389#

MSNSVEDSRKSESQNAERASRSVLVTGGNRGIGLAVAQRLLADGHKVAVTHRGSGVPDGLFGVKCDVTDSESVDRAFSEVEAHQGPVEVLVANAGITDDTLLMRMTEEQFTRVIDANLTGAFRCAKRANRAMLRARWGRMIFLGSVVGLGGGPGQINYASSKAGVIGLARSVTRELGSRNITANVVAPGFIETDMTAELPEEMRETAKKFIPLQRLGAPEEVAAVISFLASEDSRYVSGAVIPVDGGMGMGH

>CORE_REP|Org2_Gene6607#

MNSLTPAVSLLTSNNDGVNTTSSSVPAASVLVAEDDPHVRSTLDQLLRFEGYQVYLAADGQEALELLAQQRPDLAVVDVEMPRLDGLSLCRLLRRRGDRLPILVLTARQQIGDRVAGLDAGADDYLPKPFATDELLARLRALLRRSTFDEDDDTVLAVGDLTLNTATRQVHRGDRPIELTKTEFDVLELLLRNARIVLSRSRIYEHIWGFDFDTESRSLDVYIGYLRRKTEENGEPRLIHTVRNVGYSVRPA

>CORE_REP|Org45_Gene4290#

MSGHSKWATTKHKKAALDAKRGKLFAKLIKNIEVAARTGGGDPDGNPTLYDAIQKARKNSVPLDNIERARKRGGGEEAGGADWQTIMYEGYGPSGVAVLVECLTDNRNRAAGEVRVAVTRNGGNMADPGSVSYLFSRKGVVTLEKNGLSEDDVLMAVLDAGAEEVNDLGEEFEIISEPSDLVAVRSALQGAGIDYNSAESGFQPSVSVPADADLAKKVFKLVDALEDCDDVQNVYTNIDVSDEVLAQLDAE

>CORE_REP|Org5_Gene560#

MSRMNGVAGDRIPEARVLVVDDEPMIVELLSVSLRYQGFEVAAAGNGAEGLDRAKQFRPDALIVDVMMPGMDGFGLLRRLRADGIDAPVLFLTARDEVDDKITGLTLGADDYVTKPFSLEEVVARLRVILRRSGHVVEETKSSRIRFEDIELDDDTHEVWKAGEPVALSPTEFTLLRYFMVNAGTVLSKPRILDHVWRYDFGGEVGVVETYVSYLRKKVDTGPDRLIHTLRGVGYVMRAPSRSRSSAK

>CORE_REP|Org31_Gene655#

MVPLPDAYRTSELVSTPKVLVVDDDEDVLASVERGLRLSGFHVLVARDGAQALRSVSEHAPDAIVLDMNMPVLDGAGVVTALRAMGNEVPICVLSARASVDERISGLESGADDYLVKPFVLAELVARIRALLRRRTDTPPAATPGAITVGPLEVDIAGYRAVLHGNEIELTKREFELLSTLARNVGVVLSRERLLELVWGYDFAADTNVVDVFVGYLRRKLEVDGAPRLLHTIRGVGFVLRAPK

>CORE_REP|Org114_Gene4108#

MSANLMIVEDDDRVRVALRLAMEDEGYDVAEAEEAEVALRQLRDNGAPDFMIVDLMLGGMDGFTCIREIRRDHDVPIIVVSARDDTHDVVAALEAGADDFVTKPFEVKEITARMRAVARRARFAEQAAAEEDPDSELGTMVLDEQAGNPLVLSTESGIVRRGDEEIHLTLTEYRLLCELAGSAGRVLSRGTLLERVWDRGFFGDERIVDVHIRRLRTKIERDASDPQLIVTVRGLGYRLDVQR

>CORE_REP|Org102_Gene4053#

MHDEGVKQVKDLVDTTEMYLRTIYDLEEEGVTPLRARIAERLEQSGPTVSQTVARMERDGLLTVAGDRHLELTEKGRAMAVAVMRKHRLAERLLVDIIGLDWQNVHAEACRWEHVMSEEVERRLVEVLNHPTTSPYGNPIPGLDELGVTGTSNAEEKLVRLSDLPSGQSAAVVVRRLSEHIQTDPEIINQLREAGVVPDARVNVETKPGAVVILVPGHAGFELSDEMAHAVQVKLV

>CORE_REP|Org44_Gene3372#

MTSVLIVEDEESLADPLAFLLRKEGFEVTVVGDGPSALAEFDRSGADIVLLDLMLPGMSGTDVCKQLRTRSGVPVIMVTARDSEIDKVVGLELGADDYVTKPYSARELIARIRAVLRRGAGDELDGNGESGVLEAGPVRMDVDRHTVMVNGKPVTLPLKEFDLLEYLLRNSGRVLTRGQLIDRVWGADYVGDTKTLDVHVKRLRSKIEADPAKPEHLVTVRGLGYKLEA

>CORE_REP|Org96_Gene3994#

MITMRNVTKSYKTSTRPALDNITVDVDKGEFVFIIGPSGSGKSTFMRLLLKEESPTAGEIRVADFRVDRLPGRKVPKLRQRMGCVFQDFRLLQQKTVQENVAFALEVIGKRRQVIERTVPEVLDMVGLGGKADRLPSELSGGEQQRVAIARAFVNRPLVLLADEPTGNLDPDTSGEIMLLLERINRTGTTVLMATHDNHIVDAMRRRVVELDHGRLVRDEATGVYGVGR

>CORE_REP|Org102_Gene5430#

MNTTSRGDSVDGGGRVTNGRTKRLALLDYGSGNLHSAERALVRAGAEVTVTADPDIALNADGLVVPGVGAFAACMAGLQEVRGERIIGKRLAGGRPVLGICVGMQILFERGVEHGIETAGCAEWPGTVERLDAPVLPHMGWNTVRAPEDSVLFAGMDADTRFYFVHSYAAQKWEWNGDGTIAPAKLTWAEHGVPFLAAVENGPLSATQFHPEKSGDAGAQLLRNWVRSL

>CORE_REP|Org2_Gene5611#

MSTPTYARPAVPMIAPSILSADFAHLADEARAVEGADWLHVDVMDAHFVPNLTLGLPVVESLLKATDIPLDCHLMIEDPGRWAPPYAEAGAYNVTFHAEATDDPIAVARDIRAAGAKAGLSVKPNTPIEPYLEILREFDTLLVMSVEPGFGGQSFIADVLDKARTVRRLVDAGELRLLVEIDGGINANTIEAAAEAGIDCFVAGSAVYNTADPAATVQALRKQAAAHR

>CORE_REP|Org134_Gene5164#

MVESAREVREPAARQGRDIVAEYTLPDLDYDYSALEPHISGQINEIHHSKHHAAYVAGVNTALEKLEAAREAGDHGAIFLNEKNLAFHLGGHVNHSIWWKNLSPNGGDKPVGELAAAIDDQFGSFDKFRAQFTAAANGLQGSGWAVLGYDTLGQKLLTFQLYDQQANVPLGIIPLLQVDMWEHAFYLQYKNVKADYVTAFWNVVNWADVQERFARAVSEGKGLIFG

>CORE_REP|Org45_Gene5167#

MSNMFDPRAAGITFAAAGSSLPQARYILPSYTEQTSFGVKETNPYTKLFEERIIFLGAPIDDTSANDIMAQLLVLESLDPDRDITMYINSPGGSPVSLMAIYDTMQYVRADVATVCLGQAASAAAVLLAAGAPGKRAALPNARVLIHQPYTQGGFQGQVSDLEIQAAEIERTRSLLDTILARHTGKEAEIIRRDTDRDKILTADEAKDYGIVDTVFEYRKLSAQR

>CORE_REP|Org7_Gene3709#

MTAVLLAEDDEAIAAPLSRALGREGYSVTVERFGPAVLERALEGHHDLLILDLGLPGMDGLEVCRQVRASGADIAVLMLTARTDEVDFVVGLDAGADDYVGKPFRLAELLARVRALLRRSGIGDDTVEVGGIRLEPAARRVLVNGAEIGLANKEYELLKVLIDRAGQVVPRETILREVWGDAELRGSKTLDMHMSWLRRKIGDEGPMAERRIVTVRGVGFRLNTD

>CORE_REP|Org4_Gene7730#

MAVGQRAGMRRQVSVTSNQAGIAMTSATAGLNLSDSVYERLLRERIIFLGTQVDDDIANKICAQILLLTAEDPTKDISLYINSPGGSVTAGMAIYDTMQFAECDIRTVGMGLAASMGQFLLTAGTKGKRLALPHARIMMHQPSAGIGGSAADIAIMAEQFAHTKRELNELQAQHTGKSVEQVTADADRDRWFTAKEALEYGFIDRVVSHANQTGGVSDN

>CORE_REP|Org1_Gene1099#

MTMARVLVASRNAKKLAELRRILDDAGVAGVQIVGLDDVPPYDEAPETGATFEENALAKARDGAAATGLPCVADDSGLAVDALNGMPGVLSARWSGTHGDDAANNALLLAQLRDVPDERRGARFVSACALVVPGGTETVVRGEWPGTIGRKPMGEGGFGYDPLFVPDGGDVTAAQLTPAAKDAASHRGRALRHLLPALAALADRTE

>CORE_REP|Org152_Gene5548#

MTVEIRELSVPGAWEFTPRLHGDARGVFLEQFKASEFEKAVGRPFDLQQVNVSTSAAGVLRGIHYTANPPGQAKYVTCVRGAFLDVVVDLRPDSPTFGRWDAVVIDDVTRRSVFLAEGLGHALLSLADDSTVTYLCSLEYTPEFDAEVDAFDPAIGIEWPTMGRDGQPLTVIRSAKDAAAPPLSDARLLY

>CORE_REP|Org151_Gene30#

MLRTMMKSKIHRATVTHADLHYVGSVTVDQDLLDAADLLEGEQVCIVDIDNGARLETYVIAGERGSGVIGINGAAAHLVHPGDLVILIAYGQMNEQEIAEYDPKVVFVDERNRPVELGSDPAHAPEGSGLTSPRSLSFAG
