# Supplementary material for: Design of a multi-epitope vaccine against six Nocardia species based on reverse vaccinology combined with immunoinformatics
Source: Front Immunol. 2023 Feb 2;14:1100188. doi: 10.3389/fimmu.2023.1100188 (PMC9952739; doi:10.3389/fimmu.2023.1100188)
Supplement: Supplementary file 12 [file Table_5.docx]

>CORE_REP|Org63_Gene886#

MRGSQIALRTVPDAATAVLTKPPAAPVVTRNDFRTARLIALVAGLLGALFALATPFLPVTQTTAVLNWPQGGTLGNVQAPLMSQVPIDLKATIPCETIAQLPERGGMLLATAPPQGDRAALEAMFVRVSETSVDVVDRNAVVVSADRSRMGECAALSISSDSERTYAVFTGLTKQVERPVEGGAPGATELATVPVEGQLGGDLRPQVVGVFSDLKGAAPAGLAFDMTVDTRFSSSPTAIKLVAMIAAVLCTLIALAALARLDGSDGRGHRRFLPANWLKPTWADGAVAGTLLLWHFAGANTSDDGYILSMVRVAPHAGYMANYFRWYGVPEAPFGWYYYVIQVFSEISTASPWVRLPALACAILCWLVISREVVPRLGRGVRTSKVALWTGGLVFLAFWLPFDNGLRSEPIVALGALLTWVSIERAIATGRLLPAAVAILVAAFTLAAAPTGLMCVAALLAGIRPLVRIVVRKHRQFAALGAGRWGSTLPLLAPIAAAGVLVLTVVYSDQTFAGIQEANRVRQVTGPNLAWYEDYLRYYYLFVETVDGSLSRRFAFLVMLLCLFTTMLVLLRRRQVPGIASGPTWRLMGVVFGTIFFMMFNPTKWTHHFGAYAGIAGSLAAVTAVAVSASALRARKNRAIFLAGLLFVLAVAFSGINGYWYVSSFGVPWFDKRISLQGYQSNTVMLMLFGLALALVGWYALREDYTKPQPSAKTARGRRIRRFAAIPLTVVAALMVALEVLSLVKGAVSQYPAYSLARSNIDALGGSTCGLANDVLVEPDPNGGRLEPIIDPARPLTDPLAGVDSVGFDPNGVPNDLSADSVEVKPGTGNTSTQSVGAAFAEGQSAGTGGGQGALGVNGSTVALPFGLDPASTPILGSYQNGMQQPANVTSSWYQLPARSADKPLVVISAAGRILSFDDTGAMKYGQSLTVDYGKHLPDGTVQKLGTYLPRDIGPFPSWRNLRVPLDEIAPDADAVRIVANDPILIGDQWLAFTPPRMPKLQTLNSLLGSQQPILLDWAVGLQFPCQRPFDHENGVAEVPGYRILPDRPLAISSTNTWQAEEFGGPLGFAQMLAKSTTVPTYLKHDWARDWGSLERYDQYDRNAVPAKLDTGTTTRSGLWSPGNLRVF

>CORE_REP|Org19_Gene3335#

MADETSTRNAYPRVDFTAADAGSRQGASVSFPELERRVLDYWAADDTFRASIDNRAEGCDEFVFYDGPPFANGLPHYGHLLTGYVKDLVPRFQTMRGKRVERRFGWDTHGLPAEIEAEKQLGITDKSQIDAMGLAEFNAACKSSVLRYTNEWRDYVTRQARWVDFDNDYKTLDLDFMESVMWAFKSLYDKGLVYQGFRVLPYSWYEQTPLSNQEARLDDAYRMRQDPAVTVDMLLEVAADHPLHELDGANALIWTTTPWTLPSNLAIAVHPDITYAHVRGKDGKRYVLATERVSHYARELGTADDGLEVLSEHSGAALAGLRYRPPFDFFLGHPHAHRVLNADYVTTDSGTGVVHLAPAFGEEDMDVASANGIEVVQPLDAGGKFTSMVPPYEGLMVFDANPVIIKDLKAAGKLLRHETIEHSYPHSWRSGQPLIYMAVPSWFVAVTKFRDRMVELNQQITWVPEHIRDGQFGKWLENARDWNISRNRYWGAPIPVWVSDDPAYPRVDVYGSLDELERDFGVRPADLHRPGIDELVRPNPDDPTGKSMMRRTPEVLDCWFESGSMPYAQVHYPFENKEWFDGGAVTSDTAGTSVARAHSPGDFIVEYNGQTRGWFYNLHVLSTALFDRPAFKSVVAHGIVLGDDGLKMSKSKGNYPDVNEVFDRDGSDAMRWFLMASPVLRGGNLIVTERGIREGVSHALRPLWNAWTFLQLYASKPGVWRTDSAHVLDRYILAKLAAARDVMTEALEVYDIATACDELRSFADALTNWYVRRSRSRFWEEDRDAIDTLHTVLEVTCRLAAPLLPLITEVIWRGLTGERSVHLTDWPGETELPRDPELVSAMDEVRSVCSTVLSLRKAQNLRVRLPLSEVTIAAPDAERLRPFADLVADEVNVKKVDLTTDVAAHGRFELVVNARAAGPRLGKDVQHVIKAVKSGQWREDDGVVKALVPGHDDGIALLPEEYTQRLVAAEPESTAALPGNAGLVVLNSEVTEELEAEGWARDLIRDLQETRKSLGLDVSDRITVVLEVPQERAEWARTHRDLIAGEILATALDFGAAGAGAADVVGGVRAAIAKA

>CORE_REP|Org125_Gene1111#

MEIQAVTSPYDDGPNGGRPPRGPQSGPGGQPPRPAGGNPPGARPLPPRRQAPPPGPGGPRGGQPGGPPNPAGGPPRRPGPPPGGDRTPPMRGPAGGPPRTGGQPTVRGGQPNPAGGPPRRSANPAPRPGAGATQKIAKPGEQKPQATQKIAAGTLGEAMAQRGPRSTAANRSAPGGAGTRSGSGPGTGGRRAVAGGTPPSGPPPRKGNGGGDGPGGSAGKGPKTKKKSPWRIVRRVIYVLVALAIVVPSAVFLIAYTTVSIPQPGDLKTPQVATILASDGTTQISKIVPPEGNRTDVTIDQIPPHVRNAVIAAEDRDFYSNPGFSISGFARAARDNLMGKDTAGGGSTITQQYVKNAMVGNQHSLSRKMRELVISAKMARQWSKDDILTAYLNTIPFGRGTFGIDAAAKAYFGKSVEQLTVEEGAMLAATINQPYGLDPENNPKGAEQRWNYVLDGMVKAGSVPAAERAKMVYPKVLPSSANNDDSESKTAGPNGLIKRQVLSELSEAGISDTQLNTEGLQITTTIDQKAQQAAIDSVHKNMQGERDEVRTAVVSVDPKSGAVRAYYGGDNATGWDFANAGLQSGSTFKVFGLAENLELGKPLSTMYDSSDLTVNGIKITNAEGETCGTCTIAEALKRSLNTSFYRMELDMPDGPAKIAAMAHRMGIPDTIPGVGQTLTEPDGSGPNNGIILGQYQVRPLDMASAYATIAASGVYHKPHFVQKVVTADGQVLLDRGQVAGEQRISAAVADNLASAMQPIAASSRNHGLAGGRPSGSKTGTTQLGDTGQNKDAWMIGFTPSLSTAVWVGTADGVALKTPGGSIMYGSGLPSDIWKDTMDGALEGTPKENFPKPAAIGGQAGVPSYSAPYTAPTTTQQEYQPPVVVKPSQVEILPGITIPVPGIQPNPRSQPQQNQPQSQDTGPLPGQPVAPADGSSPSTSNSGDTSGNSRSQRPGAGVGNSTDGTGDGYTNSHR

>CORE_REP|Org144_Gene202#

MTDTTLPPFGGSGGDRIDPVDIQQEMQNSYIDYAMSVIVGRALPEVRDGLKPVHRRVLYAMYDNGYRPDRGYVKSARPVAETMGNYHPHGDASIYDTLVRMAQPWSLRYPLVDGQGNFGSRGNDGAAAMRYTECRLTPLAMEMLREIDHETVDFIPNYDGRSQEPTVLPSRVPALLMNGSNGIAVGMATNIPPHNLTELAEAIYWALDNHDADEEATLAACMERVKGPDFPTHGLIVGSQGIHDAYTTGRGSIRMRGVVEIEEDNKGRTTLVITELPYQVNTDNFINSIAEQVRDGKIAGISDIHDESSDRAGMRIVVTVKRDAVAKVVLNNLYKHTQLQTSFGANMLSIVDGVPRTLRLDQMIRLYVKHQLDVIVRRTKYLLRKAEERAHILRGLVKALDALDEVIALIRRSANTDTARTGLMQLLDIDEIQATAILDMQLRRLSALERQKIIDELAKIELEIADLKDILAKEERQRAIVRDELAEIVEKYGDDRRTRIIAADGDVADEDLIAREDVVVTITETGYAKRTKTDLYRSQKRGGKGVQGAGLKQDDLVKHFFISSTHDWLLFFTNKGRVYRAKAYELPEANRTARGQHVANLLAFQPDEKIAQIIQIKNYEVAPYLVLATKNGLVKKSKLSDFDSNRSGGIVAVNLRDEDELVGAVLCSADDDLLLVSALGQSIRFSATDEALRPMGRATSGVQGMRFNASDELLSLNVVRPDTYLLVATAGGYAKRTAIEEYTPQGRGGKGVLTVQYDPKRGTLVGALIVEDDDELYAITSGGGVIRTVAKQVRKAGRQTKGVRLMNLGEGDTLLAIARNADEPDPDLLAGDTSDTGSSE

>CORE_REP|Org97_Gene925#

MGRAQDRARIFWYARPCGSLATSCHGAPPTAHRACDGGPIRLGRVPISQTLARLAGACVLAAVLVAGLLFPLAGGFGYMSNRAADAVDNVSAELVAGTAPAVSTMVDATGAPIAWLYEQRRFEVPSDKIANDMKLAIVSIEDKRFAEHGGVDWQGTLRAFLTNTSSGEVQQGASTIDQQYVKNFQLLVVAKTDAERRAAIETTPARKLREIRMALTLEKELTKDEILTRYLNLVPFGNGSYGIQDAAQTYFGVDAKDLKVAQAAMLAGMVQSSSKLNPYTNPKGVLERRNTVLDTLIQNIPSRADEFRAAKEQPLGVLPEPKGLPRGCIAAGDRGYFCDYALQYLANAGISKDQMDKGGYLIRTTLDPAVQNSVKAAVTANTDPNLENIAEVTSIIAPGQDSHHILAMTSSRTYGLDQGAHQTVQPQPYSMVGDGAGSIFKIFTTAAAMEKGLGTSAQLDVPSFFAAKGMGNGGAAGCPPATYCVKNAGNYRSPMSVTEALAQSPNTAFVKLIQDVGVTPTVDMAVRLGMRSYAEAGTSGHGNQSLADMIKQQNLGSFTLGPVAINPLELSNVAATLASGGKWCPPSPIAEVIDRDGKQVPLTQQACEQVVEPGLANTLANALSQDAVGGTAAGSARAVGWNAPVSAKTGTTETHRSSAFLGFTNSMAGAAYIYGDSPTPGEICSFPLRTCGDGNLYGGNEPARSWFGGIKPVLDKFPPPALPPLDDKYVRGSNNAQIPDVNGMSESEARSVLIGAGFQVSTVTTPGSAAKGTVTATTPNGSAIPGSVITVLVSDGTQREIPKPGPPPAPPVLPGLPQIPRLPPIPIPIPR

>CORE_REP|Org141_Gene5255#

MVSGSLLLCGIDLTVLHVAVPSVSRDLRPSAAQLLWIVDVYSLALAAMLVTCGTLGDRVGRRRMVLSGFLTFGLASAACALSTSTAQLIAARAALGVGAAMIMASTVAIIRVVFTDGRERAFAIGVWTSAHSVGATIGPLVGGLVAERWGWNAVFLVNIPVIIVILAVGARVIPESKNPAPRRWDLASVALSIAGLASVVYALKQAGEHAGVSTAILVTALSGAALLYAFVHRQRRLAEPLLDLSLFADRRFATAAVCVIGCFGSYVALLFFLTQWLQQVGGYSPLHAGLALMPLAAANAVGAVTAPRTASRWGNRGALTAALLLFALAYAVIAAVGDTAHYGTILPALLAAGYGAGIVMTLGADAIMSAAQPERSGEAAAIQETSFELGAGLGVAVLGTVMTVVYRTGMPHVPGLGPDERVIVGESFTAAQDLTAHLPSATADAVLDAARQSYDHGFTTIAVIATVTLVITAAMAAVLLRCKQNEPRNYRFQGGQASVPDTTHPPVTVLGLGAMGQAFVATLLKGGRTVTIWNRTPGKDAELVTAGARTTAAVDEAVTASPVIIAVLLDHRSVHSTLDPIADQLAGRQLINVTSTTAEESRELAFWAAGHGIEYLDGGIMAAPSMIGQPGASILYSGSRAVFDDHRGTLDLLASAEYFGTDAGMASMLDFSLLSAMYGMYGGFFNGVAMTRSVGVSAEAYAERAAAWVKAMTDYLPMLGKLIDARDYENGVQDIAFHKAAVDAIVRATRDAGAAPDFLAPLQHLIDRQIAEGNSALAFEHTVEEIV

>CORE_REP|Org79_Gene341#

MAQEVLKDLNKVRNIGIMAHIDAGKTTTTERILFYTGINRKVGETHDGGATTDWMEQEKERGITITSAAVTCFWNNNQINIIDTPGHVDFTVEVERSLRVLDGAVAVFDGKEGVEPQSEQVWRQAAKYDVPRICFVNKMDKLGADFYYTVGTIVDRLGAKPLVMQLPIGAEDDFDGVVDLIDMKALLWPGKVETGTPPQIQEIPEDLKEKAEEYREKLLETVAESDEELMEKYFGGEELTKEEIQAAIRKLTIASEVYPVFCGTAYRNKGIEPILDAVVSYLPSPIDIGEVHGTSVDGEEDLTRKPSVEEPFSALAFKIAVHPFFGKLTYVRVYSGQAIPGEQMLNSTKSKKERVGKLFQMHANKENPVEHADAGNIYAFIGLKETTTGDTLCNPDHPIILESMDFPDPVIQVAIEPKTKADQEKLGTAIQKLAEEDPTFTVQLDEETGQTVIGGMGELHLDVLVDRMKREFKVEANIGSPQVAYRETIRKKVESLDYTHKKQTGGSGQFAKVIVTIEPYSPDPEELEEGESASYKFENAVTGGRVPKEYIPSVDAGIQDAMQYGFLAGFPLVNIKATLEDGAYHDVDSSEMAFKLAGSQVLKEAVAKAKPVLLEPVMAVEVVTPEEYMGTVNGDISSRRGQVFAMEDRSGAKVVKAKVPLSEMFGYIGDLRSSTAGRANFTMVFDSYAEVPQSVAQEIIDERNGNK

>CORE_REP|Org112_Gene6280#

MSRGRDSGRDRAENGRGRGRFGGGRGNSGGGRASSGKRAASARSAAGPARARKPSRPRPAPGLDASTRFRFGVGRIVMLVALLVAALQLLWIQSVSAPRLSAEAASQRTVHQIDAATRGPILDRNGKSLAFTVNAKALTFQPVRVRKDLQEAHDENSAKPEPDQRMQAIAKYIHDKLGTAAPEQDLLKKLRSDEPFVYLVRNVDPRVAADISLKFPEVGTERQDLREYPGGSLAANVIGATGWDGHGQIGLESALDAILAGTDGSHTYDRGSDGAVIPGSWRDRQPAVNGYGVELTLDSDLQYYVQQQTQQAKELSGAQAASAVVLDARTGQVLAMANDSTFNPALGPQHWSSSSLGNPSVQEVYEPGSVNKIVTAAAAIEYGLTTPDEVLQVPGNIFMGGVTVNDAWQHGVMPFTTTGIFGKSSNVGTLMLAQRIGEDRYYDMLQKFGLGQRTGVGLPGESAGVVPSREQWSGSTFANLPIGQGLSMTTLQMTAMYQAIANDGVRVPPRIVKSKIDPDGNRTEEEPPEGVRVVSPETAATLREMFQAVVQRDPMGVQMGTGVPAAVEGYQVAGKTGTAQQIDPGCRCYSTSSYWITFAGMAPADNPRYVIGLMLDAPVRSSDGSGGQSAAPLFHAIASWALQRDRVPPSPPAKPLILQAS

>CORE_REP|Org4_Gene3693#

MRTRSVPWLRAVSVPPPRSPEGSMSNLINLEQVSKSFGITPLLDNVSLGVHAGERIGVVGLNGGGKTTLLEVLTGLEPPDSGRVSRVGGLRLAVVTQRGVLPAGATVGSVVLAGLADDLNGVAGGPDEVAEHEWAANPRIRSVLEGIGIAGLGLQTSIDNLSGGERRRVALAAALVRDLDLLVLDEPTNHLDVEGVQWLAAHLLERRSALVVVTHDRWFLDTVATDTWEVVGGKVESYEGGYGDWIFARAERARQADASEARRSNLARKELAWLRRGAKARTSKPRYRVEAAEALIADVPPPRDSVSLAAFARKRLGRVVIELEDTTLTTPDGRELVRDLTWRLAPGERVGLVGVNGSGKTTLLRTLAGDTEPAAGKRIQGQTVQIGWLRQELDDLPTDMRVLEAVQQVAQRIMLGDKEISAGQLAERLGFSPARQRTPVGDLSGGERRRLQLTRILMAEPNVLLLDEPTNDLDIDTLQQLEDLLDNWAGTLVVISHDRYLIERICDTTWALFGDGKLTNLPGGIDEYLKKRAAQGQSATRAADKPTGPVTDAAAQRAARKELSRLERAIEKFDEREQRLHTALADAAIDPDKLVTLNAELKQVVADKEAAEERWMELAEDV

>CORE_REP|Org145_Gene4689#

MGGSCACPVLTLPVRNHCRSERCRFVRLPVRETGRAESAQHCRRTARRSQPVITATDLEVRAGVRTLLSAPGPALRVQAGDRIGLVGRNGAGKTTTLRILAGEGEPYAGKILRSTEIGYLPQDPREGDLDVLARDRVLSARGLDTLIRDMEKQQALMAEVADEAEREKAVRKYGRLEERFSALGGYVAESEAARICHSLGLPDRVLGQPLRTLSGGQRRRIELARILFSASDGSGGRSDRILLLDEPTNHLDADSITWLRGFLQNHDGGLIVISHDVELLEAVVNKVWFLDAVRGEVDVYNMGWKKYLDARATDEQRRRRERANAEKKASALKAQAAKLGAKATKAVAAQNMVKRAERLLDELDEVRVADKVARIKFPEPAPCGKTPLMAENLTKVYGSLEIFTGVDLAIDRGSRVVVLGLNGAGKTTLLRLLAGVEQPTAGQLVPGHGLKVGYFAQEHDTLDDQATVWENIRHAAPDAGEQDLRGLLGAFMFSGPQLDQPAGTLSGGEKTRLALAGLVSSAANVLLLDEPTNNLDPISREQVLDALRTYAGAVVLVTHDPGAAEALSPERVILLPDGTEDHWSAEYLELIQLA

>CORE_REP|Org216_Gene3082#

MLIRLLRTYLSPYRAQLAGVVALQLVSVIAMLYLPSLNADLIDNGVTKGDIDYIWHTGLWMLAVTAVQIVASASSVFLGAQAAMSAGRDLRAALVHRVGTFSAREVGLFGAPSLITRNTNDVQQVQLLVVMSVTVLVMAPIMCVGGIIMALREDLKLSWLLLIAVPALALAMGLVVARLVPGFREMQARIDVVNRVLREQITGIRVVRAFVRERQETWRFGLANTDLTEASLRVGRLMALMFPVVMLISNVTTVAVIWFGGHLIDDGELQIGSLTAMLSYIMQILMAVMMASFLAMMAPRAAVSADRIGAVLTTESSVVPPEFPKPFAGDPGRVEFAAAEFAFPGAEKPVLRGIRFTVEPGTTTAIVGSTGAGKTTLLNLIPRLIDVTAGAVYVGGTDVRELDMELLREQIGLVPQKAYLFSGTVASNLRYGRPEATDEELWRALEIAQAADFVRDMPQGLETPVAQGGTTVSGGQRQRLAIARALVRRPRVYLFDDSFSALDVATDARLREALRPETRDASVIIVAQRVSTIRDADQIIVLEDGEMAGIGTHEQLLRDCAEYQEIVASQLSAQEEVR

>CORE_REP|Org113_Gene5713#

MRPEVVVVRWIFMLDRARVTKGDKVILDDVSLTVLPGAKIGVVGPNGAGKSTVLRVMAGLELTAGGEAVLAPGITVGILAQEPELDETATVRGNVEAAVAGTQALLARYTEIAERLADDADEELLAELGALQEQLDRRGAWDLDSRLDQAMDALRCPPPDAGVTTLSGGERRRVALCRLLLQRPDLLLLDEPTNHLDAESVQWLEQHLSGYPGTVVAVTHDRYFLDNLAEWILELDRGHAHPYRGNYGIYLDTKATRLRVEGRKDAERLRRLRRELEWIRSGPAARQAKGAARLRRYEEMAAAADGARVRTFDEIRIPPGPRLGGLVVEADHVDKSFGDHTVIRDLSFSLPRNGIVGVLGPNGAGKTTLFRLLIGELTPDAGKIRIGDSVEISYVDQNRVRIDPGRTAWDVVSGGHAVIGVGTMEVPSRAYLAAFGFRGVDQQKPSRLFSGGERNRLNLALTLKQGGNVLLLDEPANDLDTETLDSLENAIDEFAGCVIVTAHDRWFLDRLATHILAWEGTAADPGRWFWFEGNFAAYEQNKLARLGPDAARPHRLTHRRLTRD

>CORE_REP|Org202_Gene4973#

MRVRRPGRLDPVSTTLHARGLSAGHGERTLFDDLDLTIAPGDVIGLVGVNGAGKSTLLRMLAARETPTGTITLSPPDATVGYLAQEPERVPGETVLDFLGRRTGVTAAQRAMDAAAERLAEGGTDEYSPALERWLALGGADLEARAQEVAADLGLAESLADGLGTPMTALSGGQAARAGLASVLLSRYDILLLDEPTNDLDLDGLARLEDFVRGVRVPLVVISHDREFLARTVNRIVELDLAQQQVGLYDGGYEAYLAEREIARRHAREAFEEYADTRAALETRAQMQRNWLEHGVRNARRKARDPRKLDSDKAGRKMRAEATEKQAAKARQTQRRIERLEVVEEPRKEWELRMTIAAAPRSGAVVATATDAVVTRGDFRLGPVTTQIDWADRIVLTGANGAGKSTLLGLLLGRIAPDSGSAALGSGVEIGEVDQARSLFRGTTPLAERFGREMPDWPDAEIRTLLAKFGLRGPHVLRACDTLSPGERTRAALALLQARGVNLLVLDEPTNHLDLPAIEQLEQAVDSFTGTLLLVTHDRRMLDSVRATRRWHLRDGLLHED

>CORE_REP|Org18_Gene2143#

MLSHPERLPLLTASTDGAVSSGPRGLPAEVSRRRTFAVISHPDAGKSTLTEALALHAKMISEAGAIHGKAGRKSTVSDWMEMEKARGISVSSTALQFNYRAAGSDIDNVINLVDTPGHSDFSEDTYRVLTAVDAAVMLIDAAKGLEPQTLKLFQVCRHRGIPVITVINKWDRPGRAPLELLDEIDERIGLTPTPLFLPVGIAGDFRGLLRRGPDGEAVEYIHFTRTAGGATIAPEESLTPEQAQAREGEAWETAAEESELLSATGQDHDQELFLAGQTSPVIYASAMLNFGVRQLLETLVALAPAPAGRRDVDGGMRETSDPFSAVVFKVQAGMDTAHRDRLAFMRIVSGEFERGMVVTHAQTGRPFATKYALTVFGRERATVDTAYPGDVVGLVNATALAPGHTLFVDKKVEFPPIPSFAPEHFAVLRAQSAGKYKQFRKAIDQLDSEGVVQVLRNDARGDASPVLAAVGPMQFEVVTARMQAEYNVETQMDHLPYTLARRTDAASAEELGRQRGVEVFTRSDGVLLALFSDKWRLQYIEKEHPGLTLEPLVATAD

>CORE_REP|Org15_Gene6278#

MSDPRASGVRGRESADSTEQLDTGDRVHVTKSTGARVETAVPSNGNESSPDTYWRRAGRFRHRISRRLSAVPLRVTLALALVSLTGLGLLISGVAVTSAMRNVLMDNVDRQLFGAAHDWAGPDAPPPQRLPGPVGRERPPGLFYVRIEDPSGKVRSLFPTGPSVPDFPADLGKHPRTIGSVGNPDEHWRAERVTTPGGSSWVAIRLSETENIIDRLIGLQVAVGLMVLAVLAIVAQFVIRRSLRPLGEVEKTAAAIASGDLHRRVPVQGTNTEVDRLSQSLNGMLSQIQSAFAATEASEESARRSEARMRRFVADASHELRTPLTTIKGFAELYRQGALADPDMFMDRIERESKRMSLLVEDLLMLARLDAQRPVERRPVDLLALASDAVHNARAVDAAQRPEEPRRPIDLEIRPGTGTLEVRGDEARLRQVLGNLVNNALLHTPPEAAVTVALTPAPDEVVIEVADTGPGLPTEDAERIFERFYRTDTSRSRDSGGTGLGLSIVQALVAAHGGTVSVRSAVGQGTTFAVRLPRSQE

>CORE_REP|Org29_Gene4463#

MARTTSKRQAKSGANETVAPLGSSRRGADEPAPMRPPTPLTRTVSLRWRVTLLAASVVAIAVAVTSIAAYAMVARALYGDVDAQLRARAATMINGDIDSMAFQSLGVATLFSNNIGVGLIYPFSVSSPPSTPEGERTLDSLPVYIPPQPTKPPIGTEEIAVAKGEHTSSLRTYNNQRVLARRMDSGVTLVISQRLEPTREVLDRLAWLLFVVGGCGVLLAAAAGTAVGRTGLRPIARLTAATERVARTDDLTPIPVTGDDELARLTESFNTMLRALAESRDRQRRLVADAGHELRTPLTSLRTNMELLIAAGRPGAPRIPDEDMAELRMDVVAQIEELSTLVGDLVDLAREDAPETVYERVDLGEVAERALERARRRRGSIEFVAALRPWFVYGHEAGLERAILNVLDNAAKWSPAGAQVRVSMAEVGRGLLELSVDDAGPGIPPAERELVFERFYRTTASRSMPGSGLGLAIVKQVVTKHGGTITIDTSERGGALIRIVLPGEAGAPVATAEDEPDP

>CORE_REP|Org19_Gene2296#

MGTETVENRRHRVVVIGSGFGGLFACKHLEHDNVDVVLISKTSTHLFQPLLYQVATGILSTGEIAPATRIVLRKHHNTQVILGEVHDIDLVNKTVTSKLLNQDTVTSFDSLIVATGAQQSYFGNDRFATYAPGMKTIDDALELRARILGSFEEAELAKTQEERDRFLTFVVVGAGPTGVELAGQIAELADRTLVGTFRNIDPRDARVLLVEGAGAVLAPMGPKLGGKAQRRLEKMGVEIQLNAMVTDVDARGVTVKDKDGTERRIESACKVWSAGVQASELGKMLAERSKGTETDRAGRVVVEPDLTIKGYPNVFVVGDLMAVPGVPGQAQGAIQGATYAAKQIKAEVAGKQTPDQRKPFKYFNKGSMATVSRFNAVCQIGKLEFSGFLAWLIWLVLHLYYLIGYRSRTVTVFQWFVAFLGRNRGQMAATEQWVFARLALEAMNGNETDARDVQAEVGNTTPPAAPGEPSAKSAAATPDGEKASGTSESAGSGESTATSKSGASSESTTSGSSQPKAG

>CORE_REP|Org5_Gene2466#

MSTTDHNPSGATQHMPTTVTSPQVAVNDIGSAEDFLAAIDKTIKYFNDGDIVEGTIVKVDRDEVLLDIGYKTEGVIPSRELSIKHDVDPNEVVSVGDEVEALVLTKEDKEGRLILSKKRAQYERAWGTIEELKEKDEAVKGTVIEVVKGGLILDIGLRGFLPASLVEMRRVRDLQPYVGKEIEAKIIELDKNRNNVVLSRRAWLEQTQSEVRSEFLHQLQKGQVRKGVVSSIVNFGAFVDLGGVDGLVHVSELSWKHIDHPSEVVEVGMEVTVEVLDVDLDRERVSLSLKATQEDPWRQFARTHAIGQIVPGKVTKLVPFGAFVRVEEGIEGLVHISELAERHVEVPDQVVAVGDDAMVKVIDIDLERRRISLSLKQANEDYHAEFDPSKYGMADSYDEQGNYIFPEGFDPETNEWLEGFDKQREEWEGRYAEAERRHKMHTAQMEKMAADAAAEAANGGGAGNYSSESGAQASSSSSSSSESAGGSLASDAQLAALREKLSGNA

>CORE_REP|Org162_Gene2884#

MPADISAPPSRGPAPTGGKTPTVIRLLVLATFVVILNETIMINAIPRLMHDLDVTERAAQWVSTAFMLTMAAVIPVTGWFLQRVSTRQAYAIAMGVFLAGTALSAVAPTFAVLLVGRIIQAGGTAVMMPLLMTTLMTVVPEQDRGRVMGNVTLAISVAPAMGPVISGLVLQAGSWRWLFVLVLPIAGTVTWLGLRRLDNIGEPQTGDIDWLSVAFAAFGFGGLVYGLSKFETDHVAVPALLVAAGLALIAVFAFRQLRLQRSGVPLLDLRILLSGTYTKALVLMSVAFLAMLGSMILLPLYLQNLRHLSPLETGLLVMPGGLAMGLLGPTVGRLFDRFGGRPLVIPGAVGVTVALAGFTQISMSMPYWQLLALHILLMISLAGLFTPVFTLGLGALPPHLYSHGSSMLGTLQQVAAAFGTALVVTVMSARMTQLMETGTEPVTAQLDGMRLAFAVSAALSVLVIVTAILLPSRAPAPEETGEDDASEAETAESAAPLLVKD

>CORE_REP|Org3_Gene1380#

MTGPDETDGPDFAREAGNAEPEPQHGTGAPLGSGPSPVDLAEMALVEAELDRRWPETKIEPSLTRIATLMDLLGSPQQSYPAIHIAGTNGKTSVTRMIDALLTALHRRTGRITSPHLQLATERISIDNAPITPARYVEVYRELAPYIEMIDQQSAAAGGPAMSKFEVLTGMAYAAFAEAPVDVAVVETGMGGTWDATNVIDGQVAVITPIGLDHTEYLGPDLTAIAREKAGIIKRAPESLIPRDNVAVIAEQDPEAMDVLLRRAVEVDAAVAREGAEFRVLARKIAVGGQQLELQGLGGVYDEIFLPLHGEHQARNAVLALAAVEAFFGAGAQRQLDVDAVRAGFASVTSPGRLERMRSAPTIFIDAAHNPAGAKALAATLTSEFDFRKLVGVVAVLGDKDAAGILEALEPVFDEIVVTTNGSPRALDVDSLTDLAVQRFGDERVVPAYTLPDALETAIAIAEDVADTGEMVSGAGVIVTGSVVTAGAARALFGKEPA

>CORE_REP|Org106_Gene2979#

MLENPAATTQFPVTQRAFGLAILVLSGLQLMVVLDGTVVIFALPRLQDQMGLSSAGSAWIVTAYGLTFAGLMLLGGRLGDAFGRKRMLIAGVGLFTVASLLCGLAHWQAMLIAARALQGAGAAIAAPVAFALVATTFAPGKARNQAIAIVGSMVGIGSVGGLVVGGALTQLSWRWIFLINVPIGALIILGAIYCLADTGHHRVALDARGAVLGTLACAAIVFGATEGPELGWSHPAVIGALIGGAILLVVFVIAERNVDDPLLPWSLFDSRDRVTTFVLILLAGGVLGAMTYFVAQFLQNVLGYGPLQAGVASIPFTVGIGIGGALASKLAMTVAPRWLLFGAALVLAVGLLFGSTLDGEVSYLPTLLPLLIVIGFGVGVAMVVTPLCVLVGVPPSDIGPLSAVGQMFMNLGTPMAIGILTPVAVSRTLSLGGTTGKVSAMTDAQIVALGEGYTLVLAVCAGVAAVIGLIALTLRFTPEQIARAQHAQEEAQRS

>CORE_REP|Org63_Gene113#

MDSVSQRLDLRPNRIAVLSVHTSPLAQPGTGDAGGMNVYVLQTAVELARRGTEVEIFTRATASNLPPVQEAAPGVLVRNVVAGPFEGLDKHDLPTQLCPFTAEVLRQEARHLPGYYDLVHSHYWLSGQVGWLARDRWRVPLVHTAHTLAAVKNAALAEGDCPEPATREIGEKQVIAESDRLVANTAEEARQLVELYGADPERIDVVPPGADLTLYRPGDKAAARAALGLSADEQIVAFVGRIQPLKAPDVLVRAAAEVLRADPERPLRVLIVGGPSGSGLDRPDALIELAAELGIAARVSFLPPQPPQRLVLVYRAADLVAVPSYNESFGLVAIEAQASGTPVLAADVGGLGTAVRHDVSGLLVPGHRTSDWANALRHLLDDPGRLHRMGERAVAHAANFSWAHTADGLLASYAAALAGFRDERSALGGRGLAHSLVRDDAYDRAAADRTNLAGERTAALLAESSQARSRALWRRRMGVRR

>CORE_REP|Org101_Gene5725#

MTDQKPESFPLRRSVAASAMGNATEWFDYGVYAATATYLTDAFFPGELGTLGTMLGFAVSFVLRPLGGMVWGPLGDRIGRKAVLATTILLMAAATGAIGILPTHSSVGVFAPILLIGLRVVQGFSTGGEYGGAATYLAECASDKRRGFLGSFLEFGTLAGFVGGSATVLACQLAIGSDAMHDWGWRIPFLLAVPLGLVGWYLRSRLDESPVFTEVAEVAEQTDQEHRPGGLHGLRELVTTYRRELLTLGGLVVALNVVNYTLLTYQPTYLQKTIGISESGTTAMMLIGQTVMMVTLPFFGRLSDRVGRRPMWLFSLVGLAVLALPMYWLMGQGTAWAITGFIVLGLLYVPQLSTISSTFPAIFPTQVRYAGFALAYNVSTAAFGGTAPLVNEAAIESTGWSLFPAAYMIGASLIGLVAWCFLRETAGTSLRGTEVPDAGEDAPAIIPAGPAGAALAP

>CORE_REP|Org105_Gene2713#

MINRGVVRMRNTKAVQAAEPEFVEVVIVGSGFGGLAAAKQLAKSGVPYVLISSTPEHLFQPLLYQVATGVLAADEIAPPIASILRRHEKADVRLGKVTAIDPDAAELVYETADGPRRIRYGSLIAATGANQSYFGRDDFAEKTFALKTIDDAKRLRAQIDHVFTQAKHADKETRERLLSFVVVGAGATGVEVAGQLAELAKRYYHQDVSVTLVEGAGEVLPPFGGGLSEYAKQSLTKGGVEVLLGTFVTDIEPGKVTVKDKQGVEHRIAAETVVWSAGVQASGFTKILAEATGAETDRAGRLLINPDLTVGGYADIYAIGDMTSLKGYPGQSPVAMQEGRHAADIIRRKKLPGTEFEYWDKGSMAVIRRRSAIAKVSDKIKFKGLIAWYMWLAVHLFYLVGFRNRFMAVMGWLVAFTGNGRPGFAEIDKDRPAVGHKPPIAA

>CORE_REP|Org103_Gene5459#

MACDLRGTSRTRRWSRGRRPYDSGVSVPQAVLLAVLAAVVGLAVGGLLIPYVNARQAARRQADSGLTMSQVLDLIVLASESGIAVVDQYRDVVLVNPRAEELGLVRNRLLDERAWAAVEKVLATGESAEFDLTAKNPLPGRSRIAVRGVARPLSQEETGFTVLFADDDSEQARMEATRRDFVANVSHELKTPVGAMSLLAEALLESADDPEAVRHFGQRVLGESRRLGKMVTELIALSRLQGAEKLPELEVVDVDTVVMQAVDRSRTAAEAAGITVSTDRPSGLEVLGDETLLVTALSNLVENAIAYSPPGSHVSVSRSLRGKYVAMAVTDRGIGIAKEDQERVFERFFRSDKARSRATGGTGLGLAIVKHVAANHNGEITLWSKLGTGSTFTLRIPAHLEADSGDDDVDADGAAVSTKENGSRPSGPGRPNGVEARR

>CORE_REP|Org100_Gene1857#

MDKLPGVSERFLVTGGNRLVGEVAVGGAKNSVLKLMAAALLAEGTTTITNCPDILDVPLMAEVLRGLGCEVTITDDAPGDRSVVTITTPAEPKYHADFPAVTQFRASVCVLGPLMARCKRAVVALPGGDAIGSRPLDMHQAGLRLLGATSEIEHGCVVARAEELRGARIRLDFPSVGATENILMAAVLAEGETVIDNAAREPDIVDLCNMLVQMGARISGAGTSVLTIQGVERLHPTEHRVIGDRIVAATWGIAAAMTMGDVRVTGVNPKHLALVLDKLRSAGARISFDVDGFRVVQPDRPRAVNFSTLPFPGFPTDLQPMAIGLAAIADGTSMITENIFEARFRFVEEMIRLGADARTDGHHAVVRGIPRLSSAPVWSSDIRAGAGLVLAGLVADGTTEVHDVFHIDRGYPNFVEQLQSLGGLVERVGGAE

>CORE_REP|Org12_Gene4586#

MRTLDTSFSTRNGGFREVVVTAVEITTSIGADTESTWQALLSGASGIKVLTDEDITRHDLPNAIGGKLIHDPTADLDRVRKRRMCYVQQMSYAMGQRLWETAGAPEVDKDRLGVCIGTGLGGADVIVEANDTMREHGYRKVSPFAVPMSMPNGVSGVVGLDIGARASLVTPVSACASGNEALVHAWRSIVLGDADMVVAGGVEGYINPMAIAGFTMARALSSRVDEPERASRPFDRDRDGFVFGEAAALLLVESEEHARARGATPLARLLGAGLTADGYHMVAPDPEGLGCARAMRRAIETAGVSAADVDHVNAHATGTSIGDLAEAKGIAAAIGTHPAVYAPKSALGHSVGAVGALEAAISVLTLRDQVIPPTLNLDNQDPEIDLDIVHDKPRHTDVEFAMNNSFGFGGHNAAVLFGRY

>CORE_REP|Org105_Gene3182#

MRVARARPSVYVESVSFAHAAAMTGPADRLRVAMLTREYPPEVYGGAGVHVTELVSELRALAEVTVHCMGAPRDDAVVHQPDTHLYAANPAIQMMSAQLRMADATGEVDVVHSHTWYTGLAGHLSATLYGIPHVLTAHSLEPRRPWKAEQLGGGYRLSSWSERNAVEHADAIIAVSAGMRRDVLDAYPAVDPARVHVVHNGIDASVWHPGPPEAGGEPFLWQLGVRTDRPIAAFVGRITRQKGVAHLLAAARDFDSEIQVVLCAGAADTPELAAEVASAVEELSRRRGNVFWVQDMLPTEQIRQVLAAATVFVCPSVYEPLGIVNLEAMACGTAVVASDVGGIPEVVADRNTGRLVHYDPAAPSEYERGLAEAVNELAADQVLASEYGAAGRARAVAEFDWSRIAAQTLEVYDRVRKP

>CORE_REP|Org134_Gene3544#

MELKVQVYRHASSIPGGCPPPTTPVVAGSGIVNAQVETVVDLDAIAHNVRILREHAGDAAVMTVVKADGYNHGAVEVGRAALAAGAAELGVTTISEAVHLREAGITAPILCWLNNSGADYGAGIAADIEIGISSMSQLRAVEAAARRLGRTATLTLKVDTGLNRNGVSVTEYRDVLTALRPLVDEQVLRFRAIFSHLAHADQPHHPTIDVQRDRFVDAIATAKEYGLVPEVTHLANSAAALTRPDLAFDMVRPGIAMYGLSPVPELGDFGLRPAMTFQAEISLIKHVAAGEGVSYGHEWIAPHDTTVALIPAGYADGVSRRLGGRCEVWVRGARRPSIGRVCMDQMVIDLGDNLDGVAEGDTAILFGTGESGEPHAQDWADLLDTIHYEVVCSPRGRVVRRFRGGQQ

>CORE_REP|Org102_Gene3646#

MGGVISAARPGTRATALGWDTGEVMTAPLPLVFDAPRRGMPPRHLADLDAEERRAVMADLGLPKFRADQIARQYYGRLQADPEQMTDLPADMRAKVGEALFPPLLTPVRHIACDDGSTRKTLWKAGDGTLLESVLMRYPDRATLCISSQAGCGMACPFCATGQGGLNRNLSTAEIVDQVRAAAAALRDGEVAGGPGRLSNIVFMGMGEPLANYKRVVNAVRRITSPAPDGLGISQRNVVVSTVGLAPAIRKLADEDLSVTLAVSLHTPDDELRDTLVPVNNRWPVAEVLDAARYYADKSGRRVSIEYALIRDINDQPWRADMLGKKLHKALGSRVHVNVIPLNPTPGSKWDASPKPVEREFVRRVEAQGVPCTVRDTRGQEIAAACGQLAAEG

>CORE_REP|Org150_Gene5131#

MPSLDNAGSHTPREDSGATDAAASQAGTDAAAAHADTGAAASRAGTDPAASRVGTDAAAPRAGSDAAGGADTAVRVSGADPAVVVDDVRKSFGEVQALQGISFTAARASVLGILGPNGAGKTTTVKILSTLLRPDSGSASVAGHDVVADAAGVRRSIMMTGQYAALDENLSGRENLELFGRLMGLPKKDARRRADTLLEEFDLVGAGKRAVRHYSGGMRRRVDIACGLVVRPEVVFLDEPTTGLDPRSRQGVWDLVNALKEQGITVLLTTQYLEEADVLSDNIIVIDKGTVIAEGTADELKEKTGGSYCEVVPLDPTQLRKAVTALGELVPEALRHEFAGDRISIPAPDGASTLAEAVRRLDAAGLELADIALRRPSLDDVFLSITGHSGGHQ

>CORE_REP|Org15_Gene485#

MTSYAPAEALAIEADELVKVFGEQRAVDGVSLAVPQGAVYGVLGPNGAGKTTTIRMLATLLRPDGGRARIFGHDVVAEPTAVRSLIGVTGQYASVDEKLSATENLIIFSRLLGLSRSEAKRRAAELLEEFGLTEAATKALENFSGGMRRRLDLAASLIATPPLLFLDEPTTGLDPRTRAQMWETIRRLVREGATVLLTTQYLDEADQLADRIAVIDHGRVIADGTSDELKGSVGQSALQITVADRDVIERARTLIGEFLSRADGKLVEASISPEAGRVTAPLSDPSVTADLLIRLRDNDIRVDEITVSKPSLDEVFFALTGHAAESDAAESDSAESDSAGSNSEGTAA

>CORE_REP|Org201_Gene1319#

MGDAIVAEGLVKRYGQQVALDGLDLTVPEGTVTALLGPNGAGKTTTVRVLTTLLIPDGGRATVAGIDVLRDPRALRRRIGASGQYAAVDEYLTGFENLEMVGRLYHMGVQRSKERARELLDRFRLSDAADRPVKGYSGGMRRRLDLAGALVAAPPVLFLDEPTTGLDPRARLDLWDVIEELVAGGTTLLLTTQYMEEADRLADSIAVIDRGKVIAKGTADELKTMVGGDRIELTVDHVDNLAIAQQALAGLADGEIHLEPGLRRIIVPVSNGSQALVEAVGRLNDHSVKIHDVGLRRPSLDDVFLTLTGHEAEELINADDAADGLGALEATEGKTR

>CORE_REP|Org134_Gene5162#

MGRVRAIRLNGFGGPEVMEWAETPDPQAGPGEVLIDVAAAGVNRADVMQRKGHYPPPPGASEVPGLECSGVIAAVGDGVRGWSVGDRVCALLSGGGYAERAVAPAGQLLPIPDGLDLGAAAGLPEVAATVWSNLVMTAGLHAGQLVLIHGGGSGIGTHAIQVAKRLGARVAVTAGSAGKLERCRELGADILINYREEDFVAVIRAEQGSGGPGADIILDNMGAAYLARNVEALATYGQLVVIGLQGGVDAELNLAALLGKRAAVRATNLRGRPANGVGSKAEIIAEVREHVWPLVTEGAVVPVIHAELPINEVGDAHALLDSADTVGKVVLHIGDY

>CORE_REP|Org1_Gene5346#

MLDSMIEVRGLTKHYGRTAAVEDLTFTVKPGQVTGFLGPNGAGKSTTMRMILGLDTPTAGTALIDGKPYHQLKQPLRTVGALLDAKWVHPNRSARAHLEWLAASNGIARSRVEEVLRLVGLSEVAGKNAGGYSLGMSQRLGLAGALLGDPKVLLFDEPVNGLDPEGILWIRRFMQRLASEGRTVLVSSHLLSEMAQTAEHLIVIGRGKLIADTPTKEFIERASEQTVRVRSPQLDQLRSLLTSNGMTVREDGTGAEGPALLVAGVTSDAVGKLAGANDITLFELSPQRASLEEAFMRMTGGAVQYHGEGAEAVGVPGPGGPYTAMGGAL

>CORE_REP|Org114_Gene5945#

MARRARVDAELVRRGLARSREHAVELISAGRVLINGTVATKPATGVETATPLLVREEPDEVRWASRGAHKLLGALAAFEPQGVTVAGKRCLDAGASTGGFTDVLLSKGAAAVVAADVGYGQLVWRLRSDDRVEVHDRTNVRALTPELIGGTVELVVADLSFISLGLVLPALALCCAPGADLLPMVKPQFEVGKERVGSGGVVRDPALRAEAVRAVAAAAARLGLRTHGVVASPLPGPSGNVEYFLWLRKELSGADHSTGSITGAAADSSASAHPGVQSVPEDGAGTGLPAAPGAAAVGAAAYDAVEEERVAALIQRAVEEGPQ

>CORE_REP|Org19_Gene6366#

MSALITPRDGRSCVVMGVVNVTSDSFSDGGRYLDPAVAVAHGVRLYEAGADIIDVGGESTRPGAVRIDPETEAQRVVPVIRGLVEAGVPTSVDTMRASVAAAAIDAGVSVVNDVSGGRADAEMVKVVAAAEIPWILMHWRANADHRHIGPADHYDDVVREVLAELSSQVDLAMAAGVHPSRLVLDPGLGFAKNAEHNWALLGALPELTAQGLPILVGASRKRFLGSLLGDESGPRPPDGREVATATISALAAQHGAWGVRVHDVRSSLDAIAVADAWRRAAESAERRAAESAERWAAESVERRAAEAGSHNQGSE

>CORE_REP|Org1_Gene4747#

MVMFSPPAAPLPTLCGKPVATDRALVMAIVNRTPDSFYDRGATFTDEAAMAAVDRAVAEGADLVDIGGVKAGPGSEVDAAVDIGGVKAGPGSEVDAAEETRRVVPFVAAIRAAYPDLLISVDTWRSEVARAAVAEGADLINDTWAGADPELVRVAAEHGAGIVCSHTGGAVPRTRPHRVRYADVVAEVTETVVAAAERAAAAGVRTDSILIDPTHDFGKNTYHGLELLRGLDVLVNSGWPVLMALSNKDFIGETLGVGLSERLEGTLAATAWSAAAGARVFRVHEVAHTRRVVDMIAAIQGIRPPARTLRGLV

>CORE_REP|Org127_Gene4988#

MTDNSERICAGRTVIVTGAGRGIGRAHALAFAAAGANVVVNDLGAELDGAPSADSPAAQVVEEIVQAGGRAVVNGDDVADWAGAKRLIGQAVETFGGLDVVVNNAGIVRDRMLVNLAEDEWDAVIRVHLKGHFATMRHAIEYWRAESKAGRARDARIINTSSGAGLQGSVGQGNYAAAKAGIAALTITAAAEFGRYGVTVNAIAPSARTRMTETVFADMMARPDDGFDAMAPENVSPLVVWLGSPDSAGVTGRMFEVEGGKVALADGWRHGVAEDRGARWQPSELGPVVRELIAKATDPEPVYGA

>CORE_REP|Org66_Gene2436#

MAAAVSVHPRRHDLRRFERSAAQHHRRASARAPSGGSAVSGPLSVAPQPIPGHGLLTGRVAVITAAAGTGIGSATARRLLAEGADVVISDWHERRLGETEVELKGEFPERRVAAIACDVQSTTQVDELVRGAAAALGRIDIMVNNAGLGGETPVVDMTDEQWDRVLDITLNGTFRCTRAALNYFRAAGHGGVIVNNASVLGWRAQYGQAHYAAAKAGVMALTRCSAIEAAELGVRINAVAPSIARHAFLDKVSSSELLDRLSEREAFGRAAEPWEVAATIAMLASDYTTYLTGEVVSISSQRA

>CORE_REP|Org102_Gene5292#

MPDNPAPANLTPAEPTAADRAPADSAPLNLAAGAPAPGNPAQPVAFVTGAARGIGAAIAQRLAADGATVAVVDLDENSCAAAVDTIVAAGGKAIAVACDVTAEDQVDAAVDRVAAELGSLDILVNNAGVLRDNLLFKMSVAEWDTVMSVHLRGAFLCSRAAQRHMVAQRSGKIVNTSSVSALGNRGQANYSAAKMGIQGFTRTLAMELGPYGINVNAVAPGFIVTEMTAATAARLGVSSEELQAKTAEITPLRRVGQPADIADVVAFLASENAAFVTGQTIYVDGGRRL

>CORE_REP|Org5_Gene5353#

MTDAADATPGAPGTGNTAEPDATTAAGASARTDAAPPMISMRNVDKHFGDLHVLRDVNLEVPRGQVVIVLGPSGSGKSTLCRTINRLEPIDSGTIAVDGVELPAEGRALAKLRADVGMVFQSFNLFAHKTILDNVLLGPVKVRRVDKKRARARAMELLERVGIADQADKYPAQLSGGQQQRVAIARALAMDPKVMLFDEPTSALDPEMVNEVLDVMVALAKEGMTMLVVTHEMGFARRAGDRVLFMADGRIVEDAPPETFFTAPASERARDFLGKILSH

>CORE_REP|Org19_Gene2600#

MRNPLATPTGCGCPARHRRRREQHCAARSRRRPGAGVPQDTGGVVTSAERPPAATRVLVVDDEPQILRALRINLSVRGYEVITAATGAAALRAAAEKHPDVVVLDLGLPDIDGVEVLAGIRGWSSMPVIVLSARTDSSDKVQALDTGADDYVTKPFGMDELLARLRAAVRRSASTAEESAPIVETSSFTVDLAAKKVIRGGRDVHLTPTEWGVLEMLVRNQGKLVGRRELLREVWGPTYATETHYLRVYLAQLRRKLEDDPSQPKHLLTEAGMGYRFQA

>CORE_REP|Org31_Gene3788#

MGGLLEGKTILVTGIITDSSIAFHAAAVAQEQGAKVIITGIPERLRLIDRIAKRLPQEVPPAIPLDVTSEENLAELADKLRELAPEGIDGVLHSIAFAPRTLMGPEALPFLDGPGPDAAKAFEISAWSYASLARAVLPVMNERGSIVGMDFDPRTAMPFYNWMGVAKAALESVNRYVAREVGAAKKIRSNLIAAGPIKTLAAKAIAGTATDDAAKLNQLNEYWDGASPIGWDVDDPTVVAKSIVAMLSDWLPGTTASIIYVDGGASHNTWFPEDMSIN

>CORE_REP|Org103_Gene1084#

MTDLTAAFAASVACGAMSSDLLGKSALVSGASRGIGKAVAAELLRRGANVLITARKPEPLAEAAAELRALGHQGEVATIAGNSGDAQARAEAVGRAVTEFGSLDILINNTGINPVFGALMDADLDAVRKIFDVNVVAALGYAQEAYKAWMGEHGGAIVNVASVAGLRSTGVIAAYGASKAALIRLTEELAWQLGPKIRVNAVAPGVVKTKFADALYSADEERAASVYPMKRLGSPEDVARLIGFLASDEAAWITGETVRVDGGLLATGGI

>CORE_REP|Org77_Gene3533#

MALEIDLSGRVVLVTGGVRGVGAGVSRALLAAGATVLACARRPGDAPVEYEGRQAEFLPCDVRDGDAVRELIDTVIARHGRLDHLVNNAGGAPFALAADASAKFHAKIVELNLLAPLLVSQLANAVMQAQPDGGTIVNISSVSAHRPSPGTAAYGAAKAGVDSLTASLAVEWAPKVRVNSVVVGPVETELSLLHYGDADGVAAVGATIPLGRLARPEDVGRCVAFLASPLAGYVSGATLEVHGGGERPAFLDAATVNTAAPNGAPKP

>CORE_REP|Org15_Gene4889#

MSDGTGLLADKVVVISGVGPGLGRSLCVQAAAAGAKVVLAARTESRLREVADEIDGAGGTSLIVPTDITDDAAVANLVERTVATFGRVDALINNAFAMPSMKSLARTDFQQISDSLELTVLGTLRATQAFTDELAKTRGAVVMINSSVLRHSEPRYGSYKVAKSALLAMSQTLATELGAKGIRVNSVAPGYIWADRLKWYFGEVAKKYGITVEQVYEQTASRSDLKRLPEPDEIARAVVFLASEWASAITGQTLDVNCGEYHA

>CORE_REP|Org37_Gene1288#

MPAPGRRSRRGPASARRIRGHGPAAPGHDGNGEPVSGRADSDGDTPIRVLLVDDEQLVRSGFRLLLDIEDDITVVGEAANGAEAVRKARALRPDVVLMDIRMPTMDGIQATREIAATTGLQDVRILILTTYDTDAYVFEGLQAGASGFLLKDAGPAELLHAIRVVAAGEALLAPRITRRLIAQFTARRAADRAAEQRLAVLTDREREVLALVGQGMSNAEIGAELFLSPATARTHVSRAMVKLGARDRAQLVVIAYRTGLVAP

>CORE_REP|Org71_Gene936#

MADGRAAGHPDVEGSTRTRCGLRPFGADQTGERVTTAATETTTAVLVVDDQELVRGGLRRILRRRDGFVLTECADGDEVVPAISAEPPDVILMDLRMKRVGGIEATRLVRMRADAPPVLVLTTFDDDQLLSGALRAGAAGFILKDSPAEDLIRAVRTVAAGGAWLDPAVTGRVLSAYRTVRPATPTDARLAELTAREYEVLELIGRGRVNSEIARELGISEVTVKSHVGHIFGKLDLRDRAAAIVFAFDHGVVSPGQSTV

>CORE_REP|Org114_Gene5983#

MLELTDVTKEYRVGEQTVRALDGISLRIEPGEFTAIIGPSGSGKSTLLHMLGALDSPDSGSIRFQDAEIGGLDDDRQSEFRRHRVGFVFQFFNLLPTLSAWENVAIPKLLDGTGLRKAKPRALELLELVGLADRAEHRPAELSGGQMQRVAVARALIMDPPLILADEPTGNLDSKTGASILELLGDITRQGNSVVMVTHDMGAVRYCDRLITLRDGKIGSNELVEHTENGEVRTVPVELTASLSEDGSEPAQAVRP

>CORE_REP|Org35_Gene4389#

MSNSVEDSRKSESQNAERASRSVLVTGGNRGIGLAVAQRLLADGHKVAVTHRGSGVPDGLFGVKCDVTDSESVDRAFSEVEAHQGPVEVLVANAGITDDTLLMRMTEEQFTRVIDANLTGAFRCAKRANRAMLRARWGRMIFLGSVVGLGGGPGQINYASSKAGVIGLARSVTRELGSRNITANVVAPGFIETDMTAELPEEMRETAKKFIPLQRLGAPEEVAAVISFLASEDSRYVSGAVIPVDGGMGMGH

>CORE_REP|Org2_Gene6607#

MNSLTPAVSLLTSNNDGVNTTSSSVPAASVLVAEDDPHVRSTLDQLLRFEGYQVYLAADGQEALELLAQQRPDLAVVDVEMPRLDGLSLCRLLRRRGDRLPILVLTARQQIGDRVAGLDAGADDYLPKPFATDELLARLRALLRRSTFDEDDDTVLAVGDLTLNTATRQVHRGDRPIELTKTEFDVLELLLRNARIVLSRSRIYEHIWGFDFDTESRSLDVYIGYLRRKTEENGEPRLIHTVRNVGYSVRPA

>CORE_REP|Org5_Gene560#

MSRMNGVAGDRIPEARVLVVDDEPMIVELLSVSLRYQGFEVAAAGNGAEGLDRAKQFRPDALIVDVMMPGMDGFGLLRRLRADGIDAPVLFLTARDEVDDKITGLTLGADDYVTKPFSLEEVVARLRVILRRSGHVVEETKSSRIRFEDIELDDDTHEVWKAGEPVALSPTEFTLLRYFMVNAGTVLSKPRILDHVWRYDFGGEVGVVETYVSYLRKKVDTGPDRLIHTLRGVGYVMRAPSRSRSSAK

>CORE_REP|Org31_Gene655#

MVPLPDAYRTSELVSTPKVLVVDDDEDVLASVERGLRLSGFHVLVARDGAQALRSVSEHAPDAIVLDMNMPVLDGAGVVTALRAMGNEVPICVLSARASVDERISGLESGADDYLVKPFVLAELVARIRALLRRRTDTPPAATPGAITVGPLEVDIAGYRAVLHGNEIELTKREFELLSTLARNVGVVLSRERLLELVWGYDFAADTNVVDVFVGYLRRKLEVDGAPRLLHTIRGVGFVLRAPK

>CORE_REP|Org114_Gene4108#

MSANLMIVEDDDRVRVALRLAMEDEGYDVAEAEEAEVALRQLRDNGAPDFMIVDLMLGGMDGFTCIREIRRDHDVPIIVVSARDDTHDVVAALEAGADDFVTKPFEVKEITARMRAVARRARFAEQAAAEEDPDSELGTMVLDEQAGNPLVLSTESGIVRRGDEEIHLTLTEYRLLCELAGSAGRVLSRGTLLERVWDRGFFGDERIVDVHIRRLRTKIERDASDPQLIVTVRGLGYRLDVQR

>CORE_REP|Org12_Gene5846#

MSTDTVKSEKATNLTLPVKEPGGKTNGTVELPAEIFDATANIALMHQVVVAQQAAARQGTHATKTRGDVRGGGKKPYRQKGTGRARQGSTRAPQFTGGGTVHGPQPRDYSQRLPKKMKAAALRGALSDRARNERIHVISELVAGQTPSTKAAKNFLAELSDRKKVLVVVGREDVTAWKSVANLQGVHPIAPDQLNTYDVLLSDDVVFSVEALNAFVHGPTESAQESQPGSAAQEESK

>CORE_REP|Org44_Gene3372#

MTSVLIVEDEESLADPLAFLLRKEGFEVTVVGDGPSALAEFDRSGADIVLLDLMLPGMSGTDVCKQLRTRSGVPVIMVTARDSEIDKVVGLELGADDYVTKPYSARELIARIRAVLRRGAGDELDGNGESGVLEAGPVRMDVDRHTVMVNGKPVTLPLKEFDLLEYLLRNSGRVLTRGQLIDRVWGADYVGDTKTLDVHVKRLRSKIEADPAKPEHLVTVRGLGYKLEA

>CORE_REP|Org96_Gene3994#

MITMRNVTKSYKTSTRPALDNITVDVDKGEFVFIIGPSGSGKSTFMRLLLKEESPTAGEIRVADFRVDRLPGRKVPKLRQRMGCVFQDFRLLQQKTVQENVAFALEVIGKRRQVIERTVPEVLDMVGLGGKADRLPSELSGGEQQRVAIARAFVNRPLVLLADEPTGNLDPDTSGEIMLLLERINRTGTTVLMATHDNHIVDAMRRRVVELDHGRLVRDEATGVYGVGR

>CORE_REP|Org125_Gene428#

MTEKKQGPATDAKTRPAAGILGTKLGMTQVFDEKNRVVPVTVVKAGPNVVTQIRTEERDGYSAVQLAFGAIDPRKVTKPVAGQFAKAGVTPRRHVAEIRVADAAAFEVGQELSADVFEEGTYVDVTGTSKGKGYAGVMKRHGFRGQGAAHGAQAVHRRPGSIGGCSTPGRVFKGMRMAGRMGNDRVTTQNLSVHKVDAENGLLLIKGAIPGRKGGIVIVKSAAKGGARA

>CORE_REP|Org7_Gene3709#

MTAVLLAEDDEAIAAPLSRALGREGYSVTVERFGPAVLERALEGHHDLLILDLGLPGMDGLEVCRQVRASGADIAVLMLTARTDEVDFVVGLDAGADDYVGKPFRLAELLARVRALLRRSGIGDDTVEVGGIRLEPAARRVLVNGAEIGLANKEYELLKVLIDRAGQVVPRETILREVWGDAELRGSKTLDMHMSWLRRKIGDEGPMAERRIVTVRGVGFRLNTD

>CORE_REP|Org170_Gene1954#

MSRIGKQPIAIPSGVEVTINGQDIAVKGPKGQLSLTVSEPITVTKGEDGQLQVARPDDERRSRALHGLTRTLVANMIEGVTKGYEKKLEIAGVGYRVALKGQNLEFALGYSHPVVAEPPQGITFAVESPTKFSVAGIDKQLVGEVAANIRKYRKPEPYKGKGIRYAGENVRRKVGKTGK

>CORE_REP|Org57_Gene1684#

MPTINQLVRKGRRDKVAKTKTAALKGSPQRRGVCTRVYTTTPKKPNSALRKVARVRLTSAVEVTAYIPGEGHNLQEHSMVLVRGGRVKDLPGVRYKIIRGSLDTQGVKNRKQARSRYGAKKEKS

>CORE_REP|Org56_Gene4690#

MADRVLRGSRLGAVSYETDRDHDLAPRRVARYRTDNGEEFDVPFADDAEIPPTWLCRNGQEGILIEGTTQEPKKVKPPRTHWDMLLERRSKEELEELLQERLELLKTRRGR
