# Supplementary material for: Design of a multi-epitope vaccine against six Nocardia species based on reverse vaccinology combined with immunoinformatics
Source: Front Immunol. 2023 Feb 2;14:1100188. doi: 10.3389/fimmu.2023.1100188 (PMC9952739; doi:10.3389/fimmu.2023.1100188)
Supplement: Supplementary file 13 [file Table_6.docx]

>CORE_REP|Org216_Gene3082#

MLIRLLRTYLSPYRAQLAGVVALQLVSVIAMLYLPSLNADLIDNGVTKGDIDYIWHTGLWMLAVTAVQIVASASSVFLGAQAAMSAGRDLRAALVHRVGTFSAREVGLFGAPSLITRNTNDVQQVQLLVVMSVTVLVMAPIMCVGGIIMALREDLKLSWLLLIAVPALALAMGLVVARLVPGFREMQARIDVVNRVLREQITGIRVVRAFVRERQETWRFGLANTDLTEASLRVGRLMALMFPVVMLISNVTTVAVIWFGGHLIDDGELQIGSLTAMLSYIMQILMAVMMASFLAMMAPRAAVSADRIGAVLTTESSVVPPEFPKPFAGDPGRVEFAAAEFAFPGAEKPVLRGIRFTVEPGTTTAIVGSTGAGKTTLLNLIPRLIDVTAGAVYVGGTDVRELDMELLREQIGLVPQKAYLFSGTVASNLRYGRPEATDEELWRALEIAQAADFVRDMPQGLETPVAQGGTTVSGGQRQRLAIARALVRRPRVYLFDDSFSALDVATDARLREALRPETRDASVIIVAQRVSTIRDADQIIVLEDGEMAGIGTHEQLLRDCAEYQEIVASQLSAQEEVR

>CORE_REP|Org15_Gene6278#

MSDPRASGVRGRESADSTEQLDTGDRVHVTKSTGARVETAVPSNGNESSPDTYWRRAGRFRHRISRRLSAVPLRVTLALALVSLTGLGLLISGVAVTSAMRNVLMDNVDRQLFGAAHDWAGPDAPPPQRLPGPVGRERPPGLFYVRIEDPSGKVRSLFPTGPSVPDFPADLGKHPRTIGSVGNPDEHWRAERVTTPGGSSWVAIRLSETENIIDRLIGLQVAVGLMVLAVLAIVAQFVIRRSLRPLGEVEKTAAAIASGDLHRRVPVQGTNTEVDRLSQSLNGMLSQIQSAFAATEASEESARRSEARMRRFVADASHELRTPLTTIKGFAELYRQGALADPDMFMDRIERESKRMSLLVEDLLMLARLDAQRPVERRPVDLLALASDAVHNARAVDAAQRPEEPRRPIDLEIRPGTGTLEVRGDEARLRQVLGNLVNNALLHTPPEAAVTVALTPAPDEVVIEVADTGPGLPTEDAERIFERFYRTDTSRSRDSGGTGLGLSIVQALVAAHGGTVSVRSAVGQGTTFAVRLPRSQE

>CORE_REP|Org29_Gene4463#

MARTTSKRQAKSGANETVAPLGSSRRGADEPAPMRPPTPLTRTVSLRWRVTLLAASVVAIAVAVTSIAAYAMVARALYGDVDAQLRARAATMINGDIDSMAFQSLGVATLFSNNIGVGLIYPFSVSSPPSTPEGERTLDSLPVYIPPQPTKPPIGTEEIAVAKGEHTSSLRTYNNQRVLARRMDSGVTLVISQRLEPTREVLDRLAWLLFVVGGCGVLLAAAAGTAVGRTGLRPIARLTAATERVARTDDLTPIPVTGDDELARLTESFNTMLRALAESRDRQRRLVADAGHELRTPLTSLRTNMELLIAAGRPGAPRIPDEDMAELRMDVVAQIEELSTLVGDLVDLAREDAPETVYERVDLGEVAERALERARRRRGSIEFVAALRPWFVYGHEAGLERAILNVLDNAAKWSPAGAQVRVSMAEVGRGLLELSVDDAGPGIPPAERELVFERFYRTTASRSMPGSGLGLAIVKQVVTKHGGTITIDTSERGGALIRIVLPGEAGAPVATAEDEPDP

>CORE_REP|Org103_Gene5459#

MACDLRGTSRTRRWSRGRRPYDSGVSVPQAVLLAVLAAVVGLAVGGLLIPYVNARQAARRQADSGLTMSQVLDLIVLASESGIAVVDQYRDVVLVNPRAEELGLVRNRLLDERAWAAVEKVLATGESAEFDLTAKNPLPGRSRIAVRGVARPLSQEETGFTVLFADDDSEQARMEATRRDFVANVSHELKTPVGAMSLLAEALLESADDPEAVRHFGQRVLGESRRLGKMVTELIALSRLQGAEKLPELEVVDVDTVVMQAVDRSRTAAEAAGITVSTDRPSGLEVLGDETLLVTALSNLVENAIAYSPPGSHVSVSRSLRGKYVAMAVTDRGIGIAKEDQERVFERFFRSDKARSRATGGTGLGLAIVKHVAANHNGEITLWSKLGTGSTFTLRIPAHLEADSGDDDVDADGAAVSTKENGSRPSGPGRPNGVEARR

>CORE_REP|Org12_Gene4586#

MRTLDTSFSTRNGGFREVVVTAVEITTSIGADTESTWQALLSGASGIKVLTDEDITRHDLPNAIGGKLIHDPTADLDRVRKRRMCYVQQMSYAMGQRLWETAGAPEVDKDRLGVCIGTGLGGADVIVEANDTMREHGYRKVSPFAVPMSMPNGVSGVVGLDIGARASLVTPVSACASGNEALVHAWRSIVLGDADMVVAGGVEGYINPMAIAGFTMARALSSRVDEPERASRPFDRDRDGFVFGEAAALLLVESEEHARARGATPLARLLGAGLTADGYHMVAPDPEGLGCARAMRRAIETAGVSAADVDHVNAHATGTSIGDLAEAKGIAAAIGTHPAVYAPKSALGHSVGAVGALEAAISVLTLRDQVIPPTLNLDNQDPEIDLDIVHDKPRHTDVEFAMNNSFGFGGHNAAVLFGRY

>CORE_REP|Org150_Gene5131#

MPSLDNAGSHTPREDSGATDAAASQAGTDAAAAHADTGAAASRAGTDPAASRVGTDAAAPRAGSDAAGGADTAVRVSGADPAVVVDDVRKSFGEVQALQGISFTAARASVLGILGPNGAGKTTTVKILSTLLRPDSGSASVAGHDVVADAAGVRRSIMMTGQYAALDENLSGRENLELFGRLMGLPKKDARRRADTLLEEFDLVGAGKRAVRHYSGGMRRRVDIACGLVVRPEVVFLDEPTTGLDPRSRQGVWDLVNALKEQGITVLLTTQYLEEADVLSDNIIVIDKGTVIAEGTADELKEKTGGSYCEVVPLDPTQLRKAVTALGELVPEALRHEFAGDRISIPAPDGASTLAEAVRRLDAAGLELADIALRRPSLDDVFLSITGHSGGHQ

>CORE_REP|Org15_Gene485#

MTSYAPAEALAIEADELVKVFGEQRAVDGVSLAVPQGAVYGVLGPNGAGKTTTIRMLATLLRPDGGRARIFGHDVVAEPTAVRSLIGVTGQYASVDEKLSATENLIIFSRLLGLSRSEAKRRAAELLEEFGLTEAATKALENFSGGMRRRLDLAASLIATPPLLFLDEPTTGLDPRTRAQMWETIRRLVREGATVLLTTQYLDEADQLADRIAVIDHGRVIADGTSDELKGSVGQSALQITVADRDVIERARTLIGEFLSRADGKLVEASISPEAGRVTAPLSDPSVTADLLIRLRDNDIRVDEITVSKPSLDEVFFALTGHAAESDAAESDSAESDSAGSNSEGTAA

>CORE_REP|Org201_Gene1319#

MGDAIVAEGLVKRYGQQVALDGLDLTVPEGTVTALLGPNGAGKTTTVRVLTTLLIPDGGRATVAGIDVLRDPRALRRRIGASGQYAAVDEYLTGFENLEMVGRLYHMGVQRSKERARELLDRFRLSDAADRPVKGYSGGMRRRLDLAGALVAAPPVLFLDEPTTGLDPRARLDLWDVIEELVAGGTTLLLTTQYMEEADRLADSIAVIDRGKVIAKGTADELKTMVGGDRIELTVDHVDNLAIAQQALAGLADGEIHLEPGLRRIIVPVSNGSQALVEAVGRLNDHSVKIHDVGLRRPSLDDVFLTLTGHEAEELINADDAADGLGALEATEGKTR

>CORE_REP|Org134_Gene5162#

MGRVRAIRLNGFGGPEVMEWAETPDPQAGPGEVLIDVAAAGVNRADVMQRKGHYPPPPGASEVPGLECSGVIAAVGDGVRGWSVGDRVCALLSGGGYAERAVAPAGQLLPIPDGLDLGAAAGLPEVAATVWSNLVMTAGLHAGQLVLIHGGGSGIGTHAIQVAKRLGARVAVTAGSAGKLERCRELGADILINYREEDFVAVIRAEQGSGGPGADIILDNMGAAYLARNVEALATYGQLVVIGLQGGVDAELNLAALLGKRAAVRATNLRGRPANGVGSKAEIIAEVREHVWPLVTEGAVVPVIHAELPINEVGDAHALLDSADTVGKVVLHIGDY

>CORE_REP|Org1_Gene5346#

MLDSMIEVRGLTKHYGRTAAVEDLTFTVKPGQVTGFLGPNGAGKSTTMRMILGLDTPTAGTALIDGKPYHQLKQPLRTVGALLDAKWVHPNRSARAHLEWLAASNGIARSRVEEVLRLVGLSEVAGKNAGGYSLGMSQRLGLAGALLGDPKVLLFDEPVNGLDPEGILWIRRFMQRLASEGRTVLVSSHLLSEMAQTAEHLIVIGRGKLIADTPTKEFIERASEQTVRVRSPQLDQLRSLLTSNGMTVREDGTGAEGPALLVAGVTSDAVGKLAGANDITLFELSPQRASLEEAFMRMTGGAVQYHGEGAEAVGVPGPGGPYTAMGGAL

>CORE_REP|Org127_Gene4988#

MTDNSERICAGRTVIVTGAGRGIGRAHALAFAAAGANVVVNDLGAELDGAPSADSPAAQVVEEIVQAGGRAVVNGDDVADWAGAKRLIGQAVETFGGLDVVVNNAGIVRDRMLVNLAEDEWDAVIRVHLKGHFATMRHAIEYWRAESKAGRARDARIINTSSGAGLQGSVGQGNYAAAKAGIAALTITAAAEFGRYGVTVNAIAPSARTRMTETVFADMMARPDDGFDAMAPENVSPLVVWLGSPDSAGVTGRMFEVEGGKVALADGWRHGVAEDRGARWQPSELGPVVRELIAKATDPEPVYGA

>CORE_REP|Org66_Gene2436#

MAAAVSVHPRRHDLRRFERSAAQHHRRASARAPSGGSAVSGPLSVAPQPIPGHGLLTGRVAVITAAAGTGIGSATARRLLAEGADVVISDWHERRLGETEVELKGEFPERRVAAIACDVQSTTQVDELVRGAAAALGRIDIMVNNAGLGGETPVVDMTDEQWDRVLDITLNGTFRCTRAALNYFRAAGHGGVIVNNASVLGWRAQYGQAHYAAAKAGVMALTRCSAIEAAELGVRINAVAPSIARHAFLDKVSSSELLDRLSEREAFGRAAEPWEVAATIAMLASDYTTYLTGEVVSISSQRA

>CORE_REP|Org102_Gene5292#

MPDNPAPANLTPAEPTAADRAPADSAPLNLAAGAPAPGNPAQPVAFVTGAARGIGAAIAQRLAADGATVAVVDLDENSCAAAVDTIVAAGGKAIAVACDVTAEDQVDAAVDRVAAELGSLDILVNNAGVLRDNLLFKMSVAEWDTVMSVHLRGAFLCSRAAQRHMVAQRSGKIVNTSSVSALGNRGQANYSAAKMGIQGFTRTLAMELGPYGINVNAVAPGFIVTEMTAATAARLGVSSEELQAKTAEITPLRRVGQPADIADVVAFLASENAAFVTGQTIYVDGGRRL

>CORE_REP|Org5_Gene5353#

MTDAADATPGAPGTGNTAEPDATTAAGASARTDAAPPMISMRNVDKHFGDLHVLRDVNLEVPRGQVVIVLGPSGSGKSTLCRTINRLEPIDSGTIAVDGVELPAEGRALAKLRADVGMVFQSFNLFAHKTILDNVLLGPVKVRRVDKKRARARAMELLERVGIADQADKYPAQLSGGQQQRVAIARALAMDPKVMLFDEPTSALDPEMVNEVLDVMVALAKEGMTMLVVTHEMGFARRAGDRVLFMADGRIVEDAPPETFFTAPASERARDFLGKILSH

>CORE_REP|Org19_Gene2600#

MRNPLATPTGCGCPARHRRRREQHCAARSRRRPGAGVPQDTGGVVTSAERPPAATRVLVVDDEPQILRALRINLSVRGYEVITAATGAAALRAAAEKHPDVVVLDLGLPDIDGVEVLAGIRGWSSMPVIVLSARTDSSDKVQALDTGADDYVTKPFGMDELLARLRAAVRRSASTAEESAPIVETSSFTVDLAAKKVIRGGRDVHLTPTEWGVLEMLVRNQGKLVGRRELLREVWGPTYATETHYLRVYLAQLRRKLEDDPSQPKHLLTEAGMGYRFQA

>CORE_REP|Org103_Gene1084#

MTDLTAAFAASVACGAMSSDLLGKSALVSGASRGIGKAVAAELLRRGANVLITARKPEPLAEAAAELRALGHQGEVATIAGNSGDAQARAEAVGRAVTEFGSLDILINNTGINPVFGALMDADLDAVRKIFDVNVVAALGYAQEAYKAWMGEHGGAIVNVASVAGLRSTGVIAAYGASKAALIRLTEELAWQLGPKIRVNAVAPGVVKTKFADALYSADEERAASVYPMKRLGSPEDVARLIGFLASDEAAWITGETVRVDGGLLATGGI

>CORE_REP|Org77_Gene3533#

MALEIDLSGRVVLVTGGVRGVGAGVSRALLAAGATVLACARRPGDAPVEYEGRQAEFLPCDVRDGDAVRELIDTVIARHGRLDHLVNNAGGAPFALAADASAKFHAKIVELNLLAPLLVSQLANAVMQAQPDGGTIVNISSVSAHRPSPGTAAYGAAKAGVDSLTASLAVEWAPKVRVNSVVVGPVETELSLLHYGDADGVAAVGATIPLGRLARPEDVGRCVAFLASPLAGYVSGATLEVHGGGERPAFLDAATVNTAAPNGAPKP

>CORE_REP|Org15_Gene4889#

MSDGTGLLADKVVVISGVGPGLGRSLCVQAAAAGAKVVLAARTESRLREVADEIDGAGGTSLIVPTDITDDAAVANLVERTVATFGRVDALINNAFAMPSMKSLARTDFQQISDSLELTVLGTLRATQAFTDELAKTRGAVVMINSSVLRHSEPRYGSYKVAKSALLAMSQTLATELGAKGIRVNSVAPGYIWADRLKWYFGEVAKKYGITVEQVYEQTASRSDLKRLPEPDEIARAVVFLASEWASAITGQTLDVNCGEYHA

>CORE_REP|Org37_Gene1288#

MPAPGRRSRRGPASARRIRGHGPAAPGHDGNGEPVSGRADSDGDTPIRVLLVDDEQLVRSGFRLLLDIEDDITVVGEAANGAEAVRKARALRPDVVLMDIRMPTMDGIQATREIAATTGLQDVRILILTTYDTDAYVFEGLQAGASGFLLKDAGPAELLHAIRVVAAGEALLAPRITRRLIAQFTARRAADRAAEQRLAVLTDREREVLALVGQGMSNAEIGAELFLSPATARTHVSRAMVKLGARDRAQLVVIAYRTGLVAP

>CORE_REP|Org114_Gene5983#

MLELTDVTKEYRVGEQTVRALDGISLRIEPGEFTAIIGPSGSGKSTLLHMLGALDSPDSGSIRFQDAEIGGLDDDRQSEFRRHRVGFVFQFFNLLPTLSAWENVAIPKLLDGTGLRKAKPRALELLELVGLADRAEHRPAELSGGQMQRVAVARALIMDPPLILADEPTGNLDSKTGASILELLGDITRQGNSVVMVTHDMGAVRYCDRLITLRDGKIGSNELVEHTENGEVRTVPVELTASLSEDGSEPAQAVRP

>CORE_REP|Org35_Gene4389#

MSNSVEDSRKSESQNAERASRSVLVTGGNRGIGLAVAQRLLADGHKVAVTHRGSGVPDGLFGVKCDVTDSESVDRAFSEVEAHQGPVEVLVANAGITDDTLLMRMTEEQFTRVIDANLTGAFRCAKRANRAMLRARWGRMIFLGSVVGLGGGPGQINYASSKAGVIGLARSVTRELGSRNITANVVAPGFIETDMTAELPEEMRETAKKFIPLQRLGAPEEVAAVISFLASEDSRYVSGAVIPVDGGMGMGH

>CORE_REP|Org2_Gene6607#

MNSLTPAVSLLTSNNDGVNTTSSSVPAASVLVAEDDPHVRSTLDQLLRFEGYQVYLAADGQEALELLAQQRPDLAVVDVEMPRLDGLSLCRLLRRRGDRLPILVLTARQQIGDRVAGLDAGADDYLPKPFATDELLARLRALLRRSTFDEDDDTVLAVGDLTLNTATRQVHRGDRPIELTKTEFDVLELLLRNARIVLSRSRIYEHIWGFDFDTESRSLDVYIGYLRRKTEENGEPRLIHTVRNVGYSVRPA

>CORE_REP|Org5_Gene560#

MSRMNGVAGDRIPEARVLVVDDEPMIVELLSVSLRYQGFEVAAAGNGAEGLDRAKQFRPDALIVDVMMPGMDGFGLLRRLRADGIDAPVLFLTARDEVDDKITGLTLGADDYVTKPFSLEEVVARLRVILRRSGHVVEETKSSRIRFEDIELDDDTHEVWKAGEPVALSPTEFTLLRYFMVNAGTVLSKPRILDHVWRYDFGGEVGVVETYVSYLRKKVDTGPDRLIHTLRGVGYVMRAPSRSRSSAK

>CORE_REP|Org31_Gene655#

MVPLPDAYRTSELVSTPKVLVVDDDEDVLASVERGLRLSGFHVLVARDGAQALRSVSEHAPDAIVLDMNMPVLDGAGVVTALRAMGNEVPICVLSARASVDERISGLESGADDYLVKPFVLAELVARIRALLRRRTDTPPAATPGAITVGPLEVDIAGYRAVLHGNEIELTKREFELLSTLARNVGVVLSRERLLELVWGYDFAADTNVVDVFVGYLRRKLEVDGAPRLLHTIRGVGFVLRAPK

>CORE_REP|Org114_Gene4108#

MSANLMIVEDDDRVRVALRLAMEDEGYDVAEAEEAEVALRQLRDNGAPDFMIVDLMLGGMDGFTCIREIRRDHDVPIIVVSARDDTHDVVAALEAGADDFVTKPFEVKEITARMRAVARRARFAEQAAAEEDPDSELGTMVLDEQAGNPLVLSTESGIVRRGDEEIHLTLTEYRLLCELAGSAGRVLSRGTLLERVWDRGFFGDERIVDVHIRRLRTKIERDASDPQLIVTVRGLGYRLDVQR

>CORE_REP|Org44_Gene3372#

MTSVLIVEDEESLADPLAFLLRKEGFEVTVVGDGPSALAEFDRSGADIVLLDLMLPGMSGTDVCKQLRTRSGVPVIMVTARDSEIDKVVGLELGADDYVTKPYSARELIARIRAVLRRGAGDELDGNGESGVLEAGPVRMDVDRHTVMVNGKPVTLPLKEFDLLEYLLRNSGRVLTRGQLIDRVWGADYVGDTKTLDVHVKRLRSKIEADPAKPEHLVTVRGLGYKLEA

>CORE_REP|Org96_Gene3994#

MITMRNVTKSYKTSTRPALDNITVDVDKGEFVFIIGPSGSGKSTFMRLLLKEESPTAGEIRVADFRVDRLPGRKVPKLRQRMGCVFQDFRLLQQKTVQENVAFALEVIGKRRQVIERTVPEVLDMVGLGGKADRLPSELSGGEQQRVAIARAFVNRPLVLLADEPTGNLDPDTSGEIMLLLERINRTGTTVLMATHDNHIVDAMRRRVVELDHGRLVRDEATGVYGVGR

>CORE_REP|Org7_Gene3709#

MTAVLLAEDDEAIAAPLSRALGREGYSVTVERFGPAVLERALEGHHDLLILDLGLPGMDGLEVCRQVRASGADIAVLMLTARTDEVDFVVGLDAGADDYVGKPFRLAELLARVRALLRRSGIGDDTVEVGGIRLEPAARRVLVNGAEIGLANKEYELLKVLIDRAGQVVPRETILREVWGDAELRGSKTLDMHMSWLRRKIGDEGPMAERRIVTVRGVGFRLNT
