# Supplementary material for: Design of a multi-epitope vaccine against six Nocardia species based on reverse vaccinology combined with immunoinformatics
Source: Front Immunol. 2023 Feb 2;14:1100188. doi: 10.3389/fimmu.2023.1100188 (PMC9952739; doi:10.3389/fimmu.2023.1100188)
Supplement: Supplementary file 14 [file Table_7.docx]

>CORE_REP|Org119_Gene7073#

MSSLATDSVDQCSSGESSAQPFVLSPAQTALWYAQRIRPDVPLTIAQYVEIHGDLDVGRLLYAIERFGAESEVGKLRLAEIDGIPHQIVDPARRPGWARVDLRGERDPHAAALRWMHEYTGSPIDLERDPLTANVVLRTGDSDYIWYSRAHHIVIDGYGAMNALTRTAEIYTALENRTEPVVSRAAPLAEIYADEVRYRETSRFRADRDYWLEQLAGAGEPMSLGGSTVTAATQDAGRRIAAGVLDDRAQAAMDAAVTTFGTANSALFVAALGAYVRSVTGNPDVVLSLPVSARTTVSLRRSAGVVSNVVPIRLRFGAETTLAEVVKATELQITGALRHQRYRHDDIRRDCGYSRDARGFFGPMVNIMLFHDELTFGSLVGSLNVLATGPVEDLSVNLYNGVGGRIHVDFEANPRLYGEAEVSVHHDRFLDFLTRFLGAAPDTHAETLTAITAAEHERVLHEWNATEAPRQPGTLAELFAERAAACPDAIALESGDDTADPSPVHPVTTLTYRELDERANRLARLLIERGAGPETVVGLCLRRSIDLVVGMYAIVKTGAAYLPLDPEHPADRLDQIVRQASPVCVLTAARDELAMPESAAALAIDTVELSGYRGAPITDAERTAALRADHLAYVIFTSGSTGKPKGVGVSHAAIVNRLRWMQHEYSLDRTDVVLQKTPATFDVSVWEFFWPLQIGARLVVAAHDGHRDPAYLARLIAEKGITTAHFVPSMLSVFVTDTDVRGCTALRQVFCSGEALPAATVRDFHAALPRPALHNLYGPTEAAVDVTYWPCPADPATVPIGSPVWNTQTYVLDSRLRPVPPGVVGELYLAGVQLARGYLGQPRLTADRFVANPFGAGVRMYRTGDLARWQLGTDRPGVLEYMGRSDFQVKIRGLRIELGEIEAALLDDARVARAVCVAHPGRNGDELVAYVVATPAAGRLDTTALLTELRRTLPAYMVPSALLELDELPLSANGKIDRKALPAPVGVRATGRSTAEPRTEVERVLARVFAEMLGTEVGVEDSFFDLGGNSLVAARAVARINAALGTGLTIRDLFEASTIAALTQRFATHPADVSSPKLVAAQRPERIPLSLAQQRLWILNRFAEHAAAYNMPLAVRIEGALDVEALRAGLVDVIERHESLRTTFPESAEGAVQLVHPAAEIPLTLDPIDAAGADVAELATEFAGYGFDLRSQAPIRVALYRTGPDQWVFLVVLHHICGDGWSIAPLARDLMTAVAARGAGAAPQWAPLPVQYADFALWQRELLGNESDPASALSGQLTHWRSALAGLPDQLDLPLDRPRPLRRSTTGGRVDFTISPEIRRAASELAAARGVSMFMVLHAALATLLSRLCASTDIAIGTPIAGRSDPALDELVGMFVNTLVLRTEIDPAAGFDRMLDVVRETDLNAFANADVPFERLVEVVNPERSAARHPLFQVMLSYDRDPDLRIELPGVRAEVLPIVSDIAKFDLQLVVHDDVTDGPLTAEFGYATDIFDRATVESFARRFVAVLNAVVAAPSMPIGDLSILDRREIANLVPIAGAPAEPFTTLARLLTDTAERVPDAVAVRYLGVDTTYRELDESSNRLARVLIEHGAGPEVVVAIALPRGLDAITAVWAVAKTGAAYVPIDPSYPGERIAHMIGDSGAILGLTDAACLAAMPEWPAPRGKHRKNYVDWLVLGSAELAAEAVHCSTAPITDADRHHSLCTVHPAYLIYTSGSTGKPKAVVVTHAGLASLANEQTHLFGVTDSARTLHFSSPSFDASVLELLLGFAAGATIVVAPAGMYGGAELATLLRTERVTHAFVTPAALATVPTDGLDELEAVIVGGEACSEELVETWSAEHRMHNMYGPSEATVAATATGPMVPGRPVPLGQPIRGMRLFVLDGRLHPVPPGTPGELYLSGPGLARGYHGRYGLTAQRFLANPHGRRGERMYRTGDLVVVETGGQVRFLGRADDQIKIRGFRIELREIDHVLRAHPGVNFALTVVHTDEHGQPRLASYVTVDHPVAAADLTETARQRLPGYMVPASVTVLAELPVTPAGKLDRKALPEPVFATGGSSRAPATELESRVAGVFGEILGRPVTGAEDSFFDVGGNSLLATRLAAALHAEFGVDLPVRVIFEAPTVAGVAERLTEAPRTQRLALAVQTTRPGRIPLSLPQQRLWFLNRYSPESSAYNIAFVIRIAGDLDVAALRAALTDLVERHEVLRTVFPEDSAGAQQVVLPTARALPAIEAIDTDEAGATAALGALAHRGFDLIRDTPLRMTLLRTGSERYLLGIVVHHIAADGWSLGPLTRDLAAAYVARHGGAAPAWTPLPVQYADFGLWQRACLGDEGEPGSLAAEQLAYWRSALADLPAELPLPYDRPRPAEPTQYAGAVPFTVPDPVQRALAELAKEQGVSMFMVLRSALAVLLRSVTGGRDIVIGTPVAGRTDTKLDELVGMFVNTLVLRSDVDPDRPFAGLLRADRDTELAAMAHADIPFERVVEELASGTTRGRHPLFQVALTVQDGPVPTLELPGLELRAEELDIALAKFDLELRVAHIGCDAGPGEPGRAFEFVYAAELFDEATIHTLADRFLRVLAAVTADPRVLVRDIDTRTERERRLLAPATGGPTTPQCTLAAYFTATAHMHPHRTAVRSGATTLTYAELDKRSNRLARALLARDIGIGDRVALGLTRSVESVLTVLAVVKTGAAFVPVDPNYPADRVRHMLADAGCWVGVTVGAHAERLRTAAADGPATDWLLLDDPAVRAELETYDDALVDDLDRMCTIEAADLAYLIYTSGSTGKPKGVAVTHAGLSNFADELRDRMRVDRESRTLHFASPSFDAAVLDLLLAVGSGAAMVLCPPDVYGGDELAALLERERITHTFMTPAALATIDHERWPLPHLRALMVGGEACAPDLVARWAPGRTMLNGYGPTETTIVATIATLTAEQPVTIGTLVRGARALVLDERLRPVPAGVPGDLYLGGHGVARGYFDRFGLTALRFVADPFGPAGARLYRTGDVVRWNDAGELCYLGRSDHQVKVRGFRIELGEITAALGEHPAVRFAHTEVRQIAGADRIVAFVQPADEHTGVDVEAVRDRLGAQLPAHMVPASITVLERIPLTPVGKLDSAALPEPQLAVAAATREPSTPSERLVARVMGELVGVDAVRADDSFFDIGGNSLLATQLVARLAAASNTRLEVRTVFAAPRVAELAAHLDSGPAGARSRPALVRQARPDRIPLSAAQRRLWFLNRFNGIGEAAADGADLSAGAYNVPVVLRMNGKLNVDALVVALHAVQDRHETLRTVFPEVGGEPTQRVLDLVTAAITLFVATVRPDEVDDAVRRFAAPGFDLAGVVPMRAALISVSPDGDRGVRNPAEVSDEHVLVLVVHHIAMDGQSLAPLALDVATAYRAACADRSPEWDELAVQYVDYTLWQQDTLGTEDDPDSVIRRQLDYWRHQLDGVPELLTLPADRRRPPVPSYRGGLVECEIDAFTHRDLHRVATSNNVSMFMVLHAALAVLLHRMSATDDITVGTPIAGRGHPALDRLIGMFVNTLVLRTRIDPDARFTDLLHTVRDVDLDAFAHADLPFERLVEVLNPARSQAHHPMFQVMLSVQNHPVGGLELPGLRIEAADVDTGIAKFDLQFTLTEAQTPERDPAGITLSVNYASDLFDEQTALRLGHRLARLLAAVAANPTTAVGDLELLDPAEWSGLAPVRGAEPDRPVTFPEVFAAAAAVDRAAIALRADGTQISYDALDRWTNRLARVLMRRGVGPETLVALGIPRSVESVATVLAVAKAGAAFVPVDPNYPAPRIAHMLSDSGAALGITLSAHRDELPGDVEWIVLDDPIFRGLVLDSPDGPIAAAERTAPLRIDNPAYVIYTSGSTGTPKGVVVTHGGLSNFAAETAQRFDVRPGCRVLHFATPSFDAAMLDLLLALGGAATLVITPPGVVGGEDLARVFIDEAITHAFITTSALGTVDPTGVTALRHVLVGGEALPPDLVTRWAPNRNLYNVYGPTETTIVTVISQPMTPGGPITIGGPIRGVSATILDGRLHPAPVGVTGELHLAGSALARGYLNRPGLTAQKFVANPFGKPGERMYRTGDLVRWWTGQGSPAAGRDHGGSREIEYVGRTDHQVKIRGFRIELGEIDAALAKHGGVEFATTIGHRTPAGSTALVSYVKARNGIGLTAAELTEHVAGLVPNYMVPQSIMLLDRVPLSPVGKLDRKALPEPVFSAADGYRAPATPTEVALCAAFAAVLGVETVGADDGFFELGGNSLLATKVVAQVRANGLDLPVQAMFGEATPAAIAARLDGSGAGIVAALGPVLPIRPNGKAAPLFCVHPAIGLAWCYSGLLAHLAPDRPVYGLQAPHVAGEDGFASIAEAAQQYVAHIKSIQPTGPYHLLGWSLGGLIAHEVAVQLQEAGDEVALLSMMDSYRLSDAWLEHAIPSVAEIIEEFGSDQLDAPLDPAMNLRDAAELLRARPGPFAALTVEHLERLYAGYTNGTLLAHGFRPRVFDGDLLFFTAAADEINRADPERTAAAWQPFVTGAIRDHELPCRHSAMTAPESLAAIGQVLRGALDGAAVLLPAGAQPAKNGVRRTKSGARKEKQR

>CORE_REP|Org107_Gene6262#

MATEGFVRRPRIAPPRAPGGEVALTPPPEVTRALPAPLMMKLMPVVMVVAVIGMIAMMAMMGRNLLANPLSMMFPMMMLMSMVGMMAGFRGGTGKRAVELNEERKDYFRYLDQVRKDVRRTGNKQLETLVWSHPEPADLPSLIGTRRMWERRPNDPDFGHVRVGMGSHRLATKLARPETGPLEDLEPVSTVALRRFVRTHSVVHGLPTAVSLRAFPAINISGSPEDSRMLVRSMLMELVTFHGPDHLAVAIVCADPDGAWGWAKWLPHLQHPTQRDGMGSARMMYTSLGELETALAAELMERGRFMRNPQPTQGRLHLVVIIDDGYVNGNERLISESGLDSVTVLDLTAPEGGLAARRGLQLIASDGDVSARSAAGVEKFATADMVSPAEAEAFSRTLSRYRLATAAQIVSLGEGSTADPGLMALLKIPDAAQIDPARVWRPRTARERLRVPIGITPDGTPVEIDIKESAENGMGPHGLCIGATGSGKSEFLRTLVLSLVTTHSPDALNLVLVDFKGGATFLGLDSLPHVAAVITNLEEELSLVDRMKDALAGEMNRRQELLRSAGNYANVTDYEKARAAGVPLDPLPALFVVVDEFSELLSQKPDFAELFVMIGRLGRSLHVHLLLASQRLEENKLRGLESHLSYRIGLRTFSANESRAVLGITDAYHLPSVPGAGYLKSDASDPLRFNASYVSGPYVAPQGTVTGEDGTPVGGQRLALFTAAPVEMPAPPEEEEASPLDLPPSPTNPMLELPPPPSALGLPGAPGSDEGIPDSLLDVVVKRLTGHGRPAHEVWLPPLDESPTVDMLLPDPDWRSPVNRHGQLWMPIGVIDKPYEQRRDVLTISLAGAQGNVAVVGGPQSGKSTTLRAIIMAAAATHTPQHVQFYCLDFGGGSMAGLVGLPHVGSVAGRLDSDRVRRTIAELTSLMRQREERFAELGIESMAEFRRRKFAAAAHVPEGAASSGNPLADDRFGDVFLVIDGWAVIREEFDVLESQINAIAAQGLSYGIHVIIGASRWAEIRPVVKDQIGTRLELRLGDPTDSEMGRRTAFQVPVGRPGRGLTPEQLHMLIALPRLDSDSDPSTLADGVSRARQELAELHAGRHAPEVRMLPMQFSRDELLATTRAQGIELSPTKVVVGLGESELQPLVLDFQTEPHFMAFADVESGKTTLLRNIVMGVVENSDPEQAKIIMIDYRRTMLGVVEGEHLAGYSTSSQTCGPMIQEVAEFLSKRIPGSDITPQQLRDRSWWEGPEIYIVVDDYDMVATGGINPFAPLIEYMPQARDIGMHFVVTRRMGGVSRALYDPIIGGLKNMSVDTLIMSGSRDEGKIIGEIRPSKLPPGRGTLASRSKGQEMVQIAYLPPV

>CORE_REP|Org63_Gene886#

MRGSQIALRTVPDAATAVLTKPPAAPVVTRNDFRTARLIALVAGLLGALFALATPFLPVTQTTAVLNWPQGGTLGNVQAPLMSQVPIDLKATIPCETIAQLPERGGMLLATAPPQGDRAALEAMFVRVSETSVDVVDRNAVVVSADRSRMGECAALSISSDSERTYAVFTGLTKQVERPVEGGAPGATELATVPVEGQLGGDLRPQVVGVFSDLKGAAPAGLAFDMTVDTRFSSSPTAIKLVAMIAAVLCTLIALAALARLDGSDGRGHRRFLPANWLKPTWADGAVAGTLLLWHFAGANTSDDGYILSMVRVAPHAGYMANYFRWYGVPEAPFGWYYYVIQVFSEISTASPWVRLPALACAILCWLVISREVVPRLGRGVRTSKVALWTGGLVFLAFWLPFDNGLRSEPIVALGALLTWVSIERAIATGRLLPAAVAILVAAFTLAAAPTGLMCVAALLAGIRPLVRIVVRKHRQFAALGAGRWGSTLPLLAPIAAAGVLVLTVVYSDQTFAGIQEANRVRQVTGPNLAWYEDYLRYYYLFVETVDGSLSRRFAFLVMLLCLFTTMLVLLRRRQVPGIASGPTWRLMGVVFGTIFFMMFNPTKWTHHFGAYAGIAGSLAAVTAVAVSASALRARKNRAIFLAGLLFVLAVAFSGINGYWYVSSFGVPWFDKRISLQGYQSNTVMLMLFGLALALVGWYALREDYTKPQPSAKTARGRRIRRFAAIPLTVVAALMVALEVLSLVKGAVSQYPAYSLARSNIDALGGSTCGLANDVLVEPDPNGGRLEPIIDPARPLTDPLAGVDSVGFDPNGVPNDLSADSVEVKPGTGNTSTQSVGAAFAEGQSAGTGGGQGALGVNGSTVALPFGLDPASTPILGSYQNGMQQPANVTSSWYQLPARSADKPLVVISAAGRILSFDDTGAMKYGQSLTVDYGKHLPDGTVQKLGTYLPRDIGPFPSWRNLRVPLDEIAPDADAVRIVANDPILIGDQWLAFTPPRMPKLQTLNSLLGSQQPILLDWAVGLQFPCQRPFDHENGVAEVPGYRILPDRPLAISSTNTWQAEEFGGPLGFAQMLAKSTTVPTYLKHDWARDWGSLERYDQYDRNAVPAKLDTGTTTRSGLWSPGNLRVF

>CORE_REP|Org125_Gene1111#

MEIQAVTSPYDDGPNGGRPPRGPQSGPGGQPPRPAGGNPPGARPLPPRRQAPPPGPGGPRGGQPGGPPNPAGGPPRRPGPPPGGDRTPPMRGPAGGPPRTGGQPTVRGGQPNPAGGPPRRSANPAPRPGAGATQKIAKPGEQKPQATQKIAAGTLGEAMAQRGPRSTAANRSAPGGAGTRSGSGPGTGGRRAVAGGTPPSGPPPRKGNGGGDGPGGSAGKGPKTKKKSPWRIVRRVIYVLVALAIVVPSAVFLIAYTTVSIPQPGDLKTPQVATILASDGTTQISKIVPPEGNRTDVTIDQIPPHVRNAVIAAEDRDFYSNPGFSISGFARAARDNLMGKDTAGGGSTITQQYVKNAMVGNQHSLSRKMRELVISAKMARQWSKDDILTAYLNTIPFGRGTFGIDAAAKAYFGKSVEQLTVEEGAMLAATINQPYGLDPENNPKGAEQRWNYVLDGMVKAGSVPAAERAKMVYPKVLPSSANNDDSESKTAGPNGLIKRQVLSELSEAGISDTQLNTEGLQITTTIDQKAQQAAIDSVHKNMQGERDEVRTAVVSVDPKSGAVRAYYGGDNATGWDFANAGLQSGSTFKVFGLAENLELGKPLSTMYDSSDLTVNGIKITNAEGETCGTCTIAEALKRSLNTSFYRMELDMPDGPAKIAAMAHRMGIPDTIPGVGQTLTEPDGSGPNNGIILGQYQVRPLDMASAYATIAASGVYHKPHFVQKVVTADGQVLLDRGQVAGEQRISAAVADNLASAMQPIAASSRNHGLAGGRPSGSKTGTTQLGDTGQNKDAWMIGFTPSLSTAVWVGTADGVALKTPGGSIMYGSGLPSDIWKDTMDGALEGTPKENFPKPAAIGGQAGVPSYSAPYTAPTTTQQEYQPPVVVKPSQVEILPGITIPVPGIQPNPRSQPQQNQPQSQDTGPLPGQPVAPADGSSPSTSNSGDTSGNSRSQRPGAGVGNSTDGTGDGYTNSHR

>CORE_REP|Org144_Gene202#

MTDTTLPPFGGSGGDRIDPVDIQQEMQNSYIDYAMSVIVGRALPEVRDGLKPVHRRVLYAMYDNGYRPDRGYVKSARPVAETMGNYHPHGDASIYDTLVRMAQPWSLRYPLVDGQGNFGSRGNDGAAAMRYTECRLTPLAMEMLREIDHETVDFIPNYDGRSQEPTVLPSRVPALLMNGSNGIAVGMATNIPPHNLTELAEAIYWALDNHDADEEATLAACMERVKGPDFPTHGLIVGSQGIHDAYTTGRGSIRMRGVVEIEEDNKGRTTLVITELPYQVNTDNFINSIAEQVRDGKIAGISDIHDESSDRAGMRIVVTVKRDAVAKVVLNNLYKHTQLQTSFGANMLSIVDGVPRTLRLDQMIRLYVKHQLDVIVRRTKYLLRKAEERAHILRGLVKALDALDEVIALIRRSANTDTARTGLMQLLDIDEIQATAILDMQLRRLSALERQKIIDELAKIELEIADLKDILAKEERQRAIVRDELAEIVEKYGDDRRTRIIAADGDVADEDLIAREDVVVTITETGYAKRTKTDLYRSQKRGGKGVQGAGLKQDDLVKHFFISSTHDWLLFFTNKGRVYRAKAYELPEANRTARGQHVANLLAFQPDEKIAQIIQIKNYEVAPYLVLATKNGLVKKSKLSDFDSNRSGGIVAVNLRDEDELVGAVLCSADDDLLLVSALGQSIRFSATDEALRPMGRATSGVQGMRFNASDELLSLNVVRPDTYLLVATAGGYAKRTAIEEYTPQGRGGKGVLTVQYDPKRGTLVGALIVEDDDELYAITSGGGVIRTVAKQVRKAGRQTKGVRLMNLGEGDTLLAIARNADEPDPDLLAGDTSDTGSSE

>CORE_REP|Org158_Gene3705#

MTQHLEQANAGQSNNDASATPPTPNSMPQRQGDPTSQRQSDPTSQRQGDPAAQRQGDTTAAQRPGDTTAQRPSLPVAQRQGGAPAAVPTSASRRVRARLARRMTGQRGIAAVKPVLEPLATVHRELYPKANLQLLQRAFDVADEKHAHQFRKSGDPYITHPLAVANILAELGMDTTTLVAALLHDTVEDTGYSLDELTNEFGQEVAHLVDGVTKLDKVNLGAAAEAETIRKMIIAMARDPRVLVIKVADRLHNMRTMRFLPPEKQAKKARETLEVIAPLAHRLGMATVKWELEDLAFAILHPKKYDEIVRLVADRAPSRDTYLAKVRAEIVNTLAASRINAIVEGRPKHYWSIYQKMIVKGKDFDDIHDLVGIRILCDEVRDCYAAVGVVHSLWQPMAGRFKDYIAQPRYGVYQSLHTTVVGPDGKPLEVQIRTQDMHRTAEFGIAAHWRYKETKGKHSNDSTEVDDMAWMRQLLDWQREAADPAEFLESLRFDLKSPEIFVFTPKGDVITLPQKSTPVDFAYAVHTEVGHRCIGARVNGRLVALERQLENGEVVEIFTSKAQNAGPSRDWQNFVVSPRAKAKIRQWFAKERREEALEAGKEAISKEVRRSGLPLQRLMSADAMSALAHELHYPDISALYAAVGESQVSAHHVVQRLMAQLGGVGDVENELAERSTPSTVPARQRGTGDAGVEIPGASGTVAKLAKCCTPVPGDEIMGFVTRGGAVSVHRTDCTNADSLRSEPERIIEVKWAPSPSSVFLVAIQIEALDRTRLLSDVTKVLADEKVNILSASVMTSGDRVAISKFTFEMGDPKHLGHLLNVVRNVEGVYDVYRVTSAA

>CORE_REP|Org97_Gene925#

MGRAQDRARIFWYARPCGSLATSCHGAPPTAHRACDGGPIRLGRVPISQTLARLAGACVLAAVLVAGLLFPLAGGFGYMSNRAADAVDNVSAELVAGTAPAVSTMVDATGAPIAWLYEQRRFEVPSDKIANDMKLAIVSIEDKRFAEHGGVDWQGTLRAFLTNTSSGEVQQGASTIDQQYVKNFQLLVVAKTDAERRAAIETTPARKLREIRMALTLEKELTKDEILTRYLNLVPFGNGSYGIQDAAQTYFGVDAKDLKVAQAAMLAGMVQSSSKLNPYTNPKGVLERRNTVLDTLIQNIPSRADEFRAAKEQPLGVLPEPKGLPRGCIAAGDRGYFCDYALQYLANAGISKDQMDKGGYLIRTTLDPAVQNSVKAAVTANTDPNLENIAEVTSIIAPGQDSHHILAMTSSRTYGLDQGAHQTVQPQPYSMVGDGAGSIFKIFTTAAAMEKGLGTSAQLDVPSFFAAKGMGNGGAAGCPPATYCVKNAGNYRSPMSVTEALAQSPNTAFVKLIQDVGVTPTVDMAVRLGMRSYAEAGTSGHGNQSLADMIKQQNLGSFTLGPVAINPLELSNVAATLASGGKWCPPSPIAEVIDRDGKQVPLTQQACEQVVEPGLANTLANALSQDAVGGTAAGSARAVGWNAPVSAKTGTTETHRSSAFLGFTNSMAGAAYIYGDSPTPGEICSFPLRTCGDGNLYGGNEPARSWFGGIKPVLDKFPPPALPPLDDKYVRGSNNAQIPDVNGMSESEARSVLIGAGFQVSTVTTPGSAAKGTVTATTPNGSAIPGSVITVLVSDGTQREIPKPGPPPAPPVLPGLPQIPRLPPIPIPIPR

>CORE_REP|Org141_Gene5255#

MVSGSLLLCGIDLTVLHVAVPSVSRDLRPSAAQLLWIVDVYSLALAAMLVTCGTLGDRVGRRRMVLSGFLTFGLASAACALSTSTAQLIAARAALGVGAAMIMASTVAIIRVVFTDGRERAFAIGVWTSAHSVGATIGPLVGGLVAERWGWNAVFLVNIPVIIVILAVGARVIPESKNPAPRRWDLASVALSIAGLASVVYALKQAGEHAGVSTAILVTALSGAALLYAFVHRQRRLAEPLLDLSLFADRRFATAAVCVIGCFGSYVALLFFLTQWLQQVGGYSPLHAGLALMPLAAANAVGAVTAPRTASRWGNRGALTAALLLFALAYAVIAAVGDTAHYGTILPALLAAGYGAGIVMTLGADAIMSAAQPERSGEAAAIQETSFELGAGLGVAVLGTVMTVVYRTGMPHVPGLGPDERVIVGESFTAAQDLTAHLPSATADAVLDAARQSYDHGFTTIAVIATVTLVITAAMAAVLLRCKQNEPRNYRFQGGQASVPDTTHPPVTVLGLGAMGQAFVATLLKGGRTVTIWNRTPGKDAELVTAGARTTAAVDEAVTASPVIIAVLLDHRSVHSTLDPIADQLAGRQLINVTSTTAEESRELAFWAAGHGIEYLDGGIMAAPSMIGQPGASILYSGSRAVFDDHRGTLDLLASAEYFGTDAGMASMLDFSLLSAMYGMYGGFFNGVAMTRSVGVSAEAYAERAAAWVKAMTDYLPMLGKLIDARDYENGVQDIAFHKAAVDAIVRATRDAGAAPDFLAPLQHLIDRQIAEGNSALAFEHTVEEIV

>CORE_REP|Org46_Gene6654#

MASSCSPPNKPLPGDRAGHHGVAVTGRTTRVAPQAATARAASRSSGASTSERQETVAYRADLDGLRGVAIGLVVIFHVWFGRVSGGVDVFLVLSGFFFTGLLLRRADSTGSPGVGTTLRRTVRRLLPAMVVVLAAVVVASVIVRPYTQWWELSAQTLSSLLYVQNWRLALTWSDYLAADPSVSPLQHLWSMSVQGQFYLAALATVAVAAWTTRRSMRSAALRPVLAVTVGVLGVVSFWYAWRGGQTQQGWNYYDSIARCWELLAGALLAAIAPLLSPPRMARAGLAALGLFGVVGCGWLILDGANRFPGPAALLPVAAAAGVIVSGNNLPLDQRPWPNRILATPTARWLGDIAYPLYLWHWPILIFYLTERGQPHAGVAGGIVIVTLSIVLAWVTHRWVEEPLRLRSRPRAEAAGAEGTTISRRVAGVAVVALGAVVIAAAGGWLTVMARINPPHAVGALDPRLYPGAEALASGAAVPQAPMRPTVFEAPGELPPPTVDGCIADWDTREVITCTYGVPDAERTLAVVGSSHAEHWLPALQVLAGEYSFRIQVYLKMGCPLTLAEDAMYKGEPIPDCRDWSREVIDRLGADRPDWVFTTGTRPREDIGDETPPEYLDVWSALSERGLNVIAIRDTPWLRREKVRYMAIDCLAKGGDRIGCGMRRQDALDEVNPALEPASRYPNVFPVDLSDAVCEPTVCAVIEGNVLIYHDEHHFTVSYSRSLADALGRRLQPLLGWW

>CORE_REP|Org24_Gene1345#

MCGLLGYLTVDTSGAPEGTTAEAIAAQLHEALVCQRHRGPDERGTWHDEHMVFGFNRLSIIDIEHSHQPLRWGPPENRQRYAMTFNGEIYNYLELREQLTAEHGAEFGADPMFATEGDTETIAAAFHYWGPEAAARLRGMFAFAIWDTETRKLFIARDPFGIKPLFLATGPGGTAFSSEKKSLLDLLPQLGLSDALDPRALEHYTVLQYVPEPETLHRDVRRLESGCYAWVEPGQAPKITRYFDPRFRVVPFAKPGEVTAQPPTTRPRPAAQRPNTAEYRYREIAEALEDSVAKHMRADVTVGAFLSGGIDSTAIAALAIRHNPNLLTFTSAFEREGYSEADVAAETAAAIGAKHYIRTVSPEEFAASIPEIVWYLDEPVADPALVPLYFVAKEARKHVKVVLSGEGSDELFGGYTIYREPLSLKPFEYLPKPLRRLAGRLSERIPDGTRGKSLLHRGSLTLEDRYYGNARSFNDAQLRSVLRDFRPEWTHRDVTDPIWAMQGRDWDPVARMQHLDLFTWLRGDILVKADKMTMANSLELRVPFLDPEVFAVAEKIPVDQKITKDTTKYALRRALEDIVPPHVLHRAKLGFPVPLRHWLRGPELYDWARQQIIDSATDHLLDKTAVLGMLDAHRAGTSDHSRRLWTLLVFMIWHGIFVEQRIKPEIQEPTYPVSL

>CORE_REP|Org112_Gene6280#

MSRGRDSGRDRAENGRGRGRFGGGRGNSGGGRASSGKRAASARSAAGPARARKPSRPRPAPGLDASTRFRFGVGRIVMLVALLVAALQLLWIQSVSAPRLSAEAASQRTVHQIDAATRGPILDRNGKSLAFTVNAKALTFQPVRVRKDLQEAHDENSAKPEPDQRMQAIAKYIHDKLGTAAPEQDLLKKLRSDEPFVYLVRNVDPRVAADISLKFPEVGTERQDLREYPGGSLAANVIGATGWDGHGQIGLESALDAILAGTDGSHTYDRGSDGAVIPGSWRDRQPAVNGYGVELTLDSDLQYYVQQQTQQAKELSGAQAASAVVLDARTGQVLAMANDSTFNPALGPQHWSSSSLGNPSVQEVYEPGSVNKIVTAAAAIEYGLTTPDEVLQVPGNIFMGGVTVNDAWQHGVMPFTTTGIFGKSSNVGTLMLAQRIGEDRYYDMLQKFGLGQRTGVGLPGESAGVVPSREQWSGSTFANLPIGQGLSMTTLQMTAMYQAIANDGVRVPPRIVKSKIDPDGNRTEEEPPEGVRVVSPETAATLREMFQAVVQRDPMGVQMGTGVPAAVEGYQVAGKTGTAQQIDPGCRCYSTSSYWITFAGMAPADNPRYVIGLMLDAPVRSSDGSGGQSAAPLFHAIASWALQRDRVPPSPPAKPLILQAS

>CORE_REP|Org8_Gene457#

MSRTRGTWTSVVAAILLVAGMATACSSDDTDEAADVCATTPNGTLVAASPTGPTGSKDISTNPELSTGYRSGMVAARTKTFAVATANTLASKAACEVLRDGGTAADALITAQTMLGLVEPQSSGIGGGAFLMYYDAASKSVEAYDGREVAPAAATENYLRWVSDTDRTEPKPNTRASGRSIGVPGVLRMLEMVHREHGKTGWRELFDPAIGLADRGFSISPRLAAQVAEQAKNLALDEAAKAYFLNPDGTPKPADTLLTNPAMAKTLGAIASEGAQAFYTGAIAQDIVAAATSTSGGRTPSLITTADLAGYQAKKRTALCTDYRNHQICGMPNPSSGGSTVAATLGILENFDLAALPPDNLGAGSDTARNGGKPKAEAVHLIAEAERLAYADRNKYVADTDFVPLPGNSLQTLLNKDYLKQRSALIDRNRSMGTAQPGDFGPVPLGVGPQPPEHGTSHISVVDQYGNAAAMTTTVESEFGSFHLVDGFVLNNQLTDFSADPLGTDGAPVANRLQPNKRPRSSMSPTLVFDKAPDGARGNLTHVAGSPGGSVIIQFVVKTLVGMLDWGLDPQQAVSALSFGAGNSPATGVGGEHPSINTADNGDHDALVLRLRELGHQVSVAPQSSGLSALTRDGTAWVGGADPRREGAVLGDNR

>CORE_REP|Org15_Gene5535#

MSPCCVCLGGEGMTDETFDDYLDETGNIAIPEGRTLVDYVEKHTRNDANDLAYRYIDYSRERDGEYQDLTWKEFGVRLRAVAARLQQVTKPGDRVAILAPQGLDYVISFFAAIYAGTIAVPLFDPDEPGHTDRLHAVLGDCTPSAILTASSSAAGVRQFFRPLPAAQRPRIIAVDAVPDTLGESWVRPDLAVDDIAYLQYTSGSTRTPAGVEITHRAVGTNLLQMVHAINLDWNSRGVTWLPLYHDMGLLCVILPAIGGKYITIMSPSAFVRRPGRWISELAAVSDGAGTFAAAPNFAFEHAAARGLPKNGETLDLSNVIGLINGSEPVTTSSMKKFNEAFAPYGLPKTAIKPCYGMAEATLFVSATRAEDEAKVIYVDRNELNAGRVVKVDHSAPNAIAQVSCGYVALSQWAAIVDSESIDSPEGAQELPEGRVGEIWLHGNNIGIGYWGREEETRQTFKNLLTNRQAEGSHAAGAPDDAIWLRTGDYGVYVDGELYITGRVKDLVIVDGRNHYPQDLEFSAQEASKMLRPGFIAAFSVPANQLPAEVFAADSHAGLKYDADDASEQLVIVAERGPGAHKADSQPIADAVRGALSQRHGVTVRDVLLVPAGSIPRTSSGKLARRACRAAYLEGTLRGGYQQQAFPDAPDEE

>CORE_REP|Org105_Gene5106#

MYRTGHADAIYVAAGPNSSVSAAVMKISGFHAMTGNRQAQRAFDAGILSLGLSIDGQESTRDLEYAKLAFQRATEWDPTMCDAWLGRAAAGEVTDEVIRNLHRTSTSTLYREQRRLGLAPRALAGRFVSGLYIDYPLASYTEIWLAYAANLIGSKQYDEAERVLDELAEYRAGMLSDPDREIDDRISAYIRGVLHFNTQRWPDVMSVLAGSAEWEDPYLATGAHVMVGSACAQLGLFGEAIRRMEQAENGPIPAARTTAMFCRGLCLRETGSEDEAQALFEQVYSQAPDFTANTEAMRDKSYRITITTKESIDARTDRWDPASAPSVEQLQTADAEDRAKKILTEARAELDRQIGLTAVKTQVAKLQATAQLAKIRAEKGMASVPRGNHLAFTGPPGTGKTTIARVVAKIYCGVGLLKTDKVVEAKRMDFVGQHLGSTAIKTDKLIDTAMDGVLFIDEAYTLIQTGLSGGDAFGREAVDTLLARMENDRDRLVVIIAGYDGEIDRLLAANDGLASRFAKRLQFPSYTPPELGQIGKLIASSRDSELSEDAVRLLEQACERLYNSERTDQSGQPRRGIDLAGNGRFVRNVIEAAEEEREFRLANDESLDLTAVDESVLMRIEAPDMEAALAGVLSSLGVS

>CORE_REP|Org98_Gene7027#

MLTEIRIDGLGVIATATAQFHAGLTCLTGETGAGKTMVVTSLHLLSGARADAGRVRLGAPRAVVEGRFTVDDVNDAARAEVAQVLEAAAAEPDDDGSVIAIRTVGSDGRSRAHLGGRGVPASVLADFTASLLTVHGQNDQLRLQRPDQQLSALDQFAGDAVGTALRKYQVLRRSWLDARTELLERTARSRELALEADRLKHSLNEIDAIAPEPGEDVRIVDEVRRLSDLDSLRDAAATAHGALAGPADTPEDGSGALEALGTARARIEAADDPALVALAPRLADAIAVVIDVTTELSGYLSDLPSDPGALDSLLTRQAELKTLTRKYAPDIDGVLAWAQEARTRLGSLDVSEEALAKLAAEVDTAADRVREAAKKLSGVRAKAAGKLAAAVSAELGGLAMGKARLEVEVRPLLAGAQDTAPLTVDGQELHAGHTGIDEAEFRLSAHSGAQSLPLSKSASGGELSRVMLALEVVLASSDHGATMVFDEVDAGVGGRAAVEIGRRLARLARTHQVIVVTHLPQVAAFADTHLVVDKSDDGKGAVNSGVRALTNDERVVELARMLAGLDDTETGRAHAEELLATARAEKAGAEAATR

>CORE_REP|Org18_Gene2143#

MLSHPERLPLLTASTDGAVSSGPRGLPAEVSRRRTFAVISHPDAGKSTLTEALALHAKMISEAGAIHGKAGRKSTVSDWMEMEKARGISVSSTALQFNYRAAGSDIDNVINLVDTPGHSDFSEDTYRVLTAVDAAVMLIDAAKGLEPQTLKLFQVCRHRGIPVITVINKWDRPGRAPLELLDEIDERIGLTPTPLFLPVGIAGDFRGLLRRGPDGEAVEYIHFTRTAGGATIAPEESLTPEQAQAREGEAWETAAEESELLSATGQDHDQELFLAGQTSPVIYASAMLNFGVRQLLETLVALAPAPAGRRDVDGGMRETSDPFSAVVFKVQAGMDTAHRDRLAFMRIVSGEFERGMVVTHAQTGRPFATKYALTVFGRERATVDTAYPGDVVGLVNATALAPGHTLFVDKKVEFPPIPSFAPEHFAVLRAQSAGKYKQFRKAIDQLDSEGVVQVLRNDARGDASPVLAAVGPMQFEVVTARMQAEYNVETQMDHLPYTLARRTDAASAEELGRQRGVEVFTRSDGVLLALFSDKWRLQYIEKEHPGLTLEPLVATAD

>CORE_REP|Org15_Gene6278#

MSDPRASGVRGRESADSTEQLDTGDRVHVTKSTGARVETAVPSNGNESSPDTYWRRAGRFRHRISRRLSAVPLRVTLALALVSLTGLGLLISGVAVTSAMRNVLMDNVDRQLFGAAHDWAGPDAPPPQRLPGPVGRERPPGLFYVRIEDPSGKVRSLFPTGPSVPDFPADLGKHPRTIGSVGNPDEHWRAERVTTPGGSSWVAIRLSETENIIDRLIGLQVAVGLMVLAVLAIVAQFVIRRSLRPLGEVEKTAAAIASGDLHRRVPVQGTNTEVDRLSQSLNGMLSQIQSAFAATEASEESARRSEARMRRFVADASHELRTPLTTIKGFAELYRQGALADPDMFMDRIERESKRMSLLVEDLLMLARLDAQRPVERRPVDLLALASDAVHNARAVDAAQRPEEPRRPIDLEIRPGTGTLEVRGDEARLRQVLGNLVNNALLHTPPEAAVTVALTPAPDEVVIEVADTGPGLPTEDAERIFERFYRTDTSRSRDSGGTGLGLSIVQALVAAHGGTVSVRSAVGQGTTFAVRLPRSQE

>CORE_REP|Org29_Gene4463#

MARTTSKRQAKSGANETVAPLGSSRRGADEPAPMRPPTPLTRTVSLRWRVTLLAASVVAIAVAVTSIAAYAMVARALYGDVDAQLRARAATMINGDIDSMAFQSLGVATLFSNNIGVGLIYPFSVSSPPSTPEGERTLDSLPVYIPPQPTKPPIGTEEIAVAKGEHTSSLRTYNNQRVLARRMDSGVTLVISQRLEPTREVLDRLAWLLFVVGGCGVLLAAAAGTAVGRTGLRPIARLTAATERVARTDDLTPIPVTGDDELARLTESFNTMLRALAESRDRQRRLVADAGHELRTPLTSLRTNMELLIAAGRPGAPRIPDEDMAELRMDVVAQIEELSTLVGDLVDLAREDAPETVYERVDLGEVAERALERARRRRGSIEFVAALRPWFVYGHEAGLERAILNVLDNAAKWSPAGAQVRVSMAEVGRGLLELSVDDAGPGIPPAERELVFERFYRTTASRSMPGSGLGLAIVKQVVTKHGGTITIDTSERGGALIRIVLPGEAGAPVATAEDEPDP

>CORE_REP|Org19_Gene2296#

MGTETVENRRHRVVVIGSGFGGLFACKHLEHDNVDVVLISKTSTHLFQPLLYQVATGILSTGEIAPATRIVLRKHHNTQVILGEVHDIDLVNKTVTSKLLNQDTVTSFDSLIVATGAQQSYFGNDRFATYAPGMKTIDDALELRARILGSFEEAELAKTQEERDRFLTFVVVGAGPTGVELAGQIAELADRTLVGTFRNIDPRDARVLLVEGAGAVLAPMGPKLGGKAQRRLEKMGVEIQLNAMVTDVDARGVTVKDKDGTERRIESACKVWSAGVQASELGKMLAERSKGTETDRAGRVVVEPDLTIKGYPNVFVVGDLMAVPGVPGQAQGAIQGATYAAKQIKAEVAGKQTPDQRKPFKYFNKGSMATVSRFNAVCQIGKLEFSGFLAWLIWLVLHLYYLIGYRSRTVTVFQWFVAFLGRNRGQMAATEQWVFARLALEAMNGNETDARDVQAEVGNTTPPAAPGEPSAKSAAATPDGEKASGTSESAGSGESTATSKSGASSESTTSGSSQPKAG

>CORE_REP|Org142_Gene5069#

MLDEGRTQFYGGHVPAPLTTRQQVNGYRFLLRRLDHALVRRDVRMLHDPMRSQLRSLLVGAVLGLLVVAGAAILAFIRPQGAIGDAKIVMGKDSGALYVVVADNDGGNTLHPVLNLASARLISGSSESPASVKDDKLADMPRGPLLGIPGAPSALPGSAQGTSSEWSLCDTVELSITGSAASASGVDTAVLAARPDLSERIRRADPDEAVLVRRSDRTYLIYEGKRAQVDPENSAIARALSLSGERPRPAGAGLLGAATPVPPIAVPEIPNAGKPGPGALSDIPVGGVISVAATGRGERAELYVVLADGVQHISDFTADVIRTANSQGMSQIETVPPDALTGIAVLSQLPVDHFPAAAPTILSAEDAPVTCVSWSKTEQSDADAVDGPTDRASAALLVGARLPLPEGAQPVSLATADGSGDRVDQAYLRPSSGEFVHVTGMEPGSPRRGSLFYIADNGIRYGVPDIDTAMVLGLGDAPALAPWAIVGQLVPGPTLASTDALTRHDVLPQSN

>CORE_REP|Org5_Gene2466#

MSTTDHNPSGATQHMPTTVTSPQVAVNDIGSAEDFLAAIDKTIKYFNDGDIVEGTIVKVDRDEVLLDIGYKTEGVIPSRELSIKHDVDPNEVVSVGDEVEALVLTKEDKEGRLILSKKRAQYERAWGTIEELKEKDEAVKGTVIEVVKGGLILDIGLRGFLPASLVEMRRVRDLQPYVGKEIEAKIIELDKNRNNVVLSRRAWLEQTQSEVRSEFLHQLQKGQVRKGVVSSIVNFGAFVDLGGVDGLVHVSELSWKHIDHPSEVVEVGMEVTVEVLDVDLDRERVSLSLKATQEDPWRQFARTHAIGQIVPGKVTKLVPFGAFVRVEEGIEGLVHISELAERHVEVPDQVVAVGDDAMVKVIDIDLERRRISLSLKQANEDYHAEFDPSKYGMADSYDEQGNYIFPEGFDPETNEWLEGFDKQREEWEGRYAEAERRHKMHTAQMEKMAADAAAEAANGGGAGNYSSESGAQASSSSSSSSESAGGSLASDAQLAALREKLSGNA

>CORE_REP|Org5_Gene928#

MVRNGRAAGWLRVAVAGAVLGAASGAGPAVMVGAGPASAVAPPAIDDGALGQAQAVNAKNGPPDETEKRAICAEPYLTGAVPRDPPLPQRILDLDRAWKFSRGAGQKVAVIDTGVNRHPRLPDLQPGGDFVTAGDGTEDCDGHGTLVAGLIAARPSPEDAFSGVAPEAQILAIRQLSLQYEAKNHRDDDTGKVAAGGYGDVLTMAAAVVRAVDMGATVINISEVSCSPAGSGTADGPLGAAVKYAADRNVVVVAAAGNLDQSACSVQNQTSGWNGVSTVISPAWFSPYVLSVASTDPDGATSPFSIHGPWVGVAAPGRTIISLDSKPGGTGLVDTEHGDEGPLTIDGTSFSAAFVSGLAALVRSRFPDLSAAQVIDRIERTAHNPGAGRDDRVGFGLIDPLAALTAQLPPPADRTGALPRAIAPPAPDPGPDPVPRRVAVIGSIALLALLVIGWAAALPYRRGRPGRGTGDPADGFVGTAETASPERISASSGPAGTDSPGGE

>CORE_REP|Org5_Gene949#

MPSKPTTRWQVSGYRFLVRRMEHALVRRDVRMLHDPMRSQSRAYAVGLVLGIVALAGCGVLALLKPQGSIGDNKILLGKDSGAVYAVIDGVVHPALNLSSARLAVGEPAKAVSIKESELAKKPRGALIGIPGAPSSLNFDGSGKGRAWSICDGLKNDGSQDLSTTVIAGDPSLGSKASRLGEGAALLVQGRDAAYLVYDNQRARVDMNDPKVTEALGIRGKTPRPISPGLLNAIPEVLPIEPPKIVDPGGMPTYSLNNHRIGDVVHVATKDQYYVVLRTGLQSISPLTADIIRNSNTAVSTDPEIDQSQAVQQNVSNELPVQKYPVKAPTIVEAKDQPVACMSWKPVAGASDKTDGSKRATLAVITGYSLPIPDNAQTTPLAQADGSGQNVDAFYSTPGSGFFVQTTGIETDSQRRDSMFFIADTGVRYGIKDANAQKALGMDAEKAKPELAPDQIVGLLAAGPTLGRQEAMVAHDGVAPDPAPAKQLVQSKQDQQAQQQSPN

>CORE_REP|Org162_Gene2884#

MPADISAPPSRGPAPTGGKTPTVIRLLVLATFVVILNETIMINAIPRLMHDLDVTERAAQWVSTAFMLTMAAVIPVTGWFLQRVSTRQAYAIAMGVFLAGTALSAVAPTFAVLLVGRIIQAGGTAVMMPLLMTTLMTVVPEQDRGRVMGNVTLAISVAPAMGPVISGLVLQAGSWRWLFVLVLPIAGTVTWLGLRRLDNIGEPQTGDIDWLSVAFAAFGFGGLVYGLSKFETDHVAVPALLVAAGLALIAVFAFRQLRLQRSGVPLLDLRILLSGTYTKALVLMSVAFLAMLGSMILLPLYLQNLRHLSPLETGLLVMPGGLAMGLLGPTVGRLFDRFGGRPLVIPGAVGVTVALAGFTQISMSMPYWQLLALHILLMISLAGLFTPVFTLGLGALPPHLYSHGSSMLGTLQQVAAAFGTALVVTVMSARMTQLMETGTEPVTAQLDGMRLAFAVSAALSVLVIVTAILLPSRAPAPEETGEDDASEAETAESAAPLLVKD

>CORE_REP|Org3_Gene1380#

MTGPDETDGPDFAREAGNAEPEPQHGTGAPLGSGPSPVDLAEMALVEAELDRRWPETKIEPSLTRIATLMDLLGSPQQSYPAIHIAGTNGKTSVTRMIDALLTALHRRTGRITSPHLQLATERISIDNAPITPARYVEVYRELAPYIEMIDQQSAAAGGPAMSKFEVLTGMAYAAFAEAPVDVAVVETGMGGTWDATNVIDGQVAVITPIGLDHTEYLGPDLTAIAREKAGIIKRAPESLIPRDNVAVIAEQDPEAMDVLLRRAVEVDAAVAREGAEFRVLARKIAVGGQQLELQGLGGVYDEIFLPLHGEHQARNAVLALAAVEAFFGAGAQRQLDVDAVRAGFASVTSPGRLERMRSAPTIFIDAAHNPAGAKALAATLTSEFDFRKLVGVVAVLGDKDAAGILEALEPVFDEIVVTTNGSPRALDVDSLTDLAVQRFGDERVVPAYTLPDALETAIAIAEDVADTGEMVSGAGVIVTGSVVTAGAARALFGKEPA

>CORE_REP|Org210_Gene726#

MPQQTAVVVLAAGAGTRMRSKTPKVLHSLAGRSMLEHALHAANEIDPTALITVIGHDREQVGAAVNSVAAELGREITSAVQEQQLGTGHAVQCALTALPADFAGDLLVTSADVPLLDGHTLSALLDEHRSYQPRSAVTVLTFVPEDPNGYGRIVRDADGGVLEIVEHADATPEQAAINEVNSGVYVFDVAVLRTMISRLTTANAQHELYLTDVLKLAREAGNPVHGARLVDAAKVTGVNDRVQMAQAARTLNRYILERHMRAGVTVIDPATTWVDASVRIGRDAVLRPGVQLLGNTVIGEDAEVGPDSTLTDVLVGEGAKVVRTHGEGATIAAAATIGPFAYLRPGTIVGESGKIGAFVETKNASIGAHSKVPHLTYVGDATIGEYSNIGASSVFVNYDGVKKHHTVVGSHVRTGSDTMFVAPVTVGDGAYSAAGTVLRRNVPPGALAVSGGAQKNIEGWVQRYRPGTAAAQAAAEAIAADDRASQATEQKDGNTE

>CORE_REP|Org106_Gene2979#

MLENPAATTQFPVTQRAFGLAILVLSGLQLMVVLDGTVVIFALPRLQDQMGLSSAGSAWIVTAYGLTFAGLMLLGGRLGDAFGRKRMLIAGVGLFTVASLLCGLAHWQAMLIAARALQGAGAAIAAPVAFALVATTFAPGKARNQAIAIVGSMVGIGSVGGLVVGGALTQLSWRWIFLINVPIGALIILGAIYCLADTGHHRVALDARGAVLGTLACAAIVFGATEGPELGWSHPAVIGALIGGAILLVVFVIAERNVDDPLLPWSLFDSRDRVTTFVLILLAGGVLGAMTYFVAQFLQNVLGYGPLQAGVASIPFTVGIGIGGALASKLAMTVAPRWLLFGAALVLAVGLLFGSTLDGEVSYLPTLLPLLIVIGFGVGVAMVVTPLCVLVGVPPSDIGPLSAVGQMFMNLGTPMAIGILTPVAVSRTLSLGGTTGKVSAMTDAQIVALGEGYTLVLAVCAGVAAVIGLIALTLRFTPEQIARAQHAQEEAQRS

>CORE_REP|Org5_Gene6276#

MTVARSAESVHAVVKAYDVRGVVGEQIDAAFVRDVGAAFARLMRDSATRIAIGHDMRESSPELAAAFADGVLDQGLDVVHIGLASTDQLYFASGHLQCPGAMFTASHNPARYNGIKLCKANALPVGQETGLATIADELIEGVPAGPGPRGTATEQNLLEAYAEFLRGLVDLSGIRPLKVAVDAGNGMGGYTVPAVLGAVSQLTIEPLYFELDGSFPNHEANPLDPKNLVDLQKFVRETGADIGLAFDGDADRCFVVDERGEPVSPSAVTALVAERELAKEPGATIIHNLITSQSVPELVTELGGTPVRTRVGHSFIKQQMASTGAIFGGEHSAHYYFRDFWGADSGMLAALHVLAALGGSDGPEGGSLRNHEGPRGRRIEHSEKDDRTMSELSSSYSTYAASGEINSTVADAKDRTLAVVTAFEGRARSVDRLDGVTVRLPGNAWFNLRASNTEPLLRLNVEARSQEEVDALVTEILSIVRG

>CORE_REP|Org63_Gene113#

MDSVSQRLDLRPNRIAVLSVHTSPLAQPGTGDAGGMNVYVLQTAVELARRGTEVEIFTRATASNLPPVQEAAPGVLVRNVVAGPFEGLDKHDLPTQLCPFTAEVLRQEARHLPGYYDLVHSHYWLSGQVGWLARDRWRVPLVHTAHTLAAVKNAALAEGDCPEPATREIGEKQVIAESDRLVANTAEEARQLVELYGADPERIDVVPPGADLTLYRPGDKAAARAALGLSADEQIVAFVGRIQPLKAPDVLVRAAAEVLRADPERPLRVLIVGGPSGSGLDRPDALIELAAELGIAARVSFLPPQPPQRLVLVYRAADLVAVPSYNESFGLVAIEAQASGTPVLAADVGGLGTAVRHDVSGLLVPGHRTSDWANALRHLLDDPGRLHRMGERAVAHAANFSWAHTADGLLASYAAALAGFRDERSALGGRGLAHSLVRDDAYDRAAADRTNLAGERTAALLAESSQARSRALWRRRMGVRR

>CORE_REP|Org112_Gene2784#

MVATNTRETAESADAADSADTTAARPVKKAAAKKAPAKKAAAKKTAAKKTAAKKTAKATKATKAAKKAAPKKAGEGADGAETENLDDESLEIDDLGDLEVDEEDLGDEELEVEDDEAEDEAEEAEAETEEEADEPTAKDKASGDFVWDEEESEALRQARKDAELTASADSVRAYLKQIGKVALLNAEEEVELAKRIEAGLYATEKIREYADKGEKLNVQLRRDLNWIMRDGNRAKNHLLEANLRLVVSLAKRYTGRGMAFLDLIQEGNLGLIRAVEKFDYTKGYKFSTYATWWIRQAITRAMADQARTIRIPVHMVEVINKLGRIQRELLQDLGREPTPEELAKEMDITPEKVLEIQQYAREPISLDQTIGDEGDSQLGDFIEDSEAVVAVDAVSFTLLQDQLQSVLETLSEREAGVVRLRFGLTDGQPRTLDEIGQVYGVTRERIRQIESKTMSKLRHPSRSQVLRDYLD

>CORE_REP|Org13_Gene5085#

MEDHVTTPESVRLTSAVDPATAVSRLACSGHFSDYIVYERPGRWVFAAAPLGRVELDTDELRVSTSAGSARERWTGRPVDALERALDSLRVTCGPGASGTAYGWIAFEFCADALGAQRHLTERADLAHVIIPRIEVTVTESGVGVDGATAAEVEVIEQLLLSPAEPLPTPHPVDVRADTCGYRARVAAAVAEIAAGDYQKVILSRRVELPFRVDLPASYRLGRAHNTPARSFLLRLGGLAAAGFSPELVVSVDDEGVVTTEPLAGTRALGQGVAADLAARNDLESDPKEIVEHAVSVKTSFAEIASIAEPGTTTVADFMAVRERGSVQHLASTVRGRLARHRTRWDALDALFPAVTASGIPKRAAVDAVFRHDAARGLYSGAVVTLSESGSLEAALVLRAVYQDADAAWVRAGAGIVAQSRPDREFEETCEKLGSVAPYLVPAISHGPGSTPRSPMVTGRRSHRS

>CORE_REP|Org215_Gene1707#

MNSLFLLALAIVLVPLGGIFAALDSSLNTISAARVDDMVRAERPGAARLAHIITDRPRYVNLMVLLRVLCEITATVLLAAVLLDWMDQLWALVVTAAVMVLVDYLVIGVGPRTLGRQHAYSLALAASLPLQAIGTLLGPVSRLLILIGNAITPGKGFRNGPFASEIELREVVDLAGERGVVADDERRMIQSVFELGDTAARAVMVPRTEMVWIESEKTVAQAMSLAVRSGHSRIPVIGENVDDIVGVVYLKDMVPYADRSRKVRVHEVMRAAVFVPDSKPLDDLLDEMQRRRNHMAVLVDEYGGIAGLVTIEDVLEEIVGEIVDEYDQNEVPDVEDLGNGKYRVSARLSVEDLGELFGMAIEEEDVDTVGGLLAHELGRVPLPGSKAVAHGLVLKGEGGSDARGRVRVHTVVVKRAAEKTGAEKSDAGRSTSERVDGESAGVNGVGGSGANEDGEADD

>CORE_REP|Org101_Gene5725#

MTDQKPESFPLRRSVAASAMGNATEWFDYGVYAATATYLTDAFFPGELGTLGTMLGFAVSFVLRPLGGMVWGPLGDRIGRKAVLATTILLMAAATGAIGILPTHSSVGVFAPILLIGLRVVQGFSTGGEYGGAATYLAECASDKRRGFLGSFLEFGTLAGFVGGSATVLACQLAIGSDAMHDWGWRIPFLLAVPLGLVGWYLRSRLDESPVFTEVAEVAEQTDQEHRPGGLHGLRELVTTYRRELLTLGGLVVALNVVNYTLLTYQPTYLQKTIGISESGTTAMMLIGQTVMMVTLPFFGRLSDRVGRRPMWLFSLVGLAVLALPMYWLMGQGTAWAITGFIVLGLLYVPQLSTISSTFPAIFPTQVRYAGFALAYNVSTAAFGGTAPLVNEAAIESTGWSLFPAAYMIGASLIGLVAWCFLRETAGTSLRGTEVPDAGEDAPAIIPAGPAGAALAP

>CORE_REP|Org117_Gene5341#

MNRRVRDSGRDDGYGRRTALAREWESGVETLLVVGAGPKALAVAAKSHVLRQLGLSAPRVIAVEAHAVGGNWLASGGWTDGRHRLGTSPEKDIGFPYHSTWARGHNREINEAMMAFSWTSFLVEHGTYAEWIDRGRPSPQHHVWAKYLQWVARKIDLELVLGKVRTIRQRPTDGGAGWSVEVAGADGATTELEADGLMITGPGQSTKALAKHPRVLSIAEFWDLAGKRKLPISSRAAVIGGGETAGSALDELVRHEMLTISVISPMATIYTRGESYFENSLFSDPTKWNALSIQERRDVIRRTDRGVFSVRVQESLLGDNRVHHLQGRVTRIVGQGDGVAVTLRNEMRADQVHNFDLVVDATGGQPLWFLDLFDSESADLLELAVGGPLTQQRIESSIGYDLAVTGLGAKLYLPNMAALAQGPGFPNLSCLGELSDRVLRAEPARVRAGARQLAAQ

>CORE_REP|Org4_Gene7917#

MPLQQFESFIRSAVGHIDLNEEVESEMSNAGTPKTAAEIQQDWDTNPRWKGVTRNYTAEQVSKLQGTVVEEATLARRGSEILWDLVNNEDYINSLGALTGNQAVQQVRAGLKAIYLSGWQVAGDANLSGHTYPDQSLYPANSVPSVVRRINNALLRADEIAKVEGDDSVKNWLAPIVADAEAGFGGALNAYELQKAMIAAGAAGVHWEDQLASEKKCGHLGGKVLIPTQQHIRTLTSARLAADVADVPSVIIARTDAEAATLITSDVDERDREFLDGTRTAEGFFGVKNGIEPCIARAKAYAPYADLIWMETGVPDLEVARKFAEAVRGEFPDQLLAYNCSPSFNWKAHLDDATIAKFQRELGAMGFKFQFITLAGFHSLNYGMFDLAYGYAREGMTAFVDLQEREFKAASERGFTAIKHQREVGAGYFDTIATTVDPNTSTAALKGSTEEGQFH

>CORE_REP|Org105_Gene2713#

MINRGVVRMRNTKAVQAAEPEFVEVVIVGSGFGGLAAAKQLAKSGVPYVLISSTPEHLFQPLLYQVATGVLAADEIAPPIASILRRHEKADVRLGKVTAIDPDAAELVYETADGPRRIRYGSLIAATGANQSYFGRDDFAEKTFALKTIDDAKRLRAQIDHVFTQAKHADKETRERLLSFVVVGAGATGVEVAGQLAELAKRYYHQDVSVTLVEGAGEVLPPFGGGLSEYAKQSLTKGGVEVLLGTFVTDIEPGKVTVKDKQGVEHRIAAETVVWSAGVQASGFTKILAEATGAETDRAGRLLINPDLTVGGYADIYAIGDMTSLKGYPGQSPVAMQEGRHAADIIRRKKLPGTEFEYWDKGSMAVIRRRSAIAKVSDKIKFKGLIAWYMWLAVHLFYLVGFRNRFMAVMGWLVAFTGNGRPGFAEIDKDRPAVGHKPPIAA

>CORE_REP|Org103_Gene5459#

MACDLRGTSRTRRWSRGRRPYDSGVSVPQAVLLAVLAAVVGLAVGGLLIPYVNARQAARRQADSGLTMSQVLDLIVLASESGIAVVDQYRDVVLVNPRAEELGLVRNRLLDERAWAAVEKVLATGESAEFDLTAKNPLPGRSRIAVRGVARPLSQEETGFTVLFADDDSEQARMEATRRDFVANVSHELKTPVGAMSLLAEALLESADDPEAVRHFGQRVLGESRRLGKMVTELIALSRLQGAEKLPELEVVDVDTVVMQAVDRSRTAAEAAGITVSTDRPSGLEVLGDETLLVTALSNLVENAIAYSPPGSHVSVSRSLRGKYVAMAVTDRGIGIAKEDQERVFERFFRSDKARSRATGGTGLGLAIVKHVAANHNGEITLWSKLGTGSTFTLRIPAHLEADSGDDDVDADGAAVSTKENGSRPSGPGRPNGVEARR

>CORE_REP|Org100_Gene1857#

MDKLPGVSERFLVTGGNRLVGEVAVGGAKNSVLKLMAAALLAEGTTTITNCPDILDVPLMAEVLRGLGCEVTITDDAPGDRSVVTITTPAEPKYHADFPAVTQFRASVCVLGPLMARCKRAVVALPGGDAIGSRPLDMHQAGLRLLGATSEIEHGCVVARAEELRGARIRLDFPSVGATENILMAAVLAEGETVIDNAAREPDIVDLCNMLVQMGARISGAGTSVLTIQGVERLHPTEHRVIGDRIVAATWGIAAAMTMGDVRVTGVNPKHLALVLDKLRSAGARISFDVDGFRVVQPDRPRAVNFSTLPFPGFPTDLQPMAIGLAAIADGTSMITENIFEARFRFVEEMIRLGADARTDGHHAVVRGIPRLSSAPVWSSDIRAGAGLVLAGLVADGTTEVHDVFHIDRGYPNFVEQLQSLGGLVERVGGAE

>CORE_REP|Org105_Gene3182#

MRVARARPSVYVESVSFAHAAAMTGPADRLRVAMLTREYPPEVYGGAGVHVTELVSELRALAEVTVHCMGAPRDDAVVHQPDTHLYAANPAIQMMSAQLRMADATGEVDVVHSHTWYTGLAGHLSATLYGIPHVLTAHSLEPRRPWKAEQLGGGYRLSSWSERNAVEHADAIIAVSAGMRRDVLDAYPAVDPARVHVVHNGIDASVWHPGPPEAGGEPFLWQLGVRTDRPIAAFVGRITRQKGVAHLLAAARDFDSEIQVVLCAGAADTPELAAEVASAVEELSRRRGNVFWVQDMLPTEQIRQVLAAATVFVCPSVYEPLGIVNLEAMACGTAVVASDVGGIPEVVADRNTGRLVHYDPAAPSEYERGLAEAVNELAADQVLASEYGAAGRARAVAEFDWSRIAAQTLEVYDRVRKP

>CORE_REP|Org102_Gene1805#

MLGSTRLPRALPIRWAPVTVASPFDLIVVGSGFFGLTIAERTANLLGKRVLVVERRYHLGGNAYSEADPETGIEIHKYGAHLFHTSNKRVWDYVNQFTEFTGYQHRVFAMHKGQAYQFPMGLGLLSQFFGRYFTPDEARKLIAEQSAEIDTKDAANLEEKAISLIGRPLYEAFIRDYTAKQWQTDPKELPPGNITRLPVRYTFDNRYFNDTYEGLPKHGYTAWLAKMAESDLIEVRLDTDWFEVRDEIRAQNPDAPVVYTGPLDRYFDYAEGELGWRTIDFETEHLETGDFQGTSVMNYNDADVPYTRIIEPRHFHPERDYPTDKTVIMREYSRFAQTGDEPYYPINTPDDRAKLLAYRERAKTETAAAKVLFGGRLGTYQYLDMHMAIGSALNMFDNVLRPHLESGAPLVDTAE

>CORE_REP|Org134_Gene3544#

MELKVQVYRHASSIPGGCPPPTTPVVAGSGIVNAQVETVVDLDAIAHNVRILREHAGDAAVMTVVKADGYNHGAVEVGRAALAAGAAELGVTTISEAVHLREAGITAPILCWLNNSGADYGAGIAADIEIGISSMSQLRAVEAAARRLGRTATLTLKVDTGLNRNGVSVTEYRDVLTALRPLVDEQVLRFRAIFSHLAHADQPHHPTIDVQRDRFVDAIATAKEYGLVPEVTHLANSAAALTRPDLAFDMVRPGIAMYGLSPVPELGDFGLRPAMTFQAEISLIKHVAAGEGVSYGHEWIAPHDTTVALIPAGYADGVSRRLGGRCEVWVRGARRPSIGRVCMDQMVIDLGDNLDGVAEGDTAILFGTGESGEPHAQDWADLLDTIHYEVVCSPRGRVVRRFRGGQQ

>CORE_REP|Org142_Gene4996#

MRRDSVRRPGERRGSRPVSEDRVIFSRPYRAAAEVENLRAVLDSDHSHGDGRFTKTATAKIKAITNSPHALLTTSCTHALELGALLLELGQDDEVIVPSFAFTSAATAVALRGATCVFVDIDPATGNIDPMSVADAVTDRTKAVLVMHYGGVAADMAPLLEIAGEHGLALIEDNAHGLGGTWRGRALGTIGTIGTQSFHDTKNVHCGEGGALLLSDEILMGRAEIIREKGTDRARFLRGQVDKYSWQDIGSSYLPSELNAAVLDAQLAEFDRIQTGRHRVWDAYASALPEWARRNDVRLMQVPGDREHTAHLFYLRLPSEDIRDTMIRHLADRGIVAPFHYVPLDSSPAGLKYGRTPVPCTHSAEFSATIVRLPLWPMLGDDQIQRVVDAVTAFAV

>CORE_REP|Org102_Gene3646#

MGGVISAARPGTRATALGWDTGEVMTAPLPLVFDAPRRGMPPRHLADLDAEERRAVMADLGLPKFRADQIARQYYGRLQADPEQMTDLPADMRAKVGEALFPPLLTPVRHIACDDGSTRKTLWKAGDGTLLESVLMRYPDRATLCISSQAGCGMACPFCATGQGGLNRNLSTAEIVDQVRAAAAALRDGEVAGGPGRLSNIVFMGMGEPLANYKRVVNAVRRITSPAPDGLGISQRNVVVSTVGLAPAIRKLADEDLSVTLAVSLHTPDDELRDTLVPVNNRWPVAEVLDAARYYADKSGRRVSIEYALIRDINDQPWRADMLGKKLHKALGSRVHVNVIPLNPTPGSKWDASPKPVEREFVRRVEAQGVPCTVRDTRGQEIAAACGQLAAEG

>CORE_REP|Org1_Gene5478#

MPGPVVVSDTSPTVPASTGLWSGIMNGFLLARPDGVLRASGVRAAFDAVNDARAALRTGGAAIVVGALPFDPARPAALVAPAEQVHTAGPWRPAALPPLPRVQVVSEFPSAGEHLARVTKLVEQLDDPDTELRKVVAARSVLAEADGVLEPETVAAQLAARHPGASVFAVDLTAAGRTGATLIGASPELLVARRGRTVTLHPLAGTAPRRADPDADAAQAAELLDSAKNREEHSYVIEWIRDVLTPLCTELRIPEGPRLVETHDVWHLATPIVGTLREPAPTALDLAVLLHPTPAVCGTPTAAALETITRIEGDRGFYGGAVGWCDADGDGTWVVAIRCAELAADGRSLRAYAGGGIVAASQPQAELDETTAKLRTFLGGLDCAVPTH

>CORE_REP|Org105_Gene4411#

MLVRRGPRVSDEEAGVVAIRLLTGRSGERSSRMRSARRPGRTTRSGSWVKRAAIGVAAAMLVPMGVSIAGPAAPASAAFNPAGFDFWVDSGMGPIKSRIFRAKDGNTNRVVYALDGLRAPETLSGWEIDTNVAQLLTDWNINVVMPVGGMSSFYADWNAPSSFAGIPPGTGSSSGSGALNALAAGPGKSYRYQWETFLTQNLRWALRDRLGFNPNRNGVFGLSMGGSAALTLAAYHPDQFSFAGSYSGYLNISAPGMREAIRLAMLDAGGYNVDSMAPPWGPQWLRMDPFVFAPLLRDNNTRLWVSAGSGLPGPADGPTAGTVNGMALEALALANTRAFQLRMATLGANNVVYSFPNVGIHAWSYWAEEVARMTPDLSAHIG

>CORE_REP|Org145_Gene4685#

MNTVQVARPPRARGAVALRRSPPEGISAVTQAPVQTDILEIAREQVLERGEGLTQDQTLAVLRLGDDRLEELLGLAHEVRMKWCGPEVEVEGIISLKTGGCPEDCHFCSQSGLFQSPVRAAWLDIPSLVEAAKQTAKTGATEFCIVAAVRGPDARLMAQVAAGVEAIRNEVDIQVACSLGMLTQEQVDQLAAMGVHRYNHNLETAKSHFPNVVTTHTWEERWDTLRMVREAGMEVCCGGILGMGETLEQRAEFAAQLAELEPDEVPLNFLNPRPGTPFGDLEVLPAAEALKAVAAFRLALPRTILRFAGGREITLGDLGAKQGILGGINAVIVGNYLTTLGRPAESDLDLLGELKMPIKALNETL

>CORE_REP|Org216_Gene3268#

MLRRSLLRGLLKCKCVRCRRRHSLVRHRAVRPGRCGGAAARRCGMVVLNPEGPSAQARLLTAACRGVVRPVLRAAPITRATIPVGALAIDGLARLRPHPRGIEREQVTMPGFAMEIIRPAGAARAMRHGALLYLHGGGFAVCGLETHRPVAASLARRTGLPVVNVAYRQLPVRSITESIDDCLAAYRWLLRHGAEPDRIVFAGDSAGGYLTFATALRALECGLPAPAGLVGLSPLLDLDYAAKRDYVNVARDPYIPLSALAAVVRLGAEREGRLDPLLSPVNGALAHLPPVLLVAAEDEVLRFDAELMAARLDAAGVPNSVELWRGQVHAFMSIAPGLPESRAALGRVARFVRGRLADSQRARTA

>CORE_REP|Org4_Gene2114#

MAAHRRQGTLRLDTKRLVGGALAAGVLATTTVYGAGPVGADPVALPATAADAVQRMVDLSRQSEQLNEQALNAQSDLDTKLGLQREADAKLAASTDQVNRARDEVRKYQPIIDRTAIAAYQGARTNRLFAVLVSDSPQQLLDQMSTLDVLAAQTSDQLALYKKATDAAEGAEADARRASDEARAAADKAETVRGELERKRSDLSGAIVQVVQAWTGLSTKDKSALAGPAFPPGFDRDTLLQGLVPGSGTSALAASLTRIGDPYVWGATGPHQFDCSGLVQWAFKQVGKDVPRTSSQQASYGTPVAQNDLQPGDVVFFYNDISHVGIYAGNGLMVHASTFGVPVAVAPISTTPYHSARRY

>CORE_REP|Org13_Gene6761#

MRFGRSAAPVSHSALAPESAPRGRRGAQRGWRTRLLAAGAAMALPIAAGIMAPAAIAAPVHAPVHQTPAGGYDELMVPSSMGPIKVQVQWARNGGNAALLLLDGLRARDDRNAWSFETNAQQMFGNDNVTLVMPVGGQSSWYADWQGPSNTNGQKFTYKWETFLTKELPDFLSNYGVSRTNYAVAGLSMSGPAALRLAAFHRDQFKYAASFSGPLNWNAPGMREAIRVMMLDAGRFNVDSMAAPWSPQWLRSDPMVFAPQLRGLPMYISAASGLPGQYDHPNGLVGAFNTGNAMGIELISMVSTHSFKARLDSLGIPAAYDFPPTGTHAWLYWQDELAKARTGILAALNA

>CORE_REP|Org127_Gene3382#

MSNSGAKKLTPEQLRKLYRPGELTVVHEPAGVSAVTSALRGVGRTVALVPTMGALHEGHLELVRRAKRTNQVVVVSIFVNPLQFGENEDFDKYPRTLDSDVALLREEGVALVFAPSVAQMYPDGPRTSVHPGPLGAELEGASRPTHFAGMLTVVAKLLQIVRPHEAFFGEKDYQQLTLIRQMVRDLNFDVDIVAVPTVRESDGLALSSRNRYLDEQQRELAITLSAALAAGRHAAGRGPDAVLAAARSVLDGATGVDVDYLELRGSDLGPIPSSGNARLLVAARIGATRLIDNVPVSVPPAVSDAASAGPSVVTQAAASGPVAPADGHNAVPDFQPAQADA

>CORE_REP|Org114_Gene5945#

MARRARVDAELVRRGLARSREHAVELISAGRVLINGTVATKPATGVETATPLLVREEPDEVRWASRGAHKLLGALAAFEPQGVTVAGKRCLDAGASTGGFTDVLLSKGAAAVVAADVGYGQLVWRLRSDDRVEVHDRTNVRALTPELIGGTVELVVADLSFISLGLVLPALALCCAPGADLLPMVKPQFEVGKERVGSGGVVRDPALRAEAVRAVAAAAARLGLRTHGVVASPLPGPSGNVEYFLWLRKELSGADHSTGSITGAAADSSASAHPGVQSVPEDGAGTGLPAAPGAAAVGAAAYDAVEEERVAALIQRAVEEGPQ

>CORE_REP|Org105_Gene1446#

MRYAAPPLECAQRHARSACGDLGGEHVIDGGERVTGLKTGLEAVLARAHELPSPPARGAVTLRARGVSVDRRGGGAKARRVLAEVDFEVAAGEVVALVGPNGAGKSTLLAALAGELDPTEGSVELDGRPLTQWTPLDMARRRAVLPQSHTVGFPFSAGAVVAMGRAPWQRTALRERDQEIIAASMAATDVTHLAEQAFPTLSGGERARVALARVLAQDTATLLLDEPTAALDLGHQETVLRLADERAAAGAAVVIVLHDLGVAAAYADRVAVLDAGRIAADGPPRDVLTTELLTRVYQYPVEVLDHPVTGAQLVLPVRGGGGE

>CORE_REP|Org113_Gene6136#

MTADAGSDATQAVPPATSCFRTAVVPAAGLGTRFLPATKTVPKELLPVVDTPGIELVAAEAAESGAQRLVIVTSPGKDGVVAHFVEDLVLESTLAERGKFHLLEKVRKAPGLLDVSSVVQEEPLGLGHAVSQAEQVLDDDEDAIAVLLPDDLVLPCGVLDVMTRVRRKRGGSVLCAIDVPKQEVSAYGVFDVVPVPDATNPDVLRVVGMVEKPKLADAPSTFAAAGRYLLDRAIFDALRRIEPGAGGELQLTDAISLLIAEGHPVHVVVHRGSRHDLGNPGGYLRAAVDFALERDEYGPALREWLQRRLAPDWNPQLTSPQ

>CORE_REP|Org24_Gene597#

MATARRGRRRSGRSHRVAARSARRARLTGVTLPSVTPVTGRLVVTGARGQLGRALLDLAPDARGYTHADLDITDLDAVRAALRCGDVVINCAAYTAVDRAETDIDAACAVNARGPMALAVACGEVGARLIHVSTDYVFPGTGSRPYETADPTGPTSVYGKSKLAGERAVADLLPETGHIVRTAWVYTGTGSDFVATMRRLERERETVDVVDDQIGSPTYAPDLAAALVELAEQPDAPRILHAANAGQASWFDLARAVFAGVGADPDRVRPCSTSAFPRPAPRPAYSVLSTASWTAAGLSPLRPWQDALNDALAAASD

>CORE_REP|Org19_Gene6366#

MSALITPRDGRSCVVMGVVNVTSDSFSDGGRYLDPAVAVAHGVRLYEAGADIIDVGGESTRPGAVRIDPETEAQRVVPVIRGLVEAGVPTSVDTMRASVAAAAIDAGVSVVNDVSGGRADAEMVKVVAAAEIPWILMHWRANADHRHIGPADHYDDVVREVLAELSSQVDLAMAAGVHPSRLVLDPGLGFAKNAEHNWALLGALPELTAQGLPILVGASRKRFLGSLLGDESGPRPPDGREVATATISALAAQHGAWGVRVHDVRSSLDAIAVADAWRRAAESAERRAAESAERWAAESVERRAAEAGSHNQGSE

>CORE_REP|Org1_Gene4747#

MVMFSPPAAPLPTLCGKPVATDRALVMAIVNRTPDSFYDRGATFTDEAAMAAVDRAVAEGADLVDIGGVKAGPGSEVDAAVDIGGVKAGPGSEVDAAEETRRVVPFVAAIRAAYPDLLISVDTWRSEVARAAVAEGADLINDTWAGADPELVRVAAEHGAGIVCSHTGGAVPRTRPHRVRYADVVAEVTETVVAAAERAAAAGVRTDSILIDPTHDFGKNTYHGLELLRGLDVLVNSGWPVLMALSNKDFIGETLGVGLSERLEGTLAATAWSAAAGARVFRVHEVAHTRRVVDMIAAIQGIRPPARTLRGLV

>CORE_REP|Org113_Gene136#

MRGIILAGGTGSRLHPITRGVSKQLVPVYDKPMVYYPLSTLMLAGVRDVLVITTPEDAESFRRLLGDGTQFGMSIDYVVQPEPDGLARAFVLGADHIGTDCAALVLGDNIFHGPGLGTRLRRFDGLDGGTVFAYRVSDPSAYGVIEFVGGKAVSIEEKPKLPRSSYAVPGLYFYDNDVVEIARGLRPSARGEYEITDINRTYLEQGRLRVETLARGTAWLDTGTFDSLLDAANYVRTIEERQGLKIGVPEEVAWRMGFIDDEQLSRLAEPLVRSGYGTYLMDLLTRGKNDGTTADEYRDEQDD

>CORE_REP|Org120_Gene2356#

MIGPMKIRKAVIPAAGIGSRLLPLTKAIPKEMLPVGDKPVIEHTVRELVSSGITDITIVVSSGKSLIQDHFRPNPALVAQLRADGKTAYADAVEEVGELSRLGHITYLDQHGPYGNGTPVLNAARNLGDEPMLVLWPDDVFVADVPRAQQLINAYEQTGAPVLALMPMDPTESQRYGVPVVADDQGHGLLRITGLREKPKPEDAPSNYAAIGGYVVTPGVIEELRTQTRAWYEHRTGEVYLTDAINVHAADNPVYGQVIRGRWYDTGNPADYLVAQFASALANPQYGPLLRTLAEDTAS

>CORE_REP|Org49_Gene5817#

MSVGPRGHSDVPATQYEEESVKHIHAGKVRDLYEDGDELILVASDRVSVYDVVLPTPIPEKGALLTQLSNWWFRFFADVPNHLISTTDVPAEFAGRAVRAKKLSMVKVECIARGYLTGSGLAEYRRTGSVSGVALPPGLVEGDKLPEPIFTPTTKADEGHDEFITFDDVVNQEGREVAERLRDLTLDVYARGAEHAASRGVIIADTKLEWGWDGDVLTLGDEVLTSDSSRFWPADEYAPGRPQPSFDKQFVRDWSTSTGWNKEYPGPEIPADIVAATRAKYQQAYELITGETWTGVS

>CORE_REP|Org128_Gene4969#

MRLPRSRVGGHPIHKVDAAREHATLPESSLPIGVSADYELPGAARSDVRTEVEVRPEAEVGPQARAVVANGADFDDTESVAGDAESVAGDAESVAGDAADDALSGTAAFDATGDRTMMPSWDELVREHADRVYRLAYRLTGDPQDAEDLTQETFIRVFRSLQNYQPGTFEGWLHRITTNLFLDMVRRRNRIRMEALPEDYDRVPSEGPGPEQVYHDARLDPDLQRALDALAPEFRAAVVLCDIEGLSYEEIGATLGVKLGTVRSRIHRGRQALREYLAHNGSQQRFAAEEKVG

>CORE_REP|Org43_Gene3246#

MSRVSIDTHQAWVEFPIFDAKSRSLKKAFLGKAGGAIGRNQSDVVVVEALRDINLSLREGDRIGLVGHNGAGKSTLLRLLSGIYEPSRGSARIRGRVAPVFDLGVGMDPEISGYENIIIRGLFLGQTRKQMMSKIDEIADFTELGEYLHMPLRTYSTGMRVRLAMGVVTSIDPEILLLDEGIGAVDAEFMKKARLRLQELVARSGILVFASHSNEFLAQLCDSALWIDHGQIRLRGGIEEVVRAYEGPDAGNHVATVLREMAAERAGRAEGSADERELEQNAT

>CORE_REP|Org19_Gene2600#

MRNPLATPTGCGCPARHRRRREQHCAARSRRRPGAGVPQDTGGVVTSAERPPAATRVLVVDDEPQILRALRINLSVRGYEVITAATGAAALRAAAEKHPDVVVLDLGLPDIDGVEVLAGIRGWSSMPVIVLSARTDSSDKVQALDTGADDYVTKPFGMDELLARLRAAVRRSASTAEESAPIVETSSFTVDLAAKKVIRGGRDVHLTPTEWGVLEMLVRNQGKLVGRRELLREVWGPTYATETHYLRVYLAQLRRKLEDDPSQPKHLLTEAGMGYRFQA

>CORE_REP|Org31_Gene3788#

MGGLLEGKTILVTGIITDSSIAFHAAAVAQEQGAKVIITGIPERLRLIDRIAKRLPQEVPPAIPLDVTSEENLAELADKLRELAPEGIDGVLHSIAFAPRTLMGPEALPFLDGPGPDAAKAFEISAWSYASLARAVLPVMNERGSIVGMDFDPRTAMPFYNWMGVAKAALESVNRYVAREVGAAKKIRSNLIAAGPIKTLAAKAIAGTATDDAAKLNQLNEYWDGASPIGWDVDDPTVVAKSIVAMLSDWLPGTTASIIYVDGGASHNTWFPEDMSIN

>CORE_REP|Org15_Gene4889#

MSDGTGLLADKVVVISGVGPGLGRSLCVQAAAAGAKVVLAARTESRLREVADEIDGAGGTSLIVPTDITDDAAVANLVERTVATFGRVDALINNAFAMPSMKSLARTDFQQISDSLELTVLGTLRATQAFTDELAKTRGAVVMINSSVLRHSEPRYGSYKVAKSALLAMSQTLATELGAKGIRVNSVAPGYIWADRLKWYFGEVAKKYGITVEQVYEQTASRSDLKRLPEPDEIARAVVFLASEWASAITGQTLDVNCGEYHA

>CORE_REP|Org37_Gene1288#

MPAPGRRSRRGPASARRIRGHGPAAPGHDGNGEPVSGRADSDGDTPIRVLLVDDEQLVRSGFRLLLDIEDDITVVGEAANGAEAVRKARALRPDVVLMDIRMPTMDGIQATREIAATTGLQDVRILILTTYDTDAYVFEGLQAGASGFLLKDAGPAELLHAIRVVAAGEALLAPRITRRLIAQFTARRAADRAAEQRLAVLTDREREVLALVGQGMSNAEIGAELFLSPATARTHVSRAMVKLGARDRAQLVVIAYRTGLVAP

>CORE_REP|Org71_Gene936#

MADGRAAGHPDVEGSTRTRCGLRPFGADQTGERVTTAATETTTAVLVVDDQELVRGGLRRILRRRDGFVLTECADGDEVVPAISAEPPDVILMDLRMKRVGGIEATRLVRMRADAPPVLVLTTFDDDQLLSGALRAGAAGFILKDSPAEDLIRAVRTVAAGGAWLDPAVTGRVLSAYRTVRPATPTDARLAELTAREYEVLELIGRGRVNSEIARELGISEVTVKSHVGHIFGKLDLRDRAAAIVFAFDHGVVSPGQSTV

>CORE_REP|Org117_Gene417#

MTVAVRVIPCLDVDAGRVVKGVNFQNLRDAGDPVELAATYDAQGADELTFLDVTASTGDRGTMIDVVTRTAEQIFIPLTVGGGVRTVEDVDRLLRAGADKVSVNTAAIARPEVLREMSERFGSQCIVLSVDARTVPDGQPDTPSGWEVTTHGGKRGTGIDAVEWAERGAELGVGEILLNSMDADGTKTGFDLPMIRAVRAAVSIPVIASGGAGAVEHFAPAVQAGADAVLAASVFHFGDLTIGQVKDSLRDAHLVVR

>CORE_REP|Org114_Gene5983#

MLELTDVTKEYRVGEQTVRALDGISLRIEPGEFTAIIGPSGSGKSTLLHMLGALDSPDSGSIRFQDAEIGGLDDDRQSEFRRHRVGFVFQFFNLLPTLSAWENVAIPKLLDGTGLRKAKPRALELLELVGLADRAEHRPAELSGGQMQRVAVARALIMDPPLILADEPTGNLDSKTGASILELLGDITRQGNSVVMVTHDMGAVRYCDRLITLRDGKIGSNELVEHTENGEVRTVPVELTASLSEDGSEPAQAVRP

>CORE_REP|Org2_Gene6607#

MNSLTPAVSLLTSNNDGVNTTSSSVPAASVLVAEDDPHVRSTLDQLLRFEGYQVYLAADGQEALELLAQQRPDLAVVDVEMPRLDGLSLCRLLRRRGDRLPILVLTARQQIGDRVAGLDAGADDYLPKPFATDELLARLRALLRRSTFDEDDDTVLAVGDLTLNTATRQVHRGDRPIELTKTEFDVLELLLRNARIVLSRSRIYEHIWGFDFDTESRSLDVYIGYLRRKTEENGEPRLIHTVRNVGYSVRPA

>CORE_REP|Org5_Gene560#

MSRMNGVAGDRIPEARVLVVDDEPMIVELLSVSLRYQGFEVAAAGNGAEGLDRAKQFRPDALIVDVMMPGMDGFGLLRRLRADGIDAPVLFLTARDEVDDKITGLTLGADDYVTKPFSLEEVVARLRVILRRSGHVVEETKSSRIRFEDIELDDDTHEVWKAGEPVALSPTEFTLLRYFMVNAGTVLSKPRILDHVWRYDFGGEVGVVETYVSYLRKKVDTGPDRLIHTLRGVGYVMRAPSRSRSSAK

>CORE_REP|Org31_Gene655#

MVPLPDAYRTSELVSTPKVLVVDDDEDVLASVERGLRLSGFHVLVARDGAQALRSVSEHAPDAIVLDMNMPVLDGAGVVTALRAMGNEVPICVLSARASVDERISGLESGADDYLVKPFVLAELVARIRALLRRRTDTPPAATPGAITVGPLEVDIAGYRAVLHGNEIELTKREFELLSTLARNVGVVLSRERLLELVWGYDFAADTNVVDVFVGYLRRKLEVDGAPRLLHTIRGVGFVLRAPK

>CORE_REP|Org114_Gene4108#

MSANLMIVEDDDRVRVALRLAMEDEGYDVAEAEEAEVALRQLRDNGAPDFMIVDLMLGGMDGFTCIREIRRDHDVPIIVVSARDDTHDVVAALEAGADDFVTKPFEVKEITARMRAVARRARFAEQAAAEEDPDSELGTMVLDEQAGNPLVLSTESGIVRRGDEEIHLTLTEYRLLCELAGSAGRVLSRGTLLERVWDRGFFGDERIVDVHIRRLRTKIERDASDPQLIVTVRGLGYRLDVQR

>CORE_REP|Org12_Gene5846#

MSTDTVKSEKATNLTLPVKEPGGKTNGTVELPAEIFDATANIALMHQVVVAQQAAARQGTHATKTRGDVRGGGKKPYRQKGTGRARQGSTRAPQFTGGGTVHGPQPRDYSQRLPKKMKAAALRGALSDRARNERIHVISELVAGQTPSTKAAKNFLAELSDRKKVLVVVGREDVTAWKSVANLQGVHPIAPDQLNTYDVLLSDDVVFSVEALNAFVHGPTESAQESQPGSAAQEESK

>CORE_REP|Org102_Gene4053#

MHDEGVKQVKDLVDTTEMYLRTIYDLEEEGVTPLRARIAERLEQSGPTVSQTVARMERDGLLTVAGDRHLELTEKGRAMAVAVMRKHRLAERLLVDIIGLDWQNVHAEACRWEHVMSEEVERRLVEVLNHPTTSPYGNPIPGLDELGVTGTSNAEEKLVRLSDLPSGQSAAVVVRRLSEHIQTDPEIINQLREAGVVPDARVNVETKPGAVVILVPGHAGFELSDEMAHAVQVKLV

>CORE_REP|Org44_Gene3372#

MTSVLIVEDEESLADPLAFLLRKEGFEVTVVGDGPSALAEFDRSGADIVLLDLMLPGMSGTDVCKQLRTRSGVPVIMVTARDSEIDKVVGLELGADDYVTKPYSARELIARIRAVLRRGAGDELDGNGESGVLEAGPVRMDVDRHTVMVNGKPVTLPLKEFDLLEYLLRNSGRVLTRGQLIDRVWGADYVGDTKTLDVHVKRLRSKIEADPAKPEHLVTVRGLGYKLEA

>CORE_REP|Org96_Gene3994#

MITMRNVTKSYKTSTRPALDNITVDVDKGEFVFIIGPSGSGKSTFMRLLLKEESPTAGEIRVADFRVDRLPGRKVPKLRQRMGCVFQDFRLLQQKTVQENVAFALEVIGKRRQVIERTVPEVLDMVGLGGKADRLPSELSGGEQQRVAIARAFVNRPLVLLADEPTGNLDPDTSGEIMLLLERINRTGTTVLMATHDNHIVDAMRRRVVELDHGRLVRDEATGVYGVGR

>CORE_REP|Org102_Gene5430#

MNTTSRGDSVDGGGRVTNGRTKRLALLDYGSGNLHSAERALVRAGAEVTVTADPDIALNADGLVVPGVGAFAACMAGLQEVRGERIIGKRLAGGRPVLGICVGMQILFERGVEHGIETAGCAEWPGTVERLDAPVLPHMGWNTVRAPEDSVLFAGMDADTRFYFVHSYAAQKWEWNGDGTIAPAKLTWAEHGVPFLAAVENGPLSATQFHPEKSGDAGAQLLRNWVRSL

>CORE_REP|Org7_Gene3709#

MTAVLLAEDDEAIAAPLSRALGREGYSVTVERFGPAVLERALEGHHDLLILDLGLPGMDGLEVCRQVRASGADIAVLMLTARTDEVDFVVGLDAGADDYVGKPFRLAELLARVRALLRRSGIGDDTVEVGGIRLEPAARRVLVNGAEIGLANKEYELLKVLIDRAGQVVPRETILREVWGDAELRGSKTLDMHMSWLRRKIGDEGPMAERRIVTVRGVGFRLNTD

>CORE_REP|Org1_Gene1099#

MTMARVLVASRNAKKLAELRRILDDAGVAGVQIVGLDDVPPYDEAPETGATFEENALAKARDGAAATGLPCVADDSGLAVDALNGMPGVLSARWSGTHGDDAANNALLLAQLRDVPDERRGARFVSACALVVPGGTETVVRGEWPGTIGRKPMGEGGFGYDPLFVPDGGDVTAAQLTPAAKDAASHRGRALRHLLPALAALADRTE

>CORE_REP|Org152_Gene5548#

MTVEIRELSVPGAWEFTPRLHGDARGVFLEQFKASEFEKAVGRPFDLQQVNVSTSAAGVLRGIHYTANPPGQAKYVTCVRGAFLDVVVDLRPDSPTFGRWDAVVIDDVTRRSVFLAEGLGHALLSLADDSTVTYLCSLEYTPEFDAEVDAFDPAIGIEWPTMGRDGQPLTVIRSAKDAAAPPLSDARLLY

>CORE_REP|Org170_Gene1954#

MSRIGKQPIAIPSGVEVTINGQDIAVKGPKGQLSLTVSEPITVTKGEDGQLQVARPDDERRSRALHGLTRTLVANMIEGVTKGYEKKLEIAGVGYRVALKGQNLEFALGYSHPVVAEPPQGITFAVESPTKFSVAGIDKQLVGEVAANIRKYRKPEPYKGKGIRYAGENVRRKVGKTGK

>CORE_REP|Org151_Gene30#

MLRTMMKSKIHRATVTHADLHYVGSVTVDQDLLDAADLLEGEQVCIVDIDNGARLETYVIAGERGSGVIGINGAAAHLVHPGDLVILIAYGQMNEQEIAEYDPKVVFVDERNRPVELGSDPAHAPEGSGLTSPRSLSFAG

>CORE_REP|Org56_Gene4690#

MADRVLRGSRLGAVSYETDRDHDLAPRRVARYRTDNGEEFDVPFADDAEIPPTWLCRNGQEGILIEGTTQEPKKVKPPRTHWDMLLERRSKEELEELLQERLELLKTRRGR
